# A systems biology approach identifies a regulator, *BplERF1*, of cold tolerance in *Betula platyphylla*

**DOI:** 10.48130/FR-2021-0011

**Published:** 2021-06-30

**Authors:** Kaiwen Lv, Wenqi Wu, Hairong Wei, Guifeng Liu

**Affiliations:** 1 State Key Laboratory of Tree Genetics and Breeding, Northeast Forestry University, Harbin 150040, China; 2 Beijing Advanced Innovation Center for Tree Breeding by Molecular Design, Beijing Forestry University, Beijing 100083, China; 3 College of Forest Resources and Environmental Science, Michigan Technological University, Houghton, MI 49931, United States of America

**Keywords:** *Betula platyphylla*, gene regulatory network, cold stress, *BplERF1*

## Abstract

Cold is an abiotic stress that can greatly affect the growth and survival of plants. Here, we reported that an *AP2/ERF* family gene, *BplERF1*, isolated from *Betula platyphylla* played a contributing role in cold stress tolerance. Overexpression of *BplERF1* in *B. platyphylla* transgenic lines enhanced cold stress tolerance by increasing the scavenging capability and reducing H_2_O_2_ and malondialdehyde (MDA) content in transgenic plants. Construction of BplERF-mediated multilayered hierarchical gene regulatory network (ML-hGRN), using Top-down GGM algorithm and the transcriptomic data of *BplERF1* overexpression lines, led to the identification of five candidate target genes of BplERF1 which include *MPK20*, *ERF9*, *WRKY53*, *WRKY70*, and *GIA1*. All of them were then verified to be the true target genes of BplERF1 by chromatin-immunoprecipitation PCR (ChIP-PCR) assay. Our results indicate that BplERF1 is a positive regulator of cold tolerance and is capable of exerting regulation on the expression of cold signaling and regulatory genes, causing mitigation of reactive oxygen species.

## INTRODUCTION

Cold stress is a major environmental factor that affects plant growth, geographic distribution, and productivity^[1]^. Under cold stress, plants first initiate stress signaling transduction pathways, which then cause re-programming of both biochemical and physiological responses, leading to differential gene expression^[2]^, hormone turnover and homeostasis^[3]^, and reactive oxygen species (ROS) scavenging capability^[4]^. The mitogen-activated protein kinase (MAPK) signaling pathway plays an important role in regulating cell growth and development, cell proliferation and the transmission of abiotic stress signals^[5−7]^. The MAPK cascade signaling pathway comprises MAPKKK, MAPKK and MAPK components^[6]^. When cells senses a signal through the receptors or sensors, MAPKKK can be activated in multiple mechanisms, which then modulates downstream reactions through phosphorylation of substrates, such as transcription factors and protein kinases, and the signal is transmitted through the MAPKKK-MAPKK-MAPK signaling cascade^[8,9]^. Cold induced reactive oxygen species (ROS) can activate a MAPK cascade (MEKK1-MKK2-MPK4) that regulates tolerance to cold acclimation^[10]^. In *Arabidopsis thaliana*, MPK3 and MPK6 reduce the stability and transcriptional activity of ICE1, a basic helix-loop-helix transcription factor that regulates the expression of CBF genes, through phosphorylation, thereby negatively regulating CBFs^[11,12]^. In *A. thaliana*, MKK2 is specifically activated by cold, and can directly regulate MPK4 and MPK6^[13]^. In rice, the OsMKK6-OsMPK3 cascade phosphorylates OsbHLH002, which promotes the expression of the trehalose biosynthesis gene, OsTPP1, leading to an accumulation of trehalose content and augmented resistance to chilling damage^[14]^.

The Ethylene Response Factor (ERF) family is primarily involved in plant growth and biotic/abiotic stress response processes. For example, overexpression of poplar ERF76 in tobacco transgenic lines altered leaf length-to-width ratios, boosted root and height growth, as well as the salt tolerance^[15]^. Overexpression of *TERF2* in transgenic rice lines enhances cold tolerance without significantly affecting the growth and other agronomic traits, through reducing ROS, electrolyte leakage, malondialdehyde (MDA)^[16]^. Overexpression of *CdERF1* cloned from Bermudagrass enhances cold tolerance of A. thaliana transgenic plants, and further physiological assays reveal the elevated activities of superoxide dismutase (SOD) and peroxidase (POD), and reduced contents of electrolyte leakage, MDA, H_2_O_2_ and $   {\text O}_{\text 2}^{\text -} $^[17]^. The study of four phylogenetically closely related ERF family genes, *ERF102*/*ERF5*, *ERF103*/*ERF6*, *ERF104* and *ERF105*, unveiled their little impact on shoot and root growth but significant contributing roles in the cold stress response^[18]^ and tolerance^[19]^. Overexpression of cold or low-temperature-inducible *VaERF092* or its target gene *VaWRKY33* from *Amur grape*, enhances cold tolerance of *A. thaliana* transgenic plants^[20]^. Antisense suppression and over-expression of rice *OsERF3* reveal that it positively regulates two MAPKs and two WRKY genes as well affecting the concentrations of jasmonate (JA), salicylate (SA) and the activity of trypsin protease inhibitors (TrypPIs)^[21]^.

*Betula platyphylla*, also referred to as white birch, is a pioneer tree species that is primarily distributed in temperate or subarctic regions in Asia, including Japan, China, Korea, and Siberia^[22]^, where it can survive very low temperatures, far below freezing point. In Heilongjiang province, China, it can tolerate −40 °C~−50 °C^[23]^. In this study, *B. platyphylla* was used to identify genes that contribute to birch's cold tolerance. To do this, we first employed the Algorithm for the Reconstruction of Gene Regulatory Networks (ARACNE) to construct the gene regulatory network centered on MAPK signaling genes using a merged data set from two time-course RNA-Seq data sets of *B. platyphylla* under cold stress treatment. There were 17 ERF family genes that emerged in this network, among which *BplERF1* was ranked as one of the highest ethylene response genes based on their connectivity to the MARK signaling transduction pathway genes. We then developed transgenic lines of *B. platyphylla* overexpressing *BplERF1* and performed freezing experiments to substantiate that *BplERF1* boosted the antifreeze capability of overexpression lines, and then characterized the *BplERF1* transgenic lines. We found that the POD and SOD activities increased while the H_2_O_2_, $   {\text O}_{\text 2}^{\text -} $ and MDA content decreased in the *BplERF1* transgenic lines. We used Top-down GGM^[27]^ to infer the five target genes of BplERF1 whose orthologs have been demonstrated to regulate stress response and tolerance in other plant species. Finally, we used ChIP-PCR assay to substantiate that all of the five target genes predicted are the true target genes of *BplERF1.*

## RESULTS

### Identification of differentially expressed genes (DEGs) in response to cold stress treatment (4 °C)

The RNA-seq data sets yielded from two cold treatment experiments were aligned to the silver birch genome using Bowtie2^[24]^ and TopHat^[25]^ to obtain raw counts. The modified software package of *Pop's Pipe*^[26]^ was then employed to identify the DEGs. DEGs were identified by comparing the adjacent time points, as shown in Table 1. A total of 8,583 unique DEGs were identified from all comparisons of all adjacent time points (Supplemental Table S1).

**Table 1:** Differentially expressed genes in leaves of *Betula Platyphylla* under cold treatment.

Cold treatments (wild type at 4 °C)	Up regulated genes	Down regulated genes	Total DEGs
1 h versus 0.5 h	2,193	2,256	4,449
1.5 h versus 1 h	813	517	1,330
2 h versus 1.5	9	58	67
2.5 h versus 2 h	3	2	5
3 h versus 2.5 h	10	2	12
24 h versus 6 h	2,573	2,569	5,142
2 d versus 24 h	179	240	419
4 d versus 2 d	41	51	92
7 d versus 4 d	0	0	0
14 d versus 7 d	134	311	445

### The association network between MAPK signaling transduction pathway and transcription factors

**Figure 1:**
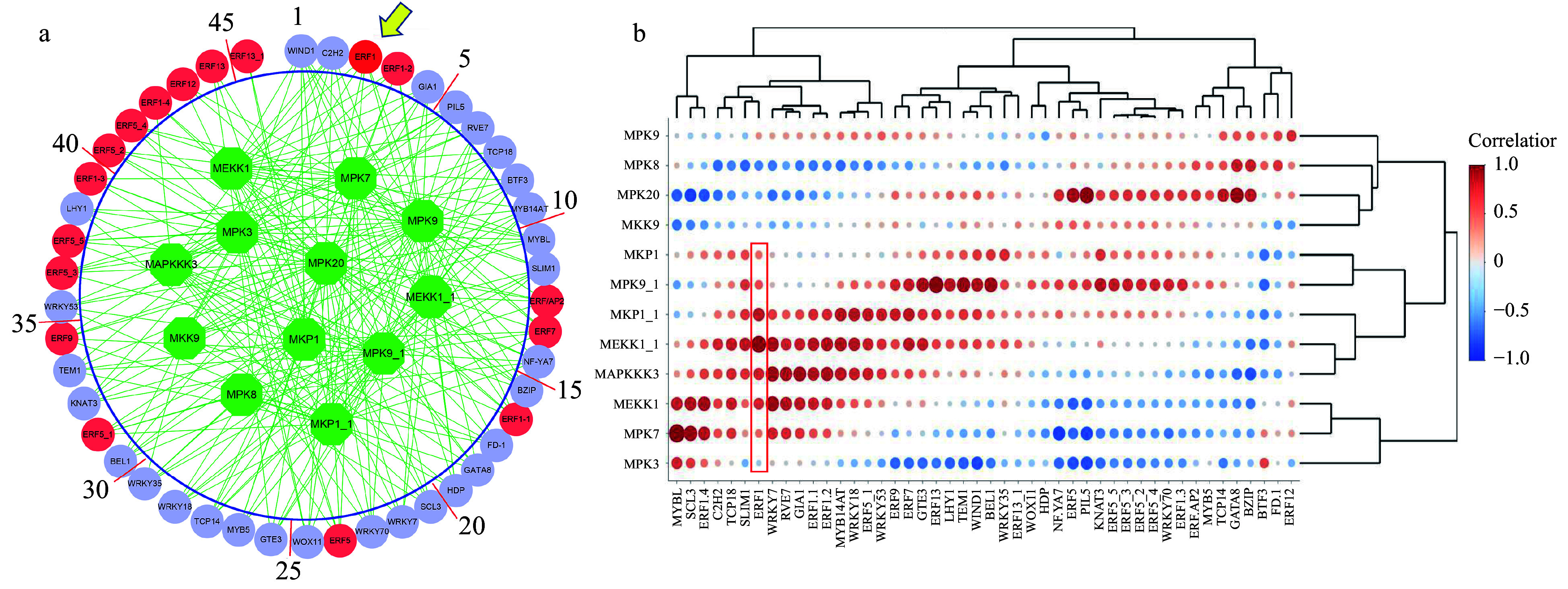
The association between MAKP signaling transduction pathway and transcription factors. (a) The association network was inferred by the Algorithm for Reconstruction of Gene Regulatory Network (ARACNE). Green circles represent MAPK signaling transduction pathway genes. Red circles represent ERF family genes, while lilac circles represent other regulatory genes inferred. (b) The heatmap of the logged correlation *p*-values (with a base of 10) between MAPK signaling transduction pathway genes and transcription factors. The sizes of the dots in the heatmap are negatively proportional to the *p*-values.

We constructed an association network between all transcription factors in the DEGs and MAPK signaling pathway genes using RNA-seq data sets normalized with the TMM (weighted trimmed mean of M-values) algorithm. The method used for association is ARACNE and the resulting network is shown in Fig. 1a. The 12 MAPK-pathway genes involved in cold stress are shown in the green nodes within the big circle. We obtained 17 ERF family genes (red nodes represent ERF family genes). The lilac circles represent other predicted regulatory genes that were conjectured to regulate the MAPK pathway genes or vice versa under cold stress. Among all these ERF genes, *BplERF1* ranked as one of the top genes. Therefore, we speculated that *BplERF1* gene might play a more important role in response to cold and regulate MAPK cold stress signaling pathway or vice versa. The correlation matrix of MAPK genes and these transcription factors are shown in Fig. 1b.

### Tissue-specificity and subcellular localization of BplERF1

**Figure 2:**
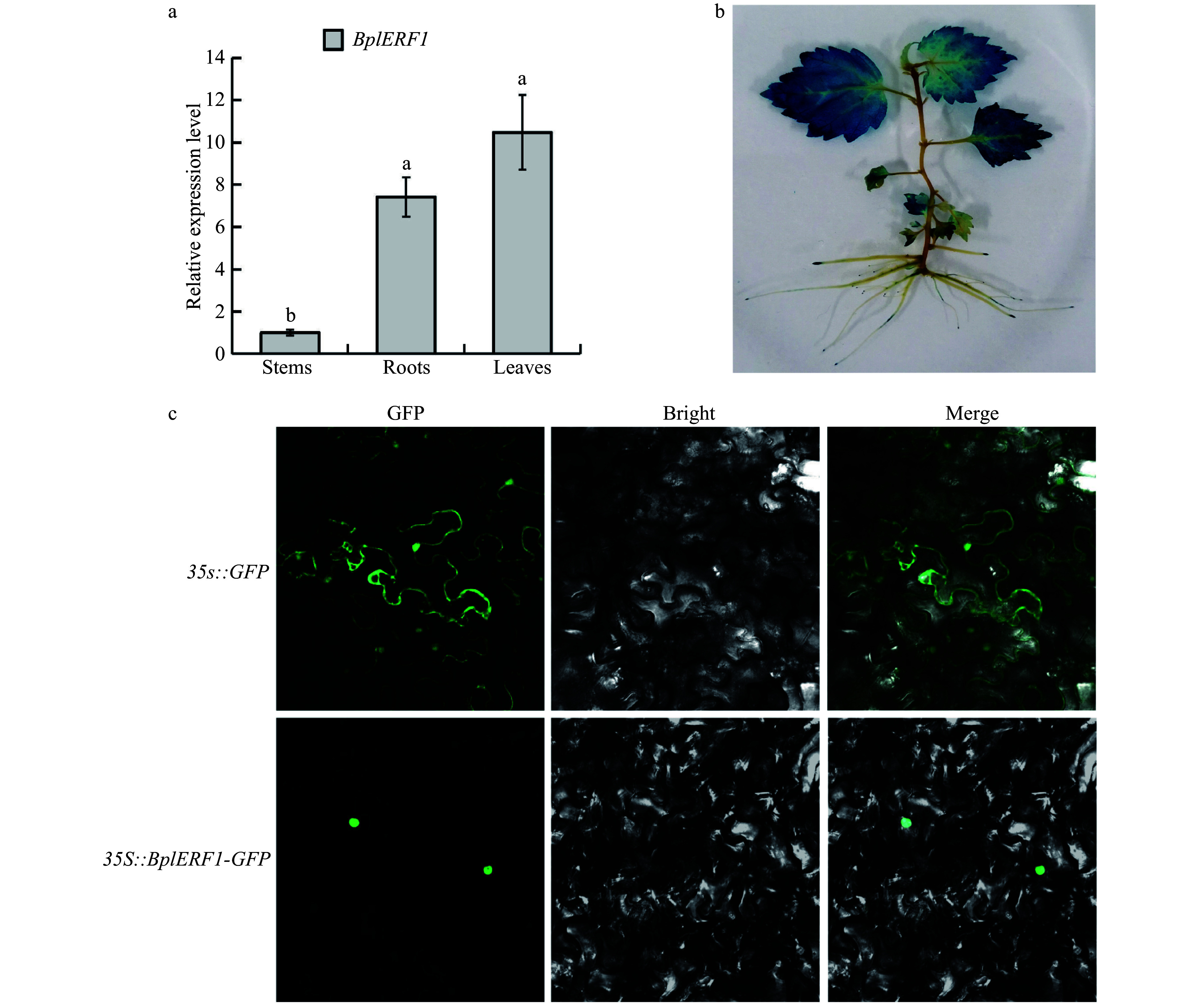
Tissue-specific expression patterns of *BplERF1* gene and subcellular localization of BplERF protein. (a) Expression patterns and temporal, tissue-specific expression pattern of *BplERF1* in the two-month-old wild-type *Betula platyphylla* plants measured by RT-qPCR under normal conditions. One-way ANOVA was performed to test the significant differences among tissues with a threshold *F* statistic (0.05). Multiple comparisons of *BplERF1* expression levels in roots, stems and leaves were carried out by the Fisher's LSD method, and statistically significant differences are denoted by different lowercase letters. (b) GUS staining of the *pBI101-BplERF1pro::GUS* transgenic lines. (c) Subcellular localization of BplERF1 proteins in the onion epidermis cells. Two plasmid constructs, *pBI121*-*35S::BplERF1-GFP* and *35S::GFP *(control), were used to transform tobacco epidermal cells. Images were obtained using a confocal laser scanning fluorescence microscope.

We cloned the 564 base pair (bp) cDNA fragment of* BplERF1* from *B. platyphylla*; the protein has a molecular weight of 20.12 kDa. According to the results of RT-qPCR (Fig. 2a), we found that *BplERF1* had the highest expression in the leaves, which was also supported by the GUS staining of* BplERF1* overexpression transgenic lines where the GUS gene was driven by *BplERF1* promoter*.* The GUS proteins were highly expressed in the leaves. *BplERF1* promoter drove GUS expression in roots too, but the GUS proteins were primarily shown at the root tips (Fig. 2b). As shown in Fig. 2c, we found that BplERF1 was localized in the nucleus, indicating that it was involved in transcriptional regulation.

### Identification and validation of *BplERF1* transgenic lines

**Figure 3:**
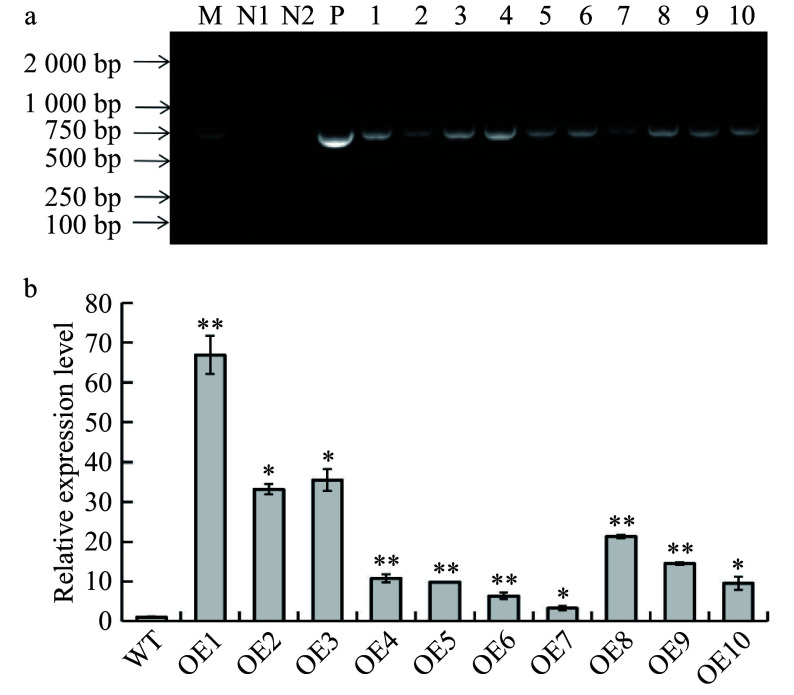
Identification and validation of *BplERF1* transgenic lines. (a) Identification *BplERF1* transgene in the transgenic lines using PCR with specifically designed primer pair. M: DL2000 Marker; N1: distilled water; N2: wild-type genomic DNA; P: positive plasmid carrying* BplERF1.* 1−10: Genomic DNA of transgenic lines. (b) RT-qPCR detection of cDNA from different transgenic lines. The relative expression levels in other transgenic lines were normalized using that of wild type, which was set as 1. Asterisks indicate levels of the significant difference of *BplERF1* overexpression lines in comparison with wild type. Three biological replicates were used to calculate the means and standard deviations of each transgenic line, which are shown as bar heights and error bars, respectively (Student's* t* test, *p < 0.05, **p < 0.01).

A total of 12 putative *B. platyphylla* transgenic lines were obtained through gene transformation and positive medium selection. These putative lines were subsequently examined with PCR and RT-qPCR assays. Ten transgenic lines were verified to harbor the *BplERF1* transgene and thus are true transgenic lines (Fig. 3a). The *BplERF1* was highly but differentially expressed in different transgenic lines (Fig. 3b). The relative expression level of *BplERF1* in OE1 line was the highest as compared to all other transgenic lines, and was ~60 times higher than that of the wild type. The three lines with the highest relative expression levels (OE1, OE2, OE3, > 30 times) were selected for further analysis.

### Comparison of root lengths between *BplERF1* transgenic lines and wild type plants

**Figure 4:**
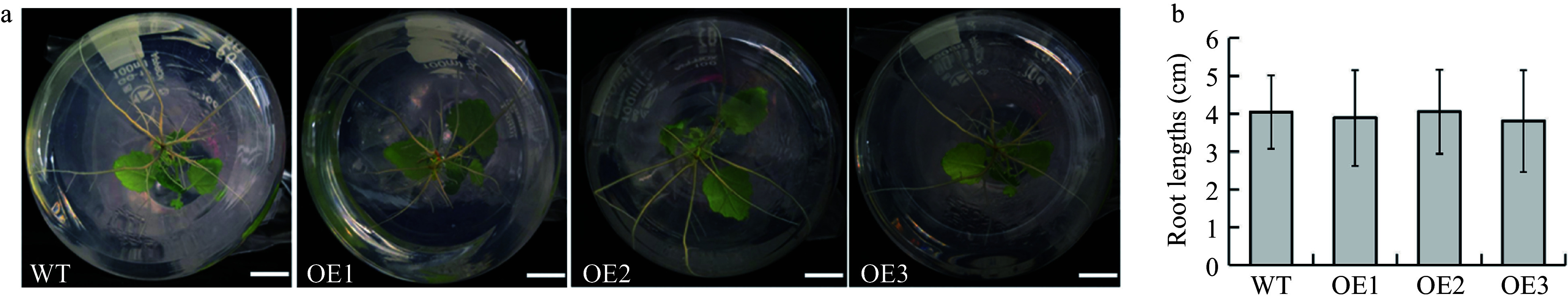
Comparison of root lengths between *BplERF1* transgenic lines and wild type. (a) Morphological phenotypes of roots in *BplERF1* transgenic lines and wild type plants. Bar represents 1 cm. (b) The root lengths of *BplERF1* transgenic lines and wild type. Values are mean ± SD with 20 replicates.

*BplERF1* overexpression transgenic lines and wild type plants were cultured in tissue bottles for two months. Based on the results shown in Fig. 4a and b, the average root length of each *BplERF1* transgenic line had no significant change as compared to that of wild type. All the *p* values for *BplERF1 OE1* vs wild type, *OE2* vs wild type and* OE3* vs wild type comparisons were insignificant because their *p*-values fell into the range of 0.50 to 0.96 (> 0.05 cut-off threshold).

### Assessment of the stress tolerance of *BplERF1* overexpression transgenic plants

**Figure 5:**
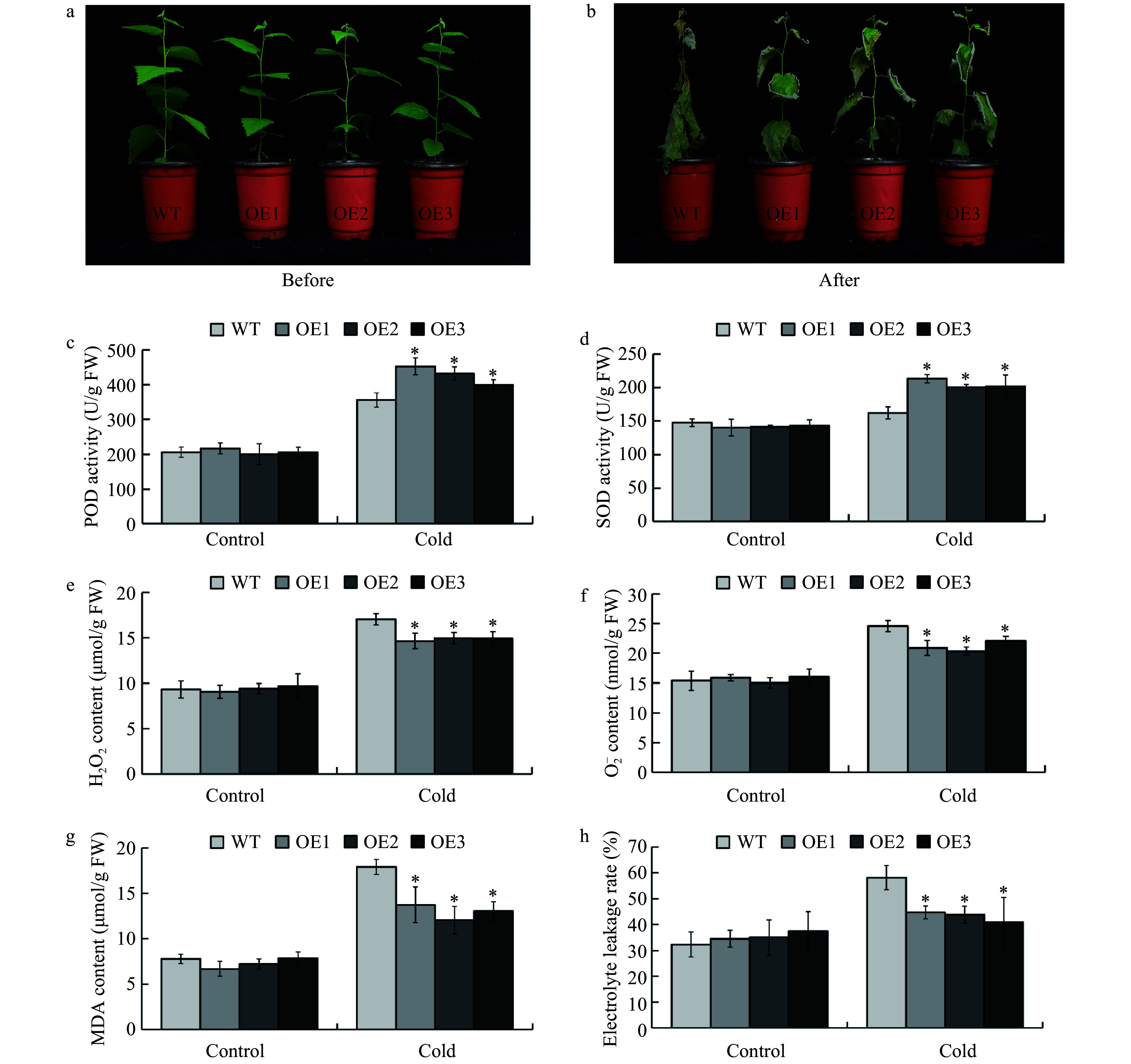
Overexpression of *BplERF1* conferred augmented cold tolerance of transgenic lines through enhancing the scavenging capability. (a−b) Phenotypic performance of two-month-old *BplERF1* transgenic lines and wild-type after a 4 °C for overnight treatment followed by a −5 °C freeze for 2 h. Photos were taken immediately before the cold treatment and after the cold treatment with a 10-d recovery. (c) POD activity. (d) SOD activity. (e) H_2_O_2_ content. (f) ${\rm O}_2^- $ content. (g) MDA content. (h) Electrolyte leakage. Three biological replicates were sampled and measured immediately after freezing treatment. An asterisk indicates a statistically significant difference between a treated group and a control group determined by Student's *t* test (*p* < 0.05).

The *BplERF1-OE* transgenic lines and wild type were cultivated in a greenhouse for two months (Fig. 5a). After cold treatment, namely 4 °C overnight prior to −5 °C for 2 h, and a 10-d recovery in the greenhouse, all wild-type plants withered and eventually died. Although the transgenic lines suffered severe damage from freezing and the leaf edges became curled, some transgenic plants survived but their leaves were partially dead (Fig. 5b). We harvested the leaves immediately after freezing treatment and measured the activities of POD and SOD, which are the enzymes responsible for removing H_2_O_2_ and superoxide radicals (${\text O}_{\text 2}^{\text -} $), respectively. The results were shown in Fig. 5c and d, respectively. After cold stress treatment, the POD and SOD enzyme activities in the transgenic lines were significantly higher than those in the wild type plants. Correspondingly, the contents of H_2_O_2_ and ${\text O}_{\text 2}^{\text -} $ were significantly lower than those in the wild type plants (Fig. 5e, f). Thus, *BplERF1* overexpression in transgenic lines eliminated ROS and diminished the toxicity of ROS. The MDA and electrolyte leakage rate are important indicators for membrane damage under abiotic stresses. Based on the results shown in Fig. 5g−h, the MDA and electrolyte leakage rate in the transgenic lines were significantly lower than those in the wild type upon freezing treatment. Taken together, overexpression of *BplERF1* augmented the cold tolerance through enhancing the scavenging capability of the transgenic plants.

### BplERF1-mediated multilayered hierarchical gene regulatory network (ML-hGRN)

**Figure 6:**
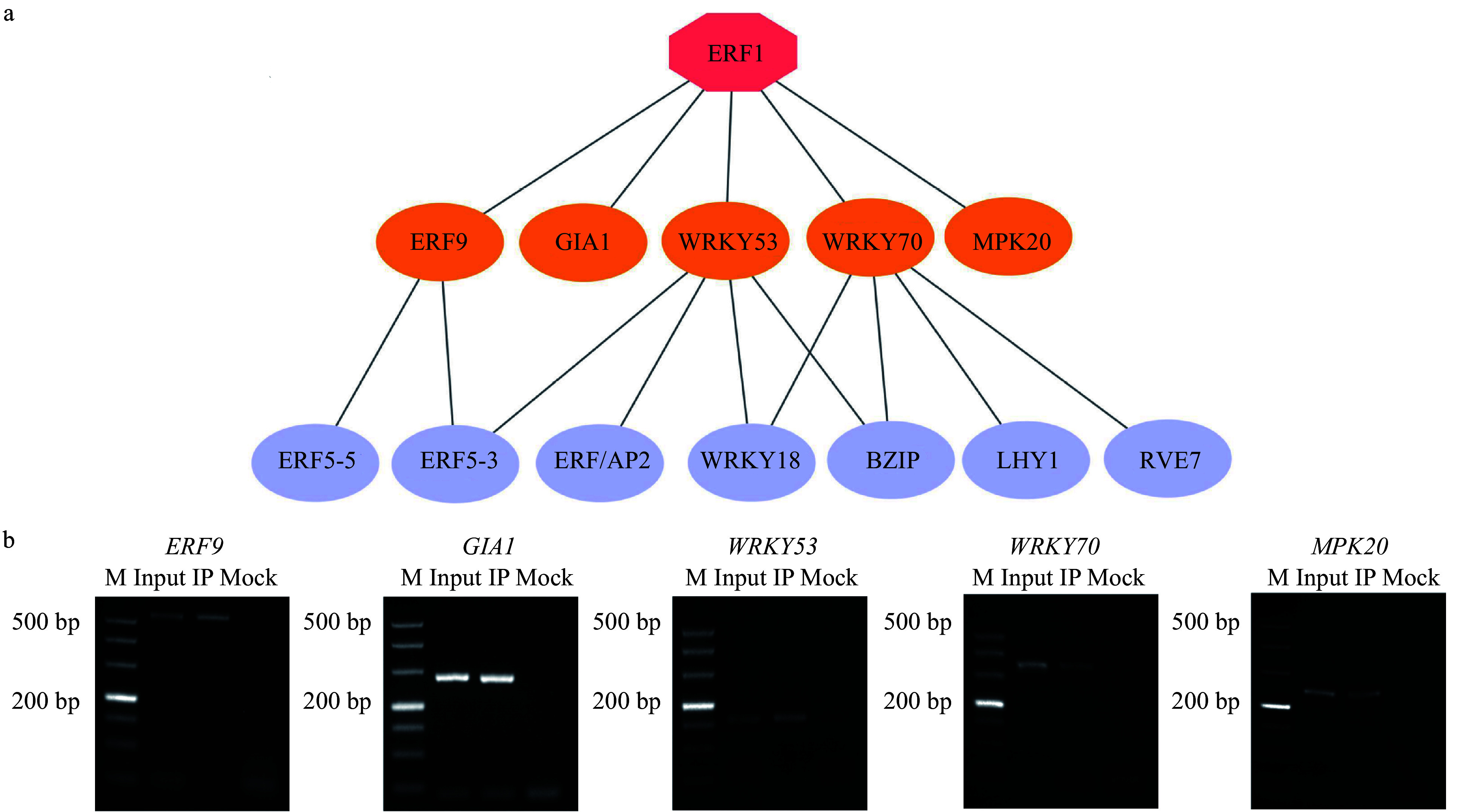
Construction of BplERF1-mediated multilayered gene regulatory network (ML-hGRN) in *Betula platyphylla*. (a) ML-hGRN-mediated by BplERF1 predicted using the Top-down GGM algorithm. Each orange oval represents a direct target gene of BplERF1. Each lilac oval represents an indirect gene of BplERF1. (b) ChIP-PCR analysis of the association of BplERF1 with its five putative target gene promoters in vivo using an anti-GFP tag antibody. M: DL500 Marker, the band is 500 bp, 400 bp, 300 bp, 200 bp, 150 bp, 100 bp, 50 bp from top to bottom.

We extracted 634 DEGs that were transcription factors and MAPK pathway genes, and then used the expression profiles of these DEGs and BplERF1 to build the BplERF1-mediated ML-hGRN using Top-down GGM algorithm^[27]^. The result was shown in Fig. 6a and Supplemental Table S2. In the ML-hGRN built, BplERF1 directly regulated five target genes, *ERF9*, *GIA1*, *WRKY53*, *WRKY70*, and *MPK20*. Using the TF-centered Y1H assay^[28]^, we found that BplERF1 could bind to the *cis*-acting element of WRKY71OS (TGAC) or MYBCORE (CNGTTR) (Table 2). We then performed ChIP-PCR to examine the binding of BplERF1 to these *cis*-elements, and the results were presented in Fig. 6b. These results indicated that BplERF1 directly binds to all five target genes predicted. All the five amplified promoter fragments of five genes harbored the motif of WRKY71OS (TGAC) and only the amplified promoter fragments of ERF9 harbored MYBCORE (CNGTTR) element.

**Table 2:** BplERF1-binding sites (*cis*-elements) identified by TF-centered Y1H method.

Sequences	Core sequences	Motif prediction
T**CAACAG**GAT A**GTCA**GCATA	CNGTTR TGAC	MYBCORE WRKY71OS

## DISCUSSION

Previous studies have shown that MAPK signaling pathway genes^[29,30]^ and the genes from several transcription factor families including ERF^[31]^, WRKY^[32]^, MYB^[33]^ and bHLH^[34]^, are actively involved in cold stress response and tolerance^[35]^. In this study, a varying number of genes were induced at different time points upon cold stress. We first identified a potential signaling pathway regulator, BplERF1, through gene association using ARACNE^[36]^, a pairwise gene association method that can recognize both linear and non-linear mutual dependency. However, pairwise correlation may not reflect causality though the data processing inequality method integrated in ARACNE can remove some weakest association. Our analysis suggested that BplERF1 potentially regulates the MAPK signaling pathway.

Although we do not know the regulatory directions in the TF-MAPK association network (Fig. 1a) the relationships recognized by ARACNE represent either a linear or nonlinear dependency. In this association network, as many as 17 ERF genes were shown to be associated with MAPK signaling pathway genes. Previous studies have demonstrated that ERF family members regulate plant cold response and tolerance. For example, overexpression *BpERF13* enhances cold tolerance in white birch through upregulating *CBF* genes and mitigating reactive oxygen species^[31]^; the suppression of Sl-ERF.B.3 in transgenic tomato plants reduces cell injury and enhances the tolerance against 14-d cold stress^[37]^; another study showed 12 and 19 AP2/ERF gene family members in *Zoysia japonica* are responsive to 2-h and 3-d cold treatments, respectively^[38]^. In addition, MPK pathway is shown to be involved in signaling transduction under cold stress. For instance, *A. thaliana* MEKK1 is phosphorylated via Ca^2+^ signaling as part of the cold stress response^[39]^. MPK3, MPK4, and MPK6 are rapidly activated after cold treatment, whereas the MKK4/5-MPK3/6 cascade negatively regulates the cold response by modulating ICE1 protein stability^[12]^. Moreover, ERF3 in rice positively regulates two MAPKs and two WRKY genes as well as concentrations of jasmonate (JA) and salicylate (SA)^[21]^. In our study, *MPK20* had a negative correlation with *BplERF1* (Fig. 1b). Given the fact that BplERF1 is a positive regulator of cold stress, MPK20 may, like *A. thaliana* MPK3 and MPK6, negatively regulate cold stress. In the BplERF1-mediated ML-hGRN we built (Fig. 6a), BplERF1 directly regulated *MPK20*, *ERF9*, *GIA1*, *WRKY53*, and *WRKY70*. All the orthologs of these five genes have been shown to play a contributing role in abiotic stress response and tolerance in other species^[40−45]^. In several grasses (*Brachypodium distachyon*), foxtail millet (*Setaria italica*), and sorghum (*Sorghum bicolor*), ERF9 and ERF2 are located in DNase I-hypersensitive sites (DHSs) and are also highly expressed in cold treated tissues^[46]^. *ERF9* is a key gene of the mannitol-responsive network^[47]^. *WRKY53* in* A. thaliana* negatively regulates drought tolerance^[48]^. Under cold stress, *WRKY70* in *A. thaliana* is negatively involved in drought responses^[49]^. *GIA1* expression is regulated by ABF3, which enhances salt stress tolerance in *A. thaliana*^[50]^.

Low temperature reduces the activities of enzymes^[51]^, affects the permeability of cell membranes^[52]^ and increases the accumulation of ROS^[53]^. In our study, *BplERF1-OE* lines showed increased POD and SOD activities, and reduced H_2_O_2_, ${\text O}_{\text 2}^{\text -} $ and MDA content, which indicates that overexpression of *BplERF1* can confer augmented cold tolerance of transgenic lines through increasing the scavenging reactive oxygen species.

It is worth mentioning that the *ERF1* gene in *A. thaliana* was reported to be highly expressed in the roots where it inhibits root elongation through upregulating the *ASA1* gene, the rate-limiting gene in the auxin biosynthesis pathway^[54]^. Transgenic lines overexpressing *A. thaliana ERF1* were more tolerant to drought and salt stress^[55]^. Overexpression of *HbERF-IXc5*, a *ERF1* orthologue in *Hevea brasiliensis*, enhances the tolerance to water deficit, cold and salt stresses^[56]^, and at the same time, boosts the development of root systems in *HbERF-IXc5* transgenic lines. In our study, *BplERF1* had the highest expression in the leaves rather than the roots. Although we noticed that GUS proteins were indeed accumulated to a higher concentration at the root tips (Fig. 2b), the root lengths of *BplERF1* overexpression transgenic lines had no significant changes as compared to those in the wild-type (Fig. 4); this indicates that BplERF1 may have evolved to regulate cold stress response and tolerance rather than growth in *B. platyphylla*. Since different plant species have experienced different environmental conditions and evolutionary trajectories, the distinct functions of orthologous TF genes in different species have frequently been observed. For example, *A. thaliana* MYC2 negatively regulates adventitious root (AR) formation^[57]^, while *Populus ussuriensis* PuMYC2 promotes AR formation^[58]^. PtaNAC1 in *Populus tremula x Populus alba* is predominantly expressed in roots and promotes lateral root growth under low nitrogen condition^[59]^ while NAC1 in *Medicago truncatula* does not have any obvious effects on lateral root formation^[60]^. These results indicate that orthologue genes in different species may have evolved to have different regulatory roles.

## CONCLUSION

We found that overexpression of *BplERF1* in *B. platyphylla* transgenic lines could significantly improve cold stress tolerance. Physiological assays of the overexpression transgenic lines revealed that *BplERF1* could not only increase activities of SOD and POD, but also reduce the content of electrolyte leakage rate, MDA, H_2_O_2_, and ${\text O}_{\text 2}^{\text -} $ in transgenic plants. In addition, TF-centered Y1H and ChIP-PCR experiments showed that BplERF1 protein bound to the promoters of *ERF9*, *GIA1*, *WRKY53*, *WRKY70* and *MPK20* through the WRKY71OS (TGAC) and MYBCORE (CNGTTR) element. Therefore, we conclude that *BplERF1* can be used to improve the antifreeze ability of plants because it could modulate the expression of multiple stress responsive and tolerance regulatory genes and increase the ROS-scavenging capacity of plants.

## MATERIALS AND METHODS

### Materials

Plants of wild type *B. platyphylla* were cultured in tissue bottles containing 1/2 MS + 0.02 mg/L NAA + 2% (w/v) sucrose medium, and placed in the tissue culture room set to 16/8-h light/dark cycles and an average temperature of 25 °C. Differentiation media containing WPM + 0.8 mg/L 6-BA + 0.02 mg/L NAA + 2% (w/v) sucrose, with pH being adjusted to 5.8−6.0, were used.

### RNA-seq cold treatment experiments

The two RNA-seq time-course data sets were generated from two-month-old *Betula platyphylla* seedlings after the 4 °C low temperature treatment. In the first time-course experiment, all the leaf samples were harvested at the same time after 0.5 h, 1 h, 1.5 h, 2 h, and 3 h cold treatment respectively, and the controls under room temperature (25 °C) were also harvested at the same times. The RNA-seq data were submitted to NCBI Bioproject (PRJNA727859). In the second time-course experiment, all samples of the leaves were harvested at the same time after 6 h, 12 h, 24 h, 2 d, 4 d, 7 d, and 14 d cold treatment respectively, and the controls were also harvested at the same times. The RNA-seq data were submitted to NCBI Bioproject (PRJNA285437)^[61]^. Two biological replicates were harvested from each time point in both experiments.

### Identification of DEGs of cold treatments

The RNA-seq data sets yielded from two cold treatment experiments were analyzed with the modified software package of *Pop's Pipe*^[26]^ to identify DEGs. The DEGs between a time interval were identified by comparing the data of a current time point with the previous time point.

### Construction of gene regulatory network

Algorithm for the Reconstruction of Accurate Cellular Networks (ARACNE)^[36]^ was used to construct an association network between the MAPK signaling pathway and differentially expressed transcription factors.

### Vector construction and plant transformation

Total RNA was isolated from *B. platyphylla* using the cetyltrimethylammonium bromide (CTAB) method^[62]^. Total RNA was reverse transcribed into cDNA using a Toyo Kit (TOYOBO ReverTra Ace® qPCR RT Master Mix with gDNA Remover, FSQ-301). The cDNA was used as a template for PCR amplification of the coding sequence (CDS) of *BplERF1*, and the CDS was cloned into the *pENTR-D* vector (Invitrogen) and eventually transferred into the binary vector *pGWB2* using LR-recombination (Invitrogen).

We designed a pair of primers with adaptors containing specific restriction sites, and used birch cDNA as a template for PCR amplification of *BplERF1*. The PCR products were cloned into the binary vector of *pBI121-GFP*, and obtained *pBI121-35S::BplERF1-GFP* (BplERF1-GFP fusing protein).

We used birch genomic DNA as a template for PCR amplification of *BplERF1* promoter sequence, and inserted the promoter sequence into the *pBI101-GUS* vector by the double enzyme digestion method and obtained the *pBI101-BplERF1pro::GUS* vector.

The three binary vectors, namely, *pGWB2-BplERF1, pBI121-BplERF1-GFP* and *pBI101-BplERF1pro::GUS* harboring* BplERF1* were then transformed into *Agrobacterium* strain EHA105 by freeze-thaw method^[63]^. The *B. platyphylla* transgenic lines were developed by the leaf disc method^[31]^. These three types of transgenic plants were used to verify the function of *BplERF1* gene, BplERF1's target genes via chromosome immunoprecipitation (ChIP) experiment, and the temporal and spatial expression of *BplERF1* gene, respectively. The primer sequences used are shown in Supplemental Table S3.

### Tissue-specific expression and subcellular localization of *BplERF1*

Total RNA was isolated from three tissues (roots, stems and leaves) of two-month-old wild type *B. platyphylla* plants using CTAB method. Total RNA was reverse transcribed into cDNA for RT-qPCR analysis. Real-time RT-PCR was performed with a Toyo SYBR qPCR Kit (TOYOBO SYBR qPCR Mix, QPS-201) using the primer sequences listed in Supplemental Table S3.

At the same time, the *pBI101-BplERF1pro::GUS* transgenic lines were stained with GUS reaction buffer^[64]^ overnight at 37 °C in the dark. After staining, plant material was cleared with absolute alcohol.

In order to localize BplERF1 proteins in the cells, the *pBI121-35S::BplERF1-GFP* plasmid was transferred into *Nicotiana benthamiana* epidermal cells using transient expression assay as described previously^[65]^. After the infection, the materials were placed at room temperature for 48 h before the fluorescence signals emerged. The fluorescence was observed and photographed under a confocal laser scanning microscope (LSM 700, Zeiss, Germany).

### Transformation and identification of *BplERF1* transgenic lines

The *pBI121-35S::BplERF1-GFP* and *pBI101-BplERF1pro::GUS* constructs were introduced into *Betula platyphylla* leaves using *Agrobacterium tumefaciens*-mediated transformation following the procedure as described previously^[31]^. The DNA was extracted from each putative transgenic line using a DNA extraction kit (TIANGEN, Beijing, China) for PCR assay. Meanwhile, the total RNA was reversely transcribed into cDNA, which was used for RT-qPCR assay to further validate transgenic lines. The putative transgenic lines with the correct sized *BplERF1* bands were considered to be true positive transgenic lines.

### Plant materials and cold stress treatments

Birch seedlings were planted in soil in the greenhouse under controlled conditions (16/8 h light/dark, 24 °C). After two months, the seedlings were placed in a refrigerator and treated at 4 °C overnight in the dark, and then −5 °C for 2 h. Following cold treatment, the plants were placed in the greenhouse for a 10-d recovery before the phenotypic assessment was conducted. Photos were taken immediately before and after cold treatment with a 10-d recovery.

Immediately after the freezing (−5 °C for 2 h) treatment as previously described, the activities of SOD and POD, the contents of H_2_O_2 _and ${\text O}_{\text 2}^{\text -} $ in cold treated plants, were determined with the SOD and POD microdetermination kits (Suzhou Comin biotechnology), respectively. MDA^[66]^ and electrolyte leakage rate^[67]^ were measured following the methods as described.

### TF-centered yeast one-hybrid (Y1H)

The TF-centered Y1H method^[28]^ was used to identify the binding *cis*-elements of BplERF1. First, *BplERF1* was constructed into the *pGADT7-Rec2* vector using homologous recombination, the *pGADT7-Rec2-BplERF1* plasmid and the *pHIS2* element library plasmid were then co-transferred into Y187 yeast using yeast transformation kit (the Yeastmaker^TM^ Yeast Transformation System 2, Takara), and cultured on SD/-His/-Leu/-Trp + 50 mM 3-Amino-1, 2, 4-triazole (3-AT) solid medium. Positive colonies were chosen and sequenced to identify candidate *cis*-element sequences.

### Construction of the BplERF1-mediated ML-hGRN

The BplERF1-mediated ML-hGRN was constructed using *BplERF1-OE* RNA-seq data (PRJNA722021). Raw read counts were harvested from the leaves of the wild-type birch and seven *BplERF1*-OE transgenic lines, two biological replicates, and the data were normalized using the TMM algorithm contained in the edgeR package. In order to study the regulatory relationships between BplERF1 and MAPK-pathway-targeted genes in the network, BplERF1-mediated ML-hGRN was constructed by using Top-down GGM algorithm^[27]^.

### ChIP experiments and ChIP-PCR

*pBI121-BplERF1-GFP* transgenic plants were harvested for ChIP experiments following a method described previously^[68]^ using an anti-GFP antibody. The precipitated DNA was used for ChIP-PCR as previously described^[31]^. We designed the primers that span the positions of WRKY71OS and MYBCORE motif in the promoter regions of the target genes, and used the precipitated DNA as a template for PCR amplification. The primer sequences used are shown in Supplemental Table S3.

### Statistical analysis

The Student’s *t* test and one-way analysis of variance (ANOVA) were used to test the significant differences. The thresholds for statistically significant differences and very significant difference were set to *p* < 0.05 and *p* < 0.01, respectively.

## SUPPLEMENTARY DATA

Supplementary data to this article can be found online.
