# Supplementary material for: A systems biology approach identifies a regulator, BplERF1, of cold tolerance in Betula platyphylla
Source: For Res (Fayettev). 2021 Jun 30;1:11. doi: 10.48130/FR-2021-0011 (PMC11524244; doi:10.48130/FR-2021-0011)
Supplement: Supplementary file 1 — Supplementary data to this article can be found online. [file FR-2021-0011-S1.zip › 10.48130_FR-2021-0011-Suppl-TableS2.pdf]

Supplemental Table 2 Inferred gene regulatory network-mediated by BplERF1 using Top

TF	symbol	description
Bpev01.c0343.g0032.m0001	ERF1	ethylene response factor 1
Bpev01.c0343.g0032.m0001	ERF1	ethylene response factor 1
Bpev01.c0343.g0032.m0001	ERF1	ethylene response factor 1
Bpev01.c0343.g0032.m0001	ERF1	ethylene response factor 1
Bpev01.c0343.g0032.m0001	ERF1	ethylene response factor 1
Bpev01.c0505.g0019.m0001	WRKY53	WRKY DNA-binding protein 53
Bpev01.c0505.g0019.m0001	WRKY53	WRKY DNA-binding protein 53
Bpev01.c0505.g0019.m0001	WRKY53	WRKY DNA-binding protein 53
Bpev01.c0505.g0019.m0001	WRKY53	WRKY DNA-binding protein 53
Bpev01.c0224.g0049.m0001	WRKY70	WRKY DNA-binding protein 70
Bpev01.c0224.g0049.m0001	WRKY70	WRKY DNA-binding protein 70
Bpev01.c0224.g0049.m0001	WRKY70	WRKY DNA-binding protein 70
Bpev01.c0224.g0049.m0001	WRKY70	WRKY DNA-binding protein 70
Bpev01.c0500.g0009.m0001	ERF9	erf domain protein 9
Bpev01.c0500.g0009.m0001	ERF9	erf domain protein 9
Bpev01.c0500.g0009.m0001	ERF9	erf domain protein 9

p-down GGM algorithm

target gene	target_symbol
Bpev01.c0224.g0049.m0001	WRKY70
Bpev01.c0327.g0058.m0001	GIA1
Bpev01.c0500.g0009.m0001	ERF9
Bpev01.c0505.g0019.m0001	WRKY53
Bpev01.c1138.g0008.m0001	MPK20
Bpev01.c0051.g0164.m0001	ERF/AP2
Bpev01.c0089.g0075.m0001	BZIP
Bpev01.c0511.g0003.m0001	WRKY18
Bpev01.c1161.g0014.m0001	ERF5-3
Bpev01.c0089.g0075.m0001	BZIP
Bpev01.c1464.g0001.m0001	RVE7
Bpev01.c0001.g0063.m0001	LHY1
Bpev01.c0511.g0003.m0001	WRKY18
Bpev01.c0343.g0032.m0001	ERF1
Bpev01.c1161.g0014.m0001	ERF5-3
Bpev01.c1161.g0016.m0001	ERF5-5

terget_description	number_interfere	Layer
WRKY DNA-binding protein 70	1	Layer1
GROWTH-INSENSITIVITY TO ABA	1	Layer1
erf domain protein 9	1	Layer1
---	1	Layer1
---	1	Layer1
Integrase-type DNA-binding superfa	1	Layer2
Basic-leucine zipper (bZIP) transcrip	1	Layer2
WRKY DNA-binding protein 18	1	Layer2
ethylene responsive element binding	1	Layer2
Basic-leucine zipper (bZIP) transcrip	2	Layer2
REVEILLE 7	2	Layer2
LATE ELONGATED HYPOCOTYL 1	1	Layer2
WRKY DNA-binding protein 18	1	Layer2
ethylene response factor 1	2	Layer2
ethylene responsive element binding	1	Layer2
ethylene responsive element binding	1	Layer2
