# Supplementary material for: A systems biology approach identifies a regulator, BplERF1, of cold tolerance in Betula platyphylla
Source: For Res (Fayettev). 2021 Jun 30;1:11. doi: 10.48130/FR-2021-0011 (PMC11524244; doi:10.48130/FR-2021-0011)
Supplement: Supplementary file 1 — Supplementary data to this article can be found online. [file FR-2021-0011-S1.zip › 10.48130_FR-2021-0011-Suppl-TableS3.pdf]

Supplemental Table 3. Primer pairs used in this study for gene cloning, vector construction, transgenic line validation, RT-qPCR and ChIP-PCR

Primer sequences for gene cloning

Name	5' primers	3' primers
BplERF1	CACCATGGTTTTAGCCTCCCAAAG	TCAAGTTGTTTCTATTTTTGGAAAACCTAGAAC

Primer sequences for vector construction

Vector	5' primers	3' primers
pBI121-*BplERF1*	CGCTCTAGAATGGTTTTAGCCTCCCAAAGCGAG	CGCGTCGACCAGTTGTTTCTATTTTTGGAAAACC
pBI101-*BplERF1*	CCAAGCTTTTGGTTTCTGAGCTGCAATGATC	CCCCCGGGGGCTTTTTTTTCTTTCTTTT
pGADT7-Rec2-*BplERF1*	GAGTGGCCATTATGGCCCATGGTTTTAGCCTCCCAAAGCGAGC	TCTAGAGGCCGAGGCGGCCGACATGTCAAGTTGTTTCTATTTTTGGAAAAC

Primer sequences for transgenic line validation

Name	5' primers	3' primers
DNA detection primer	GTTCATTTCATTTGGAGAGAACACG	GGAAATTCGAGCTCTAAGCGCTGT

Primer sequences for RT-qPCR

Name	5' primers	3' primers
tubulin	TCAACCGCCTTGTCTCTCAGG	TGGCTCGAATGCACTGTTGG
UBQ	TCTGACAGGGAAGACCATA	TCAATTAGAGCTGACCACC
BplERF1	ACGAGAACGATCCACAAGACATG	CATTGATTTGGTGCCTTTGC

Primer sequences for ChIP-PCR

Name	5' primers	3' primers
ERF9	CTTCAGCCTCTCGTAGGGGTC	GTTCGTCCTGACCAGCCGTGTC
GIA1	CCGTTGCTTGCTGATTGCTTGG	GACCTATTCCAACCAATGAGCTC
WRKY53	GGTCTCCTCGTCTCCAAGCTC	GCTTCTTCGCAACCTCTTCACTC
WRKY70	GTTGCAGGTTTGAGTTGTGTTG	GGATTTGAATCCCTTTGAATGCTAAC
MPK20	GGTCTTAAACTTCAACGCGC	TGTTGGCATTTGATAAGAGCACC
