# Supplementary material for: A systems biology approach identifies a regulator, BplERF1, of cold tolerance in Betula platyphylla
Source: For Res (Fayettev). 2021 Jun 30;1:11. doi: 10.48130/FR-2021-0011 (PMC11524244; doi:10.48130/FR-2021-0011)
Supplement: Supplementary file 1 — Supplementary data to this article can be found online. [file 10.48130_FR-2021-0011-Suppl-TableS1.pdf › 10.48130_FR-2021-0011-Suppl-TableS1.pdf]

	Cold treatments<br><br>(Wild type at 4°C)	Up regulated genes	Down regulated genes	Total DEGs
1	1h versus 0.5h	2193	2256	4449
2	1.5h versus 1h	813	517	1330
3	2 h versus 1.5	9	58	67
4	2.5 h versus 2h	3	2	5
5	3h versus 2.5h	10	2	12
6	24 versus 6h	2573	2569	5142
7	2d versus 24h	179	240	419
8	4d versus 2d	41	51	92
9	7d versus 4d	0	0	0
10	14d versus 7d	134	311	445

Supplemental Table 1 Differentially expressed genes from different time points in comparison \

GeneID	Comparison_1_FC	Comparison_1	Comparison_1	DEG/nonDEG
Bpev01.c0015.g0194.m0002	0.830929589	0.00692796	0.04689633	DEG
Bpev01.c2169.g0001.m0001	1.340382227	0.00051536	0.0061687	DEG
Bpev01.c0016.g0033.m0001	-1.26548097	3.67E-05	0.00071737	DEG
Bpev01.c1072.g0013.m0001	1.248698783	0.00040195	0.00503654	DEG
Bpev01.c0452.g0009.m0001	-1.042186427	0.00143661	0.01383456	DEG
Bpev01.c0992.g0003.m0001	-1.984493219	0.00022026	0.0030716	DEG
Bpev01.c0394.g0029.m0001	1.907454589	0.00053676	0.00636651	DEG
Bpev01.c0957.g0013.m0001	-1.03920676	0.0008762	0.00935935	DEG
Bpev01.c0129.g0003.m0002	1.503726652	0.00013433	0.00207807	DEG
Bpev01.c0555.g0029.m0001	-2.226280023	2.74E-10	2.83E-08	DEG
Bpev01.c1382.g0018.m0001	-1.097373582	0.00743952	0.04943838	DEG
Bpev01.c0024.g0043.m0001	3.836966888	8.50E-14	1.80E-11	DEG
Bpev01.c0668.g0020.m0001	-1.755073057	0.00085234	0.00916438	DEG
Bpev01.c1666.g0003.m0001	1.278811007	0.00107486	0.01092259	DEG
Bpev01.c0126.g0053.m0001	1.84084968	0.00737325	0.04908661	DEG
Bpev01.c0717.g0033.m0001	-1.298653239	0.00588109	0.04136998	DEG
Bpev01.c0488.g0021.m0001	-1.806008627	0.00011684	0.00184962	DEG
Bpev01.c0115.g0107.m0001	3.506193361	1.21E-09	1.06E-07	DEG
Bpev01.c0157.g0015.m0001	6.581472326	2.21E-29	8.13E-26	DEG
Bpev01.c1539.g0004.m0001	1.076243804	0.00405757	0.03136011	DEG
Bpev01.c1610.g0010.m0001	1.891354372	1.91E-05	0.00042022	DEG
Bpev01.c1859.g0001.m0001	4.292395494	1.59E-08	1.00E-06	DEG
Bpev01.c0617.g0016.m0001	1.225222316	7.96E-05	0.00134599	DEG
Bpev01.c0774.g0013.m0002	-2.506091733	0.0018721	0.01720661	DEG
Bpev01.c0051.g0180.m0001	-2.648529129	0.00021119	0.00297196	DEG
Bpev01.c0043.g0025.m0001	0.976906211	0.00221702	0.01958204	DEG
Bpev01.c0857.g0015.m0001	-0.916602546	0.0053932	0.03887618	DEG
Bpev01.c0224.g0002.m0001	-2.488806688	2.01E-14	4.96E-12	DEG
Bpev01.c0169.g0043.m0001	1.561912831	4.35E-06	0.00012123	DEG
Bpev01.c0327.g0060.m0001	-3.04056551	2.78E-08	1.62E-06	DEG
Bpev01.c0389.g0051.m0001	-1.209105643	0.00591397	0.04151203	DEG
Bpev01.c0154.g0009.m0001	4.114776262	0.00237619	0.02064717	DEG
Bpev01.c1226.g0002.m0001	2.046088949	0.00031062	0.00405336	DEG
Bpev01.c0018.g0027.m0001	1.467418978	0.00046146	0.00563452	DEG
Bpev01.c0162.g0058.m0001	-1.054488765	0.00410332	0.03163901	DEG
Bpev01.c0224.g0010.m0001	-1.421143866	1.40E-05	0.0003278	DEG
Bpev01.c1537.g0011.m0001	-1.751046087	0.00336499	0.02722232	DEG
Bpev01.c0038.g0089.m0001	-2.69063297	0.0008905	0.00947432	DEG
Bpev01.c0621.g0018.m0001	-1.274864443	0.00118041	0.01179573	DEG
Bpev01.c1064.g0004.m0001	4.042546791	7.32E-07	2.73E-05	DEG
Bpev01.c1202.g0019.m0001	-2.198594929	0.00019769	0.00282097	DEG

Bpev01.c0426.g0010.m0001	1.59812884	0.00604086	0.04216139	DEG
Bpev01.c0374.g0019.m0001	-2.210942612	1.89E-06	6.06E-05	DEG
Bpev01.c2812.g0001.m0001	2.871426122	0.00177674	0.01647937	DEG
Bpev01.c1653.g0009.m0002	1.801699778	7.35E-07	2.73E-05	DEG
Bpev01.c2330.g0007.m0001	0.972193172	0.00644673	0.0444566	DEG
Bpev01.c1202.g0001.m0001	1.741482965	4.54E-05	0.00085427	DEG
Bpev01.c0215.g0043.m0001	-1.358403132	0.00030394	0.0039856	DEG
Bpev01.c1972.g0007.m0003	0.84811728	0.00723386	0.0484766	DEG
Bpev01.c0281.g0013.m0002	1.915398931	1.39E-08	8.89E-07	DEG
Bpev01.c0317.g0001.m0003	2.703069901	1.25E-07	5.91E-06	DEG
Bpev01.c1579.g0012.m0001	0.907785707	0.00499954	0.03663056	DEG
Bpev01.c1733.g0002.m0001	-1.3895217	9.96E-05	0.00161302	DEG
Bpev01.c0118.g0021.m0001	-1.119300911	0.0005458	0.00643058	DEG
Bpev01.c1579.g0004.m0001	-1.262699947	0.00241307	0.02091206	DEG
Bpev01.c0451.g0001.m0001	0.920201418	0.00354585	0.02837551	DEG
Bpev01.c0082.g0014.m0001	1.820549051	3.52E-05	0.00069457	DEG
Bpev01.c0660.g0012.m0001	4.546801173	2.79E-08	1.62E-06	DEG
Bpev01.c0141.g0032.m0001	1.927954809	0.00578902	0.04081988	DEG
Bpev01.c1370.g0002.m0001	1.75989173	0.00022215	0.00308918	DEG
Bpev01.c0292.g0015.m0001	1.517691054	0.00080053	0.00867063	DEG
Bpev01.c0862.g0006.m0001	1.826113696	5.69E-06	0.00015277	DEG
Bpev01.c0154.g0011.m0001	2.929152042	1.11E-05	0.00026869	DEG
Bpev01.c0402.g0015.m0001	1.306066542	0.00220057	0.01946005	DEG
Bpev01.c0843.g0004.m0001	-1.129791399	0.00638487	0.04410235	DEG
Bpev01.c0807.g0006.m0001	2.032467146	0.00025669	0.0034743	DEG
Bpev01.c0237.g0005.m0001	1.168405473	0.00417262	0.03196401	DEG
Bpev01.c0299.g0035.m0002	1.361726872	1.87E-05	0.00041378	DEG
Bpev01.c0597.g0040.m0001	-1.935347881	2.00E-05	0.00043605	DEG
Bpev01.c0516.g0006.m0001	-2.463352179	0.00227675	0.0199837	DEG
Bpev01.c0889.g0013.m0001	-2.551853146	0.00033392	0.00430496	DEG
Bpev01.c0154.g0029.m0001	1.057502226	0.00406365	0.03139893	DEG
Bpev01.c0288.g0015.m0001	-1.104498301	0.00464207	0.03471118	DEG
Bpev01.c0038.g0134.m0001	-1.197704207	0.00010028	0.00162118	DEG
Bpev01.c0283.g0017.m0001	1.716449258	9.87E-05	0.00160052	DEG
Bpev01.c1085.g0015.m0001	-3.00612893	8.24E-12	1.15E-09	DEG
Bpev01.c0826.g0014.m0001	-1.659976506	1.04E-06	3.70E-05	DEG
Bpev01.c0161.g0053.m0002	1.248330003	0.00011297	0.00179762	DEG
Bpev01.c0396.g0011.m0001	1.195015046	0.00069392	0.00777038	DEG
Bpev01.c0415.g0034.m0001	-0.922794188	0.00628552	0.04354893	DEG
Bpev01.c0257.g0025.m0001	1.150232003	0.0016961	0.01586528	DEG
Bpev01.c1160.g0005.m0001	1.410272149	0.00090336	0.00955584	DEG
Bpev01.c0044.g0057.m0001	1.246818238	0.00011632	0.00184305	DEG
Bpev01.c1891.g0001.m0001	-1.087533544	0.00029972	0.00394563	DEG

Bpev01.c0294.g0013.m0001	1.382802129	6.23E-06	0.00016499	DEG
Bpev01.c1327.g0005.m0001	0.880091658	0.00409743	0.03160188	DEG
Bpev01.c0112.g0014.m0001	1.271245241	0.00237066	0.02061704	DEG
Bpev01.c0104.g0010.m0001	2.609981976	2.32E-05	0.00049153	DEG
Bpev01.c0278.g0007.m0001	-2.057224437	0.00031464	0.0040967	DEG
Bpev01.c0135.g0082.m0001	-1.762502064	1.49E-05	0.00034496	DEG
Bpev01.c0510.g0008.m0001	-2.69267062	7.02E-05	0.00121168	DEG
Bpev01.c2051.g0002.m0001	-0.89232593	0.00630534	0.0436657	DEG
Bpev01.c0190.g0068.m0001	6.39395589	0.0044206	0.0334211	DEG
Bpev01.c0080.g0106.m0001	2.108453488	4.68E-05	0.00087562	DEG
Bpev01.c0843.g0017.m0001	2.414812856	4.32E-06	0.0001209	DEG
Bpev01.c0469.g0018.m0001	0.955981559	0.00358053	0.02858888	DEG
Bpev01.c0476.g0001.m0001	-2.081055734	0.00161787	0.01528022	DEG
Bpev01.c1938.g0014.m0001	0.976729915	0.00235428	0.02049308	DEG
Bpev01.c0568.g0029.m0001	-1.064516548	0.00366695	0.02913375	DEG
Bpev01.c0425.g0033.m0001	1.130133389	0.0020591	0.01846416	DEG
Bpev01.c0449.g0018.m0001	-1.044225204	0.00227419	0.01996716	DEG
Bpev01.c0989.g0003.m0002	0.925362689	0.00669183	0.04568149	DEG
Bpev01.c3050.g0001.m0001	1.660192887	0.00472948	0.0352038	DEG
Bpev01.c0105.g0017.m0001	-2.228825006	7.60E-08	3.87E-06	DEG
Bpev01.c0115.g0113.m0001	1.216962661	0.0004449	0.00547776	DEG
Bpev01.c0177.g0032.m0001	3.825495352	7.40E-05	0.00126209	DEG
Bpev01.c0015.g0194.m0001	0.828748763	0.00703521	0.04742607	DEG
Bpev01.c0857.g0011.m0001	1.162663277	0.00019307	0.00277391	DEG
Bpev01.c0564.g0008.m0001	-1.662387984	1.13E-07	5.37E-06	DEG
Bpev01.c0158.g0013.m0001	3.458672292	5.77E-19	3.40E-16	DEG
Bpev01.c1699.g0013.m0001	-1.493088434	0.00222412	0.01960819	DEG
Bpev01.c0357.g0022.m0001	0.938986497	0.00387174	0.03028971	DEG
Bpev01.c0038.g0077.m0001	-1.437646029	0.00012096	0.00190223	DEG
Bpev01.c2211.g0001.m0001	3.010299449	0.00200213	0.01805779	DEG
Bpev01.c0044.g0053.m0001	-0.93334991	0.0041474	0.03184538	DEG
Bpev01.c0454.g0031.m0001	-1.261498128	0.00067612	0.00763197	DEG
Bpev01.c0036.g0058.m0001	2.930166747	2.35E-09	1.87E-07	DEG
Bpev01.c0480.g0065.m0001	-2.113195234	0.00569062	0.04033688	DEG
Bpev01.c0146.g0008.m0001	1.611037433	0.00189635	0.01736988	DEG
Bpev01.c0157.g0017.m0001	1.268583929	4.17E-05	0.00079647	DEG
Bpev01.c0281.g0056.m0001	-0.899794412	0.00369828	0.02926709	DEG
Bpev01.c0167.g0023.m0001	-1.556653218	0.00106516	0.01084743	DEG
Bpev01.c0945.g0002.m0001	1.123886812	0.00022231	0.00308992	DEG
Bpev01.c0732.g0002.m0001	-2.551583166	1.29E-08	8.42E-07	DEG
Bpev01.c1189.g0014.m0001	-2.368026913	9.31E-14	1.93E-11	DEG
Bpev01.c2879.g0001.m0001	1.244624256	0.00021484	0.00301178	DEG
Bpev01.c0249.g0002.m0001	-3.565933683	2.08E-05	0.00045057	DEG

Bpev01.c2937.g0003.m0001	-1.877943602	3.96E-05	0.00076115	DEG
Bpev01.c1021.g0001.m0001	2.370908713	6.30E-05	0.00110866	DEG
Bpev01.c1947.g0003.m0001	0.851649924	0.00524474	0.03802017	DEG
Bpev01.c0956.g0006.m0001	1.484239101	0.00278065	0.0234622	DEG
Bpev01.c1101.g0001.m0001	1.24224153	0.00070795	0.00791238	DEG
Bpev01.c0940.g0001.m0001	4.00340805	1.34E-13	2.74E-11	DEG
Bpev01.c2059.g0003.m0001	-1.176674665	0.00072578	0.00803846	DEG
Bpev01.c0624.g0025.m0002	-1.192813536	0.00032682	0.00423102	DEG
Bpev01.c0088.g0011.m0001	1.380911123	0.0001763	0.00257683	DEG
Bpev01.c1112.g0016.m0001	1.697237895	8.67E-08	4.30E-06	DEG
Bpev01.c0146.g0011.m0001	-2.224737181	3.64E-06	0.00010557	DEG
Bpev01.c0278.g0001.m0001	-2.94095871	0.00582591	0.04106033	DEG
Bpev01.c0706.g0010.m0001	2.899862992	6.84E-05	0.00118777	DEG
Bpev01.c0364.g0032.m0001	4.789336626	2.64E-18	1.44E-15	DEG
Bpev01.c0052.g0167.m0001	-1.132433215	0.00018269	0.002652	DEG
Bpev01.c0298.g0013.m0001	-1.364497394	0.0006406	0.00730618	DEG
Bpev01.c0996.g0008.m0001	1.070437958	0.00489136	0.03608941	DEG
Bpev01.c1398.g0007.m0001	-1.526640861	1.13E-05	0.0002721	DEG
Bpev01.c3105.g0003.m0001	2.136417725	0.00024553	0.00335039	DEG
Bpev01.c0984.g0030.m0001	-1.544826729	0.00133682	0.01304434	DEG
Bpev01.c1272.g0023.m0001	-2.506959946	1.47E-06	4.89E-05	DEG
Bpev01.c0188.g0002.m0001	1.369327011	0.00033814	0.0043488	DEG
Bpev01.c0602.g0009.m0001	-1.632771485	1.17E-05	0.00027951	DEG
Bpev01.c1242.g0005.m0001	0.894539823	0.00592808	0.0415894	DEG
Bpev01.c1343.g0002.m0001	7.584973775	6.44E-05	0.00113007	DEG
Bpev01.c0605.g0010.m0001	3.909434054	1.79E-15	5.81E-13	DEG
Bpev01.c0068.g0007.m0001	-1.453227139	3.99E-06	0.00011305	DEG
Bpev01.c0549.g0006.m0001	-3.3194413	0.0001841	0.00266977	DEG
Bpev01.c0000.g0157.m0002	5.106391173	7.30E-13	1.27E-10	DEG
Bpev01.c1346.g0008.m0001	-1.192549096	0.00103892	0.01063447	DEG
Bpev01.c1161.g0004.m0001	-0.892182067	0.00703454	0.04742607	DEG
Bpev01.c0101.g0018.m0001	-0.995289112	0.00298901	0.02479532	DEG
Bpev01.c0146.g0007.m0001	1.355576596	0.00200031	0.01804691	DEG
Bpev01.c1615.g0006.m0001	-2.271021325	5.08E-05	0.00093542	DEG
Bpev01.c0798.g0003.m0001	3.972999464	1.01E-05	0.00024808	DEG
Bpev01.c0710.g0012.m0001	-1.352022083	0.00015807	0.00236217	DEG
Bpev01.c0022.g0111.m0001	1.361074739	0.00024686	0.0033645	DEG
Bpev01.c0101.g0047.m0001	-1.907875364	0.00361859	0.02885368	DEG
Bpev01.c1911.g0002.m0001	2.109264519	0.00593458	0.04161698	DEG
Bpev01.c0457.g0004.m0001	1.710414926	0.00193845	0.01765106	DEG
Bpev01.c1092.g0018.m0001	-2.466599146	0.00286876	0.02402069	DEG
Bpev01.c0919.g0021.m0001	-1.263652942	0.00454266	0.03408902	DEG
Bpev01.c0192.g0008.m0001	-1.341496388	6.91E-05	0.00119785	DEG

Bpev01.c1922.g0001.m0001	-1.695502207	1.47E-05	0.00034002	DEG
Bpev01.c2228.g0004.m0002	-1.349299173	9.61E-05	0.00156729	DEG
Bpev01.c0402.g0037.m0001	-1.654151899	7.41E-05	0.00126392	DEG
Bpev01.c0015.g0123.m0001	-2.557961637	5.22E-06	0.00014215	DEG
Bpev01.c0237.g0054.m0001	-1.818326792	3.29E-08	1.88E-06	DEG
Bpev01.c0159.g0037.m0001	-3.648448186	1.22E-06	4.24E-05	DEG
Bpev01.c0367.g0017.m0001	0.850152246	0.00500593	0.03665911	DEG
Bpev01.c0000.g0106.m0001	1.24863259	0.00496943	0.03650079	DEG
Bpev01.c0053.g0039.m0001	8.277692249	0.00034403	0.00441841	DEG
Bpev01.c0552.g0010.m0001	-1.423506026	3.35E-06	9.84E-05	DEG
Bpev01.c1239.g0009.m0001	-0.957256481	0.0049906	0.03658321	DEG
Bpev01.c1714.g0001.m0001	-2.119838598	3.69E-06	0.0001065	DEG
Bpev01.c2063.g0005.m0001	-1.464896999	0.00010244	0.00164705	DEG
Bpev01.c0161.g0002.m0001	-2.937466847	0.00010064	0.00162365	DEG
Bpev01.c0622.g0002.m0002	3.22467242	2.66E-13	5.15E-11	DEG
Bpev01.c0401.g0026.m0001	-1.05779377	0.00241973	0.02095745	DEG
Bpev01.c0275.g0067.m0001	-1.902629878	0.0005645	0.00659058	DEG
Bpev01.c0768.g0013.m0001	0.957044703	0.00755351	0.0499904	DEG
Bpev01.c0710.g0003.m0001	1.727049769	4.37E-08	2.42E-06	DEG
Bpev01.c1581.g0003.m0002	-3.228212399	5.97E-17	2.47E-14	DEG
Bpev01.c1531.g0001.m0001	-1.808650415	0.0070124	0.04731563	DEG
Bpev01.c1448.g0002.m0001	1.84471813	1.79E-05	0.00040005	DEG
Bpev01.c0022.g0177.m0001	-1.068624866	0.00225273	0.01982604	DEG
Bpev01.c0362.g0009.m0001	1.627620751	1.78E-05	0.00039769	DEG
Bpev01.c0142.g0044.m0001	-1.86752591	2.79E-05	0.00057402	DEG
Bpev01.c0016.g0061.m0001	4.02807818	2.27E-14	5.47E-12	DEG
Bpev01.c0286.g0011.m0001	1.744264532	1.40E-05	0.00032634	DEG
Bpev01.c0857.g0012.m0001	-2.137400257	4.69E-10	4.54E-08	DEG
Bpev01.c0285.g0006.m0001	-1.839124584	0.00063466	0.00724735	DEG
Bpev01.c0210.g0009.m0001	1.132093297	0.00258756	0.02213753	DEG
Bpev01.c1456.g0001.m0001	-2.010990852	0.00271732	0.02304005	DEG
Bpev01.c0051.g0106.m0001	-2.25990446	5.75E-06	0.00015434	DEG
Bpev01.c0679.g0008.m0001	1.535847542	0.00059011	0.00683139	DEG
Bpev01.c3068.g0001.m0001	-1.893621581	0.00153506	0.01461538	DEG
Bpev01.c0016.g0173.m0001	-1.171962697	0.00064637	0.00735832	DEG
Bpev01.c0327.g0068.m0001	-1.198232063	0.00056913	0.00663524	DEG
Bpev01.c0575.g0009.m0001	2.895550736	1.48E-06	4.90E-05	DEG
Bpev01.c2577.g0002.m0001	-1.119635695	0.00139568	0.01348895	DEG
Bpev01.c0570.g0009.m0001	1.517423904	3.50E-06	0.00010225	DEG
Bpev01.c0396.g0028.m0001	-1.247525715	0.00165026	0.01554123	DEG
Bpev01.c3201.g0002.m0001	1.098424907	0.00087128	0.00932164	DEG
Bpev01.c0707.g0001.m0001	-1.522481569	0.00716988	0.04817944	DEG
Bpev01.c1404.g0004.m0001	2.810801983	1.05E-06	3.71E-05	DEG

Bpev01.c0800.g0026.m0001	0.918606391	0.00321274	0.02626712	DEG
Bpev01.c1475.g0004.m0001	-1.046393094	0.0011562	0.01159706	DEG
Bpev01.c1747.g0010.m0001	-2.310459543	3.02E-05	0.00061179	DEG
Bpev01.c0053.g0030.m0001	-2.380975889	0.00017881	0.00260463	DEG
Bpev01.c0281.g0067.m0001	3.645577762	7.85E-09	5.47E-07	DEG
Bpev01.c0000.g0140.m0001	1.317549476	0.00017758	0.00258929	DEG
Bpev01.c1577.g0027.m0001	-1.568747565	0.00090029	0.0095371	DEG
Bpev01.c0578.g0005.m0001	4.013103275	0.00034914	0.00447279	DEG
Bpev01.c0389.g0003.m0001	-1.682243401	0.00013985	0.00214199	DEG
Bpev01.c0167.g0007.m0001	1.06075629	0.00040618	0.00507746	DEG
Bpev01.c0027.g0056.m0001	1.179464704	0.00555431	0.03964764	DEG
Bpev01.c1338.g0018.m0001	-1.379254859	0.00621424	0.04314646	DEG
Bpev01.c1552.g0003.m0001	1.295432258	0.0032732	0.02666289	DEG
Bpev01.c0298.g0026.m0001	-1.71018135	6.96E-07	2.61E-05	DEG
Bpev01.c2361.g0001.m0001	-2.025377865	0.0001983	0.00282705	DEG
Bpev01.c0953.g0004.m0001	2.795846597	3.52E-06	0.00010266	DEG
Bpev01.c2582.g0001.m0001	5.458974114	1.04E-05	0.00025541	DEG
Bpev01.c1439.g0010.m0001	-2.092427832	1.93E-05	0.00042447	DEG
Bpev01.c1671.g0001.m0001	2.145007314	0.00477795	0.0354839	DEG
Bpev01.c0863.g0013.m0001	0.975883978	0.00233788	0.02039863	DEG
Bpev01.c0052.g0170.m0001	-3.798139954	0.00224641	0.01978221	DEG
Bpev01.c0726.g0018.m0001	1.660889683	5.39E-06	0.00014576	DEG
Bpev01.c0134.g0075.m0001	-2.12221622	0.00036842	0.00468705	DEG
Bpev01.c0261.g0078.m0001	-2.600120413	0.00475039	0.03532379	DEG
Bpev01.c0962.g0026.m0001	1.140169747	0.00026168	0.0035321	DEG
Bpev01.c0764.g0007.m0001	4.515573303	2.71E-15	8.15E-13	DEG
Bpev01.c1913.g0005.m0001	4.058317347	0.00226887	0.01993383	DEG
Bpev01.c0916.g0001.m0001	2.18293146	1.70E-07	7.69E-06	DEG
Bpev01.c0660.g0016.m0001	2.279571451	0.00409515	0.03159253	DEG
Bpev01.c1047.g0013.m0001	-1.178467087	0.00059732	0.00690402	DEG
Bpev01.c0029.g0039.m0001	-2.080246511	0.00030652	0.00401225	DEG
Bpev01.c0791.g0008.m0001	1.812032046	0.00015296	0.0022974	DEG
Bpev01.c2087.g0003.m0001	3.178846012	4.38E-06	0.00012212	DEG
Bpev01.c0803.g0015.m0001	-1.03564311	0.0058914	0.04143262	DEG
Bpev01.c2029.g0004.m0001	1.903152016	0.00052359	0.0062529	DEG
Bpev01.c0716.g0009.m0001	2.599283453	4.95E-10	4.72E-08	DEG
Bpev01.c1736.g0002.m0001	2.638869639	1.35E-08	8.77E-07	DEG
Bpev01.c0391.g0004.m0001	2.029524312	0.00020876	0.00294778	DEG
Bpev01.c1308.g0002.m0001	2.204655956	6.75E-07	2.54E-05	DEG
Bpev01.c0088.g0129.m0001	5.329057415	2.97E-28	7.29E-25	DEG
Bpev01.c0023.g0013.m0001	1.895856617	6.97E-05	0.00120569	DEG
Bpev01.c0000.g0178.m0001	1.14465287	0.00050105	0.00604034	DEG
Bpev01.c0066.g0036.m0001	-1.640302198	0.00150854	0.01441661	DEG

Bpev01.c1135.g0010.m0001	-1.911740962	0.00471568	0.03511886	DEG
Bpev01.c0015.g0094.m0001	-1.603695481	0.00014403	0.00219014	DEG
Bpev01.c1390.g0012.m0001	1.940922124	1.25E-06	4.32E-05	DEG
Bpev01.c0082.g0103.m0001	3.962973308	0.00102265	0.01050076	DEG
Bpev01.c0135.g0076.m0001	-1.221870995	0.00293534	0.02446622	DEG
Bpev01.c0570.g0019.m0001	1.70602769	1.36E-06	4.60E-05	DEG
Bpev01.c0189.g0065.m0001	-2.017429642	0.00031136	0.0040612	DEG
Bpev01.c0558.g0009.m0001	-1.757070613	1.21E-06	4.19E-05	DEG
Bpev01.c0422.g0011.m0001	1.312638527	0.00360794	0.0287765	DEG
Bpev01.c2500.g0001.m0001	1.264946654	0.00173233	0.01613858	DEG
Bpev01.c1710.g0001.m0001	1.623696055	0.00053353	0.00634371	DEG
Bpev01.c0932.g0005.m0001	-2.165040364	0.00010503	0.00168692	DEG
Bpev01.c0379.g0002.m0001	-1.102479898	0.00052918	0.00630456	DEG
Bpev01.c2446.g0001.m0001	1.923502373	0.00027234	0.00365096	DEG
Bpev01.c0301.g0021.m0001	-2.034707433	0.00055207	0.00649057	DEG
Bpev01.c0866.g0022.m0001	-1.712124409	0.00232059	0.02028979	DEG
Bpev01.c1893.g0003.m0001	2.50343515	4.24E-09	3.13E-07	DEG
Bpev01.c0660.g0014.m0001	4.244759652	1.30E-05	0.00030921	DEG
Bpev01.c0024.g0009.m0004	1.291427907	0.0007885	0.00857506	DEG
Bpev01.c1187.g0006.m0001	-2.715862993	2.06E-14	5.06E-12	DEG
Bpev01.c3074.g0002.m0001	-2.523143707	0.00100114	0.01034487	DEG
Bpev01.c0136.g0016.m0001	-2.605936111	3.30E-05	0.00065998	DEG
Bpev01.c0000.g0014.m0001	1.972329488	8.00E-08	4.06E-06	DEG
Bpev01.c0817.g0033.m0001	-2.681545841	1.77E-14	4.44E-12	DEG
Bpev01.c0212.g0010.m0001	1.091132147	0.00122115	0.01211646	DEG
Bpev01.c1782.g0007.m0001	-2.04676913	0.00470786	0.03508729	DEG
Bpev01.c1632.g0002.m0001	-2.157059973	9.86E-07	3.55E-05	DEG
Bpev01.c1074.g0005.m0001	2.0366896	2.80E-06	8.45E-05	DEG
Bpev01.c0698.g0007.m0001	2.823907724	0.00570267	0.04037004	DEG
Bpev01.c0552.g0003.m0001	1.32341842	1.38E-05	0.00032343	DEG
Bpev01.c2454.g0003.m0001	-1.812140808	0.00133777	0.01304696	DEG
Bpev01.c0015.g0201.m0001	-2.314902495	1.65E-05	0.00037446	DEG
Bpev01.c2242.g0004.m0001	3.463436542	0.00040125	0.00503294	DEG
Bpev01.c0489.g0009.m0001	1.785878652	6.80E-05	0.00118186	DEG
Bpev01.c0496.g0009.m0001	-2.869786137	0.0010153	0.01044729	DEG
Bpev01.c1084.g0005.m0001	-1.694073571	1.04E-07	5.01E-06	DEG
Bpev01.c0861.g0002.m0001	-1.929086153	0.00027177	0.00364665	DEG
Bpev01.c1031.g0016.m0001	-0.895578537	0.00485902	0.03593185	DEG
Bpev01.c1093.g0003.m0001	2.125957534	8.19E-08	4.13E-06	DEG
Bpev01.c0038.g0140.m0001	-1.544436295	4.51E-07	1.78E-05	DEG
Bpev01.c0030.g0007.m0001	-1.868139139	0.00242146	0.0209601	DEG
Bpev01.c0135.g0077.m0001	-2.197240855	0.00535825	0.03864321	DEG
Bpev01.c0352.g0015.m0001	-0.821048877	0.0068177	0.04634178	DEG

Bpev01.c0739.g0024.m0001	-0.927767889	0.00554483	0.03961995	DEG
Bpev01.c0015.g0105.m0001	0.994495767	0.00216161	0.01918466	DEG
Bpev01.c1239.g0010.m0001	-1.127524685	0.00603782	0.0421591	DEG
Bpev01.c0853.g0002.m0001	6.340077135	0.00565897	0.04017216	DEG
Bpev01.c1335.g0007.m0001	-1.349713007	2.16E-05	0.00046515	DEG
Bpev01.c0015.g0027.m0001	-0.974782487	0.00448736	0.03376016	DEG
Bpev01.c0343.g0006.m0001	0.937897449	0.00668951	0.04568149	DEG
Bpev01.c0349.g0036.m0001	2.000622946	0.00015107	0.0022761	DEG
Bpev01.c0053.g0012.m0001	1.701555156	4.08E-06	0.00011501	DEG
Bpev01.c0301.g0033.m0001	-2.61615788	8.55E-14	1.80E-11	DEG
Bpev01.c0191.g0010.m0001	3.804424233	6.86E-18	3.54E-15	DEG
Bpev01.c0044.g0102.m0001	-1.842688005	0.00119348	0.011894	DEG
Bpev01.c0358.g0007.m0001	-1.777270723	3.15E-05	0.00063229	DEG
Bpev01.c0129.g0107.m0001	3.546014264	1.68E-25	2.47E-22	DEG
Bpev01.c0602.g0002.m0001	-0.874375246	0.00752297	0.04986457	DEG
Bpev01.c0275.g0052.m0001	1.213458969	0.00045653	0.00559519	DEG
Bpev01.c0213.g0054.m0001	-4.248572055	0.00086415	0.00926765	DEG
Bpev01.c0138.g0006.m0001	1.094194927	0.00267702	0.02274633	DEG
Bpev01.c1202.g0045.m0001	2.090732428	0.00011689	0.00184962	DEG
Bpev01.c0640.g0020.m0001	-2.261404576	1.39E-08	8.89E-07	DEG
Bpev01.c0027.g0054.m0001	-1.539974256	4.53E-06	0.00012587	DEG
Bpev01.c0075.g0005.m0001	3.12162907	0.00208604	0.01867162	DEG
Bpev01.c2315.g0007.m0001	-3.302977035	2.08E-06	6.51E-05	DEG
Bpev01.c1040.g0034.m0001	1.601892466	7.20E-06	0.00018712	DEG
Bpev01.c0051.g0142.m0001	-2.465615348	1.05E-09	9.36E-08	DEG
Bpev01.c0082.g0037.m0001	-1.225593186	0.00065921	0.0074755	DEG
Bpev01.c0557.g0036.m0001	6.335960122	0.00669773	0.04570574	DEG
Bpev01.c0473.g0013.m0001	3.692975138	2.11E-09	1.70E-07	DEG
Bpev01.c1019.g0003.m0001	1.271118259	0.00013566	0.00209199	DEG
Bpev01.c0556.g0018.m0001	3.891555533	1.44E-06	4.81E-05	DEG
Bpev01.c0045.g0076.m0001	4.707564685	0.00043858	0.00541814	DEG
Bpev01.c0280.g0015.m0001	1.472170238	0.00076806	0.00839309	DEG
Bpev01.c4626.g0001.m0001	2.208738964	0.00689056	0.04676148	DEG
Bpev01.c0118.g0029.m0001	-2.093855895	0.00340589	0.02750027	DEG
Bpev01.c0038.g0054.m0001	1.428902128	0.00363302	0.02893737	DEG
Bpev01.c0203.g0003.m0001	2.274947744	0.00401587	0.03114416	DEG
Bpev01.c0902.g0015.m0001	-1.786864432	1.62E-08	1.01E-06	DEG
Bpev01.c0510.g0006.m0001	1.689978393	5.49E-05	0.00099343	DEG
Bpev01.c0473.g0029.m0001	-1.999968512	0.0001037	0.00166644	DEG
Bpev01.c1356.g0004.m0001	-2.707885651	0.00371257	0.02934862	DEG
Bpev01.c0717.g0010.m0001	0.88691241	0.00557028	0.03971504	DEG
Bpev01.c0470.g0001.m0001	-7.346819575	0.00160441	0.01517747	DEG
Bpev01.c0389.g0066.m0001	-1.037179433	0.00099916	0.0103353	DEG

Bpev01.c0190.g0043.m0001	1.853670886	0.00134493	0.01310602	DEG
Bpev01.c0604.g0016.m0001	6.216283966	0.00665659	0.04556229	DEG
Bpev01.c0154.g0021.m0001	-1.931050684	0.00273609	0.02315258	DEG
Bpev01.c0154.g0013.m0001	1.280485131	0.00650594	0.04472875	DEG
Bpev01.c0115.g0060.m0001	1.667228529	0.00069036	0.00774228	DEG
Bpev01.c0477.g0004.m0001	-1.513898092	4.27E-05	0.00081082	DEG
Bpev01.c0327.g0073.m0001	-1.433823122	0.00020549	0.0029084	DEG
Bpev01.c1281.g0005.m0001	1.038156914	0.00202902	0.01823892	DEG
Bpev01.c0996.g0005.m0001	-1.402748458	0.0019862	0.0179685	DEG
Bpev01.c1709.g0004.m0001	-1.089219759	0.00319139	0.02613051	DEG
Bpev01.c0046.g0009.m0001	1.668156155	7.38E-05	0.00126076	DEG
Bpev01.c0115.g0086.m0001	-2.274755334	0.00346391	0.02786941	DEG
Bpev01.c0154.g0079.m0001	1.845430467	0.00059065	0.00683226	DEG
Bpev01.c0426.g0020.m0001	-1.527371062	4.21E-05	0.00080132	DEG
Bpev01.c0706.g0007.m0001	2.479048175	0.00055295	0.00649568	DEG
Bpev01.c0496.g0022.m0001	-1.331830549	3.68E-05	0.0007193	DEG
Bpev01.c0974.g0025.m0001	1.632971602	7.76E-06	0.00019925	DEG
Bpev01.c0154.g0088.m0001	-4.160492931	0.00021457	0.00300944	DEG
Bpev01.c0083.g0024.m0002	1.939170059	0.0016392	0.01545198	DEG
Bpev01.c0134.g0018.m0001	-2.10220779	2.26E-10	2.39E-08	DEG
Bpev01.c0688.g0008.m0001	-1.263691976	0.00352851	0.02825697	DEG
Bpev01.c0667.g0025.m0001	-1.527128072	0.00077529	0.00845634	DEG
Bpev01.c0253.g0008.m0001	-1.96165409	4.29E-08	2.38E-06	DEG
Bpev01.c2423.g0003.m0001	-1.652042469	1.01E-05	0.0002482	DEG
Bpev01.c1350.g0010.m0001	-3.015682091	0.0012218	0.01211876	DEG
Bpev01.c1238.g0009.m0001	1.433314164	0.00017567	0.0025704	DEG
Bpev01.c0095.g0046.m0001	-1.586704478	0.00076633	0.00839087	DEG
Bpev01.c0745.g0003.m0001	1.378454111	9.01E-05	0.00148768	DEG
Bpev01.c0727.g0003.m0001	-1.731499319	0.00011198	0.00178476	DEG
Bpev01.c0145.g0051.m0001	-1.737517337	6.78E-06	0.00017768	DEG
Bpev01.c1476.g0003.m0001	1.241770927	7.17E-05	0.00123107	DEG
Bpev01.c0062.g0014.m0001	-1.086932181	0.00124444	0.01230189	DEG
Bpev01.c1283.g0002.m0002	-1.227011206	0.00025378	0.00344447	DEG
Bpev01.c1349.g0005.m0001	1.832991044	3.08E-08	1.77E-06	DEG
Bpev01.c0384.g0002.m0001	-1.456596611	3.96E-05	0.00076115	DEG
Bpev01.c0127.g0065.m0001	-2.172309438	3.67E-06	0.00010607	DEG
Bpev01.c0286.g0020.m0001	1.198749094	0.00076192	0.00835072	DEG
Bpev01.c1069.g0005.m0001	-1.353248043	0.00087383	0.0093375	DEG
Bpev01.c0640.g0016.m0001	1.258864377	0.00011398	0.00180992	DEG
Bpev01.c0000.g0055.m0001	-2.132181324	0.00665025	0.04554004	DEG
Bpev01.c0121.g0005.m0001	1.00097188	0.00256222	0.02196542	DEG
Bpev01.c0038.g0101.m0001	-1.93850791	0.00025351	0.00344233	DEG
Bpev01.c0045.g0072.m0002	2.905771158	3.96E-15	1.14E-12	DEG

Bpev01.c0101.g0040.m0001	0.944258204	0.00445796	0.03359901	DEG
Bpev01.c4481.g0001.m0001	-2.740023075	0.00479772	0.03558584	DEG
Bpev01.c1206.g0016.m0001	-1.803724921	8.11E-09	5.64E-07	DEG
Bpev01.c0349.g0034.m0001	-1.28831984	0.00513098	0.03737033	DEG
Bpev01.c0411.g0003.m0001	0.971321005	0.0043282	0.03287358	DEG
Bpev01.c1067.g0004.m0001	2.751772085	0.00056933	0.00663524	DEG
Bpev01.c2306.g0001.m0001	-0.984295005	0.00247683	0.02137654	DEG
Bpev01.c0566.g0050.m0001	2.105735495	9.41E-06	0.00023407	DEG
Bpev01.c0170.g0035.m0001	-1.090092691	0.0024589	0.02123424	DEG
Bpev01.c0171.g0027.m0001	1.512631281	0.00287888	0.02408396	DEG
Bpev01.c0015.g0113.m0001	1.285884149	2.21E-05	0.00047355	DEG
Bpev01.c2470.g0005.m0001	0.923751235	0.00466825	0.03485207	DEG
Bpev01.c0281.g0012.m0001	1.600780467	4.09E-06	0.00011505	DEG
Bpev01.c0515.g0005.m0001	-0.921734091	0.00716528	0.0481595	DEG
Bpev01.c0327.g0021.m0001	1.317982096	4.10E-05	0.00078496	DEG
Bpev01.c0158.g0014.m0001	-2.279203184	1.84E-10	1.99E-08	DEG
Bpev01.c1040.g0032.m0001	1.730831737	0.00179356	0.01661439	DEG
Bpev01.c0964.g0011.m0001	-0.97853549	0.00624772	0.04333801	DEG
Bpev01.c0261.g0041.m0001	-1.373694594	0.0002855	0.00379627	DEG
Bpev01.c0055.g0024.m0001	-0.882433599	0.00648044	0.04463152	DEG
Bpev01.c0650.g0008.m0001	-0.881593034	0.00650141	0.04471844	DEG
Bpev01.c1962.g0002.m0001	2.396524571	1.10E-07	5.24E-06	DEG
Bpev01.c1938.g0004.m0001	-1.900238716	0.0008926	0.00949319	DEG
Bpev01.c0062.g0076.m0001	-2.16209246	0.00070242	0.00785957	DEG
Bpev01.c0633.g0002.m0001	1.507972182	7.07E-05	0.00121798	DEG
Bpev01.c0884.g0005.m0001	0.909155827	0.00405712	0.03136011	DEG
Bpev01.c1115.g0008.m0001	-1.287438624	0.0013863	0.01341599	DEG
Bpev01.c0166.g0027.m0001	1.192691433	0.001329	0.01298521	DEG
Bpev01.c0067.g0005.m0001	1.995493886	0.00077125	0.00842478	DEG
Bpev01.c0038.g0059.m0001	-3.215055663	0.0001403	0.00214567	DEG
Bpev01.c0215.g0026.m0001	-1.692708442	0.00346082	0.02785975	DEG
Bpev01.c0101.g0010.m0001	0.899854331	0.00543054	0.03906882	DEG
Bpev01.c0575.g0023.m0001	0.87767902	0.00391963	0.03058306	DEG
Bpev01.c0089.g0052.m0001	2.554497346	2.98E-12	4.53E-10	DEG
Bpev01.c0096.g0001.m0001	-0.939697624	0.00216795	0.01922935	DEG
Bpev01.c0445.g0002.m0001	1.231272011	0.00401502	0.03114416	DEG
Bpev01.c1338.g0010.m0001	1.469813435	0.00055353	0.0065	DEG
Bpev01.c0214.g0051.m0001	-1.212786451	0.00013325	0.00206352	DEG
Bpev01.c2473.g0001.m0001	-1.37057639	0.00637634	0.04406407	DEG
Bpev01.c1563.g0005.m0001	1.443057555	0.00053619	0.00636355	DEG
Bpev01.c0349.g0037.m0001	-6.513671484	0.00329322	0.02678153	DEG
Bpev01.c0281.g0043.m0001	-2.049714339	4.54E-05	0.00085427	DEG
Bpev01.c0473.g0011.m0001	2.221048004	1.98E-08	1.20E-06	DEG

Bpev01.c0665.g0017.m0001	-2.68313025	0.00145211	0.01396561	DEG
Bpev01.c2410.g0002.m0001	1.873272128	1.41E-06	4.73E-05	DEG
Bpev01.c0515.g0004.m0001	1.428649008	5.06E-05	0.0009332	DEG
Bpev01.c0191.g0006.m0001	0.970695862	0.00175455	0.01632515	DEG
Bpev01.c0212.g0007.m0001	3.541045216	1.97E-10	2.10E-08	DEG
Bpev01.c0828.g0010.m0001	-1.091925524	0.00119142	0.01187747	DEG
Bpev01.c0419.g0022.m0001	-0.917617907	0.00412128	0.03172761	DEG
Bpev01.c0127.g0108.m0001	-2.142668447	3.06E-07	1.27E-05	DEG
Bpev01.c2530.g0002.m0001	-1.63083361	0.00654507	0.04494525	DEG
Bpev01.c1465.g0010.m0001	-1.422587681	0.00215234	0.01911973	DEG
Bpev01.c0910.g0004.m0001	-1.418097208	0.00202471	0.0182113	DEG
Bpev01.c0106.g0010.m0001	2.129915958	9.68E-05	0.00157369	DEG
Bpev01.c1040.g0039.m0001	-1.773094671	0.00521319	0.0378474	DEG
Bpev01.c0278.g0044.m0001	2.723034902	3.78E-08	2.12E-06	DEG
Bpev01.c0015.g0034.m0001	-1.117062465	0.00076713	0.00839087	DEG
Bpev01.c0427.g0019.m0001	-2.643720751	0.00444231	0.03354117	DEG
Bpev01.c0547.g0013.m0001	-0.941597871	0.00452585	0.03400622	DEG
Bpev01.c0870.g0001.m0001	4.826579531	6.21E-15	1.72E-12	DEG
Bpev01.c0053.g0025.m0001	-0.898041921	0.00537836	0.03877875	DEG
Bpev01.c0019.g0005.m0001	-2.385496907	1.70E-06	5.51E-05	DEG
Bpev01.c0462.g0003.m0001	1.973414544	2.26E-09	1.82E-07	DEG
Bpev01.c0344.g0039.m0001	-1.854663026	0.00263022	0.02244376	DEG
Bpev01.c0210.g0048.m0001	-1.565427441	2.34E-06	7.22E-05	DEG
Bpev01.c0127.g0071.m0001	-1.339109469	0.0037008	0.02927919	DEG
Bpev01.c0001.g0015.m0001	-1.528915157	3.72E-06	0.0001069	DEG
Bpev01.c0223.g0008.m0001	-2.193708702	0.00196348	0.01783494	DEG
Bpev01.c0951.g0001.m0001	-3.719814474	2.28E-09	1.83E-07	DEG
Bpev01.c1283.g0005.m0001	2.057271067	2.51E-09	1.99E-07	DEG
Bpev01.c0640.g0001.m0001	1.017685991	0.00313716	0.02577589	DEG
Bpev01.c1353.g0001.m0001	2.361712091	0.00016222	0.00241197	DEG
Bpev01.c0088.g0027.m0001	2.213483945	1.47E-08	9.33E-07	DEG
Bpev01.c0189.g0012.m0001	-1.693977403	9.41E-08	4.58E-06	DEG
Bpev01.c0606.g0023.m0001	-4.082894223	6.51E-05	0.00113928	DEG
Bpev01.c0169.g0007.m0001	-1.583262197	3.31E-05	0.00066206	DEG
Bpev01.c0272.g0001.m0001	2.534364886	5.64E-08	3.01E-06	DEG
Bpev01.c3211.g0006.m0001	-1.242886856	5.36E-05	0.00097566	DEG
Bpev01.c0127.g0100.m0002	-1.184279648	0.00075744	0.00831407	DEG
Bpev01.c1227.g0005.m0001	1.556840962	0.0013077	0.01282816	DEG
Bpev01.c1302.g0003.m0001	2.201928454	2.08E-06	6.53E-05	DEG
Bpev01.c0444.g0011.m0001	2.993061503	2.07E-12	3.29E-10	DEG
Bpev01.c0537.g0004.m0001	-1.246691097	0.00736512	0.04905803	DEG
Bpev01.c1189.g0018.m0001	-2.526862765	0.00071746	0.00798841	DEG
Bpev01.c0066.g0006.m0001	1.008430394	0.00458853	0.03438945	DEG

Bpev01.c0051.g0184.m0002	1.101271607	0.00094929	0.00993461	DEG
Bpev01.c0862.g0023.m0001	5.850829516	4.37E-27	8.57E-24	DEG
Bpev01.c1910.g0003.m0001	1.930044022	5.23E-06	0.00014215	DEG
Bpev01.c0190.g0084.m0001	1.901740035	1.28E-06	4.39E-05	DEG
Bpev01.c2136.g0004.m0001	-1.342719549	2.07E-05	0.00044794	DEG
Bpev01.c0188.g0037.m0001	2.31502568	2.26E-08	1.36E-06	DEG
Bpev01.c0066.g0046.m0001	1.106419002	0.0015702	0.01489438	DEG
Bpev01.c0051.g0120.m0001	-1.849079212	2.76E-08	1.61E-06	DEG
Bpev01.c0015.g0085.m0001	-2.788905606	4.61E-18	2.42E-15	DEG
Bpev01.c0082.g0027.m0001	1.073685551	0.00664034	0.04550395	DEG
Bpev01.c0154.g0008.m0001	4.017979719	5.20E-28	1.18E-24	DEG
Bpev01.c0038.g0149.m0001	-2.527532955	0.00269364	0.02285243	DEG
Bpev01.c0847.g0007.m0001	-2.589273071	3.72E-05	0.00072498	DEG
Bpev01.c0739.g0003.m0001	2.777884543	5.50E-12	7.86E-10	DEG
Bpev01.c0205.g0006.m0001	-0.951483052	0.00211591	0.01884725	DEG
Bpev01.c0134.g0139.m0001	1.105854216	0.00193335	0.01761554	DEG
Bpev01.c0027.g0123.m0001	-2.342709573	0.00322148	0.0263235	DEG
Bpev01.c0281.g0040.m0001	-1.09064193	0.00186345	0.01714855	DEG
Bpev01.c0488.g0031.m0001	2.516432384	0.00066596	0.00753002	DEG
Bpev01.c1543.g0004.m0001	-3.962463752	0.00058342	0.00677793	DEG
Bpev01.c0531.g0031.m0001	1.398627924	6.45E-05	0.00113007	DEG
Bpev01.c0495.g0005.m0001	-2.529937633	0.00029495	0.00389726	DEG
Bpev01.c0019.g0001.m0001	1.814018859	7.37E-06	0.00019081	DEG
Bpev01.c0037.g0017.m0001	0.831136315	0.006745	0.04595369	DEG
Bpev01.c2362.g0001.m0001	5.466548011	5.37E-12	7.71E-10	DEG
Bpev01.c1200.g0002.m0001	2.589873103	1.61E-08	1.01E-06	DEG
Bpev01.c0015.g0010.m0001	1.187947356	0.00089659	0.00950823	DEG
Bpev01.c1459.g0002.m0001	-1.18726851	0.0005454	0.00643008	DEG
Bpev01.c0275.g0035.m0001	2.035763791	1.57E-06	5.14E-05	DEG
Bpev01.c0505.g0015.m0001	-1.685183947	6.14E-05	0.001088	DEG
Bpev01.c0740.g0002.m0001	2.340358028	1.07E-08	7.20E-07	DEG
Bpev01.c0843.g0011.m0001	-1.036769715	0.00204034	0.01833504	DEG
Bpev01.c0092.g0020.m0001	-1.915737803	0.00046119	0.0056336	DEG
Bpev01.c1456.g0019.m0001	-2.798177066	1.33E-06	4.53E-05	DEG
Bpev01.c0051.g0059.m0001	1.624777659	0.00725163	0.04857357	DEG
Bpev01.c0094.g0045.m0001	1.292641471	7.98E-05	0.00134672	DEG
Bpev01.c0429.g0015.m0001	-2.518382451	4.04E-09	3.00E-07	DEG
Bpev01.c0389.g0004.m0001	-1.757531251	5.51E-05	0.00099562	DEG
Bpev01.c0274.g0066.m0001	-2.394432922	1.89E-11	2.41E-09	DEG
Bpev01.c0493.g0001.m0001	2.457947984	0.00045702	0.00559661	DEG
Bpev01.c0213.g0023.m0001	-1.324769594	0.00397528	0.03091898	DEG
Bpev01.c1653.g0001.m0001	1.570649767	0.00235069	0.02048001	DEG
Bpev01.c0157.g0034.m0001	-1.427471109	0.00037523	0.00476136	DEG

Bpev01.c0245.g0032.m0001	2.58646164	1.60E-07	7.29E-06	DEG
Bpev01.c1517.g0001.m0002	1.642678127	0.0012691	0.01249536	DEG
Bpev01.c0752.g0004.m0001	1.225066998	0.00096943	0.01008807	DEG
Bpev01.c1259.g0014.m0001	-1.4384442	0.0026464	0.02252961	DEG
Bpev01.c2336.g0001.m0001	1.908692967	0.00414691	0.03184538	DEG
Bpev01.c0215.g0016.m0002	1.442936671	0.00021058	0.0029661	DEG
Bpev01.c0578.g0010.m0001	1.79274045	2.89E-06	8.66E-05	DEG
Bpev01.c1378.g0009.m0001	-1.732129495	4.12E-05	0.00078807	DEG
Bpev01.c0082.g0091.m0001	2.006883419	3.43E-09	2.62E-07	DEG
Bpev01.c1721.g0008.m0001	3.901379164	2.04E-12	3.27E-10	DEG
Bpev01.c0050.g0103.m0001	4.072789351	0.00016539	0.00245044	DEG
Bpev01.c0387.g0010.m0001	-2.216256594	5.63E-07	2.17E-05	DEG
Bpev01.c0862.g0003.m0001	4.2124949	1.72E-06	5.57E-05	DEG
Bpev01.c0154.g0092.m0001	2.36288981	0.00037582	0.00476549	DEG
Bpev01.c0001.g0057.m0001	-2.154336711	2.02E-10	2.15E-08	DEG
Bpev01.c0286.g0019.m0001	1.101251709	0.00694448	0.0469758	DEG
Bpev01.c0907.g0001.m0001	-1.314213206	0.00024359	0.00333849	DEG
Bpev01.c2093.g0006.m0001	1.392266285	0.00210191	0.01877371	DEG
Bpev01.c0015.g0255.m0001	1.060081859	0.00098973	0.01025577	DEG
Bpev01.c0027.g0027.m0001	-2.025124972	0.00053376	0.00634371	DEG
Bpev01.c0368.g0001.m0001	3.212523724	1.62E-18	9.18E-16	DEG
Bpev01.c2707.g0004.m0001	1.052396459	0.00750212	0.04977571	DEG
Bpev01.c0051.g0037.m0001	1.254561381	0.00321301	0.02626712	DEG
Bpev01.c0478.g0008.m0001	-1.563445443	0.00606131	0.0422841	DEG
Bpev01.c1202.g0004.m0001	3.415686871	1.62E-06	5.28E-05	DEG
Bpev01.c0938.g0001.m0001	-4.46117523	0.00520655	0.03780848	DEG
Bpev01.c1134.g0003.m0001	-0.965685228	0.00213734	0.01900365	DEG
Bpev01.c0764.g0004.m0001	6.684355183	0.00493479	0.03630068	DEG
Bpev01.c0383.g0018.m0001	-1.245704972	0.0008714	0.00932164	DEG
Bpev01.c0447.g0017.m0001	0.983823654	0.00200735	0.01809938	DEG
Bpev01.c2385.g0002.m0001	1.126376037	0.00124898	0.01233849	DEG
Bpev01.c0549.g0001.m0001	-2.069933687	5.51E-05	0.00099492	DEG
Bpev01.c0237.g0045.m0001	-2.194027767	2.00E-05	0.00043518	DEG
Bpev01.c0403.g0037.m0001	1.136854189	0.00733749	0.04894954	DEG
Bpev01.c1289.g0001.m0001	-1.300106128	5.44E-05	0.00098754	DEG
Bpev01.c1763.g0006.m0001	-3.698633806	0.00282039	0.02370239	DEG
Bpev01.c1649.g0008.m0001	-2.270020431	8.32E-08	4.18E-06	DEG
Bpev01.c0992.g0020.m0001	1.411885368	0.000163	0.00241979	DEG
Bpev01.c0990.g0007.m0001	2.230295719	2.91E-07	1.21E-05	DEG
Bpev01.c0332.g0002.m0001	-2.038593669	8.46E-07	3.11E-05	DEG
Bpev01.c0333.g0001.m0001	2.348909546	1.10E-08	7.36E-07	DEG
Bpev01.c0080.g0065.m0001	-1.476958017	0.00031895	0.00414545	DEG
Bpev01.c0962.g0002.m0001	1.400108615	5.08E-05	0.00093542	DEG

Bpev01.c0576.g0003.m0001	2.073783215	0.00168257	0.01578494	DEG
Bpev01.c0467.g0013.m0001	0.94700824	0.00543823	0.03909549	DEG
Bpev01.c1378.g0036.m0001	-1.854628239	0.00056436	0.00659058	DEG
Bpev01.c0934.g0002.m0001	1.357799068	0.00718399	0.04824119	DEG
Bpev01.c0136.g0018.m0001	1.014299235	0.00331781	0.02693683	DEG
Bpev01.c1339.g0005.m0001	0.989610181	0.00137627	0.01335843	DEG
Bpev01.c0079.g0001.m0001	3.862279846	9.76E-09	6.65E-07	DEG
Bpev01.c0294.g0010.m0001	-2.125157291	2.86E-07	1.20E-05	DEG
Bpev01.c0304.g0029.m0001	4.339740264	2.00E-16	7.55E-14	DEG
Bpev01.c0327.g0041.m0001	1.670243557	9.07E-07	3.29E-05	DEG
Bpev01.c0224.g0008.m0001	1.352366927	1.63E-05	0.00037099	DEG
Bpev01.c1262.g0001.m0001	1.225425096	0.00021589	0.00302363	DEG
Bpev01.c1088.g0003.m0001	4.554183154	4.83E-06	0.00013257	DEG
Bpev01.c0169.g0024.m0001	-1.086445858	0.00072777	0.00805748	DEG
Bpev01.c0114.g0065.m0001	-2.083796519	0.00306432	0.02529739	DEG
Bpev01.c0051.g0037.m0002	1.546576147	0.00060501	0.00697378	DEG
Bpev01.c0038.g0053.m0001	1.006794657	0.00589755	0.04144189	DEG
Bpev01.c0142.g0090.m0001	-2.196855694	2.17E-05	0.00046551	DEG
Bpev01.c0804.g0004.m0001	1.330355787	0.0003806	0.00481912	DEG
Bpev01.c0547.g0004.m0001	3.426998336	0.00142555	0.01373709	DEG
Bpev01.c0215.g0015.m0001	2.789860369	0.00341267	0.02753992	DEG
Bpev01.c0759.g0001.m0001	1.9679728	0.0069147	0.04684859	DEG
Bpev01.c1030.g0009.m0001	-1.197533623	0.00093688	0.00983622	DEG
Bpev01.c1161.g0006.m0001	1.611219611	0.00073173	0.00808826	DEG
Bpev01.c1185.g0015.m0001	-1.871894709	1.56E-05	0.00035753	DEG
Bpev01.c0157.g0019.m0001	-2.284326983	2.07E-08	1.25E-06	DEG
Bpev01.c0120.g0008.m0001	-2.062278799	0.0018675	0.01717501	DEG
Bpev01.c0137.g0019.m0001	2.13575347	6.39E-06	0.00016814	DEG
Bpev01.c1170.g0013.m0001	-5.119056963	7.77E-30	3.81E-26	DEG
Bpev01.c0335.g0038.m0001	-2.114543103	0.0071832	0.04824119	DEG
Bpev01.c0051.g0185.m0002	-1.505339522	1.99E-06	6.31E-05	DEG
Bpev01.c0671.g0015.m0001	-0.950630218	0.00318459	0.0260927	DEG
Bpev01.c0044.g0095.m0001	4.522371647	2.11E-15	6.68E-13	DEG
Bpev01.c0145.g0066.m0001	-1.755911671	5.31E-06	0.00014409	DEG
Bpev01.c0357.g0017.m0001	-1.987182823	2.42E-05	0.00050717	DEG
Bpev01.c0327.g0036.m0001	-2.397590004	0.00019535	0.00279717	DEG
Bpev01.c0027.g0069.m0001	-1.475021442	7.25E-06	0.00018793	DEG
Bpev01.c0443.g0026.m0001	1.727814776	5.86E-05	0.00105156	DEG
Bpev01.c1289.g0003.m0001	-4.226614965	0.0004087	0.00510468	DEG
Bpev01.c0145.g0021.m0001	1.600632653	0.00017329	0.00254314	DEG
Bpev01.c0105.g0002.m0001	-1.542315633	0.00058665	0.00680871	DEG
Bpev01.c0050.g0011.m0001	-3.120832845	0.00030997	0.00404842	DEG
Bpev01.c2532.g0001.m0001	2.989888413	0.00078734	0.00856562	DEG

Bpev01.c1180.g0004.m0001	1.828363937	1.97E-07	8.68E-06	DEG
Bpev01.c1708.g0006.m0001	0.956514424	0.00312205	0.0257019	DEG
Bpev01.c0624.g0009.m0001	2.602199343	0.00016836	0.00248681	DEG
Bpev01.c1382.g0002.m0001	-1.174523805	0.00051344	0.00615432	DEG
Bpev01.c0555.g0027.m0001	1.680683545	1.60E-05	0.00036429	DEG
Bpev01.c2291.g0001.m0001	2.867512916	0.00112899	0.01139402	DEG
Bpev01.c1577.g0009.m0001	-1.904197479	6.92E-07	2.60E-05	DEG
Bpev01.c1390.g0002.m0001	-1.544298569	3.86E-06	0.00011021	DEG
Bpev01.c2085.g0001.m0001	-6.679052543	2.75E-08	1.61E-06	DEG
Bpev01.c0427.g0034.m0001	-1.308674666	0.00012839	0.00199889	DEG
Bpev01.c0015.g0028.m0001	-1.748897607	0.00018652	0.00270222	DEG
Bpev01.c1226.g0003.m0001	2.169210148	2.11E-05	0.00045456	DEG
Bpev01.c0025.g0035.m0001	2.346686829	5.97E-06	0.00015923	DEG
Bpev01.c0402.g0029.m0001	-1.731371906	0.00015943	0.00237759	DEG
Bpev01.c0223.g0051.m0001	-0.862135283	0.00567298	0.04023294	DEG
Bpev01.c0470.g0043.m0001	-1.212724806	0.00048818	0.00591422	DEG
Bpev01.c0425.g0050.m0001	1.270984519	0.00071562	0.00797694	DEG
Bpev01.c0340.g0001.m0001	3.636439468	1.83E-19	1.32E-16	DEG
Bpev01.c1101.g0006.m0001	5.983456632	6.54E-07	2.48E-05	DEG
Bpev01.c0038.g0087.m0001	2.210232003	1.90E-10	2.04E-08	DEG
Bpev01.c0044.g0060.m0001	2.858356458	1.66E-06	5.41E-05	DEG
Bpev01.c0462.g0005.m0001	2.090947746	3.76E-06	0.00010794	DEG
Bpev01.c0126.g0023.m0001	-1.408599885	0.0001142	0.00181236	DEG
Bpev01.c0115.g0056.m0001	-1.333164132	4.38E-05	0.00082842	DEG
Bpev01.c0044.g0015.m0001	-1.741074832	1.51E-05	0.00034911	DEG
Bpev01.c0142.g0059.m0001	-1.344665828	1.36E-05	0.00031916	DEG
Bpev01.c0261.g0042.m0001	-1.208498217	0.00029157	0.00386118	DEG
Bpev01.c0343.g0010.m0001	-2.029556114	0.00188149	0.01728209	DEG
Bpev01.c2310.g0003.m0001	0.88566032	0.00659049	0.04520443	DEG
Bpev01.c0022.g0158.m0001	1.429578115	0.00735781	0.0490504	DEG
Bpev01.c0191.g0020.m0002	-1.208649693	0.00051818	0.00619854	DEG
Bpev01.c1999.g0005.m0001	-2.037061927	9.04E-06	0.00022711	DEG
Bpev01.c2035.g0007.m0001	-1.432544397	4.92E-05	0.00091432	DEG
Bpev01.c0531.g0011.m0001	-0.997749432	0.00120191	0.01195779	DEG
Bpev01.c2000.g0004.m0001	-3.576483936	1.72E-05	0.0003869	DEG
Bpev01.c0031.g0012.m0001	-1.933994898	9.01E-06	0.00022644	DEG
Bpev01.c0088.g0088.m0001	-1.939556139	0.00100808	0.01039911	DEG
Bpev01.c0480.g0070.m0001	-0.842366991	0.00558647	0.03975345	DEG
Bpev01.c0115.g0082.m0001	2.200068048	1.77E-09	1.47E-07	DEG
Bpev01.c2343.g0001.m0001	1.253083459	0.00117193	0.01173489	DEG
Bpev01.c2660.g0002.m0001	-2.387104582	1.15E-06	4.02E-05	DEG
Bpev01.c0029.g0131.m0001	-2.504768091	0.00220549	0.01949189	DEG
Bpev01.c0451.g0002.m0001	1.105783706	0.0006147	0.00707157	DEG

Bpev01.c1135.g0008.m0001	1.215409009	0.00720805	0.04832561	DEG
Bpev01.c0154.g0065.m0001	5.014551908	3.33E-19	2.15E-16	DEG
Bpev01.c0148.g0006.m0001	-1.754241457	6.06E-06	0.00016089	DEG
Bpev01.c1054.g0003.m0001	1.126229599	0.00026535	0.00357515	DEG
Bpev01.c0018.g0006.m0001	2.374277834	1.55E-06	5.10E-05	DEG
Bpev01.c0744.g0008.m0001	-1.156528063	0.0003075	0.00402338	DEG
Bpev01.c0594.g0002.m0001	-2.043983908	3.47E-09	2.64E-07	DEG
Bpev01.c0961.g0001.m0001	-1.900125495	1.40E-06	4.71E-05	DEG
Bpev01.c0383.g0005.m0001	1.489605704	0.00040119	0.00503294	DEG
Bpev01.c0229.g0019.m0001	-1.435081535	0.00019506	0.00279663	DEG
Bpev01.c2648.g0002.m0001	1.706275162	0.00048402	0.00587587	DEG
Bpev01.c0126.g0051.m0001	1.749720293	1.87E-05	0.00041296	DEG
Bpev01.c0963.g0002.m0001	0.967033588	0.00544046	0.03910197	DEG
Bpev01.c2062.g0001.m0001	-1.98961677	4.72E-06	0.00012977	DEG
Bpev01.c1160.g0007.m0001	1.797434935	1.65E-06	5.39E-05	DEG
Bpev01.c2103.g0005.m0001	-1.380756191	1.59E-05	0.00036296	DEG
Bpev01.c2330.g0001.m0001	0.945884913	0.00493027	0.03629464	DEG
Bpev01.c0015.g0056.m0001	-2.143254119	5.44E-05	0.00098754	DEG
Bpev01.c0485.g0032.m0001	-3.134347207	1.53E-08	9.64E-07	DEG
Bpev01.c2243.g0001.m0001	1.135532719	0.0013077	0.01282816	DEG
Bpev01.c0285.g0004.m0001	3.500127308	8.06E-08	4.08E-06	DEG
Bpev01.c0420.g0010.m0001	-1.966484405	0.00190569	0.01743908	DEG
Bpev01.c0215.g0035.m0001	2.176548887	0.00018216	0.00264719	DEG
Bpev01.c1382.g0026.m0001	-1.566172831	6.34E-06	0.00016706	DEG
Bpev01.c0363.g0022.m0001	-2.125038633	9.28E-09	6.34E-07	DEG
Bpev01.c1233.g0009.m0001	-1.798921501	0.0002478	0.00337573	DEG
Bpev01.c0505.g0034.m0001	1.447613717	0.0001194	0.00188062	DEG
Bpev01.c0015.g0012.m0001	1.331077408	0.00079551	0.00863529	DEG
Bpev01.c0850.g0003.m0001	-2.414737315	3.87E-09	2.89E-07	DEG
Bpev01.c0215.g0035.m0002	2.065420667	0.00019871	0.0028301	DEG
Bpev01.c0335.g0029.m0001	2.050971468	0.00027268	0.0036533	DEG
Bpev01.c1816.g0001.m0001	1.857537595	0.00232252	0.020295	DEG
Bpev01.c0120.g0042.m0001	1.213873469	7.74E-05	0.00131247	DEG
Bpev01.c0634.g0024.m0001	3.094710035	1.64E-07	7.46E-06	DEG
Bpev01.c0222.g0045.m0001	-2.865535069	1.25E-06	4.30E-05	DEG
Bpev01.c3010.g0003.m0001	-1.34164534	0.0002456	0.00335039	DEG
Bpev01.c1577.g0011.m0001	-1.422002451	0.00056665	0.00661185	DEG
Bpev01.c1419.g0003.m0001	1.257571259	0.00029982	0.00394563	DEG
Bpev01.c0634.g0009.m0001	1.393853472	0.00430973	0.03275019	DEG
Bpev01.c0789.g0006.m0001	2.022762818	6.64E-07	2.51E-05	DEG
Bpev01.c0427.g0002.m0001	-0.948206391	0.00470393	0.03506684	DEG
Bpev01.c0169.g0035.m0001	-0.873474488	0.00547245	0.03927304	DEG
Bpev01.c0077.g0011.m0001	1.591558736	2.08E-05	0.00044989	DEG

Bpev01.c0027.g0143.m0001	1.167325603	0.00126524	0.01247148	DEG
Bpev01.c0020.g0001.m0001	-1.070245308	0.00049763	0.00601174	DEG
Bpev01.c0391.g0002.m0001	2.009128264	1.77E-05	0.0003959	DEG
Bpev01.c0473.g0035.m0001	-0.966677552	0.00313178	0.02573882	DEG
Bpev01.c1278.g0005.m0001	2.909999654	1.60E-06	5.25E-05	DEG
Bpev01.c1044.g0016.m0001	-1.673372681	0.00139847	0.01351149	DEG
Bpev01.c1897.g0003.m0001	-2.886287141	0.00243844	0.02108231	DEG
Bpev01.c0683.g0006.m0001	-2.570900188	6.50E-08	3.37E-06	DEG
Bpev01.c2257.g0004.m0001	2.058403339	0.00096841	0.01008807	DEG
Bpev01.c0443.g0026.m0002	1.467681097	0.00014447	0.00219575	DEG
Bpev01.c0578.g0019.m0001	-0.941601797	0.00253231	0.02175967	DEG
Bpev01.c1753.g0004.m0002	-2.223876535	1.89E-09	1.56E-07	DEG
Bpev01.c0136.g0011.m0001	-1.786510378	3.61E-05	0.00070661	DEG
Bpev01.c0052.g0190.m0001	1.040651833	0.0035988	0.02871915	DEG
Bpev01.c0076.g0001.m0001	0.925400887	0.00407341	0.03144132	DEG
Bpev01.c3036.g0001.m0001	2.928407448	3.67E-08	2.06E-06	DEG
Bpev01.c0327.g0018.m0001	-1.710634167	4.69E-06	0.00012953	DEG
Bpev01.c0080.g0089.m0001	-1.929526021	0.00145817	0.01400561	DEG
Bpev01.c0134.g0030.m0001	2.853945662	8.69E-18	4.41E-15	DEG
Bpev01.c0213.g0038.m0001	0.949297986	0.00586829	0.04129967	DEG
Bpev01.c2156.g0004.m0001	3.092562473	0.00050805	0.00610224	DEG
Bpev01.c0621.g0016.m0001	1.939171761	0.00310757	0.02559699	DEG
Bpev01.c0505.g0016.m0001	1.168597443	0.00178571	0.0165521	DEG
Bpev01.c0280.g0012.m0001	-1.319626607	9.63E-05	0.00156738	DEG
Bpev01.c0015.g0062.m0001	1.740210541	0.00055794	0.00654293	DEG
Bpev01.c1972.g0009.m0002	1.582693836	0.00068885	0.00773122	DEG
Bpev01.c1100.g0018.m0001	-2.074349089	9.17E-11	1.05E-08	DEG
Bpev01.c0772.g0010.m0001	1.665594191	0.00014354	0.00218491	DEG
Bpev01.c0392.g0008.m0001	0.965580851	0.00337402	0.02728782	DEG
Bpev01.c0205.g0022.m0001	1.122367286	0.00213651	0.01900202	DEG
Bpev01.c0275.g0002.m0001	2.699421508	2.52E-06	7.69E-05	DEG
Bpev01.c0022.g0135.m0002	3.31683619	3.77E-09	2.82E-07	DEG
Bpev01.c0212.g0006.m0001	3.012433508	1.16E-07	5.51E-06	DEG
Bpev01.c0015.g0120.m0001	-1.943047383	4.61E-06	0.00012754	DEG
Bpev01.c1770.g0002.m0001	8.951306554	2.64E-08	1.55E-06	DEG
Bpev01.c0169.g0027.m0001	-2.067535244	0.00221963	0.01959922	DEG
Bpev01.c1064.g0009.m0001	2.82678098	0.00578426	0.04079611	DEG
Bpev01.c2743.g0002.m0001	1.882038322	0.00051902	0.0062061	DEG
Bpev01.c0210.g0046.m0001	1.166424697	0.00016118	0.00239764	DEG
Bpev01.c1367.g0002.m0001	0.959502797	0.0054381	0.03909549	DEG
Bpev01.c3876.g0002.m0001	1.407897202	0.00052371	0.0062529	DEG
Bpev01.c0042.g0002.m0001	3.961626807	6.71E-29	2.20E-25	DEG
Bpev01.c1030.g0005.m0001	2.558122558	0.00317955	0.02606591	DEG

Bpev01.c0114.g0058.m0001	-2.024171795	6.13E-05	0.00108577	DEG
Bpev01.c0447.g0022.m0001	3.085969199	3.36E-05	0.00067071	DEG
Bpev01.c0444.g0010.m0001	2.933644493	8.98E-05	0.00148477	DEG
Bpev01.c0095.g0017.m0001	-1.933839168	0.00186741	0.01717501	DEG
Bpev01.c1574.g0002.m0001	-2.222200735	0.00483082	0.03575926	DEG
Bpev01.c0879.g0017.m0002	-1.055245027	0.00504983	0.03690717	DEG
Bpev01.c3221.g0001.m0001	1.821128257	5.72E-05	0.00103005	DEG
Bpev01.c0157.g0051.m0001	-4.761439422	1.43E-05	0.00033334	DEG
Bpev01.c0222.g0047.m0001	-3.392279893	0.00014745	0.00222771	DEG
Bpev01.c0023.g0006.m0001	-2.126764206	9.32E-08	4.56E-06	DEG
Bpev01.c0549.g0003.m0001	-1.504338001	0.00010514	0.00168766	DEG
Bpev01.c0222.g0026.m0001	1.654805467	1.68E-05	0.00037935	DEG
Bpev01.c0804.g0009.m0001	-1.200846941	0.0003008	0.00395494	DEG
Bpev01.c0667.g0008.m0002	1.273763533	0.0001437	0.00218619	DEG
Bpev01.c0024.g0024.m0001	1.905716836	3.31E-08	1.89E-06	DEG
Bpev01.c0919.g0011.m0001	7.263712693	0.00026636	0.00358704	DEG
Bpev01.c0029.g0070.m0001	2.062729399	2.59E-05	0.00053915	DEG
Bpev01.c1308.g0001.m0001	1.982307159	7.31E-05	0.00125294	DEG
Bpev01.c1069.g0004.m0001	-1.061009522	0.00415661	0.03189118	DEG
Bpev01.c0022.g0039.m0001	2.250157705	8.68E-13	1.49E-10	DEG
Bpev01.c0214.g0014.m0001	2.635582767	0.00606799	0.04229114	DEG
Bpev01.c0062.g0077.m0001	0.957879302	0.00295568	0.02457975	DEG
Bpev01.c0090.g0017.m0004	4.418415605	1.36E-11	1.81E-09	DEG
Bpev01.c2235.g0012.m0001	2.101142414	1.24E-06	4.30E-05	DEG
Bpev01.c1786.g0012.m0001	-1.384254473	3.15E-05	0.00063285	DEG
Bpev01.c0484.g0038.m0001	-1.440624115	2.60E-06	7.88E-05	DEG
Bpev01.c0802.g0001.m0001	2.504213441	0.00023027	0.00318704	DEG
Bpev01.c1562.g0004.m0001	3.310756725	4.41E-12	6.46E-10	DEG
Bpev01.c0381.g0015.m0001	-0.935644484	0.00454177	0.03408902	DEG
Bpev01.c0219.g0018.m0001	-3.014537304	4.07E-06	0.00011499	DEG
Bpev01.c0652.g0023.m0001	-1.697996429	7.35E-05	0.00125806	DEG
Bpev01.c0702.g0011.m0001	-1.380988979	4.94E-05	0.00091714	DEG
Bpev01.c0556.g0010.m0001	2.716212539	0.00015558	0.00233319	DEG
Bpev01.c1067.g0001.m0001	1.55540343	1.52E-05	0.00035065	DEG
Bpev01.c0454.g0005.m0001	2.110211695	4.89E-07	1.91E-05	DEG
Bpev01.c0863.g0009.m0001	0.924713924	0.00210636	0.01879633	DEG
Bpev01.c2035.g0003.m0001	-2.616587521	1.85E-07	8.24E-06	DEG
Bpev01.c0439.g0047.m0001	-1.744917333	0.00061575	0.00708088	DEG
Bpev01.c1786.g0014.m0001	-1.839506888	0.00159692	0.01512115	DEG
Bpev01.c1806.g0002.m0001	1.412721991	7.50E-05	0.00127697	DEG
Bpev01.c0171.g0001.m0001	-1.544994619	1.03E-05	0.00025151	DEG
Bpev01.c0253.g0034.m0001	-2.417982211	9.03E-05	0.00148875	DEG
Bpev01.c1132.g0006.m0001	-2.307533376	1.55E-06	5.10E-05	DEG

Bpev01.c1037.g0001.m0001	-2.483154258	0.00131708	0.01289624	DEG
Bpev01.c0639.g0023.m0001	-1.527134963	4.54E-06	0.0001259	DEG
Bpev01.c0299.g0012.m0002	1.317029152	0.00025857	0.00349817	DEG
Bpev01.c0031.g0028.m0001	-1.88627258	8.23E-08	4.14E-06	DEG
Bpev01.c0015.g0150.m0001	-1.777578784	2.31E-08	1.39E-06	DEG
Bpev01.c0278.g0022.m0001	1.464251939	7.68E-06	0.0001975	DEG
Bpev01.c1028.g0007.m0001	-1.107106222	0.00045672	0.00559519	DEG
Bpev01.c0764.g0003.m0001	1.069521028	0.00077466	0.00845263	DEG
Bpev01.c0274.g0059.m0001	-1.635767237	1.13E-05	0.00027227	DEG
Bpev01.c0000.g0150.m0001	-1.515793124	5.34E-05	0.00097326	DEG
Bpev01.c0531.g0022.m0001	-1.023011417	0.00119131	0.01187747	DEG
Bpev01.c0658.g0012.m0002	1.353947844	7.13E-05	0.00122556	DEG
Bpev01.c0169.g0029.m0001	-1.864393672	0.00127612	0.01255031	DEG
Bpev01.c2283.g0001.m0001	2.169119777	9.17E-08	4.50E-06	DEG
Bpev01.c0162.g0074.m0001	0.998810754	0.00295512	0.02457975	DEG
Bpev01.c0091.g0033.m0001	-2.448622745	5.03E-09	3.67E-07	DEG
Bpev01.c0950.g0004.m0001	1.215928164	0.00013928	0.00213452	DEG
Bpev01.c0127.g0058.m0001	-3.280553883	5.97E-14	1.31E-11	DEG
Bpev01.c0480.g0025.m0001	1.368531623	0.00020266	0.00287103	DEG
Bpev01.c0050.g0106.m0001	1.093290859	0.00124039	0.01227424	DEG
Bpev01.c0854.g0001.m0001	3.045104808	1.14E-12	1.92E-10	DEG
Bpev01.c0146.g0020.m0001	-2.468924384	1.00E-07	4.83E-06	DEG
Bpev01.c0390.g0006.m0001	-2.835587963	6.38E-08	3.32E-06	DEG
Bpev01.c0581.g0004.m0001	1.130665513	0.00054374	0.00641824	DEG
Bpev01.c0015.g0203.m0001	-0.968959305	0.00175668	0.01633961	DEG
Bpev01.c0001.g0077.m0001	-1.297418935	0.0063783	0.04406731	DEG
Bpev01.c1119.g0006.m0001	-2.296710547	0.00436781	0.03308905	DEG
Bpev01.c0295.g0001.m0001	-0.908871104	0.00528188	0.03819538	DEG
Bpev01.c2319.g0001.m0001	4.087363541	0.00105486	0.01076399	DEG
Bpev01.c0000.g0146.m0001	-1.489018529	0.00363102	0.02892928	DEG
Bpev01.c0217.g0001.m0001	-2.929260722	1.11E-10	1.24E-08	DEG
Bpev01.c0115.g0019.m0001	-1.007051936	0.00277327	0.02341336	DEG
Bpev01.c0161.g0053.m0001	1.249492593	0.00013463	0.00208159	DEG
Bpev01.c0931.g0003.m0001	2.102993851	2.03E-07	8.93E-06	DEG
Bpev01.c0261.g0077.m0001	1.568338843	0.0020124	0.01812823	DEG
Bpev01.c1120.g0016.m0001	-1.052115219	0.00098287	0.01020981	DEG
Bpev01.c0245.g0055.m0001	3.015141017	2.55E-11	3.18E-09	DEG
Bpev01.c1347.g0008.m0001	2.13634978	0.0027893	0.02350147	DEG
Bpev01.c0414.g0003.m0001	3.40757451	8.58E-08	4.27E-06	DEG
Bpev01.c0000.g0167.m0001	1.085003712	0.007539	0.04993042	DEG
Bpev01.c0088.g0127.m0001	4.036133721	4.54E-08	2.50E-06	DEG
Bpev01.c0878.g0015.m0001	-1.682963639	0.00017618	0.0025766	DEG
Bpev01.c0094.g0283.m0001	-1.151270039	0.00025599	0.00346802	DEG

Bpev01.c0029.g0036.m0001	-1.117990777	0.00106906	0.01087495	DEG
Bpev01.c1756.g0002.m0001	-1.155244307	0.00151454	0.01445733	DEG
Bpev01.c1429.g0001.m0001	-2.41423941	1.21E-06	4.19E-05	DEG
Bpev01.c2297.g0004.m0001	5.724842339	8.10E-34	5.96E-30	DEG
Bpev01.c1416.g0001.m0001	-1.317351029	0.00654359	0.04494525	DEG
Bpev01.c0304.g0006.m0001	-1.440908982	5.08E-05	0.00093565	DEG
Bpev01.c0457.g0003.m0001	2.832408615	0.00126328	0.01245886	DEG
Bpev01.c0289.g0008.m0001	1.48899936	0.00384288	0.03012386	DEG
Bpev01.c0134.g0098.m0001	-1.742580205	0.00086747	0.00929312	DEG
Bpev01.c0232.g0014.m0001	-2.219611801	0.00074537	0.00820912	DEG
Bpev01.c0172.g0007.m0001	-0.908885368	0.0050754	0.03703883	DEG
Bpev01.c0082.g0005.m0001	2.938293387	1.03E-06	3.67E-05	DEG
Bpev01.c1179.g0010.m0001	1.83461783	1.21E-05	0.00028881	DEG
Bpev01.c0473.g0048.m0001	-1.76819087	2.92E-07	1.22E-05	DEG
Bpev01.c0101.g0017.m0001	1.508269379	1.76E-05	0.00039462	DEG
Bpev01.c0023.g0035.m0001	-1.058247551	0.00116611	0.01168436	DEG
Bpev01.c0362.g0001.m0001	-1.259815846	0.00012416	0.00194731	DEG
Bpev01.c0709.g0003.m0001	0.982784135	0.00271824	0.02304129	DEG
Bpev01.c0777.g0012.m0001	-0.937604341	0.00290182	0.02422076	DEG
Bpev01.c1455.g0008.m0001	2.446652303	0.00018523	0.00268481	DEG
Bpev01.c0774.g0017.m0001	3.301001096	7.04E-12	9.97E-10	DEG
Bpev01.c0575.g0024.m0001	-1.367730423	3.36E-05	0.00066998	DEG
Bpev01.c0157.g0002.m0001	-1.108297763	0.00038252	0.00483933	DEG
Bpev01.c0000.g0208.m0001	1.75249222	8.38E-05	0.00140534	DEG
Bpev01.c0429.g0029.m0001	0.886881923	0.00323973	0.02642686	DEG
Bpev01.c0157.g0032.m0001	-1.056267806	0.00708897	0.04773382	DEG
Bpev01.c0794.g0003.m0001	-1.185299577	0.00027368	0.00366378	DEG
Bpev01.c1964.g0001.m0001	2.535810345	7.27E-06	0.00018836	DEG
Bpev01.c0263.g0013.m0001	-2.476885092	8.97E-06	0.00022569	DEG
Bpev01.c2675.g0003.m0001	-1.803041906	0.00027193	0.00364704	DEG
Bpev01.c0396.g0018.m0001	2.723080661	8.46E-05	0.00141466	DEG
Bpev01.c1250.g0014.m0001	0.921785438	0.00284111	0.02383571	DEG
Bpev01.c0357.g0029.m0001	-1.560944761	1.05E-06	3.71E-05	DEG
Bpev01.c1083.g0003.m0001	-7.076214525	0.00156369	0.01484468	DEG
Bpev01.c0850.g0007.m0001	-1.186170866	0.00366128	0.02911519	DEG
Bpev01.c0428.g0006.m0001	-2.21941787	0.00023067	0.00319009	DEG
Bpev01.c0222.g0027.m0001	0.898332504	0.00552051	0.03952294	DEG
Bpev01.c1167.g0020.m0001	-2.531073529	0.00074113	0.00817463	DEG
Bpev01.c2689.g0001.m0001	-2.177731337	0.00086809	0.0092964	DEG
Bpev01.c0089.g0048.m0001	-2.398359388	9.19E-05	0.00151137	DEG
Bpev01.c0380.g0002.m0001	1.564654117	8.33E-05	0.00139849	DEG
Bpev01.c0015.g0053.m0001	0.842935518	0.0062013	0.04308708	DEG
Bpev01.c1270.g0017.m0001	-1.235217494	4.54E-05	0.00085413	DEG

Bpev01.c1453.g0004.m0001	-1.250593964	0.00079752	0.00865206	DEG
Bpev01.c1607.g0005.m0001	2.217221658	5.24E-05	0.00096138	DEG
Bpev01.c0414.g0008.m0001	1.913385594	1.03E-08	6.95E-07	DEG
Bpev01.c1539.g0005.m0001	0.953423552	0.00689424	0.04677563	DEG
Bpev01.c0148.g0015.m0001	-1.38399819	5.04E-05	0.00093173	DEG
Bpev01.c2535.g0009.m0001	-1.641530775	0.00037514	0.00476136	DEG
Bpev01.c0192.g0007.m0001	-1.231821607	0.00230274	0.0201724	DEG
Bpev01.c1160.g0009.m0001	1.468407255	0.00645297	0.04447405	DEG
Bpev01.c0044.g0082.m0001	1.026865996	0.00224637	0.01978221	DEG
Bpev01.c0422.g0020.m0001	1.431065287	4.85E-05	0.00090451	DEG
Bpev01.c0550.g0005.m0001	-0.915469134	0.00308764	0.02546132	DEG
Bpev01.c1560.g0005.m0001	-1.875087619	0.00086397	0.00926765	DEG
Bpev01.c0522.g0046.m0001	-1.681361658	0.00025093	0.00341054	DEG
Bpev01.c0158.g0052.m0001	-1.0583142	0.00232406	0.0203021	DEG
Bpev01.c0429.g0027.m0001	-1.648240342	0.00335519	0.02715794	DEG
Bpev01.c0522.g0049.m0001	-1.456330029	1.14E-05	0.00027303	DEG
Bpev01.c0215.g0016.m0001	1.395127818	0.00035291	0.00451319	DEG
Bpev01.c0506.g0024.m0001	-1.569000872	0.00071708	0.00798718	DEG
Bpev01.c0145.g0072.m0001	1.180668522	0.00054197	0.00639993	DEG
Bpev01.c0329.g0015.m0001	-1.449760424	0.00035021	0.00448443	DEG
Bpev01.c1501.g0008.m0001	1.408256108	0.00013723	0.00211185	DEG
Bpev01.c0000.g0195.m0001	2.051327663	0.00329746	0.02680386	DEG
Bpev01.c0470.g0010.m0001	1.354845297	0.00133617	0.0130423	DEG
Bpev01.c0158.g0059.m0001	2.620491403	0.00048445	0.0058787	DEG
Bpev01.c0213.g0015.m0001	-1.485059585	9.62E-05	0.00156737	DEG
Bpev01.c0353.g0023.m0001	-1.302022879	0.00011705	0.00184962	DEG
Bpev01.c0053.g0015.m0002	1.795667275	1.98E-06	6.28E-05	DEG
Bpev01.c1403.g0005.m0001	2.210214455	0.0023467	0.02046339	DEG
Bpev01.c0798.g0006.m0001	-2.382091473	0.0001058	0.00169734	DEG
Bpev01.c0393.g0001.m0001	1.827453256	0.00613949	0.04267781	DEG
Bpev01.c2320.g0001.m0001	1.523439037	1.46E-06	4.86E-05	DEG
Bpev01.c0053.g0056.m0001	2.519645837	3.45E-07	1.41E-05	DEG
Bpev01.c1155.g0005.m0001	1.960349719	0.00222335	0.01960819	DEG
Bpev01.c0745.g0008.m0001	1.61177123	0.00014669	0.00222143	DEG
Bpev01.c0890.g0008.m0001	1.081320733	0.00554001	0.03960477	DEG
Bpev01.c0389.g0057.m0001	-1.930110318	8.83E-06	0.00022364	DEG
Bpev01.c0401.g0022.m0001	1.230079784	0.00032174	0.00417251	DEG
Bpev01.c1418.g0012.m0001	3.800507634	0.00300737	0.02491109	DEG
Bpev01.c0389.g0012.m0001	3.091158729	6.42E-10	6.00E-08	DEG
Bpev01.c3377.g0001.m0001	3.377098075	3.09E-13	5.83E-11	DEG
Bpev01.c0114.g0035.m0001	-1.155599502	0.00099999	0.01034025	DEG
Bpev01.c1631.g0001.m0001	-1.48967924	2.84E-05	0.00058236	DEG
Bpev01.c0232.g0007.m0001	-1.990138301	0.00233026	0.02034295	DEG

Bpev01.c1286.g0001.m0001	1.771351782	1.76E-06	5.70E-05	DEG
Bpev01.c0337.g0007.m0001	-1.264327077	0.00182543	0.01686719	DEG
Bpev01.c0344.g0036.m0001	-1.466072386	0.00024945	0.00339347	DEG
Bpev01.c0455.g0003.m0001	2.414573056	4.86E-08	2.64E-06	DEG
Bpev01.c1272.g0021.m0001	1.600612397	4.29E-06	0.00012032	DEG
Bpev01.c0052.g0011.m0001	-2.488813204	0.00017984	0.00261702	DEG
Bpev01.c0064.g0001.m0001	-1.439649548	0.00198673	0.0179685	DEG
Bpev01.c1391.g0002.m0001	0.970522783	0.00519367	0.03773357	DEG
Bpev01.c0575.g0011.m0001	-6.443444219	0.00365718	0.02909828	DEG
Bpev01.c0015.g0143.m0002	2.465086153	1.02E-06	3.63E-05	DEG
Bpev01.c0719.g0014.m0001	1.056390961	0.00384525	0.03013053	DEG
Bpev01.c0018.g0046.m0001	-3.465303819	2.02E-13	3.98E-11	DEG
Bpev01.c0320.g0003.m0001	-1.032430837	0.0008226	0.00888681	DEG
Bpev01.c0473.g0002.m0001	1.690768897	0.00106639	0.01085522	DEG
Bpev01.c0953.g0007.m0001	3.628678725	0.0029378	0.02446622	DEG
Bpev01.c2343.g0004.m0001	-0.872326439	0.00392298	0.03058695	DEG
Bpev01.c1114.g0001.m0001	-1.2975524	0.00334121	0.02708423	DEG
Bpev01.c0102.g0011.m0001	1.118198421	0.00056439	0.00659058	DEG
Bpev01.c1851.g0003.m0001	3.145333108	3.20E-17	1.47E-14	DEG
Bpev01.c1910.g0002.m0001	2.103290819	6.55E-08	3.38E-06	DEG
Bpev01.c0423.g0001.m0002	1.716791982	2.82E-05	0.00057849	DEG
Bpev01.c0158.g0017.m0001	-2.105344013	2.86E-06	8.60E-05	DEG
Bpev01.c0018.g0033.m0001	6.279693761	0.00588054	0.04136998	DEG
Bpev01.c0391.g0003.m0001	2.219092413	2.84E-06	8.54E-05	DEG
Bpev01.c1152.g0001.m0001	-1.311232484	0.00011827	0.00186587	DEG
Bpev01.c0444.g0014.m0001	-2.183114576	2.71E-05	0.0005624	DEG
Bpev01.c3059.g0001.m0001	2.632247925	0.00081817	0.00884547	DEG
Bpev01.c0298.g0004.m0001	2.266543553	1.38E-05	0.00032374	DEG
Bpev01.c3708.g0002.m0001	3.375091798	2.30E-06	7.13E-05	DEG
Bpev01.c0134.g0040.m0001	-5.169518611	0.00041294	0.00515548	DEG
Bpev01.c0137.g0017.m0001	3.756794586	4.99E-26	8.64E-23	DEG
Bpev01.c1372.g0005.m0001	-1.998137523	4.71E-06	0.00012977	DEG
Bpev01.c0120.g0038.m0001	-1.264220568	4.02E-05	0.0007712	DEG
Bpev01.c0115.g0035.m0001	1.300985639	7.10E-05	0.0012218	DEG
Bpev01.c1159.g0015.m0001	-1.526755251	7.44E-06	0.00019197	DEG
Bpev01.c0862.g0018.m0001	-1.218808217	0.00034415	0.00441841	DEG
Bpev01.c1684.g0004.m0001	1.013360885	0.00171091	0.01596434	DEG
Bpev01.c0588.g0005.m0001	1.094879433	0.00170744	0.01593986	DEG
Bpev01.c0867.g0008.m0001	-2.730570712	0.00416758	0.03195039	DEG
Bpev01.c0004.g0001.m0001	-2.216849528	1.06E-06	3.75E-05	DEG
Bpev01.c0403.g0027.m0001	-2.544212072	2.72E-05	0.00056355	DEG
Bpev01.c0821.g0019.m0001	1.533779575	5.26E-05	0.00096402	DEG
Bpev01.c1104.g0001.m0001	-2.37716328	0.00019761	0.00282097	DEG

Bpev01.c0018.g0113.m0001	2.379905374	9.22E-08	4.52E-06	DEG
Bpev01.c0276.g0028.m0001	-2.357700639	9.58E-05	0.00156375	DEG
Bpev01.c2125.g0002.m0001	2.863520328	2.01E-06	6.35E-05	DEG
Bpev01.c0154.g0042.m0001	1.547873854	0.00539527	0.03888163	DEG
Bpev01.c1607.g0007.m0001	0.898458622	0.00475452	0.03534555	DEG
Bpev01.c0742.g0001.m0001	1.310377785	0.00012629	0.00197439	DEG
Bpev01.c0344.g0044.m0001	-1.606417965	0.00044227	0.00545914	DEG
Bpev01.c1185.g0022.m0001	4.767524603	3.18E-14	7.38E-12	DEG
Bpev01.c0485.g0023.m0001	1.175113091	0.00034789	0.00445863	DEG
Bpev01.c0667.g0016.m0001	-0.961942433	0.00207193	0.01855662	DEG
Bpev01.c1851.g0001.m0001	2.328350527	2.54E-06	7.74E-05	DEG
Bpev01.c0082.g0068.m0001	2.832213135	2.41E-05	0.00050676	DEG
Bpev01.c1398.g0014.m0001	-1.57283491	4.46E-05	0.00084219	DEG
Bpev01.c0006.g0002.m0001	2.57447769	0.00159878	0.01513396	DEG
Bpev01.c1159.g0006.m0001	1.68277209	3.38E-05	0.00067262	DEG
Bpev01.c0162.g0035.m0001	2.142644932	4.97E-08	2.68E-06	DEG
Bpev01.c1228.g0004.m0001	-1.25458727	0.00691015	0.0468404	DEG
Bpev01.c0114.g0059.m0001	-2.804477333	4.46E-18	2.38E-15	DEG
Bpev01.c1177.g0001.m0001	-1.104731526	0.00072357	0.00802912	DEG
Bpev01.c0051.g0183.m0001	-3.168719011	0.00188581	0.01731096	DEG
Bpev01.c0083.g0003.m0001	-2.435910611	0.00236713	0.02059279	DEG
Bpev01.c0426.g0009.m0001	1.724104822	0.00753208	0.0498958	DEG
Bpev01.c0134.g0046.m0001	-1.905137322	8.53E-06	0.00021706	DEG
Bpev01.c0454.g0028.m0001	-1.80928497	1.58E-07	7.22E-06	DEG
Bpev01.c1979.g0001.m0001	4.642854216	9.73E-05	0.00158104	DEG
Bpev01.c0018.g0041.m0001	1.691704777	2.89E-05	0.00059097	DEG
Bpev01.c0052.g0102.m0001	-1.204292765	0.0002523	0.00342759	DEG
Bpev01.c0299.g0043.m0001	-1.048127855	0.00079307	0.00861519	DEG
Bpev01.c0909.g0001.m0001	-1.462176335	6.62E-05	0.0011566	DEG
Bpev01.c0015.g0162.m0001	-1.138177344	0.00267727	0.02274633	DEG
Bpev01.c0257.g0006.m0001	1.148981348	0.00114525	0.01151867	DEG
Bpev01.c0972.g0004.m0001	1.372014592	4.40E-05	0.00083214	DEG
Bpev01.c0157.g0033.m0001	-1.638357157	0.00073712	0.00814261	DEG
Bpev01.c0820.g0014.m0001	1.658360371	0.00014652	0.0022199	DEG
Bpev01.c0428.g0033.m0001	1.153818683	0.0002443	0.00334503	DEG
Bpev01.c1165.g0001.m0001	-2.082754141	0.00666076	0.04556459	DEG
Bpev01.c0022.g0174.m0001	-2.623855158	1.96E-09	1.60E-07	DEG
Bpev01.c0205.g0023.m0001	1.643012837	0.00101425	0.01044729	DEG
Bpev01.c0671.g0002.m0001	5.96207482	0.00125387	0.01237432	DEG
Bpev01.c3211.g0003.m0001	-1.181353593	0.00023535	0.00324066	DEG
Bpev01.c0015.g0020.m0001	-1.941177142	0.00423698	0.03232239	DEG
Bpev01.c1577.g0005.m0001	-1.564512854	0.00420888	0.03217483	DEG
Bpev01.c0464.g0036.m0001	1.874107884	0.00540704	0.03893782	DEG

Bpev01.c1653.g0003.m0001	3.246034471	0.00755469	0.0499904	DEG
Bpev01.c0458.g0012.m0001	1.534515383	2.50E-06	7.62E-05	DEG
Bpev01.c0827.g0015.m0001	-3.92235988	6.17E-16	2.09E-13	DEG
Bpev01.c0265.g0017.m0001	-1.056743343	0.00211063	0.01881497	DEG
Bpev01.c0018.g0103.m0001	-2.014061459	0.00664341	0.0455144	DEG
Bpev01.c1526.g0006.m0001	-2.600143324	1.67E-07	7.58E-06	DEG
Bpev01.c0894.g0013.m0001	-0.915031405	0.00421177	0.03218855	DEG
Bpev01.c0106.g0012.m0001	-3.115523228	3.20E-07	1.32E-05	DEG
Bpev01.c0142.g0074.m0001	1.348660673	0.00573192	0.04049043	DEG
Bpev01.c0171.g0039.m0001	3.105814228	1.31E-06	4.47E-05	DEG
Bpev01.c1193.g0015.m0001	2.046629008	1.37E-08	8.82E-07	DEG
Bpev01.c0498.g0010.m0001	1.15662635	0.00121455	0.01205905	DEG
Bpev01.c0699.g0024.m0001	3.08904654	0.00258165	0.0220998	DEG
Bpev01.c0646.g0003.m0001	4.529266075	0.00095897	0.01001107	DEG
Bpev01.c1159.g0003.m0001	1.547396323	0.00011216	0.00178668	DEG
Bpev01.c0101.g0077.m0001	-2.699827343	4.26E-05	0.00081077	DEG
Bpev01.c0052.g0031.m0001	1.728530159	0.0001073	0.00171853	DEG
Bpev01.c0304.g0026.m0001	3.983420275	8.46E-12	1.17E-09	DEG
Bpev01.c0568.g0004.m0001	3.292039423	4.06E-13	7.47E-11	DEG
Bpev01.c1229.g0023.m0001	0.921158048	0.00239728	0.02079975	DEG
Bpev01.c0752.g0005.m0001	-2.094818317	1.11E-08	7.38E-07	DEG
Bpev01.c0995.g0001.m0004	3.666170461	1.33E-07	6.22E-06	DEG
Bpev01.c0827.g0002.m0001	-1.411282739	0.00046411	0.0056622	DEG
Bpev01.c0121.g0011.m0001	-0.947988949	0.00422335	0.03226033	DEG
Bpev01.c0896.g0007.m0001	-1.553515774	8.97E-06	0.00022569	DEG
Bpev01.c0711.g0003.m0001	-2.560477554	0.00033437	0.00430789	DEG
Bpev01.c0448.g0002.m0001	1.281513549	7.00E-05	0.00120972	DEG
Bpev01.c0000.g0158.m0001	1.620963306	4.45E-06	0.00012364	DEG
Bpev01.c0828.g0011.m0001	-1.231426888	5.03E-05	0.00093015	DEG
Bpev01.c0265.g0012.m0001	-1.609331837	8.70E-05	0.00144577	DEG
Bpev01.c0038.g0069.m0001	-1.041468687	0.00668536	0.04567427	DEG
Bpev01.c1594.g0002.m0001	3.407187723	9.87E-06	0.00024377	DEG
Bpev01.c0652.g0026.m0001	-1.74681516	1.13E-06	3.95E-05	DEG
Bpev01.c0892.g0019.m0001	-1.610050881	0.00386966	0.03028146	DEG
Bpev01.c0051.g0040.m0001	-1.922416348	6.49E-09	4.62E-07	DEG
Bpev01.c1530.g0004.m0001	3.070248697	0.00326988	0.0266432	DEG
Bpev01.c0403.g0031.m0001	1.169466396	0.00157235	0.01490771	DEG
Bpev01.c0082.g0035.m0001	1.328765901	0.00018218	0.00264719	DEG
Bpev01.c0189.g0061.m0001	-1.887932738	0.00100957	0.01040276	DEG
Bpev01.c1432.g0002.m0001	-1.178688101	0.0001401	0.00214475	DEG
Bpev01.c0299.g0030.m0001	1.335799538	0.00022595	0.00313612	DEG
Bpev01.c1844.g0009.m0001	1.026293248	0.00290408	0.02422592	DEG
Bpev01.c0402.g0027.m0001	-1.260916806	0.00052415	0.00625472	DEG

Bpev01.c0522.g0023.m0001	-3.202913096	4.84E-09	3.55E-07	DEG
Bpev01.c0214.g0043.m0001	-2.361875034	1.08E-05	0.00026242	DEG
Bpev01.c0080.g0073.m0001	2.431505641	1.28E-07	6.01E-06	DEG
Bpev01.c0454.g0027.m0001	-1.180866874	0.00042464	0.00527907	DEG
Bpev01.c0389.g0002.m0001	-2.616412424	1.96E-07	8.62E-06	DEG
Bpev01.c1584.g0010.m0001	2.038594557	0.00283703	0.02382183	DEG
Bpev01.c0114.g0033.m0001	-1.428399129	0.0016844	0.01578699	DEG
Bpev01.c0145.g0056.m0001	0.92019038	0.00558579	0.03975345	DEG
Bpev01.c1374.g0003.m0001	-1.579890227	0.0063113	0.04369668	DEG
Bpev01.c1952.g0002.m0001	-0.895669067	0.00666592	0.04556459	DEG
Bpev01.c0023.g0025.m0001	-1.673578993	0.00099092	0.01026444	DEG
Bpev01.c1259.g0020.m0001	-1.230781957	0.00050654	0.00609155	DEG
Bpev01.c1725.g0001.m0001	2.659031355	0.00513769	0.03740352	DEG
Bpev01.c0157.g0053.m0001	-1.079159151	0.0044866	0.03376016	DEG
Bpev01.c0889.g0012.m0001	-1.116199921	0.00114706	0.0115307	DEG
Bpev01.c0470.g0049.m0002	-2.883028867	8.91E-05	0.00147644	DEG
Bpev01.c0000.g0191.m0001	-2.451353326	4.86E-09	3.56E-07	DEG
Bpev01.c0134.g0017.m0001	3.689475138	0.00032164	0.00417251	DEG
Bpev01.c0129.g0092.m0001	1.195139977	0.00070914	0.00792124	DEG
Bpev01.c1458.g0006.m0001	1.742615341	0.00379641	0.02987502	DEG
Bpev01.c0206.g0006.m0001	-1.419487514	3.70E-05	0.00072277	DEG
Bpev01.c0066.g0059.m0002	0.977136203	0.00255543	0.02191357	DEG
Bpev01.c0817.g0036.m0001	-1.949785849	3.17E-07	1.30E-05	DEG
Bpev01.c0129.g0036.m0001	2.801045331	3.09E-09	2.40E-07	DEG
Bpev01.c0344.g0025.m0001	-1.476263448	0.00126848	0.01249342	DEG
Bpev01.c0142.g0050.m0001	1.952121831	0.00307579	0.02538493	DEG
Bpev01.c0052.g0169.m0001	-2.393511108	0.00154861	0.01472054	DEG
Bpev01.c0439.g0046.m0001	1.518945626	0.00742419	0.04936896	DEG
Bpev01.c0044.g0106.m0001	-1.785964721	8.64E-06	0.0002196	DEG
Bpev01.c0275.g0020.m0001	-0.871187447	0.00559397	0.03978761	DEG
Bpev01.c0101.g0065.m0001	0.982253774	0.00153043	0.01459055	DEG
Bpev01.c0280.g0059.m0001	-2.829123749	1.67E-11	2.15E-09	DEG
Bpev01.c0000.g0120.m0001	1.621182379	1.78E-05	0.00039876	DEG
Bpev01.c1157.g0009.m0001	-2.638641615	9.67E-05	0.00157315	DEG
Bpev01.c0443.g0028.m0001	-1.171320682	0.00394129	0.03068705	DEG
Bpev01.c0690.g0002.m0001	1.176873263	0.00035326	0.00451567	DEG
Bpev01.c1044.g0012.m0001	3.020648971	0.00503018	0.03679092	DEG
Bpev01.c0215.g0042.m0001	-1.33781659	9.89E-05	0.00160303	DEG
Bpev01.c0015.g0006.m0001	1.099564748	0.00112259	0.01133333	DEG
Bpev01.c0044.g0066.m0001	-1.060239785	0.00594941	0.04168128	DEG
Bpev01.c1383.g0002.m0001	1.344458035	6.25E-05	0.00110432	DEG
Bpev01.c0300.g0002.m0001	3.84804751	0.00044568	0.00548513	DEG
Bpev01.c0449.g0030.m0002	-0.950713102	0.00316219	0.02593806	DEG

Bpev01.c0052.g0206.m0001	-1.850904036	2.04E-06	6.44E-05	DEG
Bpev01.c0243.g0039.m0002	1.027218555	0.00198152	0.01795237	DEG
Bpev01.c0191.g0028.m0001	0.929355809	0.00325966	0.02656734	DEG
Bpev01.c1283.g0002.m0001	-1.346706232	4.93E-05	0.00091588	DEG
Bpev01.c0464.g0013.m0001	1.399832451	1.98E-05	0.00043375	DEG
Bpev01.c1915.g0001.m0001	-1.148256275	0.00572409	0.04044926	DEG
Bpev01.c0036.g0023.m0001	-1.637257218	0.00686744	0.04662608	DEG
Bpev01.c0016.g0182.m0001	2.578036999	2.42E-09	1.92E-07	DEG
Bpev01.c2145.g0002.m0001	2.044756581	1.16E-06	4.03E-05	DEG
Bpev01.c0497.g0001.m0001	1.682522844	0.00289261	0.02418504	DEG
Bpev01.c1398.g0006.m0001	2.209032712	1.34E-05	0.00031634	DEG
Bpev01.c0190.g0017.m0001	1.479762525	2.82E-05	0.00057933	DEG
Bpev01.c0029.g0143.m0001	-1.499758607	3.13E-05	0.00062902	DEG
Bpev01.c0036.g0020.m0001	-1.851673071	0.00448297	0.03375297	DEG
Bpev01.c0114.g0039.m0001	1.09566517	0.00605348	0.04223948	DEG
Bpev01.c0090.g0006.m0001	-2.2355397	4.21E-05	0.00080177	DEG
Bpev01.c0443.g0012.m0001	-4.34049889	0.00015159	0.00227913	DEG
Bpev01.c0337.g0011.m0001	-0.951897688	0.00300689	0.02491109	DEG
Bpev01.c0088.g0130.m0001	2.561830894	5.07E-05	0.00093459	DEG
Bpev01.c0745.g0007.m0001	2.074971923	8.20E-05	0.00137852	DEG
Bpev01.c0169.g0022.m0001	-1.28251259	0.00010957	0.00174929	DEG
Bpev01.c0552.g0011.m0002	1.360027434	3.52E-05	0.00069457	DEG
Bpev01.c3139.g0002.m0001	-1.511315484	0.0004448	0.00547776	DEG
Bpev01.c0052.g0216.m0001	-1.986718798	0.00170035	0.01589102	DEG
Bpev01.c2233.g0007.m0001	1.020120983	0.00355595	0.02843662	DEG
Bpev01.c0281.g0064.m0002	-1.316262668	0.00313893	0.02578322	DEG
Bpev01.c0018.g0057.m0001	0.972806658	0.0012327	0.01221049	DEG
Bpev01.c0292.g0006.m0001	-1.044014947	0.00305256	0.02521448	DEG
Bpev01.c0671.g0006.m0002	1.291859312	0.000551	0.00648313	DEG
Bpev01.c0658.g0012.m0001	1.248950634	0.00012264	0.00192766	DEG
Bpev01.c0094.g0141.m0001	4.140870613	0.00352936	0.02825697	DEG
Bpev01.c0522.g0028.m0001	-2.690410325	1.27E-06	4.35E-05	DEG
Bpev01.c0197.g0002.m0001	-1.244840466	0.00517649	0.03763664	DEG
Bpev01.c1002.g0001.m0001	-2.662023803	1.27E-07	5.95E-06	DEG
Bpev01.c0167.g0024.m0001	-1.441293576	0.0035705	0.02852975	DEG
Bpev01.c0447.g0010.m0001	-1.548852023	0.00013257	0.00205519	DEG
Bpev01.c0146.g0013.m0001	-1.077299438	0.00073084	0.00808237	DEG
Bpev01.c0230.g0004.m0001	-1.040925293	0.00414913	0.03185041	DEG
Bpev01.c0389.g0049.m0001	-1.801043092	0.00535582	0.03863511	DEG
Bpev01.c0364.g0034.m0001	3.799349884	4.03E-06	0.00011403	DEG
Bpev01.c2368.g0004.m0001	-1.971338171	2.35E-05	0.00049692	DEG
Bpev01.c1376.g0001.m0001	4.45530408	5.05E-12	7.32E-10	DEG
Bpev01.c2195.g0002.m0001	1.319836455	2.04E-05	0.00044184	DEG

Bpev01.c0478.g0003.m0001	1.200470782	8.40E-05	0.00140665	DEG
Bpev01.c0357.g0014.m0001	1.311658985	0.00310064	0.02554711	DEG
Bpev01.c0413.g0002.m0001	-3.141400442	2.45E-05	0.00051287	DEG
Bpev01.c0276.g0019.m0001	3.557753173	9.15E-07	3.31E-05	DEG
Bpev01.c0640.g0017.m0001	-1.383845428	1.02E-05	0.00025112	DEG
Bpev01.c2160.g0012.m0001	-1.201116715	0.00056461	0.00659058	DEG
Bpev01.c0574.g0011.m0001	-1.762930834	0.0011785	0.01178062	DEG
Bpev01.c1238.g0013.m0002	-1.150875229	0.00038428	0.00485943	DEG
Bpev01.c0753.g0004.m0001	-2.714312502	0.00240023	0.02081305	DEG
Bpev01.c0223.g0057.m0001	1.284260377	0.00368606	0.0292266	DEG
Bpev01.c0051.g0115.m0001	0.944375882	0.00255537	0.02191357	DEG
Bpev01.c0828.g0012.m0001	1.151317425	0.00049781	0.00601174	DEG
Bpev01.c1105.g0009.m0001	-1.375629757	0.0002555	0.00346296	DEG
Bpev01.c1080.g0002.m0001	-1.757145859	0.00072542	0.00803793	DEG
Bpev01.c0157.g0014.m0001	1.072347326	0.00167006	0.01569765	DEG
Bpev01.c0874.g0035.m0001	-6.162083397	0.00697958	0.04714821	DEG
Bpev01.c0286.g0013.m0001	-1.986811652	0.00601548	0.04203414	DEG
Bpev01.c0304.g0027.m0001	3.628219973	6.37E-15	1.75E-12	DEG
Bpev01.c0191.g0037.m0001	-1.207545001	0.00013671	0.00210603	DEG
Bpev01.c0951.g0007.m0001	-2.760203018	0.00322828	0.02636267	DEG
Bpev01.c0191.g0019.m0001	-2.656814355	0.00014713	0.00222533	DEG
Bpev01.c0461.g0001.m0001	2.166297334	3.72E-07	1.51E-05	DEG
Bpev01.c2381.g0003.m0001	-4.4220514	0.00122462	0.0121386	DEG
Bpev01.c1378.g0031.m0001	1.284876494	7.43E-05	0.00126665	DEG
Bpev01.c0170.g0024.m0001	-1.235422427	0.0002143	0.00300844	DEG
Bpev01.c0261.g0077.m0002	1.558550109	0.00105368	0.01075934	DEG
Bpev01.c1226.g0004.m0001	1.727225772	3.82E-05	0.00074008	DEG
Bpev01.c0515.g0006.m0001	-3.144651178	1.21E-07	5.74E-06	DEG
Bpev01.c1815.g0002.m0001	-1.818801267	1.47E-06	4.89E-05	DEG
Bpev01.c2324.g0002.m0001	-0.946239825	0.00650379	0.04472436	DEG
Bpev01.c0134.g0068.m0001	-2.925201537	1.12E-05	0.00027005	DEG
Bpev01.c0161.g0106.m0001	-1.360158441	0.00355663	0.02843662	DEG
Bpev01.c0096.g0031.m0001	3.131733202	8.74E-08	4.32E-06	DEG
Bpev01.c0177.g0023.m0001	3.225741328	7.53E-12	1.06E-09	DEG
Bpev01.c0529.g0012.m0001	5.294310066	9.48E-21	8.21E-18	DEG
Bpev01.c0281.g0004.m0001	1.881073411	1.11E-09	9.86E-08	DEG
Bpev01.c1000.g0019.m0001	2.244588974	0.00035256	0.00451063	DEG
Bpev01.c0864.g0005.m0001	-2.521507904	1.38E-09	1.19E-07	DEG
Bpev01.c0042.g0043.m0001	-1.108936862	0.0038375	0.03010183	DEG
Bpev01.c0281.g0027.m0001	-1.219016054	0.00010911	0.00174289	DEG
Bpev01.c0646.g0009.m0001	1.266827808	0.00028345	0.00377581	DEG
Bpev01.c1054.g0005.m0001	2.994551166	3.36E-19	2.15E-16	DEG
Bpev01.c0299.g0020.m0001	1.660965023	2.28E-07	9.85E-06	DEG

Bpev01.c1547.g0001.m0001	1.77649105	7.31E-07	2.73E-05	DEG
Bpev01.c0104.g0007.m0001	2.731031218	0.0005027	0.00605524	DEG
Bpev01.c1063.g0002.m0001	1.95325982	7.78E-05	0.00131931	DEG
Bpev01.c0536.g0019.m0001	1.047456753	0.00251066	0.02161147	DEG
Bpev01.c0404.g0005.m0001	-1.38156543	0.00443914	0.03352755	DEG
Bpev01.c0289.g0011.m0001	-2.038839751	5.78E-07	2.21E-05	DEG
Bpev01.c0464.g0027.m0001	1.452350718	1.31E-05	0.00030972	DEG
Bpev01.c0389.g0050.m0001	-1.408036569	0.00031336	0.00408183	DEG
Bpev01.c0022.g0106.m0001	2.679691109	1.49E-11	1.96E-09	DEG
Bpev01.c0566.g0061.m0001	-1.160837213	0.0017066	0.01593932	DEG
Bpev01.c0437.g0013.m0002	-1.076329334	0.00218645	0.01937596	DEG
Bpev01.c1938.g0017.m0001	6.376157237	0.00446158	0.03361774	DEG
Bpev01.c1379.g0002.m0001	4.209462003	8.70E-05	0.00144577	DEG
Bpev01.c0389.g0047.m0001	-1.790413769	0.00218088	0.01933238	DEG
Bpev01.c0279.g0019.m0001	-1.9346782	2.44E-07	1.05E-05	DEG
Bpev01.c0016.g0056.m0001	1.568961662	1.34E-05	0.00031531	DEG
Bpev01.c0659.g0014.m0001	2.646681027	2.81E-05	0.00057717	DEG
Bpev01.c0658.g0003.m0001	2.631906881	1.29E-05	0.00030552	DEG
Bpev01.c1659.g0005.m0001	-1.228999779	0.00101621	0.01044729	DEG
Bpev01.c1782.g0020.m0001	4.692408315	2.16E-17	1.03E-14	DEG
Bpev01.c0147.g0001.m0001	-1.677519142	3.39E-06	9.92E-05	DEG
Bpev01.c0429.g0004.m0001	2.324778001	0.00413984	0.03181223	DEG
Bpev01.c1968.g0001.m0001	3.2706344	2.19E-11	2.76E-09	DEG
Bpev01.c1724.g0002.m0001	0.916229683	0.00369314	0.02923432	DEG
Bpev01.c0335.g0043.m0001	1.68624981	5.66E-06	0.00015204	DEG
Bpev01.c1354.g0009.m0001	2.463374897	8.86E-12	1.23E-09	DEG
Bpev01.c3241.g0001.m0001	8.109002769	2.87E-05	0.00058613	DEG
Bpev01.c1884.g0002.m0001	1.465993829	6.49E-06	0.00017058	DEG
Bpev01.c0299.g0041.m0001	-1.709174147	1.75E-05	0.0003937	DEG
Bpev01.c0473.g0046.m0001	1.034815009	0.00103844	0.01063329	DEG
Bpev01.c2146.g0004.m0001	2.125755581	9.39E-06	0.00023407	DEG
Bpev01.c0022.g0070.m0001	3.330857643	1.48E-07	6.85E-06	DEG
Bpev01.c0449.g0029.m0001	-1.023174222	0.00279184	0.02350947	DEG
Bpev01.c0367.g0004.m0001	-1.442813229	3.61E-05	0.00070717	DEG
Bpev01.c0863.g0017.m0001	1.429430323	0.00044379	0.00546871	DEG
Bpev01.c2170.g0002.m0002	2.554472559	2.40E-05	0.00050427	DEG
Bpev01.c1957.g0002.m0001	1.023668555	0.00110108	0.01113911	DEG
Bpev01.c0082.g0060.m0001	-1.711398381	0.00010056	0.00162365	DEG
Bpev01.c0213.g0011.m0001	-1.239257456	0.00038843	0.00490555	DEG
Bpev01.c0443.g0025.m0001	-1.972373113	0.00045353	0.00556548	DEG
Bpev01.c2470.g0006.m0001	1.299634399	2.89E-05	0.00059097	DEG
Bpev01.c1288.g0010.m0001	-2.220977312	6.06E-05	0.00107753	DEG
Bpev01.c0051.g0134.m0001	1.756285947	1.26E-05	0.00029986	DEG

Bpev01.c1024.g0015.m0001	-1.689048133	1.56E-05	0.00035827	DEG
Bpev01.c0398.g0005.m0001	2.661880075	7.16E-13	1.26E-10	DEG
Bpev01.c1334.g0001.m0001	-1.735540627	1.97E-06	6.25E-05	DEG
Bpev01.c0444.g0037.m0001	-2.145649113	0.00226904	0.01993383	DEG
Bpev01.c0263.g0011.m0001	1.658377438	0.00021257	0.00298558	DEG
Bpev01.c0467.g0010.m0001	1.275159764	0.00026955	0.00362347	DEG
Bpev01.c0603.g0006.m0001	-0.887303984	0.00530053	0.03829258	DEG
Bpev01.c0364.g0023.m0001	2.976928003	5.34E-05	0.00097326	DEG
Bpev01.c0964.g0009.m0001	2.152777357	3.05E-09	2.38E-07	DEG
Bpev01.c1687.g0001.m0001	1.666242077	0.00031728	0.00412749	DEG
Bpev01.c0016.g0067.m0001	-2.461518293	1.62E-07	7.38E-06	DEG
Bpev01.c1006.g0020.m0001	1.003085243	0.00161082	0.01522827	DEG
Bpev01.c0062.g0034.m0001	2.200362594	6.95E-07	2.61E-05	DEG
Bpev01.c0284.g0002.m0001	3.165416393	1.49E-11	1.96E-09	DEG
Bpev01.c0289.g0004.m0001	2.575413716	8.76E-06	0.00022203	DEG
Bpev01.c1700.g0005.m0001	-1.722405068	4.07E-08	2.26E-06	DEG
Bpev01.c0029.g0100.m0001	-1.18605885	0.00261133	0.0223214	DEG
Bpev01.c0624.g0022.m0001	-1.507462287	0.00210302	0.01877791	DEG
Bpev01.c0088.g0074.m0001	-2.138204189	0.00341783	0.02757397	DEG
Bpev01.c0051.g0122.m0001	1.112941356	0.00150114	0.0143667	DEG
Bpev01.c0854.g0013.m0001	-1.138702716	0.00046197	0.00563846	DEG
Bpev01.c0052.g0223.m0003	1.714692185	0.0001909	0.00275221	DEG
Bpev01.c1974.g0001.m0001	-1.106477572	0.00162641	0.01534119	DEG
Bpev01.c1080.g0001.m0001	-2.801279204	0.00026038	0.0035211	DEG
Bpev01.c0092.g0001.m0001	1.916127883	5.75E-07	2.20E-05	DEG
Bpev01.c2819.g0001.m0001	1.237628961	7.38E-05	0.00126076	DEG
Bpev01.c1499.g0001.m0001	-1.238045959	0.00048839	0.0059143	DEG
Bpev01.c1122.g0007.m0001	-1.239060545	0.0005401	0.00638704	DEG
Bpev01.c0292.g0004.m0001	1.662663192	1.25E-06	4.30E-05	DEG
Bpev01.c1267.g0011.m0001	5.533012524	0.00012034	0.00189343	DEG
Bpev01.c2054.g0001.m0001	-2.294813087	2.03E-05	0.00044098	DEG
Bpev01.c1097.g0007.m0001	-0.990397594	0.00188786	0.01731904	DEG
Bpev01.c0127.g0079.m0001	-1.500397432	3.18E-06	9.41E-05	DEG
Bpev01.c1189.g0015.m0001	1.905911142	0.00530643	0.03832583	DEG
Bpev01.c1699.g0005.m0001	-1.188780576	0.00128798	0.01266	DEG
Bpev01.c0821.g0007.m0001	-1.35746194	0.00399837	0.03104113	DEG
Bpev01.c0557.g0012.m0001	-1.118768585	0.00037004	0.00470367	DEG
Bpev01.c0118.g0014.m0001	-1.278095707	0.00020654	0.00292189	DEG
Bpev01.c1652.g0002.m0001	1.55584359	0.00098939	0.01025577	DEG
Bpev01.c0328.g0053.m0001	-1.264357916	0.00189243	0.01733935	DEG
Bpev01.c0000.g0113.m0001	-1.577312952	0.00023774	0.00326587	DEG
Bpev01.c0141.g0019.m0001	-1.454019076	5.61E-06	0.00015091	DEG
Bpev01.c3249.g0002.m0001	2.817999647	1.94E-07	8.57E-06	DEG

Bpev01.c0533.g0015.m0001	2.426774342	3.23E-06	9.51E-05	DEG
Bpev01.c2238.g0002.m0001	3.382776726	5.69E-10	5.39E-08	DEG
Bpev01.c0798.g0008.m0001	5.790158146	4.41E-25	6.18E-22	DEG
Bpev01.c0597.g0041.m0001	-1.206297936	0.00018746	0.00271345	DEG
Bpev01.c0091.g0012.m0001	-1.014483047	0.00198631	0.0179685	DEG
Bpev01.c0414.g0009.m0001	-0.950935126	0.00365274	0.02907087	DEG
Bpev01.c0254.g0005.m0001	-1.434568946	3.11E-05	0.00062685	DEG
Bpev01.c2716.g0001.m0001	-2.164899824	1.44E-05	0.00033445	DEG
Bpev01.c2000.g0003.m0001	-1.148127403	0.00026418	0.00356095	DEG
Bpev01.c0356.g0002.m0001	4.869853698	8.46E-09	5.86E-07	DEG
Bpev01.c0995.g0001.m0002	2.568581034	1.96E-06	6.22E-05	DEG
Bpev01.c1912.g0004.m0001	-3.159019867	2.39E-09	1.90E-07	DEG
Bpev01.c0188.g0006.m0001	2.006097579	2.29E-07	9.87E-06	DEG
Bpev01.c0192.g0003.m0001	-2.240507177	5.45E-09	3.93E-07	DEG
Bpev01.c1042.g0001.m0001	1.04078169	0.00075661	0.00830799	DEG
Bpev01.c1930.g0004.m0001	1.599242129	3.63E-07	1.47E-05	DEG
Bpev01.c0127.g0055.m0001	1.971344483	5.20E-06	0.0001418	DEG
Bpev01.c0540.g0019.m0001	1.375001418	0.00062751	0.00718524	DEG
Bpev01.c0115.g0054.m0001	1.244318757	0.0003384	0.00435031	DEG
Bpev01.c0615.g0003.m0001	0.989503867	0.00484294	0.03583992	DEG
Bpev01.c0322.g0010.m0001	3.703519922	4.83E-19	2.90E-16	DEG
Bpev01.c0523.g0001.m0001	1.202475295	0.00019886	0.0028301	DEG
Bpev01.c0152.g0008.m0001	2.801075449	5.76E-14	1.28E-11	DEG
Bpev01.c0074.g0017.m0001	1.210307296	0.00047753	0.00580909	DEG
Bpev01.c0981.g0003.m0001	-1.977278149	1.85E-05	0.00041064	DEG
Bpev01.c1044.g0004.m0001	-1.509705422	2.17E-06	6.76E-05	DEG
Bpev01.c0645.g0001.m0001	-1.332342551	4.58E-05	0.00086035	DEG
Bpev01.c0170.g0044.m0001	-1.347133118	3.16E-05	0.00063407	DEG
Bpev01.c0094.g0011.m0001	-2.28998654	0.00021978	0.00306785	DEG
Bpev01.c0774.g0002.m0001	3.458825052	6.66E-14	1.45E-11	DEG
Bpev01.c0027.g0048.m0001	-1.433329897	5.99E-06	0.00015971	DEG
Bpev01.c2055.g0007.m0001	-1.731628936	1.91E-06	6.10E-05	DEG
Bpev01.c0425.g0017.m0001	0.926833532	0.0055577	0.03965415	DEG
Bpev01.c1289.g0007.m0001	-2.655039943	0.0040503	0.03133018	DEG
Bpev01.c1519.g0012.m0001	-1.076820492	0.00063164	0.00722696	DEG
Bpev01.c0189.g0023.m0001	-2.522448773	6.37E-05	0.00111967	DEG
Bpev01.c0616.g0019.m0001	-1.718783807	5.32E-06	0.00014429	DEG
Bpev01.c0029.g0064.m0001	-1.516860183	0.00078392	0.00853157	DEG
Bpev01.c0504.g0003.m0001	-1.87086739	2.73E-05	0.00056436	DEG
Bpev01.c0036.g0042.m0001	1.141775284	0.00500439	0.03665697	DEG
Bpev01.c0445.g0026.m0001	2.400154108	1.51E-05	0.00034922	DEG
Bpev01.c0044.g0009.m0001	0.820270354	0.00679025	0.04618718	DEG
Bpev01.c1810.g0004.m0001	2.667262063	9.83E-13	1.67E-10	DEG

Bpev01.c0112.g0008.m0001	-1.615981923	3.07E-05	0.000622	DEG
Bpev01.c0485.g0008.m0001	-1.671008357	9.67E-07	3.48E-05	DEG
Bpev01.c1802.g0004.m0001	1.813036274	1.02E-06	3.65E-05	DEG
Bpev01.c0052.g0157.m0001	0.918935907	0.00585497	0.04124539	DEG
Bpev01.c0161.g0011.m0001	1.454367921	5.56E-06	0.00014978	DEG
Bpev01.c0210.g0015.m0001	0.977721047	0.00298598	0.02479193	DEG
Bpev01.c0080.g0055.m0001	1.689916896	3.61E-05	0.00070659	DEG
Bpev01.c0080.g0063.m0001	-1.051766751	0.0013211	0.01291803	DEG
Bpev01.c0154.g0082.m0001	1.903898128	0.00547018	0.03926764	DEG
Bpev01.c0444.g0006.m0001	-1.861417752	0.00418264	0.03201575	DEG
Bpev01.c0051.g0071.m0001	1.240589783	0.00138157	0.01339232	DEG
Bpev01.c0237.g0007.m0001	-1.120222424	0.00254173	0.0218215	DEG
Bpev01.c0866.g0020.m0001	-2.096818349	4.04E-10	3.98E-08	DEG
Bpev01.c1379.g0005.m0001	2.253621366	0.00039352	0.00495712	DEG
Bpev01.c0972.g0005.m0001	-1.262108456	6.62E-05	0.0011566	DEG
Bpev01.c0858.g0002.m0001	-1.817387233	0.00010126	0.00163068	DEG
Bpev01.c0624.g0031.m0001	2.10785527	1.31E-05	0.00030972	DEG
Bpev01.c0772.g0013.m0001	-1.125345843	0.0004367	0.00540168	DEG
Bpev01.c0044.g0114.m0001	-2.561368374	8.78E-08	4.33E-06	DEG
Bpev01.c0115.g0116.m0001	2.151717144	3.34E-08	1.90E-06	DEG
Bpev01.c1081.g0009.m0001	4.839631375	0.00249016	0.0214664	DEG
Bpev01.c0515.g0003.m0001	-1.343284584	1.38E-05	0.00032366	DEG
Bpev01.c0449.g0056.m0001	1.67063421	0.00012982	0.0020168	DEG
Bpev01.c0000.g0135.m0001	-2.56567289	0.00016403	0.00243148	DEG
Bpev01.c0275.g0066.m0001	-1.316219191	4.88E-05	0.00090927	DEG
Bpev01.c0062.g0082.m0001	-2.105047017	7.63E-05	0.00129698	DEG
Bpev01.c1081.g0003.m0001	-2.161569337	0.00017648	0.00257714	DEG
Bpev01.c0168.g0002.m0001	-1.51090606	0.00305125	0.0252107	DEG
Bpev01.c0796.g0010.m0001	-1.437284917	0.00052769	0.00628936	DEG
Bpev01.c0075.g0006.m0001	4.566934212	3.27E-13	6.13E-11	DEG
Bpev01.c0402.g0003.m0001	-1.361130568	0.00261938	0.02236426	DEG
Bpev01.c0044.g0101.m0001	-1.734554251	2.03E-06	6.42E-05	DEG
Bpev01.c0711.g0002.m0001	-2.635114241	0.00029134	0.00385991	DEG
Bpev01.c0042.g0074.m0001	4.686332363	0.0020978	0.01874834	DEG
Bpev01.c0808.g0006.m0001	1.039807108	0.00096921	0.01008807	DEG
Bpev01.c1109.g0003.m0001	-0.968900447	0.00436781	0.03308905	DEG
Bpev01.c1414.g0002.m0001	-1.452588023	2.88E-05	0.00058864	DEG
Bpev01.c1183.g0023.m0001	1.922472228	1.52E-07	7.00E-06	DEG
Bpev01.c0130.g0020.m0001	1.299467967	0.00113551	0.01144805	DEG
Bpev01.c0088.g0017.m0001	-2.136439154	2.45E-10	2.54E-08	DEG
Bpev01.c1453.g0003.m0001	1.302721314	0.00231933	0.02028484	DEG
Bpev01.c0926.g0008.m0001	2.523480851	1.10E-10	1.24E-08	DEG
Bpev01.c0349.g0004.m0001	2.182875635	8.57E-07	3.14E-05	DEG

Bpev01.c0566.g0050.m0002	2.107687224	5.38E-08	2.89E-06	DEG
Bpev01.c0566.g0056.m0001	2.142692584	0.0053984	0.03888511	DEG
Bpev01.c0278.g0043.m0001	2.632632739	2.58E-07	1.10E-05	DEG
Bpev01.c1354.g0005.m0001	-0.951661577	0.00596996	0.04180918	DEG
Bpev01.c0849.g0004.m0001	-1.699913949	0.00068628	0.00771706	DEG
Bpev01.c0699.g0012.m0001	-1.760663714	0.00030152	0.00396273	DEG
Bpev01.c1267.g0006.m0001	-2.045592965	0.00198084	0.01795237	DEG
Bpev01.c0894.g0003.m0001	3.261154757	0.00059986	0.0069225	DEG
Bpev01.c0571.g0019.m0001	-1.312357206	0.00028243	0.00376566	DEG
Bpev01.c0621.g0019.m0001	-1.325672364	8.05E-05	0.00135492	DEG
Bpev01.c1323.g0003.m0001	2.899981871	0.00198239	0.01795237	DEG
Bpev01.c0074.g0007.m0001	-0.986605156	0.00222431	0.01960819	DEG
Bpev01.c0237.g0005.m0002	1.123058142	0.00448196	0.03375297	DEG
Bpev01.c1106.g0008.m0001	-6.237152968	0.00559748	0.03980293	DEG
Bpev01.c1033.g0006.m0001	1.057902336	0.00199689	0.0180382	DEG
Bpev01.c0261.g0045.m0001	1.758589111	1.68E-05	0.00037994	DEG
Bpev01.c0095.g0031.m0002	2.998871311	0.00285454	0.02392792	DEG
Bpev01.c0558.g0001.m0001	-1.425683057	0.00090455	0.00956506	DEG
Bpev01.c1673.g0004.m0001	-1.836879646	1.06E-08	7.11E-07	DEG
Bpev01.c1505.g0005.m0001	-1.205589948	0.00184976	0.01705455	DEG
Bpev01.c0719.g0004.m0001	-1.467280916	3.00E-06	8.92E-05	DEG
Bpev01.c0500.g0009.m0001	1.166870285	0.00043239	0.00535515	DEG
Bpev01.c0142.g0097.m0001	1.055401445	0.00116403	0.0116676	DEG
Bpev01.c0251.g0003.m0001	-1.410963223	0.00251589	0.02164388	DEG
Bpev01.c0129.g0034.m0001	-1.639585106	0.00190254	0.01741568	DEG
Bpev01.c0000.g0193.m0001	2.330634521	0.00438938	0.03321827	DEG
Bpev01.c1674.g0010.m0001	0.860038011	0.00742572	0.04936896	DEG
Bpev01.c0779.g0002.m0001	1.895516055	0.00251206	0.02161726	DEG
Bpev01.c0929.g0024.m0001	1.023682888	0.00330088	0.02681422	DEG
Bpev01.c0165.g0001.m0001	-0.914953159	0.00480247	0.03560712	DEG
Bpev01.c0275.g0002.m0002	2.828857921	5.52E-07	2.13E-05	DEG
Bpev01.c0267.g0025.m0001	-1.926997121	1.11E-08	7.42E-07	DEG
Bpev01.c0629.g0014.m0001	-2.246487907	0.00443936	0.03352755	DEG
Bpev01.c0349.g0068.m0001	-1.016850997	0.00268587	0.02280625	DEG
Bpev01.c1405.g0007.m0001	-2.580860544	0.00281876	0.02370223	DEG
Bpev01.c0017.g0017.m0001	-1.229967251	9.81E-05	0.0015913	DEG
Bpev01.c0401.g0033.m0001	1.688572868	2.04E-06	6.44E-05	DEG
Bpev01.c2230.g0004.m0001	-2.34637834	2.25E-10	2.38E-08	DEG
Bpev01.c1926.g0003.m0001	-1.872635747	5.38E-08	2.89E-06	DEG
Bpev01.c0240.g0009.m0001	1.870074172	3.57E-05	0.00070144	DEG
Bpev01.c0030.g0004.m0001	-2.3159054	0.00040105	0.00503294	DEG
Bpev01.c0029.g0054.m0001	1.28543394	0.00034473	0.00442198	DEG
Bpev01.c1816.g0002.m0001	2.325283601	1.31E-06	4.47E-05	DEG

Bpev01.c0169.g0048.m0001	-1.834997432	2.64E-05	0.00054776	DEG
Bpev01.c3196.g0001.m0001	-1.573347753	0.00033646	0.00433103	DEG
Bpev01.c1294.g0003.m0001	-1.052366645	0.00365816	0.02909828	DEG
Bpev01.c0597.g0039.m0001	-1.276877317	0.00026913	0.00362122	DEG
Bpev01.c0121.g0056.m0001	1.060099508	0.00131727	0.01289624	DEG
Bpev01.c0939.g0009.m0001	-2.614011173	1.83E-07	8.20E-06	DEG
Bpev01.c1172.g0008.m0001	2.181245789	1.84E-08	1.12E-06	DEG
Bpev01.c0428.g0016.m0002	1.121164198	0.00031701	0.00412569	DEG
Bpev01.c0018.g0108.m0001	1.709156298	0.00514064	0.0374129	DEG
Bpev01.c0739.g0017.m0001	-2.983483873	3.23E-08	1.85E-06	DEG
Bpev01.c1120.g0009.m0001	-1.362690034	2.41E-05	0.00050677	DEG
Bpev01.c0580.g0013.m0001	-2.473882226	2.06E-05	0.00044582	DEG
Bpev01.c0365.g0029.m0001	-1.860827558	0.00155937	0.01481329	DEG
Bpev01.c0134.g0056.m0001	-2.15538684	1.73E-09	1.44E-07	DEG
Bpev01.c0531.g0014.m0001	-1.554311038	4.46E-05	0.00084244	DEG
Bpev01.c3054.g0001.m0001	1.543843779	0.00290348	0.02422592	DEG
Bpev01.c0639.g0007.m0001	2.474947452	1.16E-08	7.65E-07	DEG
Bpev01.c0936.g0004.m0001	-1.169453782	0.00136875	0.01329854	DEG
Bpev01.c1167.g0024.m0001	2.702066721	5.66E-09	4.07E-07	DEG
Bpev01.c0833.g0004.m0001	-1.120736089	0.00370445	0.02930019	DEG
Bpev01.c0245.g0040.m0001	-6.313121329	0.00499033	0.03658321	DEG
Bpev01.c1418.g0011.m0001	-2.300933231	0.00333736	0.02706791	DEG
Bpev01.c0015.g0191.m0001	-1.640208133	1.51E-05	0.00034911	DEG
Bpev01.c0502.g0009.m0001	-1.553963022	0.00073875	0.00815754	DEG
Bpev01.c0892.g0015.m0001	-2.545440045	1.09E-09	9.77E-08	DEG
Bpev01.c0364.g0019.m0001	1.568977739	1.72E-05	0.0003869	DEG
Bpev01.c0094.g0254.m0001	-1.04042538	0.00089009	0.00947337	DEG
Bpev01.c0094.g0007.m0001	1.703020599	2.27E-05	0.00048239	DEG
Bpev01.c0352.g0008.m0001	0.886741793	0.00445787	0.03359901	DEG
Bpev01.c0045.g0045.m0001	-1.061456557	0.00052378	0.0062529	DEG
Bpev01.c0915.g0019.m0001	1.050663782	0.00522723	0.03793996	DEG
Bpev01.c1204.g0003.m0001	1.440985378	2.31E-05	0.00048837	DEG
Bpev01.c2075.g0004.m0001	-0.960833237	0.00192507	0.01756188	DEG
Bpev01.c0349.g0040.m0002	1.291765716	0.00199895	0.01804019	DEG
Bpev01.c0050.g0008.m0001	-1.589103901	2.39E-06	7.32E-05	DEG
Bpev01.c1923.g0005.m0001	-2.661393977	0.00016863	0.00248957	DEG
Bpev01.c0610.g0010.m0001	5.448294639	0.00017932	0.00261078	DEG
Bpev01.c2709.g0001.m0001	2.462776466	6.40E-05	0.0011247	DEG
Bpev01.c1221.g0002.m0001	-3.041504102	0.00442073	0.0334211	DEG
Bpev01.c1206.g0009.m0001	-0.854818203	0.00557611	0.03973367	DEG
Bpev01.c0344.g0033.m0001	1.91527974	0.00026056	0.00352184	DEG
Bpev01.c0018.g0097.m0001	-2.249896331	2.37E-06	7.28E-05	DEG
Bpev01.c1211.g0010.m0001	-1.226575846	0.00126963	0.01249635	DEG

Bpev01.c0841.g0006.m0001	2.112630234	3.01E-07	1.25E-05	DEG
Bpev01.c1761.g0002.m0001	-1.152131523	0.00062138	0.00713166	DEG
Bpev01.c0712.g0003.m0001	0.896339067	0.0074352	0.04942081	DEG
Bpev01.c0294.g0012.m0001	-2.125394277	6.40E-08	3.33E-06	DEG
Bpev01.c0161.g0075.m0001	2.956526592	7.32E-05	0.00125355	DEG
Bpev01.c0671.g0007.m0001	0.91862816	0.00349333	0.02805244	DEG
Bpev01.c0629.g0022.m0001	-1.387245123	3.47E-05	0.00068725	DEG
Bpev01.c1013.g0007.m0001	-3.108548149	0.00076757	0.00839087	DEG
Bpev01.c1985.g0008.m0001	0.963694996	0.00271646	0.0230394	DEG
Bpev01.c0114.g0064.m0001	3.339223845	0.00397889	0.03093071	DEG
Bpev01.c0190.g0069.m0001	-1.24719914	9.97E-05	0.00161321	DEG
Bpev01.c0745.g0003.m0002	1.320481126	0.00016115	0.00239764	DEG
Bpev01.c0484.g0008.m0001	-4.280808195	3.63E-08	2.05E-06	DEG
Bpev01.c0279.g0009.m0001	-1.399756121	8.47E-06	0.00021561	DEG
Bpev01.c3322.g0001.m0001	-6.386581208	0.00677904	0.04613225	DEG
Bpev01.c0279.g0010.m0001	-2.88273886	2.57E-07	1.10E-05	DEG
Bpev01.c0024.g0029.m0001	0.913028952	0.00237348	0.0206297	DEG
Bpev01.c0162.g0006.m0001	3.109567212	0.00339893	0.02745916	DEG
Bpev01.c0480.g0057.m0002	2.613675462	0.00042496	0.00528084	DEG
Bpev01.c0016.g0118.m0001	2.15892535	9.31E-10	8.48E-08	DEG
Bpev01.c0396.g0006.m0001	-2.165678957	1.69E-07	7.63E-06	DEG
Bpev01.c0037.g0036.m0001	1.269051605	0.00648356	0.04463281	DEG
Bpev01.c0115.g0079.m0001	-1.43991903	3.12E-05	0.00062762	DEG
Bpev01.c0292.g0002.m0001	-1.054780246	0.00086497	0.00926971	DEG
Bpev01.c1047.g0003.m0001	-2.913840858	5.38E-16	1.84E-13	DEG
Bpev01.c1290.g0001.m0001	1.360388366	0.00132125	0.01291803	DEG
Bpev01.c0375.g0006.m0001	-1.812443902	0.00053462	0.00635131	DEG
Bpev01.c1458.g0005.m0001	1.980651586	4.68E-05	0.00087597	DEG
Bpev01.c0867.g0016.m0001	-1.697654772	0.00137169	0.01331835	DEG
Bpev01.c1042.g0008.m0001	-1.566064014	0.0004271	0.00530294	DEG
Bpev01.c0051.g0127.m0001	-2.074092263	2.24E-05	0.00047739	DEG
Bpev01.c2576.g0001.m0001	5.527762561	3.39E-05	0.00067423	DEG
Bpev01.c0037.g0034.m0001	1.706188549	3.88E-06	0.00011023	DEG
Bpev01.c0053.g0038.m0001	-2.183753374	0.00019509	0.00279663	DEG
Bpev01.c1009.g0008.m0001	-2.08183777	0.00027277	0.0036533	DEG
Bpev01.c1371.g0007.m0001	-1.163736391	0.00105898	0.0108023	DEG
Bpev01.c0082.g0029.m0001	0.94568068	0.00266543	0.02265883	DEG
Bpev01.c0023.g0032.m0001	-1.971071551	2.44E-10	2.54E-08	DEG
Bpev01.c0457.g0040.m0002	1.331660402	0.00072309	0.00802912	DEG
Bpev01.c0237.g0047.m0001	2.782517959	8.76E-07	3.20E-05	DEG
Bpev01.c0082.g0001.m0001	-2.582381118	1.05E-05	0.00025721	DEG
Bpev01.c0485.g0024.m0001	-2.436170816	0.00392324	0.03058695	DEG
Bpev01.c0803.g0014.m0001	-2.287401929	0.00161465	0.01525965	DEG

Bpev01.c0565.g0023.m0001	-3.253483708	2.84E-07	1.19E-05	DEG
Bpev01.c0038.g0017.m0001	1.572561766	9.43E-06	0.00023445	DEG
Bpev01.c0038.g0045.m0001	-0.903729068	0.00283977	0.02383121	DEG
Bpev01.c1046.g0006.m0001	1.525585165	1.43E-06	4.79E-05	DEG
Bpev01.c1902.g0002.m0001	1.260052408	0.00724591	0.04854627	DEG
Bpev01.c0557.g0046.m0001	-1.395377532	1.54E-05	0.00035433	DEG
Bpev01.c0281.g0077.m0001	-2.04275438	4.11E-11	5.02E-09	DEG
Bpev01.c0645.g0027.m0001	-0.903201771	0.00690266	0.04680044	DEG
Bpev01.c0044.g0068.m0001	0.898215969	0.00648441	0.04463281	DEG
Bpev01.c1079.g0002.m0001	1.813862869	0.00170315	0.01591212	DEG
Bpev01.c0051.g0172.m0001	-1.875331124	0.00180269	0.01668845	DEG
Bpev01.c0771.g0002.m0001	-1.018022016	0.00603356	0.04214044	DEG
Bpev01.c0162.g0005.m0001	1.648850078	2.31E-06	7.13E-05	DEG
Bpev01.c0759.g0007.m0001	-2.823976622	1.24E-05	0.00029687	DEG
Bpev01.c1968.g0006.m0001	1.151935072	0.00392685	0.0305989	DEG
Bpev01.c0422.g0024.m0001	-2.06522615	8.78E-06	0.00022239	DEG
Bpev01.c0154.g0078.m0001	4.001329323	1.96E-07	8.62E-06	DEG
Bpev01.c0126.g0032.m0001	3.864430733	7.93E-06	0.00020328	DEG
Bpev01.c1359.g0001.m0001	-1.050416286	0.00180896	0.01673602	DEG
Bpev01.c0383.g0017.m0001	-1.083002305	0.00056039	0.00656217	DEG
Bpev01.c1599.g0003.m0001	-1.212310096	0.00010622	0.00170314	DEG
Bpev01.c0484.g0020.m0001	-2.65275791	0.00100852	0.01039911	DEG
Bpev01.c3334.g0001.m0001	3.69851397	0.00013291	0.0020594	DEG
Bpev01.c0172.g0001.m0003	0.830133137	0.00697836	0.04714821	DEG
Bpev01.c0613.g0010.m0001	0.89587424	0.00736677	0.04905803	DEG
Bpev01.c1837.g0003.m0001	-1.143734557	0.00014565	0.00220798	DEG
Bpev01.c0094.g0001.m0001	1.730172222	8.12E-08	4.11E-06	DEG
Bpev01.c1584.g0011.m0001	3.754907518	0.00079999	0.00866793	DEG
Bpev01.c1814.g0005.m0001	1.535649939	0.00113696	0.01145877	DEG
Bpev01.c1031.g0009.m0001	-3.672153756	5.42E-13	9.72E-11	DEG
Bpev01.c0135.g0010.m0001	2.896270169	4.16E-19	2.55E-16	DEG
Bpev01.c0505.g0033.m0001	1.273913224	0.00017147	0.00252267	DEG
Bpev01.c0956.g0008.m0001	2.1162438	0.00506397	0.0369829	DEG
Bpev01.c0884.g0008.m0001	-1.451790321	1.85E-05	0.00041076	DEG
Bpev01.c2692.g0001.m0001	-1.194067776	0.00095713	0.00999533	DEG
Bpev01.c0029.g0154.m0001	-0.947819729	0.00395558	0.03078364	DEG
Bpev01.c2361.g0013.m0001	1.159620161	0.00025625	0.00347001	DEG
Bpev01.c1800.g0003.m0001	3.144676523	7.61E-07	2.81E-05	DEG
Bpev01.c0379.g0003.m0001	-1.153778132	0.00230305	0.0201724	DEG
Bpev01.c0366.g0007.m0001	-0.88046864	0.00646965	0.04457289	DEG
Bpev01.c0516.g0003.m0001	4.146912623	4.48E-05	0.00084523	DEG
Bpev01.c0134.g0035.m0001	-1.450768924	2.06E-06	6.48E-05	DEG
Bpev01.c0671.g0021.m0001	-2.0121242	0.00053371	0.00634371	DEG

Bpev01.c0229.g0027.m0001	-0.948018394	0.00366254	0.02911733	DEG
Bpev01.c0149.g0014.m0001	-5.431691402	1.32E-06	4.47E-05	DEG
Bpev01.c1770.g0004.m0001	5.599360326	0.00108333	0.01100113	DEG
Bpev01.c0042.g0011.m0001	1.238369038	0.00025516	0.00346003	DEG
Bpev01.c1577.g0016.m0001	-1.471912746	0.00011263	0.00179329	DEG
Bpev01.c1653.g0011.m0001	4.409395767	0.00347288	0.02793395	DEG
Bpev01.c2361.g0006.m0001	3.243711962	1.10E-07	5.26E-06	DEG
Bpev01.c1891.g0002.m0001	1.191252765	0.00222658	0.01961939	DEG
Bpev01.c0363.g0011.m0001	1.563848627	1.10E-06	3.86E-05	DEG
Bpev01.c0051.g0184.m0001	1.07843734	0.00204766	0.01838394	DEG
Bpev01.c0082.g0034.m0001	-1.372349398	1.26E-05	0.00029982	DEG
Bpev01.c1029.g0016.m0001	0.862242502	0.00736434	0.04905803	DEG
Bpev01.c0029.g0033.m0001	-0.910152307	0.00575254	0.04061161	DEG
Bpev01.c0578.g0008.m0001	1.20816177	0.0001361	0.00209773	DEG
Bpev01.c0418.g0002.m0001	-1.564313099	0.00020055	0.00284809	DEG
Bpev01.c0483.g0022.m0001	-1.992132103	8.92E-08	4.38E-06	DEG
Bpev01.c0395.g0023.m0001	-2.234420503	0.00233081	0.02034295	DEG
Bpev01.c0106.g0009.m0001	-0.923308694	0.00292676	0.02440817	DEG
Bpev01.c0155.g0038.m0001	4.18216143	0.00014094	0.00215421	DEG
Bpev01.c0349.g0061.m0001	-1.189912447	0.00084518	0.00909888	DEG
Bpev01.c1699.g0003.m0001	3.230914564	1.12E-05	0.00027156	DEG
Bpev01.c0630.g0003.m0001	1.964212051	0.00490221	0.03614761	DEG
Bpev01.c0095.g0020.m0001	-1.239819063	6.75E-05	0.00117634	DEG
Bpev01.c1086.g0006.m0001	-0.848050158	0.00692657	0.04689633	DEG
Bpev01.c1229.g0003.m0001	-2.522969698	0.00024349	0.00333849	DEG
Bpev01.c0320.g0019.m0001	-1.845206484	0.00039904	0.0050116	DEG
Bpev01.c0594.g0001.m0001	1.723236714	0.00276784	0.0233742	DEG
Bpev01.c0541.g0010.m0001	1.768222497	0.00214079	0.01902862	DEG
Bpev01.c1060.g0035.m0001	-1.191062785	0.0001957	0.00279947	DEG
Bpev01.c0716.g0002.m0001	-5.075225543	0.00065381	0.00743437	DEG
Bpev01.c0052.g0046.m0001	-1.718096521	1.52E-06	5.00E-05	DEG
Bpev01.c1078.g0001.m0001	4.711008818	0.0002376	0.00326557	DEG
Bpev01.c0699.g0014.m0001	-2.070163018	4.27E-10	4.19E-08	DEG
Bpev01.c0616.g0017.m0001	-1.840924644	0.00196728	0.01785839	DEG
Bpev01.c2315.g0006.m0001	-3.973245934	1.14E-09	1.01E-07	DEG
Bpev01.c0050.g0094.m0001	4.543442062	1.96E-10	2.10E-08	DEG
Bpev01.c1475.g0011.m0001	-1.86601775	1.73E-08	1.07E-06	DEG
Bpev01.c0195.g0016.m0001	-1.266612297	0.0003654	0.00465268	DEG
Bpev01.c0051.g0133.m0001	2.309594591	2.56E-10	2.66E-08	DEG
Bpev01.c0451.g0024.m0001	3.176891625	3.09E-17	1.44E-14	DEG
Bpev01.c1030.g0007.m0001	3.506594052	0.00061775	0.00709835	DEG
Bpev01.c0852.g0003.m0001	5.82463772	7.23E-19	4.17E-16	DEG
Bpev01.c0074.g0020.m0001	5.318267255	3.62E-33	2.13E-29	DEG

Bpev01.c1154.g0002.m0001	-1.876515933	0.00148494	0.01423241	DEG
Bpev01.c0038.g0024.m0001	1.88491861	4.23E-08	2.35E-06	DEG
Bpev01.c1478.g0001.m0001	1.667988698	0.00042933	0.00532615	DEG
Bpev01.c0120.g0043.m0001	-1.849299628	5.57E-08	2.98E-06	DEG
Bpev01.c0016.g0141.m0001	-3.619759304	0.00092445	0.00973006	DEG
Bpev01.c0080.g0085.m0001	-1.849904185	2.53E-06	7.70E-05	DEG
Bpev01.c1163.g0018.m0001	3.006929072	2.77E-05	0.00057096	DEG
Bpev01.c0080.g0123.m0001	1.931333281	6.32E-05	0.00111157	DEG
Bpev01.c1275.g0004.m0001	1.70711828	0.00096636	0.01008102	DEG
Bpev01.c0276.g0004.m0001	-1.700646253	1.44E-05	0.00033405	DEG
Bpev01.c0449.g0055.m0001	-1.825363085	1.41E-06	4.73E-05	DEG
Bpev01.c0598.g0006.m0001	1.791563185	0.00499362	0.03659626	DEG
Bpev01.c1792.g0002.m0001	-2.590695344	1.39E-05	0.00032461	DEG
Bpev01.c0016.g0088.m0001	2.743696214	4.94E-05	0.00091714	DEG
Bpev01.c0001.g0064.m0001	0.966576589	0.0047344	0.03523156	DEG
Bpev01.c0981.g0007.m0001	-0.935749245	0.00502255	0.03674423	DEG
Bpev01.c0896.g0005.m0001	-1.673923964	0.00024631	0.00335857	DEG
Bpev01.c1225.g0008.m0001	-1.51153608	6.28E-05	0.00110697	DEG
Bpev01.c0774.g0016.m0001	-0.89180023	0.00493473	0.03630068	DEG
Bpev01.c0052.g0076.m0001	3.937995659	6.12E-05	0.00108577	DEG
Bpev01.c1536.g0007.m0001	-1.813442974	5.77E-05	0.00103632	DEG
Bpev01.c0210.g0013.m0001	-1.620838904	0.00328154	0.0267013	DEG
Bpev01.c1938.g0006.m0001	1.334413244	0.00109424	0.01109653	DEG
Bpev01.c0080.g0063.m0002	-1.350451342	2.28E-05	0.00048327	DEG
Bpev01.c0131.g0002.m0001	-1.263514447	0.00012847	0.00199889	DEG
Bpev01.c0022.g0072.m0001	-1.955670839	0.002111	0.01881497	DEG
Bpev01.c0042.g0003.m0001	1.06319816	0.00099168	0.01026873	DEG
Bpev01.c0257.g0020.m0001	-1.261927119	0.0001413	0.00215871	DEG
Bpev01.c4257.g0001.m0001	-0.993675465	0.00633974	0.04384203	DEG
Bpev01.c0115.g0031.m0001	-2.263689071	4.76E-10	4.58E-08	DEG
Bpev01.c0949.g0008.m0002	1.628249244	0.00106484	0.01084743	DEG
Bpev01.c1383.g0003.m0001	2.275974383	8.86E-06	0.00022405	DEG
Bpev01.c1526.g0007.m0001	-2.588675382	1.17E-14	3.06E-12	DEG
Bpev01.c0800.g0023.m0001	-0.886404041	0.0042342	0.03231806	DEG
Bpev01.c0949.g0009.m0001	5.234995771	3.98E-36	1.17E-31	DEG
Bpev01.c0054.g0003.m0001	-1.189440594	0.00037588	0.00476549	DEG
Bpev01.c0732.g0005.m0001	5.318374715	5.70E-26	9.32E-23	DEG
Bpev01.c0445.g0013.m0001	1.034469442	0.00706124	0.04757976	DEG
Bpev01.c0278.g0018.m0001	3.05264285	0.00030214	0.00396904	DEG
Bpev01.c0080.g0084.m0001	-1.197106131	0.00051319	0.00615389	DEG
Bpev01.c0127.g0096.m0001	-1.73468238	0.00590546	0.04146219	DEG
Bpev01.c0454.g0009.m0001	-1.468238709	1.74E-05	0.00039219	DEG
Bpev01.c0357.g0073.m0001	-1.529267748	4.95E-06	0.00013575	DEG

Bpev01.c0663.g0007.m0001	-1.405688031	0.00136478	0.01326878	DEG
Bpev01.c0791.g0006.m0001	5.614118435	2.91E-08	1.69E-06	DEG
Bpev01.c1287.g0001.m0001	-1.75017425	0.00278739	0.02349885	DEG
Bpev01.c1243.g0009.m0001	-2.973490216	8.76E-05	0.00145316	DEG
Bpev01.c0224.g0027.m0001	-1.646917227	2.26E-07	9.76E-06	DEG
Bpev01.c2091.g0001.m0001	4.190283056	0.00505927	0.03695778	DEG
Bpev01.c1649.g0012.m0002	1.231058567	0.00010906	0.00174289	DEG
Bpev01.c1033.g0003.m0001	-1.309132001	0.00027139	0.00364314	DEG
Bpev01.c0264.g0005.m0001	-2.392976669	3.52E-05	0.00069457	DEG
Bpev01.c0650.g0003.m0001	-2.680960959	3.78E-17	1.66E-14	DEG
Bpev01.c1763.g0007.m0001	-2.614843273	0.00088619	0.00944213	DEG
Bpev01.c2228.g0004.m0001	-1.316654762	0.00014457	0.00219611	DEG
Bpev01.c0159.g0001.m0001	-1.271002335	3.38E-05	0.00067325	DEG
Bpev01.c0089.g0040.m0001	-1.088229454	0.00079175	0.00860405	DEG
Bpev01.c0357.g0039.m0001	-1.348892022	0.00561478	0.0399163	DEG
Bpev01.c1759.g0006.m0001	-2.411715906	2.61E-06	7.90E-05	DEG
Bpev01.c1739.g0001.m0001	2.503100219	6.11E-09	4.35E-07	DEG
Bpev01.c0887.g0005.m0001	-1.153150874	0.00017638	0.00257683	DEG
Bpev01.c0127.g0009.m0001	-0.953995167	0.00221447	0.01956538	DEG
Bpev01.c1617.g0003.m0001	1.311695743	0.00101654	0.01044729	DEG
Bpev01.c1502.g0001.m0001	3.070087316	7.46E-06	0.00019222	DEG
Bpev01.c1238.g0015.m0001	-0.988379745	0.00738757	0.04917086	DEG
Bpev01.c0010.g0003.m0001	1.541352315	0.00199225	0.0180024	DEG
Bpev01.c0918.g0017.m0001	-1.422102137	9.92E-06	0.00024459	DEG
Bpev01.c1127.g0009.m0001	-1.098570297	0.00084325	0.00908324	DEG
Bpev01.c0357.g0062.m0001	-2.5223066	0.00726649	0.04862882	DEG
Bpev01.c0484.g0027.m0001	2.704104998	0.00216726	0.01922905	DEG
Bpev01.c0394.g0022.m0001	-3.492033127	1.32E-07	6.17E-06	DEG
Bpev01.c0142.g0036.m0001	-1.379158659	4.91E-05	0.00091362	DEG
Bpev01.c0146.g0025.m0001	-2.023533967	1.46E-05	0.00033961	DEG
Bpev01.c0710.g0004.m0001	1.387841321	1.82E-05	0.0004045	DEG
Bpev01.c1778.g0005.m0001	-1.854345723	3.65E-09	2.76E-07	DEG
Bpev01.c1161.g0013.m0001	2.066875612	1.76E-08	1.09E-06	DEG
Bpev01.c0232.g0005.m0001	0.876918402	0.00622394	0.04319341	DEG
Bpev01.c0102.g0002.m0001	4.629010831	2.17E-15	6.78E-13	DEG
Bpev01.c1000.g0009.m0001	-2.222738611	0.00666386	0.04556459	DEG
Bpev01.c0045.g0055.m0001	1.625978459	0.00023199	0.00320335	DEG
Bpev01.c0052.g0017.m0002	-0.825723834	0.00744395	0.04944737	DEG
Bpev01.c0468.g0001.m0001	2.591096381	3.95E-10	3.91E-08	DEG
Bpev01.c0146.g0044.m0001	-1.079880883	0.00063216	0.00723001	DEG
Bpev01.c0281.g0029.m0001	-1.483404513	0.00172661	0.01609045	DEG
Bpev01.c0984.g0029.m0001	-2.546940637	1.48E-12	2.44E-10	DEG
Bpev01.c1256.g0002.m0001	-1.089713255	0.00726611	0.04862882	DEG

Bpev01.c0261.g0034.m0001	-0.950867326	0.00366757	0.02913375	DEG
Bpev01.c0597.g0011.m0001	-0.931183888	0.00374429	0.02955176	DEG
Bpev01.c0062.g0037.m0001	2.59093546	4.83E-09	3.55E-07	DEG
Bpev01.c1880.g0002.m0001	1.407099792	0.00132508	0.01295116	DEG
Bpev01.c0094.g0258.m0001	-2.342726436	0.0001728	0.00253718	DEG
Bpev01.c0253.g0022.m0001	2.806614177	0.0060261	0.04209835	DEG
Bpev01.c0671.g0017.m0001	0.951970442	0.00389858	0.03045204	DEG
Bpev01.c0147.g0004.m0001	-2.500328696	0.00154568	0.01470216	DEG
Bpev01.c0403.g0032.m0001	1.347541575	0.00059883	0.00691603	DEG
Bpev01.c1762.g0002.m0001	-3.267861872	0.00109723	0.01112045	DEG
Bpev01.c1104.g0014.m0001	-2.200741088	2.22E-11	2.79E-09	DEG
Bpev01.c1040.g0033.m0001	1.434714911	0.00068751	0.00772507	DEG
Bpev01.c2742.g0001.m0001	2.431760281	0.00380271	0.02990751	DEG
Bpev01.c1051.g0010.m0001	4.301481001	5.20E-09	3.79E-07	DEG
Bpev01.c2275.g0002.m0001	-1.506678168	0.00109375	0.01109541	DEG
Bpev01.c0566.g0009.m0001	-3.401340006	0.00520313	0.03779296	DEG
Bpev01.c0030.g0023.m0001	-2.488341497	0.00286516	0.02401006	DEG
Bpev01.c0206.g0010.m0001	2.645858354	4.45E-11	5.37E-09	DEG
Bpev01.c1863.g0001.m0001	2.055503948	1.93E-06	6.16E-05	DEG
Bpev01.c0214.g0062.m0001	1.268218814	0.00085734	0.009208	DEG
Bpev01.c0473.g0031.m0001	-2.958275924	5.15E-06	0.00014072	DEG
Bpev01.c0826.g0010.m0002	1.778153069	5.54E-07	2.14E-05	DEG
Bpev01.c0706.g0006.m0001	1.921472128	6.34E-08	3.31E-06	DEG
Bpev01.c0343.g0021.m0001	5.813990928	1.94E-09	1.59E-07	DEG
Bpev01.c1233.g0004.m0001	-1.064970541	0.00191219	0.01748224	DEG
Bpev01.c1405.g0004.m0001	1.16702273	0.00352683	0.02825697	DEG
Bpev01.c0038.g0126.m0001	-1.82089913	4.27E-07	1.71E-05	DEG
Bpev01.c0945.g0004.m0001	2.804372294	4.67E-06	0.00012907	DEG
Bpev01.c0015.g0172.m0001	1.543481095	0.00100784	0.01039911	DEG
Bpev01.c0787.g0003.m0001	1.742264508	0.00126541	0.01247148	DEG
Bpev01.c0329.g0021.m0001	-1.58681402	0.00186209	0.01714133	DEG
Bpev01.c0016.g0152.m0001	-1.964128195	0.00206226	0.01848685	DEG
Bpev01.c0015.g0146.m0001	-1.339151786	0.00068329	0.00769229	DEG
Bpev01.c0026.g0010.m0001	-1.028311622	0.00298709	0.02479193	DEG
Bpev01.c0455.g0029.m0001	2.543385364	2.37E-13	4.64E-11	DEG
Bpev01.c1041.g0002.m0001	-2.439032135	2.89E-08	1.68E-06	DEG
Bpev01.c0727.g0024.m0001	1.221647469	0.00019913	0.00283202	DEG
Bpev01.c0597.g0021.m0001	-1.195809138	0.00049921	0.00602304	DEG
Bpev01.c2000.g0009.m0001	-1.207712727	0.00014198	0.00216686	DEG
Bpev01.c0650.g0002.m0001	-3.009250751	9.98E-07	3.58E-05	DEG
Bpev01.c1086.g0007.m0001	-1.566122677	2.20E-05	0.00047089	DEG
Bpev01.c0352.g0003.m0001	2.932159602	0.00289629	0.02420895	DEG
Bpev01.c0328.g0007.m0001	-1.590977855	7.03E-06	0.00018337	DEG

Bpev01.c0142.g0073.m0001	7.838740918	3.49E-05	0.00069021	DEG
Bpev01.c0190.g0067.m0001	3.678775583	9.09E-06	0.00022806	DEG
Bpev01.c0050.g0113.m0001	2.230395863	8.55E-08	4.27E-06	DEG
Bpev01.c1347.g0001.m0001	-1.762961377	1.13E-05	0.00027227	DEG
Bpev01.c0120.g0017.m0001	-1.24136327	0.00033753	0.00434284	DEG
Bpev01.c0565.g0010.m0001	0.884667032	0.006424	0.04431023	DEG
Bpev01.c0484.g0028.m0001	4.198142762	6.82E-11	7.94E-09	DEG
Bpev01.c0800.g0015.m0001	-1.315467529	0.00093823	0.00984693	DEG
Bpev01.c1828.g0001.m0003	-1.242188987	0.00039275	0.00494959	DEG
Bpev01.c0597.g0014.m0001	-0.988915092	0.0020501	0.01839467	DEG
Bpev01.c0127.g0077.m0001	2.080728602	0.00089981	0.00953547	DEG
Bpev01.c1627.g0007.m0001	-2.518652424	0.00013559	0.00209199	DEG
Bpev01.c1300.g0004.m0001	-1.541794478	0.0012042	0.01197353	DEG
Bpev01.c0077.g0011.m0002	1.449403924	0.00029727	0.00392107	DEG
Bpev01.c0866.g0014.m0001	-1.14669927	0.00054139	0.00639563	DEG
Bpev01.c0015.g0121.m0001	-1.081437134	0.00192429	0.01756016	DEG
Bpev01.c1061.g0010.m0001	1.527075684	4.34E-06	0.00012118	DEG
Bpev01.c0545.g0012.m0001	-2.096443383	5.36E-10	5.09E-08	DEG
Bpev01.c0146.g0046.m0001	1.372605647	0.00024283	0.00333274	DEG
Bpev01.c1530.g0008.m0001	2.013144755	0.000334	0.00430496	DEG
Bpev01.c0155.g0014.m0001	1.064817162	0.00136114	0.01324661	DEG
Bpev01.c1790.g0002.m0001	1.47749999	0.00077889	0.00849255	DEG
Bpev01.c0732.g0007.m0001	3.428274246	7.87E-06	0.00020171	DEG
Bpev01.c0451.g0020.m0001	2.268378059	0.00016278	0.00241777	DEG
Bpev01.c0812.g0009.m0001	0.876492087	0.00606807	0.04229114	DEG
Bpev01.c0018.g0016.m0001	-0.848708243	0.00729187	0.04873218	DEG
Bpev01.c0148.g0010.m0001	4.264421641	2.87E-20	2.29E-17	DEG
Bpev01.c0555.g0002.m0001	1.009567395	0.0072901	0.04873143	DEG
Bpev01.c0470.g0044.m0001	-1.127724098	0.00065496	0.00744457	DEG
Bpev01.c0115.g0068.m0001	0.947184898	0.00204428	0.01836233	DEG
Bpev01.c1623.g0016.m0001	-1.192424272	0.00301729	0.02497219	DEG
Bpev01.c1477.g0004.m0001	3.123525611	0.00092406	0.00972941	DEG
Bpev01.c0038.g0096.m0001	4.029111743	3.25E-17	1.47E-14	DEG
Bpev01.c0579.g0016.m0001	3.627632163	7.35E-14	1.57E-11	DEG
Bpev01.c0237.g0047.m0002	3.142044719	1.81E-10	1.96E-08	DEG
Bpev01.c1885.g0001.m0001	-2.16473189	1.16E-05	0.00027851	DEG
Bpev01.c0150.g0003.m0001	-2.156891355	0.00632513	0.04376157	DEG
Bpev01.c0154.g0089.m0001	4.556723355	2.87E-12	4.40E-10	DEG
Bpev01.c2710.g0002.m0001	0.98692454	0.00379559	0.02987502	DEG
Bpev01.c1351.g0004.m0001	0.916985063	0.00443249	0.03349284	DEG
Bpev01.c2388.g0001.m0001	-3.000326766	0.00230673	0.02019861	DEG
Bpev01.c0016.g0162.m0001	-1.181746694	0.00050033	0.00603406	DEG
Bpev01.c0147.g0009.m0001	-1.516618069	0.00354787	0.02838197	DEG

Bpev01.c0540.g0031.m0001	1.6890034	0.00011076	0.00176641	DEG
Bpev01.c1517.g0002.m0001	-1.867814456	0.00160006	0.01514114	DEG
Bpev01.c1561.g0001.m0001	0.909785235	0.00490293	0.03614761	DEG
Bpev01.c1152.g0015.m0001	-1.397717338	0.00032402	0.00419655	DEG
Bpev01.c0960.g0002.m0001	-1.967827295	1.30E-05	0.00030864	DEG
Bpev01.c0617.g0018.m0001	1.398497135	0.0018384	0.01697108	DEG
Bpev01.c0343.g0017.m0001	-2.54852259	0.00253803	0.02179612	DEG
Bpev01.c0845.g0009.m0001	1.277632308	4.17E-05	0.00079651	DEG
Bpev01.c2587.g0001.m0001	2.007774676	0.00195428	0.01777276	DEG
Bpev01.c1334.g0008.m0001	-0.924356953	0.00368825	0.0292316	DEG
Bpev01.c1709.g0005.m0001	2.616692314	0.00375041	0.02958423	DEG
Bpev01.c0577.g0023.m0001	9.008500566	0.00026105	0.0035269	DEG
Bpev01.c0134.g0106.m0001	0.998760995	0.00628331	0.04354384	DEG
Bpev01.c0114.g0054.m0001	2.375958277	4.81E-07	1.89E-05	DEG
Bpev01.c1833.g0004.m0001	-2.260605454	5.48E-05	0.00099263	DEG
Bpev01.c2271.g0001.m0001	-3.086701166	7.43E-06	0.00019197	DEG
Bpev01.c2235.g0004.m0001	2.592115719	1.79E-06	5.77E-05	DEG
Bpev01.c0429.g0030.m0001	-1.178602535	0.00015845	0.00236657	DEG
Bpev01.c2282.g0005.m0001	1.596263825	0.00028792	0.00382317	DEG
Bpev01.c3687.g0001.m0001	-1.351074131	0.00019512	0.00279663	DEG
Bpev01.c0090.g0017.m0003	-1.02974579	0.00708003	0.04768451	DEG
Bpev01.c0162.g0017.m0001	3.594652117	0.00342313	0.02760921	DEG
Bpev01.c0617.g0005.m0001	-1.563158248	3.07E-06	9.11E-05	DEG
Bpev01.c0522.g0025.m0001	-4.777840549	0.00364508	0.02902556	DEG
Bpev01.c0279.g0033.m0001	1.472971223	9.36E-06	0.00023358	DEG
Bpev01.c1923.g0001.m0001	-2.642225104	0.00714891	0.04807145	DEG
Bpev01.c0017.g0015.m0001	-1.205606098	0.00293787	0.02446622	DEG
Bpev01.c0999.g0009.m0001	2.706753574	0.00066606	0.00753002	DEG
Bpev01.c0129.g0022.m0001	1.226559695	0.0008139	0.00880902	DEG
Bpev01.c0015.g0157.m0001	0.913930708	0.00589835	0.04144189	DEG
Bpev01.c0449.g0016.m0001	-0.994836317	0.00663629	0.04548678	DEG
Bpev01.c0389.g0010.m0001	1.157194724	0.0061339	0.04264902	DEG
Bpev01.c1789.g0001.m0001	3.195388086	1.68E-05	0.00037935	DEG
Bpev01.c0578.g0017.m0001	1.548572899	4.39E-07	1.74E-05	DEG
Bpev01.c0397.g0012.m0001	2.872021587	1.80E-15	5.81E-13	DEG
Bpev01.c0142.g0048.m0002	1.113778922	0.00262557	0.02241061	DEG
Bpev01.c0668.g0011.m0001	1.312447187	0.00047341	0.00576369	DEG
Bpev01.c0001.g0013.m0001	-1.624145043	6.65E-05	0.00116189	DEG
Bpev01.c0616.g0024.m0001	-1.310173155	3.75E-05	0.00072881	DEG
Bpev01.c0333.g0016.m0001	-2.205263728	4.26E-06	0.00011953	DEG
Bpev01.c0399.g0007.m0001	-1.220983728	5.41E-05	0.00098332	DEG
Bpev01.c0298.g0040.m0001	-1.52126628	0.00022346	0.00310452	DEG
Bpev01.c0401.g0032.m0001	1.418941327	0.00029063	0.00385404	DEG

Bpev01.c0934.g0013.m0001	3.248411321	9.08E-12	1.25E-09	DEG
Bpev01.c0263.g0010.m0001	3.009775175	1.48E-12	2.44E-10	DEG
Bpev01.c0854.g0010.m0001	3.136221411	4.07E-19	2.55E-16	DEG
Bpev01.c0038.g0113.m0002	1.798355714	2.43E-06	7.45E-05	DEG
Bpev01.c0214.g0029.m0001	-1.914245926	9.46E-10	8.57E-08	DEG
Bpev01.c0467.g0020.m0001	-2.077350014	9.60E-05	0.00156626	DEG
Bpev01.c0485.g0015.m0001	-1.528949012	0.00748912	0.04970068	DEG
Bpev01.c1142.g0003.m0001	6.412167505	0.00590276	0.0414531	DEG
Bpev01.c0000.g0153.m0001	-1.906425896	0.00470297	0.03506684	DEG
Bpev01.c1838.g0003.m0001	1.314899501	0.00117404	0.01174394	DEG
Bpev01.c0951.g0008.m0001	-3.869732847	3.87E-06	0.00011023	DEG
Bpev01.c0843.g0002.m0002	-1.187976938	0.00599662	0.04195585	DEG
Bpev01.c0402.g0020.m0001	-2.796783478	5.83E-07	2.23E-05	DEG
Bpev01.c0281.g0064.m0001	-1.356992045	0.0053093	0.03833714	DEG
Bpev01.c1484.g0004.m0001	-1.025612577	0.00245017	0.02117131	DEG
Bpev01.c0036.g0003.m0001	6.84965221	0.00245242	0.02118447	DEG
Bpev01.c0403.g0005.m0001	-2.698611065	1.36E-05	0.00031987	DEG
Bpev01.c0242.g0014.m0001	2.999217661	4.53E-13	8.24E-11	DEG
Bpev01.c0817.g0020.m0001	-0.958660995	0.00242053	0.02095827	DEG
Bpev01.c0022.g0034.m0001	1.467775441	0.00024539	0.00335039	DEG
Bpev01.c0001.g0029.m0001	-1.204849023	0.00263598	0.02247343	DEG
Bpev01.c0680.g0004.m0001	-2.650361731	2.83E-12	4.36E-10	DEG
Bpev01.c0961.g0006.m0001	1.127125979	0.00060832	0.00700644	DEG
Bpev01.c0018.g0030.m0001	4.834994936	1.14E-06	3.98E-05	DEG
Bpev01.c1040.g0034.m0002	1.462021736	0.00013746	0.00211424	DEG
Bpev01.c0582.g0001.m0001	-2.57573842	0.00138159	0.01339232	DEG
Bpev01.c0094.g0065.m0001	-1.665897959	0.00195575	0.01777567	DEG
Bpev01.c0177.g0049.m0001	-1.318912881	8.42E-05	0.00140861	DEG
Bpev01.c0415.g0023.m0001	-2.075979462	5.79E-11	6.84E-09	DEG
Bpev01.c0127.g0068.m0001	-1.699090712	2.50E-05	0.00052229	DEG
Bpev01.c0318.g0003.m0001	3.257127587	2.94E-12	4.48E-10	DEG
Bpev01.c1227.g0006.m0001	1.649957624	0.00057494	0.00668733	DEG
Bpev01.c0774.g0017.m0002	3.59806755	1.20E-21	1.10E-18	DEG
Bpev01.c1034.g0007.m0001	-4.86191621	0.00029189	0.00386371	DEG
Bpev01.c0425.g0005.m0001	2.89964907	3.97E-05	0.00076279	DEG
Bpev01.c0015.g0243.m0001	-0.885107972	0.00423922	0.03232239	DEG
Bpev01.c0684.g0007.m0001	-2.247753017	5.58E-07	2.15E-05	DEG
Bpev01.c0147.g0020.m0001	-3.643297163	0.00015764	0.00235805	DEG
Bpev01.c1106.g0007.m0001	1.13975242	0.00174769	0.01627143	DEG
Bpev01.c0407.g0017.m0001	-2.495547616	2.42E-15	7.42E-13	DEG
Bpev01.c0349.g0049.m0002	1.913576404	2.03E-05	0.00044098	DEG
Bpev01.c0832.g0006.m0001	2.151479505	0.0002984	0.00393221	DEG
Bpev01.c0437.g0013.m0001	-1.185548259	0.00100874	0.01039911	DEG

Bpev01.c0652.g0023.m0002	-1.716102286	2.58E-05	0.0005374	DEG
Bpev01.c0162.g0004.m0001	-1.014989037	0.00230964	0.02021814	DEG
Bpev01.c1935.g0006.m0001	-2.26857014	0.00553263	0.03956573	DEG
Bpev01.c1150.g0002.m0001	-1.50919849	1.42E-06	4.76E-05	DEG
Bpev01.c0142.g0045.m0001	-1.099260748	0.00168438	0.01578699	DEG
Bpev01.c0161.g0031.m0001	1.175034583	0.00094386	0.00989185	DEG
Bpev01.c0170.g0029.m0001	-1.707845138	1.71E-06	5.56E-05	DEG
Bpev01.c0276.g0006.m0001	-4.783371738	1.78E-08	1.10E-06	DEG
Bpev01.c0328.g0006.m0001	1.057828465	0.00516854	0.03758812	DEG
Bpev01.c0480.g0073.m0001	-2.060169435	1.45E-09	1.23E-07	DEG
Bpev01.c0470.g0029.m0001	-2.721996996	0.00733935	0.04894954	DEG
Bpev01.c0004.g0009.m0001	-2.003095671	0.00271953	0.02304555	DEG
Bpev01.c0624.g0012.m0001	2.153931333	3.13E-05	0.00062902	DEG
Bpev01.c0022.g0035.m0001	1.34571827	0.00182795	0.01688325	DEG
Bpev01.c0574.g0048.m0001	1.022473402	0.0025753	0.02205185	DEG
Bpev01.c0016.g0040.m0001	1.388242499	0.00348415	0.02800861	DEG
Bpev01.c0426.g0018.m0001	1.382028411	0.000212	0.00297903	DEG
Bpev01.c0449.g0026.m0001	-1.267064598	0.00030426	0.00398623	DEG
Bpev01.c1960.g0001.m0002	1.745664522	3.30E-06	9.69E-05	DEG
Bpev01.c2510.g0005.m0001	-2.521185085	1.14E-06	3.98E-05	DEG
Bpev01.c0230.g0022.m0001	-1.950937672	0.00406732	0.03141077	DEG
Bpev01.c0015.g0129.m0001	-1.455353131	2.92E-05	0.00059477	DEG
Bpev01.c0214.g0035.m0001	-0.989057385	0.00235289	0.02049307	DEG
Bpev01.c0062.g0046.m0001	0.903994231	0.00541849	0.03900249	DEG
Bpev01.c0585.g0005.m0001	3.461279602	1.67E-08	1.04E-06	DEG
Bpev01.c0167.g0003.m0001	-1.088429758	0.00688591	0.04674065	DEG
Bpev01.c0803.g0013.m0001	-2.663199907	0.00113212	0.01141776	DEG
Bpev01.c0278.g0036.m0001	1.334378984	0.00024478	0.00335006	DEG
Bpev01.c0022.g0083.m0001	3.405588101	3.53E-06	0.0001027	DEG
Bpev01.c1272.g0024.m0001	-2.567874075	5.48E-08	2.93E-06	DEG
Bpev01.c0485.g0014.m0001	-1.978043435	0.00068541	0.0077103	DEG
Bpev01.c0037.g0039.m0001	2.272420533	5.10E-05	0.0009375	DEG
Bpev01.c0274.g0057.m0001	-1.299674184	0.00012849	0.00199889	DEG
Bpev01.c1238.g0010.m0001	-1.647454515	4.42E-07	1.76E-05	DEG
Bpev01.c0224.g0015.m0001	0.889047086	0.00328099	0.0267013	DEG
Bpev01.c0425.g0049.m0001	1.29950229	5.33E-05	0.00097326	DEG
Bpev01.c0045.g0035.m0001	-1.871335131	0.00414664	0.03184538	DEG
Bpev01.c0389.g0062.m0001	2.025497715	7.71E-05	0.00130816	DEG
Bpev01.c0588.g0021.m0001	-2.091906117	0.00050475	0.00607495	DEG
Bpev01.c1124.g0006.m0001	-4.298044803	2.29E-27	4.81E-24	DEG
Bpev01.c3046.g0003.m0001	1.843428704	1.79E-05	0.00040058	DEG
Bpev01.c1031.g0015.m0002	-1.866179114	6.32E-07	2.40E-05	DEG
Bpev01.c0269.g0002.m0001	-0.834990588	0.00590053	0.04144734	DEG

Bpev01.c0617.g0006.m0001	-1.979114369	0.00273568	0.02315258	DEG
Bpev01.c0566.g0028.m0001	1.580170332	2.83E-05	0.00058096	DEG
Bpev01.c1567.g0001.m0001	-1.971717023	3.18E-09	2.47E-07	DEG
Bpev01.c0427.g0003.m0001	2.710788377	0.00131374	0.01287884	DEG
Bpev01.c0322.g0012.m0002	4.772120094	8.88E-24	1.09E-20	DEG
Bpev01.c0000.g0206.m0001	-0.889454012	0.0038987	0.03045204	DEG
Bpev01.c0765.g0002.m0001	-1.331173513	5.48E-05	0.00099254	DEG
Bpev01.c1851.g0002.m0001	3.336893399	4.28E-15	1.22E-12	DEG
Bpev01.c0018.g0090.m0001	-1.040279484	0.00061628	0.00708421	DEG
Bpev01.c0082.g0084.m0001	3.062351907	2.87E-07	1.20E-05	DEG
Bpev01.c0051.g0021.m0001	-1.97865268	4.67E-08	2.56E-06	DEG
Bpev01.c0962.g0028.m0001	1.867626627	2.70E-07	1.14E-05	DEG
Bpev01.c1159.g0002.m0001	2.274256063	1.00E-05	0.00024639	DEG
Bpev01.c0046.g0006.m0001	-2.138604803	0.00043629	0.00539889	DEG
Bpev01.c0154.g0017.m0001	-2.745864843	0.00081476	0.008815	DEG
Bpev01.c0191.g0008.m0001	-1.275723973	0.00019528	0.00279717	DEG
Bpev01.c1674.g0004.m0001	-1.370302322	0.00452172	0.03398386	DEG
Bpev01.c0015.g0104.m0001	-1.201651097	0.00043549	0.0053913	DEG
Bpev01.c1601.g0002.m0001	1.280020538	0.00057223	0.0066611	DEG
Bpev01.c1606.g0001.m0001	3.169235785	0.0064968	0.0446972	DEG
Bpev01.c0283.g0018.m0001	1.980848183	9.32E-05	0.00152958	DEG
Bpev01.c0015.g0142.m0001	-0.877676753	0.00441045	0.03336057	DEG
Bpev01.c0051.g0185.m0001	-1.503481317	2.09E-06	6.54E-05	DEG
Bpev01.c0886.g0007.m0001	1.292108177	4.18E-05	0.00079778	DEG
Bpev01.c1000.g0004.m0001	-1.210118634	0.0002738	0.00366378	DEG
Bpev01.c0062.g0062.m0001	-1.38044846	1.94E-05	0.00042574	DEG
Bpev01.c0281.g0089.m0001	-1.229778837	0.00622575	0.04319578	DEG
Bpev01.c1057.g0006.m0002	-1.285991566	0.0018742	0.0172205	DEG
Bpev01.c0328.g0005.m0001	-1.228314386	0.00681422	0.0463288	DEG
Bpev01.c0420.g0024.m0001	1.284035882	0.00016992	0.00250364	DEG
Bpev01.c3149.g0001.m0001	1.132307373	0.00477	0.03543377	DEG
Bpev01.c0934.g0011.m0001	1.680888302	0.00192155	0.01755697	DEG
Bpev01.c0213.g0049.m0001	4.289475658	6.55E-15	1.79E-12	DEG
Bpev01.c0514.g0008.m0001	-1.318315209	8.54E-05	0.0014252	DEG
Bpev01.c0015.g0175.m0001	-2.946125493	2.91E-13	5.56E-11	DEG
Bpev01.c0557.g0023.m0001	1.026654813	0.00242675	0.02099975	DEG
Bpev01.c0896.g0009.m0001	-2.204590989	6.03E-05	0.00107583	DEG
Bpev01.c1141.g0004.m0001	2.726404462	0.000774	0.00844876	DEG
Bpev01.c2528.g0001.m0001	-1.871321618	8.85E-07	3.23E-05	DEG
Bpev01.c0915.g0017.m0001	-1.127918955	0.00140822	0.01359248	DEG
Bpev01.c0015.g0014.m0001	-2.323291441	2.63E-11	3.26E-09	DEG
Bpev01.c0862.g0009.m0001	-1.16393005	0.00367217	0.0291624	DEG
Bpev01.c0366.g0013.m0001	-1.353476361	8.67E-06	0.00022024	DEG

Bpev01.c2300.g0005.m0001	2.341968108	1.75E-12	2.82E-10	DEG
Bpev01.c0038.g0083.m0001	2.319548711	9.48E-08	4.61E-06	DEG
Bpev01.c0327.g0054.m0001	-1.488539824	1.51E-06	5.00E-05	DEG
Bpev01.c3684.g0001.m0001	1.826905996	9.23E-07	3.34E-05	DEG
Bpev01.c0038.g0088.m0001	-2.275228628	0.00290106	0.02422076	DEG
Bpev01.c1092.g0005.m0001	1.066893654	0.00057859	0.00672454	DEG
Bpev01.c1140.g0010.m0001	-1.679680057	0.00480301	0.03560712	DEG
Bpev01.c0557.g0009.m0001	-1.019459083	0.00188401	0.01729988	DEG
Bpev01.c1593.g0003.m0001	-2.725840004	0.00066598	0.00753002	DEG
Bpev01.c1005.g0010.m0001	-1.399417706	0.00023303	0.00321457	DEG
Bpev01.c0863.g0008.m0001	-1.294216186	0.0002307	0.00319009	DEG
Bpev01.c0826.g0013.m0001	-1.76916548	1.83E-08	1.12E-06	DEG
Bpev01.c1238.g0001.m0001	1.99078643	0.00228434	0.0200383	DEG
Bpev01.c1577.g0026.m0001	3.515885902	0.0001432	0.00218237	DEG
Bpev01.c1076.g0006.m0001	1.390869184	4.67E-05	0.00087397	DEG
Bpev01.c3201.g0001.m0001	1.337174522	0.00108443	0.0110085	DEG
Bpev01.c1229.g0011.m0001	1.817565664	2.63E-07	1.11E-05	DEG
Bpev01.c0000.g0173.m0001	0.990188451	0.00131486	0.01288559	DEG
Bpev01.c0992.g0016.m0001	-0.984329084	0.00312904	0.02572349	DEG
Bpev01.c0023.g0015.m0001	-0.973318195	0.00398182	0.03094532	DEG
Bpev01.c0162.g0068.m0001	1.698776039	0.00086458	0.00926894	DEG
Bpev01.c1967.g0003.m0001	-1.120930356	0.00060015	0.00692318	DEG
Bpev01.c0219.g0003.m0001	1.765630084	6.43E-07	2.44E-05	DEG
Bpev01.c0778.g0020.m0001	3.917321373	4.05E-13	7.47E-11	DEG
Bpev01.c0848.g0041.m0001	-1.137774734	0.00101969	0.01047404	DEG
Bpev01.c0886.g0006.m0001	1.811711435	0.00250952	0.02160801	DEG
Bpev01.c0480.g0007.m0001	-2.05731634	2.49E-07	1.06E-05	DEG
Bpev01.c0155.g0021.m0001	-1.4105892	5.16E-05	0.00094837	DEG
Bpev01.c0557.g0013.m0001	-0.936984024	0.00250519	0.02157701	DEG
Bpev01.c0022.g0073.m0001	-1.111270961	0.00078243	0.00852481	DEG
Bpev01.c1748.g0002.m0001	-1.974340473	0.00348872	0.02803068	DEG
Bpev01.c0158.g0001.m0001	1.048307556	0.00376357	0.02965627	DEG
Bpev01.c0029.g0020.m0001	5.313263701	9.00E-05	0.0014862	DEG
Bpev01.c0134.g0103.m0001	2.863990055	4.11E-17	1.78E-14	DEG
Bpev01.c0794.g0008.m0001	-1.210134765	0.00744423	0.04944737	DEG
Bpev01.c2083.g0003.m0001	2.480897342	5.25E-09	3.82E-07	DEG
Bpev01.c0588.g0018.m0001	1.475346818	0.00642153	0.0443036	DEG
Bpev01.c3779.g0001.m0001	1.449267045	0.003387	0.02738527	DEG
Bpev01.c0995.g0001.m0003	2.27402594	0.00068528	0.0077103	DEG
Bpev01.c1699.g0001.m0001	1.068020774	0.00043694	0.00540212	DEG
Bpev01.c0027.g0126.m0001	4.446710101	1.75E-19	1.29E-16	DEG
Bpev01.c1233.g0003.m0001	0.933819756	0.00412748	0.03175869	DEG
Bpev01.c0449.g0014.m0001	-0.941957288	0.00322546	0.0263469	DEG

Bpev01.c0765.g0009.m0001	-1.118615651	0.00385733	0.03021708	DEG
Bpev01.c1081.g0001.m0001	1.234258415	0.00029729	0.00392107	DEG
Bpev01.c0818.g0007.m0001	7.784632723	3.49E-05	0.00069021	DEG
Bpev01.c0364.g0031.m0001	3.932672108	5.11E-12	7.37E-10	DEG
Bpev01.c0214.g0025.m0001	-3.012840605	6.28E-05	0.00110697	DEG
Bpev01.c0286.g0007.m0001	1.020336271	0.00369181	0.02923166	DEG
Bpev01.c0489.g0008.m0001	1.697551924	0.00129892	0.01275906	DEG
Bpev01.c0938.g0002.m0001	0.886325212	0.005517	0.03950741	DEG
Bpev01.c0484.g0002.m0001	1.685372686	2.72E-05	0.0005624	DEG
Bpev01.c0327.g0020.m0001	2.116991286	1.37E-08	8.82E-07	DEG
Bpev01.c3100.g0003.m0001	-1.278070769	7.61E-05	0.00129421	DEG
Bpev01.c0357.g0086.m0001	-2.300373197	2.96E-05	0.00060287	DEG
Bpev01.c0022.g0006.m0002	-1.18542581	0.00552273	0.03952924	DEG
Bpev01.c0158.g0021.m0001	-1.787477284	3.29E-07	1.35E-05	DEG
Bpev01.c1515.g0002.m0003	2.012083926	6.54E-08	3.38E-06	DEG
Bpev01.c0051.g0165.m0001	-0.904988976	0.00486633	0.0359769	DEG
Bpev01.c0018.g0112.m0001	1.367092826	3.06E-05	0.00061859	DEG
Bpev01.c0015.g0098.m0001	1.319639239	0.00107045	0.0108853	DEG
Bpev01.c0274.g0009.m0001	-1.350611108	1.16E-05	0.00027827	DEG
Bpev01.c0645.g0015.m0001	2.662166179	1.72E-07	7.76E-06	DEG
Bpev01.c2770.g0001.m0001	3.589052011	6.94E-10	6.44E-08	DEG
Bpev01.c0651.g0012.m0001	-1.433732388	8.87E-05	0.0014711	DEG
Bpev01.c0088.g0111.m0001	-1.149905494	0.00065278	0.00742548	DEG
Bpev01.c0423.g0001.m0001	2.004974998	5.36E-06	0.00014505	DEG
Bpev01.c0224.g0032.m0001	-0.913687624	0.00526373	0.03811094	DEG
Bpev01.c0367.g0012.m0001	-2.786951822	2.19E-06	6.81E-05	DEG
Bpev01.c0480.g0087.m0001	-0.874642518	0.004451	0.03357605	DEG
Bpev01.c0135.g0033.m0001	-1.821413625	0.00153047	0.01459055	DEG
Bpev01.c1534.g0010.m0001	-2.092786866	1.68E-09	1.41E-07	DEG
Bpev01.c0127.g0016.m0002	-1.1038686	0.0051816	0.0376645	DEG
Bpev01.c0328.g0048.m0001	-1.108042363	0.00140824	0.01359248	DEG
Bpev01.c0180.g0001.m0002	4.019616257	5.70E-08	3.03E-06	DEG
Bpev01.c0469.g0005.m0001	1.925791419	0.00054023	0.00638704	DEG
Bpev01.c1119.g0005.m0001	-1.835015588	0.00084965	0.00914208	DEG
Bpev01.c0848.g0038.m0001	-1.161645778	0.0057039	0.04037004	DEG
Bpev01.c0588.g0014.m0001	1.147864725	0.0046116	0.03451842	DEG
Bpev01.c0346.g0002.m0002	3.34284218	1.48E-07	6.85E-06	DEG
Bpev01.c0157.g0008.m0001	-1.82038265	3.87E-06	0.00011021	DEG
Bpev01.c1097.g0002.m0001	-1.11396943	0.00077402	0.00844876	DEG
Bpev01.c0518.g0035.m0001	-1.656364659	0.0005244	0.00625519	DEG
Bpev01.c0027.g0068.m0001	2.847651644	3.10E-12	4.68E-10	DEG
Bpev01.c0532.g0011.m0001	-1.939978676	0.00055155	0.00648709	DEG
Bpev01.c0278.g0002.m0001	-1.895359005	1.17E-06	4.08E-05	DEG

Bpev01.c0094.g0054.m0002	1.264091305	7.91E-05	0.00133785	DEG
Bpev01.c0094.g0201.m0001	-1.459576237	3.00E-05	0.00060822	DEG
Bpev01.c0776.g0012.m0001	2.327312009	9.61E-05	0.00156729	DEG
Bpev01.c1092.g0004.m0001	3.700162507	1.13E-11	1.53E-09	DEG
Bpev01.c1874.g0008.m0001	2.760490186	1.86E-13	3.75E-11	DEG
Bpev01.c0671.g0005.m0001	4.148418635	2.00E-05	0.00043518	DEG
Bpev01.c0781.g0024.m0001	-3.782942415	8.37E-05	0.00140439	DEG
Bpev01.c0363.g0005.m0003	1.335964261	7.06E-05	0.00121771	DEG
Bpev01.c0331.g0006.m0001	-1.310030811	0.00433298	0.03290134	DEG
Bpev01.c2505.g0002.m0001	-2.971813109	0.00136582	0.01327444	DEG
Bpev01.c1505.g0006.m0001	1.681600849	1.51E-06	4.99E-05	DEG
Bpev01.c0240.g0013.m0001	-1.393689683	3.74E-05	0.00072697	DEG
Bpev01.c1217.g0012.m0001	1.240854623	0.00074916	0.0082385	DEG
Bpev01.c0261.g0074.m0001	-1.214384872	0.0001566	0.00234737	DEG
Bpev01.c0430.g0012.m0001	1.709135093	0.00167418	0.01571627	DEG
Bpev01.c0101.g0072.m0001	1.793096751	0.00127623	0.01255031	DEG
Bpev01.c1527.g0009.m0001	-1.004192254	0.00088017	0.00938913	DEG
Bpev01.c3869.g0001.m0001	2.141198971	0.0024996	0.02153517	DEG
Bpev01.c0118.g0001.m0001	1.04764641	0.00557999	0.03973618	DEG
Bpev01.c0803.g0008.m0001	0.984252333	0.00664869	0.04553999	DEG
Bpev01.c0788.g0008.m0001	3.029949238	0.0031488	0.02584264	DEG
Bpev01.c0300.g0001.m0001	2.27380371	0.00057118	0.00665151	DEG
Bpev01.c0105.g0001.m0001	-2.917704151	3.96E-06	0.00011247	DEG
Bpev01.c0615.g0010.m0003	-2.756915379	4.40E-15	1.24E-12	DEG
Bpev01.c0349.g0076.m0001	-2.685295391	4.33E-11	5.25E-09	DEG
Bpev01.c0066.g0068.m0001	1.203867894	0.00418487	0.03202451	DEG
Bpev01.c0192.g0016.m0001	-1.197357935	0.00323896	0.02642686	DEG
Bpev01.c0172.g0006.m0001	-1.129000865	0.00161078	0.01522827	DEG
Bpev01.c1112.g0007.m0001	1.330578624	0.00225443	0.01983509	DEG
Bpev01.c2300.g0008.m0001	-2.719098471	0.00013904	0.00213194	DEG
Bpev01.c0828.g0021.m0001	1.804700154	1.34E-08	8.67E-07	DEG
Bpev01.c0088.g0009.m0001	-1.654527789	0.0006813	0.00767874	DEG
Bpev01.c0327.g0007.m0001	-0.896214883	0.00556041	0.03966391	DEG
Bpev01.c0170.g0059.m0001	2.413243923	1.48E-05	0.00034261	DEG
Bpev01.c1382.g0003.m0001	-2.500689003	0.001554	0.014767	DEG
Bpev01.c0518.g0051.m0001	1.620533965	0.00428596	0.03261162	DEG
Bpev01.c0130.g0019.m0001	1.040307903	0.00118518	0.01183533	DEG
Bpev01.c0224.g0013.m0001	-1.800607622	0.00045254	0.00555786	DEG
Bpev01.c0402.g0034.m0001	-1.486391029	1.87E-06	5.99E-05	DEG
Bpev01.c0150.g0007.m0001	-2.903635018	0.00028587	0.00379942	DEG
Bpev01.c0926.g0006.m0001	-2.414289226	0.00078201	0.00852331	DEG
Bpev01.c1499.g0005.m0001	-1.577746009	2.37E-05	0.00049945	DEG
Bpev01.c0289.g0009.m0001	1.203097128	0.0004213	0.0052443	DEG

Bpev01.c2046.g0009.m0001	-0.853630958	0.00456883	0.03425924	DEG
Bpev01.c2230.g0002.m0001	1.387586812	2.92E-05	0.0005944	DEG
Bpev01.c0130.g0016.m0001	0.853882516	0.00672325	0.04583736	DEG
Bpev01.c0796.g0012.m0001	2.689196139	0.00021762	0.0030463	DEG
Bpev01.c1753.g0004.m0001	-2.477197012	1.34E-11	1.79E-09	DEG
Bpev01.c0245.g0079.m0001	1.542390919	3.05E-05	0.00061823	DEG
Bpev01.c0500.g0004.m0001	1.33705297	9.54E-05	0.00155865	DEG
Bpev01.c0027.g0075.m0001	1.538600012	7.43E-06	0.00019193	DEG
Bpev01.c0284.g0021.m0001	-1.063409743	0.00162065	0.01529669	DEG
Bpev01.c0080.g0112.m0001	-1.635444331	7.37E-07	2.74E-05	DEG
Bpev01.c0022.g0059.m0001	-2.312866439	0.00094794	0.00992402	DEG
Bpev01.c0640.g0005.m0001	1.884067851	6.13E-07	2.34E-05	DEG
Bpev01.c0191.g0028.m0002	0.950056962	0.00265846	0.02261267	DEG
Bpev01.c0017.g0006.m0001	-1.310980651	0.00026979	0.00362502	DEG
Bpev01.c2226.g0001.m0001	-1.44013934	0.00343161	0.02767002	DEG
Bpev01.c0281.g0014.m0001	-1.090788933	0.00062979	0.00720851	DEG
Bpev01.c0051.g0089.m0001	1.581928465	6.23E-05	0.00110086	DEG
Bpev01.c0022.g0061.m0001	-2.817679038	0.00603909	0.0421591	DEG
Bpev01.c1169.g0003.m0001	-1.094069041	0.00068055	0.00767325	DEG
Bpev01.c0344.g0038.m0001	-1.865573133	0.0002211	0.00308046	DEG
Bpev01.c1259.g0017.m0001	5.847976628	1.17E-16	4.61E-14	DEG
Bpev01.c0015.g0225.m0001	-2.025739039	0.00057588	0.00669557	DEG
Bpev01.c1709.g0002.m0001	-2.926194616	0.0010802	0.01097313	DEG
Bpev01.c0025.g0024.m0001	-1.101747433	0.00161859	0.01528217	DEG
Bpev01.c1985.g0010.m0001	-2.647609823	0.00017247	0.00253485	DEG
Bpev01.c0522.g0042.m0001	1.916391605	0.00020952	0.00295547	DEG
Bpev01.c0052.g0133.m0001	-1.019039679	0.0019235	0.01756016	DEG
Bpev01.c1041.g0001.m0001	-1.455931306	0.00367753	0.02917352	DEG
Bpev01.c1074.g0001.m0001	3.043435302	3.40E-07	1.39E-05	DEG
Bpev01.c0574.g0036.m0001	2.700526653	3.00E-07	1.25E-05	DEG
Bpev01.c0114.g0031.m0001	-1.112188292	0.00274056	0.02317705	DEG
Bpev01.c0161.g0004.m0001	-1.471658049	9.12E-05	0.00150301	DEG
Bpev01.c0317.g0001.m0002	2.949244331	2.08E-12	3.29E-10	DEG
Bpev01.c0901.g0021.m0001	2.036603006	1.39E-09	1.19E-07	DEG
Bpev01.c0027.g0187.m0001	2.519522593	0.00671751	0.04580882	DEG
Bpev01.c0223.g0056.m0001	1.840009965	9.89E-06	0.00024413	DEG
Bpev01.c0264.g0010.m0002	1.603093321	0.00137062	0.01331234	DEG
Bpev01.c0000.g0021.m0001	-2.699831678	0.00045459	0.00557612	DEG
Bpev01.c2880.g0001.m0001	-1.03555656	0.00145496	0.01398842	DEG
Bpev01.c0597.g0009.m0001	-1.085725481	0.00046729	0.00569389	DEG
Bpev01.c0501.g0001.m0001	-1.883834591	0.00463516	0.03466834	DEG
Bpev01.c0104.g0009.m0001	1.572166779	0.00071301	0.00795389	DEG
Bpev01.c1668.g0003.m0001	1.095280923	0.00381285	0.02994836	DEG

Bpev01.c0783.g0010.m0001	2.350504539	3.83E-06	0.00010948	DEG
Bpev01.c1243.g0008.m0001	-0.848161351	0.00653245	0.04489004	DEG
Bpev01.c1916.g0002.m0001	4.392784965	1.43E-15	4.79E-13	DEG
Bpev01.c0029.g0137.m0001	-2.536867311	0.0036517	0.02907043	DEG
Bpev01.c0015.g0076.m0001	3.24082599	3.75E-07	1.51E-05	DEG
Bpev01.c0667.g0010.m0001	-2.381436243	0.00393985	0.03068394	DEG
Bpev01.c0532.g0008.m0001	-1.8391771	1.11E-06	3.91E-05	DEG
Bpev01.c0115.g0064.m0001	3.25168595	1.94E-16	7.42E-14	DEG
Bpev01.c0956.g0001.m0001	1.328523723	0.00072419	0.00802991	DEG
Bpev01.c1435.g0007.m0001	-1.738409548	0.00051056	0.00612486	DEG
Bpev01.c0739.g0005.m0001	1.836743328	9.76E-05	0.00158502	DEG
Bpev01.c0716.g0007.m0001	-1.334779371	2.17E-05	0.00046515	DEG
Bpev01.c0953.g0005.m0001	3.464108407	0.00405274	0.03133928	DEG
Bpev01.c0866.g0009.m0001	1.321176697	0.00092744	0.00974756	DEG
Bpev01.c0610.g0006.m0001	1.089533584	0.00166637	0.01567291	DEG
Bpev01.c0157.g0035.m0001	-1.117761355	0.00062308	0.00714013	DEG
Bpev01.c1272.g0020.m0001	-2.737201092	3.51E-05	0.00069283	DEG
Bpev01.c0722.g0004.m0001	2.001195828	0.00088596	0.00944213	DEG
Bpev01.c0575.g0010.m0001	1.588981354	1.69E-06	5.48E-05	DEG
Bpev01.c0425.g0007.m0001	3.078677832	2.10E-05	0.00045273	DEG
Bpev01.c0283.g0006.m0001	2.444578196	2.46E-08	1.46E-06	DEG
Bpev01.c0480.g0021.m0001	-1.08116021	0.00042742	0.00530477	DEG
Bpev01.c0094.g0018.m0001	-1.310246714	0.00030338	0.00398176	DEG
Bpev01.c0833.g0002.m0001	-1.264115843	4.82E-05	0.00089998	DEG
Bpev01.c0280.g0042.m0001	2.579681131	0.00087826	0.00937461	DEG
Bpev01.c0286.g0009.m0001	2.517751739	7.10E-09	5.00E-07	DEG
Bpev01.c0383.g0007.m0001	1.239904816	0.00211722	0.01885325	DEG
Bpev01.c2102.g0006.m0001	3.378647831	0.00509027	0.03711977	DEG
Bpev01.c0127.g0038.m0001	-1.725831743	0.00021803	0.00305065	DEG
Bpev01.c0094.g0156.m0001	-1.506543997	0.00072029	0.00801114	DEG
Bpev01.c0134.g0080.m0001	0.953278015	0.00500951	0.03666704	DEG
Bpev01.c0552.g0011.m0001	1.416923988	2.63E-05	0.00054605	DEG
Bpev01.c0029.g0054.m0002	1.247679957	0.00050726	0.00609521	DEG
Bpev01.c0245.g0043.m0001	-1.601246659	0.00019496	0.00279663	DEG
Bpev01.c1882.g0008.m0001	2.348262743	4.69E-08	2.56E-06	DEG
Bpev01.c0080.g0082.m0001	1.644287065	5.64E-07	2.17E-05	DEG
Bpev01.c1686.g0002.m0001	1.775030397	4.69E-08	2.56E-06	DEG
Bpev01.c0101.g0046.m0001	1.36344896	0.00013883	0.00212973	DEG
Bpev01.c0134.g0104.m0001	-1.467101248	0.00528564	0.03819801	DEG
Bpev01.c0555.g0025.m0001	1.080902228	0.0007899	0.00858715	DEG
Bpev01.c1004.g0001.m0001	-2.458471293	1.78E-06	5.76E-05	DEG
Bpev01.c1338.g0021.m0001	1.194765721	0.00147745	0.01416768	DEG
Bpev01.c0640.g0012.m0001	-2.712133253	4.53E-07	1.79E-05	DEG

Bpev01.c1335.g0015.m0001	-1.715682924	0.00133462	0.01303579	DEG
Bpev01.c0915.g0021.m0001	1.411421674	0.0007641	0.00837152	DEG
Bpev01.c1060.g0018.m0001	0.830242172	0.00709371	0.04775482	DEG
Bpev01.c1406.g0003.m0001	-2.164119737	5.71E-08	3.03E-06	DEG
Bpev01.c0022.g0003.m0001	-1.373126867	0.00096788	0.01008807	DEG
Bpev01.c0510.g0006.m0002	1.418495336	6.76E-05	0.00117709	DEG
Bpev01.c2092.g0001.m0001	3.504847784	0.00552513	0.03953676	DEG
Bpev01.c1518.g0010.m0001	-0.924550724	0.003512	0.02816396	DEG
Bpev01.c0703.g0001.m0002	0.923124592	0.00686618	0.04662608	DEG
Bpev01.c0105.g0021.m0001	-1.425489711	0.00268891	0.02282551	DEG
Bpev01.c1465.g0001.m0001	1.512376434	6.46E-05	0.00113211	DEG
Bpev01.c0815.g0012.m0001	1.463935073	1.25E-05	0.00029812	DEG
Bpev01.c0549.g0008.m0001	1.295864593	1.95E-05	0.00042728	DEG
Bpev01.c1332.g0003.m0002	-1.343084901	7.98E-05	0.00134672	DEG
Bpev01.c0042.g0049.m0001	1.928927108	2.39E-06	7.32E-05	DEG
Bpev01.c0346.g0005.m0001	-0.841608346	0.00700734	0.0472976	DEG
Bpev01.c0621.g0008.m0001	1.164945672	0.00024556	0.00335039	DEG
Bpev01.c1074.g0007.m0001	1.691796416	0.00391347	0.03055119	DEG
Bpev01.c0533.g0013.m0001	4.000189689	0.00035824	0.00457138	DEG
Bpev01.c0335.g0002.m0001	1.170026777	0.00318354	0.02609139	DEG
Bpev01.c0115.g0010.m0001	1.707483642	0.00010684	0.00171212	DEG
Bpev01.c0663.g0001.m0001	-1.92300864	2.34E-05	0.00049362	DEG
Bpev01.c0415.g0014.m0001	-0.817767557	0.0070215	0.04736616	DEG
Bpev01.c0127.g0100.m0001	-1.198789487	0.00068269	0.00768844	DEG
Bpev01.c0135.g0101.m0001	1.288171801	7.32E-05	0.00125355	DEG
Bpev01.c0579.g0017.m0001	3.053558767	6.19E-12	8.80E-10	DEG
Bpev01.c1661.g0001.m0001	-3.219698779	0.00032355	0.0041923	DEG
Bpev01.c0584.g0004.m0001	3.152320924	2.20E-07	9.57E-06	DEG
Bpev01.c0863.g0013.m0002	1.030106585	0.00142063	0.01369868	DEG
Bpev01.c0610.g0002.m0001	-1.607403347	4.69E-07	1.84E-05	DEG
Bpev01.c0305.g0005.m0001	2.458076833	1.95E-05	0.00042843	DEG
Bpev01.c1910.g0001.m0001	2.193028681	2.84E-07	1.19E-05	DEG
Bpev01.c0044.g0059.m0001	1.417019494	0.00018263	0.002652	DEG
Bpev01.c0015.g0030.m0002	-1.359485869	3.78E-05	0.00073445	DEG
Bpev01.c1120.g0013.m0001	1.111364648	0.0005613	0.00657021	DEG
Bpev01.c0432.g0002.m0001	1.30815008	0.00251712	0.02164809	DEG
Bpev01.c1117.g0018.m0001	-1.230675219	0.0024828	0.02140922	DEG
Bpev01.c0617.g0021.m0001	-1.309368366	0.00302018	0.02498905	DEG
Bpev01.c1060.g0006.m0001	-1.070501833	0.00070928	0.00792124	DEG
Bpev01.c0161.g0038.m0001	-1.096582866	0.00471816	0.03512845	DEG
Bpev01.c0159.g0023.m0001	2.431586988	1.44E-05	0.00033405	DEG
Bpev01.c3140.g0001.m0001	3.821753373	2.53E-05	0.00052756	DEG
Bpev01.c0413.g0003.m0001	-3.068030755	4.08E-06	0.00011501	DEG

Bpev01.c0633.g0019.m0001	2.531740477	0.00013148	0.00204152	DEG
Bpev01.c0148.g0007.m0001	3.016766438	1.58E-06	5.17E-05	DEG
Bpev01.c0480.g0051.m0001	-1.823846216	1.62E-07	7.38E-06	DEG
Bpev01.c1029.g0016.m0002	0.911556216	0.00396921	0.03087993	DEG
Bpev01.c0129.g0093.m0001	-1.561324672	0.00063727	0.0072744	DEG
Bpev01.c0062.g0008.m0001	5.226671902	5.13E-22	5.40E-19	DEG
Bpev01.c0044.g0047.m0001	-1.621514521	0.00380352	0.02990751	DEG
Bpev01.c1051.g0005.m0001	4.359574821	2.11E-06	6.59E-05	DEG
Bpev01.c0480.g0057.m0001	2.314118054	3.88E-05	0.00074998	DEG
Bpev01.c1594.g0006.m0001	4.519149321	0.00487234	0.03600452	DEG
Bpev01.c0289.g0007.m0001	1.728244699	9.48E-05	0.00155227	DEG
Bpev01.c0557.g0014.m0001	-1.299979615	5.25E-05	0.00096257	DEG
Bpev01.c0556.g0008.m0001	1.69217616	0.00528614	0.03819801	DEG
Bpev01.c0650.g0001.m0001	-3.095324295	1.62E-06	5.28E-05	DEG
Bpev01.c0328.g0033.m0001	2.724120952	1.51E-06	5.00E-05	DEG
Bpev01.c0024.g0032.m0001	1.983362318	4.25E-07	1.70E-05	DEG
Bpev01.c0027.g0041.m0001	3.311475021	5.05E-14	1.13E-11	DEG
Bpev01.c0029.g0019.m0001	3.557625014	2.88E-09	2.25E-07	DEG
Bpev01.c0101.g0006.m0001	-1.638507732	5.49E-05	0.00099346	DEG
Bpev01.c0739.g0009.m0001	-1.167475031	0.00015171	0.00227978	DEG
Bpev01.c0015.g0005.m0001	1.20362818	0.00122108	0.01211646	DEG
Bpev01.c0449.g0010.m0001	-1.049909285	0.00094084	0.00986724	DEG
Bpev01.c0364.g0003.m0001	-2.274017107	0.00185546	0.01709105	DEG
Bpev01.c0281.g0013.m0001	1.954277877	6.51E-09	4.62E-07	DEG
Bpev01.c0224.g0010.m0002	-1.503278308	8.91E-06	0.00022467	DEG
Bpev01.c2581.g0003.m0001	-1.636436433	0.00253551	0.02178084	DEG
Bpev01.c0127.g0010.m0001	-1.103945659	0.00039248	0.00494959	DEG
Bpev01.c0294.g0019.m0001	-0.889127223	0.00539779	0.03888511	DEG
Bpev01.c0695.g0001.m0001	-1.511830726	0.0003927	0.00494959	DEG
Bpev01.c0229.g0015.m0001	-2.343257647	5.63E-09	4.05E-07	DEG
Bpev01.c0485.g0031.m0001	-2.533028924	2.50E-11	3.13E-09	DEG
Bpev01.c0974.g0024.m0001	1.392659148	2.49E-05	0.00052064	DEG
Bpev01.c0096.g0021.m0001	1.188074911	0.00546684	0.03925328	DEG
Bpev01.c0016.g0130.m0001	-1.080588691	0.00194262	0.01768198	DEG
Bpev01.c0188.g0009.m0001	1.688628511	0.00648653	0.04463697	DEG
Bpev01.c0052.g0052.m0001	-1.413566999	3.72E-05	0.00072498	DEG
Bpev01.c0036.g0034.m0001	1.859694223	6.19E-05	0.00109439	DEG
Bpev01.c0330.g0010.m0001	1.560810346	1.05E-06	3.71E-05	DEG
Bpev01.c0121.g0025.m0001	1.892713705	7.01E-08	3.60E-06	DEG
Bpev01.c0580.g0011.m0001	3.128592728	9.66E-10	8.72E-08	DEG
Bpev01.c0016.g0153.m0001	-0.965726255	0.00168881	0.01582337	DEG
Bpev01.c0246.g0004.m0001	1.089693236	0.00244181	0.02110522	DEG
Bpev01.c0031.g0027.m0001	-1.465552518	4.67E-05	0.00087397	DEG

Bpev01.c0163.g0030.m0001	2.304679726	2.67E-12	4.16E-10	DEG
Bpev01.c0135.g0035.m0001	-0.851240718	0.00497581	0.03652938	DEG
Bpev01.c1765.g0001.m0001	-2.272088574	0.00575614	0.04062696	DEG
Bpev01.c1347.g0009.m0001	-1.470266152	0.0004238	0.00527089	DEG
Bpev01.c0030.g0016.m0001	-0.855794227	0.00631674	0.04372407	DEG
Bpev01.c0863.g0003.m0001	-1.159021541	0.00296851	0.02467249	DEG
Bpev01.c0777.g0027.m0001	-1.884484253	0.00403645	0.03127082	DEG
Bpev01.c1659.g0003.m0001	-1.990529483	3.10E-05	0.00062619	DEG
Bpev01.c0891.g0004.m0001	-0.890715937	0.00375375	0.02959846	DEG
Bpev01.c0566.g0058.m0001	1.266268636	0.00131893	0.01290566	DEG
Bpev01.c0213.g0025.m0001	0.99872222	0.00298515	0.02479193	DEG
Bpev01.c0826.g0010.m0001	1.983239559	6.34E-08	3.31E-06	DEG
Bpev01.c0791.g0010.m0001	4.237052709	1.66E-08	1.03E-06	DEG
Bpev01.c0161.g0069.m0001	-1.148504578	0.00033064	0.00427109	DEG
Bpev01.c0055.g0019.m0001	-2.375429258	0.0002359	0.00324514	DEG
Bpev01.c0657.g0008.m0001	3.01871596	2.94E-10	3.01E-08	DEG
Bpev01.c1915.g0002.m0001	1.564335142	0.00323799	0.02642686	DEG
Bpev01.c0365.g0033.m0001	-2.068706127	7.76E-06	0.00019925	DEG
Bpev01.c1468.g0008.m0001	2.367056285	1.13E-08	7.48E-07	DEG
Bpev01.c1377.g0002.m0001	3.58807409	4.95E-08	2.68E-06	DEG
Bpev01.c0893.g0011.m0001	2.545500654	2.52E-09	1.99E-07	DEG
Bpev01.c1028.g0013.m0001	1.590657387	0.00019665	0.00281077	DEG
Bpev01.c1837.g0004.m0001	-1.385230146	0.00087315	0.00933486	DEG
Bpev01.c0082.g0026.m0001	-1.156363329	0.00434858	0.03298811	DEG
Bpev01.c0127.g0092.m0001	-1.146558616	0.00599038	0.04193833	DEG
Bpev01.c0275.g0025.m0001	-1.412380824	4.96E-05	0.00092054	DEG
Bpev01.c0164.g0002.m0001	5.495508115	1.55E-07	7.10E-06	DEG
Bpev01.c0374.g0013.m0001	6.307674656	0.00559079	0.03977459	DEG
Bpev01.c0622.g0010.m0001	1.102234693	0.00063905	0.00729184	DEG
Bpev01.c0343.g0010.m0002	-2.089111304	0.0020159	0.01814867	DEG
Bpev01.c0357.g0075.m0001	-0.94856457	0.00413232	0.03178766	DEG
Bpev01.c0627.g0002.m0001	2.269110133	2.19E-07	9.55E-06	DEG
Bpev01.c0288.g0016.m0001	-0.929230627	0.00219148	0.01940887	DEG
Bpev01.c1503.g0010.m0001	1.18193845	0.0011903	0.01187434	DEG
Bpev01.c0485.g0030.m0001	-1.020014893	0.00304347	0.02516394	DEG
Bpev01.c0149.g0013.m0001	-1.722408323	0.00185997	0.0171272	DEG
Bpev01.c1189.g0009.m0001	-1.41350182	3.55E-05	0.00069873	DEG
Bpev01.c0439.g0019.m0001	2.84561141	0.00023328	0.003215	DEG
Bpev01.c0166.g0029.m0001	-1.475040233	1.43E-05	0.0003336	DEG
Bpev01.c0022.g0125.m0001	0.870980367	0.00376345	0.02965627	DEG
Bpev01.c0449.g0056.m0002	1.616344918	0.00091954	0.00969915	DEG
Bpev01.c0480.g0074.m0001	5.443414751	2.11E-28	5.64E-25	DEG
Bpev01.c0577.g0015.m0001	-0.963186031	0.0030279	0.02504592	DEG

Bpev01.c0051.g0027.m0001	-1.083423299	0.00069071	0.00774323	DEG
Bpev01.c0088.g0119.m0001	1.072438465	0.00201847	0.01816069	DEG
Bpev01.c0096.g0025.m0001	1.302472059	0.00021132	0.00297234	DEG
Bpev01.c0284.g0008.m0001	1.238674339	0.00252304	0.02169273	DEG
Bpev01.c1069.g0002.m0001	4.051414683	0.00082169	0.00888019	DEG
Bpev01.c0169.g0055.m0001	-1.048715556	0.00308032	0.02540809	DEG
Bpev01.c0149.g0008.m0001	-2.715495691	3.67E-10	3.66E-08	DEG
Bpev01.c0464.g0002.m0001	-0.876189662	0.00719282	0.04828947	DEG
Bpev01.c1238.g0013.m0001	-1.164912198	0.00040212	0.00503654	DEG
Bpev01.c1717.g0004.m0001	-1.3046529	0.00012805	0.00199777	DEG
Bpev01.c1092.g0013.m0001	1.495003196	0.00123975	0.01227201	DEG
Bpev01.c0011.g0001.m0001	2.609330575	0.00011705	0.00184962	DEG
Bpev01.c1678.g0001.m0001	2.071311156	0.00193639	0.01763784	DEG
Bpev01.c0146.g0041.m0001	-1.239644572	0.00020909	0.00295077	DEG
Bpev01.c0480.g0018.m0001	-3.466627038	6.20E-06	0.00016445	DEG
Bpev01.c0565.g0029.m0001	1.148665697	0.00019874	0.0028301	DEG
Bpev01.c0135.g0102.m0001	1.255567896	0.00038085	0.00482027	DEG
Bpev01.c0166.g0035.m0001	-0.919271462	0.00527829	0.03818816	DEG
Bpev01.c0449.g0049.m0001	-1.611415612	1.30E-06	4.44E-05	DEG
Bpev01.c0094.g0079.m0001	-1.134934326	0.00424026	0.03232239	DEG
Bpev01.c0522.g0009.m0001	1.049345458	0.00345272	0.02780978	DEG
Bpev01.c0919.g0022.m0001	1.514469344	8.93E-07	3.25E-05	DEG
Bpev01.c1270.g0002.m0001	-1.348818389	0.00249439	0.0214966	DEG
Bpev01.c0134.g0031.m0001	2.906402732	3.73E-07	1.51E-05	DEG
Bpev01.c0214.g0031.m0001	-1.258784764	0.00012647	0.00197522	DEG
Bpev01.c0817.g0012.m0001	1.665831352	0.0011189	0.01129997	DEG
Bpev01.c0119.g0005.m0002	-2.501577401	4.68E-07	1.84E-05	DEG
Bpev01.c1044.g0002.m0001	3.090559379	1.93E-07	8.55E-06	DEG
Bpev01.c0298.g0027.m0001	-2.500364677	0.00362345	0.02888454	DEG
Bpev01.c0118.g0012.m0001	3.581057135	3.77E-17	1.66E-14	DEG
Bpev01.c0889.g0001.m0001	-2.317167141	1.88E-13	3.76E-11	DEG
Bpev01.c1959.g0002.m0001	4.007083071	0.00617226	0.04289546	DEG
Bpev01.c0092.g0006.m0001	2.014915242	1.95E-05	0.0004282	DEG
Bpev01.c0564.g0032.m0001	-2.024323431	0.00079481	0.00863092	DEG
Bpev01.c0135.g0028.m0001	-1.837497131	6.92E-09	4.89E-07	DEG
Bpev01.c0395.g0041.m0001	-1.567626172	2.49E-05	0.00052083	DEG
Bpev01.c0114.g0014.m0001	-1.747013187	0.00048931	0.00592308	DEG
Bpev01.c0015.g0101.m0001	1.042249339	0.00570843	0.04037004	DEG
Bpev01.c0984.g0004.m0001	1.866061983	9.93E-07	3.56E-05	DEG
Bpev01.c1481.g0003.m0001	-1.501117385	8.68E-06	0.00022025	DEG
Bpev01.c0896.g0003.m0001	0.934614205	0.00641652	0.04428982	DEG
Bpev01.c0157.g0007.m0001	0.973881446	0.0047885	0.03554607	DEG
Bpev01.c0962.g0028.m0002	1.861642641	3.69E-07	1.50E-05	DEG

Bpev01.c0219.g0013.m0001	-1.974949772	0.0023985	0.02080418	DEG
Bpev01.c0142.g0001.m0001	-1.442608697	0.00464688	0.03472951	DEG
Bpev01.c2146.g0002.m0001	2.085538765	0.00666214	0.04556459	DEG
Bpev01.c0105.g0005.m0001	-1.983527024	4.89E-10	4.67E-08	DEG
Bpev01.c0354.g0006.m0001	1.285958431	6.26E-05	0.0011055	DEG
Bpev01.c1918.g0003.m0001	2.149593886	0.00046108	0.0056336	DEG
Bpev01.c1104.g0021.m0001	1.366031705	6.92E-05	0.00119787	DEG
Bpev01.c0042.g0021.m0001	-1.307231904	0.00044051	0.00543967	DEG
Bpev01.c0114.g0061.m0001	-2.858358575	2.55E-16	9.28E-14	DEG
Bpev01.c0803.g0011.m0001	1.789901546	1.27E-06	4.38E-05	DEG
Bpev01.c0396.g0005.m0001	-1.1783558	0.00564579	0.04008838	DEG
Bpev01.c1789.g0004.m0001	4.145048025	0.00487616	0.03600452	DEG
Bpev01.c0126.g0032.m0002	2.89426577	9.53E-08	4.62E-06	DEG
Bpev01.c0624.g0002.m0001	-1.393908807	0.00053511	0.0063546	DEG
Bpev01.c2187.g0005.m0001	2.17214587	1.08E-05	0.00026346	DEG
Bpev01.c0781.g0006.m0001	-1.247240487	0.00283838	0.0238264	DEG
Bpev01.c1517.g0006.m0001	-2.324755463	9.96E-07	3.57E-05	DEG
Bpev01.c2429.g0001.m0001	-2.42427235	0.00131197	0.01286579	DEG
Bpev01.c0838.g0002.m0001	-1.185483756	0.00140906	0.01359595	DEG
Bpev01.c0349.g0024.m0001	-1.071062584	0.00044294	0.00546285	DEG
Bpev01.c0597.g0042.m0001	1.127643557	0.00197086	0.01787988	DEG
Bpev01.c0027.g0132.m0001	2.623434584	1.90E-07	8.44E-06	DEG
Bpev01.c2438.g0002.m0001	1.813901209	0.00264126	0.02250534	DEG
Bpev01.c0158.g0004.m0001	0.959696231	0.00264612	0.02252961	DEG
Bpev01.c0817.g0031.m0001	-1.537074866	0.00257149	0.02203205	DEG
Bpev01.c0629.g0030.m0001	1.834928311	8.58E-05	0.00143059	DEG
Bpev01.c1345.g0001.m0001	1.039190625	0.00527435	0.038169	DEG
Bpev01.c1800.g0007.m0001	2.155889407	1.84E-07	8.23E-06	DEG
Bpev01.c0121.g0042.m0001	3.772134822	0.00042708	0.00530294	DEG
Bpev01.c1283.g0006.m0001	1.520910925	0.00031772	0.00413135	DEG
Bpev01.c0665.g0015.m0002	1.237502437	0.00431308	0.03276717	DEG
Bpev01.c0243.g0044.m0001	2.677823621	1.38E-08	8.83E-07	DEG
Bpev01.c0027.g0076.m0001	-1.37331635	5.03E-05	0.00093011	DEG
Bpev01.c0188.g0007.m0001	1.639176395	0.00058997	0.00683139	DEG
Bpev01.c1635.g0001.m0001	1.660995801	0.0010146	0.01044729	DEG
Bpev01.c0149.g0011.m0001	-1.507079729	1.58E-05	0.00036099	DEG
Bpev01.c0102.g0009.m0001	-1.073578517	0.00372035	0.02939439	DEG
Bpev01.c1784.g0001.m0001	5.5179456	1.75E-28	5.14E-25	DEG
Bpev01.c0580.g0012.m0001	3.226268817	3.61E-08	2.04E-06	DEG
Bpev01.c0706.g0011.m0001	3.255611376	1.90E-05	0.0004193	DEG
Bpev01.c0557.g0024.m0001	-1.212220406	0.00732381	0.04889017	DEG
Bpev01.c2937.g0004.m0001	3.468051246	8.92E-09	6.12E-07	DEG
Bpev01.c0717.g0024.m0001	-1.710709475	0.00058918	0.00682867	DEG

Bpev01.c0043.g0008.m0001	-1.516849794	0.00040222	0.00503654	DEG
Bpev01.c0024.g0031.m0001	-1.066316061	0.00098674	0.01023958	DEG
Bpev01.c0962.g0034.m0001	-1.164435991	0.0005906	0.00683226	DEG
Bpev01.c0022.g0078.m0001	-3.272368864	0.00128872	0.0126631	DEG
Bpev01.c0052.g0072.m0001	-1.420641025	1.04E-05	0.00025457	DEG
Bpev01.c1148.g0007.m0001	-2.796851078	3.67E-12	5.51E-10	DEG
Bpev01.c2101.g0001.m0001	1.360926214	0.00166833	0.01568633	DEG
Bpev01.c0158.g0057.m0001	6.013513586	2.17E-34	3.19E-30	DEG
Bpev01.c0858.g0003.m0001	6.093774877	2.34E-05	0.00049362	DEG
Bpev01.c0494.g0004.m0001	-1.641346925	1.07E-05	0.00026133	DEG
Bpev01.c0356.g0001.m0001	2.113711953	0.0015137	0.01445407	DEG
Bpev01.c0522.g0039.m0001	-1.260079254	0.00015125	0.00227753	DEG
Bpev01.c0149.g0004.m0001	-2.624218353	7.91E-05	0.00133757	DEG
Bpev01.c2272.g0004.m0001	-1.613388588	6.18E-07	2.36E-05	DEG
Bpev01.c0744.g0016.m0001	1.314746533	0.00023575	0.00324456	DEG
Bpev01.c0171.g0025.m0001	-1.191877362	0.00430438	0.03271798	DEG
Bpev01.c0161.g0039.m0001	-2.5377736	7.39E-07	2.74E-05	DEG
Bpev01.c0884.g0014.m0001	-1.618493169	0.0003762	0.00476749	DEG
Bpev01.c0506.g0009.m0001	-1.921000958	9.13E-05	0.00150352	DEG
Bpev01.c0210.g0052.m0001	-2.296268528	8.41E-05	0.00140858	DEG
Bpev01.c1907.g0009.m0001	-1.71435073	0.00569802	0.04035736	DEG
Bpev01.c2017.g0003.m0001	-1.124878588	0.00049786	0.00601174	DEG
Bpev01.c0365.g0031.m0001	1.473975085	0.00066322	0.00750939	DEG
Bpev01.c0906.g0006.m0001	3.576877024	0.00235404	0.02049308	DEG
Bpev01.c0874.g0031.m0001	-1.503627629	0.00469974	0.03505339	DEG
Bpev01.c0439.g0028.m0001	-1.317738805	0.00010084	0.00162579	DEG
Bpev01.c0222.g0044.m0001	-1.095236304	0.00417226	0.03196401	DEG
Bpev01.c0518.g0034.m0001	-2.078698124	3.67E-06	0.00010607	DEG
Bpev01.c0052.g0174.m0001	-6.159547673	0.00693491	0.0469326	DEG
Bpev01.c1171.g0011.m0001	-2.099480129	1.92E-08	1.17E-06	DEG
Bpev01.c0389.g0015.m0002	-1.175795146	0.00309764	0.02553669	DEG
Bpev01.c1040.g0033.m0002	2.074310182	7.95E-06	0.00020359	DEG
Bpev01.c0015.g0248.m0001	-3.30344521	1.13E-05	0.00027287	DEG
Bpev01.c0135.g0032.m0001	-1.045436307	0.00118894	0.01186486	DEG
Bpev01.c0137.g0004.m0001	-1.102696482	0.00196268	0.01783314	DEG
Bpev01.c0423.g0007.m0001	-1.45206658	2.34E-06	7.21E-05	DEG
Bpev01.c0159.g0038.m0001	-4.33527832	9.38E-08	4.58E-06	DEG
Bpev01.c1078.g0008.m0001	-1.059573165	0.0006425	0.00731699	DEG
Bpev01.c1335.g0021.m0001	1.233108514	0.00014718	0.00222533	DEG
Bpev01.c1203.g0001.m0001	-1.043157582	0.00096878	0.01008807	DEG
Bpev01.c0461.g0006.m0002	2.294552233	4.90E-05	0.00091167	DEG
Bpev01.c0115.g0112.m0001	1.595532696	0.00682535	0.04637235	DEG
Bpev01.c0646.g0002.m0001	3.081836172	0.00042291	0.00526212	DEG

Bpev01.c1786.g0008.m0001	-2.259644635	5.15E-05	0.00094595	DEG
Bpev01.c0050.g0099.m0001	-7.626875981	0.00012385	0.00194455	DEG
Bpev01.c1211.g0019.m0001	-0.956520274	0.00607203	0.04230879	DEG
Bpev01.c0245.g0060.m0001	-2.089121445	3.39E-06	9.92E-05	DEG
Bpev01.c0441.g0004.m0001	4.853137498	0.00072059	0.00801114	DEG
Bpev01.c0508.g0005.m0001	1.3730647	7.15E-05	0.00122854	DEG
Bpev01.c0445.g0023.m0001	0.854630957	0.00720738	0.04832561	DEG
Bpev01.c0753.g0018.m0001	1.555037182	0.00015151	0.00227912	DEG
Bpev01.c1833.g0005.m0001	-2.28687634	3.88E-05	0.00075024	DEG
Bpev01.c1602.g0005.m0001	-2.111688336	6.79E-05	0.00118186	DEG
Bpev01.c3390.g0001.m0001	-1.377255849	5.96E-06	0.00015917	DEG
Bpev01.c0817.g0027.m0001	-1.597164055	7.87E-05	0.00133152	DEG
Bpev01.c1242.g0004.m0001	1.716548462	0.00013525	0.00208784	DEG
Bpev01.c0001.g0014.m0001	2.644794245	1.90E-09	1.56E-07	DEG
Bpev01.c3039.g0001.m0001	-2.391259438	7.25E-05	0.00124314	DEG
Bpev01.c1588.g0010.m0001	1.879787377	6.78E-08	3.49E-06	DEG
Bpev01.c0088.g0124.m0001	4.888070748	3.35E-15	9.77E-13	DEG
Bpev01.c0243.g0018.m0001	-1.386631109	4.15E-05	0.00079335	DEG
Bpev01.c0298.g0028.m0001	-1.593406192	1.88E-07	8.35E-06	DEG
Bpev01.c0633.g0007.m0001	-0.891697194	0.00742283	0.04936896	DEG
Bpev01.c0094.g0036.m0001	2.049911997	0.00048773	0.00591368	DEG
Bpev01.c0283.g0011.m0001	-2.787682251	2.62E-09	2.06E-07	DEG
Bpev01.c1352.g0002.m0001	-2.354722555	6.32E-08	3.31E-06	DEG
Bpev01.c0000.g0217.m0001	-1.23872059	9.17E-05	0.00150895	DEG
Bpev01.c0788.g0005.m0001	1.684656018	0.0008032	0.00869639	DEG
Bpev01.c0403.g0022.m0001	-1.881783882	1.97E-08	1.20E-06	DEG
Bpev01.c1185.g0013.m0001	-2.228322154	7.32E-10	6.78E-08	DEG
Bpev01.c0979.g0007.m0001	2.008874051	0.00115558	0.0115948	DEG
Bpev01.c0147.g0008.m0001	-4.113529869	0.00046494	0.00566996	DEG
Bpev01.c0349.g0040.m0001	1.377246591	0.00089976	0.00953547	DEG
Bpev01.c0045.g0019.m0001	6.601976732	5.66E-07	2.17E-05	DEG
Bpev01.c1543.g0001.m0001	-1.144926111	0.00046675	0.00568972	DEG
Bpev01.c0739.g0026.m0001	1.136809982	0.00418752	0.03203647	DEG
Bpev01.c0223.g0027.m0001	2.787681314	3.55E-10	3.56E-08	DEG
Bpev01.c2777.g0002.m0001	-1.335709418	0.0040708	0.03142941	DEG
Bpev01.c0051.g0119.m0001	-2.682856736	1.83E-12	2.94E-10	DEG
Bpev01.c1085.g0019.m0001	-1.536586123	0.00277415	0.02341408	DEG
Bpev01.c0458.g0015.m0001	-3.029857358	2.34E-12	3.69E-10	DEG
Bpev01.c0050.g0027.m0001	1.160193044	0.00487618	0.03600452	DEG
Bpev01.c0584.g0011.m0001	6.14670893	2.86E-05	0.00058522	DEG
Bpev01.c0052.g0040.m0001	-1.705678644	7.53E-07	2.79E-05	DEG
Bpev01.c1484.g0012.m0001	-1.440791101	4.27E-05	0.00081077	DEG
Bpev01.c0000.g0066.m0001	-3.21872347	7.03E-07	2.63E-05	DEG

Bpev01.c1371.g0007.m0002	-1.242624913	0.00054017	0.00638704	DEG
Bpev01.c0015.g0054.m0001	-1.197376814	0.00210857	0.01881033	DEG
Bpev01.c0029.g0104.m0001	-1.403360439	5.92E-05	0.00106146	DEG
Bpev01.c0577.g0019.m0001	1.517850669	0.00023214	0.00320393	DEG
Bpev01.c0294.g0011.m0001	-2.413478852	1.49E-08	9.43E-07	DEG
Bpev01.c0523.g0003.m0001	-2.004450167	0.00452934	0.03402381	DEG
Bpev01.c2507.g0004.m0001	-1.168712924	0.00026188	0.00353321	DEG
Bpev01.c0142.g0005.m0001	-1.144085796	0.00263192	0.02245175	DEG
Bpev01.c2063.g0002.m0001	-1.951499982	0.00022186	0.00308674	DEG
Bpev01.c0092.g0010.m0001	-2.569423589	2.95E-06	8.82E-05	DEG
Bpev01.c0000.g0003.m0001	-1.835110378	0.00294892	0.02455126	DEG
Bpev01.c2256.g0001.m0001	1.503836249	1.31E-06	4.45E-05	DEG
Bpev01.c0357.g0037.m0001	-1.949229965	4.51E-07	1.78E-05	DEG
Bpev01.c0616.g0004.m0001	-1.828918132	0.00675232	0.04599295	DEG
Bpev01.c0000.g0018.m0001	-1.66782808	0.00055977	0.00655756	DEG
Bpev01.c0365.g0002.m0001	1.721991376	6.59E-07	2.49E-05	DEG
Bpev01.c1638.g0007.m0001	-2.742437542	1.02E-13	2.11E-11	DEG
Bpev01.c0447.g0021.m0001	2.666560836	3.59E-08	2.03E-06	DEG
Bpev01.c1897.g0006.m0001	-1.962675425	2.36E-06	7.26E-05	DEG
Bpev01.c1054.g0001.m0001	1.992336097	6.64E-08	3.42E-06	DEG
Bpev01.c0437.g0018.m0001	-1.167050151	0.00082449	0.00890215	DEG
Bpev01.c1723.g0002.m0001	1.199937679	0.00042082	0.00524049	DEG
Bpev01.c1295.g0001.m0001	-1.803933562	0.00367455	0.02916558	DEG
Bpev01.c2749.g0001.m0001	-1.367099214	5.47E-05	0.00099254	DEG
Bpev01.c0158.g0064.m0001	1.412651105	0.00487319	0.03600452	DEG
Bpev01.c1086.g0011.m0001	1.584214895	2.62E-05	0.00054372	DEG
Bpev01.c0267.g0008.m0001	-1.978685182	0.00665225	0.04554317	DEG
Bpev01.c1281.g0009.m0001	-1.232806648	0.00386898	0.03028146	DEG
Bpev01.c1117.g0013.m0001	2.989497771	3.46E-11	4.26E-09	DEG
Bpev01.c0023.g0019.m0001	-2.536488679	1.81E-09	1.50E-07	DEG
Bpev01.c0505.g0020.m0001	1.486535376	5.56E-05	0.00100248	DEG
Bpev01.c0505.g0022.m0001	4.108074969	1.61E-09	1.36E-07	DEG
Bpev01.c1783.g0001.m0001	2.673047298	0.00506727	0.03699788	DEG
Bpev01.c0383.g0019.m0001	-2.085962869	4.97E-05	0.00092054	DEG
Bpev01.c0245.g0022.m0001	-2.438348284	0.00022187	0.00308674	DEG
Bpev01.c0000.g0039.m0001	-1.318068041	0.00012593	0.00197155	DEG
Bpev01.c0066.g0066.m0001	1.367612903	9.81E-06	0.00024277	DEG
Bpev01.c0479.g0011.m0001	-2.612277191	0.0001955	0.00279797	DEG
Bpev01.c1060.g0037.m0001	-1.544547856	1.06E-06	3.75E-05	DEG
Bpev01.c1649.g0010.m0001	1.075742544	0.00375016	0.02958423	DEG
Bpev01.c0029.g0085.m0001	-1.52894114	7.70E-05	0.00130816	DEG
Bpev01.c0585.g0004.m0001	1.566883035	1.65E-05	0.00037574	DEG
Bpev01.c0189.g0055.m0001	4.019944228	3.69E-26	6.78E-23	DEG

Bpev01.c0089.g0060.m0001	0.992693755	0.00334458	0.02710188	DEG
Bpev01.c0863.g0030.m0001	0.974478877	0.00196484	0.01784178	DEG
Bpev01.c0692.g0004.m0001	-1.324974329	0.00068683	0.00772036	DEG
Bpev01.c0365.g0001.m0001	3.590979109	0.00150895	0.01441661	DEG
Bpev01.c1047.g0008.m0001	1.26669586	0.00349059	0.02803804	DEG
Bpev01.c0050.g0028.m0001	-1.949770087	2.16E-05	0.00046486	DEG
Bpev01.c1142.g0005.m0001	1.110052518	0.00752672	0.04987154	DEG
Bpev01.c0561.g0002.m0001	-1.204547389	0.00413731	0.03180943	DEG
Bpev01.c0240.g0016.m0001	1.121394793	0.00127639	0.01255031	DEG
Bpev01.c0080.g0025.m0001	-0.958759494	0.0066623	0.04556459	DEG
Bpev01.c2002.g0003.m0001	-1.728674684	0.00119535	0.01190862	DEG
Bpev01.c0389.g0016.m0001	-1.108036577	0.00032882	0.00425128	DEG
Bpev01.c0263.g0016.m0001	1.543920388	3.32E-05	0.00066337	DEG
Bpev01.c0066.g0059.m0001	0.90131339	0.0032217	0.0263235	DEG
Bpev01.c0437.g0001.m0001	2.097356608	1.76E-05	0.0003937	DEG
Bpev01.c1396.g0002.m0001	2.068340306	9.40E-10	8.54E-08	DEG
Bpev01.c0328.g0016.m0001	1.276953356	0.00454914	0.03412031	DEG
Bpev01.c1347.g0003.m0001	-2.434182345	0.00042003	0.00523286	DEG
Bpev01.c0063.g0029.m0001	2.949626905	1.91E-06	6.10E-05	DEG
Bpev01.c0278.g0023.m0001	-2.714720887	0.00010115	0.00162985	DEG
Bpev01.c0649.g0001.m0001	3.133011487	2.14E-05	0.00046057	DEG
Bpev01.c0138.g0004.m0001	-1.044080596	0.00082749	0.00892655	DEG
Bpev01.c0162.g0032.m0001	2.19857677	1.81E-07	8.13E-06	DEG
Bpev01.c0264.g0027.m0001	3.59056479	0.00383806	0.03010183	DEG
Bpev01.c0136.g0009.m0001	-1.420530471	0.00029093	0.00385619	DEG
Bpev01.c2282.g0001.m0001	1.943065636	4.35E-05	0.00082366	DEG
Bpev01.c0220.g0003.m0001	-1.063364113	0.00054525	0.00643008	DEG
Bpev01.c0127.g0067.m0001	-1.255825278	0.00021939	0.00306388	DEG
Bpev01.c0517.g0018.m0001	-1.073210072	0.00068171	0.00768043	DEG
Bpev01.c0349.g0012.m0001	-2.282936947	4.75E-10	4.58E-08	DEG
Bpev01.c0660.g0011.m0001	-1.391779584	0.0016763	0.01573118	DEG
Bpev01.c0645.g0032.m0001	-0.859660876	0.00489835	0.03613191	DEG
Bpev01.c0688.g0002.m0001	0.901417975	0.00435001	0.03298811	DEG
Bpev01.c0755.g0005.m0001	-3.070328223	0.00120431	0.01197353	DEG
Bpev01.c1550.g0005.m0001	-1.940007876	3.60E-06	0.00010455	DEG
Bpev01.c0166.g0037.m0001	1.475801794	3.87E-06	0.00011021	DEG
Bpev01.c0659.g0006.m0001	-1.474408927	5.54E-05	0.00099919	DEG
Bpev01.c0579.g0020.m0001	1.937712064	8.14E-08	4.11E-06	DEG
Bpev01.c0962.g0008.m0001	-1.768085565	0.00074184	0.00817937	DEG
Bpev01.c0094.g0034.m0001	-1.130167047	0.00054114	0.00639522	DEG
Bpev01.c0892.g0022.m0001	1.152277591	0.00233921	0.02040414	DEG
Bpev01.c1114.g0003.m0001	3.64298237	1.87E-11	2.41E-09	DEG
Bpev01.c2001.g0009.m0001	-2.057167386	2.68E-11	3.32E-09	DEG

Bpev01.c0299.g0012.m0003	1.128358052	0.00136129	0.01324661	DEG
Bpev01.c0052.g0206.m0003	-1.771251567	5.96E-05	0.00106683	DEG
Bpev01.c0415.g0018.m0001	1.349278283	0.00021508	0.00301374	DEG
Bpev01.c0066.g0023.m0001	-1.518885406	8.47E-07	3.11E-05	DEG
Bpev01.c0473.g0041.m0001	-1.57319906	0.00016943	0.00249774	DEG
Bpev01.c1153.g0001.m0001	1.551617536	7.64E-06	0.00019667	DEG
Bpev01.c2616.g0001.m0001	3.529137951	0.00169228	0.01584068	DEG
Bpev01.c1333.g0014.m0001	-2.907552112	6.54E-05	0.00114402	DEG
Bpev01.c1844.g0003.m0001	-1.147462105	0.00016913	0.00249452	DEG
Bpev01.c0154.g0004.m0001	-1.681150227	1.12E-06	3.93E-05	DEG
Bpev01.c0720.g0007.m0001	-1.279998489	0.00100886	0.01039911	DEG
Bpev01.c0092.g0004.m0001	1.090609421	0.00427849	0.03256324	DEG
Bpev01.c0577.g0030.m0002	-2.422202228	8.15E-05	0.00137128	DEG
Bpev01.c0727.g0018.m0001	3.268354767	1.37E-14	3.55E-12	DEG
Bpev01.c0096.g0004.m0001	2.54840197	1.02E-07	4.92E-06	DEG
Bpev01.c2229.g0003.m0001	1.64154261	0.00133582	0.0130423	DEG
Bpev01.c0532.g0017.m0001	2.087901477	0.00153263	0.01459695	DEG
Bpev01.c0916.g0003.m0001	-2.185478373	5.36E-05	0.00097566	DEG
Bpev01.c0261.g0046.m0001	-1.083578128	0.00324118	0.02643133	DEG
Bpev01.c1235.g0002.m0001	3.871055414	0.00471349	0.03511148	DEG
Bpev01.c0017.g0021.m0001	-0.987844201	0.00201169	0.01812739	DEG
Bpev01.c3203.g0002.m0001	1.158433531	0.00017075	0.00251464	DEG
Bpev01.c0094.g0039.m0001	-1.846050099	9.81E-08	4.74E-06	DEG
Bpev01.c0018.g0078.m0001	2.542958871	1.42E-11	1.88E-09	DEG
Bpev01.c0383.g0016.m0001	1.161186534	0.00035487	0.00453033	DEG
Bpev01.c1044.g0007.m0001	1.700333766	2.88E-06	8.63E-05	DEG
Bpev01.c0210.g0027.m0001	2.088132656	2.39E-08	1.43E-06	DEG
Bpev01.c0066.g0016.m0001	-1.261370081	6.86E-05	0.00119064	DEG
Bpev01.c2235.g0014.m0001	2.627730661	3.82E-06	0.00010932	DEG
Bpev01.c0104.g0008.m0001	2.702413836	0.00014911	0.00225103	DEG
Bpev01.c1756.g0001.m0001	-2.165069383	0.00657363	0.04510979	DEG
Bpev01.c0422.g0018.m0001	0.981978466	0.00635569	0.04394202	DEG
Bpev01.c0896.g0006.m0001	-0.980933484	0.00315724	0.0259047	DEG
Bpev01.c0679.g0010.m0001	1.044305154	0.00219585	0.01943032	DEG
Bpev01.c0447.g0018.m0001	2.478560994	8.71E-08	4.31E-06	DEG
Bpev01.c0015.g0051.m0001	-0.96181708	0.00669263	0.04568149	DEG
Bpev01.c1051.g0007.m0001	3.556043303	2.51E-05	0.00052375	DEG
Bpev01.c0015.g0110.m0001	1.550000906	0.00633848	0.04384203	DEG
Bpev01.c0016.g0074.m0001	-1.052638662	0.00124101	0.01227628	DEG
Bpev01.c1649.g0012.m0001	1.287313962	7.79E-05	0.00131945	DEG
Bpev01.c1456.g0002.m0001	-1.842589333	0.00180844	0.01673602	DEG
Bpev01.c0518.g0061.m0001	2.081614806	4.32E-05	0.00081899	DEG
Bpev01.c2158.g0002.m0001	3.254507747	3.88E-06	0.00011025	DEG

Bpev01.c3113.g0001.m0001	4.53151514	0.00079764	0.00865206	DEG
Bpev01.c0015.g0071.m0001	-1.888886794	1.99E-05	0.00043467	DEG
Bpev01.c1288.g0001.m0001	-1.429026627	4.33E-05	0.00082113	DEG
Bpev01.c1273.g0001.m0001	1.41582596	0.00040566	0.00507313	DEG
Bpev01.c0797.g0009.m0001	1.101023674	0.00264425	0.02252436	DEG
Bpev01.c0473.g0040.m0001	-3.514433265	7.60E-22	7.22E-19	DEG
Bpev01.c0212.g0023.m0001	-2.000064605	0.00018966	0.0027383	DEG
Bpev01.c1836.g0006.m0001	-2.567577109	1.41E-07	6.61E-06	DEG
Bpev01.c0094.g0259.m0001	-2.305331651	0.00219412	0.01942636	DEG
Bpev01.c0237.g0057.m0001	-1.434756517	6.22E-05	0.00110007	DEG
Bpev01.c0429.g0002.m0001	-0.93377621	0.00279074	0.02350688	DEG
Bpev01.c1674.g0007.m0001	1.087835137	0.00065143	0.00741303	DEG
Bpev01.c1193.g0014.m0001	-0.925556221	0.00332628	0.02699819	DEG
Bpev01.c0588.g0028.m0001	-1.753746986	0.00728288	0.04870148	DEG
Bpev01.c0163.g0015.m0001	-1.39128677	8.92E-06	0.00022485	DEG
Bpev01.c0166.g0028.m0001	-1.018584457	0.0038595	0.03022608	DEG
Bpev01.c1100.g0010.m0001	-1.539574091	5.29E-05	0.00096841	DEG
Bpev01.c0365.g0015.m0001	-0.906207737	0.00333646	0.02706791	DEG
Bpev01.c1280.g0013.m0001	2.154449217	4.54E-06	0.0001259	DEG
Bpev01.c1723.g0003.m0001	-1.549314834	0.00429909	0.03268617	DEG
Bpev01.c0700.g0005.m0001	-2.348977054	9.33E-11	1.07E-08	DEG
Bpev01.c2462.g0003.m0001	2.168784043	3.74E-05	0.00072697	DEG
Bpev01.c0000.g0058.m0001	1.387064426	0.00211211	0.01881909	DEG
Bpev01.c0450.g0018.m0001	3.724234293	0.00469479	0.03502533	DEG
Bpev01.c0522.g0029.m0001	-2.640414318	6.74E-07	2.54E-05	DEG
Bpev01.c2326.g0005.m0001	1.756337968	2.58E-05	0.0005374	DEG
Bpev01.c0082.g0075.m0001	1.85986294	0.00283459	0.02381492	DEG
Bpev01.c0163.g0040.m0001	2.550495005	4.64E-10	4.51E-08	DEG
Bpev01.c0945.g0001.m0001	3.600116569	3.27E-10	3.32E-08	DEG
Bpev01.c2275.g0003.m0001	-1.264663447	0.00012853	0.00199889	DEG
Bpev01.c0842.g0013.m0001	1.830874204	3.96E-05	0.00076115	DEG
Bpev01.c0784.g0010.m0001	1.319935881	0.001385	0.01341222	DEG
Bpev01.c0682.g0008.m0001	-1.060710774	0.00218802	0.01938405	DEG
Bpev01.c0824.g0002.m0001	2.597233302	0.00020877	0.00294778	DEG
Bpev01.c0281.g0021.m0001	1.771095787	3.53E-05	0.00069457	DEG
Bpev01.c0286.g0025.m0001	0.851115395	0.00728396	0.04870148	DEG
Bpev01.c0327.g0001.m0001	3.401740339	9.57E-08	4.63E-06	DEG
Bpev01.c0588.g0025.m0001	2.043510443	0.0055058	0.03944636	DEG
Bpev01.c0038.g0094.m0001	3.807091376	0.00381766	0.02997816	DEG
Bpev01.c0022.g0081.m0001	-0.947210414	0.0042563	0.03243621	DEG
Bpev01.c1439.g0009.m0001	-1.758409548	0.00448695	0.03376016	DEG
Bpev01.c0018.g0039.m0001	-1.030822491	0.00232256	0.020295	DEG
Bpev01.c0016.g0080.m0001	2.220652701	0.00142218	0.01370908	DEG

Bpev01.c0029.g0091.m0001	1.271172249	0.00014337	0.00218341	DEG
Bpev01.c0649.g0021.m0001	2.879497061	0.00110864	0.01120783	DEG
Bpev01.c0022.g0010.m0001	-1.258796906	3.90E-05	0.00075315	DEG
Bpev01.c0038.g0013.m0001	-0.973800204	0.0028922	0.02418504	DEG
Bpev01.c0951.g0003.m0001	-3.961176062	2.33E-16	8.57E-14	DEG
Bpev01.c3010.g0004.m0001	-1.717187674	9.57E-05	0.00156324	DEG
Bpev01.c0121.g0047.m0001	-1.161701565	0.00035091	0.00449145	DEG
Bpev01.c2269.g0002.m0001	2.687521722	0.00248059	0.02139642	DEG
Bpev01.c0025.g0039.m0001	2.323594965	1.84E-05	0.00040881	DEG
Bpev01.c0483.g0010.m0001	-1.314043262	1.80E-05	0.00040129	DEG
Bpev01.c0052.g0080.m0001	-1.733874693	0.00175457	0.01632515	DEG
Bpev01.c2456.g0002.m0001	1.655569436	0.00609369	0.04241953	DEG
Bpev01.c1755.g0001.m0001	-1.161610865	0.00504285	0.0368653	DEG
Bpev01.c0901.g0023.m0001	-2.365026649	0.00438441	0.0331892	DEG
Bpev01.c3885.g0001.m0001	-1.953997694	0.0046465	0.03472951	DEG
Bpev01.c0080.g0122.m0001	2.524372238	0.00055413	0.00650444	DEG
Bpev01.c0276.g0012.m0001	3.500930245	3.75E-14	8.55E-12	DEG
Bpev01.c1029.g0003.m0001	-1.178597189	0.00044896	0.00552084	DEG
Bpev01.c0177.g0048.m0001	-1.623118816	0.00281164	0.02365585	DEG
Bpev01.c0847.g0009.m0001	-1.822222934	2.20E-05	0.00047123	DEG
Bpev01.c1119.g0015.m0001	-2.232118251	0.00098675	0.01023958	DEG
Bpev01.c0114.g0037.m0001	-1.197505371	0.00039444	0.00496659	DEG
Bpev01.c0957.g0010.m0001	-1.063019547	0.0007046	0.00788097	DEG
Bpev01.c0557.g0031.m0001	0.808781612	0.00697508	0.04713942	DEG
Bpev01.c0518.g0031.m0001	-1.294172501	0.00022737	0.00315145	DEG
Bpev01.c0727.g0004.m0001	-1.007553789	0.00727802	0.04868385	DEG
Bpev01.c0000.g0209.m0001	1.273531447	0.00010853	0.00173548	DEG
Bpev01.c0757.g0002.m0001	-2.117662082	0.00053629	0.00636355	DEG
Bpev01.c2083.g0005.m0001	2.4934191	1.32E-08	8.60E-07	DEG
Bpev01.c0571.g0008.m0001	-2.58039656	4.16E-11	5.06E-09	DEG
Bpev01.c0157.g0056.m0001	1.609029335	1.65E-05	0.00037432	DEG
Bpev01.c0427.g0034.m0002	-1.515277818	8.66E-05	0.00144173	DEG
Bpev01.c0463.g0001.m0001	2.421052403	6.06E-05	0.00107753	DEG
Bpev01.c1117.g0009.m0001	1.112356222	0.00065567	0.00744683	DEG
Bpev01.c1784.g0002.m0001	4.095175477	1.83E-09	1.51E-07	DEG
Bpev01.c0040.g0001.m0001	2.104807267	0.00014474	0.00219752	DEG
Bpev01.c0441.g0006.m0001	-1.398725527	4.36E-05	0.00082493	DEG
Bpev01.c1216.g0002.m0001	3.151836904	3.84E-05	0.00074396	DEG
Bpev01.c1871.g0006.m0002	1.25343172	0.00359057	0.02866128	DEG
Bpev01.c0889.g0015.m0001	1.31417498	0.00017821	0.00259718	DEG
Bpev01.c0038.g0011.m0001	-2.717794535	1.65E-17	8.09E-15	DEG
Bpev01.c0169.g0033.m0001	-2.56576529	1.89E-06	6.06E-05	DEG
Bpev01.c0420.g0011.m0001	-3.248222583	2.04E-07	8.94E-06	DEG

Bpev01.c0929.g0019.m0001	-1.017099286	0.00135569	0.01320215	DEG
Bpev01.c0135.g0041.m0001	-1.259294251	0.00341045	0.02752951	DEG
Bpev01.c0398.g0016.m0001	-1.994763992	6.28E-07	2.39E-05	DEG
Bpev01.c0145.g0053.m0001	-2.262950974	0.00023191	0.00320335	DEG
Bpev01.c0925.g0005.m0001	-1.183943986	0.00024545	0.00335039	DEG
Bpev01.c1053.g0003.m0001	3.427047009	3.01E-08	1.74E-06	DEG
Bpev01.c0286.g0023.m0001	-2.191192449	0.00104471	0.01068179	DEG
Bpev01.c2482.g0002.m0001	2.667565361	0.00017667	0.00257854	DEG
Bpev01.c2346.g0006.m0001	3.440091401	1.00E-17	4.99E-15	DEG
Bpev01.c0668.g0016.m0001	-1.894002465	2.86E-05	0.00058594	DEG
Bpev01.c0134.g0049.m0001	2.475710068	5.98E-09	4.28E-07	DEG
Bpev01.c0170.g0066.m0001	3.12411605	1.70E-12	2.76E-10	DEG
Bpev01.c0396.g0001.m0001	-2.05547598	0.00021027	0.00296459	DEG
Bpev01.c1092.g0006.m0001	-1.248183574	0.00557233	0.03972002	DEG
Bpev01.c0522.g0015.m0001	4.388312527	8.82E-15	2.36E-12	DEG
Bpev01.c0050.g0157.m0001	1.860187196	1.48E-05	0.00034177	DEG
Bpev01.c0431.g0002.m0001	1.306567091	0.00069271	0.00775972	DEG
Bpev01.c1723.g0001.m0001	-1.121615565	0.00319187	0.02613051	DEG
Bpev01.c0038.g0032.m0001	-2.729060716	2.31E-16	8.57E-14	DEG
Bpev01.c0769.g0003.m0001	-2.372362633	0.00238278	0.02069225	DEG
Bpev01.c0651.g0015.m0001	4.554708036	9.67E-13	1.66E-10	DEG
Bpev01.c0129.g0056.m0001	-2.235112682	0.00375423	0.02959846	DEG
Bpev01.c0134.g0114.m0001	3.193617262	1.36E-10	1.49E-08	DEG
Bpev01.c0029.g0002.m0001	-2.114655969	4.10E-07	1.64E-05	DEG
Bpev01.c0015.g0200.m0001	-0.926514672	0.00625127	0.04334256	DEG
Bpev01.c0089.g0001.m0001	1.728623771	0.00690132	0.04680044	DEG
Bpev01.c1339.g0009.m0001	1.379238997	0.00013829	0.00212481	DEG
Bpev01.c0820.g0010.m0001	0.90433941	0.00481043	0.03565315	DEG
Bpev01.c0403.g0036.m0001	-1.605762112	8.32E-05	0.00139791	DEG
Bpev01.c0936.g0008.m0001	-1.676015713	3.00E-07	1.25E-05	DEG
Bpev01.c0016.g0143.m0001	-1.33050685	0.00117275	0.01173508	DEG
Bpev01.c0127.g0016.m0001	-1.123280076	0.00352866	0.02825697	DEG
Bpev01.c0500.g0003.m0001	-2.164976912	0.00097443	0.01012575	DEG
Bpev01.c0000.g0176.m0001	-1.208130835	0.00028168	0.00376069	DEG
Bpev01.c1577.g0007.m0001	-2.209902117	2.75E-05	0.00056829	DEG
Bpev01.c1040.g0016.m0001	-1.938084352	5.86E-08	3.10E-06	DEG
Bpev01.c0127.g0039.m0001	1.606557709	1.48E-05	0.00034255	DEG
Bpev01.c0804.g0007.m0001	1.409589783	1.67E-05	0.00037877	DEG
Bpev01.c0114.g0055.m0001	-3.542620823	0.00019171	0.00275837	DEG
Bpev01.c0050.g0095.m0001	-1.548660781	0.0056275	0.03997782	DEG
Bpev01.c0381.g0014.m0002	1.6874919	1.01E-06	3.60E-05	DEG
Bpev01.c0616.g0018.m0001	-2.099153475	8.83E-08	4.35E-06	DEG
Bpev01.c1284.g0004.m0001	2.632511789	1.54E-06	5.06E-05	DEG

Bpev01.c1285.g0003.m0001	-1.716108804	0.00476842	0.03543098	DEG
Bpev01.c0667.g0002.m0002	-2.357692176	1.66E-05	0.00037758	DEG
Bpev01.c2409.g0002.m0001	1.531800058	3.09E-06	9.17E-05	DEG
Bpev01.c0016.g0176.m0001	-1.610629982	0.00075523	0.00829746	DEG
Bpev01.c0778.g0005.m0001	1.174029488	0.00032338	0.00419194	DEG
Bpev01.c0644.g0005.m0001	1.246352436	0.00114241	0.01150155	DEG
Bpev01.c1748.g0003.m0001	-1.622825471	0.00612999	0.04264197	DEG
Bpev01.c0357.g0068.m0001	2.859554437	1.35E-09	1.16E-07	DEG
Bpev01.c0451.g0020.m0002	2.509698611	9.53E-06	0.00023661	DEG
Bpev01.c0536.g0007.m0001	1.302821921	3.16E-05	0.00063443	DEG
Bpev01.c0449.g0028.m0001	-1.036554721	0.00336452	0.02722232	DEG
Bpev01.c0279.g0034.m0001	1.459992471	1.10E-05	0.00026685	DEG
Bpev01.c0408.g0005.m0001	-3.343092842	0.00039687	0.00498653	DEG
Bpev01.c0286.g0039.m0001	-1.970091491	0.00105186	0.01074452	DEG
Bpev01.c1136.g0002.m0001	-2.006504105	2.59E-09	2.04E-07	DEG
Bpev01.c0016.g0110.m0001	0.912636572	0.00498434	0.03658292	DEG
Bpev01.c0350.g0003.m0001	0.961318146	0.00172257	0.01605786	DEG
Bpev01.c0052.g0017.m0001	-0.936971822	0.00214483	0.01905878	DEG
Bpev01.c1668.g0001.m0001	2.870023434	0.00193055	0.01759937	DEG
Bpev01.c1114.g0005.m0001	-1.418926752	6.56E-06	0.00017221	DEG
Bpev01.c0929.g0016.m0002	0.891369532	0.0045405	0.03408902	DEG
Bpev01.c1074.g0002.m0001	3.930803635	1.38E-12	2.31E-10	DEG
Bpev01.c0112.g0019.m0001	1.937722917	0.00739975	0.04922965	DEG
Bpev01.c0518.g0058.m0001	-2.296566309	7.09E-06	0.00018467	DEG
Bpev01.c1874.g0004.m0001	-1.289579687	0.00019792	0.00282292	DEG
Bpev01.c0037.g0035.m0001	2.211486665	1.00E-09	9.03E-08	DEG
Bpev01.c0265.g0015.m0001	1.14313497	0.00089338	0.00949461	DEG
Bpev01.c0000.g0189.m0001	1.11703152	0.00099224	0.01027089	DEG
Bpev01.c0237.g0055.m0001	-1.948563401	0.00682093	0.04635304	DEG
Bpev01.c0052.g0090.m0001	1.32418787	4.09E-05	0.00078467	DEG
Bpev01.c0016.g0045.m0001	-1.39278586	0.00109811	0.01112045	DEG
Bpev01.c0245.g0030.m0001	4.176066704	5.90E-17	2.47E-14	DEG
Bpev01.c0455.g0024.m0001	-1.007374099	0.00092126	0.00971035	DEG
Bpev01.c0828.g0018.m0001	-0.906584721	0.00350197	0.02810642	DEG
Bpev01.c0051.g0072.m0001	1.767551438	0.00098937	0.01025577	DEG
Bpev01.c0984.g0027.m0001	2.508684796	0.00073039	0.00808036	DEG
Bpev01.c0624.g0010.m0001	1.416454871	0.00389567	0.03045204	DEG
Bpev01.c1610.g0008.m0002	3.313194752	3.09E-07	1.28E-05	DEG
Bpev01.c0837.g0010.m0001	-1.057236356	0.0013616	0.01324661	DEG
Bpev01.c0094.g0167.m0001	-2.854523953	5.28E-15	1.48E-12	DEG
Bpev01.c0633.g0004.m0001	2.005025059	4.82E-08	2.63E-06	DEG
Bpev01.c0243.g0005.m0001	1.228285018	0.00053198	0.00633539	DEG
Bpev01.c1090.g0001.m0001	-2.676385998	3.52E-10	3.53E-08	DEG

Bpev01.c0080.g0007.m0001	-2.729648159	8.69E-05	0.00144454	DEG
Bpev01.c0050.g0083.m0001	-1.51619626	1.35E-05	0.00031876	DEG
Bpev01.c0275.g0056.m0001	-2.430928704	6.31E-06	0.0001665	DEG
Bpev01.c1241.g0001.m0001	0.970790855	0.00567616	0.04024577	DEG
Bpev01.c0566.g0043.m0001	-2.258919335	0.00072038	0.00801114	DEG
Bpev01.c1727.g0002.m0001	2.378618006	1.03E-08	6.95E-07	DEG
Bpev01.c0022.g0177.m0003	-1.078559134	0.00478995	0.03554607	DEG
Bpev01.c0663.g0003.m0001	2.914863412	0.00013177	0.00204375	DEG
Bpev01.c0154.g0072.m0001	-2.0953612	0.00036535	0.00465268	DEG
Bpev01.c0337.g0005.m0001	1.33267147	1.87E-05	0.00041378	DEG
Bpev01.c0229.g0023.m0001	-2.278867887	0.00411811	0.03171151	DEG
Bpev01.c0154.g0066.m0001	3.06670779	0.00016321	0.00242172	DEG
Bpev01.c1675.g0003.m0001	2.816071982	0.00011807	0.00186371	DEG
Bpev01.c0819.g0010.m0001	1.096447131	0.00045963	0.00561918	DEG
Bpev01.c0162.g0023.m0001	-1.411302699	3.82E-06	0.00010932	DEG
Bpev01.c0214.g0044.m0001	2.445557593	0.0003728	0.00473466	DEG
Bpev01.c1476.g0002.m0001	-1.951585488	0.00122369	0.01213344	DEG
Bpev01.c0511.g0003.m0001	1.65076584	0.00036015	0.00459179	DEG
Bpev01.c0038.g0146.m0001	-1.504431617	4.61E-06	0.00012761	DEG
Bpev01.c0018.g0099.m0001	3.152079434	3.05E-10	3.10E-08	DEG
Bpev01.c0613.g0010.m0002	0.914461166	0.00523431	0.03798194	DEG
Bpev01.c0191.g0021.m0001	-1.452624549	0.00119939	0.01194478	DEG
Bpev01.c0652.g0011.m0001	-2.114240372	0.00041613	0.00518861	DEG
Bpev01.c0818.g0006.m0001	5.217938658	7.26E-34	5.96E-30	DEG
Bpev01.c0120.g0048.m0001	0.899984836	0.00321202	0.02626712	DEG
Bpev01.c0978.g0008.m0001	3.246873065	6.08E-05	0.00108058	DEG
Bpev01.c1017.g0001.m0001	1.486613677	0.0029004	0.02422076	DEG
Bpev01.c0794.g0011.m0001	-1.189775363	0.00015758	0.00235805	DEG
Bpev01.c0088.g0126.m0001	3.803273901	0.00041508	0.00517771	DEG
Bpev01.c0397.g0016.m0001	1.236844112	0.00305359	0.02521591	DEG
Bpev01.c0190.g0088.m0001	-2.045851656	3.47E-07	1.41E-05	DEG
Bpev01.c0483.g0016.m0001	-3.15173571	0.00109967	0.01113247	DEG
Bpev01.c0276.g0018.m0002	4.616566831	3.10E-19	2.10E-16	DEG
Bpev01.c1372.g0003.m0001	1.425412572	0.00085848	0.00921687	DEG
Bpev01.c1123.g0002.m0001	-1.284762476	0.00044973	0.0055257	DEG
Bpev01.c0671.g0012.m0002	-2.342055978	1.03E-06	3.67E-05	DEG
Bpev01.c0214.g0023.m0001	-1.786293193	0.00501162	0.03667339	DEG
Bpev01.c0298.g0002.m0001	1.45986499	0.00024984	0.00339727	DEG
Bpev01.c1327.g0002.m0001	-1.142074716	0.00059956	0.00692171	DEG
Bpev01.c0171.g0024.m0001	-0.903100058	0.00369078	0.02923166	DEG
Bpev01.c0018.g0075.m0001	2.389050036	3.43E-05	0.00068024	DEG
Bpev01.c1031.g0014.m0001	-1.992434733	7.88E-08	4.01E-06	DEG
Bpev01.c1042.g0004.m0001	-1.338848539	0.0013861	0.01341599	DEG

Bpev01.c0657.g0004.m0001	1.582947194	0.00069121	0.00774595	DEG
Bpev01.c0525.g0009.m0001	-1.038150494	0.00243342	0.02104506	DEG
Bpev01.c0112.g0002.m0001	-3.252187171	2.79E-05	0.00057501	DEG
Bpev01.c0299.g0032.m0001	1.18946026	0.00101596	0.01044729	DEG
Bpev01.c0580.g0009.m0001	3.114866949	2.34E-08	1.40E-06	DEG
Bpev01.c0121.g0041.m0001	1.026466955	0.00476173	0.03539021	DEG
Bpev01.c0129.g0020.m0001	-0.979452275	0.00429069	0.03263078	DEG
Bpev01.c0025.g0007.m0001	1.018631051	0.00110069	0.01113895	DEG
Bpev01.c0349.g0049.m0001	1.80246607	4.13E-06	0.00011599	DEG
Bpev01.c1553.g0007.m0001	-1.278635934	5.70E-05	0.00102813	DEG
Bpev01.c0848.g0030.m0001	-1.418352914	4.67E-05	0.00087397	DEG
Bpev01.c0298.g0008.m0001	-1.415849932	3.60E-05	0.00070548	DEG
Bpev01.c1938.g0015.m0001	-0.922023751	0.00513808	0.03740352	DEG
Bpev01.c0118.g0050.m0001	1.30352439	0.00124223	0.01228418	DEG
Bpev01.c0974.g0010.m0001	1.471173722	0.00010004	0.00161814	DEG
Bpev01.c0351.g0001.m0001	-1.970180271	0.00307705	0.02538826	DEG
Bpev01.c0594.g0005.m0001	2.219174664	0.00505361	0.03692556	DEG
Bpev01.c2192.g0001.m0001	-1.009858219	0.00471226	0.03511118	DEG
Bpev01.c0015.g0025.m0001	1.443744627	0.00298636	0.02479193	DEG
Bpev01.c0037.g0016.m0001	-7.031725474	0.00114941	0.01154863	DEG
Bpev01.c0163.g0009.m0001	-1.679552872	8.50E-05	0.00141991	DEG
Bpev01.c1259.g0004.m0001	3.241888517	0.00087327	0.00933486	DEG
Bpev01.c0190.g0049.m0001	0.933282721	0.00532482	0.03843976	DEG
Bpev01.c0714.g0012.m0001	-1.916434365	0.00149761	0.01433757	DEG
Bpev01.c1116.g0010.m0001	-6.24204997	0.00731071	0.04882483	DEG
Bpev01.c2476.g0003.m0001	3.448114148	6.24E-06	0.000165	DEG
Bpev01.c0146.g0005.m0001	6.646069877	0.00312452	0.02570459	DEG
Bpev01.c0574.g0029.m0001	-1.997329092	0.00241397	0.02091377	DEG
Bpev01.c0162.g0002.m0001	-1.577848195	0.0001403	0.00214567	DEG
Bpev01.c0447.g0014.m0001	0.894347135	0.00607776	0.04232862	DEG
Bpev01.c0706.g0004.m0001	2.524160889	1.21E-06	4.19E-05	DEG
Bpev01.c0161.g0052.m0001	1.806103415	1.48E-07	6.85E-06	DEG
Bpev01.c0772.g0009.m0001	-2.118822004	9.22E-06	0.00023055	DEG
Bpev01.c0483.g0011.m0001	1.558173259	0.00015353	0.00230487	DEG
Bpev01.c0795.g0009.m0001	1.30306761	0.00403765	0.03127187	DEG
Bpev01.c2139.g0004.m0001	5.209286914	4.93E-23	5.81E-20	DEG
Bpev01.c1871.g0006.m0001	1.399214311	1.09E-05	0.00026488	DEG
Bpev01.c1877.g0001.m0001	-2.147493127	5.85E-06	0.00015646	DEG
Bpev01.c0001.g0048.m0001	-0.84110768	0.00694031	0.04695835	DEG
Bpev01.c2218.g0001.m0001	2.783485801	6.19E-07	2.36E-05	DEG
Bpev01.c2516.g0002.m0001	2.086174449	0.00085484	0.00918784	DEG
Bpev01.c1404.g0002.m0001	3.879814091	1.74E-05	0.00039155	DEG
Bpev01.c1584.g0007.m0001	4.701046338	2.20E-07	9.57E-06	DEG

Bpev01.c0280.g0032.m0001	1.986539492	0.00402758	0.03122286	DEG
Bpev01.c0451.g0004.m0001	1.80376892	1.46E-07	6.80E-06	DEG
Bpev01.c0243.g0036.m0001	-1.063683506	0.00091812	0.00968766	DEG
Bpev01.c1093.g0006.m0001	4.604159212	2.57E-07	1.09E-05	DEG
Bpev01.c1141.g0006.m0001	-1.743862265	0.00348435	0.02800861	DEG
Bpev01.c0577.g0026.m0001	-2.026384846	3.52E-06	0.00010266	DEG
Bpev01.c0344.g0046.m0001	-1.552051596	0.00406519	0.03140256	DEG
Bpev01.c2215.g0004.m0001	1.498322453	0.00293661	0.02446622	DEG
Bpev01.c0089.g0052.m0003	1.527492279	2.95E-06	8.82E-05	DEG
Bpev01.c0594.g0020.m0001	-3.731779815	0.00555544	0.03964764	DEG
Bpev01.c0022.g0033.m0001	-1.72793375	4.37E-08	2.42E-06	DEG
Bpev01.c1247.g0001.m0001	2.56915041	1.52E-07	7.00E-06	DEG
Bpev01.c4891.g0001.m0001	-1.814245453	2.49E-06	7.60E-05	DEG
Bpev01.c1167.g0008.m0001	-1.726055873	0.00189112	0.01733919	DEG
Bpev01.c0268.g0003.m0001	2.615426347	2.98E-14	6.97E-12	DEG
Bpev01.c1006.g0017.m0001	-2.03868973	2.09E-08	1.26E-06	DEG
Bpev01.c3848.g0001.m0001	2.25799453	5.95E-05	0.00106507	DEG
Bpev01.c0264.g0009.m0001	1.084710479	0.00040245	0.00503735	DEG
Bpev01.c1803.g0004.m0001	2.618274406	5.92E-06	0.00015817	DEG
Bpev01.c0022.g0110.m0001	-0.860793376	0.00563549	0.04002487	DEG
Bpev01.c0121.g0063.m0001	1.213919368	0.00013867	0.00212886	DEG
Bpev01.c0045.g0072.m0001	2.878988725	3.68E-14	8.46E-12	DEG
Bpev01.c0066.g0003.m0001	-1.383965319	0.00284253	0.02384084	DEG
Bpev01.c2045.g0004.m0001	-1.098473642	0.000969	0.01008807	DEG
Bpev01.c1431.g0003.m0001	3.066032526	1.52E-09	1.29E-07	DEG
Bpev01.c0564.g0019.m0001	-1.034416893	0.00190882	0.01745691	DEG
Bpev01.c0080.g0100.m0001	2.776608454	0.00197498	0.01790619	DEG
Bpev01.c0439.g0017.m0001	1.250302616	0.0007464	0.00821122	DEG
Bpev01.c0564.g0023.m0001	2.246059442	6.73E-11	7.86E-09	DEG
Bpev01.c1167.g0021.m0001	-2.812074462	6.06E-08	3.19E-06	DEG
Bpev01.c1958.g0003.m0001	-2.648858633	0.00390387	0.03048436	DEG
Bpev01.c0597.g0024.m0001	-0.903776504	0.00346224	0.02786354	DEG
Bpev01.c0062.g0079.m0001	1.654706244	4.60E-05	0.00086404	DEG
Bpev01.c1565.g0010.m0001	-6.44008693	0.00670653	0.04574458	DEG
Bpev01.c1447.g0005.m0001	3.224993116	0.00295738	0.02458696	DEG
Bpev01.c0122.g0009.m0001	3.400358898	4.50E-20	3.48E-17	DEG
Bpev01.c1338.g0009.m0001	-1.152348264	0.00068779	0.00772518	DEG
Bpev01.c0161.g0067.m0001	0.859260648	0.00621347	0.04314646	DEG
Bpev01.c0105.g0009.m0001	-1.315521538	5.49E-05	0.00099346	DEG
Bpev01.c0029.g0146.m0001	-1.255618202	0.00020694	0.00292461	DEG
Bpev01.c0038.g0046.m0001	3.060369126	2.88E-14	6.85E-12	DEG
Bpev01.c1382.g0011.m0001	-2.145190297	1.83E-06	5.87E-05	DEG
Bpev01.c3008.g0002.m0001	6.743004933	0.00360616	0.02877013	DEG

Bpev01.c1216.g0006.m0001	2.217633407	3.65E-07	1.48E-05	DEG
Bpev01.c0657.g0006.m0001	1.852805063	0.00044289	0.00546285	DEG
Bpev01.c0386.g0005.m0001	-3.17634729	0.00024331	0.00333772	DEG
Bpev01.c1077.g0011.m0001	-2.630379679	0.00210945	0.0188125	DEG
Bpev01.c0053.g0034.m0001	-1.223764788	0.0065513	0.04497753	DEG
Bpev01.c0115.g0018.m0001	1.17866477	0.00048798	0.00591416	DEG
Bpev01.c0053.g0013.m0001	2.287141216	8.22E-07	3.03E-05	DEG
Bpev01.c0169.g0041.m0001	0.948809622	0.00329779	0.02680386	DEG
Bpev01.c2510.g0001.m0001	-1.199600249	0.00088825	0.00946062	DEG
Bpev01.c0518.g0060.m0001	-0.889517032	0.00340561	0.02750027	DEG
Bpev01.c2003.g0005.m0001	1.454504537	0.00678935	0.04618718	DEG
Bpev01.c0045.g0031.m0001	1.581953939	7.98E-05	0.00134672	DEG
Bpev01.c0389.g0025.m0001	-1.189848287	0.00645379	0.04447405	DEG
Bpev01.c0062.g0014.m0002	-0.985592117	0.00374122	0.02953547	DEG
Bpev01.c0720.g0003.m0001	1.409573978	8.01E-05	0.00135005	DEG
Bpev01.c0171.g0038.m0001	2.30090508	0.002093	0.01871685	DEG
Bpev01.c1851.g0007.m0001	-2.108342931	0.00052176	0.00623636	DEG
Bpev01.c0045.g0032.m0001	1.032054337	0.00068044	0.00767325	DEG
Bpev01.c0444.g0018.m0001	0.862484905	0.00427189	0.03252983	DEG
Bpev01.c0374.g0015.m0001	-1.707901445	0.00466278	0.03483063	DEG
Bpev01.c0115.g0009.m0002	-1.013362689	0.00524126	0.03800431	DEG
Bpev01.c2653.g0002.m0001	-1.733477099	1.22E-07	5.78E-06	DEG
Bpev01.c0437.g0015.m0001	-1.069164478	0.00239076	0.02074932	DEG
Bpev01.c0242.g0009.m0001	1.54041223	0.00019178	0.00275837	DEG
Bpev01.c0574.g0037.m0001	-2.26662858	0.00017263	0.00253602	DEG
Bpev01.c0015.g0130.m0001	-1.252896504	0.00064096	0.00730618	DEG
Bpev01.c0051.g0121.m0001	-1.498300568	0.00028696	0.00381213	DEG
Bpev01.c0580.g0018.m0001	4.505022513	6.45E-10	6.01E-08	DEG
Bpev01.c1764.g0013.m0001	1.601973152	1.18E-05	0.00028253	DEG
Bpev01.c0281.g0097.m0001	-1.641619029	0.00677511	0.04612686	DEG
Bpev01.c0652.g0013.m0001	-3.117622893	0.00067885	0.00765986	DEG
Bpev01.c0050.g0160.m0001	1.846939954	5.96E-06	0.00015913	DEG
Bpev01.c0018.g0076.m0001	2.045346106	4.02E-09	2.99E-07	DEG
Bpev01.c0089.g0057.m0001	1.171247408	0.00281418	0.0236705	DEG
Bpev01.c0015.g0124.m0001	-2.340004018	0.00325656	0.02654939	DEG
Bpev01.c0700.g0011.m0001	-2.318678135	5.33E-06	0.00014429	DEG
Bpev01.c1560.g0007.m0001	-1.36770453	0.00417871	0.03200234	DEG
Bpev01.c0683.g0005.m0001	-1.763227199	2.25E-05	0.00047893	DEG
Bpev01.c0579.g0026.m0001	-1.946524072	1.38E-05	0.00032366	DEG
Bpev01.c1183.g0022.m0001	-2.269853772	0.00104122	0.01065061	DEG
Bpev01.c1152.g0003.m0001	1.036451266	0.00232891	0.02033845	DEG
Bpev01.c1805.g0002.m0001	1.065879372	0.00499055	0.03658321	DEG
Bpev01.c0051.g0186.m0001	-2.208727594	0.00084532	0.00909888	DEG

Bpev01.c0803.g0018.m0001	-1.642516237	0.0012519	0.01236321	DEG
Bpev01.c0135.g0021.m0001	-1.154589044	0.00541908	0.03900249	DEG
Bpev01.c1130.g0008.m0001	-1.961079968	0.00279748	0.02355018	DEG
Bpev01.c0082.g0003.m0001	-0.932683713	0.00189214	0.01733935	DEG
Bpev01.c0029.g0135.m0002	1.600070194	0.0041234	0.03173564	DEG
Bpev01.c0478.g0020.m0001	1.217459778	0.00599715	0.04195585	DEG
Bpev01.c3876.g0002.m0003	1.227773139	0.00746474	0.04957244	DEG
Bpev01.c1024.g0015.m0002	-1.819398748	1.53E-06	5.04E-05	DEG
Bpev01.c0812.g0007.m0001	-2.516576886	0.00515457	0.03750505	DEG
Bpev01.c0015.g0250.m0001	-1.925647115	1.69E-09	1.41E-07	DEG
Bpev01.c0349.g0058.m0001	-1.172398605	0.00172084	0.01604679	DEG
Bpev01.c0080.g0091.m0001	0.848936113	0.00466434	0.03483346	DEG
Bpev01.c0053.g0026.m0001	0.874413158	0.00400895	0.0311069	DEG
Bpev01.c1893.g0004.m0001	6.03633077	2.84E-19	1.99E-16	DEG
Bpev01.c0343.g0032.m0001	1.83021826	2.22E-05	0.00047498	DEG
Bpev01.c0115.g0047.m0001	1.117057779	0.00031942	0.00414982	DEG
Bpev01.c0237.g0038.m0001	2.119883036	4.86E-05	0.00090682	DEG
Bpev01.c1402.g0005.m0001	-1.015371137	0.00335337	0.02715551	DEG
Bpev01.c0478.g0014.m0001	-2.568080414	0.0029746	0.02471612	DEG
Bpev01.c0240.g0005.m0001	-0.908728608	0.00422589	0.03226299	DEG
Bpev01.c0158.g0032.m0001	1.493182762	1.88E-05	0.00041431	DEG
Bpev01.c1644.g0004.m0001	1.828931805	0.00644919	0.04446319	DEG
Bpev01.c0565.g0016.m0001	-1.2808962	0.00028206	0.00376237	DEG
Bpev01.c0114.g0007.m0001	-0.895553036	0.00459718	0.03442799	DEG
Bpev01.c0994.g0010.m0001	-1.977131221	0.00020019	0.0028457	DEG
Bpev01.c1060.g0020.m0001	-1.643713022	0.00402814	0.03122286	DEG
Bpev01.c0428.g0017.m0001	-1.044719294	0.00654433	0.04494525	DEG
Bpev01.c0485.g0007.m0001	-6.313121254	0.0049876	0.03658321	DEG
Bpev01.c0015.g0011.m0001	-1.701129657	0.00039191	0.00494538	DEG
Bpev01.c0401.g0038.m0001	1.667254587	0.00566778	0.04021003	DEG
Bpev01.c2973.g0002.m0001	-2.382304764	0.00314592	0.02582616	DEG
Bpev01.c0001.g0065.m0001	-2.968962719	0.00095703	0.00999533	DEG
Bpev01.c1577.g0023.m0001	-2.508307274	8.37E-06	0.00021332	DEG
Bpev01.c1072.g0028.m0001	-1.970607997	2.32E-06	7.18E-05	DEG
Bpev01.c0029.g0009.m0001	-2.482081303	0.00029822	0.00393168	DEG
Bpev01.c0294.g0014.m0001	3.07571736	1.63E-05	0.0003709	DEG
Bpev01.c1647.g0003.m0001	4.599921589	5.37E-20	4.05E-17	DEG
Bpev01.c0474.g0001.m0001	-1.665912247	6.05E-05	0.00107753	DEG
Bpev01.c0115.g0058.m0001	-2.877553642	0.00014748	0.00222771	DEG
Bpev01.c0000.g0142.m0001	3.857425292	7.14E-22	7.22E-19	DEG
Bpev01.c0180.g0001.m0001	4.227726857	1.12E-12	1.90E-10	DEG
Bpev01.c0401.g0013.m0001	1.308442323	2.20E-05	0.00047187	DEG
Bpev01.c1560.g0014.m0001	0.946697927	0.00189124	0.01733919	DEG

Bpev01.c0572.g0011.m0001	1.462437139	0.00201783	0.01816049	DEG
Bpev01.c0223.g0045.m0001	-3.090039531	1.56E-14	3.98E-12	DEG
Bpev01.c0189.g0059.m0001	2.585133066	8.60E-07	3.15E-05	DEG
Bpev01.c0552.g0004.m0001	-2.263869348	3.25E-06	9.58E-05	DEG
Bpev01.c0331.g0010.m0001	-0.994040905	0.00176221	0.01637551	DEG
Bpev01.c0157.g0081.m0001	1.19052753	0.00066119	0.00749226	DEG
Bpev01.c4073.g0001.m0001	4.409092962	1.00E-08	6.82E-07	DEG
Bpev01.c0015.g0055.m0001	2.542038077	1.04E-07	4.98E-06	DEG
Bpev01.c0068.g0004.m0001	6.478496187	6.08E-24	7.79E-21	DEG
Bpev01.c1185.g0009.m0001	1.758940986	2.98E-05	0.00060563	DEG
Bpev01.c0922.g0003.m0001	-1.983913172	0.00459132	0.03440157	DEG
Bpev01.c0416.g0005.m0001	2.343822108	1.82E-09	1.51E-07	DEG
Bpev01.c0029.g0108.m0001	2.864996813	1.88E-14	4.69E-12	DEG
Bpev01.c0224.g0011.m0001	1.552939933	0.00032763	0.0042396	DEG
Bpev01.c0088.g0024.m0001	-1.069787126	0.00058984	0.00683139	DEG
Bpev01.c0119.g0005.m0001	-2.729823174	2.77E-07	1.17E-05	DEG
Bpev01.c0527.g0002.m0001	1.430048746	0.00036146	0.00460655	DEG
Bpev01.c1356.g0004.m0002	-2.61776374	0.00231424	0.02025235	DEG
Bpev01.c0451.g0003.m0001	1.634002315	0.0036296	0.02892575	DEG
Bpev01.c0066.g0042.m0001	3.713674059	2.11E-07	9.21E-06	DEG
Bpev01.c1211.g0014.m0001	4.22020154	1.91E-06	6.11E-05	DEG
Bpev01.c0441.g0005.m0001	-1.953931477	9.46E-05	0.00155012	DEG
Bpev01.c0659.g0007.m0001	-1.990542215	8.71E-05	0.00144577	DEG
Bpev01.c0281.g0007.m0001	-0.996780002	0.00509787	0.03716595	DEG
Bpev01.c0095.g0012.m0001	-1.45176881	0.00022095	0.00307975	DEG
Bpev01.c0497.g0003.m0001	1.895253251	9.84E-06	0.00024324	DEG
Bpev01.c0198.g0001.m0001	-1.209772764	0.000138	0.00212139	DEG
Bpev01.c1758.g0003.m0001	-1.003286493	0.00333763	0.02706791	DEG
Bpev01.c0557.g0043.m0001	8.088887306	1.33E-05	0.00031405	DEG
Bpev01.c1422.g0004.m0001	0.998449794	0.00676846	0.0460922	DEG
Bpev01.c0374.g0014.m0001	1.201598746	0.00525648	0.03808652	DEG
Bpev01.c1456.g0013.m0001	1.013569327	0.00099786	0.01032547	DEG
Bpev01.c0566.g0065.m0001	1.111382797	0.00555133	0.03963752	DEG
Bpev01.c0243.g0055.m0001	-0.856925519	0.00656531	0.04506323	DEG
Bpev01.c0036.g0013.m0001	-1.603116628	0.00100341	0.01036108	DEG
Bpev01.c0118.g0006.m0002	1.795270762	1.78E-08	1.10E-06	DEG
Bpev01.c0259.g0005.m0001	2.441926242	0.00028362	0.00377638	DEG
Bpev01.c1499.g0005.m0002	-1.518447056	3.39E-05	0.00067423	DEG
Bpev01.c1972.g0007.m0001	0.89458376	0.00510969	0.03722446	DEG
Bpev01.c0329.g0003.m0001	2.749026129	9.36E-15	2.48E-12	DEG
Bpev01.c0415.g0033.m0001	-2.241721241	0.00029225	0.00386671	DEG
Bpev01.c0445.g0003.m0001	2.312688373	0.00020194	0.00286233	DEG
Bpev01.c0452.g0022.m0001	-2.407524636	8.74E-07	3.20E-05	DEG

Bpev01.c2029.g0005.m0001	2.19333987	4.10E-05	0.00078496	DEG
Bpev01.c1118.g0002.m0001	0.95612278	0.00145148	0.01396406	DEG
Bpev01.c0127.g0111.m0001	-1.292832333	0.0009723	0.01011072	DEG
Bpev01.c2140.g0003.m0001	1.043582247	0.00171949	0.0160393	DEG
Bpev01.c0000.g0015.m0001	1.22976747	0.00103268	0.01058169	DEG
Bpev01.c2085.g0004.m0002	0.918660209	0.00293781	0.02446622	DEG
Bpev01.c0000.g0201.m0001	3.034923328	6.92E-09	4.89E-07	DEG
Bpev01.c2346.g0005.m0001	2.887967107	1.59E-11	2.06E-09	DEG
Bpev01.c0415.g0019.m0003	-1.551282799	2.02E-05	0.00043938	DEG
Bpev01.c0450.g0009.m0001	5.052789442	3.87E-07	1.56E-05	DEG
Bpev01.c0915.g0021.m0002	1.363018418	0.00100269	0.01035721	DEG
Bpev01.c0363.g0006.m0001	1.460990049	0.00117258	0.01173508	DEG
Bpev01.c0496.g0025.m0001	1.260297336	0.00058386	0.00678043	DEG
Bpev01.c0324.g0002.m0001	-0.948214958	0.00314155	0.02579755	DEG
Bpev01.c0171.g0036.m0001	1.144081936	0.00148627	0.0142383	DEG
Bpev01.c2937.g0002.m0001	-1.24201734	0.00026162	0.0035321	DEG
Bpev01.c1061.g0004.m0001	-2.137235067	0.0038049	0.02990989	DEG
Bpev01.c3054.g0002.m0001	1.794375252	1.27E-05	0.00030307	DEG
Bpev01.c1952.g0004.m0001	1.343549782	6.82E-05	0.00118495	DEG
Bpev01.c1560.g0002.m0001	-1.029729695	0.00263949	0.02249676	DEG
Bpev01.c0702.g0009.m0001	-0.931702645	0.00383852	0.03010183	DEG
Bpev01.c0142.g0050.m0002	1.975761344	0.00097275	0.01011188	DEG
Bpev01.c0878.g0016.m0001	-2.513337171	1.39E-06	4.71E-05	DEG
Bpev01.c0518.g0029.m0001	1.665780962	5.29E-05	0.00096785	DEG
Bpev01.c0224.g0037.m0001	-1.248490863	0.00024869	0.00338468	DEG
Bpev01.c0038.g0056.m0001	3.133721602	7.25E-07	2.71E-05	DEG
Bpev01.c0001.g0060.m0001	-1.641519705	0.00098678	0.01023958	DEG
Bpev01.c0044.g0004.m0001	1.059072853	0.00231608	0.02026244	DEG
Bpev01.c1337.g0006.m0001	1.763712807	1.72E-07	7.76E-06	DEG
Bpev01.c0843.g0002.m0001	-1.408991527	0.00103068	0.0105669	DEG
Bpev01.c0942.g0002.m0001	1.123296857	0.00044945	0.00552452	DEG
Bpev01.c0415.g0016.m0001	1.548006595	2.48E-06	7.59E-05	DEG
Bpev01.c0556.g0007.m0001	1.933598121	0.00013399	0.00207388	DEG
Bpev01.c0130.g0004.m0001	-1.495436465	6.03E-06	0.00016029	DEG
Bpev01.c1681.g0005.m0001	2.19225134	1.94E-06	6.17E-05	DEG
Bpev01.c1721.g0010.m0001	6.713004857	0.00188773	0.01731904	DEG
Bpev01.c1028.g0014.m0001	1.28895241	0.00608382	0.04236079	DEG
Bpev01.c0984.g0012.m0001	-1.611496247	2.53E-05	0.00052791	DEG
Bpev01.c0332.g0014.m0001	-1.199392441	0.00698534	0.04717625	DEG
Bpev01.c1043.g0003.m0001	1.415606236	0.00445064	0.03357605	DEG
Bpev01.c0478.g0017.m0001	-2.137941932	0.00054587	0.00643058	DEG
Bpev01.c0531.g0023.m0001	1.281586926	0.00018913	0.00273193	DEG
Bpev01.c1051.g0002.m0001	1.813464762	0.00186954	0.01718848	DEG

Bpev01.c0566.g0037.m0001	4.693167031	1.12E-29	4.70E-26	DEG
Bpev01.c0015.g0096.m0001	-1.938254269	1.18E-09	1.04E-07	DEG
Bpev01.c0050.g0152.m0001	1.039418686	0.00156428	0.01484556	DEG
Bpev01.c0755.g0009.m0001	2.776418965	1.93E-06	6.15E-05	DEG
Bpev01.c0715.g0009.m0001	0.864447419	0.00702814	0.04740008	DEG
Bpev01.c0062.g0082.m0002	-2.200654733	6.31E-05	0.00111044	DEG
Bpev01.c0177.g0045.m0001	-1.775946989	6.48E-08	3.36E-06	DEG
Bpev01.c0015.g0070.m0001	1.222323823	0.00036948	0.0046985	DEG
Bpev01.c0159.g0029.m0001	2.014961045	0.00138482	0.01341222	DEG
Bpev01.c1161.g0008.m0001	1.156806554	0.00136243	0.0132503	DEG
Bpev01.c0967.g0003.m0001	2.252356541	0.00032105	0.00416728	DEG
Bpev01.c1456.g0014.m0001	1.125410217	0.00095042	0.00994296	DEG
Bpev01.c0129.g0100.m0001	-2.164378927	0.0008552	0.00918844	DEG
Bpev01.c0213.g0046.m0001	-2.272206065	8.68E-09	5.97E-07	DEG
Bpev01.c0610.g0005.m0001	1.580620599	4.10E-05	0.00078506	DEG
Bpev01.c2082.g0001.m0001	2.852547555	8.49E-09	5.87E-07	DEG
Bpev01.c0963.g0005.m0001	-1.113977871	0.00557968	0.03973618	DEG
Bpev01.c0001.g0016.m0001	-1.712842413	1.50E-07	6.93E-06	DEG
Bpev01.c0089.g0032.m0001	-1.020645651	0.00137791	0.01336989	DEG
Bpev01.c0292.g0019.m0001	1.043034186	0.00051014	0.00612483	DEG
Bpev01.c0657.g0009.m0001	3.142414797	3.77E-09	2.82E-07	DEG
Bpev01.c1234.g0001.m0001	1.194684859	0.00712702	0.04795108	DEG
Bpev01.c0473.g0007.m0001	-2.213399755	5.37E-09	3.89E-07	DEG
Bpev01.c0278.g0046.m0001	2.055365776	0.00038657	0.00488423	DEG
Bpev01.c2227.g0002.m0001	2.272204581	0.00170002	0.01589102	DEG
Bpev01.c0376.g0001.m0001	1.5588331	0.00204897	0.01839017	DEG
Bpev01.c0177.g0043.m0001	-1.396445061	0.00448059	0.03375229	DEG
Bpev01.c0396.g0004.m0001	-1.468081346	1.96E-05	0.00043037	DEG
Bpev01.c0624.g0021.m0001	-0.880921628	0.00577487	0.04073959	DEG
Bpev01.c0874.g0032.m0001	-1.271803065	0.0004989	0.00602172	DEG
Bpev01.c1699.g0004.m0001	1.440872856	2.82E-06	8.49E-05	DEG
Bpev01.c0142.g0092.m0001	2.592134453	5.27E-08	2.84E-06	DEG
Bpev01.c0531.g0015.m0001	-1.355879004	6.80E-05	0.00118186	DEG
Bpev01.c1115.g0007.m0001	2.110746935	6.90E-07	2.60E-05	DEG
Bpev01.c0506.g0011.m0001	-2.00885774	8.46E-08	4.23E-06	DEG
Bpev01.c0164.g0005.m0001	1.244960114	0.00017498	0.00256533	DEG
Bpev01.c0038.g0154.m0001	1.40150925	0.00198254	0.01795237	DEG
Bpev01.c0989.g0005.m0001	1.169740681	0.00366365	0.02911833	DEG
Bpev01.c0944.g0009.m0001	-1.727852276	5.88E-08	3.10E-06	DEG
Bpev01.c0018.g0007.m0001	2.502335193	6.11E-05	0.00108471	DEG
Bpev01.c0420.g0023.m0001	1.02385836	0.00100055	0.0103424	DEG
Bpev01.c0092.g0005.m0001	1.308974898	0.0003797	0.00480979	DEG
Bpev01.c0344.g0029.m0001	-1.185458746	0.00252487	0.0217021	DEG

Bpev01.c0758.g0005.m0001	1.056927229	0.00410658	0.03165581	DEG
Bpev01.c0371.g0006.m0001	-1.978973467	0.00019275	0.00277065	DEG
Bpev01.c0171.g0020.m0001	-1.868140226	3.64E-06	0.00010557	DEG
Bpev01.c1292.g0010.m0001	3.537904963	3.29E-09	2.53E-07	DEG
Bpev01.c0045.g0058.m0001	-1.270385734	0.00053278	0.00634234	DEG
Bpev01.c1424.g0002.m0001	-2.149244429	2.82E-10	2.90E-08	DEG
Bpev01.c0257.g0017.m0001	1.027544295	0.00201499	0.01814599	DEG
Bpev01.c2297.g0002.m0001	-2.349404821	6.98E-05	0.00120613	DEG
Bpev01.c0062.g0080.m0001	1.224007779	0.00217497	0.01928587	DEG
Bpev01.c0762.g0011.m0001	1.191214637	0.00700812	0.0472976	DEG
Bpev01.c0210.g0051.m0001	1.532659274	1.98E-05	0.00043375	DEG
Bpev01.c0205.g0024.m0001	1.004587846	0.00147356	0.01413956	DEG
Bpev01.c0594.g0004.m0001	1.76800269	0.00125988	0.01242953	DEG
Bpev01.c0274.g0017.m0001	-2.90245481	0.00368883	0.0292316	DEG
Bpev01.c1491.g0003.m0001	1.909341274	0.00011315	0.00179956	DEG
Bpev01.c0080.g0108.m0001	2.200497855	0.00335397	0.02715551	DEG
Bpev01.c1031.g0021.m0001	1.631192049	6.28E-06	0.00016588	DEG
Bpev01.c1610.g0004.m0001	-2.332391454	1.96E-13	3.91E-11	DEG
Bpev01.c0015.g0030.m0001	-1.356317529	3.75E-05	0.00072881	DEG
Bpev01.c0727.g0006.m0001	-4.50595839	3.42E-08	1.95E-06	DEG
Bpev01.c0000.g0106.m0002	1.343633614	0.003125	0.02570459	DEG
Bpev01.c1060.g0031.m0001	2.006681782	4.49E-07	1.78E-05	DEG
Bpev01.c0432.g0002.m0002	1.37796279	0.00050194	0.00604856	DEG
Bpev01.c0689.g0004.m0001	-1.726716989	1.80E-06	5.81E-05	DEG
Bpev01.c0659.g0015.m0001	-2.88506983	1.56E-10	1.70E-08	DEG
Bpev01.c0136.g0003.m0001	-1.589890646	1.08E-05	0.0002626	DEG
Bpev01.c0083.g0021.m0001	2.005007876	5.51E-07	2.13E-05	DEG
Bpev01.c0963.g0018.m0001	2.42501472	3.63E-08	2.05E-06	DEG
Bpev01.c0957.g0014.m0001	1.937195538	0.0048453	0.0358484	DEG
Bpev01.c0841.g0011.m0001	1.306452569	0.00222258	0.01960819	DEG
Bpev01.c0597.g0004.m0001	0.992204748	0.00690012	0.04680044	DEG
Bpev01.c0464.g0035.m0001	2.132703369	1.38E-05	0.00032412	DEG
Bpev01.c0506.g0035.m0001	3.909457279	0.00189713	0.01737158	DEG
Bpev01.c0050.g0046.m0002	-1.184323993	0.00181316	0.01676962	DEG
Bpev01.c0322.g0012.m0001	4.813088717	7.59E-22	7.22E-19	DEG
Bpev01.c1126.g0006.m0001	-1.102826202	0.00154514	0.01470193	DEG
Bpev01.c0365.g0045.m0001	-1.86424199	4.27E-05	0.00081082	DEG
Bpev01.c0000.g0020.m0001	-0.991500949	0.0027891	0.02350147	DEG
Bpev01.c0703.g0001.m0001	1.03625988	0.00554234	0.03961178	DEG
Bpev01.c0205.g0027.m0001	-1.690351526	2.23E-05	0.00047698	DEG
Bpev01.c1854.g0004.m0001	2.521555124	9.18E-06	0.0002299	DEG
Bpev01.c1383.g0004.m0001	1.217700042	0.00192086	0.01755611	DEG
Bpev01.c0439.g0044.m0001	2.755705881	1.50E-06	4.96E-05	DEG

Bpev01.c0671.g0012.m0001	-2.276881203	1.92E-06	6.14E-05	DEG
Bpev01.c2340.g0002.m0001	-6.452073526	0.00500821	0.03666668	DEG
Bpev01.c1288.g0011.m0001	-1.484076464	0.00154829	0.01472054	DEG
Bpev01.c1577.g0015.m0001	-1.367175057	0.0034385	0.02771041	DEG
Bpev01.c0134.g0102.m0001	-0.952620522	0.00435598	0.03301639	DEG
Bpev01.c1135.g0005.m0001	1.283488627	8.60E-05	0.00143263	DEG
Bpev01.c0779.g0001.m0001	1.791823776	0.00106675	0.01085522	DEG
Bpev01.c0224.g0010.m0003	-1.313971019	8.01E-05	0.00135005	DEG
Bpev01.c0141.g0010.m0001	-1.113650209	0.00133798	0.01304696	DEG
Bpev01.c1456.g0008.m0001	-1.568002059	3.96E-07	1.59E-05	DEG
Bpev01.c1948.g0001.m0001	-1.656636576	0.00105106	0.01074007	DEG
Bpev01.c0261.g0071.m0001	0.821060318	0.00611609	0.04255533	DEG
Bpev01.c0170.g0061.m0001	-1.76070489	6.79E-06	0.00017778	DEG
Bpev01.c1271.g0001.m0001	-2.930588606	2.32E-15	7.18E-13	DEG
Bpev01.c0947.g0002.m0001	-1.977730195	0.00261768	0.02236269	DEG
Bpev01.c0274.g0041.m0001	4.190365318	5.50E-11	6.53E-09	DEG
Bpev01.c1161.g0012.m0001	2.004951532	1.43E-08	9.08E-07	DEG
Bpev01.c0145.g0042.m0001	-2.132251826	2.37E-05	0.00049968	DEG
Bpev01.c0565.g0026.m0001	-1.188302836	0.00120138	0.01195657	DEG
Bpev01.c2160.g0006.m0001	-0.944111061	0.00300736	0.02491109	DEG
Bpev01.c0942.g0009.m0001	-1.007834499	0.00168942	0.01582402	DEG
Bpev01.c0129.g0094.m0001	-1.457454944	0.00028838	0.00382767	DEG
Bpev01.c0820.g0008.m0001	-1.943798938	3.66E-09	2.76E-07	DEG
Bpev01.c1445.g0003.m0001	4.778442549	2.31E-06	7.13E-05	DEG
Bpev01.c0134.g0043.m0001	-2.961208297	0.00733513	0.04894954	DEG
Bpev01.c0956.g0005.m0001	1.392524956	0.00030389	0.0039856	DEG
Bpev01.c0044.g0051.m0001	3.27331874	5.30E-05	0.00096841	DEG
Bpev01.c1332.g0003.m0001	-1.392657102	0.00015013	0.00226424	DEG
Bpev01.c0495.g0007.m0001	2.013729233	0.00111043	0.01122211	DEG
Bpev01.c1064.g0005.m0001	-2.198768026	0.0026159	0.02235399	DEG
Bpev01.c0415.g0019.m0001	-1.451197845	0.00045591	0.00559001	DEG
Bpev01.c0215.g0029.m0001	-2.174454344	0.00076715	0.00839087	DEG
Bpev01.c0142.g0076.m0001	-1.822507356	0.00071444	0.00796687	DEG
Bpev01.c0367.g0013.m0001	-2.933023454	0.00056281	0.00658002	DEG
Bpev01.c0422.g0005.m0001	2.189540503	4.59E-11	5.51E-09	DEG
Bpev01.c0800.g0032.m0001	-1.441287665	0.00548787	0.03933707	DEG
Bpev01.c0147.g0015.m0001	-2.034432397	0.00030411	0.00398606	DEG
Bpev01.c1675.g0005.m0001	4.474133559	3.21E-05	0.00064323	DEG
Bpev01.c0346.g0002.m0001	3.227487258	7.02E-05	0.0012116	DEG
Bpev01.c0571.g0007.m0001	1.651792899	0.00023313	0.00321457	DEG
Bpev01.c1071.g0002.m0001	2.60843651	5.85E-08	3.10E-06	DEG
Bpev01.c0042.g0028.m0001	1.994987774	2.56E-08	1.52E-06	DEG
Bpev01.c1477.g0007.m0001	1.765740939	6.99E-06	0.00018245	DEG

Bpev01.c0403.g0017.m0001	-1.906794862	0.00016899	0.00249373	DEG
Bpev01.c2482.g0001.m0001	2.437028328	4.04E-06	0.00011412	DEG
Bpev01.c0274.g0052.m0001	-1.71671931	0.00083167	0.00896832	DEG
Bpev01.c0018.g0045.m0001	-3.354950367	9.46E-17	3.76E-14	DEG
Bpev01.c0241.g0002.m0001	3.298343291	4.36E-10	4.27E-08	DEG
Bpev01.c1350.g0009.m0001	-2.148151874	5.09E-11	6.06E-09	DEG
Bpev01.c0993.g0007.m0001	1.434069034	0.00752398	0.04986457	DEG
Bpev01.c0134.g0065.m0001	-0.972739096	0.00592923	0.0415894	DEG
Bpev01.c0050.g0029.m0001	2.766647929	7.00E-14	1.50E-11	DEG
Bpev01.c2571.g0004.m0001	3.613752947	6.04E-05	0.00107668	DEG
Bpev01.c0565.g0034.m0001	-2.206471901	0.00168426	0.01578699	DEG
Bpev01.c1153.g0009.m0001	-1.28659476	0.00140466	0.01356687	DEG
Bpev01.c0918.g0018.m0001	-1.147287292	0.00524782	0.03803315	DEG
Bpev01.c0893.g0006.m0001	-1.619385799	9.36E-06	0.00023358	DEG
Bpev01.c1004.g0008.m0001	1.240343437	0.00033235	0.00428751	DEG
Bpev01.c0305.g0003.m0001	1.10295793	0.00050583	0.00608549	DEG
Bpev01.c0555.g0007.m0001	-1.206084642	0.00016819	0.00248679	DEG
Bpev01.c0579.g0028.m0001	-4.01438336	1.24E-09	1.08E-07	DEG
Bpev01.c0443.g0020.m0001	-1.686249964	3.90E-08	2.18E-06	DEG
Bpev01.c0571.g0004.m0001	-1.026272951	0.00114723	0.0115307	DEG
Bpev01.c1051.g0006.m0001	2.849045722	1.14E-06	3.98E-05	DEG
Bpev01.c0283.g0009.m0001	-1.596935864	0.00139534	0.01348895	DEG
Bpev01.c0029.g0102.m0001	-1.123941227	0.00419909	0.03211669	DEG
Bpev01.c0104.g0011.m0001	2.121762484	5.77E-05	0.00103632	DEG
Bpev01.c0960.g0003.m0001	3.534986554	2.37E-07	1.02E-05	DEG
Bpev01.c0797.g0016.m0001	1.753734646	0.00016083	0.00239494	DEG
Bpev01.c0096.g0030.m0001	4.399392472	1.13E-05	0.00027283	DEG
Bpev01.c1534.g0002.m0001	4.115362392	7.18E-12	1.01E-09	DEG
Bpev01.c0170.g0039.m0001	0.966703449	0.00423697	0.03232239	DEG
Bpev01.c0089.g0082.m0001	-1.465579016	1.84E-05	0.00040881	DEG
Bpev01.c1400.g0001.m0001	-1.365205959	7.20E-06	0.00018712	DEG
Bpev01.c0261.g0014.m0001	-1.404766359	0.00299731	0.02484868	DEG
Bpev01.c1803.g0003.m0001	2.828416156	3.96E-07	1.59E-05	DEG
Bpev01.c1207.g0002.m0001	-1.155412664	0.00107343	0.01091187	DEG
Bpev01.c1414.g0008.m0001	-2.665493637	1.40E-06	4.73E-05	DEG
Bpev01.c0713.g0004.m0001	-0.969050941	0.00555046	0.03963752	DEG
Bpev01.c0080.g0077.m0001	-0.951191134	0.0039161	0.03056363	DEG
Bpev01.c1028.g0001.m0002	-2.332642157	0.00672721	0.04584312	DEG
Bpev01.c0052.g0091.m0001	1.374476729	0.00562473	0.03996773	DEG
Bpev01.c2436.g0003.m0001	0.889882084	0.0054736	0.03927304	DEG
Bpev01.c0088.g0117.m0001	-2.060700887	0.00153119	0.01459263	DEG
Bpev01.c0052.g0060.m0001	-2.416292611	4.87E-07	1.90E-05	DEG
Bpev01.c0088.g0020.m0001	-1.063872575	0.00095465	0.0099801	DEG

Bpev01.c1179.g0005.m0001	1.580536976	0.00179266	0.01661132	DEG
Bpev01.c0525.g0007.m0001	-1.113376261	0.00060867	0.00700765	DEG
Bpev01.c0264.g0023.m0001	0.979573606	0.00329167	0.02677633	DEG
Bpev01.c1422.g0009.m0001	-1.189028092	0.00059201	0.00684535	DEG
Bpev01.c1184.g0002.m0001	-2.148420889	7.20E-06	0.00018712	DEG
Bpev01.c0292.g0023.m0001	-1.539799373	0.00072353	0.00802912	DEG
Bpev01.c1791.g0005.m0001	1.804186455	0.00045772	0.00560047	DEG
Bpev01.c0090.g0017.m0002	-1.037856422	0.00668256	0.04566572	DEG
Bpev01.c1874.g0014.m0001	-1.81269833	0.00069545	0.00778455	DEG
Bpev01.c0063.g0018.m0001	1.214949043	0.00011876	0.00187162	DEG
Bpev01.c1456.g0005.m0001	2.059421834	2.11E-06	6.60E-05	DEG
Bpev01.c2129.g0001.m0001	-1.492406342	6.87E-05	0.00119135	DEG
Bpev01.c0089.g0087.m0001	4.065621727	5.72E-10	5.39E-08	DEG
Bpev01.c0557.g0032.m0001	-2.016742193	7.34E-09	5.16E-07	DEG
Bpev01.c0772.g0011.m0001	0.975676762	0.00212916	0.01894815	DEG
Bpev01.c0597.g0035.m0001	-1.829570184	5.26E-05	0.00096402	DEG
Bpev01.c2233.g0004.m0001	-0.920412059	0.00599565	0.04195585	DEG
Bpev01.c0280.g0055.m0001	1.433593339	0.00030874	0.00403418	DEG
Bpev01.c0989.g0007.m0001	0.813819268	0.0067015	0.04572088	DEG
Bpev01.c0096.g0016.m0001	0.92273187	0.00736729	0.04905803	DEG
Bpev01.c0261.g0037.m0001	-1.759747216	4.05E-07	1.63E-05	DEG
Bpev01.c3057.g0001.m0001	-1.033136113	0.00298943	0.02479532	DEG
Bpev01.c1241.g0006.m0001	-1.448377419	8.90E-06	0.00022467	DEG
Bpev01.c0164.g0003.m0001	1.751037905	3.80E-07	1.53E-05	DEG
Bpev01.c0717.g0023.m0001	-3.01449566	2.40E-18	1.34E-15	DEG
Bpev01.c0518.g0030.m0001	-0.828524031	0.00601523	0.04203414	DEG
Bpev01.c0874.g0004.m0001	-1.725422642	0.00450876	0.03389512	DEG
Bpev01.c0115.g0053.m0001	2.513949594	1.01E-05	0.00024867	DEG
Bpev01.c1034.g0004.m0001	-1.403326853	3.49E-05	0.00069021	DEG
Bpev01.c0165.g0005.m0001	2.188118653	1.63E-08	1.01E-06	DEG
Bpev01.c0031.g0008.m0001	2.024058705	1.76E-05	0.0003937	DEG
Bpev01.c0161.g0043.m0001	-1.066952627	0.00062219	0.00713539	DEG
Bpev01.c0245.g0046.m0001	-1.915584666	3.27E-09	2.52E-07	DEG
Bpev01.c0000.g0102.m0001	-1.316822344	0.00069036	0.00774228	DEG
Bpev01.c0029.g0055.m0001	-1.550033936	7.42E-06	0.00019193	DEG
Bpev01.c1288.g0008.m0001	-6.57178928	0.00222207	0.01960819	DEG
Bpev01.c0029.g0063.m0001	-0.940203488	0.00207374	0.01856717	DEG
Bpev01.c0095.g0043.m0001	-2.347319853	0.00067447	0.00761629	DEG
Bpev01.c1040.g0010.m0001	0.88642777	0.00462167	0.03458498	DEG
Bpev01.c0082.g0016.m0001	-2.037771802	3.99E-08	2.23E-06	DEG
Bpev01.c1240.g0001.m0001	-1.144097052	0.00040328	0.00504555	DEG
Bpev01.c0036.g0009.m0001	1.146974439	0.00014308	0.00218237	DEG
Bpev01.c0464.g0003.m0001	-1.840458312	0.00027775	0.00371336	DEG

Bpev01.c0171.g0006.m0001	1.50822623	5.82E-06	0.00015579	DEG
Bpev01.c0016.g0107.m0001	1.757865889	5.44E-06	0.00014684	DEG
Bpev01.c1897.g0014.m0001	4.351549928	6.69E-05	0.00116899	DEG
Bpev01.c0027.g0083.m0001	-1.184417638	0.00087001	0.00931349	DEG
Bpev01.c0050.g0069.m0001	0.917291732	0.00589438	0.04143378	DEG
Bpev01.c2264.g0001.m0001	2.741698352	0.00073906	0.00815787	DEG
Bpev01.c0210.g0026.m0001	2.551299207	5.13E-06	0.00014043	DEG
Bpev01.c1349.g0009.m0001	0.908393152	0.00309941	0.02554409	DEG
Bpev01.c1653.g0009.m0001	1.995186098	2.55E-08	1.51E-06	DEG
Bpev01.c0439.g0038.m0001	-1.652916001	2.16E-06	6.73E-05	DEG
Bpev01.c1764.g0008.m0001	-1.04182699	0.00090244	0.00954961	DEG
Bpev01.c0111.g0002.m0001	4.453565323	6.04E-05	0.00107668	DEG
Bpev01.c0137.g0021.m0001	-1.481092838	1.28E-05	0.00030425	DEG
Bpev01.c0025.g0036.m0001	1.675173657	0.00011765	0.00185809	DEG
Bpev01.c0261.g0053.m0001	1.323649349	0.00053774	0.00637049	DEG
Bpev01.c0134.g0038.m0001	1.4618955	0.00016046	0.00239186	DEG
Bpev01.c0280.g0019.m0001	-1.662721057	0.0066911	0.04568149	DEG
Bpev01.c1072.g0009.m0001	-1.04961503	0.00224748	0.01978575	DEG
Bpev01.c1092.g0013.m0002	1.433593847	0.001855	0.01709105	DEG
Bpev01.c1518.g0002.m0001	-1.103230769	0.00106198	0.01082647	DEG
Bpev01.c0094.g0054.m0001	1.35493363	3.70E-05	0.00072277	DEG
Bpev01.c0027.g0057.m0001	1.272732526	9.69E-05	0.00157548	DEG
Bpev01.c0162.g0064.m0001	-1.21492286	0.00033209	0.00428696	DEG
Bpev01.c0440.g0007.m0001	2.034845026	0.00162142	0.015299	DEG
Bpev01.c0485.g0027.m0001	1.853667274	8.44E-08	4.23E-06	DEG
Bpev01.c1084.g0007.m0001	2.564386786	1.81E-05	0.00040428	DEG
Bpev01.c0718.g0008.m0001	-1.426789139	0.00041938	0.00522696	DEG
Bpev01.c1202.g0014.m0001	-2.067566711	1.28E-10	1.41E-08	DEG
Bpev01.c3433.g0001.m0001	2.918337011	2.28E-05	0.00048377	DEG
Bpev01.c0356.g0034.m0001	1.012030001	0.00318796	0.0261131	DEG
Bpev01.c0010.g0002.m0001	1.878911394	3.49E-08	1.98E-06	DEG
Bpev01.c2282.g0005.m0002	1.52793938	0.00053901	0.00638033	DEG
Bpev01.c0467.g0001.m0001	-1.5635852	6.09E-05	0.00108126	DEG
Bpev01.c1034.g0001.m0001	2.069616359	2.36E-05	0.00049863	DEG
Bpev01.c0626.g0011.m0001	1.315720011	0.00445214	0.03357605	DEG
Bpev01.c1047.g0004.m0001	-2.314808249	1.48E-07	6.85E-06	DEG
Bpev01.c1563.g0006.m0001	2.655220408	1.59E-11	2.06E-09	DEG
Bpev01.c2384.g0001.m0001	1.135128108	0.00695734	0.04705193	DEG
Bpev01.c1116.g0008.m0001	-1.31291949	0.00020049	0.00284809	DEG
Bpev01.c0169.g0054.m0001	-1.741693149	0.00036556	0.00465277	DEG
Bpev01.c0518.g0006.m0001	-0.864217095	0.00527017	0.03814815	DEG
Bpev01.c3303.g0001.m0001	-1.631157618	2.76E-05	0.00056886	DEG
Bpev01.c0240.g0032.m0001	-0.879302636	0.00416582	0.03194963	DEG

Bpev01.c0343.g0011.m0001	-1.210188298	0.00062286	0.00714013	DEG
Bpev01.c0037.g0002.m0001	-1.433132601	0.00275683	0.02328789	DEG
Bpev01.c0473.g0018.m0001	-1.223199563	0.00130055	0.01277082	DEG
Bpev01.c0547.g0018.m0001	2.182580974	7.85E-05	0.00132952	DEG
Bpev01.c2235.g0008.m0001	-0.907035823	0.00493948	0.03631828	DEG
Bpev01.c0299.g0023.m0001	-2.736369148	1.71E-14	4.33E-12	DEG
Bpev01.c0154.g0005.m0002	2.10384608	0.00148517	0.01423241	DEG
Bpev01.c0313.g0016.m0001	0.926974181	0.00339654	0.02744733	DEG
Bpev01.c0159.g0039.m0001	-2.706036721	0.0007241	0.00802991	DEG
Bpev01.c0142.g0095.m0001	1.053959856	0.00197172	0.01788217	DEG
Bpev01.c0740.g0001.m0001	2.521423711	6.25E-10	5.86E-08	DEG
Bpev01.c0240.g0002.m0001	-4.028804811	0.00075537	0.00829746	DEG
Bpev01.c0129.g0088.m0001	1.24921507	9.41E-05	0.0015435	DEG
Bpev01.c0564.g0017.m0001	-1.806472799	4.98E-05	0.00092325	DEG
Bpev01.c0415.g0039.m0001	1.273389612	0.00575259	0.04061161	DEG
Bpev01.c0469.g0006.m0001	-0.929000201	0.00433477	0.03290645	DEG
Bpev01.c1419.g0009.m0001	-0.845596177	0.00754805	0.04997911	DEG
Bpev01.c0145.g0018.m0001	1.082365082	0.00548728	0.03933707	DEG
Bpev01.c0818.g0013.m0001	-2.266186514	0.00029413	0.00388816	DEG
Bpev01.c0159.g0009.m0001	1.711981928	9.26E-06	0.00023148	DEG
Bpev01.c0029.g0040.m0001	-1.766745317	0.00071053	0.00793221	DEG
Bpev01.c0774.g0017.m0003	3.498870023	2.49E-15	7.55E-13	DEG
Bpev01.c2900.g0001.m0001	1.64812721	4.34E-06	0.0001212	DEG
Bpev01.c0162.g0012.m0001	6.49972355	9.97E-26	1.55E-22	DEG
Bpev01.c0806.g0008.m0001	3.282710761	0.00176115	0.01637279	DEG
Bpev01.c2379.g0004.m0001	2.513261053	0.00058858	0.00682437	DEG
Bpev01.c0115.g0036.m0001	-2.143109783	1.82E-08	1.11E-06	DEG
Bpev01.c0536.g0015.m0001	3.564850858	7.57E-07	2.80E-05	DEG
Bpev01.c0042.g0040.m0001	-0.909143166	0.00528318	0.03819539	DEG
Bpev01.c0762.g0008.m0001	-1.250831998	0.00033216	0.00428696	DEG
Bpev01.c0022.g0112.m0001	-2.347732825	0.00018334	0.0026601	DEG
Bpev01.c0023.g0014.m0001	-2.744152396	3.42E-07	1.40E-05	DEG
Bpev01.c1606.g0002.m0001	2.744496533	4.68E-06	0.00012936	DEG
Bpev01.c0261.g0013.m0001	-2.668054117	1.63E-13	3.30E-11	DEG
Bpev01.c1577.g0010.m0001	-2.669517086	0.0004302	0.00533478	DEG
Bpev01.c0118.g0006.m0001	1.722958212	3.81E-06	0.00010927	DEG
Bpev01.c0000.g0156.m0001	3.41219024	4.08E-12	6.04E-10	DEG
Bpev01.c0000.g0215.m0001	-2.967550563	0.00219967	0.01945793	DEG
Bpev01.c0261.g0040.m0001	-1.425852643	6.23E-06	0.00016497	DEG
Bpev01.c0189.g0060.m0001	-2.183934683	0.00023625	0.0032484	DEG
Bpev01.c1989.g0006.m0001	-1.404298412	0.00057471	0.00668729	DEG
Bpev01.c0398.g0003.m0001	3.094622261	0.00256504	0.0219832	DEG
Bpev01.c0645.g0006.m0001	1.171326312	0.00078333	0.00853142	DEG

Bpev01.c1179.g0001.m0001	1.085335099	0.00091118	0.00962135	DEG
Bpev01.c0261.g0049.m0001	2.555505434	0.0009265	0.0097411	DEG
Bpev01.c0796.g0003.m0001	2.262973966	8.65E-09	5.96E-07	DEG
Bpev01.c0606.g0013.m0001	-1.139720176	0.00102796	0.01054845	DEG
Bpev01.c0615.g0010.m0002	-2.858176043	7.05E-15	1.90E-12	DEG
Bpev01.c0089.g0075.m0001	-1.566745911	7.66E-06	0.00019703	DEG
Bpev01.c0389.g0018.m0002	-1.335496521	0.00487436	0.03600452	DEG
Bpev01.c0794.g0013.m0001	-1.025852327	0.00228152	0.02001962	DEG
Bpev01.c0094.g0086.m0001	-1.127105024	0.00182832	0.01688325	DEG
Bpev01.c0080.g0029.m0001	-1.324056816	0.00176331	0.01638054	DEG
Bpev01.c0020.g0003.m0001	3.177505407	8.24E-06	0.00021062	DEG
Bpev01.c0080.g0006.m0001	-0.932164496	0.00546663	0.03925328	DEG
Bpev01.c0581.g0013.m0002	-1.121211794	0.00045822	0.00560428	DEG
Bpev01.c0018.g0005.m0001	2.677444188	5.76E-06	0.00015434	DEG
Bpev01.c0329.g0017.m0001	1.02444002	0.00176565	0.01639194	DEG
Bpev01.c0624.g0017.m0001	2.456564818	3.66E-06	0.00010607	DEG
Bpev01.c0473.g0039.m0001	-3.115862884	9.77E-06	0.00024178	DEG
Bpev01.c0365.g0041.m0001	1.427562378	0.00102815	0.01054845	DEG
Bpev01.c2060.g0002.m0001	5.102233087	1.39E-06	4.70E-05	DEG
Bpev01.c1063.g0008.m0001	-1.859700341	3.10E-05	0.00062612	DEG
Bpev01.c0682.g0010.m0001	-1.487335345	3.56E-05	0.00069873	DEG
Bpev01.c0101.g0054.m0001	-1.845448327	0.00022856	0.00316635	DEG
Bpev01.c0116.g0005.m0001	-2.99431775	2.75E-15	8.19E-13	DEG
Bpev01.c0000.g0035.m0001	1.897920322	0.00399663	0.03103578	DEG
Bpev01.c0029.g0051.m0001	-1.816238691	0.0035461	0.02837551	DEG
Bpev01.c1588.g0010.m0002	1.444867076	6.01E-06	0.00015995	DEG
Bpev01.c0141.g0022.m0001	-2.297570026	0.00015528	0.00232998	DEG
Bpev01.c1195.g0006.m0001	1.134679936	0.00047431	0.00577232	DEG
Bpev01.c1012.g0005.m0001	1.552009687	0.00124848	0.01233765	DEG
Bpev01.c0906.g0020.m0001	3.683634295	3.77E-13	7.02E-11	DEG
Bpev01.c0169.g0039.m0001	-0.953627101	0.00205367	0.0184211	DEG
Bpev01.c0038.g0113.m0001	1.870231317	1.41E-06	4.73E-05	DEG
Bpev01.c0027.g0052.m0002	1.016867253	0.00295094	0.02455485	DEG
Bpev01.c0454.g0030.m0001	-1.365914598	3.90E-05	0.00075315	DEG
Bpev01.c0901.g0004.m0001	-2.581571327	1.02E-06	3.63E-05	DEG
Bpev01.c0470.g0049.m0001	-2.674691371	0.00017553	0.0025696	DEG
Bpev01.c1936.g0015.m0001	-2.133173003	0.00246934	0.02131816	DEG
Bpev01.c0889.g0017.m0001	-1.695263792	0.0001015	0.00163374	DEG
Bpev01.c0172.g0001.m0005	0.903532806	0.00661933	0.04538111	DEG
Bpev01.c1307.g0001.m0001	1.855898403	3.77E-06	0.00010817	DEG
Bpev01.c0817.g0011.m0001	1.563531264	0.00031041	0.00405238	DEG
Bpev01.c0746.g0011.m0001	1.692609544	0.00082493	0.00890215	DEG
Bpev01.c0667.g0002.m0001	-2.315106634	4.33E-06	0.00012092	DEG

Bpev01.c0452.g0008.m0001	1.068301461	0.00571014	0.04037004	DEG
Bpev01.c1972.g0008.m0001	2.005683558	3.35E-09	2.56E-07	DEG
Bpev01.c0491.g0001.m0001	4.905145575	0.00039559	0.00497472	DEG
Bpev01.c0828.g0020.m0001	1.75671749	2.47E-07	1.06E-05	DEG
Bpev01.c0161.g0082.m0001	1.471214372	3.88E-05	0.00074998	DEG
Bpev01.c0557.g0027.m0001	-1.4319547	0.0012083	0.01200508	DEG
Bpev01.c1167.g0017.m0001	-1.183951993	0.00074631	0.00821122	DEG
Bpev01.c2673.g0002.m0001	-2.354419949	5.31E-05	0.00097034	DEG
Bpev01.c1782.g0008.m0001	-1.998381128	0.00371722	0.02937755	DEG
Bpev01.c0404.g0024.m0001	1.786290211	0.00028434	0.00378418	DEG
Bpev01.c1608.g0003.m0001	-1.001328788	0.00492012	0.03624037	DEG
Bpev01.c1985.g0009.m0001	-2.481536696	0.00035371	0.00451863	DEG
Bpev01.c1649.g0017.m0001	1.438528519	5.42E-06	0.00014651	DEG
Bpev01.c1260.g0007.m0001	-1.851108837	0.00088957	0.00947129	DEG
Bpev01.c0275.g0039.m0001	1.462438809	0.00030066	0.00395494	DEG
Bpev01.c1115.g0015.m0001	-1.169270711	0.00093557	0.009826	DEG
Bpev01.c3283.g0003.m0001	1.74923534	0.00569545	0.04035339	DEG
Bpev01.c1592.g0005.m0001	1.247670233	0.00039177	0.00494538	DEG
Bpev01.c0210.g0012.m0001	-2.3574587	0.00064109	0.00730618	DEG
Bpev01.c1849.g0004.m0001	1.166780515	0.00600084	0.04197168	DEG
Bpev01.c0893.g0016.m0001	1.616390417	5.85E-05	0.00105098	DEG
Bpev01.c0245.g0049.m0001	0.95086578	0.00413698	0.03180943	DEG
Bpev01.c0052.g0184.m0001	1.081208975	0.00110614	0.01118637	DEG
Bpev01.c0022.g0043.m0001	1.886112887	2.31E-05	0.00048855	DEG
Bpev01.c0349.g0041.m0001	-2.772173912	1.40E-10	1.53E-08	DEG
Bpev01.c0782.g0015.m0001	2.214071914	0.00241239	0.02091206	DEG
Bpev01.c0286.g0026.m0001	-1.494788804	2.93E-06	8.78E-05	DEG
Bpev01.c3685.g0001.m0001	2.887560635	1.87E-05	0.00041378	DEG
Bpev01.c0517.g0019.m0001	0.985828966	0.00395579	0.03078364	DEG
Bpev01.c0713.g0014.m0001	-1.191727782	0.0002273	0.00315145	DEG
Bpev01.c0444.g0032.m0001	0.99857592	0.00225566	0.01983999	DEG
Bpev01.c0467.g0007.m0001	-3.051636998	0.00435116	0.03298833	DEG
Bpev01.c0415.g0019.m0002	-1.494584214	3.72E-05	0.00072498	DEG
Bpev01.c0091.g0035.m0001	-1.695840232	0.00012618	0.00197375	DEG
Bpev01.c0082.g0032.m0001	1.292726783	0.00012406	0.00194673	DEG
Bpev01.c0134.g0111.m0001	0.978806052	0.00377227	0.02971687	DEG
Bpev01.c1345.g0004.m0001	-2.433986459	0.00017403	0.00255275	DEG
Bpev01.c0396.g0012.m0001	-0.985966251	0.00438431	0.0331892	DEG
Bpev01.c0335.g0004.m0001	-2.884876578	3.38E-07	1.39E-05	DEG
Bpev01.c1146.g0001.m0001	-0.991334826	0.00209196	0.01871326	DEG
Bpev01.c0115.g0096.m0001	-1.749184914	5.93E-05	0.00106167	DEG
Bpev01.c0015.g0184.m0001	-1.785128739	0.00193098	0.01759937	DEG
Bpev01.c1805.g0001.m0001	-1.17881472	0.00012841	0.00199889	DEG

Bpev01.c1229.g0006.m0001	-1.150892188	0.00510633	0.03721312	DEG
Bpev01.c0411.g0010.m0001	-0.952840611	0.00442467	0.03344236	DEG
Bpev01.c0062.g0004.m0001	1.71144162	2.97E-06	8.85E-05	DEG
Bpev01.c0157.g0049.m0001	2.253945127	1.12E-06	3.92E-05	DEG
Bpev01.c0821.g0008.m0001	-3.3522748	0.00273498	0.02315258	DEG
Bpev01.c0120.g0037.m0001	-1.45229721	0.00012954	0.00201344	DEG
Bpev01.c1371.g0015.m0001	-0.871120365	0.00573267	0.04049043	DEG
Bpev01.c1159.g0018.m0001	1.656189594	0.00066006	0.00748233	DEG
Bpev01.c0651.g0010.m0001	0.837785738	0.00735552	0.04904622	DEG
Bpev01.c2218.g0004.m0001	2.267371852	2.08E-09	1.69E-07	DEG
Bpev01.c0979.g0001.m0001	-1.133707158	0.00028137	0.00375832	DEG
Bpev01.c0052.g0107.m0001	1.18966677	0.00176449	0.01638642	DEG
Bpev01.c0404.g0022.m0001	2.417897283	1.25E-08	8.17E-07	DEG
Bpev01.c1354.g0001.m0001	2.217799118	0.00103487	0.01060044	DEG
Bpev01.c0878.g0014.m0001	2.424471204	6.94E-08	3.56E-06	DEG
Bpev01.c1936.g0005.m0001	-3.248103989	3.73E-05	0.00072616	DEG
Bpev01.c1366.g0001.m0001	2.271385674	0.00031303	0.0040794	DEG
Bpev01.c0575.g0014.m0001	-1.233384086	0.00013507	0.00208619	DEG
Bpev01.c0158.g0051.m0001	-1.420113268	0.00642118	0.0443036	DEG
Bpev01.c0162.g0031.m0001	-1.746158981	0.00625618	0.04336607	DEG
Bpev01.c0027.g0012.m0001	1.22653753	0.00023338	0.003215	DEG
Bpev01.c1034.g0012.m0001	-6.504318537	0.00495921	0.0364439	DEG
Bpev01.c0022.g0050.m0001	0.905621494	0.00691179	0.04684077	DEG
Bpev01.c0001.g0071.m0001	0.876964172	0.00495151	0.03639641	DEG
Bpev01.c0051.g0086.m0001	1.610669213	3.43E-05	0.00068012	DEG
Bpev01.c0564.g0002.m0001	1.183739329	0.00016827	0.00248679	DEG
Bpev01.c0018.g0088.m0001	-1.205960889	0.00012292	0.0019309	DEG
Bpev01.c1610.g0008.m0001	3.125112916	2.24E-07	9.70E-06	DEG
Bpev01.c0126.g0032.m0003	2.241267178	8.05E-06	0.00020591	DEG
Bpev01.c4793.g0001.m0001	2.588354338	0.00066311	0.00750939	DEG
Bpev01.c1463.g0001.m0001	-1.717918907	0.00635806	0.04394809	DEG
Bpev01.c1159.g0021.m0001	-2.614792292	4.73E-11	5.66E-09	DEG
Bpev01.c0335.g0025.m0001	-1.315213024	0.00234823	0.02046461	DEG
Bpev01.c1115.g0006.m0001	-2.10019866	1.16E-05	0.00027769	DEG
Bpev01.c0029.g0008.m0001	-1.47453016	2.49E-06	7.60E-05	DEG
Bpev01.c1335.g0009.m0001	-1.604018537	5.04E-06	0.00013814	DEG
Bpev01.c1217.g0003.m0001	-1.037104273	0.00093871	0.0098484	DEG
Bpev01.c0051.g0199.m0001	-1.107047544	0.0068073	0.04629244	DEG
Bpev01.c0094.g0057.m0001	1.18385766	0.00570654	0.04037004	DEG
Bpev01.c0276.g0026.m0001	-3.303413781	8.86E-07	3.23E-05	DEG
Bpev01.c1918.g0005.m0001	1.501335429	1.37E-05	0.00032296	DEG
Bpev01.c1228.g0006.m0001	3.148246369	8.88E-06	0.00022449	DEG
Bpev01.c1423.g0006.m0001	-1.372066581	0.00225969	0.01986946	DEG

Bpev01.c0727.g0002.m0001	0.965741569	0.00199836	0.01804019	DEG
Bpev01.c0532.g0009.m0001	0.913367797	0.00600458	0.04198784	DEG
Bpev01.c0063.g0012.m0001	0.945044228	0.00544683	0.03913819	DEG
Bpev01.c1119.g0009.m0001	-1.920010843	0.00213141	0.01896236	DEG
Bpev01.c1229.g0010.m0001	1.898717539	7.51E-09	5.26E-07	DEG
Bpev01.c1591.g0001.m0001	4.631604748	2.95E-14	6.96E-12	DEG
Bpev01.c0154.g0041.m0001	1.735844651	4.72E-06	0.00012981	DEG
Bpev01.c0995.g0004.m0001	-1.326596164	0.00210177	0.01877371	DEG
Bpev01.c1150.g0004.m0001	1.186026065	0.00017511	0.00256599	DEG
Bpev01.c0161.g0102.m0001	-2.202098406	2.60E-07	1.10E-05	DEG
Bpev01.c0906.g0021.m0001	2.748132549	1.22E-08	7.97E-07	DEG
Bpev01.c0163.g0042.m0001	-0.999201968	0.00163178	0.01538696	DEG
Bpev01.c0473.g0019.m0001	0.865535256	0.00528054	0.03819507	DEG
Bpev01.c1764.g0010.m0001	-1.978305524	7.37E-10	6.80E-08	DEG
Bpev01.c0088.g0037.m0001	-2.726904633	2.69E-09	2.11E-07	DEG
Bpev01.c2197.g0002.m0001	-1.181204199	0.00023199	0.00320335	DEG
Bpev01.c0357.g0045.m0001	1.729825548	5.53E-06	0.00014921	DEG
Bpev01.c0230.g0017.m0001	0.899022012	0.00484658	0.03584887	DEG
Bpev01.c1178.g0006.m0001	4.089179698	2.78E-05	0.00057293	DEG
Bpev01.c1034.g0006.m0001	-2.595239552	0.00658261	0.0451609	DEG
Bpev01.c1045.g0004.m0001	2.105240477	4.73E-05	0.00088465	DEG
Bpev01.c0679.g0012.m0001	-1.762224685	0.00181828	0.01681171	DEG
Bpev01.c0536.g0012.m0001	-1.457687943	0.00027054	0.00363336	DEG
Bpev01.c1594.g0005.m0001	6.759576507	0.00209166	0.01871326	DEG
Bpev01.c0052.g0206.m0002	-1.901857031	6.91E-06	0.00018067	DEG
Bpev01.c3036.g0002.m0001	3.230506662	1.40E-09	1.19E-07	DEG
Bpev01.c0115.g0027.m0001	-1.030777365	0.00114142	0.01149584	DEG
Bpev01.c0420.g0016.m0001	1.146326873	0.00094664	0.00991754	DEG
Bpev01.c0080.g0016.m0001	3.953391432	0.00029614	0.00390942	DEG
Bpev01.c1869.g0005.m0001	3.028999133	5.82E-10	5.47E-08	DEG
Bpev01.c0349.g0008.m0001	-2.19096904	2.38E-10	2.51E-08	DEG
Bpev01.c0579.g0012.m0001	0.94265014	0.00198312	0.01795237	DEG
Bpev01.c0578.g0002.m0001	-1.105349225	0.00640881	0.0442573	DEG
Bpev01.c1334.g0012.m0001	-1.079272071	0.00130295	0.01279008	DEG
Bpev01.c1647.g0001.m0001	4.623897914	4.70E-06	0.00012956	DEG
Bpev01.c1018.g0006.m0001	1.548090039	1.10E-05	0.00026761	DEG
Bpev01.c1517.g0001.m0001	1.746036144	0.00089398	0.00949482	DEG
Bpev01.c0279.g0015.m0001	-1.049821426	0.00076731	0.00839087	DEG
Bpev01.c1086.g0008.m0001	1.269896748	8.43E-05	0.00140983	DEG
Bpev01.c0480.g0081.m0001	2.10476097	1.13E-08	7.50E-07	DEG
Bpev01.c0469.g0009.m0001	-2.19548231	1.36E-08	8.78E-07	DEG
Bpev01.c0461.g0002.m0001	2.249514808	4.65E-12	6.77E-10	DEG
Bpev01.c0404.g0021.m0001	0.931604275	0.00474927	0.03532379	DEG

Bpev01.c1112.g0014.m0001	-2.337361807	0.00558426	0.03975345	DEG
Bpev01.c1945.g0001.m0001	-2.276928693	0.00115015	0.01155216	DEG
Bpev01.c0872.g0002.m0001	2.411166654	0.0005583	0.00654293	DEG
Bpev01.c0771.g0016.m0001	3.134210074	1.10E-06	3.89E-05	DEG
Bpev01.c0214.g0067.m0001	2.060774756	0.00526122	0.03810212	DEG
Bpev01.c0565.g0006.m0001	-2.444157277	0.00648119	0.04463152	DEG
Bpev01.c0900.g0002.m0001	2.531893542	3.50E-09	2.65E-07	DEG
Bpev01.c1475.g0006.m0001	-2.404567544	3.23E-09	2.49E-07	DEG
Bpev01.c0000.g0103.m0001	-1.849966653	5.08E-07	1.98E-05	DEG
Bpev01.c1108.g0001.m0001	-1.115417081	0.00199779	0.01804019	DEG
Bpev01.c0095.g0013.m0001	2.495104426	1.14E-10	1.28E-08	DEG
Bpev01.c0274.g0001.m0001	4.178912975	1.81E-08	1.11E-06	DEG
Bpev01.c0553.g0007.m0001	1.987905955	0.00023997	0.00329507	DEG
Bpev01.c0158.g0067.m0001	1.748460755	2.00E-05	0.00043605	DEG
Bpev01.c0340.g0036.m0001	1.649951058	8.93E-05	0.00147768	DEG
Bpev01.c0404.g0023.m0001	-1.322491183	4.83E-05	0.00090185	DEG
Bpev01.c1189.g0007.m0001	-1.507845616	3.04E-06	9.04E-05	DEG
Bpev01.c0031.g0026.m0001	0.97937305	0.00112994	0.01139966	DEG
Bpev01.c1588.g0004.m0001	-2.228823863	0.00312315	0.02570376	DEG
Bpev01.c0522.g0041.m0001	-1.282126221	0.00199232	0.0180024	DEG
Bpev01.c0038.g0016.m0001	1.742169624	1.00E-05	0.00024629	DEG
Bpev01.c0118.g0010.m0001	3.591451535	3.82E-08	2.14E-06	DEG
Bpev01.c1949.g0001.m0001	2.547541963	8.58E-08	4.27E-06	DEG
Bpev01.c0146.g0019.m0001	1.276921576	0.00090928	0.00960535	DEG
Bpev01.c0778.g0023.m0001	-1.261648702	0.00372309	0.02940022	DEG
Bpev01.c0162.g0060.m0001	-1.118045725	0.00029534	0.00390064	DEG
Bpev01.c1229.g0001.m0001	3.553354637	1.67E-09	1.41E-07	DEG
Bpev01.c0147.g0019.m0001	-1.782632804	0.00011333	0.00180141	DEG
Bpev01.c2167.g0006.m0002	2.077379529	0.00725848	0.04860836	DEG
Bpev01.c2242.g0005.m0001	3.032775501	0.00053733	0.00636815	DEG
Bpev01.c0122.g0011.m0001	3.105158906	2.52E-20	2.06E-17	DEG
Bpev01.c0984.g0002.m0001	1.212696091	0.00013168	0.00204354	DEG
Bpev01.c0129.g0003.m0003	1.693218762	0.00372192	0.02939889	DEG
Bpev01.c0340.g0010.m0001	-2.549110877	8.64E-10	7.90E-08	DEG
Bpev01.c0044.g0036.m0001	-1.531216305	4.16E-05	0.00079493	DEG
Bpev01.c0088.g0053.m0001	-1.709190134	1.17E-07	5.54E-06	DEG
Bpev01.c0088.g0087.m0002	-0.869319795	0.0058943	0.04143378	DEG
Bpev01.c0038.g0147.m0001	-2.411322922	0.00024821	0.00337979	DEG
Bpev01.c2165.g0006.m0002	1.248667206	0.00234781	0.02046461	DEG
Bpev01.c1335.g0003.m0001	2.966580584	1.49E-15	4.92E-13	DEG
Bpev01.c0518.g0010.m0001	-1.01677642	0.00227155	0.01994992	DEG
Bpev01.c2080.g0005.m0001	2.891583625	7.64E-11	8.85E-09	DEG
Bpev01.c0584.g0005.m0001	4.195773794	8.02E-07	2.96E-05	DEG

Bpev01.c0874.g0006.m0001	-1.986905769	0.00018005	0.00261883	DEG
Bpev01.c0811.g0005.m0001	2.337403961	2.87E-06	8.61E-05	DEG
Bpev01.c0190.g0014.m0001	2.206061565	2.56E-06	7.79E-05	DEG
Bpev01.c1117.g0016.m0001	0.923766722	0.00651662	0.04479166	DEG
Bpev01.c1651.g0001.m0001	1.773275848	8.34E-06	0.00021263	DEG
Bpev01.c1100.g0017.m0001	-1.400277138	0.00012646	0.00197522	DEG
Bpev01.c0557.g0054.m0001	-2.384341155	0.00075934	0.00832873	DEG
Bpev01.c0284.g0013.m0001	-1.085372061	0.00089424	0.00949482	DEG
Bpev01.c0896.g0010.m0001	-3.033080578	0.00357527	0.02855465	DEG
Bpev01.c1405.g0008.m0001	-0.847619305	0.00720725	0.04832561	DEG
Bpev01.c0455.g0030.m0001	-1.960668717	0.00720631	0.04832561	DEG
Bpev01.c2218.g0002.m0001	2.377153031	6.70E-11	7.86E-09	DEG
Bpev01.c0962.g0003.m0001	1.100158413	0.00549774	0.03939824	DEG
Bpev01.c0577.g0031.m0001	-2.692618094	1.50E-10	1.63E-08	DEG
Bpev01.c0265.g0003.m0001	-1.277480533	2.55E-05	0.00053135	DEG
Bpev01.c2230.g0005.m0001	-1.186534762	0.00026287	0.00354501	DEG
Bpev01.c1431.g0003.m0002	2.825291937	1.15E-10	1.29E-08	DEG
Bpev01.c0577.g0030.m0001	-2.436673845	7.38E-05	0.00126076	DEG
Bpev01.c1783.g0003.m0001	3.950455517	5.71E-05	0.00102813	DEG
Bpev01.c1773.g0006.m0001	-1.160103618	0.00018881	0.00272877	DEG
Bpev01.c0261.g0035.m0001	-0.920770603	0.00357119	0.02852975	DEG
Bpev01.c0280.g0034.m0001	2.514337713	1.24E-09	1.08E-07	DEG
Bpev01.c0746.g0005.m0001	1.220157799	0.00535335	0.0386268	DEG
Bpev01.c0025.g0028.m0001	3.281378013	1.05E-10	1.18E-08	DEG
Bpev01.c0022.g0121.m0001	1.765523573	9.22E-06	0.00023055	DEG
Bpev01.c2139.g0001.m0001	3.013091729	1.76E-06	5.70E-05	DEG
Bpev01.c1170.g0009.m0001	-1.131334708	0.00045751	0.00560031	DEG
Bpev01.c0550.g0004.m0001	-0.910711494	0.00348502	0.02800861	DEG
Bpev01.c0050.g0038.m0001	1.980207506	0.00459398	0.03441277	DEG
Bpev01.c0130.g0002.m0001	2.897712361	5.73E-05	0.00103006	DEG
Bpev01.c0000.g0038.m0001	1.131398368	0.00139025	0.0134453	DEG
Bpev01.c0094.g0068.m0001	3.992536328	3.06E-08	1.76E-06	DEG
Bpev01.c0020.g0007.m0001	2.792239483	6.68E-07	2.52E-05	DEG
Bpev01.c1155.g0004.m0001	1.872105924	0.00278636	0.02349687	DEG
Bpev01.c0566.g0015.m0001	-1.222638979	0.00030547	0.0040003	DEG
Bpev01.c0015.g0160.m0001	1.356161709	0.00167402	0.01571627	DEG
Bpev01.c0051.g0200.m0001	2.292068451	4.87E-10	4.67E-08	DEG
Bpev01.c0029.g0089.m0001	2.157123073	8.88E-07	3.23E-05	DEG
Bpev01.c0335.g0018.m0001	2.748119688	0.00195483	0.01777276	DEG
Bpev01.c3139.g0003.m0001	1.247364169	0.00541999	0.03900249	DEG
Bpev01.c2450.g0001.m0001	1.78562592	1.44E-06	4.81E-05	DEG
Bpev01.c0027.g0026.m0001	-2.024045145	0.0001918	0.00275837	DEG
Bpev01.c0568.g0002.m0001	1.505136562	3.35E-06	9.84E-05	DEG

Bpev01.c0803.g0012.m0001	-1.414019577	0.0046947	0.03502533	DEG
Bpev01.c0142.g0012.m0001	-1.165462423	0.00050328	0.00605973	DEG
Bpev01.c0352.g0006.m0001	2.55308317	0.00044626	0.00548993	DEG
Bpev01.c0121.g0001.m0001	-3.057265886	0.00380634	0.02991317	DEG
Bpev01.c0263.g0003.m0001	-2.258982436	4.22E-06	0.00011861	DEG
Bpev01.c0154.g0006.m0001	1.116732357	0.00053322	0.00634371	DEG
Bpev01.c0586.g0003.m0001	-1.078351875	0.00087785	0.00937358	DEG
Bpev01.c1148.g0013.m0001	-1.215832117	0.00150221	0.01437227	DEG
Bpev01.c0878.g0013.m0001	-6.308482612	0.00493412	0.03630068	DEG
Bpev01.c3351.g0001.m0001	-1.684271	0.00068865	0.00773122	DEG
Bpev01.c1957.g0003.m0001	1.106260796	0.0004316	0.00534762	DEG
Bpev01.c0892.g0006.m0001	1.264625254	0.00021069	0.00296622	DEG
Bpev01.c1043.g0003.m0002	1.592894497	0.00274346	0.02319493	DEG
Bpev01.c0212.g0002.m0001	2.507950097	2.44E-08	1.46E-06	DEG
Bpev01.c0288.g0031.m0001	-2.71040383	0.00028938	0.00383914	DEG
Bpev01.c0243.g0044.m0002	2.952944701	6.24E-08	3.27E-06	DEG
Bpev01.c1700.g0004.m0001	1.186350763	0.00285202	0.02391358	DEG
Bpev01.c1671.g0002.m0001	3.611217939	0.0039774	0.03092725	DEG
Bpev01.c1702.g0009.m0001	-1.221836103	0.00380358	0.02990751	DEG
Bpev01.c0457.g0022.m0001	1.268081178	0.00021901	0.00306004	DEG
Bpev01.c0088.g0023.m0001	1.354737052	9.38E-05	0.00153967	DEG
Bpev01.c1233.g0005.m0001	-1.300826409	0.00032015	0.00415743	DEG
Bpev01.c0112.g0010.m0001	2.305279269	0.00121096	0.01202744	DEG
Bpev01.c0994.g0009.m0001	3.601858225	3.77E-12	5.63E-10	DEG
Bpev01.c0808.g0002.m0001	-0.809805899	0.00696608	0.04710026	DEG
Bpev01.c0210.g0004.m0001	-1.002303094	0.00450312	0.0338614	DEG
Bpev01.c0253.g0008.m0002	-1.890584359	7.13E-08	3.64E-06	DEG
Bpev01.c1688.g0007.m0001	2.321601099	0.00164452	0.01549219	DEG
Bpev01.c0215.g0012.m0001	-1.511032519	6.93E-06	0.00018104	DEG
Bpev01.c0213.g0003.m0001	-1.193180854	0.00019675	0.00281077	DEG
Bpev01.c1243.g0010.m0001	-2.574911208	2.29E-05	0.00048464	DEG
Bpev01.c0123.g0019.m0001	1.174377229	0.00387893	0.03033155	DEG
Bpev01.c1142.g0009.m0001	3.248782681	3.05E-13	5.80E-11	DEG
Bpev01.c0000.g0072.m0001	-1.936596068	1.91E-05	0.00041997	DEG
Bpev01.c0461.g0006.m0001	2.500347375	2.58E-05	0.00053739	DEG
Bpev01.c0332.g0003.m0001	1.796517267	1.69E-07	7.63E-06	DEG
Bpev01.c0782.g0006.m0001	1.803814173	0.00202591	0.01821645	DEG
Bpev01.c1077.g0009.m0001	1.865864519	1.87E-07	8.33E-06	DEG
Bpev01.c0022.g0085.m0002	1.27953137	0.00755485	0.0499904	DEG
Bpev01.c1709.g0001.m0001	-0.953842803	0.006725	0.04583868	DEG
Bpev01.c0794.g0009.m0001	-1.921828791	2.62E-07	1.11E-05	DEG
Bpev01.c0120.g0007.m0001	0.900132451	0.00692076	0.04686917	DEG
Bpev01.c0462.g0001.m0001	2.448666733	1.09E-05	0.00026488	DEG

Bpev01.c0015.g0258.m0001	1.809814745	0.00075811	0.00831834	DEG
Bpev01.c1341.g0002.m0001	1.852917579	6.08E-08	3.20E-06	DEG
Bpev01.c0374.g0023.m0001	1.574931618	4.44E-06	0.00012364	DEG
Bpev01.c1579.g0008.m0001	0.923236472	0.0035235	0.02824073	DEG
Bpev01.c1468.g0005.m0001	-1.425579414	0.00104003	0.01064217	DEG
Bpev01.c0142.g0035.m0001	1.124983755	0.0004789	0.00582338	DEG
Bpev01.c2413.g0001.m0001	1.108241565	0.00413907	0.03181223	DEG
Bpev01.c1671.g0003.m0001	2.008387983	0.00088026	0.00938913	DEG
Bpev01.c1657.g0004.m0001	1.636514182	0.0041664	0.03194963	DEG
Bpev01.c0464.g0033.m0001	1.079979649	0.00556809	0.03970907	DEG
Bpev01.c0364.g0008.m0001	-2.013601521	0.00084263	0.00907985	DEG
Bpev01.c1063.g0012.m0001	-1.850332779	0.00491809	0.03624037	DEG
Bpev01.c1963.g0004.m0001	3.870893446	0.00041321	0.00515665	DEG
Bpev01.c0052.g0007.m0001	2.446082863	2.12E-06	6.63E-05	DEG
Bpev01.c0245.g0042.m0001	-2.397942249	7.26E-08	3.71E-06	DEG
Bpev01.c0190.g0042.m0001	1.670063512	0.00092619	0.0097411	DEG
Bpev01.c0455.g0012.m0001	-1.120447756	0.00134204	0.01308221	DEG
Bpev01.c1869.g0002.m0001	-1.267387285	0.00703845	0.047437	DEG
Bpev01.c0842.g0018.m0001	-0.874546519	0.00691613	0.04684859	DEG
Bpev01.c1160.g0005.m0002	1.359491303	0.00122647	0.01215283	DEG
Bpev01.c1179.g0007.m0001	9.28564651	4.47E-08	2.46E-06	DEG
Bpev01.c0754.g0001.m0001	2.412242032	0.00157044	0.01489438	DEG
Bpev01.c0348.g0002.m0001	0.878514355	0.00482185	0.03570954	DEG
Bpev01.c0282.g0001.m0001	1.885425564	3.97E-05	0.00076234	DEG
Bpev01.c0257.g0003.m0001	1.357325957	5.00E-05	0.00092552	DEG
Bpev01.c0522.g0040.m0001	1.945269844	4.98E-07	1.94E-05	DEG
Bpev01.c0145.g0054.m0001	1.372794564	9.05E-06	0.00022722	DEG
Bpev01.c0473.g0052.m0001	-1.917936648	6.11E-05	0.00108498	DEG
Bpev01.c0222.g0008.m0001	-2.768350531	0.00280339	0.02359321	DEG
Bpev01.c0395.g0027.m0001	-1.007986883	0.00169652	0.01586528	DEG
Bpev01.c1653.g0002.m0001	2.239250222	0.00569875	0.04035736	DEG
Bpev01.c1377.g0003.m0001	3.317797191	7.12E-07	2.66E-05	DEG
Bpev01.c0241.g0029.m0001	-2.192182848	0.00327829	0.02668962	DEG
Bpev01.c0127.g0069.m0001	-1.500890131	0.00169537	0.01586461	DEG
Bpev01.c0171.g0010.m0001	1.050561763	0.00072546	0.00803793	DEG
Bpev01.c0317.g0001.m0001	2.34634337	1.56E-07	7.16E-06	DEG
Bpev01.c0454.g0012.m0001	-2.663432949	3.09E-08	1.77E-06	DEG
Bpev01.c1435.g0004.m0001	-1.734249236	1.43E-05	0.00033405	DEG
Bpev01.c3181.g0001.m0001	2.254893713	3.21E-05	0.00064323	DEG
Bpev01.c0257.g0027.m0001	-2.435513968	0.00488876	0.03607929	DEG
Bpev01.c1553.g0003.m0001	1.916362012	0.0011443	0.01151302	DEG
Bpev01.c0564.g0022.m0001	-1.113937219	0.00044379	0.00546871	DEG
Bpev01.c0782.g0011.m0001	3.102619717	0.00109776	0.01112045	DEG

Bpev01.c5598.g0001.m0001	-1.939712997	0.00287508	0.02405896	DEG
Bpev01.c0614.g0010.m0001	-0.943232715	0.00220386	0.01948333	DEG
Bpev01.c0364.g0033.m0001	3.888211061	2.99E-15	8.81E-13	DEG
Bpev01.c0080.g0126.m0001	1.290460671	0.00015148	0.00227912	DEG
Bpev01.c0134.g0139.m0002	1.054206218	0.00367687	0.02917352	DEG
Bpev01.c1031.g0015.m0001	-1.899730376	1.85E-07	8.23E-06	DEG
Bpev01.c1112.g0010.m0001	1.236125326	0.00024503	0.00335039	DEG
Bpev01.c1069.g0009.m0001	-1.492289637	0.00095971	0.01001522	DEG
Bpev01.c0528.g0002.m0001	-1.404822907	0.0001752	0.00256606	DEG
Bpev01.c1043.g0005.m0001	2.457876106	0.00116264	0.01165765	DEG
Bpev01.c0863.g0021.m0001	1.096806866	0.00350609	0.02813184	DEG
Bpev01.c1565.g0007.m0001	1.788391637	0.00192259	0.01756016	DEG
Bpev01.c0024.g0013.m0001	1.073003326	0.00487904	0.03601658	DEG
Bpev01.c0894.g0011.m0001	-2.28447793	2.09E-08	1.26E-06	DEG
Bpev01.c1827.g0011.m0001	1.604392739	0.00054715	0.00644303	DEG
Bpev01.c0764.g0015.m0001	6.266061247	1.17E-14	3.06E-12	DEG
Bpev01.c0052.g0090.m0002	1.237509017	0.00011442	0.0018149	DEG
Bpev01.c0644.g0005.m0002	1.366720181	0.00061943	0.00711238	DEG
Bpev01.c0652.g0015.m0001	-0.998389146	0.00571012	0.04037004	DEG
Bpev01.c0469.g0002.m0001	-1.91048295	1.85E-06	5.96E-05	DEG
Bpev01.c0066.g0044.m0001	-1.017851353	0.00102837	0.01054845	DEG
Bpev01.c0940.g0005.m0001	-1.077172763	0.00222465	0.01960819	DEG
Bpev01.c0622.g0002.m0001	3.197231016	1.44E-12	2.40E-10	DEG
Bpev01.c1972.g0007.m0002	0.8351045	0.00733814	0.04894954	DEG
Bpev01.c0052.g0089.m0001	-1.079911603	0.00571721	0.04041028	DEG
Bpev01.c0033.g0002.m0001	1.39482545	0.00032794	0.00424175	DEG
Bpev01.c0690.g0001.m0001	-0.845926229	0.00739948	0.04922965	DEG
Bpev01.c0115.g0018.m0002	1.224337803	0.00150852	0.01441661	DEG
Bpev01.c0135.g0081.m0001	0.915749957	0.00497074	0.03650126	DEG
Bpev01.c0062.g0022.m0001	-1.159986754	0.00194304	0.01768198	DEG
Bpev01.c1527.g0012.m0001	1.059931916	0.00278284	0.02347394	DEG
Bpev01.c2582.g0002.m0001	2.460194211	0.00269067	0.02283389	DEG
Bpev01.c1515.g0002.m0002	2.291587386	3.19E-06	9.41E-05	DEG
Bpev01.c0191.g0041.m0001	2.323847865	2.88E-07	1.21E-05	DEG
Bpev01.c0694.g0001.m0001	2.602312482	2.78E-06	8.37E-05	DEG
Bpev01.c1040.g0007.m0001	-1.422266941	0.00014555	0.00220759	DEG
Bpev01.c0328.g0003.m0001	-0.892706031	0.00379139	0.02985147	DEG
Bpev01.c0802.g0013.m0001	3.941169149	1.32E-09	1.14E-07	DEG
Bpev01.c1378.g0007.m0001	1.568278414	7.23E-05	0.00124069	DEG
Bpev01.c2934.g0001.m0001	2.554046701	0.00098835	0.0102523	DEG
Bpev01.c0062.g0081.m0001	3.480140002	1.56E-08	9.85E-07	DEG
Bpev01.c1534.g0003.m0001	-1.151017977	0.00034209	0.00439579	DEG
Bpev01.c0422.g0018.m0002	1.047965812	0.00387915	0.03033155	DEG

Bpev01.c0776.g0011.m0001	2.554599941	9.88E-05	0.00160213	DEG
Bpev01.c0135.g0105.m0001	-1.063841716	0.00071113	0.00793589	DEG
Bpev01.c0320.g0016.m0001	-1.10833921	0.0007408	0.00817406	DEG
Bpev01.c0800.g0005.m0001	-1.138357497	0.00019678	0.00281077	DEG
Bpev01.c2105.g0003.m0001	-1.912290851	0.00621734	0.04315783	DEG
Bpev01.c0102.g0008.m0001	-1.23103823	0.00097149	0.01010594	DEG
Bpev01.c0088.g0061.m0001	-2.543909894	9.57E-12	1.31E-09	DEG
Bpev01.c0177.g0039.m0001	-1.73514389	1.09E-07	5.22E-06	DEG
Bpev01.c2047.g0001.m0001	3.551263807	0.00076707	0.00839087	DEG
Bpev01.c3332.g0001.m0001	2.644921084	2.99E-05	0.00060658	DEG
Bpev01.c0321.g0008.m0001	-1.082091176	0.00065552	0.00744683	DEG
Bpev01.c0082.g0089.m0001	2.634541731	1.53E-05	0.00035207	DEG
Bpev01.c1178.g0005.m0001	3.332187612	5.44E-17	2.32E-14	DEG
Bpev01.c0022.g0138.m0001	1.077305028	0.00570519	0.04037004	DEG
Bpev01.c0860.g0011.m0001	0.859931022	0.00747896	0.04964443	DEG
Bpev01.c0320.g0009.m0001	-2.18413091	0.00143996	0.01386232	DEG
Bpev01.c0345.g0005.m0001	2.186419132	0.00548355	0.03933487	DEG
Bpev01.c1152.g0014.m0001	-2.116181406	2.44E-07	1.05E-05	DEG
Bpev01.c0224.g0046.m0001	-2.307137845	0.00027453	0.00367198	DEG
Bpev01.c0441.g0008.m0001	-2.134074072	0.00286719	0.02402022	DEG
Bpev01.c0274.g0056.m0001	-1.823753052	0.00717812	0.0482238	DEG
Bpev01.c1202.g0050.m0001	-1.198095256	0.00426284	0.03247769	DEG
Bpev01.c0001.g0043.m0001	3.517856136	1.22E-10	1.35E-08	DEG
Bpev01.c0023.g0049.m0001	3.349799039	0.0019875	0.01796993	DEG
Bpev01.c0027.g0082.m0001	-1.412470353	1.88E-05	0.00041548	DEG
Bpev01.c0073.g0013.m0001	4.351499277	8.93E-05	0.00147768	DEG
Bpev01.c0111.g0005.m0001	1.636100927	0.00370817	0.0293217	DEG
Bpev01.c0213.g0014.m0001	-2.065079791	2.90E-05	0.00059097	DEG
Bpev01.c0457.g0023.m0001	1.377248939	0.00381031	0.02993644	DEG
Bpev01.c0781.g0012.m0001	1.24682552	0.00449782	0.03383016	DEG
Bpev01.c1117.g0020.m0001	-2.193884367	3.13E-07	1.29E-05	DEG
Bpev01.c0022.g0032.m0001	-2.180764229	0.00030802	0.0040265	DEG
Bpev01.c0617.g0019.m0001	1.125242439	0.00193041	0.01759937	DEG
Bpev01.c0146.g0024.m0001	-1.958906011	0.00034642	0.00444177	DEG
Bpev01.c0304.g0002.m0001	-1.880132273	0.00606343	0.04228471	DEG
Bpev01.c0661.g0011.m0001	-1.108385854	0.00028016	0.00374385	DEG
Bpev01.c0173.g0004.m0001	-1.410663195	0.00255103	0.02188858	DEG
Bpev01.c1565.g0011.m0001	-6.933866899	0.0008958	0.00950363	DEG
Bpev01.c1524.g0002.m0001	-1.60577576	0.00115279	0.01157077	DEG
Bpev01.c0043.g0005.m0001	-2.402846613	5.29E-06	0.00014365	DEG
Bpev01.c1979.g0002.m0001	-3.352876829	1.02E-09	9.15E-08	DEG
Bpev01.c0473.g0006.m0001	-1.20246265	8.58E-05	0.00143028	DEG
Bpev01.c0027.g0078.m0001	0.958705417	0.0027387	0.02316798	DEG

Bpev01.c0018.g0060.m0001	-1.066403543	0.00056258	0.00657992	DEG
Bpev01.c0354.g0003.m0001	0.888068505	0.0042218	0.03225686	DEG
Bpev01.c0229.g0010.m0001	-0.873905413	0.00492044	0.03624037	DEG
Bpev01.c0228.g0009.m0001	-1.027731203	0.00116649	0.01168436	DEG
Bpev01.c1040.g0043.m0001	0.914788527	0.00415163	0.0318613	DEG
Bpev01.c2462.g0002.m0001	2.458964615	0.00014553	0.00220759	DEG
Bpev01.c1600.g0016.m0001	-1.042117262	0.00242964	0.02101855	DEG
Bpev01.c0027.g0140.m0001	1.865941956	1.64E-05	0.00037268	DEG
Bpev01.c0166.g0016.m0001	-0.923877442	0.00712775	0.04795108	DEG
Bpev01.c0645.g0025.m0001	-3.451500548	0.00065844	0.00747173	DEG
Bpev01.c1051.g0001.m0001	2.034309065	0.00427513	0.03254606	DEG
Bpev01.c0089.g0056.m0001	0.810380483	0.00752164	0.04986457	DEG
Bpev01.c0457.g0040.m0001	1.16243603	0.00736184	0.04905803	DEG
Bpev01.c2673.g0001.m0001	-2.415331199	0.00726563	0.04862882	DEG
Bpev01.c0029.g0038.m0001	-2.108167223	0.0013191	0.01290566	DEG
Bpev01.c1267.g0012.m0001	1.743784849	1.85E-05	0.00041076	DEG
Bpev01.c0817.g0038.m0001	-7.117149056	0.00117731	0.01177273	DEG
Bpev01.c0089.g0055.m0001	-1.947150541	4.27E-07	1.71E-05	DEG
Bpev01.c1150.g0012.m0001	-0.849518489	0.00594035	0.04163149	DEG
Bpev01.c1281.g0013.m0001	-0.978112003	0.00236553	0.02058496	DEG
Bpev01.c1202.g0015.m0001	-1.200887263	0.00074577	0.00821042	DEG
Bpev01.c0281.g0061.m0001	-1.739919387	1.40E-08	8.93E-07	DEG
Bpev01.c0444.g0024.m0001	-0.966592213	0.00660188	0.04527199	DEG
Bpev01.c0289.g0005.m0001	1.650114707	0.00345596	0.02782825	DEG
Bpev01.c0865.g0004.m0001	-3.099253794	0.00420618	0.03216249	DEG
Bpev01.c0404.g0030.m0001	-2.259488857	0.00386286	0.03024438	DEG
Bpev01.c0572.g0007.m0001	-1.535054559	0.00014982	0.00226071	DEG
Bpev01.c2934.g0002.m0001	4.036377816	2.11E-11	2.69E-09	DEG
Bpev01.c0115.g0111.m0001	0.956028655	0.00257322	0.02204046	DEG
Bpev01.c1290.g0004.m0001	1.339533245	0.00339102	0.0274103	DEG
Bpev01.c1067.g0007.m0001	1.07135454	0.00403949	0.03127789	DEG
Bpev01.c0602.g0010.m0001	1.717303133	8.83E-05	0.00146451	DEG
Bpev01.c0016.g0046.m0001	-1.353992013	1.10E-05	0.00026596	DEG
Bpev01.c0154.g0054.m0001	-2.128243891	0.00062573	0.00716763	DEG
Bpev01.c1607.g0006.m0001	1.753374552	1.05E-05	0.00025721	DEG
Bpev01.c0018.g0056.m0001	1.081556999	0.00039484	0.00496957	DEG
Bpev01.c0288.g0023.m0001	1.194090332	9.52E-05	0.00155787	DEG
Bpev01.c0122.g0002.m0001	-1.635381207	3.19E-06	9.42E-05	DEG
Bpev01.c0950.g0015.m0001	1.014886748	0.00178207	0.01652353	DEG
Bpev01.c0909.g0005.m0001	-1.526416204	9.61E-06	0.0002383	DEG
Bpev01.c0327.g0038.m0001	1.362415114	6.41E-05	0.00112477	DEG
Bpev01.c0129.g0047.m0001	-1.875178017	2.75E-08	1.61E-06	DEG
Bpev01.c0552.g0014.m0001	1.003787477	0.00184561	0.01702155	DEG

Bpev01.c0575.g0013.m0001	-0.901893047	0.00526104	0.03810212	DEG
Bpev01.c0253.g0010.m0001	3.33359347	8.54E-17	3.44E-14	DEG
Bpev01.c0241.g0002.m0002	3.703814639	3.38E-10	3.41E-08	DEG
Bpev01.c0129.g0031.m0001	1.776094225	1.82E-05	0.0004045	DEG
Bpev01.c0031.g0018.m0001	0.827530725	0.00727106	0.04864835	DEG
Bpev01.c0018.g0031.m0001	3.422138592	7.60E-17	3.11E-14	DEG
Bpev01.c0268.g0006.m0001	-1.532256789	0.00226379	0.01989961	DEG
Bpev01.c1253.g0001.m0001	-1.661420405	2.27E-05	0.00048239	DEG
Bpev01.c0000.g0111.m0001	-1.236228866	9.53E-05	0.0015585	DEG
Bpev01.c0848.g0020.m0001	1.982339106	8.25E-06	0.00021068	DEG
Bpev01.c0141.g0013.m0001	0.83467009	0.00613339	0.04264902	DEG
Bpev01.c0553.g0013.m0001	3.512665782	1.14E-08	7.51E-07	DEG
Bpev01.c0301.g0024.m0001	-0.890387181	0.00301359	0.02495173	DEG
Bpev01.c0022.g0159.m0001	1.590661598	0.00666621	0.04556459	DEG
Bpev01.c2213.g0001.m0001	1.612416307	0.00070505	0.007883	DEG
Bpev01.c0347.g0002.m0001	-2.827767823	2.44E-10	2.54E-08	DEG
Bpev01.c0161.g0065.m0001	-1.165288653	0.0002273	0.00315145	DEG
Bpev01.c0276.g0018.m0001	3.423679359	4.38E-16	1.52E-13	DEG
Bpev01.c0095.g0011.m0001	-2.28053024	5.47E-05	0.00099196	DEG
Bpev01.c1100.g0013.m0001	-1.932341952	0.00283639	0.02382183	DEG
Bpev01.c2742.g0002.m0001	3.116723567	1.31E-05	0.00031074	DEG
Bpev01.c0082.g0009.m0001	-1.782669266	3.61E-07	1.47E-05	DEG
Bpev01.c0141.g0035.m0001	-3.088366626	1.52E-07	7.00E-06	DEG
Bpev01.c1382.g0001.m0001	-1.367675835	0.00054511	0.00643008	DEG
Bpev01.c0022.g0054.m0001	-1.304861012	0.0003538	0.00451863	DEG
Bpev01.c0101.g0034.m0001	2.935940947	0.00377521	0.02973207	DEG
Bpev01.c0027.g0147.m0001	-2.447449835	6.82E-13	1.22E-10	DEG
Bpev01.c1161.g0016.m0001	2.934072619	2.03E-15	6.50E-13	DEG
Bpev01.c1002.g0007.m0001	2.350022676	1.33E-09	1.15E-07	DEG
Bpev01.c1273.g0001.m0002	1.543847385	0.00011609	0.00184033	DEG
Bpev01.c2260.g0002.m0001	0.961204902	0.00496193	0.03645475	DEG
Bpev01.c1152.g0011.m0001	3.792764231	2.03E-20	1.71E-17	DEG
Bpev01.c0324.g0019.m0001	1.328987459	9.28E-05	0.0015253	DEG
Bpev01.c0672.g0007.m0001	-2.296304365	5.52E-07	2.13E-05	DEG
Bpev01.c0038.g0161.m0001	1.435108628	0.00037116	0.00471578	DEG
Bpev01.c0027.g0141.m0001	1.265512673	0.00049518	0.00599013	DEG
Bpev01.c0865.g0005.m0001	0.875986531	0.00523953	0.03800113	DEG
Bpev01.c0135.g0083.m0001	-2.382584881	0.00072095	0.00801212	DEG
Bpev01.c0088.g0116.m0001	1.01900962	0.00507891	0.03705527	DEG
Bpev01.c0275.g0004.m0001	-1.458518744	0.00123527	0.01223179	DEG
Bpev01.c0442.g0012.m0001	3.174844969	0.00015698	0.00235188	DEG
Bpev01.c0561.g0004.m0001	2.697334076	0.0004371	0.00540212	DEG
Bpev01.c1095.g0002.m0001	1.604582304	0.00092366	0.00972862	DEG

Bpev01.c3692.g0001.m0001	2.422786827	6.50E-06	0.00017058	DEG
Bpev01.c0229.g0021.m0001	-1.684934792	0.00393334	0.03064132	DEG
Bpev01.c1222.g0002.m0001	-1.549699387	2.77E-06	8.37E-05	DEG
Bpev01.c0367.g0007.m0001	-1.669139134	1.57E-05	0.00035934	DEG
Bpev01.c0772.g0012.m0001	-0.842494604	0.00720196	0.04832561	DEG
Bpev01.c0050.g0163.m0001	2.408049841	1.29E-06	4.43E-05	DEG
Bpev01.c0018.g0095.m0001	-1.549047966	0.00629866	0.04362968	DEG
Bpev01.c0053.g0019.m0001	1.508654356	0.00074297	0.00818578	DEG
Bpev01.c0044.g0072.m0001	-1.142537001	0.0006413	0.00730618	DEG
Bpev01.c0480.g0075.m0001	2.324222889	3.55E-05	0.00069873	DEG
Bpev01.c0848.g0019.m0001	2.022094226	2.05E-05	0.00044495	DEG
Bpev01.c1230.g0003.m0001	-1.240811892	0.00012534	0.00196379	DEG
Bpev01.c0889.g0016.m0001	-2.628296734	4.91E-08	2.66E-06	DEG
Bpev01.c0030.g0030.m0001	1.489715562	1.31E-05	0.00030972	DEG
Bpev01.c2783.g0001.m0001	2.443028014	5.30E-08	2.85E-06	DEG
Bpev01.c0420.g0021.m0001	2.328233672	2.71E-08	1.59E-06	DEG
Bpev01.c0141.g0024.m0001	-1.792384247	2.61E-08	1.54E-06	DEG
Bpev01.c1579.g0015.m0001	-1.798430629	2.13E-07	9.28E-06	DEG
Bpev01.c0403.g0018.m0001	-2.003566531	0.00011694	0.00184962	DEG
Bpev01.c0824.g0003.m0001	-1.18183357	0.00237693	0.02064748	DEG
Bpev01.c1145.g0003.m0001	1.348412065	0.00209563	0.01873468	DEG
Bpev01.c0776.g0009.m0001	3.856222502	3.53E-09	2.68E-07	DEG
Bpev01.c0480.g0058.m0001	-1.611021539	0.00408159	0.0314962	DEG
Bpev01.c0389.g0015.m0001	-1.325636538	0.00179613	0.01663303	DEG
Bpev01.c2242.g0002.m0001	3.061886257	4.32E-06	0.0001209	DEG
Bpev01.c0425.g0048.m0001	-1.506082822	8.07E-05	0.00135847	DEG
Bpev01.c0390.g0001.m0001	-1.717435199	9.40E-06	0.00023407	DEG
Bpev01.c1600.g0013.m0001	2.47809813	3.82E-05	0.00074008	DEG
Bpev01.c0145.g0051.m0002	-1.733495922	4.69E-07	1.84E-05	DEG
Bpev01.c0062.g0048.m0001	-1.341476614	0.00019503	0.00279663	DEG
Bpev01.c1027.g0003.m0001	-1.406202651	0.00331052	0.0268851	DEG
Bpev01.c0038.g0151.m0001	-2.174681451	0.00120043	0.01195111	DEG
Bpev01.c0038.g0009.m0001	-2.045316044	0.00089436	0.00949482	DEG
Bpev01.c0504.g0023.m0001	-2.300699919	0.00268511	0.02280625	DEG
Bpev01.c0016.g0166.m0001	-1.584770783	0.00147014	0.01411131	DEG
Bpev01.c0243.g0032.m0001	0.818860277	0.00677774	0.04613225	DEG
Bpev01.c0052.g0067.m0001	-1.501925587	2.17E-05	0.00046515	DEG
Bpev01.c0837.g0008.m0001	1.081996286	0.00707389	0.04765404	DEG
Bpev01.c1177.g0005.m0001	-1.751849878	0.00072018	0.00801114	DEG
Bpev01.c0362.g0012.m0001	-1.050718785	0.00067191	0.00759029	DEG
Bpev01.c0027.g0162.m0001	-1.149888708	0.00482289	0.03570954	DEG
Bpev01.c0863.g0011.m0001	1.151204248	0.00282014	0.02370239	DEG
Bpev01.c0229.g0008.m0001	-2.030081551	0.00272952	0.02312353	DEG

Bpev01.c0563.g0004.m0001	1.068861847	0.0017684	0.01641232	DEG
Bpev01.c0495.g0009.m0001	1.954155428	0.00061056	0.00702666	DEG
Bpev01.c0154.g0049.m0001	-1.702575341	0.00012597	0.00197155	DEG
Bpev01.c0727.g0010.m0001	-1.345139728	0.00012698	0.00198207	DEG
Bpev01.c1581.g0003.m0001	-3.236950372	3.12E-16	1.12E-13	DEG
Bpev01.c0364.g0006.m0001	-1.105956133	0.00201055	0.01812263	DEG
Bpev01.c0101.g0051.m0001	-0.930942205	0.0042401	0.03232239	DEG
Bpev01.c0803.g0017.m0001	-1.448016921	1.28E-05	0.00030534	DEG
Bpev01.c0038.g0158.m0001	1.328761329	0.00138867	0.01343445	DEG
Bpev01.c1600.g0007.m0001	-1.561145682	3.49E-06	0.0001021	DEG
Bpev01.c0645.g0017.m0001	3.237031642	6.94E-14	1.50E-11	DEG
Bpev01.c0473.g0025.m0001	1.01135455	0.00289896	0.02421749	DEG
Bpev01.c0373.g0006.m0001	-2.170554339	5.99E-05	0.00107103	DEG
Bpev01.c0624.g0025.m0001	-1.286793353	0.00020363	0.00288345	DEG
Bpev01.c0357.g0035.m0001	1.252814138	0.00031209	0.00406898	DEG
Bpev01.c0489.g0013.m0001	1.794120564	0.00350991	0.02815483	DEG
Bpev01.c1876.g0005.m0001	-1.925990832	3.53E-05	0.00069457	DEG
Bpev01.c0485.g0036.m0001	-1.613201621	9.02E-05	0.00148768	DEG
Bpev01.c0962.g0029.m0001	-1.060556256	0.00120797	0.01200508	DEG
Bpev01.c0454.g0029.m0001	-1.904629704	4.31E-07	1.72E-05	DEG
Bpev01.c0265.g0026.m0001	-1.357293397	0.00410841	0.03166166	DEG
Bpev01.c0606.g0002.m0001	-1.329562394	3.39E-05	0.0006735	DEG
Bpev01.c1448.g0005.m0001	1.99829376	0.0001908	0.00275201	DEG
Bpev01.c0808.g0007.m0001	-1.632362311	0.00170775	0.01593986	DEG
Bpev01.c0353.g0029.m0001	2.206638679	4.05E-06	0.00011447	DEG
Bpev01.c2439.g0003.m0001	3.282062677	1.83E-07	8.19E-06	DEG
Bpev01.c0588.g0039.m0001	-1.204809219	0.00034466	0.00442198	DEG
Bpev01.c1577.g0001.m0001	-1.690362619	0.00722756	0.04844539	DEG
Bpev01.c0659.g0001.m0001	1.314905859	0.00058676	0.00680871	DEG
Bpev01.c0155.g0045.m0001	-2.386250093	1.02E-08	6.95E-07	DEG
Bpev01.c1252.g0001.m0001	3.167422355	4.39E-13	8.04E-11	DEG
Bpev01.c0000.g0073.m0001	-1.321072465	0.00055595	0.00652315	DEG
Bpev01.c0016.g0149.m0001	2.52199597	0.00103088	0.0105669	DEG
Bpev01.c0848.g0048.m0001	1.527598394	1.47E-06	4.89E-05	DEG
Bpev01.c0210.g0046.m0002	1.162569051	0.00016703	0.00247218	DEG
Bpev01.c0261.g0005.m0001	-1.081980155	0.00106525	0.01084743	DEG
Bpev01.c0964.g0003.m0001	-1.231819534	0.00076034	0.0083366	DEG
Bpev01.c0050.g0102.m0001	0.908489227	0.00481754	0.03568784	DEG
Bpev01.c0522.g0018.m0001	-3.724776819	2.24E-07	9.70E-06	DEG
Bpev01.c0243.g0022.m0001	1.940462688	4.29E-07	1.71E-05	DEG
Bpev01.c0480.g0028.m0001	1.753381476	0.00035846	0.00457218	DEG
Bpev01.c2165.g0006.m0001	1.187914899	0.00411247	0.03168463	DEG
Bpev01.c0015.g0134.m0001	-1.226432704	0.0009556	0.00998651	DEG

Bpev01.c0000.g0100.m0001	1.728105145	0.00026914	0.00362122	DEG
Bpev01.c0129.g0052.m0001	4.636796003	4.93E-08	2.67E-06	DEG
Bpev01.c0488.g0027.m0001	2.740214273	3.97E-12	5.91E-10	DEG
Bpev01.c0115.g0072.m0001	-1.088604824	0.00196999	0.01787745	DEG
Bpev01.c1590.g0007.m0001	-1.795036255	9.65E-07	3.48E-05	DEG
Bpev01.c0727.g0011.m0001	-2.569057407	0.00019115	0.00275308	DEG
Bpev01.c0094.g0089.m0001	-3.87128838	0.00454227	0.03408902	DEG
Bpev01.c0871.g0001.m0001	3.801552737	0.00625132	0.04334256	DEG
Bpev01.c1480.g0002.m0001	2.212295379	3.96E-10	3.91E-08	DEG
Bpev01.c0207.g0008.m0001	-2.091848977	3.00E-06	8.92E-05	DEG
Bpev01.c3211.g0004.m0001	-1.01724246	0.00092041	0.00970486	DEG
Bpev01.c0052.g0075.m0001	-1.018123674	0.00150596	0.01440353	DEG
Bpev01.c0146.g0017.m0001	-1.357693546	0.00683555	0.04643094	DEG
Bpev01.c0055.g0015.m0001	1.564933725	0.00192395	0.01756016	DEG
Bpev01.c0170.g0003.m0001	1.003096769	0.00165509	0.01557676	DEG
Bpev01.c0630.g0018.m0001	2.040233098	0.00065862	0.00747173	DEG
Bpev01.c0044.g0104.m0001	-2.073621601	2.03E-07	8.93E-06	DEG
Bpev01.c0213.g0024.m0001	-1.292499699	0.00066878	0.00755784	DEG
Bpev01.c0566.g0050.m0003	2.142098987	4.47E-09	3.30E-07	DEG
Bpev01.c1051.g0003.m0001	1.772811306	0.00094144	0.00987003	DEG
Bpev01.c0661.g0018.m0001	-0.9322495	0.00731532	0.0488446	DEG
Bpev01.c0286.g0021.m0001	-2.149148002	0.00111628	0.01127736	DEG
Bpev01.c0640.g0008.m0001	8.35521001	3.71E-06	0.0001069	DEG
Bpev01.c0253.g0028.m0001	2.124528314	3.13E-07	1.29E-05	DEG
Bpev01.c0214.g0045.m0001	-1.18145749	0.00411404	0.03168848	DEG
Bpev01.c1494.g0002.m0001	1.366127347	6.27E-05	0.00110646	DEG
Bpev01.c0101.g0017.m0002	1.443837007	1.40E-05	0.00032701	DEG
Bpev01.c0088.g0123.m0001	3.759490355	0.00010786	0.0017267	DEG
Bpev01.c0566.g0030.m0001	1.372854999	0.00228518	0.0200397	DEG
Bpev01.c0123.g0018.m0001	1.677961399	0.00351434	0.02817498	DEG
Bpev01.c1077.g0010.m0001	3.058974017	2.77E-06	8.37E-05	DEG
Bpev01.c0866.g0008.m0001	-1.712316078	0.00079874	0.00866081	DEG
Bpev01.c0053.g0021.m0001	1.095664215	0.00157334	0.01491232	DEG
Bpev01.c0038.g0095.m0001	3.01314873	6.34E-11	7.46E-09	DEG
Bpev01.c1158.g0012.m0001	1.980959714	0.00030764	0.00402341	DEG
Bpev01.c1285.g0008.m0001	1.32189673	0.00011071	0.00176641	DEG
Bpev01.c1137.g0002.m0001	1.838090854	0.00063265	0.00723218	DEG
Bpev01.c0228.g0003.m0001	2.690508965	1.32E-08	8.60E-07	DEG
Bpev01.c1434.g0001.m0001	3.635732945	5.73E-05	0.00103006	DEG
Bpev01.c1227.g0001.m0001	1.655289565	0.00016711	0.00247218	DEG
Bpev01.c1833.g0002.m0001	-1.074799559	0.00118567	0.01183617	DEG
Bpev01.c0051.g0171.m0001	-2.657869186	3.63E-16	1.29E-13	DEG
Bpev01.c1292.g0002.m0001	1.40275684	0.00464891	0.03473585	DEG

Bpev01.c1104.g0015.m0001	0.84654223	0.00700731	0.0472976	DEG
Bpev01.c0497.g0002.m0001	1.651111987	0.00017753	0.00258929	DEG
Bpev01.c0288.g0009.m0001	1.692810875	0.00022915	0.00317312	DEG
Bpev01.c0030.g0015.m0001	-2.061389427	0.0002011	0.00285324	DEG
Bpev01.c3283.g0001.m0001	5.437130688	0.00016241	0.00241345	DEG
Bpev01.c1072.g0016.m0001	1.911211784	0.00072338	0.00802912	DEG
Bpev01.c0063.g0020.m0001	-1.138877921	0.00083354	0.00898525	DEG
Bpev01.c2269.g0003.m0001	1.625485877	1.57E-05	0.00036045	DEG
Bpev01.c1423.g0001.m0001	2.909596175	0.00056733	0.00661708	DEG
Bpev01.c0018.g0095.m0002	-1.673043904	0.00601461	0.04203414	DEG
Bpev01.c0280.g0025.m0001	1.101540072	0.00151604	0.01446696	DEG
Bpev01.c1372.g0006.m0001	2.814460942	9.49E-08	4.61E-06	DEG
Bpev01.c1756.g0008.m0001	-1.915468725	0.00126841	0.01249342	DEG
Bpev01.c2104.g0005.m0001	-1.54713482	0.00016061	0.0023928	DEG
Bpev01.c0149.g0006.m0001	-2.699405672	5.96E-05	0.00106683	DEG
Bpev01.c0018.g0055.m0001	-1.715056402	0.00048096	0.00584356	DEG
Bpev01.c0016.g0039.m0001	2.092812249	0.00295102	0.02455485	DEG
Bpev01.c0359.g0001.m0001	1.552740532	1.64E-06	5.35E-05	DEG
Bpev01.c0051.g0174.m0001	-1.630399627	5.20E-07	2.02E-05	DEG
Bpev01.c0265.g0027.m0001	-3.131022754	0.00043145	0.00534762	DEG
Bpev01.c0349.g0009.m0001	1.916912852	1.98E-06	6.27E-05	DEG
Bpev01.c2003.g0004.m0001	-2.157305906	0.00219589	0.01943032	DEG
Bpev01.c0901.g0005.m0001	-2.76469661	7.09E-08	3.63E-06	DEG
Bpev01.c0848.g0011.m0001	-1.759868857	4.66E-07	1.84E-05	DEG
Bpev01.c0443.g0015.m0001	-0.851385538	0.00641047	0.04425838	DEG
Bpev01.c0127.g0064.m0001	-2.16586936	7.52E-10	6.92E-08	DEG
Bpev01.c0027.g0021.m0001	-2.57251677	1.63E-12	2.67E-10	DEG
Bpev01.c0190.g0036.m0001	-0.915342404	0.0040015	0.03105724	DEG
Bpev01.c1391.g0008.m0001	1.404808596	2.30E-05	0.00048653	DEG
Bpev01.c1122.g0006.m0001	-2.103160486	1.71E-05	0.00038557	DEG
Bpev01.c0038.g0150.m0001	-2.585142532	4.19E-05	0.00079812	DEG
Bpev01.c1356.g0003.m0001	-3.289364697	4.65E-05	0.0008733	DEG
Bpev01.c1874.g0007.m0001	-2.232678704	0.0040505	0.03133018	DEG
Bpev01.c0667.g0008.m0001	1.165923868	0.00030293	0.00397769	DEG
Bpev01.c2668.g0001.m0001	1.681921252	0.00032957	0.00425914	DEG
Bpev01.c1135.g0011.m0001	-2.215053714	0.00607635	0.04232862	DEG
Bpev01.c0115.g0108.m0001	2.981610298	0.00063284	0.00723218	DEG
Bpev01.c1206.g0012.m0001	2.578483118	4.81E-14	1.08E-11	DEG
Bpev01.c1851.g0006.m0001	-1.939369308	0.0001387	0.00212886	DEG
Bpev01.c2998.g0001.m0001	1.547300448	9.26E-07	3.34E-05	DEG
Bpev01.c0569.g0008.m0001	1.253163887	9.19E-05	0.00151097	DEG
Bpev01.c0050.g0106.m0002	1.068563312	0.00165699	0.01558965	DEG
Bpev01.c0308.g0001.m0001	2.909085717	1.47E-05	0.00034002	DEG

Bpev01.c0027.g0087.m0001	1.22107863	0.00059854	0.00691541	DEG
Bpev01.c0652.g0024.m0001	1.147898521	0.00066381	0.00751319	DEG
Bpev01.c1163.g0019.m0001	3.686145742	6.20E-06	0.00016456	DEG
Bpev01.c0091.g0038.m0001	-1.836129552	0.00154515	0.01470193	DEG
Bpev01.c0212.g0003.m0001	2.657027153	2.27E-05	0.00048239	DEG
Bpev01.c0783.g0011.m0001	2.844766974	3.49E-06	0.0001021	DEG
Bpev01.c0286.g0027.m0001	-1.034162952	0.00176136	0.01637279	DEG
Bpev01.c0129.g0033.m0001	-1.559726302	7.22E-05	0.00123956	DEG
Bpev01.c2931.g0001.m0001	-1.383112329	0.00029253	0.00386878	DEG
Bpev01.c1878.g0001.m0001	-1.115774493	0.00147648	0.01416295	DEG
Bpev01.c0243.g0039.m0001	0.966093888	0.00289813	0.02421746	DEG
Bpev01.c0979.g0002.m0001	-1.101211057	0.00053854	0.00637739	DEG
Bpev01.c0451.g0010.m0001	-0.973080915	0.00138209	0.0133928	DEG
Bpev01.c0051.g0096.m0001	-1.804309478	0.00460594	0.0344848	DEG
Bpev01.c1260.g0016.m0001	-1.175953839	0.00207144	0.01855662	DEG
Bpev01.c0845.g0008.m0001	-1.02044593	0.00195312	0.01776822	DEG
Bpev01.c0000.g0246.m0001	1.906722591	0.0013791	0.01337707	DEG
Bpev01.c0304.g0030.m0001	4.11492887	7.61E-13	1.32E-10	DEG
Bpev01.c1286.g0004.m0001	1.119545005	0.00045345	0.00556548	DEG
Bpev01.c0016.g0134.m0001	-1.069357485	0.00392495	0.03059214	DEG
Bpev01.c0566.g0019.m0001	1.559589325	7.70E-05	0.00130816	DEG
Bpev01.c0188.g0034.m0001	3.071723705	4.68E-13	8.46E-11	DEG
Bpev01.c0044.g0083.m0001	-1.9386336	0.00012839	0.00199889	DEG
Bpev01.c0555.g0018.m0001	3.244931836	4.04E-22	4.41E-19	DEG
Bpev01.c2850.g0001.m0001	3.513050422	0.00073192	0.00808826	DEG
Bpev01.c1584.g0004.m0001	5.253496076	6.96E-05	0.00120388	DEG
Bpev01.c1382.g0010.m0001	-2.36553379	0.00086118	0.00924255	DEG
Bpev01.c2235.g0002.m0001	-1.074155224	0.0005583	0.00654293	DEG
Bpev01.c0706.g0001.m0001	2.765578705	1.16E-09	1.03E-07	DEG
Bpev01.c0120.g0024.m0001	-0.924964984	0.00343358	0.02767831	DEG
Bpev01.c1031.g0001.m0001	-2.129076322	9.64E-11	1.10E-08	DEG
Bpev01.c0828.g0019.m0001	-2.154042724	0.00426844	0.03251191	DEG
Bpev01.c1229.g0017.m0001	0.941025687	0.00417103	0.03196401	DEG
Bpev01.c0547.g0007.m0001	4.867952487	0.00048082	0.00584356	DEG
Bpev01.c0270.g0001.m0001	6.380699167	1.20E-08	7.87E-07	DEG
Bpev01.c0506.g0017.m0001	-1.735783553	1.03E-07	4.96E-06	DEG
Bpev01.c0094.g0012.m0001	1.364910798	0.0001579	0.00236086	DEG
Bpev01.c0197.g0001.m0001	0.911044214	0.00263501	0.02247162	DEG
Bpev01.c0040.g0003.m0001	2.4500942	0.00237132	0.02061704	DEG
Bpev01.c0615.g0010.m0001	-3.06781948	1.42E-16	5.52E-14	DEG
Bpev01.c0127.g0057.m0001	1.625087608	0.00114277	0.01150155	DEG
Bpev01.c0373.g0001.m0001	2.431396861	0.00055248	0.00649283	DEG
Bpev01.c1418.g0007.m0001	-1.169222077	0.00033541	0.00431944	DEG

Bpev01.c0033.g0001.m0001	1.038363672	0.00266328	0.02264708	DEG
Bpev01.c0671.g0006.m0001	1.271351421	0.00090934	0.00960535	DEG
Bpev01.c0212.g0018.m0001	-2.193243747	1.53E-11	2.01E-09	DEG
Bpev01.c0597.g0005.m0001	1.190730709	0.00051501	0.00616818	DEG
Bpev01.c0015.g0031.m0001	-1.564586469	8.94E-05	0.00147791	DEG
Bpev01.c0025.g0006.m0001	2.190782575	2.11E-09	1.70E-07	DEG
Bpev01.c0564.g0031.m0001	-2.927635635	1.61E-08	1.01E-06	DEG
Bpev01.c1158.g0003.m0001	1.766675983	0.00184376	0.01700989	DEG
Bpev01.c1468.g0003.m0001	-1.887721755	0.00258298	0.02210472	DEG
Bpev01.c0015.g0143.m0001	1.896271076	2.41E-05	0.00050676	DEG
Bpev01.c0154.g0024.m0001	1.328731677	1.59E-05	0.00036288	DEG
Bpev01.c0089.g0052.m0002	2.77053822	2.18E-11	2.76E-09	DEG
Bpev01.c0577.g0001.m0001	-0.921789631	0.00384338	0.03012386	DEG
Bpev01.c0455.g0032.m0001	-0.987571275	0.00444784	0.03357438	DEG
Bpev01.c0451.g0014.m0001	-1.443587766	0.00389798	0.03045204	DEG
Bpev01.c0145.g0007.m0001	-1.500445896	5.23E-06	0.00014215	DEG
Bpev01.c1426.g0006.m0001	3.330903389	3.10E-06	9.18E-05	DEG
Bpev01.c0462.g0004.m0001	1.032025144	0.00091323	0.00963951	DEG
Bpev01.c0219.g0021.m0001	-2.617801769	1.33E-10	1.47E-08	DEG
Bpev01.c1893.g0005.m0001	4.977319724	7.01E-13	1.24E-10	DEG
Bpev01.c0000.g0181.m0001	-1.404985824	0.00586795	0.04129967	DEG
Bpev01.c0129.g0067.m0001	-2.03878809	0.00060478	0.00697378	DEG
Bpev01.c1848.g0004.m0001	2.582648936	1.43E-07	6.67E-06	DEG
Bpev01.c1577.g0014.m0001	-3.99521812	0.00434638	0.03298613	DEG
Bpev01.c0044.g0099.m0001	-1.447826677	4.47E-05	0.00084325	DEG
Bpev01.c1782.g0001.m0001	-2.246611955	4.42E-10	4.31E-08	DEG
Bpev01.c1070.g0006.m0001	-1.543789803	0.0018228	0.0168482	DEG
Bpev01.c0426.g0011.m0001	1.944692013	0.00092592	0.0097411	DEG
Bpev01.c0467.g0019.m0001	-1.650419546	0.00286887	0.02402069	DEG
Bpev01.c0114.g0030.m0001	-1.092975423	0.00072863	0.00806393	DEG
Bpev01.c0166.g0015.m0001	4.035477924	0.00204462	0.01836233	DEG
Bpev01.c0045.g0037.m0001	1.565818058	1.14E-05	0.00027388	DEG
Bpev01.c0991.g0002.m0001	-1.623120091	4.01E-06	0.00011361	DEG
Bpev01.c0027.g0106.m0002	-0.86462516	0.00713718	0.0480035	DEG
Bpev01.c1115.g0011.m0001	-1.418232331	3.43E-05	0.00068012	DEG
Bpev01.c0027.g0171.m0001	0.96958792	0.00156166	0.01483019	DEG
Bpev01.c0024.g0044.m0001	3.46729464	1.01E-11	1.37E-09	DEG
Bpev01.c0566.g0024.m0001	-2.142208217	2.07E-06	6.51E-05	DEG
Bpev01.c0126.g0029.m0001	-2.300013141	3.63E-11	4.45E-09	DEG
Bpev01.c0122.g0005.m0001	2.97855928	5.84E-09	4.19E-07	DEG
Bpev01.c1447.g0002.m0001	-1.04328742	0.0010162	0.01044729	DEG
Bpev01.c1319.g0002.m0001	1.600430149	0.0042258	0.03226299	DEG
Bpev01.c0022.g0160.m0001	1.257439367	0.00018801	0.0027198	DEG

Bpev01.c0478.g0018.m0001	-1.223204269	0.00017147	0.00252267	DEG
Bpev01.c0001.g0041.m0001	-1.237914672	0.00021448	0.00300944	DEG
Bpev01.c0401.g0024.m0001	-1.443783016	0.00016684	0.00247057	DEG
Bpev01.c0096.g0012.m0001	-0.995267414	0.00323348	0.02639777	DEG
Bpev01.c0037.g0034.m0002	1.758756116	1.28E-06	4.38E-05	DEG
Bpev01.c0427.g0027.m0001	-2.615028309	1.55E-09	1.31E-07	DEG
Bpev01.c0517.g0009.m0001	1.6167548	0.00580984	0.04095685	DEG
Bpev01.c0364.g0007.m0001	-1.026171496	0.00074242	0.00818273	DEG
Bpev01.c0050.g0007.m0001	1.562130755	0.00144675	0.01392318	DEG
Bpev01.c1158.g0005.m0001	2.098566624	0.00056257	0.00657992	DEG
Bpev01.c0525.g0006.m0001	-1.24509448	0.00167128	0.01570408	DEG
Bpev01.c0094.g0206.m0001	-1.679334668	0.00535043	0.03861516	DEG
Bpev01.c1518.g0009.m0001	1.59022812	5.18E-06	0.0001415	DEG
Bpev01.c0455.g0002.m0001	1.399819925	0.00106209	0.01082647	DEG
Bpev01.c0615.g0006.m0001	1.552117564	0.00096844	0.01008807	DEG
Bpev01.c0283.g0010.m0001	-0.999106734	0.00265159	0.02256072	DEG
Bpev01.c1446.g0006.m0001	-1.079711622	0.0041819	0.03201575	DEG
Bpev01.c0301.g0007.m0001	1.179600284	0.00158726	0.01503935	DEG
Bpev01.c0005.g0002.m0001	-1.278436565	0.00020673	0.00292317	DEG
Bpev01.c0275.g0003.m0001	-0.903918153	0.00557694	0.03973367	DEG
Bpev01.c0889.g0003.m0001	-2.388642645	2.77E-12	4.30E-10	DEG
Bpev01.c1161.g0015.m0001	2.530560504	8.11E-11	9.37E-09	DEG
Bpev01.c0566.g0003.m0001	-1.803820362	4.47E-05	0.00084261	DEG
Bpev01.c0863.g0020.m0001	-2.612964371	0.00020176	0.00286112	DEG
Bpev01.c0365.g0049.m0001	-1.834641447	1.05E-07	5.01E-06	DEG
Bpev01.c0207.g0006.m0001	-2.072754684	0.00048284	0.00586403	DEG
Bpev01.c0878.g0011.m0001	2.652862128	1.00E-10	1.14E-08	DEG
Bpev01.c0045.g0074.m0001	1.4280034	6.03E-05	0.00107583	DEG
Bpev01.c0015.g0235.m0001	0.933245109	0.0046692	0.03485207	DEG
Bpev01.c0055.g0031.m0001	1.625402299	0.00075386	0.00828708	DEG
Bpev01.c1749.g0001.m0001	4.891822719	1.68E-17	8.09E-15	DEG
Bpev01.c1339.g0011.m0001	1.455284394	6.71E-06	0.00017599	DEG
Bpev01.c2411.g0002.m0001	1.14667414	0.00404557	0.03130861	DEG
Bpev01.c0480.g0061.m0001	-0.92228172	0.00463454	0.03466834	DEG
Bpev01.c0960.g0001.m0002	0.857258605	0.00606427	0.04228471	DEG
Bpev01.c0166.g0033.m0001	-1.457144183	0.00022169	0.00308674	DEG
Bpev01.c0191.g0001.m0001	-1.375430792	0.00215325	0.01912204	DEG
Bpev01.c0243.g0016.m0001	-1.601197935	3.08E-07	1.28E-05	DEG
Bpev01.c1525.g0004.m0001	-2.919025001	3.27E-06	9.62E-05	DEG
Bpev01.c0267.g0010.m0001	-2.286907298	0.00013483	0.00208363	DEG
Bpev01.c0616.g0011.m0001	-1.438303803	0.00481541	0.03568104	DEG
Bpev01.c0038.g0128.m0001	-0.878802706	0.00551174	0.03947933	DEG
Bpev01.c0420.g0012.m0001	-1.056506686	0.00049526	0.00599013	DEG

Bpev01.c0470.g0035.m0001	1.026753175	0.00148915	0.01426121	DEG
Bpev01.c0672.g0001.m0001	1.267001668	0.00085077	0.00915086	DEG
Bpev01.c0275.g0063.m0001	-0.97375927	0.00206677	0.01852169	DEG
Bpev01.c1560.g0001.m0001	2.884906143	3.95E-10	3.91E-08	DEG
Bpev01.c0428.g0016.m0001	1.242173029	8.59E-05	0.00143093	DEG
Bpev01.c0961.g0007.m0001	1.045195833	0.00089584	0.00950363	DEG
Bpev01.c0892.g0002.m0001	-1.135807248	0.0001989	0.0028301	DEG
Bpev01.c0437.g0004.m0001	2.365497852	2.86E-10	2.93E-08	DEG
Bpev01.c0968.g0003.m0001	1.299448112	0.00094786	0.00992402	DEG
Bpev01.c0522.g0007.m0001	0.90053167	0.00349923	0.02809212	DEG
Bpev01.c1592.g0005.m0002	1.30121228	0.00029967	0.00394563	DEG
Bpev01.c0135.g0099.m0001	-1.796389558	8.17E-09	5.67E-07	DEG
Bpev01.c0000.g0117.m0001	1.447305359	5.04E-06	0.00013814	DEG
Bpev01.c0022.g0120.m0001	1.362604148	0.00131709	0.01289624	DEG
Bpev01.c0423.g0002.m0001	2.150811738	5.10E-05	0.00093814	DEG
Bpev01.c0401.g0011.m0002	1.276107727	0.00382407	0.03002045	DEG
Bpev01.c0274.g0044.m0001	-1.157151261	0.00018748	0.00271345	DEG
Bpev01.c1193.g0007.m0001	-1.849429479	0.00554797	0.03963281	DEG
Bpev01.c1650.g0001.m0001	1.908709882	0.0001247	0.0019548	DEG
Bpev01.c3876.g0002.m0002	1.269452262	0.00429046	0.03263078	DEG
Bpev01.c0407.g0008.m0001	1.032261119	0.00553321	0.03956573	DEG
Bpev01.c2035.g0007.m0002	-1.406750043	6.91E-05	0.00119787	DEG
Bpev01.c0613.g0002.m0001	-1.045070816	0.00118446	0.01183209	DEG
Bpev01.c2499.g0008.m0001	-1.864332591	1.75E-05	0.00039219	DEG
Bpev01.c0191.g0020.m0001	-1.250177801	0.00050711	0.00609521	DEG
Bpev01.c0147.g0003.m0001	1.566092349	9.62E-06	0.0002383	DEG
Bpev01.c0222.g0039.m0001	1.984030248	0.00010065	0.00162365	DEG
Bpev01.c2152.g0006.m0001	-2.270963931	8.54E-05	0.0014252	DEG
Bpev01.c0420.g0028.m0001	-2.151561471	1.99E-05	0.00043508	DEG
Bpev01.c3330.g0001.m0001	4.261948033	1.83E-22	2.07E-19	DEG
Bpev01.c0304.g0031.m0001	4.374047644	2.85E-14	6.82E-12	DEG
Bpev01.c2077.g0003.m0001	-2.176525707	1.01E-11	1.37E-09	DEG
Bpev01.c0029.g0086.m0001	-2.052976002	0.00569175	0.04033688	DEG
Bpev01.c0115.g0026.m0001	2.652727944	8.79E-14	1.83E-11	DEG
Bpev01.c0299.g0012.m0001	1.252449863	0.00051493	0.00616818	DEG
Bpev01.c1063.g0004.m0001	1.7465964	1.34E-06	4.54E-05	DEG
Bpev01.c0018.g0093.m0001	-1.20558539	7.45E-05	0.00126978	DEG
Bpev01.c0279.g0038.m0001	-1.254596184	0.0005881	0.00682155	DEG
Bpev01.c0580.g0005.m0001	1.107270401	0.00355882	0.02844637	DEG
Bpev01.c0000.g0224.m0001	2.537084461	0.00152046	0.01450444	DEG
Bpev01.c0327.g0069.m0001	1.253729916	0.00051038	0.00612486	DEG
Bpev01.c1840.g0004.m0001	-1.194103093	0.00025484	0.00345726	DEG
Bpev01.c1229.g0012.m0001	-1.23142117	0.00062198	0.00713539	DEG

Bpev01.c1754.g0005.m0001	1.438278719	0.00011859	0.00186998	DEG
Bpev01.c0044.g0076.m0001	-0.977729146	0.00548624	0.03933707	DEG
Bpev01.c0094.g0038.m0001	1.442042506	0.00729811	0.04876283	DEG
Bpev01.c0214.g0032.m0001	-1.336152491	2.64E-05	0.00054733	DEG
Bpev01.c0484.g0005.m0001	2.063466271	9.74E-07	3.51E-05	DEG
Bpev01.c1077.g0004.m0001	-2.002690118	1.03E-08	6.95E-07	DEG
Bpev01.c0257.g0005.m0001	1.150790527	0.00061946	0.00711238	DEG
Bpev01.c0094.g0261.m0001	-2.156357479	3.90E-05	0.00075315	DEG
Bpev01.c0352.g0004.m0001	-1.172295752	0.00026926	0.00362122	DEG
Bpev01.c1476.g0006.m0001	1.850432381	1.56E-05	0.00035753	DEG
Bpev01.c1908.g0004.m0001	-0.998098091	0.00190804	0.01745515	DEG
Bpev01.c1272.g0015.m0001	-1.179249953	0.00015936	0.00237759	DEG
Bpev01.c0630.g0019.m0001	1.982213092	0.00014159	0.00216189	DEG
Bpev01.c0489.g0002.m0001	1.492165827	0.00114116	0.01149584	DEG
Bpev01.c0675.g0004.m0001	-1.712164828	1.71E-05	0.00038557	DEG
Bpev01.c2549.g0001.m0001	-1.648665321	0.00018993	0.0027408	DEG
Bpev01.c1798.g0004.m0001	1.255297289	3.37E-05	0.00067132	DEG
Bpev01.c0373.g0007.m0001	-2.634206339	7.77E-09	5.43E-07	DEG
Bpev01.c0016.g0119.m0001	2.844058482	2.60E-07	1.10E-05	DEG
Bpev01.c2168.g0003.m0001	1.738997674	0.00597051	0.04180918	DEG
Bpev01.c0533.g0012.m0001	3.950714182	3.11E-05	0.00062685	DEG
Bpev01.c0726.g0007.m0001	1.357914282	0.00566839	0.04021003	DEG
Bpev01.c0407.g0006.m0001	3.69757894	0.00299003	0.02479532	DEG
Bpev01.c2000.g0002.m0001	-2.904103944	4.35E-12	6.40E-10	DEG
Bpev01.c0090.g0017.m0001	-1.183920381	0.00146505	0.01406703	DEG
Bpev01.c1786.g0017.m0001	-0.997931897	0.00108938	0.01105495	DEG
Bpev01.c0552.g0009.m0001	-1.058304899	0.00141219	0.01362172	DEG
Bpev01.c1936.g0010.m0001	-0.875971594	0.00508856	0.03711651	DEG
Bpev01.c0115.g0042.m0001	-2.289660226	0.00142875	0.01376343	DEG
Bpev01.c0381.g0004.m0001	1.612781786	0.00011382	0.00180826	DEG
Bpev01.c0429.g0024.m0001	-1.023211774	0.00260563	0.02227913	DEG
Bpev01.c0223.g0009.m0001	-1.079402459	0.00264749	0.02253237	DEG
Bpev01.c1709.g0006.m0001	-1.281893393	0.00711303	0.04787391	DEG
Bpev01.c0149.g0012.m0001	-2.457904722	0.00254345	0.02182995	DEG
Bpev01.c0042.g0029.m0001	-1.558436351	1.41E-06	4.73E-05	DEG
Bpev01.c2033.g0004.m0001	2.357459992	0.00229265	0.02009928	DEG
Bpev01.c1537.g0001.m0001	-8.433028091	8.31E-07	3.06E-05	DEG
Bpev01.c0490.g0001.m0001	3.08463977	0.00020111	0.00285324	DEG
Bpev01.c0522.g0008.m0001	3.355280283	4.03E-16	1.41E-13	DEG
Bpev01.c0001.g0096.m0001	-1.272362498	0.00051547	0.0061687	DEG
Bpev01.c1854.g0005.m0001	2.459440371	1.52E-05	0.00035065	DEG
Bpev01.c0092.g0016.m0001	1.228401245	6.15E-05	0.00108864	DEG
Bpev01.c0597.g0001.m0001	2.021110082	3.35E-09	2.56E-07	DEG

Bpev01.c0480.g0085.m0001	-1.134541364	0.00053704	0.00636731	DEG
Bpev01.c0192.g0004.m0001	-0.949557719	0.00311972	0.02568992	DEG
Bpev01.c2604.g0001.m0001	1.495409038	0.00015104	0.0022761	DEG
Bpev01.c2165.g0008.m0001	-1.936237129	0.00065731	0.0074626	DEG
Bpev01.c0879.g0017.m0001	-1.040250698	0.00594089	0.04163149	DEG
Bpev01.c0042.g0004.m0001	1.110637099	0.00440483	0.03332656	DEG
Bpev01.c5230.g0001.m0001	1.523547685	0.00150929	0.01441661	DEG
Bpev01.c1335.g0008.m0001	-0.932625916	0.005434	0.03908415	DEG
Bpev01.c0118.g0043.m0001	-2.036511189	2.37E-06	7.29E-05	DEG
Bpev01.c0062.g0003.m0001	-1.88156812	8.46E-08	4.23E-06	DEG
Bpev01.c0127.g0060.m0001	-2.580137744	7.22E-13	1.26E-10	DEG
Bpev01.c0052.g0031.m0002	1.686653795	0.00039503	0.00496972	DEG
Bpev01.c0842.g0017.m0002	-0.939823251	0.00503586	0.03682331	DEG
Bpev01.c0024.g0003.m0001	4.921937495	4.84E-07	1.89E-05	DEG
Bpev01.c1260.g0017.m0001	-0.922429489	0.00631912	0.04373026	DEG
Bpev01.c0827.g0011.m0001	-1.194087726	0.00016403	0.00243148	DEG
Bpev01.c0164.g0012.m0001	-1.286312642	0.00019115	0.00275308	DEG
Bpev01.c2114.g0002.m0001	1.595679878	0.00048748	0.00591302	DEG
Bpev01.c0478.g0015.m0001	1.032215912	0.0021183	0.01885717	DEG
Bpev01.c0029.g0113.m0001	-2.262648734	0.00081693	0.00883523	DEG
Bpev01.c1402.g0003.m0001	1.347574917	0.00145746	0.01400333	DEG
Bpev01.c0074.g0021.m0001	2.139889152	5.51E-07	2.13E-05	DEG
Bpev01.c1272.g0013.m0001	-2.076504164	1.26E-07	5.95E-06	DEG
Bpev01.c2499.g0005.m0001	2.147939703	1.39E-06	4.70E-05	DEG
Bpev01.c0479.g0008.m0001	-0.904540888	0.00335229	0.02715551	DEG
Bpev01.c0328.g0028.m0001	3.518778054	2.17E-07	9.48E-06	DEG
Bpev01.c0052.g0010.m0001	-1.550531296	5.19E-06	0.00014164	DEG
Bpev01.c0636.g0002.m0001	-1.328145882	0.00109757	0.01112045	DEG
Bpev01.c0511.g0002.m0001	1.450258683	3.21E-05	0.00064323	DEG
Bpev01.c0818.g0012.m0001	-1.182469204	0.00095106	0.00994615	DEG
Bpev01.c0146.g0002.m0001	-1.686147712	0.00474518	0.03530284	DEG
Bpev01.c1082.g0001.m0001	-0.843239902	0.00566032	0.04017216	DEG
Bpev01.c1854.g0001.m0001	2.869324116	2.16E-06	6.73E-05	DEG
Bpev01.c0114.g0064.m0002	3.25568115	3.12E-05	0.00062762	DEG
Bpev01.c0050.g0124.m0001	-0.952930493	0.0023857	0.02071148	DEG
Bpev01.c0161.g0096.m0001	2.343566596	1.42E-09	1.21E-07	DEG
Bpev01.c1527.g0011.m0001	-1.269199946	0.00014706	0.00222533	DEG
Bpev01.c0214.g0037.m0001	-1.84958898	0.00125249	0.01236483	DEG
Bpev01.c2099.g0001.m0001	3.600976152	0.00092239	0.00971875	DEG
Bpev01.c1104.g0025.m0001	1.914358349	0.00493012	0.03629464	DEG
Bpev01.c2819.g0002.m0001	-1.29904726	3.91E-05	0.00075393	DEG
Bpev01.c0022.g0134.m0001	1.031008039	0.0039873	0.03097969	DEG
Bpev01.c0230.g0002.m0001	-1.768583961	2.94E-08	1.70E-06	DEG

Bpev01.c0051.g0185.m0003	-1.502423341	1.30E-06	4.45E-05	DEG
Bpev01.c0223.g0018.m0001	1.22741139	0.00057108	0.00665151	DEG
Bpev01.c0018.g0083.m0001	-0.943916083	0.00546146	0.03923378	DEG
Bpev01.c1238.g0013.m0003	-1.152465646	0.00049636	0.00600092	DEG
Bpev01.c0757.g0003.m0001	-2.710917699	1.75E-07	7.89E-06	DEG
Bpev01.c1112.g0013.m0001	-3.674625753	0.00090145	0.009546	DEG
Bpev01.c1393.g0005.m0001	0.913790873	0.00715269	0.0480859	DEG
Bpev01.c0957.g0008.m0001	-1.515694704	0.0057576	0.04062754	DEG
Bpev01.c0516.g0011.m0001	-1.466572013	4.50E-05	0.00084749	DEG
Bpev01.c0427.g0018.m0001	-2.559059409	6.24E-06	0.000165	DEG
Bpev01.c4564.g0001.m0001	-1.54294706	0.00216065	0.01918198	DEG
Bpev01.c0557.g0042.m0001	5.532497045	8.27E-25	1.11E-21	DEG
Bpev01.c0716.g0010.m0001	1.7706246	0.00060688	0.0069925	DEG
Bpev01.c0344.g0028.m0001	-1.304439368	5.31E-05	0.00097034	DEG
Bpev01.c0245.g0076.m0001	0.860058929	0.00620444	0.04309875	DEG
Bpev01.c1077.g0001.m0001	-1.712305636	8.69E-08	4.31E-06	DEG
Bpev01.c2085.g0004.m0001	1.036313669	0.00161661	0.01527324	DEG
Bpev01.c0506.g0024.m0002	-1.582492162	0.00024386	0.00334055	DEG
Bpev01.c0226.g0001.m0001	-1.785973689	0.00185526	0.01709105	DEG
Bpev01.c0279.g0032.m0001	1.503559811	2.27E-06	7.03E-05	DEG
Bpev01.c0022.g0135.m0001	3.38477022	1.20E-08	7.87E-07	DEG
Bpev01.c0052.g0180.m0001	1.84466276	0.00134982	0.01314934	DEG
Bpev01.c0194.g0001.m0001	2.927919607	0.00592493	0.04157905	DEG
Bpev01.c0397.g0002.m0001	-1.064277827	0.00305045	0.0252107	DEG
Bpev01.c0805.g0001.m0001	-1.874477621	0.00275554	0.02328365	DEG
Bpev01.c0121.g0049.m0001	1.687853122	0.00382528	0.03002197	DEG
Bpev01.c0471.g0005.m0001	-2.647437979	0.00013691	0.00210803	DEG
Bpev01.c0557.g0020.m0001	-1.012805461	0.00368621	0.0292266	DEG
Bpev01.c0613.g0017.m0001	-1.343082403	3.10E-05	0.0006267	DEG
Bpev01.c0022.g0128.m0001	-1.05756328	0.00167249	0.01571045	DEG
Bpev01.c2316.g0001.m0001	2.241709666	0.00055088	0.00648313	DEG
Bpev01.c0640.g0014.m0001	-1.560784973	2.24E-06	6.97E-05	DEG
Bpev01.c0192.g0009.m0001	-1.002199991	0.00115258	0.01157077	DEG
Bpev01.c2452.g0001.m0001	-1.255458281	0.00720722	0.04832561	DEG
Bpev01.c0050.g0052.m0001	-1.372285975	1.69E-05	0.0003826	DEG
Bpev01.c0001.g0020.m0001	2.172798226	2.44E-07	1.05E-05	DEG
Bpev01.c0344.g0008.m0001	1.038149058	0.00092886	0.00975902	DEG
Bpev01.c0622.g0015.m0001	1.154120086	0.00038639	0.00488407	DEG
Bpev01.c1479.g0009.m0001	-1.680644142	2.79E-07	1.18E-05	DEG
Bpev01.c1250.g0007.m0001	-2.166254137	1.60E-08	1.00E-06	DEG
Bpev01.c0263.g0001.m0001	0.976959131	0.00523914	0.03800113	DEG
Bpev01.c0634.g0012.m0001	-2.70738964	0.00010037	0.00162168	DEG
Bpev01.c0001.g0066.m0001	0.952609397	0.00159622	0.01511943	DEG

Bpev01.c0581.g0013.m0001	-1.163695153	0.0004064	0.00507813	DEG
Bpev01.c0088.g0120.m0001	2.165872972	9.07E-07	3.29E-05	DEG
Bpev01.c0129.g0008.m0001	-1.457070081	6.73E-05	0.00117361	DEG
Bpev01.c0399.g0006.m0001	-2.364670872	0.00105414	0.01076038	DEG
Bpev01.c2499.g0004.m0001	2.447160981	8.29E-05	0.00139362	DEG
Bpev01.c0381.g0014.m0001	1.292816414	0.0001202	0.00189222	DEG
Bpev01.c0096.g0007.m0001	1.42477625	1.84E-05	0.00040881	DEG
Bpev01.c1140.g0002.m0001	-1.338076444	0.00169016	0.0158259	DEG
Bpev01.c1378.g0027.m0001	-2.021098423	7.24E-06	0.00018793	DEG
Bpev01.c0237.g0020.m0001	-1.673665734	0.00518789	0.03770086	DEG
Bpev01.c0389.g0018.m0001	-1.626469038	0.00258896	0.02214302	DEG
Bpev01.c0411.g0004.m0001	-3.182051208	2.38E-13	4.64E-11	DEG
Bpev01.c1154.g0004.m0001	-0.996104294	0.00404558	0.03130861	DEG
Bpev01.c0488.g0002.m0001	1.262631916	0.0040328	0.03125073	DEG
Bpev01.c0022.g0014.m0001	-1.348473933	0.00021149	0.00297332	DEG
Bpev01.c0518.g0047.m0001	-1.407093559	0.00438408	0.0331892	DEG
Bpev01.c0052.g0192.m0001	-1.494953574	3.60E-05	0.00070548	DEG
Bpev01.c0091.g0028.m0001	-2.114461381	0.00021888	0.00305961	DEG
Bpev01.c1354.g0002.m0001	1.292733057	0.00078385	0.00853157	DEG
Bpev01.c2170.g0002.m0001	2.112868981	0.00046906	0.00571318	DEG
Bpev01.c0063.g0031.m0001	3.992234328	1.45E-07	6.75E-06	DEG
Bpev01.c1530.g0006.m0001	2.406860209	3.72E-09	2.79E-07	DEG
Bpev01.c1547.g0004.m0001	-1.761304636	0.00478874	0.03554607	DEG
Bpev01.c0051.g0110.m0001	1.600369018	0.00507418	0.03703883	DEG
Bpev01.c0276.g0023.m0001	4.033252443	2.17E-09	1.75E-07	DEG
Bpev01.c0025.g0001.m0001	-1.239663273	0.00145716	0.01400333	DEG
Bpev01.c0959.g0006.m0001	-1.37363635	3.04E-05	0.00061634	DEG
Bpev01.c0161.g0019.m0001	-1.198826502	0.00163988	0.01545343	DEG
Bpev01.c0363.g0001.m0001	1.278408187	0.00063325	0.00723411	DEG
Bpev01.c0307.g0004.m0001	-1.081152521	0.00491051	0.03619438	DEG
Bpev01.c0415.g0018.m0002	1.378703253	0.00017221	0.00253231	DEG
Bpev01.c3115.g0001.m0001	-3.08699978	9.06E-05	0.00149352	DEG
Bpev01.c2062.g0002.m0001	-2.385822976	1.20E-09	1.05E-07	DEG
Bpev01.c0155.g0012.m0001	-1.316130944	0.00586355	0.0412861	DEG
Bpev01.c0154.g0002.m0001	-2.830981746	0.00015932	0.00237759	DEG
Bpev01.c1602.g0006.m0001	-1.02421895	0.00344975	0.02779345	DEG
Bpev01.c3211.g0002.m0001	-1.314901663	6.74E-05	0.00117531	DEG
Bpev01.c1061.g0002.m0001	1.648289524	0.00493965	0.03631828	DEG
Bpev01.c1378.g0033.m0001	1.99326328	1.60E-06	5.24E-05	DEG
Bpev01.c0458.g0013.m0001	0.835694761	0.00562412	0.03996773	DEG
Bpev01.c0793.g0003.m0001	-1.512317464	1.24E-06	4.28E-05	DEG
Bpev01.c2784.g0002.m0001	-1.401440756	0.00287493	0.02405896	DEG
Bpev01.c1378.g0021.m0001	-1.095969691	0.00177631	0.01647937	DEG

Bpev01.c0584.g0009.m0001	1.483383417	2.43E-05	0.0005102	DEG
Bpev01.c1971.g0001.m0001	-6.162083326	0.0069741	0.04713942	DEG
Bpev01.c1519.g0004.m0001	-2.33075643	4.63E-08	2.54E-06	DEG
Bpev01.c0027.g0080.m0001	-1.743112782	0.00018826	0.0027221	DEG
Bpev01.c1349.g0014.m0001	1.645832709	1.66E-05	0.00037758	DEG
Bpev01.c1355.g0003.m0001	3.215832758	3.69E-06	0.00010651	DEG
Bpev01.c0581.g0014.m0001	-1.352815618	0.00510686	0.03721312	DEG
Bpev01.c0123.g0001.m0001	4.194470018	3.14E-19	2.10E-16	DEG
Bpev01.c0027.g0028.m0001	-2.696570477	0.00079962	0.00866716	DEG
Bpev01.c0149.g0021.m0001	1.06029211	0.00751103	0.04981241	DEG
Bpev01.c0261.g0009.m0001	-1.37599065	0.00317723	0.02605415	DEG
Bpev01.c0469.g0004.m0001	-2.004256857	3.49E-10	3.52E-08	DEG
Bpev01.c0050.g0046.m0001	-1.260057837	0.00089327	0.00949461	DEG
Bpev01.c0333.g0020.m0001	-1.666287743	2.77E-06	8.37E-05	DEG
Bpev01.c1753.g0003.m0001	-2.295081577	3.54E-06	0.00010291	DEG
Bpev01.c0489.g0005.m0001	2.208392656	2.82E-05	0.00057849	DEG
Bpev01.c2812.g0002.m0001	4.709609934	0.00261871	0.02236426	DEG
Bpev01.c0518.g0028.m0001	-1.400551138	0.00028503	0.00379169	DEG
Bpev01.c1259.g0015.m0001	6.485181311	5.41E-05	0.00098332	DEG
Bpev01.c0129.g0004.m0001	1.442966831	0.00010219	0.00164397	DEG
Bpev01.c1961.g0002.m0001	-2.842189361	9.06E-09	6.20E-07	DEG
Bpev01.c0088.g0062.m0001	-1.520245226	2.96E-05	0.00060314	DEG
Bpev01.c0621.g0021.m0001	-0.916780715	0.00398935	0.03098743	DEG
Bpev01.c0053.g0015.m0001	1.777612899	1.06E-05	0.00025792	DEG
Bpev01.c1070.g0023.m0001	0.893040791	0.00434926	0.03298811	DEG
Bpev01.c1908.g0005.m0001	1.600364158	7.07E-05	0.00121798	DEG
Bpev01.c0364.g0004.m0001	-2.132975166	0.00750975	0.04981241	DEG
Bpev01.c1062.g0010.m0001	-1.433401966	0.0045472	0.0341144	DEG
Bpev01.c1456.g0018.m0001	-1.557589448	0.00445263	0.03357605	DEG
Bpev01.c0091.g0005.m0001	-1.402826839	0.00022482	0.00312194	DEG
Bpev01.c0351.g0005.m0001	2.294705176	0.00052474	0.00625665	DEG
Bpev01.c0066.g0069.m0001	1.12447953	0.000283	0.0037715	DEG
Bpev01.c0979.g0006.m0001	-1.126986712	0.00101673	0.01044729	DEG
Bpev01.c0456.g0001.m0001	1.706642789	0.00609993	0.04245289	DEG
Bpev01.c1856.g0002.m0001	1.290631144	0.00229725	0.02013359	DEG
Bpev01.c2135.g0001.m0001	-1.183853234	0.00021827	0.00305256	DEG
Bpev01.c0951.g0004.m0001	-4.121146214	2.17E-14	5.27E-12	DEG
Bpev01.c0395.g0010.m0001	-2.516920594	6.50E-06	0.00017058	DEG
Bpev01.c0592.g0007.m0001	-2.397524459	2.24E-05	0.00047734	DEG
Bpev01.c0995.g0001.m0001	2.299894664	6.03E-05	0.00107583	DEG
Bpev01.c0398.g0004.m0001	-1.51196197	2.11E-05	0.00045496	DEG
Bpev01.c0466.g0002.m0001	1.197325901	0.00321235	0.02626712	DEG
Bpev01.c2594.g0003.m0001	-0.989474756	0.00329974	0.02681236	DEG

Bpev01.c0029.g0135.m0001	1.582763727	0.0017437	0.01623941	DEG
Bpev01.c0444.g0029.m0001	-1.513402425	3.03E-06	9.02E-05	DEG
Bpev01.c1637.g0001.m0001	-2.360379719	0.00392132	0.03058695	DEG
Bpev01.c0154.g0058.m0001	1.370175887	0.00275454	0.02328188	DEG
Bpev01.c0261.g0050.m0001	-0.998031	0.00312902	0.02572349	DEG
Bpev01.c0091.g0053.m0001	0.810807435	0.00747889	0.04964443	DEG
Bpev01.c1814.g0003.m0001	1.675081796	0.00319307	0.0261331	DEG
Bpev01.c2165.g0002.m0001	2.785530251	0.00024548	0.00335039	DEG
Bpev01.c0038.g0029.m0001	4.537370236	2.80E-13	5.40E-11	DEG
Bpev01.c0574.g0016.m0001	1.911729233	2.54E-08	1.51E-06	DEG
Bpev01.c0771.g0013.m0001	1.001599978	0.00479472	0.03557251	DEG
Bpev01.c1474.g0001.m0001	2.716271424	0.00104499	0.01068179	DEG
Bpev01.c0349.g0019.m0001	2.270870454	1.30E-11	1.75E-09	DEG
Bpev01.c0213.g0019.m0001	1.298358806	0.00730867	0.04882228	DEG
Bpev01.c0470.g0050.m0001	-2.255036456	0.00165375	0.01556916	DEG
Bpev01.c0196.g0007.m0001	0.888839651	0.00457722	0.03431338	DEG
Bpev01.c0668.g0010.m0001	-3.208487117	9.42E-05	0.00154463	DEG
Bpev01.c0358.g0006.m0001	-0.938098255	0.00273254	0.02314252	DEG
Bpev01.c1998.g0002.m0001	3.43224634	0.00090811	0.00959928	DEG
Bpev01.c0672.g0009.m0001	0.905604552	0.00369137	0.02923166	DEG
Bpev01.c1227.g0004.m0001	2.336452284	0.00499006	0.03658321	DEG
Bpev01.c0145.g0064.m0001	-2.141193242	0.00082476	0.00890215	DEG
Bpev01.c1562.g0003.m0002	1.462916074	0.00248004	0.02139642	DEG
Bpev01.c0016.g0066.m0001	-2.753428093	2.65E-06	8.03E-05	DEG
Bpev01.c0268.g0017.m0001	-0.939067442	0.00552813	0.03954863	DEG
Bpev01.c1108.g0012.m0001	1.75121432	3.64E-06	0.00010557	DEG
Bpev01.c0411.g0017.m0001	-3.026317589	0.00071646	0.00798334	DEG
Bpev01.c0278.g0006.m0001	1.23753003	0.00583621	0.0411231	DEG
Bpev01.c0062.g0021.m0001	3.13218849	1.31E-21	1.17E-18	DEG
Bpev01.c0522.g0033.m0001	-1.837740096	0.00039609	0.00497886	DEG
Bpev01.c0188.g0031.m0001	4.006274927	2.09E-05	0.00045083	DEG
Bpev01.c5346.g0001.m0001	5.630906721	0.0001571	0.0023524	DEG
Bpev01.c2030.g0002.m0001	-1.58846479	0.00327601	0.02667843	DEG
Bpev01.c0384.g0010.m0001	2.088468432	2.05E-05	0.00044417	DEG
Bpev01.c2321.g0001.m0001	2.234757039	0.00028182	0.00376082	DEG
Bpev01.c1063.g0013.m0001	-1.653902098	0.0002105	0.0029661	DEG
Bpev01.c0322.g0003.m0001	1.129866928	0.00586167	0.04128274	DEG
Bpev01.c0281.g0046.m0001	-1.678699665	3.72E-06	0.0001069	DEG
Bpev01.c0478.g0006.m0001	-2.243879923	5.30E-07	2.06E-05	DEG
Bpev01.c0606.g0017.m0001	3.158004934	0.00014322	0.00218237	DEG
Bpev01.c1110.g0002.m0001	-2.309440264	0.0015324	0.01459695	DEG
Bpev01.c0142.g0051.m0001	1.467048028	5.00E-05	0.00092552	DEG
Bpev01.c3912.g0001.m0001	1.450526494	0.00010802	0.00172834	DEG

Bpev01.c0094.g0028.m0001	-3.296222806	0.00594056	0.04163149	DEG
Bpev01.c1161.g0014.m0001	2.888457812	4.74E-14	1.07E-11	DEG
Bpev01.c0121.g0043.m0001	3.257949247	0.00304388	0.02516394	DEG
Bpev01.c1189.g0012.m0001	-1.239627598	0.0002201	0.00307086	DEG
Bpev01.c0092.g0003.m0001	1.337984747	0.00090192	0.00954754	DEG
Bpev01.c0658.g0014.m0001	-1.131517547	0.00210469	0.01878713	DEG
Bpev01.c0031.g0016.m0001	4.084744647	5.72E-08	3.03E-06	DEG
Bpev01.c0017.g0018.m0001	-1.751537403	0.00088538	0.00944036	DEG
Bpev01.c1517.g0007.m0001	-3.418616501	7.05E-05	0.00121578	DEG
Bpev01.c0101.g0037.m0001	4.727544759	5.35E-09	3.88E-07	DEG
Bpev01.c1588.g0002.m0001	1.884290915	9.46E-06	0.00023511	DEG
Bpev01.c0580.g0002.m0001	-3.842000213	2.61E-12	4.09E-10	DEG
Bpev01.c0420.g0018.m0001	-2.305800984	2.25E-05	0.00047892	DEG
Bpev01.c0023.g0048.m0001	4.299127064	0.00367371	0.02916558	DEG
Bpev01.c0826.g0006.m0001	-2.179756468	3.91E-05	0.00075393	DEG
Bpev01.c0114.g0022.m0001	-1.162443959	0.00040221	0.00503654	DEG
Bpev01.c0115.g0061.m0001	-2.916174145	0.00516285	0.03755598	DEG
Bpev01.c1897.g0004.m0001	-2.325420379	0.00301397	0.02495173	DEG
Bpev01.c1093.g0013.m0001	2.201131557	8.56E-10	7.85E-08	DEG
Bpev01.c0645.g0033.m0001	-1.723006817	0.0018426	0.01700452	DEG
Bpev01.c0953.g0001.m0001	3.301795712	2.21E-09	1.78E-07	DEG
Bpev01.c0442.g0027.m0001	-0.902456753	0.00334148	0.02708423	DEG
Bpev01.c0622.g0007.m0001	-0.67148032	0.47221602	0.80076738	nonDEG
Bpev01.c2193.g0003.m0001	-0.764665206	0.0163085	0.08892496	nonDEG
Bpev01.c1413.g0004.m0001	-0.726869142	0.03361021	0.1506243	nonDEG
Bpev01.c1127.g0001.m0001	-0.085463127	0.85005032	1	nonDEG
Bpev01.c0080.g0027.m0003	-0.56930699	0.07418229	0.26292469	nonDEG
Bpev01.c0449.g0002.m0001	-0.870420497	0.008977	0.0569924	nonDEG
Bpev01.c0278.g0041.m0001	-1.006192904	0.01311635	0.07569735	nonDEG
Bpev01.c2419.g0001.m0001	0.415716655	0.30048346	0.63135627	nonDEG
Bpev01.c0292.g0030.m0001	-0.954321539	0.0598632	0.2269857	nonDEG
Bpev01.c0171.g0015.m0001	-0.577352513	0.0985535	0.31723331	nonDEG
Bpev01.c1100.g0005.m0001	-0.276027521	0.42144011	0.75288987	nonDEG
Bpev01.c0044.g0017.m0001	-0.674838147	0.02721863	0.12930548	nonDEG
Bpev01.c0425.g0055.m0001	-0.766810853	0.58812722	0.89802267	nonDEG
Bpev01.c0387.g0007.m0001	-0.505691086	0.1627046	0.44125846	nonDEG
Bpev01.c0142.g0047.m0001	-5.89647772	0.01750709	0.09348651	nonDEG
Bpev01.c0189.g0075.m0001	-0.282586067	0.35806299	0.69626189	nonDEG
Bpev01.c0233.g0001.m0001	-1.239267083	0.13001859	0.38241754	nonDEG
Bpev01.c0443.g0018.m0001	-0.484098291	0.1155915	0.35369179	nonDEG
Bpev01.c0123.g0003.m0001	-0.691750202	0.02778972	0.13135865	nonDEG
Bpev01.c0089.g0081.m0001	-0.233389433	0.50886696	0.82991932	nonDEG
Bpev01.c1085.g0007.m0001	-0.518026306	0.1547769	0.42755721	nonDEG

Bpev01.c0134.g0028.m0001	-0.537559471	0.37962496	0.71647215	nonDEG
Bpev01.c1239.g0004.m0001	-0.742957342	0.07716283	0.26962571	nonDEG
Bpev01.c1044.g0003.m0001	-0.814010184	0.11446937	0.3511738	nonDEG
Bpev01.c1172.g0004.m0001	0.489118583	0.38591642	0.71914078	nonDEG
Bpev01.c0190.g0074.m0001	-0.808733279	0.0240443	0.11836793	nonDEG
Bpev01.c0863.g0023.m0001	0.052361038	0.87057207	1	nonDEG
Bpev01.c0394.g0003.m0001	0.41093231	0.31071453	0.64112138	nonDEG
Bpev01.c1411.g0003.m0001	-0.758287671	0.07028852	0.25348815	nonDEG
Bpev01.c0094.g0136.m0001	-0.136668702	0.70812976	0.96218186	nonDEG
Bpev01.c0425.g0001.m0001	-1.169946779	0.01406042	0.07957457	nonDEG
Bpev01.c2535.g0002.m0001	-0.277635019	0.65107523	0.93220195	nonDEG
Bpev01.c0312.g0022.m0001	-0.58118947	0.05227403	0.20684231	nonDEG
Bpev01.c0510.g0010.m0001	-0.664170527	0.08363202	0.28436624	nonDEG
Bpev01.c1968.g0005.m0001	-0.818236781	0.02322795	0.11523553	nonDEG
Bpev01.c0044.g0087.m0001	0.306888549	0.3613712	0.6995008	nonDEG
Bpev01.c0645.g0019.m0001	0.896179984	0.01667823	0.0904551	nonDEG
Bpev01.c0862.g0021.m0001	1.408426962	0.01701924	0.09166291	nonDEG
Bpev01.c0332.g0009.m0002	0.924180009	0.00792032	0.05186086	nonDEG
Bpev01.c1561.g0011.m0001	1.169407996	0.14665372	0.41318203	nonDEG
Bpev01.c0415.g0040.m0001	-0.853130913	0.01134333	0.06790022	nonDEG
Bpev01.c0402.g0030.m0001	-0.825820092	0.00945208	0.05912874	nonDEG
Bpev01.c0018.g0047.m0001	0.361885888	0.3318194	0.66741975	nonDEG
Bpev01.c0606.g0019.m0001	1.880738215	0.08459961	0.28643221	nonDEG
Bpev01.c0094.g0284.m0001	-0.011798615	0.98931035	1	nonDEG
Bpev01.c2625.g0003.m0001	-0.303383155	0.66226225	0.93220195	nonDEG
Bpev01.c0094.g0185.m0001	-0.974834917	0.04498168	0.1860376	nonDEG
Bpev01.c0333.g0009.m0001	-0.244741306	0.58168637	0.89235358	nonDEG
Bpev01.c0022.g0044.m0001	-0.303718855	0.36998091	0.70970893	nonDEG
Bpev01.c0022.g0002.m0002	-0.675181901	0.05617605	0.21731494	nonDEG
Bpev01.c0015.g0039.m0001	-0.834155913	0.01573718	0.08657951	nonDEG
Bpev01.c0430.g0005.m0001	-0.17614106	0.74314337	0.98399997	nonDEG
Bpev01.c1291.g0001.m0001	-0.43240161	0.30384899	0.63584095	nonDEG
Bpev01.c2330.g0012.m0001	1.154591094	0.02188516	0.11022709	nonDEG
Bpev01.c0452.g0003.m0001	-4.070116963	0.37985749	0.71647215	nonDEG
Bpev01.c0430.g0002.m0001	-0.916610666	0.07699302	0.26941608	nonDEG
Bpev01.c0170.g0049.m0001	-0.248738071	0.43055738	0.76219432	nonDEG
Bpev01.c0671.g0004.m0001	0.85856142	0.35037951	0.68805044	nonDEG
Bpev01.c0095.g0031.m0001	2.419077323	0.05191821	0.2058377	nonDEG
Bpev01.c0223.g0048.m0001	-0.900607453	0.01566937	0.08628934	nonDEG
Bpev01.c0940.g0002.m0001	-0.481679016	0.1451998	0.41059004	nonDEG
Bpev01.c0266.g0001.m0001	-1.371411359	0.01031291	0.06330313	nonDEG
Bpev01.c0356.g0038.m0001	-0.483686238	0.67787042	0.93994337	nonDEG
Bpev01.c0396.g0015.m0001	-0.113161127	0.75621928	0.99113216	nonDEG

Bpev01.c1272.g0004.m0001	-0.782485543	0.0266655	0.12737395	nonDEG
Bpev01.c0066.g0048.m0001	0.062588505	0.87592416	1	nonDEG
Bpev01.c0015.g0079.m0001	-1.095658534	0.02540285	0.1232294	nonDEG
Bpev01.c1529.g0009.m0001	-0.38902896	0.48750883	0.81424325	nonDEG
Bpev01.c0261.g0060.m0001	0.005019819	0.99306136	1	nonDEG
Bpev01.c0415.g0040.m0002	-0.850148722	0.01159074	0.06890544	nonDEG
Bpev01.c1041.g0005.m0001	-0.95668917	0.01406117	0.07957457	nonDEG
Bpev01.c0161.g0025.m0001	-0.688200855	0.02341087	0.11594762	nonDEG
Bpev01.c0496.g0021.m0001	0.352856338	0.35319943	0.6912254	nonDEG
Bpev01.c0220.g0004.m0001	0.079813523	0.79123498	1	nonDEG
Bpev01.c0478.g0012.m0001	-0.922936676	0.01336258	0.07674229	nonDEG
Bpev01.c0025.g0017.m0001	0.632329364	0.34333696	0.680152	nonDEG
Bpev01.c1111.g0005.m0001	2.276903613	0.03000094	0.13874067	nonDEG
Bpev01.c0572.g0001.m0001	-3.006416668	0.12401006	0.37150957	nonDEG
Bpev01.c0210.g0018.m0001	-1.224789363	0.00764063	0.05043327	nonDEG
Bpev01.c0403.g0023.m0002	0.812914177	0.02023361	0.10368631	nonDEG
Bpev01.c0015.g0183.m0001	-0.828231286	0.01190896	0.07032001	nonDEG
Bpev01.c0038.g0093.m0001	-0.702850993	0.02902758	0.13557716	nonDEG
Bpev01.c0699.g0016.m0001	0.173322044	0.62858531	0.92606861	nonDEG
Bpev01.c0168.g0011.m0001	-0.25599946	0.66545473	0.93220195	nonDEG
Bpev01.c0080.g0113.m0001	0.160894012	0.81790657	1	nonDEG
Bpev01.c0274.g0018.m0001	0.814238872	0.01999622	0.10282422	nonDEG
Bpev01.c0606.g0018.m0001	0	1	1	nonDEG
Bpev01.c0051.g0092.m0001	0.748879891	0.12105697	0.3654426	nonDEG
Bpev01.c0483.g0002.m0001	0.325507067	0.32179962	0.65516711	nonDEG
Bpev01.c2094.g0002.m0001	0.662883004	0.03541332	0.15644247	nonDEG
Bpev01.c0050.g0128.m0001	-0.787741955	0.00903644	0.05729567	nonDEG
Bpev01.c0879.g0016.m0001	-0.422778343	0.21214103	0.51825598	nonDEG
Bpev01.c1272.g0010.m0001	0.706787925	0.22783463	0.54024011	nonDEG
Bpev01.c1435.g0003.m0001	-0.64384921	0.0494822	0.19875923	nonDEG
Bpev01.c0224.g0041.m0001	-0.516096472	0.08608961	0.28971101	nonDEG
Bpev01.c1328.g0004.m0001	-1.263356687	0.0222688	0.11166263	nonDEG
Bpev01.c1673.g0003.m0001	-0.674277615	0.04151882	0.17599316	nonDEG
Bpev01.c0870.g0008.m0001	0.843171078	0.09879226	0.31771305	nonDEG
Bpev01.c0009.g0001.m0001	-0.658433718	0.25520773	0.57554891	nonDEG
Bpev01.c0157.g0004.m0001	0.099024235	0.89266393	1	nonDEG
Bpev01.c1688.g0009.m0001	-0.641580723	0.06113702	0.23027287	nonDEG
Bpev01.c0606.g0022.m0001	1.847550624	0.02658346	0.12718912	nonDEG
Bpev01.c0237.g0031.m0001	-1.318061158	0.00994821	0.0616299	nonDEG
Bpev01.c0237.g0009.m0001	-0.766296234	0.01935108	0.1005607	nonDEG
Bpev01.c1211.g0017.m0001	-1.424341842	0.01167948	0.06920936	nonDEG
Bpev01.c0700.g0009.m0001	-0.25845066	0.40561024	0.73886268	nonDEG
Bpev01.c0776.g0001.m0001	0.246618439	0.57290255	0.88491936	nonDEG

Bpev01.c0177.g0038.m0001	-0.662271768	0.04222077	0.17812229	nonDEG
Bpev01.c1239.g0003.m0001	-0.49550157	0.10221559	0.32538114	nonDEG
Bpev01.c0454.g0025.m0001	-0.049230898	0.89475918	1	nonDEG
Bpev01.c0930.g0001.m0001	-3.687376652	0.08190069	0.28057824	nonDEG
Bpev01.c0094.g0137.m0001	-1.261239796	0.3850977	0.71876591	nonDEG
Bpev01.c2346.g0004.m0001	0.428877947	0.25850992	0.57890411	nonDEG
Bpev01.c1167.g0011.m0001	-0.70834752	0.02198039	0.11053648	nonDEG
Bpev01.c0778.g0006.m0001	-0.823765234	0.00814915	0.05293528	nonDEG
Bpev01.c1163.g0014.m0001	2.157777591	0.14477492	0.41000759	nonDEG
Bpev01.c0159.g0008.m0001	0.900184392	0.27941188	0.60626059	nonDEG
Bpev01.c1202.g0029.m0001	-0.502586831	0.14109929	0.40283556	nonDEG
Bpev01.c0015.g0154.m0001	-1.169965355	0.01501311	0.08364325	nonDEG
Bpev01.c0115.g0009.m0001	-0.836303213	0.01482043	0.08280485	nonDEG
Bpev01.c0080.g0027.m0002	-0.217156702	0.4810624	0.80999748	nonDEG
Bpev01.c0245.g0075.m0001	-0.639532739	0.10292727	0.3268915	nonDEG
Bpev01.c0892.g0013.m0001	-0.512192299	0.55106175	0.86669018	nonDEG
Bpev01.c1838.g0008.m0001	0.188179459	0.6463541	0.93220195	nonDEG
Bpev01.c0189.g0039.m0002	-0.442592488	0.17435599	0.46201827	nonDEG
Bpev01.c0332.g0009.m0001	0.87306041	0.00988222	0.06131143	nonDEG
Bpev01.c0588.g0006.m0001	-0.698422231	0.02287862	0.11392485	nonDEG
Bpev01.c0699.g0015.m0001	0.040715464	0.91391695	1	nonDEG
Bpev01.c0196.g0003.m0001	-0.560758616	0.06426685	0.23831111	nonDEG
Bpev01.c1800.g0004.m0001	-0.450121244	0.1476278	0.41529046	nonDEG
Bpev01.c0018.g0084.m0002	-0.758362436	0.06375173	0.23702356	nonDEG
Bpev01.c0094.g0287.m0001	-0.309848211	0.6332194	0.92895529	nonDEG
Bpev01.c0565.g0017.m0001	-0.025883148	0.95254882	1	nonDEG
Bpev01.c0004.g0010.m0001	0.612414053	0.15373205	0.42586975	nonDEG
Bpev01.c1769.g0003.m0001	-0.414627062	0.17466927	0.4623679	nonDEG
Bpev01.c0394.g0002.m0002	0.249149642	0.60777635	0.91157994	nonDEG
Bpev01.c0120.g0052.m0001	-1.222476169	0.00850923	0.05471894	nonDEG
Bpev01.c0901.g0006.m0001	0.251435831	0.51886141	0.83876563	nonDEG
Bpev01.c0135.g0024.m0001	-0.968205397	0.03173318	0.14421011	nonDEG
Bpev01.c0079.g0002.m0001	2.478769989	0.00869156	0.05554521	nonDEG
Bpev01.c0665.g0011.m0001	-0.201155909	0.5504869	0.86636733	nonDEG
Bpev01.c1536.g0004.m0001	-0.29511598	0.60729348	0.91126524	nonDEG
Bpev01.c1522.g0002.m0001	-2.618321476	0.15185773	0.42305938	nonDEG
Bpev01.c1786.g0015.m0001	0.863031207	0.26393522	0.58694584	nonDEG
Bpev01.c0580.g0008.m0001	-0.39503749	0.3227471	0.65639738	nonDEG
Bpev01.c0394.g0001.m0001	0.523535718	0.22401439	0.53398863	nonDEG
Bpev01.c2581.g0009.m0001	0.844396529	0.03137268	0.14325737	nonDEG
Bpev01.c0577.g0029.m0002	-0.933837691	0.06322541	0.23559548	nonDEG
Bpev01.c0094.g0029.m0001	-0.225587046	0.52222282	0.84176509	nonDEG
Bpev01.c0744.g0001.m0001	-0.865083568	0.0123338	0.07235845	nonDEG

Bpev01.c0135.g0096.m0001	-0.310356554	0.35535539	0.69326093	nonDEG
Bpev01.c1649.g0014.m0001	-2.140030271	0.12271358	0.36883217	nonDEG
Bpev01.c0000.g0198.m0001	-0.256507102	0.43829691	0.77028373	nonDEG
Bpev01.c0115.g0033.m0001	-0.166537888	0.75995842	0.99384371	nonDEG
Bpev01.c0051.g0198.m0001	-0.731908996	0.02179213	0.10985246	nonDEG
Bpev01.c0094.g0285.m0001	-0.262269137	0.48860678	0.81427283	nonDEG
Bpev01.c0027.g0051.m0001	-0.412256677	0.20527847	0.50791671	nonDEG
Bpev01.c0170.g0032.m0001	-0.759291266	0.01779336	0.09458626	nonDEG
Bpev01.c2858.g0001.m0001	-0.756792661	0.23069212	0.54453922	nonDEG
Bpev01.c0165.g0003.m0001	1.943850743	0.04576728	0.18809759	nonDEG
Bpev01.c0267.g0017.m0001	-0.446542388	0.307252	0.63797373	nonDEG
Bpev01.c0051.g0136.m0001	1.072911811	0.03247948	0.14667342	nonDEG
Bpev01.c0018.g0004.m0001	1.797464762	0.0312133	0.14275405	nonDEG
Bpev01.c0335.g0046.m0001	0.700620777	0.13207332	0.38580139	nonDEG
Bpev01.c0000.g0054.m0001	0.38174718	0.3349211	0.67059391	nonDEG
Bpev01.c3045.g0005.m0001	1.128841121	0.01901787	0.09926209	nonDEG
Bpev01.c1736.g0006.m0001	-0.896923806	0.0238046	0.11752197	nonDEG
Bpev01.c0094.g0210.m0001	-0.556357338	0.23794409	0.55485154	nonDEG
Bpev01.c0190.g0027.m0001	-0.902079865	0.01033049	0.06338458	nonDEG
Bpev01.c0275.g0036.m0001	-1.108828767	0.03542507	0.15646403	nonDEG
Bpev01.c0640.g0010.m0001	-0.550000288	0.06903409	0.25040594	nonDEG
Bpev01.c0679.g0009.m0001	-0.13123349	0.74976583	0.98722409	nonDEG
Bpev01.c0820.g0009.m0001	-0.994332891	0.01012667	0.06239409	nonDEG
Bpev01.c0029.g0059.m0001	-0.19644295	0.51538476	0.8355327	nonDEG
Bpev01.c1188.g0007.m0002	-0.698381071	0.03851694	0.16662751	nonDEG
Bpev01.c0027.g0170.m0001	-0.815072638	0.01785299	0.09478344	nonDEG
Bpev01.c1891.g0005.m0001	-0.35016328	0.38579887	0.71910428	nonDEG
Bpev01.c1956.g0005.m0001	-0.955133706	0.06788133	0.24746231	nonDEG
Bpev01.c0682.g0007.m0001	-0.81768936	0.01354185	0.07739442	nonDEG
Bpev01.c1545.g0002.m0001	-0.702927571	0.02176583	0.10974792	nonDEG
Bpev01.c0094.g0055.m0001	1.261177388	0.0249528	0.12172089	nonDEG
Bpev01.c1466.g0005.m0001	-1.568469206	0.00930483	0.05851846	nonDEG
Bpev01.c0094.g0227.m0001	0.078968701	0.83322026	1	nonDEG
Bpev01.c0038.g0157.m0001	1.102972591	0.31278331	0.64387301	nonDEG
Bpev01.c0387.g0008.m0001	-1.359928869	0.02190072	0.11024999	nonDEG
Bpev01.c0412.g0005.m0001	0.173869635	0.74438795	0.98458853	nonDEG
Bpev01.c0727.g0009.m0001	-0.8540282	0.05995663	0.22719892	nonDEG
Bpev01.c0939.g0008.m0001	0.446113545	0.24676806	0.56591143	nonDEG
Bpev01.c0051.g0109.m0001	-0.198696575	0.66155337	0.93220195	nonDEG
Bpev01.c0614.g0007.m0001	0.067119708	0.89674	1	nonDEG
Bpev01.c0265.g0004.m0001	-0.631767531	0.26643459	0.59040479	nonDEG
Bpev01.c0157.g0027.m0001	-0.712210688	0.01745156	0.09332545	nonDEG
Bpev01.c0855.g0006.m0001	-0.847513387	0.01628334	0.08885558	nonDEG

Bpev01.c0106.g0003.m0001	-0.8320802	0.02650119	0.1269599	nonDEG
Bpev01.c1138.g0008.m0001	-0.768926373	0.01972399	0.10181562	nonDEG
Bpev01.c2566.g0003.m0001	-0.356403541	0.23257597	0.54730647	nonDEG
Bpev01.c0399.g0008.m0001	-0.649840734	0.10628509	0.33353873	nonDEG
Bpev01.c0340.g0004.m0001	-1.104911027	0.31906603	0.65210005	nonDEG
Bpev01.c0213.g0061.m0001	-0.513317813	0.18707843	0.48131277	nonDEG
Bpev01.c0469.g0008.m0001	-0.708747069	0.0677772	0.24724686	nonDEG
Bpev01.c0800.g0001.m0001	-0.922333862	0.01132237	0.06780296	nonDEG
Bpev01.c1938.g0001.m0001	-0.658074479	0.05136964	0.20416778	nonDEG
Bpev01.c1758.g0008.m0001	0.037140905	0.91147599	1	nonDEG
Bpev01.c0278.g0045.m0001	1.04149398	0.02761335	0.13075588	nonDEG
Bpev01.c0088.g0039.m0001	-2.188976272	0.05069734	0.20242494	nonDEG
Bpev01.c1972.g0012.m0001	-0.367142281	0.58023329	0.89096014	nonDEG
Bpev01.c0037.g0010.m0001	-0.949458297	0.06690231	0.24493379	nonDEG
Bpev01.c0142.g0037.m0001	1.248648982	0.03981502	0.17073456	nonDEG
Bpev01.c0163.g0007.m0001	0.772534263	0.01864765	0.09780683	nonDEG
Bpev01.c0038.g0049.m0001	-0.485296365	0.16126073	0.43961057	nonDEG
Bpev01.c0349.g0020.m0001	-0.197036246	0.55920375	0.87419668	nonDEG
Bpev01.c1082.g0005.m0001	-0.199692451	0.5134977	0.83398757	nonDEG
Bpev01.c1891.g0007.m0001	-0.438275494	0.16209825	0.44050682	nonDEG
Bpev01.c0433.g0001.m0001	-0.34000892	0.58153287	0.89216459	nonDEG
Bpev01.c0161.g0112.m0001	-0.878872834	0.09944488	0.31928866	nonDEG
Bpev01.c0080.g0027.m0001	-0.789263836	0.01303896	0.07538372	nonDEG
Bpev01.c0177.g0042.m0001	0.456169083	0.25586037	0.57598389	nonDEG
Bpev01.c0042.g0053.m0001	-0.763202176	0.10824811	0.33768317	nonDEG
Bpev01.c0455.g0007.m0001	-1.048690712	0.01278401	0.07428906	nonDEG
Bpev01.c0029.g0120.m0001	-0.578721446	0.05643464	0.21808532	nonDEG
Bpev01.c0094.g0103.m0001	0.251807784	0.54102037	0.85823979	nonDEG
Bpev01.c0051.g0042.m0001	-0.20897986	0.63199266	0.92831514	nonDEG
Bpev01.c0394.g0002.m0001	0.465208173	0.31482797	0.64686074	nonDEG
Bpev01.c1687.g0008.m0001	-1.334328443	0.01038652	0.06364879	nonDEG
Bpev01.c1346.g0003.m0001	1.841180561	0.11170155	0.34530944	nonDEG
Bpev01.c0261.g0027.m0001	-1.197382412	0.01781323	0.09465769	nonDEG
Bpev01.c1315.g0002.m0001	0.022567133	0.945672	1	nonDEG
Bpev01.c0751.g0003.m0001	-0.336506108	0.57024171	0.88307974	nonDEG
Bpev01.c0000.g0219.m0001	-0.347760006	0.35934142	0.69762125	nonDEG
Bpev01.c1188.g0007.m0001	-0.464012965	0.14268454	0.40594461	nonDEG
Bpev01.c0223.g0036.m0001	1.083449953	0.08376507	0.28470977	nonDEG
Bpev01.c0892.g0016.m0001	1.247546806	0.08647981	0.29045969	nonDEG
Bpev01.c2361.g0009.m0001	-0.85071692	0.04453718	0.18510943	nonDEG
Bpev01.c0027.g0020.m0001	-0.94141771	0.02727886	0.12948442	nonDEG
Bpev01.c0044.g0115.m0001	0.929979418	0.0448499	0.18580585	nonDEG
Bpev01.c0094.g0286.m0001	-0.551942161	0.29403277	0.62402044	nonDEG

Bpev01.c0577.g0029.m0001	-1.178083565	0.02007775	0.10311738	nonDEG
Bpev01.c0710.g0010.m0001	-0.465046545	0.12156012	0.36643542	nonDEG
Bpev01.c0932.g0006.m0001	-0.634049794	0.05326274	0.20959585	nonDEG
Bpev01.c0343.g0015.m0001	-0.043030279	0.88819232	1	nonDEG
Bpev01.c2535.g0005.m0001	-0.147581446	0.79323083	1	nonDEG
Bpev01.c0699.g0010.m0001	-0.391491855	0.30467349	0.63602914	nonDEG
Bpev01.c0942.g0008.m0001	-0.896242219	0.0408957	0.17410391	nonDEG
Bpev01.c0598.g0015.m0001	-0.577327125	0.05483341	0.21383506	nonDEG
Bpev01.c0529.g0006.m0001	0.061972501	0.88002136	1	nonDEG
Bpev01.c0901.g0022.m0001	-0.914627676	0.01323492	0.07618748	nonDEG
Bpev01.c0073.g0003.m0001	-0.625796411	0.03787482	0.16447806	nonDEG
Bpev01.c0018.g0084.m0001	-0.68962751	0.06391936	0.23741129	nonDEG
Bpev01.c0483.g0004.m0001	-0.644091609	0.2715286	0.59720064	nonDEG
Bpev01.c0162.g0062.m0001	-0.970491657	0.15474377	0.42754126	nonDEG
Bpev01.c0815.g0014.m0001	-0.608570697	0.10372295	0.32843927	nonDEG
Bpev01.c0857.g0013.m0001	-0.193046301	0.52358102	0.84310807	nonDEG
Bpev01.c0052.g0146.m0001	-1.349284845	0.01159542	0.06891933	nonDEG
Bpev01.c0597.g0019.m0001	1.769385798	0.01470142	0.08235875	nonDEG
Bpev01.c0082.g0064.m0002	-0.580134533	0.18056047	0.47169399	nonDEG
Bpev01.c0094.g0282.m0001	0.583951577	0.54145246	0.85864139	nonDEG
Bpev01.c0089.g0019.m0001	0.652561188	0.07090234	0.25479664	nonDEG
Bpev01.c0574.g0005.m0001	-0.561057092	0.06313216	0.23549769	nonDEG
Bpev01.c0348.g0006.m0001	-0.116888462	0.70528063	0.96019182	nonDEG
Bpev01.c2535.g0007.m0001	-0.333285117	0.56890206	0.88179371	nonDEG
Bpev01.c0134.g0012.m0001	-1.809322855	0.00950679	0.05940784	nonDEG
Bpev01.c3373.g0001.m0001	-0.513858099	0.16630325	0.44796425	nonDEG
Bpev01.c0134.g0011.m0001	-1.060615417	0.0242131	0.11898005	nonDEG
Bpev01.c2157.g0001.m0001	0.029483784	0.93087433	1	nonDEG
Bpev01.c0017.g0012.m0001	0.205302343	0.60580583	0.91023925	nonDEG
Bpev01.c0051.g0107.m0001	-0.420363031	0.28914104	0.61802244	nonDEG
Bpev01.c0015.g0219.m0001	0.025796551	0.95457799	1	nonDEG
Bpev01.c0066.g0043.m0001	-0.501514685	0.09495954	0.3093067	nonDEG
Bpev01.c0878.g0004.m0001	-0.0252304	0.94013462	1	nonDEG
Bpev01.c0605.g0012.m0001	-0.80470333	0.03069383	0.1408866	nonDEG
Bpev01.c0154.g0046.m0001	-0.781547572	0.01123469	0.06749754	nonDEG
Bpev01.c0449.g0020.m0001	-0.438276642	0.27589578	0.60261877	nonDEG
Bpev01.c1490.g0001.m0001	-0.122437129	0.68833382	0.9475735	nonDEG
Bpev01.c0220.g0005.m0001	0.168460633	0.59989202	0.90630305	nonDEG
Bpev01.c1577.g0002.m0001	-1.160554808	0.0368268	0.16118704	nonDEG
Bpev01.c0115.g0109.m0001	3.978560923	0.00806989	0.05254799	nonDEG
Bpev01.c0338.g0001.m0001	-1.341447465	0.01066186	0.0648769	nonDEG
Bpev01.c0918.g0011.m0001	-0.149962513	0.69679657	0.95418152	nonDEG
Bpev01.c0848.g0010.m0001	-0.69225027	0.13189793	0.38566561	nonDEG

Bpev01.c0274.g0062.m0001	-0.053739469	0.86305574	1	nonDEG
Bpev01.c0919.g0012.m0001	1.497108722	0.12504476	0.37311907	nonDEG
Bpev01.c0414.g0007.m0001	-1.238297214	0.00833732	0.05382506	nonDEG
Bpev01.c0984.g0025.m0001	0.778069677	0.01050805	0.06420091	nonDEG
Bpev01.c0095.g0032.m0001	2.163712932	0.0173823	0.09312422	nonDEG
Bpev01.c0518.g0009.m0001	-2.304435851	1	1	nonDEG
Bpev01.c0018.g0012.m0001	-0.757161776	0.01294642	0.07496474	nonDEG
Bpev01.c0848.g0015.m0001	-0.278056724	0.39661215	0.72972424	nonDEG
Bpev01.c0430.g0006.m0001	-0.315119764	0.6021455	0.90770583	nonDEG
Bpev01.c0995.g0003.m0001	0.6570396	0.0311364	0.14248786	nonDEG
Bpev01.c1328.g0005.m0001	-0.845722498	0.06739218	0.24622384	nonDEG
Bpev01.c0166.g0013.m0001	-1.036733384	0.0147949	0.08270925	nonDEG
Bpev01.c1891.g0006.m0001	0.432394676	0.19085681	0.48658818	nonDEG
Bpev01.c0477.g0013.m0001	-1.791120592	0.01274594	0.074141	nonDEG
Bpev01.c0306.g0006.m0001	1.614502521	0.04933178	0.19837352	nonDEG
Bpev01.c3139.g0001.m0001	0.608800291	0.17694842	0.46545819	nonDEG
Bpev01.c0016.g0133.m0001	-0.804129662	0.02911024	0.13581242	nonDEG
Bpev01.c0402.g0019.m0001	-1.990581736	0.01582315	0.08697117	nonDEG
Bpev01.c0454.g0026.m0001	-0.795982094	0.01357857	0.07754404	nonDEG
Bpev01.c1315.g0001.m0001	0.0381687	0.90447286	1	nonDEG
Bpev01.c0094.g0211.m0001	-1.111936423	0.06563585	0.2417005	nonDEG
Bpev01.c0659.g0005.m0001	0.172197388	0.59595346	0.90369187	nonDEG
Bpev01.c0699.g0017.m0001	0.148960979	0.6503467	0.93220195	nonDEG
Bpev01.c0022.g0002.m0001	-0.670785635	0.06255685	0.2338851	nonDEG
Bpev01.c0135.g0022.m0001	-2.063836756	0.01269089	0.07390844	nonDEG
Bpev01.c0044.g0018.m0001	-0.761899303	0.0355514	0.15686417	nonDEG
Bpev01.c0531.g0025.m0001	2.601441777	0.01719291	0.09241228	nonDEG
Bpev01.c0213.g0002.m0001	-0.255760874	0.63080833	0.92786882	nonDEG
Bpev01.c0833.g0005.m0001	-0.475595679	0.11683626	0.35598662	nonDEG
Bpev01.c0445.g0017.m0001	-0.864709068	0.14363915	0.40761452	nonDEG
Bpev01.c0412.g0006.m0001	-1.090826278	0.08371192	0.28457219	nonDEG
Bpev01.c0257.g0001.m0001	-0.82820381	0.0103097	0.06330144	nonDEG
Bpev01.c0469.g0012.m0001	-2.198969264	0.07071307	0.2544002	nonDEG
Bpev01.c0163.g0010.m0001	-0.901110724	0.00850085	0.05470091	nonDEG
Bpev01.c0120.g0064.m0001	-0.296195335	1	1	nonDEG
Bpev01.c1263.g0004.m0001	-0.012911689	0.97700096	1	nonDEG
Bpev01.c0420.g0004.m0001	-0.601624926	0.05357086	0.21030437	nonDEG
Bpev01.c0313.g0008.m0001	-0.613804994	0.29875723	0.62939132	nonDEG
Bpev01.c0617.g0008.m0001	-0.622112464	0.0592812	0.22528763	nonDEG
Bpev01.c0699.g0011.m0001	-0.061483051	0.8765775	1	nonDEG
Bpev01.c0700.g0010.m0001	-0.68828223	0.03966893	0.17030971	nonDEG
Bpev01.c0464.g0005.m0001	-0.82765194	0.01329635	0.07645144	nonDEG
Bpev01.c1263.g0002.m0001	-0.047054998	0.91168831	1	nonDEG

Bpev01.c1577.g0006.m0001	-1.065821046	0.02634196	0.12642337	nonDEG
Bpev01.c0337.g0003.m0001	-0.857932503	0.01081998	0.06563558	nonDEG
Bpev01.c0135.g0021.m0002	-1.107259583	0.01651804	0.08980138	nonDEG
Bpev01.c2045.g0002.m0001	1.496359787	0.03405512	0.15197497	nonDEG
Bpev01.c0082.g0053.m0001	1.204769074	0.035143	0.15569222	nonDEG
Bpev01.c0082.g0058.m0001	1.35849571	0.08996742	0.29851264	nonDEG
Bpev01.c0719.g0019.m0001	1.021663752	0.03829107	0.16579656	nonDEG
Bpev01.c2741.g0001.m0001	-2.272071509	0.07317267	0.26049817	nonDEG
Bpev01.c0082.g0052.m0001	-0.616710185	0.40622348	0.73947645	nonDEG
Bpev01.c0660.g0004.m0001	0.690504304	0.34021811	0.67634129	nonDEG
Bpev01.c0082.g0056.m0001	1.710799588	0.0419087	0.17718661	nonDEG
Bpev01.c0157.g0070.m0001	0.852042329	0.24878149	0.56818295	nonDEG
Bpev01.c0865.g0004.m0002	-3.039047473	0.00833649	0.05382506	nonDEG
Bpev01.c1323.g0001.m0001	0.722899687	0.52300306	0.84254964	nonDEG
Bpev01.c0442.g0002.m0001	0.046155134	0.93897346	1	nonDEG
Bpev01.c0082.g0054.m0001	0.249397938	0.77072333	1	nonDEG
Bpev01.c2944.g0001.m0001	1.035522216	0.01119734	0.06732874	nonDEG
Bpev01.c0660.g0009.m0001	0.901190017	0.03485288	0.15466437	nonDEG
Bpev01.c0173.g0005.m0001	1.29286403	0.02320444	0.11517107	nonDEG
Bpev01.c0023.g0002.m0001	-0.133261712	0.79956826	1	nonDEG
Bpev01.c0082.g0049.m0001	1.570888187	0.10277254	0.32665542	nonDEG
Bpev01.c0105.g0025.m0001	1.35698445	0.13277548	0.3872377	nonDEG
Bpev01.c0373.g0022.m0001	1.85847158	0.01251394	0.07318195	nonDEG
Bpev01.c1736.g0001.m0001	1.40291983	0.01043411	0.06390049	nonDEG
Bpev01.c0027.g0118.m0001	-0.555890776	0.50366122	0.82624991	nonDEG
Bpev01.c2382.g0002.m0001	2.53550645	0.01442094	0.0811024	nonDEG
Bpev01.c0023.g0001.m0001	-0.573105951	0.20396694	0.5062954	nonDEG
Bpev01.c1085.g0028.m0001	2.008459034	0.02225648	0.11161983	nonDEG
Bpev01.c0328.g0027.m0001	1.289421078	0.06238535	0.23362962	nonDEG
Bpev01.c1807.g0007.m0001	0.441120533	0.2777397	0.60481743	nonDEG
Bpev01.c1879.g0002.m0001	-1.33950351	0.08264042	0.28213515	nonDEG
Bpev01.c1439.g0013.m0001	-2.084883233	0.02322385	0.11523461	nonDEG
Bpev01.c0660.g0010.m0001	1.247961893	0.17038582	0.45515285	nonDEG
Bpev01.c0022.g0053.m0001	-0.177479129	0.56247707	0.87686732	nonDEG
Bpev01.c1081.g0005.m0001	0.818986212	0.55378439	0.86930035	nonDEG
Bpev01.c0744.g0009.m0001	-0.669790078	0.03288762	0.14799429	nonDEG
Bpev01.c0629.g0028.m0001	-0.220203619	0.48626224	0.81311074	nonDEG
Bpev01.c0566.g0066.m0001	0.280615376	0.37375387	0.7132879	nonDEG
Bpev01.c0162.g0011.m0001	-0.824173236	0.3380568	0.6740106	nonDEG
Bpev01.c1006.g0031.m0001	-0.248325876	0.43863999	0.77071798	nonDEG
Bpev01.c1060.g0022.m0001	-0.822149967	0.00873088	0.05574246	nonDEG
Bpev01.c1093.g0012.m0001	-0.812406075	0.0140559	0.07957457	nonDEG
Bpev01.c1600.g0019.m0001	0.143613158	0.63311114	0.92884276	nonDEG

Bpev01.c1049.g0002.m0001	-0.466342079	0.13438391	0.39045873	nonDEG
Bpev01.c0281.g0074.m0001	0.302506301	0.39589283	0.7291016	nonDEG
Bpev01.c0042.g0046.m0001	-0.353532986	0.4623555	0.79130768	nonDEG
Bpev01.c0142.g0043.m0001	-0.110419902	0.78915031	1	nonDEG
Bpev01.c0145.g0050.m0001	0.641281495	0.32720388	0.66200905	nonDEG
Bpev01.c2182.g0006.m0001	1.083988443	0.04980426	0.19972585	nonDEG
Bpev01.c0919.g0018.m0001	-0.176801584	0.64926769	0.93220195	nonDEG
Bpev01.c1378.g0015.m0001	0.624571462	0.04743621	0.19272351	nonDEG
Bpev01.c0442.g0006.m0001	-0.924087121	0.04387834	0.18318623	nonDEG
Bpev01.c1087.g0001.m0001	1.641870498	0.04698842	0.19145757	nonDEG
Bpev01.c0914.g0004.m0001	-0.140388089	0.66905356	0.93342816	nonDEG
Bpev01.c0627.g0006.m0001	-0.308991078	0.31746344	0.65009782	nonDEG
Bpev01.c0473.g0003.m0001	0.12839815	0.80260202	1	nonDEG
Bpev01.c0558.g0004.m0002	0.731063334	0.12804047	0.37894676	nonDEG
Bpev01.c0789.g0011.m0001	-0.488997951	0.24193067	0.55946902	nonDEG
Bpev01.c0023.g0053.m0001	-0.130529076	0.68356174	0.9442364	nonDEG
Bpev01.c0353.g0022.m0002	0.361649194	0.2841601	0.61119231	nonDEG
Bpev01.c0029.g0075.m0001	-0.398522788	0.44036959	0.77222066	nonDEG
Bpev01.c0483.g0020.m0001	0.882799424	0.00881264	0.05616697	nonDEG
Bpev01.c0038.g0100.m0001	0.714972475	0.04118729	0.17498947	nonDEG
Bpev01.c0379.g0015.m0001	-0.239376135	0.42214097	0.75382689	nonDEG
Bpev01.c0223.g0026.m0001	0.709335824	0.02615714	0.1258441	nonDEG
Bpev01.c0874.g0012.m0001	-0.224734264	0.49189467	0.81591656	nonDEG
Bpev01.c0223.g0014.m0001	1.249412002	0.62151043	0.92162607	nonDEG
Bpev01.c0044.g0108.m0001	-0.457290185	0.22729748	0.53949331	nonDEG
Bpev01.c1165.g0005.m0001	-0.542675991	0.22444759	0.53459904	nonDEG
Bpev01.c0629.g0026.m0001	-0.882831115	0.01026288	0.0630749	nonDEG
Bpev01.c2147.g0005.m0001	0.515626234	1	1	nonDEG
Bpev01.c0142.g0070.m0001	0.264750935	0.73626496	0.97983383	nonDEG
Bpev01.c0224.g0012.m0001	-0.380846253	0.24368993	0.56152677	nonDEG
Bpev01.c1917.g0001.m0001	0.681273727	0.49546725	0.81913398	nonDEG
Bpev01.c0571.g0012.m0001	-0.409212751	0.18276501	0.47498294	nonDEG
Bpev01.c0052.g0030.m0001	-0.034802316	0.93896283	1	nonDEG
Bpev01.c2153.g0001.m0001	-1.07141172	0.01090816	0.06607518	nonDEG
Bpev01.c0577.g0010.m0001	0.471277929	0.23789752	0.55485154	nonDEG
Bpev01.c0001.g0076.m0001	0.342246435	0.27369022	0.59979711	nonDEG
Bpev01.c0210.g0032.m0001	0.749988985	0.01525671	0.08451603	nonDEG
Bpev01.c1702.g0011.m0001	-0.680604967	0.08816694	0.29421206	nonDEG
Bpev01.c0480.g0037.m0002	1.188522536	0.03376874	0.15112768	nonDEG
Bpev01.c0045.g0018.m0001	-1.826449957	0.03635256	0.15961239	nonDEG
Bpev01.c0051.g0088.m0001	0.596730373	0.68073637	0.94218138	nonDEG
Bpev01.c0029.g0116.m0001	0.056320115	0.89227802	1	nonDEG
Bpev01.c0051.g0036.m0001	-0.450248491	0.16645075	0.44819755	nonDEG

Bpev01.c0497.g0027.m0001	0.337733982	0.74308221	0.98399997	nonDEG
Bpev01.c0121.g0014.m0001	-0.171881926	0.69384577	0.95164581	nonDEG
Bpev01.c0222.g0010.m0001	-0.269800576	0.64903986	0.93220195	nonDEG
Bpev01.c0167.g0016.m0001	-0.050640953	0.86848739	1	nonDEG
Bpev01.c1527.g0010.m0001	2.133936877	0.05413885	0.2117998	nonDEG
Bpev01.c0261.g0024.m0001	0.466083858	0.18917214	0.4843906	nonDEG
Bpev01.c0029.g0156.m0001	0.438019918	0.46279218	0.79176351	nonDEG
Bpev01.c0000.g0131.m0001	0.958532003	0.14230571	0.40539362	nonDEG
Bpev01.c0030.g0028.m0001	0.74384673	0.11761836	0.35762931	nonDEG
Bpev01.c0080.g0127.m0001	-0.628137196	0.06511185	0.24038472	nonDEG
Bpev01.c0425.g0024.m0001	-0.62468366	0.06346905	0.23618575	nonDEG
Bpev01.c0127.g0086.m0001	-0.470322162	0.14652246	0.41293075	nonDEG
Bpev01.c0050.g0110.m0001	2.237571108	0.02525019	0.1227242	nonDEG
Bpev01.c2640.g0001.m0001	-0.598860638	0.04744918	0.19274962	nonDEG
Bpev01.c1206.g0020.m0001	0.476111443	0.12477863	0.37258327	nonDEG
Bpev01.c0444.g0013.m0001	0.206418734	0.50555236	0.82773576	nonDEG
Bpev01.c0015.g0254.m0001	0.038158688	0.90115401	1	nonDEG
Bpev01.c0404.g0002.m0001	0.042820052	0.89955717	1	nonDEG
Bpev01.c0115.g0017.m0001	1.257085804	0.01318024	0.07594698	nonDEG
Bpev01.c1159.g0004.m0001	-0.586734201	0.1074692	0.33589402	nonDEG
Bpev01.c0095.g0021.m0001	-0.258546101	0.39511697	0.72843158	nonDEG
Bpev01.c0214.g0064.m0001	-0.311831729	0.34636506	0.68300518	nonDEG
Bpev01.c0088.g0003.m0001	-1.099673758	0.20162039	0.50275306	nonDEG
Bpev01.c1172.g0001.m0001	-0.905128414	0.32595515	0.66018531	nonDEG
Bpev01.c1938.g0011.m0001	0.173894484	0.66498899	0.93220195	nonDEG
Bpev01.c0804.g0014.m0001	-0.35893842	0.59060495	0.89993168	nonDEG
Bpev01.c0383.g0030.m0002	-0.103137666	0.74146973	0.98341259	nonDEG
Bpev01.c0228.g0011.m0001	0.365266076	0.31781363	0.65057392	nonDEG
Bpev01.c0281.g0037.m0001	0.903578265	0.04527592	0.18670373	nonDEG
Bpev01.c0485.g0006.m0001	-0.618883372	0.66622555	0.93220195	nonDEG
Bpev01.c0027.g0149.m0001	-1.622637991	0.09039316	0.29915208	nonDEG
Bpev01.c0115.g0105.m0001	-0.297279259	0.33326165	0.66901246	nonDEG
Bpev01.c0062.g0063.m0001	-0.693264682	0.02040381	0.10434754	nonDEG
Bpev01.c0275.g0005.m0001	0.112631398	0.7868876	1	nonDEG
Bpev01.c1945.g0002.m0001	1.431936659	0.27230686	0.59821333	nonDEG
Bpev01.c0462.g0006.m0001	0.597323984	0.50141459	0.82395446	nonDEG
Bpev01.c0782.g0013.m0001	-0.086286347	0.78098181	1	nonDEG
Bpev01.c0557.g0030.m0001	2.245804498	0.06755827	0.24660233	nonDEG
Bpev01.c0006.g0001.m0001	-0.054423577	1	1	nonDEG
Bpev01.c0135.g0104.m0001	0.568063307	0.09146359	0.3017643	nonDEG
Bpev01.c1029.g0006.m0001	0.032594622	0.92918962	1	nonDEG
Bpev01.c0050.g0097.m0001	0.018616862	0.9618617	1	nonDEG
Bpev01.c1837.g0005.m0001	-0.450887239	0.15430511	0.42674609	nonDEG

Bpev01.c1610.g0009.m0001	0.632401053	0.1644522	0.44456457	nonDEG
Bpev01.c0022.g0085.m0001	1.279623225	0.01836787	0.09688799	nonDEG
Bpev01.c0665.g0010.m0001	-0.125916224	0.74373972	0.98416996	nonDEG
Bpev01.c0549.g0005.m0001	0.234384872	0.59410518	0.90238771	nonDEG
Bpev01.c0793.g0002.m0001	0.422428319	0.1623931	0.44078866	nonDEG
Bpev01.c0505.g0006.m0001	-1.313145234	0.0146447	0.08208782	nonDEG
Bpev01.c0161.g0037.m0001	-0.140494588	0.63957848	0.93036198	nonDEG
Bpev01.c1025.g0002.m0001	-0.173764657	0.74168356	0.98341259	nonDEG
Bpev01.c0672.g0004.m0001	-0.355829679	0.25440126	0.57509421	nonDEG
Bpev01.c0401.g0016.m0001	0.614915148	0.06072491	0.22916043	nonDEG
Bpev01.c0396.g0007.m0001	-0.6616471	0.27041894	0.59577498	nonDEG
Bpev01.c0665.g0008.m0001	-0.18359707	0.57233207	0.88459239	nonDEG
Bpev01.c0080.g0017.m0001	-0.022069249	1	1	nonDEG
Bpev01.c1338.g0011.m0001	-0.656926051	0.2092597	0.51422339	nonDEG
Bpev01.c1270.g0012.m0001	0.363236381	0.37093121	0.71046833	nonDEG
Bpev01.c0042.g0071.m0001	-0.506983428	0.55497895	0.87043289	nonDEG
Bpev01.c0062.g0010.m0001	0.002090875	0.99457467	1	nonDEG
Bpev01.c0452.g0020.m0001	0.460597423	0.70474801	0.95975931	nonDEG
Bpev01.c0568.g0012.m0001	-0.132493945	0.73850942	0.98153403	nonDEG
Bpev01.c1925.g0003.m0001	-1.424414814	0.22635608	0.53791546	nonDEG
Bpev01.c1040.g0038.m0001	-0.598526945	0.0646066	0.23912882	nonDEG
Bpev01.c0118.g0030.m0001	-0.635198229	0.08472784	0.28655499	nonDEG
Bpev01.c2361.g0012.m0002	0.618110101	0.04196949	0.17739266	nonDEG
Bpev01.c0759.g0006.m0001	0.620154076	0.73674797	0.98012217	nonDEG
Bpev01.c3426.g0001.m0001	0.614278714	0.32446532	0.65852738	nonDEG
Bpev01.c0050.g0047.m0002	-0.393621126	0.28485152	0.61214981	nonDEG
Bpev01.c2394.g0001.m0001	1.193034	0.23089734	0.5448807	nonDEG
Bpev01.c0050.g0049.m0001	-0.171685716	0.58314954	0.89357378	nonDEG
Bpev01.c0337.g0004.m0001	0.073006044	0.93927281	1	nonDEG
Bpev01.c0588.g0010.m0001	1.182069173	0.00971624	0.06040893	nonDEG
Bpev01.c0062.g0028.m0001	0.141527627	0.70578912	0.96033189	nonDEG
Bpev01.c0080.g0037.m0001	1.078862013	0.30431671	0.63589769	nonDEG
Bpev01.c0245.g0009.m0001	-0.185285802	0.62084724	0.92132505	nonDEG
Bpev01.c0582.g0018.m0001	-0.42544194	0.3952914	0.72843158	nonDEG
Bpev01.c0411.g0012.m0001	-1.608861093	0.08997258	0.29851264	nonDEG
Bpev01.c0449.g0044.m0001	-0.819598424	0.10824289	0.33768317	nonDEG
Bpev01.c0114.g0045.m0001	-0.629971308	0.05520366	0.21465334	nonDEG
Bpev01.c0022.g0115.m0001	1.033776619	0.06896004	0.2502811	nonDEG
Bpev01.c0565.g0014.m0001	-0.209824609	0.67970844	0.94146687	nonDEG
Bpev01.c0879.g0003.m0001	-0.239704714	0.57401118	0.88595401	nonDEG
Bpev01.c1150.g0013.m0001	-0.395255719	0.23062827	0.54450764	nonDEG
Bpev01.c0386.g0002.m0001	-0.238078343	0.56652542	0.87944311	nonDEG
Bpev01.c0473.g0032.m0001	-0.879037321	0.00999124	0.06176651	nonDEG

Bpev01.c0147.g0005.m0001	-0.106845364	0.72565715	0.97377248	nonDEG
Bpev01.c0404.g0029.m0001	0.691686494	0.04571631	0.18796681	nonDEG
Bpev01.c1202.g0049.m0001	0.538437693	0.08358692	0.28425386	nonDEG
Bpev01.c0120.g0004.m0001	-0.132107948	0.74957693	0.98706366	nonDEG
Bpev01.c0597.g0017.m0001	-0.388180516	0.21096596	0.51673408	nonDEG
Bpev01.c0560.g0010.m0001	-2.457731318	0.04891931	0.19716806	nonDEG
Bpev01.c0015.g0106.m0002	0.680539329	0.10563088	0.33233428	nonDEG
Bpev01.c2443.g0001.m0001	-0.939397358	0.305122	0.63636974	nonDEG
Bpev01.c0483.g0021.m0001	2.04349797	0.29268888	0.62230737	nonDEG
Bpev01.c2183.g0007.m0001	-0.206279495	0.50631808	0.82845143	nonDEG
Bpev01.c0613.g0005.m0001	-0.55614137	0.07831892	0.2723722	nonDEG
Bpev01.c0237.g0023.m0001	2.016488733	0.01325222	0.07625724	nonDEG
Bpev01.c1638.g0003.m0001	-1.625913937	0.01591907	0.08743312	nonDEG
Bpev01.c1847.g0004.m0001	-0.458834519	0.13627506	0.39428025	nonDEG
Bpev01.c0171.g0018.m0001	-0.331376328	0.29333324	0.62322729	nonDEG
Bpev01.c0029.g0150.m0001	-0.335526468	0.26562026	0.58953518	nonDEG
Bpev01.c0000.g0041.m0001	0.21224288	0.53391724	0.8520621	nonDEG
Bpev01.c0395.g0001.m0001	-0.922673466	0.00800213	0.05222228	nonDEG
Bpev01.c0016.g0081.m0001	-0.218587188	0.62652795	0.92459054	nonDEG
Bpev01.c3064.g0001.m0001	0.027485502	0.97539068	1	nonDEG
Bpev01.c0389.g0049.m0002	-1.970215608	0.01610304	0.08811477	nonDEG
Bpev01.c0353.g0034.m0001	-0.343705498	0.25127734	0.57162148	nonDEG
Bpev01.c0893.g0015.m0001	-0.020258461	0.95986938	1	nonDEG
Bpev01.c1479.g0004.m0001	0.817567645	0.02593799	0.12515793	nonDEG
Bpev01.c0555.g0013.m0002	-0.507590127	0.11388679	0.34993353	nonDEG
Bpev01.c0238.g0001.m0001	-0.773181617	0.08583777	0.28916745	nonDEG
Bpev01.c0022.g0055.m0001	0.165221687	0.58827781	0.89815945	nonDEG
Bpev01.c0126.g0049.m0003	0.499027729	0.13210895	0.38586719	nonDEG
Bpev01.c1250.g0010.m0001	-0.25278659	0.40308582	0.73631444	nonDEG
Bpev01.c0777.g0025.m0001	-0.800203432	0.01612515	0.08820294	nonDEG
Bpev01.c0136.g0013.m0001	0.525197743	0.08259279	0.28207065	nonDEG
Bpev01.c0265.g0016.m0001	0.074603141	0.80427009	1	nonDEG
Bpev01.c0887.g0017.m0001	-1.465351592	0.63720094	0.93006545	nonDEG
Bpev01.c0575.g0004.m0001	-0.541191725	0.66336027	0.93220195	nonDEG
Bpev01.c2183.g0004.m0001	-0.150070864	0.65210674	0.93220195	nonDEG
Bpev01.c0190.g0031.m0001	-0.103538885	0.74761995	0.98577955	nonDEG
Bpev01.c0739.g0020.m0001	-0.547215366	0.07384923	0.26209133	nonDEG
Bpev01.c0022.g0079.m0001	-0.327596126	0.2928282	0.62251367	nonDEG
Bpev01.c0245.g0005.m0001	-0.907368331	0.00803656	0.0524121	nonDEG
Bpev01.c0036.g0014.m0001	0.53121239	0.45176861	0.78330192	nonDEG
Bpev01.c0920.g0016.m0001	-0.518750169	0.09465493	0.30877448	nonDEG
Bpev01.c3072.g0001.m0001	0.098135122	0.77586402	1	nonDEG
Bpev01.c0609.g0011.m0001	1.202736509	0.07922424	0.27432161	nonDEG

Bpev01.c2314.g0005.m0001	0.01896041	0.96104595	1	nonDEG
Bpev01.c1560.g0018.m0001	1.408621965	0.00933305	0.058598	nonDEG
Bpev01.c0506.g0034.m0001	-0.151761619	0.62197508	0.92198954	nonDEG
Bpev01.c0494.g0001.m0001	-1.748706566	0.31331944	0.6446217	nonDEG
Bpev01.c1529.g0004.m0001	-0.379301596	0.24940623	0.56925647	nonDEG
Bpev01.c0333.g0013.m0001	-0.059353058	0.86845976	1	nonDEG
Bpev01.c0428.g0011.m0001	1.249047036	0.03525353	0.15604642	nonDEG
Bpev01.c0016.g0060.m0001	0.623331281	0.03792532	0.16462454	nonDEG
Bpev01.c0344.g0032.m0001	0.42251929	0.18046338	0.47152404	nonDEG
Bpev01.c0051.g0164.m0001	1.338430935	0.04255836	0.17921262	nonDEG
Bpev01.c0080.g0031.m0001	-0.115438448	0.70419232	0.95949794	nonDEG
Bpev01.c0929.g0002.m0002	-0.831197868	0.02801034	0.13216802	nonDEG
Bpev01.c0547.g0015.m0001	0.090009265	0.77373533	1	nonDEG
Bpev01.c2715.g0005.m0001	0.039180517	0.93939747	1	nonDEG
Bpev01.c0096.g0015.m0001	0.053458456	0.86655138	1	nonDEG
Bpev01.c0719.g0018.m0001	4.15579491	0.29604426	0.62617553	nonDEG
Bpev01.c0349.g0064.m0001	0.408631049	0.17174875	0.45773144	nonDEG
Bpev01.c2868.g0001.m0001	0.399710397	0.64555028	0.93220195	nonDEG
Bpev01.c0571.g0013.m0001	-0.726302037	0.02131432	0.10798009	nonDEG
Bpev01.c1588.g0012.m0001	0.784741732	0.23798339	0.55485154	nonDEG
Bpev01.c0016.g0146.m0001	0.33041252	0.90269819	1	nonDEG
Bpev01.c0245.g0050.m0001	0.176467076	0.79004609	1	nonDEG
Bpev01.c0671.g0013.m0001	0.06659586	0.82421662	1	nonDEG
Bpev01.c0051.g0067.m0001	-0.254283764	0.3965787	0.72972424	nonDEG
Bpev01.c0321.g0017.m0001	0.442556948	0.15069957	0.42103489	nonDEG
Bpev01.c1159.g0010.m0001	0.103381388	0.87978396	1	nonDEG
Bpev01.c0944.g0007.m0001	-0.552500525	0.0698439	0.25236344	nonDEG
Bpev01.c0127.g0045.m0001	0.010418558	1	1	nonDEG
Bpev01.c0051.g0129.m0001	0.64089932	0.07033659	0.25359939	nonDEG
Bpev01.c0088.g0085.m0001	0.384305696	0.42611717	0.75824851	nonDEG
Bpev01.c0027.g0046.m0001	-0.744691506	0.06854549	0.24930945	nonDEG
Bpev01.c1087.g0002.m0001	-0.132103158	0.83135783	1	nonDEG
Bpev01.c0137.g0001.m0001	0.305597172	0.37746583	0.71507186	nonDEG
Bpev01.c0536.g0022.m0001	1.260325337	0.02433786	0.11947344	nonDEG
Bpev01.c0154.g0025.m0001	0.465341925	0.14258042	0.40576485	nonDEG
Bpev01.c1116.g0007.m0001	0.816354411	0.00919959	0.05804259	nonDEG
Bpev01.c1441.g0003.m0001	0.147708259	0.63852835	0.93020764	nonDEG
Bpev01.c1236.g0002.m0001	-0.05241861	1	1	nonDEG
Bpev01.c0092.g0019.m0001	1.02350828	0.237737	0.55476579	nonDEG
Bpev01.c1266.g0001.m0001	-0.029341582	0.92792544	1	nonDEG
Bpev01.c0828.g0003.m0001	0.924782764	0.03148735	0.14349163	nonDEG
Bpev01.c0517.g0034.m0001	-0.17835094	0.57824972	0.88939882	nonDEG
Bpev01.c0571.g0006.m0001	0.081364158	0.81787197	1	nonDEG

Bpev01.c1484.g0003.m0001	-0.674245116	0.06017861	0.22777041	nonDEG
Bpev01.c1828.g0001.m0002	-0.516578223	0.13430481	0.39034447	nonDEG
Bpev01.c0166.g0017.m0001	-0.341141532	0.29385104	0.62390493	nonDEG
Bpev01.c0191.g0018.m0001	-0.376025146	0.27255728	0.59847943	nonDEG
Bpev01.c0015.g0024.m0001	-4.604348074	0.25725635	0.57715056	nonDEG
Bpev01.c0022.g0071.m0001	1.2934618	0.01796094	0.09520203	nonDEG
Bpev01.c0813.g0012.m0001	-0.007210561	0.98173976	1	nonDEG
Bpev01.c0050.g0042.m0001	-0.73659083	0.02682654	0.12785278	nonDEG
Bpev01.c0759.g0004.m0001	1.397841217	0.03003224	0.13885959	nonDEG
Bpev01.c1427.g0006.m0001	-0.600984702	0.06600079	0.24272296	nonDEG
Bpev01.c0161.g0006.m0001	0.165179138	0.66238791	0.93220195	nonDEG
Bpev01.c0597.g0018.m0001	-0.761092462	0.029024	0.13557716	nonDEG
Bpev01.c0350.g0018.m0003	0.046438168	0.88569388	1	nonDEG
Bpev01.c2419.g0002.m0001	-1.268507352	0.13092637	0.38402191	nonDEG
Bpev01.c0457.g0009.m0001	-0.81564049	0.02214243	0.11116148	nonDEG
Bpev01.c0018.g0053.m0001	0.296794976	0.34212786	0.67862114	nonDEG
Bpev01.c1836.g0003.m0001	0.377109617	0.24252596	0.56014723	nonDEG
Bpev01.c1019.g0004.m0001	-0.278638746	0.52326198	0.8429132	nonDEG
Bpev01.c0616.g0021.m0001	-0.70979411	0.0227739	0.11353781	nonDEG
Bpev01.c0145.g0020.m0001	-1.242434989	0.20755209	0.51169299	nonDEG
Bpev01.c1544.g0004.m0001	0.441870977	0.30007238	0.63080769	nonDEG
Bpev01.c0383.g0010.m0001	0.755470959	0.06331438	0.23577968	nonDEG
Bpev01.c1120.g0006.m0001	-0.273429152	0.42027335	0.75175764	nonDEG
Bpev01.c0443.g0024.m0001	-3.135298191	0.66454518	0.93220195	nonDEG
Bpev01.c0146.g0048.m0001	-0.590518233	0.07511553	0.2646896	nonDEG
Bpev01.c0514.g0012.m0001	-0.295228261	0.44473852	0.77641329	nonDEG
Bpev01.c0442.g0036.m0001	-0.898926382	0.02793998	0.13187832	nonDEG
Bpev01.c0415.g0025.m0001	0.432619263	0.20139591	0.50244866	nonDEG
Bpev01.c1300.g0003.m0001	0.68139186	0.02642797	0.12679484	nonDEG
Bpev01.c0695.g0005.m0001	-0.543314645	0.07951692	0.27519107	nonDEG
Bpev01.c0488.g0012.m0001	0.364803545	0.24401758	0.561748	nonDEG
Bpev01.c0069.g0001.m0001	-0.725805619	0.02575688	0.12454941	nonDEG
Bpev01.c1519.g0013.m0001	-0.697182555	0.06134592	0.23082342	nonDEG
Bpev01.c2152.g0003.m0001	-0.308764647	0.33241021	0.66789336	nonDEG
Bpev01.c0195.g0011.m0001	-0.148319602	0.6362186	0.93006545	nonDEG
Bpev01.c0162.g0003.m0001	-0.393351248	0.21519731	0.52237947	nonDEG
Bpev01.c0579.g0025.m0001	-0.04620957	0.87969818	1	nonDEG
Bpev01.c1892.g0003.m0001	-0.752317551	0.02509767	0.12228843	nonDEG
Bpev01.c0605.g0021.m0001	0.601758437	0.07580118	0.26644908	nonDEG
Bpev01.c0222.g0004.m0001	-0.637869589	0.09405935	0.30729255	nonDEG
Bpev01.c0948.g0002.m0001	0.43237661	0.17523169	0.46332366	nonDEG
Bpev01.c0148.g0008.m0001	-0.183809402	0.65536539	0.93220195	nonDEG
Bpev01.c1333.g0004.m0001	-0.628806348	0.0707396	0.2544002	nonDEG

Bpev01.c2009.g0003.m0001	0.068163633	0.83598188	1	nonDEG
Bpev01.c0166.g0034.m0001	-0.110736055	0.76063096	0.99426612	nonDEG
Bpev01.c0000.g0242.m0002	-0.785647233	0.0116239	0.06900225	nonDEG
Bpev01.c1690.g0001.m0001	0.816502561	0.09033904	0.29905273	nonDEG
Bpev01.c0438.g0007.m0001	0.487373367	0.15802888	0.43419098	nonDEG
Bpev01.c0962.g0006.m0001	0.731875444	0.04660721	0.19061817	nonDEG
Bpev01.c0031.g0004.m0001	-0.460434048	0.61467554	0.91706395	nonDEG
Bpev01.c1280.g0008.m0002	-0.103426174	0.83956255	1	nonDEG
Bpev01.c0050.g0148.m0001	0.953675994	0.00885792	0.05638233	nonDEG
Bpev01.c0120.g0034.m0001	0.008907581	0.98041518	1	nonDEG
Bpev01.c1702.g0006.m0001	0.285247011	0.41605867	0.74817367	nonDEG
Bpev01.c1318.g0005.m0001	3.703905005	0.02015539	0.103462	nonDEG
Bpev01.c0022.g0141.m0001	-0.369077009	0.53591943	0.85377629	nonDEG
Bpev01.c0996.g0015.m0001	-0.548115674	0.07500864	0.26445271	nonDEG
Bpev01.c0281.g0079.m0001	0.009094688	0.97906802	1	nonDEG
Bpev01.c0366.g0009.m0001	0.594774376	0.05684218	0.21909705	nonDEG
Bpev01.c0001.g0017.m0001	-0.673205971	0.0775009	0.27049889	nonDEG
Bpev01.c0442.g0034.m0001	-0.672277471	0.05348347	0.21015748	nonDEG
Bpev01.c0913.g0005.m0001	-0.103637163	0.7445086	0.98465673	nonDEG
Bpev01.c0029.g0066.m0001	0.08750401	0.83391716	1	nonDEG
Bpev01.c0038.g0118.m0001	1.501468424	0.04532873	0.18688484	nonDEG
Bpev01.c0294.g0016.m0002	0.865837772	0.01249036	0.07307306	nonDEG
Bpev01.c0000.g0232.m0001	1.497227489	0.34542773	0.68193715	nonDEG
Bpev01.c0121.g0065.m0001	-0.657699094	0.10583008	0.33267824	nonDEG
Bpev01.c0032.g0012.m0001	-0.072766982	0.91566539	1	nonDEG
Bpev01.c0815.g0002.m0001	0.195662393	0.51532676	0.83552925	nonDEG
Bpev01.c0327.g0062.m0002	4.199656272	0.37584993	0.71439917	nonDEG
Bpev01.c0879.g0010.m0001	1.012813512	0.02927184	0.13626401	nonDEG
Bpev01.c1561.g0002.m0001	0.23258382	0.43943186	0.77148474	nonDEG
Bpev01.c0801.g0010.m0001	-0.066485708	0.86150133	1	nonDEG
Bpev01.c2007.g0004.m0001	0.289404956	0.48916322	0.81435641	nonDEG
Bpev01.c1559.g0001.m0001	0.319044849	0.32329575	0.65707396	nonDEG
Bpev01.c0127.g0050.m0001	-0.328730867	0.8573198	1	nonDEG
Bpev01.c1371.g0010.m0001	-0.579513139	0.15418417	0.42652018	nonDEG
Bpev01.c0733.g0003.m0001	-0.333610726	0.47535677	0.80398692	nonDEG
Bpev01.c0597.g0033.m0001	-0.088040773	0.83105591	1	nonDEG
Bpev01.c1171.g0020.m0001	0.579252181	0.18941475	0.48459033	nonDEG
Bpev01.c0052.g0070.m0001	0.386410516	0.22911383	0.54209436	nonDEG
Bpev01.c0430.g0007.m0001	-0.929141153	0.06415064	0.23800009	nonDEG
Bpev01.c0349.g0075.m0001	0.623216034	0.07826029	0.27226478	nonDEG
Bpev01.c0689.g0002.m0001	-0.419115743	0.1935143	0.4914058	nonDEG
Bpev01.c0540.g0024.m0001	0.127178644	0.85826313	1	nonDEG
Bpev01.c0015.g0151.m0001	-0.376668111	0.24128544	0.55858089	nonDEG

Bpev01.c1270.g0013.m0001	0.082373621	0.88391713	1	nonDEG
Bpev01.c1161.g0018.m0001	0.862203442	0.01524524	0.08448197	nonDEG
Bpev01.c0652.g0014.m0002	-0.654680179	0.1034417	0.32783078	nonDEG
Bpev01.c0281.g0095.m0001	-0.056264459	0.85596101	1	nonDEG
Bpev01.c1193.g0003.m0001	0.91924858	0.01155765	0.06881989	nonDEG
Bpev01.c2016.g0005.m0001	-1.536593124	0.13052981	0.38326584	nonDEG
Bpev01.c0177.g0046.m0001	2.303486793	0.06719054	0.24568655	nonDEG
Bpev01.c0241.g0001.m0001	-0.833517262	0.67633984	0.93896574	nonDEG
Bpev01.c0190.g0046.m0001	0.021438639	1	1	nonDEG
Bpev01.c0327.g0049.m0001	-0.713589408	0.02476423	0.12100151	nonDEG
Bpev01.c0329.g0004.m0001	0.165313024	0.59350529	0.90216359	nonDEG
Bpev01.c0768.g0008.m0001	-0.184428981	0.70460542	0.95975931	nonDEG
Bpev01.c1561.g0006.m0001	-0.812600135	0.02829101	0.13306119	nonDEG
Bpev01.c1040.g0017.m0001	-0.507061442	0.13378758	0.38934091	nonDEG
Bpev01.c0091.g0006.m0001	0.544856506	0.07846548	0.27270982	nonDEG
Bpev01.c0052.g0059.m0001	-0.08759048	1	1	nonDEG
Bpev01.c1557.g0009.m0001	0.170469697	1	1	nonDEG
Bpev01.c0279.g0023.m0001	-0.351239279	0.62351164	0.92299286	nonDEG
Bpev01.c0126.g0062.m0001	-1.641839372	0.05919301	0.22508177	nonDEG
Bpev01.c1602.g0010.m0001	0.401430225	0.19043205	0.48625732	nonDEG
Bpev01.c1662.g0004.m0001	-0.402350754	0.19856354	0.49867483	nonDEG
Bpev01.c0442.g0022.m0001	-0.116691879	0.74344915	0.98412999	nonDEG
Bpev01.c1762.g0003.m0001	0.061769234	0.84731609	1	nonDEG
Bpev01.c2315.g0004.m0002	0.237145578	0.49854622	0.82136323	nonDEG
Bpev01.c0803.g0006.m0001	0.200133167	0.50483492	0.82701846	nonDEG
Bpev01.c0050.g0002.m0002	0.108217012	0.80956841	1	nonDEG
Bpev01.c0536.g0006.m0001	-1.272233326	0.01266765	0.0738023	nonDEG
Bpev01.c0120.g0016.m0001	0.120210604	0.70903624	0.96281138	nonDEG
Bpev01.c0402.g0010.m0001	-0.017395668	0.95886028	1	nonDEG
Bpev01.c0168.g0008.m0001	-0.312129571	0.6035783	0.90856408	nonDEG
Bpev01.c0147.g0010.m0001	-0.680370923	0.03543678	0.15646599	nonDEG
Bpev01.c2464.g0002.m0001	-1.053314136	0.08425544	0.28566116	nonDEG
Bpev01.c2001.g0001.m0001	-0.685172144	0.02932558	0.13644705	nonDEG
Bpev01.c1534.g0004.m0001	-1.00147954	0.02158014	0.10912021	nonDEG
Bpev01.c1378.g0034.m0001	-0.213509482	0.5723434	0.88459239	nonDEG
Bpev01.c0042.g0014.m0001	0.965524281	0.00781515	0.05125202	nonDEG
Bpev01.c2330.g0002.m0001	-0.14090192	0.65743632	0.93220195	nonDEG
Bpev01.c0445.g0025.m0001	0.755712657	0.31127094	0.64183688	nonDEG
Bpev01.c1004.g0014.m0001	-0.027958108	0.92929514	1	nonDEG
Bpev01.c1847.g0001.m0001	0.079970143	0.82901113	1	nonDEG
Bpev01.c1116.g0009.m0001	-0.160919091	0.61815447	0.91944472	nonDEG
Bpev01.c0027.g0073.m0001	0.614146646	0.05878681	0.22397113	nonDEG
Bpev01.c0478.g0022.m0001	1.206438288	0.0488115	0.1968171	nonDEG

Bpev01.c1448.g0007.m0001	1.666522728	0.00766379	0.05054083	nonDEG
Bpev01.c2055.g0006.m0001	0.207921678	0.52223233	0.84176509	nonDEG
Bpev01.c1165.g0003.m0001	1.140314488	0.01307497	0.07551775	nonDEG
Bpev01.c1170.g0005.m0001	-0.704292767	0.05190868	0.2058377	nonDEG
Bpev01.c0757.g0004.m0001	-0.134716869	0.72367136	0.97270392	nonDEG
Bpev01.c0497.g0026.m0001	-0.508362061	0.38016819	0.71647704	nonDEG
Bpev01.c1085.g0008.m0001	-0.629364227	0.08114858	0.27908098	nonDEG
Bpev01.c0866.g0002.m0001	0.751373636	0.02289569	0.11397129	nonDEG
Bpev01.c0570.g0018.m0001	0.019419673	0.95118337	1	nonDEG
Bpev01.c2197.g0001.m0001	-0.441873555	0.16456906	0.44479881	nonDEG
Bpev01.c0237.g0021.m0001	-0.035957784	0.94178164	1	nonDEG
Bpev01.c0281.g0085.m0001	-0.547683729	0.11142534	0.34463655	nonDEG
Bpev01.c0083.g0022.m0001	-0.399488902	0.26103983	0.58306157	nonDEG
Bpev01.c0594.g0016.m0001	0.877960443	0.14598043	0.41209892	nonDEG
Bpev01.c0353.g0010.m0001	0.959030675	0.45981239	0.78878952	nonDEG
Bpev01.c0189.g0026.m0001	0.501222966	0.11459561	0.35145782	nonDEG
Bpev01.c1465.g0009.m0001	-0.220146354	0.79724263	1	nonDEG
Bpev01.c0582.g0014.m0001	1.116605579	0.46042637	0.7890797	nonDEG
Bpev01.c1623.g0009.m0001	-0.080598493	0.82822495	1	nonDEG
Bpev01.c0863.g0005.m0001	0.141503293	0.72001805	0.97015264	nonDEG
Bpev01.c0374.g0024.m0001	-1.62045858	0.13880522	0.39930983	nonDEG
Bpev01.c1238.g0012.m0001	-0.730586184	0.01613391	0.08822359	nonDEG
Bpev01.c2165.g0009.m0001	0.221611803	0.47243784	0.80076738	nonDEG
Bpev01.c0094.g0177.m0001	-0.319586323	0.6074089	0.91134552	nonDEG
Bpev01.c3157.g0002.m0001	0.025324492	0.95848865	1	nonDEG
Bpev01.c0848.g0023.m0001	-1.235340044	0.26302067	0.58561174	nonDEG
Bpev01.c1838.g0001.m0001	0.314391552	0.43619613	0.76870266	nonDEG
Bpev01.c2295.g0002.m0001	-1.159703001	0.02102935	0.10694128	nonDEG
Bpev01.c0704.g0008.m0001	-0.419388314	0.2042795	0.50672263	nonDEG
Bpev01.c2535.g0004.m0001	-0.36856095	0.46294234	0.79189771	nonDEG
Bpev01.c0652.g0019.m0001	0.302612326	0.50956155	0.83034333	nonDEG
Bpev01.c0929.g0007.m0001	0.532090695	0.10912708	0.33938222	nonDEG
Bpev01.c1315.g0010.m0002	0.748500902	0.12162806	0.36656517	nonDEG
Bpev01.c1074.g0004.m0001	-0.878303131	0.20315219	0.50513317	nonDEG
Bpev01.c1829.g0002.m0001	-0.775357694	0.21357764	0.52031714	nonDEG
Bpev01.c2029.g0001.m0001	0.489549597	0.4331691	0.7650168	nonDEG
Bpev01.c0015.g0231.m0001	0.8617738	0.01442206	0.0811024	nonDEG
Bpev01.c0001.g0049.m0001	0.696268532	0.13026391	0.38293861	nonDEG
Bpev01.c0281.g0048.m0001	-0.699790421	0.05308661	0.20912842	nonDEG
Bpev01.c0964.g0017.m0001	0.338650759	0.27317142	0.59928893	nonDEG
Bpev01.c0555.g0004.m0001	0.035272109	0.91972486	1	nonDEG
Bpev01.c0449.g0046.m0001	-0.543643843	0.13724344	0.39645658	nonDEG
Bpev01.c0016.g0006.m0001	1.284405131	0.02953954	0.13718481	nonDEG

Bpev01.c2445.g0001.m0001	-0.162245428	0.64015797	0.93095693	nonDEG
Bpev01.c0120.g0051.m0001	0	1	1	nonDEG
Bpev01.c1070.g0015.m0001	-0.265525301	0.37656401	0.7144404	nonDEG
Bpev01.c1331.g0001.m0001	0.102896907	0.76171405	0.99507876	nonDEG
Bpev01.c1163.g0016.m0001	-0.238045291	0.90146991	1	nonDEG
Bpev01.c0142.g0062.m0001	-0.606946901	0.07370883	0.2617508	nonDEG
Bpev01.c2609.g0001.m0001	-0.808281554	0.02782737	0.13151549	nonDEG
Bpev01.c0386.g0012.m0001	-0.681001252	0.02658792	0.12718912	nonDEG
Bpev01.c1463.g0002.m0001	0.747935997	0.02838584	0.13334143	nonDEG
Bpev01.c0051.g0201.m0001	-0.115795635	0.75582692	0.99092823	nonDEG
Bpev01.c1519.g0015.m0001	-0.320641373	0.31126659	0.64183688	nonDEG
Bpev01.c1202.g0030.m0001	-0.470761574	0.14562293	0.4113013	nonDEG
Bpev01.c0394.g0025.m0001	0.752182246	0.0132329	0.07618748	nonDEG
Bpev01.c1838.g0006.m0003	0.150851951	0.64365214	0.93220195	nonDEG
Bpev01.c1922.g0002.m0001	-0.876958245	0.34670802	0.6834106	nonDEG
Bpev01.c0000.g0060.m0001	0.497162882	0.12674039	0.37612809	nonDEG
Bpev01.c0001.g0007.m0001	0.731864628	0.16408861	0.44378545	nonDEG
Bpev01.c0649.g0010.m0001	-0.546259647	0.09000366	0.29858211	nonDEG
Bpev01.c0157.g0020.m0001	-0.28616647	0.40344811	0.73663587	nonDEG
Bpev01.c0089.g0046.m0001	0.321747724	0.32675044	0.66152301	nonDEG
Bpev01.c1070.g0013.m0001	-0.056163394	0.87821214	1	nonDEG
Bpev01.c1009.g0005.m0001	-0.395664901	0.19475559	0.4932157	nonDEG
Bpev01.c0052.g0183.m0001	-0.047064088	0.90298541	1	nonDEG
Bpev01.c0510.g0002.m0001	0.341935209	0.4163103	0.74822626	nonDEG
Bpev01.c3935.g0002.m0001	-0.227556404	0.72767618	0.97507154	nonDEG
Bpev01.c2152.g0004.m0001	-0.038694287	0.89984575	1	nonDEG
Bpev01.c2813.g0003.m0001	0.100943976	0.75626926	0.99113256	nonDEG
Bpev01.c1806.g0003.m0001	0.96869992	0.07407776	0.26277565	nonDEG
Bpev01.c0455.g0026.m0001	-0.455476699	0.2104581	0.51604832	nonDEG
Bpev01.c0455.g0023.m0001	-1.313901857	0.0438126	0.1831841	nonDEG
Bpev01.c0355.g0003.m0001	0.415724338	0.3014115	0.63277653	nonDEG
Bpev01.c0324.g0021.m0001	0.200226076	0.540196	0.85759119	nonDEG
Bpev01.c1167.g0015.m0001	-0.819057457	0.15257188	0.4239862	nonDEG
Bpev01.c0190.g0045.m0001	-0.45623231	0.38564162	0.71909228	nonDEG
Bpev01.c0121.g0033.m0001	2.01187599	0.05322559	0.20950772	nonDEG
Bpev01.c0082.g0081.m0001	-0.018594718	0.9529782	1	nonDEG
Bpev01.c0080.g0101.m0001	0.031018085	0.91961023	1	nonDEG
Bpev01.c0190.g0071.m0001	-0.314772726	0.31029188	0.64062575	nonDEG
Bpev01.c0089.g0065.m0001	-0.536814957	0.09536061	0.31021634	nonDEG
Bpev01.c0400.g0003.m0001	-3.670528638	0.41410862	0.74697103	nonDEG
Bpev01.c0082.g0073.m0001	-0.988540506	0.20716114	0.5108147	nonDEG
Bpev01.c0051.g0153.m0001	0.766173544	0.0314726	0.14349107	nonDEG
Bpev01.c0800.g0013.m0001	0.287210495	0.43151392	0.76319245	nonDEG

Bpev01.c2297.g0001.m0001	0.83493329	0.05424082	0.21211419	nonDEG
Bpev01.c0468.g0007.m0001	4.396819881	0.01811447	0.09580882	nonDEG
Bpev01.c0031.g0013.m0001	-0.033149945	0.91598066	1	nonDEG
Bpev01.c0032.g0010.m0001	0.788087977	0.2312038	0.54525336	nonDEG
Bpev01.c2048.g0004.m0001	-0.359003197	0.33777151	0.67359813	nonDEG
Bpev01.c1550.g0007.m0001	0.464846047	0.23531916	0.55138179	nonDEG
Bpev01.c1233.g0008.m0001	0.599879012	0.05226975	0.20684231	nonDEG
Bpev01.c0284.g0020.m0001	-0.397638845	0.32315687	0.65695843	nonDEG
Bpev01.c0247.g0005.m0001	0.417618455	0.22851471	0.54112329	nonDEG
Bpev01.c1206.g0008.m0001	-0.067018188	0.85465795	1	nonDEG
Bpev01.c2423.g0002.m0001	0.145418218	0.63759247	0.93006545	nonDEG
Bpev01.c0134.g0064.m0001	0.708702549	0.03900291	0.1681615	nonDEG
Bpev01.c0702.g0012.m0001	-0.54127729	0.08809133	0.29412731	nonDEG
Bpev01.c0044.g0048.m0001	0.179847135	0.70866253	0.96268359	nonDEG
Bpev01.c0088.g0022.m0001	0.235368015	0.47724035	0.80573889	nonDEG
Bpev01.c0356.g0039.m0001	0.876493226	0.04406447	0.18379341	nonDEG
Bpev01.c0027.g0106.m0001	-0.716575233	0.02455503	0.12017019	nonDEG
Bpev01.c0089.g0051.m0001	-0.405016354	0.18020434	0.47105626	nonDEG
Bpev01.c0038.g0112.m0001	-0.22407207	0.58850504	0.89822004	nonDEG
Bpev01.c1206.g0021.m0001	0.721543602	0.01555862	0.08582436	nonDEG
Bpev01.c1901.g0002.m0001	-0.369301415	0.26894554	0.59382642	nonDEG
Bpev01.c0848.g0018.m0001	-0.050390853	0.88873859	1	nonDEG
Bpev01.c0990.g0005.m0001	-0.871365534	0.01509754	0.08398649	nonDEG
Bpev01.c0594.g0019.m0001	0.674474462	0.03072883	0.14097338	nonDEG
Bpev01.c0082.g0022.m0001	-0.651006015	0.03527066	0.15604642	nonDEG
Bpev01.c1176.g0011.m0001	-0.867041934	0.01006671	0.0621157	nonDEG
Bpev01.c2093.g0001.m0001	0.771163651	0.0822113	0.28118944	nonDEG
Bpev01.c1178.g0003.m0001	0.331371996	0.29714699	0.62734582	nonDEG
Bpev01.c0477.g0024.m0001	0.014833064	0.96273595	1	nonDEG
Bpev01.c1202.g0013.m0001	0.506566922	0.12188372	0.3671853	nonDEG
Bpev01.c0000.g0231.m0001	0.820594438	0.08600919	0.28953968	nonDEG
Bpev01.c0480.g0067.m0001	-0.178847579	0.58217702	0.8927869	nonDEG
Bpev01.c2046.g0003.m0001	1.705666721	0.05776377	0.22165116	nonDEG
Bpev01.c0954.g0008.m0001	-0.231277448	0.63552696	0.93006545	nonDEG
Bpev01.c0607.g0003.m0001	-2.010571119	0.01282467	0.07449588	nonDEG
Bpev01.c0090.g0018.m0001	0.949395555	0.03193503	0.1448814	nonDEG
Bpev01.c0029.g0144.m0001	0.248260827	0.53035438	0.84962001	nonDEG
Bpev01.c1225.g0009.m0002	-0.612429699	0.04986067	0.19987042	nonDEG
Bpev01.c0000.g0114.m0001	0.566770476	0.05900233	0.22453072	nonDEG
Bpev01.c0038.g0156.m0001	0.111578349	0.72344989	0.97258386	nonDEG
Bpev01.c0169.g0053.m0001	0.232991347	0.44794707	0.77938025	nonDEG
Bpev01.c1136.g0010.m0001	-0.30959793	0.34094097	0.67698375	nonDEG
Bpev01.c2320.g0002.m0001	0.541004402	0.0865521	0.29063616	nonDEG

Bpev01.c1544.g0005.m0001	-0.859728839	0.02165376	0.10936095	nonDEG
Bpev01.c0486.g0011.m0001	0.10350091	0.73615989	0.97977421	nonDEG
Bpev01.c0511.g0004.m0001	1.330978857	0.07125845	0.25582653	nonDEG
Bpev01.c0147.g0024.m0001	-0.441858297	0.15927728	0.43629139	nonDEG
Bpev01.c0188.g0024.m0001	0.649580699	0.05722057	0.22019823	nonDEG
Bpev01.c0501.g0003.m0001	-0.71162232	0.04591221	0.18850902	nonDEG
Bpev01.c0645.g0034.m0001	-0.487241858	0.11680307	0.35592231	nonDEG
Bpev01.c0488.g0016.m0001	0.162452689	0.60261426	0.90808565	nonDEG
Bpev01.c0215.g0010.m0001	-0.861334966	0.01947688	0.10092939	nonDEG
Bpev01.c0640.g0015.m0001	-0.576876846	0.13059014	0.38329442	nonDEG
Bpev01.c0481.g0004.m0001	-0.628485077	0.04387965	0.18318623	nonDEG
Bpev01.c0094.g0098.m0001	-0.590693669	0.04818269	0.1949047	nonDEG
Bpev01.c0574.g0001.m0001	0.255208054	0.78538626	1	nonDEG
Bpev01.c0206.g0011.m0001	0.364003078	0.27018014	0.59556968	nonDEG
Bpev01.c0572.g0003.m0001	-0.080217236	0.8012463	1	nonDEG
Bpev01.c0042.g0012.m0001	-0.385824755	0.19518142	0.49393501	nonDEG
Bpev01.c0470.g0025.m0001	-0.056815295	0.89783256	1	nonDEG
Bpev01.c1159.g0012.m0001	0.321338429	0.28262706	0.60945342	nonDEG
Bpev01.c2535.g0001.m0001	-0.633550198	0.3371741	0.67286254	nonDEG
Bpev01.c0797.g0014.m0001	-0.06333063	0.83178408	1	nonDEG
Bpev01.c0862.g0001.m0002	0.103732077	0.78294006	1	nonDEG
Bpev01.c0629.g0028.m0002	-0.20545334	0.5546665	0.8700819	nonDEG
Bpev01.c1116.g0006.m0001	-0.132457229	0.66075376	0.93220195	nonDEG
Bpev01.c0094.g0074.m0001	0.69952565	0.60220805	0.90770583	nonDEG
Bpev01.c0245.g0054.m0001	0.043791869	0.89905374	1	nonDEG
Bpev01.c0658.g0005.m0002	-0.67815949	0.04281122	0.18004566	nonDEG
Bpev01.c0213.g0047.m0001	-0.781704021	0.0173523	0.09301428	nonDEG
Bpev01.c0702.g0014.m0001	-0.267258463	0.4000027	0.7329942	nonDEG
Bpev01.c0777.g0009.m0001	0.667651181	0.04258705	0.17930241	nonDEG
Bpev01.c1873.g0005.m0001	1.639422884	0.0143635	0.08086574	nonDEG
Bpev01.c2194.g0003.m0001	0.205674103	0.5828824	0.89339694	nonDEG
Bpev01.c2158.g0001.m0001	-0.65580483	0.04440237	0.18478389	nonDEG
Bpev01.c1104.g0008.m0001	-0.033660869	0.91061638	1	nonDEG
Bpev01.c0867.g0007.m0001	-0.713468721	0.18145773	0.47301854	nonDEG
Bpev01.c0546.g0012.m0001	2.079234182	0.41304829	0.74651835	nonDEG
Bpev01.c0702.g0015.m0001	0.343073979	0.26133602	0.58342263	nonDEG
Bpev01.c0015.g0266.m0001	0.608176435	0.07624587	0.26766242	nonDEG
Bpev01.c0395.g0015.m0001	0.206083246	0.49599957	0.81925017	nonDEG
Bpev01.c0333.g0022.m0001	-0.419862438	0.25935032	0.58030053	nonDEG
Bpev01.c2235.g0007.m0001	-0.271337855	0.43994911	0.77198915	nonDEG
Bpev01.c1520.g0001.m0001	-0.53307693	0.12820967	0.37933311	nonDEG
Bpev01.c0051.g0058.m0001	-1.17793638	0.05693846	0.21931327	nonDEG
Bpev01.c0154.g0073.m0001	-0.285504407	0.39066568	0.72351611	nonDEG

Bpev01.c0374.g0022.m0001	-0.766695845	0.01216231	0.0715663	nonDEG
Bpev01.c0062.g0067.m0001	-0.578312227	0.07896436	0.2737115	nonDEG
Bpev01.c0088.g0019.m0001	0.285792094	0.46338217	0.7923187	nonDEG
Bpev01.c0510.g0011.m0001	-0.511757843	0.10321627	0.32736675	nonDEG
Bpev01.c1239.g0008.m0001	0.156772917	0.81589027	1	nonDEG
Bpev01.c0925.g0003.m0001	-0.50392497	0.12058331	0.36444117	nonDEG
Bpev01.c0066.g0060.m0001	-0.61712593	0.14284969	0.40624346	nonDEG
Bpev01.c0134.g0047.m0001	-0.479492293	0.26080974	0.58259185	nonDEG
Bpev01.c0051.g0019.m0001	0.062821243	0.88059268	1	nonDEG
Bpev01.c0617.g0012.m0001	0.693824263	0.02424312	0.11909843	nonDEG
Bpev01.c0848.g0042.m0001	-0.279382727	0.40113123	0.73397367	nonDEG
Bpev01.c1140.g0009.m0001	0.03206692	0.92677875	1	nonDEG
Bpev01.c0404.g0026.m0001	-0.72593846	0.03337046	0.14965256	nonDEG
Bpev01.c0929.g0016.m0001	0.818324793	0.00864639	0.05534706	nonDEG
Bpev01.c0214.g0022.m0001	0.484917039	0.13134554	0.3848976	nonDEG
Bpev01.c0570.g0012.m0001	-0.474301477	0.26907735	0.59394212	nonDEG
Bpev01.c1397.g0005.m0001	-0.720760051	0.53729815	0.85481627	nonDEG
Bpev01.c0169.g0038.m0001	-3.299999448	0.06501379	0.24020342	nonDEG
Bpev01.c0237.g0004.m0001	-0.100060695	0.88721582	1	nonDEG
Bpev01.c2153.g0005.m0001	2.824823899	0.04360804	0.18248431	nonDEG
Bpev01.c2220.g0001.m0001	-1.476493505	0.02070332	0.10555679	nonDEG
Bpev01.c0027.g0154.m0001	1.587606532	0.01586438	0.08718152	nonDEG
Bpev01.c0126.g0005.m0001	-0.833987559	0.01017307	0.06260139	nonDEG
Bpev01.c1188.g0004.m0001	0.269574982	0.37292416	0.71278108	nonDEG
Bpev01.c0957.g0001.m0001	-0.13324463	0.69200874	0.95018868	nonDEG
Bpev01.c1862.g0001.m0001	-0.039160811	0.89695873	1	nonDEG
Bpev01.c1176.g0003.m0001	0.473246207	0.49370357	0.81748914	nonDEG
Bpev01.c0843.g0010.m0002	-0.509638255	0.09348466	0.30595829	nonDEG
Bpev01.c0082.g0087.m0001	0.232577153	0.48601689	0.81287415	nonDEG
Bpev01.c0037.g0013.m0001	1.087248483	0.48473059	0.81256284	nonDEG
Bpev01.c0598.g0013.m0001	-0.127079035	1	1	nonDEG
Bpev01.c0365.g0027.m0001	-0.341311258	0.77227898	1	nonDEG
Bpev01.c1235.g0001.m0001	0.905677611	0.06144097	0.23109246	nonDEG
Bpev01.c1274.g0002.m0001	0.444995758	0.21263135	0.51899048	nonDEG
Bpev01.c0496.g0014.m0001	-0.391783818	0.42589695	0.75804164	nonDEG
Bpev01.c0245.g0019.m0001	-0.023806373	0.9499617	1	nonDEG
Bpev01.c0001.g0044.m0001	-0.816049696	0.23268547	0.54752038	nonDEG
Bpev01.c0052.g0215.m0001	0.069605261	0.83085315	1	nonDEG
Bpev01.c0044.g0005.m0001	0.941476751	0.03029558	0.13963314	nonDEG
Bpev01.c0401.g0004.m0001	-0.061293079	0.84460993	1	nonDEG
Bpev01.c1081.g0008.m0001	-0.205560075	0.51464478	0.83497534	nonDEG
Bpev01.c1217.g0008.m0001	1.598329013	0.01203915	0.07095507	nonDEG
Bpev01.c1024.g0003.m0001	1.01218018	0.27380498	0.59986949	nonDEG

Bpev01.c1185.g0017.m0001	-0.262406633	0.38498221	0.71876591	nonDEG
Bpev01.c0163.g0037.m0001	-0.648974386	0.3033269	0.635128	nonDEG
Bpev01.c0029.g0158.m0001	-0.2441062	0.44550518	0.77717359	nonDEG
Bpev01.c1833.g0007.m0001	-0.543344733	0.20620127	0.5092156	nonDEG
Bpev01.c2366.g0005.m0001	1.905455283	0.63679809	0.93006545	nonDEG
Bpev01.c0114.g0001.m0001	1.918564811	0.10147472	0.32361762	nonDEG
Bpev01.c0488.g0036.m0001	0.981244874	0.25894302	0.57956538	nonDEG
Bpev01.c1260.g0006.m0001	-0.083745895	0.81264867	1	nonDEG
Bpev01.c0042.g0072.m0001	-0.646135793	0.38607083	0.71914078	nonDEG
Bpev01.c1475.g0003.m0001	-0.808461428	0.01495353	0.083406	nonDEG
Bpev01.c0366.g0025.m0001	0.461813265	0.69515136	0.95294812	nonDEG
Bpev01.c0558.g0008.m0001	-0.32349449	0.29268101	0.62230737	nonDEG
Bpev01.c0506.g0006.m0001	-0.679291516	0.0363881	0.1596705	nonDEG
Bpev01.c0299.g0010.m0001	-0.657895804	0.04462828	0.1852523	nonDEG
Bpev01.c0219.g0009.m0001	-0.320062172	0.35916987	0.69746713	nonDEG
Bpev01.c1401.g0014.m0001	0.545866917	0.08394213	0.28495524	nonDEG
Bpev01.c0134.g0146.m0003	0.39732413	0.20393604	0.5062954	nonDEG
Bpev01.c0726.g0004.m0001	0.100221911	0.94706979	1	nonDEG
Bpev01.c0455.g0004.m0001	1.112521632	0.16607996	0.44756755	nonDEG
Bpev01.c0274.g0030.m0001	0.841535399	0.04805709	0.19462824	nonDEG
Bpev01.c1378.g0010.m0001	-0.286126955	0.40946962	0.74327143	nonDEG
Bpev01.c0264.g0035.m0001	0.56536371	0.11261409	0.34736444	nonDEG
Bpev01.c0588.g0011.m0001	-0.471471822	0.26028043	0.58198356	nonDEG
Bpev01.c1395.g0004.m0001	0.224461343	0.47081094	0.79957179	nonDEG
Bpev01.c0162.g0055.m0001	0.168496715	0.71504116	0.96715353	nonDEG
Bpev01.c1551.g0001.m0001	-0.277149131	0.46261572	0.79161499	nonDEG
Bpev01.c1382.g0014.m0001	-0.343908776	0.59952818	0.90617191	nonDEG
Bpev01.c0298.g0043.m0003	0.728681156	0.03415035	0.15227867	nonDEG
Bpev01.c0261.g0015.m0001	-0.253109916	0.45401328	0.78418781	nonDEG
Bpev01.c0016.g0092.m0001	0.088856065	0.76700653	0.99840401	nonDEG
Bpev01.c1953.g0001.m0001	0.306887799	0.31961063	0.65279483	nonDEG
Bpev01.c0286.g0032.m0001	0.411254519	0.42088057	0.75234196	nonDEG
Bpev01.c0506.g0025.m0001	-0.033125188	0.91949636	1	nonDEG
Bpev01.c1736.g0007.m0001	1.932617135	0.2376621	0.55475218	nonDEG
Bpev01.c0340.g0005.m0001	0.197092083	0.59825571	0.90541075	nonDEG
Bpev01.c1583.g0004.m0001	0.318514801	0.50183781	0.82432782	nonDEG
Bpev01.c0565.g0019.m0001	-0.190469098	0.61018923	0.91365448	nonDEG
Bpev01.c0420.g0017.m0001	0.5305303	0.08320502	0.28333981	nonDEG
Bpev01.c0390.g0009.m0001	-0.451653145	0.24568667	0.56413461	nonDEG
Bpev01.c0299.g0007.m0001	-0.138873566	0.64840565	0.93220195	nonDEG
Bpev01.c0457.g0012.m0001	0.141075039	0.64297626	0.93220195	nonDEG
Bpev01.c0480.g0082.m0002	0.260771633	0.3834071	0.71773634	nonDEG
Bpev01.c0567.g0008.m0001	0.797644942	0.28151505	0.60830961	nonDEG

Bpev01.c2919.g0001.m0001	1.012388133	0.38963448	0.72265973	nonDEG
Bpev01.c0497.g0004.m0001	-0.505012712	0.42713422	0.7591864	nonDEG
Bpev01.c1535.g0001.m0001	0.782899283	0.24409425	0.56179272	nonDEG
Bpev01.c0158.g0066.m0001	-0.282231569	0.63634888	0.93006545	nonDEG
Bpev01.c0281.g0001.m0001	0.695952601	0.04103465	0.17446839	nonDEG
Bpev01.c1180.g0003.m0001	-0.709473876	0.02582385	0.12479126	nonDEG
Bpev01.c0190.g0020.m0001	0.549132805	0.09621793	0.31184627	nonDEG
Bpev01.c1040.g0047.m0001	-0.236699198	0.50533324	0.82752991	nonDEG
Bpev01.c0781.g0007.m0001	-0.639020678	0.06636256	0.24368808	nonDEG
Bpev01.c0744.g0011.m0001	-0.385143189	0.21965044	0.52807588	nonDEG
Bpev01.c1132.g0001.m0001	-0.291786569	0.35659892	0.69471832	nonDEG
Bpev01.c0484.g0032.m0001	-0.675873047	0.70769147	0.96190825	nonDEG
Bpev01.c0037.g0006.m0001	-0.846489986	0.04517072	0.18642657	nonDEG
Bpev01.c0442.g0028.m0001	-0.654339296	0.57737202	0.88873134	nonDEG
Bpev01.c0427.g0012.m0001	-0.852165387	0.06254757	0.2338801	nonDEG
Bpev01.c0025.g0022.m0001	0.743393123	0.02933433	0.13644705	nonDEG
Bpev01.c0427.g0015.m0001	-0.279997722	0.39463582	0.72783385	nonDEG
Bpev01.c0439.g0007.m0001	0.255364387	0.41946273	0.75119758	nonDEG
Bpev01.c0052.g0055.m0001	-0.182837882	0.56012953	0.87492191	nonDEG
Bpev01.c0022.g0068.m0001	-1.101298079	0.01085484	0.06578521	nonDEG
Bpev01.c0841.g0003.m0001	1.189808984	0.05352137	0.21020399	nonDEG
Bpev01.c0000.g0059.m0001	-0.383509207	0.30455727	0.6359669	nonDEG
Bpev01.c0666.g0007.m0001	0.290682383	0.55400738	0.86941859	nonDEG
Bpev01.c0245.g0063.m0001	-0.400955436	0.17757925	0.46630592	nonDEG
Bpev01.c0224.g0050.m0001	-0.064505007	0.83084579	1	nonDEG
Bpev01.c0126.g0019.m0001	1.134654231	0.01217414	0.07157866	nonDEG
Bpev01.c0129.g0082.m0001	-1.470769929	0.02190095	0.11024999	nonDEG
Bpev01.c0083.g0016.m0001	0.719124536	0.02263537	0.11294281	nonDEG
Bpev01.c0349.g0001.m0001	0.081532095	0.79288303	1	nonDEG
Bpev01.c1302.g0001.m0001	1.699265936	0.05382511	0.21096492	nonDEG
Bpev01.c2187.g0004.m0001	-0.267402746	0.38310704	0.71761551	nonDEG
Bpev01.c0089.g0033.m0001	-0.591638037	0.07567566	0.26619855	nonDEG
Bpev01.c1328.g0008.m0001	-0.172646756	0.57949074	0.89058447	nonDEG
Bpev01.c0159.g0004.m0001	0.012841778	0.96618083	1	nonDEG
Bpev01.c0383.g0027.m0001	-0.395749712	0.27653913	0.60339722	nonDEG
Bpev01.c0225.g0003.m0001	0.065917229	0.86722	1	nonDEG
Bpev01.c0714.g0005.m0001	-0.044377656	0.94164591	1	nonDEG
Bpev01.c0101.g0041.m0001	-0.521141903	0.08300365	0.28288313	nonDEG
Bpev01.c0134.g0027.m0001	0.636589846	0.30689988	0.63779608	nonDEG
Bpev01.c1519.g0010.m0001	0.452140064	0.19327418	0.49088073	nonDEG
Bpev01.c0052.g0149.m0001	0.696153686	0.24239318	0.5601147	nonDEG
Bpev01.c1281.g0010.m0001	-0.840844213	0.05261348	0.20782076	nonDEG
Bpev01.c0848.g0014.m0001	-0.403710574	0.2107204	0.51645208	nonDEG

Bpev01.c0720.g0002.m0001	0.658022361	0.07137514	0.2560304	nonDEG
Bpev01.c0349.g0026.m0001	0.54780348	0.15248739	0.4239377	nonDEG
Bpev01.c0915.g0020.m0001	0.03107017	0.92096331	1	nonDEG
Bpev01.c1418.g0008.m0001	-0.687563176	0.16625104	0.44786459	nonDEG
Bpev01.c0383.g0026.m0001	0.391980789	0.43825999	0.77026483	nonDEG
Bpev01.c0518.g0046.m0001	0.842144135	0.00811427	0.052732	nonDEG
Bpev01.c1992.g0001.m0001	-0.099187629	0.88871319	1	nonDEG
Bpev01.c0280.g0030.m0001	-0.609353601	0.04130975	0.17532301	nonDEG
Bpev01.c0171.g0041.m0001	0.092525992	0.77629237	1	nonDEG
Bpev01.c0000.g0192.m0001	-0.216774874	0.49107282	0.81526453	nonDEG
Bpev01.c0267.g0028.m0001	0.505918025	0.1527465	0.42431835	nonDEG
Bpev01.c2473.g0004.m0002	0.112646651	0.80436402	1	nonDEG
Bpev01.c0135.g0075.m0001	0.186655455	0.54631312	0.86267833	nonDEG
Bpev01.c0150.g0002.m0001	0.280041066	0.36610704	0.70530864	nonDEG
Bpev01.c1981.g0010.m0001	-0.066613584	0.88116301	1	nonDEG
Bpev01.c1197.g0002.m0001	0.790623847	0.03175591	0.14424659	nonDEG
Bpev01.c0045.g0027.m0001	0.152510403	0.79193928	1	nonDEG
Bpev01.c0389.g0027.m0001	0.449040831	0.14076788	0.40219561	nonDEG
Bpev01.c0505.g0001.m0001	0.226708539	0.48283658	0.81159222	nonDEG
Bpev01.c0043.g0004.m0001	-0.256920309	0.43775923	0.76990532	nonDEG
Bpev01.c0286.g0014.m0001	0.786552153	0.02518519	0.12259039	nonDEG
Bpev01.c1378.g0030.m0001	-0.04615007	0.89564814	1	nonDEG
Bpev01.c0668.g0002.m0001	0.79636768	0.0121694	0.07157866	nonDEG
Bpev01.c0169.g0019.m0001	0.404194161	0.33741921	0.67312253	nonDEG
Bpev01.c1029.g0005.m0001	0.100152782	0.79706564	1	nonDEG
Bpev01.c0022.g0099.m0002	0.137753784	0.65464384	0.93220195	nonDEG
Bpev01.c0016.g0180.m0001	0.417811487	0.1706533	0.45563781	nonDEG
Bpev01.c0264.g0039.m0001	0.278831776	0.50669829	0.82857808	nonDEG
Bpev01.c0688.g0011.m0001	-1.541052052	0.00861759	0.05523476	nonDEG
Bpev01.c0579.g0019.m0001	0.691983568	0.02785839	0.13161984	nonDEG
Bpev01.c0449.g0045.m0001	0.167604716	0.84092223	1	nonDEG
Bpev01.c1236.g0006.m0001	-1.268725185	0.05479699	0.21372132	nonDEG
Bpev01.c0038.g0068.m0001	-0.22706535	0.51642044	0.83669007	nonDEG
Bpev01.c0027.g0061.m0001	0.888644529	0.10735305	0.33563789	nonDEG
Bpev01.c1202.g0005.m0001	-0.48320126	0.13609993	0.39396715	nonDEG
Bpev01.c0877.g0002.m0001	0.392061129	0.22084936	0.52987883	nonDEG
Bpev01.c0452.g0021.m0001	-0.898737444	0.026479	0.12692086	nonDEG
Bpev01.c0778.g0012.m0001	-0.35099012	0.48565823	0.81271092	nonDEG
Bpev01.c0155.g0053.m0001	0.491687037	0.11163669	0.34518146	nonDEG
Bpev01.c0575.g0005.m0001	-0.679851251	0.54002575	0.85748749	nonDEG
Bpev01.c0298.g0014.m0001	-0.250544835	0.44152084	0.77304678	nonDEG
Bpev01.c2873.g0003.m0001	0.39790684	0.47319832	0.80147778	nonDEG
Bpev01.c2434.g0001.m0001	1.265847309	0.29589088	0.6260408	nonDEG

Bpev01.c1046.g0002.m0001	-0.414953881	0.17743258	0.46621486	nonDEG
Bpev01.c0671.g0019.m0001	-0.287619875	0.62335601	0.92299286	nonDEG
Bpev01.c1769.g0007.m0001	1.046938627	0.84464804	1	nonDEG
Bpev01.c0732.g0006.m0001	3.124841669	0.02924433	0.13617901	nonDEG
Bpev01.c1206.g0018.m0001	-0.551038693	0.08118989	0.2791578	nonDEG
Bpev01.c0972.g0002.m0001	0.808198487	0.01149399	0.06855179	nonDEG
Bpev01.c0324.g0010.m0001	-0.446362982	0.51208495	0.8329338	nonDEG
Bpev01.c0391.g0007.m0001	1.004934329	0.24057746	0.5577979	nonDEG
Bpev01.c0639.g0027.m0001	1.332725035	0.58353026	0.89397103	nonDEG
Bpev01.c0418.g0001.m0001	-0.654748158	0.07701439	0.26944434	nonDEG
Bpev01.c0280.g0033.m0001	0.473189905	0.25334431	0.57463619	nonDEG
Bpev01.c1957.g0004.m0002	-0.268928255	0.3802625	0.71647704	nonDEG
Bpev01.c0614.g0011.m0001	-0.750211084	0.01538183	0.0850856	nonDEG
Bpev01.c0940.g0006.m0001	0.074981194	0.80323898	1	nonDEG
Bpev01.c0452.g0004.m0001	0.061523422	0.88256682	1	nonDEG
Bpev01.c1467.g0001.m0001	-1.663077374	0.01295486	0.07497683	nonDEG
Bpev01.c0522.g0044.m0001	1.646891743	0.14484044	0.41011424	nonDEG
Bpev01.c1185.g0004.m0001	-1.215564375	0.01677114	0.09073362	nonDEG
Bpev01.c0574.g0031.m0001	-0.838489295	0.01149239	0.06855179	nonDEG
Bpev01.c0464.g0034.m0001	0.581628874	0.64460928	0.93220195	nonDEG
Bpev01.c0243.g0050.m0001	0.097667578	0.75564592	0.99088595	nonDEG
Bpev01.c0428.g0035.m0001	-0.170282043	0.69545807	0.95310228	nonDEG
Bpev01.c0759.g0003.m0001	0.779719502	0.06577055	0.24208791	nonDEG
Bpev01.c0134.g0069.m0001	0.142491801	0.65619037	0.93220195	nonDEG
Bpev01.c0027.g0107.m0001	0.265852094	0.41735207	0.74895397	nonDEG
Bpev01.c2165.g0010.m0001	0.937352594	0.00794881	0.0520011	nonDEG
Bpev01.c0230.g0030.m0001	-0.291995345	0.58246456	0.89297875	nonDEG
Bpev01.c0505.g0005.m0001	0.43903073	0.33178671	0.66741975	nonDEG
Bpev01.c0261.g0024.m0002	0.479532531	0.17652381	0.46487724	nonDEG
Bpev01.c1684.g0005.m0001	-0.333522015	0.26723001	0.59154706	nonDEG
Bpev01.c0283.g0013.m0001	-0.185944494	0.55906279	0.87418332	nonDEG
Bpev01.c0191.g0038.m0001	-0.261994367	0.3832495	0.7176976	nonDEG
Bpev01.c2630.g0001.m0001	0.193433353	0.59209981	0.90105072	nonDEG
Bpev01.c0959.g0004.m0001	-0.139535271	0.65330034	0.93220195	nonDEG
Bpev01.c0088.g0132.m0001	0.089889319	0.79820729	1	nonDEG
Bpev01.c0051.g0084.m0001	0.51616785	0.12149953	0.36629029	nonDEG
Bpev01.c0142.g0061.m0001	-0.394378666	0.50374513	0.82626213	nonDEG
Bpev01.c0841.g0007.m0001	0.227673423	0.52988182	0.84927865	nonDEG
Bpev01.c0784.g0006.m0001	0.764912297	0.30425309	0.63589769	nonDEG
Bpev01.c1087.g0012.m0001	0.142518599	0.71877938	0.9695398	nonDEG
Bpev01.c0557.g0004.m0001	-0.256964598	0.41110487	0.74500233	nonDEG
Bpev01.c1072.g0003.m0001	0.040344781	0.90599677	1	nonDEG
Bpev01.c1688.g0003.m0001	-0.072505749	0.90063127	1	nonDEG

Bpev01.c0051.g0020.m0001	0.134928149	0.67619075	0.93880303	nonDEG
Bpev01.c0320.g0008.m0001	0.061698029	0.86653493	1	nonDEG
Bpev01.c2334.g0001.m0001	-0.422905829	0.16960101	0.45380015	nonDEG
Bpev01.c2326.g0003.m0001	-0.668412924	0.10294772	0.32689871	nonDEG
Bpev01.c0000.g0050.m0001	-0.062012557	0.8519342	1	nonDEG
Bpev01.c3346.g0002.m0001	2.068394622	0.29667243	0.62683842	nonDEG
Bpev01.c0147.g0022.m0001	-0.655852632	0.06725264	0.24588306	nonDEG
Bpev01.c0848.g0024.m0001	0.43669935	0.27605254	0.60282701	nonDEG
Bpev01.c1431.g0004.m0001	-0.331321116	0.48144093	0.81037973	nonDEG
Bpev01.c0472.g0011.m0001	0.123227085	0.6814001	0.94267087	nonDEG
Bpev01.c0324.g0014.m0002	0.57375067	0.10657641	0.33399858	nonDEG
Bpev01.c0929.g0001.m0001	-0.710667186	0.0225798	0.11275179	nonDEG
Bpev01.c0540.g0014.m0001	-0.327557261	0.3033357	0.635128	nonDEG
Bpev01.c1442.g0001.m0001	-0.230332396	0.53181797	0.85057649	nonDEG
Bpev01.c0015.g0149.m0001	-1.365895072	0.05391089	0.21121674	nonDEG
Bpev01.c0051.g0062.m0001	0.412542884	0.36140518	0.6995008	nonDEG
Bpev01.c0344.g0019.m0001	0.658623614	0.03376399	0.15112768	nonDEG
Bpev01.c0452.g0030.m0001	0.036684661	0.92352443	1	nonDEG
Bpev01.c0521.g0001.m0001	-0.427467019	0.16982782	0.45421652	nonDEG
Bpev01.c0442.g0037.m0001	0.076363545	0.86737465	1	nonDEG
Bpev01.c0333.g0003.m0001	0.040242857	0.90265995	1	nonDEG
Bpev01.c0161.g0048.m0001	-0.60715546	0.07428673	0.26304149	nonDEG
Bpev01.c1031.g0010.m0001	-0.64299835	0.03940308	0.16941851	nonDEG
Bpev01.c1229.g0019.m0001	-0.425131626	0.20798699	0.51237899	nonDEG
Bpev01.c0704.g0010.m0001	1.434686063	0.03665474	0.16055333	nonDEG
Bpev01.c0718.g0007.m0002	-0.518116952	0.1119032	0.34578758	nonDEG
Bpev01.c0950.g0014.m0001	-0.210932202	0.50582318	0.82787172	nonDEG
Bpev01.c1320.g0002.m0001	0.281361885	0.51199129	0.83282747	nonDEG
Bpev01.c0215.g0034.m0001	0.834351248	0.03762553	0.16383051	nonDEG
Bpev01.c0190.g0007.m0001	-0.640068617	0.05531792	0.21487058	nonDEG
Bpev01.c0778.g0013.m0001	-0.270876191	0.41809035	0.74985755	nonDEG
Bpev01.c0243.g0043.m0002	1.27620986	0.0162837	0.08885558	nonDEG
Bpev01.c0595.g0009.m0001	-0.289330871	0.35320846	0.6912254	nonDEG
Bpev01.c2123.g0003.m0002	0.437869464	0.2110675	0.51693405	nonDEG
Bpev01.c1741.g0002.m0001	-0.608217372	0.04327162	0.18146344	nonDEG
Bpev01.c0667.g0030.m0001	-0.13387501	0.68132634	0.94267087	nonDEG
Bpev01.c2312.g0002.m0001	0.363279635	0.90396371	1	nonDEG
Bpev01.c0158.g0030.m0001	-0.67480163	0.04479403	0.18565274	nonDEG
Bpev01.c0000.g0136.m0001	-0.617253446	0.04458881	0.18521941	nonDEG
Bpev01.c0356.g0007.m0001	2.875189736	0.01952421	0.10108568	nonDEG
Bpev01.c0329.g0001.m0001	-0.013839451	0.97378376	1	nonDEG
Bpev01.c0480.g0010.m0001	-0.160019089	0.59655266	0.90438462	nonDEG
Bpev01.c1099.g0006.m0001	-0.344483749	0.30576855	0.6369601	nonDEG

Bpev01.c0015.g0093.m0001	0.929578837	0.0098222	0.06097759	nonDEG
Bpev01.c1654.g0001.m0001	-3.135394404	0.66056109	0.93220195	nonDEG
Bpev01.c0094.g0084.m0001	-0.832923449	0.0479549	0.19429259	nonDEG
Bpev01.c2000.g0014.m0001	-0.793937929	0.01180528	0.06978625	nonDEG
Bpev01.c1455.g0010.m0001	-1.47666983	0.46478522	0.79365993	nonDEG
Bpev01.c0902.g0003.m0001	-0.556421595	0.06622491	0.24330404	nonDEG
Bpev01.c0118.g0036.m0002	0.219115352	0.46558854	0.79425514	nonDEG
Bpev01.c1670.g0003.m0001	-0.353750244	0.46439271	0.7932264	nonDEG
Bpev01.c0863.g0012.m0001	-0.834770583	0.00866631	0.05546251	nonDEG
Bpev01.c0135.g0050.m0001	-0.157722074	0.78605611	1	nonDEG
Bpev01.c0043.g0017.m0001	-0.010722941	0.97712124	1	nonDEG
Bpev01.c0350.g0018.m0002	0.127963899	0.69014394	0.94890558	nonDEG
Bpev01.c0166.g0047.m0001	0.040127972	0.90037815	1	nonDEG
Bpev01.c0232.g0015.m0001	-0.356869128	0.25977163	0.58106657	nonDEG
Bpev01.c1159.g0011.m0001	-5.01062927	0.09131632	0.30147595	nonDEG
Bpev01.c0237.g0064.m0001	-0.260568784	0.52646628	0.84571869	nonDEG
Bpev01.c1773.g0008.m0001	-0.750084358	0.01562105	0.08608538	nonDEG
Bpev01.c1721.g0003.m0001	0.671841317	0.58910333	0.89872068	nonDEG
Bpev01.c0158.g0022.m0001	-0.111220556	0.90751515	1	nonDEG
Bpev01.c0000.g0240.m0001	-1.884947323	0.06251743	0.23382678	nonDEG
Bpev01.c0998.g0005.m0001	0.661119672	0.04489879	0.18590373	nonDEG
Bpev01.c0666.g0008.m0001	0.11148311	0.71535422	0.96731717	nonDEG
Bpev01.c0439.g0021.m0001	0.462251298	0.15020444	0.42020987	nonDEG
Bpev01.c1450.g0004.m0001	-0.245485435	0.42922294	0.76101506	nonDEG
Bpev01.c0504.g0010.m0001	0.093735583	0.97383926	1	nonDEG
Bpev01.c0357.g0072.m0001	-0.112889128	0.71576796	0.96760312	nonDEG
Bpev01.c0223.g0042.m0001	0.857728595	0.00950237	0.05939282	nonDEG
Bpev01.c5566.g0001.m0001	-0.094816658	1	1	nonDEG
Bpev01.c0910.g0002.m0001	0.699413525	0.22205286	0.53137816	nonDEG
Bpev01.c1030.g0010.m0001	0.018534472	0.9552782	1	nonDEG
Bpev01.c1424.g0004.m0001	0.011384715	0.97116296	1	nonDEG
Bpev01.c0095.g0045.m0001	0.119454973	0.70906658	0.96281138	nonDEG
Bpev01.c0445.g0011.m0001	0.252777129	0.42817117	0.76029501	nonDEG
Bpev01.c0091.g0058.m0001	0.141315329	0.91661327	1	nonDEG
Bpev01.c1280.g0011.m0001	-0.255836741	0.41109688	0.74500233	nonDEG
Bpev01.c2138.g0001.m0001	-0.432483094	0.15354532	0.42543509	nonDEG
Bpev01.c0190.g0079.m0001	0.641772207	0.03329037	0.14944119	nonDEG
Bpev01.c1397.g0006.m0001	-0.336427579	0.30538054	0.63669247	nonDEG
Bpev01.c1130.g0005.m0001	0.36705561	0.42123214	0.75264949	nonDEG
Bpev01.c0812.g0005.m0001	-0.637370951	0.04373937	0.18295584	nonDEG
Bpev01.c0384.g0007.m0002	0.524630584	0.08600817	0.28953968	nonDEG
Bpev01.c0286.g0018.m0001	0.123413455	0.73222007	0.97727826	nonDEG
Bpev01.c0455.g0017.m0001	-0.372884959	0.24216738	0.55976487	nonDEG

Bpev01.c0154.g0084.m0001	-0.906230675	0.05332218	0.20971967	nonDEG
Bpev01.c0540.g0027.m0001	0.282758768	0.58099805	0.89164126	nonDEG
Bpev01.c0088.g0113.m0001	0.602239208	0.08878162	0.29566087	nonDEG
Bpev01.c0029.g0095.m0001	-3.135235619	0.66719249	0.93220195	nonDEG
Bpev01.c1608.g0004.m0001	-0.672552593	0.36590969	0.70502097	nonDEG
Bpev01.c0574.g0007.m0001	1.123702708	0.01754368	0.09364792	nonDEG
Bpev01.c0162.g0040.m0001	0.339146729	0.34091994	0.67698375	nonDEG
Bpev01.c1968.g0008.m0001	-0.634928272	0.04606834	0.18895335	nonDEG
Bpev01.c1221.g0005.m0001	0.280433143	0.42251419	0.75424381	nonDEG
Bpev01.c0962.g0032.m0001	1.401337491	0.01489095	0.08313572	nonDEG
Bpev01.c1897.g0005.m0001	-0.856925135	0.00828677	0.05360455	nonDEG
Bpev01.c0001.g0037.m0001	-0.235429032	0.44383696	0.77527213	nonDEG
Bpev01.c0038.g0135.m0001	-0.025331663	0.93423381	1	nonDEG
Bpev01.c1140.g0012.m0001	0.775287684	0.01382436	0.07868817	nonDEG
Bpev01.c0001.g0026.m0001	0.189388582	0.55198585	0.86758736	nonDEG
Bpev01.c0051.g0177.m0003	0.056536656	0.86258675	1	nonDEG
Bpev01.c0976.g0005.m0001	0.01692011	0.96110237	1	nonDEG
Bpev01.c0419.g0013.m0001	-0.414368987	0.28243536	0.60918669	nonDEG
Bpev01.c1558.g0002.m0001	1.783916349	0.13562781	0.39306429	nonDEG
Bpev01.c0411.g0016.m0001	1.140728495	0.00826826	0.05350834	nonDEG
Bpev01.c1999.g0003.m0001	2.863843096	0.30702744	0.63779608	nonDEG
Bpev01.c0016.g0102.m0001	0.64559281	0.11080834	0.34308864	nonDEG
Bpev01.c0289.g0003.m0002	0.201026558	0.74119201	0.98341259	nonDEG
Bpev01.c0817.g0001.m0001	-0.732713448	0.02540437	0.1232294	nonDEG
Bpev01.c0597.g0010.m0002	-0.522141543	0.12857903	0.38008419	nonDEG
Bpev01.c1764.g0014.m0001	0.447704045	0.17468634	0.4623679	nonDEG
Bpev01.c0127.g0074.m0001	-0.221879923	0.49069632	0.81484708	nonDEG
Bpev01.c2783.g0003.m0001	0.245990255	0.43486828	0.76718927	nonDEG
Bpev01.c0949.g0005.m0001	-0.563755652	0.07974335	0.27563476	nonDEG
Bpev01.c0717.g0002.m0001	-0.532701208	0.14140142	0.40357053	nonDEG
Bpev01.c0314.g0003.m0001	0.811955633	0.02451291	0.12011244	nonDEG
Bpev01.c2054.g0002.m0001	0.008305918	0.97815041	1	nonDEG
Bpev01.c0123.g0024.m0001	0.11242095	0.72442244	0.97304705	nonDEG
Bpev01.c0080.g0090.m0001	0.114962394	0.74087022	0.98329442	nonDEG
Bpev01.c0243.g0033.m0001	-0.448319232	0.18790774	0.48258012	nonDEG
Bpev01.c0312.g0010.m0001	-0.729057668	0.03156241	0.14371314	nonDEG
Bpev01.c1162.g0008.m0001	-0.33574717	0.26632191	0.59029143	nonDEG
Bpev01.c1567.g0002.m0001	-0.708569059	0.36724206	0.70672699	nonDEG
Bpev01.c0472.g0006.m0001	0.351869797	0.24196337	0.55946902	nonDEG
Bpev01.c0044.g0014.m0001	-2.271461396	0.13616104	0.39409737	nonDEG
Bpev01.c0190.g0061.m0001	0.71618187	0.15609823	0.43031528	nonDEG
Bpev01.c1105.g0008.m0001	-1.317553669	0.08680525	0.29128686	nonDEG
Bpev01.c2531.g0004.m0001	0.782606226	0.16208754	0.44050682	nonDEG

Bpev01.c1117.g0004.m0001	2.579107644	0.02967917	0.13771366	nonDEG
Bpev01.c0390.g0007.m0001	0.203562051	0.70586157	0.96033189	nonDEG
Bpev01.c1103.g0002.m0001	-0.945229379	0.02829454	0.13306119	nonDEG
Bpev01.c0088.g0089.m0001	-0.407002035	0.20664553	0.50997046	nonDEG
Bpev01.c2723.g0001.m0001	0.840087765	0.00968938	0.06025467	nonDEG
Bpev01.c1767.g0008.m0001	-5.168891108	0.160055	0.43749852	nonDEG
Bpev01.c0000.g0187.m0001	-0.130154314	0.66370004	0.93220195	nonDEG
Bpev01.c0387.g0005.m0001	0.148139372	0.71694308	0.96831905	nonDEG
Bpev01.c0001.g0061.m0001	-0.848456201	0.07297287	0.25984931	nonDEG
Bpev01.c0764.g0002.m0002	-0.007166445	0.9850595	1	nonDEG
Bpev01.c0224.g0051.m0001	0.108848022	0.73734423	0.9806495	nonDEG
Bpev01.c0533.g0016.m0001	-0.120632652	0.94186452	1	nonDEG
Bpev01.c1633.g0001.m0001	1.153445929	0.02602318	0.12543847	nonDEG
Bpev01.c0616.g0014.m0001	-0.651154015	0.1047612	0.33073082	nonDEG
Bpev01.c0215.g0038.m0001	-0.061273807	0.8503697	1	nonDEG
Bpev01.c0016.g0117.m0001	0.286415227	0.33934837	0.67541591	nonDEG
Bpev01.c0036.g0017.m0001	-0.548042845	0.4964657	0.81948157	nonDEG
Bpev01.c0775.g0001.m0001	0.074060831	0.87701972	1	nonDEG
Bpev01.c0157.g0048.m0001	-1.327207926	0.03715279	0.16225204	nonDEG
Bpev01.c0142.g0039.m0001	-0.296265448	0.32536073	0.65930843	nonDEG
Bpev01.c0044.g0079.m0001	-0.510182993	0.09584009	0.31107346	nonDEG
Bpev01.c0145.g0052.m0001	-0.531548739	0.13795792	0.39789783	nonDEG
Bpev01.c0914.g0005.m0001	-0.039784896	0.8981488	1	nonDEG
Bpev01.c0363.g0018.m0001	-1.341332982	0.63949774	0.93036198	nonDEG
Bpev01.c0734.g0001.m0001	0.635206411	0.076041	0.26710069	nonDEG
Bpev01.c0027.g0116.m0001	-0.773273403	0.02892716	0.13527305	nonDEG
Bpev01.c0052.g0196.m0001	-0.372450927	0.26107663	0.58309952	nonDEG
Bpev01.c0120.g0045.m0001	0.005917916	0.98678393	1	nonDEG
Bpev01.c0020.g0012.m0001	-0.620980654	0.8070411	1	nonDEG
Bpev01.c0038.g0090.m0001	-0.145110922	0.70144313	0.95746868	nonDEG
Bpev01.c1445.g0004.m0001	1.64983219	0.26641678	0.59040479	nonDEG
Bpev01.c0026.g0007.m0001	-0.356043364	0.7668562	0.99835094	nonDEG
Bpev01.c0592.g0008.m0001	0.081841281	0.81484168	1	nonDEG
Bpev01.c0403.g0004.m0001	-0.085083074	0.84872216	1	nonDEG
Bpev01.c0190.g0081.m0001	0.295536403	0.50868237	0.82978336	nonDEG
Bpev01.c0149.g0019.m0001	-0.083190943	0.78999399	1	nonDEG
Bpev01.c0682.g0002.m0001	-0.209200125	0.52981489	0.84925004	nonDEG
Bpev01.c0962.g0023.m0001	-0.665716303	0.02577183	0.12456031	nonDEG
Bpev01.c0394.g0008.m0001	-0.567534246	0.11617203	0.35470146	nonDEG
Bpev01.c0162.g0026.m0001	-1.111216824	0.06733256	0.24606201	nonDEG
Bpev01.c0906.g0023.m0002	0.198421983	0.59002293	0.8994555	nonDEG
Bpev01.c1807.g0005.m0001	1.335994166	0.57592447	0.88730586	nonDEG
Bpev01.c0679.g0017.m0002	0.894295368	0.07229015	0.25820792	nonDEG

Bpev01.c1157.g0006.m0001	0.058648779	0.94156011	1	nonDEG
Bpev01.c1502.g0002.m0001	0.037541474	0.9156847	1	nonDEG
Bpev01.c1260.g0011.m0001	-0.196596057	0.51610252	0.83637246	nonDEG
Bpev01.c0051.g0055.m0001	1.494824707	0.09720865	0.31402534	nonDEG
Bpev01.c0265.g0008.m0001	-0.976548069	0.01478845	0.08270925	nonDEG
Bpev01.c2504.g0004.m0001	0.632449293	0.41993752	0.75148728	nonDEG
Bpev01.c1130.g0007.m0001	0.019530319	0.95391045	1	nonDEG
Bpev01.c0727.g0007.m0001	-0.210441217	0.56396297	0.87792771	nonDEG
Bpev01.c2001.g0002.m0001	0.257668266	0.50752486	0.82885967	nonDEG
Bpev01.c0024.g0021.m0001	-0.142198819	0.64429686	0.93220195	nonDEG
Bpev01.c1378.g0019.m0001	0.463139754	0.49615836	0.81931645	nonDEG
Bpev01.c0639.g0020.m0001	0.161055001	0.62221646	0.92218923	nonDEG
Bpev01.c0389.g0033.m0001	0.552397	0.07290573	0.259682	nonDEG
Bpev01.c0445.g0010.m0001	0.299710134	0.36560888	0.70457972	nonDEG
Bpev01.c0029.g0151.m0001	-3.136496727	0.13097325	0.38411253	nonDEG
Bpev01.c1318.g0004.m0001	2.305848103	0.24373073	0.56152677	nonDEG
Bpev01.c0848.g0022.m0001	0.636291975	0.03453774	0.15354222	nonDEG
Bpev01.c0718.g0004.m0001	-0.136740739	0.66364412	0.93220195	nonDEG
Bpev01.c2361.g0012.m0001	0.671451543	0.02803087	0.13222389	nonDEG
Bpev01.c0170.g0007.m0001	0.479980365	0.1495582	0.41872029	nonDEG
Bpev01.c0050.g0130.m0001	-0.119209512	0.70693361	0.9611922	nonDEG
Bpev01.c0401.g0034.m0001	0.556375331	0.15049603	0.42055612	nonDEG
Bpev01.c1600.g0001.m0001	0.637968977	0.50246483	0.82503553	nonDEG
Bpev01.c0428.g0036.m0001	0.091157951	0.85029906	1	nonDEG
Bpev01.c0564.g0026.m0001	-0.02066012	1	1	nonDEG
Bpev01.c2409.g0001.m0001	0.08473764	0.80897391	1	nonDEG
Bpev01.c0887.g0008.m0001	1.788536704	0.44927115	0.78079864	nonDEG
Bpev01.c0327.g0059.m0001	-0.635779558	0.21620862	0.52261808	nonDEG
Bpev01.c0280.g0049.m0001	0.355596679	0.38553466	0.71909228	nonDEG
Bpev01.c0552.g0006.m0001	-0.643443789	0.0605956	0.22881913	nonDEG
Bpev01.c0036.g0024.m0001	-0.122396809	0.6873747	0.94675627	nonDEG
Bpev01.c0083.g0023.m0001	2.435782423	0.02632014	0.12635985	nonDEG
Bpev01.c0190.g0072.m0001	-0.668337568	0.05026619	0.2011385	nonDEG
Bpev01.c0472.g0002.m0001	-0.886517857	0.00821357	0.0532363	nonDEG
Bpev01.c0127.g0094.m0001	-0.716427628	0.06980625	0.25227428	nonDEG
Bpev01.c0286.g0005.m0001	-0.15371233	0.6168518	0.9185382	nonDEG
Bpev01.c0000.g0200.m0001	-2.82902692	0.09984638	0.32015877	nonDEG
Bpev01.c0727.g0015.m0001	0.111086071	0.77236636	1	nonDEG
Bpev01.c2042.g0001.m0001	-1.017959448	0.15716076	0.43247855	nonDEG
Bpev01.c0027.g0151.m0001	0.596845658	0.09415858	0.30750143	nonDEG
Bpev01.c0052.g0108.m0001	-0.288384409	0.37686437	0.71458531	nonDEG
Bpev01.c0919.g0004.m0001	0.079888543	0.81753967	1	nonDEG
Bpev01.c1346.g0010.m0001	0.70528165	0.07134367	0.2560304	nonDEG

Bpev01.c1072.g0010.m0001	0.379974044	0.33000798	0.66565694	nonDEG
Bpev01.c1207.g0005.m0001	-0.177928449	0.67338955	0.93685799	nonDEG
Bpev01.c0264.g0012.m0001	-0.185443051	0.58428246	0.89458424	nonDEG
Bpev01.c0158.g0040.m0001	0.234306272	0.49643384	0.81948157	nonDEG
Bpev01.c0344.g0018.m0001	1.372559933	0.16232438	0.44075516	nonDEG
Bpev01.c0902.g0006.m0001	-0.069538429	0.82307073	1	nonDEG
Bpev01.c0157.g0060.m0001	0.731471842	0.70242557	0.95827918	nonDEG
Bpev01.c0915.g0014.m0001	0.264242376	0.37900702	0.71647215	nonDEG
Bpev01.c0015.g0238.m0001	0.774142139	0.02094096	0.10657064	nonDEG
Bpev01.c0101.g0050.m0001	-0.725668217	0.0935193	0.30600362	nonDEG
Bpev01.c1078.g0007.m0001	-0.935058501	0.19773817	0.49758217	nonDEG
Bpev01.c0105.g0008.m0001	-0.317556323	0.33891914	0.67497231	nonDEG
Bpev01.c1040.g0029.m0001	-0.368669663	0.22249137	0.53182623	nonDEG
Bpev01.c0142.g0020.m0001	-1.925100899	0.02647752	0.12692086	nonDEG
Bpev01.c0024.g0033.m0001	-0.318532407	0.36950437	0.70930521	nonDEG
Bpev01.c0038.g0097.m0001	-0.534956663	0.31166138	0.64241698	nonDEG
Bpev01.c0532.g0013.m0001	-0.030183124	0.92753096	1	nonDEG
Bpev01.c0154.g0048.m0001	2.39585494	1	1	nonDEG
Bpev01.c0030.g0005.m0001	0.623895643	0.3161209	0.64861187	nonDEG
Bpev01.c0996.g0001.m0001	-0.208702706	0.49866323	0.82140481	nonDEG
Bpev01.c0532.g0015.m0001	0.03008276	1	1	nonDEG
Bpev01.c3621.g0001.m0001	-0.53003537	0.10349033	0.32787902	nonDEG
Bpev01.c0127.g0098.m0001	-0.050108503	0.86954254	1	nonDEG
Bpev01.c1916.g0005.m0001	0.5361175	0.79616363	1	nonDEG
Bpev01.c0826.g0001.m0001	-0.204738199	0.51483023	0.83509214	nonDEG
Bpev01.c0804.g0001.m0001	-0.23057331	0.78438993	1	nonDEG
Bpev01.c1758.g0007.m0001	0.549666677	0.06832299	0.24871009	nonDEG
Bpev01.c0333.g0007.m0002	-0.201269348	0.63882829	0.93036198	nonDEG
Bpev01.c2961.g0001.m0001	0.903436117	0.1029228	0.3268915	nonDEG
Bpev01.c0929.g0002.m0001	-0.949434993	0.02297426	0.11422721	nonDEG
Bpev01.c3157.g0001.m0001	0.41957276	0.42894836	0.76099568	nonDEG
Bpev01.c1786.g0006.m0001	0.751261502	0.04731725	0.1924793	nonDEG
Bpev01.c0745.g0001.m0001	-0.095618167	0.75030012	0.98762058	nonDEG
Bpev01.c0894.g0008.m0001	0.309724699	0.31332733	0.6446217	nonDEG
Bpev01.c0162.g0072.m0001	-0.03343465	0.94080316	1	nonDEG
Bpev01.c0647.g0002.m0001	-0.41224373	0.21219308	0.51831664	nonDEG
Bpev01.c0995.g0006.m0001	0.456933238	0.18640909	0.48068724	nonDEG
Bpev01.c0051.g0025.m0001	-0.056324162	0.92566015	1	nonDEG
Bpev01.c0000.g0148.m0001	-0.322363267	0.51367076	0.83411192	nonDEG
Bpev01.c0996.g0012.m0002	0.219215233	0.47845779	0.80699578	nonDEG
Bpev01.c0024.g0026.m0001	-0.08207143	0.79678053	1	nonDEG
Bpev01.c2114.g0004.m0001	-1.711015643	0.01055479	0.06435576	nonDEG
Bpev01.c0169.g0062.m0001	-0.313890708	0.52689488	0.84589946	nonDEG

Bpev01.c1183.g0018.m0001	1.755495618	0.01425948	0.08041854	nonDEG
Bpev01.c1684.g0010.m0001	-0.358088622	0.72461989	0.97308867	nonDEG
Bpev01.c1150.g0011.m0001	0.558059723	0.0929941	0.30486574	nonDEG
Bpev01.c0089.g0072.m0001	-0.396696144	0.28159053	0.60830961	nonDEG
Bpev01.c0281.g0009.m0001	-0.410250905	0.28591187	0.61341512	nonDEG
Bpev01.c0455.g0020.m0001	0.720315225	0.08291942	0.28267799	nonDEG
Bpev01.c0237.g0044.m0001	-0.292113673	0.37183245	0.71134876	nonDEG
Bpev01.c0879.g0011.m0001	2.172368206	0.01644508	0.08950464	nonDEG
Bpev01.c0335.g0040.m0001	-0.510257809	0.13638899	0.39445481	nonDEG
Bpev01.c0119.g0001.m0001	0.144114063	0.86685615	1	nonDEG
Bpev01.c0813.g0006.m0001	0.504903347	0.39923565	0.73224046	nonDEG
Bpev01.c0094.g0080.m0001	-0.505385223	0.12464749	0.37231101	nonDEG
Bpev01.c1071.g0001.m0001	-0.186672275	0.68506142	0.9452788	nonDEG
Bpev01.c1161.g0017.m0001	0.502834864	0.13340687	0.38850181	nonDEG
Bpev01.c0038.g0136.m0001	-0.123598349	0.69613129	0.95371412	nonDEG
Bpev01.c0001.g0052.m0001	-0.169425388	0.57147147	0.8839147	nonDEG
Bpev01.c0169.g0012.m0001	-0.747794986	0.02396372	0.11801067	nonDEG
Bpev01.c0396.g0025.m0001	-0.744666285	0.01461986	0.08201106	nonDEG
Bpev01.c0082.g0063.m0001	-0.376125577	0.484493	0.81256284	nonDEG
Bpev01.c0751.g0007.m0001	1.876190079	0.20054065	0.50129203	nonDEG
Bpev01.c0147.g0029.m0001	0.341418819	0.3103478	0.64069142	nonDEG
Bpev01.c1858.g0003.m0001	1.439150327	0.13039097	0.3830918	nonDEG
Bpev01.c0080.g0014.m0001	-0.338585306	0.27704984	0.60401913	nonDEG
Bpev01.c1577.g0019.m0001	-0.346330448	0.7885271	1	nonDEG
Bpev01.c0045.g0010.m0001	0.256547043	0.44369994	0.77519777	nonDEG
Bpev01.c0015.g0189.m0001	-0.476569624	0.18503517	0.47842619	nonDEG
Bpev01.c0053.g0063.m0001	1.28669798	0.1273601	0.3775024	nonDEG
Bpev01.c0918.g0028.m0001	0.079610792	0.79019372	1	nonDEG
Bpev01.c0118.g0003.m0001	1.091178803	0.08254234	0.28202926	nonDEG
Bpev01.c0169.g0011.m0001	0.492050541	0.1328331	0.38730226	nonDEG
Bpev01.c0714.g0004.m0001	-0.23700935	0.55996399	0.87478557	nonDEG
Bpev01.c0044.g0092.m0001	0.692384102	0.02874754	0.13469663	nonDEG
Bpev01.c0129.g0085.m0001	-0.635932216	0.0431802	0.1811798	nonDEG
Bpev01.c1588.g0005.m0001	-1.524999979	0.00949422	0.05935452	nonDEG
Bpev01.c0477.g0037.m0001	-0.293120753	0.65027295	0.93220195	nonDEG
Bpev01.c1206.g0007.m0001	-0.069070325	0.83049096	1	nonDEG
Bpev01.c0304.g0038.m0001	1.072240543	0.09396534	0.30705373	nonDEG
Bpev01.c0769.g0002.m0001	-6.433717526	0.04567124	0.18783399	nonDEG
Bpev01.c0121.g0014.m0002	-0.033403481	0.94095296	1	nonDEG
Bpev01.c0051.g0161.m0001	-0.050614007	0.86870532	1	nonDEG
Bpev01.c0304.g0019.m0001	-0.271662794	0.4465418	0.7779007	nonDEG
Bpev01.c0425.g0026.m0001	-0.172229719	0.57943916	0.89057687	nonDEG
Bpev01.c0062.g0085.m0001	-0.718943085	0.01887814	0.09876553	nonDEG

Bpev01.c0964.g0010.m0001	-0.356306594	0.25249059	0.57356989	nonDEG
Bpev01.c0051.g0004.m0001	0.141391745	0.71240828	0.96505733	nonDEG
Bpev01.c0146.g0001.m0001	0.582887684	0.05214427	0.20646606	nonDEG
Bpev01.c0425.g0042.m0001	-0.37445681	0.2509368	0.57111159	nonDEG
Bpev01.c0306.g0008.m0001	-0.419556427	1	1	nonDEG
Bpev01.c1315.g0010.m0001	0.476721185	0.28404762	0.61103967	nonDEG
Bpev01.c1117.g0011.m0001	0.806716053	0.01094054	0.06621682	nonDEG
Bpev01.c1206.g0017.m0001	0.244993726	0.41233975	0.74599742	nonDEG
Bpev01.c0052.g0032.m0001	0.329858784	0.34388528	0.68030635	nonDEG
Bpev01.c0229.g0003.m0001	1.49972831	0.05502472	0.21429723	nonDEG
Bpev01.c0402.g0025.m0001	0.625932501	0.1319307	0.38566561	nonDEG
Bpev01.c0134.g0042.m0001	-0.805272641	0.74740707	0.98577955	nonDEG
Bpev01.c2159.g0005.m0001	-0.405624876	0.19250201	0.48921501	nonDEG
Bpev01.c0353.g0022.m0001	0.399689643	0.26892168	0.59381828	nonDEG
Bpev01.c0536.g0018.m0001	1.418972508	0.00863125	0.05528877	nonDEG
Bpev01.c0015.g0168.m0001	0.14657727	0.67590516	0.93860504	nonDEG
Bpev01.c0504.g0018.m0001	1.130942721	0.67576777	0.93854759	nonDEG
Bpev01.c0042.g0030.m0001	-0.79405228	0.22333737	0.53289259	nonDEG
Bpev01.c0101.g0031.m0002	-0.3110004	0.31057793	0.64094663	nonDEG
Bpev01.c0224.g0020.m0001	0.71527273	0.31182395	0.64266208	nonDEG
Bpev01.c0283.g0021.m0001	0.266731343	0.64657658	0.93220195	nonDEG
Bpev01.c0428.g0015.m0001	-0.627515794	0.04659781	0.19060622	nonDEG
Bpev01.c0162.g0025.m0001	-1.161696715	0.01046215	0.06400075	nonDEG
Bpev01.c0509.g0004.m0001	-1.469824173	0.05534307	0.21491158	nonDEG
Bpev01.c0957.g0011.m0001	0.984093975	0.14622215	0.41255834	nonDEG
Bpev01.c0547.g0003.m0001	1.605846016	0.02058824	0.1050834	nonDEG
Bpev01.c0389.g0028.m0001	-0.434123744	0.18016752	0.47104366	nonDEG
Bpev01.c0320.g0006.m0001	0.221456212	0.50693025	0.82857808	nonDEG
Bpev01.c0396.g0019.m0001	-0.714194788	0.16290966	0.44157619	nonDEG
Bpev01.c0170.g0017.m0001	-0.332091288	0.6575903	0.93220195	nonDEG
Bpev01.c0428.g0029.m0001	-0.332616481	0.29080348	0.61998975	nonDEG
Bpev01.c0281.g0053.m0001	0.057663838	0.86295174	1	nonDEG
Bpev01.c0978.g0009.m0001	-0.198361169	0.80661118	1	nonDEG
Bpev01.c0155.g0010.m0001	0.458086812	0.15673487	0.43154862	nonDEG
Bpev01.c0636.g0006.m0001	0.019702809	0.9653511	1	nonDEG
Bpev01.c1085.g0005.m0001	-0.204131434	0.59915472	0.90574694	nonDEG
Bpev01.c0778.g0018.m0001	-0.799844801	0.01464357	0.08208782	nonDEG
Bpev01.c0458.g0024.m0001	-0.602538032	0.20425754	0.50671087	nonDEG
Bpev01.c0574.g0015.m0001	0.276190491	0.58835571	0.89818522	nonDEG
Bpev01.c1200.g0001.m0001	0.305687858	0.38657115	0.7194505	nonDEG
Bpev01.c1267.g0001.m0001	0.328422038	0.31265585	0.64383573	nonDEG
Bpev01.c0349.g0021.m0001	-0.601410378	0.18496115	0.47839319	nonDEG
Bpev01.c0644.g0008.m0001	-0.023855096	0.94023479	1	nonDEG

Bpev01.c0744.g0004.m0001	-1.918697121	0.00804249	0.0524392	nonDEG
Bpev01.c1457.g0002.m0001	0.866134265	0.32263219	0.65625434	nonDEG
Bpev01.c1447.g0006.m0001	0.319650671	0.83378697	1	nonDEG
Bpev01.c0911.g0003.m0001	-0.578866544	0.24039522	0.55763888	nonDEG
Bpev01.c0051.g0039.m0001	-0.743197171	0.45386208	0.78408739	nonDEG
Bpev01.c0803.g0007.m0001	-0.501188276	0.1077035	0.33655486	nonDEG
Bpev01.c2182.g0001.m0001	0.191130767	0.64448497	0.93220195	nonDEG
Bpev01.c0134.g0091.m0001	0.2087198	0.51708111	0.83726698	nonDEG
Bpev01.c0402.g0009.m0001	0.391755784	0.24154038	0.55888606	nonDEG
Bpev01.c0195.g0007.m0001	-0.787787605	0.01540498	0.08517877	nonDEG
Bpev01.c0016.g0125.m0001	0.395720677	0.23199714	0.5463503	nonDEG
Bpev01.c0781.g0010.m0001	-0.709786993	0.02929432	0.13634711	nonDEG
Bpev01.c0333.g0008.m0002	0.211173273	0.52632813	0.84567388	nonDEG
Bpev01.c0022.g0051.m0001	-1.1778847	0.01555877	0.08582436	nonDEG
Bpev01.c1153.g0003.m0001	-0.526188865	0.10611212	0.33322623	nonDEG
Bpev01.c0161.g0030.m0001	0.636819375	0.05273366	0.20821166	nonDEG
Bpev01.c0139.g0004.m0001	1.989941805	0.00959288	0.05985687	nonDEG
Bpev01.c0366.g0009.m0002	0.551258905	0.07902675	0.27379882	nonDEG
Bpev01.c0497.g0005.m0001	-0.619392282	0.34778025	0.68482639	nonDEG
Bpev01.c1354.g0008.m0001	-0.099656839	0.75389658	0.99005047	nonDEG
Bpev01.c1058.g0009.m0001	0.2829431	0.35375182	0.69163199	nonDEG
Bpev01.c0842.g0010.m0001	0.565783007	0.1296542	0.38178752	nonDEG
Bpev01.c0455.g0022.m0001	1.091662876	0.00788607	0.05168257	nonDEG
Bpev01.c0442.g0015.m0001	1.631791664	0.3219302	0.65532451	nonDEG
Bpev01.c0438.g0020.m0001	0.855507474	0.04101108	0.17441853	nonDEG
Bpev01.c0094.g0271.m0001	-0.004052248	1	1	nonDEG
Bpev01.c0470.g0028.m0001	-1.651839238	0.01158857	0.06890544	nonDEG
Bpev01.c0397.g0011.m0001	0.75790065	0.01932156	0.1004605	nonDEG
Bpev01.c1975.g0002.m0001	-0.616041187	0.10657912	0.33399858	nonDEG
Bpev01.c0939.g0002.m0001	0.1088388	0.76880042	0.99947556	nonDEG
Bpev01.c0647.g0006.m0001	0.053082272	0.89987165	1	nonDEG
Bpev01.c0390.g0019.m0001	-0.464945948	0.20124042	0.50227357	nonDEG
Bpev01.c0135.g0100.m0001	-0.817626818	0.01497306	0.08348334	nonDEG
Bpev01.c1324.g0001.m0001	-0.26159589	0.44977936	0.78122925	nonDEG
Bpev01.c0464.g0012.m0001	-0.417743571	0.17974507	0.4702315	nonDEG
Bpev01.c0454.g0017.m0001	0.278851731	0.60709944	0.9112063	nonDEG
Bpev01.c0022.g0177.m0002	-1.095798632	0.01603847	0.08782931	nonDEG
Bpev01.c0588.g0009.m0001	0.241200633	0.43372997	0.765594	nonDEG
Bpev01.c1414.g0004.m0001	0.382589101	0.46343823	0.7923187	nonDEG
Bpev01.c0862.g0025.m0001	-1.413630406	0.55826304	0.87353597	nonDEG
Bpev01.c0915.g0010.m0001	0.15836926	0.59724969	0.90476398	nonDEG
Bpev01.c0281.g0003.m0001	-0.709076999	0.12517609	0.37339738	nonDEG
Bpev01.c1000.g0011.m0001	-0.317258486	0.29526876	0.62524917	nonDEG

Bpev01.c0279.g0025.m0001	0.468631393	0.24008479	0.55713827	nonDEG
Bpev01.c1202.g0006.m0001	-0.652691503	0.0347686	0.15438852	nonDEG
Bpev01.c1148.g0004.m0001	-0.195924145	0.6381846	0.93006545	nonDEG
Bpev01.c2685.g0003.m0001	0.222797838	0.56411236	0.87792771	nonDEG
Bpev01.c0080.g0040.m0001	0.301440968	0.34862966	0.68568336	nonDEG
Bpev01.c0813.g0017.m0001	0.113718851	0.85964406	1	nonDEG
Bpev01.c0281.g0079.m0002	-0.049288579	0.88112293	1	nonDEG
Bpev01.c0134.g0113.m0001	-0.419034546	0.20295028	0.50482311	nonDEG
Bpev01.c1206.g0015.m0001	0.023719005	0.93921256	1	nonDEG
Bpev01.c0267.g0016.m0001	-0.923418348	0.01132182	0.06780296	nonDEG
Bpev01.c1089.g0004.m0001	0.357679858	0.26883813	0.59367833	nonDEG
Bpev01.c0449.g0034.m0001	0.574364445	0.13368542	0.38908206	nonDEG
Bpev01.c1879.g0001.m0002	1.11958513	0.07984426	0.27585203	nonDEG
Bpev01.c0053.g0037.m0001	-0.51695785	0.10053548	0.32163268	nonDEG
Bpev01.c0454.g0016.m0001	-0.104459931	0.74844935	0.98641718	nonDEG
Bpev01.c1906.g0001.m0001	-0.685445082	0.22815617	0.54070391	nonDEG
Bpev01.c0389.g0054.m0001	-0.557618578	0.08763218	0.29309292	nonDEG
Bpev01.c0162.g0065.m0001	-0.616852351	0.06540466	0.24116454	nonDEG
Bpev01.c0050.g0134.m0001	-0.690417289	0.03049837	0.14029663	nonDEG
Bpev01.c1165.g0004.m0001	1.016299209	0.04049556	0.17302594	nonDEG
Bpev01.c2035.g0008.m0001	0.628370582	0.07579738	0.26644908	nonDEG
Bpev01.c0744.g0012.m0001	0.203292489	0.5257361	0.84541683	nonDEG
Bpev01.c0577.g0014.m0001	0.970700723	0.80456388	1	nonDEG
Bpev01.c1949.g0002.m0001	-1.263755441	0.0689666	0.2502811	nonDEG
Bpev01.c1838.g0006.m0001	0.160094471	0.6139828	0.91663639	nonDEG
Bpev01.c0123.g0014.m0001	-0.517985523	0.19018647	0.48590305	nonDEG
Bpev01.c1115.g0004.m0001	0.444465374	0.16395414	0.44346252	nonDEG
Bpev01.c0472.g0008.m0001	-0.181305127	0.63875942	0.93036198	nonDEG
Bpev01.c0429.g0016.m0001	-0.589382159	0.58183901	0.89249471	nonDEG
Bpev01.c0257.g0024.m0001	0.125450605	0.8285841	1	nonDEG
Bpev01.c0219.g0011.m0001	-0.066171201	0.83126216	1	nonDEG
Bpev01.c0864.g0001.m0001	-2.067074854	0.08932824	0.29697729	nonDEG
Bpev01.c0142.g0015.m0001	-0.063448645	0.84665923	1	nonDEG
Bpev01.c0597.g0003.m0001	-0.432068634	0.24785241	0.56685264	nonDEG
Bpev01.c0862.g0004.m0001	-0.050592002	0.88106117	1	nonDEG
Bpev01.c0755.g0001.m0001	0.248627503	0.89086478	1	nonDEG
Bpev01.c0752.g0016.m0001	0.011208597	0.98570507	1	nonDEG
Bpev01.c0121.g0006.m0001	0.603076717	0.0513053	0.20402225	nonDEG
Bpev01.c0381.g0006.m0001	0.397987881	0.33387472	0.66928552	nonDEG
Bpev01.c1054.g0012.m0001	-0.734453064	0.09798429	0.3158738	nonDEG
Bpev01.c2890.g0001.m0001	0.252994935	0.41139482	0.74506078	nonDEG
Bpev01.c0523.g0022.m0001	-0.114032853	0.72739077	0.97489902	nonDEG
Bpev01.c0878.g0002.m0001	-0.87843133	0.04414579	0.18400007	nonDEG

Bpev01.c4242.g0002.m0001	-1.412568309	0.03552487	0.15677058	nonDEG
Bpev01.c0240.g0031.m0001	-0.278906744	0.44128454	0.7729043	nonDEG
Bpev01.c0640.g0002.m0001	0.808198702	0.06615506	0.24313841	nonDEG
Bpev01.c0501.g0005.m0001	0.565673738	0.24179258	0.55924981	nonDEG
Bpev01.c0338.g0005.m0001	-0.229206775	0.45919158	0.7883677	nonDEG
Bpev01.c0038.g0086.m0001	-0.628089736	0.05227442	0.20684231	nonDEG
Bpev01.c0015.g0236.m0001	-1.984154629	0.19563001	0.49447512	nonDEG
Bpev01.c1044.g0005.m0001	0.330029494	0.56102009	0.87533763	nonDEG
Bpev01.c0956.g0004.m0001	-0.702257965	0.73948111	0.98211605	nonDEG
Bpev01.c1060.g0030.m0002	-0.806299889	0.02899984	0.13551212	nonDEG
Bpev01.c0557.g0018.m0001	-0.517690542	0.10527675	0.33175361	nonDEG
Bpev01.c0053.g0052.m0001	-0.329426308	0.27236155	0.59821333	nonDEG
Bpev01.c0551.g0003.m0001	-0.097570304	0.74755188	0.98577955	nonDEG
Bpev01.c0115.g0013.m0001	0.269165945	0.50244943	0.82503553	nonDEG
Bpev01.c2356.g0002.m0001	0.397361834	0.60671551	0.9109087	nonDEG
Bpev01.c0874.g0015.m0001	0.136589506	0.6618115	0.93220195	nonDEG
Bpev01.c1457.g0003.m0001	1.627309789	0.02432849	0.11944738	nonDEG
Bpev01.c2315.g0004.m0001	0.252212621	0.43734818	0.7696146	nonDEG
Bpev01.c1171.g0013.m0001	-5.914300867	0.07629156	0.26775717	nonDEG
Bpev01.c0051.g0100.m0001	-0.332295949	0.36085922	0.69925333	nonDEG
Bpev01.c1112.g0003.m0001	0.886823441	0.03893633	0.16797283	nonDEG
Bpev01.c1818.g0010.m0001	-0.538070709	0.1004724	0.32160564	nonDEG
Bpev01.c0518.g0048.m0002	0.248627436	0.61530243	0.91716533	nonDEG
Bpev01.c0299.g0028.m0001	-0.07706829	0.85356955	1	nonDEG
Bpev01.c1653.g0010.m0001	0.578356258	0.10877479	0.33877864	nonDEG
Bpev01.c0367.g0001.m0001	0.762147339	0.16048647	0.43815807	nonDEG
Bpev01.c0223.g0049.m0001	0.624912519	0.03767872	0.16398932	nonDEG
Bpev01.c0030.g0037.m0001	-0.147409249	0.62397881	0.923313	nonDEG
Bpev01.c1849.g0005.m0001	0.880638117	0.02309779	0.11468643	nonDEG
Bpev01.c0159.g0025.m0001	-2.292220257	1	1	nonDEG
Bpev01.c1645.g0002.m0001	0.003037332	0.9928671	1	nonDEG
Bpev01.c0480.g0026.m0001	-0.563688121	0.06939867	0.25114486	nonDEG
Bpev01.c0115.g0051.m0001	-0.002395567	0.99521624	1	nonDEG
Bpev01.c0501.g0002.m0001	-0.404510787	0.32079574	0.65430301	nonDEG
Bpev01.c0667.g0020.m0001	-0.583738915	0.06201859	0.23261215	nonDEG
Bpev01.c0018.g0020.m0001	0.226375837	0.59004349	0.8994555	nonDEG
Bpev01.c0472.g0020.m0001	-0.129494887	0.77725735	1	nonDEG
Bpev01.c0363.g0023.m0001	0.090965935	0.76193755	0.99510579	nonDEG
Bpev01.c0032.g0011.m0001	0.288553735	0.49749037	0.82048286	nonDEG
Bpev01.c0471.g0007.m0001	-0.221963557	0.59316572	0.9018598	nonDEG
Bpev01.c0015.g0107.m0001	-1.166299751	0.01091643	0.06611166	nonDEG
Bpev01.c0445.g0018.m0001	-0.661581273	0.03411302	0.15218265	nonDEG
Bpev01.c0163.g0025.m0001	0.28605934	0.35264852	0.69073104	nonDEG

Bpev01.c0480.g0080.m0001	0.610625345	0.04316879	0.18116121	nonDEG
Bpev01.c0919.g0014.m0001	-0.405601223	0.17751449	0.46621904	nonDEG
Bpev01.c0223.g0030.m0001	0.499422702	0.09403812	0.30725728	nonDEG
Bpev01.c0517.g0035.m0001	-0.424961984	0.18216221	0.47415326	nonDEG
Bpev01.c0807.g0007.m0001	1.827297756	0.16433884	0.44433973	nonDEG
Bpev01.c2320.g0004.m0001	0.214100423	0.49198896	0.81598101	nonDEG
Bpev01.c0758.g0003.m0001	0.696132816	0.07525034	0.26500542	nonDEG
Bpev01.c0022.g0173.m0001	-0.244705209	0.44156411	0.77304678	nonDEG
Bpev01.c0944.g0008.m0001	0.31949914	0.29044261	0.61963475	nonDEG
Bpev01.c0497.g0029.m0001	0.308359464	0.30620354	0.6373251	nonDEG
Bpev01.c0597.g0027.m0001	-0.447335776	0.17928531	0.46944589	nonDEG
Bpev01.c1167.g0014.m0001	-0.4158558	0.59003811	0.8994555	nonDEG
Bpev01.c0353.g0031.m0001	-0.08049656	0.78692791	1	nonDEG
Bpev01.c0053.g0016.m0003	0.4946069	0.17688843	0.46544601	nonDEG
Bpev01.c0000.g0154.m0001	-0.056067501	0.86002255	1	nonDEG
Bpev01.c0025.g0009.m0001	-0.262317168	0.386674	0.71945131	nonDEG
Bpev01.c0172.g0002.m0001	-0.545080721	0.08341258	0.283784	nonDEG
Bpev01.c0121.g0038.m0001	0.128430178	0.70419894	0.95949794	nonDEG
Bpev01.c0537.g0001.m0001	0.022445198	0.95772436	1	nonDEG
Bpev01.c0129.g0042.m0001	-0.401354587	0.33359995	0.66919231	nonDEG
Bpev01.c0055.g0008.m0001	-0.657294682	0.0381097	0.16525429	nonDEG
Bpev01.c1159.g0024.m0001	-0.357389747	0.36125628	0.6995008	nonDEG
Bpev01.c1560.g0011.m0001	0.055781299	0.97505889	1	nonDEG
Bpev01.c0915.g0018.m0001	0.087823214	0.77492523	1	nonDEG
Bpev01.c0344.g0037.m0001	-0.014722081	0.96378351	1	nonDEG
Bpev01.c0052.g0109.m0001	-0.261525619	0.41638829	0.74822626	nonDEG
Bpev01.c1187.g0005.m0001	0.716505223	0.05507137	0.21434451	nonDEG
Bpev01.c0177.g0002.m0001	0.198848202	0.56364263	0.87775472	nonDEG
Bpev01.c0457.g0006.m0001	0.267341061	0.42646753	0.75859469	nonDEG
Bpev01.c1283.g0001.m0001	0.467986804	0.13086644	0.38391403	nonDEG
Bpev01.c2656.g0004.m0001	-0.238765881	0.46733418	0.79597116	nonDEG
Bpev01.c3204.g0001.m0001	0.527656735	0.14355633	0.40750942	nonDEG
Bpev01.c1838.g0006.m0002	0.116438074	0.7211265	0.97092353	nonDEG
Bpev01.c0727.g0013.m0001	-0.165994806	0.71929837	0.96997817	nonDEG
Bpev01.c0647.g0003.m0001	-0.405429255	0.19180993	0.48825703	nonDEG
Bpev01.c0335.g0012.m0001	-0.221891899	0.6595996	0.93220195	nonDEG
Bpev01.c0620.g0005.m0001	0.360388326	0.67965205	0.94146687	nonDEG
Bpev01.c1384.g0003.m0001	0.411641878	0.3250841	0.65928291	nonDEG
Bpev01.c0094.g0099.m0001	-0.290349863	0.34383026	0.68030635	nonDEG
Bpev01.c0088.g0055.m0001	-0.001575523	0.99617558	1	nonDEG
Bpev01.c1185.g0006.m0001	-0.478641383	0.18148576	0.47301854	nonDEG
Bpev01.c0565.g0007.m0002	-2.437181176	0.01109645	0.06694023	nonDEG
Bpev01.c0815.g0005.m0001	-0.212937552	0.49039395	0.81462965	nonDEG

Bpev01.c0428.g0018.m0001	0.09719158	0.79075378	1	nonDEG
Bpev01.c0027.g0004.m0001	1.237324455	0.06673608	0.24462899	nonDEG
Bpev01.c0449.g0024.m0001	-0.269961899	0.4496669	0.7811261	nonDEG
Bpev01.c0932.g0008.m0001	-0.340475854	0.42059484	0.75205851	nonDEG
Bpev01.c0264.g0034.m0001	-3.135269212	0.66576562	0.93220195	nonDEG
Bpev01.c1132.g0002.m0001	-0.765281069	0.03919953	0.16870961	nonDEG
Bpev01.c0504.g0009.m0001	-2.81053421	0.00799103	0.05219611	nonDEG
Bpev01.c1100.g0004.m0001	-0.211962365	0.5441851	0.86063566	nonDEG
Bpev01.c0159.g0017.m0001	1.186714926	0.08407447	0.28526133	nonDEG
Bpev01.c0396.g0026.m0001	-0.851189224	0.00846412	0.054548	nonDEG
Bpev01.c0488.g0025.m0001	-0.234329241	0.60818138	0.91194681	nonDEG
Bpev01.c0540.g0008.m0001	-1.097516026	0.0332811	0.14944119	nonDEG
Bpev01.c2637.g0003.m0001	-0.452704705	0.17338192	0.46079176	nonDEG
Bpev01.c0038.g0031.m0003	0.098233218	0.77161924	1	nonDEG
Bpev01.c0805.g0005.m0001	-0.180032426	0.56008487	0.87489858	nonDEG
Bpev01.c0264.g0030.m0001	-4.049354376	0.38536956	0.71901413	nonDEG
Bpev01.c1638.g0010.m0001	-0.58513688	0.05714048	0.21991878	nonDEG
Bpev01.c1158.g0004.m0001	1.711956092	0.00887957	0.05645911	nonDEG
Bpev01.c1135.g0002.m0001	-0.77099971	0.01493691	0.08332912	nonDEG
Bpev01.c0483.g0017.m0001	-0.427407016	0.1540444	0.42632575	nonDEG
Bpev01.c1688.g0008.m0001	0.370099735	0.35002419	0.68751895	nonDEG
Bpev01.c0470.g0014.m0001	0.030857977	0.92720495	1	nonDEG
Bpev01.c2874.g0002.m0001	0.813112776	0.01774788	0.09439562	nonDEG
Bpev01.c0142.g0101.m0001	0.210814314	0.56596946	0.87900686	nonDEG
Bpev01.c1163.g0006.m0001	-0.670536162	0.09559945	0.3107035	nonDEG
Bpev01.c0190.g0044.m0001	-0.246194641	0.44161515	0.77306942	nonDEG
Bpev01.c1104.g0030.m0001	0.09633714	0.88351947	1	nonDEG
Bpev01.c0073.g0011.m0001	-0.019897901	0.95554796	1	nonDEG
Bpev01.c0357.g0077.m0001	0.435337703	0.15026746	0.42026637	nonDEG
Bpev01.c0044.g0055.m0001	-0.628070256	0.0723583	0.25838864	nonDEG
Bpev01.c0458.g0021.m0001	0.585140618	0.34996247	0.68751895	nonDEG
Bpev01.c0112.g0009.m0001	0.968503319	0.01761737	0.0939051	nonDEG
Bpev01.c1079.g0003.m0001	1.790980753	0.08734094	0.29241784	nonDEG
Bpev01.c1339.g0006.m0001	0.274090315	0.39901496	0.73210355	nonDEG
Bpev01.c2310.g0001.m0001	1.656160609	0.25812656	0.57830956	nonDEG
Bpev01.c0069.g0005.m0001	-0.475399574	0.15872062	0.4352656	nonDEG
Bpev01.c1382.g0024.m0001	0.461044476	0.17341763	0.46079179	nonDEG
Bpev01.c0892.g0026.m0001	-0.062233011	0.85226759	1	nonDEG
Bpev01.c0134.g0127.m0001	0.089889389	0.77332263	1	nonDEG
Bpev01.c0096.g0026.m0001	-0.083503588	0.79449677	1	nonDEG
Bpev01.c1370.g0001.m0001	0.773357335	0.0225251	0.11256433	nonDEG
Bpev01.c1063.g0003.m0001	1.146810183	0.59198907	0.90098781	nonDEG
Bpev01.c0486.g0008.m0001	0.319992492	0.34932219	0.68681601	nonDEG

Bpev01.c0477.g0012.m0001	0.651686796	0.11930558	0.36152721	nonDEG
Bpev01.c2080.g0003.m0001	0.369250307	0.24270798	0.56031056	nonDEG
Bpev01.c0555.g0013.m0001	-0.587627552	0.06878834	0.24985316	nonDEG
Bpev01.c0522.g0004.m0001	-0.121808984	0.71801161	0.96896658	nonDEG
Bpev01.c0134.g0055.m0001	-0.731060055	0.02883912	0.13495388	nonDEG
Bpev01.c0278.g0038.m0001	0.112562165	0.74216046	0.98359129	nonDEG
Bpev01.c0403.g0037.m0002	0.870001023	0.0099816	0.06172408	nonDEG
Bpev01.c0301.g0013.m0001	-0.900294824	0.33905258	0.67504074	nonDEG
Bpev01.c0566.g0048.m0001	0.404329257	0.28507568	0.61244566	nonDEG
Bpev01.c0134.g0130.m0001	-0.558242712	0.13288368	0.38736138	nonDEG
Bpev01.c1602.g0004.m0001	0.279915071	0.35146247	0.68941257	nonDEG
Bpev01.c1172.g0006.m0001	0.12531091	0.69030533	0.94891196	nonDEG
Bpev01.c0449.g0046.m0002	-0.564860614	0.10861211	0.33863928	nonDEG
Bpev01.c0644.g0003.m0001	0.794498909	0.39926802	0.73224839	nonDEG
Bpev01.c1450.g0006.m0001	1.283089938	0.44760556	0.7789702	nonDEG
Bpev01.c0016.g0062.m0001	0.485966104	0.10387784	0.32875293	nonDEG
Bpev01.c0425.g0041.m0001	0.166724025	0.62451952	0.92373026	nonDEG
Bpev01.c1657.g0009.m0001	0.552428314	0.17743306	0.46621486	nonDEG
Bpev01.c0158.g0005.m0001	0.161431195	0.58853107	0.89822004	nonDEG
Bpev01.c0188.g0026.m0001	-0.309445762	0.32452866	0.65856477	nonDEG
Bpev01.c1456.g0011.m0001	-0.769697548	0.01432678	0.08075168	nonDEG
Bpev01.c0213.g0021.m0001	-0.75641541	0.17641376	0.46487724	nonDEG
Bpev01.c1031.g0003.m0001	-1.554796778	0.0314836	0.14349163	nonDEG
Bpev01.c0043.g0013.m0001	-0.228137115	0.45774592	0.78707915	nonDEG
Bpev01.c1254.g0001.m0001	1.226005428	0.1378527	0.39780778	nonDEG
Bpev01.c0022.g0086.m0001	0.777909275	0.08062937	0.27791219	nonDEG
Bpev01.c0407.g0010.m0001	-0.36928557	0.22478407	0.53510304	nonDEG
Bpev01.c2249.g0004.m0001	0.574619125	0.12200032	0.36734861	nonDEG
Bpev01.c0980.g0009.m0001	0.2819844	0.37140187	0.71094996	nonDEG
Bpev01.c0154.g0077.m0001	0.296583262	0.34317913	0.68005187	nonDEG
Bpev01.c0887.g0013.m0001	-2.331364033	0.21237123	0.51860992	nonDEG
Bpev01.c1401.g0002.m0001	0.399334276	0.2145509	0.52176282	nonDEG
Bpev01.c0169.g0040.m0001	-0.207090884	0.5156823	0.8358295	nonDEG
Bpev01.c0357.g0056.m0001	-0.576481693	0.1055058	0.33219623	nonDEG
Bpev01.c0222.g0017.m0001	1.118419469	0.41192245	0.74556317	nonDEG
Bpev01.c0384.g0011.m0001	-0.230346913	0.55070933	0.86636733	nonDEG
Bpev01.c1390.g0011.m0001	-1.265211531	0.00881158	0.05616697	nonDEG
Bpev01.c0561.g0001.m0001	-0.389915746	0.22657067	0.53820818	nonDEG
Bpev01.c0357.g0070.m0001	0.540446889	0.10101128	0.32266397	nonDEG
Bpev01.c0919.g0009.m0001	0.50833811	0.30582533	0.63698826	nonDEG
Bpev01.c1414.g0007.m0001	-0.82550071	0.01294866	0.07496474	nonDEG
Bpev01.c0038.g0155.m0001	0.599508804	0.20763744	0.51177484	nonDEG
Bpev01.c2298.g0001.m0001	0.613340837	0.30301831	0.63467163	nonDEG

Bpev01.c1579.g0010.m0001	0.871219454	0.01116765	0.06723197	nonDEG
Bpev01.c0298.g0019.m0001	0.510031733	0.09703711	0.31374799	nonDEG
Bpev01.c1250.g0010.m0002	-0.190685249	0.53891498	0.85645745	nonDEG
Bpev01.c0516.g0002.m0001	1.283846511	0.05778296	0.22169588	nonDEG
Bpev01.c1155.g0001.m0001	-0.057661104	0.85220422	1	nonDEG
Bpev01.c1117.g0015.m0001	-0.467120616	0.13331368	0.38830725	nonDEG
Bpev01.c1480.g0001.m0001	0.993320451	0.34579241	0.68239121	nonDEG
Bpev01.c0717.g0022.m0001	0.7557808	0.0260334	0.12544001	nonDEG
Bpev01.c0147.g0012.m0001	0.026248793	0.9759064	1	nonDEG
Bpev01.c1076.g0003.m0001	-0.732586273	0.01740093	0.09314188	nonDEG
Bpev01.c0022.g0146.m0001	-0.090027256	0.85353505	1	nonDEG
Bpev01.c2393.g0004.m0001	0.670697724	0.21549696	0.52238538	nonDEG
Bpev01.c2136.g0005.m0001	-0.254001066	0.47543946	0.80407091	nonDEG
Bpev01.c0015.g0037.m0001	-0.431109634	0.18115848	0.47266902	nonDEG
Bpev01.c1280.g0008.m0001	-0.174022391	0.716953	0.96831905	nonDEG
Bpev01.c0518.g0040.m0001	-0.029300855	0.95600143	1	nonDEG
Bpev01.c0655.g0001.m0001	-0.341118353	0.63223811	0.92831514	nonDEG
Bpev01.c0120.g0058.m0001	-0.618623604	0.21644504	0.52297484	nonDEG
Bpev01.c0094.g0072.m0001	0.977080846	0.02006308	0.10307802	nonDEG
Bpev01.c1240.g0004.m0001	0.137985541	0.65655074	0.93220195	nonDEG
Bpev01.c0043.g0006.m0001	0.1024109	0.74153902	0.98341259	nonDEG
Bpev01.c0052.g0027.m0001	-0.051939582	0.93538424	1	nonDEG
Bpev01.c1468.g0010.m0001	0.756170815	0.01953568	0.1011273	nonDEG
Bpev01.c0052.g0013.m0001	-0.544663128	0.10199087	0.32481559	nonDEG
Bpev01.c1398.g0012.m0001	-0.275609906	0.72477092	0.97315991	nonDEG
Bpev01.c0051.g0079.m0001	0.09814085	0.80044627	1	nonDEG
Bpev01.c0225.g0002.m0001	-0.095094082	0.83708965	1	nonDEG
Bpev01.c0387.g0003.m0001	0.185053429	0.56628476	0.87935737	nonDEG
Bpev01.c0617.g0002.m0001	-1.774926774	0.05167926	0.20514911	nonDEG
Bpev01.c1328.g0007.m0001	0.600553245	0.15739536	0.43296225	nonDEG
Bpev01.c0050.g0002.m0001	-0.353076845	0.35644479	0.69464969	nonDEG
Bpev01.c0996.g0029.m0001	-2.827671286	0.03732462	0.16283337	nonDEG
Bpev01.c1453.g0007.m0001	0.790784886	0.05203365	0.20616669	nonDEG
Bpev01.c0616.g0016.m0001	0.594815241	0.09353864	0.30603291	nonDEG
Bpev01.c0684.g0004.m0001	0.80388243	0.03741163	0.16311625	nonDEG
Bpev01.c1411.g0011.m0001	1.178597782	0.01338779	0.07679719	nonDEG
Bpev01.c1118.g0001.m0001	0.343116632	0.25825971	0.57851983	nonDEG
Bpev01.c0449.g0047.m0001	0.314935166	0.48608165	0.81287415	nonDEG
Bpev01.c0577.g0017.m0001	0.104588891	0.73756859	0.98081497	nonDEG
Bpev01.c0659.g0012.m0001	-4.049371943	0.38381304	0.71813093	nonDEG
Bpev01.c0286.g0015.m0001	0.62716294	0.06967674	0.25192994	nonDEG
Bpev01.c0438.g0012.m0002	0.621717296	0.17958084	0.46993012	nonDEG
Bpev01.c0522.g0022.m0001	0.530000755	0.14398919	0.40817503	nonDEG

Bpev01.c0138.g0006.m0002	0.897152151	0.01857704	0.09755622	nonDEG
Bpev01.c1520.g0008.m0001	0.176262719	0.57022975	0.88307974	nonDEG
Bpev01.c2361.g0002.m0001	0.181607342	0.55064824	0.86636733	nonDEG
Bpev01.c0457.g0041.m0001	-0.085771123	0.82024265	1	nonDEG
Bpev01.c0146.g0016.m0002	0.018182488	0.96111642	1	nonDEG
Bpev01.c1758.g0001.m0001	0.658178252	0.08906071	0.29638887	nonDEG
Bpev01.c0230.g0018.m0001	0.506333251	0.12874296	0.38026233	nonDEG
Bpev01.c0327.g0078.m0001	0.949300801	0.81008084	1	nonDEG
Bpev01.c0702.g0013.m0001	-0.48164517	0.29009627	0.61914151	nonDEG
Bpev01.c1577.g0018.m0001	-1.185291677	0.2659104	0.58973453	nonDEG
Bpev01.c1280.g0012.m0001	-0.915432648	0.01268937	0.07390844	nonDEG
Bpev01.c1087.g0004.m0001	1.340901387	0.09827	0.31658685	nonDEG
Bpev01.c0155.g0015.m0001	-0.274395143	0.43606301	0.76851406	nonDEG
Bpev01.c0036.g0011.m0001	-0.409194494	0.68612774	0.94600738	nonDEG
Bpev01.c1006.g0015.m0001	-0.815405478	0.00773203	0.05084281	nonDEG
Bpev01.c0114.g0068.m0001	0.257760443	0.50052628	0.82309201	nonDEG
Bpev01.c0088.g0001.m0001	0.318490355	0.29908267	0.62985154	nonDEG
Bpev01.c0051.g0087.m0001	0.557513943	0.60494424	0.90973404	nonDEG
Bpev01.c2715.g0002.m0001	0.097636392	0.91152581	1	nonDEG
Bpev01.c0038.g0117.m0001	-0.265913871	0.78819513	1	nonDEG
Bpev01.c0420.g0009.m0001	0.729890625	0.01554427	0.08579074	nonDEG
Bpev01.c0135.g0055.m0001	-0.491188825	0.13212741	0.38586829	nonDEG
Bpev01.c0340.g0012.m0001	-0.807015074	0.10634675	0.33357023	nonDEG
Bpev01.c1767.g0010.m0001	-0.628886285	0.04866768	0.1962641	nonDEG
Bpev01.c1657.g0014.m0002	0.023099915	0.94305914	1	nonDEG
Bpev01.c0051.g0158.m0001	-0.012181798	0.97175631	1	nonDEG
Bpev01.c0080.g0093.m0001	0.520900479	0.19723621	0.49699072	nonDEG
Bpev01.c0486.g0001.m0001	-0.032567699	0.91628556	1	nonDEG
Bpev01.c0050.g0060.m0001	0.610458674	0.30161241	0.63294267	nonDEG
Bpev01.c2046.g0008.m0002	0.370180649	0.26496588	0.58848213	nonDEG
Bpev01.c0135.g0040.m0001	0.219653001	0.48159621	0.81038968	nonDEG
Bpev01.c2319.g0002.m0001	3.114429638	0.02032919	0.10400955	nonDEG
Bpev01.c0275.g0041.m0001	-0.490594581	0.25635335	0.57621593	nonDEG
Bpev01.c2037.g0001.m0001	0.331638435	0.31121685	0.64183688	nonDEG
Bpev01.c0052.g0009.m0001	-0.044704585	0.9558217	1	nonDEG
Bpev01.c1332.g0005.m0001	0.133248792	0.71228522	0.96502207	nonDEG
Bpev01.c0224.g0049.m0001	0.817376341	0.01548878	0.0855486	nonDEG
Bpev01.c0692.g0002.m0001	-0.190620056	0.60356835	0.90856408	nonDEG
Bpev01.c0051.g0166.m0002	0.434508373	0.16663272	0.44851218	nonDEG
Bpev01.c0488.g0015.m0001	0.477606396	0.54735724	0.86358592	nonDEG
Bpev01.c0575.g0020.m0001	0.071375595	0.85663859	1	nonDEG
Bpev01.c0134.g0146.m0002	0.344635077	0.27382201	0.59986949	nonDEG
Bpev01.c1758.g0010.m0001	0.34933078	0.24408559	0.56179272	nonDEG

Bpev01.c1874.g0005.m0001	-0.309911323	0.35774895	0.69590137	nonDEG
Bpev01.c0974.g0002.m0001	-0.582333688	0.08257428	0.28207065	nonDEG
Bpev01.c0142.g0021.m0001	0.609344362	0.05143357	0.20439428	nonDEG
Bpev01.c0364.g0025.m0001	0.334090321	0.26195335	0.58420127	nonDEG
Bpev01.c0343.g0008.m0001	-0.267052832	0.45735782	0.78673254	nonDEG
Bpev01.c0346.g0007.m0001	-0.121462336	0.72583147	0.97377891	nonDEG
Bpev01.c0053.g0043.m0001	-1.737924116	0.02845398	0.13357624	nonDEG
Bpev01.c0066.g0039.m0001	0.854318487	0.0332986	0.14945027	nonDEG
Bpev01.c0134.g0110.m0001	-0.550603014	0.15233412	0.42379077	nonDEG
Bpev01.c0443.g0011.m0001	0.284790075	0.53104297	0.84997731	nonDEG
Bpev01.c0213.g0058.m0001	0.715197446	0.02443911	0.1198306	nonDEG
Bpev01.c0210.g0033.m0001	-0.559563678	0.07450318	0.26342771	nonDEG
Bpev01.c0403.g0033.m0001	-0.799970425	0.10910422	0.33938222	nonDEG
Bpev01.c0257.g0018.m0001	0.214162579	0.48432178	0.81256284	nonDEG
Bpev01.c1664.g0002.m0001	0.649745119	0.14436013	0.40902962	nonDEG
Bpev01.c0158.g0020.m0001	0.608549117	0.2934817	0.62331777	nonDEG
Bpev01.c0044.g0045.m0001	-0.255706119	0.41906587	0.75073816	nonDEG
Bpev01.c1086.g0012.m0001	-0.064555003	0.84573522	1	nonDEG
Bpev01.c0440.g0003.m0001	0.961444365	0.03290941	0.14806973	nonDEG
Bpev01.c0101.g0030.m0001	0.112010703	0.71643046	0.96813962	nonDEG
Bpev01.c0038.g0139.m0001	-0.737692247	0.0320267	0.14520776	nonDEG
Bpev01.c0931.g0001.m0001	-0.416320754	0.23812151	0.55491488	nonDEG
Bpev01.c1287.g0003.m0001	-0.218610646	0.53806625	0.8555297	nonDEG
Bpev01.c0105.g0015.m0002	-0.754900391	0.13017446	0.38283776	nonDEG
Bpev01.c0029.g0110.m0001	-0.063095064	0.83939219	1	nonDEG
Bpev01.c0826.g0011.m0001	0.771812144	0.0330181	0.14844529	nonDEG
Bpev01.c0274.g0004.m0001	1.233064752	0.05683725	0.21909705	nonDEG
Bpev01.c0679.g0007.m0001	-0.811522169	0.11670787	0.3557838	nonDEG
Bpev01.c1300.g0007.m0001	2.301892806	1	1	nonDEG
Bpev01.c0545.g0008.m0001	0.290484432	0.37408004	0.71343239	nonDEG
Bpev01.c0547.g0017.m0001	-0.123521176	0.7054723	0.96021078	nonDEG
Bpev01.c1040.g0011.m0001	-0.329753818	0.30517409	0.63639726	nonDEG
Bpev01.c1333.g0006.m0001	-0.227187115	0.59988136	0.90630305	nonDEG
Bpev01.c0395.g0005.m0001	0.035419349	0.92756	1	nonDEG
Bpev01.c0483.g0009.m0001	0.788245605	0.01183104	0.0699105	nonDEG
Bpev01.c0649.g0020.m0001	0.906540847	0.84390837	1	nonDEG
Bpev01.c0136.g0027.m0001	0.067880077	0.8333314	1	nonDEG
Bpev01.c0094.g0097.m0001	-0.263799293	0.67671568	0.93918227	nonDEG
Bpev01.c2783.g0004.m0001	0.390485039	0.4458782	0.77748079	nonDEG
Bpev01.c0152.g0007.m0001	-0.064683664	0.86561708	1	nonDEG
Bpev01.c0566.g0068.m0001	-1.151277706	0.01979208	0.10209551	nonDEG
Bpev01.c0205.g0003.m0001	-0.541429988	0.1021775	0.32529505	nonDEG
Bpev01.c1484.g0013.m0001	-0.074125819	0.80412362	1	nonDEG

Bpev01.c1698.g0002.m0001	0.127084265	1	1	nonDEG
Bpev01.c0016.g0069.m0001	-0.791970231	0.05803856	0.22233685	nonDEG
Bpev01.c0571.g0014.m0001	-0.191953179	0.59554388	0.90339652	nonDEG
Bpev01.c0328.g0042.m0001	-0.611967168	0.05532971	0.21488805	nonDEG
Bpev01.c0566.g0040.m0001	-0.6680068	0.03347532	0.15006547	nonDEG
Bpev01.c0275.g0042.m0001	-0.012981705	0.98745011	1	nonDEG
Bpev01.c0636.g0005.m0001	-0.054358696	0.92043001	1	nonDEG
Bpev01.c1374.g0001.m0001	0.497236777	0.16896923	0.45245455	nonDEG
Bpev01.c0661.g0017.m0001	0.130947047	0.67671518	0.93918227	nonDEG
Bpev01.c0004.g0013.m0001	-0.43389561	0.14516726	0.41059004	nonDEG
Bpev01.c0486.g0005.m0001	0.10017816	0.76098594	0.99448062	nonDEG
Bpev01.c0848.g0016.m0001	2.570826604	0.01868683	0.09792124	nonDEG
Bpev01.c0016.g0034.m0001	-0.155560774	0.67871703	0.94053618	nonDEG
Bpev01.c0661.g0015.m0001	0.385894436	0.23696467	0.55374958	nonDEG
Bpev01.c2530.g0001.m0001	0.858589444	0.17436519	0.46201827	nonDEG
Bpev01.c0062.g0075.m0001	-0.361430289	0.32855439	0.66376013	nonDEG
Bpev01.c0281.g0068.m0001	-0.812329446	0.24928008	0.56905679	nonDEG
Bpev01.c0652.g0008.m0001	-0.173218147	0.57274466	0.88490763	nonDEG
Bpev01.c1055.g0003.m0001	0.865389729	0.08270677	0.2822962	nonDEG
Bpev01.c0841.g0003.m0002	1.312921512	0.02766641	0.13092292	nonDEG
Bpev01.c0224.g0019.m0001	1.402405402	0.02647499	0.12692086	nonDEG
Bpev01.c0710.g0015.m0001	0.727771457	0.36387371	0.70245695	nonDEG
Bpev01.c0018.g0122.m0002	-0.043370301	0.93213909	1	nonDEG
Bpev01.c0015.g0261.m0001	-0.652779824	0.06280095	0.23464872	nonDEG
Bpev01.c1289.g0004.m0001	0.342084014	0.29069811	0.6198654	nonDEG
Bpev01.c1427.g0006.m0002	-0.58293263	0.09536526	0.31021634	nonDEG
Bpev01.c0990.g0001.m0001	-0.86461954	0.00894959	0.05685522	nonDEG
Bpev01.c3379.g0001.m0001	-0.558211131	0.17326147	0.46067959	nonDEG
Bpev01.c1788.g0003.m0001	0.401278938	0.5703688	0.88309136	nonDEG
Bpev01.c0169.g0021.m0001	-0.481861994	0.12030656	0.36390821	nonDEG
Bpev01.c0597.g0018.m0002	-0.726934736	0.02324331	0.11527286	nonDEG
Bpev01.c0023.g0027.m0001	0.152020105	0.69093408	0.94933666	nonDEG
Bpev01.c1063.g0001.m0001	0.040823978	0.91916873	1	nonDEG
Bpev01.c0229.g0002.m0001	-0.436584786	0.19117538	0.48714724	nonDEG
Bpev01.c1623.g0014.m0001	1.033544895	0.31153311	0.64219756	nonDEG
Bpev01.c1455.g0015.m0001	1.317858425	0.03925188	0.16888864	nonDEG
Bpev01.c0000.g0025.m0001	-0.176624804	0.58528391	0.89545007	nonDEG
Bpev01.c0837.g0005.m0001	-1.507108857	0.06936527	0.25111217	nonDEG
Bpev01.c0120.g0033.m0001	-0.236737566	0.45359394	0.78378541	nonDEG
Bpev01.c0281.g0049.m0001	0.146245147	0.67752079	0.9398479	nonDEG
Bpev01.c0395.g0056.m0001	0.722620878	0.47911874	0.80760243	nonDEG
Bpev01.c0848.g0025.m0001	0.73886779	0.14179766	0.40438316	nonDEG
Bpev01.c0591.g0001.m0001	0.123601494	1	1	nonDEG

Bpev01.c3916.g0001.m0001	0.704228205	0.08263292	0.28213515	nonDEG
Bpev01.c0062.g0049.m0001	-0.264050848	0.51727428	0.83744157	nonDEG
Bpev01.c0402.g0031.m0002	2.392149159	0.0565625	0.21835082	nonDEG
Bpev01.c1173.g0002.m0001	0.050537309	0.87441387	1	nonDEG
Bpev01.c0356.g0016.m0001	-0.320586533	0.59145451	0.9005801	nonDEG
Bpev01.c0613.g0003.m0001	-0.148413022	0.62080347	0.92132505	nonDEG
Bpev01.c1020.g0011.m0001	1.415860944	0.01622027	0.08859155	nonDEG
Bpev01.c0356.g0021.m0001	0.232822847	0.48150163	0.81037973	nonDEG
Bpev01.c1260.g0002.m0001	0.325972741	0.29574965	0.62581808	nonDEG
Bpev01.c0509.g0011.m0001	0.181810078	0.83190369	1	nonDEG
Bpev01.c0717.g0013.m0001	0.181387845	0.57701542	0.88846087	nonDEG
Bpev01.c0027.g0139.m0001	0.68704478	0.04447273	0.18490753	nonDEG
Bpev01.c0157.g0040.m0001	0.538762047	0.11666352	0.355718	nonDEG
Bpev01.c1087.g0012.m0002	0.110920093	0.74779862	0.9858685	nonDEG
Bpev01.c0094.g0017.m0001	0.750314058	0.02381593	0.11753851	nonDEG
Bpev01.c0443.g0007.m0003	-0.313361532	0.29433252	0.62419922	nonDEG
Bpev01.c1229.g0002.m0001	-1.508588534	0.01055654	0.06435576	nonDEG
Bpev01.c0257.g0028.m0001	-0.144729084	0.62957422	0.92680274	nonDEG
Bpev01.c0440.g0002.m0001	0.111015419	0.76177494	0.99508385	nonDEG
Bpev01.c1633.g0002.m0001	0.681185769	0.37160327	0.71119677	nonDEG
Bpev01.c1335.g0017.m0001	-0.846423611	0.12893249	0.38050137	nonDEG
Bpev01.c0062.g0039.m0001	0.297703936	0.64618365	0.93220195	nonDEG
Bpev01.c1756.g0007.m0001	0.403689842	0.57414712	0.88595855	nonDEG
Bpev01.c0500.g0010.m0001	-0.244083402	0.90904846	1	nonDEG
Bpev01.c0051.g0103.m0001	0.176047775	0.60604904	0.91034513	nonDEG
Bpev01.c0996.g0010.m0001	0.602008114	0.05807781	0.22236346	nonDEG
Bpev01.c0026.g0003.m0001	1.078454084	0.06680364	0.24472777	nonDEG
Bpev01.c0036.g0049.m0001	0.482230351	0.12870428	0.38022332	nonDEG
Bpev01.c0541.g0012.m0001	0.569726515	0.57801778	0.88929983	nonDEG
Bpev01.c1382.g0025.m0001	-0.067955949	0.86794304	1	nonDEG
Bpev01.c0837.g0003.m0001	0.116375378	0.72043983	0.97054099	nonDEG
Bpev01.c0044.g0116.m0001	-0.472857281	0.59300854	0.90183045	nonDEG
Bpev01.c0281.g0080.m0001	-0.22616835	0.49281532	0.81673271	nonDEG
Bpev01.c0442.g0021.m0001	-0.819026581	0.03213539	0.14563329	nonDEG
Bpev01.c1099.g0005.m0001	0.378264443	0.22169095	0.53087955	nonDEG
Bpev01.c0884.g0007.m0001	-0.247812743	0.41485457	0.74697103	nonDEG
Bpev01.c2858.g0003.m0001	1.142473792	0.00761249	0.05029267	nonDEG
Bpev01.c2719.g0002.m0001	-0.50359314	0.46348219	0.7923187	nonDEG
Bpev01.c1529.g0003.m0001	-0.419229311	0.20495623	0.50737525	nonDEG
Bpev01.c0134.g0078.m0001	1.836666693	0.24109874	0.55834006	nonDEG
Bpev01.c0713.g0002.m0001	0.116231759	0.73289492	0.97781941	nonDEG
Bpev01.c1952.g0005.m0001	-0.125490449	0.83623438	1	nonDEG
Bpev01.c0564.g0034.m0001	0.72226375	0.02606879	0.1255216	nonDEG

Bpev01.c0051.g0111.m0001	1.425561501	0.30968885	0.63987438	nonDEG
Bpev01.c0484.g0019.m0001	-0.21907785	0.49311471	0.81697359	nonDEG
Bpev01.c1167.g0022.m0001	0.247671743	0.47848471	0.80699578	nonDEG
Bpev01.c0438.g0012.m0001	1.103636357	0.01076234	0.06536674	nonDEG
Bpev01.c0485.g0035.m0001	0.227853778	0.45784275	0.78710773	nonDEG
Bpev01.c0080.g0056.m0001	0.213661542	0.50671065	0.82857808	nonDEG
Bpev01.c1868.g0003.m0001	1.211049888	0.55982958	0.87473186	nonDEG
Bpev01.c0695.g0009.m0001	0.487317207	0.16615626	0.44773218	nonDEG
Bpev01.c1365.g0008.m0001	1.516755766	0.00917257	0.05792174	nonDEG
Bpev01.c0449.g0045.m0002	0.190422915	0.80245441	1	nonDEG
Bpev01.c0289.g0022.m0001	0.15137489	0.71474639	0.96706459	nonDEG
Bpev01.c0120.g0032.m0001	-0.424481165	0.3336827	0.66919477	nonDEG
Bpev01.c0998.g0004.m0001	0.657177269	0.05904248	0.22459639	nonDEG
Bpev01.c0015.g0205.m0001	0.649762572	0.04446635	0.18490753	nonDEG
Bpev01.c0015.g0187.m0001	-0.350445834	0.34413339	0.68054939	nonDEG
Bpev01.c0518.g0004.m0001	-0.049549651	0.87784812	1	nonDEG
Bpev01.c0514.g0009.m0001	-0.564251548	0.22588102	0.53712476	nonDEG
Bpev01.c0753.g0007.m0001	0.682868348	0.04236174	0.17851234	nonDEG
Bpev01.c1634.g0002.m0001	-0.203888481	0.52867103	0.84796482	nonDEG
Bpev01.c0816.g0001.m0001	0.222872472	0.56657896	0.87944311	nonDEG
Bpev01.c1520.g0010.m0002	-0.575608547	0.07963318	0.27541367	nonDEG
Bpev01.c0051.g0019.m0002	-0.082327495	0.86261361	1	nonDEG
Bpev01.c0027.g0104.m0001	-0.081194583	0.80178881	1	nonDEG
Bpev01.c1225.g0009.m0001	-0.557760387	0.07040546	0.25378552	nonDEG
Bpev01.c0115.g0059.m0001	0.536380113	0.09931215	0.31896689	nonDEG
Bpev01.c0452.g0030.m0002	0.183453104	0.69096031	0.94933666	nonDEG
Bpev01.c0575.g0003.m0001	1.290003222	0.10963345	0.34048941	nonDEG
Bpev01.c0999.g0007.m0001	0.000662973	0.99863534	1	nonDEG
Bpev01.c0263.g0008.m0001	-2.760522409	0.01171209	0.06938868	nonDEG
Bpev01.c0556.g0015.m0001	-0.54642993	0.6097481	0.9131385	nonDEG
Bpev01.c0505.g0028.m0001	0.396001516	0.19857893	0.49867483	nonDEG
Bpev01.c0024.g0019.m0001	-0.110363895	0.72064273	0.97062506	nonDEG
Bpev01.c0299.g0003.m0001	0.115069433	0.86569854	1	nonDEG
Bpev01.c1101.g0008.m0001	-0.313417198	0.33281893	0.66843835	nonDEG
Bpev01.c0051.g0069.m0001	-0.591772232	0.07962143	0.27540537	nonDEG
Bpev01.c0169.g0010.m0001	1.508940345	0.0179446	0.09513255	nonDEG
Bpev01.c0444.g0020.m0001	-0.224701642	0.61523234	0.91716533	nonDEG
Bpev01.c1341.g0003.m0001	-0.423302159	0.1788603	0.46881944	nonDEG
Bpev01.c0538.g0002.m0001	0.485581285	0.17155575	0.45746646	nonDEG
Bpev01.c0605.g0009.m0001	-0.88237694	0.00859837	0.05516672	nonDEG
Bpev01.c0237.g0058.m0001	-0.218988079	0.50136486	0.82392023	nonDEG
Bpev01.c0044.g0085.m0001	1.067780893	0.18394853	0.47708091	nonDEG
Bpev01.c0531.g0012.m0001	0.70132637	0.04757338	0.19314752	nonDEG

Bpev01.c1470.g0005.m0001	0.672887514	0.64504683	0.93220195	nonDEG
Bpev01.c0161.g0008.m0001	-0.514860283	0.09741793	0.31449572	nonDEG
Bpev01.c1968.g0011.m0001	-0.475383765	0.1863184	0.4806089	nonDEG
Bpev01.c0112.g0015.m0001	0.176345709	0.698817	0.95539282	nonDEG
Bpev01.c0161.g0055.m0001	0.210242423	0.50686349	0.82857808	nonDEG
Bpev01.c0083.g0002.m0002	0.439016435	0.20993302	0.51523285	nonDEG
Bpev01.c1389.g0003.m0001	0.769110143	0.0651421	0.24040594	nonDEG
Bpev01.c0483.g0014.m0001	-0.071081214	0.8242279	1	nonDEG
Bpev01.c0722.g0002.m0001	4.34281474	0.29942276	0.63014748	nonDEG
Bpev01.c0713.g0003.m0002	0.347420077	0.25859108	0.57904183	nonDEG
Bpev01.c0550.g0001.m0001	-0.697090078	0.03336835	0.14965256	nonDEG
Bpev01.c0974.g0003.m0001	-0.829042379	0.04123707	0.17515194	nonDEG
Bpev01.c0026.g0005.m0001	-0.185970207	0.61372345	0.91642447	nonDEG
Bpev01.c0157.g0082.m0001	0.057186912	0.88752554	1	nonDEG
Bpev01.c0210.g0011.m0003	0.488241786	0.43064334	0.76229386	nonDEG
Bpev01.c0430.g0015.m0001	0.100785104	0.76456466	0.99687634	nonDEG
Bpev01.c0843.g0010.m0001	-0.524746974	0.08336082	0.28367347	nonDEG
Bpev01.c0324.g0014.m0001	0.446081849	0.20703732	0.51059493	nonDEG
Bpev01.c0214.g0054.m0001	1.18352029	0.05685459	0.2191049	nonDEG
Bpev01.c0280.g0021.m0001	0.771826246	0.01364136	0.07784223	nonDEG
Bpev01.c0616.g0006.m0001	0.288974032	0.33154113	0.66726109	nonDEG
Bpev01.c0438.g0009.m0001	-0.243122156	0.69082	0.94930915	nonDEG
Bpev01.c0753.g0012.m0002	-0.057152194	0.85618799	1	nonDEG
Bpev01.c1518.g0005.m0001	-0.767236232	0.01720448	0.0924407	nonDEG
Bpev01.c0114.g0017.m0001	-0.273351615	0.37465246	0.71375625	nonDEG
Bpev01.c0864.g0015.m0001	-0.737415912	0.2688183	0.59367833	nonDEG
Bpev01.c0449.g0022.m0001	-0.588165301	0.20332059	0.50540868	nonDEG
Bpev01.c0145.g0017.m0001	-0.641534688	0.03751534	0.16349579	nonDEG
Bpev01.c0229.g0007.m0001	-0.576018118	0.06272474	0.23445321	nonDEG
Bpev01.c0817.g0003.m0001	-0.706326376	0.03522216	0.15599596	nonDEG
Bpev01.c1167.g0025.m0001	-0.503380603	0.32410517	0.65805955	nonDEG
Bpev01.c1132.g0004.m0001	-0.573186294	0.05759545	0.22117823	nonDEG
Bpev01.c1225.g0001.m0001	1.145328657	0.04575573	0.18807636	nonDEG
Bpev01.c1103.g0002.m0002	-0.740109592	0.13855757	0.39879669	nonDEG
Bpev01.c0044.g0011.m0001	0.589511607	0.05231217	0.20693605	nonDEG
Bpev01.c0147.g0014.m0001	0.618734201	0.06227405	0.23328406	nonDEG
Bpev01.c0261.g0069.m0001	-0.426941407	0.27560748	0.6021434	nonDEG
Bpev01.c0182.g0005.m0001	0.387122259	0.69787294	0.95454544	nonDEG
Bpev01.c0384.g0007.m0001	0.612979827	0.04444205	0.18489675	nonDEG
Bpev01.c2046.g0001.m0001	0.067301182	0.84462584	1	nonDEG
Bpev01.c1127.g0008.m0001	0.645729435	0.21901488	0.52706722	nonDEG
Bpev01.c0001.g0065.m0002	-0.815634007	0.01659854	0.0901059	nonDEG
Bpev01.c0350.g0017.m0001	1.266952715	0.01899345	0.09916007	nonDEG

Bpev01.c0443.g0023.m0001	-0.371497481	0.26865864	0.59346002	nonDEG
Bpev01.c0000.g0241.m0001	-0.739083249	0.0292092	0.13610156	nonDEG
Bpev01.c0560.g0008.m0001	0.513981211	0.10630215	0.33355671	nonDEG
Bpev01.c0243.g0038.m0001	-0.037949348	0.91826185	1	nonDEG
Bpev01.c0190.g0057.m0001	0.93475379	0.08203689	0.28081734	nonDEG
Bpev01.c0016.g0083.m0001	0.048760459	0.87139242	1	nonDEG
Bpev01.c1418.g0004.m0001	-0.575189118	0.09097716	0.30065969	nonDEG
Bpev01.c0015.g0111.m0001	-0.0879687	0.80749954	1	nonDEG
Bpev01.c1950.g0001.m0001	-0.989519918	0.30673353	0.63779608	nonDEG
Bpev01.c2103.g0002.m0001	-0.287306319	0.34832027	0.68526968	nonDEG
Bpev01.c0950.g0013.m0001	-0.338130899	0.78903523	1	nonDEG
Bpev01.c2183.g0002.m0001	0.067634419	0.83015704	1	nonDEG
Bpev01.c0411.g0007.m0001	-0.121796268	0.68486844	0.94518793	nonDEG
Bpev01.c0597.g0010.m0001	-0.795017941	0.02385808	0.11768735	nonDEG
Bpev01.c0488.g0014.m0001	0.272777536	0.37558089	0.71435107	nonDEG
Bpev01.c0901.g0024.m0001	0.05105916	0.86454957	1	nonDEG
Bpev01.c0015.g0050.m0001	-0.261528516	0.39267645	0.72562114	nonDEG
Bpev01.c1350.g0008.m0002	-0.073895784	0.91270325	1	nonDEG
Bpev01.c1767.g0002.m0001	-1.030423851	0.04344992	0.18200374	nonDEG
Bpev01.c0328.g0038.m0001	-0.554365908	0.09596921	0.31137732	nonDEG
Bpev01.c0444.g0012.m0002	-0.153453478	0.66813039	0.93262639	nonDEG
Bpev01.c0051.g0192.m0001	-0.023556456	0.94849455	1	nonDEG
Bpev01.c0264.g0040.m0001	0.501384518	0.09941996	0.31924349	nonDEG
Bpev01.c0732.g0003.m0001	-1.623197699	0.01340528	0.0768513	nonDEG
Bpev01.c0301.g0010.m0001	-0.896400215	0.00772713	0.05082191	nonDEG
Bpev01.c0371.g0009.m0001	-0.346309909	0.36936734	0.70930521	nonDEG
Bpev01.c0225.g0004.m0001	-0.23684838	0.63549613	0.93006545	nonDEG
Bpev01.c0142.g0061.m0002	-0.042417754	0.93945424	1	nonDEG
Bpev01.c0892.g0001.m0001	1.640626564	0.01886676	0.09872353	nonDEG
Bpev01.c0333.g0011.m0001	-0.3948754	0.22292774	0.53247624	nonDEG
Bpev01.c0129.g0024.m0001	0.792536096	0.10112734	0.32288735	nonDEG
Bpev01.c0145.g0034.m0001	-0.427330316	0.17054135	0.45538021	nonDEG
Bpev01.c1117.g0002.m0001	1.461872352	0.09238535	0.30367713	nonDEG
Bpev01.c1085.g0016.m0001	-0.534158976	0.08315303	0.28326107	nonDEG
Bpev01.c1657.g0008.m0001	-0.440602302	0.14040663	0.40173301	nonDEG
Bpev01.c1475.g0010.m0001	-0.23460016	0.47861263	0.80700614	nonDEG
Bpev01.c0094.g0026.m0001	-0.064309611	0.90520181	1	nonDEG
Bpev01.c0031.g0025.m0001	0.399005343	0.18147418	0.47301854	nonDEG
Bpev01.c0719.g0011.m0001	0.031108297	0.91901636	1	nonDEG
Bpev01.c0306.g0002.m0001	-0.375885068	0.60721732	0.91126524	nonDEG
Bpev01.c0980.g0010.m0001	0.196476759	0.55314418	0.8687599	nonDEG
Bpev01.c1733.g0004.m0001	0.393395869	0.21030997	0.51582252	nonDEG
Bpev01.c1406.g0004.m0001	-0.252189943	0.39614844	0.7292074	nonDEG

Bpev01.c0015.g0106.m0001	0.932328515	0.03243496	0.14651724	nonDEG
Bpev01.c0080.g0022.m0001	0.39791351	0.27815025	0.60534415	nonDEG
Bpev01.c0022.g0179.m0001	-0.017930519	0.96303101	1	nonDEG
Bpev01.c2862.g0001.m0001	-0.731332107	0.03293227	0.14814993	nonDEG
Bpev01.c0386.g0004.m0001	-0.07107198	0.82815836	1	nonDEG
Bpev01.c1120.g0007.m0001	0.079119898	0.81074942	1	nonDEG
Bpev01.c0015.g0060.m0001	0.391009022	0.20902643	0.51386463	nonDEG
Bpev01.c0405.g0014.m0001	-0.876062289	0.02357025	0.11660473	nonDEG
Bpev01.c0420.g0022.m0001	1.216954773	0.01134553	0.06790022	nonDEG
Bpev01.c0080.g0128.m0001	0.492935087	0.46979514	0.79847002	nonDEG
Bpev01.c0022.g0023.m0001	0.200177738	0.50846977	0.8297124	nonDEG
Bpev01.c0190.g0023.m0002	0.576193764	0.07901536	0.27379882	nonDEG
Bpev01.c1020.g0008.m0001	-0.205567247	0.49562805	0.81913398	nonDEG
Bpev01.c1148.g0009.m0001	-0.162053734	0.74684996	0.9856171	nonDEG
Bpev01.c0172.g0007.m0002	-0.961435644	0.00886148	0.05639283	nonDEG
Bpev01.c1764.g0005.m0001	-0.271694293	0.39970568	0.73277716	nonDEG
Bpev01.c0471.g0003.m0001	-0.130491821	0.74707229	0.9857402	nonDEG
Bpev01.c0826.g0002.m0001	1.386422383	0.01667044	0.09042956	nonDEG
Bpev01.c2327.g0001.m0001	-0.669093675	0.03186466	0.14467361	nonDEG
Bpev01.c1378.g0020.m0001	0.563743169	0.12608828	0.37528186	nonDEG
Bpev01.c1382.g0004.m0001	-0.319557642	0.32795999	0.66301429	nonDEG
Bpev01.c0349.g0045.m0001	0.102834513	0.74562552	0.98490016	nonDEG
Bpev01.c0439.g0016.m0001	0.747626945	0.19225134	0.48898721	nonDEG
Bpev01.c1329.g0004.m0001	-0.824152795	0.01342665	0.07690887	nonDEG
Bpev01.c1159.g0017.m0001	-0.796136601	0.29907426	0.62985154	nonDEG
Bpev01.c0066.g0065.m0001	-0.430703154	0.27074048	0.59609071	nonDEG
Bpev01.c0001.g0063.m0001	0.716307585	0.02676213	0.12764912	nonDEG
Bpev01.c0800.g0025.m0001	-0.108241563	0.72250566	0.97187084	nonDEG
Bpev01.c0037.g0019.m0001	0.649471116	0.27681555	0.60373189	nonDEG
Bpev01.c0241.g0007.m0001	0.313945028	0.5865923	0.89656578	nonDEG
Bpev01.c0588.g0034.m0001	0.120209732	0.69594702	0.95355042	nonDEG
Bpev01.c0114.g0025.m0001	0.764080925	0.07494854	0.26436739	nonDEG
Bpev01.c0329.g0006.m0001	0.639272784	0.07684431	0.26908318	nonDEG
Bpev01.c0327.g0029.m0001	-0.184880042	0.54990654	0.86589103	nonDEG
Bpev01.c0357.g0054.m0001	-1.089443382	0.01078434	0.06547331	nonDEG
Bpev01.c0066.g0012.m0001	-0.297522788	0.33413764	0.66949246	nonDEG
Bpev01.c0470.g0030.m0001	-1.443671262	0.04130376	0.17532301	nonDEG
Bpev01.c0996.g0021.m0001	-0.716919497	0.03310026	0.14874651	nonDEG
Bpev01.c0613.g0016.m0001	0.932555486	0.00985686	0.06116699	nonDEG
Bpev01.c0018.g0044.m0001	1.055217157	0.03284865	0.14786411	nonDEG
Bpev01.c1939.g0001.m0001	0.981937019	0.00798622	0.05217628	nonDEG
Bpev01.c0545.g0009.m0001	0.547031112	0.08741954	0.29258115	nonDEG
Bpev01.c0192.g0013.m0001	-1.395223108	0.06508399	0.24034215	nonDEG

Bpev01.c0088.g0067.m0001	-0.327526965	0.3387468	0.67476602	nonDEG
Bpev01.c1674.g0005.m0001	-0.099597077	0.75748423	0.99202368	nonDEG
Bpev01.c0422.g0007.m0001	-0.197580811	0.55895323	0.87418332	nonDEG
Bpev01.c0327.g0058.m0001	-0.798298277	0.34460198	0.68094628	nonDEG
Bpev01.c0129.g0030.m0001	0.092701502	0.84678399	1	nonDEG
Bpev01.c1256.g0006.m0001	0.751618416	0.02630065	0.1262869	nonDEG
Bpev01.c0828.g0017.m0001	-0.448090554	0.21406672	0.52112049	nonDEG
Bpev01.c0963.g0016.m0001	-0.346241214	0.25044044	0.57055534	nonDEG
Bpev01.c0439.g0025.m0001	-0.675340295	0.09505358	0.30951026	nonDEG
Bpev01.c0288.g0026.m0001	0.158693357	0.64870016	0.93220195	nonDEG
Bpev01.c0506.g0004.m0001	-0.679230859	0.04833661	0.19527671	nonDEG
Bpev01.c0240.g0014.m0001	0.555097004	0.07708339	0.26950675	nonDEG
Bpev01.c0918.g0006.m0001	0.971561647	0.00773479	0.05084957	nonDEG
Bpev01.c1687.g0007.m0001	1.728824602	0.04944691	0.19869883	nonDEG
Bpev01.c1029.g0014.m0001	0.398585957	0.59194504	0.90098781	nonDEG
Bpev01.c0441.g0010.m0001	-0.779758126	0.10867872	0.33870252	nonDEG
Bpev01.c0889.g0006.m0001	-0.141496425	0.6568149	0.93220195	nonDEG
Bpev01.c0101.g0012.m0001	0.72017467	0.01851949	0.09740847	nonDEG
Bpev01.c0190.g0040.m0001	-0.245725438	0.4460679	0.77757808	nonDEG
Bpev01.c1425.g0002.m0001	-4.117559482	0.01237238	0.07255587	nonDEG
Bpev01.c0037.g0026.m0001	0.588747851	0.13247587	0.38659368	nonDEG
Bpev01.c0373.g0011.m0001	-0.235006089	0.46258103	0.79160164	nonDEG
Bpev01.c0162.g0027.m0001	-0.323268065	0.39006153	0.72316943	nonDEG
Bpev01.c0517.g0008.m0001	-0.215580894	0.52528735	0.84493139	nonDEG
Bpev01.c0279.g0043.m0001	-0.162606308	0.5959404	0.90369187	nonDEG
Bpev01.c0518.g0005.m0001	-0.543692137	0.2460025	0.56463961	nonDEG
Bpev01.c0018.g0052.m0001	-0.684112217	0.08227091	0.28133038	nonDEG
Bpev01.c2911.g0003.m0001	0.45092422	0.19501017	0.49354405	nonDEG
Bpev01.c0118.g0004.m0001	0.54293738	0.35835199	0.69650163	nonDEG
Bpev01.c0044.g0063.m0001	0.884220434	0.01034758	0.06344981	nonDEG
Bpev01.c1041.g0007.m0001	-0.054814556	0.87416063	1	nonDEG
Bpev01.c0394.g0010.m0001	-0.912025382	0.01647306	0.08962309	nonDEG
Bpev01.c1104.g0019.m0001	0.463927645	0.1371259	0.39619681	nonDEG
Bpev01.c0161.g0029.m0002	-0.85973434	0.04549703	0.18737929	nonDEG
Bpev01.c0052.g0079.m0001	-0.197844089	0.53386358	0.85203247	nonDEG
Bpev01.c0726.g0016.m0001	0.643601985	0.05505689	0.21434451	nonDEG
Bpev01.c1782.g0014.m0001	0.666094686	0.02922777	0.13616525	nonDEG
Bpev01.c0867.g0005.m0001	0.666116279	0.06456566	0.23908785	nonDEG
Bpev01.c0015.g0167.m0001	0.139554718	0.68818265	0.94749832	nonDEG
Bpev01.c0764.g0013.m0001	-0.034320931	0.94703143	1	nonDEG
Bpev01.c0066.g0049.m0001	0.246252273	0.43540928	0.76749977	nonDEG
Bpev01.c0523.g0015.m0001	0.561853926	0.35799281	0.69623772	nonDEG
Bpev01.c0154.g0053.m0001	-0.00721541	0.98095647	1	nonDEG

Bpev01.c0514.g0005.m0001	-0.394925253	0.23202435	0.5463503	nonDEG
Bpev01.c0094.g0092.m0001	-0.83123761	0.00935864	0.05868712	nonDEG
Bpev01.c0488.g0019.m0001	0.242894843	0.41784683	0.74946644	nonDEG
Bpev01.c0365.g0012.m0003	0.666544215	0.04916753	0.19787324	nonDEG
Bpev01.c1446.g0011.m0001	-5.896477982	0.01749612	0.09344843	nonDEG
Bpev01.c0551.g0005.m0001	0.432599673	0.34968908	0.68726196	nonDEG
Bpev01.c0365.g0039.m0001	-0.362666997	0.34093164	0.67698375	nonDEG
Bpev01.c0498.g0001.m0001	0.34657278	0.26984668	0.59505741	nonDEG
Bpev01.c0045.g0004.m0001	-0.233957837	0.46574044	0.79442537	nonDEG
Bpev01.c0994.g0002.m0001	0.179002821	0.55050758	0.86636733	nonDEG
Bpev01.c0161.g0070.m0001	0.121729096	0.68323765	0.94395119	nonDEG
Bpev01.c0166.g0046.m0001	0.336788418	0.36109079	0.69934202	nonDEG
Bpev01.c1070.g0005.m0001	-0.362807321	0.40455569	0.73771634	nonDEG
Bpev01.c0874.g0011.m0001	-0.557667445	0.07488201	0.26419602	nonDEG
Bpev01.c0795.g0004.m0001	-0.342986796	0.31692748	0.64976864	nonDEG
Bpev01.c0080.g0094.m0001	-0.309696419	0.34140705	0.67777208	nonDEG
Bpev01.c2499.g0002.m0001	0.697100593	0.12034373	0.36392388	nonDEG
Bpev01.c0473.g0055.m0001	0.591837449	0.04981787	0.19972604	nonDEG
Bpev01.c0477.g0036.m0001	-0.316625098	0.71877625	0.9695398	nonDEG
Bpev01.c2890.g0005.m0001	1.250183108	0.48830066	0.81424325	nonDEG
Bpev01.c0063.g0026.m0001	0.266859035	0.46127353	0.79018404	nonDEG
Bpev01.c0455.g0028.m0001	-0.837632587	0.04215865	0.17802195	nonDEG
Bpev01.c0265.g0024.m0001	0.487298089	0.11439813	0.35099181	nonDEG
Bpev01.c0403.g0010.m0001	-0.26103533	0.42004974	0.75154031	nonDEG
Bpev01.c0022.g0011.m0001	-0.113948996	0.74536525	0.98482149	nonDEG
Bpev01.c0135.g0017.m0001	-0.803681031	0.04425794	0.18439139	nonDEG
Bpev01.c0142.g0016.m0001	0.861081354	0.08712186	0.29198321	nonDEG
Bpev01.c0022.g0007.m0001	-0.0462729	0.89825832	1	nonDEG
Bpev01.c1346.g0002.m0001	0.317466099	0.93974192	1	nonDEG
Bpev01.c0207.g0010.m0001	-0.252267925	0.6768851	0.93919124	nonDEG
Bpev01.c1876.g0006.m0001	0.012569832	0.97560731	1	nonDEG
Bpev01.c0931.g0002.m0001	0.716031388	0.02411386	0.1186112	nonDEG
Bpev01.c0574.g0018.m0001	-0.275072774	0.38283734	0.7175209	nonDEG
Bpev01.c0083.g0008.m0001	0.765627995	0.01594159	0.08749151	nonDEG
Bpev01.c0091.g0006.m0002	0.580366352	0.05985115	0.2269857	nonDEG
Bpev01.c1040.g0031.m0001	0.531144983	0.1104922	0.34228978	nonDEG
Bpev01.c1782.g0006.m0001	0.876882166	0.09330111	0.30559541	nonDEG
Bpev01.c0038.g0100.m0002	0.673112747	0.04686432	0.19121813	nonDEG
Bpev01.c0304.g0022.m0001	-0.164912872	0.63358695	0.92930926	nonDEG
Bpev01.c0090.g0009.m0001	-0.459741639	0.40302363	0.73631444	nonDEG
Bpev01.c0818.g0014.m0001	0.01123347	0.97274287	1	nonDEG
Bpev01.c0617.g0011.m0001	0.026498714	0.94105431	1	nonDEG
Bpev01.c0349.g0066.m0001	-0.719062519	0.03209173	0.1454578	nonDEG

Bpev01.c2165.g0004.m0001	-0.112525315	0.73211585	0.97718346	nonDEG
Bpev01.c0401.g0023.m0001	-1.050442736	0.07074406	0.2544002	nonDEG
Bpev01.c0051.g0032.m0001	-0.066409707	0.85541667	1	nonDEG
Bpev01.c0166.g0024.m0001	-0.061684394	0.8483317	1	nonDEG
Bpev01.c0320.g0001.m0001	-0.764090905	0.60600948	0.91034513	nonDEG
Bpev01.c0241.g0016.m0001	0.016605513	0.96107882	1	nonDEG
Bpev01.c0439.g0034.m0001	-0.563067067	0.11739014	0.35711981	nonDEG
Bpev01.c0558.g0015.m0001	0.585184346	0.28019925	0.60728746	nonDEG
Bpev01.c0243.g0057.m0001	0.065867533	0.83531902	1	nonDEG
Bpev01.c0015.g0045.m0001	0.419551436	0.22876688	0.54150263	nonDEG
Bpev01.c0265.g0020.m0001	0.348456772	0.24891035	0.56843315	nonDEG
Bpev01.c0649.g0008.m0001	0.626452422	0.11813739	0.35876281	nonDEG
Bpev01.c1115.g0003.m0001	0.47305679	0.12862635	0.38010623	nonDEG
Bpev01.c0253.g0020.m0001	0.542624287	0.12327632	0.37031956	nonDEG
Bpev01.c0700.g0002.m0001	-0.055828986	0.85868631	1	nonDEG
Bpev01.c2645.g0005.m0001	-0.008595571	0.97896043	1	nonDEG
Bpev01.c0038.g0041.m0001	-0.767301806	0.01492853	0.08329816	nonDEG
Bpev01.c0090.g0002.m0001	-0.546918971	0.14093859	0.40250585	nonDEG
Bpev01.c0127.g0056.m0001	-0.159053329	0.65796123	0.93220195	nonDEG
Bpev01.c0350.g0018.m0001	0.110530899	0.72926083	0.97576589	nonDEG
Bpev01.c2078.g0001.m0001	0.765155779	0.12534211	0.37377901	nonDEG
Bpev01.c0498.g0007.m0001	-0.197257326	0.53555792	0.85343129	nonDEG
Bpev01.c0051.g0160.m0001	-0.016634105	0.95596463	1	nonDEG
Bpev01.c0148.g0023.m0001	-0.016254098	0.96027684	1	nonDEG
Bpev01.c1136.g0004.m0001	0.422470267	0.18788118	0.48258012	nonDEG
Bpev01.c0989.g0008.m0001	0.07126555	0.83344745	1	nonDEG
Bpev01.c1242.g0001.m0001	0.663222256	0.03224648	0.14595696	nonDEG
Bpev01.c0572.g0004.m0001	-0.280854827	0.4275727	0.75964471	nonDEG
Bpev01.c0029.g0140.m0001	-0.579241026	0.08169997	0.28015904	nonDEG
Bpev01.c0299.g0033.m0001	-0.867443668	0.01060953	0.06463867	nonDEG
Bpev01.c0215.g0006.m0001	0.141108606	0.65170551	0.93220195	nonDEG
Bpev01.c0571.g0003.m0001	-0.434271394	0.16751059	0.44994017	nonDEG
Bpev01.c1000.g0017.m0001	0.26185548	0.68251726	0.94344864	nonDEG
Bpev01.c0091.g0008.m0001	0.447012619	0.29404571	0.62402044	nonDEG
Bpev01.c0457.g0020.m0001	0.653453389	0.0389094	0.16793736	nonDEG
Bpev01.c0032.g0001.m0001	0.543023571	0.34571115	0.6823594	nonDEG
Bpev01.c1114.g0009.m0001	-0.042803566	0.88809785	1	nonDEG
Bpev01.c0261.g0051.m0001	0.669206894	0.42791454	0.76002103	nonDEG
Bpev01.c0000.g0149.m0001	-0.95265283	0.01099748	0.06645213	nonDEG
Bpev01.c0024.g0017.m0001	-0.603294034	0.11824039	0.35896452	nonDEG
Bpev01.c1000.g0013.m0001	-0.593994014	0.19919474	0.49970974	nonDEG
Bpev01.c0214.g0016.m0001	0.191307641	0.76899974	0.99962836	nonDEG
Bpev01.c0088.g0082.m0001	0.305106423	0.91080102	1	nonDEG

Bpev01.c0027.g0015.m0001	-0.062880668	0.87892157	1	nonDEG
Bpev01.c0820.g0007.m0001	-0.780025167	0.02438607	0.11961038	nonDEG
Bpev01.c2293.g0001.m0001	-0.306558558	0.48669249	0.81356767	nonDEG
Bpev01.c2541.g0001.m0001	-1.280999765	0.00871314	0.05564127	nonDEG
Bpev01.c0275.g0033.m0001	-0.041924933	0.88979965	1	nonDEG
Bpev01.c0901.g0010.m0001	-0.197882733	0.85090799	1	nonDEG
Bpev01.c1078.g0003.m0001	0.894189358	0.238189	0.55492609	nonDEG
Bpev01.c0582.g0021.m0001	0.453055877	0.19476316	0.4932157	nonDEG
Bpev01.c0259.g0007.m0001	-0.63435223	0.48537991	0.81262661	nonDEG
Bpev01.c1828.g0005.m0001	0.599592427	0.05400462	0.21138703	nonDEG
Bpev01.c1856.g0003.m0001	-0.608919913	0.56573233	0.87882403	nonDEG
Bpev01.c0023.g0011.m0001	0.655640015	0.14662823	0.41314973	nonDEG
Bpev01.c0170.g0052.m0001	-0.198100259	0.50750567	0.82885967	nonDEG
Bpev01.c0015.g0018.m0001	0.458607912	0.40690121	0.74025242	nonDEG
Bpev01.c0129.g0030.m0002	0.083253293	0.83226889	1	nonDEG
Bpev01.c1855.g0001.m0001	0.100799686	0.92151067	1	nonDEG
Bpev01.c1951.g0001.m0001	0.043340675	0.92442429	1	nonDEG
Bpev01.c1070.g0026.m0001	0.571370397	0.08990534	0.29839046	nonDEG
Bpev01.c1627.g0006.m0001	-2.723038641	0.02126506	0.10780476	nonDEG
Bpev01.c1088.g0002.m0001	0.847709477	0.24540713	0.5636686	nonDEG
Bpev01.c1426.g0009.m0001	0.405209759	0.25757344	0.57750987	nonDEG
Bpev01.c0263.g0002.m0001	0.815661131	0.02956527	0.13726098	nonDEG
Bpev01.c2307.g0001.m0001	0.097849653	0.76170279	0.99507876	nonDEG
Bpev01.c0001.g0002.m0002	-0.988521764	0.00881219	0.05616697	nonDEG
Bpev01.c0796.g0007.m0001	-0.816446217	0.25223849	0.57314362	nonDEG
Bpev01.c0123.g0007.m0001	-0.153195552	0.62466226	0.9238115	nonDEG
Bpev01.c0396.g0014.m0001	0.578984836	0.43529321	0.76749977	nonDEG
Bpev01.c0847.g0010.m0001	-0.504666429	0.09501708	0.30945987	nonDEG
Bpev01.c0029.g0088.m0001	-0.04410951	0.89723475	1	nonDEG
Bpev01.c0357.g0087.m0001	2.295929848	0.01207067	0.07109812	nonDEG
Bpev01.c0287.g0010.m0001	1.255761681	0.0647454	0.23948231	nonDEG
Bpev01.c0598.g0020.m0001	-0.474822044	0.16763893	0.4501619	nonDEG
Bpev01.c1419.g0004.m0001	0.168608411	0.59859799	0.90546328	nonDEG
Bpev01.c1371.g0006.m0001	-0.553151863	0.07643104	0.26808691	nonDEG
Bpev01.c0052.g0150.m0001	-0.219663191	0.56941952	0.88231704	nonDEG
Bpev01.c1657.g0006.m0001	0.77397147	0.0305003	0.14029663	nonDEG
Bpev01.c0088.g0070.m0001	0.266089695	0.61375131	0.91642447	nonDEG
Bpev01.c1639.g0005.m0001	-0.728102985	0.20271951	0.50455357	nonDEG
Bpev01.c0517.g0007.m0001	1.141116144	0.04946601	0.19874845	nonDEG
Bpev01.c1520.g0011.m0002	0.324555533	0.41631331	0.74822626	nonDEG
Bpev01.c0154.g0031.m0001	-1.10099223	0.0491201	0.1977635	nonDEG
Bpev01.c0337.g0009.m0001	0.093847746	0.78999481	1	nonDEG
Bpev01.c0190.g0004.m0001	0.373962762	0.28457357	0.61176875	nonDEG

Bpev01.c0324.g0018.m0001	0.48750626	0.12784553	0.37852203	nonDEG
Bpev01.c1868.g0004.m0001	1.24373045	0.03968157	0.17032684	nonDEG
Bpev01.c0344.g0017.m0001	-0.843282383	0.01131069	0.06776059	nonDEG
Bpev01.c0105.g0016.m0001	-2.374091313	0.15330699	0.42509225	nonDEG
Bpev01.c0214.g0028.m0001	-0.4099535	0.25249636	0.57356989	nonDEG
Bpev01.c0018.g0034.m0001	0.050168369	0.87491778	1	nonDEG
Bpev01.c0847.g0001.m0001	0.03610677	0.94775125	1	nonDEG
Bpev01.c0155.g0011.m0001	-0.04383068	0.88979871	1	nonDEG
Bpev01.c0389.g0035.m0001	0.313165096	0.34648417	0.68305046	nonDEG
Bpev01.c0444.g0002.m0001	-0.110855264	0.71514971	0.9672496	nonDEG
Bpev01.c0652.g0022.m0001	0.614140575	0.09683348	0.31329605	nonDEG
Bpev01.c0522.g0003.m0001	-0.725089899	0.04934756	0.19838081	nonDEG
Bpev01.c1818.g0009.m0001	0.13463556	0.65468111	0.93220195	nonDEG
Bpev01.c0018.g0054.m0001	0.220064697	0.51288829	0.83340737	nonDEG
Bpev01.c0130.g0011.m0001	0.288069826	0.45889103	0.78789764	nonDEG
Bpev01.c0101.g0050.m0002	-0.513247574	0.242413	0.5601147	nonDEG
Bpev01.c1952.g0006.m0001	0.388159566	0.28012861	0.60717908	nonDEG
Bpev01.c1087.g0007.m0001	-0.14291719	0.88278148	1	nonDEG
Bpev01.c1154.g0007.m0001	-0.552964811	0.39086476	0.72361573	nonDEG
Bpev01.c0230.g0031.m0001	0.600036349	0.52366763	0.84320142	nonDEG
Bpev01.c1324.g0003.m0001	-0.263648972	0.40457999	0.73771634	nonDEG
Bpev01.c0313.g0004.m0001	-3.078753042	0.13171038	0.38549465	nonDEG
Bpev01.c0329.g0008.m0001	0.50936876	0.10450171	0.33015945	nonDEG
Bpev01.c0022.g0041.m0001	0.732615225	0.04432558	0.18459483	nonDEG
Bpev01.c0408.g0002.m0001	-0.137242707	0.91922537	1	nonDEG
Bpev01.c0328.g0008.m0001	0.736224499	0.02704326	0.12871051	nonDEG
Bpev01.c3277.g0001.m0001	0.62815919	0.2013324	0.50239138	nonDEG
Bpev01.c0253.g0004.m0001	0.51069067	0.09927496	0.31891703	nonDEG
Bpev01.c0253.g0032.m0001	-1.280345708	0.01890128	0.09886899	nonDEG
Bpev01.c0132.g0003.m0001	1.500663515	0.03269905	0.14739356	nonDEG
Bpev01.c0177.g0015.m0001	-0.108362638	0.73809693	0.98116298	nonDEG
Bpev01.c0055.g0010.m0001	-0.133201105	0.65548394	0.93220195	nonDEG
Bpev01.c0206.g0001.m0001	0.083578188	0.82227155	1	nonDEG
Bpev01.c1557.g0002.m0001	0.24509135	0.85666716	1	nonDEG
Bpev01.c2054.g0003.m0001	-0.525989749	0.08666906	0.2908962	nonDEG
Bpev01.c1070.g0010.m0001	1.750276319	0.01757678	0.09377363	nonDEG
Bpev01.c0364.g0005.m0001	2.044752686	0.03426738	0.15266305	nonDEG
Bpev01.c3728.g0002.m0001	2.814812047	0.01114036	0.06711754	nonDEG
Bpev01.c0560.g0004.m0001	-4.604364042	0.25548777	0.57585256	nonDEG
Bpev01.c0166.g0031.m0001	0.21480871	0.47715616	0.80572446	nonDEG
Bpev01.c0821.g0002.m0001	-0.778492538	0.01005823	0.06207636	nonDEG
Bpev01.c0089.g0077.m0001	-2.022988432	0.4085308	0.74234116	nonDEG
Bpev01.c0127.g0022.m0001	-0.140522467	0.78611564	1	nonDEG

Bpev01.c0015.g0210.m0001	0.481158922	0.19486038	0.4933346	nonDEG
Bpev01.c0044.g0061.m0001	0.41752418	0.23304143	0.54805135	nonDEG
Bpev01.c0394.g0020.m0001	0.756806427	0.02028352	0.10388421	nonDEG
Bpev01.c0136.g0005.m0001	0.251512855	0.42286422	0.75447915	nonDEG
Bpev01.c0442.g0017.m0001	0.961140489	0.28807049	0.61644131	nonDEG
Bpev01.c2298.g0001.m0002	0.416474796	0.52640966	0.84567388	nonDEG
Bpev01.c0383.g0022.m0001	0.02300248	0.94159855	1	nonDEG
Bpev01.c1586.g0006.m0001	0.082110899	0.85403125	1	nonDEG
Bpev01.c2874.g0001.m0001	-0.408894266	0.17293425	0.4600173	nonDEG
Bpev01.c0052.g0165.m0001	-0.327883897	0.27193333	0.59774842	nonDEG
Bpev01.c0051.g0075.m0001	0.478662599	0.24367563	0.56152677	nonDEG
Bpev01.c0654.g0005.m0001	0.131346288	0.76463299	0.99687634	nonDEG
Bpev01.c0205.g0002.m0001	0.452900723	0.27032036	0.59570036	nonDEG
Bpev01.c0366.g0011.m0001	0.225082484	0.75455168	0.99053132	nonDEG
Bpev01.c1879.g0001.m0001	0.92177344	0.15466534	0.42739203	nonDEG
Bpev01.c1280.g0007.m0001	0.0726681	0.8273341	1	nonDEG
Bpev01.c0312.g0002.m0001	-0.82920041	0.00921098	0.05810203	nonDEG
Bpev01.c0171.g0016.m0001	-0.740014554	0.05092059	0.20298804	nonDEG
Bpev01.c0139.g0003.m0001	-0.388793617	0.25827998	0.57852122	nonDEG
Bpev01.c0261.g0032.m0001	0.32196402	0.2907508	0.61993284	nonDEG
Bpev01.c1060.g0023.m0001	-0.364365485	0.24740613	0.56652461	nonDEG
Bpev01.c0668.g0019.m0001	0.301977809	0.38855289	0.72130199	nonDEG
Bpev01.c0050.g0035.m0001	-0.227990673	0.6350895	0.93006545	nonDEG
Bpev01.c0052.g0130.m0001	-0.601927628	0.20919354	0.51414045	nonDEG
Bpev01.c0350.g0022.m0002	-0.414882953	0.24312529	0.56089085	nonDEG
Bpev01.c1602.g0013.m0001	0.640839611	0.11326624	0.34860898	nonDEG
Bpev01.c0136.g0019.m0001	-0.795816744	0.01150009	0.06856039	nonDEG
Bpev01.c0042.g0066.m0001	-0.322516064	0.6020119	0.90764254	nonDEG
Bpev01.c0027.g0167.m0001	0.622522076	0.18376813	0.47673159	nonDEG
Bpev01.c0331.g0008.m0001	-0.176067343	0.68209761	0.94311442	nonDEG
Bpev01.c2115.g0003.m0001	-0.631406049	0.04153061	0.17600656	nonDEG
Bpev01.c0407.g0013.m0001	-0.814645184	0.00903839	0.05729567	nonDEG
Bpev01.c1610.g0005.m0001	-0.590025415	0.12627023	0.37541457	nonDEG
Bpev01.c0120.g0065.m0001	0.450141156	0.34017417	0.67634129	nonDEG
Bpev01.c0798.g0004.m0001	0.910993776	0.1795723	0.46993012	nonDEG
Bpev01.c1164.g0001.m0001	5.311190939	0.08382184	0.28470977	nonDEG
Bpev01.c1758.g0002.m0001	-0.388039716	0.36051613	0.69892883	nonDEG
Bpev01.c0134.g0145.m0001	0.062190893	0.85218477	1	nonDEG
Bpev01.c0050.g0076.m0001	0.73815411	0.06164693	0.23157594	nonDEG
Bpev01.c0149.g0015.m0001	-0.173748914	0.59683193	0.90460461	nonDEG
Bpev01.c0245.g0083.m0001	0.914886559	0.00909836	0.05756431	nonDEG
Bpev01.c0088.g0109.m0001	0.271322378	0.38412394	0.71839303	nonDEG
Bpev01.c0365.g0006.m0001	1.082047406	0.08073446	0.27820925	nonDEG

Bpev01.c1481.g0008.m0001	0.130579289	0.67741546	0.9397679	nonDEG
Bpev01.c0167.g0006.m0001	0.296191981	0.46408724	0.79288865	nonDEG
Bpev01.c0588.g0008.m0001	0.524449975	0.1018886	0.32455079	nonDEG
Bpev01.c0051.g0170.m0001	-0.589989577	0.06080396	0.22939996	nonDEG
Bpev01.c0120.g0049.m0001	0.315507317	0.52771721	0.84680404	nonDEG
Bpev01.c0996.g0012.m0001	0.172505505	0.57764126	0.88892739	nonDEG
Bpev01.c0651.g0010.m0002	0.834714024	0.00981006	0.06091508	nonDEG
Bpev01.c0870.g0016.m0001	0.343398483	0.28964078	0.61855168	nonDEG
Bpev01.c0763.g0002.m0001	0.449549772	0.13404611	0.38982221	nonDEG
Bpev01.c0427.g0016.m0001	-0.700800608	0.0687493	0.2497856	nonDEG
Bpev01.c0000.g0026.m0001	-0.709377575	0.05192265	0.2058377	nonDEG
Bpev01.c0483.g0018.m0001	0.286890729	0.34419307	0.68054939	nonDEG
Bpev01.c0161.g0063.m0001	-0.277587246	0.71558459	0.96748851	nonDEG
Bpev01.c0312.g0023.m0001	0.495103075	0.17653325	0.46487724	nonDEG
Bpev01.c1076.g0001.m0001	1.061965964	0.01057053	0.06442771	nonDEG
Bpev01.c0423.g0006.m0001	-0.816376037	0.02672025	0.12759405	nonDEG
Bpev01.c0051.g0006.m0001	0.265124775	0.4174891	0.74905301	nonDEG
Bpev01.c0052.g0063.m0002	0.519920254	0.4205936	0.75205851	nonDEG
Bpev01.c1338.g0004.m0001	0.828710084	0.01083178	0.06569362	nonDEG
Bpev01.c2295.g0006.m0001	-0.187653829	0.54956799	0.86549682	nonDEG
Bpev01.c0016.g0051.m0001	0.491264277	0.1279836	0.37881653	nonDEG
Bpev01.c0025.g0031.m0001	-0.120383162	0.69532589	0.95300987	nonDEG
Bpev01.c0663.g0004.m0001	-0.851555819	0.01019542	0.06269957	nonDEG
Bpev01.c0395.g0043.m0001	-0.545299712	0.10325228	0.3274391	nonDEG
Bpev01.c0571.g0018.m0001	-0.854206206	0.0125778	0.07342413	nonDEG
Bpev01.c0080.g0086.m0001	-0.143505349	0.74706457	0.9857402	nonDEG
Bpev01.c1269.g0007.m0001	-0.458636344	0.20401252	0.5062954	nonDEG
Bpev01.c0791.g0002.m0001	-1.139427672	0.3579039	0.69615678	nonDEG
Bpev01.c0114.g0002.m0001	0.957892431	0.67467609	0.93789439	nonDEG
Bpev01.c0091.g0048.m0001	-0.0526838	0.91966337	1	nonDEG
Bpev01.c0488.g0014.m0002	0.2014218	0.51885407	0.83876563	nonDEG
Bpev01.c0134.g0015.m0001	-0.098263232	0.76639988	0.99827645	nonDEG
Bpev01.c0845.g0001.m0001	-0.384927704	0.45532422	0.78525424	nonDEG
Bpev01.c0016.g0172.m0001	0.154378478	0.62695	0.92482743	nonDEG
Bpev01.c0892.g0027.m0001	-0.866532993	0.01037612	0.06359829	nonDEG
Bpev01.c0038.g0145.m0001	-0.165157511	0.68884339	0.94792037	nonDEG
Bpev01.c0052.g0035.m0001	0.312874892	0.64731428	0.93220195	nonDEG
Bpev01.c0748.g0006.m0001	-0.000975641	0.99824903	1	nonDEG
Bpev01.c0597.g0010.m0004	-0.529390078	0.1829012	0.47511061	nonDEG
Bpev01.c0665.g0007.m0001	0.279386721	0.34810229	0.68524128	nonDEG
Bpev01.c0154.g0005.m0001	1.615421291	0.01294707	0.07496474	nonDEG
Bpev01.c0774.g0001.m0001	-0.511709444	0.36133689	0.6995008	nonDEG
Bpev01.c1143.g0008.m0001	-0.251301113	0.43941583	0.77148474	nonDEG

Bpev01.c1427.g0002.m0001	-0.320023707	0.31434351	0.64618104	nonDEG
Bpev01.c1891.g0009.m0001	-1.242109742	0.01865895	0.09780683	nonDEG
Bpev01.c0114.g0004.m0001	1.651064093	0.01801775	0.09543443	nonDEG
Bpev01.c0101.g0052.m0001	-0.065954466	0.87830517	1	nonDEG
Bpev01.c0261.g0033.m0001	0.360044252	0.25549852	0.57585256	nonDEG
Bpev01.c3045.g0003.m0001	-0.372414449	0.31123349	0.64183688	nonDEG
Bpev01.c1239.g0011.m0001	-0.47568291	0.1246469	0.37231101	nonDEG
Bpev01.c1475.g0007.m0001	-0.506192054	0.12926823	0.3811025	nonDEG
Bpev01.c0930.g0004.m0001	0.081797964	0.7882021	1	nonDEG
Bpev01.c0215.g0014.m0001	-0.4886908	0.12979267	0.38204778	nonDEG
Bpev01.c0129.g0043.m0001	0.072590376	0.8140069	1	nonDEG
Bpev01.c1560.g0004.m0001	0.716006744	0.07243217	0.25855333	nonDEG
Bpev01.c1074.g0006.m0001	1.591449448	0.03735929	0.16291217	nonDEG
Bpev01.c0134.g0142.m0001	0.671911324	0.03372559	0.1509805	nonDEG
Bpev01.c0189.g0058.m0001	-0.227815948	0.45476076	0.78471082	nonDEG
Bpev01.c0778.g0004.m0001	-0.099783547	0.79949971	1	nonDEG
Bpev01.c0931.g0020.m0001	0.033526118	1	1	nonDEG
Bpev01.c0398.g0015.m0001	0.511833472	0.21940797	0.52770843	nonDEG
Bpev01.c0004.g0008.m0001	0.486067394	0.20448073	0.50699651	nonDEG
Bpev01.c1122.g0002.m0001	1.541338136	0.24208627	0.55962133	nonDEG
Bpev01.c0103.g0001.m0001	1.926247155	0.01678292	0.09075539	nonDEG
Bpev01.c1591.g0004.m0001	0.25586782	0.42702504	0.75917569	nonDEG
Bpev01.c1064.g0008.m0001	0.664931693	0.02675066	0.12763433	nonDEG
Bpev01.c1238.g0011.m0001	0.635532892	0.10922391	0.33957575	nonDEG
Bpev01.c1899.g0002.m0001	-0.482663578	0.17656569	0.46487724	nonDEG
Bpev01.c0000.g0091.m0001	-0.574697681	0.08050847	0.27759297	nonDEG
Bpev01.c1013.g0003.m0001	-0.620556115	0.03951379	0.16978271	nonDEG
Bpev01.c2401.g0001.m0001	-0.031549049	0.92074241	1	nonDEG
Bpev01.c0223.g0058.m0001	0.927420463	0.07942003	0.27493489	nonDEG
Bpev01.c0015.g0260.m0001	0.004577818	0.99080474	1	nonDEG
Bpev01.c0088.g0073.m0001	0.611211796	0.59087045	0.90014905	nonDEG
Bpev01.c0091.g0001.m0001	-0.593755383	0.10470891	0.33063665	nonDEG
Bpev01.c0842.g0011.m0001	0.452385891	0.2598915	0.58129037	nonDEG
Bpev01.c0349.g0006.m0001	0.476429392	0.12096582	0.36535476	nonDEG
Bpev01.c1845.g0003.m0001	-1.307510183	0.25109049	0.57128472	nonDEG
Bpev01.c0918.g0019.m0003	-0.454014042	0.28947355	0.61834965	nonDEG
Bpev01.c0190.g0048.m0001	-0.000427681	1	1	nonDEG
Bpev01.c0195.g0017.m0001	-0.114685342	0.70370687	0.95918263	nonDEG
Bpev01.c0652.g0018.m0001	0.083990944	0.80898469	1	nonDEG
Bpev01.c0052.g0212.m0001	-0.579289444	0.19172453	0.48808185	nonDEG
Bpev01.c1564.g0002.m0001	-0.225855772	0.92782304	1	nonDEG
Bpev01.c0189.g0014.m0001	-0.719964808	0.04446871	0.18490753	nonDEG
Bpev01.c0418.g0001.m0002	-0.429185763	0.28598849	0.61341512	nonDEG

Bpev01.c0671.g0018.m0001	-0.462924134	0.36137958	0.6995008	nonDEG
Bpev01.c0101.g0031.m0001	-0.272492304	0.39434017	0.72765328	nonDEG
Bpev01.c0299.g0022.m0001	0.020545051	0.95242531	1	nonDEG
Bpev01.c0092.g0017.m0001	0.206327698	0.53486104	0.85287479	nonDEG
Bpev01.c0096.g0023.m0001	0.616104387	0.22281448	0.53237063	nonDEG
Bpev01.c0022.g0099.m0001	0.107005395	0.72924447	0.97576589	nonDEG
Bpev01.c0050.g0100.m0001	-0.065081469	1	1	nonDEG
Bpev01.c0245.g0064.m0001	0.097846852	0.92985224	1	nonDEG
Bpev01.c0094.g0046.m0001	0.834725655	0.01544156	0.08533584	nonDEG
Bpev01.c0960.g0005.m0001	-1.54043504	0.35747357	0.6955955	nonDEG
Bpev01.c0275.g0006.m0001	0.192553654	0.6349317	0.93006545	nonDEG
Bpev01.c0274.g0036.m0001	-0.223924584	0.48938621	0.81450931	nonDEG
Bpev01.c0558.g0006.m0001	0.173921021	0.6385265	0.93020764	nonDEG
Bpev01.c1179.g0011.m0001	0.900996902	0.05874849	0.22385411	nonDEG
Bpev01.c0038.g0162.m0001	-1.157635344	0.01854329	0.09746346	nonDEG
Bpev01.c0523.g0011.m0001	-0.065635017	0.83970866	1	nonDEG
Bpev01.c0038.g0047.m0001	-0.639902616	0.35312889	0.6912254	nonDEG
Bpev01.c1477.g0002.m0001	0.005294841	0.98852626	1	nonDEG
Bpev01.c0925.g0002.m0001	-0.323686376	0.41996893	0.75148728	nonDEG
Bpev01.c0327.g0010.m0001	-0.26811824	0.44861482	0.78021325	nonDEG
Bpev01.c0996.g0026.m0001	0.106587367	0.74283209	0.98399997	nonDEG
Bpev01.c0613.g0012.m0001	1.10448513	0.31854265	0.65146197	nonDEG
Bpev01.c0473.g0030.m0001	-0.408613382	0.47501036	0.80371457	nonDEG
Bpev01.c0473.g0021.m0001	0.117122208	0.72560332	0.9737598	nonDEG
Bpev01.c0657.g0010.m0001	0.128455181	0.75419944	0.99024515	nonDEG
Bpev01.c0043.g0013.m0002	-0.140930488	0.66376626	0.93220195	nonDEG
Bpev01.c2165.g0005.m0001	0.517769889	0.09254345	0.30404474	nonDEG
Bpev01.c0129.g0097.m0001	0.536909949	0.0861167	0.28971243	nonDEG
Bpev01.c0161.g0079.m0001	-0.036421506	0.90861916	1	nonDEG
Bpev01.c1650.g0003.m0001	0.367197219	0.5017338	0.82420296	nonDEG
Bpev01.c0281.g0038.m0001	-1.001909336	0.01129177	0.06770231	nonDEG
Bpev01.c1155.g0003.m0001	-2.292304475	1	1	nonDEG
Bpev01.c0044.g0008.m0001	1.091045158	0.03981846	0.17073456	nonDEG
Bpev01.c0089.g0031.m0001	1.678810403	0.32153371	0.65489692	nonDEG
Bpev01.c1949.g0003.m0001	-0.673572521	0.22445791	0.53459904	nonDEG
Bpev01.c0088.g0041.m0001	0.270990403	0.69759985	0.95454544	nonDEG
Bpev01.c0401.g0014.m0001	0.554514883	0.20842629	0.5130319	nonDEG
Bpev01.c0170.g0030.m0001	-0.438689617	0.18122874	0.47272812	nonDEG
Bpev01.c0038.g0131.m0001	-0.338258678	0.86209874	1	nonDEG
Bpev01.c1333.g0008.m0001	-0.026780363	0.93192291	1	nonDEG
Bpev01.c1020.g0005.m0001	0.474726409	0.23826514	0.55501144	nonDEG
Bpev01.c0088.g0094.m0001	0.478506823	0.18966877	0.48494519	nonDEG
Bpev01.c0320.g0006.m0002	0.368774215	0.28803665	0.61642273	nonDEG

Bpev01.c1600.g0024.m0001	-0.003578812	1	1	nonDEG
Bpev01.c0384.g0011.m0002	-0.114144113	0.78880651	1	nonDEG
Bpev01.c1649.g0005.m0001	0.669150728	0.1317283	0.38549465	nonDEG
Bpev01.c1223.g0003.m0001	-0.648260805	0.03726594	0.16262556	nonDEG
Bpev01.c0886.g0003.m0001	0.637369881	0.14159847	0.40396524	nonDEG
Bpev01.c0036.g0022.m0001	0.282356425	0.39984362	0.73289312	nonDEG
Bpev01.c0038.g0106.m0001	-0.801399029	0.01173958	0.06946765	nonDEG
Bpev01.c0693.g0001.m0001	-0.338853306	0.2651886	0.5886756	nonDEG
Bpev01.c0609.g0013.m0001	1.083738358	0.23468759	0.55073479	nonDEG
Bpev01.c0000.g0036.m0001	0.40776996	0.46025764	0.7890797	nonDEG
Bpev01.c0974.g0019.m0001	-0.079649838	0.79598726	1	nonDEG
Bpev01.c0189.g0003.m0001	1.233019707	0.01104427	0.06670749	nonDEG
Bpev01.c0002.g0004.m0001	-4.362441658	0.20038605	0.50114495	nonDEG
Bpev01.c0052.g0134.m0001	0.662267294	0.03666966	0.16059478	nonDEG
Bpev01.c0264.g0007.m0001	0.877127005	0.05302407	0.20893043	nonDEG
Bpev01.c0044.g0064.m0001	0.236610225	0.45743496	0.78677423	nonDEG
Bpev01.c0237.g0068.m0001	0.538119733	0.1590887	0.43599072	nonDEG
Bpev01.c0148.g0018.m0001	-0.769260589	0.01788505	0.09488519	nonDEG
Bpev01.c2206.g0003.m0001	0.706391335	0.24522318	0.56346592	nonDEG
Bpev01.c1479.g0003.m0001	0.1476323	0.69423061	0.95199622	nonDEG
Bpev01.c2104.g0002.m0001	0.224501486	0.5746371	0.88629652	nonDEG
Bpev01.c0337.g0027.m0001	-0.534411614	0.27171238	0.59756019	nonDEG
Bpev01.c0094.g0073.m0001	0.471170629	0.36018467	0.69847023	nonDEG
Bpev01.c0000.g0088.m0001	0.324549865	0.31770182	0.65045023	nonDEG
Bpev01.c0261.g0057.m0002	0.229941492	0.51500667	0.83523262	nonDEG
Bpev01.c0906.g0022.m0001	-0.305213631	0.34742658	0.68427719	nonDEG
Bpev01.c2581.g0006.m0001	-0.080787206	0.79336579	1	nonDEG
Bpev01.c0480.g0068.m0001	0.35915551	0.29521654	0.62524917	nonDEG
Bpev01.c0000.g0087.m0001	-0.085774329	0.8048636	1	nonDEG
Bpev01.c0582.g0023.m0001	-0.318278322	0.29101043	0.62012711	nonDEG
Bpev01.c0051.g0157.m0001	0.57327505	0.66319501	0.93220195	nonDEG
Bpev01.c0751.g0011.m0001	-0.499724313	0.12628433	0.37541457	nonDEG
Bpev01.c0616.g0005.m0001	-0.513082574	0.19542172	0.49433063	nonDEG
Bpev01.c0783.g0001.m0001	1.316018522	0.1693051	0.45323023	nonDEG
Bpev01.c0241.g0019.m0001	1.032116048	0.01137589	0.06802656	nonDEG
Bpev01.c0044.g0077.m0001	-0.129828801	0.82956378	1	nonDEG
Bpev01.c0094.g0066.m0001	0.484082744	0.24822532	0.56736253	nonDEG
Bpev01.c0329.g0016.m0001	2.787030308	0.04952406	0.19890023	nonDEG
Bpev01.c0018.g0001.m0001	-0.584290644	0.18194581	0.47375754	nonDEG
Bpev01.c0616.g0016.m0002	0.609572736	0.05530106	0.21487058	nonDEG
Bpev01.c0038.g0031.m0001	0.576851297	0.09612047	0.31165305	nonDEG
Bpev01.c0891.g0010.m0001	-0.497743927	0.10064161	0.32182392	nonDEG
Bpev01.c0566.g0057.m0001	0.538295177	0.11515068	0.35267592	nonDEG

Bpev01.c1114.g0004.m0001	-0.926079819	0.00878511	0.05603701	nonDEG
Bpev01.c0452.g0015.m0001	1.77910454	0.08484768	0.28690914	nonDEG
Bpev01.c0327.g0014.m0001	-0.108297743	0.73980496	0.98245242	nonDEG
Bpev01.c0349.g0032.m0001	-0.740784674	0.32003227	0.65335853	nonDEG
Bpev01.c0331.g0011.m0001	-0.690484321	0.03252798	0.14684727	nonDEG
Bpev01.c0518.g0048.m0001	0.331112193	0.45443587	0.78440427	nonDEG
Bpev01.c1382.g0013.m0001	1.853336168	0.05151725	0.20458875	nonDEG
Bpev01.c0350.g0005.m0001	0.028473892	0.92881713	1	nonDEG
Bpev01.c0660.g0003.m0001	-0.607846456	0.46843937	0.79688125	nonDEG
Bpev01.c0652.g0025.m0001	0.117387892	0.70330588	0.95893076	nonDEG
Bpev01.c0043.g0027.m0001	0.031177575	0.91823952	1	nonDEG
Bpev01.c0471.g0001.m0001	-0.195557881	0.71999909	0.97015264	nonDEG
Bpev01.c0480.g0088.m0001	0.041334316	0.89687834	1	nonDEG
Bpev01.c0522.g0014.m0001	0.191665945	0.54654529	0.86285981	nonDEG
Bpev01.c1999.g0004.m0001	-1.820913351	0.27143588	0.59704131	nonDEG
Bpev01.c0649.g0018.m0001	0.737564914	0.07203113	0.25762659	nonDEG
Bpev01.c1130.g0006.m0001	-0.43171693	0.16669321	0.44860421	nonDEG
Bpev01.c0374.g0012.m0001	-0.012740457	1	1	nonDEG
Bpev01.c0890.g0006.m0001	0.575996364	0.05984277	0.2269857	nonDEG
Bpev01.c0919.g0007.m0001	0.150904481	1	1	nonDEG
Bpev01.c1391.g0007.m0001	0.016506387	0.96136975	1	nonDEG
Bpev01.c0118.g0031.m0001	-0.534829672	0.19844154	0.4984999	nonDEG
Bpev01.c0632.g0002.m0001	0.725517796	0.11302908	0.348258	nonDEG
Bpev01.c1136.g0005.m0001	-0.355430125	0.24368468	0.56152677	nonDEG
Bpev01.c0368.g0007.m0001	0.169106755	0.60003118	0.90642025	nonDEG
Bpev01.c0553.g0006.m0001	0.946577189	0.12448604	0.37220644	nonDEG
Bpev01.c3442.g0001.m0001	1.212488811	0.15432382	0.42674609	nonDEG
Bpev01.c1350.g0005.m0001	0.61274419	0.12779956	0.37850013	nonDEG
Bpev01.c0566.g0073.m0001	-0.421702462	0.67908235	0.94082099	nonDEG
Bpev01.c0914.g0001.m0001	0.524434467	1	1	nonDEG
Bpev01.c0796.g0016.m0001	-0.746855669	0.02655779	0.12713534	nonDEG
Bpev01.c0051.g0003.m0001	-1.902476951	0.07452716	0.26343404	nonDEG
Bpev01.c2497.g0003.m0002	-0.236918425	0.49275325	0.81673271	nonDEG
Bpev01.c0036.g0006.m0001	0.210991011	0.49744101	0.82044741	nonDEG
Bpev01.c1545.g0001.m0001	-0.655015	0.11367252	0.34967663	nonDEG
Bpev01.c0915.g0011.m0001	0.799239236	0.03645645	0.15985128	nonDEG
Bpev01.c0398.g0014.m0001	-0.231491539	0.44986938	0.78124733	nonDEG
Bpev01.c0161.g0100.m0001	1.045421507	0.23369192	0.5490986	nonDEG
Bpev01.c0240.g0031.m0002	-0.431752628	0.22916418	0.54209436	nonDEG
Bpev01.c0050.g0024.m0001	0.318312613	0.35554461	0.69344626	nonDEG
Bpev01.c0096.g0005.m0001	-0.827146626	0.02415028	0.11873082	nonDEG
Bpev01.c0947.g0004.m0001	0.636139715	0.34584638	0.68239121	nonDEG
Bpev01.c1202.g0011.m0002	0.255189043	0.41996905	0.75148728	nonDEG

Bpev01.c0038.g0133.m0001	-0.050748767	0.87020909	1	nonDEG
Bpev01.c0039.g0018.m0001	-0.25151639	0.64244901	0.93220195	nonDEG
Bpev01.c0803.g0005.m0001	0.080613346	0.79550727	1	nonDEG
Bpev01.c0719.g0020.m0001	0.072486736	1	1	nonDEG
Bpev01.c0597.g0030.m0001	-0.697848394	0.77721131	1	nonDEG
Bpev01.c0862.g0001.m0001	0.155830901	0.66134748	0.93220195	nonDEG
Bpev01.c0426.g0006.m0002	0.067793554	0.82793849	1	nonDEG
Bpev01.c0042.g0036.m0001	-0.230418697	0.48358875	0.81243903	nonDEG
Bpev01.c0088.g0064.m0001	-0.009876845	0.97987016	1	nonDEG
Bpev01.c0328.g0034.m0001	-0.60539986	0.1005184	0.32163268	nonDEG
Bpev01.c0016.g0063.m0001	-0.157185363	0.62587808	0.92449698	nonDEG
Bpev01.c0015.g0112.m0001	-0.445946332	0.17402858	0.4616352	nonDEG
Bpev01.c1159.g0022.m0001	-0.41591534	0.19244924	0.48912313	nonDEG
Bpev01.c0016.g0160.m0001	-0.43952451	0.16803856	0.45090577	nonDEG
Bpev01.c0118.g0044.m0001	0.809900324	0.01623283	0.08864369	nonDEG
Bpev01.c1649.g0009.m0001	-0.068842276	0.83064574	1	nonDEG
Bpev01.c0301.g0036.m0001	-0.507593329	0.19064179	0.48637696	nonDEG
Bpev01.c0588.g0037.m0001	0.051614074	0.9045697	1	nonDEG
Bpev01.c0389.g0046.m0001	0.270768013	0.53270482	0.85113807	nonDEG
Bpev01.c0540.g0008.m0002	-1.065830344	0.01439579	0.08098559	nonDEG
Bpev01.c0120.g0018.m0002	-0.586290945	0.12173123	0.36683854	nonDEG
Bpev01.c0915.g0008.m0001	-0.573164576	0.05895703	0.22439156	nonDEG
Bpev01.c0161.g0103.m0001	0.637329066	0.4510231	0.78253483	nonDEG
Bpev01.c0167.g0022.m0001	-0.842566308	0.00907091	0.05745235	nonDEG
Bpev01.c0051.g0193.m0001	0.102534694	0.731695	0.97692159	nonDEG
Bpev01.c1520.g0006.m0001	-1.972094149	0.03532944	0.15621258	nonDEG
Bpev01.c0820.g0013.m0001	-0.055480664	0.87201582	1	nonDEG
Bpev01.c0751.g0002.m0001	-0.304951688	0.5957377	0.90353904	nonDEG
Bpev01.c0350.g0015.m0001	-0.507416889	0.58978664	0.89934367	nonDEG
Bpev01.c0045.g0002.m0001	-0.273106013	0.39220157	0.72521764	nonDEG
Bpev01.c0318.g0004.m0001	0.523595189	0.13655867	0.39479041	nonDEG
Bpev01.c1539.g0002.m0001	-0.287483202	0.43778101	0.76990532	nonDEG
Bpev01.c0083.g0025.m0001	-0.136711196	0.68981379	0.94876791	nonDEG
Bpev01.c3512.g0001.m0001	-0.797275641	0.01083764	0.06570206	nonDEG
Bpev01.c0253.g0023.m0001	0.015478083	0.95941676	1	nonDEG
Bpev01.c0089.g0084.m0001	-0.879337444	0.0088907	0.05651767	nonDEG
Bpev01.c0557.g0022.m0001	-0.361063281	0.2338948	0.54931224	nonDEG
Bpev01.c0245.g0004.m0001	0.197385372	0.53939964	0.85679065	nonDEG
Bpev01.c0598.g0004.m0001	-0.200812238	0.61359073	0.91636517	nonDEG
Bpev01.c0327.g0052.m0001	-0.270093164	0.47346848	0.80179697	nonDEG
Bpev01.c0479.g0002.m0001	0.416375899	0.32716916	0.66200905	nonDEG
Bpev01.c0395.g0051.m0001	-0.422152641	0.31722376	0.6499687	nonDEG
Bpev01.c0732.g0017.m0001	-0.023197924	1	1	nonDEG

Bpev01.c0001.g0054.m0001	0.552254939	0.08459069	0.28643221	nonDEG
Bpev01.c0345.g0007.m0001	0.239106215	0.7508115	0.98791208	nonDEG
Bpev01.c0029.g0149.m0001	0.27650098	0.35485251	0.69285757	nonDEG
Bpev01.c0719.g0013.m0001	-6.002348373	0.025191	0.12259842	nonDEG
Bpev01.c1160.g0008.m0001	1.236963743	0.08626755	0.29001145	nonDEG
Bpev01.c0357.g0055.m0002	0.078583466	1	1	nonDEG
Bpev01.c1000.g0010.m0001	-0.146805143	0.65215927	0.93220195	nonDEG
Bpev01.c0145.g0004.m0001	0.607498136	0.05517531	0.21464092	nonDEG
Bpev01.c0800.g0018.m0001	0.827700286	0.02113443	0.10733661	nonDEG
Bpev01.c0142.g0048.m0001	0.974231439	0.01923192	0.10013594	nonDEG
Bpev01.c1202.g0032.m0001	-0.078264813	0.86473479	1	nonDEG
Bpev01.c0679.g0019.m0001	2.351728939	0.1390596	0.39973717	nonDEG
Bpev01.c0088.g0068.m0001	0.569427323	0.07353638	0.26139067	nonDEG
Bpev01.c0212.g0012.m0001	-0.041373001	0.88955758	1	nonDEG
Bpev01.c0127.g0043.m0001	0.923209703	0.02697437	0.12845334	nonDEG
Bpev01.c0516.g0007.m0001	-1.036451928	0.01093238	0.06618102	nonDEG
Bpev01.c0051.g0011.m0001	-0.378937163	0.42106313	0.75248838	nonDEG
Bpev01.c0639.g0003.m0001	0.33514317	0.27640571	0.60324025	nonDEG
Bpev01.c0163.g0028.m0001	0.597811702	0.08217129	0.28112035	nonDEG
Bpev01.c1693.g0002.m0001	-0.4632069	0.13743467	0.39681633	nonDEG
Bpev01.c0765.g0010.m0001	-0.525371828	0.1084531	0.33817932	nonDEG
Bpev01.c0357.g0033.m0001	-0.576679354	0.08318942	0.28331945	nonDEG
Bpev01.c0305.g0001.m0001	-0.196999123	0.89444698	1	nonDEG
Bpev01.c0080.g0060.m0001	0.176987617	0.66277141	0.93220195	nonDEG
Bpev01.c0621.g0014.m0001	1.497686443	0.03471878	0.1542306	nonDEG
Bpev01.c0580.g0007.m0001	0.768113608	0.01941915	0.10077042	nonDEG
Bpev01.c4635.g0001.m0001	0.121756177	0.80912287	1	nonDEG
Bpev01.c1289.g0002.m0001	0.373971904	0.26114086	0.58315449	nonDEG
Bpev01.c0889.g0018.m0001	-0.68381414	0.02517872	0.12257918	nonDEG
Bpev01.c0126.g0013.m0001	0.227157181	0.45858465	0.78772909	nonDEG
Bpev01.c1544.g0011.m0001	-0.087794937	0.7796506	1	nonDEG
Bpev01.c0094.g0033.m0001	0.18969566	0.70211127	0.95802798	nonDEG
Bpev01.c0121.g0014.m0003	0.053830681	0.91531872	1	nonDEG
Bpev01.c0374.g0016.m0001	0.831109287	0.0416177	0.17625186	nonDEG
Bpev01.c0115.g0075.m0001	0.423384528	0.1686404	0.45190286	nonDEG
Bpev01.c0166.g0006.m0001	-0.022049926	0.95840899	1	nonDEG
Bpev01.c0331.g0001.m0001	0.167295519	0.61810765	0.91944472	nonDEG
Bpev01.c0127.g0052.m0001	-1.593698147	0.24952788	0.56937769	nonDEG
Bpev01.c0505.g0025.m0001	-0.025496177	0.93455632	1	nonDEG
Bpev01.c1479.g0006.m0001	-0.396614955	0.18361457	0.47641717	nonDEG
Bpev01.c0442.g0016.m0001	0.913308673	0.42790439	0.76002103	nonDEG
Bpev01.c1628.g0003.m0001	-1.133980138	0.18347587	0.47616999	nonDEG
Bpev01.c0088.g0087.m0001	-0.801360662	0.00926286	0.0583322	nonDEG

Bpev01.c0815.g0006.m0001	0.706742013	0.02827862	0.13302883	nonDEG
Bpev01.c1401.g0001.m0001	0.366177059	0.37476649	0.71377051	nonDEG
Bpev01.c0015.g0267.m0001	0.125087672	0.80701497	1	nonDEG
Bpev01.c0126.g0049.m0001	0.481840432	0.14962448	0.4188262	nonDEG
Bpev01.c0322.g0008.m0001	0.523376004	0.11673272	0.3557838	nonDEG
Bpev01.c0428.g0010.m0001	-0.17780511	0.58194946	0.89261763	nonDEG
Bpev01.c1280.g0016.m0001	0.699094579	0.02370854	0.11718529	nonDEG
Bpev01.c0946.g0009.m0001	-0.674808028	0.38426821	0.71842431	nonDEG
Bpev01.c2000.g0008.m0001	0.022225683	0.94317099	1	nonDEG
Bpev01.c2235.g0011.m0001	-0.132784998	0.66003015	0.93220195	nonDEG
Bpev01.c0419.g0025.m0001	0.923581946	0.29781373	0.62816782	nonDEG
Bpev01.c0032.g0005.m0001	0.301584974	0.73105082	0.97666405	nonDEG
Bpev01.c1782.g0003.m0001	-0.397123802	0.45208928	0.78330192	nonDEG
Bpev01.c0121.g0030.m0001	2.260146052	0.02449674	0.12005853	nonDEG
Bpev01.c1653.g0007.m0001	0.779012998	0.30251412	0.6341294	nonDEG
Bpev01.c0038.g0031.m0002	0.142390779	0.67803545	0.93994563	nonDEG
Bpev01.c0104.g0005.m0001	0.108081013	0.72977784	0.97576589	nonDEG
Bpev01.c0510.g0005.m0001	-0.364993245	0.25051921	0.57064647	nonDEG
Bpev01.c2198.g0001.m0001	-0.362852099	0.22446463	0.53459904	nonDEG
Bpev01.c0101.g0079.m0001	0.353810338	0.30002034	0.63074333	nonDEG
Bpev01.c0000.g0230.m0001	-0.158623698	1	1	nonDEG
Bpev01.c1407.g0002.m0001	-0.266731894	0.38063353	0.71669142	nonDEG
Bpev01.c1031.g0023.m0001	-0.992376094	0.01505203	0.08379664	nonDEG
Bpev01.c0016.g0151.m0001	1.181590656	0.0336167	0.15063043	nonDEG
Bpev01.c0366.g0020.m0001	1.038569404	0.51015451	0.83085083	nonDEG
Bpev01.c0150.g0006.m0001	-0.860672247	0.1273092	0.37746556	nonDEG
Bpev01.c0540.g0015.m0001	0.379362171	0.67885607	0.9406403	nonDEG
Bpev01.c0292.g0013.m0001	-0.346482903	0.2454032	0.5636686	nonDEG
Bpev01.c0001.g0011.m0001	0.637907961	0.04890328	0.1971602	nonDEG
Bpev01.c0212.g0024.m0001	-1.481766476	0.00869244	0.05554521	nonDEG
Bpev01.c0127.g0013.m0001	0.00830623	0.97868093	1	nonDEG
Bpev01.c2643.g0001.m0001	-0.17159796	0.58625684	0.89618938	nonDEG
Bpev01.c0062.g0044.m0001	0.3500875	0.43507807	0.76731797	nonDEG
Bpev01.c0389.g0045.m0001	0.342184892	0.28951123	0.61836472	nonDEG
Bpev01.c1233.g0001.m0001	1.176237349	0.28496385	0.61229478	nonDEG
Bpev01.c0285.g0005.m0001	0.070820083	0.83439384	1	nonDEG
Bpev01.c0091.g0016.m0001	-0.006096041	0.98448794	1	nonDEG
Bpev01.c1140.g0013.m0001	0.094109753	0.76650467	0.9983246	nonDEG
Bpev01.c0038.g0076.m0001	0.101819964	0.73898741	0.98179669	nonDEG
Bpev01.c0992.g0007.m0001	-0.024377287	0.94105662	1	nonDEG
Bpev01.c0121.g0026.m0001	-0.084269645	0.83280754	1	nonDEG
Bpev01.c0015.g0202.m0001	0.172812405	0.57689205	0.88841633	nonDEG
Bpev01.c0480.g0050.m0001	-0.035492154	0.93824526	1	nonDEG

Bpev01.c0022.g0087.m0001	-1.152535842	0.01070908	0.06508353	nonDEG
Bpev01.c0963.g0015.m0001	-0.369100035	0.23968834	0.55652536	nonDEG
Bpev01.c0177.g0052.m0001	0.017121072	0.95688038	1	nonDEG
Bpev01.c1418.g0003.m0001	0.48414311	0.26183586	0.58404197	nonDEG
Bpev01.c0018.g0100.m0002	0.276106255	0.37493143	0.71392967	nonDEG
Bpev01.c0118.g0016.m0001	-0.210404829	0.50866909	0.82978336	nonDEG
Bpev01.c0051.g0177.m0001	0.439865465	0.17790837	0.46687864	nonDEG
Bpev01.c0645.g0029.m0001	0.671079578	0.03445435	0.15332776	nonDEG
Bpev01.c3074.g0001.m0001	-0.44166705	0.15568974	0.42959511	nonDEG
Bpev01.c0161.g0105.m0001	0.720302011	0.01838409	0.09692145	nonDEG
Bpev01.c0157.g0055.m0001	0.949833744	0.41019448	0.74412838	nonDEG
Bpev01.c0540.g0013.m0001	-0.124478315	0.78656451	1	nonDEG
Bpev01.c0449.g0045.m0003	0.91613529	0.32235205	0.65595307	nonDEG
Bpev01.c0480.g0056.m0001	0.24287501	0.41983807	0.75143562	nonDEG
Bpev01.c0170.g0005.m0001	-0.043203457	0.89043468	1	nonDEG
Bpev01.c0337.g0001.m0001	-1.087641547	0.01276931	0.07423297	nonDEG
Bpev01.c1280.g0014.m0001	0.625252174	0.06522769	0.24066147	nonDEG
Bpev01.c0127.g0054.m0001	-0.389367044	0.23716917	0.55408485	nonDEG
Bpev01.c0188.g0016.m0001	-0.200648748	0.51731244	0.83745729	nonDEG
Bpev01.c1138.g0006.m0001	0.700132064	0.02107888	0.10713762	nonDEG
Bpev01.c0679.g0001.m0001	0.390045502	0.239738	0.55655286	nonDEG
Bpev01.c0268.g0004.m0001	0.243028506	0.47751939	0.8060604	nonDEG
Bpev01.c0954.g0005.m0001	-1.748824683	0.01649446	0.08968975	nonDEG
Bpev01.c0506.g0038.m0001	-0.742286075	0.28116156	0.60830961	nonDEG
Bpev01.c1089.g0007.m0001	0.57781364	0.06629163	0.24351876	nonDEG
Bpev01.c1134.g0007.m0001	-0.15622381	0.62502298	0.92415465	nonDEG
Bpev01.c0022.g0133.m0001	-0.189825195	0.54171499	0.85878015	nonDEG
Bpev01.c0000.g0062.m0001	0.219526308	0.47152913	0.80016734	nonDEG
Bpev01.c0652.g0006.m0001	-0.818549814	0.07664052	0.26862963	nonDEG
Bpev01.c0157.g0010.m0001	-0.481443745	0.13048775	0.38326584	nonDEG
Bpev01.c0631.g0003.m0001	0.04257066	0.97384898	1	nonDEG
Bpev01.c0157.g0054.m0001	0.271861789	0.45975621	0.78878952	nonDEG
Bpev01.c0088.g0108.m0001	-0.666678733	0.35041748	0.68805044	nonDEG
Bpev01.c1084.g0006.m0001	0.602384577	0.17460979	0.46234374	nonDEG
Bpev01.c1411.g0004.m0001	0.236433198	0.68573592	0.94576621	nonDEG
Bpev01.c0019.g0002.m0001	-0.037679096	0.90450498	1	nonDEG
Bpev01.c0467.g0016.m0001	-0.296300602	0.3418419	0.67822374	nonDEG
Bpev01.c0420.g0025.m0001	-0.529239798	0.109024	0.33927669	nonDEG
Bpev01.c0029.g0031.m0001	-0.27522722	0.40557923	0.73885191	nonDEG
Bpev01.c0205.g0004.m0001	-0.537717528	0.09954093	0.31951842	nonDEG
Bpev01.c0015.g0156.m0001	1.063156982	0.46028828	0.7890797	nonDEG
Bpev01.c0357.g0010.m0001	-0.518107563	0.09378163	0.30669101	nonDEG
Bpev01.c2314.g0006.m0001	-1.124749857	0.0131304	0.07571802	nonDEG

Bpev01.c0278.g0024.m0001	0.318289558	0.30080069	0.63188751	nonDEG
Bpev01.c0050.g0003.m0001	-0.669706729	0.04838795	0.1954573	nonDEG
Bpev01.c1448.g0006.m0001	1.320570921	0.24485461	0.56299071	nonDEG
Bpev01.c0088.g0134.m0001	0.070186614	0.83237414	1	nonDEG
Bpev01.c0640.g0004.m0001	-0.640823473	0.27376394	0.59985407	nonDEG
Bpev01.c1518.g0008.m0001	0.075630763	0.82723704	1	nonDEG
Bpev01.c1202.g0054.m0001	-0.675311075	0.04471633	0.18543513	nonDEG
Bpev01.c1087.g0008.m0001	0.526281118	0.49550435	0.81913398	nonDEG
Bpev01.c1064.g0010.m0001	0.012144551	0.97319661	1	nonDEG
Bpev01.c0203.g0002.m0001	0.378111799	0.24476154	0.56288843	nonDEG
Bpev01.c0134.g0127.m0002	0.139048652	0.65298106	0.93220195	nonDEG
Bpev01.c0151.g0004.m0001	-0.725942945	0.02572842	0.12443223	nonDEG
Bpev01.c0159.g0031.m0001	0.094503464	0.90879034	1	nonDEG
Bpev01.c0404.g0014.m0001	0.239820509	0.44298614	0.77427233	nonDEG
Bpev01.c0321.g0009.m0001	-0.985091935	0.01105227	0.06674209	nonDEG
Bpev01.c0251.g0001.m0001	-0.866659692	0.0077809	0.05107289	nonDEG
Bpev01.c0786.g0011.m0001	-0.806601797	0.04071135	0.17356343	nonDEG
Bpev01.c2294.g0005.m0001	1.101781468	0.37086415	0.71046833	nonDEG
Bpev01.c1389.g0007.m0001	-0.213312954	0.50443877	0.82670896	nonDEG
Bpev01.c0053.g0016.m0001	0.477954267	0.13953048	0.40027653	nonDEG
Bpev01.c0157.g0064.m0001	0.344648218	0.52640393	0.84567388	nonDEG
Bpev01.c0051.g0167.m0001	0.401385585	0.25439343	0.57509421	nonDEG
Bpev01.c0050.g0014.m0001	0.724297519	0.08279368	0.28237745	nonDEG
Bpev01.c0147.g0021.m0001	0.477296282	0.13579044	0.3933807	nonDEG
Bpev01.c0483.g0007.m0001	0.387456235	0.60112505	0.90705641	nonDEG
Bpev01.c0000.g0075.m0001	-0.160274321	0.61464949	0.91706395	nonDEG
Bpev01.c0365.g0019.m0002	0.355432774	0.28228313	0.60909233	nonDEG
Bpev01.c0496.g0017.m0001	0.481471056	0.14245773	0.40558147	nonDEG
Bpev01.c0237.g0059.m0001	-0.061321985	0.85450206	1	nonDEG
Bpev01.c0052.g0037.m0001	-0.529960454	0.29064182	0.61983517	nonDEG
Bpev01.c1339.g0013.m0001	1.627545818	0.06294925	0.23493444	nonDEG
Bpev01.c0357.g0034.m0001	0.384395264	0.41911443	0.75077947	nonDEG
Bpev01.c0992.g0006.m0001	0.052072192	0.86806299	1	nonDEG
Bpev01.c0135.g0048.m0001	0.374862457	0.23442185	0.55015504	nonDEG
Bpev01.c1700.g0001.m0001	0.415391367	0.39725529	0.7302403	nonDEG
Bpev01.c0120.g0039.m0001	-0.37143997	0.31796421	0.65071592	nonDEG
Bpev01.c0817.g0016.m0001	0.483028024	0.1407216	0.40214222	nonDEG
Bpev01.c0043.g0028.m0001	0.235148174	0.44040589	0.77223831	nonDEG
Bpev01.c0157.g0059.m0001	0.667150486	0.41637893	0.74822626	nonDEG
Bpev01.c1042.g0003.m0001	1.064268419	0.44994749	0.78133687	nonDEG
Bpev01.c1064.g0006.m0001	0	1	1	nonDEG
Bpev01.c0366.g0019.m0001	0.106731628	0.72065689	0.97062506	nonDEG
Bpev01.c2148.g0002.m0001	3.275739567	0.02357128	0.11660473	nonDEG

Bpev01.c0170.g0058.m0001	0.379962957	0.23814655	0.55491488	nonDEG
Bpev01.c1069.g0008.m0001	-0.005729644	0.98580713	1	nonDEG
Bpev01.c1115.g0002.m0001	0.116406665	0.78834514	1	nonDEG
Bpev01.c0402.g0028.m0001	0.223392107	0.46180888	0.79073189	nonDEG
Bpev01.c0016.g0144.m0001	0.521680994	0.89991126	1	nonDEG
Bpev01.c0488.g0012.m0002	0.363379419	0.25451057	0.57513921	nonDEG
Bpev01.c0095.g0008.m0001	0.879664766	0.34968674	0.68726196	nonDEG
Bpev01.c1715.g0001.m0001	-0.064468988	0.85192347	1	nonDEG
Bpev01.c0645.g0026.m0001	0.636561319	0.041446	0.17578575	nonDEG
Bpev01.c0050.g0088.m0001	3.105242455	0.03892075	0.16795484	nonDEG
Bpev01.c0289.g0018.m0001	-0.704177053	0.02024181	0.10370671	nonDEG
Bpev01.c0094.g0069.m0001	0.218128153	0.5411918	0.85836676	nonDEG
Bpev01.c0016.g0169.m0002	-0.361159245	0.22970933	0.54294766	nonDEG
Bpev01.c0439.g0045.m0001	1.019454628	0.01335932	0.07673855	nonDEG
Bpev01.c2795.g0002.m0001	-1.025946294	0.0086762	0.05550168	nonDEG
Bpev01.c0055.g0028.m0001	-0.65086129	0.03825119	0.16567262	nonDEG
Bpev01.c0114.g0003.m0002	2.492068237	0.05105873	0.20345396	nonDEG
Bpev01.c1349.g0011.m0001	-0.168177105	0.58450687	0.89481511	nonDEG
Bpev01.c0276.g0022.m0001	0.909527596	0.02822836	0.13283485	nonDEG
Bpev01.c0044.g0103.m0001	-0.342168146	0.33018772	0.66587178	nonDEG
Bpev01.c1163.g0005.m0001	0.209264261	0.55180063	0.86748152	nonDEG
Bpev01.c0629.g0011.m0001	-0.295327501	0.47305364	0.80141717	nonDEG
Bpev01.c0188.g0019.m0001	0.535488747	0.20714853	0.5108147	nonDEG
Bpev01.c0000.g0097.m0001	-0.15998914	0.75061547	0.98790096	nonDEG
Bpev01.c0760.g0002.m0001	-0.773665059	0.03781142	0.16431656	nonDEG
Bpev01.c0462.g0002.m0001	-0.192525589	0.57100284	0.88364489	nonDEG
Bpev01.c0155.g0023.m0001	-0.005141396	0.98904126	1	nonDEG
Bpev01.c1511.g0001.m0001	0.140313761	0.69654168	0.95399409	nonDEG
Bpev01.c0518.g0015.m0001	0.081776053	0.78825327	1	nonDEG
Bpev01.c0483.g0009.m0002	0.761560214	0.01910138	0.0995619	nonDEG
Bpev01.c0022.g0021.m0001	0.092848601	0.83449315	1	nonDEG
Bpev01.c0114.g0029.m0001	0.641770999	0.04244251	0.17877595	nonDEG
Bpev01.c0051.g0166.m0001	0.431656155	0.17049431	0.45533721	nonDEG
Bpev01.c1407.g0003.m0001	-0.04640457	0.88847688	1	nonDEG
Bpev01.c0420.g0001.m0001	-0.180775936	0.61536906	0.91716533	nonDEG
Bpev01.c0335.g0013.m0001	-0.495198459	0.40102048	0.73390787	nonDEG
Bpev01.c0968.g0009.m0001	-0.672355461	0.03027807	0.13960159	nonDEG
Bpev01.c0121.g0039.m0001	0.155691506	0.61734408	0.9190658	nonDEG
Bpev01.c0051.g0190.m0001	0.095426751	0.76441422	0.99687634	nonDEG
Bpev01.c3736.g0001.m0001	0.482040446	0.12725068	0.37733006	nonDEG
Bpev01.c0050.g0104.m0001	3.420707714	0.01606761	0.08795357	nonDEG
Bpev01.c0099.g0001.m0001	0.563351609	0.54274709	0.85953691	nonDEG
Bpev01.c0473.g0033.m0001	0.358289477	0.26171209	0.58398713	nonDEG

Bpev01.c2517.g0006.m0001	-0.864853362	0.01714039	0.09216363	nonDEG
Bpev01.c1938.g0016.m0001	-0.01887726	1	1	nonDEG
Bpev01.c0206.g0008.m0001	-0.015546107	0.97683656	1	nonDEG
Bpev01.c0913.g0007.m0001	-0.845464436	0.01016413	0.06257303	nonDEG
Bpev01.c0667.g0013.m0001	0.386458358	0.31023754	0.64060342	nonDEG
Bpev01.c0090.g0024.m0001	0.546324079	0.10029279	0.32127162	nonDEG
Bpev01.c3359.g0001.m0001	-0.60020464	0.0536432	0.21047611	nonDEG
Bpev01.c0574.g0042.m0001	0.250715201	0.41731527	0.74895397	nonDEG
Bpev01.c1561.g0004.m0001	-0.031441715	0.98674021	1	nonDEG
Bpev01.c0940.g0011.m0001	-0.614434492	0.09069142	0.29993166	nonDEG
Bpev01.c0155.g0028.m0001	-0.64837683	0.20693146	0.5103766	nonDEG
Bpev01.c0135.g0054.m0001	0.352085726	0.25157466	0.5721993	nonDEG
Bpev01.c0603.g0003.m0001	1.117142409	0.02916119	0.13594241	nonDEG
Bpev01.c0392.g0006.m0001	0.422559865	0.23535682	0.55142613	nonDEG
Bpev01.c0340.g0007.m0001	0.870403981	0.00957182	0.05975078	nonDEG
Bpev01.c0062.g0032.m0001	-0.068881352	0.82392394	1	nonDEG
Bpev01.c0414.g0012.m0001	-0.15254754	0.61486367	0.91706395	nonDEG
Bpev01.c0845.g0004.m0001	1.110572897	0.19471649	0.4932157	nonDEG
Bpev01.c0887.g0011.m0001	0.406091017	0.82216207	1	nonDEG
Bpev01.c1192.g0005.m0001	0.31606598	0.34035079	0.67639145	nonDEG
Bpev01.c0090.g0016.m0001	0.804192645	0.01449864	0.08145526	nonDEG
Bpev01.c0091.g0050.m0001	-0.695843902	0.02455168	0.12017019	nonDEG
Bpev01.c1333.g0007.m0001	-0.237096427	0.72834751	0.97560389	nonDEG
Bpev01.c3534.g0001.m0001	0.134249727	0.74302672	0.98399997	nonDEG
Bpev01.c0992.g0019.m0001	-0.222913134	0.50036941	0.82288	nonDEG
Bpev01.c0063.g0027.m0001	1.180352831	0.03381618	0.15128402	nonDEG
Bpev01.c1270.g0001.m0001	-0.375473969	0.23051399	0.54428146	nonDEG
Bpev01.c0051.g0081.m0001	-0.651733771	0.05111831	0.20366383	nonDEG
Bpev01.c1350.g0008.m0001	-0.146685327	0.7763134	1	nonDEG
Bpev01.c0451.g0025.m0001	-0.519191614	0.08654075	0.2906312	nonDEG
Bpev01.c0167.g0022.m0002	-0.796935726	0.01245845	0.07293915	nonDEG
Bpev01.c0470.g0038.m0001	-0.926995115	0.05928541	0.22528763	nonDEG
Bpev01.c0188.g0025.m0001	-0.709361655	0.02390178	0.11781755	nonDEG
Bpev01.c0522.g0043.m0001	1.09384642	0.22250417	0.53182623	nonDEG
Bpev01.c2396.g0003.m0001	0.069069579	0.85863553	1	nonDEG
Bpev01.c0689.g0003.m0001	-0.04985915	0.88135201	1	nonDEG
Bpev01.c0874.g0005.m0001	0.208453683	0.50413482	0.82640398	nonDEG
Bpev01.c0760.g0003.m0001	0.165346907	0.63068197	0.92772931	nonDEG
Bpev01.c0789.g0009.m0001	-0.10586817	0.72777641	0.9751495	nonDEG
Bpev01.c2411.g0001.m0001	0.834472794	0.08547053	0.28823506	nonDEG
Bpev01.c0016.g0147.m0001	0.182880289	0.58039125	0.8911562	nonDEG
Bpev01.c2192.g0005.m0001	-0.472567396	0.14326854	0.40699435	nonDEG
Bpev01.c0803.g0019.m0002	-0.3051974	0.36683704	0.70639166	nonDEG

Bpev01.c0089.g0005.m0001	-0.668259539	0.12198899	0.36734861	nonDEG
Bpev01.c1692.g0003.m0001	0.777117175	0.087517	0.29287405	nonDEG
Bpev01.c2348.g0001.m0001	0.007238763	0.98571599	1	nonDEG
Bpev01.c0304.g0024.m0001	-0.335437669	0.65392539	0.93220195	nonDEG
Bpev01.c1365.g0002.m0001	-0.475446825	0.12668348	0.37606484	nonDEG
Bpev01.c0253.g0007.m0001	0.278372147	0.43730365	0.7696146	nonDEG
Bpev01.c0000.g0095.m0001	0.509009257	0.11914815	0.36116171	nonDEG
Bpev01.c1298.g0006.m0001	-0.727364955	0.03617458	0.15897053	nonDEG
Bpev01.c0042.g0007.m0001	-0.188863069	0.55631389	0.87201559	nonDEG
Bpev01.c0327.g0032.m0001	-0.043344041	0.88554972	1	nonDEG
Bpev01.c0195.g0013.m0001	0.410404949	0.2775707	0.60461738	nonDEG
Bpev01.c1072.g0020.m0001	0.41734943	0.22532109	0.53610503	nonDEG
Bpev01.c1320.g0003.m0001	0.102608399	0.93474179	1	nonDEG
Bpev01.c0442.g0009.m0001	0.18520671	0.5806214	0.89118422	nonDEG
Bpev01.c1106.g0004.m0001	0	1	1	nonDEG
Bpev01.c0618.g0007.m0001	-0.210811131	0.55683482	0.87222839	nonDEG
Bpev01.c0603.g0002.m0001	-0.35201087	0.24082827	0.55802783	nonDEG
Bpev01.c0588.g0034.m0002	0.078714576	0.81581303	1	nonDEG
Bpev01.c0395.g0018.m0001	0.371110903	0.21253191	0.51884294	nonDEG
Bpev01.c1779.g0002.m0001	0.347588194	0.44687906	0.77820881	nonDEG
Bpev01.c0506.g0016.m0001	0.796544565	0.02514463	0.12245374	nonDEG
Bpev01.c1856.g0001.m0001	0.30739938	0.7366006	0.98005899	nonDEG
Bpev01.c1099.g0010.m0001	0.437937466	0.24666959	0.56572967	nonDEG
Bpev01.c0025.g0016.m0001	0.335950152	0.61971116	0.92050364	nonDEG
Bpev01.c0115.g0118.m0001	-0.251742742	0.47497128	0.80369465	nonDEG
Bpev01.c0148.g0009.m0001	0.454215221	0.2528512	0.57409275	nonDEG
Bpev01.c1579.g0014.m0004	-0.338411357	0.54707551	0.86328023	nonDEG
Bpev01.c1782.g0014.m0002	0.592270822	0.04973575	0.19956138	nonDEG
Bpev01.c0328.g0030.m0001	0.124415018	0.77676897	1	nonDEG
Bpev01.c0088.g0083.m0001	-0.213189924	0.58888851	0.89859732	nonDEG
Bpev01.c0038.g0155.m0002	0.870368467	0.04259186	0.17930241	nonDEG
Bpev01.c0027.g0142.m0001	-0.209537726	0.48772087	0.81424325	nonDEG
Bpev01.c0016.g0071.m0001	-0.291493579	0.347845	0.68482639	nonDEG
Bpev01.c1534.g0006.m0001	-0.271887349	0.48021684	0.80903648	nonDEG
Bpev01.c1721.g0007.m0001	-0.499543509	0.12575802	0.37486722	nonDEG
Bpev01.c0777.g0008.m0001	0.399200317	0.57872343	0.889802	nonDEG
Bpev01.c0029.g0073.m0001	0.704069575	0.02391249	0.11781755	nonDEG
Bpev01.c0383.g0023.m0001	-0.792511205	0.01529985	0.08467988	nonDEG
Bpev01.c0129.g0076.m0001	-0.107739491	0.8039482	1	nonDEG
Bpev01.c0050.g0030.m0001	-0.645808214	0.06752681	0.24651808	nonDEG
Bpev01.c0854.g0008.m0001	0.118569892	0.73123428	0.97675014	nonDEG
Bpev01.c0517.g0010.m0001	0.541287818	0.11551506	0.3535712	nonDEG
Bpev01.c1005.g0013.m0001	-0.307087311	0.33241528	0.66789336	nonDEG

Bpev01.c0016.g0150.m0001	0.906040233	0.07821225	0.27218383	nonDEG
Bpev01.c0863.g0006.m0001	0.615162642	0.04780592	0.19382431	nonDEG
Bpev01.c0182.g0004.m0001	0.959119648	0.04117284	0.17495487	nonDEG
Bpev01.c0015.g0178.m0001	0.50088138	0.0953465	0.31021634	nonDEG
Bpev01.c0080.g0096.m0001	0.523677889	0.17453276	0.46218134	nonDEG
Bpev01.c0577.g0037.m0001	-0.137307376	0.66716802	0.93220195	nonDEG
Bpev01.c0376.g0004.m0001	-0.451110696	0.14064937	0.40205565	nonDEG
Bpev01.c0018.g0042.m0001	0.485170626	0.10549675	0.33219623	nonDEG
Bpev01.c0121.g0020.m0001	-0.564973672	0.0957638	0.31099666	nonDEG
Bpev01.c0684.g0006.m0001	0.674464189	0.06961868	0.25180124	nonDEG
Bpev01.c1426.g0001.m0001	-0.070647298	0.86239601	1	nonDEG
Bpev01.c1270.g0015.m0001	-0.336831345	0.31242876	0.64354815	nonDEG
Bpev01.c1972.g0013.m0001	-0.556351938	0.11633886	0.35498547	nonDEG
Bpev01.c0984.g0011.m0001	0.944720265	0.18278752	0.47498294	nonDEG
Bpev01.c0016.g0148.m0001	0.281856379	0.69098637	0.94933666	nonDEG
Bpev01.c2444.g0002.m0001	0.325433604	0.59405019	0.90238771	nonDEG
Bpev01.c0480.g0091.m0001	0.410086194	0.18347749	0.47616999	nonDEG
Bpev01.c0050.g0047.m0001	-0.453485284	0.16200693	0.44050263	nonDEG
Bpev01.c0088.g0118.m0001	-0.579469202	0.05850962	0.22334865	nonDEG
Bpev01.c0080.g0088.m0001	0.899778985	0.01498195	0.08350125	nonDEG
Bpev01.c1140.g0001.m0001	-0.290636951	0.33665567	0.67226003	nonDEG
Bpev01.c0120.g0011.m0001	-0.647191283	0.28160071	0.60830961	nonDEG
Bpev01.c2226.g0002.m0001	-0.451534699	0.13821812	0.39853117	nonDEG
Bpev01.c0162.g0069.m0001	0.625068796	0.0365098	0.16001368	nonDEG
Bpev01.c1204.g0002.m0001	-0.152693228	0.66425223	0.93220195	nonDEG
Bpev01.c1029.g0011.m0001	-0.375477364	0.23033543	0.54398342	nonDEG
Bpev01.c2499.g0001.m0001	0.207064782	0.71106262	0.96423727	nonDEG
Bpev01.c0575.g0006.m0001	0.166477676	0.63813261	0.93006545	nonDEG
Bpev01.c1381.g0001.m0001	0.588712171	0.17616699	0.46471148	nonDEG
Bpev01.c0066.g0058.m0001	0.52071041	0.2309312	0.54491692	nonDEG
Bpev01.c1522.g0001.m0001	0.386075451	0.47527066	0.80397017	nonDEG
Bpev01.c0082.g0021.m0001	0.274747381	0.40171811	0.7347735	nonDEG
Bpev01.c0961.g0002.m0001	-0.760408838	0.07529382	0.2650771	nonDEG
Bpev01.c0570.g0020.m0001	-0.753281132	0.02329873	0.11552825	nonDEG
Bpev01.c0537.g0006.m0001	-0.631248122	0.0513347	0.20411158	nonDEG
Bpev01.c0015.g0127.m0001	0.308750433	0.35110111	0.68893326	nonDEG
Bpev01.c0281.g0008.m0001	0.791806621	0.01994498	0.1026504	nonDEG
Bpev01.c0365.g0012.m0002	0.638747342	0.05841768	0.22320027	nonDEG
Bpev01.c0177.g0016.m0001	-0.641780486	0.04068393	0.17352797	nonDEG
Bpev01.c0281.g0017.m0001	0.045794073	0.87867773	1	nonDEG
Bpev01.c0454.g0013.m0001	-0.131668442	0.69063084	0.94918214	nonDEG
Bpev01.c0307.g0002.m0001	0.206742493	0.49189264	0.81591656	nonDEG
Bpev01.c0038.g0062.m0001	0.637602026	0.04619854	0.189315	nonDEG

Bpev01.c4451.g0001.m0001	-1.272309058	0.57996189	0.8908084	nonDEG
Bpev01.c0517.g0037.m0001	-0.088530221	0.78297826	1	nonDEG
Bpev01.c0030.g0006.m0001	1.628473724	0.05362583	0.21046404	nonDEG
Bpev01.c0659.g0011.m0001	0.068898148	0.82382358	1	nonDEG
Bpev01.c0327.g0040.m0001	0.239689456	0.43294444	0.76475767	nonDEG
Bpev01.c0189.g0015.m0001	-0.192532825	0.64495375	0.93220195	nonDEG
Bpev01.c1350.g0007.m0001	-0.545306179	0.17724743	0.46593331	nonDEG
Bpev01.c0023.g0044.m0001	0.225508758	0.51314049	0.83359138	nonDEG
Bpev01.c0470.g0009.m0001	-0.229789198	0.49858255	0.82136323	nonDEG
Bpev01.c0135.g0023.m0001	0.366004979	0.3825318	0.71751627	nonDEG
Bpev01.c1639.g0006.m0001	-0.857134236	0.0363586	0.15961239	nonDEG
Bpev01.c0991.g0005.m0001	-0.55870785	0.09201574	0.30283415	nonDEG
Bpev01.c0547.g0016.m0001	0.778750274	0.07572794	0.26635065	nonDEG
Bpev01.c0121.g0017.m0001	-0.829595284	0.0198119	0.10214244	nonDEG
Bpev01.c0169.g0049.m0001	-0.370661632	0.26040307	0.58208095	nonDEG
Bpev01.c1741.g0004.m0001	0.354621198	0.26348022	0.58619969	nonDEG
Bpev01.c1223.g0004.m0001	-0.318870189	0.4674389	0.79597116	nonDEG
Bpev01.c0142.g0030.m0001	-0.030977844	0.92415709	1	nonDEG
Bpev01.c0602.g0006.m0001	0.358193342	0.35718552	0.69516273	nonDEG
Bpev01.c0279.g0037.m0001	0.401405417	0.20253448	0.50430622	nonDEG
Bpev01.c2499.g0003.m0001	-0.564579081	0.22143974	0.53064423	nonDEG
Bpev01.c0015.g0052.m0001	-0.624868741	0.07434211	0.26311624	nonDEG
Bpev01.c0213.g0012.m0001	0.585427747	0.06120262	0.23040203	nonDEG
Bpev01.c0045.g0065.m0001	-0.449548086	0.16601576	0.44751745	nonDEG
Bpev01.c0713.g0003.m0001	0.462059289	0.12661397	0.37597224	nonDEG
Bpev01.c0135.g0013.m0001	0.648596627	0.03534469	0.15625656	nonDEG
Bpev01.c1185.g0021.m0001	-0.927904952	0.0112989	0.06772429	nonDEG
Bpev01.c0036.g0005.m0001	0.034833908	0.91151025	1	nonDEG
Bpev01.c0661.g0003.m0001	0.215765136	0.48424386	0.81256284	nonDEG
Bpev01.c0408.g0003.m0001	0.100179443	0.86482344	1	nonDEG
Bpev01.c1645.g0001.m0001	-1.194413079	0.38179551	0.71703402	nonDEG
Bpev01.c0242.g0007.m0002	0.366611609	0.29288198	0.62258305	nonDEG
Bpev01.c1837.g0007.m0001	0.445619723	0.34832622	0.68526968	nonDEG
Bpev01.c0650.g0011.m0001	0.808353957	0.28820088	0.6166397	nonDEG
Bpev01.c0240.g0018.m0001	-0.65835449	0.04603133	0.18884007	nonDEG
Bpev01.c0595.g0005.m0001	-0.208859955	0.62053028	0.92108057	nonDEG
Bpev01.c1814.g0004.m0001	-1.067752837	0.05410814	0.21173211	nonDEG
Bpev01.c0055.g0016.m0001	-0.448822404	0.45308436	0.78341188	nonDEG
Bpev01.c1087.g0009.m0001	-0.247438523	0.81157578	1	nonDEG
Bpev01.c0998.g0003.m0001	0.013579741	1	1	nonDEG
Bpev01.c0225.g0001.m0001	-0.242818328	0.56913877	0.8820569	nonDEG
Bpev01.c1892.g0004.m0001	0.352296853	0.27353814	0.5996172	nonDEG
Bpev01.c0027.g0166.m0001	-0.779247117	0.01081527	0.06563402	nonDEG

Bpev01.c2063.g0004.m0001	0.029309227	0.92757152	1	nonDEG
Bpev01.c0222.g0046.m0001	-0.254782071	0.63658131	0.93006545	nonDEG
Bpev01.c0148.g0002.m0001	1.382435244	0.03012047	0.1391237	nonDEG
Bpev01.c0210.g0011.m0002	-0.049125099	0.87685591	1	nonDEG
Bpev01.c0118.g0019.m0001	-0.574421669	0.06375861	0.23702356	nonDEG
Bpev01.c0265.g0001.m0001	0.37931506	0.21832339	0.5258848	nonDEG
Bpev01.c0000.g0144.m0001	-0.331898015	0.28877875	0.61747223	nonDEG
Bpev01.c2774.g0002.m0001	1.332292256	0.50196306	0.82444156	nonDEG
Bpev01.c0129.g0040.m0001	2.920609485	0.08274754	0.28233712	nonDEG
Bpev01.c0222.g0024.m0001	-0.519027807	0.13349909	0.38865504	nonDEG
Bpev01.c3412.g0001.m0001	-0.124003486	0.72628677	0.97408456	nonDEG
Bpev01.c0480.g0034.m0001	-1.559655659	0.13838861	0.39859859	nonDEG
Bpev01.c1527.g0014.m0001	-1.008965405	0.06996176	0.25255724	nonDEG
Bpev01.c0146.g0012.m0001	0.107481183	0.79896266	1	nonDEG
Bpev01.c1295.g0005.m0001	0.795237798	0.25073551	0.57083683	nonDEG
Bpev01.c0168.g0009.m0001	-0.995595634	0.04947795	0.19875923	nonDEG
Bpev01.c0243.g0056.m0001	0.499424863	0.17420661	0.46191592	nonDEG
Bpev01.c1242.g0001.m0002	0.580587827	0.06391323	0.23741129	nonDEG
Bpev01.c0210.g0033.m0002	-0.524257256	0.09915204	0.31862644	nonDEG
Bpev01.c1708.g0002.m0001	-0.142359681	0.679827	0.94150805	nonDEG
Bpev01.c3472.g0001.m0001	0.72014387	0.02836079	0.13326631	nonDEG
Bpev01.c0597.g0016.m0001	-0.487356257	0.15393126	0.42618099	nonDEG
Bpev01.c0999.g0005.m0001	-0.225067444	0.4672325	0.79590659	nonDEG
Bpev01.c0915.g0022.m0001	1.215691616	0.02170772	0.1095395	nonDEG
Bpev01.c0566.g0016.m0001	0.124168481	0.75369394	0.99000443	nonDEG
Bpev01.c0120.g0031.m0001	1.093354739	0.36246019	0.7008516	nonDEG
Bpev01.c0245.g0044.m0001	0.12626565	0.67598601	0.93860731	nonDEG
Bpev01.c0157.g0031.m0001	-0.468111801	0.14310761	0.40677276	nonDEG
Bpev01.c0141.g0021.m0001	0.303887612	0.50692333	0.82857808	nonDEG
Bpev01.c0274.g0014.m0001	0.988687448	0.01363092	0.07779771	nonDEG
Bpev01.c1868.g0005.m0001	1.307287951	0.08738176	0.29252121	nonDEG
Bpev01.c0112.g0013.m0001	-0.577001291	0.17361753	0.46084407	nonDEG
Bpev01.c0088.g0128.m0001	1.269361664	0.202906	0.50478047	nonDEG
Bpev01.c1831.g0003.m0001	-0.124439796	0.76326646	0.99631096	nonDEG
Bpev01.c0582.g0007.m0001	0.500927283	0.26937547	0.59432994	nonDEG
Bpev01.c0706.g0015.m0001	-0.206957293	0.51503851	0.83523262	nonDEG
Bpev01.c1152.g0010.m0001	0.075367702	0.84204316	1	nonDEG
Bpev01.c0051.g0005.m0001	0.061727223	0.85244899	1	nonDEG
Bpev01.c0483.g0001.m0001	0.916829294	0.34208564	0.67861585	nonDEG
Bpev01.c1089.g0014.m0001	0.682308391	0.07869065	0.27310758	nonDEG
Bpev01.c1233.g0011.m0001	0.277394138	0.43729897	0.7696146	nonDEG
Bpev01.c0848.g0036.m0001	-0.034958021	0.91879499	1	nonDEG
Bpev01.c1122.g0010.m0001	0.042036803	0.90575416	1	nonDEG

Bpev01.c0995.g0002.m0001	-0.39005736	0.28187367	0.60849739	nonDEG
Bpev01.c0364.g0028.m0001	-0.086022747	0.78790461	1	nonDEG
Bpev01.c0568.g0031.m0001	-0.38462122	0.19994817	0.50083164	nonDEG
Bpev01.c0558.g0004.m0001	0.776240117	0.12390212	0.37136575	nonDEG
Bpev01.c1677.g0002.m0001	1.453107247	0.17080159	0.45582433	nonDEG
Bpev01.c0051.g0066.m0001	0.344304153	0.33630043	0.6719648	nonDEG
Bpev01.c1917.g0002.m0001	0.878295956	0.49119837	0.81535882	nonDEG
Bpev01.c1828.g0004.m0001	0.167675096	0.6826194	0.94350122	nonDEG
Bpev01.c0261.g0002.m0001	0.400749133	0.26150864	0.58361022	nonDEG
Bpev01.c0918.g0021.m0001	-0.735219255	0.02426425	0.11917116	nonDEG
Bpev01.c0254.g0002.m0001	-0.940869252	0.46859776	0.79698709	nonDEG
Bpev01.c0224.g0042.m0001	-1.132156516	0.01865913	0.09780683	nonDEG
Bpev01.c1307.g0002.m0001	-0.126305508	0.80272673	1	nonDEG
Bpev01.c0126.g0036.m0001	-0.433064947	0.23875788	0.55537242	nonDEG
Bpev01.c0015.g0197.m0001	0.807896624	0.00803138	0.05238993	nonDEG
Bpev01.c0170.g0045.m0001	0.629975932	0.06091154	0.22967569	nonDEG
Bpev01.c1040.g0028.m0001	0.21662021	0.47226654	0.80076738	nonDEG
Bpev01.c0395.g0047.m0001	-0.576962661	0.06939895	0.25114486	nonDEG
Bpev01.c0992.g0002.m0001	0.367347836	0.61071725	0.91407316	nonDEG
Bpev01.c0457.g0048.m0001	0.364391951	0.34585573	0.68239121	nonDEG
Bpev01.c0038.g0061.m0001	-0.816014654	0.02776401	0.13132143	nonDEG
Bpev01.c0215.g0032.m0001	0.026179786	0.97912331	1	nonDEG
Bpev01.c0104.g0004.m0001	0.752409964	0.02524892	0.1227242	nonDEG
Bpev01.c0328.g0004.m0001	0.143436714	0.63692411	0.93006545	nonDEG
Bpev01.c0320.g0014.m0001	0.050607099	0.91748674	1	nonDEG
Bpev01.c0759.g0006.m0002	1.070870824	0.46648397	0.79518364	nonDEG
Bpev01.c1006.g0007.m0001	0.379338645	0.22169788	0.53087955	nonDEG
Bpev01.c0162.g0029.m0001	-0.02529135	0.93888849	1	nonDEG
Bpev01.c0353.g0034.m0002	-0.42646979	0.15138165	0.42217926	nonDEG
Bpev01.c1405.g0006.m0001	0	1	1	nonDEG
Bpev01.c0340.g0029.m0002	0.631544662	0.05282508	0.20854467	nonDEG
Bpev01.c1511.g0004.m0001	-0.117060833	0.75233803	0.98901664	nonDEG
Bpev01.c0605.g0017.m0001	0.10442231	0.79533066	1	nonDEG
Bpev01.c0263.g0007.m0001	0.23906219	0.48059948	0.8093569	nonDEG
Bpev01.c0719.g0016.m0001	-0.914999699	0.01956727	0.10122136	nonDEG
Bpev01.c0219.g0001.m0001	1.015232439	0.0117279	0.06942645	nonDEG
Bpev01.c0214.g0020.m0001	0.492074142	0.72624333	0.97407068	nonDEG
Bpev01.c0022.g0147.m0001	0.385708959	0.25641912	0.57623835	nonDEG
Bpev01.c0082.g0023.m0001	0.128852736	0.68755638	0.94690177	nonDEG
Bpev01.c1344.g0003.m0001	-0.109247425	0.72640732	0.97411311	nonDEG
Bpev01.c1530.g0011.m0001	-0.701795724	0.02487183	0.12144666	nonDEG
Bpev01.c1105.g0007.m0001	0.249179958	0.49737758	0.82043473	nonDEG
Bpev01.c0726.g0009.m0001	-0.153573456	0.66117637	0.93220195	nonDEG

Bpev01.c0768.g0006.m0001	1.369044344	0.19426151	0.49262337	nonDEG
Bpev01.c0145.g0040.m0001	0.053735063	0.87625415	1	nonDEG
Bpev01.c0053.g0045.m0001	0.061003778	0.84729213	1	nonDEG
Bpev01.c0161.g0005.m0001	-1.948718871	0.00853006	0.05480499	nonDEG
Bpev01.c1043.g0008.m0001	-0.250040718	0.40644382	0.73964886	nonDEG
Bpev01.c0223.g0039.m0001	-0.702735968	0.52896063	0.84824446	nonDEG
Bpev01.c0615.g0004.m0001	0.67796043	0.49152728	0.81553693	nonDEG
Bpev01.c0936.g0007.m0001	-0.203392634	0.7418273	0.98341259	nonDEG
Bpev01.c0365.g0019.m0001	0.220823217	0.49131745	0.81545172	nonDEG
Bpev01.c0155.g0019.m0001	0.581527834	0.07428327	0.26304149	nonDEG
Bpev01.c0581.g0008.m0001	-1.487465171	0.63558433	0.93006545	nonDEG
Bpev01.c0171.g0022.m0001	0.302870832	0.33289994	0.66854774	nonDEG
Bpev01.c2521.g0002.m0001	0.197907514	0.79394346	1	nonDEG
Bpev01.c0062.g0074.m0001	-0.278497897	0.42045301	0.75202246	nonDEG
Bpev01.c0134.g0090.m0001	0.021159399	0.95020446	1	nonDEG
Bpev01.c0472.g0009.m0001	-0.82798896	0.13714262	0.39620623	nonDEG
Bpev01.c0253.g0029.m0001	0.067034884	0.84510983	1	nonDEG
Bpev01.c1422.g0003.m0001	-1.02600822	0.01156524	0.06883724	nonDEG
Bpev01.c0343.g0022.m0001	2.400244879	0.10698479	0.33480656	nonDEG
Bpev01.c0120.g0027.m0001	-0.178138784	0.56541577	0.87842497	nonDEG
Bpev01.c0080.g0048.m0001	-0.298130632	0.32718922	0.66200905	nonDEG
Bpev01.c0902.g0019.m0001	0.837325809	0.01345764	0.07697286	nonDEG
Bpev01.c0605.g0031.m0001	0.259778528	0.41417306	0.74697103	nonDEG
Bpev01.c1436.g0005.m0001	-0.615144557	0.25035189	0.57045076	nonDEG
Bpev01.c0509.g0006.m0001	-0.641233001	0.74586747	0.98508715	nonDEG
Bpev01.c0127.g0044.m0001	1.655839422	0.04494375	0.18603755	nonDEG
Bpev01.c0016.g0129.m0001	-0.837102564	0.01323453	0.07618748	nonDEG
Bpev01.c1040.g0025.m0001	-0.752082445	0.42640726	0.75855514	nonDEG
Bpev01.c0327.g0063.m0001	-0.388925422	0.21668358	0.52321078	nonDEG
Bpev01.c0757.g0011.m0001	-0.386608616	0.51289522	0.83340737	nonDEG
Bpev01.c0588.g0003.m0001	-0.392156917	0.22339883	0.53299605	nonDEG
Bpev01.c0024.g0028.m0001	-2.57290727	0.02127906	0.10782003	nonDEG
Bpev01.c0154.g0052.m0001	-0.638878673	0.11823144	0.35896452	nonDEG
Bpev01.c1490.g0003.m0001	0.226704673	0.46362872	0.79250511	nonDEG
Bpev01.c0328.g0039.m0001	0.79688531	0.01402656	0.07948562	nonDEG
Bpev01.c0357.g0082.m0001	0.936814929	0.05744769	0.22084128	nonDEG
Bpev01.c0115.g0057.m0001	-0.512263728	0.09254883	0.30404474	nonDEG
Bpev01.c2048.g0003.m0002	-0.323056315	0.35867524	0.69692035	nonDEG
Bpev01.c0043.g0022.m0001	-0.702934401	0.05006475	0.20044285	nonDEG
Bpev01.c0077.g0009.m0001	-0.478029107	0.13501436	0.39171063	nonDEG
Bpev01.c0616.g0020.m0001	-0.706145171	0.04084869	0.17397923	nonDEG
Bpev01.c1105.g0006.m0001	0.263844637	0.3810459	0.71687811	nonDEG
Bpev01.c0386.g0004.m0002	-0.053944957	0.8677958	1	nonDEG

Bpev01.c0863.g0029.m0001	0.738080644	0.01748707	0.09343049	nonDEG
Bpev01.c2017.g0001.m0001	0.17271603	0.57598429	0.88735158	nonDEG
Bpev01.c1406.g0002.m0001	0.115338202	0.7329587	0.97781941	nonDEG
Bpev01.c0126.g0045.m0001	-0.502484453	0.152876	0.42449089	nonDEG
Bpev01.c0387.g0005.m0002	0.014208667	0.97309245	1	nonDEG
Bpev01.c0804.g0010.m0001	-0.465405564	0.4265819	0.75867304	nonDEG
Bpev01.c0135.g0003.m0001	-0.563122116	0.23012593	0.54367043	nonDEG
Bpev01.c2261.g0001.m0001	0.59972064	0.05381888	0.21096492	nonDEG
Bpev01.c2182.g0003.m0001	0.515259305	0.2341204	0.54966667	nonDEG
Bpev01.c0275.g0054.m0001	1.875419486	0.03500283	0.15518801	nonDEG
Bpev01.c1615.g0004.m0001	0.307396162	0.38030063	0.71647704	nonDEG
Bpev01.c0052.g0063.m0001	1.135537237	0.06557767	0.2415592	nonDEG
Bpev01.c0496.g0013.m0001	2.094311198	0.00935956	0.05868712	nonDEG
Bpev01.c1849.g0001.m0001	-1.284660515	0.09610047	0.31165305	nonDEG
Bpev01.c0455.g0033.m0001	-0.042953879	0.91626624	1	nonDEG
Bpev01.c0768.g0007.m0001	0.206990197	0.53872485	0.85620693	nonDEG
Bpev01.c0134.g0036.m0001	1.016184376	0.07974392	0.27563476	nonDEG
Bpev01.c0457.g0019.m0001	0.155543838	0.61327056	0.91629674	nonDEG
Bpev01.c0324.g0006.m0001	-0.647587054	0.04025264	0.17226153	nonDEG
Bpev01.c0281.g0075.m0001	-0.353027021	0.30035918	0.63114019	nonDEG
Bpev01.c0597.g0015.m0001	-0.173553151	0.61817601	0.91944472	nonDEG
Bpev01.c0051.g0102.m0001	-0.849199705	0.00789933	0.05174643	nonDEG
Bpev01.c1154.g0008.m0001	-0.157289471	0.62725623	0.92511462	nonDEG
Bpev01.c1274.g0004.m0001	0.257708167	0.68774351	0.94707087	nonDEG
Bpev01.c1604.g0002.m0001	1.745864144	0.05122788	0.20385207	nonDEG
Bpev01.c0016.g0019.m0001	0.644332941	0.08574664	0.28898628	nonDEG
Bpev01.c0129.g0078.m0001	-0.726601134	0.01855926	0.0974957	nonDEG
Bpev01.c0800.g0016.m0001	-0.460712951	0.47127547	0.79987769	nonDEG
Bpev01.c0127.g0078.m0001	0.117910045	0.75943288	0.99346536	nonDEG
Bpev01.c0190.g0023.m0001	0.556023096	0.07809503	0.27197913	nonDEG
Bpev01.c0371.g0016.m0001	-0.033065044	0.95408799	1	nonDEG
Bpev01.c0429.g0001.m0001	-0.779569536	0.01361174	0.0777033	nonDEG
Bpev01.c0116.g0004.m0001	0.578340517	0.08924517	0.29676816	nonDEG
Bpev01.c1339.g0004.m0001	-0.244322305	0.49971913	0.8223159	nonDEG
Bpev01.c0294.g0006.m0001	-0.268592917	0.62015516	0.9208812	nonDEG
Bpev01.c0329.g0010.m0001	0.658106599	0.11700175	0.35630646	nonDEG
Bpev01.c0001.g0089.m0001	-0.745199339	0.02483175	0.12129115	nonDEG
Bpev01.c0038.g0058.m0001	0.051005312	0.87468799	1	nonDEG
Bpev01.c0223.g0025.m0001	0.602874133	0.0457901	0.18816511	nonDEG
Bpev01.c0243.g0040.m0001	-1.107178532	0.03097341	0.14192504	nonDEG
Bpev01.c0027.g0159.m0001	-0.515903434	0.15034258	0.42036822	nonDEG
Bpev01.c0044.g0110.m0001	-0.272387851	0.44622143	0.77759202	nonDEG
Bpev01.c0088.g0064.m0002	-0.012677649	0.97437983	1	nonDEG

Bpev01.c0335.g0027.m0001	0.107212303	0.76731482	0.99858455	nonDEG
Bpev01.c0991.g0004.m0001	-0.004871798	0.99345476	1	nonDEG
Bpev01.c1709.g0003.m0001	-0.553415506	0.87730698	1	nonDEG
Bpev01.c0834.g0018.m0001	-0.387340638	0.22319276	0.53280666	nonDEG
Bpev01.c0343.g0029.m0001	3.011389299	0.04407492	0.18380498	nonDEG
Bpev01.c0052.g0005.m0001	-0.077792294	0.80634525	1	nonDEG
Bpev01.c0015.g0109.m0001	0.754466133	0.36891118	0.70890185	nonDEG
Bpev01.c1723.g0005.m0001	-0.188764303	1	1	nonDEG
Bpev01.c0166.g0032.m0001	-0.252603695	0.53063544	0.84975496	nonDEG
Bpev01.c4638.g0001.m0001	0.393131739	0.70186963	0.95778705	nonDEG
Bpev01.c0353.g0019.m0001	-0.424110932	0.87312386	1	nonDEG
Bpev01.c0954.g0001.m0001	0.578595377	0.41366123	0.74687353	nonDEG
Bpev01.c1211.g0012.m0001	0.73647737	0.04602736	0.18884007	nonDEG
Bpev01.c0106.g0004.m0001	0.448676854	0.16155212	0.43984091	nonDEG
Bpev01.c1985.g0003.m0001	-0.07010249	0.93765741	1	nonDEG
Bpev01.c1158.g0009.m0001	0.949690828	0.74611311	0.98518768	nonDEG
Bpev01.c0018.g0018.m0001	-0.346050369	0.27535596	0.60196604	nonDEG
Bpev01.c0383.g0009.m0001	-0.516383688	0.1087834	0.33877864	nonDEG
Bpev01.c0636.g0009.m0001	2.182529081	0.01162952	0.06900225	nonDEG
Bpev01.c0422.g0002.m0002	0.161558749	0.74509482	0.98468642	nonDEG
Bpev01.c1402.g0011.m0001	0.903446086	0.04484355	0.18580565	nonDEG
Bpev01.c0726.g0010.m0001	-0.251248116	0.50649497	0.82857808	nonDEG
Bpev01.c0126.g0049.m0002	0.65215293	0.04364509	0.1826134	nonDEG
Bpev01.c0036.g0046.m0001	0.196308438	0.53223548	0.8509752	nonDEG
Bpev01.c0112.g0001.m0001	-0.276549858	0.35504651	0.6929798	nonDEG
Bpev01.c1587.g0002.m0001	-0.935042024	0.61689437	0.91855512	nonDEG
Bpev01.c0363.g0025.m0001	0.495765449	0.15850924	0.43485014	nonDEG
Bpev01.c0488.g0010.m0001	-0.89443449	0.01409456	0.07971751	nonDEG
Bpev01.c0944.g0005.m0001	0.137860176	0.66730033	0.93220195	nonDEG
Bpev01.c0505.g0019.m0001	-0.048935213	0.93671635	1	nonDEG
Bpev01.c0092.g0013.m0001	0.086921592	0.79988663	1	nonDEG
Bpev01.c0511.g0010.m0001	-0.002685228	0.99435046	1	nonDEG
Bpev01.c1605.g0002.m0001	1.850995141	0.07697978	0.26940177	nonDEG
Bpev01.c0161.g0099.m0001	-0.656142144	0.04382075	0.18318623	nonDEG
Bpev01.c0920.g0001.m0001	0	1	1	nonDEG
Bpev01.c3008.g0001.m0001	0.164445051	0.59680341	0.90460461	nonDEG
Bpev01.c0891.g0006.m0001	-0.013375078	0.96810236	1	nonDEG
Bpev01.c0015.g0171.m0001	0.580238997	0.09563595	0.31076911	nonDEG
Bpev01.c1209.g0003.m0001	-0.312730877	0.5085649	0.82972967	nonDEG
Bpev01.c1579.g0014.m0003	-0.92059636	0.1223553	0.36812015	nonDEG
Bpev01.c0404.g0008.m0001	-0.47679284	0.13024115	0.38291912	nonDEG
Bpev01.c0597.g0010.m0003	-0.110828634	0.75280714	0.98930826	nonDEG
Bpev01.c1974.g0006.m0001	-0.108110003	0.76430968	0.99687634	nonDEG

Bpev01.c1100.g0001.m0001	-0.23376645	0.485179	0.81256284	nonDEG
Bpev01.c1195.g0004.m0001	-0.067630967	1	1	nonDEG
Bpev01.c1379.g0008.m0001	4.199465371	0.37376199	0.7132879	nonDEG
Bpev01.c1281.g0011.m0001	0.441973116	0.24313552	0.56089085	nonDEG
Bpev01.c1776.g0002.m0001	-0.439506918	0.66530718	0.93220195	nonDEG
Bpev01.c1349.g0008.m0001	0.823064647	0.01437505	0.08091533	nonDEG
Bpev01.c0241.g0010.m0001	0.608244271	0.04773564	0.19364605	nonDEG
Bpev01.c1402.g0009.m0001	0.949301386	0.02380167	0.11752197	nonDEG
Bpev01.c0757.g0010.m0001	-0.442577077	0.22843629	0.54098109	nonDEG
Bpev01.c1583.g0001.m0001	0.693338937	0.11238356	0.3469078	nonDEG
Bpev01.c0709.g0002.m0001	-0.60629858	0.1193234	0.36154399	nonDEG
Bpev01.c1583.g0003.m0001	0.484440605	0.11601109	0.35449352	nonDEG
Bpev01.c0394.g0012.m0001	0.288148961	0.42841973	0.76046116	nonDEG
Bpev01.c1477.g0004.m0002	1.826118123	0.05003225	0.20039446	nonDEG
Bpev01.c1270.g0005.m0001	0	1	1	nonDEG
Bpev01.c0114.g0025.m0002	0.644267631	0.15182223	0.42304638	nonDEG
Bpev01.c0774.g0019.m0001	-0.797988467	0.34355009	0.680152	nonDEG
Bpev01.c1152.g0007.m0001	0.01862668	0.95237374	1	nonDEG
Bpev01.c2732.g0001.m0001	0.552934487	0.16036519	0.4379804	nonDEG
Bpev01.c0777.g0011.m0001	0.377102042	0.39998664	0.7329942	nonDEG
Bpev01.c0280.g0060.m0001	-0.887606856	0.31235745	0.64344629	nonDEG
Bpev01.c0703.g0003.m0001	0.138534265	0.83329367	1	nonDEG
Bpev01.c0257.g0032.m0001	-0.030522363	0.92640428	1	nonDEG
Bpev01.c0699.g0007.m0001	-0.879071342	0.01369775	0.07808834	nonDEG
Bpev01.c0038.g0165.m0001	0.208597382	0.51415402	0.834431	nonDEG
Bpev01.c1152.g0012.m0001	-0.549877888	0.0903146	0.29903434	nonDEG
Bpev01.c0215.g0027.m0001	-0.376356995	0.23366838	0.54908712	nonDEG
Bpev01.c0425.g0043.m0001	0.479539339	0.28092561	0.60819359	nonDEG
Bpev01.c0999.g0003.m0001	-0.27506681	0.40101207	0.73390787	nonDEG
Bpev01.c0088.g0133.m0001	-0.211350016	0.52425583	0.84368705	nonDEG
Bpev01.c0848.g0043.m0001	-0.680844946	0.02861007	0.1341	nonDEG
Bpev01.c0279.g0005.m0001	0.813125292	0.1735918	0.46084407	nonDEG
Bpev01.c0022.g0116.m0001	-0.107052842	0.84092509	1	nonDEG
Bpev01.c0932.g0001.m0001	1.117792097	0.02612196	0.12571993	nonDEG
Bpev01.c0652.g0022.m0002	0.473900087	0.14188723	0.40459302	nonDEG
Bpev01.c0022.g0097.m0001	1.167076406	0.03383482	0.15128541	nonDEG
Bpev01.c0016.g0077.m0001	-0.305560093	0.32091719	0.65430301	nonDEG
Bpev01.c0401.g0037.m0001	0.278502147	0.54647695	0.86280768	nonDEG
Bpev01.c0357.g0046.m0001	-0.672557726	0.04289913	0.18031233	nonDEG
Bpev01.c0477.g0006.m0001	0	1	1	nonDEG
Bpev01.c0815.g0009.m0001	-0.062032425	0.85492036	1	nonDEG
Bpev01.c0428.g0002.m0001	-0.50909648	0.14809863	0.41633124	nonDEG
Bpev01.c1171.g0021.m0001	-0.240275831	0.42649209	0.75859469	nonDEG

Bpev01.c1200.g0003.m0001	0.051576396	0.87253145	1	nonDEG
Bpev01.c2417.g0001.m0001	-0.962083565	0.04826739	0.19510416	nonDEG
Bpev01.c1206.g0014.m0001	0.324045137	0.44843896	0.7799595	nonDEG
Bpev01.c0594.g0009.m0001	-0.52369067	0.12419861	0.3718758	nonDEG
Bpev01.c2333.g0002.m0001	-2.203916724	0.13116648	0.384526	nonDEG
Bpev01.c0095.g0039.m0001	-0.475149398	0.16495681	0.44537374	nonDEG
Bpev01.c0872.g0001.m0001	-0.02718934	0.95779169	1	nonDEG
Bpev01.c0129.g0103.m0001	-0.305093465	0.43733944	0.7696146	nonDEG
Bpev01.c0304.g0007.m0001	-0.463994107	0.13916595	0.39973717	nonDEG
Bpev01.c0095.g0015.m0001	-0.413369655	0.2160881	0.52254127	nonDEG
Bpev01.c0726.g0006.m0001	-0.204768429	0.57471569	0.88632486	nonDEG
Bpev01.c0299.g0046.m0001	-0.69751677	0.07172614	0.25680826	nonDEG
Bpev01.c1550.g0002.m0001	0.317000509	0.42522934	0.75735534	nonDEG
Bpev01.c0381.g0002.m0001	-0.152105472	0.68846665	0.9476068	nonDEG
Bpev01.c3046.g0004.m0001	-0.226572967	0.62910416	0.92638043	nonDEG
Bpev01.c0052.g0150.m0002	-0.337879633	0.37097662	0.71046833	nonDEG
Bpev01.c0170.g0014.m0001	-0.545252508	0.08327251	0.28347127	nonDEG
Bpev01.c0237.g0032.m0001	0.547918483	0.15289427	0.42449089	nonDEG
Bpev01.c2535.g0003.m0001	-1.205353877	0.01048857	0.06410069	nonDEG
Bpev01.c0121.g0048.m0001	-0.786432065	0.84550735	1	nonDEG
Bpev01.c0996.g0028.m0001	-1.338669552	0.18778074	0.4824644	nonDEG
Bpev01.c0264.g0036.m0001	-0.826259891	0.00790947	0.05180132	nonDEG
Bpev01.c0055.g0011.m0001	-1.065040647	0.37081806	0.71043297	nonDEG
Bpev01.c1335.g0011.m0001	-1.813121447	0.03083106	0.14139828	nonDEG
Bpev01.c0641.g0004.m0001	0.07315853	0.881062	1	nonDEG
Bpev01.c1470.g0004.m0001	2.105707833	0.01858072	0.09755622	nonDEG
Bpev01.c0604.g0011.m0001	1.06468852	0.01948118	0.10093388	nonDEG
Bpev01.c1067.g0002.m0001	1.305308585	0.02426819	0.11917116	nonDEG
Bpev01.c1610.g0005.m0002	-0.750887118	0.06276717	0.23455225	nonDEG
Bpev01.c0365.g0012.m0001	0.677003053	0.04454348	0.18510949	nonDEG
Bpev01.c0427.g0008.m0001	-0.330521295	0.31604812	0.6485458	nonDEG
Bpev01.c0036.g0022.m0002	0.404007108	0.2349693	0.55111948	nonDEG
Bpev01.c0577.g0041.m0001	0.385192959	0.20608486	0.50901352	nonDEG
Bpev01.c0367.g0019.m0001	1.443587362	0.15832402	0.43461624	nonDEG
Bpev01.c2314.g0001.m0001	-0.132789271	0.6694854	0.93349977	nonDEG
Bpev01.c0395.g0042.m0001	0.866647524	0.00928415	0.05843832	nonDEG
Bpev01.c0115.g0041.m0001	-0.147804212	0.63703739	0.93006545	nonDEG
Bpev01.c0780.g0002.m0001	-0.960371936	0.00883702	0.05629802	nonDEG
Bpev01.c0765.g0001.m0001	-0.59306749	0.39058862	0.72349704	nonDEG
Bpev01.c0777.g0003.m0001	-0.385100152	0.30572675	0.63694102	nonDEG
Bpev01.c1325.g0001.m0001	-0.402223099	0.23842508	0.55501144	nonDEG
Bpev01.c0145.g0044.m0001	0.638300793	0.11025149	0.34185475	nonDEG
Bpev01.c0439.g0043.m0001	0.583177252	0.06016575	0.22777041	nonDEG

Bpev01.c1060.g0034.m0001	0.811346756	0.07492046	0.26429999	nonDEG
Bpev01.c0395.g0039.m0001	-0.004084183	0.98981592	1	nonDEG
Bpev01.c2182.g0004.m0001	0.928819501	0.07356878	0.26146339	nonDEG
Bpev01.c0022.g0084.m0001	-0.089815519	0.77724963	1	nonDEG
Bpev01.c0027.g0050.m0001	-0.409326601	0.17990643	0.47052821	nonDEG
Bpev01.c0313.g0012.m0001	-0.387359534	0.20906691	0.51392124	nonDEG
Bpev01.c1229.g0009.m0001	0.540998088	0.11809673	0.35870863	nonDEG
Bpev01.c1475.g0010.m0002	-0.27417289	0.42396614	0.75574563	nonDEG
Bpev01.c0050.g0133.m0001	0.577047253	0.09552621	0.31049973	nonDEG
Bpev01.c0517.g0020.m0001	0.724662715	0.02976537	0.13790726	nonDEG
Bpev01.c0121.g0027.m0001	3.359456902	0.06242373	0.23365444	nonDEG
Bpev01.c0796.g0008.m0001	0.735966642	0.01932631	0.10046748	nonDEG
Bpev01.c0616.g0013.m0001	-0.600268559	0.07113781	0.25554925	nonDEG
Bpev01.c2549.g0002.m0001	-0.56453459	0.10119554	0.32300722	nonDEG
Bpev01.c0000.g0174.m0001	-0.130146514	0.96786765	1	nonDEG
Bpev01.c0134.g0088.m0001	-0.193448851	0.5417517	0.85879212	nonDEG
Bpev01.c0594.g0028.m0001	0.350349769	0.33899298	0.67502803	nonDEG
Bpev01.c0817.g0015.m0001	-0.39755833	0.2297532	0.54300774	nonDEG
Bpev01.c0275.g0005.m0002	0.368966941	0.35619661	0.69425794	nonDEG
Bpev01.c0162.g0051.m0001	-1.733387006	0.02265773	0.11300945	nonDEG
Bpev01.c0042.g0031.m0001	-0.337200199	0.27130835	0.59699875	nonDEG
Bpev01.c0358.g0005.m0001	-0.54086366	0.12337919	0.37050955	nonDEG
Bpev01.c0083.g0026.m0001	-0.159364206	0.64972869	0.93220195	nonDEG
Bpev01.c1055.g0002.m0001	1.005367082	0.24486297	0.56299071	nonDEG
Bpev01.c0052.g0220.m0001	-0.565678245	0.0619634	0.23249304	nonDEG
Bpev01.c0401.g0042.m0001	0.195867931	0.5135766	0.83402369	nonDEG
Bpev01.c0082.g0028.m0001	0.563505263	0.27063612	0.59595008	nonDEG
Bpev01.c0798.g0010.m0001	0.157518095	0.61413537	0.91673132	nonDEG
Bpev01.c0799.g0004.m0001	-0.044315457	0.8896557	1	nonDEG
Bpev01.c0469.g0013.m0001	1.269743151	0.25611465	0.57617513	nonDEG
Bpev01.c1685.g0001.m0001	0.593239077	0.07868781	0.27310758	nonDEG
Bpev01.c0280.g0016.m0001	0.411963909	0.23385076	0.54925263	nonDEG
Bpev01.c0960.g0001.m0001	0.620574492	0.06116566	0.23031289	nonDEG
Bpev01.c0887.g0007.m0001	2.09068149	0.26131552	0.58342263	nonDEG
Bpev01.c0127.g0048.m0001	-0.954184659	0.46000541	0.78889079	nonDEG
Bpev01.c0473.g0009.m0001	0.906484605	0.02032451	0.10400368	nonDEG
Bpev01.c1029.g0013.m0001	0.553255271	0.10678608	0.33436241	nonDEG
Bpev01.c0349.g0017.m0001	0.419399665	0.19237014	0.48902882	nonDEG
Bpev01.c0000.g0147.m0001	-0.558948034	0.36721982	0.70672699	nonDEG
Bpev01.c0711.g0004.m0001	-1.071782602	0.12103619	0.36541732	nonDEG
Bpev01.c0704.g0002.m0001	-1.608422873	0.02253968	0.11261807	nonDEG
Bpev01.c0264.g0006.m0001	-0.551714357	0.10568443	0.33236235	nonDEG
Bpev01.c0639.g0017.m0001	0.28219185	0.37661785	0.7144404	nonDEG

Bpev01.c1188.g0001.m0001	0.242952928	0.53275999	0.85113807	nonDEG
Bpev01.c0066.g0045.m0001	0.033110163	0.93401562	1	nonDEG
Bpev01.c0131.g0005.m0001	0.265716562	0.37609345	0.71439917	nonDEG
Bpev01.c0984.g0005.m0001	0.868065247	0.01004018	0.06200399	nonDEG
Bpev01.c0367.g0002.m0001	1.591068327	0.038576	0.1668585	nonDEG
Bpev01.c0477.g0033.m0001	0.983654168	0.59321353	0.9018598	nonDEG
Bpev01.c0356.g0037.m0001	-0.503919339	0.1252306	0.37352216	nonDEG
Bpev01.c0189.g0035.m0001	-0.503918656	0.12851515	0.38000778	nonDEG
Bpev01.c0114.g0003.m0001	2.14599447	0.01722894	0.09252147	nonDEG
Bpev01.c1216.g0007.m0001	0.694936724	0.30431092	0.63589769	nonDEG
Bpev01.c0225.g0009.m0001	0.839990399	0.0106681	0.06490149	nonDEG
Bpev01.c0045.g0008.m0001	0.845090451	0.00775793	0.05094486	nonDEG
Bpev01.c0419.g0020.m0001	-0.478736301	0.19960182	0.50037919	nonDEG
Bpev01.c1719.g0001.m0001	0.231299747	0.60964316	0.91311549	nonDEG
Bpev01.c1104.g0005.m0001	0.682697412	0.21175402	0.51772074	nonDEG
Bpev01.c0083.g0002.m0001	0.536662676	0.12234479	0.36812015	nonDEG
Bpev01.c1335.g0020.m0001	-0.648994601	0.04791005	0.19413958	nonDEG
Bpev01.c0162.g0073.m0001	-0.286903251	0.37054798	0.71014661	nonDEG
Bpev01.c0883.g0001.m0001	-0.886182632	0.15713441	0.43244646	nonDEG
Bpev01.c1401.g0009.m0001	-0.021987933	0.94692444	1	nonDEG
Bpev01.c0127.g0049.m0001	2.656131812	0.30564438	0.63694102	nonDEG
Bpev01.c1662.g0004.m0002	-0.42139398	0.17631008	0.46485888	nonDEG
Bpev01.c0134.g0146.m0001	0.38453932	0.20840649	0.51302607	nonDEG
Bpev01.c0688.g0005.m0001	0.999742346	0.03225515	0.14597378	nonDEG
Bpev01.c0430.g0004.m0001	-0.27899022	0.6282466	0.92567326	nonDEG
Bpev01.c0094.g0078.m0001	1.230016926	0.52726538	0.84635582	nonDEG
Bpev01.c0018.g0048.m0001	-1.089315094	0.04616404	0.18920301	nonDEG
Bpev01.c0983.g0005.m0001	-0.204489056	0.59130785	0.90052757	nonDEG
Bpev01.c0557.g0033.m0001	1.961361659	0.10874032	0.33877864	nonDEG
Bpev01.c1343.g0004.m0001	0.526620462	0.33128363	0.66702771	nonDEG
Bpev01.c0561.g0005.m0001	0.666254467	0.40514209	0.73837541	nonDEG
Bpev01.c0485.g0002.m0001	-0.538999189	0.13912505	0.39973717	nonDEG
Bpev01.c2102.g0003.m0001	1.123016531	0.01902026	0.09926209	nonDEG
Bpev01.c0050.g0147.m0001	0.371803075	0.22956732	0.54274272	nonDEG
Bpev01.c0288.g0022.m0001	0.227629412	0.459362	0.78843039	nonDEG
Bpev01.c0142.g0089.m0001	-0.066684972	0.84093972	1	nonDEG
Bpev01.c1099.g0008.m0001	-0.528227334	0.11561453	0.35369179	nonDEG
Bpev01.c0902.g0018.m0001	-0.388024451	0.24398932	0.561748	nonDEG
Bpev01.c0018.g0115.m0001	1.449651057	0.01587211	0.08720774	nonDEG
Bpev01.c0887.g0014.m0001	0.41633777	0.91205906	1	nonDEG
Bpev01.c0036.g0039.m0001	-0.984554293	0.45488061	0.78481101	nonDEG
Bpev01.c0051.g0090.m0001	-0.55298301	0.07757154	0.27063622	nonDEG
Bpev01.c1099.g0001.m0001	-0.741700236	0.12495984	0.37290348	nonDEG

Bpev01.c1818.g0006.m0001	-0.228410907	0.60605282	0.91034513	nonDEG
Bpev01.c1067.g0006.m0001	0.462369325	0.19101816	0.48685818	nonDEG
Bpev01.c0892.g0014.m0001	0.447618454	0.54329116	0.86010127	nonDEG
Bpev01.c0480.g0084.m0001	0.069943593	0.81457815	1	nonDEG
Bpev01.c0832.g0002.m0001	0.122677219	0.68519399	0.94541741	nonDEG
Bpev01.c2776.g0001.m0001	-0.092358578	0.87636966	1	nonDEG
Bpev01.c0050.g0122.m0001	0.394825607	0.32532221	0.65930843	nonDEG
Bpev01.c0163.g0041.m0001	0.90527196	0.0104714	0.06403562	nonDEG
Bpev01.c0604.g0005.m0001	-0.250533108	0.4103891	0.7443897	nonDEG
Bpev01.c0044.g0027.m0001	-0.591606499	0.10789266	0.33697321	nonDEG
Bpev01.c0000.g0037.m0001	0.2266406	0.4524839	0.7833866	nonDEG
Bpev01.c1165.g0007.m0001	0.524807486	0.22225121	0.53172316	nonDEG
Bpev01.c1464.g0001.m0001	-0.715290062	0.03144949	0.14349107	nonDEG
Bpev01.c0425.g0020.m0001	-0.020163736	0.95005251	1	nonDEG
Bpev01.c0018.g0120.m0001	0.773261269	0.27537201	0.60196604	nonDEG
Bpev01.c0667.g0028.m0001	0.374262494	0.22643544	0.53803658	nonDEG
Bpev01.c1002.g0005.m0001	0.903619517	0.01140598	0.06819268	nonDEG
Bpev01.c0082.g0043.m0001	-0.308857426	0.42885345	0.76099568	nonDEG
Bpev01.c1558.g0007.m0001	0.638056105	0.16354633	0.44271104	nonDEG
Bpev01.c0518.g0045.m0001	-0.536832982	0.099549	0.31951842	nonDEG
Bpev01.c0768.g0011.m0001	-0.035297975	0.91220985	1	nonDEG
Bpev01.c0604.g0004.m0001	0.863979745	0.05592415	0.21656814	nonDEG
Bpev01.c0863.g0033.m0001	-0.730007813	0.10606043	0.33314116	nonDEG
Bpev01.c0634.g0005.m0001	-0.121866489	0.74136965	0.98341259	nonDEG
Bpev01.c0053.g0062.m0001	-1.545741575	0.20121779	0.50227357	nonDEG
Bpev01.c1373.g0001.m0001	0.121957878	0.72259314	0.97192029	nonDEG
Bpev01.c0281.g0038.m0002	-0.965226642	0.0163511	0.08910772	nonDEG
Bpev01.c0136.g0006.m0001	-0.965538062	0.01002043	0.06192094	nonDEG
Bpev01.c0189.g0030.m0001	-1.098445368	0.16073679	0.43862907	nonDEG
Bpev01.c1160.g0004.m0001	0.522376674	0.14954256	0.41871633	nonDEG
Bpev01.c0558.g0017.m0001	-0.153024524	0.63430926	0.92965477	nonDEG
Bpev01.c0045.g0028.m0001	0.723076066	0.02201671	0.11066243	nonDEG
Bpev01.c0379.g0016.m0001	-0.041444781	0.90646719	1	nonDEG
Bpev01.c0134.g0097.m0001	0.030112734	0.92842311	1	nonDEG
Bpev01.c0279.g0020.m0001	0.514457985	0.10064992	0.32182392	nonDEG
Bpev01.c1339.g0008.m0001	0.48778307	0.16831421	0.45123413	nonDEG
Bpev01.c0862.g0012.m0001	-0.387901396	0.31451793	0.64644929	nonDEG
Bpev01.c0177.g0019.m0001	-0.279132002	0.38707759	0.71971055	nonDEG
Bpev01.c0484.g0026.m0001	-0.819608386	0.01105878	0.06676769	nonDEG
Bpev01.c2346.g0003.m0001	-0.877359125	0.39880293	0.73203388	nonDEG
Bpev01.c0018.g0089.m0001	0.510565006	0.14561546	0.4113013	nonDEG
Bpev01.c0281.g0080.m0002	-0.202304431	0.52743158	0.84653028	nonDEG
Bpev01.c1092.g0009.m0001	0.734292586	0.02884876	0.13495608	nonDEG

Bpev01.c2286.g0003.m0001	-0.095769976	0.79362752	1	nonDEG
Bpev01.c1427.g0004.m0001	-0.187886108	0.56798139	0.88076924	nonDEG
Bpev01.c0566.g0074.m0001	0.313254666	0.32706966	0.66194168	nonDEG
Bpev01.c0887.g0017.m0002	-0.464884749	0.82820306	1	nonDEG
Bpev01.c0016.g0092.m0002	0.073020259	0.81500583	1	nonDEG
Bpev01.c1122.g0008.m0001	-0.308333424	0.31855005	0.65146197	nonDEG
Bpev01.c1365.g0001.m0001	-0.099538528	0.74257966	0.9838672	nonDEG
Bpev01.c0363.g0014.m0001	-0.614099948	0.0539348	0.2112542	nonDEG
Bpev01.c0661.g0013.m0001	-0.723322889	0.02251367	0.11252631	nonDEG
Bpev01.c0205.g0005.m0001	0.014895731	0.98170778	1	nonDEG
Bpev01.c0161.g0033.m0001	0.203992106	0.5327361	0.85113807	nonDEG
Bpev01.c0162.g0022.m0001	0.111480558	0.92466778	1	nonDEG
Bpev01.c0970.g0002.m0001	0.13179595	0.65896061	0.93220195	nonDEG
Bpev01.c0874.g0037.m0001	-0.354329643	0.25694283	0.57669851	nonDEG
Bpev01.c0142.g0052.m0001	-0.08142592	0.79282355	1	nonDEG
Bpev01.c0298.g0043.m0001	1.017745494	0.01676502	0.09072524	nonDEG
Bpev01.c2824.g0001.m0001	-0.666667464	0.5692371	0.88212722	nonDEG
Bpev01.c0671.g0016.m0001	-0.755344335	0.03300933	0.14842852	nonDEG
Bpev01.c0134.g0067.m0001	0.123388148	0.85260975	1	nonDEG
Bpev01.c0470.g0007.m0001	-1.362799427	0.01155111	0.0687949	nonDEG
Bpev01.c0177.g0034.m0001	3.036970168	0.07575652	0.26638749	nonDEG
Bpev01.c1028.g0010.m0001	0.399125967	0.22379343	0.53363477	nonDEG
Bpev01.c0080.g0042.m0001	0.305354105	0.33653914	0.67210483	nonDEG
Bpev01.c0324.g0001.m0001	-0.346339016	0.27233772	0.59821333	nonDEG
Bpev01.c0016.g0163.m0001	-0.109354462	0.72748417	0.97492493	nonDEG
Bpev01.c1777.g0001.m0001	-2.297959865	0.45417167	0.78423132	nonDEG
Bpev01.c0001.g0058.m0001	0.221867064	0.53183062	0.85057649	nonDEG
Bpev01.c0029.g0148.m0001	-0.145684233	0.62519971	0.92415465	nonDEG
Bpev01.c0598.g0005.m0001	0.088985462	0.7686401	0.99943102	nonDEG
Bpev01.c0397.g0007.m0001	3.122818776	0.01673181	0.09061215	nonDEG
Bpev01.c2627.g0003.m0001	-0.955915637	0.1818104	0.47361439	nonDEG
Bpev01.c1836.g0001.m0001	0.449436409	0.17603988	0.46454266	nonDEG
Bpev01.c1334.g0005.m0001	-0.570666593	0.08221577	0.28118944	nonDEG
Bpev01.c0994.g0004.m0001	0.550384517	0.08276602	0.28236747	nonDEG
Bpev01.c3006.g0002.m0001	0.549357209	0.11172712	0.34535224	nonDEG
Bpev01.c0274.g0040.m0001	0.641950361	0.18292493	0.47513031	nonDEG
Bpev01.c0901.g0020.m0001	0.10981409	0.81181753	1	nonDEG
Bpev01.c0152.g0009.m0001	-0.688238281	0.02584497	0.12483182	nonDEG
Bpev01.c0614.g0008.m0001	0.63202034	0.04333854	0.18168416	nonDEG
Bpev01.c0229.g0013.m0001	-0.132014893	0.67520874	0.93819182	nonDEG
Bpev01.c1153.g0008.m0001	-0.779543213	0.01938602	0.10068896	nonDEG
Bpev01.c0025.g0033.m0001	0.301481087	0.3727075	0.71251059	nonDEG
Bpev01.c3896.g0001.m0001	-0.08774293	0.86054664	1	nonDEG

Bpev01.c2549.g0004.m0001	-0.5593756	0.08536009	0.28811233	nonDEG
Bpev01.c2008.g0001.m0001	0.620689828	0.03799578	0.16488174	nonDEG
Bpev01.c0401.g0015.m0001	-2.031340155	0.09609628	0.31165305	nonDEG
Bpev01.c0841.g0003.m0003	1.118301565	0.03307657	0.14866277	nonDEG
Bpev01.c0000.g0139.m0001	1.933102733	0.00906345	0.05742984	nonDEG
Bpev01.c0280.g0023.m0001	-1.057088214	0.02678154	0.12767966	nonDEG
Bpev01.c1764.g0004.m0001	1.32843037	0.14929495	0.41814234	nonDEG
Bpev01.c0452.g0032.m0001	0.205194607	0.55593342	0.87160484	nonDEG
Bpev01.c1828.g0003.m0001	0.416078068	0.2807897	0.60806006	nonDEG
Bpev01.c0243.g0028.m0001	-1.202130177	0.29726411	0.62741942	nonDEG
Bpev01.c1352.g0007.m0001	0.119891999	0.73282492	0.97781941	nonDEG
Bpev01.c0774.g0001.m0002	-0.800477508	0.38985906	0.72301001	nonDEG
Bpev01.c0129.g0066.m0001	0.114512372	0.70484618	0.95975931	nonDEG
Bpev01.c1525.g0003.m0001	-1.336394828	0.09362119	0.30626895	nonDEG
Bpev01.c0718.g0007.m0001	-0.63949796	0.04701926	0.19150528	nonDEG
Bpev01.c0597.g0025.m0001	1.137746319	0.01481186	0.08277263	nonDEG
Bpev01.c0658.g0005.m0001	-0.62200418	0.05783712	0.22178807	nonDEG
Bpev01.c0447.g0008.m0001	0.019910006	0.96888501	1	nonDEG
Bpev01.c0254.g0006.m0001	-0.132172538	0.73268432	0.9777649	nonDEG
Bpev01.c0080.g0018.m0001	-0.594830443	0.08135268	0.27948903	nonDEG
Bpev01.c0129.g0084.m0001	0.375081899	0.22634714	0.53791546	nonDEG
Bpev01.c1047.g0001.m0001	-0.733559762	0.02651398	0.12699048	nonDEG
Bpev01.c0753.g0014.m0001	-0.570236152	0.08817063	0.29421206	nonDEG
Bpev01.c0115.g0017.m0002	1.164517134	0.01506237	0.0838273	nonDEG
Bpev01.c1378.g0032.m0001	0.663910676	0.03568494	0.15728282	nonDEG
Bpev01.c1031.g0022.m0001	0.020154711	0.95642609	1	nonDEG
Bpev01.c0016.g0101.m0001	-0.927080976	0.01302908	0.07534843	nonDEG
Bpev01.c0840.g0009.m0001	-0.734407679	0.04779197	0.19382116	nonDEG
Bpev01.c0504.g0016.m0001	-0.765860345	0.0264807	0.12692086	nonDEG
Bpev01.c1044.g0013.m0001	1.964400783	0.01367076	0.07796462	nonDEG
Bpev01.c1435.g0002.m0001	-0.071752297	0.8249672	1	nonDEG
Bpev01.c0634.g0022.m0001	0.06279838	0.84124341	1	nonDEG
Bpev01.c0464.g0024.m0001	-0.341671103	0.37995104	0.71647215	nonDEG
Bpev01.c1290.g0005.m0001	0.65775183	0.28576428	0.6132653	nonDEG
Bpev01.c0018.g0100.m0001	0.315708589	0.31468076	0.64669367	nonDEG
Bpev01.c0617.g0017.m0001	-0.056319002	0.88380893	1	nonDEG
Bpev01.c0939.g0003.m0001	0.803767717	0.07855179	0.27283371	nonDEG
Bpev01.c1779.g0002.m0003	0.5293648	0.20135589	0.50239138	nonDEG
Bpev01.c0379.g0018.m0001	-0.292035916	0.45431201	0.78430652	nonDEG
Bpev01.c0088.g0112.m0001	0.816274769	0.20782187	0.51209516	nonDEG
Bpev01.c1047.g0009.m0001	-0.93196604	0.02234099	0.11185313	nonDEG
Bpev01.c0438.g0003.m0001	-0.667083585	0.3878494	0.72055747	nonDEG
Bpev01.c0478.g0009.m0001	-0.503133542	0.10027825	0.32126361	nonDEG

Bpev01.c0817.g0028.m0001	0.957336064	0.03405615	0.15197497	nonDEG
Bpev01.c0329.g0002.m0001	-0.23670612	0.46498266	0.7937259	nonDEG
Bpev01.c1702.g0006.m0002	0.229616153	0.53463878	0.8526477	nonDEG
Bpev01.c0605.g0006.m0001	0.59973989	0.41229018	0.74599553	nonDEG
Bpev01.c0501.g0004.m0001	-0.436301337	0.21792502	0.52517146	nonDEG
Bpev01.c1566.g0001.m0001	0.935132181	0.03240105	0.14639377	nonDEG
Bpev01.c0480.g0037.m0001	1.455268238	0.04493466	0.18602608	nonDEG
Bpev01.c1031.g0019.m0001	-0.480618635	0.11958095	0.36210212	nonDEG
Bpev01.c0668.g0009.m0001	-0.343276633	0.30989428	0.64011911	nonDEG
Bpev01.c0261.g0016.m0001	0.25139586	0.68988633	0.94881857	nonDEG
Bpev01.c0732.g0010.m0001	0.260465024	0.93311379	1	nonDEG
Bpev01.c0000.g0242.m0001	-0.814741244	0.0088416	0.05631496	nonDEG
Bpev01.c1663.g0008.m0001	-0.089127291	0.77553494	1	nonDEG
Bpev01.c0237.g0037.m0001	-0.306151626	0.35475844	0.69283096	nonDEG
Bpev01.c0036.g0027.m0001	-0.281833542	0.46402374	0.79287218	nonDEG
Bpev01.c0523.g0005.m0001	-0.90142783	0.01280366	0.0743886	nonDEG
Bpev01.c0155.g0025.m0001	0.677953872	0.02502822	0.12204831	nonDEG
Bpev01.c0504.g0013.m0001	1.455820089	0.1013054	0.3232177	nonDEG
Bpev01.c0732.g0016.m0001	0.199242099	0.57740274	0.88873134	nonDEG
Bpev01.c0045.g0049.m0001	1.139991494	0.02331822	0.11558596	nonDEG
Bpev01.c0279.g0014.m0002	-0.243571397	0.49208742	0.8160767	nonDEG
Bpev01.c0444.g0015.m0001	-0.01902326	0.9592219	1	nonDEG
Bpev01.c1352.g0003.m0001	0.893114394	0.65808405	0.93220195	nonDEG
Bpev01.c0823.g0004.m0001	-0.314752023	0.29601714	0.62617293	nonDEG
Bpev01.c1104.g0006.m0001	0.085388939	0.78300898	1	nonDEG
Bpev01.c0383.g0001.m0001	0.418718132	0.20862265	0.51334243	nonDEG
Bpev01.c0994.g0005.m0001	-0.827543116	0.01846814	0.09726004	nonDEG
Bpev01.c2265.g0003.m0001	0.018840124	0.97181216	1	nonDEG
Bpev01.c0281.g0069.m0001	-0.306066143	0.32337446	0.65717388	nonDEG
Bpev01.c0279.g0013.m0001	-0.153024803	0.61078047	0.91412132	nonDEG
Bpev01.c0050.g0161.m0001	-0.639357465	0.05014178	0.20072394	nonDEG
Bpev01.c0679.g0017.m0001	1.319345054	0.01866167	0.09780683	nonDEG
Bpev01.c1659.g0001.m0001	-0.541806695	0.08818667	0.29421206	nonDEG
Bpev01.c0718.g0006.m0001	-0.754153964	0.04764283	0.19332286	nonDEG
Bpev01.c0568.g0003.m0001	0.74748455	0.01688231	0.09107089	nonDEG
Bpev01.c1017.g0003.m0001	1.070906956	0.06214282	0.23298807	nonDEG
Bpev01.c0692.g0003.m0001	-0.546004711	0.07625547	0.26766242	nonDEG
Bpev01.c1503.g0011.m0001	0.749690079	0.07220526	0.25796729	nonDEG
Bpev01.c0279.g0011.m0001	0.647345097	0.05662138	0.21852083	nonDEG
Bpev01.c0744.g0015.m0001	-0.466972241	0.12860709	0.38008875	nonDEG
Bpev01.c0056.g0006.m0001	-0.844875691	0.01085582	0.06578521	nonDEG
Bpev01.c0263.g0006.m0001	0.453034422	0.16664378	0.44851218	nonDEG
Bpev01.c0142.g0088.m0001	-0.771143614	0.01113014	0.06707474	nonDEG

Bpev01.c0027.g0125.m0001	-0.146395788	0.87946726	1	nonDEG
Bpev01.c1188.g0005.m0001	0.586414986	0.08444487	0.28610572	nonDEG
Bpev01.c1228.g0002.m0001	1.297437196	0.02227362	0.11166777	nonDEG
Bpev01.c0390.g0005.m0001	0.151592899	0.62895581	0.92638043	nonDEG
Bpev01.c0169.g0016.m0001	0.151908915	0.62224827	0.92218923	nonDEG
Bpev01.c1670.g0002.m0001	0.03691213	0.9019218	1	nonDEG
Bpev01.c0477.g0016.m0001	-0.383551044	0.23965529	0.55649252	nonDEG
Bpev01.c1000.g0014.m0001	-0.765002595	0.03951734	0.16978271	nonDEG
Bpev01.c0957.g0002.m0001	-0.610848082	0.04130278	0.17532301	nonDEG
Bpev01.c0214.g0027.m0001	0.211071765	0.49451573	0.8183093	nonDEG
Bpev01.c0083.g0019.m0001	0.531323881	0.18498001	0.47839994	nonDEG
Bpev01.c0470.g0027.m0001	-1.099494851	0.06992178	0.2525058	nonDEG
Bpev01.c1393.g0001.m0001	0.427997655	0.18513302	0.47862747	nonDEG
Bpev01.c0189.g0062.m0001	0.525995538	0.2182412	0.52576127	nonDEG
Bpev01.c1275.g0002.m0001	0.878159521	0.01094632	0.06622457	nonDEG
Bpev01.c0106.g0015.m0001	0.639052658	0.37837882	0.71610964	nonDEG
Bpev01.c0395.g0007.m0001	-0.094439283	0.76924977	0.99976789	nonDEG
Bpev01.c0298.g0043.m0002	0.758988412	0.10941313	0.34002039	nonDEG
Bpev01.c0522.g0011.m0001	0.019602297	0.94826188	1	nonDEG
Bpev01.c0052.g0181.m0001	0.788278629	0.04335101	0.18169283	nonDEG
Bpev01.c1534.g0001.m0001	0.794271219	0.01338439	0.07679719	nonDEG
Bpev01.c0707.g0005.m0001	-0.674771602	0.05805575	0.22233685	nonDEG
Bpev01.c1307.g0004.m0001	0.514042113	0.09263331	0.30417435	nonDEG
Bpev01.c1273.g0002.m0001	-1.300042938	0.32180836	0.65516711	nonDEG
Bpev01.c1126.g0005.m0001	0.580642499	0.06899992	0.25031282	nonDEG
Bpev01.c0574.g0032.m0001	0.739796044	0.04073041	0.17360107	nonDEG
Bpev01.c0045.g0079.m0001	-0.580077405	0.08817994	0.29421206	nonDEG
Bpev01.c0328.g0014.m0001	-0.812391503	0.13034131	0.38302235	nonDEG
Bpev01.c0439.g0040.m0001	0.738576444	0.02019362	0.10354989	nonDEG
Bpev01.c1981.g0011.m0001	0.085162582	0.82608803	1	nonDEG
Bpev01.c0050.g0006.m0001	-0.568618912	0.06139318	0.23094223	nonDEG
Bpev01.c0327.g0056.m0001	0.303262716	0.50651529	0.82857808	nonDEG
Bpev01.c2346.g0002.m0001	0.66788333	0.02596938	0.12526832	nonDEG
Bpev01.c0949.g0002.m0001	0.177302664	0.66706606	0.93220195	nonDEG
Bpev01.c1250.g0008.m0001	-0.458038156	0.24691447	0.56609718	nonDEG
Bpev01.c0962.g0030.m0001	-0.010277811	0.97415165	1	nonDEG
Bpev01.c0280.g0046.m0001	-0.082950072	0.80812361	1	nonDEG
Bpev01.c1105.g0003.m0001	0.105108003	0.89301887	1	nonDEG
Bpev01.c0214.g0008.m0001	-0.132769686	0.83068455	1	nonDEG
Bpev01.c0304.g0019.m0002	-0.343835254	0.3201205	0.65349333	nonDEG
Bpev01.c0622.g0017.m0001	-0.484108435	0.31276997	0.64387301	nonDEG
Bpev01.c1221.g0005.m0002	0.146077825	0.67177542	0.93558504	nonDEG
Bpev01.c2388.g0003.m0001	-0.695431917	0.02017135	0.10350537	nonDEG

Bpev01.c0332.g0010.m0001	0.352565624	0.23774331	0.55476579	nonDEG
Bpev01.c1130.g0005.m0002	0.379869953	0.35910354	0.69738434	nonDEG
Bpev01.c0397.g0006.m0001	-1.169599866	0.64035017	0.93106479	nonDEG
Bpev01.c0051.g0033.m0001	-0.034787821	0.92206368	1	nonDEG
Bpev01.c0155.g0020.m0001	0.493402193	0.1348535	0.39139821	nonDEG
Bpev01.c0022.g0021.m0002	0.058598718	0.88575683	1	nonDEG
Bpev01.c0114.g0024.m0001	0.063332935	0.84016468	1	nonDEG
Bpev01.c0089.g0054.m0001	-0.497377681	0.10140243	0.32345717	nonDEG
Bpev01.c0115.g0099.m0001	-0.11892353	0.73333323	0.97781941	nonDEG
Bpev01.c0169.g0051.m0001	-0.1274852	0.69960207	0.95597778	nonDEG
Bpev01.c1854.g0006.m0001	0.748359792	0.36824224	0.70803565	nonDEG
Bpev01.c0265.g0021.m0001	0.493116641	0.1086414	0.3386947	nonDEG
Bpev01.c0439.g0041.m0001	0.098072664	0.93242807	1	nonDEG
Bpev01.c0962.g0013.m0001	-0.34158287	0.45589211	0.78548539	nonDEG
Bpev01.c0962.g0012.m0001	-0.025983946	0.94789649	1	nonDEG
Bpev01.c3320.g0002.m0001	0.396155011	0.21299562	0.51941502	nonDEG
Bpev01.c0429.g0003.m0001	-0.026850347	0.93576734	1	nonDEG
Bpev01.c0052.g0135.m0001	0.56273381	0.08526735	0.28789833	nonDEG
Bpev01.c1537.g0006.m0001	-0.242991367	0.45168433	0.78329024	nonDEG
Bpev01.c0120.g0018.m0001	-0.431424286	0.20493642	0.50736887	nonDEG
Bpev01.c0018.g0117.m0001	-0.425677183	0.21351742	0.52031714	nonDEG
Bpev01.c0142.g0063.m0001	-0.650589161	0.04736365	0.19253502	nonDEG
Bpev01.c0488.g0006.m0001	-1.033668699	0.03690217	0.16144493	nonDEG
Bpev01.c0186.g0001.m0001	0.837561973	0.06846747	0.24914879	nonDEG
Bpev01.c0468.g0011.m0001	-0.353348866	0.84147342	1	nonDEG
Bpev01.c0789.g0017.m0001	-0.227460108	0.56845887	0.8813503	nonDEG
Bpev01.c1047.g0007.m0001	-0.127937808	0.66838307	0.93276707	nonDEG
Bpev01.c0395.g0050.m0001	0.004574349	0.98871876	1	nonDEG
Bpev01.c0278.g0021.m0001	-0.609556311	0.08710101	0.29196616	nonDEG
Bpev01.c3071.g0001.m0001	-0.463729946	0.16978755	0.45415008	nonDEG
Bpev01.c0127.g0073.m0002	0.81478022	0.0307939	0.14124986	nonDEG
Bpev01.c0026.g0014.m0001	0.519403118	0.16259013	0.44104814	nonDEG
Bpev01.c0435.g0006.m0001	0.105093074	0.80491372	1	nonDEG
Bpev01.c1799.g0001.m0001	0.953712622	0.04966112	0.19936913	nonDEG
Bpev01.c0320.g0004.m0001	-0.423945617	0.16896829	0.45245455	nonDEG
Bpev01.c0699.g0022.m0001	0.62529588	0.04641314	0.19000926	nonDEG
Bpev01.c0089.g0002.m0001	-0.273626495	0.37969269	0.71647215	nonDEG
Bpev01.c0155.g0028.m0002	-0.51361198	0.13464951	0.3909989	nonDEG
Bpev01.c0428.g0008.m0001	-0.638963296	0.04216071	0.17802195	nonDEG
Bpev01.c0732.g0012.m0001	-0.751316565	0.03370586	0.15091512	nonDEG
Bpev01.c0284.g0014.m0001	-0.196405543	0.62666114	0.92461009	nonDEG
Bpev01.c0281.g0019.m0001	-0.883448416	0.01160605	0.06895472	nonDEG
Bpev01.c0445.g0009.m0001	-0.789460899	0.01760287	0.09386179	nonDEG

Bpev01.c1638.g0012.m0001	-0.334079738	0.28407116	0.61104566	nonDEG
Bpev01.c1072.g0008.m0001	-0.724779972	0.02723458	0.12935226	nonDEG
Bpev01.c0574.g0019.m0001	0.41367398	0.17949772	0.46991849	nonDEG
Bpev01.c0210.g0043.m0002	-0.018344388	0.95866712	1	nonDEG
Bpev01.c0001.g0018.m0001	4.703161342	0.1488572	0.41731333	nonDEG
Bpev01.c0442.g0007.m0001	0.442583855	0.17730101	0.46603255	nonDEG
Bpev01.c0757.g0005.m0001	-0.601055407	0.45707491	0.7864766	nonDEG
Bpev01.c1520.g0010.m0001	-0.513198174	0.11024222	0.34185475	nonDEG
Bpev01.c0015.g0245.m0001	-0.379213783	0.23560632	0.55179113	nonDEG
Bpev01.c0129.g0053.m0001	-0.174149298	0.62228588	0.92219854	nonDEG
Bpev01.c0027.g0136.m0001	-0.365899819	0.33282276	0.66843835	nonDEG
Bpev01.c1378.g0014.m0001	-0.617380204	0.0654748	0.2413778	nonDEG
Bpev01.c1047.g0005.m0001	1.247892978	0.41369123	0.7468819	nonDEG
Bpev01.c0572.g0014.m0002	0.553980313	0.13655244	0.39479041	nonDEG
Bpev01.c3424.g0003.m0001	-0.059626614	0.86755427	1	nonDEG
Bpev01.c0328.g0001.m0001	0.819069657	0.0332032	0.14911807	nonDEG
Bpev01.c3071.g0002.m0001	0.898998725	0.26339305	0.58605003	nonDEG
Bpev01.c0043.g0029.m0001	0.397725794	0.27932326	0.60623691	nonDEG
Bpev01.c0055.g0021.m0001	-0.416789705	0.20269228	0.50454753	nonDEG
Bpev01.c0145.g0022.m0001	0.358832914	0.30631601	0.63735239	nonDEG
Bpev01.c0038.g0163.m0001	0.857715721	0.55992613	0.87478557	nonDEG
Bpev01.c0962.g0007.m0001	0.310227498	0.34004884	0.67617056	nonDEG
Bpev01.c0834.g0013.m0001	0.206997669	0.51015522	0.83085083	nonDEG
Bpev01.c0604.g0006.m0001	0.424937886	0.17575578	0.46412579	nonDEG
Bpev01.c0045.g0033.m0001	0.313739781	0.30880906	0.63895347	nonDEG
Bpev01.c1568.g0001.m0001	0.81373987	0.08160772	0.28000577	nonDEG
Bpev01.c0142.g0027.m0001	1.722776099	0.01066116	0.0648769	nonDEG
Bpev01.c1901.g0004.m0002	-0.214113994	0.48474269	0.81256284	nonDEG
Bpev01.c0353.g0033.m0001	-0.491365907	0.11565944	0.35379243	nonDEG
Bpev01.c0120.g0040.m0001	-1.01287447	0.33303044	0.66867298	nonDEG
Bpev01.c0096.g0020.m0001	0.163447723	0.66656171	0.93220195	nonDEG
Bpev01.c1414.g0004.m0002	0.775772646	0.04395731	0.18339843	nonDEG
Bpev01.c0038.g0155.m0003	1.0863426	0.06551203	0.2413778	nonDEG
Bpev01.c0803.g0019.m0001	-0.268222804	0.41845198	0.75010634	nonDEG
Bpev01.c1260.g0011.m0002	-0.208163855	0.49082486	0.81496859	nonDEG
Bpev01.c0857.g0008.m0001	-0.296951957	0.33331119	0.66901246	nonDEG
Bpev01.c0781.g0018.m0001	-0.01780506	0.95706721	1	nonDEG
Bpev01.c0411.g0008.m0001	-1.047772454	0.38732267	0.72002981	nonDEG
Bpev01.c1517.g0005.m0001	0.854791266	0.01435267	0.08083568	nonDEG
Bpev01.c2233.g0003.m0001	0.269555584	0.44610738	0.77757808	nonDEG
Bpev01.c2630.g0001.m0002	0.077162225	0.83538539	1	nonDEG
Bpev01.c0841.g0007.m0002	0.374844977	0.30362891	0.63547074	nonDEG
Bpev01.c0797.g0011.m0001	0.84005804	0.05687517	0.21915048	nonDEG

Bpev01.c0794.g0015.m0001	-0.596726606	0.30959438	0.63976899	nonDEG
Bpev01.c0606.g0016.m0001	1.014701691	0.55150286	0.8671523	nonDEG
Bpev01.c1200.g0005.m0001	-0.12256662	0.69582357	0.95342566	nonDEG
Bpev01.c0027.g0168.m0001	-0.715688209	0.02323416	0.11524692	nonDEG
Bpev01.c0558.g0010.m0001	-0.356317673	0.31912328	0.65210005	nonDEG
Bpev01.c0932.g0007.m0001	0.109271081	0.73107005	0.97666405	nonDEG
Bpev01.c0433.g0002.m0001	1.85135014	0.14903197	0.41764417	nonDEG
Bpev01.c0557.g0051.m0001	-0.545507112	0.17870695	0.4684732	nonDEG
Bpev01.c0168.g0010.m0001	-0.322684805	0.58604879	0.89601092	nonDEG
Bpev01.c0344.g0012.m0001	-0.505936823	0.13140613	0.38498567	nonDEG
Bpev01.c0301.g0003.m0001	-0.660682857	0.84031501	1	nonDEG
Bpev01.c0115.g0118.m0002	-0.261655365	0.41896754	0.75065337	nonDEG
Bpev01.c0518.g0050.m0001	-0.511924832	0.15525183	0.42854745	nonDEG
Bpev01.c1670.g0006.m0001	-0.084446802	0.78830605	1	nonDEG
Bpev01.c1188.g0002.m0001	-0.030803101	0.91999052	1	nonDEG
Bpev01.c0001.g0002.m0001	-0.840880969	0.0302204	0.13942302	nonDEG
Bpev01.c0951.g0009.m0001	-1.522226366	0.06935081	0.25109686	nonDEG
Bpev01.c0053.g0027.m0001	-0.749779994	0.01391314	0.07896453	nonDEG
Bpev01.c0029.g0139.m0001	0.634335891	0.49352763	0.81733574	nonDEG
Bpev01.c0327.g0051.m0001	-0.065440039	0.83755643	1	nonDEG
Bpev01.c1067.g0009.m0001	-0.244156433	0.47805319	0.8066433	nonDEG
Bpev01.c1297.g0002.m0001	0.478445701	0.12859626	0.38008875	nonDEG
Bpev01.c1649.g0011.m0001	-0.711793634	0.02837563	0.13331475	nonDEG
Bpev01.c0166.g0021.m0001	0.205134134	0.58938959	0.89892447	nonDEG
Bpev01.c0458.g0018.m0001	0.429912929	0.16968926	0.4539284	nonDEG
Bpev01.c0778.g0007.m0001	0.827398538	0.01015149	0.06253393	nonDEG
Bpev01.c1407.g0004.m0001	3.277053157	0.0144424	0.08118574	nonDEG
Bpev01.c0579.g0009.m0001	0.36575709	0.29445667	0.62439747	nonDEG
Bpev01.c1221.g0001.m0001	2.555163458	0.0132489	0.07625303	nonDEG
Bpev01.c0366.g0003.m0001	0.154267413	0.6139578	0.91663639	nonDEG
Bpev01.c0029.g0124.m0001	-0.177655541	0.6262554	0.92459054	nonDEG
Bpev01.c0496.g0002.m0001	0.165432808	0.5883996	0.89820565	nonDEG
Bpev01.c0402.g0018.m0001	-0.021926728	0.9426623	1	nonDEG
Bpev01.c0449.g0025.m0001	-0.648303658	0.03879549	0.16756169	nonDEG
Bpev01.c0337.g0016.m0001	0.057369442	0.86394773	1	nonDEG
Bpev01.c0807.g0003.m0001	-0.717412966	0.0278857	0.13168547	nonDEG
Bpev01.c0540.g0010.m0001	-0.598895949	0.14298343	0.40649822	nonDEG
Bpev01.c0937.g0001.m0001	1.292605795	0.02883536	0.13495388	nonDEG
Bpev01.c0151.g0004.m0002	-0.719613482	0.03436838	0.15302036	nonDEG
Bpev01.c1062.g0009.m0001	0.847423564	0.02066288	0.10540494	nonDEG
Bpev01.c0558.g0005.m0001	0.016555832	0.95884078	1	nonDEG
Bpev01.c0275.g0018.m0001	-0.047751461	0.93425715	1	nonDEG
Bpev01.c0053.g0016.m0002	0.598820544	0.06344389	0.23612196	nonDEG

Bpev01.c0243.g0059.m0001	-0.23577561	0.45073867	0.78224935	nonDEG
Bpev01.c0671.g0009.m0001	0.542886485	0.19928393	0.4998483	nonDEG
Bpev01.c0565.g0004.m0001	1.001497369	0.02304831	0.11451798	nonDEG
Bpev01.c0874.g0013.m0001	0.918557213	0.74181262	0.98341259	nonDEG
Bpev01.c0787.g0004.m0001	3.315490154	0.00804837	0.05245427	nonDEG
Bpev01.c2235.g0007.m0002	-0.20035495	0.55873008	0.87394161	nonDEG
Bpev01.c0759.g0009.m0002	-0.355875625	0.42047249	0.75202246	nonDEG
Bpev01.c0073.g0008.m0001	-0.325373678	0.29808346	0.62860165	nonDEG
Bpev01.c1156.g0004.m0001	0.627746884	0.09217185	0.3032801	nonDEG
Bpev01.c0029.g0114.m0001	-0.803573461	0.06249182	0.23379291	nonDEG
Bpev01.c1446.g0009.m0001	0.680277721	0.05369963	0.21055721	nonDEG
Bpev01.c0532.g0016.m0001	1.352591032	0.08125352	0.2792784	nonDEG
Bpev01.c0419.g0014.m0001	0.229079514	0.68708145	0.94667011	nonDEG
Bpev01.c0052.g0078.m0001	1.075781404	0.21150574	0.51760985	nonDEG
Bpev01.c1036.g0001.m0001	0.799126415	0.0112778	0.06768749	nonDEG
Bpev01.c0451.g0023.m0001	-0.201403855	0.5369979	0.8545695	nonDEG
Bpev01.c0118.g0036.m0001	0.207941799	0.48586939	0.81279174	nonDEG
Bpev01.c0015.g0241.m0001	-0.416984112	0.18040578	0.47141539	nonDEG
Bpev01.c0796.g0011.m0001	-0.158611909	0.62806611	0.92548249	nonDEG
Bpev01.c1420.g0007.m0001	0.483015314	0.14331112	0.40707603	nonDEG
Bpev01.c0606.g0010.m0001	0.555557004	0.11796498	0.35839872	nonDEG
Bpev01.c0052.g0066.m0001	0.092753378	0.77747298	1	nonDEG
Bpev01.c0427.g0021.m0001	0.354275437	0.65056795	0.93220195	nonDEG
Bpev01.c0702.g0003.m0001	-0.304461623	0.32777463	0.66278354	nonDEG
Bpev01.c0015.g0008.m0001	-0.388860729	0.23678932	0.55350492	nonDEG
Bpev01.c0811.g0006.m0001	-0.42749698	0.90195916	1	nonDEG
Bpev01.c2783.g0002.m0001	-0.305880057	0.39131638	0.72420714	nonDEG
Bpev01.c0215.g0030.m0001	-0.839765575	0.00940214	0.0588789	nonDEG
Bpev01.c0327.g0039.m0001	0.073682567	0.866702	1	nonDEG
Bpev01.c0629.g0002.m0001	-0.890412281	0.2017808	0.50302522	nonDEG
Bpev01.c0870.g0013.m0001	-0.308917009	0.31104104	0.64154257	nonDEG
Bpev01.c0016.g0169.m0001	-0.386118972	0.20264374	0.50449293	nonDEG
Bpev01.c1058.g0007.m0001	-1.235771932	0.72659179	0.97418914	nonDEG
Bpev01.c0129.g0102.m0001	-0.89776973	0.01194754	0.07051393	nonDEG
Bpev01.c0477.g0008.m0001	-0.592222825	0.15225324	0.4236468	nonDEG
Bpev01.c1552.g0002.m0001	-0.132912258	0.73768427	0.98087419	nonDEG
Bpev01.c0045.g0039.m0001	-0.285013242	0.50631273	0.82845143	nonDEG
Bpev01.c0866.g0018.m0001	0.250944752	0.46190418	0.79073189	nonDEG
Bpev01.c0659.g0002.m0001	0.157235132	0.63171531	0.92823022	nonDEG
Bpev01.c0159.g0013.m0001	0	1	1	nonDEG
Bpev01.c0367.g0005.m0001	0.721237858	0.15215009	0.42348345	nonDEG
Bpev01.c0480.g0082.m0001	0.241003358	0.42015262	0.7516787	nonDEG
Bpev01.c0261.g0039.m0001	0.82139607	0.05820193	0.22260707	nonDEG

Bpev01.c1524.g0001.m0001	-0.341104044	0.40681477	0.7401409	nonDEG
Bpev01.c1371.g0013.m0001	-0.145984385	0.64117435	0.93184893	nonDEG
Bpev01.c0817.g0026.m0001	-0.315719962	0.46424206	0.7930611	nonDEG
Bpev01.c0605.g0002.m0001	0.066620641	0.87127142	1	nonDEG
Bpev01.c0038.g0143.m0001	-0.17028894	0.58955467	0.89908309	nonDEG
Bpev01.c0265.g0013.m0001	1.587715943	0.13180113	0.3855802	nonDEG
Bpev01.c0717.g0014.m0001	0.27625509	0.42289685	0.75447915	nonDEG
Bpev01.c1117.g0007.m0001	0.927068195	0.74080646	0.98325525	nonDEG
Bpev01.c0036.g0050.m0001	0.61789519	0.06707717	0.24536343	nonDEG
Bpev01.c0015.g0224.m0001	-0.171329511	0.66132981	0.93220195	nonDEG
Bpev01.c0980.g0005.m0001	0	1	1	nonDEG
Bpev01.c0467.g0011.m0001	-0.831741869	0.01124313	0.06751731	nonDEG
Bpev01.c0215.g0036.m0001	0.134434736	0.65643708	0.93220195	nonDEG
Bpev01.c0036.g0057.m0001	0.113913013	0.74468845	0.98468642	nonDEG
Bpev01.c0427.g0022.m0001	1.177918238	0.00826597	0.05350522	nonDEG
Bpev01.c0445.g0020.m0001	-0.297157211	0.33971492	0.67577996	nonDEG
Bpev01.c0018.g0019.m0001	-0.542009427	0.15211027	0.42348345	nonDEG
Bpev01.c1206.g0002.m0001	-0.407422152	0.24319399	0.56094868	nonDEG
Bpev01.c0184.g0003.m0001	0.675288364	0.25588331	0.57598389	nonDEG
Bpev01.c1159.g0016.m0001	2.260010053	0.24294036	0.56068533	nonDEG
Bpev01.c0419.g0028.m0001	-0.639990606	0.09792604	0.31575518	nonDEG
Bpev01.c0387.g0002.m0001	0.003625767	0.99208212	1	nonDEG
Bpev01.c0614.g0013.m0001	-0.547888354	0.11713662	0.35645905	nonDEG
Bpev01.c1634.g0004.m0001	-0.572434159	0.07179475	0.25699971	nonDEG
Bpev01.c0015.g0080.m0001	0.180310773	0.62147736	0.92162607	nonDEG
Bpev01.c0350.g0022.m0001	-0.337847944	0.33134713	0.66704353	nonDEG
Bpev01.c0470.g0037.m0001	-0.455725999	0.15134921	0.42216878	nonDEG
Bpev01.c5491.g0001.m0001	0.343618177	0.69710373	0.95431661	nonDEG
Bpev01.c0821.g0001.m0001	1.315993864	0.02217727	0.11131741	nonDEG
Bpev01.c0155.g0027.m0001	0.528300703	0.11110631	0.34375814	nonDEG
Bpev01.c1072.g0012.m0001	0.845896395	0.02719703	0.1292836	nonDEG
Bpev01.c0571.g0009.m0001	-0.135053014	0.66639236	0.93220195	nonDEG
Bpev01.c1134.g0008.m0001	-0.037168552	0.90178681	1	nonDEG
Bpev01.c0892.g0004.m0001	1.287462713	0.01460448	0.08194039	nonDEG
Bpev01.c1078.g0011.m0001	0.948962779	0.04522339	0.18659163	nonDEG
Bpev01.c0157.g0005.m0001	-0.886653834	0.43326974	0.76506881	nonDEG
Bpev01.c0379.g0006.m0001	-0.060248437	0.842998	1	nonDEG
Bpev01.c0189.g0010.m0001	0.26437998	0.45841314	0.78768685	nonDEG
Bpev01.c0344.g0014.m0001	-0.211269154	0.62501769	0.92415465	nonDEG
Bpev01.c0000.g0077.m0001	0.51012088	0.10668795	0.33419734	nonDEG
Bpev01.c2131.g0002.m0001	-0.025823562	0.95202593	1	nonDEG
Bpev01.c0883.g0013.m0001	-0.563422611	0.0669098	0.24493379	nonDEG
Bpev01.c0413.g0001.m0001	0.461823996	0.28084859	0.6081128	nonDEG

Bpev01.c0866.g0013.m0001	-0.744113918	0.0538593	0.21107081	nonDEG
Bpev01.c0603.g0003.m0002	1.060119387	0.0333707	0.14965256	nonDEG
Bpev01.c0183.g0003.m0001	-0.251888356	0.57480994	0.88637734	nonDEG
Bpev01.c0828.g0013.m0001	0.097255144	0.76473376	0.99690019	nonDEG
Bpev01.c0018.g0024.m0001	0.208229289	0.52673928	0.84589946	nonDEG
Bpev01.c0540.g0029.m0001	0.778563903	0.01120734	0.06736075	nonDEG
Bpev01.c0135.g0011.m0001	-0.308402488	0.48879355	0.81435641	nonDEG
Bpev01.c1422.g0003.m0002	-0.987990853	0.00930813	0.05852393	nonDEG
Bpev01.c1307.g0003.m0001	0.393076221	0.28733261	0.61527384	nonDEG
Bpev01.c0149.g0015.m0002	-0.166074259	0.61861111	0.9198087	nonDEG
Bpev01.c1871.g0001.m0001	0.750827744	0.04051015	0.17305099	nonDEG
Bpev01.c0518.g0012.m0001	-0.618055009	0.07621043	0.26760005	nonDEG
Bpev01.c0134.g0010.m0001	-0.452271313	0.14036075	0.40173301	nonDEG
Bpev01.c0101.g0067.m0001	-0.714930703	0.01895756	0.09901401	nonDEG
Bpev01.c0078.g0001.m0001	0.581593493	0.09716097	0.31401052	nonDEG
Bpev01.c1229.g0022.m0001	0.657092161	0.03861152	0.16696307	nonDEG
Bpev01.c1072.g0005.m0001	0.330406392	0.29718165	0.62737402	nonDEG
Bpev01.c0442.g0038.m0001	-0.572385983	0.17536904	0.46353413	nonDEG
Bpev01.c0960.g0007.m0001	2.948187215	0.03448223	0.15338808	nonDEG
Bpev01.c0051.g0007.m0001	-0.875180899	0.04388878	0.18319041	nonDEG
Bpev01.c0690.g0006.m0001	0.642183901	0.05862837	0.22362795	nonDEG
Bpev01.c0365.g0011.m0001	-0.454226452	0.16239101	0.44078866	nonDEG
Bpev01.c1818.g0003.m0001	-0.53293174	0.20429998	0.50673075	nonDEG
Bpev01.c0768.g0004.m0001	-0.248590605	0.43194837	0.76359383	nonDEG
Bpev01.c0023.g0051.m0001	0.187365703	0.66700212	0.93220195	nonDEG
Bpev01.c1529.g0007.m0003	-0.592894501	0.26197282	0.58420127	nonDEG
Bpev01.c1405.g0005.m0001	-1.021140209	0.03595613	0.1582704	nonDEG
Bpev01.c0091.g0019.m0001	0.471686538	0.11877523	0.36006838	nonDEG
Bpev01.c1211.g0015.m0001	-0.772962748	0.01741491	0.09318024	nonDEG
Bpev01.c1354.g0006.m0001	0.027255259	0.93244461	1	nonDEG
Bpev01.c0066.g0007.m0001	-0.26123131	0.38538415	0.71901413	nonDEG
Bpev01.c0038.g0102.m0001	-0.757198116	0.05066982	0.20234249	nonDEG
Bpev01.c0321.g0023.m0001	-0.215190103	0.50125363	0.82387391	nonDEG
Bpev01.c2326.g0002.m0001	-0.631599852	0.06662796	0.24435786	nonDEG
Bpev01.c0141.g0007.m0001	0.162139834	0.62746645	0.92511462	nonDEG
Bpev01.c0044.g0096.m0001	0.305321676	0.30573775	0.63694102	nonDEG
Bpev01.c1990.g0002.m0001	0.470925099	0.18450792	0.4780189	nonDEG
Bpev01.c2473.g0004.m0001	0.319654903	0.48370565	0.81253758	nonDEG
Bpev01.c0042.g0059.m0001	-0.243387972	0.57410284	0.88595855	nonDEG
Bpev01.c0022.g0047.m0001	-0.696333875	0.06640621	0.24375718	nonDEG
Bpev01.c2342.g0002.m0001	-0.282368619	0.37699402	0.71458531	nonDEG
Bpev01.c2197.g0009.m0001	-0.03974737	0.89465717	1	nonDEG
Bpev01.c0944.g0008.m0002	0.397561701	0.18751682	0.48203874	nonDEG

Bpev01.c0286.g0005.m0002	0.011755941	0.97116282	1	nonDEG
Bpev01.c0016.g0170.m0001	0.376775632	0.22204308	0.53137816	nonDEG
Bpev01.c0127.g0051.m0001	1.471782094	0.37400131	0.71337602	nonDEG
Bpev01.c0299.g0021.m0001	0.032768065	0.95878579	1	nonDEG
Bpev01.c0127.g0095.m0001	0.776720823	0.14414446	0.40853651	nonDEG
Bpev01.c0568.g0025.m0001	0.510161459	0.09164063	0.30214007	nonDEG
Bpev01.c0906.g0023.m0001	0.068231095	0.86064316	1	nonDEG
Bpev01.c0598.g0009.m0001	0.003214347	0.99171993	1	nonDEG
Bpev01.c1029.g0015.m0001	0.214948487	0.71493117	0.96714319	nonDEG
Bpev01.c0237.g0035.m0001	-0.232385816	0.45852041	0.78772909	nonDEG
Bpev01.c1577.g0024.m0001	-0.325815767	0.34070912	0.67687382	nonDEG
Bpev01.c2022.g0001.m0001	0.00239245	0.99715435	1	nonDEG
Bpev01.c0189.g0044.m0001	-0.749483522	0.01749678	0.09344843	nonDEG
Bpev01.c0022.g0066.m0001	-0.042449626	0.89707197	1	nonDEG
Bpev01.c1560.g0012.m0001	0.855429485	0.02924252	0.13617901	nonDEG
Bpev01.c1391.g0003.m0001	0.011168904	0.97883863	1	nonDEG
Bpev01.c0575.g0002.m0001	0.910283238	0.74969746	0.98717822	nonDEG
Bpev01.c2190.g0001.m0001	-0.377619949	0.24016351	0.55725567	nonDEG
Bpev01.c0518.g0042.m0001	-0.830675295	0.01098356	0.06640894	nonDEG
Bpev01.c0121.g0045.m0001	1.012263425	0.05065801	0.20232278	nonDEG
Bpev01.c0349.g0052.m0001	-0.506727562	0.13970931	0.40059437	nonDEG
Bpev01.c0090.g0023.m0001	-0.109612537	0.82178163	1	nonDEG
Bpev01.c1537.g0005.m0001	-1.169971225	0.01011954	0.06237113	nonDEG
Bpev01.c0127.g0021.m0001	0.343815236	0.37870552	0.71646079	nonDEG
Bpev01.c0001.g0079.m0002	0.155491592	0.6032399	0.90856408	nonDEG
Bpev01.c1372.g0007.m0001	-0.245054945	0.45235369	0.78338169	nonDEG
Bpev01.c2195.g0001.m0001	2.239170352	0.02438121	0.11960649	nonDEG
Bpev01.c1344.g0004.m0001	0.311828376	0.33211618	0.66770254	nonDEG
Bpev01.c0172.g0007.m0003	-0.907001354	0.01760957	0.09388052	nonDEG
Bpev01.c0050.g0033.m0001	0.212615833	0.55966905	0.87452745	nonDEG
Bpev01.c0001.g0073.m0001	-0.424162658	0.18684354	0.48131277	nonDEG
Bpev01.c4423.g0001.m0001	-2.131002361	0.02721349	0.12930548	nonDEG
Bpev01.c1472.g0002.m0001	-0.343013674	0.32452642	0.65856477	nonDEG
Bpev01.c0107.g0003.m0001	-0.071081367	0.81998122	1	nonDEG
Bpev01.c1146.g0002.m0001	-0.290944752	0.4628101	0.79176351	nonDEG
Bpev01.c0051.g0132.m0001	-0.069391845	0.85713805	1	nonDEG
Bpev01.c2272.g0003.m0001	0.743608816	0.08155818	0.27987912	nonDEG
Bpev01.c0984.g0001.m0001	0.068115255	0.83114654	1	nonDEG
Bpev01.c0115.g0029.m0001	-0.380842231	0.73143317	0.97684802	nonDEG
Bpev01.c0120.g0015.m0001	0.698694341	0.03145935	0.14349107	nonDEG
Bpev01.c0022.g0164.m0001	0.277622576	0.40303508	0.73631444	nonDEG
Bpev01.c1779.g0002.m0002	0.473327927	0.29800548	0.62848223	nonDEG
Bpev01.c0127.g0110.m0001	-0.514660119	0.19843543	0.4984999	nonDEG

Bpev01.c0324.g0017.m0001	0.319847666	0.29366887	0.62358034	nonDEG
Bpev01.c0174.g0002.m0001	-0.537448908	0.0836677	0.28445472	nonDEG
Bpev01.c1211.g0011.m0001	-0.788652986	0.00933628	0.05860358	nonDEG
Bpev01.c0394.g0023.m0001	0.093157026	0.83513959	1	nonDEG
Bpev01.c1524.g0001.m0002	-0.363672044	0.40707502	0.74043135	nonDEG
Bpev01.c0045.g0011.m0001	0.563551062	0.08558318	0.28853449	nonDEG
Bpev01.c0038.g0164.m0001	1.028102193	0.65678759	0.93220195	nonDEG
Bpev01.c0568.g0030.m0001	0.400493369	0.21915639	0.52723258	nonDEG
Bpev01.c1202.g0005.m0002	-0.426402207	0.1764692	0.46487724	nonDEG
Bpev01.c0237.g0044.m0002	-0.441386411	0.18697049	0.48131277	nonDEG
Bpev01.c0618.g0004.m0001	0.441521229	0.20474696	0.5070704	nonDEG
Bpev01.c0237.g0006.m0001	0.470061013	0.17586463	0.46424666	nonDEG
Bpev01.c0000.g0239.m0001	-1.105038583	0.05506011	0.21434451	nonDEG
Bpev01.c0027.g0130.m0001	-0.165381076	0.58897606	0.89861966	nonDEG
Bpev01.c2401.g0002.m0001	-0.102705756	0.76745084	0.99867327	nonDEG
Bpev01.c0274.g0028.m0001	-0.206034374	0.48752342	0.81424325	nonDEG
Bpev01.c1072.g0014.m0001	-0.607544463	0.29227814	0.62200557	nonDEG
Bpev01.c1588.g0008.m0001	0.716343011	0.01848491	0.09729611	nonDEG
Bpev01.c0094.g0048.m0001	-0.657949331	0.04456955	0.18519165	nonDEG
Bpev01.c0617.g0003.m0001	0.885285422	0.04027903	0.17230083	nonDEG
Bpev01.c0455.g0031.m0001	-0.09017484	0.7769019	1	nonDEG
Bpev01.c0667.g0022.m0001	-0.139543314	0.73050043	0.97617229	nonDEG
Bpev01.c1152.g0004.m0001	-0.890841986	0.01204764	0.07099088	nonDEG
Bpev01.c1070.g0016.m0001	0.156551328	0.70641808	0.96080508	nonDEG
Bpev01.c0159.g0024.m0001	0.358840852	0.66645128	0.93220195	nonDEG
Bpev01.c0281.g0034.m0001	0.061872944	0.87556046	1	nonDEG
Bpev01.c0286.g0040.m0001	0.488793224	0.12341206	0.37053825	nonDEG
Bpev01.c2581.g0001.m0001	0.714799406	0.03564325	0.15717522	nonDEG
Bpev01.c0357.g0081.m0001	0.418527416	0.19913266	0.49959659	nonDEG
Bpev01.c0525.g0010.m0001	-0.673129983	0.06234331	0.23350186	nonDEG
Bpev01.c1758.g0004.m0001	-0.557607738	0.06739603	0.24622384	nonDEG
Bpev01.c0332.g0009.m0003	0.872368691	0.0105327	0.06429032	nonDEG
Bpev01.c0883.g0002.m0001	0.124396486	0.71677099	0.96831905	nonDEG
Bpev01.c0038.g0015.m0001	0.345530182	0.36161639	0.69972558	nonDEG
Bpev01.c0887.g0015.m0001	-1.230062044	0.37102693	0.71046833	nonDEG
Bpev01.c2300.g0002.m0001	-0.54289996	0.07073583	0.2544002	nonDEG
Bpev01.c0029.g0122.m0001	-1.637038667	0.22447022	0.53459904	nonDEG
Bpev01.c0298.g0034.m0001	0.631987575	0.06190107	0.23231839	nonDEG
Bpev01.c1657.g0013.m0001	0.801497997	0.01626037	0.08876116	nonDEG
Bpev01.c1067.g0013.m0001	0.887505635	0.0160737	0.08797058	nonDEG
Bpev01.c1040.g0024.m0001	1.412596182	0.0140532	0.07957457	nonDEG
Bpev01.c0768.g0003.m0001	1.669618437	0.01356599	0.07750227	nonDEG
Bpev01.c0038.g0025.m0001	0.216431543	0.49050889	0.81474556	nonDEG

Bpev01.c1320.g0007.m0001	0.717325537	0.04497152	0.1860376	nonDEG
Bpev01.c4734.g0001.m0001	0.029494221	0.94456034	1	nonDEG
Bpev01.c0620.g0009.m0001	-0.924083619	0.03586118	0.15797055	nonDEG
Bpev01.c1183.g0019.m0001	0.02640209	0.94222294	1	nonDEG
Bpev01.c0480.g0008.m0001	-0.853092789	0.06015405	0.22777041	nonDEG
Bpev01.c1957.g0004.m0001	-0.268157044	0.38169318	0.71703402	nonDEG
Bpev01.c0606.g0020.m0001	0	1	1	nonDEG
Bpev01.c2293.g0001.m0002	-0.136117238	0.72890651	0.97576589	nonDEG
Bpev01.c0189.g0071.m0001	-0.27303182	0.48720485	0.81405434	nonDEG
Bpev01.c0000.g0022.m0001	-0.403163504	0.17629349	0.46485888	nonDEG
Bpev01.c0438.g0015.m0001	0.924906647	0.00818545	0.05311248	nonDEG
Bpev01.c0930.g0005.m0001	-0.277772296	0.38635538	0.71927629	nonDEG
Bpev01.c0571.g0011.m0001	-0.70331741	0.04087471	0.17403973	nonDEG
Bpev01.c0089.g0038.m0001	-0.164282837	0.61509939	0.91713487	nonDEG
Bpev01.c0050.g0137.m0001	-0.388226751	0.21401002	0.52102556	nonDEG
Bpev01.c0511.g0008.m0001	-0.150079363	0.62660551	0.92459724	nonDEG
Bpev01.c0052.g0195.m0001	-0.437298216	0.15773523	0.43365403	nonDEG
Bpev01.c0786.g0007.m0001	0.107033301	0.73342268	0.97781941	nonDEG
Bpev01.c1081.g0004.m0001	0.828883074	0.04824877	0.19505569	nonDEG
Bpev01.c0029.g0024.m0001	0.393510908	0.25581109	0.57597115	nonDEG
Bpev01.c0874.g0002.m0001	-0.073992574	0.86699268	1	nonDEG
Bpev01.c0286.g0029.m0001	0.438723951	0.16213484	0.44052493	nonDEG
Bpev01.c0038.g0120.m0001	0.64424614	0.0589331	0.22435426	nonDEG
Bpev01.c0679.g0014.m0001	-0.19602356	0.52328821	0.8429132	nonDEG
Bpev01.c0167.g0017.m0001	-0.45004924	0.30298081	0.63467163	nonDEG
Bpev01.c0531.g0016.m0001	-0.161453646	0.6070546	0.91118546	nonDEG
Bpev01.c0050.g0122.m0002	0.614635012	0.16333923	0.4422919	nonDEG
Bpev01.c0161.g0055.m0002	-0.119229547	0.70828808	0.96225069	nonDEG
Bpev01.c0328.g0024.m0001	0.688858101	0.04108686	0.17464576	nonDEG
Bpev01.c0394.g0018.m0001	0.924965024	0.01123772	0.06750201	nonDEG
Bpev01.c0402.g0021.m0001	-0.244761684	0.489701	0.81451458	nonDEG
Bpev01.c0015.g0044.m0001	-0.402758045	0.26414265	0.58727403	nonDEG
Bpev01.c0210.g0011.m0001	0.103240293	0.82236669	1	nonDEG
Bpev01.c0052.g0016.m0001	0.597111766	0.07349139	0.2612623	nonDEG
Bpev01.c0518.g0033.m0002	0.034926905	0.92015875	1	nonDEG
Bpev01.c1371.g0008.m0001	0.987888945	0.03393384	0.15159003	nonDEG
Bpev01.c0455.g0001.m0001	1.1230655	0.4333025	0.76506881	nonDEG
Bpev01.c0506.g0037.m0001	0.428748308	0.46328002	0.7923187	nonDEG
Bpev01.c0349.g0038.m0001	1.205322111	0.20460219	0.50699651	nonDEG
Bpev01.c2423.g0001.m0001	0.351275382	0.66086709	0.93220195	nonDEG
Bpev01.c0046.g0005.m0001	-0.814806161	0.04192039	0.17721057	nonDEG
Bpev01.c1070.g0024.m0001	-0.288908522	0.5032097	0.82584404	nonDEG
Bpev01.c1043.g0009.m0001	-0.637736333	0.0586602	0.22369141	nonDEG

Bpev01.c0714.g0006.m0001	-0.134755758	0.8223332	1	nonDEG
Bpev01.c1732.g0001.m0001	1.866817416	0.15215138	0.42348345	nonDEG
Bpev01.c0038.g0119.m0001	0.562918973	0.06860286	0.24941736	nonDEG
Bpev01.c0242.g0007.m0001	0.491217667	0.15960674	0.43681837	nonDEG
Bpev01.c1469.g0003.m0001	0.080062737	0.8028279	1	nonDEG
Bpev01.c0394.g0020.m0002	0.802857094	0.0168361	0.09095941	nonDEG
Bpev01.c3512.g0005.m0001	-0.477149246	0.29523157	0.62524917	nonDEG
Bpev01.c1930.g0003.m0001	-0.981343964	0.01548416	0.08553915	nonDEG
Bpev01.c0062.g0025.m0001	-1.267961192	0.46447297	0.79327144	nonDEG
Bpev01.c0387.g0004.m0001	0.38075856	0.21668487	0.52321078	nonDEG
Bpev01.c0995.g0013.m0001	-0.013777926	0.96757827	1	nonDEG
Bpev01.c0335.g0009.m0001	0.57032996	0.12351003	0.37071899	nonDEG
Bpev01.c0215.g0021.m0001	0.007981018	0.98000457	1	nonDEG
Bpev01.c0358.g0004.m0001	0.932298692	0.03381365	0.15128402	nonDEG
Bpev01.c0145.g0006.m0001	0.537729112	0.16676767	0.44869	nonDEG
Bpev01.c0016.g0010.m0001	-0.919685408	0.02166813	0.10941474	nonDEG
Bpev01.c0145.g0002.m0001	0.582822451	0.11235367	0.34685188	nonDEG
Bpev01.c0796.g0014.m0001	-0.666734999	0.06022987	0.22789447	nonDEG
Bpev01.c0148.g0011.m0001	0.50559051	0.19637705	0.49563991	nonDEG
Bpev01.c0088.g0063.m0001	2.282623364	0.02601107	0.12542831	nonDEG
Bpev01.c0299.g0036.m0001	-0.737495925	0.13979746	0.40080808	nonDEG
Bpev01.c0564.g0014.m0001	-0.791240535	0.01054903	0.06433661	nonDEG
Bpev01.c0954.g0009.m0001	-0.282942733	0.63240209	0.92831514	nonDEG
Bpev01.c0215.g0018.m0001	-0.160227857	0.59875562	0.90554913	nonDEG
Bpev01.c0324.g0023.m0001	0.474518011	0.1699792	0.45453881	nonDEG
Bpev01.c1233.g0002.m0001	-0.236230076	0.55324116	0.86886458	nonDEG
Bpev01.c3589.g0003.m0001	0.29665361	0.79820145	1	nonDEG
Bpev01.c1259.g0021.m0001	3.430375108	0.12932048	0.38116395	nonDEG
Bpev01.c0082.g0074.m0001	0.382112669	0.26763462	0.59201235	nonDEG
Bpev01.c1638.g0011.m0001	0.740123615	0.0147343	0.08248012	nonDEG
Bpev01.c0130.g0018.m0001	-0.909646806	0.03548782	0.15665408	nonDEG
Bpev01.c0088.g0031.m0001	0.311943119	0.52567222	0.84541683	nonDEG
Bpev01.c0265.g0002.m0001	-0.098062945	0.7565205	0.99137355	nonDEG
Bpev01.c2566.g0005.m0001	1.709898161	0.00961664	0.05997975	nonDEG
Bpev01.c0565.g0007.m0001	-2.288527307	0.02948431	0.13701477	nonDEG
Bpev01.c0523.g0012.m0001	0.788148897	0.01409421	0.07971751	nonDEG
Bpev01.c0415.g0028.m0001	0.542150714	0.13201374	0.38566561	nonDEG
Bpev01.c0629.g0025.m0001	-0.379939653	0.2351927	0.55125615	nonDEG
Bpev01.c0817.g0032.m0001	-5.168889179	0.16029714	0.43791644	nonDEG
Bpev01.c0224.g0017.m0001	0.586395426	0.19587141	0.49491532	nonDEG
Bpev01.c0349.g0043.m0001	0.122730413	0.71880925	0.9695398	nonDEG
Bpev01.c0094.g0188.m0001	-0.352436265	0.32682462	0.6616277	nonDEG
Bpev01.c1033.g0002.m0001	-0.983924144	0.11193559	0.34579289	nonDEG

Bpev01.c0555.g0012.m0001	-1.899689472	0.29053978	0.61979713	nonDEG
Bpev01.c1475.g0001.m0001	0	1	1	nonDEG
Bpev01.c0115.g0077.m0001	-0.242842046	0.42557717	0.75780302	nonDEG
Bpev01.c1824.g0002.m0001	-0.398024309	0.4148666	0.74697103	nonDEG
Bpev01.c0333.g0028.m0001	-0.051460472	0.86375522	1	nonDEG
Bpev01.c0230.g0026.m0001	-0.429696765	0.37003467	0.70971243	nonDEG
Bpev01.c0022.g0108.m0001	0.079521269	0.89094482	1	nonDEG
Bpev01.c0889.g0008.m0001	0.226221972	0.45830405	0.78767609	nonDEG
Bpev01.c1320.g0004.m0001	1.06847215	0.00805426	0.05246941	nonDEG
Bpev01.c2530.g0003.m0001	-0.632738126	0.0475881	0.19318066	nonDEG
Bpev01.c0615.g0001.m0001	0.737637485	0.01697564	0.09149504	nonDEG
Bpev01.c0929.g0022.m0001	-0.312255977	0.37127913	0.71077823	nonDEG
Bpev01.c0906.g0014.m0001	-0.654142848	0.03043444	0.1401031	nonDEG
Bpev01.c0340.g0022.m0001	-0.063156042	0.91896091	1	nonDEG
Bpev01.c0922.g0001.m0001	0.184837656	0.53870198	0.85620693	nonDEG
Bpev01.c0435.g0006.m0004	-0.314254089	0.49727779	0.82040802	nonDEG
Bpev01.c0000.g0157.m0001	0.955933796	0.22951461	0.54270571	nonDEG
Bpev01.c2361.g0011.m0001	0.456856012	0.12927007	0.3811025	nonDEG
Bpev01.c0931.g0012.m0001	0.294854092	0.94368687	1	nonDEG
Bpev01.c0115.g0078.m0001	-0.994586219	0.34243287	0.67893401	nonDEG
Bpev01.c1817.g0003.m0001	1.159497448	0.05117313	0.20377192	nonDEG
Bpev01.c1681.g0003.m0001	-0.45473939	0.78128594	1	nonDEG
Bpev01.c0903.g0001.m0001	1.343930839	0.04147271	0.17584834	nonDEG
Bpev01.c0328.g0020.m0001	-0.422262796	0.21958697	0.52800955	nonDEG
Bpev01.c1029.g0018.m0001	4.342816547	0.29940051	0.63014748	nonDEG
Bpev01.c0365.g0022.m0001	0.166202566	0.66536352	0.93220195	nonDEG
Bpev01.c1317.g0001.m0001	0.459334815	0.54501962	0.86142552	nonDEG
Bpev01.c1108.g0011.m0001	-0.747569871	0.11704866	0.35633873	nonDEG
Bpev01.c0261.g0012.m0001	0.096715043	0.78284154	1	nonDEG
Bpev01.c0077.g0013.m0001	-0.85204157	0.06316461	0.23556507	nonDEG
Bpev01.c0930.g0008.m0001	-1.88988662	0.23804392	0.55485154	nonDEG
Bpev01.c0190.g0070.m0001	-0.645275663	0.29676546	0.62686113	nonDEG
Bpev01.c0435.g0006.m0003	0.005830184	0.98950041	1	nonDEG
Bpev01.c0425.g0045.m0001	-0.567842883	0.08543726	0.28817457	nonDEG
Bpev01.c0415.g0026.m0001	-0.362404313	0.23537759	0.55143092	nonDEG
Bpev01.c0130.g0014.m0001	0.45775769	0.49885997	0.82168291	nonDEG
Bpev01.c0022.g0104.m0001	-0.414816495	0.23983425	0.55668852	nonDEG
Bpev01.c0161.g0044.m0001	0.137723441	0.67899253	0.94074082	nonDEG
Bpev01.c0870.g0002.m0001	2.795891432	0.04575033	0.18807636	nonDEG
Bpev01.c0880.g0001.m0001	1.276019066	0.01008742	0.06221739	nonDEG
Bpev01.c1172.g0012.m0001	0.592756539	0.10876507	0.33877864	nonDEG
Bpev01.c0177.g0008.m0001	0.816892867	0.22817231	0.54070391	nonDEG
Bpev01.c2579.g0001.m0001	0.157235624	0.61180673	0.91495952	nonDEG

Bpev01.c0115.g0119.m0001	0.456481432	0.1263867	0.37552779	nonDEG
Bpev01.c0415.g0009.m0001	-1.679398677	0.0082934	0.05362386	nonDEG
Bpev01.c0015.g0204.m0001	-0.510398679	0.09308451	0.30502169	nonDEG
Bpev01.c0042.g0013.m0001	0.381171729	0.21347149	0.52031687	nonDEG
Bpev01.c1550.g0001.m0001	0.500036072	0.28222486	0.60909233	nonDEG
Bpev01.c0629.g0005.m0001	-0.55060874	0.32154218	0.65489692	nonDEG
Bpev01.c0806.g0001.m0001	0.002821968	0.99807492	1	nonDEG
Bpev01.c0094.g0049.m0001	0.891844566	0.34357622	0.680152	nonDEG
Bpev01.c0328.g0035.m0001	1.268078169	0.01924447	0.10018354	nonDEG
Bpev01.c0112.g0020.m0001	0.925527736	0.01550345	0.08559157	nonDEG
Bpev01.c1663.g0006.m0001	3.580544292	0.03042664	0.14008912	nonDEG
Bpev01.c0865.g0001.m0001	3.843935483	0.0281204	0.13247504	nonDEG
Bpev01.c2911.g0002.m0001	-0.803138776	0.10320267	0.32736675	nonDEG
Bpev01.c0038.g0124.m0001	-0.822474417	0.05577076	0.21608785	nonDEG
Bpev01.c1223.g0006.m0001	1.880894154	0.27483267	0.60132285	nonDEG
Bpev01.c0088.g0131.m0001	0.699602625	0.0622432	0.23328406	nonDEG
Bpev01.c0441.g0009.m0001	-1.043288415	0.01153788	0.06872999	nonDEG
Bpev01.c0177.g0012.m0001	-1.014130239	0.03109191	0.14232855	nonDEG
Bpev01.c1596.g0001.m0001	2.395413686	1	1	nonDEG
Bpev01.c0224.g0034.m0001	-0.559804266	0.3917187	0.72472391	nonDEG
Bpev01.c2000.g0001.m0001	-0.177527998	0.58248686	0.89297875	nonDEG
Bpev01.c0604.g0013.m0001	-0.61696413	0.20049519	0.50122095	nonDEG
Bpev01.c0602.g0008.m0001	-0.37704507	0.23342756	0.54872252	nonDEG
Bpev01.c0038.g0091.m0001	-0.082890306	0.78150489	1	nonDEG
Bpev01.c0415.g0005.m0001	-1.169986727	0.03734909	0.16289183	nonDEG
Bpev01.c0304.g0034.m0001	-1.180712577	0.03910249	0.16840726	nonDEG
Bpev01.c1837.g0011.m0001	-3.872312869	0.01604827	0.08786404	nonDEG
Bpev01.c0001.g0036.m0001	-0.864423625	0.00930968	0.05852393	nonDEG
Bpev01.c1352.g0008.m0001	0.811211098	0.142438	0.40557426	nonDEG
Bpev01.c0605.g0025.m0001	0.683739549	0.20067811	0.50142276	nonDEG
Bpev01.c1427.g0003.m0001	0.059767321	0.8568599	1	nonDEG
Bpev01.c2001.g0007.m0001	-0.59962725	0.12026273	0.36382845	nonDEG
Bpev01.c0948.g0004.m0001	-0.23932788	0.82254603	1	nonDEG
Bpev01.c0016.g0049.m0001	-0.655948044	0.44867982	0.78021325	nonDEG
Bpev01.c0962.g0009.m0001	-0.795052151	0.01466389	0.08217977	nonDEG
Bpev01.c0045.g0077.m0001	0.193732519	0.66296358	0.93220195	nonDEG
Bpev01.c0582.g0013.m0001	1.178217364	0.11480446	0.35187178	nonDEG
Bpev01.c0000.g0049.m0001	-0.049656364	0.8802629	1	nonDEG
Bpev01.c0052.g0068.m0001	0.303283992	0.31962485	0.65279483	nonDEG
Bpev01.c0936.g0005.m0001	0.723616665	0.04689666	0.19132297	nonDEG
Bpev01.c0312.g0025.m0001	1.25472033	0.43593107	0.76832753	nonDEG
Bpev01.c0245.g0045.m0001	1.824222801	0.00934217	0.05861557	nonDEG
Bpev01.c0115.g0097.m0001	-0.594929908	0.04664787	0.19075796	nonDEG

Bpev01.c3172.g0001.m0001	0.820338369	0.01524687	0.08448197	nonDEG
Bpev01.c1427.g0005.m0001	0.812574781	0.00912531	0.05770997	nonDEG
Bpev01.c0132.g0001.m0001	2.854416216	0.07870664	0.27310758	nonDEG
Bpev01.c0365.g0010.m0001	0.440601688	0.36936289	0.70930521	nonDEG
Bpev01.c0237.g0008.m0001	-1.02283323	0.00968521	0.06024791	nonDEG
Bpev01.c0018.g0038.m0001	0.441803672	0.16172562	0.44010359	nonDEG
Bpev01.c1961.g0003.m0001	0.072395577	0.85954513	1	nonDEG
Bpev01.c0214.g0041.m0001	-1.681102716	0.01785246	0.09478344	nonDEG
Bpev01.c0088.g0110.m0001	1.57115805	0.01681833	0.09088011	nonDEG
Bpev01.c0321.g0005.m0001	-0.606410171	0.7094876	0.96291574	nonDEG
Bpev01.c0223.g0002.m0001	-2.403374069	0.01031049	0.06330144	nonDEG
Bpev01.c0444.g0034.m0001	-0.979531681	0.00995566	0.0616631	nonDEG
Bpev01.c2405.g0004.m0001	-2.319789721	0.01274289	0.07413795	nonDEG
Bpev01.c2405.g0005.m0001	-2.187445765	0.01630034	0.08891341	nonDEG
Bpev01.c0564.g0024.m0001	2.650138897	0.04943736	0.19868756	nonDEG
Bpev01.c0280.g0014.m0001	1.302563722	0.07117985	0.25562837	nonDEG
Bpev01.c1453.g0005.m0001	0.756213804	0.06677521	0.24465406	nonDEG
Bpev01.c0956.g0002.m0001	0.927525376	0.01839473	0.09694278	nonDEG
Bpev01.c0044.g0030.m0001	1.823534931	0.01837556	0.09691115	nonDEG
Bpev01.c0210.g0038.m0001	-1.182391727	0.06017775	0.22777041	nonDEG
Bpev01.c0226.g0001.m0002	-1.722291829	0.01803897	0.09551246	nonDEG
Bpev01.c0704.g0004.m0001	1.973544331	0.00868273	0.05553136	nonDEG
Bpev01.c0253.g0013.m0001	-0.774534516	0.01372678	0.07822353	nonDEG
Bpev01.c1100.g0009.m0001	0.259538499	0.72577836	0.97377891	nonDEG
Bpev01.c0214.g0041.m0002	-1.084960615	0.01106628	0.06679932	nonDEG
Bpev01.c0864.g0013.m0001	-2.170862774	0.01705328	0.09175362	nonDEG
Bpev01.c0298.g0039.m0001	0.602766681	0.08023446	0.27684396	nonDEG
Bpev01.c0278.g0003.m0001	0.188957778	0.72384776	0.97283081	nonDEG

with the previous nearby time points.

Comparison_2	Comparison_2	Comparison_2	DEG/nonDEG	Comparison_3	Comparison_3	Comparison_3
-0.4254412	0.08371079	0.52900194	nonDEG	-0.115906	0.75236288	1
-0.251202	0.42028433	0.96323058	nonDEG	0.25055453	0.50272493	1
1.26655451	4.01E-06	0.00032968	DEG	0.06130471	0.87303369	1
-0.5346871	0.0461931	0.38621947	nonDEG	-0.0315103	0.93847678	1
0.40927842	0.12341845	0.64067117	nonDEG	0.18529469	0.64619348	1
2.56622704	7.94E-11	3.00E-08	DEG	-0.6910304	0.17951606	1
-0.6499361	0.23520958	0.81654893	nonDEG	0.75146057	0.19942177	1
0.49656671	0.04717127	0.39084574	nonDEG	0.14707478	0.68857715	1
-0.3614499	0.36887041	0.92821405	nonDEG	-0.5446564	0.26550262	1
1.24246219	0.00208258	0.04734293	DEG	0.59721479	0.23349991	1
-0.1185475	0.79167665	1	nonDEG	0.77089456	0.13047551	1
-1.6899734	9.35E-09	1.85E-06	DEG	-0.6844096	0.09584074	1
0.21946467	0.66353859	1	nonDEG	0.56667947	0.29118649	1
-0.6630817	0.04038134	0.35647711	nonDEG	-0.4169476	0.34374199	1
-1.1388444	0.03632338	0.3355268	nonDEG	0.91558315	0.15085807	1
0.70974334	0.13792476	0.66530676	nonDEG	0.72439539	0.15838478	1
0.510349	0.29360037	0.87686936	nonDEG	0.50572438	0.35080243	1
-1.0070715	0.03554977	0.33139637	nonDEG	-2.1315428	0.0036701	0.49008216
-3.9032461	4.71E-30	1.39E-25	DEG	0.47714608	0.55083652	1
-0.7694466	0.0236379	0.25678087	nonDEG	0.16641296	0.71839051	1
-0.473787	0.20358647	0.77664663	nonDEG	-0.4372833	0.35341031	1
-2.2826138	4.03E-08	6.48E-06	DEG	-1.14313	0.1234189	1
-1.0262438	0.00064388	0.01944111	DEG	0.01876671	0.96551248	1
0.08323516	0.9407493	1	nonDEG	0.03175574	1	1
-0.4391962	0.4487245	0.98329655	nonDEG	0.82171135	0.11173672	1
-0.54433	0.03556755	0.3314571	nonDEG	-0.2309035	0.54709011	1
0.44594016	0.11678403	0.62509182	nonDEG	0.4967349	0.19195416	1
1.1917748	0.00544923	0.09425382	nonDEG	0.72020328	0.16236503	1
-1.7911769	3.71E-10	1.08E-07	DEG	-0.3388807	0.42631101	1
1.24987646	0.0469189	0.39007214	nonDEG	1.15766828	0.06585741	1
0.29860415	0.48332316	0.99599492	nonDEG	0.48415911	0.32721893	1
-2.9164315	0.01012765	0.14719589	nonDEG	1.80555585	0.32947132	1
-1.4402396	0.00384185	0.07435909	nonDEG	-0.9314824	0.22034985	1
-0.529363	0.13823097	0.66596638	nonDEG	-0.5284009	0.26071231	1
0.53657712	0.13760188	0.66494775	nonDEG	0.28357878	0.49684633	1
0.64892509	0.01469956	0.18763285	nonDEG	0.87873301	0.01594863	0.90504819
-0.3434236	0.57201858	1	nonDEG	0.74454703	0.26850016	1
-0.3871339	0.66515868	1	nonDEG	1.79742685	0.02283477	1
-0.0932633	0.71299436	1	nonDEG	0.03382857	0.92762662	1
-1.1449985	0.06274316	0.45833648	nonDEG	-1.5482538	0.09535716	1
0.19888588	0.69238461	1	nonDEG	0.79072795	0.09325109	1

-0.1096255	0.70282626	1	nonDEG	-0.2113294	0.65260578	1
0.82121123	0.05711825	0.43505932	nonDEG	0.83122049	0.07978992	1
2.0720354	1.05E-05	0.00072916	DEG	2.08846817	0.00017082	0.06208357
-0.4902494	0.09532597	0.56333484	nonDEG	-0.6498382	0.14235098	1
-0.0631107	0.82495094	1	nonDEG	-0.45877	0.30713876	1
-0.6521511	0.17414887	0.73544234	nonDEG	-0.9765162	0.11677451	1
2.55980328	1.46E-12	8.10E-10	DEG	-0.7801817	0.06951336	1
-0.4600313	0.085343	0.53493735	nonDEG	-0.2632066	0.48468365	1
-0.9467141	0.00133869	0.03374116	DEG	-0.7552939	0.09355144	1
-1.2492191	0.00023096	0.00859883	DEG	-1.2410155	0.00620591	0.62141396
-0.2370755	0.35424612	0.91917773	nonDEG	-0.4182073	0.2501392	1
0.29640356	0.3743159	0.93251128	nonDEG	0.1986709	0.62803515	1
0.45194283	0.1254899	0.64431252	nonDEG	0.42345363	0.25107001	1
0.01696018	0.97390776	1	nonDEG	0.64138689	0.17798609	1
0.22498749	0.37005133	0.92872472	nonDEG	-0.5560177	0.12246989	1
-1.1854354	0.00116323	0.03027793	DEG	-0.0740882	0.87898552	1
2.31587352	0.00448403	0.08226984	nonDEG	-3.7751964	9.46E-05	0.04219605
0.50069521	0.38917411	0.94606909	nonDEG	-1.060008	0.12282052	1
-0.9778115	0.02059523	0.2335528	nonDEG	-0.8908269	0.1167594	1
-0.8684668	0.03008652	0.29847765	nonDEG	-0.2162486	0.71160146	1
-1.4634411	5.36E-06	0.00042516	DEG	-0.1872152	0.63938886	1
0.33547005	0.76381486	1	nonDEG	-0.2566891	0.83877305	1
-0.7430644	0.06225573	0.45672559	nonDEG	-0.1252634	0.89267764	1
0.6312262	0.07691604	0.50793676	nonDEG	0.28620299	0.49251838	1
-0.6483632	0.1840803	0.75161441	nonDEG	-0.4500832	0.46697673	1
-0.7335295	0.03586775	0.33267511	nonDEG	-0.0226079	0.9649347	1
-0.6411129	0.01508982	0.19114859	nonDEG	-0.3504405	0.38968239	1
0.77609454	0.06577721	0.46863877	nonDEG	0.50862231	0.21751451	1
0.63198095	0.51450983	1	nonDEG	0.64232366	0.48072861	1
0.25943031	0.74072921	1	nonDEG	1.43732693	0.06356572	1
-0.5355295	0.08899057	0.54533586	nonDEG	-0.1724173	0.68362025	1
0.03902922	0.92858662	1	nonDEG	0.21287361	0.66899389	1
1.05643976	0.00028397	0.0102291	DEG	-0.0849963	0.83299485	1
-0.4997431	0.1066131	0.59793922	nonDEG	-0.4322985	0.388103	1
1.84117954	0.00193144	0.04473627	DEG	0.04298991	0.95104457	1
2.58832373	1.51E-13	1.03E-10	DEG	0.29265525	0.62492805	1
-0.4887983	0.05286855	0.41681767	nonDEG	-0.2505413	0.49812077	1
-0.9997465	0.0009856	0.02644954	DEG	0.46020645	0.39666581	1
0.28413175	0.26991653	0.85268624	nonDEG	0.39421674	0.30180283	1
-0.3472402	0.27349125	0.85674092	nonDEG	-0.427906	0.27758045	1
-1.1974262	0.00221473	0.0492815	DEG	-0.0846866	0.88481826	1
-0.7246252	0.00671965	0.10941366	nonDEG	-0.4664047	0.19716634	1
0.16828284	0.57020179	1	nonDEG	0.79339483	0.06156782	1

0.04669769	0.87170761	1	nonDEG	-0.6866574	0.14935423	1
-0.45725	0.06701204	0.47329651	nonDEG	0.07155414	0.85841916	1
-0.4435879	0.0858164	0.53616486	nonDEG	-0.7787963	0.09405091	1
-0.809742	0.05371579	0.41978737	nonDEG	-0.624898	0.27956303	1
1.67818709	0.00484684	0.0865881	nonDEG	0.41576375	0.56836624	1
0.60494353	0.11220584	0.61193546	nonDEG	0.46111147	0.23644123	1
-0.1668151	0.82534572	1	nonDEG	1.13105066	0.15168135	1
0.21537882	0.4680213	0.986734	nonDEG	0.48502933	0.18867217	1
-3.8356261	0.01612137	0.19924305	nonDEG	2.68171175	0.30841772	1
0.66510692	0.09541288	0.56368848	nonDEG	-1.3646674	0.03976571	1
-0.7183425	0.01262899	0.17015329	nonDEG	-0.6436625	0.11762648	1
-0.1698294	0.55341391	1	nonDEG	-0.5296713	0.17710985	1
1.19403178	0.08361232	0.52875977	nonDEG	0.16152955	0.81649332	1
-0.4876829	0.04294918	0.36894687	nonDEG	0.41785181	0.35712645	1
1.71855694	4.77E-05	0.00243063	DEG	-0.7877171	0.11133813	1
-0.4608531	0.12776709	0.64928971	nonDEG	-0.4525427	0.24599156	1
-0.5517501	0.13178298	0.65533095	nonDEG	0.91140237	0.05283517	1
-0.6752979	0.02309325	0.25179337	nonDEG	-0.1485771	0.73132159	1
-1.24858	0.02342754	0.25496607	nonDEG	-0.4571453	0.52041163	1
1.46453251	0.00089048	0.02452275	DEG	-0.0001305	1	1
-0.4862732	0.08650443	0.53862182	nonDEG	-0.4534767	0.26391472	1
-2.5522066	2.99E-06	0.00025435	DEG	-1.9285242	0.02472683	1
-0.4159082	0.09063708	0.54902571	nonDEG	-0.1239308	0.73268399	1
-0.6404703	0.01245371	0.16873447	nonDEG	0.05754888	0.88849514	1
0.98237792	0.00051386	0.01635409	DEG	0.17366449	0.656995	1
-1.4713667	2.74E-07	3.18E-05	DEG	-1.0339191	0.0805976	1
0.91638299	0.12853183	0.65114404	nonDEG	0.02296426	0.97423634	1
-0.3576094	0.15917216	0.70687421	nonDEG	-0.391095	0.30153173	1
0.47634272	0.06206012	0.45594902	nonDEG	0.54727133	0.15499886	1
-1.5416154	0.0348095	0.32718929	nonDEG	-0.1459625	0.88995959	1
0.13758786	0.571805	1	nonDEG	0.37019065	0.39438866	1
0.96690866	0.00234982	0.05131777	nonDEG	0.81561627	0.04761318	1
-0.6577174	0.01889124	0.22104103	nonDEG	-0.1032719	0.86613781	1
-0.3082075	0.71330494	1	nonDEG	1.0473171	0.16703468	1
-0.7941872	0.02471555	0.2630517	nonDEG	-0.8074603	0.0685514	1
-0.7855459	0.00214604	0.04821374	DEG	-0.107881	0.7745423	1
0.45953444	0.08222223	0.52438049	nonDEG	0.17813319	0.72904237	1
-0.5662145	0.2199042	0.79942699	nonDEG	1.2706094	0.01286592	0.82858811
-0.7077356	0.00392012	0.07513316	nonDEG	-0.1953033	0.618793	1
1.29621406	2.27E-05	0.00137827	DEG	0.76351242	0.05851219	1
1.26896819	5.74E-06	0.0004472	DEG	0.47773568	0.22104829	1
-0.192618	0.48151404	0.9944442	nonDEG	-0.1880446	0.62982746	1
0.37483258	0.67721698	1	nonDEG	1.57212775	0.01226605	0.81648099

0.83771763	0.07411302	0.49784845	nonDEG	0.1430383	0.78038441	1
-1.0799641	0.02874515	0.29080021	nonDEG	-1.1627599	0.0937541	1
-0.5557625	0.02839007	0.28839265	nonDEG	-0.0232393	0.94957907	1
-0.4405344	0.25694617	0.83898637	nonDEG	-0.5353322	0.33271628	1
-0.0620899	0.83194971	1	nonDEG	-0.3002086	0.45148215	1
-1.9863466	7.17E-09	1.48E-06	DEG	-0.2148102	0.75055922	1
0.48468254	0.09020816	0.54766713	nonDEG	0.37991194	0.30040939	1
0.81644305	0.00215846	0.04838117	DEG	-0.085675	0.81594199	1
-0.3361295	0.20727705	0.78320256	nonDEG	0.11344155	0.81475141	1
-0.8507969	0.00192584	0.04467463	DEG	-0.4677148	0.24854764	1
0.03023552	0.95908391	1	nonDEG	1.21078706	0.06391285	1
1.71719176	0.14349395	0.67589095	nonDEG	1.00770706	0.28354847	1
-1.3951978	0.01285771	0.17221029	nonDEG	-0.4617737	0.53479324	1
-2.2152697	3.72E-07	4.12E-05	DEG	-1.1890963	0.14881592	1
0.77726458	0.00228208	0.05029026	nonDEG	-0.130394	0.73177576	1
0.40282992	0.10735064	0.59980305	nonDEG	0.54964245	0.16138356	1
-0.7669717	0.01865253	0.2189195	nonDEG	-0.2696383	0.55602218	1
1.3876241	1.31E-05	0.00087189	DEG	0.26004108	0.52526205	1
-0.5284674	0.28265505	0.86438122	nonDEG	-1.2856822	0.04840924	1
0.75812727	0.12483518	0.64346707	nonDEG	0.28353417	0.64456429	1
-0.0994122	0.91597626	1	nonDEG	1.42079463	0.15416972	1
-0.6725127	0.01955824	0.22611878	nonDEG	-0.2288349	0.5778864	1
0.95356427	0.02004262	0.2294327	nonDEG	0.46439511	0.29435071	1
-0.2098526	0.41974073	0.96294915	nonDEG	-0.1999719	0.59022192	1
-2.7030116	0.01379992	0.17983881	nonDEG	1.68202767	0.38500683	1
-1.4430434	0.0007129	0.02087336	DEG	-0.3962759	0.48617852	1
1.71017996	6.82E-08	1.00E-05	DEG	-0.3292738	0.41108405	1
1.8191839	0.05641908	0.43212952	nonDEG	1.20589201	0.1341935	1
-3.8608404	7.77E-08	1.12E-05	DEG	0.58553564	0.6750646	1
0.1547767	0.65249062	1	nonDEG	0.51955961	0.21320841	1
0.18070232	0.45748531	0.98449693	nonDEG	0.35917539	0.34246631	1
0.40576936	0.11135001	0.60975417	nonDEG	0.33588666	0.36109469	1
-0.6361038	0.05427755	0.42171463	nonDEG	-0.4143496	0.31836643	1
-0.0911344	0.90962579	1	nonDEG	1.83678549	0.00566374	0.58916916
-0.7618504	0.34428227	0.91869633	nonDEG	-1.8402034	0.07016133	1
0.53351314	0.11308591	0.61468538	nonDEG	0.12161683	0.79003446	1
-2.0528576	2.26E-11	9.91E-09	DEG	0.21421386	0.6276011	1
1.11361311	0.10230372	0.58344038	nonDEG	0.24799596	0.71225388	1
0.19109591	0.78353882	1	nonDEG	-3.8172488	0.00017019	0.06208357
-0.3584204	0.23669578	0.81852308	nonDEG	-0.3926075	0.39150971	1
2.05345681	0.0140866	0.18284636	nonDEG	0.15498226	0.87647269	1
-0.4839445	0.23428643	0.81498817	nonDEG	1.13735562	0.01121987	0.79590774
0.98119163	0.00066324	0.0198628	DEG	0.18570252	0.60872072	1

0.60983904	0.10909523	0.60426235	nonDEG	0.60598893	0.14722022	1
0.38147907	0.19023542	0.7571097	nonDEG	0.67585572	0.09332502	1
0.44265306	0.13542404	0.66192067	nonDEG	0.81612666	0.03998117	1
0.75588548	0.26384691	0.847847	nonDEG	0.53638137	0.41381659	1
1.32353092	2.31E-06	0.00020391	DEG	0.2723741	0.49239344	1
2.86210174	1.88E-06	0.00017049	DEG	-0.7578476	0.19286675	1
-0.4095543	0.09282535	0.55553681	nonDEG	-0.0120878	0.97464673	1
-0.4579299	0.2095917	0.78582018	nonDEG	-0.4346816	0.38878315	1
-2.8541008	0.04080542	0.35848132	nonDEG	-1.9796261	0.33452266	1
0.93338029	0.00134672	0.03381419	DEG	0.42191757	0.30122268	1
0.2023584	0.41633145	0.96166196	nonDEG	0.37254522	0.38650137	1
0.60958234	0.23380729	0.81412023	nonDEG	0.50564335	0.34910617	1
0.39308709	0.25685427	0.83898637	nonDEG	0.53139378	0.18051268	1
0.39014795	0.68421322	1	nonDEG	0.50447278	0.55900233	1
-1.8644855	1.02E-11	4.94E-09	DEG	-0.7598323	0.06578613	1
-0.1039512	0.72283816	1	nonDEG	0.66398166	0.06973108	1
-0.3514402	0.52705525	1	nonDEG	1.08345189	0.07321883	1
-0.2482121	0.41237536	0.95908664	nonDEG	-0.5618896	0.17670833	1
-0.8502253	0.00099685	0.02670257	DEG	-0.1258451	0.78326302	1
1.54360862	1.91E-05	0.0011999	DEG	1.18621659	0.14608383	1
1.88652361	0.00624872	0.10403281	nonDEG	0.59516691	0.44172904	1
-0.4254602	0.32034382	0.90340087	nonDEG	-0.9907406	0.07769324	1
1.16016128	0.00164107	0.03947003	DEG	0.11096839	0.80719319	1
-0.5178803	0.1169094	0.62512815	nonDEG	-0.1874719	0.6295209	1
0.91627963	0.04301645	0.36920155	nonDEG	0.37852234	0.44882436	1
-1.7155496	6.14E-08	9.18E-06	DEG	-0.6791432	0.26775951	1
-1.0471198	0.00046086	0.01509189	DEG	0.03753799	0.9258652	1
1.61640755	9.69E-11	3.53E-08	DEG	0.34055008	0.39112252	1
0.08374319	0.87927656	1	nonDEG	0.51892762	0.33016365	1
-0.3323568	0.18001376	0.74514618	nonDEG	-0.1157054	0.76110954	1
0.63966941	0.47365959	0.99070103	nonDEG	0.29684114	0.74425995	1
0.55275623	0.34721305	0.91879594	nonDEG	1.65751713	0.02664251	1
-0.5975533	0.02297719	0.25108593	nonDEG	0.16127599	0.78813562	1
-0.3492092	0.51920477	1	nonDEG	1.39573431	0.00689473	0.65687369
0.39489082	0.10453036	0.59178253	nonDEG	0.29551647	0.46748706	1
0.4917532	0.14515075	0.67913112	nonDEG	0.35933679	0.37359011	1
-1.5540272	0.00219728	0.0490788	DEG	-0.9759714	0.19813593	1
0.41984488	0.13983247	0.66813245	nonDEG	0.47292294	0.20667034	1
-1.0516394	6.81E-05	0.00322694	DEG	-0.0977385	0.81095782	1
0.05125521	0.83875046	1	nonDEG	0.21870561	0.54189855	1
-0.5662515	0.03877898	0.34784104	nonDEG	-0.4168514	0.27913999	1
1.04566966	0.0602977	0.44791923	nonDEG	0.30087298	0.60802996	1
-1.4692965	0.00272148	0.05722682	nonDEG	-1.3530275	0.07408151	1

-0.1268994	0.62354329	1	nonDEG	-0.0878879	0.82515476	1
0.81036404	0.00272903	0.05734467	nonDEG	0.1704333	0.64760782	1
0.22927007	0.78103126	1	nonDEG	0.84733432	0.32106126	1
-0.0291395	0.96091517	1	nonDEG	0.74142544	0.16915663	1
-1.537849	0.00469817	0.08485241	nonDEG	0.30287736	0.70410966	1
-0.4860508	0.09649981	0.5670375	nonDEG	-0.6360634	0.09213577	1
1.50545552	1.69E-07	2.16E-05	DEG	-0.3393752	0.35982682	1
-1.5928317	0.05725293	0.43563425	nonDEG	0.36426267	0.77511902	1
0.26445393	0.59583259	1	nonDEG	0.50396173	0.33812914	1
-0.4413141	0.07201582	0.49000913	nonDEG	-0.0574228	0.88185142	1
-0.3626409	0.35767965	0.92075237	nonDEG	-0.1187576	0.81239434	1
0.24924006	0.61489522	1	nonDEG	0.29082803	0.60340521	1
1.04959453	0.00430743	0.08035893	nonDEG	-0.2985905	0.55051691	1
0.76176671	0.00557951	0.09577556	nonDEG	0.59544381	0.10048881	1
-0.1001094	0.89018638	1	nonDEG	1.85358766	0.00747965	0.67511746
-1.4684133	0.00094982	0.0257943	DEG	-0.828488	0.20875649	1
-0.6058035	0.5318017	1	nonDEG	-1.6244434	0.15207678	1
1.11445969	0.00757198	0.11996367	nonDEG	-0.092112	0.81988999	1
-0.9278371	0.12544298	0.64431252	nonDEG	-0.301904	0.72444573	1
-0.543057	0.03916813	0.34952124	nonDEG	-0.2407683	0.57293292	1
2.49977796	0.07538828	0.50302707	nonDEG	-0.0743461	0.96936724	1
-0.6715743	0.02730403	0.28105013	nonDEG	0.08365223	0.86130815	1
0.10645456	0.87082889	1	nonDEG	1.02953012	0.08697388	1
-0.7113773	0.6148117	1	nonDEG	1.79668829	0.22427542	1
-0.6691454	0.00921303	0.13766588	nonDEG	-0.1192224	0.78502974	1
-0.7386236	0.10209565	0.58261617	nonDEG	-1.1821709	0.12743321	1
-0.4345624	0.61666093	1	nonDEG	-1.2262194	0.23112423	1
-0.602002	0.02509439	0.26586493	nonDEG	-0.9606892	0.01119797	0.79590774
2.15962369	0.01751722	0.20980046	nonDEG	-4.76818	2.20E-06	0.00412442
1.10732748	0.0010959	0.02896059	DEG	-0.470893	0.26657606	1
1.1897315	0.05857268	0.44089009	nonDEG	-0.0625444	0.91809193	1
-1.1016315	0.00725571	0.1160243	nonDEG	-0.4449713	0.38729788	1
-2.7361052	6.72E-15	5.71E-12	DEG	-1.2009933	0.00485436	0.55667563
0.51740957	0.14372525	0.67633117	nonDEG	0.19547425	0.63589764	1
-1.3328081	0.00342162	0.06847966	nonDEG	-0.9081995	0.17338291	1
-0.9122708	0.00021911	0.008259	DEG	0.15885415	0.77591505	1
-1.0592866	0.02617896	0.27367988	nonDEG	0.45188092	0.42244667	1
-1.1893924	0.00399559	0.07574128	nonDEG	-0.4619353	0.41330791	1
-0.063465	0.79801249	1	nonDEG	-0.9372237	0.01047516	0.77777211
-2.0578562	5.24E-11	2.06E-08	DEG	-0.5143081	0.47982756	1
-1.2309056	0.00034537	0.01208956	DEG	0.14532109	0.80448711	1
-0.5571235	0.04346883	0.37153991	nonDEG	-0.3796093	0.30689514	1
1.01153338	0.05035204	0.4060021	nonDEG	-0.4123803	0.48102413	1

-0.4590802	0.41856629	0.96257729	nonDEG	1.31301842	0.02090409	1
0.38721442	0.38584925	0.94210008	nonDEG	0.4737042	0.35811794	1
-1.4743962	0.00016162	0.00643821	DEG	-0.8080724	0.22943166	1
-2.6614972	0.00015773	0.00631741	DEG	-0.9960212	0.31877652	1
-0.0232941	0.95266074	1	nonDEG	0.90255028	0.04878912	1
-0.9154278	0.00206175	0.04701458	DEG	-0.4269016	0.31400686	1
0.34402552	0.74041259	1	nonDEG	1.29649769	0.22441112	1
0.14773371	0.61511048	1	nonDEG	0.91426088	0.01495952	0.8820345
-0.5230461	0.12205748	0.63647417	nonDEG	-0.6988349	0.13662558	1
-0.4604654	0.14279456	0.67492321	nonDEG	-0.7653547	0.06794725	1
-1.4765549	0.00047492	0.01536385	DEG	-0.1150639	0.84340019	1
1.56757952	0.00583644	0.09897412	nonDEG	0.27102115	0.65257959	1
1.33227766	1.07E-07	1.46E-05	DEG	-0.1000066	0.79148694	1
-0.7029823	0.11481618	0.61909498	nonDEG	-0.7692006	0.23074165	1
0.14605675	0.84064806	1	nonDEG	1.23820242	0.06385241	1
0.13458408	0.77250417	1	nonDEG	0.41347838	0.45432984	1
-1.141964	0.00092905	0.02539484	DEG	-0.1528546	0.80671636	1
2.3223352	0.00122382	0.03154832	DEG	-3.793209	0.00033382	0.10757186
-0.4012857	0.17885657	0.744789	nonDEG	-0.7551608	0.08780601	1
1.42586216	1.24E-06	0.00012004	DEG	0.98190795	0.02818798	1
0.27536411	0.80818744	1	nonDEG	1.06719884	0.29325975	1
1.38276066	0.04126193	0.36087637	nonDEG	0.45618398	0.56730456	1
-0.8874844	0.00089602	0.02460777	DEG	-0.5437209	0.13992432	1
0.65281365	0.10174476	0.58137887	nonDEG	1.2359764	0.00819752	0.70001992
-0.5427333	0.06174988	0.45469104	nonDEG	-0.1597735	0.69569198	1
-0.5998243	0.42367276	0.96604312	nonDEG	1.27824181	0.06637617	1
0.28965336	0.57103426	1	nonDEG	1.14946978	0.03406974	1
1.16352796	1.07E-05	0.00074088	DEG	0.35156401	0.36630551	1
-0.6381463	0.50478232	1	nonDEG	-1.0027479	0.36255516	1
-0.4758972	0.05279869	0.4164841	nonDEG	-0.13489	0.72832107	1
0.59406049	0.32267265	0.90571702	nonDEG	0.58210887	0.35244952	1
0.1493977	0.85662919	1	nonDEG	1.21192002	0.17884399	1
-1.2796886	0.00024642	0.00906799	DEG	-2.2674266	0.00184515	0.33121517
-0.8875069	0.01701055	0.20607968	nonDEG	-0.4717392	0.37169888	1
-1.9126029	0.17194483	0.73059581	nonDEG	3.49649669	0.03107016	1
1.22113077	1.14E-05	0.00077033	DEG	-0.3109532	0.39924182	1
-0.0562435	0.92933516	1	nonDEG	0.8401351	0.22866525	1
0.34374282	0.17138055	0.72989601	nonDEG	0.14724713	0.69710992	1
-1.1863807	1.09E-05	0.00074904	DEG	-0.0065366	0.98980282	1
1.52235159	1.41E-07	1.83E-05	DEG	-0.269365	0.49036927	1
1.22594374	0.03230094	0.31228481	nonDEG	0.02019432	0.98067586	1
0.53846855	0.55829454	1	nonDEG	-0.1569084	0.87205213	1
0.71209768	0.00635049	0.10520171	nonDEG	0.18008243	0.63608366	1

0.2782935	0.33720625	0.91869633	nonDEG	0.44797542	0.25242668	1
-0.18769	0.45171866	0.98329655	nonDEG	0.05826967	0.8731803	1
0.84272699	0.02647428	0.27530073	nonDEG	0.16014186	0.71245041	1
-0.6108892	0.64972125	1	nonDEG	-0.6936681	0.71907552	1
1.38565122	7.40E-06	0.00055552	DEG	-0.2597853	0.62261173	1
0.41805651	0.1780367	0.74368492	nonDEG	0.28219571	0.47336226	1
-0.3705016	0.21659477	0.79477343	nonDEG	-0.1292016	0.74894157	1
-1.0942704	0.00636964	0.10534603	nonDEG	-0.5028602	0.3691299	1
-0.7254058	0.00437877	0.08142707	nonDEG	-0.0699863	0.86803445	1
1.87462937	2.84E-10	9.08E-08	DEG	0.2988834	0.46215268	1
-0.5460258	0.18995066	0.75659012	nonDEG	0.61771801	0.48890876	1
1.73021496	0.00212697	0.04798161	DEG	-0.1404532	0.81358454	1
0.83572176	0.00564987	0.09664522	nonDEG	0.0275657	0.94340257	1
-2.4503282	1.49E-18	2.44E-15	DEG	0.9252453	0.11707915	1
0.152912	0.53541608	1	nonDEG	0.51174869	0.15630777	1
-0.413599	0.13400086	0.65914049	nonDEG	-0.2856884	0.45329282	1
2.32386758	0.09131914	0.5510019	nonDEG	0.54058153	0.60041503	1
-0.3460218	0.27981301	0.86183461	nonDEG	-0.6734944	0.10227813	1
-0.3503305	0.41832926	0.96254612	nonDEG	-0.3994953	0.47743555	1
0.57354859	0.12476935	0.64346707	nonDEG	0.58430575	0.17237628	1
1.12549024	2.55E-05	0.00148934	DEG	0.29630652	0.472633	1
2.83279068	2.27E-07	2.74E-05	DEG	1.85480334	0.0005595	0.14973747
0.82233458	0.43550849	0.97371721	nonDEG	1.61786915	0.11693295	1
-0.1362942	0.62385469	1	nonDEG	-0.7581172	0.05744707	1
1.30902745	1.10E-05	0.0007521	DEG	0.33538551	0.36129312	1
0.32549263	0.24624695	0.82791961	nonDEG	0.47156482	0.20062407	1
-1.992788	0.13075813	0.65446487	nonDEG	-4.0646698	0.27840674	1
-0.4723653	0.33536046	0.91869633	nonDEG	-2.0864022	0.0011654	0.24332051
-0.623839	0.01153143	0.16150043	nonDEG	-0.0872895	0.81773488	1
-0.4990899	0.3024669	0.88706147	nonDEG	-0.2746917	0.72678603	1
-0.7948884	0.37772286	0.93522146	nonDEG	-0.2025904	0.86618177	1
-0.567178	0.06272438	0.45832913	nonDEG	-0.363697	0.35594858	1
-1.2063273	0.15030432	0.68725272	nonDEG	-0.2508534	0.80675515	1
-0.1820553	0.78442903	1	nonDEG	0.51120482	0.40657038	1
-0.4929511	0.18468159	0.752504	nonDEG	-0.2170175	0.69481776	1
-1.271432	0.0754887	0.50324091	nonDEG	-0.1371765	0.90756162	1
1.53931777	4.60E-09	1.00E-06	DEG	0.14506741	0.70991235	1
-0.4249582	0.14510538	0.67913112	nonDEG	-0.9542106	0.02279573	1
0.93185273	0.09474681	0.56132341	nonDEG	0.92495829	0.11171534	1
1.64957824	0.05292884	0.41684651	nonDEG	0.06952966	0.92974296	1
-0.422775	0.10373567	0.58898253	nonDEG	-0.2509919	0.57608499	1
5.87954322	0.03665358	0.33741237	nonDEG	0.53301796	0.72799657	1
0.98247651	0.00025147	0.0091963	DEG	0.07645269	0.84289956	1

-1.1679683	0.02209639	0.24491556	nonDEG		-0.7385111	0.26620829	1
-2.7922808	0.05423694	0.42151037	nonDEG		2.96725146	0.0684258	1
1.10263949	0.09578743	0.56510742	nonDEG		0.45837938	0.42417678	1
0.42183232	0.36340144	0.92416624	nonDEG		-0.9081612	0.07523692	1
-0.0107499	0.98260702	1	nonDEG		-1.297367	0.01945008	0.98654907
0.73826826	0.01247745	0.16873447	nonDEG		0.28379051	0.46984615	1
0.66105328	0.04908592	0.40042859	nonDEG		0.26884963	0.52400446	1
-0.3751487	0.14084638	0.67020778	nonDEG		0.1595016	0.69693312	1
1.02818468	0.01547711	0.19413322	nonDEG		0.00783851	0.98978901	1
1.50065885	1.74E-06	0.00015952	DEG		0.30506696	0.45170481	1
-0.8834651	0.0657729	0.46863877	nonDEG		-0.8784109	0.15384579	1
0.91019681	0.23664268	0.81852308	nonDEG		0.21968871	0.763074	1
-0.2330017	0.51671485	1	nonDEG		-1.4305586	0.00416413	0.52387911
0.51512495	0.16191946	0.71251696	nonDEG		0.68624628	0.08758646	1
-1.9398309	0.00442997	0.08187808	nonDEG		0.42344764	0.62335763	1
1.01935586	0.00038177	0.01300817	DEG		0.32101255	0.38516497	1
-0.5948381	0.03844432	0.34568182	nonDEG		-0.6103217	0.1953241	1
3.72885988	0.00078013	0.02226206	DEG		-0.6908499	0.38895552	1
-0.7001897	0.18859221	0.75429997	nonDEG		-0.9022354	0.20084651	1
1.17212532	0.00386046	0.07457224	nonDEG		0.1486054	0.76713724	1
-0.2492273	0.48892504	0.99836609	nonDEG		0.76013618	0.07365036	1
0.85525743	0.05121526	0.40931023	nonDEG		0.40436952	0.36021357	1
0.47994234	0.14686638	0.68238624	nonDEG		0.85334499	0.03894069	1
1.3692257	0.00013711	0.00569311	DEG		-0.1794525	0.67097964	1
0.50473993	0.66604032	1	nonDEG		-0.036749	1	1
-0.6555699	0.02438177	0.26130459	nonDEG		0.12186824	0.74268422	1
1.25906243	0.01177508	0.16392237	nonDEG		-0.1916155	0.73026713	1
-0.4869048	0.06627677	0.47058314	nonDEG		-0.3537145	0.36782283	1
1.37417773	0.00033967	0.01193264	DEG		0.13338156	0.78485893	1
1.44380815	5.36E-05	0.00268647	DEG		0.20644772	0.61024283	1
-0.8370067	0.00082726	0.023172	DEG		0.06676768	0.85801802	1
0.3483993	0.20661344	0.78183639	nonDEG		0.24422122	0.5154539	1
0.48461786	0.1117285	0.61091667	nonDEG		0.57500519	0.11896878	1
-0.620913	0.0173544	0.20869945	nonDEG		-0.0992039	0.8654135	1
0.69092755	0.01641362	0.20111157	nonDEG		0.57120405	0.11248891	1
1.20732173	0.00034467	0.01207963	DEG		-0.1809565	0.6392428	1
-0.337008	0.25242231	0.83371668	nonDEG		-0.1920157	0.64200642	1
-0.1956302	0.45036494	0.98329655	nonDEG		0.25048864	0.50356154	1
-0.6383273	0.0126392	0.17021291	nonDEG		-0.1902843	0.65924047	1
0.29109761	0.67305092	1	nonDEG		1.58429147	0.00455988	0.53910928
-0.4166983	0.10393938	0.58968421	nonDEG		-0.3096884	0.40036193	1
-0.0728995	0.89585867	1	nonDEG		0.833211	0.1800234	1
-1.5472454	8.02E-08	1.15E-05	DEG		-0.3419696	0.64357772	1

-0.3761613	0.17877694	0.74462568	nonDEG	-0.2826532	0.45819764	1
-1.3686868	0.36087578	0.92354131	nonDEG	2.0565245	0.2907534	1
1.23924808	5.81E-06	0.00045106	DEG	0.39141631	0.43618548	1
0.97209438	0.02980569	0.29683682	nonDEG	0.24270384	0.6216203	1
-0.3986461	0.13495239	0.6610751	nonDEG	-0.0859721	0.81996871	1
-0.9619144	0.07253309	0.4914105	nonDEG	-0.1235078	0.87603616	1
1.13692977	1.78E-05	0.00113193	DEG	0.08174401	0.8239311	1
-0.839359	0.00967074	0.14249091	nonDEG	-0.2699029	0.58241671	1
0.26960361	0.34570108	0.91869633	nonDEG	0.42969784	0.24187349	1
-0.4643898	0.25556113	0.83779145	nonDEG	-1.3672317	0.00318845	0.4469745
-0.5259948	0.07656455	0.50685493	nonDEG	-0.7758373	0.09544707	1
-0.1998952	0.469044	0.98763939	nonDEG	-0.3845146	0.37900491	1
-1.1206676	3.62E-05	0.00198055	DEG	-0.144109	0.69292696	1
0.15150356	0.55331459	1	nonDEG	0.32846806	0.39489306	1
-0.0700843	0.80606028	1	nonDEG	-0.4635839	0.23342151	1
1.02504099	0.00390929	0.07507213	nonDEG	0.79447629	0.0589012	1
0.1005924	0.79745632	1	nonDEG	-0.3014915	0.59635414	1
0.25955575	0.42985339	0.97110382	nonDEG	0.31747676	0.42284145	1
0.17476389	0.67723212	1	nonDEG	0.76303915	0.11390959	1
0.06071641	0.83503135	1	nonDEG	0.78095827	0.03597291	1
0.4082431	0.16115078	0.71104884	nonDEG	0.14227013	0.70091564	1
-1.2747206	0.00026815	0.00975788	DEG	-0.4063613	0.44742225	1
-0.4145788	0.66397267	1	nonDEG	1.48319362	0.13981572	1
1.68552826	0.0069078	0.11185852	nonDEG	-0.2457197	0.70809814	1
-0.4668882	0.06521077	0.46677686	nonDEG	-0.3983991	0.31830475	1
-0.3518101	0.15102578	0.68824233	nonDEG	0.28523133	0.58150787	1
0.71245019	0.07062022	0.48619944	nonDEG	0.42739956	0.38171571	1
-0.5880086	0.06640941	0.47120429	nonDEG	-0.1610799	0.70666079	1
-1.0525589	0.03214288	0.31147271	nonDEG	-0.2476357	0.73296568	1
1.9300986	0.01925632	0.2233886	nonDEG	0.57902012	0.51863597	1
0.13814153	0.82446848	1	nonDEG	1.00420109	0.11136959	1
-0.4739646	0.07464709	0.50034968	nonDEG	-0.3771354	0.45023665	1
-0.3011851	0.21902774	0.79791581	nonDEG	-0.2492125	0.51545225	1
-1.1019828	2.04E-05	0.00126416	DEG	-0.8835572	0.05516191	1
1.23325861	7.08E-07	7.31E-05	DEG	-0.1961215	0.58669863	1
-0.3083739	0.21475811	0.79425429	nonDEG	-0.0316652	0.9343739	1
-0.8570358	0.02046752	0.23246267	nonDEG	0.03430213	0.94988647	1
0.73076131	0.01310486	0.17425207	nonDEG	0.23572261	0.56223335	1
0.37876458	0.48579636	0.99670526	nonDEG	0.76460938	0.20904369	1
-0.7682914	0.00939139	0.13942164	nonDEG	-0.5012472	0.22908621	1
5.18751503	0.0808843	0.52035682	nonDEG	0.90352577	0.56461174	1
0.24099418	0.47823059	0.99305902	nonDEG	0.7397399	0.06662571	1
-0.8435624	0.00395161	0.07536309	nonDEG	-0.6907824	0.0861358	1

1.75021314	0.2481992	0.82990493	nonDEG	1.16756499	0.40253277	1
-0.8465753	0.00234564	0.05126445	nonDEG	-0.578398	0.25038677	1
-0.5824968	0.04764216	0.3929777	nonDEG	-0.2214261	0.58782055	1
-0.415246	0.09869174	0.57226436	nonDEG	-0.3911583	0.29389519	1
-1.4007426	0.00880549	0.13348337	nonDEG	-0.5440481	0.50218541	1
1.12406973	1.48E-05	0.00096936	DEG	0.5489296	0.13783081	1
0.31133551	0.28028412	0.86186717	nonDEG	0.36056492	0.4423759	1
2.16419417	8.68E-17	1.16E-13	DEG	0.04098405	0.91455241	1
0.50899553	0.29554367	0.87965245	nonDEG	0.00869236	0.98627382	1
0.49559324	0.29214608	0.87510058	nonDEG	0.33311212	0.55052255	1
1.06370096	0.00819219	0.12656241	nonDEG	0.33474845	0.55727457	1
-0.3576412	0.40184267	0.95307326	nonDEG	-1.4678501	0.01850537	0.95785021
0.12386885	0.81293931	1	nonDEG	0.60029137	0.20615429	1
-1.2532351	0.00835509	0.1280906	nonDEG	-2.2722281	0.00892672	0.73201603
0.57298054	0.15047364	0.68725272	nonDEG	0.38934192	0.444348	1
-0.3253034	0.79887465	1	nonDEG	0.82263231	0.45063179	1
0.79112558	0.00890168	0.13438799	nonDEG	0.02003947	0.95875102	1
-0.9820996	0.09957828	0.57443019	nonDEG	-1.2059736	0.17276639	1
0.66395173	0.02248229	0.24797903	nonDEG	0.2995639	0.4202892	1
0.61953161	0.29343172	0.87663248	nonDEG	0.40875148	0.5180318	1
-0.7781193	0.00413108	0.07785846	nonDEG	-0.3656195	0.44728665	1
0.52288239	0.429668	0.97110382	nonDEG	0.22302031	0.70813109	1
0.39099869	0.13082335	0.65446487	nonDEG	0.67021218	0.09235679	1
1.02657429	0.02561456	0.26959152	nonDEG	0.16935597	0.75548789	1
1.04949222	0.00012615	0.00535132	DEG	0.56396044	0.1173229	1
-0.7126765	0.30144296	0.88626579	nonDEG	1.70144244	0.00533287	0.57843438
1.50394672	0.01134136	0.15967396	nonDEG	1.32806455	0.06080568	1
-0.7467922	0.02384649	0.25768009	nonDEG	-1.4541297	0.00857036	0.71881114
-0.598283	0.04510999	0.37991238	nonDEG	0.15129658	0.7339031	1
-0.4886007	0.26684016	0.85072996	nonDEG	-1.0470181	0.06383055	1
-0.7428135	0.01591715	0.19754846	nonDEG	0.23704007	0.56124496	1
1.58825589	2.00E-09	4.87E-07	DEG	-0.2641009	0.47829899	1
2.19403481	0.10625863	0.59731676	nonDEG	0.65942829	0.60060733	1
0.59153348	0.02024806	0.23077141	nonDEG	0.48716932	0.21613574	1
-1.8132436	7.71E-06	0.00056865	DEG	-0.2528756	0.63767346	1
0.23648355	0.39213601	0.946931	nonDEG	0.69025551	0.08374969	1
0.56713645	0.07240095	0.49099555	nonDEG	0.5327604	0.15945455	1
-0.8029968	0.06004544	0.4468115	nonDEG	-0.2709627	0.6585595	1
-0.985919	0.00801787	0.12508453	nonDEG	-0.8332611	0.07459584	1
-1.6876203	9.11E-07	9.19E-05	DEG	-0.4341162	0.38450552	1
-0.0434992	0.91716138	1	nonDEG	0.62941796	0.17362632	1
2.01108277	0.00688851	0.11160743	nonDEG	-0.0481694	0.95945535	1
-0.386257	0.20622938	0.78096048	nonDEG	-0.2324813	0.58083587	1

-0.2222732	0.39175217	0.94686517	nonDEG	-0.0991058	0.78477067	1
-3.1271615	1.74E-18	2.70E-15	DEG	-0.1128204	0.90531582	1
-0.6274571	0.05361802	0.41969541	nonDEG	-1.0250754	0.03385609	1
-1.0481794	0.00181838	0.04268833	DEG	-0.1562823	0.74613086	1
0.68529977	0.01047594	0.15140639	nonDEG	0.58339688	0.10690136	1
-1.0600962	3.63E-05	0.00198139	DEG	-0.3280815	0.51282391	1
-0.4566177	0.09847766	0.57169864	nonDEG	-0.1208258	0.7454827	1
1.58761516	4.28E-09	9.40E-07	DEG	0.30340645	0.39714038	1
1.90730824	1.19E-14	9.72E-12	DEG	0.81566597	0.06206742	1
0.35204387	0.16712352	0.72203543	nonDEG	-0.7714756	0.04717636	1
-0.0194236	0.9408979	1	nonDEG	0.42502614	0.70852558	1
1.17490477	0.22920338	0.80924485	nonDEG	1.18148385	0.20701693	1
1.14042304	0.0502433	0.4058991	nonDEG	0.42728243	0.46511139	1
-2.1088334	2.98E-15	2.83E-12	DEG	0.177829	0.70156134	1
1.01263211	0.00010499	0.00458557	DEG	0.05576859	0.8794405	1
-1.2445877	0.00012188	0.00521499	DEG	0.15283752	0.81374712	1
0.76878594	0.44480336	0.97969222	nonDEG	1.05402322	0.30603582	1
1.20130011	0.00022792	0.00852217	DEG	0.07411422	0.85963921	1
0.50274554	0.50594495	1	nonDEG	1.1718291	0.18382822	1
-5.8394512	0.0347397	0.32684625	nonDEG	3.28802144	0.63854406	1
-0.6512746	0.00670263	0.10925731	nonDEG	-0.4042475	0.26490748	1
0.3844376	0.64919066	1	nonDEG	0.79724704	0.34396285	1
-0.3141903	0.25569659	0.83785246	nonDEG	-0.8185474	0.07253343	1
-0.4153697	0.09484032	0.56143255	nonDEG	-0.2711225	0.47157375	1
-2.6243536	3.03E-06	0.00025618	DEG	-2.1119317	0.02743758	1
-1.171291	0.00112914	0.02960005	DEG	-1.0229173	0.05949121	1
-0.3738025	0.12556217	0.64431252	nonDEG	-0.0606215	0.88728684	1
0.90140341	0.00240256	0.05223713	nonDEG	-0.0718967	0.85256822	1
-0.8370407	0.01623472	0.19980521	nonDEG	-0.3551178	0.45760864	1
1.75744526	7.71E-05	0.00357614	DEG	-0.0294127	0.95368689	1
-0.7549255	0.00268449	0.05669206	nonDEG	-0.8115625	0.04489111	1
0.23359321	0.3453607	0.91869633	nonDEG	0.21650119	0.56984026	1
-0.3520711	0.61879191	1	nonDEG	1.46844818	0.09229564	1
0.78023248	0.15673896	0.70140512	nonDEG	0.94486287	0.03750925	1
-0.556046	0.29621597	0.88097874	nonDEG	0.04790794	0.95019803	1
-0.6736008	0.00592352	0.09998996	nonDEG	-0.0306959	0.93922457	1
1.15660665	0.0053246	0.09263475	nonDEG	0.60521924	0.20483792	1
0.70313437	0.13896245	0.66717926	nonDEG	0.53341962	0.27038731	1
0.81703713	0.04536588	0.38135631	nonDEG	1.14191076	0.07475086	1
-0.6667498	0.20490017	0.77892413	nonDEG	-1.4114083	0.04430476	1
0.4695386	0.35541811	0.91917773	nonDEG	0.60125444	0.30320924	1
-0.096972	0.82871372	1	nonDEG	-0.9969766	0.07139689	1
1.0393707	0.00920336	0.13766588	nonDEG	0.40902513	0.38215409	1

-1.5984351	5.10E-07	5.36E-05	DEG	-0.2689355	0.59340728	1
-0.873083	0.06969335	0.48325832	nonDEG	-0.2303117	0.70301053	1
-0.6875202	0.02093974	0.23620581	nonDEG	0.18665314	0.68343575	1
-0.1617553	0.74576097	1	nonDEG	0.58104353	0.31813658	1
-0.1427064	0.81286676	1	nonDEG	-1.4305374	0.06959963	1
-0.7370461	0.02537585	0.26766026	nonDEG	0.09024975	0.83609951	1
-0.8238149	0.00887652	0.13421469	nonDEG	-0.7778203	0.07664811	1
1.80945753	9.72E-11	3.53E-08	DEG	-0.2723666	0.49507424	1
0.4188779	0.24513358	0.8265359	nonDEG	-0.9985461	0.03925966	1
-2.5912438	1.93E-12	1.01E-09	DEG	-0.3704632	0.63120093	1
-1.4471545	0.09842346	0.57152136	nonDEG	-0.9961192	0.35994923	1
0.80588136	0.04915109	0.40070868	nonDEG	1.05993175	0.01561771	0.89863185
-6.0623649	3.05E-11	1.23E-08	DEG	1.49956725	0.26084429	1
-1.6312347	0.00018963	0.00734531	DEG	-0.7344268	0.05308163	1
1.32633904	3.46E-06	0.00028765	DEG	0.40539928	0.27414709	1
-0.4823952	0.17564657	0.73940089	nonDEG	-0.4273296	0.37442395	1
1.12643133	0.00928429	0.13824992	nonDEG	-0.1808859	0.70978517	1
-0.479543	0.19841766	0.77062507	nonDEG	-0.2955587	0.56453097	1
-0.3659079	0.16571733	0.71880838	nonDEG	-0.366644	0.39091057	1
0.1665578	0.78465069	1	nonDEG	1.3757496	0.02065183	1
-1.8347416	4.22E-10	1.21E-07	DEG	-0.404544	0.5298458	1
-0.1939426	0.5517179	1	nonDEG	-0.6906215	0.11848317	1
-1.4623425	0.00019923	0.0076368	DEG	0.08872731	0.8794652	1
0.77953431	0.19595661	0.76693341	nonDEG	0.44827813	0.47822371	1
-1.2639168	0.06221975	0.45672559	nonDEG	-2.3726443	0.00490831	0.5578981
3.5728182	0.16751427	0.7227101	nonDEG	0.40255498	0.84047181	1
0.22638306	0.38027893	0.93639703	nonDEG	0.39751731	0.32863134	1
-4.1214282	0.01579622	0.19679425	nonDEG	4.52873715	0.00870868	0.72219089
0.88665997	0.00241504	0.05243102	nonDEG	0.2963887	0.49681134	1
-0.1020333	0.69095317	1	nonDEG	-0.4940321	0.1806122	1
-0.3417162	0.2383487	0.82044546	nonDEG	-0.3618928	0.34254528	1
0.54782159	0.24139465	0.82326427	nonDEG	0.59891055	0.25078403	1
-0.2968477	0.56852757	1	nonDEG	0.91296478	0.09390776	1
-0.1781795	0.61409357	1	nonDEG	-0.637665	0.17524619	1
0.39708111	0.15181253	0.68969277	nonDEG	0.75684041	0.04082079	1
0.98103911	0.57830249	1	nonDEG	0.61506952	0.69087212	1
0.65181629	0.18699585	0.75310345	nonDEG	1.14001213	0.02570472	1
-0.5117567	0.09334381	0.55728017	nonDEG	0.18084951	0.68375747	1
-1.5704444	4.49E-05	0.00232879	DEG	0.18234417	0.78777	1
0.38781351	0.16958715	0.72663669	nonDEG	0.51964127	0.18491962	1
-0.5789853	0.02440396	0.26134168	nonDEG	-0.1183158	0.8218905	1
0.68538084	0.04157445	0.36234817	nonDEG	0.42502628	0.25536089	1
-0.7771673	0.00158813	0.03844821	DEG	0.07681148	0.84890649	1

-0.7795072	0.19570795	0.76655752	nonDEG	-1.5286214	0.06380377	1
-0.3363163	0.26280249	0.84706619	nonDEG	-0.4827673	0.23582207	1
1.02552932	0.06371523	0.46201023	nonDEG	0.34411782	0.57735731	1
-0.5679009	0.1362463	0.66329662	nonDEG	-0.6470875	0.16412953	1
-0.4566399	0.06729655	0.47405418	nonDEG	0.23677737	0.60831723	1
-0.4306712	0.07563932	0.50367471	nonDEG	-0.107279	0.77614821	1
2.00480619	4.81E-09	1.03E-06	DEG	1.74407974	0.00039025	0.11731473
1.15751363	0.01517936	0.19178765	nonDEG	0.38162762	0.55170137	1
-1.6694673	4.78E-09	1.03E-06	DEG	-1.6123972	0.0012793	0.26336648
-0.9682789	0.00022412	0.00841549	DEG	0.14407655	0.76830832	1
-0.5982065	0.01782534	0.21271188	nonDEG	0.03734445	0.93602422	1
-0.4402248	0.10999175	0.60566492	nonDEG	-0.1281619	0.74578262	1
-0.1680945	0.79851048	1	nonDEG	-0.3173447	0.7745541	1
1.52558946	1.19E-09	2.99E-07	DEG	-0.1987159	0.58291504	1
0.56868707	0.42207237	0.96420737	nonDEG	0.91775435	0.15720446	1
-1.6572134	8.72E-05	0.00397163	DEG	0.13986622	0.81659272	1
0.10536639	0.67831123	1	nonDEG	-0.0800149	0.83076751	1
1.33824174	0.01532673	0.19282211	nonDEG	0.73982503	0.37076445	1
-0.6898585	0.03154007	0.30749364	nonDEG	-0.2065961	0.65399987	1
-0.8553777	0.34593981	0.91869633	nonDEG	-1.6076621	0.20296791	1
-1.7622568	0.03283228	0.31514492	nonDEG	-1.4999223	0.24014623	1
-1.4733463	0.01193096	0.16515005	nonDEG	0.79283471	0.24655282	1
0.35682875	0.15338997	0.69343478	nonDEG	0.50914453	0.15752566	1
-0.0533789	0.90679916	1	nonDEG	-1.9519863	0.00103305	0.22695466
1.73143027	1.43E-05	0.00094297	DEG	0.20658842	0.62293585	1
0.5688591	0.16272879	0.714154	nonDEG	1.09635457	0.00974638	0.7570543
0.3358376	0.62132008	1	nonDEG	0.69786261	0.28951641	1
-0.6084499	0.01642198	0.20111157	nonDEG	0.17214608	0.74317903	1
2.18127265	2.72E-05	0.00157242	DEG	1.21830948	0.05396241	1
1.99113135	0.01010779	0.14706146	nonDEG	0.39118829	0.67909927	1
0.70490207	0.04036209	0.35647711	nonDEG	0.25926967	0.54957659	1
0.48971156	0.08980009	0.54766713	nonDEG	0.24210125	0.51405612	1
-0.3066111	0.33729224	0.91869633	nonDEG	-1.4768675	0.03208347	1
0.88759565	0.01347014	0.17663587	nonDEG	0.36074058	0.41802813	1
0.8978867	0.03454626	0.32565073	nonDEG	0.82843973	0.06816104	1
-0.4942485	0.45748558	0.98449693	nonDEG	1.7296296	0.00407558	0.52015192
1.00633497	0.00031656	0.01123456	DEG	0.37764325	0.30181043	1
-0.5614689	0.07379264	0.49665784	nonDEG	-0.1767079	0.70892417	1
-4.670663	0.22218956	0.80226969	nonDEG	7.16541913	0.00033617	0.10757186
-0.8337708	0.02385197	0.25768009	nonDEG	-0.9836191	0.05700557	1
0.39817459	0.36993798	0.92862044	nonDEG	0.53790503	0.27434167	1
-0.6900951	0.31495426	0.89811491	nonDEG	1.33951703	0.01538888	0.89532248
-0.9835853	0.36824884	0.92756771	nonDEG	-4.0998833	0.01331392	0.84289973

-0.8280936	0.00093488	0.02553045	DEG		0.25380485	0.6159672	1
-0.2592081	0.31246741	0.89535767	nonDEG		-0.2359728	0.54190376	1
-1.1138771	0.04381014	0.37347484	nonDEG		-0.5563729	0.53767902	1
0.15895065	0.52554665	1	nonDEG		0.54441674	0.17543846	1
-0.5818128	0.03756786	0.3420995	nonDEG		-0.3394284	0.3449951	1
-0.059728	0.94737964	1	nonDEG		-0.9252511	0.26952641	1
1.53582564	2.39E-05	0.00143006	DEG		-0.3289875	0.43030715	1
1.11628342	0.00088563	0.02441198	DEG		0.2403795	0.5614321	1
6.4816787	0.0018676	0.04353153	DEG		-2.6866304	0.1143529	1
1.15455916	0.00088443	0.02440185	DEG		0.82295609	0.1611747	1
1.49783103	0.00079248	0.02251915	DEG		-0.3470303	0.51341017	1
-1.358362	0.00156594	0.03803595	DEG		-0.9355797	0.16157415	1
-0.7051377	0.09386809	0.55908861	nonDEG		-1.2214101	0.03075328	1
0.53743522	0.28778663	0.87007405	nonDEG		0.53392785	0.32831394	1
0.12563308	0.61387264	1	nonDEG		0.31569527	0.38141569	1
0.44407911	0.07343801	0.49472348	nonDEG		0.50560933	0.17927236	1
-0.3921023	0.1345854	0.66012324	nonDEG		0.22209739	0.64425971	1
-1.8396456	2.83E-08	4.65E-06	DEG		-0.444081	0.24495038	1
-0.9653605	0.21263361	0.79016926	nonDEG		-0.0685313	0.98233165	1
-1.3576374	2.04E-06	0.00018271	DEG		-0.0598127	0.90145562	1
-1.3103742	0.00162221	0.03904849	DEG		-0.3298469	0.46526942	1
-0.500253	0.16906804	0.72564428	nonDEG		-0.4977963	0.32563703	1
0.76852596	0.02683959	0.27798676	nonDEG		-0.0727667	0.86237073	1
0.55333808	0.03603968	0.33386917	nonDEG		0.12347811	0.75003339	1
0.966796	0.02036112	0.23170124	nonDEG		0.47417507	0.30056083	1
1.19113883	4.13E-06	0.00033668	DEG		0.01976632	0.95805975	1
0.48865641	0.04288509	0.36871905	nonDEG		0.30472971	0.40232528	1
-0.2944563	0.68683773	1	nonDEG		1.29515869	0.05166679	1
-0.5900608	0.02469065	0.2630517	nonDEG		-0.3035158	0.4320156	1
-0.0790667	0.84402887	1	nonDEG		-0.3072397	0.53779011	1
0.45774844	0.12408521	0.64199379	nonDEG		0.44671562	0.24146525	1
-0.7273641	0.17200504	0.73059581	nonDEG		1.61871776	0.01964763	0.99212109
0.54200838	0.04941352	0.40173557	nonDEG		0.4403012	0.29877061	1
0.45316623	0.07863604	0.513638	nonDEG		0.13104767	0.73831008	1
1.28102757	0.19011223	0.75682406	nonDEG		1.39194509	0.08291794	1
1.23454503	0.00015025	0.0060995	DEG		0.45839298	0.23670962	1
1.60309521	0.00634044	0.10520171	nonDEG		-0.481831	0.47143835	1
1.04688795	3.12E-05	0.0017603	DEG		-0.2985756	0.43641027	1
-0.9323484	0.00272	0.05722682	nonDEG		-0.5599805	0.20231678	1
-0.1790533	0.56273426	1	nonDEG		-0.4147699	0.3175698	1
1.27305938	0.03998565	0.35413283	nonDEG		1.01815117	0.17008483	1
0.80549856	0.42765101	0.96944251	nonDEG		0.20199562	0.85067774	1
-0.9799108	0.00022994	0.0085793	DEG		-0.0208718	0.95901852	1

-0.6334381	0.11765939	0.62715457	nonDEG	0.12020851	0.8245167	1
-1.8153421	5.79E-10	1.59E-07	DEG	-0.6700857	0.31361169	1
1.36663322	6.42E-05	0.00307467	DEG	-0.0722494	0.87157908	1
-0.4589274	0.06915801	0.48132826	nonDEG	-0.2560872	0.48598649	1
1.56355051	1.27E-05	0.00085105	DEG	0.53475961	0.14295699	1
0.58058995	0.01915081	0.22295871	nonDEG	0.32765576	0.36790317	1
0.58406905	0.03582404	0.33244726	nonDEG	0.78129292	0.03968742	1
1.50119551	0.00013955	0.00576169	DEG	0.17129194	0.69995004	1
-0.615323	0.04736692	0.3920255	nonDEG	-0.4125092	0.35114385	1
0.42710592	0.15725732	0.70229041	nonDEG	0.48321791	0.2789696	1
-1.0931406	0.01186298	0.16465553	nonDEG	-0.3556263	0.54730671	1
-0.4794889	0.06277072	0.45842401	nonDEG	-0.6116026	0.11880911	1
-0.3311452	0.2642862	0.8486389	nonDEG	-0.5893474	0.14188019	1
0.7401978	0.13719859	0.66397983	nonDEG	0.81051101	0.11062582	1
-0.9956635	0.00083906	0.0234577	DEG	-0.9170738	0.03668929	1
1.08702336	3.83E-05	0.00206456	DEG	0.24622435	0.50131305	1
-0.3689946	0.18755549	0.75310345	nonDEG	-0.5784474	0.12003307	1
0.37493746	0.46976811	0.98843959	nonDEG	0.59960702	0.34027471	1
1.54205963	0.00461523	0.08397257	nonDEG	0.36272007	0.59865428	1
-0.5832411	0.06617768	0.47012661	nonDEG	-0.5561728	0.20574822	1
-1.9624628	1.01E-08	1.97E-06	DEG	-1.6608526	0.01019056	0.77777211
-0.4820311	0.37738048	0.93489063	nonDEG	0.97336257	0.06929636	1
-1.6466339	0.00011068	0.00477758	DEG	-0.7617932	0.20727926	1
0.50499229	0.09360023	0.55820343	nonDEG	0.46331559	0.21647289	1
1.03125264	0.00222027	0.04929297	DEG	0.52049413	0.28449969	1
0.39392922	0.44320807	0.97900292	nonDEG	1.17478826	0.0803581	1
-0.8728575	0.00915815	0.13741432	nonDEG	-0.0712321	0.90141825	1
0.10501235	0.74855	1	nonDEG	-0.7078154	0.08777839	1
1.11131248	0.0080008	0.12495257	nonDEG	0.8435205	0.04889596	1
-1.5183688	0.00044422	0.01474346	DEG	-0.9441144	0.13301362	1
-0.9348328	0.05632207	0.43207778	nonDEG	0.45653301	0.42830797	1
-1.8460707	0.00060063	0.01839949	DEG	0.60999222	0.39247243	1
-0.4144845	0.09188851	0.5524006	nonDEG	-0.0229287	0.95750862	1
-0.6308027	0.27148122	0.85427653	nonDEG	-3.6035568	0.00013291	0.05359881
1.47748951	0.02994761	0.29766155	nonDEG	1.0540639	0.09541646	1
0.30376241	0.22896891	0.80924485	nonDEG	0.59857721	0.1236129	1
1.47174163	4.80E-05	0.00244092	DEG	-0.0075521	0.98843009	1
-0.5931176	0.02471456	0.2630517	nonDEG	-0.2805638	0.45274558	1
-0.3096069	0.46113812	0.98479636	nonDEG	-1.1236598	0.04363461	1
-0.0256574	0.9455313	1	nonDEG	-0.3146506	0.49858135	1
0.58874677	0.04965406	0.4028013	nonDEG	-0.1007974	0.82109323	1
0.37536135	0.11851227	0.62868959	nonDEG	0.26283904	0.51072302	1
-0.631884	0.01470014	0.18763285	nonDEG	0.4598899	0.30442667	1

-0.5230346	0.10331185	0.58748263	nonDEG	-0.2907163	0.5135449	1
1.17540803	2.98E-06	0.00025435	DEG	-0.1704297	0.63409021	1
-0.7303349	0.05572415	0.42921589	nonDEG	0.02087825	0.97284409	1
0.36751033	0.21202923	0.78921843	nonDEG	0.27557643	0.48759459	1
-1.794174	9.64E-05	0.00430246	DEG	-1.4507937	0.03967888	1
1.31415691	0.00600695	0.10102163	nonDEG	-0.0472747	0.9442004	1
-0.6436928	0.60745265	1	nonDEG	1.52627513	0.16189549	1
0.9528668	0.06130792	0.45245519	nonDEG	1.44604646	0.00654943	0.63795339
-1.3811238	0.01091683	0.15585871	nonDEG	-0.1255332	0.86686743	1
-0.4049375	0.1746377	0.73718944	nonDEG	-0.2153458	0.63258019	1
0.64030951	0.01617874	0.19936628	nonDEG	0.0569332	0.88076706	1
1.17839696	0.00372589	0.07297836	nonDEG	0.55117683	0.32488643	1
1.3683696	0.00160765	0.03879324	DEG	0.08747709	0.85364865	1
-0.398082	0.14589009	0.68031971	nonDEG	-0.4737579	0.23820141	1
0.05012567	0.87767932	1	nonDEG	-0.5075115	0.25794554	1
-1.1542659	0.00059419	0.01827822	DEG	-1.1096989	0.06646087	1
0.60638177	0.03270609	0.31434365	nonDEG	0.69533524	0.08091324	1
-0.0940545	0.86053887	1	nonDEG	0.71716943	0.11793045	1
0.36305371	0.16467046	0.71584961	nonDEG	-0.0014762	0.99754384	1
-0.5139726	0.08349042	0.52850582	nonDEG	-0.5825724	0.13032022	1
-1.0823195	0.14327351	0.67575918	nonDEG	0.34325056	0.66929383	1
-0.6964472	0.33255194	0.91644391	nonDEG	-0.4097879	0.6462935	1
-0.87955	0.00630633	0.10476984	nonDEG	0.00556672	0.99400001	1
0.47986962	0.08456511	0.53259236	nonDEG	0.60139243	0.09995682	1
-0.1229666	0.76841809	1	nonDEG	-1.0640444	0.06001891	1
-1.2721926	0.0028225	0.05893013	nonDEG	-0.2380904	0.5372043	1
1.60924737	2.82E-10	9.08E-08	DEG	0.55006366	0.14437349	1
0.77518872	0.01513482	0.19140519	nonDEG	1.16780681	0.006598	0.63894257
-0.2565525	0.29303237	0.87606174	nonDEG	-0.0396483	0.91559943	1
-0.6346645	0.01295506	0.17296329	nonDEG	-0.0182094	0.96342487	1
-0.6218148	0.01201358	0.16595585	nonDEG	-0.4239901	0.39624936	1
-1.1934137	0.0008739	0.02420214	DEG	-1.1095657	0.0530531	1
-1.2734569	0.02760894	0.28280435	nonDEG	-0.2786545	0.71659667	1
0.75687612	0.0935827	0.55820343	nonDEG	0.91041316	0.06753397	1
-0.5309331	0.43063457	0.97169872	nonDEG	-3.4139494	0.00107568	0.2294705
0.0696974	0.92503156	1	nonDEG	0.65632535	0.31223744	1
-2.0945936	0.03389919	0.32150713	nonDEG	0.94915639	0.45345623	1
-1.2340961	0.01338446	0.17621915	nonDEG	0.09032684	0.89260212	1
-0.6491169	0.01003228	0.14620805	nonDEG	-0.1043633	0.80374768	1
-0.3844557	0.16419792	0.71505267	nonDEG	-0.1791825	0.66706736	1
-0.6840467	0.04402773	0.37471301	nonDEG	-0.2764213	0.56159225	1
-2.552219	4.22E-16	5.17E-13	DEG	-0.9120246	0.03936219	1
-1.3794335	0.09431328	0.56011472	nonDEG	-2.5554371	0.03199503	1

0.07885494	0.87779161	1	nonDEG	0.84399087	0.08258861	1
-2.3622482	0.00040941	0.01380601	DEG	-0.0784277	0.96722935	1
-1.6945441	0.01210346	0.16646771	nonDEG	-0.8068262	0.33186795	1
-0.5250653	0.38292722	0.93908647	nonDEG	1.41070447	0.01033826	0.77777211
-0.3018599	0.86980915	1	nonDEG	1.92155927	0.16543975	1
2.055095	2.69E-06	0.00023316	DEG	-0.9042661	0.07441724	1
-0.3792813	0.23010221	0.80924485	nonDEG	-1.1107286	0.01230811	0.81648099
2.48390721	0.08054252	0.51996813	nonDEG	2.16989564	0.10055827	1
-1.2862246	0.32824878	0.91232212	nonDEG	3.8586203	0.00039333	0.11731473
0.6345296	0.10611806	0.59675445	nonDEG	1.19613795	0.00606056	0.61101668
1.23802347	0.02479955	0.26366198	nonDEG	0.23217227	0.78196314	1
-0.9309879	0.00438676	0.08142707	nonDEG	-0.6324874	0.15495278	1
0.8659596	0.00135595	0.03400154	DEG	0.23464378	0.5209131	1
-0.4676185	0.07065953	0.48635627	nonDEG	-0.5943384	0.17480336	1
-1.0996283	1.66E-05	0.00105691	DEG	-0.1286416	0.7922647	1
-1.6982809	0.18641618	0.75310345	nonDEG	0.77327963	0.63334729	1
-1.5157003	4.41E-08	6.95E-06	DEG	-0.0390209	0.9251493	1
-0.2097698	0.44561409	0.98041193	nonDEG	-1.0696488	0.00762508	0.67731627
-0.0866984	0.7312024	1	nonDEG	0.12474317	0.73271563	1
-1.0743722	0.00011117	0.00479173	DEG	-0.0306316	0.96440943	1
-0.9460363	0.13082914	0.65446487	nonDEG	0.53678413	0.47477929	1
-0.5849883	0.01738714	0.20892042	nonDEG	0.07092717	0.87059636	1
-1.3258581	2.45E-06	0.00021505	DEG	-1.1566918	0.04332385	1
-0.4302573	0.19109129	0.7586698	nonDEG	-0.8020317	0.22022549	1
0.61865824	0.02172322	0.24178361	nonDEG	0.70140557	0.13306438	1
0.92744495	0.00326101	0.06611636	nonDEG	0.14951422	0.71307801	1
-0.6385366	0.26378607	0.847847	nonDEG	-0.8985025	0.26106265	1
-2.3568342	3.13E-05	0.00176095	DEG	-1.090938	0.15954173	1
0.17815273	0.48658379	0.99711758	nonDEG	0.51968392	0.19496384	1
0.87607995	0.38398179	0.94051418	nonDEG	1.58321207	0.11359093	1
0.90640323	0.03053473	0.30134491	nonDEG	0.11843837	0.80820181	1
0.69287525	0.01521042	0.19185071	nonDEG	-0.0209422	0.95923919	1
-1.9759299	0.00124734	0.03202195	DEG	0.3442451	0.68134707	1
-0.5421758	0.06580688	0.46873672	nonDEG	-0.3628422	0.39209102	1
-0.4285039	0.11949594	0.63063362	nonDEG	-0.6287033	0.15596499	1
-0.3710895	0.13351243	0.65907076	nonDEG	-0.1333086	0.73952014	1
2.76154877	6.62E-11	2.53E-08	DEG	-1.1520608	0.00681418	0.65342867
0.56635185	0.34893797	0.91917773	nonDEG	0.11774574	0.86620469	1
0.96702657	0.20959476	0.78582018	nonDEG	-0.0640688	0.94647153	1
-1.0496217	0.00161005	0.03881928	DEG	-0.6724313	0.16228275	1
0.67633242	0.02214022	0.24514654	nonDEG	0.37039939	0.30963119	1
1.51794114	0.00704727	0.11334479	nonDEG	0.24970194	0.65206831	1
0.80386779	0.32531428	0.90946768	nonDEG	0.66983635	0.44434537	1

-0.4275696	0.67364662	1	nonDEG	1.649579	0.11785677	1
0.80780401	0.0015901	0.03846418	DEG	0.32753352	0.40026727	1
-0.4876673	0.11274377	0.61361875	nonDEG	-0.4581306	0.26412518	1
1.89888795	1.82E-07	2.30E-05	DEG	-0.0545287	0.90362168	1
0.9691926	0.00015083	0.00610294	DEG	0.24322762	0.51118296	1
-0.8670584	0.00181213	0.0426592	DEG	-0.3312455	0.40405017	1
0.58517544	0.0217963	0.24231921	nonDEG	0.33467568	0.36606101	1
-0.6907373	0.01027858	0.14883975	nonDEG	-0.449356	0.24802779	1
1.06788448	0.00196949	0.0455101	DEG	0.08027653	0.8538796	1
0.45833598	0.09478375	0.56132341	nonDEG	0.59552345	0.16808441	1
0.57479138	0.02970512	0.29616776	nonDEG	0.14383211	0.69476467	1
-0.3196584	0.21801672	0.79640157	nonDEG	-0.4396414	0.25560219	1
1.23945768	0.06776301	0.47541505	nonDEG	0.52697316	0.43258083	1
-0.9110052	0.00223151	0.04946791	DEG	-0.4461525	0.34356693	1
-0.3242468	0.18946686	0.75564367	nonDEG	-0.1637092	0.66692767	1
0.58818123	0.49377853	1	nonDEG	1.62875041	0.1023035	1
-1.17681	4.47E-05	0.00232725	DEG	-0.0696295	0.87073016	1
0.63026515	0.2952502	0.87920183	nonDEG	1.37636592	0.01940515	0.98654907
-0.4542982	0.09923765	0.57396016	nonDEG	-0.6326308	0.12446045	1
-0.5718306	0.03745619	0.3417021	nonDEG	-0.5340798	0.22513803	1
-0.92182	0.0114717	0.1609701	nonDEG	-0.8696812	0.12124572	1
1.96047128	3.42E-05	0.00189334	DEG	-0.372489	0.49901144	1
0.97486657	0.09952719	0.57443019	nonDEG	1.20432792	0.09757403	1
-0.4629922	0.05891023	0.44230002	nonDEG	-0.4793647	0.18650431	1
0.86710028	0.0007832	0.02232018	DEG	0.30657027	0.39526765	1
0.300323	0.51281053	1	nonDEG	0.4319042	0.44607409	1
1.97600463	0.0122688	0.16791309	nonDEG	0.3787393	0.63180283	1
0.70001767	0.00986107	0.1445207	nonDEG	0.24044423	0.50286267	1
-0.9944204	0.28520334	0.86735764	nonDEG	-2.1098527	0.14395447	1
2.45930925	2.91E-10	9.17E-08	DEG	-1.4446594	0.00720478	0.67120689
1.74282748	0.00075162	0.0217615	DEG	0.40337768	0.52709687	1
0.03904059	0.89529943	1	nonDEG	0.17723676	0.64684464	1
-0.5457595	0.03084914	0.30332924	nonDEG	-0.19539	0.59356056	1
-0.666014	0.01997652	0.22918501	nonDEG	-0.8762503	0.0430876	1
0.47440354	0.63519482	1	nonDEG	-0.4601324	0.68856061	1
0.3529515	0.19135574	0.75941247	nonDEG	0.50557396	0.1760627	1
-1.0599797	0.02086068	0.2359014	nonDEG	0.41917986	0.47407803	1
-0.1410683	0.82143795	1	nonDEG	-1.3787401	0.15810927	1
-1.565471	2.32E-05	0.00140067	DEG	-1.024631	0.07280139	1
-0.5850085	0.07738637	0.51011587	nonDEG	-1.0856248	0.01228193	0.81648099
-1.7239569	0.00263048	0.05579158	nonDEG	-1.0355224	0.23110342	1
1.16156823	0.00621995	0.10380335	nonDEG	0.41282247	0.36939448	1
0.371457	0.16339949	0.714154	nonDEG	0.67019719	0.07271655	1

0.21848724	0.49696375	1	nonDEG		0.65401893	0.20098466	1
0.40200065	0.14401845	0.67658948	nonDEG		0.31768358	0.38271181	1
0.63219104	0.54653006	1	nonDEG		1.27868197	0.24703606	1
-2.0268819	5.49E-06	0.0004321	DEG		-0.7172501	0.37726423	1
0.56649877	0.24694474	0.82856236	nonDEG		0.65503242	0.21390078	1
0.49281048	0.0909489	0.55031281	nonDEG		0.55776261	0.13674399	1
-0.4332684	0.44099541	0.97744796	nonDEG		-0.818554	0.23927752	1
-0.3331061	0.17149668	0.72989601	nonDEG		-0.2203978	0.54874429	1
-0.2997867	0.58207913	1	nonDEG		0.64103042	0.33035858	1
0.78175869	0.47428085	0.9909109	nonDEG		0.68263006	0.53352567	1
0.52503891	0.07146868	0.48814946	nonDEG		0.39622804	0.29245748	1
-1.614634	0.0016486	0.03958649	DEG		-0.4845118	0.49430223	1
-0.684955	0.03010897	0.29847765	nonDEG		-0.4157518	0.38778474	1
0.71682924	0.0258567	0.27146768	nonDEG		0.89799824	0.02086947	1
-0.4448674	0.11440806	0.61799245	nonDEG		-0.4515177	0.25946078	1
0.50221399	0.06527937	0.4669909	nonDEG		0.52039704	0.152387	1
1.27686855	0.00053009	0.01674386	DEG		-0.0685905	0.87885393	1
-0.4612266	0.09842771	0.57152136	nonDEG		-0.3679826	0.37286938	1
0.93825509	0.0002019	0.00772904	DEG		0.0981203	0.78603032	1
-0.7885143	0.10095372	0.57899408	nonDEG		-0.5885972	0.43951849	1
-1.2389011	0.0001095	0.00474761	DEG		-0.7544366	0.18702513	1
0.95142673	0.0007231	0.02107654	DEG		0.15012924	0.68191208	1
1.11252499	1.57E-05	0.00101934	DEG		0.02982144	0.93510302	1
-1.062919	0.00825142	0.12704683	nonDEG		-0.5433142	0.32559032	1
-0.4108376	0.11278887	0.61366924	nonDEG		-0.1574497	0.67789899	1
-0.0750195	0.79843778	1	nonDEG		0.50556382	0.24070943	1
0.40499282	0.12967948	0.65346088	nonDEG		0.05725897	0.88264079	1
-2.0133989	0.00011814	0.00506989	DEG		-0.6540857	0.35840082	1
2.10450118	2.18E-08	3.79E-06	DEG		-0.071794	0.89414061	1
0.03853719	0.96125178	1	nonDEG		1.15185892	0.12080331	1
-2.82964	1.58E-05	0.0010225	DEG		-1.1784608	0.17793245	1
-0.2493828	0.3123843	0.89535767	nonDEG		-0.1125423	0.75641079	1
1.36659985	4.10E-07	4.43E-05	DEG		-0.0309906	0.93639509	1
4.71374682	0.21631698	0.79477343	nonDEG		-0.1760301	1	1
0.4823103	0.24673736	0.82843307	nonDEG		0.77973358	0.08749468	1
1.28554213	0.03908304	0.34897353	nonDEG		-0.0756813	0.90196108	1
-0.5144739	0.05740003	0.43596476	nonDEG		-0.3306877	0.36595587	1
0.38655502	0.63746811	1	nonDEG		-0.8468425	0.31248629	1
0.45137698	0.50412892	1	nonDEG		0.73103234	0.32829205	1
1.13426059	0.10160322	0.58100916	nonDEG		0.89518823	0.2364749	1
-0.5562615	0.05729694	0.43573609	nonDEG		-0.3266322	0.45994091	1
0.09434672	0.70378164	1	nonDEG		0.25984383	0.47254981	1
1.06253724	3.45E-05	0.00190756	DEG		0.01168495	0.97561218	1

0.77603835	0.02137761	0.23929105	nonDEG		0.36102225	0.35102117	1
-0.4570209	0.30327149	0.88798156	nonDEG		-0.6584129	0.24896866	1
-1.0180846	0.00010733	0.00466715	DEG		-0.3303812	0.47898325	1
-0.6488073	0.04920938	0.4007777	nonDEG		0.18727691	0.67993073	1
-0.0680697	0.82117937	1	nonDEG		0.51877097	0.20229187	1
-0.1249151	0.64242042	1	nonDEG		0.97450471	0.01112935	0.79590774
0.21304533	0.57741515	1	nonDEG		0.55780832	0.20574127	1
-0.9429002	0.05498991	0.42522933	nonDEG		-0.612626	0.38245913	1
-0.3345988	0.205107	0.77892413	nonDEG		-0.6032146	0.1114119	1
-0.7085705	0.01628355	0.2000715	nonDEG		-0.2474035	0.53765912	1
0.69637691	0.00532782	0.09263475	nonDEG		0.15952377	0.66581592	1
1.07063174	0.03906038	0.34897353	nonDEG		0.37842597	0.47899599	1
0.58233209	0.23155334	0.81081356	nonDEG		0.93566405	0.08120447	1
0.7773468	0.01303426	0.17362699	nonDEG		-0.4050591	0.28076291	1
-0.2001702	0.71165192	1	nonDEG		0.71455876	0.1613108	1
0.54718367	0.047536	0.39261181	nonDEG		0.61367206	0.09451254	1
-0.6498537	0.03698322	0.33917418	nonDEG		-0.1092339	0.803536	1
0.47280985	0.34535744	0.91869633	nonDEG		0.79769528	0.15876065	1
-0.5522461	0.08042099	0.51953337	nonDEG		-0.7097002	0.09631737	1
1.46236895	4.61E-05	0.00237211	DEG		-0.2961437	0.48852955	1
-0.514596	0.09003539	0.54766713	nonDEG		-0.6006801	0.15206978	1
-0.7045396	0.22880514	0.80924485	nonDEG		0.02933141	0.98734369	1
-0.2321287	0.4467599	0.98143159	nonDEG		-0.08395	0.845045	1
0.29132604	0.51896794	1	nonDEG		-1.5751937	0.00650753	0.63646282
0.44249798	0.15378365	0.69420532	nonDEG		0.50858824	0.17034321	1
0.96566852	0.00217687	0.04869678	DEG		-0.0593859	0.88782238	1
-0.7781049	0.00449474	0.0823914	nonDEG		-0.3761016	0.36974155	1
-1.1809571	0.05803229	0.43884871	nonDEG		-0.2511585	0.77429531	1
1.75681741	0.01289679	0.17249823	nonDEG		-0.1896795	0.76967975	1
-1.0206753	0.04755779	0.39261181	nonDEG		0.08553473	0.90333729	1
-0.7995014	0.00522927	0.09132182	nonDEG		-0.3894894	0.38248561	1
-1.1782336	0.00569568	0.0970408	nonDEG		-0.3050546	0.62774542	1
-0.7719095	0.05340048	0.41877092	nonDEG		-0.5878763	0.37864585	1
-0.6346517	0.07640216	0.50612135	nonDEG		-0.4844703	0.31129604	1
-0.7202976	0.02851859	0.28910427	nonDEG		0.17221205	0.68919157	1
0.46405827	0.29085897	0.87302174	nonDEG		0.5289075	0.30111524	1
-0.3533853	0.22077533	0.80037926	nonDEG		-0.0766278	0.84357749	1
-2.6303653	0.01254992	0.16946845	nonDEG		-0.5209767	0.7542983	1
-1.1927329	9.57E-06	0.00068227	DEG		-0.1847962	0.78632108	1
-2.1009955	7.68E-10	2.04E-07	DEG		-1.3627764	0.03037917	1
0.53200431	0.06080585	0.45021717	nonDEG		0.7253452	0.04639507	1
0.38098836	0.23716467	0.81895659	nonDEG		0.48695107	0.23612685	1
1.42072764	0.01907472	0.22256869	nonDEG		-0.740285	0.24870588	1

-0.9445195	0.00466429	0.08444543	nonDEG	0.6339576	0.17421006	1
0.61777288	0.10896123	0.60386103	nonDEG	0.59494578	0.11260456	1
0.92366455	0.01539494	0.19344122	nonDEG	0.15092144	0.71201896	1
-0.2437166	0.6056737	1	nonDEG	-2.3934603	4.32E-05	0.02564683
-0.4776423	0.12087741	0.6327947	nonDEG	-0.5361821	0.29050803	1
-0.0681597	0.97112763	1	nonDEG	1.38929706	0.12413978	1
0.22130336	0.62214255	1	nonDEG	0.54403605	0.19875742	1
-0.6456073	0.0349839	0.3281318	nonDEG	-0.3009635	0.48372651	1
5.13E-15	1	1	nonDEG	2.31087422	1	1
-1.1531741	0.0093098	0.13848972	nonDEG	-0.5540316	0.35355852	1
0.02511093	0.92503586	1	nonDEG	-0.2888466	0.45637522	1
0.9782242	0.08950871	0.54666582	nonDEG	1.37763315	0.02202299	1
0.63187883	0.011025	0.15708569	nonDEG	0.3746922	0.30275757	1
-0.5502805	0.22291138	0.80321763	nonDEG	-1.2566645	0.03457131	1
-2.8703051	0.00591032	0.09982435	nonDEG	-2.0086909	0.34030467	1
0.93283543	0.00021091	0.00801822	DEG	0.05349391	0.88344066	1
0.84975733	0.04908953	0.40042859	nonDEG	0.143892	0.76255868	1
-0.6568462	0.01863433	0.21883163	nonDEG	-0.0293065	0.9420434	1
-2.0252864	3.13E-10	9.51E-08	DEG	-0.1867526	0.76753376	1
-0.7047154	0.02444725	0.26160845	nonDEG	-0.6641589	0.16180837	1
-1.2112975	0.00041811	0.014035	DEG	0.08064092	0.87205511	1
0.40376206	0.42132428	0.96396459	nonDEG	0.62965863	0.25117898	1
-6.145148	0.00578851	0.0983312	nonDEG	0	1	1
-0.8864036	0.00748034	0.11871366	nonDEG	-0.1885013	0.67904005	1
0.48377143	0.05694888	0.43418247	nonDEG	0.2391728	0.52173873	1
0.51350874	0.32220013	0.90520806	nonDEG	0.92687056	0.0765391	1
0.89647327	0.08761607	0.5407399	nonDEG	-2.9020544	5.98E-06	0.00686876
-1.224572	0.00097241	0.02621398	DEG	-0.9929039	0.08603628	1
-2.3169449	3.67E-05	0.00200093	DEG	-1.2271052	0.14243732	1
3.0457161	0.11566982	0.62138758	nonDEG	-2.8251121	0.14976328	1
-1.0662125	6.22E-05	0.00300561	DEG	-0.7076507	0.25912412	1
0.63718537	0.08769755	0.54112938	nonDEG	0.79232431	0.05900328	1
1.06276738	3.88E-05	0.00208669	DEG	-0.0630261	0.86271468	1
-0.5971434	0.02014003	0.23016392	nonDEG	-0.3779629	0.42428194	1
1.53855417	2.67E-09	6.25E-07	DEG	0.24686943	0.49060592	1
1.04119403	0.00085262	0.02372435	DEG	0.14381193	0.70786835	1
-0.1393697	0.59836396	1	nonDEG	-0.7600324	0.04411307	1
-0.3671974	0.15666135	0.70122449	nonDEG	-0.5303579	0.18321145	1
0.73644052	0.53814686	1	nonDEG	1.62722475	0.16291981	1
0.29960241	0.69306387	1	nonDEG	1.12768639	0.17429388	1
2.39060754	4.40E-05	0.00229923	DEG	0.28177517	0.62468682	1
-1.0609657	0.00215795	0.04838117	DEG	-0.4616728	0.25052205	1
1.54911549	0.02368386	0.25718527	nonDEG	0.4869681	0.47546101	1

-1.1539141	0.00329003	0.06665875	nonDEG	-0.7715601	0.24431706	1
0.28657826	0.62618886	1	nonDEG	0.93406226	0.10748763	1
-1.0864323	0.0053336	0.09263475	nonDEG	-1.0946021	0.07959579	1
-0.439273	0.25726817	0.83898637	nonDEG	-0.409762	0.43875276	1
-0.0948411	0.71408	1	nonDEG	-0.3796402	0.32170237	1
-0.8014229	0.00667433	0.10903754	nonDEG	-0.3033329	0.47635692	1
1.55562115	0.00046827	0.01524823	DEG	-0.0594849	0.91593448	1
-2.4227702	3.34E-07	3.77E-05	DEG	0.04805562	0.97071184	1
-0.4807046	0.05375556	0.41998672	nonDEG	-0.0062552	0.98944322	1
0.83249661	0.00115327	0.03009839	DEG	0.26840299	0.45579412	1
-0.5056767	0.17180694	0.7304382	nonDEG	-0.9625316	0.17509628	1
-1.2196646	0.00015143	0.00611534	DEG	0.19156942	0.72936547	1
0.44901811	0.2555771	0.83779145	nonDEG	0.5905023	0.180005	1
-0.9423053	0.1299666	0.65373912	nonDEG	-1.5216942	0.07123514	1
-0.24957	0.30573336	0.89022852	nonDEG	-0.0839065	0.83328452	1
-0.9460751	0.00157549	0.0381902	DEG	-0.4601103	0.40393113	1
0.29627677	0.53711436	1	nonDEG	0.35618226	0.50846256	1
1.51017445	4.09E-06	0.00033448	DEG	0.4707876	0.31844133	1
0.40223645	0.21964662	0.79884354	nonDEG	0.01517857	0.97355005	1
2.79714253	0.00136098	0.03406956	DEG	-0.9038347	0.27505295	1
-0.0596199	0.96010966	1	nonDEG	0.77928394	0.26327409	1
-0.1442537	0.79911823	1	nonDEG	-0.2059559	0.76799052	1
1.26629121	0.00247567	0.05358921	nonDEG	0.07860183	0.86988602	1
1.62394677	8.38E-07	8.51E-05	DEG	0.77278243	0.14684585	1
-0.3967349	0.61616941	1	nonDEG	-8.0057308	2.50E-05	0.01792658
-0.4620326	0.20844881	0.78432061	nonDEG	0.52583317	0.46705842	1
0.24777075	0.30836056	0.89225736	nonDEG	0.28650856	0.42395601	1
0.87341259	0.00085051	0.02369576	DEG	0.17736928	0.62992901	1
0.71388208	0.1438361	0.67652836	nonDEG	-0.1049351	0.85554149	1
0.58785887	0.09055144	0.54873278	nonDEG	0.30283867	0.45691858	1
-0.3572891	0.23584162	0.81748985	nonDEG	-0.837994	0.03164375	1
-0.377492	0.16904343	0.72564428	nonDEG	-0.3431667	0.43523885	1
0.76809266	0.12664026	0.64657696	nonDEG	0.0434997	0.93940009	1
0.61221967	0.36959552	0.92855021	nonDEG	-3.0160968	4.98E-05	0.02864438
-0.6155501	0.01460036	0.18679708	nonDEG	-0.1453918	0.70279571	1
-0.9293038	0.24180018	0.82401585	nonDEG	1.33546795	0.06550727	1
0.16901527	0.82343578	1	nonDEG	1.3674253	0.1022093	1
-1.2312085	0.00376331	0.07346681	nonDEG	0.21426357	0.70197944	1
-4.3442962	0.00661139	0.10821076	nonDEG	-3.6262636	0.46635505	1
0.4122734	0.12854568	0.65114404	nonDEG	0.65765883	0.07562118	1
-0.1947553	0.73673635	1	nonDEG	0.67072572	0.18662209	1
1.49046306	0.00529546	0.09224442	nonDEG	-0.2424116	0.68167723	1
-0.7606392	0.18780588	0.75314224	nonDEG	-0.0585669	0.94683386	1

-1.4216628	0.0487938	0.39887142	nonDEG	-1.5257698	0.16148598	1
-0.8629832	0.00057654	0.0178474	DEG	-0.0382707	0.92444974	1
1.77557889	0.00010892	0.00472947	DEG	1.21258048	0.13056496	1
0.9175644	0.00220185	0.04910636	DEG	0.06005187	0.87753894	1
-0.7936572	0.35435144	0.91917773	nonDEG	1.44845354	0.07414661	1
0.37634459	0.36368934	0.92430334	nonDEG	0.62338527	0.11243108	1
0.6234534	0.0214485	0.23953814	nonDEG	0.40979405	0.2661509	1
0.16199989	0.78904283	1	nonDEG	1.75872544	0.0098505	0.75913347
-0.3463525	0.4198468	0.96294915	nonDEG	0.07395016	0.89142516	1
-1.1222402	0.00359736	0.07107563	nonDEG	-0.6305202	0.47061186	1
-0.9223206	0.00084296	0.02354462	DEG	-0.8097187	0.06181514	1
-0.3207853	0.19957519	0.77235362	nonDEG	-0.0331138	0.93737006	1
-1.4747958	0.13965192	0.66785296	nonDEG	-0.5852066	0.62450049	1
-2.5521609	0.01796583	0.21373149	nonDEG	-0.3064479	1	1
-0.443096	0.25030566	0.832419	nonDEG	-0.9844207	0.08236114	1
-0.6470946	0.35590211	0.91924469	nonDEG	2.16817511	0.00061767	0.16235277
-0.7274186	0.11402159	0.61692363	nonDEG	-0.157333	0.79606989	1
-1.4492326	2.18E-05	0.00133127	DEG	-1.2666194	0.0413795	1
-0.8329785	0.00819846	0.12656241	nonDEG	-0.0094883	0.99021011	1
-0.535625	0.02991211	0.29766155	nonDEG	0.10393742	0.79772289	1
1.27473556	0.00042886	0.01434686	DEG	0.73091444	0.06151662	1
-1.9654216	5.70E-05	0.00280626	DEG	-1.0593931	0.18346217	1
0.91782541	0.01094972	0.15625248	nonDEG	0.0604175	0.89989136	1
0.22630559	0.46935066	0.98800243	nonDEG	0.23268407	0.56167447	1
0.35473088	0.27157609	0.85427653	nonDEG	0.62846013	0.12028813	1
-0.6386334	0.21152658	0.78824441	nonDEG	1.0270081	0.00984187	0.75913347
-0.0267312	0.93087494	1	nonDEG	0.10227555	0.80457696	1
-0.8055987	0.04298864	0.36908643	nonDEG	0.44378877	0.50655686	1
0.61721026	0.01366282	0.17852636	nonDEG	0.37908877	0.29813402	1
0.6794885	0.10334099	0.58753485	nonDEG	0.0728285	0.88901841	1
0.2068756	0.59503715	1	nonDEG	0.36352839	0.43359177	1
-2.0491752	0.00173007	0.04124019	DEG	-2.0066149	0.05819861	1
0.85447419	0.02146704	0.23965425	nonDEG	0.18017126	0.76667103	1
1.79975783	0.00523009	0.09132182	nonDEG	-1.4545857	0.04106309	1
0.98277671	0.00351647	0.06971138	nonDEG	0.8228336	0.04893948	1
-2.6671201	0.00458491	0.08357591	nonDEG	0.66586786	0.57467754	1
-0.0584932	0.84658362	1	nonDEG	-0.2471815	0.54072085	1
-0.6784149	0.00542687	0.09397745	nonDEG	-0.2964467	0.41722205	1
0.79762009	0.16272571	0.714154	nonDEG	0.80464171	0.17342644	1
1.46328531	1.19E-08	2.24E-06	DEG	-0.0926786	0.8016981	1
-0.4108624	0.09176049	0.55224625	nonDEG	-0.2198029	0.5729617	1
-0.5095664	0.09180655	0.55224625	nonDEG	-0.3918205	0.30908434	1
0.54641233	0.06377601	0.46209743	nonDEG	0.32048423	0.41753317	1

0.17742553	0.75017481	1	nonDEG	2.14163628	2.67E-07	0.00098156
1.31786374	0.02614241	0.27364824	nonDEG	0.6222188	0.33932966	1
-0.2857256	0.40143152	0.95305194	nonDEG	-1.1982069	0.00942045	0.74892342
1.45846819	1.75E-06	0.00015977	DEG	0.50151888	0.32814485	1
-0.0804677	0.93931578	1	nonDEG	1.48464441	0.09009017	1
-0.77096	0.20414103	0.77789719	nonDEG	-0.8939419	0.26199679	1
0.72755731	0.11837186	0.62843136	nonDEG	0.30141445	0.55498024	1
-0.5039169	0.08081132	0.52035682	nonDEG	-0.1515956	0.70689767	1
1.11197651	0.07896965	0.51467511	nonDEG	-0.3294906	0.63796759	1
0.71726522	0.03296805	0.31599987	nonDEG	1.08174028	0.06519665	1
0.50290761	0.35575695	0.91924469	nonDEG	0.22778805	0.70428388	1
0.01939876	0.94877664	1	nonDEG	0.37136338	0.32750568	1
-1.3937198	0.08985069	0.54766713	nonDEG	-0.7599433	0.45861666	1
1.27308215	4.72E-05	0.00241502	DEG	-0.0084208	0.98386241	1
0.4462991	0.07328511	0.49429581	nonDEG	0.32744624	0.37268616	1
-0.2307334	0.8291778	1	nonDEG	1.75161581	0.10176876	1
1.49796344	0.00590835	0.09982435	nonDEG	0.70357888	0.32858092	1
-4.589913	4.11E-05	0.00217663	DEG	2.56965759	0.10393413	1
-0.6287332	0.03428004	0.32375505	nonDEG	-0.5552655	0.17551291	1
-0.6222182	0.17555878	0.73927547	nonDEG	-0.9732851	0.10661104	1
1.78154032	1.49E-05	0.00097627	DEG	-0.101623	0.84171798	1
-0.4405298	0.0922071	0.55329901	nonDEG	0.02719354	0.94572799	1
1.25211618	0.00062892	0.01906789	DEG	0.79835383	0.04357927	1
-1.0147083	0.00345995	0.06877616	nonDEG	0.18656569	0.63654705	1
1.14574639	0.00858149	0.13076106	nonDEG	-0.1416243	0.7908335	1
-0.7103507	0.18806566	0.75356653	nonDEG	-0.7125188	0.37425062	1
-0.0101543	1	1	nonDEG	1.17264209	0.07283279	1
-0.5816457	0.35040488	0.91917773	nonDEG	-0.0495988	0.95569536	1
0.87397509	0.02704266	0.27943449	nonDEG	0.28702987	0.48611359	1
0.37533517	0.17717714	0.74269189	nonDEG	0.34762779	0.35918786	1
-0.3753995	0.13274877	0.65757885	nonDEG	-0.1181347	0.75297653	1
0.77432641	0.07945843	0.51648858	nonDEG	1.14019516	0.0121656	0.81648099
-0.3822723	0.18352377	0.75100866	nonDEG	-0.460565	0.25095227	1
2.49255839	0.00046087	0.01509189	DEG	-0.4964666	0.46145216	1
0.26752212	0.44465207	0.97966661	nonDEG	0.49263239	0.2342573	1
-0.6496803	0.01870292	0.21927334	nonDEG	-0.386699	0.31773766	1
-2.3756505	0.01737607	0.20887466	nonDEG	-0.262993	0.84895589	1
2.26205136	6.78E-15	5.71E-12	DEG	-0.4376511	0.28791296	1
-0.4334162	0.13827305	0.66600461	nonDEG	-0.2472522	0.52823208	1
0.44177397	0.15691721	0.70162297	nonDEG	0.2164207	0.57388596	1
-0.4283739	0.09720573	0.56914069	nonDEG	-0.2829997	0.45420137	1
-0.9560566	0.21257935	0.79016926	nonDEG	-3.4335568	0.00138863	0.2707283
0.70130138	0.00921379	0.13766588	nonDEG	0.09270096	0.80230354	1

1.49880552	0.0003125	0.01115122	DEG		0.47441199	0.31411209	1
-0.8220977	0.00095067	0.0257943	DEG		0.26160839	0.49654306	1
-0.6378032	0.01440264	0.18561379	nonDEG		0.00961313	0.98066795	1
0.55987684	0.05882966	0.44203329	nonDEG		0.60958	0.09948856	1
-0.6786046	0.01100994	0.1570356	nonDEG		-0.3105709	0.46341744	1
0.84368078	0.02345489	0.25501106	nonDEG		-0.0907776	0.84015464	1
0.81325801	0.21502282	0.7942651	nonDEG		0.4628745	0.5266595	1
-1.722301	1.29E-06	0.00012288	DEG		-1.8333241	0.00017361	0.06232902
-0.4973613	0.06647258	0.47142526	nonDEG		-0.4912913	0.31980615	1
2.34032391	0.00081673	0.02292048	DEG		-3.8644943	2.58E-06	0.00447518
0.01025542	0.97359705	1	nonDEG		0.30411988	0.62302995	1
-0.7837949	0.00376108	0.07346681	nonDEG		-0.3375002	0.3823122	1
0.80621785	0.01522427	0.19188545	nonDEG		0.22118714	0.60828441	1
-0.2959885	0.53194577	1	nonDEG		0.59339438	0.13521511	1
-0.3220806	0.33258909	0.91644391	nonDEG		-0.0933551	0.83264361	1
1.59851902	0.0085552	0.13061862	nonDEG		-0.1036603	0.86805847	1
1.72599651	0.44363166	0.97909824	nonDEG		1.54618521	0.27550161	1
1.25486698	5.54E-06	0.00043409	DEG		-0.4763728	0.20596317	1
-1.714687	5.15E-10	1.44E-07	DEG		-0.9449293	0.02552468	1
-0.9631386	0.03139508	0.30676486	nonDEG		-0.5717904	0.3641177	1
0.80078636	0.0025691	0.05492505	nonDEG		0.5328909	0.15064561	1
-0.4718208	0.05473694	0.42427613	nonDEG		-0.2997768	0.44303308	1
0.84806684	0.04496839	0.37964291	nonDEG		0.15622844	0.74924514	1
-0.0459108	0.97772924	1	nonDEG		0.44557443	0.60166738	1
-0.2651574	0.33257634	0.91644391	nonDEG		-0.7687448	0.04052052	1
0.79937543	0.09357799	0.55820343	nonDEG		0.43138681	0.38809508	1
-0.6832482	0.00511442	0.08967438	nonDEG		0.18114082	0.6446936	1
0.45920394	0.14285247	0.67502953	nonDEG		-0.4717385	0.25023144	1
-0.4652302	0.10160927	0.58100916	nonDEG		0.06988087	0.86949002	1
-0.2521751	0.32939017	0.91339798	nonDEG		-0.4774346	0.21536301	1
-2.650959	0.0204356	0.23231812	nonDEG		1.01725014	0.65245262	1
1.9652021	0.00061654	0.01875737	DEG		0.16620208	0.77731593	1
1.22760356	0.00134215	0.03374167	DEG		0.18030659	0.68735568	1
-0.5151043	0.62001199	1	nonDEG		2.56082459	0.03315109	1
-0.945789	0.21542845	0.79470372	nonDEG		1.41416478	0.07963766	1
0.48610777	0.24749902	0.82907898	nonDEG		0.62063462	0.18064239	1
0.29675062	0.27683381	0.85887436	nonDEG		0.63925836	0.08666795	1
0.17723716	0.62915912	1	nonDEG		0.47533489	0.26076694	1
-1.0166202	0.22275579	0.80314244	nonDEG		1.96197298	0.0103697	0.77777211
-1.8869807	0.00037729	0.01290008	DEG		-1.2408659	0.2591219	1
0.95758034	0.06514611	0.46677686	nonDEG		0.42275768	0.43221414	1
-2.1064494	7.78E-12	3.82E-09	DEG		-1.095879	0.05198112	1
-0.986687	0.00055806	0.01732994	DEG		-0.3040433	0.50907324	1

-1.080767	1.51E-05	0.0009807	DEG	0.00172495	0.99731858	1
-0.9109048	0.01984149	0.22799131	nonDEG	-0.0438765	0.92478156	1
0.36083695	0.75225039	1	nonDEG	1.80550559	0.14403244	1
-2.7875052	1.06E-05	0.00073698	DEG	-0.4424473	0.65797914	1
1.28848491	1.63E-06	0.00015068	DEG	-0.0494874	0.89347529	1
0.1211761	0.67903749	1	nonDEG	0.56942565	0.18961457	1
0.68189418	0.21846874	0.79677948	nonDEG	0.74071881	0.17521597	1
0.33158389	0.23339282	0.81342102	nonDEG	0.47137063	0.24209401	1
1.37344638	0.18939766	0.75564367	nonDEG	0.62253809	0.53122242	1
-0.7862058	0.03240438	0.3128739	nonDEG	-0.0449864	0.93599952	1
-0.503257	0.04777569	0.3935669	nonDEG	-0.0085536	0.98271486	1
0.05457665	0.83285127	1	nonDEG	-0.3205379	0.41307309	1
0.4927031	0.06796045	0.47559369	nonDEG	0.39473092	0.34728206	1
-0.3932384	0.18124249	0.74675966	nonDEG	0.95898774	0.01289084	0.82858811
-0.5074756	0.04220597	0.36490497	nonDEG	-0.2275567	0.54351221	1
2.43592652	1	1	nonDEG	1.7381066	0.61367766	1
0.29895787	0.72787802	1	nonDEG	0.5508607	0.49608787	1
-2.5375414	1.65E-08	2.99E-06	DEG	-0.5725648	0.34723307	1
0.89452183	0.00044699	0.01478291	DEG	-0.0086756	0.98148203	1
0.92073163	0.38827517	0.94521067	nonDEG	0.79128387	0.44174743	1
-0.7775409	0.12448579	0.6432749	nonDEG	1.22585409	0.00624267	0.62218628
-1.3253615	7.86E-05	0.00362619	DEG	0.21585233	0.70237521	1
2.86648029	0.24786765	0.82939028	nonDEG	0.37259276	0.84897884	1
-0.7199437	0.00494966	0.08792926	nonDEG	-0.0637618	0.86804384	1
0.40928814	0.08999725	0.54766713	nonDEG	0.50791341	0.18384094	1
0.34623176	0.69823234	1	nonDEG	-0.4435318	0.67365658	1
-1.1212642	7.32E-05	0.00342	DEG	-0.548186	0.19222891	1
0.06706662	0.96165144	1	nonDEG	1.6269074	0.10447981	1
1.19544937	0.00070749	0.02078634	DEG	0.15608021	0.69365588	1
0.69485513	0.02077151	0.23518945	nonDEG	0.08264857	0.84558168	1
2.09448247	0.00223407	0.04948741	DEG	-0.1798927	0.81025174	1
0.36899222	0.40368235	0.95383155	nonDEG	0.2611049	0.58628786	1
-1.4708133	7.47E-05	0.00348658	DEG	-1.0568077	0.10246717	1
-1.9356843	2.21E-09	5.29E-07	DEG	-1.3747222	0.01132352	0.80132964
-2.1874927	1.92E-06	0.00017349	DEG	-1.7874422	0.02176969	1
-1.3641644	1.15E-07	1.56E-05	DEG	0.12923722	0.76025967	1
-0.7606899	0.10185286	0.58177076	nonDEG	-0.6971178	0.27150665	1
0.47993598	0.42315517	0.96552976	nonDEG	1.02970354	0.14113759	1
0.43215751	0.16770796	0.72307479	nonDEG	0.28203938	0.48200398	1
0.89763333	0.00309549	0.06345974	nonDEG	0.17516617	0.66840129	1
-0.3416266	0.1613151	0.7113474	nonDEG	0.11983659	0.78234619	1
-0.3709803	0.18665514	0.75310345	nonDEG	-0.5427046	0.48988059	1
-0.6275444	0.0104764	0.15140639	nonDEG	-0.2588719	0.5026089	1

-0.7417538	0.00391754	0.07513256	nonDEG	-0.1708346	0.71787841	1
-1.2036758	0.03074798	0.30263786	nonDEG	-1.0159998	0.26254862	1
-0.0055666	0.98985462	1	nonDEG	-1.5015824	0.00216102	0.35783028
-0.2835915	0.27258857	0.85574694	nonDEG	-0.006344	0.98650395	1
0.20412807	0.67750249	1	nonDEG	0.32358231	0.56137645	1
1.61345517	1.84E-05	0.00116028	DEG	-0.0320087	0.93885145	1
-0.7757622	0.00332463	0.06699103	nonDEG	0.0121771	0.98109923	1
0.21700205	0.5745101	1	nonDEG	1.06575487	0.00752194	0.67511746
-1.1112516	7.47E-06	0.00055908	DEG	-0.1819906	0.75757222	1
0.31551286	0.30631086	0.89036093	nonDEG	0.44812374	0.27015757	1
0.25561042	0.29771254	0.88261266	nonDEG	0.48445361	0.182223	1
-6.2419646	0.00362223	0.07147111	nonDEG	0	1	1
0.4120347	0.58154195	1	nonDEG	-5.2782259	4.17E-18	1.23E-13
0.10171793	0.86050384	1	nonDEG	1.11157207	0.03154947	1
0.65750737	0.01177564	0.16392237	nonDEG	0.50075243	0.20736415	1
-0.6511024	0.01645208	0.20135003	nonDEG	-0.6469174	0.09462428	1
-1.3770304	0.01237643	0.16837141	nonDEG	-1.22049	0.15905133	1
-0.4635897	0.37567241	0.93353904	nonDEG	0.03012708	0.96873914	1
0.44769315	0.06365711	0.46191806	nonDEG	0.46774416	0.22417223	1
-3.4908015	9.09E-13	5.46E-10	DEG	0.25765604	0.70342071	1
0.83305179	0.02215525	0.24519865	nonDEG	0.06229774	0.89612779	1
-0.7460091	0.13513196	0.66115168	nonDEG	-1.7047755	0.02041583	1
-1.0219598	0.00012639	0.0053535	DEG	-0.3990775	0.4720219	1
-0.1979425	0.46146944	0.98479636	nonDEG	-0.4324204	0.29820461	1
-0.6885458	0.02528036	0.26713155	nonDEG	-0.2798978	0.55075079	1
0.55263562	0.16833203	0.72428041	nonDEG	-1.5599183	0.0019417	0.34643459
-1.6434097	0.13503046	0.6610751	nonDEG	-2.4974432	0.14582726	1
-0.4960405	0.06109395	0.45160884	nonDEG	0.05308905	0.90306359	1
0.99815331	0.0221294	0.24514654	nonDEG	0.35167971	0.5676079	1
-0.7620885	0.0033217	0.06697784	nonDEG	-0.0622131	0.87780361	1
-0.935012	0.00693108	0.11217375	nonDEG	-0.2178366	0.68256472	1
-0.4961041	0.25316961	0.83442231	nonDEG	-0.0711435	0.91428417	1
0.3684353	0.16975962	0.72708731	nonDEG	0.32135982	0.37907812	1
0.48923002	0.07758069	0.51025426	nonDEG	0.77421631	0.035836	1
-1.3846375	0.00012289	0.00525086	DEG	0.13159143	0.80656426	1
-1.1569889	0.03237263	0.31266985	nonDEG	-2.0982851	0.00865936	0.72219089
-0.7210739	0.00438735	0.08142707	nonDEG	0.02291205	0.95412008	1
0.89054568	0.00285118	0.05945031	nonDEG	-0.0662208	0.86720777	1
0.23362527	0.41270067	0.95915425	nonDEG	0.54395815	0.14093332	1
0.02031118	0.97213072	1	nonDEG	0.85337755	0.08794732	1
-1.0925596	1.10E-05	0.00075304	DEG	-0.0600703	0.86957777	1
1.13994879	0.03723997	0.34056339	nonDEG	-0.0552301	0.92160877	1
-0.5127946	0.11454579	0.61862292	nonDEG	-0.9448472	0.04370022	1

0.41431241	0.28970933	0.87161191	nonDEG	0.63021248	0.13108341	1
-0.8314505	0.00363078	0.07149597	nonDEG	-1.0779579	0.01416261	0.8605661
1.64824282	8.86E-09	1.78E-06	DEG	0.12594993	0.74048239	1
0.64646391	0.42464195	0.96646612	nonDEG	0.68923198	0.38819834	1
0.03367903	0.92467942	1	nonDEG	-0.430623	0.37954911	1
-0.5031442	0.03874723	0.34766221	nonDEG	0.20642821	0.65297075	1
0.75039133	0.00704063	0.11334479	nonDEG	0.28302565	0.43770355	1
-1.5601787	0.01300604	0.17332942	nonDEG	0.01970934	0.98531205	1
-0.8797713	0.0012693	0.03247356	DEG	-0.4151226	0.33232051	1
-0.4885394	0.14105561	0.67073755	nonDEG	-0.1987672	0.63896222	1
1.3446648	0.0041743	0.07844016	nonDEG	0.54296951	0.29284655	1
-0.3725189	0.15103937	0.68824233	nonDEG	-0.5301116	0.15657381	1
0.52198921	0.06532384	0.4669909	nonDEG	-1.2280625	0.02044262	1
-0.9079461	0.00042474	0.01424137	DEG	-0.3293185	0.41222626	1
-1.9919386	0.00014214	0.00584414	DEG	-0.1510445	0.84857037	1
1.3895294	3.91E-07	4.25E-05	DEG	0.56459978	0.16553821	1
1.27722381	0.00057745	0.01785678	DEG	0.15311939	0.71405334	1
0.58766496	0.22675786	0.80853846	nonDEG	0.34439273	0.53845738	1
1.88153122	0.01057732	0.15255616	nonDEG	0.3276145	0.63362237	1
-0.1183656	0.66159657	1	nonDEG	-0.3671385	0.33116499	1
0.8523322	0.00177276	0.04191833	DEG	-0.2578548	0.49124677	1
-1.0179045	0.01311613	0.17432314	nonDEG	-0.3124019	0.66543887	1
0.44274308	0.18570267	0.75310345	nonDEG	0.14899968	0.72139533	1
1.286025	0.12481013	0.64346707	nonDEG	0.40084434	0.6862433	1
-0.9720062	0.00068367	0.0202791	DEG	-0.2993005	0.45193656	1
-0.23169	0.34804976	0.91917773	nonDEG	-0.1161663	0.76451583	1
0.43138252	0.09310527	0.55653319	nonDEG	0.52536041	0.17328986	1
0.27220652	0.28669457	0.86893869	nonDEG	0.42954957	0.31560349	1
-1.3482817	2.37E-07	2.83E-05	DEG	-0.0589747	0.87299699	1
-0.4493199	0.62203829	1	nonDEG	-1.2329738	0.22252039	1
1.13418977	0.06694421	0.47328785	nonDEG	0.5226348	0.47472084	1
0.40792627	0.14057709	0.66965193	nonDEG	0.5229237	0.15287701	1
1.21890532	2.85E-06	0.00024564	DEG	0.02977801	0.93796567	1
0.55539535	0.40528961	0.95519341	nonDEG	-2.0272502	0.0033225	0.46240087
0.19210747	0.44315728	0.97900292	nonDEG	0.61141998	0.10398181	1
0.8640736	0.07558168	0.5034048	nonDEG	0.46938617	0.33338961	1
0.48442757	0.0578643	0.43802193	nonDEG	0.50675972	0.17439695	1
0.20281206	0.5123115	1	nonDEG	0.56635394	0.14729903	1
-0.6563156	0.10485812	0.59272458	nonDEG	0.26293594	0.63799983	1
1.43932154	8.48E-05	0.00388146	DEG	0.15724455	0.70793854	1
1.32841038	0.00198635	0.0456845	DEG	0.13984163	0.78450502	1
1.52879908	6.77E-09	1.41E-06	DEG	-0.3449522	0.34721537	1
-0.7832899	0.17227538	0.73124037	nonDEG	-2.4701485	0.00161644	0.29928568

-1.4601344	0.00100445	0.02688185	DEG	-0.3493935	0.57269077	1
-1.9553223	2.02E-07	2.48E-05	DEG	-1.4337589	0.00807993	0.69551191
-1.6844283	1.99E-07	2.46E-05	DEG	-3.2694578	2.95E-06	0.00462688
0.6868131	0.01782391	0.21271188	nonDEG	0.27576053	0.45239026	1
0.63166315	0.05132782	0.40982907	nonDEG	0.09517503	0.85357085	1
0.45519138	0.10881624	0.60347345	nonDEG	0.224309	0.54509573	1
1.10719641	0.00167727	0.04024223	DEG	-0.0212716	0.96588305	1
1.07630463	0.04583725	0.3841169	nonDEG	1.08076774	0.16287129	1
1.30647657	3.40E-07	3.81E-05	DEG	0.01560509	0.96553491	1
-1.9333602	0.00344895	0.06869664	nonDEG	-1.8359519	0.08295786	1
-1.337097	0.00444232	0.08189458	nonDEG	-0.633825	0.31760136	1
0.03328506	0.97544035	1	nonDEG	2.75101894	0.0114413	0.8038671
-0.8293791	0.0030851	0.06329073	nonDEG	0.29486918	0.63206508	1
0.77855293	0.04751678	0.39260357	nonDEG	1.1340073	0.03418473	1
-0.6170546	0.01497759	0.19021792	nonDEG	-0.1934831	0.60422824	1
0.18681308	0.45598179	0.98394385	nonDEG	-0.0039226	0.9916704	1
-0.5236881	0.04175438	0.36367081	nonDEG	-0.0678531	0.88173926	1
-0.3784112	0.13824249	0.66596638	nonDEG	-0.0063111	0.98833948	1
-0.466087	0.05819058	0.4393717	nonDEG	0.15830352	0.71548315	1
-0.9720728	0.00164335	0.03949273	DEG	0.39061976	0.41695813	1
-0.7449035	0.008022	0.12508453	nonDEG	-1.8837148	1.41E-06	0.00321073
-0.5686136	0.02756077	0.28268426	nonDEG	0.03337671	0.93618387	1
-1.4694292	1.58E-06	0.00014781	DEG	0.07061855	0.92874302	1
-0.4305873	0.0855674	0.5352955	nonDEG	0.340293	0.5056803	1
1.34261488	0.01325415	0.17514359	nonDEG	1.47398209	0.117865	1
1.71725862	1.33E-08	2.48E-06	DEG	-0.0358258	0.92727803	1
0.62145692	0.04774044	0.39350089	nonDEG	0.36902495	0.35708658	1
1.10968232	0.00014045	0.00579077	DEG	0.12617621	0.73253989	1
1.04010502	0.08874858	0.54498738	nonDEG	0.18170432	0.78338773	1
-2.1023476	5.73E-14	4.22E-11	DEG	-0.0183157	0.98056603	1
0.55307666	0.03543446	0.33095019	nonDEG	0.60276804	0.09980693	1
1.10861767	0.00042706	0.01430297	DEG	0.33970046	0.40100783	1
0.01311332	0.95863713	1	nonDEG	-0.4762744	0.19057506	1
-0.402253	0.6837306	1	nonDEG	0.86471374	0.29847177	1
0.8983879	0.00028423	0.0102291	DEG	0.07985726	0.82503727	1
-0.5730594	0.3239159	0.90721721	nonDEG	1.62766615	0.00627702	0.62218628
0.33816077	0.26362374	0.847847	nonDEG	0.87589121	0.04435907	1
0.74779158	0.09507758	0.56227176	nonDEG	0.61815651	0.20250205	1
1.00860477	0.0227539	0.24961389	nonDEG	0.06442864	0.89468888	1
0.02384398	0.9428951	1	nonDEG	-0.2256458	0.5948996	1
0.05437761	0.90426481	1	nonDEG	-1.3730437	0.01561696	0.89863185
-0.2086629	0.39599643	0.9493779	nonDEG	-0.1161951	0.77672243	1
-0.1550628	0.53931185	1	nonDEG	-0.8010359	0.05468072	1

0.4858741	0.17384449	0.73489486	nonDEG	0.37154232	0.39839672	1
1.50186566	8.99E-08	1.27E-05	DEG	0.21815021	0.63809616	1
-0.5255752	0.05162237	0.41117723	nonDEG	-0.4014239	0.32964045	1
-0.556911	0.08274409	0.52622665	nonDEG	-0.6749053	0.12859212	1
-0.7307102	0.00389091	0.07487047	nonDEG	-0.1756341	0.66593117	1
-0.0306042	0.90385717	1	nonDEG	-0.0540056	0.89904978	1
-1.1178504	0.00021108	0.00801822	DEG	-0.2899674	0.46315146	1
0.44861907	0.09904278	0.57383715	nonDEG	0.17778507	0.63408543	1
-0.4029593	0.37015621	0.9288227	nonDEG	-1.1638883	0.05140271	1
-0.2190011	0.72810438	1	nonDEG	1.11095652	0.05083844	1
-0.5978025	0.01982934	0.22794067	nonDEG	-0.4809735	0.19366562	1
0.20119397	0.52038204	1	nonDEG	0.40755989	0.30701849	1
1.71684651	2.84E-09	6.38E-07	DEG	0.19622107	0.60036868	1
0.50033664	0.30657757	0.8907373	nonDEG	-2.7707055	7.61E-12	1.12E-07
0.57074885	0.08910227	0.54567957	nonDEG	0.19127434	0.65104905	1
2.63221998	5.22E-05	0.00262919	DEG	-2.0338912	0.00298731	0.4301506
-0.6371622	0.05216493	0.41404244	nonDEG	-0.8874577	0.07664615	1
0.3922048	0.11272093	0.61360789	nonDEG	0.35933364	0.39830276	1
1.20938103	0.01812261	0.21495232	nonDEG	1.24255076	0.01410556	0.8605661
0.82761271	0.00218443	0.04882869	DEG	0.40802965	0.25415333	1
-1.1220995	0.35623149	0.91924469	nonDEG	-0.6748102	0.70486583	1
1.42185847	6.85E-09	1.42E-06	DEG	-0.0040541	0.99110281	1
-0.5896755	0.05032237	0.4058991	nonDEG	-0.5863841	0.16562251	1
0.35299325	0.65729253	1	nonDEG	1.27266049	0.06201583	1
0.64514092	0.03886041	0.34814714	nonDEG	0.26504104	0.51097244	1
0.59900395	0.3096124	0.89298319	nonDEG	0.26932737	0.74175766	1
-0.3073026	0.58505077	1	nonDEG	1.04971639	0.08260158	1
0.89809892	0.08073181	0.52017157	nonDEG	-0.5600471	0.3606355	1
1.33521663	0.00202203	0.04632418	DEG	0.08363383	0.85396836	1
1.67287174	1.84E-06	0.0001668	DEG	1.56056942	0.00014805	0.0580802
0.64284151	0.17031544	0.72844924	nonDEG	-0.2072012	0.76115434	1
1.25078444	0.0001383	0.00573451	DEG	0.44554935	0.23496872	1
-0.6165349	0.25454549	0.8362895	nonDEG	1.04942592	0.01236966	0.81648099
-2.5427252	0.02961578	0.29606795	nonDEG	0.68645734	0.64027868	1
-0.214606	0.41340487	0.95962044	nonDEG	-0.0899048	0.82215896	1
0.22625992	0.41144413	0.95864691	nonDEG	0.13899837	0.7150702	1
0.58511461	0.09250015	0.55469806	nonDEG	0.91730493	0.16089066	1
-1.2194529	2.83E-05	0.00162636	DEG	-0.2348951	0.56158908	1
-0.6652294	0.04695083	0.3901947	nonDEG	-0.6073368	0.23095411	1
1.15883807	0.00077913	0.02226206	DEG	0.94432048	0.01852911	0.95785021
-0.9024151	0.03782662	0.34285037	nonDEG	-0.8940192	0.07533349	1
-1.016521	0.00013495	0.00563531	DEG	-0.3927952	0.37065237	1
-0.7708051	0.02645105	0.27515638	nonDEG	-0.4771515	0.438699	1

-0.8692879	0.00293684	0.06084276	nonDEG	-0.2763222	0.54002156	1
-1.2365203	0.06891674	0.48031248	nonDEG	0.25382819	0.76622392	1
-0.6939147	0.06757983	0.47527058	nonDEG	-2.2734719	7.02E-08	0.00034445
0.40331273	0.1342882	0.65963131	nonDEG	0.27078114	0.49459998	1
1.16767673	0.01265462	0.17034261	nonDEG	0.87254642	0.11224703	1
4.12809447	2.44E-22	7.24E-19	DEG	-0.6226732	0.37284772	1
-0.1895457	0.78112878	1	nonDEG	0.83303913	0.21141579	1
-0.8552737	0.26355066	0.847847	nonDEG	-0.3634835	0.70360122	1
0.56694907	0.10539767	0.59402755	nonDEG	0.07011592	0.87427271	1
1.0402851	0.00031675	0.01123456	DEG	0.12431302	0.73944868	1
-0.4584977	0.52853344	1	nonDEG	0.99106462	0.21585901	1
0.55727453	0.04671667	0.38914754	nonDEG	0.15020532	0.72346558	1
-0.6077362	0.07193236	0.4897356	nonDEG	-0.1986771	0.66497935	1
5.68219502	0.08083276	0.52035682	nonDEG	0.2263099	0.93732477	1
-0.4992364	0.05747976	0.43634523	nonDEG	-0.4387542	0.2643841	1
-0.5963765	0.10087807	0.57894377	nonDEG	-0.7255493	0.13207491	1
-2.2048412	0.00115225	0.03009839	DEG	-0.8493306	0.37682497	1
0.47714111	0.26657867	0.85071104	nonDEG	0.23408254	0.63970875	1
1.89264477	1.01E-10	3.61E-08	DEG	-0.5873258	0.12392851	1
0.04099772	0.90975761	1	nonDEG	0.48718959	0.2564598	1
0.91361391	0.00097014	0.02618983	DEG	0.22847356	0.60578812	1
0.37552048	0.14665894	0.68223696	nonDEG	-0.3896015	0.29710639	1
-0.7997724	0.00250235	0.05392871	nonDEG	0.05061043	0.90696574	1
0.83193945	0.08650127	0.53862182	nonDEG	0.03327722	0.95595811	1
0.00388502	1	1	nonDEG	0.49477444	0.46398604	1
-0.4577009	0.51449527	1	nonDEG	-0.0118477	1	1
-0.3217382	0.18831461	0.75385371	nonDEG	-0.3079745	0.40263859	1
-0.5452836	0.29976976	0.88467981	nonDEG	-0.7868068	0.22039749	1
-0.4357457	0.09927008	0.57403493	nonDEG	-0.6290997	0.09408676	1
0.54851866	0.05642535	0.43212952	nonDEG	0.06746128	0.89066765	1
-0.6549149	0.00696743	0.11251459	nonDEG	-0.4690795	0.34558588	1
1.2311459	2.15E-06	0.00019164	DEG	0.39593382	0.31239285	1
-0.2104344	0.74295282	1	nonDEG	0.74531579	0.11131438	1
0.97961224	0.00024498	0.00903764	DEG	0.41118056	0.40702596	1
1.86427411	0.02463565	0.26277575	nonDEG	-0.2695532	0.74970644	1
1.16231689	8.62E-06	0.00061887	DEG	-0.1788779	0.62075299	1
-0.8050241	0.01237663	0.16837141	nonDEG	-0.8429777	0.05541787	1
0.99627492	0.01558994	0.19480145	nonDEG	0.87976494	0.13647969	1
0.95157347	0.00422352	0.07914461	nonDEG	0.42678428	0.27602334	1
-0.7078711	0.05404395	0.42112225	nonDEG	-0.0414679	0.95150214	1
0.57129705	0.37141356	0.93009573	nonDEG	0.86927603	0.19568173	1
-0.7702632	0.01246342	0.16873447	nonDEG	-0.2258451	0.60803836	1
-1.6300734	0.0001817	0.00712275	DEG	0.06161351	0.92609139	1

0.68893924	0.13243106	0.65711072	nonDEG	0.5317424	0.28674722	1
0.22648268	0.61066187	1	nonDEG	0.91363857	0.06629108	1
0.63276951	0.06331583	0.46113624	nonDEG	0.23151717	0.57287006	1
0.3653809	0.14600943	0.68076602	nonDEG	0.48718672	0.22996984	1
-0.4281448	0.09357426	0.55820343	nonDEG	0.02199371	0.95493146	1
-0.1204826	0.90441343	1	nonDEG	2.17083877	0.0526643	1
-0.2724987	0.37620714	0.93394411	nonDEG	-0.7762424	0.19838736	1
-0.5945948	0.02111877	0.23725952	nonDEG	-0.2264821	0.61409878	1
-0.7337976	0.10114521	0.57997934	nonDEG	-1.1629093	0.12478752	1
0.5241153	0.3976684	0.95081663	nonDEG	0.5010678	0.42160437	1
0.98486754	0.00038154	0.01300817	DEG	0.14643272	0.68819934	1
1.11665998	0.09629503	0.56628632	nonDEG	-0.2691649	0.73048361	1
-0.3855131	0.52251628	1	nonDEG	1.17280457	0.04930069	1
1.52036462	4.59E-05	0.00236442	DEG	0.45686715	0.32949475	1
0.58872534	0.06630588	0.47058314	nonDEG	0.86414372	0.02056092	1
-0.4591657	0.20510954	0.77892413	nonDEG	-0.0994957	0.83021804	1
-0.3117439	0.20951502	0.78572136	nonDEG	-0.8619802	0.07558204	1
1.14767049	0.00051086	0.01627612	DEG	0.34250739	0.37265302	1
-0.7139491	0.04209414	0.36479284	nonDEG	-1.5359926	0.00313481	0.4415588
0.30784134	0.42980755	0.97110382	nonDEG	0.51827988	0.27766053	1
4.234773	0.34560476	0.91869633	nonDEG	0.14278335	1	1
0.6724489	0.393182	0.94736887	nonDEG	1.10508559	0.18945142	1
1.02717037	0.00432443	0.08062493	nonDEG	0.2186633	0.61309856	1
1.28631782	0.00086912	0.02411505	DEG	0.38174429	0.51294822	1
2.11085735	7.62E-07	7.79E-05	DEG	1.17191567	0.01230754	0.81648099
-1.0341824	0.00038424	0.01304696	DEG	-0.3372084	0.40160668	1
0.14175131	0.69563147	1	nonDEG	0.68223835	0.12970795	1
-0.1570883	0.61735474	1	nonDEG	-0.7552582	0.05885438	1
-0.407435	0.10506134	0.59282029	nonDEG	-0.2787549	0.45375646	1
0.792441	0.00278095	0.05818658	nonDEG	0.13257006	0.72385095	1
-1.1790039	0.00043579	0.01454556	DEG	-0.2724917	0.56156705	1
-0.6091824	0.03758109	0.3420995	nonDEG	-0.4727074	0.24182743	1
0.6147777	0.0159422	0.197776	nonDEG	0.03010435	0.93446156	1
-0.5094386	0.13488628	0.660956	nonDEG	-0.3710553	0.43085433	1
0.8497145	0.01796887	0.21373149	nonDEG	0.3398537	0.44387817	1
1.72168311	0.01875341	0.2197777	nonDEG	0.43279385	0.59353607	1
-0.2246633	0.81135048	1	nonDEG	-2.3105684	0.03764199	1
-1.2385226	0.02379788	0.25768009	nonDEG	-0.6765577	0.40372518	1
1.48086979	0.38106246	0.93723609	nonDEG	-0.7156185	0.67080279	1
0.22235773	0.3832876	0.93951737	nonDEG	0.35445193	0.45140617	1
-0.9345595	0.03250244	0.31312418	nonDEG	-0.5900045	0.32458773	1
0.55106003	0.223773	0.8044515	nonDEG	0.76952671	0.09765544	1
0.1879496	0.5864544	1	nonDEG	0.47379026	0.31615857	1

-0.9772161	0.00149691	0.03663143	DEG	-0.78097	0.04460081	1
0.29442572	0.34034148	0.91869633	nonDEG	0.81978703	0.02883077	1
-0.3403733	0.17375749	0.73480413	nonDEG	-0.5576948	0.14487671	1
0.99868207	0.00514724	0.09008894	nonDEG	-0.0956644	0.82983098	1
-2.8517757	7.18E-05	0.00336893	DEG	0.79619268	0.39120485	1
-0.3451362	0.16593295	0.71910791	nonDEG	-0.4024619	0.26426185	1
0.37246451	0.18987946	0.75640887	nonDEG	0.61888746	0.09777363	1
2.48357517	0.02929623	0.29425173	nonDEG	0.09250662	0.94183458	1
-0.4495474	0.08988452	0.54766713	nonDEG	-0.2477869	0.53154529	1
-1.2711623	0.03847576	0.3458589	nonDEG	-2.1978241	0.01050333	0.77777211
0.72889469	0.00484722	0.0865881	nonDEG	0.55726313	0.12821376	1
-0.5591752	0.03430116	0.32375505	nonDEG	-0.3070292	0.43473963	1
1.61110927	0.10255203	0.58417751	nonDEG	2.19807811	0.0115466	0.80926302
1.14504604	2.18E-05	0.00133127	DEG	0.10421495	0.77647974	1
5.13E-15	1	1	nonDEG	2.3112371	1	1
1.39973595	0.01017129	0.1475765	nonDEG	0.28505517	0.61828439	1
-0.4149376	0.09191052	0.55242013	nonDEG	-0.4074622	0.28134173	1
-1.0332226	0.227991	0.80924485	nonDEG	0.2690693	0.78786436	1
-1.260795	0.02693257	0.27868816	nonDEG	-0.1593009	0.86263117	1
-1.0188545	7.64E-05	0.00355242	DEG	-0.1339716	0.80475552	1
1.03301428	0.01323033	0.17505068	nonDEG	0.53853485	0.26155683	1
-0.7691504	0.01836475	0.21668935	nonDEG	-0.5847528	0.18758371	1
0.62536455	0.08189857	0.52356398	nonDEG	0.68065231	0.09885484	1
0.38330987	0.13658628	0.66368058	nonDEG	0.38752369	0.27898801	1
1.50234029	5.41E-05	0.00270375	DEG	0.64692895	0.15388738	1
0.09275015	0.77723915	1	nonDEG	-0.8194536	0.05751114	1
1.39112314	0.0073015	0.11651775	nonDEG	0.29278518	0.59696985	1
-0.4471256	0.17252603	0.73142193	nonDEG	-0.5572516	0.27550918	1
1.30465184	0.00466705	0.08444543	nonDEG	0.13378787	0.79703366	1
0.40170075	0.3867167	0.94352336	nonDEG	0.73091674	0.14932871	1
0.50016587	0.375028	0.93317972	nonDEG	0.84511153	0.16675129	1
-2.524641	0.02512437	0.26586493	nonDEG	-1.7150679	0.30589439	1
-1.210076	6.74E-05	0.00320635	DEG	-0.4428635	0.33243642	1
0.51955368	0.34774027	0.91917773	nonDEG	0.51863273	0.32237676	1
-0.6724441	0.21184428	0.78881213	nonDEG	1.63402053	0.00054216	0.14642867
0.51755708	0.09931404	0.57417636	nonDEG	0.15563311	0.69517395	1
-0.5947895	0.02445553	0.26160845	nonDEG	0.05143149	0.90131598	1
1.40135517	1.97E-08	3.51E-06	DEG	0.20556507	0.58799631	1
-0.1545651	0.61752934	1	nonDEG	-0.2130773	0.62465203	1
-0.4390144	0.25290797	0.83393344	nonDEG	-0.1626596	0.74905738	1
-0.0250605	0.96676833	1	nonDEG	1.36705794	0.0054657	0.58529482
1.25803717	0.14155465	0.67177078	nonDEG	-0.6264361	0.54182253	1
0.67334377	0.42621477	0.96764883	nonDEG	0.07772226	0.94278191	1

-0.2142657	0.8031124	1	nonDEG	1.82222045	0.05213584	1
-0.5059486	0.08867563	0.54465303	nonDEG	0.31007746	0.41928708	1
0.23916804	0.40628243	0.95611442	nonDEG	0.4652526	0.37227425	1
-0.7059086	0.00827144	0.12722196	nonDEG	0.18235281	0.6800336	1
-0.1152874	0.75355008	1	nonDEG	-1.0626213	0.06994653	1
0.44233013	0.09039315	0.54811204	nonDEG	0.66826152	0.06480087	1
1.59643915	0.00010232	0.00450244	DEG	0.53849889	0.35614605	1
0.26781237	0.32465557	0.90838968	nonDEG	0.54781834	0.13134169	1
-0.3912758	0.14513244	0.67913112	nonDEG	-0.1898037	0.61086835	1
-0.5299649	0.26886768	0.85210766	nonDEG	-0.5463912	0.3658362	1
1.15896616	0.05233332	0.41459648	nonDEG	0.06303281	0.93057479	1
0.01195686	0.96378709	1	nonDEG	0.45153924	0.24626828	1
-0.5788328	0.02438275	0.26130459	nonDEG	0.10618097	0.83538012	1
0.58501376	0.44841186	0.98329655	nonDEG	1.27186223	0.09168865	1
-0.1388952	0.58378988	1	nonDEG	-0.0037339	0.9937017	1
0.02219728	0.96461991	1	nonDEG	1.16103319	0.04582615	1
-1.5912175	0.01462338	0.18701035	nonDEG	-1.4421524	0.11400239	1
-1.6929492	0.00673526	0.10953391	nonDEG	-0.7800673	0.3587191	1
0.83190496	0.00815116	0.1262735	nonDEG	0.42219289	0.30183304	1
0.76165996	0.00478612	0.08586151	nonDEG	-0.293487	0.45068302	1
0.45251172	0.07504855	0.5022401	nonDEG	0.39419711	0.36027327	1
2.16508547	0.05062129	0.40650301	nonDEG	-0.3207986	0.78293785	1
-2.0438867	2.44E-05	0.00145501	DEG	-2.7033154	0.00777185	0.6829146
-0.2368918	0.36955879	0.92855021	nonDEG	-0.2592222	0.51969095	1
-0.0372546	0.89611902	1	nonDEG	-0.8370897	0.03624979	1
0.94502807	0.00019752	0.00759126	DEG	0.13107013	0.72669674	1
0.19652148	0.42095521	0.96387186	nonDEG	-1.1624537	0.00311995	0.4415588
-1.769514	0.01283189	0.17202094	nonDEG	-0.9078344	0.38572083	1
-0.3752571	0.31816269	0.90098849	nonDEG	-0.7734276	0.1195519	1
1.46784116	0.04498741	0.37964291	nonDEG	0.66896242	0.34953276	1
-1.3314095	2.55E-07	3.00E-05	DEG	-0.5932178	0.27269437	1
-0.9856229	0.00063007	0.01908308	DEG	-0.0311411	0.93502499	1
-1.6299215	0.02720609	0.28043417	nonDEG	-0.7145376	0.46732626	1
1.02790443	0.0010618	0.02818598	DEG	0.16784387	0.70476083	1
0.34900922	0.15749883	0.70294241	nonDEG	0.45775328	0.24996972	1
0.39575681	0.13100963	0.65447014	nonDEG	0.31597927	0.4112657	1
0.14411587	0.56419132	1	nonDEG	-0.3261543	0.54710163	1
-0.0561259	0.87502704	1	nonDEG	-0.7228081	0.21982059	1
1.44021784	4.87E-06	0.00039192	DEG	-0.2002536	0.61806169	1
0.36482401	0.2395949	0.82140845	nonDEG	0.01823593	0.9674792	1
-2.4323312	0.00443145	0.08187808	nonDEG	-1.4215811	0.28597413	1
0.93636238	0.00136874	0.03420582	DEG	0.23804099	0.53881043	1
1.11251938	0.06498245	0.46602151	nonDEG	-0.4758885	0.52378711	1

0.62309522	0.03637759	0.33586539	nonDEG		0.10493666	0.79255309	1
4.10798435	0.01771101	0.21177679	nonDEG		-0.7503172	0.64151413	1
-1.3423263	0.30956521	0.89293457	nonDEG		-6.2558356	0.02162879	1
-0.5002812	0.05719133	0.43543403	nonDEG		-0.3184748	0.4089918	1
1.42726271	1.65E-05	0.00105563	DEG		-0.3454868	0.39831332	1
-1.1230827	0.27590953	0.85842562	nonDEG		-0.9023738	0.48474118	1
-1.7317552	0.00047894	0.01544041	DEG		-0.628856	0.41454073	1
2.79700885	2.85E-14	2.21E-11	DEG		-1.9254398	0.00041745	0.12289217
-0.7998968	0.00173484	0.04132036	DEG		-0.2770696	0.49022625	1
-0.2818315	0.28667418	0.86893869	nonDEG		0.05176925	0.88709717	1
1.52806897	1.06E-08	2.03E-06	DEG		0.12747767	0.72864035	1
0.38249782	0.34191297	0.91869633	nonDEG		-0.9473402	0.08654784	1
0.4313125	0.10485937	0.59272458	nonDEG		0.40467187	0.28531098	1
-0.6088328	0.02388217	0.25783613	nonDEG		-0.2734339	0.4931917	1
0.98806954	0.0144626	0.1857611	nonDEG		0.27548159	0.57431948	1
0.85949209	0.00685911	0.11122554	nonDEG		0.5973321	0.1042218	1
2.16126486	0.00236146	0.05153383	nonDEG		-0.1331484	0.88090393	1
0.42235392	0.11840129	0.62843136	nonDEG		0.24664009	0.51246741	1
-2.0242781	0.00426951	0.07975268	nonDEG		-1.6818073	0.23379587	1
0.31498375	0.32631247	0.91057556	nonDEG		-0.0317394	0.93929343	1
-1.5566893	0.01220678	0.1674536	nonDEG		0.15086991	0.85979862	1
0.14715871	0.76911306	1	nonDEG		-1.4062063	0.0324547	1
0.94029523	0.00031425	0.01118661	DEG		0.39661382	0.28227291	1
0.58079716	0.02843452	0.2886496	nonDEG		0.2581737	0.49437857	1
-0.7429374	0.37812522	0.93529818	nonDEG		1.51246417	0.15539008	1
-0.1676795	0.63059322	1	nonDEG		0.23992782	0.56440246	1
-0.6136046	0.15454866	0.69582432	nonDEG		-1.3474053	0.04210715	1
-0.8239462	0.00737198	0.1173736	nonDEG		-0.9292638	0.08194029	1
0.4744088	0.07318253	0.49401735	nonDEG		0.44945664	0.21929038	1
3.6218382	0.12735082	0.64810083	nonDEG		-0.0789197	1	1
0.87180131	0.00342543	0.06850629	nonDEG		0.61478388	0.09034271	1
-2.9554432	0.00212508	0.0479756	DEG		-0.0087176	1	1
2.80167882	1.20E-18	2.20E-15	DEG		0.18370575	0.66244749	1
0.66427474	0.40823906	0.95762149	nonDEG		1.32128348	0.11365781	1
0.74035129	0.58527243	1	nonDEG		2.09029624	0.08130087	1
-0.9329414	0.03801158	0.34369589	nonDEG		-0.3647659	0.63889064	1
1.21520291	4.78E-06	0.00038574	DEG		0.34242952	0.37137968	1
0.94958878	0.00642833	0.1060189	nonDEG		0.23785511	0.57423943	1
-1.3646558	4.01E-08	6.48E-06	DEG		-0.4722102	0.25599093	1
-1.8609347	2.13E-11	9.50E-09	DEG		-0.6012164	0.15845919	1
-1.1760117	0.05695889	0.43418247	nonDEG		-2.4526018	0.05416058	1
-2.1252691	5.01E-08	7.81E-06	DEG		-0.0457232	0.94081949	1
-2.3344733	7.11E-13	4.54E-10	DEG		0.86693026	0.26628802	1

0.43039153	0.54094673	1	nonDEG	0.56680706	0.52252407	1
-0.6950061	0.025716	0.27037618	nonDEG	-1.2479961	0.00279882	0.42471307
-0.200854	0.60054514	1	nonDEG	-0.2122553	0.72822043	1
1.36099232	4.28E-07	4.56E-05	DEG	0.53463992	0.23798299	1
0.24663254	1	1	nonDEG	1.35561349	0.40119173	1
1.58964014	3.21E-07	3.67E-05	DEG	-0.1957817	0.64438687	1
-1.8514036	0.00027602	0.00999643	DEG	-1.313871	0.08360487	1
-0.3044123	0.5108882	1	nonDEG	-1.2905824	0.05711521	1
-0.8400842	0.05252481	0.41544273	nonDEG	-0.5767786	0.28092719	1
0.53249905	0.18702563	0.75310345	nonDEG	0.28851085	0.56183617	1
0.80592958	0.00485533	0.08668051	nonDEG	0.52019678	0.1651544	1
0.76926267	0.1683245	0.72428041	nonDEG	-1.7337357	0.03653822	1
0.81857377	0.22310149	0.80348199	nonDEG	0.86106078	0.31350312	1
-0.5955979	0.2748848	0.85835498	nonDEG	-1.3009205	0.07457508	1
-0.3647366	0.17788323	0.74368492	nonDEG	-0.1544936	0.68497645	1
0.04426719	0.87266245	1	nonDEG	0.74044404	0.04183079	1
0.23647424	0.59908226	1	nonDEG	0.63240008	0.13664974	1
0.98869546	0.00495347	0.08792926	nonDEG	0.58862515	0.23355804	1
0.43757816	0.08542066	0.53504228	nonDEG	0.11884743	0.74663431	1
0.20621041	0.81884672	1	nonDEG	0.0018912	1	1
1.80756971	0.00013541	0.00564654	DEG	0.70522999	0.21570756	1
0.60682371	0.27706679	0.85916381	nonDEG	0.20936092	0.73834819	1
-0.6194835	0.07037716	0.48543418	nonDEG	-0.3471152	0.47631325	1
0.80016057	0.00194395	0.04499061	DEG	0.24636387	0.52146422	1
0.58904969	0.05325141	0.41819366	nonDEG	0.23337432	0.56953243	1
0.13746908	0.82973395	1	nonDEG	0.72774315	0.31878245	1
-0.4496393	0.13435067	0.65963131	nonDEG	-0.1219713	0.83461785	1
1.49597344	7.30E-06	0.00055204	DEG	-0.1151399	0.77667606	1
0.11284303	0.66094386	1	nonDEG	0.57662348	0.1251252	1
1.46287148	3.25E-05	0.00181315	DEG	0.34739423	0.37933695	1
-0.2192481	0.61265765	1	nonDEG	-0.8601705	0.12635939	1
-0.8990853	0.03013284	0.29847941	nonDEG	-0.1585338	0.78896486	1
0.95563214	0.00133574	0.03369573	DEG	0.66971128	0.13000984	1
0.27398746	0.28410924	0.86663475	nonDEG	0.51169973	0.16066909	1
-2.372586	6.75E-20	1.53E-16	DEG	-0.3752512	0.60281954	1
1.46808166	4.41E-07	4.67E-05	DEG	-0.1593469	0.6734399	1
-2.9578275	1.22E-17	1.71E-14	DEG	0.23280396	0.73998533	1
-0.2383443	0.49309523	1	nonDEG	-0.1232118	0.79443636	1
-1.0458243	0.06148369	0.4531844	nonDEG	-1.5018425	0.05748309	1
0.39129946	0.12640076	0.64619186	nonDEG	0.49255044	0.23652351	1
-0.4902515	0.40744005	0.95694175	nonDEG	0.89841954	0.11354389	1
1.12856203	0.00044475	0.01474452	DEG	-0.0021052	0.99712286	1
1.79386411	8.66E-11	3.23E-08	DEG	-0.8658813	0.02609375	1

1.01555184	0.039657	0.35260113	nonDEG	-0.2276475	0.67164252	1
-2.253493	0.00257597	0.05499211	nonDEG	-1.6684406	0.16695221	1
0.56974335	0.42554206	0.96698651	nonDEG	0.70409112	0.37997748	1
-0.2088887	0.86163055	1	nonDEG	1.81247604	0.09656533	1
1.22485545	6.06E-06	0.00046745	DEG	0.27077573	0.45976808	1
-1.32668	0.25428817	0.83604974	nonDEG	-1.6603361	0.37355447	1
-0.6175403	0.0176325	0.21104954	nonDEG	-0.0495139	0.89687939	1
0.25197584	0.35275551	0.91917773	nonDEG	0.4704755	0.30033454	1
4.58877578	2.39E-22	7.24E-19	DEG	-0.4249966	0.55301638	1
1.09723147	0.01666571	0.20298236	nonDEG	0.573336	0.26674013	1
1.56240149	0.07212008	0.49010687	nonDEG	0.49339852	0.54045172	1
0.33144868	0.20111757	0.77336631	nonDEG	0.66405558	0.08542308	1
0.82117074	0.00198266	0.04566103	DEG	0.24185526	0.53424865	1
0.53944098	0.04618356	0.38621947	nonDEG	0.55153198	0.12519625	1
1.23395715	0.00704224	0.11334479	nonDEG	0.29248438	0.55940967	1
1.76172041	3.05E-05	0.00173001	DEG	0.16897136	0.77610466	1
-1.181199	1.08E-06	0.00010712	DEG	-0.4097603	0.40050444	1
1.04361805	3.00E-05	0.00171401	DEG	0.13534422	0.71124322	1
0.45401725	0.06684356	0.47280334	nonDEG	0.20054229	0.60796183	1
-1.1969821	0.00047911	0.01544041	DEG	0.15010952	0.7600946	1
-1.3455965	0.01900262	0.22190327	nonDEG	0.1580245	0.84775564	1
0.61803237	0.05416869	0.42120234	nonDEG	0.23813576	0.54228091	1
-0.7082071	0.08645265	0.53862182	nonDEG	-0.5270158	0.39329732	1
1.52677127	6.14E-09	1.29E-06	DEG	0.04832563	0.90568256	1
0.27619755	0.31769556	0.9005912	nonDEG	0.58507323	0.23451738	1
1.50291535	0.16298579	0.714154	nonDEG	0.50340096	0.68906023	1
1.81957282	0.00564252	0.09663188	nonDEG	-1.9141371	0.00763847	0.67731627
2.1644283	0.00442588	0.08187808	nonDEG	1.17708687	0.09584472	1
0.77562095	0.0035858	0.07089473	nonDEG	0.02528466	0.944474	1
1.89036838	0.000104	0.00455605	DEG	-0.6433907	0.30884125	1
-0.5771916	0.03113715	0.30534528	nonDEG	-0.2805317	0.52709663	1
0.79035456	0.00181462	0.04266827	DEG	0.55383445	0.15697102	1
0.17963777	0.55535468	1	nonDEG	0.80181081	0.04234419	1
-0.2969334	0.24003358	0.82187354	nonDEG	-0.2071214	0.58215028	1
-2.4705502	3.36E-08	5.46E-06	DEG	-2.128752	0.00555975	0.5887532
-1.1445717	0.16522504	0.71762465	nonDEG	1.94581019	0.00265853	0.40976087
-0.6635443	0.06418346	0.46356644	nonDEG	-0.3466589	0.49549265	1
0.5025242	0.05948774	0.44482082	nonDEG	0.17714794	0.62769791	1
-0.4583842	0.13038202	0.65446487	nonDEG	0.72096549	0.30141008	1
0.54086665	0.02882025	0.29135953	nonDEG	0.21381311	0.56892103	1
1.34468956	0.0031403	0.06411042	nonDEG	0.20926409	0.67977192	1
1.43012387	3.95E-05	0.00211924	DEG	0.27604886	0.49463767	1
0.62484093	0.0892806	0.54643065	nonDEG	0.12981986	0.75916655	1

0.30204576	0.32053204	0.90358544	nonDEG	0.11668584	0.78485491	1
0.54798403	0.05813108	0.43925074	nonDEG	-0.1231325	0.78140803	1
-1.417725	5.39E-05	0.00269641	DEG	-0.2165406	0.69936343	1
-0.4058624	0.21332779	0.79115104	nonDEG	-0.2371992	0.63174287	1
0.40347458	0.51219732	1	nonDEG	1.00327685	0.09809479	1
-1.4445533	0.03578986	0.33244726	nonDEG	-1.5882562	0.13342068	1
-0.4715873	0.06658609	0.47192054	nonDEG	-0.679726	0.064277	1
-0.2262518	0.84034017	1	nonDEG	1.2651918	0.30483261	1
-1.3546921	0.00097326	0.02621398	DEG	-0.143596	0.75246168	1
2.29911228	0.02143185	0.23953385	nonDEG	-0.1681156	0.87038407	1
0.97008406	0.00176597	0.04189226	DEG	0.54644548	0.20837477	1
-0.1280168	0.70520965	1	nonDEG	-0.9021267	0.04343594	1
-1.6362124	0.00910398	0.13695041	nonDEG	-0.2719822	0.77592596	1
-2.1679916	7.65E-05	0.00355242	DEG	-1.5279429	0.12680674	1
0.51330549	0.25315166	0.83442231	nonDEG	0.06036489	0.91197079	1
-1.5585164	0.32745593	0.91149537	nonDEG	-1.1680976	0.71269503	1
1.17750397	0.15188367	0.68990947	nonDEG	0.86540692	0.36757759	1
-0.6592539	0.03225053	0.31200237	nonDEG	-1.0850133	0.01747975	0.95300701
-0.6818073	0.006669	0.10901089	nonDEG	-0.4547734	0.22902056	1
-0.2803485	0.37054635	0.92909582	nonDEG	-0.3089068	0.47273827	1
0.16344731	0.80989244	1	nonDEG	2.37085169	5.60E-05	0.03053571
-1.1255835	3.03E-05	0.00172742	DEG	-0.3390301	0.40262386	1
-0.9992298	0.00025047	0.00917128	DEG	-0.4177494	0.34676662	1
-1.1735008	0.14398402	0.67658948	nonDEG	-8.4070784	2.79E-05	0.0195581
0.27155993	0.27120844	0.85427653	nonDEG	0.47220898	0.24351241	1
0.19307955	0.4845773	0.9962617	nonDEG	-0.9670006	0.0104253	0.77777211
1.60918201	2.05E-09	4.96E-07	DEG	0.17715026	0.64124354	1
-0.8560018	0.01204254	0.16613903	nonDEG	-1.0558771	0.08052668	1
-0.1874591	0.63457128	1	nonDEG	-0.9268162	0.07824632	1
-0.6776984	0.16035947	0.70968469	nonDEG	-0.4722742	0.43562665	1
1.18364203	0.01239189	0.16844031	nonDEG	0.09189433	0.85810908	1
-0.3094426	0.6421553	1	nonDEG	0.87750587	0.19892022	1
0.67541941	0.04123152	0.36071762	nonDEG	0.27512527	0.56123218	1
0.26190635	0.32384433	0.90721721	nonDEG	0.5202908	0.20553922	1
-1.1060713	3.20E-05	0.00179168	DEG	-0.6509244	0.14165895	1
1.02696369	0.01011082	0.14706146	nonDEG	0.93755825	0.02927109	1
-0.585961	0.04331953	0.37086528	nonDEG	-0.4770856	0.2381043	1
0.15292671	0.5544377	1	nonDEG	0.64934433	0.15091229	1
0.66221785	0.01192428	0.16515005	nonDEG	0.22453554	0.53932696	1
1.93954492	0.00071329	0.02087336	DEG	0.69821125	0.30692409	1
0.44025211	0.10293261	0.58565765	nonDEG	0.69148023	0.06698558	1
-0.2157988	0.77487157	1	nonDEG	-1.5491868	0.15173417	1
0.60124436	0.02075219	0.23515157	nonDEG	0.47325527	0.26136669	1

-7.5848125	3.60E-05	0.00197686	DEG	0.61557782	1	1
-1.3306882	0.05050551	0.40607126	nonDEG	-1.3420157	0.25775505	1
-0.3086169	0.32527399	0.90946768	nonDEG	-0.9451144	0.0531293	1
0.367725	0.23838936	0.82044546	nonDEG	0.26708677	0.51152948	1
1.05862881	5.73E-05	0.00281641	DEG	0.03047745	0.93428438	1
-0.629772	0.01507665	0.19106398	nonDEG	-0.4452897	0.22295343	1
1.07377297	0.02112571	0.23725952	nonDEG	-2.1013263	0.01163558	0.80926302
0.23110956	0.46104516	0.98479636	nonDEG	0.71296787	0.05508145	1
0.8663127	0.00169265	0.04054502	DEG	-0.3226513	0.38781539	1
0.53941758	0.0577904	0.43768758	nonDEG	-0.0838022	0.85528438	1
-0.3318764	0.42470067	0.96646612	nonDEG	-0.9096353	0.11449954	1
0.68863623	0.39945932	0.95204649	nonDEG	0.81476229	0.3647335	1
0.03477129	0.9613446	1	nonDEG	0.82413135	0.24523267	1
-0.6427472	0.02103552	0.23681245	nonDEG	0.6238861	0.15816805	1
0.63566263	0.0325068	0.31312418	nonDEG	0.45720407	0.34027456	1
1.04451245	0.00011477	0.00493228	DEG	0.18954061	0.61130836	1
-0.9378236	0.00035886	0.01244331	DEG	-0.2691953	0.49941274	1
1.98084881	4.66E-14	3.52E-11	DEG	0.14155104	0.69995563	1
-0.4778442	0.12823014	0.65074417	nonDEG	-0.5157504	0.23498429	1
-0.9015066	0.06463247	0.46487057	nonDEG	-0.9709185	0.14262264	1
-0.5895916	0.02235361	0.24711531	nonDEG	-0.1682627	0.64460162	1
-0.3553223	0.31243178	0.89535767	nonDEG	-0.7020931	0.12132292	1
-1.8603008	0.00228288	0.05029026	nonDEG	-1.3972733	0.10234576	1
-1.5568017	0.00669449	0.10923913	nonDEG	-0.743108	0.21160399	1
-0.2447299	0.33797913	0.91869633	nonDEG	-0.269139	0.46258797	1
0.44179966	0.06735964	0.47428858	nonDEG	-0.0019078	0.99606873	1
-2.4758596	1.08E-10	3.84E-08	DEG	-0.1614341	0.8039943	1
-0.2192181	0.48290732	0.99581563	nonDEG	-0.529884	0.21310173	1
0.49699749	0.07528452	0.50279711	nonDEG	0.76015046	0.03756813	1
-0.5568989	0.04079225	0.35847258	nonDEG	-0.841482	0.03131462	1
0.48652548	0.20292631	0.77486116	nonDEG	0.23057267	0.59990051	1
-1.6086336	0.04920698	0.4007777	nonDEG	-2.0679283	0.05687718	1
-1.1219323	0.0001875	0.00728201	DEG	-1.0627695	0.03832966	1
-0.7730664	0.00316011	0.06447022	nonDEG	-2.3369405	4.42E-06	0.00591756
-0.582949	0.08958842	0.54695012	nonDEG	-0.2381388	0.60631431	1
-0.0921753	0.80751593	1	nonDEG	1.48068658	0.00016339	0.06088513
0.12843977	0.95623857	1	nonDEG	0.16563082	0.88480358	1
-1.3569428	0.00070102	0.02063734	DEG	-0.4350202	0.54544373	1
-0.3302015	0.19026171	0.757112	nonDEG	0.16879598	0.71589261	1
-0.1033612	0.72128634	1	nonDEG	-0.658178	0.09155698	1
0.95435812	0.34512039	0.91869633	nonDEG	0.06738133	1	1
0.40405802	0.12148738	0.6343503	nonDEG	0.39590171	0.33020522	1
0.01550211	0.97449483	1	nonDEG	0.56260432	0.20244203	1

-0.3692908	0.30527351	0.89006022	nonDEG	-0.0856117	0.86644571	1
0.88158552	0.10467054	0.59212066	nonDEG	0.99842373	0.11803956	1
-0.3277812	0.23691533	0.81880142	nonDEG	0.1544171	0.70730013	1
0.68509887	0.03716352	0.34008606	nonDEG	0.39179447	0.29525291	1
1.02037725	0.02474141	0.26323184	nonDEG	0.86634981	0.13509569	1
-0.6808682	0.05694179	0.43418247	nonDEG	-0.7875152	0.10430817	1
0.07242797	1	1	nonDEG	0.82616713	0.49742739	1
-0.7320769	0.00464956	0.08433342	nonDEG	-0.054809	0.90192592	1
-0.3498203	0.36743461	0.92696488	nonDEG	-0.5642689	0.30845674	1
1.11413322	2.43E-05	0.00145382	DEG	0.0309264	0.93337207	1
-0.869853	0.25143269	0.83364422	nonDEG	-0.2104354	0.82686063	1
-3.7198712	0.01506314	0.19097492	nonDEG	0.13400808	1	1
0.38695046	0.12371376	0.6416507	nonDEG	0.06273572	0.90447788	1
-0.2337568	0.45592816	0.98394385	nonDEG	0.32487511	0.5966448	1
-0.2883658	0.57241568	1	nonDEG	1.65991172	0.00183625	0.33121517
1.94843002	0.00395294	0.07536309	nonDEG	0.1371465	0.83968848	1
0.78508894	0.32205768	0.90511274	nonDEG	0.01399795	0.98873393	1
1.10132407	2.88E-05	0.00164877	DEG	0.10629863	0.77485983	1
-0.6095231	0.08747911	0.54054722	nonDEG	-1.10682	0.01642035	0.920013
0.3734652	0.146447	0.68195607	nonDEG	0.39646661	0.36499041	1
1.58585715	0.00080386	0.02271523	DEG	-0.8359602	0.08731483	1
-1.9670325	0.01246072	0.16873447	nonDEG	-0.5780559	0.54192065	1
0.6872986	0.02143998	0.23953385	nonDEG	0.51307493	0.21042802	1
0.23607676	1	1	nonDEG	2.84518736	0.26695798	1
-0.2934571	0.26888054	0.85210766	nonDEG	-0.2402382	0.5679364	1
1.83001351	0.06521182	0.46677686	nonDEG	0.39880601	0.71189064	1
1.26734514	0.00047845	0.01544041	DEG	-0.3852728	0.36204961	1
-1.3913186	0.04943116	0.40176805	nonDEG	-0.817236	0.44027072	1
-0.4581424	0.08942677	0.54666582	nonDEG	-0.4204251	0.28961842	1
-0.7554081	0.00921755	0.13766588	nonDEG	0.40206751	0.34539535	1
0.29901754	0.30141975	0.88626579	nonDEG	0.28792374	0.45057197	1
0.32230509	0.36073455	0.92354131	nonDEG	-0.19721	0.6649248	1
-1.7757886	0.00098957	0.02653181	DEG	-1.3397257	0.11613472	1
-0.1886677	0.57366765	1	nonDEG	-0.9153933	0.05369072	1
-1.4193451	2.59E-06	0.00022506	DEG	-1.2968664	0.00769773	0.67884783
-0.6274256	0.04810902	0.39560933	nonDEG	0.08300236	0.84841413	1
-0.2262044	0.37772012	0.93522146	nonDEG	-0.1103007	0.7918992	1
1.17350283	0.00371184	0.07287659	nonDEG	0.13093284	0.78597002	1
0.86775931	0.00080555	0.02271523	DEG	0.23932576	0.50711631	1
0.48675025	0.32788269	0.9118557	nonDEG	0.51801757	0.31508871	1
1.00283633	8.92E-05	0.00404575	DEG	0.08993818	0.82302222	1
0.11844003	0.76360697	1	nonDEG	0.66459461	0.17651725	1
-0.6399464	0.0324835	0.31312418	nonDEG	-0.6596448	0.12314617	1

-0.5629349	0.08815363	0.54280584	nonDEG	-0.8906205	0.05821083	1
0.79362217	0.01613799	0.19925444	nonDEG	-1.5288098	0.00471293	0.55276492
-0.8120272	0.00338238	0.06787593	nonDEG	-0.8572721	0.16246963	1
-1.7784	3.32E-07	3.76E-05	DEG	-0.2780239	0.58767388	1
1.12209925	0.00014922	0.00606757	DEG	0.44008454	0.28522868	1
1.19396723	0.02247188	0.2479572	nonDEG	0.56723687	0.31578018	1
0.52913241	0.40851621	0.95788999	nonDEG	0.76775555	0.23928552	1
-6.2789548	0.00572772	0.0975236	nonDEG	0	1	1
-0.3434847	0.69438362	1	nonDEG	0.94254038	0.28441716	1
-0.7486543	0.02375994	0.25768009	nonDEG	-0.1669263	0.72477647	1
2.16217432	0.01799936	0.21392136	nonDEG	0.93505739	0.33541495	1
0.15613041	0.65399019	1	nonDEG	0.59897325	0.14452433	1
1.01466733	0.07639024	0.50612135	nonDEG	1.20823803	0.0551273	1
0.51514432	0.33027445	0.91411259	nonDEG	0.42469819	0.46815206	1
0.39409152	0.18861615	0.75429997	nonDEG	0.54441595	0.13714079	1
-2.4921924	0.11396416	0.61692363	nonDEG	0.8430976	0.74787574	1
0.11753081	0.84351413	1	nonDEG	1.03460465	0.03384894	1
-1.6257372	1.25E-06	0.00012021	DEG	-0.2598604	0.56752544	1
0.60548776	0.01920979	0.2233375	nonDEG	0.17886637	0.62853242	1
-0.7042703	0.02119269	0.23767301	nonDEG	-0.5352456	0.21918215	1
1.07445208	3.99E-05	0.00212911	DEG	0.40048917	0.37058276	1
1.55185285	2.24E-05	0.00136257	DEG	0.31049617	0.4656483	1
-0.2548085	0.35459202	0.91917773	nonDEG	0.05049316	0.90360453	1
-2.7927386	0.0002536	0.00925106	DEG	2.01283002	0.04958805	1
0.00510675	0.98750828	1	nonDEG	-1.021766	0.01108289	0.79590774
0.99550228	0.34149882	0.91869633	nonDEG	0.71323472	0.49599591	1
0.96499679	0.07752333	0.51021897	nonDEG	-0.5057709	0.42343573	1
0.43976425	0.0782979	0.51213419	nonDEG	0.51279811	0.18871449	1
1.40566129	4.41E-07	4.67E-05	DEG	0.41018167	0.31025126	1
0.57565894	0.13729748	0.66424004	nonDEG	0.5795481	0.18244618	1
-1.5000767	7.39E-06	0.00055552	DEG	-1.2101295	0.00366338	0.49008216
-0.804303	0.02673064	0.27737865	nonDEG	0.04637111	0.93052785	1
-0.9981079	8.41E-05	0.00385851	DEG	-0.829848	0.16347396	1
2.87201467	0.01127979	0.15891956	nonDEG	1.14732922	0.35241877	1
-0.5923733	0.28382201	0.86646664	nonDEG	-2.7303925	0.00107207	0.2294705
0.76473216	0.00223796	0.04953629	DEG	-0.0474553	0.89639704	1
1.01575082	0.02828678	0.28793435	nonDEG	0.94821082	0.04797919	1
2.39799567	0.03031242	0.29975389	nonDEG	0.80144737	0.2995724	1
0.43682912	0.14406746	0.67658948	nonDEG	-0.1566017	0.72897371	1
1.63974161	2.68E-09	6.25E-07	DEG	-0.4862052	0.2062767	1
-0.8258438	0.0215128	0.23980129	nonDEG	-0.8960263	0.05574096	1
-1.450766	0.01553467	0.19435827	nonDEG	-0.3067436	0.74970959	1
0.35937818	0.1476352	0.68333836	nonDEG	0.47217492	0.1952892	1

0.94699342	0.01450112	0.18609351	nonDEG		0.12772493	0.78582063	1
0.43605315	0.14484687	0.67846413	nonDEG		0.55713583	0.13941421	1
2.11290756	0.00641367	0.10587533	nonDEG		-0.7139735	0.3316505	1
2.0689723	6.33E-16	7.17E-13	DEG		0.308341	0.39230284	1
1.02848118	0.00170205	0.0407039	DEG		0.1234996	0.76245156	1
-0.4205331	0.12857951	0.65114404	nonDEG		0.27147601	0.51432223	1
0.73783351	0.03143468	0.30683212	nonDEG		0.31424569	0.45156388	1
1.50798076	0.0700383	0.48423144	nonDEG		2.51239776	0.0049538	0.5609031
-0.5917649	0.07616546	0.50539204	nonDEG		-0.1901835	0.65591651	1
1.44461718	1.67E-08	3.01E-06	DEG		0.5201115	0.20561552	1
0.08448884	0.95287217	1	nonDEG		1.57930477	0.08731186	1
-0.1778785	0.8288742	1	nonDEG		1.00425309	0.18576834	1
-0.5947993	0.15773511	0.70367692	nonDEG		-1.290956	0.03529311	1
-0.5440839	0.10594856	0.59602899	nonDEG		-0.4373119	0.34719071	1
-1.2402375	3.19E-05	0.00179105	DEG		0.1910463	0.7465641	1
-0.3761398	0.2585677	0.83934	nonDEG		-0.7130215	0.12789783	1
-0.0302249	0.9334521	1	nonDEG		-0.9881511	0.05640438	1
0.53472647	0.09828918	0.57137375	nonDEG		0.38848605	0.3670554	1
0.16424263	0.52107813	1	nonDEG		-0.811954	0.02880072	1
1.8683765	0.00051951	0.01648047	DEG		-0.7762361	0.2112469	1
-0.1454295	0.84103028	1	nonDEG		0.68298139	0.33805709	1
0.57057346	0.02403032	0.25884688	nonDEG		0.48997372	0.23557189	1
0.39218697	0.16106117	0.71096258	nonDEG		0.30912425	0.42001162	1
-0.2694837	0.33060733	0.91430575	nonDEG		-0.9222313	0.01554731	0.89863185
-1.2088652	0.00051795	0.01645256	DEG		-1.0517485	0.11547048	1
0.30968746	0.42491827	0.96658958	nonDEG		0.40672814	0.3722579	1
2.26027282	0.01173037	0.16358615	nonDEG		-0.8174344	0.38987284	1
-0.532826	0.08054884	0.51996813	nonDEG		-0.3050725	0.46650848	1
-0.9927841	0.13263669	0.65746616	nonDEG		-0.8349663	0.39047819	1
0.06448495	0.93379995	1	nonDEG		1.28335217	0.13154657	1
0.34443026	0.54548483	1	nonDEG		0.97513907	0.12266346	1
-1.7235399	0.00094748	0.0257943	DEG		-0.7546252	0.31755584	1
0.79692142	0.00840202	0.12869259	nonDEG		0.17789916	0.62901679	1
0.96439842	0.00039566	0.01340362	DEG		0.35045715	0.34457788	1
-0.5699017	0.01926446	0.2233886	nonDEG		-0.0938485	0.80135919	1
-0.7063849	0.00779239	0.12247746	nonDEG		-0.1775055	0.64462382	1
0.15960078	0.87497047	1	nonDEG		0.92022167	0.34330824	1
-0.5654342	0.18747959	0.75310345	nonDEG		-0.2479593	0.68711237	1
-0.44884	0.4808706	0.99426825	nonDEG		1.2697006	0.05794196	1
-0.1788362	0.88864953	1	nonDEG		4.09371556	0.00935581	0.74892342
-1.1740244	0.00115065	0.03008358	DEG		-0.2980846	0.57876523	1
1.61911875	4.77E-05	0.00243063	DEG		0.33661273	0.43782311	1
0.16105007	0.52129957	1	nonDEG		0.6280069	0.08280085	1

1.05551121	0.17317849	0.73281609	nonDEG	0.87547221	0.25352625	1
-0.6714792	0.0131451	0.17449596	nonDEG	-0.563394	0.38991096	1
1.13298453	9.14E-05	0.00411245	DEG	0.23163207	0.59808995	1
-0.6320756	0.28101163	0.86186717	nonDEG	-0.8408058	0.32912502	1
-0.4689608	0.15820819	0.70477861	nonDEG	-1.3635307	0.01485014	0.88143621
0.05602907	0.86528229	1	nonDEG	0.52615675	0.25204334	1
0.61536194	0.02442171	0.2614366	nonDEG	0.51198607	0.17709116	1
-1.1769366	5.18E-06	0.00041347	DEG	0.35105	0.37459353	1
0.72552745	0.00370931	0.07287659	nonDEG	0.21568708	0.55394395	1
-2.1700594	0.00029194	0.01046814	DEG	-2.1749755	0.01633216	0.91756207
-0.6837539	0.08496097	0.53408856	nonDEG	1.39663402	0.00525079	0.57678327
-1.3277268	2.16E-07	2.62E-05	DEG	-0.3247469	0.46410602	1
-0.6750195	0.11662731	0.62470156	nonDEG	-0.6217049	0.27378573	1
-0.8707489	0.20897346	0.78559183	nonDEG	1.5552373	0.01788513	0.95781535
-0.3132074	0.76358308	1	nonDEG	1.26810337	0.35759513	1
0.16313049	0.60676699	1	nonDEG	0.70550839	0.08781455	1
1.57995064	0.00045239	0.01491368	DEG	0.28779058	0.61138575	1
0.24045497	0.3740709	0.93251128	nonDEG	0.71814854	0.0477229	1
-0.1435016	0.58313162	1	nonDEG	-0.259593	0.48692864	1
-1.1364584	0.09126253	0.55084432	nonDEG	-1.2193	0.18548857	1
-0.6410714	0.0970184	0.56861423	nonDEG	-0.5013873	0.38160828	1
0.33657552	0.19823236	0.77044677	nonDEG	0.18824947	0.64607231	1
0.72325112	0.03526402	0.32988161	nonDEG	0.26382363	0.54304199	1
-0.7327214	0.00349243	0.06937488	nonDEG	-0.6713946	0.06765096	1
0.55505318	0.03356709	0.31969638	nonDEG	0.35389153	0.33689808	1
0.68796302	0.00888678	0.13430079	nonDEG	0.74333454	0.04584248	1
1.05259119	0.01442093	0.18563082	nonDEG	-0.0638269	0.89809327	1
0.99815703	0.02790165	0.28540533	nonDEG	1.11051939	0.21238045	1
0.307211	0.49202136	0.99983549	nonDEG	0.45335226	0.46614159	1
-0.9030319	0.00089251	0.02455557	DEG	0.82094636	0.0963335	1
0.087515	0.74846493	1	nonDEG	-0.4094166	0.27652994	1
-0.3021263	0.48165036	0.99447672	nonDEG	-1.1953608	0.02978317	1
-0.159744	0.61615289	1	nonDEG	-1.0755605	0.0882626	1
0.48164278	0.04889698	0.39952213	nonDEG	0.37790899	0.33512785	1
1.15619644	0.00841906	0.1288865	nonDEG	0.59460927	0.31840252	1
-0.4858624	0.06011114	0.44698457	nonDEG	-0.1174793	0.7584356	1
0.18085126	0.79504493	1	nonDEG	1.41609342	0.03233036	1
-0.8584042	0.18642934	0.75310345	nonDEG	-3.2309913	0.00236976	0.38006158
0.66218027	0.09697341	0.56857201	nonDEG	0.72359848	0.10622402	1
0.64488864	0.03672102	0.33787311	nonDEG	0.32424487	0.39394705	1
1.16556727	0.00017276	0.00679916	DEG	0.79960492	0.05961416	1
0.60929268	0.1081344	0.60205163	nonDEG	0.50066735	0.23880388	1
0.61678708	0.01296647	0.17303717	nonDEG	0.42369458	0.24474705	1

-0.171862	0.48627377	0.99711758	nonDEG	0.00991874	0.97844422	1
-0.7679381	0.00288933	0.06005192	nonDEG	-0.3883738	0.48875374	1
0.8629408	0.00055549	0.01728642	DEG	0.52589195	0.14951582	1
-1.516668	5.15E-06	0.0004116	DEG	-0.0894243	0.8479948	1
0.35753071	0.64673258	1	nonDEG	0.49584919	0.47601879	1
-0.5063196	0.03903972	0.34897353	nonDEG	0.17392075	0.66597455	1
1.28367162	0.04823159	0.39562065	nonDEG	-0.9609673	0.22023188	1
0.73150491	0.01233779	0.16835912	nonDEG	0.09518959	0.80625944	1
0.604664	0.42515961	0.9668071	nonDEG	0.6295331	0.41267429	1
0.24113298	0.50590724	1	nonDEG	0.6082718	0.16895871	1
1.11070135	0.00026578	0.00968367	DEG	0.23010339	0.57079813	1
2.25137004	1.32E-18	2.29E-15	DEG	0.33340924	0.52375189	1
-1.0423902	0.06181668	0.45495529	nonDEG	-2.0402966	0.01031541	0.77777211
-0.8209517	0.2257847	0.80685891	nonDEG	-2.1277963	0.02080778	1
-0.6474325	0.02057339	0.23348498	nonDEG	-0.2614538	0.51311743	1
-0.7022519	0.0268459	0.27798676	nonDEG	-0.6234396	0.15219856	1
-0.7544054	0.00504593	0.08894993	nonDEG	-0.7331422	0.09068989	1
-0.1873512	0.47390244	0.99085357	nonDEG	-0.4771244	0.23765527	1
1.34571868	3.55E-05	0.00195345	DEG	-0.6333333	0.11854344	1
-0.3051634	0.2508927	0.83275514	nonDEG	0.38192275	0.30726226	1
-0.0703375	0.85733649	1	nonDEG	-0.835169	0.09794906	1
0.65271657	0.01375875	0.17945226	nonDEG	0.38022839	0.29352766	1
-0.6029997	0.01361853	0.17813217	nonDEG	-0.4923789	0.22519593	1
-1.8644312	2.78E-06	0.00024028	DEG	-0.4479449	0.51146385	1
0.3851935	0.2275757	0.80922828	nonDEG	0.62748583	0.0977268	1
-0.7801162	0.13385568	0.65907076	nonDEG	-0.4792041	0.47486204	1
1.31786638	0.00059041	0.01820022	DEG	0.13536443	0.74142354	1
0.39995392	0.12564806	0.64431252	nonDEG	0.44630872	0.23344612	1
0.32186446	0.18390606	0.75158838	nonDEG	0.49580923	0.18087897	1
1.05036155	5.54E-05	0.00275725	DEG	-0.0015287	0.99706176	1
1.10284185	0.16328357	0.714154	nonDEG	-0.3219856	0.7060179	1
-0.3680156	0.22498223	0.80614069	nonDEG	-0.3159363	0.40328866	1
-4.086549	0.00199877	0.04589851	DEG	0.06627772	1	1
-1.8757665	3.89E-11	1.55E-08	DEG	0.21223781	0.68679338	1
0.18375694	0.65822753	1	nonDEG	0.48843597	0.24578034	1
-1.2214478	5.65E-05	0.0027882	DEG	-0.5126035	0.25331703	1
-0.5434308	0.14921372	0.6856099	nonDEG	-1.3649455	0.01139666	0.8038671
-0.6160756	0.17650652	0.7411461	nonDEG	-0.8830966	0.14839101	1
-0.8118654	0.14309311	0.67562441	nonDEG	-1.4969182	0.05239213	1
-0.0909407	0.70986318	1	nonDEG	-0.8231562	0.04487313	1
-2.2837225	6.08E-10	1.66E-07	DEG	-1.1639498	0.08794109	1
-0.4085572	0.11848226	0.62868959	nonDEG	-0.0379006	0.91904007	1
1.25494917	5.98E-06	0.00046317	DEG	-0.3173864	0.41439916	1

0.585092	0.11371815	0.61607445	nonDEG	0.48724248	0.28038255	1
-0.7120375	0.00644051	0.1060461	nonDEG	0.41893607	0.31724084	1
-0.8511256	0.38728536	0.94412832	nonDEG	-1.7209814	0.17858224	1
-2.4043995	1.89E-09	4.64E-07	DEG	-1.2638019	0.07523987	1
0.76990629	0.42628423	0.96769119	nonDEG	1.18629312	0.21556631	1
-0.5787656	0.04677171	0.38917814	nonDEG	0.40679855	0.28908049	1
-0.7636517	0.06480651	0.46553181	nonDEG	-0.176024	0.75433262	1
-0.3455253	0.17642991	0.74114157	nonDEG	-0.2182793	0.56149509	1
-0.7727532	0.01240368	0.16850574	nonDEG	-0.5387285	0.21223257	1
-0.607385	0.09114507	0.5505967	nonDEG	-0.2319109	0.65076424	1
0.75438112	0.0041624	0.07834841	nonDEG	0.3379723	0.3704803	1
1.91045668	0.00076898	0.02210244	DEG	0.05065356	0.94269424	1
0.54262766	0.21703127	0.79477343	nonDEG	0.41704186	0.39206973	1
0.44140411	0.20460723	0.77865662	nonDEG	0.56665535	0.16078246	1
-0.9762887	0.004952	0.08792926	nonDEG	-0.5437438	0.26983987	1
0.31358573	0.20093758	0.77325509	nonDEG	0.46921496	0.23371644	1
-0.2838284	0.27908592	0.86067572	nonDEG	-0.5372806	0.19798781	1
-0.5073502	0.05243416	0.41517191	nonDEG	-0.4582774	0.22222952	1
1.04548612	0.00426239	0.07967013	nonDEG	-0.6575389	0.14891457	1
-1.8423933	0.00012842	0.00540832	DEG	-0.1634141	0.82810037	1
-1.6944486	1.61E-05	0.00104117	DEG	-0.7072728	0.21479913	1
0.98012881	0.00666818	0.10901089	nonDEG	0.05163192	0.92161478	1
0.59556709	0.06358519	0.46173765	nonDEG	0.24361751	0.53531566	1
-1.4738135	3.02E-06	0.00025583	DEG	-0.1088094	0.82731542	1
0.22326347	0.364969	0.92544853	nonDEG	0.29531703	0.41824092	1
0.75010335	0.15274813	0.69191447	nonDEG	0.97614906	0.09893867	1
0.85541141	0.00076569	0.0220591	DEG	0.08439956	0.81761221	1
-0.2797198	0.6070282	1	nonDEG	1.25111103	0.01812925	0.95785021
0.52799769	0.07088023	0.48662383	nonDEG	0.9817565	0.02218271	1
0.59674428	0.09558928	0.5642777	nonDEG	0.06825448	0.87494565	1
0.30576273	0.33997043	0.91869633	nonDEG	0.81683411	0.03697428	1
-0.853603	0.00151566	0.03700964	DEG	-1.3942996	0.00432452	0.52515749
-0.9468832	0.07319894	0.49401735	nonDEG	-0.5091872	0.46014859	1
1.34393761	0.01184116	0.16450771	nonDEG	0.32746984	0.57410982	1
-0.0549876	0.9034941	1	nonDEG	1.36969383	0.01670212	0.92947797
-0.4801199	0.06231859	0.45672559	nonDEG	-0.3914713	0.29366136	1
-1.3367789	0.00113779	0.02980029	DEG	-0.563285	0.32368256	1
1.46742822	0.0003204	0.0113369	DEG	-0.1074198	0.81138063	1
0.9951474	0.00011066	0.00477758	DEG	0.45301324	0.36946048	1
0.73054854	0.14755561	0.68332382	nonDEG	0.26428329	0.63966115	1
-0.9268781	0.0017555	0.0417111	DEG	-0.2947947	0.55983415	1
-0.1833963	0.80120144	1	nonDEG	1.19360353	0.12397255	1
1.55295972	1.05E-08	2.03E-06	DEG	0.10176376	0.77761619	1

-0.7090403	0.00385932	0.07457224	nonDEG	0.30939119	0.52476256	1
1.07064962	0.00389625	0.07487047	nonDEG	0.67771859	0.11020852	1
-1.8403903	0.00277302	0.05806192	nonDEG	-1.1518553	0.2082352	1
-2.65479	5.66E-16	6.66E-13	DEG	-1.0461092	0.13336262	1
-1.3648615	1.70E-07	2.16E-05	DEG	-0.0916723	0.83256309	1
-3.2821704	0.00038381	0.01304696	DEG	-3.26337	0.02684422	1
2.57641206	0.00482521	0.08629981	nonDEG	-0.8028527	0.21645699	1
-0.5151666	0.04767646	0.39315049	nonDEG	-0.4813492	0.20425191	1
0.60912951	0.20000211	0.7731247	nonDEG	0.01670129	0.97891699	1
0.66515988	0.59054221	1	nonDEG	0.97613841	0.35391138	1
-0.4905887	0.09166617	0.55224625	nonDEG	-0.2618806	0.48648747	1
1.06376494	0.00015922	0.00636004	DEG	0.17793249	0.62746763	1
-0.3931229	0.11177112	0.61103621	nonDEG	-0.2001278	0.58480046	1
0.45171749	0.08098025	0.5207465	nonDEG	0.34444179	0.41763746	1
-1.2967931	0.1113502	0.60975417	nonDEG	-0.8417846	0.33995336	1
-0.5290403	0.2055237	0.7796923	nonDEG	-0.8668344	0.11917261	1
0.59994414	0.02100587	0.23665975	nonDEG	0.24545037	0.5193249	1
-1.4669661	0.00631579	0.1048678	nonDEG	-0.1390229	0.86173911	1
-0.1380557	0.69524662	1	nonDEG	-0.6840366	0.13732598	1
-0.4812079	0.06984885	0.48335992	nonDEG	-0.0036098	0.99373752	1
-0.0470629	0.97485743	1	nonDEG	-1.3437934	0.15828356	1
-1.5659819	0.00463113	0.08415784	nonDEG	-0.8919892	0.25367325	1
1.57482244	0.01062958	0.15286963	nonDEG	0.60114008	0.36362361	1
-0.7013479	0.13128729	0.65482567	nonDEG	2.74100809	7.94E-05	0.03856757
1.05291723	0.00134733	0.03381419	DEG	0.82684773	0.12083312	1
-0.5068447	0.15904021	0.70668933	nonDEG	0.18652077	0.69286664	1
0.11552986	0.71588974	1	nonDEG	0.55673845	0.17654407	1
0.36949102	0.29119909	0.87342689	nonDEG	0.63698289	0.13556214	1
-0.4951294	0.14088477	0.67024998	nonDEG	0.17445813	0.68124371	1
-0.6802082	0.18153689	0.74732027	nonDEG	1.03813093	0.01274209	0.82806693
-0.0551597	0.8764076	1	nonDEG	-1.3367664	0.00981412	0.75913347
1.05477875	0.03255124	0.31316209	nonDEG	0.14330585	0.81939372	1
0.33331461	0.18284573	0.75000403	nonDEG	0.37601194	0.34542932	1
-0.9137997	0.00017229	0.00679002	DEG	-0.2872702	0.47905152	1
1.19548679	0.14423935	0.67704211	nonDEG	0.46859983	0.50114905	1
1.69731677	1.19E-05	0.00079907	DEG	0.14335842	0.7356479	1
-0.418969	0.09842692	0.57152136	nonDEG	-0.2188781	0.62666944	1
-0.1772763	0.61938873	1	nonDEG	0.77988594	0.06903548	1
1.39637527	2.77E-08	4.58E-06	DEG	0.15510692	0.68626688	1
1.43618543	0.13268073	0.65757372	nonDEG	0.03243461	1	1
0.66770341	0.31965781	0.90293668	nonDEG	0.04810612	0.93161073	1
0.97486136	0.00472956	0.08531472	nonDEG	0.05787193	0.88026379	1
0.02610553	0.91977076	1	nonDEG	-0.4832039	0.20107051	1

-0.2911288	0.29697503	0.88208839	nonDEG	0.57783185	0.18217774	1
-0.4459379	0.07771612	0.51046073	nonDEG	-0.0233533	0.95082217	1
-0.4895518	0.05828944	0.43977008	nonDEG	-0.2240503	0.5633988	1
-1.2811836	0.03801106	0.34369589	nonDEG	-1.4895187	0.09668694	1
1.29736297	0.000598	0.0183409	DEG	0.66418583	0.25936001	1
-0.4065605	0.12381475	0.6417591	nonDEG	0.03686017	0.93971622	1
-0.3330428	0.21275207	0.79050967	nonDEG	-0.6455701	0.08416529	1
-0.4545777	0.07220672	0.49026324	nonDEG	-0.2499006	0.53157025	1
0.42005335	0.19532424	0.76607385	nonDEG	0.3892555	0.42916356	1
0.78368663	0.0224319	0.24770166	nonDEG	0.71963271	0.08368963	1
-0.8683351	0.22857091	0.80924485	nonDEG	2.03201367	0.00068201	0.1761209
-1.4068668	1.18E-05	0.00079683	DEG	-0.0292212	0.95433966	1
-0.6386888	0.01395986	0.18160156	nonDEG	-0.0454524	0.90652475	1
1.03686202	0.00126964	0.03247356	DEG	0.0524296	0.89940938	1
0.56436939	0.25180612	0.83371668	nonDEG	0.34770243	0.51951238	1
0.48501626	0.05100971	0.4084663	nonDEG	0.25730381	0.5016445	1
-0.6644032	0.0388546	0.34814714	nonDEG	0.10198314	0.82387346	1
-2.0402648	0.14563003	0.68029236	nonDEG	3.05922022	0.00240152	0.38006158
0.60288846	0.03460025	0.32595095	nonDEG	0.31650418	0.38549181	1
0.12594543	0.84993784	1	nonDEG	0.96400907	0.14758794	1
-4.9394318	1.00E-13	7.18E-11	DEG	0.48440093	0.55590231	1
1.44645235	0.00797523	0.12468547	nonDEG	0.2422065	0.68141766	1
0.60326171	0.61193038	1	nonDEG	0.8591009	0.44249215	1
0.8234152	0.00779225	0.12247746	nonDEG	0.06991711	0.85725505	1
-0.638496	0.22684116	0.80856968	nonDEG	1.16047573	0.01460099	0.87363447
-0.385537	0.37350646	0.93224331	nonDEG	0.01702746	0.97952092	1
0.28360012	0.24403535	0.82614496	nonDEG	0.37733754	0.33219583	1
0.2175905	0.62072837	1	nonDEG	0.25855042	0.55898247	1
-0.6812684	0.1383403	0.66616778	nonDEG	-1.2458864	0.05498818	1
-0.8381406	0.06225722	0.45672559	nonDEG	-0.9875883	0.12750422	1
0.12507797	0.68618799	1	nonDEG	0.45657433	0.30232329	1
0.31499318	0.37090072	0.92943023	nonDEG	0.54623758	0.1751137	1
-1.7887315	2.69E-07	3.15E-05	DEG	-1.0011559	0.04613455	1
-0.5954404	0.01825641	0.21593032	nonDEG	-0.0908709	0.87910901	1
0.54211128	0.2765525	0.85852886	nonDEG	-1.453756	0.02897295	1
-0.2961422	0.37235487	0.93131077	nonDEG	-1.2389136	0.0077944	0.6829146
-0.3378173	0.44089172	0.97742682	nonDEG	-1.1749904	0.04366271	1
-1.3202964	0.10592813	0.59602795	nonDEG	2.0900187	0.00259727	0.40455548
0.82547879	0.00812864	0.12614611	nonDEG	0.19710931	0.62834697	1
1.45624366	4.18E-06	0.00033978	DEG	-0.0201766	0.96112242	1
-0.5551203	0.43493165	0.9733779	nonDEG	1.36149311	0.07622646	1
-0.1743747	0.54210575	1	nonDEG	-1.0658429	0.01101001	0.79590774
-0.7550736	0.02114617	0.23733211	nonDEG	-0.385714	0.39375848	1

-0.7550473	0.01321908	0.17505068	nonDEG	-0.9690114	0.02665068	1
0.47056691	0.08409257	0.53067548	nonDEG	0.1094442	0.76821839	1
-2.0154817	9.96E-13	5.86E-10	DEG	-1.8721167	0.0024142	0.38006158
0.60509631	0.54281682	1	nonDEG	0.37575146	0.66084398	1
-1.1685559	0.01690326	0.20494849	nonDEG	-0.1553286	0.83806654	1
-0.7906829	0.41133857	0.95856345	nonDEG	2.08191341	0.00479012	0.55667563
1.74431692	4.67E-05	0.00239777	DEG	0.17937619	0.73768983	1
-2.9808357	1.11E-17	1.64E-14	DEG	0.5775283	0.38214149	1
-0.2756593	0.37297611	0.93165867	nonDEG	-0.3359231	0.46265647	1
0.38669806	0.40667336	0.95617157	nonDEG	0.67651755	0.17726845	1
0.83431965	0.00854782	0.13058598	nonDEG	0.13602528	0.72669105	1
0.49984864	0.0438463	0.37360097	nonDEG	0.49202834	0.17186392	1
-0.6174046	0.37826991	0.93547445	nonDEG	-0.1798463	0.85682327	1
-0.8303141	0.01648118	0.20149067	nonDEG	-0.5714072	0.25889892	1
-0.4015835	0.11918348	0.63063362	nonDEG	0.21042906	0.64456319	1
0.52067492	0.05078462	0.40746621	nonDEG	0.40352674	0.2688032	1
-0.0100568	1	1	nonDEG	1.22007331	0.30956955	1
-0.4994483	0.3034493	0.88814105	nonDEG	-0.8197294	0.19083245	1
-0.4374994	0.10989654	0.60562414	nonDEG	-0.3427031	0.38194194	1
-0.9536511	0.14581547	0.68029504	nonDEG	-2.0797422	0.03007654	1
-0.4560948	0.18818978	0.75366341	nonDEG	-0.9018665	0.06603603	1
0.47278651	0.06779505	0.47541505	nonDEG	0.27188689	0.45986297	1
1.0898912	0.00087129	0.0241525	DEG	0.11482953	0.78566813	1
1.40782399	5.39E-08	8.13E-06	DEG	0.51132729	0.31683011	1
-1.3125068	0.07205014	0.49007934	nonDEG	-0.3475628	0.69603887	1
-0.9618526	0.01720908	0.20771547	nonDEG	-0.4497242	0.382412	1
-0.2630547	0.40914845	0.95823562	nonDEG	-0.1343072	0.76275108	1
-0.42448	0.54952637	1	nonDEG	-0.5853125	0.50602588	1
0.78538646	0.13560449	0.66247272	nonDEG	0.42628636	0.45679515	1
0.20422324	0.64562101	1	nonDEG	1.14860775	0.00403616	0.52015192
-0.3597987	0.17112705	0.72989601	nonDEG	-0.1799514	0.62473303	1
-0.5424823	0.02413153	0.25949182	nonDEG	-0.2242282	0.56505157	1
-0.6799311	0.02910688	0.29304974	nonDEG	-0.2178753	0.62647733	1
0.65074672	0.08237449	0.52495927	nonDEG	0.6273144	0.10535334	1
-0.7042374	0.04237329	0.36576454	nonDEG	-0.76522	0.16539477	1
-1.0521271	7.14E-05	0.00335559	DEG	-0.0800604	0.86721663	1
-0.8002945	0.00423092	0.07918248	nonDEG	-0.1940732	0.67416047	1
-1.0305591	0.00177024	0.04189245	DEG	0.0730533	0.86772732	1
-0.0847422	0.87534969	1	nonDEG	0.88656581	0.08995354	1
-0.6929167	0.00747401	0.11867704	nonDEG	0.0640612	0.88734466	1
0.4184104	0.70128435	1	nonDEG	1.24877147	0.26443344	1
-0.5130882	0.08755902	0.54054722	nonDEG	-0.1003783	0.81195554	1
0.29706211	0.59827126	1	nonDEG	1.06042475	0.05491703	1

0.59022913	0.29041485	0.87282735	nonDEG	0.29029408	0.63440881	1
-0.8894907	0.0082773	0.12724564	nonDEG	-0.2046045	0.65195829	1
-0.2780422	0.27136793	0.85427653	nonDEG	0.13874117	0.72182133	1
0.86571614	0.02379604	0.25768009	nonDEG	0.40333746	0.41874442	1
-0.0963477	0.77040976	1	nonDEG	0.78122735	0.05327103	1
-0.4682685	0.0833413	0.52831277	nonDEG	-0.590327	0.12652403	1
-0.4954792	0.71076661	1	nonDEG	-1.2337472	0.47661207	1
1.25190566	7.66E-06	0.00056642	DEG	-0.6242823	0.12101016	1
-0.5804972	0.0437135	0.37304382	nonDEG	-0.2830114	0.47729509	1
0.20918621	0.62676366	1	nonDEG	0.42569174	0.37922263	1
-1.0109817	0.00142162	0.03525775	DEG	-0.132192	0.75897198	1
-0.7691693	0.00590418	0.09982435	nonDEG	-0.2825067	0.45523167	1
-0.7461162	0.0024948	0.05384491	nonDEG	-0.0513933	0.91095914	1
0.7970863	0.00974003	0.1432251	nonDEG	0.24190644	0.57485662	1
-0.7376334	0.02410594	0.25947156	nonDEG	0.01290171	0.97673513	1
0.47270633	0.05690969	0.43418247	nonDEG	0.35732954	0.34335171	1
-0.7517034	0.00254527	0.05452677	nonDEG	0.10159913	0.82062556	1
0.38479601	0.30001052	0.88467981	nonDEG	-0.1821585	0.69118436	1
-2.500137	0.00516692	0.09037966	nonDEG	-3.2785538	0.03001584	1
-0.9133053	0.01259853	0.1698359	nonDEG	-0.2981454	0.50168075	1
-1.0025102	0.00357233	0.07072347	nonDEG	-0.4844571	0.38451769	1
1.52817209	0.00056402	0.01749657	DEG	0.34477477	0.48353882	1
0.11145951	0.68674701	1	nonDEG	-0.1097427	0.77338071	1
0.59553825	0.04706053	0.39043916	nonDEG	0.46613802	0.20305725	1
-0.5206583	0.03906381	0.34897353	nonDEG	-0.3094896	0.41352485	1
-0.1336892	0.72471705	1	nonDEG	-2.2864013	7.46E-05	0.03852026
-0.1054155	0.91846116	1	nonDEG	1.6470514	0.07232611	1
-0.9245997	0.00615614	0.10303043	nonDEG	-1.1449027	0.05342885	1
-0.4991778	0.05422827	0.42151037	nonDEG	-0.1838195	0.66058253	1
0.87057451	0.00065906	0.01977799	DEG	0.34799773	0.36119244	1
-1.1387382	0.01868497	0.21915009	nonDEG	-0.2249247	0.75722958	1
-0.8412203	0.00988399	0.14474983	nonDEG	-0.8286137	0.08539648	1
-0.6678284	0.01268357	0.17060133	nonDEG	-0.5574627	0.15307495	1
1.47403685	1.25E-06	0.00012018	DEG	-0.0855875	0.82716746	1
-0.6308285	0.02370847	0.25726263	nonDEG	-0.0969249	0.81139953	1
-0.1275215	0.7174432	1	nonDEG	-0.3997214	0.36856449	1
0.61766468	0.13455378	0.66012324	nonDEG	0.02934957	0.9518675	1
0.53873662	0.23978866	0.82168996	nonDEG	0.16125127	0.78161083	1
0.86403278	0.00068891	0.02036011	DEG	0.04737567	0.89591236	1
0.93495108	0.01104763	0.15718248	nonDEG	0.08073008	0.84773798	1
-0.7304268	0.11466784	0.61890549	nonDEG	-1.7110945	0.00435943	0.52515749
-1.0873487	0.10652878	0.59784686	nonDEG	-2.5746021	0.00947935	0.74892342
0.10998669	0.90602146	1	nonDEG	2.20652166	0.01204237	0.81648099

-1.4451914	0.01519098	0.19185071	nonDEG	0.57292105	0.4325695	1
-0.6246945	0.30715622	0.89113747	nonDEG	-0.2837695	0.70113398	1
1.41365251	8.96E-09	1.78E-06	DEG	0.26337246	0.48939155	1
0.44198604	0.27097917	0.85406365	nonDEG	-0.8646867	0.11728806	1
1.00268547	0.02756962	0.28268426	nonDEG	0.51545847	0.29165943	1
-1.8366424	7.48E-06	0.00055908	DEG	-0.1756079	0.8138802	1
0.68768297	0.24057226	0.82231344	nonDEG	0.74017068	0.27792961	1
-2.045368	1.26E-05	0.0008419	DEG	-1.1564482	0.14736379	1
-1.0414009	0.02993662	0.29766155	nonDEG	-0.4936373	0.52771514	1
-0.4309945	0.76106346	1	nonDEG	-6.0964452	0.01498071	0.8820345
-0.3235905	0.40740749	0.95694175	nonDEG	-1.7661589	0.00108974	0.2307973
0.68560566	0.00759661	0.12023472	nonDEG	0.01621129	0.96551427	1
-0.9101363	0.02823864	0.28765305	nonDEG	-0.0148777	0.98429692	1
1.91956997	0.00121144	0.03133888	DEG	0.71568854	0.27454473	1
0.0275242	0.93287829	1	nonDEG	-1.4599994	0.00132009	0.26570141
-0.8587694	0.00068427	0.0202791	DEG	-0.2602709	0.548633	1
-2.3907181	5.36E-07	5.62E-05	DEG	-0.8991538	0.20080065	1
-0.6580113	0.06492429	0.4657179	nonDEG	-0.6950021	0.29780165	1
0.73962838	0.07749797	0.51021897	nonDEG	0.35196696	0.44978876	1
1.05859993	2.74E-05	0.00157649	DEG	0.15612336	0.67441542	1
-0.8775247	0.0053309	0.09263475	nonDEG	-0.1808767	0.67644434	1
1.02734966	0.00015198	0.00612069	DEG	0.11551014	0.76406458	1
-0.3269393	0.62517068	1	nonDEG	1.36400823	0.01099076	0.79590774
-0.9743152	0.00091858	0.02513201	DEG	-0.7618223	0.09273268	1
0.65096075	0.02275769	0.24961389	nonDEG	0.90709051	0.01412911	0.8605661
0.5768264	0.33716327	0.91869633	nonDEG	0.69297784	0.2425407	1
1.32549764	2.52E-07	2.99E-05	DEG	-0.1160938	0.76041402	1
0.70507505	0.00644079	0.1060461	nonDEG	0.24359263	0.52910377	1
0.9043453	0.05351278	0.41909088	nonDEG	0.43575259	0.43127697	1
0.66506082	0.3839005	0.94047157	nonDEG	0.92561981	0.30154964	1
0.95363132	0.00081118	0.02280822	DEG	0.80081087	0.16729144	1
-0.3961091	0.11268336	0.61354292	nonDEG	-0.4105148	0.29108973	1
-0.7092526	0.03520252	0.32962053	nonDEG	-0.1654736	0.73048263	1
0.48339752	0.07973935	0.51751469	nonDEG	0.26428279	0.51295798	1
-1.5717405	0.00514667	0.09008894	nonDEG	-1.2737071	0.11793659	1
0.45998953	0.16548876	0.7181157	nonDEG	0.87739196	0.1479667	1
-1.0352632	0.00434323	0.08092432	nonDEG	-0.6290851	0.22958148	1
-1.1327566	8.17E-06	0.00059117	DEG	-0.3238162	0.55261722	1
-0.9096699	0.00055099	0.01721917	DEG	-0.2798396	0.4494143	1
-1.1291668	0.00015172	0.0061183	DEG	-1.1106796	0.03566494	1
1.75118026	2.93E-10	9.17E-08	DEG	-0.2027853	0.60566633	1
-0.5943405	0.05193815	0.41268748	nonDEG	-0.1594229	0.73269775	1
0.27779265	0.35824638	0.92121962	nonDEG	0.44089981	0.42075103	1

-0.5593284	0.02534572	0.26753416	nonDEG		0.23102669	0.72609474	1
0.58212547	0.04347821	0.37153991	nonDEG		0.34892443	0.42123851	1
1.14819722	0.20905451	0.78572136	nonDEG		0.93913359	0.24391886	1
2.32784835	3.09E-06	0.00025929	DEG		-0.8889038	0.09929612	1
0.37774569	0.12937123	0.65257114	nonDEG		0.10901723	0.76526918	1
0.33970745	0.33102183	0.91448941	nonDEG		0.65697656	0.12071266	1
-0.4076984	0.58017116	1	nonDEG		1.23776645	0.07422296	1
0.88742714	0.01687033	0.20476882	nonDEG		0.89261084	0.05346006	1
0.08632616	0.72671297	1	nonDEG		0.39556337	0.27467713	1
0.69906038	0.05731071	0.43573609	nonDEG		-0.8588989	0.06512143	1
-0.7555123	0.01225714	0.16790974	nonDEG		-0.045496	0.90784698	1
-1.2327882	8.13E-06	0.00059045	DEG		-0.2008009	0.60893574	1
-1.6261435	5.52E-06	0.00043354	DEG		-1.8738781	0.00645996	0.63391624
0.30508753	0.25622815	0.83840175	nonDEG		0.5882619	0.10901728	1
1.32985107	0.08754592	0.54054722	nonDEG		0.48171243	0.58479597	1
-0.6880015	0.06110119	0.45160884	nonDEG		-1.2562024	0.02286102	1
-1.0614645	0.00266616	0.05634545	nonDEG		-0.4850827	0.43921332	1
0.82142904	0.14818531	0.68430231	nonDEG		0.25923828	0.69947154	1
-0.4233563	0.11407751	0.61699942	nonDEG		-0.5316494	0.1976362	1
-1.1213504	0.00824789	0.12704683	nonDEG		-1.7193714	0.01235713	0.81648099
-1.238915	0.00041659	0.01400009	DEG		-0.1914458	0.75892862	1
-0.3618439	0.23902196	0.82089086	nonDEG		-0.1062577	0.83170803	1
1.93061742	1.23E-07	1.65E-05	DEG		-0.1157226	0.80975363	1
0.225443	0.41698492	0.96211451	nonDEG		0.53533267	0.17633542	1
0.67358325	0.08363897	0.52875977	nonDEG		0.18169805	0.66558522	1
0.51976756	0.06858719	0.47869686	nonDEG		0.42540199	0.24651262	1
-3.2963965	1.11E-05	0.00076048	DEG		-1.0182276	0.41313677	1
-1.427454	0.21119315	0.7877265	nonDEG		-4.5917075	0.13568971	1
-0.5163822	0.03852996	0.34624038	nonDEG		-0.7618191	0.0338134	1
-0.4432855	0.53090886	1	nonDEG		1.40004612	0.02397858	1
0.47805704	0.09821473	0.57137375	nonDEG		0.29568143	0.4194926	1
-0.6120578	0.02537013	0.26766026	nonDEG		-1.3542972	0.00076537	0.18621308
0.268688	0.27136303	0.85427653	nonDEG		0.14896243	0.74503843	1
-0.8319485	0.00617919	0.10333109	nonDEG		0.05804513	0.88639759	1
0.89401643	0.00298722	0.06171275	nonDEG		0.12994478	0.74609957	1
2.94302	1.61E-10	5.59E-08	DEG		-0.1932653	0.72126078	1
0.55562385	0.05099002	0.4084663	nonDEG		0.77904704	0.03713427	1
-1.3693598	0.03252448	0.31312418	nonDEG		-0.1298307	0.88304434	1
1.99214188	3.91E-07	4.25E-05	DEG		-0.3423658	0.44864091	1
-0.2390437	0.38016554	0.93639703	nonDEG		-0.2990787	0.48777489	1
-0.6070153	0.06472522	0.465424	nonDEG		-0.6928609	0.14938972	1
-2.2834162	2.04E-13	1.36E-10	DEG		-0.2805835	0.74034493	1
0.5841296	0.05527295	0.42663353	nonDEG		-0.0057424	0.98903035	1

0.42949772	0.13059117	0.65446487	nonDEG	0.6387058	0.08449238	1
-0.2481518	0.33275858	0.9165494	nonDEG	-0.3532516	0.35944436	1
-0.6870694	0.04088844	0.35894269	nonDEG	-0.4712409	0.28047647	1
-0.613907	0.04699698	0.3901947	nonDEG	-0.2929486	0.61237683	1
-2.1084428	0.00990362	0.14483498	nonDEG	-0.4337224	0.6617734	1
0.94884676	0.00241909	0.05248027	nonDEG	-0.1334706	0.72614524	1
3.52417282	2.54E-22	7.24E-19	DEG	0.35096932	0.49779493	1
0.53009186	0.03253711	0.31312848	nonDEG	0.40622612	0.29611878	1
0.31391948	0.26155826	0.84531933	nonDEG	0.46101874	0.24947233	1
0.95396071	0.00256728	0.05492505	nonDEG	0.30989732	0.43870619	1
-0.3563252	0.36343781	0.92416624	nonDEG	-0.2037517	0.73724397	1
-0.5308216	0.05274994	0.41644019	nonDEG	-0.6660781	0.15591384	1
-0.2492515	0.56799272	1	nonDEG	-1.3779021	0.02110389	1
0.55928586	0.02920405	0.29372666	nonDEG	0.36246572	0.34010226	1
2.24792155	0.02613033	0.27364824	nonDEG	0.89249066	0.41922508	1
-0.5886763	0.01607163	0.19887881	nonDEG	-0.0896065	0.82559621	1
0.0102107	0.97491379	1	nonDEG	-0.7294616	0.05660488	1
0.20553321	0.48679989	0.99733295	nonDEG	0.34826845	0.35464116	1
1.08142568	0.00038502	0.01305846	DEG	0.40598343	0.28085945	1
0.92723651	0.00783392	0.12290774	nonDEG	0.02730778	0.94889655	1
0.10900268	0.72284804	1	nonDEG	-0.8010575	0.04896074	1
-1.8826181	2.31E-11	1.00E-08	DEG	0.52906011	0.21412085	1
0.6587039	0.1739713	0.73500878	nonDEG	0.02297031	0.96847494	1
-1.6656143	0.00071675	0.02093302	DEG	-0.2136697	0.76575846	1
0.81954377	0.00443336	0.08187808	nonDEG	0.43952037	0.24375733	1
-1.3271282	0.0202459	0.23077141	nonDEG	0.11514134	0.87607252	1
1.57282686	0.00494868	0.08792926	nonDEG	0.65387874	0.2536289	1
-1.3054725	0.0072298	0.11567285	nonDEG	-2.4148655	0.00098102	0.21777569
0.77357368	0.4463963	0.98124647	nonDEG	1.52730411	0.09851719	1
-1.1505772	4.23E-05	0.00222506	DEG	-0.2228589	0.73868613	1
1.36210593	3.46E-07	3.86E-05	DEG	0.72060407	0.14759064	1
-1.5690356	0.20403149	0.77765318	nonDEG	-5.2825162	0.08312139	1
-0.7519613	0.04209888	0.36479284	nonDEG	-0.8357867	0.11253879	1
1.45636495	0.01709982	0.2068102	nonDEG	0.16038597	0.79895752	1
0.96381259	0.0028206	0.05893013	nonDEG	0.47750298	0.24833477	1
0.44525106	0.08751609	0.54054722	nonDEG	0.59589655	0.14533603	1
1.065082	0.04304892	0.36926489	nonDEG	-0.0114139	0.99067439	1
-0.2384789	0.44862975	0.98329655	nonDEG	-0.5869097	0.16983143	1
-0.87235	0.00045951	0.01508089	DEG	-0.1703275	0.70084295	1
0.67282775	0.02203328	0.24440004	nonDEG	0.46077441	0.21808506	1
-0.6077115	0.01445615	0.18575932	nonDEG	-0.3104475	0.40292276	1
-0.4373595	0.1704784	0.72882858	nonDEG	-0.2786259	0.50599928	1
-1.3297869	1.95E-07	2.45E-05	DEG	-0.2898305	0.52345027	1

-0.7469277	0.25977263	0.8419516	nonDEG	1.33979748	0.03231813	1
0.62047054	0.19456671	0.76452875	nonDEG	-0.0830403	0.88549544	1
-0.5870961	0.31877965	0.90147301	nonDEG	-0.6959194	0.44243753	1
1.4650665	2.40E-08	4.10E-06	DEG	0.30531938	0.41767192	1
-0.7754872	0.00226234	0.05000073	nonDEG	-0.2528168	0.48977027	1
-1.6642881	0.003577	0.07076833	nonDEG	-0.5192056	0.49175989	1
-0.4024852	0.1088137	0.60347345	nonDEG	-0.4046603	0.27759444	1
0.42490014	0.10440759	0.59145604	nonDEG	0.47384996	0.20834411	1
1.64080539	5.01E-06	0.0004015	DEG	0.35513965	0.42511186	1
-0.8414104	0.00563023	0.0965336	nonDEG	-0.2215447	0.62167431	1
1.43278391	0.00291405	0.06049834	nonDEG	-0.1737359	0.739762	1
-1.9681862	0.11165986	0.61073012	nonDEG	-5.034595	0.10433265	1
-1.9012363	0.00615565	0.10303043	nonDEG	-0.5238644	0.55822825	1
0.61113554	0.11488853	0.61909498	nonDEG	0.40829033	0.33905716	1
-0.7675356	0.17603412	0.74032405	nonDEG	-1.4699009	0.06210039	1
0.14699959	0.73130692	1	nonDEG	0.62086929	0.22606938	1
0.065517	0.9294152	1	nonDEG	1.10878456	0.15936164	1
0.01050354	1	1	nonDEG	1.0529726	0.19303814	1
0.25040113	0.42178933	0.964132	nonDEG	0.75444264	0.04813497	1
1.08689076	1.50E-05	0.00097627	DEG	0.2553781	0.47785496	1
-0.4287557	0.14403963	0.67658948	nonDEG	-0.1278983	0.77365297	1
-1.4916978	0.00067192	0.01998038	DEG	-0.803667	0.24267225	1
-0.6436162	0.23362337	0.81386984	nonDEG	0.16197441	0.86180772	1
-0.1334739	0.60172475	1	nonDEG	-0.6430947	0.07680586	1
1.14816637	0.01788936	0.21330292	nonDEG	0.03502535	0.95595477	1
-0.6634151	0.08839987	0.54352626	nonDEG	-0.0329278	0.96239409	1
-1.0930481	0.01342007	0.17652964	nonDEG	-0.08612	0.86295067	1
-0.3444815	0.16207738	0.713	nonDEG	-0.4393378	0.26357108	1
-1.5902296	0.0651153	0.46674684	nonDEG	-1.036896	0.44187429	1
0.23951831	0.3724518	0.93135861	nonDEG	-1.7104323	1.42E-05	0.01127238
-0.2826954	0.43735679	0.97423703	nonDEG	-0.4981579	0.32402795	1
-1.4387208	0.00931884	0.13855428	nonDEG	-2.1730693	0.00153131	0.28897678
0.77045425	0.01650443	0.20152774	nonDEG	0.1368617	0.7255427	1
-1.1604555	0.00361555	0.07138705	nonDEG	-0.3454992	0.53565532	1
-0.7084949	0.04404713	0.3747137	nonDEG	-0.4872223	0.35737147	1
3.46468437	6.02E-25	2.95E-21	DEG	-0.4336006	0.46823961	1
0.55301271	0.11471936	0.61899255	nonDEG	0.37884586	0.36573853	1
-2.5235966	2.70E-08	4.52E-06	DEG	-2.8109447	0.00013828	0.05501207
-1.1864203	9.38E-05	0.00419506	DEG	-1.2690502	0.01643829	0.920013
-1.9426515	2.57E-05	0.00149559	DEG	-0.1411798	0.82854735	1
0.6881892	0.18356435	0.75101469	nonDEG	0.0556314	0.92642952	1
-0.9314002	0.03141123	0.30680662	nonDEG	-1.4292131	0.01902624	0.97073413
0.50481445	0.34414395	0.91869633	nonDEG	0.60554536	0.30283417	1

0.28316083	0.36304498	0.92393697	nonDEG		0.45989442	0.29550125	1
1.03180175	0.00020576	0.00785635	DEG		-0.1464995	0.69250213	1
0.31022509	0.21216116	0.78960966	nonDEG		0.46538961	0.26213917	1
-0.2240866	1	1	nonDEG		2.15739302	0.0650867	1
0.58746382	0.03411618	0.32289859	nonDEG		0.28176881	0.45339877	1
1.42172749	0.0002099	0.00800069	DEG		-0.0159914	0.96937989	1
-0.292738	0.26742216	0.85123186	nonDEG		0.1227236	0.77856931	1
-0.2355072	0.45989458	0.98479636	nonDEG		-0.8513697	0.34116774	1
-2.9428366	0.00594313	0.10026358	nonDEG		-2.7369332	0.08675709	1
2.09870767	2.13E-10	7.12E-08	DEG		-0.5451203	0.23847611	1
-0.7006332	0.24394566	0.82612635	nonDEG		-0.8820186	0.28173848	1
1.12154848	5.65E-05	0.0027882	DEG		0.20640386	0.56640268	1
1.66430532	0.02742201	0.28196873	nonDEG		0.20185	0.82716588	1
0.76407248	0.00697765	0.11261796	nonDEG		0.22275373	0.60278252	1
-0.7560016	0.00185999	0.04342293	DEG		0.18299441	0.6981816	1
0.52891483	0.19854148	0.77075879	nonDEG		0.45485005	0.3991005	1
0.40764245	0.47987608	0.99343606	nonDEG		0.41462155	0.51734412	1
0.89096696	0.05931012	0.44405663	nonDEG		-0.0155063	0.98419403	1
0.87425178	0.04204964	0.3647571	nonDEG		0.34919504	0.42850907	1
-0.7053527	0.25635504	0.83846016	nonDEG		1.67822978	0.01261606	0.82534259
1.01762363	0.20945541	0.78572136	nonDEG		0.58639675	0.50824334	1
0.45623069	0.09127411	0.55084432	nonDEG		0.42727153	0.23804701	1
-0.6333548	0.06041756	0.44807419	nonDEG		-0.0833971	0.84964364	1
-2.7146494	0.00917138	0.1374727	nonDEG		-0.3753086	0.82498256	1
0.83107613	0.17124762	0.72989601	nonDEG		0.44637857	0.54810143	1
0.49085134	0.08122125	0.52184628	nonDEG		0.48362503	0.20994286	1
1.82046488	0.0003731	0.01279718	DEG		-1.47768	0.00945098	0.74892342
0.5078398	0.26828487	0.85190792	nonDEG		0.54047071	0.30155706	1
6.08434575	0.0252411	0.26681248	nonDEG		-0.2216829	0.9438222	1
1.42745808	0.00146008	0.03609009	DEG		0.45844743	0.3343092	1
0.66812144	0.06570802	0.46859939	nonDEG		0.08427332	0.84140466	1
0.01494725	0.96996525	1	nonDEG		-0.7601244	0.12135706	1
0.53042958	0.52247161	1	nonDEG		1.34857831	0.19318596	1
0.34733328	0.16350528	0.714154	nonDEG		0.49374523	0.23161384	1
0.70409337	0.03064721	0.30204997	nonDEG		0.34696721	0.38885966	1
0.92301822	0.00069446	0.02048517	DEG		0.8637121	0.06991439	1
3.09742707	4.37E-05	0.00229097	DEG		-0.0864249	0.91738665	1
0.46511685	0.07341633	0.4946906	nonDEG		0.43594223	0.23602969	1
-0.4803586	0.06136289	0.4527474	nonDEG		-0.5115863	0.16061284	1
0.87246685	0.0011647	0.03028939	DEG		0.28898494	0.43091364	1
-0.6529615	0.04843666	0.39666692	nonDEG		-1.4467395	0.00546744	0.58529482
-1.1143222	0.0531793	0.41796662	nonDEG		-0.5347475	0.46839682	1
-1.1009439	0.08157673	0.52300761	nonDEG		-3.7066422	0.00049627	0.14184283

1.30013329	0.04076279	0.35845587	nonDEG		0.51187416	0.41134965	1
7.10332049	0.00053493	0.01684766	DEG		-0.3870794	0.78535496	1
0.57699226	0.09019414	0.54766713	nonDEG		0.22853493	0.59601695	1
0.12147943	0.77364313	1	nonDEG		0.64669294	0.14194692	1
-7.8588899	3.36E-05	0.00187215	DEG		5.34007113	0.0741007	1
-0.5184087	0.04224283	0.3651165	nonDEG		-0.5518576	0.13712061	1
-0.9159391	0.00052304	0.0165567	DEG		0.00273879	0.9957966	1
-0.9108106	0.01057544	0.15255616	nonDEG		-0.2297949	0.6467192	1
0.00236743	0.99845971	1	nonDEG		1.52377789	0.00247864	0.38813146
1.51805169	0.00048457	0.01559056	DEG		0.66113239	0.16866286	1
0.92168638	0.00096068	0.02599404	DEG		0.0316335	0.93454168	1
1.03046252	0.00526015	0.09173787	nonDEG		0.01080639	0.98371238	1
-0.659692	0.0780382	0.511549	nonDEG		-0.7096664	0.16772977	1
-0.9481188	0.00031525	0.01120851	DEG		0.20688357	0.67629601	1
4.55472346	1.64E-25	9.67E-22	DEG		0.21964438	0.7931172	1
-1.0735473	0.00013214	0.00554144	DEG		-0.1716314	0.65503002	1
-1.6101385	1.14E-09	2.88E-07	DEG		-0.9295433	0.14259951	1
1.11420331	2.52E-05	0.00148	DEG		-0.007397	0.98485745	1
1.13663155	5.98E-05	0.00290866	DEG		0.32627911	0.39820875	1
-0.1155559	0.70693713	1	nonDEG		0.60544678	0.11692285	1
-0.830205	0.06391925	0.46254672	nonDEG		-1.4772083	0.02487487	1
2.24902916	7.08E-06	0.00053714	DEG		-0.1662072	0.80585873	1
0.94153559	0.00771018	0.12163981	nonDEG		0.48809273	0.28512546	1
0.43066236	0.14017298	0.66891756	nonDEG		0.3164731	0.39269579	1
-0.3048211	0.32990085	0.91380796	nonDEG		-0.7883618	0.04559858	1
1.68761079	2.55E-08	4.34E-06	DEG		-0.1430204	0.7036815	1
0.87667978	0.00137222	0.03426351	DEG		0.65260365	0.13061224	1
-1.9738042	2.72E-09	6.25E-07	DEG		0.95253906	0.28530108	1
1.12320283	0.59608453	1	nonDEG		1.98260122	0.13436991	1
-0.5325628	0.10655785	0.59784686	nonDEG		-0.2966017	0.51338571	1
-2.0291451	0.06038121	0.44807419	nonDEG		-1.5247115	0.34512919	1
0.77589328	0.00788684	0.12350043	nonDEG		0.09137775	0.81005856	1
-0.8589892	0.01502106	0.19068781	nonDEG		-0.4093012	0.41502009	1
-0.6101939	0.06107656	0.45160884	nonDEG		0.19998841	0.73391381	1
0.66076618	0.13915439	0.66741056	nonDEG		0.43946943	0.34952331	1
1.79362128	1.63E-08	2.99E-06	DEG		0.56900328	0.14482836	1
0.51431451	0.10984822	0.60547121	nonDEG		0.05964194	0.9033649	1
1.04678185	0.00220022	0.04910636	DEG		1.19067119	0.00582676	0.59976888
-0.9411415	0.01118925	0.15813746	nonDEG		0.05986241	0.92430249	1
-1.8323858	0.16148539	0.71155624	nonDEG		0.35568002	0.87209863	1
1.06739856	0.00066921	0.01993081	DEG		0.26374657	0.4827131	1
0.13000336	0.73867161	1	nonDEG		0.33320783	0.52017121	1
-0.0879846	0.91076653	1	nonDEG		1.83881857	0.01684135	0.93512081

0.57619064	0.07488793	0.50162131	nonDEG		0.05082806	0.90424496	1
0.57498023	0.11279474	0.61366924	nonDEG		-0.1247821	0.7771356	1
0.26209619	0.42751075	0.96930753	nonDEG		0.50970594	0.25001682	1
0.60869146	0.05488113	0.42470945	nonDEG		-0.7310314	0.08249878	1
1.20255311	0.00269963	0.05688933	nonDEG		0.49017402	0.26471233	1
1.00979573	0.24561583	0.82672898	nonDEG		0.17486921	0.84190512	1
0.59788095	0.02660025	0.27631445	nonDEG		0.36510537	0.31481677	1
0.94743607	0.00035511	0.01232797	DEG		-0.2889486	0.52927022	1
0.44964369	0.38050844	0.93668386	nonDEG		1.07569036	0.03576466	1
-0.1652865	0.78010516	1	nonDEG		0.7342033	0.16141529	1
2.16427709	0.0004122	0.0138684	DEG		-0.3635984	0.70838319	1
-0.5477219	0.02900474	0.29222126	nonDEG		-0.3431589	0.3813187	1
0.4969784	0.11211497	0.61181363	nonDEG		0.48184061	0.19420749	1
-0.7397563	0.23449132	0.81531276	nonDEG		1.26551602	0.01255068	0.82473115
1.12913978	0.019571	0.22611878	nonDEG		0.47750224	0.32685547	1
-0.9634028	0.0001195	0.00512059	DEG		-0.0469693	0.91955758	1
0.93728189	0.0138625	0.18057446	nonDEG		0.17286553	0.69396276	1
-0.3401478	0.3651093	0.92544853	nonDEG		-0.8735236	0.20539877	1
1.06866197	0.00610492	0.1023478	nonDEG		0.42691923	0.30563171	1
-0.7816817	0.00546793	0.09444141	nonDEG		-0.8563489	0.03690654	1
0.29237779	0.24359916	0.8256221	nonDEG		0.69980051	0.06194455	1
0.54738357	0.03888779	0.34828645	nonDEG		0.03224975	0.93150856	1
-0.0970502	0.87954101	1	nonDEG		1.23343107	0.03606042	1
0.33148625	0.2223348	0.80228334	nonDEG		0.62111373	0.14220213	1
-1.0989042	0.02316676	0.25250135	nonDEG		0.17870559	0.77175918	1
-0.9386402	0.00597456	0.10073598	nonDEG		-0.6060739	0.19878226	1
-0.231546	0.72817873	1	nonDEG		0.64904343	0.30751527	1
0.36532179	0.39972498	0.95204649	nonDEG		0.80025112	0.08215163	1
-1.3933084	9.65E-06	0.00068645	DEG		-0.0102207	0.98291715	1
0.58019808	0.16314149	0.714154	nonDEG		1.59814303	0.00089309	0.20702029
-0.0907658	0.71500514	1	nonDEG		-0.2495752	0.55748487	1
-0.9175886	0.01336172	0.17615565	nonDEG		-0.9024098	0.22019793	1
-0.9521442	0.20597901	0.7803135	nonDEG		-0.361046	0.70247863	1
0.20170067	0.56138398	1	nonDEG		0.70242295	0.08562204	1
0.85973163	0.26127301	0.84479608	nonDEG		0.35663869	0.63463892	1
0.58837034	0.08122218	0.52184628	nonDEG		0.25551367	0.53498954	1
-0.7802566	0.0030575	0.0628121	nonDEG		-0.0931143	0.83010407	1
2.03744914	0.00477086	0.08569202	nonDEG		-0.038046	0.9642864	1
1.10172321	1.45E-05	0.00095637	DEG		0.1904514	0.62410656	1
-0.3775499	0.1522234	0.69070664	nonDEG		0.01924724	0.96155886	1
0.66167467	0.05944438	0.4447225	nonDEG		0.55041148	0.16629985	1
-0.7212982	0.01702781	0.20611915	nonDEG		-0.5103759	0.30146413	1
-1.1583682	7.53E-06	0.00056056	DEG		0.01846253	0.96626587	1

-0.5199077	0.03923723	0.35003173	nonDEG	-0.0383718	0.92243651	1
-0.2775268	0.26729363	0.85114736	nonDEG	0.3003304	0.49716565	1
0.61072131	0.06880764	0.4796657	nonDEG	0.39851444	0.33472232	1
-0.2781838	0.65173978	1	nonDEG	-0.4656385	0.59604891	1
-0.5705343	0.12928724	0.65245846	nonDEG	-0.003908	0.99616777	1
0.8378572	0.05485254	0.42461315	nonDEG	0.41354714	0.41076551	1
-0.3717725	0.24315974	0.82526859	nonDEG	-0.1227169	0.77970493	1
-0.0239876	0.96375467	1	nonDEG	0.61337876	0.3051327	1
-0.5943267	0.07855847	0.51343859	nonDEG	0.42799832	0.31139942	1
1.47873245	2.23E-06	0.00019851	DEG	-0.223109	0.58308649	1
0.86908793	0.09474623	0.56132341	nonDEG	0.4433721	0.43221932	1
0.40125837	0.11905303	0.63063362	nonDEG	0.70192793	0.06490536	1
-0.4033817	0.12523382	0.64420034	nonDEG	-0.2157157	0.58330042	1
-0.349606	0.16432571	0.71511831	nonDEG	0.00116862	0.99794721	1
-0.5132506	0.20596556	0.7803135	nonDEG	-1.5732721	0.00558747	0.58916916
-0.9822882	0.00014164	0.00583162	DEG	-0.3847994	0.36440572	1
-1.311546	0.00457473	0.08344208	nonDEG	0.09335282	0.88733626	1
-0.0223421	0.97949657	1	nonDEG	1.36801267	0.00419266	0.52515749
-1.7702709	1.08E-05	0.00074577	DEG	-1.3983207	0.0589581	1
-0.5930406	0.40176833	0.95307326	nonDEG	1.80676291	0.00195972	0.34754367
-1.1063981	0.17918481	0.74495434	nonDEG	-0.4932799	0.60572183	1
0.80001705	0.00157017	0.03810725	DEG	0.47986308	0.1828198	1
-2.1559166	1.15E-07	1.56E-05	DEG	0.34455789	0.65440316	1
-1.5334969	0.11837855	0.62843136	nonDEG	-0.2164837	0.86213143	1
1.4640836	2.68E-05	0.00154917	DEG	0.33951326	0.42986746	1
-0.5513584	0.14432527	0.6772062	nonDEG	-0.8528624	0.07961499	1
0.50390839	0.04370052	0.37304382	nonDEG	0.25951343	0.50211292	1
0.7956702	0.01519824	0.19185071	nonDEG	0.44526679	0.27855005	1
0.85700621	0.00210229	0.04771722	DEG	0.24821828	0.50067829	1
0.42965765	0.57145503	1	nonDEG	1.07022099	0.18609293	1
1.318469	0.00188784	0.0438298	DEG	-0.0230554	0.96600305	1
0.4800923	0.05065629	0.4066732	nonDEG	0.17698694	0.62870407	1
-0.6031777	0.03227215	0.31210905	nonDEG	-0.3693864	0.33846165	1
1.96018126	0.03926603	0.3501826	nonDEG	0.43325608	0.62364706	1
0.4263501	0.25502495	0.83678997	nonDEG	0.72317987	0.08502461	1
-0.52873	0.04828797	0.39575435	nonDEG	-0.3936388	0.34428401	1
1.99951535	3.67E-13	2.40E-10	DEG	-0.0937741	0.84263464	1
0.57103311	0.19917441	0.77158465	nonDEG	-2.196793	0.00012276	0.05194852
1.26147817	0.01345862	0.17656877	nonDEG	0.58640669	0.2080749	1
0.7032504	0.00970656	0.14294713	nonDEG	0.48811162	0.17648776	1
-0.2542406	0.41486456	0.96053724	nonDEG	-0.2486179	0.56285953	1
-1.5114973	7.54E-06	0.00056056	DEG	-1.687065	0.0028789	0.4301506
1.06022842	2.18E-05	0.00133127	DEG	0.52830737	0.17934463	1

-0.5404422	0.07248544	0.49134215	nonDEG	-0.196203	0.62958228	1
1.58036496	0.00174315	0.04145118	DEG	0.51601246	0.35203502	1
-0.8800355	0.00135816	0.03402789	DEG	-0.1639871	0.66697776	1
0.59747849	0.01834139	0.21650053	nonDEG	0.55353898	0.15463309	1
1.66594808	3.36E-10	9.89E-08	DEG	-0.0659576	0.8604279	1
-0.5690369	0.16691473	0.72155696	nonDEG	-0.8255969	0.10651378	1
-0.7947878	0.33964778	0.91869633	nonDEG	-1.3816111	0.19966931	1
0.71026444	0.47253194	0.99012003	nonDEG	1.58908119	0.06807214	1
0.6061977	0.0136863	0.17875374	nonDEG	0.14850469	0.71044935	1
1.455976	3.57E-07	3.97E-05	DEG	0.67232902	0.25271892	1
0.42486237	0.24886732	0.82990493	nonDEG	0.26862611	0.56763099	1
-0.6591916	0.02538977	0.26771108	nonDEG	-0.1617022	0.69388636	1
1.91045767	0.00034736	0.01211612	DEG	0.20357883	0.69517963	1
-1.7499743	2.15E-08	3.77E-06	DEG	0.47239444	0.55448624	1
-0.8182978	0.01821355	0.21559654	nonDEG	-0.500287	0.34369356	1
-0.8723693	0.04031226	0.35629734	nonDEG	0.04176016	0.94434469	1
-0.1803615	0.67636858	1	nonDEG	-1.1078892	0.03681167	1
0.3248093	0.57620633	1	nonDEG	1.01192429	0.08379389	1
0.42523844	0.22239642	0.80234415	nonDEG	0.18334861	0.68027865	1
-0.9013552	0.51387551	1	nonDEG	-2.5166643	0.24222689	1
0.23049322	0.34412594	0.91869633	nonDEG	0.23772999	0.58775446	1
-0.4280536	0.08277959	0.52633872	nonDEG	-0.2698962	0.50602113	1
1.45890001	1.61E-06	0.00014924	DEG	0.32725729	0.40145956	1
-0.6555436	0.02283178	0.24996092	nonDEG	-0.9055362	0.08496383	1
-0.9391046	0.0034953	0.06938507	nonDEG	-0.3171367	0.51990605	1
-0.6141829	0.02018127	0.23036691	nonDEG	-0.2945221	0.43159346	1
-1.7246374	2.87E-07	3.32E-05	DEG	-0.1338851	0.77494438	1
0.69917689	0.0115301	0.16150043	nonDEG	0.2807269	0.47639055	1
-0.8790421	0.0015588	0.03792519	DEG	-0.3394916	0.48975727	1
-1.3868185	0.00330041	0.06670357	nonDEG	-0.6891225	0.2930145	1
1.03914287	0.39141977	0.94686517	nonDEG	0.46908784	0.71422074	1
-0.3442197	0.25191124	0.83371668	nonDEG	-0.3594601	0.38161375	1
0.20005477	0.46367871	0.98535325	nonDEG	0.39973033	0.31912922	1
-0.4054375	0.15075361	0.68744653	nonDEG	-0.7721618	0.03908745	1
-1.0188631	0.00497298	0.08808644	nonDEG	-0.5002464	0.43079118	1
0.56606109	0.08528656	0.53493735	nonDEG	0.11332626	0.77896229	1
-2.0963422	0.00032433	0.0114345	DEG	-1.1373017	0.17637844	1
-0.9753007	0.0538472	0.42047949	nonDEG	-0.0015697	1	1
0.25992068	0.28571175	0.86801776	nonDEG	0.50047894	0.21380168	1
-0.5848705	0.02180863	0.24236465	nonDEG	-0.0991329	0.79261278	1
0.40826674	0.66998892	1	nonDEG	1.06518141	0.27354414	1
-0.4895626	0.25482921	0.83639093	nonDEG	-0.6428147	0.16406535	1
-1.7339808	0.00393205	0.07526366	nonDEG	0.73860505	0.35960129	1

-1.4167896	0.14949928	0.68626577	nonDEG	-3.3270057	0.02592158	1
1.64573067	3.12E-06	0.00026038	DEG	-0.0707746	0.86964897	1
1.01159316	0.00501528	0.08867551	nonDEG	-0.0337236	0.93920775	1
0.15032525	0.5444954	1	nonDEG	-0.1287168	0.77852588	1
-0.5052387	0.08952328	0.54666582	nonDEG	-0.3184858	0.44836917	1
1.19668062	0.0156584	0.19515982	nonDEG	1.62581337	0.01951262	0.98699646
0.60069401	0.38029698	0.93639703	nonDEG	0.60484252	0.37793153	1
0.42314871	0.63951398	1	nonDEG	1.18448851	0.22164218	1
0.27267613	0.73862251	1	nonDEG	1.52338268	0.02196751	1
0.83981644	0.00910301	0.13695041	nonDEG	0.43452161	0.26546471	1
0.52488791	0.04241693	0.36576454	nonDEG	0.13486685	0.73376798	1
-0.3703054	0.14951964	0.68626577	nonDEG	-0.0991718	0.82137285	1
0.65112961	0.01406176	0.1826044	nonDEG	0.17179322	0.64920495	1
-0.7856506	0.24090696	0.82264934	nonDEG	1.4688226	0.02555114	1
0.94414261	0.0006276	0.01904746	DEG	0.27299508	0.52704752	1
0.67059138	0.03870922	0.34753297	nonDEG	0.18084139	0.65785454	1
0.19205919	0.54315204	1	nonDEG	0.74761734	0.06685595	1
0.71685857	0.00828284	0.1272644	nonDEG	0.23060002	0.54523266	1
-0.4924952	0.11692576	0.62512815	nonDEG	-0.369178	0.46607409	1
-0.0455495	0.93550403	1	nonDEG	0.89264244	0.09741531	1
0.63040759	0.2231485	0.80348199	nonDEG	1.15906365	0.09169521	1
-1.0398152	0.00116103	0.03025458	DEG	-0.488604	0.33380911	1
-0.6654532	0.08037481	0.51953337	nonDEG	-0.3453251	0.48863315	1
-4.3832235	0.0004717	0.01531014	DEG	1.55455658	0.51556176	1
1.1393015	0.01975048	0.22747822	nonDEG	0.30966127	0.57246815	1
-0.7194516	0.02471218	0.2630517	nonDEG	-0.5292917	0.19492514	1
-1.140827	0.05751315	0.43635189	nonDEG	0.7014448	0.33585945	1
-1.1279415	0.00241452	0.05243102	nonDEG	-0.7072075	0.23837212	1
-2.009492	1.93E-12	1.01E-09	DEG	0.42623055	0.50917474	1
0.59348891	0.01962213	0.22662061	nonDEG	0.20338378	0.60504245	1
-1.0788234	0.00509991	0.08953675	nonDEG	-0.1794204	0.7711756	1
-0.8115269	0.00771831	0.12170283	nonDEG	-0.6973864	0.10902427	1
0.32069782	0.18219411	0.74848066	nonDEG	0.25496097	0.53010673	1
-0.2450656	0.64877121	1	nonDEG	-0.548284	0.43222947	1
-0.6819523	0.08896801	0.54533586	nonDEG	-1.2812474	0.01514537	0.88994942
-0.3685015	0.14332825	0.67575918	nonDEG	-0.0503238	0.89454952	1
-1.9351902	0.00106133	0.02818598	DEG	-1.2392522	0.17132552	1
-1.6261236	0.00185774	0.04340483	DEG	-0.1614497	0.83526196	1
-3.6721165	0.0007267	0.02114517	DEG	-5.023499	0.0833482	1
0.57228617	0.04413269	0.37517244	nonDEG	0.64211398	0.11581951	1
-0.6847345	0.16085525	0.71092698	nonDEG	0.9365689	0.02365685	1
0.54312169	0.09832465	0.57137375	nonDEG	0.2142447	0.61030644	1
-2.1749882	0.00038071	0.01300209	DEG	1.25223319	0.12607466	1

-0.8997118	0.00111451	0.02932075	DEG	0.00598505	0.98799168	1
-1.4513673	0.05177774	0.41196888	nonDEG	-0.1530376	0.90686915	1
1.00284523	0.00010038	0.00443023	DEG	0.19497686	0.6431553	1
0.58019436	0.02622256	0.27394112	nonDEG	0.5023735	0.15963578	1
1.75621139	0.00023366	0.0086832	DEG	1.17120002	0.07314737	1
0.5065951	0.32120637	0.90430629	nonDEG	0.46832018	0.40461165	1
0.44249793	0.11551336	0.62122724	nonDEG	0.35875637	0.33070011	1
-0.2760731	0.70016864	1	nonDEG	-0.2757143	0.77899394	1
-0.9930056	0.01629652	0.20014739	nonDEG	-1.1095423	0.03774576	1
1.25118913	2.36E-06	0.00020714	DEG	-0.1554781	0.67594379	1
-0.1361833	0.82736108	1	nonDEG	0.85407226	0.18349649	1
-0.2162182	0.67955502	1	nonDEG	-1.3218335	0.05595753	1
0.74334317	0.04918635	0.4007777	nonDEG	-0.2972378	0.5123061	1
1.71056132	0.03986756	0.35376105	nonDEG	0.26004412	0.72059544	1
0.78753364	0.27882166	0.86049314	nonDEG	0.90940291	0.2842126	1
-0.7573329	0.20292715	0.77486116	nonDEG	-1.5405087	0.12385981	1
-0.5503544	0.02792223	0.28551671	nonDEG	-0.1585443	0.66810764	1
0.19340152	0.50647425	1	nonDEG	0.43399393	0.25997623	1
-0.1983309	0.69637843	1	nonDEG	0.82004642	0.13925156	1
1.83026097	0.00047341	0.01534896	DEG	0.40044728	0.52291298	1
1.74401707	0.01002169	0.14612602	nonDEG	0.13392148	0.86661848	1
0.55470756	0.05161871	0.41117723	nonDEG	0.03722847	0.93162028	1
0.8464145	0.00085886	0.02387542	DEG	0.2355657	0.50835132	1
0.33316122	0.35406382	0.91917773	nonDEG	-1.0489812	0.04910509	1
0.41417923	0.15627713	0.70060637	nonDEG	0.42407825	0.26676922	1
0.65343827	0.05437302	0.42178847	nonDEG	0.21725919	0.59423295	1
-0.1638877	0.53280434	1	nonDEG	-0.3875231	0.3987103	1
-0.2641312	0.65677297	1	nonDEG	1.20540034	0.03720137	1
-1.1725432	0.00110935	0.02928982	DEG	-0.9067409	0.06410476	1
1.19642048	0.0001612	0.00643033	DEG	0.88537166	0.01829218	0.95785021
-0.7472224	0.01189576	0.16503271	nonDEG	-0.5940312	0.16335823	1
1.30505619	0.00034889	0.01215487	DEG	0.68769163	0.25507882	1
-0.6494179	0.06527023	0.4669909	nonDEG	-1.5096398	0.01576789	0.90309497
0.03181384	0.90797076	1	nonDEG	-0.6627701	0.11131222	1
-2.0431994	1.65E-05	0.00105563	DEG	-1.3672467	0.05216972	1
-1.3756958	0.00777898	0.12239731	nonDEG	-0.8234219	0.2715313	1
0.41572124	0.11948505	0.63063362	nonDEG	0.41209768	0.31144815	1
-1.2551047	0.00889669	0.13438152	nonDEG	-1.2365269	0.16119903	1
-0.3523943	0.32418668	0.90754391	nonDEG	-0.9427864	0.04459505	1
-0.1171479	0.72860001	1	nonDEG	-0.1644356	0.73230773	1
1.88428099	1.55E-12	8.42E-10	DEG	0.49994305	0.24000891	1
1.11622903	0.01777925	0.21233402	nonDEG	0.71085842	0.22141356	1
2.51105088	0.00012382	0.00527513	DEG	0.58721457	0.40066371	1

0.72857947	0.00657353	0.10774959	nonDEG	0.124932	0.73574669	1
0.52477357	0.16601892	0.71926871	nonDEG	0.24755016	0.56172135	1
0.88302116	0.00902291	0.13593939	nonDEG	0.54802985	0.2108436	1
-0.4321058	0.43620036	0.97402891	nonDEG	1.11483358	0.05906702	1
0.49012566	0.05857003	0.44089009	nonDEG	0.41977681	0.25347218	1
-1.6025152	0.00445011	0.08189458	nonDEG	-1.157507	0.16208347	1
-0.2683392	0.69735796	1	nonDEG	1.14050666	0.04992633	1
-0.3839502	0.50036763	1	nonDEG	-0.6620295	0.3327387	1
1.6702027	1.01E-07	1.41E-05	DEG	0.29902335	0.403884	1
0.84881786	0.01306787	0.17391722	nonDEG	0.33578087	0.45608579	1
-0.3136247	0.24032506	0.82199714	nonDEG	-0.2569504	0.55573994	1
-0.8961306	0.07680774	0.50766571	nonDEG	-0.5934686	0.27644299	1
0.96534163	0.10269827	0.5846711	nonDEG	-0.0144666	0.98637152	1
1.22583158	0.00474667	0.08547916	nonDEG	0.30077167	0.51259791	1
-3.1147181	3.30E-10	9.80E-08	DEG	-0.0402131	0.95114886	1
0.03669989	0.89394671	1	nonDEG	-0.5332816	0.16060078	1
-0.7562675	0.02642585	0.27499133	nonDEG	-0.125863	0.78762983	1
1.19617049	0.0003674	0.01265012	DEG	-0.3240239	0.44780866	1
1.63691393	1.53E-08	2.83E-06	DEG	0.05586595	0.88607158	1
-0.4000722	0.59923668	1	nonDEG	1.29227847	0.01617162	0.91202355
-0.9165513	0.12802728	0.65005088	nonDEG	-3.2089467	0.00469283	0.55260852
0.40121458	0.7201087	1	nonDEG	0.90707033	0.39628809	1
-1.4545912	1.07E-05	0.00074044	DEG	-1.9148277	0.00014994	0.0580802
1.21753815	0.00201471	0.04619236	DEG	0.24161569	0.59497236	1
0.51438657	0.09416821	0.55959186	nonDEG	0.14039315	0.73038757	1
0.09606847	0.84819664	1	nonDEG	-0.6926614	0.25254885	1
-0.7109626	0.00473297	0.08532397	nonDEG	-0.0711142	0.87442975	1
-0.1987366	0.45312426	0.98329655	nonDEG	0.03599526	0.92660345	1
0.63854878	0.0981127	0.57137375	nonDEG	0.96349018	0.04026692	1
0.85496094	0.00232259	0.05091593	nonDEG	0.63638242	0.09089968	1
1.22599155	6.96E-05	0.00328951	DEG	0.26080173	0.48048149	1
0.48759564	0.19259929	0.76192362	nonDEG	-0.0937823	0.839592	1
-0.2297964	0.72025648	1	nonDEG	1.22571482	0.02718485	1
0.47201286	0.07931767	0.51602934	nonDEG	0.71481535	0.0473205	1
1.52110585	0.00508928	0.0894593	nonDEG	0.44074861	0.41145236	1
0.40107591	0.26682517	0.85072996	nonDEG	0.3443196	0.39234783	1
-0.8745845	0.00184745	0.04319856	DEG	-0.3876095	0.35684473	1
-0.7227299	0.00753428	0.11947701	nonDEG	-0.1659633	0.6799212	1
1.78005682	0.10263542	0.5845394	nonDEG	0.32502004	0.81162233	1
0.12467635	0.81970271	1	nonDEG	0.60437377	0.25939195	1
-0.9381518	0.00213515	0.04805567	DEG	-0.193541	0.66042915	1
0.76629204	0.03712901	0.34008606	nonDEG	0.78354335	0.04549164	1
-1.6020673	9.90E-05	0.00438243	DEG	-1.3768465	0.03074827	1

0.73133542	0.1964304	0.76773594	nonDEG	0.00887025	0.9915915	1
0.93704507	0.10960858	0.60490056	nonDEG	0.43318365	0.48433603	1
-0.8124047	0.00260214	0.05543002	nonDEG	-0.3611214	0.36002686	1
0.74578947	0.16173393	0.71191465	nonDEG	0.17276934	0.78463038	1
0.14746072	0.56532651	1	nonDEG	-0.5368979	0.16309536	1
-0.5126014	0.10601975	0.5963155	nonDEG	0.06588044	0.88297129	1
1.35432905	0.03221575	0.31197349	nonDEG	-0.0739959	0.91606903	1
-2.4226205	3.91E-10	1.13E-07	DEG	0.2764285	0.75447964	1
-1.6216677	0.00377935	0.07362834	nonDEG	-0.6220153	0.28837924	1
-0.0057602	0.98804811	1	nonDEG	-0.4601864	0.33083327	1
1.09998193	0.00046875	0.01524823	DEG	-0.466569	0.217453	1
0.01436506	0.9600471	1	nonDEG	-0.9605211	0.01825753	0.95785021
2.2459806	0.03084485	0.30332924	nonDEG	-1.7723528	0.09051185	1
0.93021909	0.11651377	0.62432634	nonDEG	0.07253767	0.92772448	1
1.22052271	3.52E-05	0.00194255	DEG	0.5112654	0.19440726	1
-0.4851438	0.0804049	0.51953337	nonDEG	-0.2328592	0.55049601	1
-0.6380291	0.01082588	0.15510149	nonDEG	0.08996976	0.81746546	1
0.62028983	0.01467737	0.18753775	nonDEG	0.1676095	0.64514722	1
-1.4215394	0.07224609	0.49039721	nonDEG	-1.6451849	0.13355538	1
0.78232932	0.00382745	0.07417788	nonDEG	0.20831677	0.57114387	1
-0.6279415	0.01215123	0.16684709	nonDEG	-0.1847613	0.63616569	1
-1.162614	0.00716176	0.11477135	nonDEG	-1.070528	0.09587978	1
-1.0377947	0.07879598	0.51427855	nonDEG	-0.6841932	0.3966875	1
1.70309534	0.00130185	0.03309607	DEG	0.42109037	0.54326136	1
0.53557644	0.09046006	0.54840479	nonDEG	0.20644008	0.59682402	1
-1.5928847	2.66E-07	3.12E-05	DEG	-0.3225524	0.51108314	1
-0.6125609	0.01652996	0.20175184	nonDEG	-0.5106022	0.16237877	1
-0.8313044	0.00502119	0.08868937	nonDEG	-0.3198683	0.44695135	1
-0.9918586	0.19824499	0.77044677	nonDEG	1.73181961	0.01218174	0.81648099
-0.9745921	0.0001817	0.00712275	DEG	-0.0473727	0.90463169	1
0.18370951	0.55243902	1	nonDEG	0.81547986	0.05025041	1
-1.5078173	9.49E-10	2.45E-07	DEG	-0.017801	0.97540464	1
1.39468446	2.58E-08	4.36E-06	DEG	0.15877479	0.66287799	1
0.90206759	0.00055345	0.01724126	DEG	-0.370498	0.31277881	1
1.14159729	5.26E-06	0.00041862	DEG	0.78710336	0.08629718	1
-1.5845566	0.01470393	0.18763285	nonDEG	-0.1628292	0.88567027	1
-0.6426634	0.0960044	0.56558685	nonDEG	-0.3283626	0.54087616	1
-0.8977062	0.05584017	0.42965992	nonDEG	-1.1419093	0.05651152	1
0.06785308	0.78324132	1	nonDEG	0.30150505	0.45397895	1
3.54252403	7.19E-29	1.06E-24	DEG	0.34615119	0.48144598	1
-0.5696965	0.0761719	0.50539204	nonDEG	-0.7408437	0.09826529	1
-0.4062849	0.09978679	0.57465247	nonDEG	-0.2170124	0.60702207	1
0.93328442	0.02743262	0.28197936	nonDEG	0.93611822	0.04795805	1

1.71690769	0.01280521	0.17183271	nonDEG	0.31908542	0.69558101	1
0.47770978	0.10169664	0.58121674	nonDEG	0.2976406	0.46657086	1
1.072034	0.08659343	0.53906198	nonDEG	0.62375232	0.31305232	1
-0.2920477	0.31617216	0.89931324	nonDEG	-0.1235389	0.77221664	1
0.37739506	0.47885012	0.99322685	nonDEG	0.63208004	0.1824249	1
-1.3286343	0.00022572	0.00846507	DEG	-0.2351295	0.57329898	1
1.08595218	0.00378158	0.07362834	nonDEG	0.19610846	0.66605797	1
-1.4705034	0.00510424	0.08954938	nonDEG	-0.9296935	0.21464802	1
1.28134543	0.03559968	0.33165156	nonDEG	0.36601478	0.53680859	1
-0.6025593	0.01807368	0.21460783	nonDEG	-0.5431805	0.13811655	1
2.17426077	0.00439721	0.08146604	nonDEG	0.39811126	0.57414513	1
-0.8073802	0.24691018	0.82856236	nonDEG	-3.1076589	7.95E-05	0.03856757
-0.556025	0.34410416	0.91869633	nonDEG	-2.6821332	0.00052771	0.14642867
-0.3086608	0.21757954	0.79569244	nonDEG	-0.0142142	0.96893773	1
1.31078765	8.69E-07	8.79E-05	DEG	0.35927202	0.53591282	1
-2.0114825	0.00118681	0.03078286	DEG	-1.8519509	0.01267909	0.82741574
-0.1879462	0.78768775	1	nonDEG	1.2577826	0.11734889	1
-1.0358728	0.0007106	0.02083588	DEG	-0.1431224	0.72319746	1
0.57000794	0.02616055	0.27367988	nonDEG	0.50784485	0.17466769	1
-1.1750402	0.00081584	0.02291741	DEG	-1.789754	0.00299359	0.4301506
-0.1058702	0.69969725	1	nonDEG	-0.948081	0.0163237	0.91756207
1.06669706	0.05513348	0.42600382	nonDEG	0.74454394	0.2780399	1
1.28687426	0.05317606	0.41796662	nonDEG	0.02357491	0.9819041	1
-1.8909116	5.11E-10	1.44E-07	DEG	-0.0059069	0.99553729	1
-0.1967124	0.42888617	0.97063192	nonDEG	0.22356484	0.6342753	1
-2.1184198	0.00387404	0.07473655	nonDEG	0.86544635	0.4290939	1
-0.0733749	0.86606324	1	nonDEG	-0.1050258	0.84038782	1
0.0252841	0.93305338	1	nonDEG	0.85632815	0.03115085	1
-1.7324877	0.01471761	0.18772601	nonDEG	-1.0111215	0.48630739	1
-0.4121957	0.28328859	0.86547663	nonDEG	-0.5951859	0.23663219	1
0.59870491	0.08690877	0.53965561	nonDEG	0.87260708	0.04380088	1
1.99903262	0.03186824	0.3094616	nonDEG	0.50534462	0.53419658	1
-2.3654508	1.55E-09	3.83E-07	DEG	-0.0099548	1	1
-0.4553169	0.15905851	0.70668933	nonDEG	-0.5482613	0.20430842	1
0.74702344	0.01444098	0.1856454	nonDEG	0.11272481	0.77245925	1
1.79368539	0.00013926	0.00575943	DEG	0.06483463	0.90245896	1
0.5578002	0.43032694	0.9716987	nonDEG	0.43076969	0.54007412	1
-0.6615315	0.00742126	0.11797119	nonDEG	-0.0677171	0.881728	1
0.32564583	0.24441237	0.82643802	nonDEG	0.67314917	0.06476511	1
0.45682592	0.08189632	0.52356398	nonDEG	0.12574941	0.75468273	1
-0.6665726	0.17165215	0.7301355	nonDEG	0.03039081	0.97117742	1
1.80460337	6.90E-06	0.00052633	DEG	0.39021962	0.37879347	1
0.34379201	0.42393581	0.96620031	nonDEG	0.47400246	0.31589957	1

-0.8975059	0.05799738	0.43880364	nonDEG	-0.3048967	0.70757449	1
0.58378372	0.06531419	0.4669909	nonDEG	0.17993945	0.67063931	1
-1.2322934	0.04744052	0.39241397	nonDEG	2.98564447	1.69E-10	1.65E-06
0.26262367	0.39458176	0.94862012	nonDEG	-0.6466881	0.2008411	1
-1.2313973	6.21E-05	0.00300556	DEG	-0.942772	0.10514941	1
-0.5782078	0.08463414	0.53272276	nonDEG	-0.2161126	0.62713476	1
0.37535627	0.15380848	0.69420532	nonDEG	0.30413804	0.42129041	1
-0.4872215	0.06445779	0.46426144	nonDEG	0.01264158	0.9759797	1
-0.7970286	0.01234873	0.16837141	nonDEG	-0.8876926	0.0476468	1
0.49727007	0.05757191	0.436482	nonDEG	0.47287024	0.30542986	1
0.9396673	0.00151766	0.03701611	DEG	0.20795865	0.59468691	1
1.34544502	5.16E-08	7.87E-06	DEG	0.0792207	0.82584939	1
0.33175339	0.28182467	0.86297446	nonDEG	0.17897313	0.64851196	1
-0.6974759	0.05158483	0.41110063	nonDEG	-0.7743426	0.12864682	1
-0.5483922	0.09626848	0.56624334	nonDEG	-0.2163709	0.58103469	1
-1.1148629	0.09014445	0.54766713	nonDEG	2.01730012	0.00053371	0.14642867
-0.952077	0.16723341	0.72210453	nonDEG	-0.3953061	0.64462845	1
0.24766812	0.44693741	0.98167503	nonDEG	0.28804558	0.47276683	1
-0.7291889	0.05652446	0.43232624	nonDEG	-0.5091978	0.29792392	1
4.46288933	0.26880688	0.85209493	nonDEG	-0.0831419	1	1
0.23428586	0.45038966	0.98329655	nonDEG	0.75341244	0.06842994	1
-1.2814185	0.10224636	0.58333928	nonDEG	-0.7032968	0.4895014	1
-0.4862968	0.06627465	0.47058314	nonDEG	-0.3667927	0.34668249	1
0.81362711	0.22884097	0.80924485	nonDEG	0.94177551	0.14455486	1
5.13E-15	1	1	nonDEG	4.13622954	0.25891009	1
-1.4474398	0.02512083	0.26586493	nonDEG	-2.5328047	0.01172253	0.81009283
0.95640634	0.31154196	0.89482226	nonDEG	-3.6168971	0.00759907	0.67731627
-0.53282	0.37266188	0.93142022	nonDEG	1.08225458	0.02866841	1
1.17430676	0.00262397	0.05577394	nonDEG	0.31401099	0.46709195	1
-0.369073	0.12730509	0.64806063	nonDEG	-0.068588	0.84789678	1
-1.4832455	5.75E-05	0.00281928	DEG	-0.3983318	0.56162487	1
-1.2143311	1.26E-06	0.00012122	DEG	-0.0952919	0.81463102	1
0.92588821	0.05307118	0.41756807	nonDEG	0.6369592	0.19886542	1
-0.7265562	0.03583056	0.33244726	nonDEG	-0.0019503	1	1
0.09365143	0.73501815	1	nonDEG	-0.6062667	0.15406872	1
-2.2355744	5.77E-05	0.00282721	DEG	-2.1869594	0.006784	0.65266053
-0.5737938	0.03191888	0.30960786	nonDEG	-0.4827011	0.22865612	1
0.89831644	0.04913306	0.40067265	nonDEG	0.1964603	0.63362875	1
0.37299736	0.1226181	0.63832968	nonDEG	0.00566215	0.98981914	1
-1.5244856	0.00252454	0.05428767	nonDEG	-1.3497456	0.04728062	1
-1.8217247	0.00791211	0.12383014	nonDEG	0.24195815	0.77109268	1
-1.0428185	0.15407161	0.69470271	nonDEG	-1.9928265	0.05473798	1
-1.161546	0.16605954	0.71927717	nonDEG	-0.3400627	0.76843488	1

-0.1926831	0.7564157	1	nonDEG	0.1428161	0.85928119	1
-1.4828864	3.88E-07	4.25E-05	DEG	-0.4941956	0.23056594	1
0.92153282	0.00061309	0.01868401	DEG	0.10082698	0.78557906	1
-2.1801462	0.00777597	0.12239731	nonDEG	-2.2720793	0.07687201	1
0.16031867	0.8455781	1	nonDEG	1.23893199	0.10871523	1
0.96528501	0.03908139	0.34897353	nonDEG	1.14126878	0.16585592	1
1.35626048	0.01317399	0.17462252	nonDEG	0.14534319	0.85409903	1
-0.2929499	0.50484059	1	nonDEG	-0.4141589	0.46857032	1
-0.7384759	0.00324049	0.06576221	nonDEG	-0.355304	0.40340026	1
1.47928937	0.31374702	0.89645215	nonDEG	1.08208894	0.36658848	1
1.520074	6.00E-09	1.27E-06	DEG	0.42295858	0.48762045	1
-1.0801379	0.00629443	0.10463107	nonDEG	-0.5323862	0.3355649	1
0.76381033	0.04778043	0.3935669	nonDEG	0.41910859	0.3568776	1
-0.2917724	0.60545862	1	nonDEG	1.04930864	0.09860177	1
-0.8835039	0.00071057	0.02083588	DEG	0.14366414	0.81798023	1
1.29614845	6.24E-05	0.00300561	DEG	0.32068453	0.39907904	1
-0.7382532	0.1149883	0.61930855	nonDEG	-0.6590241	0.29317592	1
-0.5179193	0.04073104	0.35836253	nonDEG	-0.0276692	0.94231301	1
-0.3740135	0.41024751	0.95836418	nonDEG	-0.5219693	0.43586273	1
0.57082841	0.03142516	0.30683212	nonDEG	0.352098	0.33936735	1
0.03535775	0.88964643	1	nonDEG	-0.8002421	0.02846242	1
-1.5154136	9.40E-09	1.85E-06	DEG	-0.1096572	0.88983943	1
0.94223994	0.03112637	0.30534124	nonDEG	0.25639875	0.63347624	1
0.23532016	0.36113443	0.92354131	nonDEG	0.56116062	0.23472989	1
-1.1418403	0.00586993	0.09937032	nonDEG	-0.6425547	0.29282497	1
0.52658766	0.07134597	0.4877738	nonDEG	-0.0784305	0.86150258	1
-2.5227957	0.00244133	0.05288478	nonDEG	-1.2069218	0.25940096	1
-0.0817116	0.77986294	1	nonDEG	-0.631862	0.11244066	1
-0.5099677	0.05340713	0.41877092	nonDEG	-1.5586666	7.99E-05	0.03856757
0.71207005	0.19357463	0.76348387	nonDEG	1.26191229	0.1096845	1
1.49785223	0.13605888	0.66313188	nonDEG	0.7131297	0.48497386	1
0.60960592	0.01334291	0.17598655	nonDEG	0.21552455	0.5501312	1
-1.0217956	0.00389392	0.07487047	nonDEG	0.66533051	0.21425294	1
5.13E-15	1	1	nonDEG	3.14246924	0.64097099	1
-1.2347726	0.18008736	0.74514618	nonDEG	-1.4189799	0.24329983	1
-0.5265017	0.04142634	0.36166963	nonDEG	-0.1566913	0.75812258	1
0.19121057	0.43567578	0.97391956	nonDEG	0.37127826	0.36496196	1
-0.3342356	0.20133761	0.77336631	nonDEG	-0.3754946	0.30541417	1
0.64294409	0.019571	0.22611878	nonDEG	0.69011023	0.06208791	1
0.89870124	0.00635377	0.10520171	nonDEG	0.32186332	0.42440402	1
-1.3398958	1.36E-05	0.00090118	DEG	-0.0032123	0.99829623	1
0.66129786	0.1636454	0.714154	nonDEG	0.7007469	0.16495584	1
-2.1005211	0.13082667	0.65446487	nonDEG	-1.1984102	0.63874605	1

-0.6036287	0.10409419	0.59010761	nonDEG	-1.3767093	0.00874519	0.7231729
-0.354993	0.4263048	0.96769119	nonDEG	-0.8281468	0.14027531	1
0.10595417	0.89896912	1	nonDEG	1.16970611	0.06907665	1
1.88482712	0.02412796	0.25949182	nonDEG	-0.0078532	1	1
0.95779594	0.03015484	0.29859682	nonDEG	0.12762088	0.80800215	1
-0.4631325	0.14367946	0.67633117	nonDEG	-0.6253177	0.19701526	1
-0.6965958	0.03413365	0.32289859	nonDEG	-0.1897579	0.68749723	1
0.22337736	0.39668601	0.94951131	nonDEG	-0.4237292	0.39023185	1
0.46451523	0.15448294	0.69581138	nonDEG	0.32539101	0.43989059	1
0.63068347	0.01241834	0.16851066	nonDEG	0.18411123	0.62186515	1
-0.7120295	0.1376853	0.66503679	nonDEG	-0.9248479	0.1603247	1
-1.1037982	0.00121344	0.03134901	DEG	-0.4177349	0.37794776	1
1.2957007	0.00097703	0.0262915	DEG	-0.3139222	0.50686573	1
0.3182187	0.21940782	0.79870739	nonDEG	0.26984246	0.4587991	1
-0.9497886	0.00678544	0.11024097	nonDEG	-0.4038798	0.47154594	1
-1.143769	0.00112108	0.02946741	DEG	-0.2582958	0.54493307	1
0.9403243	0.12504138	0.64380956	nonDEG	0.21568013	0.73811246	1
0.02183403	0.92878323	1	nonDEG	-0.6503152	0.07472376	1
-0.6328446	0.01105227	0.15718248	nonDEG	-0.2757619	0.48596396	1
1.09345315	0.06946219	0.48274253	nonDEG	0.70999018	0.29042572	1
1.1460548	4.83E-05	0.00245316	DEG	0.81459922	0.03791308	1
1.64711143	1.67E-07	2.15E-05	DEG	0.03565841	0.93222335	1
0.25492325	0.29873829	0.88387501	nonDEG	0.49223261	0.17563633	1
-0.556558	0.10391706	0.58967123	nonDEG	-0.5960313	0.21685012	1
1.30253849	0.04211589	0.36479284	nonDEG	0.64953843	0.30551117	1
0.92204339	0.0066978	0.10923913	nonDEG	-0.011713	0.9792039	1
1.17934222	0.00263485	0.05584404	nonDEG	0.09418741	0.82395741	1
-1.6615371	1.90E-05	0.00119808	DEG	-1.6586416	0.01089075	0.79590774
-0.5283519	0.04178476	0.36372272	nonDEG	-0.3731745	0.33356342	1
2.94409209	9.03E-05	0.00407898	DEG	-0.9133288	0.412566	1
2.44017194	0.00104903	0.02794778	DEG	-0.3573125	0.60335959	1
-0.8628327	0.0046685	0.08444543	nonDEG	0.66626982	0.24528445	1
-0.5667367	0.04428901	0.37573954	nonDEG	-0.7187464	0.13052964	1
-0.7549863	0.06763768	0.4754102	nonDEG	-0.5813138	0.25100892	1
0.01215626	0.99158629	1	nonDEG	0.95565013	0.11364651	1
1.13726669	0.02451149	0.26192153	nonDEG	0.60274021	0.33230165	1
0.53931907	0.28845533	0.87024354	nonDEG	0.35242126	0.50348772	1
0.40665737	0.42608169	0.96763241	nonDEG	0.86134793	0.15792711	1
0.43735565	0.56136841	1	nonDEG	0.92595192	0.26497646	1
0.6631805	0.30880214	0.89262089	nonDEG	0.98101057	0.08055358	1
-0.4109957	0.15041529	0.68725272	nonDEG	-0.3770738	0.3335297	1
-0.1875189	0.51707389	1	nonDEG	-0.4961678	0.25221169	1
0.294401	0.66028703	1	nonDEG	0.33700801	0.65463748	1

0.6474676	0.21818502	0.79663858	nonDEG		0.67854159	0.23681522	1
2.45387178	9.75E-06	0.00069011	DEG		-1.0972318	0.18017206	1
-0.0825352	0.88862868	1	nonDEG		0.92947152	0.05169752	1
0.62756619	0.01431526	0.18532413	nonDEG		0.13861181	0.71891167	1
-0.6564963	0.18755229	0.75310345	nonDEG		-0.0074755	1	1
-0.493838	0.19935925	0.77176073	nonDEG		-0.3318551	0.56106169	1
-0.545449	0.17164459	0.7301355	nonDEG		-0.3606492	0.49002434	1
0.6101162	0.12641119	0.64619186	nonDEG		0.54931684	0.19836998	1
1.38502338	0.22373541	0.80443197	nonDEG		-0.5828952	0.62023323	1
1.05799805	0.00075578	0.02185591	DEG		0.27949102	0.521463	1
0.16851327	0.60492513	1	nonDEG		0.58503055	0.19120422	1
-0.315069	0.19407812	0.76383173	nonDEG		-0.0665844	0.85948739	1
-0.8281395	0.0007663	0.0220591	DEG		0.07133669	0.86219758	1
-2.4136759	5.99E-07	6.25E-05	DEG		0.40875082	0.73137834	1
-0.9970893	0.00238715	0.0519788	nonDEG		-0.8296028	0.05495131	1
0.32310004	0.18500117	0.75270911	nonDEG		0.07715293	0.83028042	1
-0.84485	0.05069905	0.40690549	nonDEG		-1.9749961	0.0009536	0.21761931
0.38621698	0.11928929	0.63063362	nonDEG		0.3495899	0.34342845	1
-1.0326575	0.30242183	0.88706147	nonDEG		2.17790143	0.00512863	0.56974199
0.17975197	0.48941864	0.99867487	nonDEG		0.61193389	0.09000044	1
-1.2733719	2.85E-05	0.00163467	DEG		-0.4051837	0.37582765	1
-0.2123738	0.71565888	1	nonDEG		0.11144849	0.8690565	1
0.56133496	0.03726915	0.34062917	nonDEG		-0.0285119	0.94030258	1
0.34831278	0.17535885	0.73885631	nonDEG		0.10195495	0.78268938	1
0.17422972	0.73566487	1	nonDEG		0.73498624	0.08729103	1
0.6637061	0.28798817	0.87024354	nonDEG		0.2776583	0.66628295	1
0.33880342	0.36157359	0.92354131	nonDEG		0.48770706	0.23619972	1
3.30309048	0.63667104	1	nonDEG		2.6899385	0.11376662	1
1.19133088	0.00288354	0.05999192	nonDEG		0.4576315	0.31136585	1
-1.0100331	0.05149833	0.41074486	nonDEG		-1.3736086	0.04705545	1
0.60876078	0.65960287	1	nonDEG		0.97045101	0.46783796	1
2.44483207	0.00988797	0.14474983	nonDEG		0.46969531	0.61806184	1
2.16073845	4.93E-05	0.00249161	DEG		-1.6968677	0.01599312	0.90504819
0.66782786	0.04511613	0.37991238	nonDEG		0.78158535	0.05436988	1
1.21037019	0.10735291	0.59980305	nonDEG		1.00652036	0.13494343	1
-0.6840261	0.27732917	0.85935509	nonDEG		-1.7141126	0.04668088	1
-1.6337348	7.59E-07	7.79E-05	DEG		-0.0167556	0.98305151	1
1.60556965	2.47E-05	0.00146252	DEG		0.1296198	0.75454715	1
-0.0708724	0.94152109	1	nonDEG		1.4983764	0.01870933	0.95785021
-0.366086	0.19740886	0.76896966	nonDEG		-0.7055712	0.29731411	1
-0.8650155	0.00228029	0.05029026	nonDEG		-1.2434998	0.01285379	0.82858811
-0.4302466	0.09034101	0.54811204	nonDEG		-0.4597757	0.22397273	1
-0.552266	0.03529889	0.32999806	nonDEG		-0.2060177	0.61408534	1

-0.7115997	0.07918137	0.51537041	nonDEG	-1.2514644	0.01368809	0.84878381
1.4375932	0.00082509	0.02313318	DEG	0.48944905	0.43295451	1
-1.2125298	0.10005767	0.57546993	nonDEG	-1.1213546	0.2113091	1
1.17401268	0.0144079	0.18561379	nonDEG	0.28316957	0.57299635	1
0.37078467	0.16002799	0.70893014	nonDEG	0.17932419	0.62033008	1
-0.5878089	0.04567981	0.38301564	nonDEG	-0.1170254	0.78399485	1
-1.6376044	0.00444265	0.08189458	nonDEG	-0.9241989	0.33674777	1
-0.5827739	0.08581843	0.53616486	nonDEG	-0.8411389	0.04904548	1
-2.0528333	0.00014742	0.00600259	DEG	-0.9451729	0.28025476	1
-0.4798667	0.16740363	0.72239744	nonDEG	-0.6499422	0.24539752	1
1.56444428	0.02894766	0.29194591	nonDEG	0.40851274	0.55304539	1
-1.0912615	4.49E-05	0.00232879	DEG	-0.5836684	0.15793355	1
-0.0813872	0.76136662	1	nonDEG	0.17066208	0.64613595	1
-0.2822961	0.28079284	0.86186717	nonDEG	-0.4651599	0.25769256	1
0.73240839	0.00450982	0.08261638	nonDEG	0.37018067	0.30600052	1
1.59346019	0.01014103	0.1472171	nonDEG	0.7199487	0.23226301	1
-0.4316382	0.11051722	0.60738686	nonDEG	-0.4370976	0.31434449	1
1.75177057	0.012502	0.16898369	nonDEG	0.02470092	0.97935752	1
-1.2568546	0.0111399	0.1578948	nonDEG	-0.0687308	0.93516172	1
-1.3741412	0.01634862	0.20053631	nonDEG	-2.2179555	0.00593836	0.60336628
-2.0947729	0.00998274	0.14570735	nonDEG	-1.8490377	0.10547172	1
1.23564674	0.01454495	0.18628913	nonDEG	0.5835131	0.32449306	1
2.03043743	4.22E-15	3.76E-12	DEG	-0.4826605	0.27523301	1
0.33392774	0.30938957	0.89277786	nonDEG	0.49288391	0.21409745	1
0.55853886	0.08160988	0.52300761	nonDEG	0.41835408	0.29638866	1
2.54070428	0.00062662	0.01903737	DEG	-3.7596184	2.24E-06	0.00412442
0.63507362	0.01927242	0.2233886	nonDEG	0.3421602	0.41869612	1
0.54141944	0.03405891	0.32260627	nonDEG	0.08777796	0.80896051	1
-2.4883117	0.01124349	0.15867556	nonDEG	-5.3089698	0.03978299	1
-0.4564847	0.12553575	0.64431252	nonDEG	-0.2347399	0.5320346	1
-0.8306363	0.02695218	0.27879316	nonDEG	0.09403888	0.85299186	1
-0.2348543	0.33870246	0.91869633	nonDEG	-0.2552544	0.47886979	1
0.72331981	0.01370064	0.17886168	nonDEG	-0.360508	0.61127138	1
0.3443885	0.17300967	0.73256216	nonDEG	0.33478521	0.36846151	1
0.41255273	0.43784819	0.97465503	nonDEG	0.30554051	0.60804512	1
-0.7246536	0.00686116	0.11122554	nonDEG	-0.5131624	0.17376316	1
-1.2260238	0.00510048	0.08953675	nonDEG	-0.5855123	0.36117971	1
1.26765151	9.99E-05	0.00441614	DEG	-0.0312498	0.93753862	1
-0.4721931	0.07603022	0.50513508	nonDEG	-0.1916751	0.62057407	1
-1.4043512	1.99E-07	2.46E-05	DEG	-0.5612122	0.15684195	1
0.1605873	0.80258344	1	nonDEG	1.20839458	0.08521948	1
-0.5781632	0.25218496	0.83371668	nonDEG	-0.9901426	0.1129988	1
0.56760203	0.34998193	0.91917773	nonDEG	0.9110529	0.14074208	1

-1.2946213	0.00383337	0.07424376	nonDEG	-1.5388269	0.00677865	0.65266053
-0.3781847	0.12113988	0.63331625	nonDEG	-0.2423173	0.50159156	1
0.37625852	0.30064032	0.88532063	nonDEG	0.50809925	0.2352253	1
-0.3940497	0.15844782	0.70535995	nonDEG	-0.4440438	0.25260525	1
-0.6899107	0.03011087	0.29847765	nonDEG	-0.1598692	0.72556976	1
-0.0643243	0.80558262	1	nonDEG	-0.2171957	0.60898272	1
-1.2943078	0.00013196	0.00554144	DEG	-0.8255097	0.11939077	1
2.17235945	2.90E-11	1.19E-08	DEG	0.38971545	0.29615057	1
1.23380651	2.48E-05	0.00146252	DEG	0.0851812	0.82344772	1
-2.0840006	0.00128377	0.03274938	DEG	-1.409943	0.10887745	1
-0.9037935	0.00857013	0.13068264	nonDEG	-0.3177408	0.49318344	1
-0.3273686	0.29126097	0.87342689	nonDEG	-0.7202808	0.07660402	1
-0.2900902	0.31422824	0.89750316	nonDEG	-0.0507953	0.91046772	1
0.38548075	0.13581726	0.66261751	nonDEG	0.08800721	0.81865431	1
-0.3074027	0.24183936	0.82401585	nonDEG	-0.4957526	0.18508254	1
0.4989122	0.04132252	0.3610802	nonDEG	0.31305635	0.43338019	1
-0.3516639	0.57250177	1	nonDEG	1.06006102	0.04286517	1
-0.8885941	0.00417527	0.07844016	nonDEG	-0.0023488	0.99888337	1
-0.0628278	0.8263341	1	nonDEG	-0.6110822	0.17212827	1
0.85644278	0.00221681	0.04929058	DEG	0.12196324	0.76225538	1
0.90030931	0.00133085	0.03362984	DEG	-0.0531757	0.88608724	1
-0.7789487	0.13745297	0.66468777	nonDEG	-0.4817768	0.46366529	1
1.8021472	0.00212887	0.0479876	DEG	0.52614457	0.40055591	1
-0.9551305	0.0050054	0.08855415	nonDEG	-0.8578245	0.09932783	1
0.41670505	0.16106126	0.71096258	nonDEG	0.37230617	0.34372674	1
-1.3914245	0.00995147	0.14539026	nonDEG	-1.9378056	0.02170864	1
0.67118343	0.17632536	0.7409355	nonDEG	0.33207907	0.56183916	1
-0.3442761	0.17852391	0.74433965	nonDEG	0.0963715	0.83398716	1
-0.6293727	0.03583189	0.33244726	nonDEG	-0.6965261	0.14365106	1
0.17399001	0.63188818	1	nonDEG	0.8502407	0.04651734	1
-0.3643716	0.15684187	0.70153162	nonDEG	-0.3569789	0.3591043	1
-0.3376605	0.20700149	0.78247522	nonDEG	0.0036455	0.9927909	1
-1.477221	0.00080095	0.02267215	DEG	0.64595107	0.32252737	1
1.63123819	0.00089947	0.02467787	DEG	0.44938163	0.54790895	1
-1.1164638	0.00493144	0.0878794	nonDEG	-0.8630342	0.13252293	1
-1.385519	0.34216922	0.91869633	nonDEG	-5.0477436	0.16361453	1
-0.5250778	0.18703763	0.75310345	nonDEG	-0.4871638	0.35755755	1
0.36286038	0.27634151	0.85842562	nonDEG	0.52552856	0.28298493	1
0.73558664	0.05736155	0.43581086	nonDEG	0.38834792	0.35120933	1
-0.3401587	0.39031548	0.94686517	nonDEG	-0.7125504	0.22574467	1
0.09487969	0.91427186	1	nonDEG	0.6621132	0.42281627	1
-0.7696867	0.00221871	0.04929297	DEG	-0.0370315	0.92252232	1
-0.6086938	0.24434704	0.82643802	nonDEG	-0.5201455	0.44180886	1

-1.9559056	1.00E-06	0.00010013	DEG	-2.3277436	9.40E-05	0.04219605
1.32388146	1.28E-07	1.70E-05	DEG	0.36210889	0.33131499	1
-0.046861	0.86135266	1	nonDEG	-0.2622357	0.50091081	1
-1.4634458	0.00464266	0.08426342	nonDEG	-1.5726661	0.04106871	1
-0.2674608	0.28393224	0.86646664	nonDEG	-0.1085426	0.76614582	1
0.58076934	0.35046781	0.91917773	nonDEG	0.40698942	0.6563749	1
1.24645432	6.96E-06	0.00052977	DEG	0.27506966	0.4547064	1
-0.4376226	0.09723125	0.56917324	nonDEG	-0.1301498	0.77678222	1
-1.1929725	0.01212332	0.16661931	nonDEG	-1.2647958	0.05326535	1
-0.6326293	0.0179089	0.21344943	nonDEG	-0.0490587	0.90544885	1
-0.3451792	0.525776	1	nonDEG	-1.7048369	0.02075917	1
-0.3968109	0.15599803	0.69999472	nonDEG	-0.7148313	0.0536779	1
0.11898723	0.83809968	1	nonDEG	0.80806368	0.12121516	1
1.11523522	0.0007502	0.02175886	DEG	0.77449806	0.04413701	1
-0.6023891	0.02634382	0.27465365	nonDEG	-0.4974765	0.1933571	1
-0.5301491	0.05864454	0.44117151	nonDEG	-0.7211863	0.15559203	1
1.42055997	1.65E-05	0.00105563	DEG	0.21816673	0.60734532	1
1.66707708	1.98E-08	3.51E-06	DEG	0.63473804	0.09617988	1
0.36359787	0.1385917	0.6665824	nonDEG	0.65403889	0.06915964	1
-0.2398152	0.32897442	0.91297524	nonDEG	-0.3787203	0.33929699	1
-0.8510006	0.03802498	0.34369589	nonDEG	-1.0331187	0.06429617	1
-0.6519906	0.0543719	0.42178847	nonDEG	-0.2238332	0.65391282	1
1.46062283	6.51E-06	0.00049942	DEG	0.01153471	0.98017869	1
-2.6690936	5.32E-08	8.07E-06	DEG	-0.6208863	0.49111598	1
-1.4482469	0.01712003	0.2068102	nonDEG	0.17425774	0.85505726	1
-0.6073212	0.25068277	0.83275219	nonDEG	-0.6663581	0.29470441	1
0.28163407	0.49619562	1	nonDEG	0.41703566	0.36956753	1
1.63115652	7.80E-06	0.0005738	DEG	-0.1839714	0.66683424	1
0.55630278	0.04819177	0.39562065	nonDEG	0.33255429	0.36898028	1
0.59340932	0.03061757	0.30185892	nonDEG	0.39738694	0.27570772	1
-0.343208	0.17045672	0.72882858	nonDEG	0.2731913	0.45485964	1
-1.0143426	0.00911001	0.13697117	nonDEG	-0.3340192	0.55624658	1
0.223229	0.42615172	0.96764883	nonDEG	0.76820013	0.03567954	1
-0.5189722	0.23964071	0.82146984	nonDEG	-1.8937352	0.00098254	0.21777569
0.98226338	0.00406857	0.07687708	nonDEG	0.3490903	0.34456492	1
-0.522136	0.04823692	0.39562065	nonDEG	-0.3679684	0.3647798	1
-0.4512353	0.16936608	0.72639395	nonDEG	-0.6474928	0.15583099	1
-0.7288421	0.02943208	0.29511279	nonDEG	-0.220746	0.61580773	1
1.16304549	5.61E-05	0.0027789	DEG	0.18184762	0.66731955	1
2.13473138	8.05E-10	2.12E-07	DEG	1.1925518	0.005856	0.60067869
-0.3295632	0.19306038	0.76268178	nonDEG	0.00975833	0.98095477	1
-0.4291311	0.09986168	0.57485879	nonDEG	-0.4769205	0.19176386	1
0.46666451	0.13924088	0.66764074	nonDEG	0.40353022	0.30647623	1

-1.0173391	0.00162079	0.0390463	DEG	0.03236854	0.94855602	1
0.06301863	0.91785017	1	nonDEG	0.7560575	0.19888112	1
0.91671984	0.02113164	0.23725952	nonDEG	0.64797291	0.12791566	1
1.07393348	0.00757541	0.11996367	nonDEG	-1.2309784	0.06311679	1
0.66053497	0.05719703	0.43543403	nonDEG	0.42014822	0.3313655	1
1.27643736	8.51E-05	0.00389046	DEG	0.12308773	0.84122593	1
-0.3925057	0.12926558	0.65245846	nonDEG	-0.2084088	0.57048551	1
-0.4596916	0.28935656	0.8709097	nonDEG	0.68250946	0.09855432	1
-0.6483794	0.09853081	0.57188242	nonDEG	-0.2035157	0.68127863	1
-0.4835954	0.20143829	0.77336631	nonDEG	-0.047735	0.92262125	1
-1.2631657	0.0002498	0.00916261	DEG	-0.4105799	0.32287078	1
-0.3614618	0.1759747	0.74017991	nonDEG	-0.4879274	0.21480805	1
-0.723285	0.131424	0.65512349	nonDEG	-1.2819888	0.0403407	1
0.68865026	0.7292066	1	nonDEG	1.10053209	0.53208886	1
-1.0486057	0.01365814	0.17852636	nonDEG	-0.9002287	0.13219065	1
-1.8591838	0.00207462	0.04720437	DEG	-2.5189609	0.00485393	0.55667563
-1.2719752	1.46E-05	0.00095656	DEG	-0.4315394	0.35378814	1
1.65318491	2.40E-09	5.71E-07	DEG	0.54717602	0.19515568	1
1.33738273	1.31E-05	0.00087189	DEG	0.00660131	0.98760126	1
1.14117026	0.2896341	0.87147774	nonDEG	2.99788199	0.00726734	0.67222413
-0.5292785	0.1386299	0.6665824	nonDEG	-0.3568481	0.46873093	1
-1.0213854	9.88E-05	0.00438169	DEG	-0.3956848	0.32145802	1
-0.2275445	0.45845766	0.98479636	nonDEG	-0.3291122	0.41807487	1
1.09603862	0.00022356	0.00840532	DEG	0.13887539	0.71936019	1
0.75458356	0.14846028	0.6851422	nonDEG	0.11099937	0.83322709	1
0.95799937	0.01608922	0.19901284	nonDEG	0.34891426	0.43806929	1
-0.8979064	0.00564771	0.09664522	nonDEG	-0.4415325	0.33944957	1
-1.0472435	0.00233765	0.05112781	nonDEG	-0.223522	0.6959375	1
-1.1093329	0.0682007	0.47678944	nonDEG	-1.3225698	0.115371	1
-0.9592891	0.01085279	0.1552455	nonDEG	0.2842802	0.57712124	1
-0.2057643	0.53082794	1	nonDEG	-0.1343074	0.74916654	1
-1.0127525	0.02141289	0.23953385	nonDEG	-1.4212811	0.01821358	0.95785021
-1.9723773	0.07497591	0.50188576	nonDEG	-0.3605152	0.794982	1
0.35263188	0.23769134	0.81948793	nonDEG	0.33081669	0.39738429	1
-0.4909584	0.15161028	0.68941225	nonDEG	-1.4739435	0.00730079	0.67222413
0.55021616	0.09583176	0.56525572	nonDEG	0.62277009	0.12179906	1
1.31555335	0.01453011	0.18622197	nonDEG	0.5910558	0.3468515	1
0.65144114	0.01033469	0.14950508	nonDEG	0.53747817	0.1396399	1
-0.4539273	0.11752016	0.62686644	nonDEG	-0.3808937	0.34727745	1
0.63895021	0.10920099	0.60460927	nonDEG	0.30007408	0.48786645	1
-0.7050393	0.13585921	0.66261751	nonDEG	-0.958513	0.13796301	1
-0.540951	0.08316236	0.52786045	nonDEG	-0.1756767	0.68675579	1
-1.1290315	0.00602693	0.10121317	nonDEG	-1.629131	0.00429785	0.52515749

1.55488048	0.00112579	0.02956467	DEG	0.0921684	0.87055872	1
4.15896175	0.34121738	0.91869633	nonDEG	-4.0092744	0.36203011	1
0.90766345	0.0530914	0.41756815	nonDEG	0.1749444	0.74253901	1
1.39832186	0.00034979	0.01217184	DEG	-0.6283936	0.16981193	1
0.17812854	0.50930191	1	nonDEG	0.10658448	0.78454982	1
-0.4231851	0.11205079	0.61181363	nonDEG	0.0896379	0.82809652	1
-0.0949117	0.80001249	1	nonDEG	-0.8586624	0.06938735	1
0.56386899	0.04748242	0.39259984	nonDEG	0.77946765	0.03267872	1
1.12955195	0.00018856	0.00731348	DEG	0.07778181	0.84366218	1
1.72568372	2.74E-09	6.25E-07	DEG	-0.1826427	0.64153326	1
1.06012878	0.03770378	0.34215831	nonDEG	0.12979957	0.83681576	1
-0.6691373	0.00672861	0.109499	nonDEG	0.17516172	0.64394837	1
1.23670317	0.00080447	0.02271523	DEG	0.38450774	0.43321775	1
2.23179766	1.86E-11	8.55E-09	DEG	0.45428923	0.53058268	1
0.78908644	0.28052638	0.86186717	nonDEG	0.50402794	0.4898608	1
-0.8786132	0.00212324	0.0479756	DEG	-1.2271003	0.04388438	1
-0.2955755	0.41127016	0.95855277	nonDEG	0.81265238	0.06107506	1
0.60448989	0.22571216	0.80685891	nonDEG	0.67311909	0.16382559	1
0.58394254	0.07610286	0.50531894	nonDEG	-0.0010584	0.99978949	1
0.35696737	0.18142686	0.74717253	nonDEG	0.27841327	0.45502335	1
0.37616277	0.15427837	0.69531549	nonDEG	0.42329179	0.27184071	1
0.642608	0.11458815	0.6186247	nonDEG	0.04120966	0.93371007	1
1.73374198	2.09E-10	7.07E-08	DEG	0.0782476	0.8290501	1
-2.8780816	0.00043572	0.01454556	DEG	0.10683781	0.94419493	1
1.33299005	0.26449013	0.84892334	nonDEG	0.48782158	0.65225675	1
-0.2561746	0.34488318	0.91869633	nonDEG	-0.3012096	0.46950216	1
-2.8939072	0.00098302	0.02641263	DEG	1.32755539	0.12938138	1
1.01712711	0.00242666	0.05260559	nonDEG	0.0430426	0.92318003	1
-0.0524477	0.92723278	1	nonDEG	-0.6878631	0.2977596	1
0.40051822	0.62324981	1	nonDEG	0.51116558	0.50056314	1
1.23313922	0.00078686	0.0224027	DEG	0.01591491	0.96925595	1
0.68306007	0.41803541	0.96254612	nonDEG	0.5694169	0.50122439	1
1.91131838	0.00013331	0.0055825	DEG	0.29770709	0.54609577	1
0.75869803	0.42977483	0.97110382	nonDEG	0.97995256	0.27423783	1
-0.8530684	0.00065164	0.01959516	DEG	-0.3464632	0.55211644	1
0.26725403	0.63436559	1	nonDEG	0.21724693	0.73756955	1
2.03868246	0.00890903	0.13443004	nonDEG	0.23378293	0.8039332	1
-1.0399676	0.19087071	0.75851009	nonDEG	-2.3724675	0.04660046	1
-1.6232313	0.00019847	0.00761785	DEG	-0.387037	0.51624207	1
-0.678862	0.15826612	0.70487086	nonDEG	-1.0919782	0.19006344	1
-2.3683755	1.71E-06	0.00015716	DEG	0.45799147	0.60316384	1
-0.8214765	0.00480963	0.08607335	nonDEG	-0.1532752	0.71830448	1
-0.401363	0.30108545	0.88559219	nonDEG	-1.5592164	0.00135332	0.26570141

0.1117024	0.82809803	1	nonDEG	0.81008454	0.11622023	1
-1.1491742	0.01109307	0.15750507	nonDEG	-1.0459882	0.14324338	1
0.52296848	0.34790432	0.91917773	nonDEG	0.35923677	0.56072647	1
0.75580803	0.06142653	0.45297871	nonDEG	1.56520967	0.00211408	0.35783028
-1.4587067	3.08E-05	0.00174202	DEG	-0.6616743	0.21447806	1
1.28602253	3.97E-05	0.00212716	DEG	0.62106414	0.12174297	1
-0.0696246	0.85142495	1	nonDEG	-0.4371782	0.36925221	1
0.38227597	0.18876669	0.75431819	nonDEG	0.43903045	0.25021048	1
-1.177554	6.76E-05	0.00321029	DEG	-1.1557035	0.00945909	0.74892342
-1.7523187	0.01941434	0.22483827	nonDEG	-0.7611216	0.48384493	1
1.57557825	0.03184025	0.3094616	nonDEG	0.5002171	0.41022813	1
0.08107327	0.8500026	1	nonDEG	0.73973976	0.20359862	1
1.83055899	1.27E-07	1.69E-05	DEG	-0.2710653	0.50598604	1
1.06638571	9.36E-05	0.00419229	DEG	0.3021719	0.43223212	1
-0.5515909	0.02428316	0.26064797	nonDEG	0.12177999	0.75316781	1
-0.6311689	0.02692539	0.27868816	nonDEG	0.26027803	0.54884161	1
1.16283569	8.33E-05	0.00382393	DEG	0.02418212	0.9496993	1
1.89594552	0.02141758	0.23953385	nonDEG	0.60306881	0.52318358	1
1.47746285	8.86E-09	1.78E-06	DEG	0.24692086	0.52237029	1
0.3826801	0.17900844	0.744789	nonDEG	-0.0167653	0.96837643	1
-2.0401805	0.00010321	0.00452799	DEG	-0.4655446	0.52972209	1
0.91993338	0.03813312	0.34416209	nonDEG	-0.0230809	0.96922753	1
1.19958407	0.00057126	0.01770238	DEG	0.16300861	0.6903449	1
-0.4998124	0.10574834	0.59518362	nonDEG	-0.953307	0.0393217	1
-1.2467537	0.02511119	0.26586493	nonDEG	-0.9489989	0.20628926	1
-0.3472945	0.3794123	0.93602044	nonDEG	-0.089855	0.85432674	1
-1.7599868	0.01062346	0.15285633	nonDEG	-2.004927	0.13912851	1
-2.6608582	4.09E-08	6.54E-06	DEG	-0.179118	0.81596711	1
-0.1033887	0.69896069	1	nonDEG	-0.7477768	0.05610509	1
0.99147501	0.00091355	0.02504098	DEG	-0.0534833	0.89919854	1
1.29267956	2.03E-07	2.48E-05	DEG	0.01877202	0.95888117	1
-0.493509	0.23342177	0.81342102	nonDEG	0.90759798	0.06218927	1
-0.8866658	0.00962283	0.14200211	nonDEG	-1.5808877	0.00436471	0.52515749
0.61403721	0.06846285	0.4782814	nonDEG	0.13622811	0.73074868	1
1.44842757	0.01665323	0.20298236	nonDEG	0.25546866	0.71221979	1
0.38145002	0.23016655	0.80924485	nonDEG	0.17262444	0.66459009	1
0.29142378	0.30415522	0.88910693	nonDEG	0.05622632	0.88582651	1
-0.384559	0.70400141	1	nonDEG	0.89275732	0.32981024	1
-0.2961209	0.44918324	0.98329655	nonDEG	-0.7711979	0.12859799	1
-0.2646819	0.28516617	0.86735764	nonDEG	-0.0399523	0.92021096	1
-0.3595913	0.43447039	0.973123	nonDEG	0.91889328	0.04068476	1
0.16512508	0.78600717	1	nonDEG	1.03689391	0.08339807	1
0.58799017	0.05414058	0.42120234	nonDEG	0.56195008	0.18917573	1

-0.5653042	0.2151825	0.79434276	nonDEG	-0.6373652	0.32769627	1
0.24858077	0.35060509	0.91917773	nonDEG	0.72368607	0.05009115	1
-0.4059797	0.09677693	0.56798563	nonDEG	0.16349046	0.71285739	1
1.10054765	0.00020237	0.00773713	DEG	0.01270124	0.97320655	1
1.22652609	0.01107312	0.15740303	nonDEG	0.49261277	0.34574694	1
1.82461898	0.0080272	0.12509943	nonDEG	0.14796875	0.83724612	1
-0.5241903	0.21853284	0.79677948	nonDEG	-1.281682	0.02746616	1
1.47308776	0.00089607	0.02460777	DEG	-0.8077435	0.07967352	1
0.89315704	0.04489693	0.37936877	nonDEG	0.10746278	0.7883505	1
-1.0534822	2.85E-05	0.00163467	DEG	0.1589564	0.73832987	1
-0.4356372	0.16617937	0.71943447	nonDEG	-0.5156025	0.24843965	1
0.96689229	0.01207273	0.1662923	nonDEG	0.05462011	0.90750292	1
-1.6318633	7.06E-10	1.91E-07	DEG	-0.491068	0.36275213	1
0.93597657	0.00198378	0.04566103	DEG	0.30253943	0.43413938	1
-0.2604594	0.29148188	0.8736995	nonDEG	-0.2234607	0.55624151	1
1.41719021	0.00123246	0.03171537	DEG	0.00883676	0.99032577	1
0.33032602	0.20198718	0.77400024	nonDEG	0.51847637	0.14987817	1
-0.7067942	0.0263363	0.27465365	nonDEG	-0.1794709	0.68547123	1
-0.3705965	0.13115298	0.65469153	nonDEG	0.0285866	0.93857649	1
-0.307524	0.27404467	0.8576791	nonDEG	-0.3405609	0.38713331	1
1.19490351	6.28E-05	0.00302168	DEG	0.15194164	0.68221202	1
0.21354598	0.40829588	0.95767847	nonDEG	0.38681197	0.32469217	1
0.94778627	0.00032982	0.01161447	DEG	0.24124023	0.51950381	1
-0.7082108	0.00816263	0.1262735	nonDEG	-0.8285639	0.03989021	1
1.12959505	0.00012785	0.00539216	DEG	1.46155471	0.03150002	1
0.4856992	0.0563316	0.43207778	nonDEG	0.40185914	0.27141164	1
-0.247337	0.73929215	1	nonDEG	1.62569053	0.05582025	1
-1.8592415	0.00027439	0.00997272	DEG	-0.3611634	0.63962983	1
1.29221815	1.11E-06	0.00010912	DEG	0.27971215	0.54893202	1
-1.0720432	0.00077633	0.02226206	DEG	-0.5745912	0.20151748	1
-1.1956625	0.00439238	0.08142781	nonDEG	-0.2800992	0.61738147	1
0.27068336	0.2744615	0.85810027	nonDEG	0.38818106	0.28329683	1
1.0991901	7.29E-05	0.00341662	DEG	0.42096543	0.38635784	1
0.16371194	0.59379807	1	nonDEG	0.57942144	0.16852577	1
0.88955897	0.00465225	0.08433342	nonDEG	0.25999287	0.52611251	1
5.72856454	0.01616231	0.19933068	nonDEG	0.19744846	1	1
0.81616243	0.0013958	0.03476581	DEG	-0.0980824	0.80867325	1
0.48101948	0.46218663	0.98479636	nonDEG	0.81939464	0.27663027	1
-0.3227215	0.21851129	0.79677948	nonDEG	-0.1141807	0.75824466	1
1.25981644	0.000247	0.00907785	DEG	0.07224927	0.85320843	1
0.58711241	0.04539054	0.38135631	nonDEG	0.10690609	0.80408502	1
-0.1611756	0.51225327	1	nonDEG	-0.5181167	0.16060369	1
0.07352739	0.84150926	1	nonDEG	0.5596097	0.20815561	1

-0.4828317	0.04945304	0.40183493	nonDEG	-0.0525435	0.90427052	1
-0.152741	0.53832456	1	nonDEG	0.38287154	0.43250643	1
-1.924287	0.02242826	0.24770166	nonDEG	-0.5108248	0.70553737	1
0.56418601	0.04117548	0.36044158	nonDEG	0.16153286	0.70545252	1
-0.7524142	0.01109635	0.15750507	nonDEG	-0.0541721	0.91106471	1
-1.2349775	0.07976997	0.51759934	nonDEG	-1.1222095	0.22240246	1
-1.5209128	0.0014646	0.03614369	DEG	0.49600813	0.4706414	1
-0.4260472	0.08288505	0.52678171	nonDEG	-0.0297497	0.94038493	1
-0.5225815	0.07074516	0.48654695	nonDEG	-0.8997812	0.04996153	1
1.50212914	6.50E-07	6.74E-05	DEG	0.31641399	0.42527257	1
0.45495405	0.07966969	0.51740481	nonDEG	0.23315022	0.52975014	1
-2.9233341	9.24E-08	1.29E-05	DEG	-2.111644	0.05265988	1
0.67156747	0.04090357	0.35894269	nonDEG	0.65486781	0.12930972	1
-0.2904133	0.45471424	0.98394385	nonDEG	-1.3742363	0.00561884	0.58916916
-0.5472156	0.08246916	0.52515887	nonDEG	-0.808131	0.09692773	1
-0.3080254	0.32590782	0.91019829	nonDEG	-0.6465645	0.21297016	1
-0.4656314	0.46982694	0.98843959	nonDEG	1.14503212	0.06421473	1
0.7459165	0.01120006	0.15813839	nonDEG	0.15134791	0.701153	1
-0.6233905	0.1283647	0.6509782	nonDEG	0.02691552	0.9671793	1
0.40789706	0.11577931	0.62174885	nonDEG	0.55040765	0.14028005	1
-0.6677806	0.0084434	0.1291919	nonDEG	0.25481703	0.60261307	1
-0.1705193	0.49158593	0.99972374	nonDEG	-0.1117124	0.7660869	1
0.35421345	0.19721683	0.76873993	nonDEG	0.2951374	0.44394574	1
-0.9097335	0.10979792	0.60530729	nonDEG	-0.6302959	0.49277194	1
-0.700208	0.00787358	0.12335836	nonDEG	-0.6014879	0.15686317	1
-2.1168594	0.00015406	0.00618731	DEG	0.66739632	0.36371935	1
0.76331075	0.0380178	0.34369589	nonDEG	0.30528025	0.46512644	1
0.78989269	0.00343803	0.06858326	nonDEG	0.8218947	0.02867934	1
-1.5809964	0.00203201	0.04651287	DEG	-0.3156094	0.63043678	1
-0.4939467	0.08555191	0.5352955	nonDEG	-0.3228876	0.42291115	1
-1.1985133	4.21E-05	0.00222294	DEG	-0.4430154	0.31644654	1
-0.6163282	0.10499268	0.59282029	nonDEG	-1.0669695	0.02648273	1
0.50490539	0.05158258	0.41110063	nonDEG	0.5438842	0.14023384	1
-1.1467565	0.00375815	0.07346681	nonDEG	-0.980876	0.07796649	1
-0.5348988	0.18544697	0.75310345	nonDEG	-0.1860529	0.72936497	1
1.47790927	0.00808642	0.12568962	nonDEG	-0.0204123	0.97880255	1
-1.0316904	4.88E-05	0.00247271	DEG	-0.7147485	0.1007019	1
-0.1728109	0.61360257	1	nonDEG	-0.5484762	0.24069344	1
0.6109919	0.02801067	0.2859244	nonDEG	0.8891196	0.01733224	0.95092622
0.75044308	0.15636132	0.70062224	nonDEG	1.3513671	0.00502389	0.56449699
0.63579734	0.01548895	0.19419894	nonDEG	-0.3359487	0.41244158	1
0.66168937	0.0460811	0.38572122	nonDEG	0.42848604	0.35679326	1
0.43737998	0.09180343	0.55224625	nonDEG	0.2632988	0.58924869	1

0.32464153	0.30325229	0.88798156	nonDEG		0.71514452	0.06192227	1
1.00723412	0.02428606	0.26064797	nonDEG		0.22833116	0.70270894	1
0.23172228	0.34909913	0.91917773	nonDEG		0.61989676	0.0911693	1
-0.7936063	0.07151699	0.48814946	nonDEG		-1.5827304	0.01193936	0.81648099
0.15043223	0.54091409	1	nonDEG		0.22347877	0.54198055	1
1.1771761	5.04E-05	0.00254263	DEG		1.22995408	0.00770186	0.67884783
-0.9676743	0.09762754	0.57010527	nonDEG		-0.1759579	0.82918702	1
-0.2965509	0.25843727	0.83909237	nonDEG		-0.4535947	0.24349373	1
2.22783229	0.0005526	0.01723297	DEG		-0.7048735	0.28016729	1
-0.3584544	0.16004284	0.70893014	nonDEG		-0.2987525	0.4321862	1
-0.9288142	0.00017011	0.00671278	DEG		-0.6719662	0.1466099	1
2.26186031	0.14989833	0.68725272	nonDEG		1.18313754	0.29630127	1
-0.7003651	0.00651712	0.1069439	nonDEG		-0.1285015	0.79473948	1
0.20031745	0.65300924	1	nonDEG		0.81419093	0.11390764	1
-0.103526	0.78561522	1	nonDEG		-0.2403711	0.60346244	1
0.32818272	0.25646448	0.83849359	nonDEG		0.3385665	0.44858828	1
0.33907243	0.22062135	0.80037926	nonDEG		0.19101807	0.60496588	1
0.2361022	0.44271105	0.97896571	nonDEG		-0.0820446	0.83734555	1
1.41963609	0.01443229	0.1856454	nonDEG		0.6121504	0.31747226	1
-0.970669	0.00111212	0.02930048	DEG		-0.2269209	0.60574045	1
0.88033857	0.08550488	0.5352955	nonDEG		0.1930606	0.72913108	1
-1.1571349	4.51E-05	0.00233123	DEG		-0.6440641	0.26412274	1
-0.31187	0.25355143	0.83493792	nonDEG		-0.8766296	0.0246718	1
-3.607762	3.07E-15	2.83E-12	DEG		-0.06283	0.97091962	1
-1.2307923	0.16110708	0.71096258	nonDEG		0.3804334	0.65267712	1
-1.5689262	0.00181669	0.04268269	DEG		-0.5814814	0.45227801	1
1.45398532	2.54E-05	0.00148578	DEG		0.00150028	0.99901324	1
-2.0425011	0.00088131	0.02438441	DEG		0.11987962	0.88147323	1
1.01611771	0.00055225	0.01723297	DEG		0.60508095	0.21658479	1
0.59500819	0.02280531	0.249764	nonDEG		0.09653523	0.80344045	1
0.11465905	0.86607798	1	nonDEG		1.33719576	0.02739367	1
1.82381922	0.001806	0.04256761	DEG		0.16743703	0.8080089	1
-0.8478072	0.05329217	0.41825335	nonDEG		-0.7678038	0.26168799	1
1.20171542	2.48E-05	0.00146252	DEG		1.00217777	0.08504529	1
1.42516871	0.11015246	0.60578707	nonDEG		0.25799554	0.76913553	1
-0.6660896	0.03612439	0.33442328	nonDEG		-0.4866746	0.2645026	1
-2.240349	5.04E-08	7.82E-06	DEG		0.4544684	0.49959193	1
1.5039159	0.24762169	0.82907898	nonDEG		1.07335367	0.28301555	1
0.87847131	0.00068507	0.0202791	DEG		0.34669301	0.34320117	1
1.06345266	0.08797328	0.54226242	nonDEG		0.67242906	0.28608852	1
-0.1517731	0.71894259	1	nonDEG		1.2812226	0.00284916	0.43013559
-2.1889995	0.01681437	0.20420722	nonDEG		-0.563269	0.54821413	1
-0.9259997	0.00155095	0.03776545	DEG		-0.3352858	0.42492413	1

-0.6088687	0.03459798	0.32595095	nonDEG	-0.388985	0.34371351	1
-0.9295447	0.11819509	0.62833119	nonDEG	-0.089653	0.91507566	1
-0.2838728	0.2694242	0.85230023	nonDEG	-0.3665352	0.32322122	1
0.47792577	0.06462964	0.46487057	nonDEG	0.42510579	0.25626839	1
-0.5550147	0.29519061	0.87920183	nonDEG	2.42497258	0.00160871	0.29928568
0.79410209	0.01551942	0.19433276	nonDEG	0.23565891	0.55542182	1
0.32724237	0.48426559	0.99623334	nonDEG	0.32512831	0.59773297	1
1.49970905	3.61E-05	0.0019793	DEG	-0.5445823	0.20261843	1
0.91520413	0.00395181	0.07536309	nonDEG	-0.0194535	0.96404217	1
0.68950237	0.13364545	0.65907076	nonDEG	0.01444049	0.98297459	1
-1.7028676	0.00114291	0.02990755	DEG	-0.6668152	0.38647346	1
0.84174334	0.00132311	0.03346634	DEG	0.24138047	0.51565446	1
0.34271045	0.21918984	0.79840753	nonDEG	0.6158855	0.21111696	1
1.82219118	6.20E-08	9.21E-06	DEG	0.42034483	0.25198623	1
-0.3609551	0.18144273	0.74717253	nonDEG	-0.0174203	0.96352817	1
-0.9082732	0.02580876	0.27125456	nonDEG	-1.1624064	0.10390729	1
-0.2464392	0.77587066	1	nonDEG	2.24686667	0.01874118	0.95785021
-0.6136476	0.1100068	0.60566492	nonDEG	-0.5399164	0.34116517	1
-1.8297095	0.05123156	0.40931023	nonDEG	-2.9254187	0.03786296	1
1.18822468	0.01022162	0.14816062	nonDEG	0.12673089	0.79952338	1
0.74443479	0.00380382	0.07391463	nonDEG	0.24074646	0.51063038	1
1.19893452	0.04434409	0.37573954	nonDEG	0.70772005	0.32678503	1
2.08053781	3.18E-10	9.56E-08	DEG	0.04148631	0.93566326	1
-1.3595279	0.02204999	0.24449326	nonDEG	-0.4585657	0.57706538	1
-0.7418583	0.29803482	0.88321393	nonDEG	0.9999104	0.1887655	1
-0.7638641	0.00259011	0.05525383	nonDEG	-0.0655972	0.86230688	1
0.52709114	0.35477584	0.91917773	nonDEG	1.01568303	0.07809946	1
-0.5564488	0.04115578	0.36037628	nonDEG	-0.3864365	0.31584183	1
-0.9012127	0.03482279	0.32720971	nonDEG	0.2557836	0.64585853	1
-1.3085786	2.67E-05	0.00154917	DEG	-1.9202379	9.37E-05	0.04219605
0.25832092	0.29084252	0.87302174	nonDEG	0.18786112	0.64208864	1
-1.8750303	5.74E-08	8.62E-06	DEG	-0.0682599	0.89521945	1
-0.2758881	0.2854581	0.8676924	nonDEG	-0.0429437	0.9066653	1
1.05355508	0.00024275	0.00896664	DEG	0.65557819	0.09550893	1
0.2000711	0.83853302	1	nonDEG	1.21464954	0.21746776	1
-0.3820066	0.6998398	1	nonDEG	1.67438555	0.1196941	1
1.16426754	0.08538526	0.53504228	nonDEG	0.49467145	0.47620768	1
0.70740194	0.10791614	0.60137462	nonDEG	0.78150403	0.17800668	1
-0.23973	0.41486637	0.96053724	nonDEG	-0.4384242	0.2971264	1
-0.6416922	0.04631074	0.38704295	nonDEG	-1.0558919	0.02616821	1
-0.7006617	0.06059979	0.44914327	nonDEG	0.08324735	0.84641729	1
-0.8220576	0.0678343	0.47541505	nonDEG	-0.2319441	0.67669137	1
0.78366386	0.14143613	0.67177078	nonDEG	0.63627359	0.27755632	1

-0.9382063	0.00514356	0.09008894	nonDEG	-0.0648571	0.89625686	1
-0.9841155	0.00079858	0.02264884	DEG	-0.3893136	0.39471816	1
-2.3038955	0.02115887	0.23738411	nonDEG	-0.3662821	0.80128034	1
-0.4729602	0.09157504	0.55209451	nonDEG	-0.2391042	0.56000078	1
-0.8671104	0.0081637	0.1262735	nonDEG	0.61933839	0.17234992	1
0.51629078	0.24472418	0.82649442	nonDEG	0.61067597	0.22738926	1
1.24373238	2.52E-05	0.00148	DEG	0.0572794	0.88283427	1
0.48168872	0.45715169	0.98426953	nonDEG	1.46358385	0.11720788	1
0.36007412	0.59920118	1	nonDEG	0.86693302	0.19046172	1
-1.2783879	0.03832653	0.34525539	nonDEG	-1.032206	0.18638949	1
0.55040257	0.06328207	0.46101478	nonDEG	0.12224399	0.74698463	1
-0.8135255	0.16121371	0.71111334	nonDEG	1.11352879	0.03171361	1
-0.4393612	0.09643951	0.56679634	nonDEG	-0.7889901	0.05950984	1
0.1818405	0.72069929	1	nonDEG	0.98422158	0.04577422	1
-0.5161002	0.08245436	0.52515887	nonDEG	-0.3493276	0.39697046	1
0.37684845	0.13340252	0.65907076	nonDEG	0.64089806	0.08248796	1
-0.2562557	0.6761133	1	nonDEG	-0.943949	0.18982594	1
-0.4932526	0.06375447	0.46209743	nonDEG	-0.1309777	0.74049353	1
0.50417265	0.58532224	1	nonDEG	0.67150185	0.41850435	1
-0.1642443	0.62984199	1	nonDEG	-0.5608558	0.22070615	1
-0.7118951	0.0169332	0.20522698	nonDEG	-0.4415303	0.24733688	1
-0.3987702	0.14574991	0.68029504	nonDEG	-0.1562971	0.67628676	1
-0.3235095	0.23631357	0.81830447	nonDEG	-0.103104	0.80119882	1
-0.469184	0.11224766	0.61193682	nonDEG	-0.7995492	0.07448203	1
1.41343372	0.00307888	0.06320723	nonDEG	0.92093377	0.14193238	1
-0.3424756	0.47921716	0.99332737	nonDEG	0.02614219	0.97199996	1
0.95854986	0.00037384	0.01279718	DEG	0.41488541	0.28789891	1
-1.0352234	0.06731025	0.47405418	nonDEG	-1.3025095	0.09605989	1
-0.4782771	0.04696755	0.3901947	nonDEG	0.08179652	0.83727624	1
0.48975032	0.06476872	0.46550937	nonDEG	0.49038566	0.18003772	1
-0.339369	0.2096603	0.7858594	nonDEG	-0.3202433	0.41942726	1
1.67718101	0.1504434	0.68725272	nonDEG	1.47543807	0.08556279	1
1.05086896	0.0002965	0.0105929	DEG	0.14096262	0.71308474	1
-0.4072392	0.51869666	1	nonDEG	1.51938143	0.03119074	1
-0.4484222	0.08714018	0.53984	nonDEG	-0.2924519	0.45645653	1
0.03671577	0.88572299	1	nonDEG	0.00689443	0.98756077	1
-0.049363	0.9509191	1	nonDEG	0.4357628	0.53498275	1
0.23028632	0.39050506	0.94686517	nonDEG	0.36201192	0.33270191	1
1.46020862	0.02017214	0.23036264	nonDEG	0.73564301	0.25254453	1
0.5956074	0.0161181	0.19924305	nonDEG	0.15503882	0.66738159	1
0.84245351	0.06252896	0.45768029	nonDEG	0.47307206	0.33810097	1
0.67101434	0.23329185	0.81342102	nonDEG	0.33605211	0.52579144	1
0.98041712	0.00054902	0.0171761	DEG	-0.0813442	0.8369286	1

1.35356011	0.00015913	0.00636004	DEG	-0.0471566	0.91064537	1
0.39071468	0.12975006	0.65346088	nonDEG	0.13512735	0.71923959	1
-0.879021	0.00925738	0.1379477	nonDEG	-0.3420735	0.47333788	1
-1.7007241	0.00024993	0.00916261	DEG	-0.6448559	0.35520417	1
2.7643397	0.1323828	0.65711072	nonDEG	0.91558092	0.51222035	1
1.1766406	3.99E-05	0.00212911	DEG	-0.0627426	0.86765861	1
0.60246159	0.02757802	0.28268426	nonDEG	1.63E-05	1	1
0.11884674	0.76545013	1	nonDEG	-0.3456653	0.6223957	1
-0.5916055	0.02023939	0.23077141	nonDEG	0.03615339	0.92615594	1
-1.0302684	6.30E-05	0.00302446	DEG	-0.104476	0.85783152	1
0.5037965	0.06803273	0.47595428	nonDEG	0.44994539	0.23706129	1
-0.1500433	0.60500287	1	nonDEG	-0.1971592	0.60942024	1
-0.373411	0.27301404	0.85608852	nonDEG	-1.2214739	0.01005814	0.77109804
-0.3295422	0.47751025	0.99294124	nonDEG	-0.5492456	0.38175836	1
0.04025938	0.92190974	1	nonDEG	0.20480895	0.65597858	1
1.40951337	0.14903911	0.6856099	nonDEG	0.34667763	0.72333489	1
-0.9948979	0.08072008	0.52017157	nonDEG	-0.9046228	0.26226789	1
0.19944792	0.50253628	1	nonDEG	0.557939	0.1437777	1
-0.028483	0.95525549	1	nonDEG	0.29400713	0.58857527	1
-0.2216917	0.72466262	1	nonDEG	0.73532198	0.22517788	1
-0.7907386	0.00226179	0.05000073	nonDEG	-0.1809185	0.64388626	1
3.24351	0.63644416	1	nonDEG	0.94127885	0.83491609	1
-0.3765802	0.1597162	0.7081789	nonDEG	-0.6444921	0.09672109	1
-0.2456998	0.32230716	0.90520806	nonDEG	-0.1682125	0.67796127	1
-0.749741	0.03041788	0.30049396	nonDEG	-0.0013531	0.99880586	1
-0.9917555	9.79E-05	0.00435493	DEG	0.15787478	0.66599319	1
0.9287108	0.00250901	0.05403284	nonDEG	0.0819318	0.85402622	1
-0.8308247	0.08945725	0.54666582	nonDEG	-1.2178957	0.06430906	1
-1.9180815	0.00012885	0.00541871	DEG	0.31471699	0.6363281	1
-1.2102252	0.04190781	0.36396333	nonDEG	0.33238208	0.68236544	1
0.39200621	0.58021723	1	nonDEG	0.29810024	0.68622116	1
1.02601252	0.06710621	0.47329651	nonDEG	0.57188243	0.3348195	1
1.06529515	0.00451447	0.08265026	nonDEG	0.13135709	0.76260149	1
0.29328719	0.38805652	0.94483467	nonDEG	0.84959207	0.04550317	1
0.70980978	0.0046699	0.08444543	nonDEG	0.35404899	0.34671742	1
1.4410764	8.14E-06	0.00059045	DEG	0.04384608	0.91525928	1
0.52915302	0.03749414	0.34173067	nonDEG	0.01513576	0.96879337	1
1.86239929	4.16E-08	6.63E-06	DEG	-0.0801759	0.83755598	1
-0.2607068	0.53588333	1	nonDEG	0.25131981	0.55737951	1
1.60991988	0.03546613	0.33114091	nonDEG	0.90908465	0.21644049	1
-0.0868821	0.77356022	1	nonDEG	-0.9821949	0.02346235	1
-1.2952912	0.01184863	0.16453383	nonDEG	-1.9164335	0.01463031	0.87363447
0.67580975	0.1342975	0.65963131	nonDEG	0.17886267	0.72168846	1

-0.7154942	0.00404752	0.07653744	nonDEG	0.1593166	0.73023579	1
0.13377484	0.60277282	1	nonDEG	-0.251161	0.51880141	1
-0.4306296	0.13434972	0.65963131	nonDEG	0.0937392	0.82627886	1
1.33983011	0.02464499	0.26277575	nonDEG	-0.0494207	0.94876748	1
-0.7599647	0.00421478	0.07903118	nonDEG	-0.6141938	0.16811827	1
-2.879313	7.66E-08	1.11E-05	DEG	-1.5951308	0.03027458	1
-1.7505198	6.07E-06	0.00046745	DEG	0.9118392	0.06631046	1
1.023131	0.002754	0.05774576	nonDEG	-0.7119082	0.09655645	1
-0.3522411	0.16289115	0.714154	nonDEG	-0.2078872	0.63155651	1
1.43327107	0.00112907	0.02960005	DEG	0.72822774	0.12532043	1
-1.1995724	0.00264302	0.05593665	nonDEG	0.06201795	0.92859358	1
0.57874331	0.01727266	0.20797135	nonDEG	0.19866986	0.58111821	1
-0.4621004	0.06345756	0.46158078	nonDEG	-0.1350013	0.71107937	1
1.14334904	7.79E-05	0.00360361	DEG	0.63094385	0.30919064	1
0.6345778	0.16357961	0.714154	nonDEG	0.79777545	0.09774254	1
0.59400749	0.05518679	0.42630384	nonDEG	-0.2434034	0.58188402	1
-1.0684316	0.00027569	0.00999643	DEG	-0.3010993	0.5523952	1
-0.5647891	0.0352787	0.32991415	nonDEG	-0.0680042	0.86616674	1
-1.7100812	0.00375984	0.07346681	nonDEG	-2.3636809	0.00416114	0.52387911
1.84691107	0.0250694	0.26586493	nonDEG	0.08916015	0.93465308	1
-0.508659	0.32046534	0.90348392	nonDEG	0.11901733	0.79694246	1
-0.074863	0.90351387	1	nonDEG	1.2133423	0.05591207	1
0.32362204	0.31743103	0.90027476	nonDEG	0.62448907	0.10155399	1
-1.2722257	0.34557456	0.91869633	nonDEG	-5.2014473	0.08601393	1
1.73693439	0.00036607	0.01261922	DEG	0.40112913	0.45877329	1
-0.8024241	0.02797537	0.28576227	nonDEG	-1.2246467	0.10841499	1
0.70306358	0.00732557	0.11682421	nonDEG	0.18292061	0.62467493	1
-0.611983	0.03742383	0.3415284	nonDEG	0.01578669	0.96961913	1
1.26785064	0.01242262	0.16851066	nonDEG	-1.6603153	0.02432017	1
-0.8693103	0.02957772	0.29606795	nonDEG	-1.3463481	0.02957249	1
1.75597795	5.52E-10	1.53E-07	DEG	0.17249531	0.63944843	1
-0.7181518	0.00377363	0.07361955	nonDEG	0.01200276	0.97433318	1
0.68100418	0.10143921	0.58087317	nonDEG	0.00955758	0.98616603	1
0.98217248	0.00211443	0.04788692	DEG	-0.1300799	0.7380489	1
-0.4748849	0.49826637	1	nonDEG	-0.8343112	0.42790004	1
-0.4234646	0.10631372	0.5973983	nonDEG	-0.4759561	0.20947274	1
-0.9549491	0.03429577	0.32375505	nonDEG	-0.2129985	0.72546319	1
0.60877017	0.01739407	0.20892042	nonDEG	0.13610046	0.7255032	1
-0.5113787	0.04458675	0.3771808	nonDEG	-0.070646	0.8557502	1
0.39503967	0.16956504	0.72663669	nonDEG	0.20780642	0.63530929	1
1.15294537	0.00208648	0.04739497	DEG	0.77522642	0.07927906	1
-1.3961739	2.18E-07	2.64E-05	DEG	0.36875874	0.53178077	1
-0.312157	0.21410206	0.79262458	nonDEG	-0.1559516	0.68723698	1

2.05327171	0.00636559	0.1053382	nonDEG	-0.9509689	0.14986812	1
-0.7648934	0.25088366	0.83275514	nonDEG	1.96577812	3.04E-05	0.0208153
-0.6976712	0.21958087	0.79884354	nonDEG	-4.2972061	1.20E-05	0.01005366
-1.997793	3.84E-07	4.24E-05	DEG	-0.7203972	0.24014548	1
0.0723765	0.91971476	1	nonDEG	0.04540843	0.969215	1
-1.2895441	0.12858928	0.65114404	nonDEG	1.73401031	0.00427904	0.52515749
-0.9647658	0.00304708	0.06277316	nonDEG	0.11329968	0.8577756	1
0.51582091	0.26676277	0.85072996	nonDEG	0.96182686	0.05149294	1
0.42747557	0.18771253	0.75310345	nonDEG	0.75200143	0.14591284	1
0.30065008	0.31654045	0.89944774	nonDEG	0.52969104	0.18257273	1
-1.246167	7.07E-05	0.00333194	DEG	-1.0274742	0.02522366	1
-2.6212023	2.09E-05	0.00129328	DEG	-0.6708207	0.52283156	1
-0.698282	0.20211633	0.77400024	nonDEG	-1.3100924	0.05964293	1
-0.4833996	0.23770314	0.81948793	nonDEG	-1.7889401	0.00133522	0.26570141
-0.7325416	0.03687815	0.3384214	nonDEG	-0.2251622	0.6400054	1
0.68819433	0.00556467	0.09557656	nonDEG	0.5030841	0.15945477	1
0.9324261	0.00043729	0.01455921	DEG	0.31726434	0.38834925	1
-0.5738998	0.01688148	0.20476882	nonDEG	0.01979736	0.96236169	1
-0.672035	0.29977702	0.88467981	nonDEG	1.43211219	0.01293824	0.82982334
0.82228952	0.04666216	0.38892621	nonDEG	0.02167235	0.97232761	1
-1.0274559	0.00077904	0.02226206	DEG	-0.3950874	0.46428029	1
-2.1922887	0.00324131	0.06576221	nonDEG	-0.0860197	0.94168234	1
-0.778721	0.02808446	0.286479	nonDEG	-0.5212272	0.28125949	1
-0.3100044	0.31549803	0.89842779	nonDEG	-0.6025249	0.22735811	1
-0.1191573	0.71101112	1	nonDEG	0.60152855	0.12885684	1
1.00586955	0.00019375	0.00747562	DEG	0.24639065	0.51151717	1
-2.8589679	3.41E-07	3.81E-05	DEG	-0.8366139	0.39980748	1
0.15360128	0.70163349	1	nonDEG	0.71989974	0.09085984	1
-0.476046	0.52666753	1	nonDEG	-2.3883004	0.00750223	0.67511746
-1.03335	0.01885808	0.22082856	nonDEG	-1.9641788	0.00297535	0.4301506
-0.5001611	0.04904201	0.40026276	nonDEG	-0.1964186	0.70241785	1
-0.6479848	0.01182229	0.16432316	nonDEG	0.17978617	0.6687557	1
-0.6303858	0.22373502	0.80443197	nonDEG	-0.3565181	0.54637136	1
1.23187284	0.001291	0.03287702	DEG	0.59616303	0.19046493	1
0.61910683	0.06039216	0.44807419	nonDEG	0.57759082	0.15935906	1
0.69794709	0.00641603	0.10587533	nonDEG	0.64491403	0.08801478	1
0.52413121	0.04418607	0.3753022	nonDEG	0.1681064	0.64483436	1
-0.1792189	0.84891026	1	nonDEG	1.80451266	0.07209334	1
-0.1129617	0.66623449	1	nonDEG	-0.3760325	0.33321625	1
-0.8382919	0.05281209	0.4164841	nonDEG	-1.1254049	0.02301611	1
1.44939094	2.21E-08	3.84E-06	DEG	0.07514739	0.89427493	1
-1.2980531	0.00016514	0.00654296	DEG	-0.3146843	0.52673616	1
-1.9770399	1.03E-05	0.00072033	DEG	-1.1829275	0.19546001	1

0.77879742	0.29147811	0.8736995	nonDEG	0.51394031	0.49899455	1
-0.0041214	0.99251332	1	nonDEG	-0.4278462	0.57514326	1
-1.2929433	0.07080993	0.48654695	nonDEG	-1.525125	0.07698739	1
-0.3056829	0.29668232	0.88169097	nonDEG	-0.3258066	0.41408979	1
-0.9464233	0.00380907	0.07396788	nonDEG	0.08457763	0.86982253	1
0.77004604	0.02173102	0.24178361	nonDEG	0.45111609	0.24687924	1
0.85892385	0.27208028	0.85514137	nonDEG	1.17551885	0.17254485	1
0.58634715	0.02502218	0.26564298	nonDEG	0.0562528	0.87962967	1
0.82556466	0.60396401	1	nonDEG	0.94148974	0.43490556	1
0.65726944	0.00924393	0.13792813	nonDEG	0.06095003	0.86714871	1
0.95282482	0.24067954	0.82233109	nonDEG	0.13891718	0.87740037	1
-1.5221071	1.36E-06	0.00012874	DEG	-0.7536688	0.09024656	1
-0.66766	0.05822552	0.4394004	nonDEG	1.02366106	0.17277091	1
1.73778162	5.86E-05	0.00285823	DEG	0.90847211	0.18111135	1
0.75927214	0.0028905	0.06005192	nonDEG	-0.015766	0.96750872	1
0.47777126	0.128426	0.65114404	nonDEG	0.21128313	0.58967853	1
-1.2032064	0.0005227	0.0165567	DEG	-0.3381449	0.59436465	1
1.9467889	6.89E-05	0.0032607	DEG	0.03408323	0.94173647	1
-0.541522	0.34886078	0.91917773	nonDEG	-0.5854499	0.64667378	1
0.55297454	0.04276939	0.36783172	nonDEG	0.40933447	0.30039106	1
0.56187645	0.03265921	0.31399559	nonDEG	0.15041289	0.68913065	1
-0.5330463	0.05100168	0.4084663	nonDEG	-0.507527	0.23152058	1
-0.7972329	0.04128815	0.36089128	nonDEG	-0.3925899	0.42374383	1
-1.2946055	4.47E-05	0.00232725	DEG	-1.6846148	0.00656612	0.63795339
0.19860043	0.65628312	1	nonDEG	-1.9076608	0.00133773	0.26570141
-1.3126744	0.00232623	0.05091593	nonDEG	-1.4143698	0.05770792	1
0.9347992	0.00248444	0.05369993	nonDEG	0.13744941	0.73623418	1
0.4002558	0.11777491	0.62754309	nonDEG	0.31022717	0.39866373	1
-1.274625	0.11989729	0.63063362	nonDEG	-1.3578286	0.22344359	1
-0.3035045	0.23436302	0.81505173	nonDEG	-1.244826	0.00344176	0.47346762
-0.8055945	0.00396771	0.07540696	nonDEG	0.05456933	0.89610665	1
-2.0293701	0.00044852	0.01480263	DEG	0.69801341	0.38429256	1
-1.9694727	6.70E-05	0.00319037	DEG	-0.5569665	0.38649974	1
-1.0423715	0.04948435	0.40190631	nonDEG	-0.8768413	0.28858663	1
0.39485914	0.12707391	0.64755605	nonDEG	0.44974606	0.27233906	1
-0.6994824	0.04062328	0.35805492	nonDEG	0.60760908	0.2447988	1
-1.0832625	0.00021524	0.00813392	DEG	-0.6217239	0.14414505	1
-1.6512378	2.67E-05	0.00154917	DEG	-0.3224207	0.57942909	1
-1.5962964	0.11167364	0.61073012	nonDEG	0.86914813	0.44745335	1
-1.2403098	0.00382269	0.07413456	nonDEG	-0.3854277	0.51536021	1
1.32766174	0.0214349	0.23953385	nonDEG	-1.5092489	0.07907912	1
0.48111082	0.38452223	0.94134613	nonDEG	1.19803116	0.04245041	1
-0.7313917	0.01219603	0.16738414	nonDEG	-1.0386335	0.00963342	0.75026005

0.44023939	0.4015071	0.95305194	nonDEG	0.23014001	0.69913431	1
0.59036219	0.03974663	0.35318476	nonDEG	0.58166208	0.11520053	1
-1.1890361	0.04007742	0.35451899	nonDEG	-2.0086913	0.0141299	0.8605661
2.3083524	0.02862432	0.28997634	nonDEG	1.91715843	0.05083782	1
0.60778884	0.27019259	0.85276106	nonDEG	0.91041643	0.08654977	1
-0.7764709	0.00381896	0.07411094	nonDEG	-0.0725113	0.85239823	1
0.51904692	0.03982716	0.35368681	nonDEG	0.49549288	0.19547898	1
0.22773146	0.46866645	0.9871869	nonDEG	0.79307845	0.0334432	1
6.741679	0.00305211	0.06278084	nonDEG	-0.343272	0.81106732	1
0.43409517	0.34605579	0.91869633	nonDEG	0.28000217	0.60945293	1
-0.7394629	0.00392877	0.07524988	nonDEG	-0.5844123	0.16585885	1
-0.5439921	0.03988776	0.35376105	nonDEG	-0.2561828	0.50009117	1
-0.4137363	0.29997769	0.88467981	nonDEG	-0.7974064	0.16237274	1
-1.502136	0.00046405	0.01516221	DEG	0.02272181	0.97283935	1
1.93678106	0.01723833	0.20781563	nonDEG	1.05562979	0.15909116	1
-1.4838073	0.00311693	0.06376611	nonDEG	-2.135961	0.00205683	0.35618226
-0.414519	0.1651271	0.71751683	nonDEG	-0.4136073	0.33886059	1
-0.9385919	0.29774199	0.88261266	nonDEG	-1.6504228	0.16094118	1
0.35276262	0.41507352	0.96059195	nonDEG	0.28232338	0.56005785	1
-0.2045837	0.43309814	0.97242402	nonDEG	-0.1279229	0.76341067	1
-0.6223381	0.01269459	0.17060133	nonDEG	0.0480212	0.90784409	1
0.98090367	8.63E-05	0.00393785	DEG	-0.6744972	0.08662362	1
0.18951673	0.7234431	1	nonDEG	-0.6259977	0.32683275	1
-1.0755384	0.00024049	0.00889408	DEG	-0.479686	0.44158185	1
1.21379388	2.13E-06	0.00019036	DEG	-0.1439042	0.69472896	1
0.3531397	0.23996113	0.82187354	nonDEG	0.37289089	0.32109703	1
0.52292002	0.0992221	0.57396016	nonDEG	0.63830574	0.11576772	1
-0.5695017	0.36140715	0.92354131	nonDEG	-0.0592675	0.96616091	1
0.61679997	0.03715106	0.34008606	nonDEG	0.36556178	0.34077751	1
1.2357515	9.72E-05	0.00432786	DEG	-0.0646681	0.88704013	1
-0.1507421	0.84638237	1	nonDEG	1.79039379	0.03308946	1
-1.0049036	0.00803519	0.12515764	nonDEG	0.27547973	0.58232362	1
-2.1764213	1.11E-08	2.13E-06	DEG	-0.8441622	0.10207349	1
1.02304097	0.01902442	0.22206973	nonDEG	0.59661407	0.20462179	1
-0.8108721	0.00706719	0.11356501	nonDEG	-1.4623842	0.00451631	0.53611112
-0.8613456	0.00311511	0.06376611	nonDEG	-0.4468701	0.22060777	1
-0.3500365	0.41401649	0.95994306	nonDEG	0.4205747	0.63682457	1
-0.3620272	0.19618832	0.76744373	nonDEG	-0.571885	0.12310918	1
-0.0472942	0.89279197	1	nonDEG	-1.0551926	0.01783385	0.95781535
0.15515953	0.5488942	1	nonDEG	0.47550879	0.18910013	1
0.64921534	0.02845858	0.28879424	nonDEG	0.73437757	0.05540485	1
-0.3215211	0.2102249	0.78699211	nonDEG	-0.2139876	0.56338392	1
-0.7037746	0.01330382	0.17562828	nonDEG	-1.4248284	0.0114305	0.8038671

-0.2471723	0.57056248	1	nonDEG	-1.1603397	0.04502958	1
-1.2974036	1.12E-05	0.00076056	DEG	-0.3808579	0.34789984	1
-0.2722469	0.29918285	0.88421282	nonDEG	0.30645017	0.51144126	1
0.68932362	0.01479178	0.18837366	nonDEG	0.0792616	0.84464782	1
0.69891243	0.07075686	0.48654695	nonDEG	0.27158107	0.54881377	1
-0.6339284	0.00881093	0.13349723	nonDEG	-0.0404396	0.91426696	1
-0.6698675	0.03506379	0.32853056	nonDEG	-0.0165014	0.97400654	1
-0.8067236	0.10761278	0.60057113	nonDEG	0.0414203	0.95223278	1
0.39136727	0.53767491	1	nonDEG	-2.4208083	0.00294463	0.4301506
-0.5861344	0.07615233	0.50539204	nonDEG	-0.2018363	0.65380552	1
0.00235429	0.99700222	1	nonDEG	1.05611236	0.00602133	0.60914768
-0.7148101	0.23366895	0.81388785	nonDEG	1.30027963	0.00869269	0.72219089
-1.3251016	0.06897076	0.4805752	nonDEG	-1.7408988	0.0865087	1
-1.0508434	0.005624	0.09648305	nonDEG	-0.521606	0.2644755	1
1.26020298	0.00012361	0.0052739	DEG	0.54493317	0.21502296	1
-0.6784328	0.08497812	0.53408856	nonDEG	-0.0904252	0.85848302	1
1.57505882	1.04E-07	1.43E-05	DEG	-0.1826588	0.63135005	1
0.67321265	0.30462439	0.88957713	nonDEG	0.38070432	0.57647227	1
1.20999018	0.00045924	0.01508089	DEG	-0.0106561	0.97983035	1
-1.0425172	0.00566953	0.09675665	nonDEG	-0.0717299	0.89517545	1
-3.0150579	0.00066738	0.01991436	DEG	-0.818399	0.62364998	1
-0.9546637	0.08848824	0.54395602	nonDEG	0.05108121	0.96410915	1
-0.3489623	0.1827792	0.74983791	nonDEG	-0.0468628	0.9000989	1
-0.0913881	0.80105997	1	nonDEG	-1.4148809	0.01163801	0.80926302
0.02806843	0.9091648	1	nonDEG	-0.2961752	0.41780657	1
-0.9239851	0.00028236	0.01018759	DEG	-0.7390922	0.04129035	1
-0.4848286	0.08686492	0.53964509	nonDEG	-0.4150821	0.36062265	1
0.57455331	0.2094566	0.78572136	nonDEG	0.67067541	0.16151611	1
2.10710507	0.02848147	0.28892692	nonDEG	0.05690959	0.96047612	1
0.97109951	0.00064828	0.01953412	DEG	0.05904056	0.87619191	1
-1.1701251	0.1201435	0.63091411	nonDEG	-1.0848623	0.38162133	1
-1.2711209	0.01890893	0.22116012	nonDEG	-1.3236368	0.07720648	1
1.90312076	0.00883829	0.13370518	nonDEG	0.22669112	0.76895602	1
0.84910343	0.0981467	0.57137375	nonDEG	-0.0578466	0.92285507	1
-0.5215796	0.03645347	0.33599056	nonDEG	0.05714854	0.89426647	1
-1.3952742	8.03E-05	0.00369835	DEG	-0.9680913	0.04094063	1
-0.0664126	0.93884855	1	nonDEG	1.6547467	0.07455045	1
0.9735175	0.01511288	0.19127607	nonDEG	0.2879818	0.49138026	1
-1.2438524	0.00873841	0.1328085	nonDEG	-1.083445	0.11369676	1
0.47330421	0.61213907	1	nonDEG	1.06069349	0.18493961	1
1.0190973	0.09601938	0.56558685	nonDEG	0.69166987	0.27934074	1
0.71571513	0.00395882	0.07536309	nonDEG	0.06221907	0.86954546	1
-2.435046	0.00172638	0.0411856	DEG	-0.4232697	0.66763062	1

1.31522406	0.06394653	0.46254672	nonDEG		0.16726694	0.82145279	1
1.13481818	8.64E-06	0.00061914	DEG		-0.4033896	0.28380617	1
-2.1250458	3.85E-12	1.92E-09	DEG		-0.4524452	0.48932334	1
-0.4042153	0.09970972	0.57443019	nonDEG		-0.0229672	0.95620923	1
-1.5593761	1.38E-06	0.00012979	DEG		0.60868449	0.33528693	1
1.67536265	1.99E-05	0.00123992	DEG		0.328283	0.45079852	1
-0.3683406	0.15003443	0.68725272	nonDEG		-0.2027532	0.60381481	1
-0.0790912	0.77332195	1	nonDEG		0.36424121	0.31373823	1
0.76219414	0.02760627	0.28280435	nonDEG		0.52922568	0.24576795	1
-1.3995189	0.04111756	0.3602077	nonDEG		-0.2667533	0.79274381	1
-0.138346	0.66193586	1	nonDEG		-0.2849962	0.52081214	1
-0.325185	0.52252451	1	nonDEG		-0.6966582	0.2387573	1
-0.5903369	0.07255695	0.4914105	nonDEG		0.14913112	0.74326933	1
1.27668438	0.00395546	0.07536309	nonDEG		0.58560518	0.21790442	1
-0.8565568	0.01374216	0.17932424	nonDEG		-0.0668424	0.88896384	1
-3.2700168	2.24E-06	0.00019851	DEG		-1.7962299	0.06603757	1
-0.9553529	0.00023104	0.00859883	DEG		0.01610584	0.96839225	1
-0.6793606	0.04333627	0.37086528	nonDEG		0.23172616	0.6125334	1
0.39841835	0.23873407	0.82081872	nonDEG		0.07948157	0.85067726	1
1.54101388	0.00183165	0.04291478	DEG		-0.4179744	0.46905417	1
-0.5605894	0.10786255	0.6012811	nonDEG		0.74365263	0.10734365	1
0.32324281	0.30334733	0.88798156	nonDEG		0.50931962	0.16396724	1
-1.9328089	1.28E-12	7.23E-10	DEG		-0.6727136	0.11411742	1
-0.4426535	0.09590291	0.56544878	nonDEG		-0.2570275	0.49239334	1
0.58600214	0.08643785	0.53862182	nonDEG		0.17584617	0.66921161	1
-0.1655072	0.65545246	1	nonDEG		-0.1803931	0.71596614	1
0.37447736	0.16679098	0.72133975	nonDEG		0.36147181	0.42977944	1
-0.5300424	0.11693946	0.62512815	nonDEG		-0.548027	0.32639355	1
-0.3282516	0.18663683	0.75310345	nonDEG		-0.0867322	0.81463607	1
0.31841498	0.30590549	0.89022852	nonDEG		0.53178816	0.18367981	1
-0.2831572	0.26539063	0.85033031	nonDEG		-0.3179255	0.39439031	1
-1.2625843	0.12285507	0.63899831	nonDEG		-1.5748934	0.16551987	1
-0.8379597	0.04525229	0.38062344	nonDEG		-0.4578816	0.43317183	1
-1.7699263	1.46E-07	1.88E-05	DEG		-0.3170886	0.52681486	1
-0.8534375	0.0067382	0.10953391	nonDEG		-0.2347443	0.62963429	1
0.04766569	0.88178582	1	nonDEG		0.52227084	0.20642821	1
0.18505243	0.44563685	0.98041193	nonDEG		0.44849229	0.21123723	1
-1.5050646	0.00496753	0.08804277	nonDEG		-0.1155372	0.90449156	1
-0.5768435	0.03596499	0.33336689	nonDEG		-0.1474659	0.73446822	1
-0.7594835	0.30778295	0.89198881	nonDEG		-0.00886	1	1
-1.2413442	0.01650475	0.20152774	nonDEG		-2.2684656	0.00216359	0.35783028
0.73701497	0.00915211	0.13739368	nonDEG		0.27849554	0.44394726	1
-0.4792498	0.10548101	0.59420043	nonDEG		-0.1841747	0.65785783	1

-2.1144546	5.42E-05	0.00270607	DEG	0.24262004	0.73941405	1
0.39963176	0.14970567	0.68679836	nonDEG	0.05049994	0.91194666	1
1.52105148	7.39E-09	1.51E-06	DEG	-0.2904159	0.44609614	1
0.95935609	0.00010204	0.00449691	DEG	0.06136695	0.86896895	1
1.34175361	0.08292106	0.52689685	nonDEG	-0.3636801	0.67862035	1
1.19987999	0.00022623	0.00847329	DEG	0.0269557	0.9509803	1
1.55466816	2.61E-08	4.39E-06	DEG	0.4374654	0.22150989	1
1.32681299	1.16E-06	0.00011422	DEG	0.64549515	0.30379834	1
-0.8245347	0.17732139	0.74306988	nonDEG	-2.5271083	0.00213904	0.35783028
-0.8828449	0.06291955	0.4592831	nonDEG	-1.2774417	0.08913701	1
0.60891479	0.01637643	0.20079369	nonDEG	0.49256962	0.19437521	1
0.6817507	0.24758083	0.82907898	nonDEG	-1.2351425	0.06713317	1
-1.4934111	1.20E-06	0.00011706	DEG	-1.1385726	0.03701319	1
-0.1972431	0.53016214	1	nonDEG	-0.5135561	0.20704812	1
-0.5615025	0.03575636	0.33237491	nonDEG	0.16759743	0.65612714	1
1.20143742	0.14456641	0.67768958	nonDEG	0.54709443	0.49446165	1
-1.8502172	0.01170021	0.16332035	nonDEG	0.21043498	0.85252993	1
1.24454238	0.00023974	0.00888873	DEG	0.34698639	0.36721121	1
0.65259379	0.32159752	0.90433751	nonDEG	0.68295461	0.37267251	1
1.89132154	0.00420239	0.07889932	nonDEG	-0.2580963	0.69182404	1
-0.0344017	0.96562292	1	nonDEG	0.74246091	0.21565076	1
0.40150852	0.34342199	0.91869633	nonDEG	0.31114169	0.52602373	1
-1.0004442	0.05297899	0.41712982	nonDEG	-0.9865484	0.16805089	1
-0.3135519	0.63763253	1	nonDEG	0.03561347	0.97422875	1
0.42663959	0.1140093	0.61692363	nonDEG	0.37699355	0.31737625	1
2.41747415	2.47E-05	0.00146252	DEG	0.77563084	0.17070059	1
-0.5627826	0.25773876	0.83898637	nonDEG	-0.4483881	0.51544867	1
0.33799427	0.39099781	0.94686517	nonDEG	0.83435611	0.06073834	1
0.44716019	0.24724714	0.8287958	nonDEG	-2.1703836	3.83E-05	0.02349846
0.64370545	0.06344274	0.46158078	nonDEG	-0.6900099	0.17365041	1
0.96587046	0.03838537	0.34546833	nonDEG	0.60587442	0.26752594	1
-0.1142753	0.82113525	1	nonDEG	1.18134535	0.0074112	0.67339017
-0.5836505	0.05759347	0.43653298	nonDEG	-0.5678948	0.17350512	1
0.90265977	0.12758667	0.64875626	nonDEG	0.15329231	0.81425266	1
-1.2003358	0.09998283	0.57533122	nonDEG	1.63993656	0.05101174	1
0.97312633	0.0003473	0.01211612	DEG	-0.0386136	0.91954086	1
-0.1043419	0.90460711	1	nonDEG	0.59676667	0.5088822	1
3.77299569	0.45196081	0.98329655	nonDEG	-0.3400994	1	1
1.48618849	0.00181279	0.0426592	DEG	0.22008708	0.66002873	1
0.31763762	0.60666924	1	nonDEG	1.4175764	0.01372401	0.84878381
0.39507822	0.66842916	1	nonDEG	1.09340723	0.22343854	1
1.3013197	4.21E-07	4.50E-05	DEG	0.09890181	0.78686842	1
-0.5110246	0.04933839	0.40134646	nonDEG	-0.2229296	0.53973883	1

0.40807833	0.12109046	0.63328868	nonDEG	0.09312536	0.81740306	1
-0.2967894	0.21772584	0.79602968	nonDEG	-0.237347	0.50823561	1
0.46737833	0.0644847	0.46426144	nonDEG	0.31632856	0.39060742	1
0.81362771	0.00257577	0.05499211	nonDEG	-0.3870989	0.31585015	1
-0.2954664	0.24448957	0.82644718	nonDEG	-0.4378711	0.23583931	1
-1.1222585	0.04186339	0.36385126	nonDEG	-0.5367181	0.53541222	1
0.21677334	0.3985411	0.95155323	nonDEG	0.37279828	0.38275749	1
-0.4911135	0.09320959	0.55676945	nonDEG	-0.7652278	0.10593791	1
0.50090756	0.0489701	0.39989762	nonDEG	-0.0211052	0.95789321	1
2.72693257	0.01937332	0.22445152	nonDEG	0.64971281	0.46032415	1
-0.4890235	0.34631811	0.91869633	nonDEG	-1.6982701	0.01356678	0.84878381
-0.3134694	0.19545924	0.76634006	nonDEG	-0.2172118	0.565249	1
-0.0407295	0.90205421	1	nonDEG	-0.1856595	0.6717571	1
0.21268672	0.88451756	1	nonDEG	0.614333	0.55678074	1
1.39197384	0.05508677	0.42575868	nonDEG	-0.4185217	0.58563817	1
-0.9774008	0.00014366	0.00589829	DEG	0.00860149	0.98517812	1
6.05338608	0.0081972	0.12656241	nonDEG	-0.5060188	0.67810103	1
0.77331095	0.00960925	0.1419401	nonDEG	0.68495988	0.0678735	1
0.35398741	0.16688597	0.72153858	nonDEG	0.0528127	0.8959058	1
1.03694781	0.00014465	0.0059267	DEG	0.25863392	0.47608425	1
0.42051948	0.20169336	0.77353451	nonDEG	0.40219727	0.31359363	1
1.40537594	4.15E-05	0.00219391	DEG	-0.1380423	0.77171055	1
0.35809553	0.20594182	0.7803135	nonDEG	0.49446016	0.1973436	1
-0.3962383	0.43114353	0.97178962	nonDEG	-1.1676552	0.0578637	1
-3.3034869	0.02302042	0.25127846	nonDEG	4.11458264	6.83E-05	0.03589312
-0.409681	0.56132737	1	nonDEG	0.67438834	0.27233064	1
0.50867405	0.16671987	0.72113815	nonDEG	0.4763186	0.24225164	1
-2.0295393	9.21E-07	9.25E-05	DEG	-1.3554614	0.04409935	1
-0.390724	0.14800205	0.68406337	nonDEG	-0.3235736	0.39669969	1
-0.7735973	0.05277119	0.41647526	nonDEG	-0.5643711	0.26784119	1
-0.0656872	0.79617173	1	nonDEG	-0.4155244	0.31697229	1
-0.3821998	0.21722042	0.79507048	nonDEG	-0.5707327	0.19090734	1
1.29554317	1.04E-05	0.00072786	DEG	0.59367168	0.34121304	1
-0.6820515	0.2257345	0.80685891	nonDEG	1.69105678	0.00015796	0.05961907
-0.6318547	0.04891871	0.39958879	nonDEG	-0.4684776	0.29930427	1
-0.6898514	0.00599352	0.10088236	nonDEG	0.02619983	0.94731876	1
-0.6056365	0.01454801	0.18628913	nonDEG	-0.1791349	0.63768252	1
1.41423825	1.95E-05	0.00121993	DEG	0.39606182	0.49159375	1
-0.2824313	0.30000685	0.88467981	nonDEG	-0.4184612	0.28551264	1
0.68248945	0.00695339	0.11241121	nonDEG	0.28144826	0.43744179	1
-0.9154611	0.00143826	0.0356406	DEG	-0.1861887	0.63009299	1
0.73009501	0.04338494	0.37106599	nonDEG	0.34254922	0.47296086	1
-0.3800478	0.17117935	0.72989601	nonDEG	-0.2254462	0.5661999	1

0.40659507	0.1123994	0.61242381	nonDEG	0.45468134	0.20550536	1
-2.6607496	8.61E-10	2.24E-07	DEG	-0.422462	0.49594812	1
-1.3436705	0.00063616	0.01922795	DEG	-1.0398796	0.09288984	1
-0.3352056	0.30776909	0.89198881	nonDEG	-0.4044928	0.34159449	1
-0.4828596	0.05292752	0.41684651	nonDEG	-0.0989104	0.78490794	1
-0.766962	0.00273402	0.05740858	nonDEG	0.06227611	0.87922565	1
0.39172021	0.43755344	0.97423703	nonDEG	0.58358687	0.20742656	1
0.54241685	0.15493386	0.69666993	nonDEG	0.73090841	0.07856858	1
0.89348248	0.00259467	0.05531104	nonDEG	-0.3145694	0.40631339	1
-0.7939483	0.05278268	0.41647526	nonDEG	-0.2923693	0.56713832	1
-0.4466019	0.06709857	0.47329651	nonDEG	-0.321528	0.39459167	1
-1.2444332	0.00391691	0.07513256	nonDEG	-1.9482207	0.00566237	0.58916916
0.33264151	0.19101387	0.75851897	nonDEG	0.31969734	0.37899897	1
-0.2994401	0.57868311	1	nonDEG	-0.1008445	0.89329882	1
-0.1674851	0.67380497	1	nonDEG	-0.5876111	0.25692287	1
0.58602363	0.36200333	0.92354131	nonDEG	0.78745252	0.25880572	1
1.30741774	5.38E-06	0.00042561	DEG	0.40209302	0.52365102	1
-1.9138584	3.40E-09	7.55E-07	DEG	-0.1484826	0.819942	1
0.50994196	0.4941054	1	nonDEG	0.46757163	0.54145586	1
-0.0519002	0.93605502	1	nonDEG	0.27072981	0.65916068	1
-1.3433278	0.02123214	0.23793408	nonDEG	-0.0003055	1	1
0.96617389	0.0013184	0.03343021	DEG	0.24704151	0.51446927	1
-0.9186958	0.16462973	0.71577825	nonDEG	2.85001089	6.29E-06	0.00686876
0.47331747	0.22864893	0.80924485	nonDEG	0.43474272	0.40258055	1
1.19165986	0.00021352	0.00808978	DEG	-0.2707414	0.47384197	1
-0.7721782	0.34556856	0.91869633	nonDEG	-0.8962474	0.39442105	1
1.29794871	0.00015092	0.00610294	DEG	0.71087759	0.10872648	1
-0.3991571	0.13050633	0.65446487	nonDEG	0.74049588	0.05033617	1
-0.9238775	0.00124831	0.03202195	DEG	-1.188534	0.00534441	0.57843438
0.13604948	0.59109177	1	nonDEG	-0.1173835	0.79568686	1
-0.1061452	0.69561676	1	nonDEG	0.09066438	0.8554375	1
-3.6458243	2.70E-22	7.24E-19	DEG	-0.0059721	1	1
-0.4470677	0.15674936	0.70140512	nonDEG	-0.5531645	0.20375589	1
0.58466539	0.23548808	0.81691546	nonDEG	0.79186809	0.10121203	1
-0.8899919	0.00952073	0.14091538	nonDEG	-0.1288986	0.79236551	1
-0.3304653	0.20823415	0.7838989	nonDEG	-0.6183003	0.10824576	1
-0.1487543	0.58936455	1	nonDEG	-0.1406411	0.74075584	1
-0.4457097	0.53260457	1	nonDEG	1.72689003	0.00438835	0.52515749
0.00448922	0.9912863	1	nonDEG	-0.9940158	0.03785491	1
0.27116227	0.57347271	1	nonDEG	0.54003665	0.31262851	1
-0.7960652	0.26573723	0.85062515	nonDEG	-2.0926657	0.07379122	1
-1.9564093	0.00021008	0.00800069	DEG	-0.657177	0.38713424	1
-0.4765954	0.22513161	0.80629737	nonDEG	-1.0263232	0.06361398	1

-1.8508508	0.00022059	0.0083041	DEG	-0.6582524	0.33823469	1
1.36574909	0.02237649	0.24727538	nonDEG	0.28742879	0.62697871	1
0.60362176	0.06005262	0.4468115	nonDEG	0.7108389	0.08149451	1
0.33258272	0.29091525	0.8730913	nonDEG	0.76533282	0.04017518	1
0.35089249	0.21830661	0.79677948	nonDEG	0.32922228	0.37763727	1
-1.4960119	0.00066765	0.01991436	DEG	-0.0408473	0.9556881	1
-0.2119808	0.6601537	1	nonDEG	0.83939256	0.15516476	1
-0.4050192	0.22939371	0.80924485	nonDEG	-0.5905249	0.21245677	1
0.43893627	0.08254531	0.52530293	nonDEG	0.2664579	0.52782844	1
-0.8180062	0.06447183	0.46426144	nonDEG	0.1724179	0.75522146	1
-0.8590958	0.06364128	0.46191708	nonDEG	-0.2503362	0.65480277	1
0.8869921	0.00105032	0.02795701	DEG	-0.1090691	0.78751488	1
0.87002214	0.03658169	0.33706675	nonDEG	0.82696813	0.16234768	1
-0.7567456	0.02808001	0.286479	nonDEG	-0.1499903	0.71747034	1
-1.262421	0.00139947	0.03482597	DEG	0.22690219	0.61599575	1
-0.9146033	0.018926	0.22127189	nonDEG	-0.6373348	0.22704501	1
1.35223284	8.09E-07	8.24E-05	DEG	0.24820848	0.50537021	1
1.14982021	0.00058329	0.01801833	DEG	0.45082672	0.30193194	1
0.88108162	0.13804288	0.6657076	nonDEG	0.13666324	0.84204116	1
0.73755489	0.04433689	0.37573954	nonDEG	0.12701314	0.76298797	1
-1.0212661	0.01090462	0.15583543	nonDEG	-1.1754253	0.03827522	1
-2.6564637	2.74E-09	6.25E-07	DEG	-1.8852366	0.01187936	0.81648099
-0.3079256	0.55043006	1	nonDEG	0.98447169	0.03983291	1
0.63294468	0.13456555	0.66012324	nonDEG	0.31295833	0.50706047	1
-0.0865616	0.88161968	1	nonDEG	-2.41808	0.00096749	0.21777569
0.84942584	0.04675687	0.3891647	nonDEG	0.40785386	0.42040992	1
0.72020579	0.06721344	0.47371232	nonDEG	0.70329078	0.10406387	1
-0.4949684	0.24073794	0.82235835	nonDEG	-0.7939384	0.17528005	1
1.46542516	2.50E-06	0.00021834	DEG	0.12514852	0.74775596	1
0.47880476	0.08071364	0.52017157	nonDEG	0.4773108	0.31450454	1
-0.2863714	0.50703493	1	nonDEG	0.35041149	0.50400358	1
1.22553	0.04257772	0.36671901	nonDEG	0.60886767	0.3273486	1
-0.5506323	0.36994669	0.92862044	nonDEG	1.16926735	0.05015012	1
0.88130224	0.37304629	0.93171372	nonDEG	0.39013096	0.69049339	1
-0.299113	0.51317328	1	nonDEG	1.10263904	0.01801814	0.95785021
-0.2989239	0.22642728	0.80748547	nonDEG	-0.02817	0.93917067	1
0.51824107	0.0718823	0.48967306	nonDEG	0.57023024	0.14895356	1
-1.0343781	0.00344093	0.06858326	nonDEG	0.1014881	0.8362146	1
0.44605656	0.48522119	0.99670526	nonDEG	0.30438554	0.65979367	1
1.17093285	4.46E-06	0.00036102	DEG	-0.1151276	0.76597388	1
0.54385417	0.1583462	0.70512084	nonDEG	0.36851804	0.39391137	1
-0.6768744	0.02198828	0.24410441	nonDEG	-0.3061961	0.45873464	1
1.19247878	0.09449355	0.56077784	nonDEG	-0.0454115	0.95814285	1

-0.5041481	0.07466782	0.50037471	nonDEG	-0.4313289	0.32718443	1
-0.3130922	0.4974712	1	nonDEG	-0.952122	0.10892084	1
0.84414039	0.02345349	0.25501106	nonDEG	0.37430242	0.46308455	1
1.16299108	0.00024039	0.00889408	DEG	0.51159377	0.30820814	1
1.55337023	3.80E-05	0.00205378	DEG	1.13616662	0.17938583	1
0.31808858	0.45674243	0.98426953	nonDEG	0.15825036	0.7417126	1
0.35479931	0.13943338	0.66785296	nonDEG	0.17195918	0.67018058	1
0.8663303	0.00205571	0.0469133	DEG	0.23466179	0.51714716	1
-0.7523001	0.01723855	0.20781563	nonDEG	-0.05674	0.89915661	1
1.54466777	2.36E-07	2.83E-05	DEG	-0.013125	0.9730355	1
-1.4003573	5.84E-05	0.00285735	DEG	0.5351028	0.57713242	1
-0.4240013	0.08216164	0.5242212	nonDEG	-0.3380604	0.36622051	1
0.92692012	0.01287757	0.17239778	nonDEG	0.65524232	0.14269501	1
0.84802885	0.00128899	0.03285429	DEG	-0.0366693	0.92278388	1
-0.0052842	0.98644029	1	nonDEG	-0.3385861	0.43493338	1
-0.8438754	0.0886008	0.54430698	nonDEG	-0.4834774	0.46622694	1
0.25604487	0.79911578	1	nonDEG	0.38837345	0.7072844	1
0.94882508	0.0159549	0.19785015	nonDEG	0.2845327	0.50551407	1
0.31439842	0.21239593	0.79008388	nonDEG	-0.0151259	0.96698911	1
1.58733488	1.13E-05	0.00076816	DEG	0.54699217	0.2601082	1
0.87606773	0.11090559	0.60877133	nonDEG	0.59233941	0.2704451	1
0.61645203	0.02533646	0.26753234	nonDEG	0.73006882	0.04794428	1
-0.5705729	0.1816578	0.74732027	nonDEG	-0.7503551	0.18083323	1
1.54610501	0.00034713	0.01211612	DEG	0.36097835	0.42944448	1
-0.2965408	0.62908847	1	nonDEG	-1.9019405	0.01731783	0.95092622
-0.1650292	0.8131527	1	nonDEG	-2.2903057	0.00718767	0.67120689
0.41342326	0.11477262	0.61909498	nonDEG	0.47247836	0.24051646	1
1.05931845	0.09273343	0.55536636	nonDEG	-0.4426393	0.5169337	1
-0.7067242	0.03464147	0.32611243	nonDEG	-0.2283815	0.64547352	1
0.32617243	0.49088506	0.99946448	nonDEG	0.69282127	0.28855507	1
-1.6616193	3.09E-06	0.00025929	DEG	-1.4350931	0.01020434	0.77777211
0.84222962	0.03411868	0.32289859	nonDEG	0.29007606	0.54137267	1
-0.2582056	0.61457478	1	nonDEG	0.38882772	0.55629883	1
-0.6289883	0.01667215	0.20298236	nonDEG	-0.1032722	0.81750298	1
-0.6745465	0.00814911	0.1262735	nonDEG	-0.0618931	0.88255836	1
0.53408766	0.0314871	0.30724185	nonDEG	0.22842135	0.52765752	1
0.47477322	0.09705786	0.56861423	nonDEG	0.46299904	0.22757893	1
-0.7958785	0.00211464	0.04788692	DEG	0.11094047	0.80644435	1
0.36164436	0.72318518	1	nonDEG	2.18127849	0.0350877	1
-0.6895755	0.00547956	0.09450486	nonDEG	-0.0943289	0.83565782	1
-0.8954611	0.06140467	0.45294217	nonDEG	-0.0867962	0.86053868	1
-0.0505481	0.84505615	1	nonDEG	-0.333623	0.39151433	1
0.45530022	0.07156499	0.48825071	nonDEG	0.21875287	0.5450263	1

-0.7506855	0.02402732	0.25884688	nonDEG	-0.3752489	0.43357424	1
-2.7292178	0.00440453	0.08155025	nonDEG	0.40935856	0.67341972	1
-1.1615466	0.00043818	0.01455921	DEG	-0.6423426	0.14061237	1
0.39843945	0.12043889	0.63156406	nonDEG	0.42590344	0.29302737	1
0.7938847	0.06005751	0.4468115	nonDEG	0.42769561	0.36871894	1
0.36802469	0.67362497	1	nonDEG	1.65401107	0.03273562	1
-3.0800831	0.60637897	1	nonDEG	5.70742827	0.02893454	1
-2.8584214	0.02377715	0.25768009	nonDEG	-4.3117336	0.2861328	1
-1.4268374	7.97E-06	0.00058182	DEG	-0.8102336	0.07977647	1
1.21887216	0.00701813	0.11314713	nonDEG	0.64391628	0.23780183	1
0.20907406	0.41127348	0.95855277	nonDEG	0.61260905	0.09871173	1
0.25113769	0.38164973	0.93796465	nonDEG	0.30572676	0.40984955	1
0.72599986	0.14576759	0.68029504	nonDEG	0.24303747	0.7034864	1
0.42551537	0.26789897	0.85190792	nonDEG	-1.3862555	0.01170777	0.81009283
-0.1190508	0.64025914	1	nonDEG	-0.5289956	0.15433281	1
-0.9519714	0.0695389	0.48286555	nonDEG	-0.3138553	0.64922509	1
1.3472491	0.00072689	0.02114517	DEG	-0.002275	0.99758605	1
0.99950436	0.0349878	0.3281318	nonDEG	0.24293049	0.66654241	1
-0.9520191	0.00066621	0.01991436	DEG	-0.2751899	0.53505346	1
-0.7109425	0.13791609	0.66530676	nonDEG	-0.7386295	0.3023439	1
0.45187457	0.15621855	0.70060637	nonDEG	0.17049107	0.66887701	1
0.13065594	0.85150254	1	nonDEG	0.19124453	0.79902959	1
-1.1296126	0.19043426	0.75718453	nonDEG	-0.6241081	0.64205747	1
-2.2582631	2.92E-12	1.48E-09	DEG	0.24847829	0.61207604	1
0.23298913	0.56182543	1	nonDEG	0.37100014	0.44881135	1
-1.1263227	0.00013384	0.00559692	DEG	-0.5224973	0.20376569	1
-0.4861306	0.07691822	0.50793676	nonDEG	-0.3884211	0.33909345	1
-2.2260098	0.00051807	0.01645256	DEG	-0.6922672	0.44902681	1
-0.7015068	0.06395218	0.46254672	nonDEG	-0.0623372	0.90085774	1
-1.291512	0.01556966	0.19463065	nonDEG	-0.3539558	0.5946351	1
-2.1771473	0.00237972	0.05185525	nonDEG	-0.3191501	0.73678166	1
0.73171194	0.13247761	0.65712017	nonDEG	0.33808467	0.50748523	1
-0.397582	0.10267062	0.58462675	nonDEG	0.19339861	0.67773481	1
-1.2427638	1.94E-05	0.0012142	DEG	-1.2055507	0.03400973	1
-0.4164613	0.32119259	0.90430629	nonDEG	-0.9755293	0.0538237	1
-0.2089106	0.44574608	0.98052148	nonDEG	-0.0762968	0.86049292	1
-0.6927013	0.13574816	0.66261751	nonDEG	-1.2521262	0.10691741	1
-0.3042056	0.36017566	0.92312867	nonDEG	-0.9059469	0.05314497	1
-1.8162963	0.02050695	0.23282069	nonDEG	-1.8058435	0.18696165	1
-0.5802137	0.07672994	0.50755195	nonDEG	-0.2150772	0.66759677	1
0.6203755	0.04218917	0.36486691	nonDEG	-0.024151	0.95421928	1
1.76576593	0.00018998	0.00734944	DEG	0.70579329	0.22417741	1
-1.0584503	0.01210113	0.16646771	nonDEG	0.01584013	0.98251858	1

-0.4478336	0.09265418	0.55530262	nonDEG	0.01029152	0.97837997	1
2.38840783	0.00078041	0.02226206	DEG	-3.6989876	1.42E-06	0.00321073
-1.2257003	0.07211503	0.49010687	nonDEG	-1.1215308	0.16704695	1
-0.7465289	0.05362911	0.41969541	nonDEG	0.65423049	0.15898512	1
-2.861491	0.01145963	0.16095422	nonDEG	-3.0311426	0.14001091	1
-0.4501921	0.23775814	0.81948793	nonDEG	-0.7958261	0.09573433	1
0.55918492	0.06599534	0.4693976	nonDEG	0.14486643	0.69721255	1
-0.7118516	0.00860456	0.13104478	nonDEG	-0.2089593	0.60775047	1
-0.2056559	0.77375277	1	nonDEG	-1.839028	0.03327354	1
0.2466498	0.63667668	1	nonDEG	0.69233301	0.25701388	1
-0.3769966	0.19260965	0.76192362	nonDEG	-0.4948934	0.29084804	1
-1.7123153	1.94E-05	0.0012142	DEG	-0.0925907	0.89155087	1
-0.4947985	0.3964093	0.9493779	nonDEG	1.35998891	0.00760162	0.67731627
-0.1625837	0.71690806	1	nonDEG	1.23575057	0.01823656	0.95785021
4.96970893	2.62E-28	2.57E-24	DEG	-0.606821	0.41888827	1
1.73649185	0.00018199	0.00712434	DEG	0.00119531	1	1
-1.0325963	0.09768773	0.57010527	nonDEG	0.31391506	0.69749293	1
-1.1191874	3.80E-05	0.00205524	DEG	-0.2431833	0.61458647	1
0.87064809	0.00095174	0.0257995	DEG	0.74736646	0.04066254	1
1.87486344	0.05174743	0.41183903	nonDEG	1.85433644	0.06576887	1
-0.4801933	0.14307843	0.67562441	nonDEG	-0.9605771	0.05952889	1
0.45021319	0.56931243	1	nonDEG	0.35005562	0.6774761	1
0.09433428	0.90497619	1	nonDEG	1.49067621	0.06900046	1
1.14932568	0.00012701	0.00537204	DEG	-0.0596003	0.87473871	1
0.41659082	0.1013455	0.58067538	nonDEG	-0.1289574	0.72294294	1
1.37689785	2.02E-06	0.00018155	DEG	0.50255589	0.24346483	1
1.44768651	1.96E-07	2.45E-05	DEG	0.85579067	0.06319936	1
0.5447747	0.04501906	0.37964291	nonDEG	0.1974314	0.59946305	1
-0.78467	0.01345072	0.17656877	nonDEG	-0.1307812	0.75134639	1
0.88238646	0.09386355	0.55908861	nonDEG	0.63443249	0.32453038	1
0.73221536	0.42146918	0.96396459	nonDEG	0.69261568	0.46439747	1
2.2144477	0.00060329	0.01844211	DEG	-0.2521491	0.68721966	1
0.21137236	0.85257261	1	nonDEG	1.51940893	0.10097386	1
-0.3415948	0.19681566	0.76824603	nonDEG	-0.7665431	0.06705505	1
-0.6041815	0.13156446	0.65533095	nonDEG	-0.4858187	0.38981467	1
-0.9528636	0.24578915	0.82694708	nonDEG	1.90890597	0.00135382	0.26570141
-0.8083173	0.17691079	0.74219628	nonDEG	-1.7016183	0.0374025	1
-0.7251388	0.02794454	0.28564558	nonDEG	-0.2471551	0.69186344	1
1.11638527	0.03078221	0.30287352	nonDEG	0.71662445	0.1782418	1
-0.5790943	0.01921648	0.2233375	nonDEG	-0.2762233	0.4630662	1
-0.7071303	0.01080103	0.15488145	nonDEG	-0.1897293	0.64659839	1
-0.5954666	0.03260901	0.31361541	nonDEG	-0.4658309	0.2772545	1
-1.7044581	0.00149219	0.03654632	DEG	-0.0474437	0.95443769	1

-0.7491746	0.00587647	0.09942375	nonDEG	0.10468598	0.78321826	1
-0.6009876	0.01540036	0.19344122	nonDEG	-0.1339424	0.71418273	1
-1.1133747	0.00147072	0.03621013	DEG	-0.5537988	0.42653223	1
0.47116362	0.45888061	0.98479636	nonDEG	0.62249211	0.39198713	1
-1.6895893	0.00254666	0.05452677	nonDEG	0.07122039	0.9250812	1
-1.7233702	8.13E-09	1.65E-06	DEG	-1.2253751	0.00215058	0.35783028
0.76586179	0.0042484	0.07945919	nonDEG	-0.0504428	0.89911092	1
0.44429577	0.27294	0.8560208	nonDEG	0.72554616	0.10752186	1
0.84041622	0.00312732	0.06388986	nonDEG	-0.3616441	0.45181793	1
0.40801819	0.19740877	0.76896966	nonDEG	0.36119529	0.35098223	1
-0.7747964	0.00216947	0.04856798	DEG	0.29847595	0.42581134	1
0.38562198	0.1186091	0.62902782	nonDEG	0.2021065	0.6056603	1
1.15659695	4.35E-05	0.00228388	DEG	0.12948443	0.75495654	1
1.99003529	0.00073107	0.02124578	DEG	-0.7741101	0.21114432	1
1.15341267	0.00078854	0.02242872	DEG	0.51495672	0.32332496	1
0.42762323	0.14138765	0.67166551	nonDEG	0.59819264	0.11118325	1
-1.0253967	0.02838359	0.28839265	nonDEG	-1.317602	0.03523882	1
-1.4099315	1.16E-07	1.56E-05	DEG	-1.3041391	0.00945437	0.74892342
-0.2149719	0.40612539	0.95607438	nonDEG	-0.072814	0.85995265	1
1.13149538	0.00064899	0.0195353	DEG	0.00968581	0.98287672	1
-0.9578894	0.00075951	0.02194236	DEG	-0.0451289	0.91144434	1
-1.5563343	1.47E-06	0.00013743	DEG	-0.3782778	0.43330235	1
1.25846847	0.01103479	0.15708569	nonDEG	0.57927959	0.21612675	1
-1.5518679	5.13E-08	7.87E-06	DEG	0.1719252	0.74492915	1
-1.5322872	0.06746472	0.47468785	nonDEG	-1.1153934	0.31526967	1
-0.4534285	0.67958531	1	nonDEG	-0.4869608	0.72080243	1
1.09944965	0.17003455	0.72767074	nonDEG	0.70044053	0.28973541	1
0.72641234	0.00329799	0.06670357	nonDEG	0.20754842	0.58535694	1
-1.7172023	1.20E-06	0.00011734	DEG	-0.2286043	0.72131628	1
0.99312564	0.00012744	0.00538284	DEG	0.29175546	0.49610335	1
1.24585874	4.29E-05	0.00225389	DEG	0.78139181	0.13982699	1
0.34795513	0.73855016	1	nonDEG	0.08917553	0.96503651	1
-0.5498479	0.05811986	0.43925074	nonDEG	-0.1537698	0.70703995	1
-1.3145962	0.24344689	0.82556597	nonDEG	-0.7712896	0.58743117	1
-4.0779701	1.18E-12	6.83E-10	DEG	0.16320657	0.86555193	1
0.98047751	0.00088314	0.02440185	DEG	0.29458301	0.47801056	1
-0.5576885	0.08711337	0.53984	nonDEG	0.85498346	0.14825689	1
-0.4033106	0.09626587	0.56624334	nonDEG	0.15494262	0.69955693	1
-1.6420397	0.04093138	0.35903915	nonDEG	-0.5778632	0.58799027	1
-0.1357897	0.73863101	1	nonDEG	2.40880392	0.00054212	0.14642867
-0.2686654	0.54791144	1	nonDEG	0.05833292	0.91309099	1
-1.266024	0.02480109	0.26366198	nonDEG	-0.1116522	0.85832401	1
1.12222581	6.59E-05	0.00314762	DEG	0.21629268	0.56627247	1

-0.0911358	0.76612843	1	nonDEG	0.04405023	0.92135862	1
-0.5861755	0.0419116	0.36396333	nonDEG	0.13211559	0.75111617	1
0.9030703	0.0034581	0.06877616	nonDEG	0.8466267	0.15565059	1
-0.3757879	0.14323045	0.67575918	nonDEG	-0.9246322	0.01339547	0.84624309
1.41624666	0.00013594	0.00566042	DEG	-0.3163291	0.47305337	1
-0.8596509	0.0007046	0.02072213	DEG	-0.8212221	0.0854556	1
0.93229398	0.11298052	0.61422597	nonDEG	0.80201578	0.20091147	1
-0.2816638	0.47956894	0.99338798	nonDEG	-0.9302417	0.06063303	1
-1.0675329	0.11354246	0.61594266	nonDEG	1.14442112	0.08536537	1
-0.9360054	0.01426226	0.18480053	nonDEG	-0.5881912	0.27377602	1
-0.6157907	0.01332091	0.17577515	nonDEG	-0.0841427	0.83573184	1
-1.3079103	3.07E-06	0.00025922	DEG	-0.913141	0.05757391	1
0.57375015	0.02823534	0.28765305	nonDEG	-0.0067783	0.98556881	1
0.52235549	0.08006512	0.5188283	nonDEG	0.09577946	0.80559835	1
0.14896855	0.78145794	1	nonDEG	0.39829861	0.49323559	1
1.23668326	1.02E-05	0.00071753	DEG	0.12231585	0.74766832	1
-0.9338474	0.02720261	0.28043417	nonDEG	-0.4096274	0.56212429	1
0.70436712	0.01052284	0.15200292	nonDEG	-0.21088	0.64906065	1
0.27167316	0.61494795	1	nonDEG	1.43712502	0.03070934	1
-1.6619304	1.36E-07	1.80E-05	DEG	-0.158751	0.88113211	1
0.53388652	0.30518746	0.89006022	nonDEG	0.61303591	0.28484941	1
0.07641546	0.92748087	1	nonDEG	1.15247507	0.17178755	1
-0.5618422	0.10541119	0.59402755	nonDEG	-0.6792336	0.2071384	1
3.7172662	0.03742554	0.3415284	nonDEG	-0.372152	0.83545041	1
1.15909157	0.00029469	0.01054742	DEG	0.18122426	0.65088022	1
1.25180949	2.32E-05	0.00140067	DEG	0.72545186	0.07794391	1
0.98465195	0.0504973	0.40607126	nonDEG	-0.1609017	0.77546857	1
-0.0067987	0.99521972	1	nonDEG	-0.2781317	0.64174673	1
1.24285225	0.01973541	0.22747822	nonDEG	0.14502767	0.8021958	1
0.4788551	0.06422679	0.46365191	nonDEG	0.22017145	0.55328296	1
-2.7743831	0.00462812	0.0841551	nonDEG	-0.0213147	1	1
-0.2840207	0.33743901	0.91869633	nonDEG	-0.956869	0.03201057	1
0.48128575	0.05756122	0.436482	nonDEG	0.69024874	0.08178404	1
0.26640003	0.36246463	0.92354131	nonDEG	0.1435293	0.75193476	1
0.3060477	0.31544745	0.89842779	nonDEG	0.14534312	0.72967985	1
-0.5755534	0.02252087	0.2481555	nonDEG	0.15420544	0.69940585	1
-1.4075847	2.97E-06	0.00025392	DEG	-0.7297929	0.08153061	1
0.38817182	0.30235363	0.88699438	nonDEG	0.84625424	0.06939825	1
1.61121934	4.31E-08	6.82E-06	DEG	0.39470217	0.29308786	1
-1.3924305	0.03674286	0.33787311	nonDEG	-0.1714521	0.83238538	1
0.31455311	0.20744081	0.78320256	nonDEG	0.29634031	0.47304223	1
0.08022956	0.90817641	1	nonDEG	-2.4148544	0.00333841	0.46240087
-0.3158133	0.20768733	0.78320256	nonDEG	0.00701441	0.98594033	1

0.6843374	0.01344925	0.17656877	nonDEG		0.46692477	0.20049293	1
0.49170389	0.10648074	0.59781215	nonDEG		-0.2260081	0.56311571	1
0.83152799	0.04038354	0.35647711	nonDEG		0.39519087	0.38262858	1
1.13266964	4.43E-06	0.00035959	DEG		-0.3300739	0.35829134	1
-1.2921963	1.97E-05	0.00122634	DEG		-0.6285136	0.17606771	1
1.0631735	0.0215043	0.23979741	nonDEG		0.73213434	0.14737936	1
-1.1608672	0.02953159	0.29570764	nonDEG		-0.4636606	0.54931037	1
1.00694606	0.00076052	0.0219499	DEG		0.02459642	0.95107293	1
-0.0584874	0.90276457	1	nonDEG		-1.9918421	0.00364955	0.49008216
-0.0821698	0.85329941	1	nonDEG		-1.1589274	0.03032511	1
0.58461952	0.13319878	0.65869963	nonDEG		0.04320214	0.93649816	1
0.31095471	0.61473778	1	nonDEG		0.64170577	0.32774393	1
-0.6993957	0.00671841	0.10941366	nonDEG		-0.4084469	0.26901213	1
1.25055922	0.0331954	0.31756445	nonDEG		-2.2620012	0.00090076	0.20716769
-0.2132886	0.42789037	0.96958523	nonDEG		-0.5047181	0.16876296	1
0.56876852	0.07556685	0.5034048	nonDEG		0.09643364	0.81258981	1
0.77088169	0.02850803	0.28909672	nonDEG		-0.1340032	0.76661217	1
-0.4896573	0.15808655	0.70450931	nonDEG		-0.3741196	0.41391372	1
0.54174082	0.03388046	0.32150713	nonDEG		0.33788538	0.34763798	1
0.41405318	0.1628595	0.714154	nonDEG		0.19525438	0.60818736	1
1.50790676	4.56E-07	4.81E-05	DEG		0.73145103	0.12588627	1
-0.1082019	0.74653254	1	nonDEG		0.83579756	0.04791848	1
0.95776506	0.02305816	0.25159719	nonDEG		0.29288452	0.4894996	1
-0.5601155	0.30098475	0.8855319	nonDEG		1.05942497	0.02546397	1
1.02926409	0.0006628	0.0198628	DEG		0.43671686	0.269915	1
-0.064009	0.92654611	1	nonDEG		1.1969926	0.04368503	1
-1.3339478	2.02E-07	2.48E-05	DEG		0.46079609	0.39619106	1
-1.059135	0.00031401	0.01118661	DEG		-0.2994311	0.41462219	1
-0.2881123	0.30056379	0.88527236	nonDEG		-0.0731358	0.85322928	1
-1.0299453	0.00807249	0.12560568	nonDEG		0.01086868	0.99036048	1
-2.3958058	1.47E-09	3.66E-07	DEG		0.62878818	0.42165266	1
-0.1329785	0.59765703	1	nonDEG		-0.5833027	0.19459093	1
-0.5250197	0.0720687	0.49007934	nonDEG		-0.1663409	0.71006954	1
0.44433975	0.07695372	0.50793676	nonDEG		0.13500144	0.71719296	1
-0.2054383	0.41441693	0.96032904	nonDEG		-0.2415505	0.50763599	1
0.35817117	0.38198506	0.93796465	nonDEG		0.58278728	0.22803888	1
0.20105404	0.59365023	1	nonDEG		0.49325166	0.25748752	1
2.25121397	6.55E-19	1.29E-15	DEG		-0.0970773	0.78596347	1
0.48881241	0.41610836	0.96166196	nonDEG		1.31260046	0.02796885	1
-0.3189373	0.73460663	1	nonDEG		1.39228635	0.16943204	1
1.55524185	0.00121009	0.03133156	DEG		0.33926421	0.52104396	1
0.6032765	0.02932595	0.29444974	nonDEG		-0.3071001	0.45498499	1
0.46068065	0.05899322	0.44272497	nonDEG		0.09088286	0.81852048	1

-0.1313586	0.61864236	1	nonDEG	-0.4461709	0.23219131	1
-0.0884902	0.74047739	1	nonDEG	-0.2584642	0.49238218	1
0.30068343	0.2581632	0.83898637	nonDEG	0.25520217	0.50459827	1
-1.3859463	9.44E-06	0.0006743	DEG	-0.7474765	0.15476812	1
-0.5960575	0.02511924	0.26586493	nonDEG	-0.1844947	0.67503313	1
-0.2993674	0.24360045	0.8256221	nonDEG	0.07685741	0.84235713	1
1.13438926	2.06E-05	0.00127973	DEG	-0.026878	0.94240665	1
-0.9828191	0.00131294	0.03334907	DEG	-0.2422942	0.71429354	1
-0.7232018	0.03091814	0.3038048	nonDEG	-0.245379	0.59384388	1
-0.280436	0.25288957	0.83393344	nonDEG	-0.1290519	0.72462019	1
-0.4883036	0.06193585	0.45537702	nonDEG	-0.279755	0.47776686	1
1.31863788	2.73E-07	3.17E-05	DEG	0.05029633	0.89382145	1
-0.873868	0.00071468	0.02089326	DEG	-0.4725418	0.23564926	1
0.25428151	0.48564599	0.99670526	nonDEG	-1.3792881	0.00288962	0.4301506
-1.4381089	0.00061677	0.01875737	DEG	-0.5054295	0.4087848	1
-0.5661484	0.14280705	0.67492321	nonDEG	-0.1846568	0.72962254	1
0.49838193	0.04951715	0.40202302	nonDEG	0.48229825	0.18368496	1
0.383432	0.63132972	1	nonDEG	1.29929076	0.07105515	1
-1.2100056	0.00046645	0.01520698	DEG	-0.3816337	0.50707326	1
-0.501499	0.18697506	0.75310345	nonDEG	-0.4122107	0.41759352	1
0.00358892	0.98929287	1	nonDEG	-0.2831899	0.48705504	1
0.53473303	0.04433246	0.37573954	nonDEG	0.4133174	0.32656568	1
0.3563654	0.15905221	0.70668933	nonDEG	0.37055733	0.37870459	1
1.41923929	2.38E-05	0.00142858	DEG	0.63914656	0.1909309	1
0.35969467	0.23924582	0.8208958	nonDEG	0.48359039	0.21093864	1
-0.5184021	0.06970056	0.48325832	nonDEG	-0.6981871	0.082413	1
-0.3736287	0.36155292	0.92354131	nonDEG	-0.545686	0.29227696	1
0.45205236	0.2996067	0.88450756	nonDEG	0.67680155	0.11998675	1
0.57920051	0.211216	0.7877265	nonDEG	0.85948172	0.06548785	1
-2.4449466	9.29E-16	1.01E-12	DEG	-0.9196301	0.05403161	1
-1.5976682	2.39E-08	4.10E-06	DEG	-1.763284	0.00035976	0.11148491
1.00667044	7.56E-05	0.00352024	DEG	0.45363149	0.24136146	1
0.90705145	0.24839758	0.82990493	nonDEG	0.05380987	0.96732919	1
-1.3779905	1.40E-07	1.83E-05	DEG	-0.4125303	0.40336979	1
-0.436739	0.13863391	0.6665824	nonDEG	-0.3698589	0.36165282	1
-0.7936429	0.00232001	0.05091593	nonDEG	-0.3101319	0.44592893	1
1.31699162	1.02E-07	1.41E-05	DEG	0.24276061	0.51791539	1
0.74151786	0.02000405	0.22920012	nonDEG	0.34453898	0.41120288	1
-0.5142889	0.11289653	0.61402178	nonDEG	-0.3428176	0.48425117	1
-2.3211545	0.00552747	0.09515976	nonDEG	1.99323504	0.01993807	0.99822614
-0.4178826	0.12226206	0.63692671	nonDEG	0.12195982	0.73651012	1
1.10698196	1.32E-05	0.00087267	DEG	0.25903529	0.48145208	1
0.5002965	0.11985404	0.63063362	nonDEG	0.44406305	0.28272958	1

-0.1803299	0.51191906	1	nonDEG	-0.0496038	0.90589238	1
0.18378619	0.46010318	0.98479636	nonDEG	0.28775981	0.49053339	1
-0.1467339	0.79734293	1	nonDEG	-2.930685	9.23E-05	0.04219605
0.87014296	0.00231687	0.05090019	nonDEG	-0.0112301	0.97706056	1
-0.9099851	0.00169977	0.04068255	DEG	-0.4986058	0.22544567	1
1.13648997	7.83E-06	0.00057447	DEG	0.38494069	0.28242859	1
-0.3152053	0.26820157	0.85190792	nonDEG	-0.4735233	0.25605914	1
0.7116971	0.09105953	0.55056513	nonDEG	0.95348088	0.0372262	1
0.49364354	0.0655177	0.46766512	nonDEG	0.41905502	0.39132326	1
-0.8292294	0.00506983	0.08921146	nonDEG	-0.1936951	0.66415878	1
0.88349662	0.00339226	0.0679814	nonDEG	0.13629156	0.74049742	1
0.56271481	0.04090671	0.35894269	nonDEG	0.28524046	0.45459371	1
-0.7969282	0.05115674	0.40907961	nonDEG	-0.6490955	0.23619542	1
-1.1666416	0.0050293	0.08876346	nonDEG	-0.0454552	0.94323236	1
1.03413887	0.00417445	0.07844016	nonDEG	0.31772476	0.43640952	1
0.50240274	0.26852322	0.85193834	nonDEG	0.46343652	0.43215569	1
-0.454506	0.06596547	0.4693976	nonDEG	0.07151503	0.86898289	1
0.8868371	0.0111617	0.15805161	nonDEG	0.63098444	0.09422229	1
-0.070584	0.77257936	1	nonDEG	-0.7464221	0.12924703	1
-0.6990729	0.19518227	0.76572106	nonDEG	-0.3161415	0.63858656	1
-2.6017917	2.37E-07	2.83E-05	DEG	-1.0557487	0.19054554	1
-0.8698275	0.05672448	0.43340566	nonDEG	0.08120546	0.89676572	1
-0.0191572	0.98704442	1	nonDEG	-2.3211901	0.042361	1
0.64486221	0.16717664	0.72210453	nonDEG	-0.1095603	0.84328289	1
1.72769688	0.00016253	0.0064642	DEG	-0.8921027	0.06511354	1
2.41495975	1.93E-20	4.74E-17	DEG	-0.8065781	0.03743192	1
0.76146997	0.00408826	0.07719965	nonDEG	0.10796998	0.77107598	1
0.28112567	0.27902128	0.86056653	nonDEG	0.34762773	0.34104202	1
1.57550794	0.03325656	0.31787013	nonDEG	0.02153842	0.97996653	1
-0.3990524	0.20136508	0.77336631	nonDEG	-0.5939692	0.14684692	1
0.67540985	0.02796774	0.28576227	nonDEG	0.5850543	0.12119472	1
0.47279328	0.09689349	0.56837975	nonDEG	0.32850878	0.37090942	1
0.98160428	0.03628965	0.3355268	nonDEG	0.08884373	0.86216334	1
2.21186163	0.00333111	0.06707556	nonDEG	-1.023777	0.24758443	1
1.19322605	3.51E-06	0.00029064	DEG	0.34115916	0.38080276	1
-1.8057593	0.00714853	0.11462182	nonDEG	0.30435605	0.78258415	1
6.86138409	0.00105557	0.02807117	DEG	-0.0337787	1	1
-1.8496777	0.00623656	0.1039039	nonDEG	-1.6005935	0.13734459	1
-0.27527	0.33929212	0.91869633	nonDEG	0.06882739	0.85802334	1
0.45103609	0.18554472	0.75310345	nonDEG	0.43033576	0.26892636	1
-0.9216311	0.03355397	0.31967482	nonDEG	-0.9840699	0.10161607	1
-1.123159	1.14E-05	0.00077015	DEG	-0.2468222	0.56133314	1
-0.1774942	0.49016396	0.99898642	nonDEG	-0.7759511	0.05484732	1

0.60038181	0.02345766	0.25501106	nonDEG	0.44847974	0.22743842	1
0.37409601	0.13614993	0.66318137	nonDEG	0.1705254	0.70850971	1
-0.3698826	0.22084218	0.80037926	nonDEG	-0.627125	0.13023434	1
0.70417062	0.16842125	0.72434671	nonDEG	0.49879772	0.3671303	1
2.07857328	2.32E-06	0.0002045	DEG	-0.9790359	0.05271107	1
-0.374087	0.23985768	0.82176136	nonDEG	-0.1587498	0.73977569	1
-0.6993052	0.08495455	0.53408856	nonDEG	-0.6785508	0.20698057	1
0.3257605	0.27680498	0.85887436	nonDEG	0.17632498	0.66342709	1
0.03844829	0.95402694	1	nonDEG	1.34422032	0.07527805	1
0.19039017	0.65051554	1	nonDEG	1.19464006	0.04325081	1
1.36132755	3.91E-06	0.00032206	DEG	0.7973534	0.03990131	1
-0.6246398	0.16049693	0.70986615	nonDEG	-0.1353686	0.81811027	1
1.31498138	0.0001841	0.00717217	DEG	-0.0142194	0.97344175	1
-2.266702	1.59E-06	0.00014819	DEG	-1.4978199	0.0987942	1
0.26148001	0.3901592	0.94686517	nonDEG	0.00531064	0.99105766	1
1.21473161	4.05E-06	0.00033201	DEG	-0.2027428	0.62769129	1
0.6795673	0.02994494	0.29766155	nonDEG	0.05308331	0.90975837	1
-1.10225	0.00480658	0.08607335	nonDEG	-0.0895156	0.88325415	1
-0.3702885	0.12439141	0.64301293	nonDEG	-0.0080398	0.98494604	1
-0.9794829	0.0894264	0.54666582	nonDEG	0.68048346	0.26317024	1
-0.8561247	0.02378865	0.25768009	nonDEG	-0.287776	0.59878659	1
-1.5023003	1.13E-08	2.14E-06	DEG	-0.5565325	0.19536481	1
0.92947882	0.01846481	0.21726041	nonDEG	0.73478361	0.09025484	1
-1.3042549	0.00054629	0.01712686	DEG	-0.089649	0.86764967	1
0.59399004	0.01824926	0.21593032	nonDEG	0.41735695	0.26352563	1
0.12674199	0.75072019	1	nonDEG	1.36920798	0.00737554	0.67222413
0.41394915	0.10709297	0.59914672	nonDEG	0.59733527	0.15812103	1
1.93365046	0.00028194	0.01018759	DEG	-1.4273376	0.01206598	0.81648099
-0.7714157	0.00925302	0.1379477	nonDEG	-0.4887458	0.20430197	1
0.66493097	0.04601435	0.38527203	nonDEG	0.31816251	0.42847619	1
0.43663671	0.40931461	0.95823562	nonDEG	0.55523961	0.38983368	1
0.9891475	5.62E-05	0.00278039	DEG	0.0633365	0.85945244	1
-0.8505079	0.05020607	0.40571408	nonDEG	-2.1218757	0.00124213	0.25751364
-1.3791936	0.03424578	0.32354346	nonDEG	-3.2769904	0.00145319	0.27900964
0.86948146	0.00168925	0.04049659	DEG	0.30761762	0.4175147	1
-0.7557277	0.0115918	0.16219151	nonDEG	-0.4683095	0.41461129	1
0.59201208	0.03103786	0.3047777	nonDEG	0.56105228	0.12500121	1
1.14143734	0.1074537	0.59991077	nonDEG	0.24090856	0.742805	1
-2.8391721	0.00141236	0.03505776	DEG	-1.9384406	0.11990033	1
1.06535906	0.01770754	0.21177679	nonDEG	-1.1500016	0.04980485	1
1.26932704	1.02E-06	0.00010177	DEG	0.05150025	0.88921151	1
0.74940538	0.05475729	0.42432218	nonDEG	-1.2357587	0.01549841	0.89863185
0.83316373	0.00396028	0.07536309	nonDEG	0.1731061	0.70009671	1

0.72165187	0.03582765	0.33244726	nonDEG	0.26863368	0.53576868	1
-0.6461568	0.013564	0.17755034	nonDEG	-0.0361457	0.92785189	1
0.23464487	0.41512145	0.96059195	nonDEG	0.3017486	0.42288955	1
0.33117328	0.23907528	0.82089086	nonDEG	0.46142283	0.25463058	1
1.63154845	0.00625137	0.10403281	nonDEG	1.16808044	0.17520923	1
2.92382777	0.00212445	0.0479756	DEG	-0.2892937	0.74767437	1
-0.5707227	0.04785872	0.39401443	nonDEG	-0.0449097	0.91107287	1
-0.0121736	0.99189045	1	nonDEG	0.83945223	0.13859369	1
1.09681537	0.00033372	0.01173753	DEG	0.21046909	0.59213012	1
0.48769046	0.44118423	0.97752398	nonDEG	1.00875381	0.12649408	1
1.31656519	0.00985591	0.1445207	nonDEG	0.10870242	0.84469455	1
-2.7381559	2.09E-15	2.05E-12	DEG	0.08187718	0.92566418	1
-0.6168969	0.13502908	0.6610751	nonDEG	-1.5413036	0.01394977	0.8555571
0.64321232	0.01362056	0.17813217	nonDEG	0.22733065	0.56293263	1
-0.6515909	0.01485408	0.1889755	nonDEG	-0.1985838	0.63387799	1
0.77433374	0.00566851	0.09675665	nonDEG	-0.205513	0.66070102	1
-0.1619796	0.57459465	1	nonDEG	-0.1670526	0.69963063	1
0.8813928	0.05410501	0.42113858	nonDEG	0.41637975	0.41469379	1
0.70587691	0.30709487	0.89104731	nonDEG	0.7046107	0.36738857	1
-0.6292715	0.01310168	0.17425207	nonDEG	-0.1055937	0.80263895	1
-1.1048508	0.00197791	0.04565219	DEG	-1.4482988	0.00870878	0.72219089
-1.4790882	0.01021448	0.14813013	nonDEG	0.24797044	0.75403846	1
-2.5245039	0.03789535	0.34316824	nonDEG	1.36981973	0.34674291	1
0.26903558	0.34667891	0.91869633	nonDEG	0.36966116	0.40799118	1
-0.1571687	0.79720892	1	nonDEG	0.70198676	0.25416045	1
-1.5589851	0.00548301	0.09450486	nonDEG	0.5818916	0.40387745	1
2.35452323	0.00047938	0.01544041	DEG	0.27901925	0.78815035	1
0.69820163	0.0259667	0.27252534	nonDEG	0.28105058	0.45579163	1
0.89943768	0.00182451	0.04279823	DEG	0.40370508	0.2693601	1
0.21132355	0.44221201	0.97851624	nonDEG	0.33790744	0.38836868	1
-0.9436879	0.07548318	0.50324091	nonDEG	-0.6435627	0.36655697	1
1.63655516	2.74E-10	9.05E-08	DEG	-0.1007349	0.7897355	1
0.4048392	0.13274314	0.65757885	nonDEG	0.46620847	0.28160056	1
-0.2856329	0.54539849	1	nonDEG	0.96242395	0.07356361	1
1.07437533	0.00016472	0.00653525	DEG	0.10811114	0.77589006	1
-1.5931021	0.00012526	0.00532778	DEG	-0.0234033	0.97688402	1
-0.3647744	0.1621954	0.71309296	nonDEG	-0.5114179	0.16847591	1
-0.5058948	0.05582342	0.42964334	nonDEG	-0.1790808	0.62691006	1
1.02729366	0.00124872	0.03202195	DEG	0.54287335	0.16015787	1
1.00226216	0.00828795	0.12727643	nonDEG	1.19934258	0.05412551	1
-0.4403605	0.17147986	0.72989601	nonDEG	-0.6534672	0.18766542	1
-0.1992425	0.74047472	1	nonDEG	0.56354344	0.33183405	1
-0.26532	0.28056766	0.86186717	nonDEG	-0.1814332	0.63947899	1

0.37961314	0.16950242	0.72663669	nonDEG	0.56548036	0.22428288	1
-0.7445649	0.30809914	0.8922025	nonDEG	-1.4253628	0.09171613	1
1.5535827	9.62E-07	9.63E-05	DEG	-0.0370422	0.92459355	1
0.02533176	0.98485747	1	nonDEG	1.05350985	0.17624571	1
-1.4052178	0.01167528	0.16316879	nonDEG	-0.6443577	0.38559716	1
-0.7507027	0.0090068	0.13576608	nonDEG	-0.2330995	0.56694227	1
-0.697043	0.00564208	0.09663188	nonDEG	-0.2139691	0.56939399	1
0.63059975	0.142218	0.67359585	nonDEG	0.36365258	0.45377958	1
1.0422668	0.04757982	0.39268359	nonDEG	0.29909055	0.61627157	1
1.69528386	0.00461091	0.0839459	nonDEG	-0.4846305	0.41057566	1
0.80128216	0.132844	0.65771855	nonDEG	0.09185068	0.88502065	1
0.57348614	0.31003319	0.89347015	nonDEG	1.52628157	0.00367907	0.49008216
0.77504851	0.01134781	0.15968848	nonDEG	-0.0795356	0.83070511	1
-0.324369	0.41010849	0.95836418	nonDEG	-0.6915	0.17737534	1
0.29099605	0.23875971	0.82081872	nonDEG	0.7174456	0.0532635	1
-0.6687408	0.11152134	0.61039373	nonDEG	0.89076255	0.04658862	1
1.30749894	1.76E-05	0.00112027	DEG	-0.0252015	0.95348855	1
1.06940781	0.08578987	0.53616486	nonDEG	0.28803617	0.66485807	1
-0.9150716	0.00647542	0.1065567	nonDEG	0.23906737	0.61093388	1
-0.8832967	0.11798451	0.6280915	nonDEG	-1.0444617	0.16789446	1
-3.0942106	8.29E-13	5.09E-10	DEG	-1.1688407	0.0841375	1
-1.6672682	1.78E-08	3.19E-06	DEG	-0.4205138	0.50845975	1
-0.4135775	0.48436918	0.99623334	nonDEG	0.89069597	0.15590327	1
-1.1943917	0.02402863	0.25884688	nonDEG	0.24668309	0.71975477	1
-2.4446097	4.11E-05	0.00217663	DEG	-0.4415266	0.65328901	1
0.9571317	0.00014542	0.00593742	DEG	-0.066554	0.8544862	1
0.32566296	0.30610049	0.89026796	nonDEG	0.65428911	0.09484044	1
-0.1121001	0.76133674	1	nonDEG	0.91987199	0.01800996	0.95785021
-0.686835	0.03873062	0.34761915	nonDEG	-0.1974315	0.65343164	1
2.02426509	1.21E-05	0.00081331	DEG	-0.9445591	0.05162118	1
-0.8751624	0.00345939	0.06877616	nonDEG	-0.140466	0.72379074	1
2.1982031	0.01622762	0.19980128	nonDEG	0.10639778	0.91373432	1
0.98362257	0.00534028	0.09269598	nonDEG	0.80625834	0.03020689	1
1.33297514	0.00371574	0.07287659	nonDEG	0.04527399	0.9404721	1
1.53085272	0.05647321	0.43215879	nonDEG	0.08214556	0.93406016	1
0.32119265	0.2884106	0.87024354	nonDEG	0.34689171	0.36682901	1
0.33197682	0.24492976	0.8265359	nonDEG	1.11466624	0.00264716	0.40976087
-0.3268284	0.52973249	1	nonDEG	-1.0278274	0.11488988	1
-0.8969882	0.00260422	0.0554344	nonDEG	-0.3662649	0.4934178	1
-0.2646996	0.28162313	0.86280604	nonDEG	0.09843754	0.78562794	1
1.06217031	0.00021283	0.00807418	DEG	0.16305837	0.69422103	1
1.07418591	0.01915377	0.22295871	nonDEG	0.22945599	0.6429242	1
0.16059019	0.62206251	1	nonDEG	0.4415265	0.27257607	1

-0.3963148	0.11368436	0.61607445	nonDEG	-0.3309463	0.42400801	1
2.39428318	1	1	nonDEG	2.0768733	0.50656904	1
0.76268143	0.0142541	0.18477604	nonDEG	0.74446055	0.1348463	1
1.34405919	0.00285347	0.05945031	nonDEG	0.9403732	0.04038647	1
-0.9903368	0.00171184	0.04087173	DEG	-0.1580397	0.72596767	1
-0.4610222	0.31817872	0.90098849	nonDEG	-1.762109	0.00407942	0.52015192
0.7198567	0.14037286	0.66901406	nonDEG	0.45707139	0.40834753	1
-2.2586804	1.63E-14	1.30E-11	DEG	-0.9041635	0.07129286	1
0.67017905	0.55104388	1	nonDEG	0.94781699	0.38308369	1
-0.0409244	0.90070557	1	nonDEG	-0.241694	0.6239311	1
0.91386654	0.04119464	0.36050205	nonDEG	0.49936624	0.29139225	1
1.69192984	2.11E-08	3.73E-06	DEG	0.13154097	0.73598045	1
0.25845543	0.42450004	0.96646612	nonDEG	0.62323372	0.12845282	1
1.013719	0.0055408	0.09527787	nonDEG	0.30247367	0.48651634	1
1.46846665	0.00211791	0.047924	DEG	0.55917827	0.36429707	1
-1.2509259	0.00302485	0.06244648	nonDEG	-0.5290369	0.38649944	1
0.99518485	0.24315511	0.82526859	nonDEG	1.0336571	0.22215916	1
0.92825197	0.01004996	0.14639322	nonDEG	0.28119492	0.49300613	1
-5.8067872	0.00015042	0.0060995	DEG	-0.655228	0.46846917	1
-0.4810444	0.127596	0.64875626	nonDEG	-0.6074378	0.11798226	1
1.33029684	0.01204766	0.16613903	nonDEG	0.83256565	0.13307781	1
0.07127256	0.8039594	1	nonDEG	0.4795928	0.19679112	1
0.37032155	0.17103923	0.72984836	nonDEG	0.48490135	0.1927102	1
-0.7143033	0.01118758	0.15813746	nonDEG	-0.4805666	0.26801029	1
-0.3126377	0.2228625	0.80321763	nonDEG	-0.1595629	0.67252998	1
-0.1158429	0.71475572	1	nonDEG	-1.0609243	0.01304859	0.83508166
-1.259285	0.19198039	0.76035391	nonDEG	3.09346437	0.000492	0.14184283
0.27899445	0.56452797	1	nonDEG	0.61123118	0.25988408	1
0.28029984	0.57866773	1	nonDEG	0.58940738	0.23436487	1
1.81714563	1.77E-05	0.00112591	DEG	-0.3055934	0.60020488	1
0.55103652	0.55148893	1	nonDEG	0.58645418	0.56529195	1
-0.753102	0.00504692	0.08894993	nonDEG	-0.209203	0.59639402	1
0.83779884	0.00409705	0.0772665	nonDEG	0.30744048	0.41247278	1
-0.7689377	0.16532374	0.71794741	nonDEG	-0.074789	0.92085237	1
-0.3695363	0.24496166	0.8265359	nonDEG	-0.7527542	0.07841589	1
0.75481206	0.00775721	0.12218543	nonDEG	0.30587804	0.40713696	1
2.19083928	9.65E-05	0.00430246	DEG	1.00406565	0.16067116	1
1.17190965	0.09219585	0.55329901	nonDEG	0.41732207	0.58360673	1
1.56604337	0.00205539	0.0469133	DEG	0.45670732	0.45262519	1
-0.7796514	0.14496732	0.67881232	nonDEG	-0.8383382	0.24074341	1
0.4563966	0.07590561	0.50462249	nonDEG	0.60160791	0.14479506	1
-0.3320994	0.26826331	0.85190792	nonDEG	-0.5348618	0.20670932	1
0.3717273	0.14357258	0.67613744	nonDEG	0.20229814	0.57750661	1

-0.5534723	0.16278585	0.714154	nonDEG	0.03931045	0.9395893	1
0.88492973	0.00609313	0.10220838	nonDEG	0.05849733	0.88342542	1
1.49491927	0.17559443	0.73931984	nonDEG	1.03800062	0.42920168	1
0.11109197	0.84609881	1	nonDEG	-1.5056895	0.03358057	1
0.43690394	0.07634527	0.50608614	nonDEG	0.13530074	0.72383763	1
-0.0153646	0.95136975	1	nonDEG	-0.0060188	0.98804469	1
-0.240722	0.46108263	0.98479636	nonDEG	-0.8862233	0.03701466	1
-1.043074	0.1246516	0.64340522	nonDEG	-0.8289264	0.42218514	1
-0.923866	0.00027607	0.00999643	DEG	-0.1003617	0.87653244	1
-1.182238	3.70E-05	0.0020123	DEG	-0.4239874	0.31410609	1
-0.6281336	0.03108031	0.30499113	nonDEG	-0.3704202	0.35489755	1
-1.7704874	0.0009409	0.02564737	DEG	-0.6748513	0.35429661	1
-1.6506426	6.16E-05	0.00299211	DEG	0.21779736	0.67079487	1
1.14422941	0.05028078	0.4058991	nonDEG	-2.4629614	0.00012586	0.05194852
0.32784805	0.68847611	1	nonDEG	0.78686965	0.37985601	1
-0.127548	0.60652971	1	nonDEG	-0.5160113	0.16589882	1
0.23052835	0.87925748	1	nonDEG	-0.0301816	1	1
0.31969773	0.19936956	0.77176073	nonDEG	0.10097951	0.78153136	1
-1.387848	0.09135004	0.5510019	nonDEG	-3.7891061	0.00381702	0.50616834
-0.4432998	0.07298292	0.49342136	nonDEG	0.16052096	0.69780119	1
-0.9817336	0.17110217	0.72989601	nonDEG	0.17466285	0.88453394	1
1.46276857	0.05008657	0.40485953	nonDEG	0.13263234	0.85183679	1
-0.3465412	0.38217641	0.93812152	nonDEG	-0.997428	0.06118192	1
0.9396199	0.09297105	0.55623323	nonDEG	1.13005799	0.04656771	1
0.18784058	0.53795274	1	nonDEG	0.35432337	0.38629663	1
-0.4768161	0.0632076	0.4607003	nonDEG	-0.2954234	0.46601894	1
-0.4202344	0.77481437	1	nonDEG	2.90181246	0.01987319	0.99822614
-0.9572153	0.01863551	0.21883163	nonDEG	0.03203901	0.95438514	1
-0.4558873	0.07610938	0.50531894	nonDEG	-1.5100326	0.00070843	0.17825087
0.27155995	0.62623061	1	nonDEG	0.50859956	0.40291914	1
-1.6932017	0.02399813	0.25884688	nonDEG	-0.6527599	0.33475904	1
-2.6650934	0.01591099	0.19754846	nonDEG	-0.28194	0.88628861	1
0.83157523	0.11888333	0.63025507	nonDEG	0.0703719	0.91355238	1
-1.0018286	0.00018389	0.00717217	DEG	0.70766149	0.18876364	1
-0.5629377	0.19033627	0.75718453	nonDEG	0.00167623	1	1
1.22513132	0.00576035	0.09796583	nonDEG	0.24986007	0.60065563	1
0.46808762	0.24725334	0.8287958	nonDEG	-0.7982536	0.08164005	1
0.48646948	0.07678379	0.50762137	nonDEG	0.60157333	0.17238491	1
0.81366953	0.03691331	0.33863851	nonDEG	0.43966681	0.30767885	1
1.69962131	0.01388859	0.18083419	nonDEG	-2.4570886	0.00237838	0.38006158
0.12276708	0.76093833	1	nonDEG	1.81645349	0.00030901	0.10107821
-0.8351883	0.00404146	0.07653744	nonDEG	-0.6778206	0.06619517	1
-0.5859953	0.05381394	0.42033127	nonDEG	0.03991649	0.93031634	1

2.45402098	0.04814969	0.39562065	nonDEG	0.10117333	0.91994677	1
0.02861923	0.91743936	1	nonDEG	0.64973761	0.0881697	1
-1.9355361	0.13241414	0.65711072	nonDEG	-1.411595	0.61222266	1
0.33746691	0.16812032	0.72400442	nonDEG	0.40390652	0.33895446	1
-0.5552331	0.05327036	0.41819366	nonDEG	-0.3237605	0.42054248	1
0.41030555	0.12082042	0.6327947	nonDEG	0.37113961	0.33007897	1
-0.7482967	0.20238037	0.77400965	nonDEG	0.23391108	0.82641982	1
1.02051197	0.06933491	0.48219949	nonDEG	0.06149068	0.93312851	1
0.65592745	0.59423158	1	nonDEG	2.57167839	0.05855174	1
-0.1270708	0.90478482	1	nonDEG	0.32473616	0.8339519	1
-0.8851692	0.00637326	0.10534658	nonDEG	-0.5919847	0.22968512	1
2.20431911	0.00477532	0.0857199	nonDEG	0.41862304	0.77056852	1
1.81318793	0.00083562	0.02338377	DEG	0.60575735	0.28032478	1
-0.2409684	0.83042351	1	nonDEG	-0.1489184	0.93916753	1
0.40604256	0.53576973	1	nonDEG	1.06943096	0.0944445	1
0.22767626	0.36578753	0.92576013	nonDEG	0.25546801	0.56279746	1
0.8355869	0.38172033	0.93796465	nonDEG	0.32903193	0.70625012	1
-0.879605	0.18943465	0.75564367	nonDEG	0.92928031	0.0749818	1
-0.9518244	0.00102645	0.02742069	DEG	-0.8471428	0.06974263	1
-0.0819144	0.88782608	1	nonDEG	1.01487965	0.05170791	1
-1.6618164	4.56E-05	0.00235293	DEG	-0.3763568	0.62523105	1
0.98028084	0.00021515	0.00813392	DEG	-0.0690339	0.85619511	1
-6.6950348	0.00101134	0.02704152	DEG	6.20789015	0.00626057	0.62218628
0.99381136	0.00046931	0.01524954	DEG	-0.1870263	0.62531139	1
0.98896773	0.00122873	0.03164701	DEG	-0.3389209	0.37962633	1
1.27785901	0.00036322	0.01255036	DEG	-0.5280359	0.31539579	1
1.31719399	5.67E-06	0.00044307	DEG	-0.8533269	0.02526311	1
1.40591092	7.12E-07	7.33E-05	DEG	-0.0225036	0.95185825	1
1.1620589	0.00131563	0.03338854	DEG	-0.1417997	0.73377229	1
1.00958624	0.00068953	0.02036011	DEG	-0.1525898	0.71059227	1
1.46547649	0.00221323	0.0492815	DEG	-0.4372712	0.3982999	1
1.29243682	1.75E-07	2.22E-05	DEG	-0.3583915	0.32271359	1
1.11918176	6.18E-05	0.00299964	DEG	0.4229658	0.3823577	1
1.21112612	1.33E-06	0.00012635	DEG	0.0743721	0.8421956	1
3.41520221	0.00116131	0.03025458	DEG	-0.6965021	0.56397486	1
1.95785932	8.17E-08	1.17E-05	DEG	-0.2715407	0.60431474	1
6.59469542	0.00145107	0.03592765	DEG	-2.3692512	0.10817774	1
1.34619278	6.72E-08	9.94E-06	DEG	-0.4733724	0.23168546	1
3.1896352	0.00035936	0.01244626	DEG	-2.6160066	0.00364037	0.49008216
1.70208474	1.19E-08	2.24E-06	DEG	-0.6169946	0.14232891	1
1.02496179	9.27E-05	0.00416576	DEG	0.15570634	0.68647224	1
1.33523418	9.56E-08	1.33E-05	DEG	-1.1469145	0.0209303	1
1.31882966	7.63E-06	0.00056605	DEG	0.08244147	0.83122317	1

1.70682548	0.00069781	0.02056348	DEG	-0.3742713	0.51387919	1
1.17537501	0.00147109	0.03621013	DEG	-0.3346283	0.43646756	1
1.25203361	4.02E-07	4.35E-05	DEG	-0.1488524	0.68077045	1
1.32093843	0.00178566	0.04216384	DEG	-0.4515518	0.28189942	1
1.22461396	2.90E-06	0.00024928	DEG	-0.4503083	0.21319761	1
0.87219969	0.00067413	0.02002599	DEG	-0.592282	0.11976624	1
1.68087792	0.00028856	0.01037225	DEG	-2.021316	3.61E-05	0.02308329
1.28694887	0.0005941	0.01827822	DEG	-0.47582	0.27402685	1
1.84386062	3.28E-07	3.73E-05	DEG	-0.998237	0.06330695	1
1.33112941	0.00043818	0.01455921	DEG	-0.0542788	0.91729951	1
-1.1478185	0.00019334	0.00746952	DEG	0.33080645	0.41230607	1
0.77965836	0.00121729	0.03140747	DEG	0.01003562	0.97769676	1
1.04331314	0.00075177	0.0217615	DEG	-0.2139476	0.61337178	1
1.61796861	3.26E-08	5.33E-06	DEG	-0.1719089	0.64579277	1
-1.1387213	0.00018273	0.00714409	DEG	0.27825928	0.52484093	1
-1.197316	0.00045945	0.01508089	DEG	-0.566713	0.26909888	1
-1.6012732	0.00088425	0.02440185	DEG	0.45218299	0.42560893	1
-0.9502575	0.00187323	0.04359372	DEG	-0.0046678	0.99495738	1
-1.9721917	0.00195909	0.04530529	DEG	-0.5891422	0.33098951	1
0.99340518	0.0008049	0.02271523	DEG	-0.1784786	0.63292227	1
1.09626529	0.00024558	0.00904829	DEG	0.07977918	0.84280347	1
1.15071465	0.00010641	0.00464108	DEG	1.08431762	0.05415847	1
3.00745223	0.00018667	0.00725939	DEG	-2.7290352	0.02193499	1
2.03967615	2.18E-05	0.00133127	DEG	-0.7312197	0.16588967	1
0.80926718	0.00108564	0.02874117	DEG	-0.7504587	0.04313582	1
2.90353028	3.22E-16	4.12E-13	DEG	-0.744069	0.06122096	1
1.15200361	0.00053094	0.01675292	DEG	-0.6116056	0.14043817	1
0.88938547	0.00095335	0.02581928	DEG	0.15785733	0.67536954	1
1.13183067	3.40E-05	0.00188882	DEG	-0.0637498	0.86182957	1
0.90788231	0.0006016	0.01841	DEG	-0.1322333	0.72125747	1
-1.042946	0.00047453	0.01536385	DEG	0.51941362	0.1602317	1
1.13071451	0.00200059	0.04590438	DEG	0.32433259	0.44417911	1
2.36978311	3.09E-05	0.00174444	DEG	-2.2247936	0.00018503	0.06333922
6.76450895	0.00085079	0.02369576	DEG	-2.9153582	0.04250195	1
-0.9408944	0.00108877	0.028798	DEG	0.9325153	0.02205222	1
2.39766165	1.72E-19	3.61E-16	DEG	-0.5416451	0.33485255	1
2.10740746	0.00187876	0.04368777	DEG	-1.0945166	0.23775615	1
-2.4558029	0.00097059	0.02618983	DEG	-0.3699089	0.75689268	1
1.36016941	7.31E-06	0.00055204	DEG	0.34351255	0.34511868	1
1.01858113	0.00031901	0.01130135	DEG	-0.3177607	0.39288108	1
2.29922241	3.04E-07	3.49E-05	DEG	-0.5272374	0.36355685	1
2.58775043	0.00021651	0.0081715	DEG	-2.9579502	0.00072772	0.18155319
1.75389421	3.41E-09	7.55E-07	DEG	-0.7341472	0.05319985	1

1.45201758	1.45E-06	0.00013649	DEG		0.42162101	0.52137846	1
1.00805422	0.00104741	0.02792998	DEG		-0.4069922	0.29095041	1
1.66934596	4.43E-05	0.00231397	DEG		0.19287075	0.75228983	1
2.29407115	7.83E-05	0.00361882	DEG		-0.4309002	0.45929501	1
1.09073534	0.00106344	0.02820408	DEG		-1.3063002	0.00140085	0.27131381
0.93639611	0.00127313	0.03253449	DEG		-0.1948707	0.59997975	1
1.46481979	3.50E-05	0.00193072	DEG		-0.2994615	0.45713707	1
1.12931705	2.38E-05	0.00142858	DEG		-0.2431502	0.51564636	1
-1.2309249	0.00076956	0.02210244	DEG		0.5560237	0.31957947	1
1.07012127	9.02E-05	0.00407898	DEG		-0.1824082	0.65086776	1
1.05015796	0.00192727	0.04467463	DEG		0.12376891	0.78554697	1
1.51172921	0.00064512	0.01945877	DEG		-0.7411605	0.14479808	1
-2.981026	0.00147405	0.03622262	DEG		-0.0620876	1	1
4.66557493	2.28E-05	0.00138284	DEG		-8.4567835	9.88E-07	0.00264462
1.23429887	0.0002339	0.0086832	DEG		-0.6582805	0.1502022	1
-0.9369586	0.00213294	0.04804256	DEG		-0.264179	0.5909354	1
1.5444485	1.50E-05	0.00097627	DEG		-0.9117825	0.0360604	1
1.26656126	2.57E-06	0.00022361	DEG		0.31256859	0.38667378	1
2.63157414	2.53E-11	1.08E-08	DEG		-1.3634872	0.0235696	1
-1.0561761	0.00025225	0.00921349	DEG		0.27960728	0.46297393	1
2.9733989	7.27E-10	1.94E-07	DEG		1.04046362	0.04395631	1
-1.0490605	0.00065355	0.01963251	DEG		0.10439634	0.81764145	1
8.03366076	3.88E-05	0.00208669	DEG		-7.8766089	0.00010233	0.04496309
-1.6597402	0.0006039	0.01844211	DEG		0.00033972	1	1
1.90242829	2.66E-11	1.12E-08	DEG		-1.4184055	0.00011676	0.05054695
-0.8097266	0.0018423	0.04311239	DEG		0.03696267	0.93384891	1
1.05514227	5.28E-05	0.0026587	DEG		0.04519565	0.90546351	1
1.63989122	1.24E-06	0.00012004	DEG		-0.593437	0.23348778	1
-2.2700104	0.00040836	0.01378627	DEG		0.28350095	0.74882454	1
0.9257255	0.00050034	0.01604533	DEG		-0.0872095	0.81728561	1
0.76787522	0.00177013	0.04189245	DEG		0.23288641	0.51777488	1
1.30653482	0.00019573	0.00754209	DEG		0.17876797	0.76298376	1
0.98572171	0.00050315	0.01610033	DEG		-0.1398662	0.70373891	1
1.13290118	0.00198196	0.04566103	DEG		0.66146263	0.16507018	1
2.65675232	1.55E-08	2.85E-06	DEG		-0.7242252	0.20173329	1
1.99697848	0.0001393	0.00575943	DEG		-0.5347757	0.52583352	1
0.98481004	0.00061306	0.01868401	DEG		0.06002177	0.87126544	1
3.26880446	7.88E-06	0.00057689	DEG		-4.0153971	3.59E-05	0.02308329
1.78290292	9.01E-05	0.00407898	DEG		-0.7197823	0.18275421	1
0.8296428	0.00153525	0.03741402	DEG		0.17688885	0.65168441	1
1.74013271	0.00133207	0.03363181	DEG		0.0422615	0.95235527	1
1.05879643	3.40E-05	0.00188882	DEG		0.02716258	0.94524859	1
-1.0842622	0.00029486	0.01054742	DEG		-0.0279816	0.94902991	1

1.23989463	3.12E-06	0.00026038	DEG	-0.1451011	0.69894352	1
0.90105337	0.000595	0.0182841	DEG	-0.2474375	0.50366782	1
0.85509758	0.00134122	0.03374167	DEG	0.01730021	0.96746048	1
4.87052393	0.00108458	0.02873903	DEG	-6.970043	0.00053317	0.14642867
3.32095016	0.00079997	0.02266637	DEG	-0.2916791	0.80476202	1
2.51253817	1.11E-13	7.78E-11	DEG	1.00829455	0.00829017	0.70535947
0.8508184	0.00086227	0.02394739	DEG	0.11279545	0.76852435	1
0.7990886	0.00207488	0.04720437	DEG	-0.0183836	0.96085146	1
-7.0285747	0.00049897	0.0160186	DEG	4.59218136	0.22472405	1
-3.3763987	0.00014495	0.0059267	DEG	2.82631342	0.0079739	0.69178488
0.97507611	0.00093889	0.02561637	DEG	-0.6267082	0.11557106	1
1.36310293	6.24E-05	0.00300561	DEG	-0.1045298	0.81570881	1
0.96040318	0.00077896	0.02226206	DEG	0.80498279	0.0410472	1
0.90825094	0.00053851	0.01691918	DEG	-1.0300045	0.00709095	0.66803023
1.27344263	0.00010469	0.00457954	DEG	-0.7474876	0.08861846	1
2.32398889	0.00147778	0.0362765	DEG	1.70729056	0.00555776	0.5887532
0.87687161	0.00214709	0.04821374	DEG	-0.1369038	0.75317845	1
1.0263017	0.00147871	0.0362765	DEG	-0.8603597	0.03484759	1
-0.936639	0.00151614	0.03700964	DEG	-0.0598082	0.9029422	1
1.06094952	1.31E-05	0.00087189	DEG	0.15727833	0.65941194	1
2.75058428	2.06E-11	9.33E-09	DEG	-1.2493704	0.03827824	1
0.77989085	0.00139337	0.03476212	DEG	0.04289003	0.90435743	1
0.92308468	0.00037281	0.01279718	DEG	-0.0998727	0.79326165	1
1.1392359	0.00050977	0.01625916	DEG	-0.0502948	0.90291614	1
2.30150678	8.56E-06	0.00061586	DEG	-0.7030396	0.19880864	1
1.98432867	7.09E-08	1.04E-05	DEG	-1.1457952	0.03601631	1
-1.5301796	0.00068541	0.0202791	DEG	-0.1058211	0.85032604	1
1.29955928	1.92E-07	2.42E-05	DEG	-0.394766	0.32008498	1
1.39024965	0.00121396	0.03134901	DEG	-1.5316063	0.00202203	0.3522274
-0.8558347	0.00198921	0.04571446	DEG	0.81737122	0.02658771	1
0.89215251	0.00111045	0.02929249	DEG	0.06342385	0.88745855	1
1.58232664	8.27E-05	0.00380325	DEG	-0.2854292	0.54621178	1
2.26209207	2.64E-05	0.00153723	DEG	2.01440387	0.00020114	0.06728831
0.96860661	0.00134184	0.03374167	DEG	-0.5118223	0.21319089	1
-0.9299783	0.00188219	0.04373299	DEG	0.1639085	0.67454901	1
3.71227206	0.00140873	0.03499725	DEG	-0.4284271	0.6522835	1
-2.7222467	0.00081029	0.02280513	DEG	1.37700969	0.17191778	1
1.16659282	3.23E-05	0.00181047	DEG	0.0581832	0.88785435	1
1.16237284	0.00036069	0.01247762	DEG	-1.2282595	0.01565944	0.89863185
0.99976549	0.00148259	0.03634147	DEG	-1.0172433	0.02991904	1
1.34247434	6.38E-06	0.00049059	DEG	-0.669207	0.10973256	1
1.01415022	0.00111274	0.02930048	DEG	-0.0746512	0.84453179	1
1.42062888	2.74E-08	4.56E-06	DEG	-0.2197815	0.56396034	1

1.38593349	4.16E-07	4.47E-05	DEG		-0.571099	0.12432176	1
3.69293839	0.0011836	0.03072669	DEG		-2.177086	0.07983554	1
1.02067558	0.0001785	0.00701584	DEG		0.0917488	0.8057619	1
1.17248065	0.00049153	0.01579715	DEG		-0.6488137	0.1023926	1
1.40527854	1.16E-07	1.56E-05	DEG		-0.1039298	0.77371369	1
2.33694455	1.21E-11	5.75E-09	DEG		-1.0587163	0.0132787	0.84289973
1.269183	1.28E-06	0.00012251	DEG		-0.1086555	0.76511676	1
0.9135258	0.00179902	0.04243693	DEG		-0.4365696	0.25836407	1
1.12900399	0.00147316	0.03622262	DEG		0.20820149	0.72515829	1
-2.38715	0.00036408	0.01256532	DEG		-2.1547464	0.01868652	0.95785021
0.96949341	0.00038293	0.01303244	DEG		-0.630177	0.11672266	1
1.54613557	5.30E-05	0.00266419	DEG		-2.472074	6.82E-08	0.00034445
2.06589961	2.15E-05	0.00132151	DEG		0.80868265	0.06882955	1
1.23998261	0.00028238	0.01018759	DEG		-0.0879824	0.85371179	1
2.68795586	3.09E-10	9.47E-08	DEG		-1.6678785	0.00519636	0.57294295
-1.5819719	0.00050148	0.01606418	DEG		0.30380602	0.61807349	1
1.3989794	0.00014478	0.0059267	DEG		-0.1657767	0.69889902	1
2.15972159	1.06E-09	2.73E-07	DEG		-0.6153327	0.14904485	1
1.39177813	1.09E-05	0.00074904	DEG		-0.4988054	0.18542232	1
3.55479333	1.17E-15	1.24E-12	DEG		-0.8360386	0.23437135	1
0.77557106	0.00187191	0.04359372	DEG		-0.2234393	0.53553074	1
0.94132654	0.00035111	0.01220344	DEG		-0.5818444	0.13461236	1
1.08552045	0.00032189	0.01136239	DEG		0.83761582	0.02579543	1
0.79911674	0.00178601	0.04216384	DEG		-0.2682661	0.4788285	1
1.10420574	0.00016539	0.00654437	DEG		-0.3664824	0.32038404	1
1.22141346	2.35E-05	0.00141445	DEG		-0.0345139	0.92417692	1
1.31949676	5.86E-05	0.00285823	DEG		0.08350216	0.8471337	1
-0.8497436	0.00197874	0.04565219	DEG		0.93356475	0.02490831	1
0.8193286	0.0020747	0.04720437	DEG		-0.2991897	0.43841	1
0.87711722	0.00040082	0.01356288	DEG		0.16005846	0.65799004	1
1.44493337	0.00050434	0.01612091	DEG		-0.2712848	0.58762434	1
1.78883759	7.32E-05	0.00342	DEG		-1.1019861	0.01686705	0.93512081
1.4592583	0.00146593	0.03614369	DEG		-2.1790953	3.26E-05	0.02179788
-6.6667466	0.00104728	0.02792998	DEG		5.20735799	0.06084894	1
1.93866484	0.00215948	0.04838117	DEG		-0.061961	0.93152227	1
1.08097772	0.00059809	0.0183409	DEG		-0.4537422	0.27237133	1
1.42124428	8.87E-05	0.0040293	DEG		0.05474825	0.90235973	1
1.7577308	1.48E-10	5.18E-08	DEG		-1.6319333	5.21E-05	0.02896648
1.44674494	1.75E-06	0.00015979	DEG		-0.265574	0.57422507	1
-1.9220131	1.65E-05	0.00105563	DEG		0.19681918	0.74350666	1
1.73649568	0.00053848	0.01691918	DEG		-0.3762238	0.66775124	1
0.79736131	0.00117156	0.03044102	DEG		0.16054007	0.65498552	1
1.08372911	0.00066957	0.01993081	DEG		0.14437481	0.7033229	1

1.40561914	6.99E-05	0.00329857	DEG	-0.6900584	0.10519163	1
0.81375425	0.00127508	0.03255595	DEG	0.16996405	0.63967491	1
1.60041437	2.80E-10	9.08E-08	DEG	-0.5951526	0.15763242	1
1.67900978	5.45E-06	0.00043032	DEG	-0.6739675	0.12025171	1
2.81584475	0.00052738	0.01667622	DEG	-0.6673993	0.4917781	1
-1.4325383	0.00041177	0.0138684	DEG	0.52833349	0.41180955	1
1.29658745	0.00222714	0.04940833	DEG	0.25761478	0.58682315	1
1.50674469	1.39E-07	1.83E-05	DEG	-0.0094327	0.98109854	1
1.03718365	0.00036872	0.01268086	DEG	-0.0530543	0.91062753	1
1.29841974	0.00044666	0.01478291	DEG	-0.8123496	0.11944868	1
1.26819145	0.00183239	0.04291478	DEG	-1.7361749	9.28E-06	0.00853991
2.66637748	0.00176783	0.04189245	DEG	-0.5935385	0.42306135	1
2.3523071	3.21E-07	3.67E-05	DEG	-0.7315452	0.17646164	1
1.66988801	0.00016623	0.00656853	DEG	-0.5369905	0.30857105	1
-2.5914888	6.60E-05	0.00314762	DEG	0.93847095	0.29241541	1
-1.0270651	0.00032185	0.01136239	DEG	0.19627443	0.61818522	1
0.87245512	0.00123718	0.03180899	DEG	0.14912373	0.68304501	1
0.84836303	0.00091515	0.02506158	DEG	-0.6021541	0.22088727	1
0.9462057	0.00012542	0.00532778	DEG	-0.0009945	0.99800942	1
2.8827575	1.10E-23	4.62E-20	DEG	-1.2273699	0.00429256	0.52515749
-1.0733729	0.00037361	0.01279718	DEG	0.20944202	0.60083745	1
2.15451768	1.61E-06	0.00014924	DEG	-0.5349473	0.24138427	1
1.13921912	2.51E-05	0.00147608	DEG	-0.9220656	0.0152325	0.89203498
0.8890394	0.00149957	0.03666595	DEG	-1.3279211	0.03259977	1
1.36496313	3.47E-06	0.00028765	DEG	-0.4876361	0.18564803	1
2.14684764	1.78E-10	6.08E-08	DEG	-0.2421408	0.53710056	1
0.93817155	0.00019614	0.00754775	DEG	-0.0526922	0.88872881	1
1.99022041	2.54E-07	3.00E-05	DEG	-1.3533974	0.00958117	0.75026005
-1.1752156	0.00210928	0.04783897	DEG	0.36719751	0.48509902	1
1.47932311	0.00074277	0.02156446	DEG	-1.7484003	0.00034418	0.10894905
1.77509066	0.00022811	0.00852217	DEG	0.08196602	0.8801729	1
-6.8891805	0.00054546	0.01711904	DEG	3.72414112	0.39057175	1
1.58548586	0.00053509	0.01684766	DEG	0.06495161	0.91430381	1
1.32556425	0.00040255	0.01360597	DEG	-0.6793865	0.18999132	1
-0.9066906	0.00203732	0.04656575	DEG	0.10212521	0.79384552	1
1.48304695	1.10E-06	0.00010888	DEG	-0.3847202	0.34183541	1
0.97828113	0.00046531	0.01518669	DEG	-0.379737	0.30990304	1
1.73935313	5.55E-05	0.00275725	DEG	-0.6770384	0.17086686	1
2.26823661	3.78E-05	0.00205028	DEG	1.00139279	0.04911098	1
1.59508454	4.53E-08	7.09E-06	DEG	-0.3170281	0.39175037	1
1.26802438	0.00010297	0.0045243	DEG	-0.2527457	0.52106732	1
-1.784781	1.83E-05	0.00116028	DEG	0.57519675	0.31009107	1
1.49150539	0.00119211	0.03089318	DEG	0.16745475	0.74169313	1

1.55474908	9.01E-08	1.27E-05	DEG	-0.6009603	0.162566	1
-0.9218274	0.00214475	0.04821374	DEG	0.72019624	0.08748979	1
1.17893098	0.00016271	0.0064642	DEG	-0.4118578	0.34963777	1
1.53843998	2.76E-09	6.25E-07	DEG	0.67530855	0.07094328	1
-1.1788126	9.88E-05	0.00438169	DEG	0.29024328	0.44730346	1
2.32134605	6.00E-11	2.32E-08	DEG	-1.1129478	0.03699321	1
1.53253135	4.09E-05	0.00217391	DEG	0.07772534	0.8530535	1
0.95019171	0.00011324	0.00487398	DEG	0.10503424	0.76893712	1
1.18117736	5.55E-05	0.00275725	DEG	0.037553	0.92391159	1
1.87018139	2.88E-11	1.19E-08	DEG	-0.2494918	0.59104661	1
1.10780125	6.82E-06	0.00052145	DEG	-0.2365098	0.50833297	1
1.06250079	0.00079508	0.02257124	DEG	-0.0915744	0.82371405	1
1.00624436	0.00159905	0.03864889	DEG	0.22357848	0.65338909	1
2.12152668	0.00066767	0.01991436	DEG	-0.0119175	0.99336072	1
1.01209358	0.00203343	0.04651287	DEG	-0.2517177	0.54987636	1
0.90056038	0.00050772	0.01621124	DEG	-0.3501207	0.34037822	1
2.38136397	0.0004381	0.01455921	DEG	-2.016352	0.00734257	0.67222413
-2.6517082	0.00010658	0.00464141	DEG	-0.2592768	0.81603526	1
0.97442133	0.00097052	0.02618983	DEG	-0.0888321	0.83307231	1
2.2917654	0.00139588	0.03476581	DEG	-1.346937	0.09161417	1
-1.3080258	3.68E-05	0.00200372	DEG	-0.056823	0.90273634	1
0.81422236	0.0009497	0.0257943	DEG	-0.2445802	0.50191569	1
0.87534592	0.00156354	0.03800905	DEG	-0.5833982	0.15175846	1
-1.0340581	0.00077677	0.02226206	DEG	0.34838793	0.38176401	1
2.59249036	4.80E-09	1.03E-06	DEG	-1.0085761	0.08952252	1
1.57184153	9.74E-06	0.00069011	DEG	-0.7334806	0.08412613	1
1.40138311	0.00140594	0.03495727	DEG	-0.4055366	0.40765484	1
2.66762789	7.24E-13	4.54E-10	DEG	-1.2433256	0.04259236	1
1.14025157	0.00018418	0.00717217	DEG	-0.4297767	0.292979	1
1.85422156	3.04E-10	9.42E-08	DEG	-0.4872358	0.33334145	1
1.56283611	2.46E-05	0.00146252	DEG	-0.6829584	0.13896242	1
-0.8955735	0.00220395	0.04911587	DEG	0.65490844	0.11259371	1
0.98115247	9.33E-05	0.00418665	DEG	0.08349168	0.82260174	1
1.01449366	0.00186316	0.04346239	DEG	0.12945242	0.74977646	1
1.06950639	4.74E-05	0.002422	DEG	0.27567183	0.47259484	1
1.1412236	0.0005567	0.01730582	DEG	-0.4143534	0.33200777	1
0.89275292	0.00029138	0.01046088	DEG	-0.2417562	0.52253713	1
1.01216159	0.00015377	0.00618422	DEG	-0.264892	0.50397832	1
1.56869395	0.00157618	0.0381902	DEG	-1.0543995	0.05759152	1
-6.9836459	0.00054787	0.01715827	DEG	0	1	1
1.0876426	0.00045435	0.01496167	DEG	-0.1684172	0.68524517	1
1.18420599	1.04E-05	0.00072786	DEG	-0.6141992	0.11986767	1
0.88398731	0.00136247	0.03407795	DEG	0.26391775	0.54642154	1

0.79435135	0.00145432	0.03597784	DEG	-0.1266219	0.75207027	1
-3.2085201	0.00098333	0.02641263	DEG	2.74373512	0.02288262	1
1.38203383	0.00063506	0.0192143	DEG	0.54858439	0.22069309	1
-0.9958293	0.00011041	0.00477758	DEG	0.08080563	0.83513652	1
-1.9697563	0.00173777	0.04135678	DEG	-2.2432071	0.02395046	1
7.39708645	9.10E-05	0.00410061	DEG	-0.7072482	0.52592174	1
0.92843356	0.00014626	0.00596345	DEG	-0.0247909	0.94570271	1
1.12699793	2.24E-05	0.00136427	DEG	0.22237724	0.54527544	1
-0.9998224	0.0017111	0.04087173	DEG	0.1895032	0.62408383	1
-0.8316368	0.00072025	0.02101417	DEG	-0.0647176	0.85799394	1
0.94621093	0.00176251	0.04184408	DEG	0.16939448	0.71800704	1
1.61539719	1.45E-07	1.88E-05	DEG	-0.3535796	0.37474441	1
2.93128592	1.07E-25	7.87E-22	DEG	-1.9724662	4.91E-06	0.00628998
1.44714818	0.00059021	0.01820022	DEG	0.18354596	0.79125863	1
-2.7873217	0.00160344	0.03872328	DEG	1.35503325	0.20352056	1
1.97925581	0.00076655	0.0220591	DEG	-1.7155313	0.01381134	0.85184879
1.94476209	3.89E-07	4.25E-05	DEG	-1.0671153	0.01120297	0.79590774
2.61337838	0.00085033	0.02369576	DEG	-1.0515652	0.22993843	1
3.19965322	1.80E-15	1.82E-12	DEG	-1.4820513	0.02120137	1
1.39006444	0.00015763	0.00631741	DEG	-0.8624104	0.09134142	1
3.09023244	1.48E-11	6.91E-09	DEG	-1.1145101	0.03988789	1
1.92159314	2.04E-12	1.06E-09	DEG	0.31117554	0.38302585	1
1.78495044	7.46E-08	1.09E-05	DEG	-0.9924041	0.02284334	1
1.18475416	2.10E-05	0.00129328	DEG	-0.1883638	0.6044246	1
3.25175641	0.00044742	0.01478291	DEG	-0.7942967	0.46431614	1
1.23019756	4.68E-05	0.00239777	DEG	-0.0091451	0.982052	1
-4.1959117	0.00129427	0.03293177	DEG	0.8832712	0.83420348	1
1.37864958	0.00132294	0.03346634	DEG	-0.7120343	0.12549525	1
1.05115929	8.73E-05	0.00397183	DEG	-0.5984435	0.1195715	1
1.3843602	6.40E-05	0.00306895	DEG	-0.0390947	0.93730628	1
1.61586733	0.00046184	0.01510663	DEG	-0.2672386	0.62059967	1
0.90102603	0.00034308	0.012038	DEG	0.05268359	0.88398736	1
3.00718694	0.00080684	0.02272975	DEG	-1.6595457	0.05596712	1
1.12641498	3.05E-05	0.00173001	DEG	0.03659347	0.92030735	1
5.24311605	1.00E-05	0.00070564	DEG	-2.6049825	0.00884511	0.72734986
1.9446867	6.21E-07	6.46E-05	DEG	-1.1314729	0.03559313	1
1.17084652	7.98E-06	0.00058182	DEG	0.12619933	0.78023162	1
1.52400966	0.00126003	0.03228363	DEG	-0.5321751	0.39331525	1
0.86500204	0.00146488	0.03614369	DEG	0.14059019	0.70135473	1
2.0014631	5.13E-08	7.87E-06	DEG	-1.2017525	0.02241408	1
0.90673435	0.00094885	0.0257943	DEG	0.02393611	0.95016921	1
0.92241751	0.00132324	0.03346634	DEG	-0.2516309	0.51767903	1
2.03020871	8.85E-08	1.26E-05	DEG	-1.1729239	0.02479847	1

1.56052445	4.06E-05	0.00215935	DEG	-0.7700782	0.08513221	1
0.93136008	0.00013676	0.00568643	DEG	-0.2071255	0.58004017	1
2.63212768	8.41E-06	0.00060701	DEG	-1.2794994	0.10454881	1
2.3800791	0.01116997	0.15809268	nonDEG	-4.9166541	7.27E-06	0.00691369
1.08794434	0.17302462	0.73256216	nonDEG	-3.9934654	6.68E-06	0.00691369
1.04250793	0.28797278	0.87024354	nonDEG	-4.7064243	5.06E-05	0.02864438
0.6818075	0.24922944	0.83045449	nonDEG	-3.288033	4.55E-07	0.00133845
-3.6659188	0.00505908	0.08907543	nonDEG	3.97623982	1.10E-08	8.07E-05
1.97203511	0.03761078	0.34215831	nonDEG	-4.9888773	3.16E-06	0.00465208
2.74944984	0.00444869	0.08189458	nonDEG	-4.4624636	6.68E-05	0.03577399
1.33038009	0.22998404	0.80924485	nonDEG	-5.4487413	1.02E-05	0.00882419
1.66044745	0.05116448	0.40907961	nonDEG	-3.7692548	2.21E-05	0.01714711
-4.931763	0.00370434	0.0728471	nonDEG	5.42881379	2.27E-05	0.01714711
1.7895792	0.14759042	0.68333836	nonDEG	-5.5406408	7.92E-05	0.03856757
1.93975275	0.0202164	0.23067851	nonDEG	-3.3087975	4.36E-05	0.02564683
1.52720176	0.08520047	0.53491714	nonDEG	-4.8188564	4.10E-06	0.00575021
0.50841816	0.09388064	0.55908861	nonDEG	-1.9315883	2.04E-06	0.00412442
1.92219019	0.00321098	0.06528188	nonDEG	-3.3557381	2.99E-06	0.00462688
0.47787938	0.35176236	0.91917773	nonDEG	-2.1704156	7.28E-06	0.00691369
1.08250293	0.04521846	0.38044766	nonDEG	-2.9938456	1.13E-07	0.00047665
0.79817495	0.43880151	0.97581792	nonDEG	-4.968431	1.02E-05	0.00882419
0.32941886	0.57246611	1	nonDEG	-3.3361752	1.33E-05	0.01090266
1.15199131	0.04974491	0.40317414	nonDEG	-3.9376561	4.15E-07	0.00133845
0.25645436	0.68134835	1	nonDEG	-2.7450393	3.80E-05	0.02349846
0.55543266	0.42018738	0.96323058	nonDEG	-4.9875917	7.07E-06	0.00691369
0.27878094	0.81743544	1	nonDEG	-5.8426877	6.30E-06	0.00686876
0.75624338	0.01213957	0.16676466	nonDEG	-1.8889164	6.18E-06	0.00686876
0.72194153	0.49060072	0.99934949	nonDEG	-4.9628314	2.44E-05	0.01792658
0.25420551	0.72772489	1	nonDEG	-2.7495534	9.44E-05	0.04219605
-0.8514858	0.02251738	0.2481555	nonDEG	-0.904126	0.03983178	1
1.12477203	0.1239347	0.6417591	nonDEG	0.12223228	0.88693799	1
0.7864426	0.41623841	0.96166196	nonDEG	0.43013882	0.69078475	1
-3.1073544	0.00232543	0.05091593	nonDEG	2.09758366	0.10905789	1
0.09560186	0.69949522	1	nonDEG	-0.1338165	0.71350476	1
0.18545691	0.88908264	1	nonDEG	-1.1675854	0.18578921	1
0.67185162	0.009666	0.14249091	nonDEG	-0.0035024	0.99259239	1
0.13153552	0.64153124	1	nonDEG	0.0718631	0.84815061	1
-0.6569541	0.0162614	0.19988277	nonDEG	0.22791141	0.54682874	1
0.36128138	0.72102991	1	nonDEG	-0.7131679	0.52780615	1
0.16962843	0.4965915	1	nonDEG	0.07179734	0.84356501	1
-0.0123087	0.97172453	1	nonDEG	0.24804846	0.54851603	1
0.19985271	0.51104265	1	nonDEG	0.25372247	0.53336872	1
-0.2343984	0.34838264	0.91917773	nonDEG	0.07961383	0.83401938	1

-0.063199	0.82573665	1	nonDEG	0.53930067	0.1787408	1
-0.0548457	0.86039394	1	nonDEG	0.09044123	0.82091886	1
0.01682678	0.97721707	1	nonDEG	0.29805367	0.57432066	1
0.1207843	0.73788322	1	nonDEG	-0.2826007	0.53371647	1
-0.0067225	0.99066321	1	nonDEG	-0.2363791	0.72741848	1
-1.0388054	0.01085	0.1552455	nonDEG	-0.2169695	0.69146422	1
0.15888949	0.65874521	1	nonDEG	-0.4352187	0.38870631	1
-0.3058203	0.21033497	0.78699211	nonDEG	0.00741201	0.98531972	1
0.75721002	0.12074378	0.6325994	nonDEG	-0.5765706	0.31290418	1
-0.2506473	0.74886082	1	nonDEG	-1.2706662	0.1562618	1
0.39080861	0.19592006	0.76693341	nonDEG	-0.0978256	0.81680451	1
0.08156937	0.74169117	1	nonDEG	0.27930068	0.45246962	1
0.3005042	0.50627871	1	nonDEG	-0.4489433	0.4104555	1
-0.3066065	0.46374093	0.98535325	nonDEG	-0.6410139	0.19423732	1
0.19763266	0.58379597	1	nonDEG	-0.0321183	0.94472925	1
-0.0152826	0.95855998	1	nonDEG	0.30356907	0.416423	1
-0.1689703	0.49942472	1	nonDEG	0.16146734	0.65923346	1
0.00139708	1	1	nonDEG	0.0588603	0.92625918	1
-0.2745737	0.37463033	0.93282096	nonDEG	-0.6785766	0.12988938	1
-0.3180244	0.27843277	0.86028362	nonDEG	-0.0214421	0.96136381	1
0.00575845	0.9819614	1	nonDEG	0.03534527	0.92314111	1
-0.2030269	0.42640328	0.96784011	nonDEG	0.11752283	0.78607826	1
0.4038151	0.14078901	0.67011925	nonDEG	-0.0987398	0.79483591	1
-1.3500109	0.48904272	0.99836609	nonDEG	1.75848837	0.31231576	1
-0.5306388	0.20997403	0.78664105	nonDEG	0.03974702	0.9445118	1
-0.2948258	0.52198536	1	nonDEG	0.72450637	0.18569926	1
0.25647762	0.41735677	0.96233775	nonDEG	0.43984654	0.26622164	1
-0.8273309	0.83419707	1	nonDEG	-3.1017818	0.64840318	1
0.39882365	0.54163169	1	nonDEG	-0.9515593	0.22874332	1
0.17669214	0.47459222	0.99095745	nonDEG	0.05689838	0.87715581	1
-0.6647009	0.62413037	1	nonDEG	-0.9007949	0.59007701	1
0.06977228	0.78617808	1	nonDEG	0.25145989	0.51741587	1
0.15654789	0.69059788	1	nonDEG	0.24891971	0.5854688	1
0.04641459	0.9245399	1	nonDEG	0.40293172	0.49969517	1
0.11440126	0.72216388	1	nonDEG	-0.2835406	0.47027126	1
0.48273787	0.11984086	0.63063362	nonDEG	-0.5853674	0.18026817	1
-0.7441692	0.00717517	0.11491859	nonDEG	0.34654245	0.41483849	1
-0.0214319	0.95617054	1	nonDEG	0.32744711	0.46697111	1
-1.0019314	0.06400332	0.4626059	nonDEG	-0.1078831	0.88104895	1
0.24833701	0.83901964	1	nonDEG	0.6601479	0.51838276	1
-1.0716983	0.46277266	0.98479636	nonDEG	-1.0944262	0.54746696	1
0.0196865	0.96105162	1	nonDEG	0.08217659	0.85958673	1
0.20216307	0.44690286	0.98167238	nonDEG	0.29349035	0.43696146	1

-1.4213912	0.13497596	0.6610751	nonDEG	0.82107772	0.41338136	1
-0.1891503	0.64312932	1	nonDEG	0.40110243	0.41028775	1
-0.3795435	0.52924391	1	nonDEG	0.43671168	0.5333542	1
-0.1113828	0.64408253	1	nonDEG	0.05340213	0.88153836	1
0.01340955	1	1	nonDEG	-0.9442149	0.36527779	1
-0.4495165	0.18316472	0.75037379	nonDEG	0.322921	0.44903286	1
0.15640708	0.77520748	1	nonDEG	0.09458173	0.89163635	1
-0.1086478	0.85296324	1	nonDEG	-0.858299	0.2327878	1
-0.3533646	0.40993049	0.95836418	nonDEG	-0.2530126	0.62548229	1
0.04147495	0.88049533	1	nonDEG	0.48040932	0.22737485	1
0.25686681	0.34953772	0.91917773	nonDEG	-0.0572507	0.87988496	1
0.57782487	0.02734634	0.28138723	nonDEG	0.1306251	0.71899992	1
-1.1540291	0.03571699	0.33221878	nonDEG	-0.490942	0.47232211	1
-0.0356514	0.88467871	1	nonDEG	0.17998341	0.69795314	1
-0.2761392	0.27556347	0.85835498	nonDEG	-0.1599958	0.65872137	1
0.32371673	0.18001385	0.74514618	nonDEG	0.01897376	0.95772338	1
0.09751076	0.69727158	1	nonDEG	0.07018605	0.84823702	1
0.55544396	0.0368541	0.33841165	nonDEG	-0.2264506	0.54068684	1
-0.6688466	0.134133	0.65933232	nonDEG	-0.4140668	0.46852894	1
0.0376828	0.9146721	1	nonDEG	0.41348102	0.31386157	1
0.07315972	0.76297843	1	nonDEG	0.20513879	0.56714271	1
-0.0850878	0.74950493	1	nonDEG	0.22417643	0.57140169	1
0.64209243	0.47433147	0.9909109	nonDEG	-0.2359399	0.81396241	1
-0.8158633	0.4615646	0.98479636	nonDEG	0.21002575	0.92680814	1
-0.2525145	0.50770483	1	nonDEG	0.23895908	0.62510067	1
-0.1309697	0.83855013	1	nonDEG	0.37203833	0.58021266	1
0.06972171	0.79276943	1	nonDEG	0.06196768	0.8702079	1
-0.178328	0.58037705	1	nonDEG	-0.0959532	0.84056159	1
0.61412263	0.08948163	0.54666582	nonDEG	-0.3591457	0.41394657	1
0.99755178	0.42689935	0.96874201	nonDEG	-3.4252533	0.04131637	1
1.77418921	0.04241721	0.36576454	nonDEG	-0.0530846	0.97291506	1
0.13208742	0.62314162	1	nonDEG	0.1275758	0.73523345	1
0.19055624	0.43744146	0.97423703	nonDEG	0.03604884	0.92103615	1
0.0101131	0.98059976	1	nonDEG	0.32456932	0.45380587	1
-0.5151557	0.69095712	1	nonDEG	0.0406385	1	1
-0.882101	0.2419714	0.82401585	nonDEG	-0.499815	0.61678968	1
-0.053732	0.84524356	1	nonDEG	-0.0893093	0.81592274	1
-1.4458052	0.0171069	0.2068102	nonDEG	-1.2999629	0.17040091	1
0.89214978	0.61405003	1	nonDEG	-0.642292	0.74155571	1
0.58941143	0.07770489	0.51046073	nonDEG	-1.1608574	0.01157628	0.80926302
-0.0855789	0.79014666	1	nonDEG	-0.0193624	0.96488067	1
-0.4274204	0.1796736	0.74514618	nonDEG	0.47606144	0.24706163	1
0.21161	0.43108718	0.97178962	nonDEG	0.03554377	0.92359645	1

0.21317776	0.5546819	1	nonDEG	-0.0836617	0.86968713	1
0.18816681	0.59725599	1	nonDEG	-1.2665785	0.00577792	0.59848676
0.26153559	0.31541235	0.89842779	nonDEG	-0.4142359	0.26368236	1
-0.1928582	0.62960712	1	nonDEG	-0.1613191	0.75132981	1
-0.4901861	0.04814258	0.39562065	nonDEG	0.51081415	0.16040002	1
0.53951078	0.31595896	0.89921847	nonDEG	0.23401212	0.69078601	1
0.12297347	0.61933374	1	nonDEG	0.07598833	0.83564826	1
0.50000085	0.29873581	0.88387501	nonDEG	0.00459508	1	1
0.03176583	0.89809387	1	nonDEG	0.18648858	0.60443347	1
0.04414927	0.86808849	1	nonDEG	-0.2579599	0.48199715	1
0.28758904	0.68770608	1	nonDEG	0.08584069	0.92073074	1
-0.1283008	0.64250124	1	nonDEG	0.19589516	0.60035156	1
-0.2920579	1	1	nonDEG	0.94124896	0.83509912	1
0.57066763	0.11560178	0.62124876	nonDEG	0.07263638	0.88725712	1
0.0104267	0.97992274	1	nonDEG	-0.2877185	0.5322377	1
0.70081373	0.42562308	0.96698651	nonDEG	-0.7357037	0.51505678	1
0.26151518	0.29406363	0.87719441	nonDEG	-0.3127566	0.38773821	1
-1.7396111	0.13058151	0.65446487	nonDEG	1.73724873	0.15339437	1
-0.2725091	0.47366615	0.99070103	nonDEG	0.07564125	0.87579078	1
1.95300926	0.01838164	0.21679329	nonDEG	-0.5836855	0.52700279	1
0.69210189	0.0131889	0.17473805	nonDEG	-0.0194388	0.9587856	1
0.48372792	0.1465784	0.68223696	nonDEG	0.01712747	0.968259	1
-0.1508273	0.53218006	1	nonDEG	-0.3189521	0.38906924	1
0.70613963	0.66926728	1	nonDEG	0.6634221	0.6911913	1
0.37813594	0.4739225	0.99085357	nonDEG	-0.4357184	0.46173579	1
0.18920489	0.55512786	1	nonDEG	0.28284091	0.47645834	1
0.20972175	0.84472507	1	nonDEG	1.90434985	0.02360641	1
-0.0505342	0.84773176	1	nonDEG	0.15124191	0.69322375	1
0.54660361	0.49113681	0.99949565	nonDEG	-0.0023101	1	1
-0.4434525	0.33421949	0.91780851	nonDEG	-0.4957993	0.3763114	1
-0.1895184	0.58619273	1	nonDEG	-0.2550957	0.56782266	1
0.03419891	1	1	nonDEG	-0.2066803	0.89903516	1
0.24215101	0.33035447	0.91411259	nonDEG	0.35539947	0.32564537	1
0.46320696	0.30587697	0.89022852	nonDEG	0.26829069	0.66801453	1
1.91801204	0.0646122	0.46487057	nonDEG	1.23395948	0.40635506	1
0.04645239	0.93438884	1	nonDEG	0.44776547	0.43758201	1
0.21749592	0.38493321	0.94140822	nonDEG	0.12216376	0.73585657	1
-0.2451913	0.62971768	1	nonDEG	-0.7576604	0.24104648	1
-0.1653343	0.74534907	1	nonDEG	0.24491932	0.68094256	1
0.11246427	0.74907456	1	nonDEG	0.41760281	0.3921485	1
-0.0759697	0.75264115	1	nonDEG	0.38325952	0.2858219	1
0.18492477	0.62999608	1	nonDEG	0.06704311	0.8875994	1
0.5108322	0.05650156	0.43226335	nonDEG	0.07858054	0.83588391	1

-0.116729	0.63957824	1	nonDEG	0.3589891	0.31862803	1
-0.56121	0.07151371	0.48814946	nonDEG	-0.0845893	0.84224794	1
0.10705089	0.68425829	1	nonDEG	0.06797784	0.85394041	1
0.50382028	0.17632218	0.7409355	nonDEG	-0.684441	0.14349679	1
0.28309831	0.25788439	0.83898637	nonDEG	-0.1221662	0.75331852	1
2.07325091	0.11439367	0.61799245	nonDEG	-0.6616922	0.67330748	1
-0.1142795	0.72336727	1	nonDEG	-0.7506338	0.09592726	1
0.55015753	0.55806114	1	nonDEG	-0.542175	0.61593193	1
1.94622421	0.08643049	0.53862182	nonDEG	0.54504707	0.69260764	1
0.11763007	0.63325411	1	nonDEG	-0.0246236	0.94761827	1
0.07829003	0.75826121	1	nonDEG	0.28873355	0.42358613	1
-0.2560695	0.7462402	1	nonDEG	-2.5709916	0.01349648	0.84878381
1.23628661	0.06112844	0.4516968	nonDEG	-0.9182492	0.20206398	1
0.11431268	0.63488004	1	nonDEG	-0.0822381	0.81887251	1
0.03194213	0.90802446	1	nonDEG	0.28091165	0.4433152	1
0.30506753	0.22075624	0.80037926	nonDEG	0.01938897	0.95786632	1
-0.0581375	0.83691776	1	nonDEG	0.05466722	0.89529475	1
0.35196372	0.18060947	0.74592623	nonDEG	0.25813471	0.48063169	1
-0.2311879	0.5969625	1	nonDEG	0.17822216	0.73594775	1
-0.3945429	0.5579507	1	nonDEG	0.04322027	0.96785114	1
0.09253311	0.94515169	1	nonDEG	1.18183171	0.16525226	1
0.10627634	0.68191826	1	nonDEG	0.20928566	0.57574899	1
-0.2024233	0.51864242	1	nonDEG	0.4050753	0.38800255	1
-0.6147994	0.0526213	0.41587071	nonDEG	-0.1802303	0.70399641	1
-0.105609	0.71298383	1	nonDEG	0.5683976	0.14062474	1
0.21814377	0.63154366	1	nonDEG	-0.1702499	0.77163318	1
-0.1551984	0.54178479	1	nonDEG	0.01010221	0.97866665	1
-0.3528343	0.21674188	0.79477343	nonDEG	-0.0661419	0.86421657	1
0.1877959	0.44838862	0.98329655	nonDEG	-0.0604283	0.86893419	1
0.38644663	0.12401271	0.64184426	nonDEG	0.2823228	0.43339894	1
-0.2103239	0.39156897	0.94686517	nonDEG	0.21322431	0.57064537	1
0.04620199	0.85247259	1	nonDEG	-0.2700757	0.45541561	1
2.79646908	0.09450113	0.56077784	nonDEG	-2.5148586	0.12328995	1
1.10599266	0.28518209	0.86735764	nonDEG	-0.3423727	0.72606771	1
0.6083635	0.02838174	0.28839265	nonDEG	-0.179659	0.63514203	1
-0.039965	0.8899691	1	nonDEG	-0.1428545	0.70418412	1
0.54212595	0.03168448	0.30845217	nonDEG	-0.2701562	0.46908178	1
0.24026843	0.34914345	0.91917773	nonDEG	-0.0713129	0.84465772	1
0.12413947	0.65076742	1	nonDEG	0.45971153	0.21850612	1
-1.828758	0.02730054	0.28105013	nonDEG	0.3822257	0.76197231	1
0.70239767	0.00447594	0.08220045	nonDEG	0.23415065	0.55457717	1
0.39275097	0.1014656	0.58089297	nonDEG	-0.2757875	0.45837032	1
0.16990766	0.7948329	1	nonDEG	-1.6135367	0.04430267	1

-0.1868538	0.57609862	1	nonDEG	0.05388508	0.90003251	1
-1.0354701	0.03051274	0.30122891	nonDEG	-0.3694565	0.56881612	1
-0.4224811	0.10953814	0.60478121	nonDEG	0.71492468	0.07868198	1
2.27792768	0.10132047	0.58067538	nonDEG	-1.1342583	0.42513793	1
0.13182608	0.65055618	1	nonDEG	-0.0269109	0.94522402	1
0.37579759	0.20948984	0.78572136	nonDEG	-0.0935339	0.83304213	1
-0.0508419	0.93975242	1	nonDEG	-0.8800613	0.21387784	1
-0.1399748	0.56785068	1	nonDEG	-0.3021835	0.44059085	1
-0.0657516	0.81094493	1	nonDEG	-0.2522289	0.50226094	1
0.67137814	0.15839029	0.70521047	nonDEG	1.03019459	0.13348138	1
-0.0418224	0.8672909	1	nonDEG	-0.2413851	0.5467659	1
0.16537262	0.64186182	1	nonDEG	0.17930801	0.72025908	1
-0.0597842	0.81383112	1	nonDEG	-0.0405792	0.91218119	1
0.18255851	0.66162226	1	nonDEG	0.14777848	0.78849242	1
-0.0870878	0.7479321	1	nonDEG	-0.0931644	0.80496917	1
-1.6278746	0.55972616	1	nonDEG	0.04511793	1	1
-0.2789988	0.25409873	0.83580029	nonDEG	-0.0902519	0.80521424	1
0.08529565	0.92324036	1	nonDEG	-1.4029652	0.14041421	1
0.21456007	0.44655843	0.98128189	nonDEG	0.42684321	0.2477432	1
-0.6294099	0.36678122	0.92635678	nonDEG	0.19007358	0.81948737	1
0.84498858	0.55292957	1	nonDEG	0.59514925	0.70880654	1
-0.1457988	0.80999397	1	nonDEG	0.46157149	0.49051523	1
0.16667965	0.49676552	1	nonDEG	-0.1267596	0.72795383	1
0.14326097	0.55911706	1	nonDEG	0.09802999	0.7901842	1
-0.1088755	0.66966989	1	nonDEG	-0.1464927	0.70120063	1
0.53478621	0.34584194	0.91869633	nonDEG	-0.4560926	0.46102639	1
0.27892379	0.27507345	0.85835498	nonDEG	0.22157579	0.54625479	1
-2.0400485	0.14297077	0.67537173	nonDEG	-4.3592531	0.1850963	1
-0.5874553	0.06265185	0.45812415	nonDEG	-0.0686987	0.8794945	1
-0.2045591	0.50477334	1	nonDEG	-0.2335317	0.62762836	1
0.16751824	0.64747877	1	nonDEG	0.52398426	0.23739141	1
0.14363369	0.79177914	1	nonDEG	-0.3262238	0.61359579	1
0.02761958	0.92968599	1	nonDEG	-0.2660957	0.5109422	1
-0.3096978	0.50323531	1	nonDEG	-0.2208053	0.68008863	1
-0.1694814	0.51564924	1	nonDEG	-0.2004834	0.59928107	1
-0.3989199	0.12559645	0.64431252	nonDEG	-0.0530448	0.88476733	1
-0.3120284	0.20125729	0.77336631	nonDEG	0.21743023	0.55420143	1
0.30847442	0.82822618	1	nonDEG	1.11661573	0.41179675	1
1.11266211	0.23410771	0.81483308	nonDEG	-2.3740643	0.03487574	1
-0.018049	0.94290242	1	nonDEG	-0.0890526	0.80814156	1
-0.5010195	0.21490735	0.7942651	nonDEG	0.19521964	0.7162607	1
-0.3268628	0.20485557	0.77887561	nonDEG	0.29552762	0.42473544	1
-0.4708078	0.13487092	0.660956	nonDEG	0.3263569	0.46994334	1

0.3608602	0.2635418	0.847847	nonDEG	0.23524548	0.55617618	1
0.50203003	0.10507613	0.59282029	nonDEG	-0.3462091	0.3945425	1
0.24691875	0.36267445	0.92389988	nonDEG	0.15824574	0.67147091	1
0.05875236	0.85001506	1	nonDEG	0.54664336	0.1628649	1
6.33986292	0.01191929	0.16515005	nonDEG	-6.1843765	0.01775495	0.95781535
0.36124527	0.21631935	0.79477343	nonDEG	-0.1811588	0.69419414	1
0.17482956	0.48231081	0.99555637	nonDEG	-0.0720701	0.84568645	1
0.10135284	0.67929401	1	nonDEG	0.20670488	0.57906634	1
-0.4184386	0.49380821	1	nonDEG	-0.3463028	0.69204528	1
-0.120124	0.64309354	1	nonDEG	0.25996149	0.47994472	1
-0.2231194	0.51502732	1	nonDEG	-0.0125891	0.97943065	1
0.27099585	0.39664621	0.94951131	nonDEG	0.24086519	0.5858707	1
-0.0306409	0.90990182	1	nonDEG	0.02942662	0.93734625	1
0.1244739	0.90889895	1	nonDEG	0.80915347	0.40495001	1
0.14926192	0.54986994	1	nonDEG	0.00278295	0.99468241	1
-0.2103196	0.40920298	0.95823562	nonDEG	-0.0519469	0.88558325	1
-0.1326491	0.6472426	1	nonDEG	-0.1360494	0.72768118	1
-0.1580102	0.70646898	1	nonDEG	0.43717808	0.48175854	1
0.15128914	0.53348956	1	nonDEG	0.15732197	0.69844644	1
0.26977443	0.789654	1	nonDEG	0.93311633	0.3338672	1
-0.5253989	0.21534381	0.79462351	nonDEG	-0.071887	0.89550408	1
-0.5455552	0.12073912	0.6325994	nonDEG	-0.4817651	0.32885708	1
0.30479373	0.2777988	0.85967939	nonDEG	-0.0698598	0.85775734	1
4.2347506	0.34519363	0.91869633	nonDEG	-4.0861667	0.35249128	1
0.09711265	0.69318645	1	nonDEG	0.35277112	0.34331211	1
-0.463565	0.18661255	0.75310345	nonDEG	0.96206268	0.02605023	1
-0.1625199	0.53715937	1	nonDEG	0.14205875	0.72190427	1
-0.0839294	0.74928367	1	nonDEG	-0.0833289	0.82650547	1
-0.4573887	0.07100788	0.48669278	nonDEG	-0.1864114	0.61050836	1
0.23203896	0.377681	0.93522146	nonDEG	0.06973584	0.88025519	1
-0.1521672	0.56636767	1	nonDEG	0.00326062	0.9939912	1
0.12382266	0.64725119	1	nonDEG	0.14626262	0.70764721	1
0.19617408	0.53337436	1	nonDEG	0.19416454	0.64035868	1
0.01013286	0.96684123	1	nonDEG	0.21947587	0.55566169	1
0.2203367	0.40390505	0.95387055	nonDEG	0.04594685	0.90095407	1
0.15049059	0.55952352	1	nonDEG	0.08303984	0.82021853	1
-0.0041261	0.98843417	1	nonDEG	-0.1096424	0.77801511	1
0.53984623	0.0790781	0.51515384	nonDEG	0.21215572	0.63036586	1
-0.3439958	0.1748808	0.73758468	nonDEG	-0.395243	0.30166916	1
0.66579461	0.05410694	0.42113858	nonDEG	-0.3329409	0.40098475	1
-0.2248478	0.41321803	0.95949897	nonDEG	-0.0200446	0.95905156	1
-0.2009083	0.56750455	1	nonDEG	0.16381809	0.7235632	1
0.56128745	0.07140751	0.48790733	nonDEG	-0.0795967	0.85518902	1

0.00701097	0.98036729	1	nonDEG	0.06659167	0.8561752	1
0.07625004	0.81663568	1	nonDEG	0.19495678	0.63994901	1
0.58930579	0.02889306	0.29159502	nonDEG	0.19357556	0.60955193	1
-0.4532445	0.27104741	0.85406365	nonDEG	-0.0557122	0.92163574	1
-0.1559531	0.57994343	1	nonDEG	-0.122209	0.75111004	1
-0.0498722	0.88686047	1	nonDEG	-1.432798	0.00518697	0.57294295
0.47106096	0.58297878	1	nonDEG	-1.095349	0.1719063	1
-0.1924037	0.6835427	1	nonDEG	-0.0096798	0.99366091	1
0.71403348	0.0798871	0.5180168	nonDEG	-1.3441824	0.0078969	0.68984185
0.03742468	0.90009209	1	nonDEG	0.02371544	0.95315679	1
-0.5534058	0.06241367	0.45706368	nonDEG	-0.0401362	0.92541523	1
-0.6217186	0.70936425	1	nonDEG	-0.0022266	1	1
0.57163658	0.31595097	0.89921847	nonDEG	-1.3599756	0.04830669	1
0.10212831	0.69160646	1	nonDEG	0.42088792	0.24735966	1
-0.1595856	0.57068826	1	nonDEG	0.1535146	0.68831073	1
-0.2402527	0.32735127	0.9112902	nonDEG	0.0751956	0.85077624	1
0.89928091	0.00330484	0.0667293	nonDEG	-0.4698641	0.23087479	1
0.08631648	0.75902092	1	nonDEG	0.28491491	0.45027312	1
-0.0242334	0.92218826	1	nonDEG	0.13514514	0.70991493	1
-0.0961475	0.80016349	1	nonDEG	-0.0829393	0.8721451	1
-0.1911676	0.77069789	1	nonDEG	0.61336452	0.41086649	1
-0.3194239	0.1924596	0.76192362	nonDEG	0.16380941	0.67526527	1
0.08880505	0.95257591	1	nonDEG	-0.0800754	1	1
-0.1048974	0.80802426	1	nonDEG	-0.1487314	0.78569225	1
0.28616238	0.61082729	1	nonDEG	-0.3369596	0.60923594	1
0.01739882	0.94368498	1	nonDEG	-0.0403718	0.91091073	1
-4.0729542	0.35468873	0.91917773	nonDEG	0	1	1
0.11648716	0.74759238	1	nonDEG	-0.3803195	0.42396233	1
-0.1340506	0.57551557	1	nonDEG	0.04337407	0.90336509	1
-0.1000378	0.77327348	1	nonDEG	0.03776209	0.93318447	1
-0.2744708	0.45003132	0.98329655	nonDEG	0.1041043	0.82470586	1
-0.2820088	0.32134452	0.90430629	nonDEG	-0.038037	0.92263844	1
-0.2185339	0.89342805	1	nonDEG	-0.4981015	0.73979725	1
0.01413687	0.97204029	1	nonDEG	0.60271905	0.17223418	1
0.24825315	0.53387328	1	nonDEG	0.04484001	0.9268375	1
0.93758671	0.00495933	0.08795036	nonDEG	-0.7008414	0.18972659	1
-0.143308	0.71646819	1	nonDEG	-0.3202003	0.53375734	1
0.22166241	0.38895686	0.94585339	nonDEG	-0.1950465	0.59835534	1
-0.7047232	0.0163412	0.20052881	nonDEG	0.84145285	0.04541826	1
-0.3489232	0.25450293	0.8362895	nonDEG	-0.2258433	0.58900318	1
0.05930425	0.8116798	1	nonDEG	0.32731868	0.36529197	1
-0.0103307	1	1	nonDEG	-0.0905379	0.92808775	1
0.00643227	0.98240832	1	nonDEG	0.21976477	0.56579839	1

0.05664402	0.908207	1	nonDEG	-0.1399164	0.816526	1
0.21450133	0.47899783	0.99332327	nonDEG	0.83783051	0.05373905	1
0.16404007	0.67579793	1	nonDEG	0.53865955	0.23652836	1
0.12846236	0.61694691	1	nonDEG	0.16066528	0.65952331	1
-0.8231781	0.02696853	0.27886427	nonDEG	0.22900916	0.66101327	1
-0.0539601	1	1	nonDEG	0.499835	0.6587904	1
0.04524281	0.94939098	1	nonDEG	-0.7133661	0.33320164	1
-0.8274309	0.83442417	1	nonDEG	0.04095168	1	1
1.07202121	0.23654292	0.81852308	nonDEG	-0.7932518	0.40498318	1
0.3039179	0.22313698	0.80348199	nonDEG	0.23805713	0.55108086	1
-0.1356199	0.60510318	1	nonDEG	-0.0109759	0.97690773	1
-0.0718961	0.88011519	1	nonDEG	-0.066686	0.9278473	1
0.0827544	0.81398398	1	nonDEG	0.06009901	0.89635366	1
0.00018579	1	1	nonDEG	0.00152608	0.99753379	1
-0.3814148	0.13986053	0.66813245	nonDEG	0.00284126	0.99464641	1
-0.1740551	1	1	nonDEG	0.24936796	1	1
-1.4123648	0.44142437	0.97752398	nonDEG	2.0952573	0.22057998	1
0.26100549	0.75575908	1	nonDEG	0.08120267	0.94585762	1
0.07198848	0.94447209	1	nonDEG	0.73509658	0.28881631	1
-0.2033122	0.425578	0.96698651	nonDEG	0.10918069	0.76547235	1
0.02839934	0.90782584	1	nonDEG	0.09315264	0.79664898	1
0.03435646	0.90636038	1	nonDEG	0.31372876	0.39726748	1
0.04994537	0.85191445	1	nonDEG	-0.091022	0.81260361	1
-0.3336515	0.30076675	0.88542723	nonDEG	-0.125435	0.76298077	1
0.60538029	0.01814983	0.21518836	nonDEG	-0.5272487	0.15343782	1
-0.0962584	0.81244397	1	nonDEG	0.15399094	0.75537749	1
0.2986106	0.58534873	1	nonDEG	-0.1246985	0.85012417	1
-0.336641	0.23927806	0.8208958	nonDEG	-0.3546382	0.42005943	1
-0.011954	0.96837806	1	nonDEG	-0.3999677	0.36886156	1
-0.9766976	0.0032087	0.06528049	nonDEG	0.14492035	0.73780431	1
0.28813761	0.23599708	0.81783615	nonDEG	0.18995188	0.59932466	1
-0.5402297	0.37316244	0.9318457	nonDEG	0.6377712	0.35720295	1
0.12269832	0.65661299	1	nonDEG	0.43671581	0.23462161	1
0.21514202	0.59775871	1	nonDEG	0.17211866	0.72551553	1
0.06268639	0.85449917	1	nonDEG	0.42005001	0.33664544	1
-0.4332353	0.10053149	0.57769793	nonDEG	0.0039809	0.99333068	1
-0.0788434	0.7445799	1	nonDEG	0.18445202	0.60670849	1
0.12245645	0.86872546	1	nonDEG	-0.6089646	0.41320519	1
-0.1691679	0.53196436	1	nonDEG	0.00256385	0.99563836	1
0.2424593	0.43545065	0.97371721	nonDEG	-0.047203	0.90907601	1
0.31086907	0.20485865	0.77887561	nonDEG	0.02920053	0.94429921	1
-0.3103893	0.25224507	0.83371668	nonDEG	-0.2818095	0.45681325	1
0.25578717	0.60627539	1	nonDEG	-0.8317709	0.17047194	1

-0.0711383	0.87752619	1	nonDEG	-1.511019	0.01205089	0.81648099
-0.1985536	0.48168224	0.99447672	nonDEG	0.13645261	0.72468251	1
-0.9086943	0.02925887	0.29417754	nonDEG	-0.0904382	0.85107271	1
0.06211534	0.8604642	1	nonDEG	0.14176883	0.74176718	1
0.36444063	0.23065864	0.80924485	nonDEG	-0.0248182	0.95255031	1
-0.1035944	0.87561682	1	nonDEG	0.59944226	0.36187672	1
0.11824811	0.72067904	1	nonDEG	0.58468664	0.14854236	1
-0.2916218	0.23316232	0.81339506	nonDEG	0.22984984	0.58042834	1
-0.0553426	0.85075475	1	nonDEG	-0.004223	0.99221392	1
0.15387058	0.54954659	1	nonDEG	0.14168533	0.69685981	1
-0.3763584	0.3696123	0.92855021	nonDEG	0.40558033	0.4367934	1
0.37603768	0.22745806	0.80910318	nonDEG	-0.1522081	0.6985191	1
0.26012926	0.4177357	0.96254612	nonDEG	-0.0170505	0.96872366	1
0.3391922	0.51788227	1	nonDEG	-0.6162589	0.33245158	1
0.42780524	0.71606525	1	nonDEG	-6.99266	0.00083048	0.19876888
-0.5646964	0.04155679	0.36234817	nonDEG	-0.1631177	0.69680939	1
-0.3482383	0.69903914	1	nonDEG	0.67214126	0.52492559	1
-0.103047	1	1	nonDEG	-0.4504126	0.87795252	1
-0.0503783	0.86956477	1	nonDEG	0.1931239	0.62067547	1
0.02176456	0.95159219	1	nonDEG	-0.0809535	0.85262817	1
0.82001265	0.33384082	0.91729884	nonDEG	0.68107733	0.46295962	1
0.48936861	0.08703256	0.53971705	nonDEG	-0.625131	0.10993147	1
0.67977223	0.01552668	0.19434101	nonDEG	-0.5145905	0.23231043	1
-0.4875491	0.24463148	0.82649442	nonDEG	-0.3484095	0.50422519	1
0.29219608	0.44836783	0.98329655	nonDEG	-0.0120441	0.98237279	1
1.90073093	0.02565453	0.26982626	nonDEG	-2.1737224	0.04669073	1
-0.4897871	0.18576222	0.75310345	nonDEG	0.13460631	0.7846398	1
1.10993474	0.0236958	0.25721998	nonDEG	0.09795729	0.83145496	1
0.32869556	0.24507141	0.8265359	nonDEG	0.16384401	0.67822899	1
-0.4815333	0.1991324	0.77155287	nonDEG	0.14289858	0.76431495	1
-0.3970339	0.36157382	0.92354131	nonDEG	-0.0784164	0.88979617	1
-0.590934	0.03605653	0.33390002	nonDEG	-0.0555723	0.88964203	1
-0.2430556	0.58949847	1	nonDEG	-0.0845217	0.88283142	1
0.73308502	0.27304709	0.85608852	nonDEG	0.17116449	0.82680152	1
0.30167467	0.63463114	1	nonDEG	-0.1503085	0.82828274	1
-0.102323	0.86877419	1	nonDEG	-0.2010403	0.77871589	1
-0.2376043	0.35171711	0.91917773	nonDEG	-0.4039878	0.26185974	1
0.21417633	0.39149859	0.94686517	nonDEG	-0.0960763	0.81588246	1
0.25437044	0.40250759	0.95352286	nonDEG	0.2588065	0.49261727	1
0.35562478	0.24935874	0.83050932	nonDEG	-0.0438966	0.91375042	1
-0.36691	0.23488315	0.81608933	nonDEG	0.00779554	0.98503504	1
0.14868957	0.64428772	1	nonDEG	0.12268259	0.75960483	1
-0.1574224	0.76903421	1	nonDEG	-1.1287534	0.08808359	1

-0.0629641	0.83795375	1	nonDEG	0.01348182	0.9752688	1
5.13E-15	1	1	nonDEG	3.14177741	0.64376366	1
0.02123928	0.93534271	1	nonDEG	0.15946857	0.66609233	1
-0.1638212	0.57418417	1	nonDEG	0.40106353	0.29773422	1
0.51988334	0.6993457	1	nonDEG	-3.5907337	0.03712009	1
0.24027989	0.38473714	0.94134613	nonDEG	0.24176248	0.51144741	1
0.54124018	0.06397942	0.46254672	nonDEG	0.54313349	0.17154074	1
0.56166123	0.02712139	0.2798551	nonDEG	-0.126348	0.75096128	1
-0.041982	0.87997759	1	nonDEG	-0.5507583	0.17494662	1
-0.0067297	0.98694858	1	nonDEG	0.08046838	0.85367925	1
-0.0722831	0.77946482	1	nonDEG	0.11152662	0.76145175	1
0.22096527	0.44583244	0.98063816	nonDEG	0.20780512	0.58363432	1
0.19809643	0.41794681	0.96254612	nonDEG	-0.0680287	0.84969424	1
-0.1979741	0.47984414	0.99343606	nonDEG	0.10001143	0.78611832	1
-1.348974	0.16609417	0.71927717	nonDEG	1.62871419	0.09963643	1
-0.0548104	0.83979896	1	nonDEG	0.06135298	0.87561996	1
-0.4468245	0.36681073	0.92635678	nonDEG	-0.4913733	0.44364415	1
0.07920251	0.74939708	1	nonDEG	0.19484049	0.60383073	1
0.02860836	0.9120193	1	nonDEG	0.20908368	0.57411275	1
0.0516037	0.84247893	1	nonDEG	-0.1416159	0.69940115	1
-0.2025099	0.54107925	1	nonDEG	0.32373895	0.43885812	1
0.23208678	0.37443369	0.93261985	nonDEG	0.01536521	0.96732046	1
0.37252336	0.25484652	0.83639093	nonDEG	-0.2475102	0.54487638	1
0.09185924	0.77554217	1	nonDEG	0.00388782	0.99439798	1
-1.1220158	0.0025359	0.05445256	nonDEG	0.11384129	0.80977214	1
-0.0531427	0.83090686	1	nonDEG	0.10262589	0.77753049	1
0.0473149	0.84887145	1	nonDEG	0.28378246	0.49199622	1
-1.3637168	0.00618112	0.10333109	nonDEG	-0.0809563	0.92057625	1
0.53263589	0.08625776	0.53811023	nonDEG	0.22858427	0.56753776	1
0.78562633	0.26537607	0.85033031	nonDEG	-0.8378777	0.2920243	1
0.32301339	0.18543657	0.75310345	nonDEG	-0.5244162	0.15397824	1
-0.2309083	0.40021437	0.95204649	nonDEG	0.01795261	0.96418856	1
0.54569155	0.27838947	0.86024013	nonDEG	-0.104669	0.86242566	1
-0.0398703	0.95176555	1	nonDEG	0.46138138	0.43417729	1
-1.1758069	0.22183745	0.8017252	nonDEG	0.5319345	0.69736672	1
0.14975078	0.55652122	1	nonDEG	0.01204626	0.97503757	1
0.04052863	0.87197292	1	nonDEG	0.01329428	0.97142421	1
0.36577597	0.13633225	0.66331422	nonDEG	0.04699916	0.90062198	1
0.29334276	0.31057761	0.89435467	nonDEG	0.58306415	0.21838345	1
4.79699625	0.18716959	0.75310345	nonDEG	-0.2161871	1	1
0.89901225	0.43144435	0.97178962	nonDEG	-1.4357873	0.24244014	1
-0.5762447	0.06445256	0.46426144	nonDEG	-0.1409787	0.75548143	1
0.01765885	0.94193825	1	nonDEG	0.11894551	0.74387018	1

-0.4267185	0.21804398	0.79640157	nonDEG	0.08670119	0.84705965	1
-1.799044	0.22070412	0.80037926	nonDEG	-0.5130081	0.8472427	1
-0.1808117	0.4541787	0.98394385	nonDEG	0.04478168	0.90421984	1
-0.6945965	0.27549977	0.85835498	nonDEG	-0.2560366	0.76165785	1
-0.0411853	0.90909309	1	nonDEG	0.21190188	0.62397102	1
-0.2605043	0.45181435	0.98329655	nonDEG	-0.3119555	0.50736807	1
-0.3187308	0.20115907	0.77336631	nonDEG	-0.1365699	0.70219355	1
0.17049021	0.64851586	1	nonDEG	0.23758278	0.60452915	1
0.1969402	0.48965781	0.99872846	nonDEG	-0.3033524	0.43799619	1
-0.2532008	0.44466396	0.97966661	nonDEG	-0.2913444	0.49401399	1
-0.248423	0.33404278	0.91768247	nonDEG	0.46203465	0.23529343	1
0.43777244	0.10855987	0.60254412	nonDEG	-0.4159049	0.26307074	1
0.61391902	0.02456083	0.26235351	nonDEG	-0.130507	0.72270818	1
-0.1916203	0.67923	1	nonDEG	-0.1326555	0.81879032	1
-0.0123699	0.96708676	1	nonDEG	-0.3453587	0.369539	1
-0.456026	0.24435692	0.82643802	nonDEG	-0.5501077	0.27151749	1
0.23621979	0.42039405	0.96333623	nonDEG	0.14698531	0.74806364	1
0.05060025	0.8351316	1	nonDEG	0.2167913	0.61537507	1
-0.0898361	0.81393283	1	nonDEG	0.37422887	0.42832125	1
-0.196909	0.41979763	0.96294915	nonDEG	-0.0662383	0.85619003	1
-0.0028019	0.99248265	1	nonDEG	0.23926821	0.51846312	1
0.41395349	0.17283331	0.7321974	nonDEG	0.21196507	0.60564312	1
-0.1918578	0.46859909	0.9871869	nonDEG	0.79976927	0.03440243	1
-0.1673931	0.53338714	1	nonDEG	-0.1453228	0.71404903	1
0.27529775	0.26370994	0.847847	nonDEG	0.21182579	0.56964603	1
0.28630092	0.26386678	0.847847	nonDEG	0.16686186	0.65145225	1
-0.1858166	0.63969503	1	nonDEG	-0.151826	0.7748901	1
-0.0308151	0.89985083	1	nonDEG	0.25732347	0.53999732	1
-0.1613275	0.53563379	1	nonDEG	0.24000487	0.51401408	1
-0.0620249	0.80282403	1	nonDEG	-0.2690946	0.48525048	1
0.54965139	0.26262059	0.84699667	nonDEG	-1.2469342	0.02861433	1
-0.010819	0.97106334	1	nonDEG	0.18334408	0.62494562	1
-1.2634388	0.10150187	0.58089297	nonDEG	-1.0065143	0.29330785	1
0.21581645	0.64750558	1	nonDEG	-0.3470634	0.54254462	1
-0.0581239	0.9673427	1	nonDEG	0.02013698	1	1
0.44631272	0.06706053	0.47329651	nonDEG	-0.1133483	0.80961127	1
0.20500187	0.51455285	1	nonDEG	-0.2504174	0.53498854	1
0.3805697	0.114828	0.61909498	nonDEG	0.1266437	0.72216671	1
-0.115607	0.64720245	1	nonDEG	-0.1680001	0.67659389	1
0.1624918	0.54232007	1	nonDEG	-0.2199261	0.54461734	1
-0.0314685	0.90449799	1	nonDEG	-0.1927667	0.60797552	1
0.14729151	0.56098054	1	nonDEG	0.23306835	0.51952692	1
-0.2529991	0.33960769	0.91869633	nonDEG	-0.3022889	0.50044448	1

0.13960266	0.7161052	1	nonDEG	0.16713573	0.73850109	1
-0.1825506	0.47261003	0.99012003	nonDEG	0.19427034	0.59868006	1
-1.7853684	0.00896525	0.13520907	nonDEG	-1.3601427	0.15211659	1
0.08233817	0.7509705	1	nonDEG	0.19519401	0.59048339	1
-0.4404987	0.08319419	0.52794861	nonDEG	-0.143246	0.69541795	1
0.14842363	0.5806009	1	nonDEG	0.16930971	0.65435452	1
-0.0306269	0.91113597	1	nonDEG	0.14005589	0.70873501	1
-0.4620558	0.08204582	0.52405012	nonDEG	0.35764441	0.33584157	1
0.2765171	0.36939259	0.92845225	nonDEG	0.37383955	0.33639069	1
-0.33333	0.36758448	0.92696488	nonDEG	0.43569447	0.32244514	1
0.43667886	0.08557018	0.5352955	nonDEG	-0.1387332	0.70104624	1
0.37183692	0.13164591	0.65533095	nonDEG	0.25590236	0.49903816	1
0.674535	0.32820785	0.91232212	nonDEG	-0.3805848	0.64841569	1
-0.2276559	0.42942918	0.97101957	nonDEG	0.01240162	0.9763128	1
-0.0059373	0.98442639	1	nonDEG	0.08809599	0.81322987	1
0.07267678	0.76602035	1	nonDEG	0.14378777	0.6912842	1
0.16076818	0.67879375	1	nonDEG	0.27303477	0.55366003	1
-0.2094593	0.39771348	0.95081663	nonDEG	-0.0305491	0.93308304	1
-0.4466937	0.4023378	0.95327344	nonDEG	0.22255706	0.73195856	1
0.07083886	0.76729042	1	nonDEG	0.1059984	0.76816974	1
-0.1748661	0.61906424	1	nonDEG	0.14181361	0.75396503	1
0.09457548	0.76515012	1	nonDEG	-0.0350244	0.93276085	1
0.03826399	0.87906183	1	nonDEG	-0.1897202	0.60464815	1
-1.209133	0.37417509	0.93251128	nonDEG	0.19914118	0.9318061	1
0.15737268	0.59305922	1	nonDEG	-0.0969392	0.80434968	1
0.28067789	0.27488674	0.85835498	nonDEG	0.2845428	0.43324577	1
0.1437496	0.56960729	1	nonDEG	0.40271177	0.27779614	1
0.56495921	0.03322094	0.31763274	nonDEG	-0.0264152	0.94371377	1
-0.2737627	0.27039509	0.85295594	nonDEG	0.14193674	0.71458505	1
-0.6998874	0.18967284	0.75575547	nonDEG	-1.3764314	0.02676231	1
-0.3162768	0.33434967	0.91780851	nonDEG	0.3156422	0.45737217	1
0.16673382	0.50821519	1	nonDEG	0.26032819	0.4777554	1
0.06480058	0.79181702	1	nonDEG	0.14774975	0.68271386	1
0.09377672	0.86327858	1	nonDEG	0.27270442	0.63274953	1
-1.1502291	0.60381442	1	nonDEG	0.10819386	1	1
0.3553402	0.15293832	0.69234986	nonDEG	-0.1187202	0.74297678	1
-0.0825673	0.76267479	1	nonDEG	-0.2070431	0.57781631	1
-0.1142326	0.65017033	1	nonDEG	0.01580753	0.96584468	1
0.11054427	0.73916155	1	nonDEG	0.17692068	0.6968692	1
0.77540934	0.01054151	0.15219793	nonDEG	-0.1996294	0.59466057	1
0.07552699	0.77234633	1	nonDEG	0.23729097	0.51054162	1
-0.1370926	0.8227197	1	nonDEG	0.65652808	0.40513011	1
0.2353475	0.41386597	0.95988343	nonDEG	-0.1587452	0.69615757	1

0.20783217	0.39799373	0.95095494	nonDEG	0.17910167	0.6729382	1
0.73200129	0.0073024	0.11651775	nonDEG	0.36894145	0.46584589	1
-0.8120482	0.02678121	0.27779566	nonDEG	0.18436619	0.70435682	1
0.30792313	0.2236967	0.80443197	nonDEG	0.16320749	0.65395031	1
-0.1985568	0.74634899	1	nonDEG	0.10338508	0.90109379	1
0.24948912	0.33375659	0.91729884	nonDEG	0.13754192	0.71907568	1
0.03510329	0.92426249	1	nonDEG	0.55740387	0.18906293	1
-0.3045919	0.41467498	0.96053724	nonDEG	0.57039436	0.21156027	1
0.04512838	0.90751547	1	nonDEG	-0.182467	0.69581171	1
-0.1282768	0.67427344	1	nonDEG	-0.3792609	0.4056343	1
0.63483274	0.03232071	0.31237337	nonDEG	0.2099622	0.60572316	1
-0.0115981	0.97170795	1	nonDEG	-0.2878861	0.48380429	1
0.06574029	0.78959017	1	nonDEG	0.37561302	0.31759609	1
-0.6126658	0.01524491	0.19195676	nonDEG	-0.1965737	0.61564879	1
-0.2123363	0.41321306	0.95949897	nonDEG	-0.1677665	0.6728826	1
0.42089093	0.23082436	0.80930446	nonDEG	-0.0010836	1	1
0.40649413	0.7042197	1	nonDEG	1.25554514	0.15476983	1
-3.0165137	0.6456824	1	nonDEG	0	1	1
-1.3608326	0.05927002	0.44386927	nonDEG	-0.3554392	0.70973946	1
-3.573952	0.00438958	0.08142707	nonDEG	0.19933371	0.87828227	1
-0.2080176	0.80450276	1	nonDEG	0.44670116	0.613054	1
-0.0476147	0.94158877	1	nonDEG	-1.0119864	0.18494137	1
0.81171429	0.00297922	0.06163403	nonDEG	0.00467606	0.99088062	1
0.15738201	0.53194929	1	nonDEG	-0.0762222	0.83599235	1
-0.1318921	0.66117071	1	nonDEG	0.04636884	0.91157434	1
0.17753012	0.47766202	0.99294124	nonDEG	0.03668948	0.91939145	1
-0.6034602	0.34984256	0.91917773	nonDEG	-0.3401102	0.66964271	1
0.48080426	0.06819985	0.47678944	nonDEG	-0.0648187	0.86035758	1
-0.030173	0.91865655	1	nonDEG	0.07156596	0.85224797	1
0.95049985	0.33575042	0.91869633	nonDEG	-1.9976537	0.06306116	1
-0.7683341	0.71409536	1	nonDEG	-4.6290621	0.16015377	1
1.04814126	0.16070078	0.71055424	nonDEG	-0.8767771	0.31944899	1
0.31425421	0.54659493	1	nonDEG	-0.9941548	0.10008278	1
-0.2233886	0.44764216	0.9825632	nonDEG	-0.0449286	0.91119218	1
-0.0614795	0.90090442	1	nonDEG	0.38062246	0.535211	1
0.26512552	0.41095784	0.95836418	nonDEG	-0.0223436	0.95825719	1
0.48561884	0.53470274	1	nonDEG	-0.2233604	0.81652608	1
-0.2801436	0.3290695	0.91297524	nonDEG	-0.0340329	0.93231508	1
-0.2896232	0.25035626	0.832419	nonDEG	-0.2122917	0.55687202	1
0.18818786	0.4726026	0.99012003	nonDEG	-0.0849218	0.81738891	1
-0.1004943	0.7166587	1	nonDEG	0.22628103	0.55000979	1
-1.0825418	0.04503456	0.37964291	nonDEG	-0.9784241	0.19530938	1
0.4956119	0.55961335	1	nonDEG	0.08863339	0.92453827	1

0.40160791	0.10010576	0.57558857	nonDEG	-0.0821756	0.82378176	1
-0.2435286	0.71169472	1	nonDEG	-0.3625036	0.6528834	1
0.2072759	0.45465374	0.98394385	nonDEG	-0.1214234	0.74584987	1
0.11005368	0.79117153	1	nonDEG	0.17007131	0.74314777	1
0.72524499	0.80645568	1	nonDEG	-0.5050324	0.87188543	1
-1.0723831	0.30827501	0.89225736	nonDEG	-1.8998549	0.14207078	1
-1.3977004	0.0520649	0.41358303	nonDEG	0.36643874	0.67699936	1
-0.0741121	0.81393425	1	nonDEG	0.07466105	0.85365181	1
1.14142051	0.09096172	0.55031281	nonDEG	-0.6662439	0.39377101	1
0.3626493	0.15756267	0.70306407	nonDEG	0.22955752	0.52369484	1
0.69061514	0.43841571	0.97547578	nonDEG	-0.2370493	0.85191144	1
0.10914311	0.65913212	1	nonDEG	0.22542422	0.53257097	1
0.28816078	0.25522248	0.83725145	nonDEG	0.39081144	0.29223902	1
0.52401788	0.07019027	0.48475818	nonDEG	0.00286249	0.99516104	1
0.02293191	0.94274	1	nonDEG	0.30395544	0.44595044	1
-0.335565	0.17924599	0.74510344	nonDEG	0.02238719	0.95389311	1
-0.0687461	0.774145	1	nonDEG	-0.1109363	0.75759346	1
0.59493118	0.50157664	1	nonDEG	-0.4550844	0.64788993	1
-0.4814763	0.49820545	1	nonDEG	-0.118328	0.905304	1
-0.5592736	0.07059017	0.48619944	nonDEG	-0.2639004	0.53594696	1
0.10381568	0.74191292	1	nonDEG	0.22158867	0.55935033	1
-0.1355164	0.62403179	1	nonDEG	0.12476439	0.74955942	1
-0.173966	0.65111306	1	nonDEG	0.28319314	0.54299549	1
-0.3286466	0.19749562	0.76905736	nonDEG	0.03457588	0.92868643	1
-0.0737651	0.86893988	1	nonDEG	0.05076081	0.92639347	1
0.57688437	0.10486447	0.59272458	nonDEG	-0.3713526	0.39358027	1
0.72467266	0.2241509	0.8048125	nonDEG	-1.2323475	0.09957581	1
-0.139342	0.62812381	1	nonDEG	-0.799476	0.04528544	1
0.04996723	0.86629236	1	nonDEG	0.04394993	0.91603729	1
0.13375435	0.59315583	1	nonDEG	-0.1946565	0.59055247	1
-0.1237588	0.63372923	1	nonDEG	0.15961546	0.68211407	1
0.11299493	0.85022121	1	nonDEG	-1.1260202	0.13494361	1
0.2596052	0.31678468	0.89948151	nonDEG	0.24636793	0.55149636	1
1.32165722	0.18823614	0.75373825	nonDEG	-0.0613978	1	1
0.23040175	0.46447924	0.98557044	nonDEG	-0.4210829	0.32426208	1
-0.0344345	0.94254051	1	nonDEG	0.0646078	0.90041302	1
0.57947013	0.06718797	0.47364623	nonDEG	0.36669927	0.36790671	1
-0.4821758	0.05736496	0.43581086	nonDEG	-0.1883487	0.64311334	1
0.16652135	0.51829231	1	nonDEG	-0.0380134	0.9207977	1
0.12262659	0.64083744	1	nonDEG	0.21414029	0.5693547	1
-0.1237013	0.62936285	1	nonDEG	0.1228542	0.73951932	1
-0.1963218	0.42088007	0.96384974	nonDEG	0.21658392	0.55736759	1
-1.4167746	0.03963582	0.35251928	nonDEG	0.59484134	0.4764852	1

-1.5047173	0.20937987	0.78572136	nonDEG	2.46079172	0.02546606	1
-0.2987746	0.64583254	1	nonDEG	0.30790639	0.69339399	1
0.26281311	0.66164929	1	nonDEG	-0.3615865	0.59983717	1
-0.2157616	0.73139928	1	nonDEG	-0.0310664	0.97696265	1
0.00580067	0.98283455	1	nonDEG	-0.0417661	0.91210283	1
0.72312401	0.00919478	0.13766588	nonDEG	0.6908674	0.19718669	1
-0.344892	0.19469273	0.7646157	nonDEG	-0.4067556	0.2994035	1
0.06434653	0.81834134	1	nonDEG	0.17723476	0.64266058	1
0.22726969	0.47061079	0.98901382	nonDEG	0.36389184	0.3796564	1
0.14116717	0.57761994	1	nonDEG	0.11349448	0.7595748	1
0.08315656	0.75013373	1	nonDEG	-0.0622881	0.86496471	1
0.7083665	0.70168463	1	nonDEG	-0.5911295	0.68032607	1
0.7945799	0.01128159	0.15891956	nonDEG	0.49352564	0.24773828	1
1.05050179	0.32386031	0.90721721	nonDEG	-2.0720293	0.08489341	1
0.35514199	0.43683706	0.97402891	nonDEG	-0.0817412	0.88277735	1
-0.2845175	0.27516121	0.85835498	nonDEG	0.03567194	0.9315345	1
0.22289135	0.46325645	0.98479636	nonDEG	-0.0770964	0.85225704	1
0.24925133	0.37419915	0.93251128	nonDEG	-0.4272713	0.31238017	1
-0.3946524	0.16046054	0.70986615	nonDEG	0.04885627	0.90290001	1
0.21020165	0.63715695	1	nonDEG	-0.0414223	0.94153449	1
-0.4968885	0.3286471	0.91273981	nonDEG	-0.400501	0.52528145	1
-0.2669239	0.40141876	0.95305194	nonDEG	0.62900249	0.14524488	1
-0.2559995	0.56821147	1	nonDEG	0.46788853	0.38291945	1
0.07514837	0.75873227	1	nonDEG	-0.0221757	0.96200775	1
0.10880783	0.65901754	1	nonDEG	0.06374053	0.85943195	1
-0.1904985	0.48374388	0.99599492	nonDEG	-0.3952329	0.28381691	1
1.17467938	0.06653366	0.47174477	nonDEG	-0.0004825	1	1
-0.2374906	0.35778151	0.92075237	nonDEG	-0.2529511	0.51484356	1
-0.1749262	0.4935211	1	nonDEG	0.08817232	0.81007933	1
1.07869201	0.23937791	0.82095133	nonDEG	-2.7164002	0.01348003	0.84878381
0.2152833	0.39071345	0.94686517	nonDEG	0.13306982	0.71166548	1
0.05600575	0.84346586	1	nonDEG	0.46059189	0.26652864	1
-0.0188061	0.94465142	1	nonDEG	0.12632983	0.73501615	1
-0.0514859	0.83421298	1	nonDEG	0.10534657	0.76995292	1
0.161521	0.62676141	1	nonDEG	0.18785979	0.66660903	1
0.12311355	0.71860015	1	nonDEG	-0.0835264	0.85090152	1
-0.2010598	0.72871419	1	nonDEG	0.40942367	0.53343435	1
0.44246801	0.10247146	0.58383147	nonDEG	-0.4194181	0.2758905	1
-0.2349726	0.62807261	1	nonDEG	-0.522122	0.35938323	1
0.10742628	0.71489354	1	nonDEG	-0.3137356	0.47088047	1
-0.6440134	0.24389894	0.82612635	nonDEG	-0.2476283	0.70245165	1
0.22397275	0.58117999	1	nonDEG	0.21304948	0.6431248	1
0.13154013	0.58707431	1	nonDEG	-0.1405665	0.70261866	1

-0.4547132	0.13237492	0.65711072	nonDEG	-0.3184414	0.43995298	1
-0.2223853	0.47298922	0.99049152	nonDEG	0.21620671	0.58302045	1
0.02492388	0.91741392	1	nonDEG	0.33591012	0.3667387	1
0.28294428	0.57265643	1	nonDEG	-0.0667897	0.91275925	1
-0.0548137	0.90994355	1	nonDEG	-0.2080089	0.74068951	1
-0.2399528	0.37876988	0.93586897	nonDEG	-0.0732468	0.84856171	1
0.03796204	0.96277212	1	nonDEG	0.49413159	0.50980602	1
0.22563776	0.36218218	0.92354131	nonDEG	-0.0166625	0.97192282	1
0.06765036	0.80940121	1	nonDEG	-0.1020875	0.78564276	1
0.35411052	0.17387489	0.73491786	nonDEG	-0.1952137	0.59615453	1
-0.4173436	0.17630521	0.7409355	nonDEG	0.14740393	0.71600545	1
-0.0592481	0.88782777	1	nonDEG	-0.0171643	0.97702952	1
-0.2488754	0.30893693	0.89264759	nonDEG	0.11674906	0.74820076	1
-0.1112653	0.67836649	1	nonDEG	0.03602234	0.92991476	1
0.36978717	0.33784439	0.91869633	nonDEG	-0.2990619	0.53039857	1
-0.3209641	0.28770571	0.87007405	nonDEG	0.03083742	0.94081704	1
0.62510752	0.21107981	0.7877265	nonDEG	-0.7718887	0.21465435	1
-0.2736122	0.27020467	0.85276106	nonDEG	0.08363549	0.83022902	1
0.3356017	0.18002787	0.74514618	nonDEG	-0.3684281	0.32599205	1
0.15955206	0.584366	1	nonDEG	-0.1774297	0.65274838	1
-0.1518568	0.58069253	1	nonDEG	-0.4792201	0.21894302	1
-0.0480414	0.86351881	1	nonDEG	-0.1593049	0.67807147	1
-0.2476931	0.33465973	0.91816031	nonDEG	-0.0308567	0.93535582	1
-0.3837714	0.31204849	0.89535767	nonDEG	-0.1151385	0.81745548	1
-0.2750827	0.43456026	0.973123	nonDEG	0.35801194	0.42696071	1
-0.2081683	0.39751252	0.95081663	nonDEG	0.12401824	0.73252166	1
-0.2387668	0.3263316	0.91057556	nonDEG	-0.0124229	0.97375322	1
0.62575101	0.08702813	0.53971705	nonDEG	-0.2017319	0.70626024	1
0.23353823	0.71187906	1	nonDEG	0.27662509	0.6809261	1
0.32910206	0.25339341	0.83481761	nonDEG	-0.9375931	0.01756829	0.95599411
0.54563402	0.44180811	0.97799065	nonDEG	-0.1361445	0.86240686	1
-0.9921218	0.10344573	0.58790327	nonDEG	1.71268284	0.00895255	0.73209494
0.22327581	0.46601173	0.98613495	nonDEG	-0.0828262	0.83393507	1
0.00868319	0.99325442	1	nonDEG	-0.4626852	0.47891978	1
-0.0183379	0.94621577	1	nonDEG	0.20172994	0.58810393	1
-0.2361653	0.34977847	0.91917773	nonDEG	-0.3418348	0.35973321	1
0.16648667	0.69941472	1	nonDEG	0.37466396	0.42305331	1
0.53010412	0.24934319	0.83050932	nonDEG	-0.1439804	0.78937944	1
-0.3831182	0.12385912	0.6417591	nonDEG	0.01165094	0.97560941	1
1.46801373	0.08224475	0.52441046	nonDEG	-0.2631778	0.768856	1
-0.086688	0.76347137	1	nonDEG	0.19526302	0.62748299	1
-0.2060135	0.69913045	1	nonDEG	-0.4899627	0.41464555	1
-1.7969051	0.18084931	0.74639324	nonDEG	-0.6457514	0.82762279	1

0.20831177	0.40987239	0.95836418	nonDEG	0.15858165	0.77731894	1
0.37105664	0.51317726	1	nonDEG	-0.9375223	0.15743155	1
0.64216944	0.89269814	1	nonDEG	-0.3329471	0.89567359	1
-0.4318201	0.64013344	1	nonDEG	-1.3103682	0.3001748	1
0.27163925	0.29263059	0.87552209	nonDEG	0.36431353	0.31901136	1
-0.2158799	0.41224386	0.95908664	nonDEG	-0.3738883	0.32301919	1
0.42987704	0.46078166	0.98479636	nonDEG	-0.5142682	0.51358802	1
-0.9914488	0.27287852	0.85596917	nonDEG	0.71044184	0.52422533	1
-1.3442132	0.46176681	0.98479636	nonDEG	-1.1741027	0.65483816	1
0.31989212	0.36907312	0.92832738	nonDEG	-0.3069978	0.50093511	1
-0.3322348	0.36752649	0.92696488	nonDEG	-0.02238	0.96971064	1
0.03686059	0.87879372	1	nonDEG	0.08052397	0.82250425	1
0.30011573	0.22516246	0.80629737	nonDEG	0.38139781	0.38111201	1
0.14157172	0.59148858	1	nonDEG	-0.3244959	0.38679765	1
-0.0385127	0.92099362	1	nonDEG	-0.1848	0.70284846	1
0.61858347	0.39579678	0.9493779	nonDEG	0.68191139	0.37387124	1
-0.8956883	0.43427658	0.973123	nonDEG	-2.175577	0.19945341	1
0.00383195	0.99566488	1	nonDEG	0.41989005	0.42110306	1
0.23017493	0.36772671	0.92707945	nonDEG	0.40848128	0.30304212	1
-2.0270152	0.08030015	0.51953337	nonDEG	2.34338952	0.09743642	1
0.23129423	0.34489783	0.91869633	nonDEG	-0.1075648	0.78911837	1
-0.0091172	0.98259574	1	nonDEG	0.11218446	0.82870051	1
-0.6569258	0.07751611	0.51021897	nonDEG	0.1668999	0.72876289	1
0.08676332	0.7362925	1	nonDEG	-0.5493221	0.13841094	1
0.03130705	0.912991	1	nonDEG	-0.249551	0.50948364	1
0.06984895	0.81932235	1	nonDEG	-0.0486469	0.91037966	1
-0.301521	0.58495608	1	nonDEG	0.16202841	0.79910033	1
-0.5849721	0.17800278	0.74368492	nonDEG	0.2157577	0.68676215	1
-0.4055108	0.20273028	0.77486116	nonDEG	0.04555298	0.91433601	1
0.50170266	0.04021727	0.35564922	nonDEG	0.06331627	0.86212374	1
0.11832508	0.6649971	1	nonDEG	0.04293525	0.90991707	1
0.2129219	0.38955344	0.94660039	nonDEG	-0.1354268	0.70711008	1
-0.217148	0.50803106	1	nonDEG	0.12350021	0.77001397	1
0.37563471	0.13601539	0.66313188	nonDEG	0.06593926	0.86386881	1
0.53613814	0.0409421	0.35903915	nonDEG	-0.1078554	0.81772794	1
-0.4085376	0.15905188	0.70668933	nonDEG	0.10484716	0.78569561	1
-0.0578891	0.92794146	1	nonDEG	0.00459572	1	1
0.21664899	0.43512251	0.97344567	nonDEG	0.34300734	0.37734419	1
-1.0233808	0.17830772	0.74433965	nonDEG	0.32875814	0.72123092	1
-0.0977264	0.78436618	1	nonDEG	0.1493541	0.73230272	1
0.39266786	0.10692997	0.59903167	nonDEG	0.30618744	0.4394657	1
-0.1384204	0.63356719	1	nonDEG	-0.0242463	0.95264153	1
-1.1422752	0.05246702	0.41532037	nonDEG	0.62274726	0.32803131	1

0.14536631	0.58187386	1	nonDEG	-0.0738983	0.84019758	1
-0.0588577	0.85493893	1	nonDEG	0.29237437	0.4633463	1
0.12710704	0.61821158	1	nonDEG	0.16078803	0.67778678	1
-0.0033566	0.99658852	1	nonDEG	0.40471213	0.3645235	1
0.38410623	0.1227691	0.63866398	nonDEG	-0.1557194	0.68689306	1
-5.0853336	0.0676499	0.4754102	nonDEG	0	1	1
0.08961498	0.77811914	1	nonDEG	0.2266194	0.56771355	1
0.37115995	0.25639654	0.83848676	nonDEG	-0.1119013	0.78074396	1
0.15198087	0.7335638	1	nonDEG	-0.0451358	0.93986193	1
-0.144596	0.54556331	1	nonDEG	0.10983279	0.76499724	1
-0.2371445	0.45113208	0.98329655	nonDEG	0.00426712	0.99316257	1
0.62092779	0.02099521	0.23663019	nonDEG	0.18677554	0.61344178	1
0.24880591	0.37488768	0.9329985	nonDEG	-0.0051938	0.99012899	1
-0.0061204	0.98887986	1	nonDEG	-0.0035739	0.9962625	1
0.33893539	0.67251875	1	nonDEG	-0.8480395	0.33221322	1
0.24739572	0.58462555	1	nonDEG	-0.8108644	0.13020763	1
-0.4067912	0.11038569	0.606843	nonDEG	0.03184507	0.93342511	1
0.79257341	0.04726933	0.39143796	nonDEG	-0.9082825	0.04660109	1
0.08043792	0.75156227	1	nonDEG	0.15523774	0.67267334	1
-0.281602	0.50632438	1	nonDEG	0.12533791	0.80778982	1
-0.0880131	0.76007812	1	nonDEG	0.13143401	0.74053591	1
0.31572805	0.29988347	0.88467981	nonDEG	0.19079002	0.6316384	1
0.19467917	0.42981347	0.97110382	nonDEG	0.16078207	0.65806569	1
0.21531221	0.43879135	0.97581792	nonDEG	-0.1155078	0.75970915	1
-0.3613549	0.52203753	1	nonDEG	-2.3808131	0.00151286	0.28733595
0.11258356	0.68911739	1	nonDEG	0.05922448	0.87610234	1
0.74283675	0.00228398	0.05029026	nonDEG	-0.0982924	0.7836345	1
-0.130475	0.75763362	1	nonDEG	0.45450365	0.35590191	1
-0.0946694	0.81548354	1	nonDEG	-1.3163658	0.01846627	0.95785021
0.14157136	0.56905238	1	nonDEG	0.30192113	0.42012368	1
0.25134298	0.37457994	0.9327744	nonDEG	0.17267396	0.65431637	1
-0.8967455	0.06188208	0.45519951	nonDEG	-0.027806	0.97296423	1
-0.0076104	0.97780465	1	nonDEG	0.29785638	0.44600267	1
-0.1313175	0.67218063	1	nonDEG	0.05641376	0.90124629	1
0.57400887	0.02045085	0.23236302	nonDEG	0.07046689	0.85238657	1
-0.1453033	0.61004661	1	nonDEG	0.15257573	0.68704528	1
0.3305961	0.87732892	1	nonDEG	-1.0771234	0.50997283	1
0.2408801	0.41575415	0.96115804	nonDEG	0.36533787	0.32953651	1
0.09021754	0.72951373	1	nonDEG	0.16903578	0.64799206	1
-1.7146261	0.04399151	0.37451301	nonDEG	-0.1534317	0.92444074	1
0.22504046	0.52635528	1	nonDEG	-0.1813898	0.68175766	1
0.1177845	0.63556301	1	nonDEG	0.05431345	0.88077396	1
0.17757693	0.504108	1	nonDEG	0.18625149	0.61639355	1

-0.3448932	0.23530068	0.81659042	nonDEG	-0.1654457	0.67207447	1
3.30290863	0.63508641	1	nonDEG	-3.1624231	0.63899192	1
0.32326094	0.43134619	0.97178962	nonDEG	0.12676626	0.79554956	1
0.22832659	0.35799908	0.92093105	nonDEG	0.19984569	0.57957879	1
0.21854671	1	1	nonDEG	0.74492238	0.77146185	1
0.52215239	0.03152394	0.30749364	nonDEG	0.03634939	0.91893514	1
-0.1361357	0.58053483	1	nonDEG	-0.0243683	0.94744664	1
0.37890356	0.37797142	0.93529818	nonDEG	0.08481163	0.86784197	1
0.62265729	0.01602134	0.19859039	nonDEG	0.16330736	0.66023439	1
-0.6935379	0.22434524	0.80513219	nonDEG	0.43423671	0.51058295	1
-0.0364893	0.90800785	1	nonDEG	0.29367216	0.45656769	1
0.00240803	0.99384778	1	nonDEG	-0.1241901	0.73393135	1
-0.0254904	0.92799297	1	nonDEG	-0.1673162	0.65904399	1
-0.1649903	0.52047077	1	nonDEG	0.2317155	0.52661214	1
4.76722949	0.13084267	0.65446487	nonDEG	-1.4547949	0.52078381	1
0.03380253	0.93366862	1	nonDEG	0.25238561	0.58959605	1
0.49482448	0.05411757	0.42113858	nonDEG	-0.0942892	0.80082599	1
-0.5792618	0.60133522	1	nonDEG	-1.314319	0.32505124	1
0.28263256	0.70729746	1	nonDEG	0.03558568	0.98139516	1
2.26562919	0.03120546	0.30563758	nonDEG	-0.4473303	0.73185656	1
-0.1632935	0.61690463	1	nonDEG	-1.0617508	0.01565103	0.89863185
-0.1736761	0.49398799	1	nonDEG	-0.0315767	0.9329762	1
-0.0464303	0.87712066	1	nonDEG	-0.3613374	0.35759848	1
0.26773611	0.2780031	0.85994885	nonDEG	0.26572735	0.50506292	1
0.0841234	0.96673138	1	nonDEG	-1.239982	0.2324713	1
0.28922078	0.25949719	0.8412441	nonDEG	0.09090007	0.80286389	1
-0.3678339	0.18253516	0.74935888	nonDEG	-0.3001768	0.46386104	1
0.06182614	1	1	nonDEG	0.46738397	0.80210495	1
-0.0395137	0.94354308	1	nonDEG	-0.8525143	0.17261021	1
-0.042133	0.88243111	1	nonDEG	0.09545293	0.82038101	1
0.11810469	0.64342871	1	nonDEG	-0.1885521	0.60671938	1
0.20685733	0.44751613	0.98254542	nonDEG	-0.1126101	0.76619452	1
-0.0434565	0.86374836	1	nonDEG	0.26965189	0.48514984	1
-0.7641923	0.59757667	1	nonDEG	1.0000623	0.41731296	1
0.13699466	0.58438054	1	nonDEG	-0.0150788	0.96692342	1
0.34431693	0.16138636	0.71144848	nonDEG	0.23401802	0.56962343	1
-0.0954318	0.71005157	1	nonDEG	-0.3241537	0.42902569	1
-0.0932824	0.78196696	1	nonDEG	-0.3682558	0.40234418	1
-0.186813	0.58509699	1	nonDEG	0.20200769	0.64742099	1
0.3636317	0.14051819	0.66947967	nonDEG	0.34556383	0.3366321	1
-0.2847531	0.24442085	0.82643802	nonDEG	0.28522099	0.486474	1
0.0821518	0.78188431	1	nonDEG	0.09234686	0.81307268	1
0.39765699	0.15049331	0.68725272	nonDEG	0.15762238	0.6860663	1

-0.1023681	0.76681213	1	nonDEG	0.23750542	0.55537598		1
0.20258463	0.6643881	1	nonDEG	-0.567546	0.31885334		1
0.06984174	0.79177876	1	nonDEG	-0.1746893	0.64052284		1
4.71371428	0.21702519	0.79477343	nonDEG	-4.561259	0.23852491		1
1.70266054	0.0052854	0.09212369	nonDEG	-0.3498953	0.64350975		1
-0.770826	0.13571116	0.66261751	nonDEG	-0.9295466	0.15835663		1
0.09970086	0.70565138	1	nonDEG	0.00749553	0.98459482		1
0.40250193	0.11360492	0.61607445	nonDEG	0.00598524	0.98788833		1
0.4097916	0.15079318	0.68750202	nonDEG	0.07947716	0.83416651		1
0.3222481	0.43751667	0.97423703	nonDEG	-0.6790411	0.20553335		1
0.77039656	0.0042051	0.07889981	nonDEG	0.34597951	0.33963425		1
0.05344022	0.83806741	1	nonDEG	0.05942949	0.87177751		1
0.16486738	0.51352279	1	nonDEG	-0.0986077	0.78503218		1
-0.1081607	0.67511045	1	nonDEG	-0.6471817	0.08800305		1
-0.181336	0.51760959	1	nonDEG	-0.0485324	0.90047477		1
-0.0950306	0.74110258	1	nonDEG	0.0495683	0.89905172		1
-0.0914942	0.72710682	1	nonDEG	0.17327719	0.64084804		1
0.1118156	0.7550006	1	nonDEG	0.17328184	0.73513496		1
-0.2773375	0.72852729	1	nonDEG	-0.9316956	0.35966222		1
-0.6483835	0.06646848	0.47142526	nonDEG	-0.3019264	0.55010972		1
-0.8797767	0.7286967	1	nonDEG	1.41995586	0.60186456		1
-0.4088411	0.26864714	0.8520756	nonDEG	0.06473875	0.89469293		1
0.71624176	0.32466418	0.90838968	nonDEG	-1.0674199	0.22091464		1
0.29158651	0.27111375	0.854165	nonDEG	0.29245841	0.44404743		1
-0.1029701	0.7431857	1	nonDEG	0.28266004	0.48192362		1
-0.3165494	0.23559837	0.81703149	nonDEG	-0.1467318	0.71690702		1
0.33081807	0.19427252	0.76398459	nonDEG	0.38368064	0.34009404		1
-0.0860063	0.73748528	1	nonDEG	0.09060984	0.80474742		1
0.26574291	0.31936367	0.90245222	nonDEG	-0.2513953	0.50575588		1
0.21009545	0.53237688	1	nonDEG	0.16609958	0.6881762		1
-0.0296803	0.91840903	1	nonDEG	-0.2422564	0.55098758		1
0.18062702	0.46719072	0.98662398	nonDEG	-0.2099434	0.56432912		1
-0.0492091	0.8396651	1	nonDEG	-0.3207538	0.37266607		1
-0.0980222	0.75230951	1	nonDEG	0.11479473	0.77450761		1
0.3201306	0.21003033	0.78674944	nonDEG	0.06148587	0.86581907		1
0.2037033	0.5092387	1	nonDEG	0.36895598	0.33215667		1
0.33875329	0.17905004	0.744789	nonDEG	-0.0504125	0.88985325		1
0.27586416	0.76533663	1	nonDEG	1.10640503	0.32354719		1
-0.2264784	0.35925819	0.92239681	nonDEG	-0.0479506	0.89489972		1
1.90222081	0.23509963	0.81632019	nonDEG	-3.4638386	0.04706374		1
-0.5521031	0.23530475	0.81659042	nonDEG	0.09851664	0.87053778		1
0.34948425	0.66717933	1	nonDEG	0.30436641	0.72157023		1
-0.503731	0.33752215	0.91869633	nonDEG	-0.1597015	0.81405743		1

-0.8164262	0.46886668	0.98740726	nonDEG		0.56381491	0.68584959	1
-0.0958125	0.80615797	1	nonDEG		0.86138764	0.13855613	1
0.37763932	0.38543702	0.94157168	nonDEG		0.42696047	0.38098252	1
0.38137615	0.1717466	0.73032618	nonDEG		-0.2882547	0.45468159	1
-0.3594984	0.16991473	0.727353	nonDEG		-0.3674885	0.35041208	1
3.2435607	0.63598442	1	nonDEG		-0.7900073	1	1
-0.1649151	0.50265608	1	nonDEG		0.12497509	0.73023818	1
0.17014491	0.62512972	1	nonDEG		-0.1114362	0.79766157	1
0.50680427	0.28468637	0.86713729	nonDEG		-0.1029753	0.85344434	1
0.02696891	0.93146817	1	nonDEG		0.05086817	0.89768942	1
0.10349613	0.71225812	1	nonDEG		0.00911029	0.9821007	1
0.61730321	0.47867146	0.99305902	nonDEG		-1.6709187	0.10187276	1
-0.139821	0.75024281	1	nonDEG		-0.6347921	0.25307279	1
0.06859574	0.83387117	1	nonDEG		0.43519015	0.2984221	1
-0.1127984	0.6925623	1	nonDEG		-0.0286523	0.94072782	1
-0.1078026	0.66073042	1	nonDEG		0.08982673	0.80424431	1
0.51709375	0.49832222	1	nonDEG		-0.0120457	1	1
-0.096744	0.80844986	1	nonDEG		0.06508545	0.89069525	1
0.69268598	0.19555055	0.76634006	nonDEG		-0.3010876	0.68116207	1
0.13481072	0.58888329	1	nonDEG		-0.055458	0.88326014	1
0.12070979	0.62425868	1	nonDEG		0.29661931	0.43254404	1
0.58393917	0.04900465	0.40006874	nonDEG		-0.3347041	0.3924415	1
0.3627497	0.15660344	0.70108546	nonDEG		-0.2173338	0.55412109	1
1.65590305	0.53772476	1	nonDEG		-4.6455393	0.23152376	1
-0.3503741	0.26190383	0.84578618	nonDEG		0.05259485	0.90000589	1
0.35831078	0.26295745	0.847237	nonDEG		-0.2970127	0.56283592	1
0.20748927	0.4830484	0.99583236	nonDEG		0.38286396	0.31443162	1
-0.8778674	0.00669595	0.10923913	nonDEG		0.40678684	0.34496719	1
1.64710402	0.21421646	0.79293932	nonDEG		0.01085808	1	1
0.71144292	0.03035657	0.30008968	nonDEG		-0.2270063	0.59196728	1
-4.0196736	0.01583379	0.19701996	nonDEG		-0.0161802	1	1
0.56861539	0.6185738	1	nonDEG		-0.6511659	0.57146025	1
0.342127	0.2399332	0.82187354	nonDEG		0.06778572	0.87135635	1
0.71222741	0.03353507	0.31967482	nonDEG		0.20458081	0.66650197	1
0.28614537	0.47954791	0.99338798	nonDEG		-0.2083284	0.67257085	1
-0.1411242	0.56234095	1	nonDEG		-0.0054602	0.98835091	1
-0.1377395	0.63590899	1	nonDEG		0.38050026	0.32111592	1
0.47384353	0.0797094	0.51749096	nonDEG		0.17581202	0.66514	1
0.18124914	0.52478557	1	nonDEG		0.16586136	0.65559063	1
1.32779216	0.01971726	0.22747822	nonDEG		-0.5717947	0.398269	1
0.09162839	0.77293143	1	nonDEG		0.35900215	0.36306299	1
-2.2027295	0.24516186	0.8265359	nonDEG		2.78994595	0.05904855	1
-0.5950803	0.19596023	0.76693341	nonDEG		0.39540061	0.47514289	1

-0.3748403	0.62661565	1	nonDEG	-0.6073678	0.52596489	1
0.75214436	0.02279526	0.24974682	nonDEG	-0.5334466	0.19506829	1
0.15281717	0.55065705	1	nonDEG	0.08410785	0.82087546	1
-1.2899595	0.07181622	0.48939761	nonDEG	-0.2913472	0.74154924	1
0.16706274	0.62368145	1	nonDEG	0.46839695	0.2519503	1
0.25020577	0.70482632	1	nonDEG	-0.7423359	0.33077933	1
0.05733636	0.84827963	1	nonDEG	-0.5764038	0.14916205	1
0.35025285	0.15563135	0.69873896	nonDEG	-0.0821297	0.82074411	1
-0.1046757	0.76794469	1	nonDEG	-0.3257198	0.44042255	1
0.07937832	0.75888215	1	nonDEG	0.14754785	0.69078813	1
-0.4483063	0.49077731	0.99944613	nonDEG	-0.0886991	0.91398163	1
-0.3667215	0.18683049	0.75310345	nonDEG	0.20650879	0.57993004	1
-0.3260021	0.2034336	0.77626464	nonDEG	-0.0177112	0.96231045	1
0.005108	0.98404957	1	nonDEG	0.17325307	0.65994626	1
2.53081205	0.30928916	0.89277786	nonDEG	0.54636425	0.77930749	1
-0.1631524	0.94921476	1	nonDEG	-0.0186504	1	1
-0.2826512	0.24888382	0.82990493	nonDEG	-0.1483499	0.68765724	1
0.1096234	0.67133826	1	nonDEG	0.2666955	0.46153851	1
-0.1813929	0.44923008	0.98329655	nonDEG	-0.292117	0.43225596	1
-0.3539613	0.22201841	0.80211073	nonDEG	0.07549852	0.85367407	1
-0.0256399	0.91890512	1	nonDEG	0.05440823	0.88158777	1
-0.2835361	0.35498202	0.91917773	nonDEG	-0.0954597	0.82186485	1
0.64318894	0.44091947	0.97742682	nonDEG	-0.7562175	0.44901384	1
-0.0824291	0.85317558	1	nonDEG	0.13819362	0.83169801	1
0.16726157	0.92391856	1	nonDEG	-0.2701001	0.82053546	1
0.05509247	0.85775571	1	nonDEG	0.11226476	0.77491725	1
-4.7955491	0.10628305	0.59733996	nonDEG	4.22896494	0.35329626	1
0.29139852	0.57742818	1	nonDEG	0.21123968	0.72582451	1
0.10608201	0.76391277	1	nonDEG	0.21890429	0.62015897	1
0.6953992	0.0185652	0.21826719	nonDEG	-0.2746123	0.4834945	1
0.23839808	0.33882037	0.91869633	nonDEG	0.1904706	0.59776632	1
-0.2941877	0.71774103	1	nonDEG	-1.204961	0.38422255	1
0.73298131	0.00998307	0.14570735	nonDEG	-0.3538221	0.38738002	1
0.32320836	0.20403523	0.77765318	nonDEG	0.3862923	0.34383833	1
0.43138032	0.20965101	0.7858594	nonDEG	-0.0350366	0.93406214	1
0.02649097	0.91866801	1	nonDEG	0.0499193	0.89269572	1
2.2790477	0.21677121	0.79477343	nonDEG	-1.1019826	0.55493772	1
0.19520631	0.6152937	1	nonDEG	-0.9173551	0.05963657	1
0.40086267	0.61050171	1	nonDEG	-0.6641285	0.42031348	1
-0.2517917	0.37374416	0.93237716	nonDEG	-0.384474	0.31940363	1
0.47227689	0.05401513	0.42112225	nonDEG	0.12687287	0.7584395	1
-0.1993052	0.53000767	1	nonDEG	0.07901734	0.86169185	1
-0.8858512	0.00298656	0.06171275	nonDEG	0.26866308	0.51352117	1

0.07469649	0.87522664	1	nonDEG	-0.2059511	0.73016926	1
0.3001812	0.42399102	0.96620031	nonDEG	-0.0286542	0.95300581	1
0.02394001	0.93889736	1	nonDEG	0.21416986	0.5823285	1
0.15843167	0.5497968	1	nonDEG	-0.0609788	0.87551403	1
-0.31403	0.76008534	1	nonDEG	-0.5489388	0.65657952	1
-0.0051303	0.9854036	1	nonDEG	-0.128536	0.73263498	1
-1.0533946	0.50346056	1	nonDEG	0.49271591	0.82947765	1
-0.320024	0.19353482	0.76342911	nonDEG	0.12134372	0.73854093	1
-0.3667934	0.20001359	0.7731247	nonDEG	-0.2826404	0.47762831	1
0.31041845	0.45519733	0.98394385	nonDEG	0.31884731	0.51816416	1
0.69433285	0.33872873	0.91869633	nonDEG	-0.593929	0.50240239	1
0.4196623	0.13219869	0.65706519	nonDEG	-0.1807767	0.63121865	1
0.10915243	0.65383777	1	nonDEG	0.13201634	0.71560887	1
1.39226773	0.11588325	0.6221935	nonDEG	0.42928184	0.64198468	1
0.43697871	0.09618377	0.56601681	nonDEG	-0.0082916	0.98306828	1
0.74358825	0.09945713	0.57437345	nonDEG	-0.2987263	0.66096373	1
-0.1095151	0.67851319	1	nonDEG	0.11049952	0.76321019	1
3.55559934	0.05525914	0.42663353	nonDEG	-1.9805343	0.27563471	1
-0.0165348	0.98886477	1	nonDEG	-0.242731	0.73524736	1
0.25341139	0.32008751	0.90313771	nonDEG	-0.3291663	0.36518892	1
1.34405093	0.18921437	0.75539488	nonDEG	-1.4039987	0.18277478	1
0.12835195	0.62041716	1	nonDEG	0.2396364	0.51335147	1
0.07422575	0.77758678	1	nonDEG	-0.3852201	0.29875428	1
0.81698889	0.52296485	1	nonDEG	-0.6231489	0.5898379	1
0.13627228	0.62442275	1	nonDEG	0.09509077	0.7984873	1
0.57618187	0.48345892	0.99599492	nonDEG	-0.6443168	0.49486331	1
-0.2216685	0.37817936	0.93532909	nonDEG	-0.1488642	0.68433358	1
0.16771444	0.68215587	1	nonDEG	0.03270759	0.95244794	1
-0.3414388	0.37107579	0.92971066	nonDEG	-0.3796449	0.44964626	1
0.24803042	0.53763227	1	nonDEG	0.32888869	0.56049181	1
-0.1635314	0.74349854	1	nonDEG	-0.0057142	0.99268367	1
-0.2627243	0.31820297	0.90098849	nonDEG	-0.3955089	0.28639602	1
0.17597707	0.47436708	0.9909109	nonDEG	-0.2237231	0.53985066	1
-0.3456904	0.19622096	0.76744373	nonDEG	-0.0319382	0.93552862	1
-0.2030463	0.60407041	1	nonDEG	0.22226858	0.65760091	1
0.36245696	0.2135295	0.79164399	nonDEG	-0.1881937	0.6280214	1
0.12356737	0.61695247	1	nonDEG	-0.6148716	0.11759071	1
0.11930202	0.84351878	1	nonDEG	0.08578592	0.89605652	1
0.63119731	0.16214104	0.71301481	nonDEG	-0.4739728	0.35440553	1
0.00172797	0.99561405	1	nonDEG	-0.0648865	0.85980694	1
-0.1266505	0.61700765	1	nonDEG	0.11138272	0.75662943	1
0.8795496	0.19366472	0.76363458	nonDEG	-0.0427279	0.95551738	1
-0.2136565	0.67193836	1	nonDEG	0.41018585	0.46773823	1

-1.1574041	0.05535488	0.42704204	nonDEG	0.67405441	0.41603999	1
-0.901837	0.47639904	0.99233789	nonDEG	0.48025467	0.78887702	1
-0.3387827	0.24359138	0.8256221	nonDEG	-0.1987239	0.61013723	1
0.14456412	0.67581513	1	nonDEG	0.19994778	0.63554808	1
-0.2483057	0.50378095	1	nonDEG	0.21569039	0.63700996	1
-0.1481812	0.64255173	1	nonDEG	-0.1850108	0.64349333	1
-0.07996	0.78359405	1	nonDEG	0.15309379	0.70718822	1
0.07113963	0.94925681	1	nonDEG	0.04363038	0.96833463	1
-0.2316782	0.46790993	0.986734	nonDEG	0.50339106	0.19366562	1
0.50111855	0.4809431	0.99426825	nonDEG	-0.5897855	0.44196918	1
-0.5517453	0.41408733	0.95994306	nonDEG	-0.5906587	0.44341032	1
0.14123701	0.62005503	1	nonDEG	0.07360959	0.84499195	1
-0.5262871	0.25146783	0.83366682	nonDEG	0.3818133	0.49368889	1
0.34320718	0.24028586	0.82195857	nonDEG	0.77009668	0.0774693	1
-0.1349609	0.60546105	1	nonDEG	0.08893983	0.80840876	1
-0.0832138	0.74393578	1	nonDEG	-0.0136664	0.97088762	1
0.22977448	0.41459791	0.96053724	nonDEG	0.24854566	0.53007212	1
0.3378554	0.20702952	0.78248067	nonDEG	0.16439178	0.66343466	1
0.23211132	0.64167311	1	nonDEG	-0.0405193	0.95404687	1
-0.1937218	0.89021977	1	nonDEG	-1.9728428	0.26874232	1
0.01357812	0.96447668	1	nonDEG	-0.4606649	0.24019258	1
-1.2037188	0.15470427	0.69606279	nonDEG	0.36237734	0.71801877	1
0.03824135	0.89609876	1	nonDEG	0.07470126	0.84839755	1
0.50830153	0.68276112	1	nonDEG	0.08817559	0.97087816	1
-0.3621035	0.23711764	0.81892791	nonDEG	0.18900217	0.63647508	1
0.09549825	0.76580043	1	nonDEG	0.23768712	0.55430005	1
1.37363576	0.02533113	0.26753234	nonDEG	0.94198063	0.25767884	1
-0.0427901	0.864276	1	nonDEG	0.11975442	0.75714085	1
-0.3973912	0.49169469	0.99972374	nonDEG	-0.7564518	0.29322498	1
-0.2638536	0.35280488	0.91917773	nonDEG	0.11265305	0.7746492	1
0.53346241	0.10968529	0.60513969	nonDEG	-0.3857871	0.3633269	1
-0.2777267	0.27556297	0.85835498	nonDEG	-0.0109552	0.97745019	1
0.39289821	0.1226093	0.63832968	nonDEG	0.15812768	0.66122118	1
-0.2669744	0.60579664	1	nonDEG	0.72895678	0.2236929	1
0.9603317	0.13166134	0.65533095	nonDEG	-0.5384774	0.43731948	1
-0.1369304	0.57643373	1	nonDEG	0.19074374	0.5962658	1
-0.2814607	0.62545331	1	nonDEG	-0.4834799	0.4825214	1
4.96497407	0.09012967	0.54766713	nonDEG	-1.1297195	0.58637336	1
-0.2559808	0.52943587	1	nonDEG	0.63472761	0.1861928	1
-0.172905	0.48541446	0.99670526	nonDEG	0.20912429	0.57872812	1
0.1746928	0.57749262	1	nonDEG	0.16336897	0.68565089	1
0.10264862	0.69388992	1	nonDEG	0.13140791	0.71929866	1
0.62676914	0.01720319	0.20771547	nonDEG	-0.0543911	0.88666966	1

0.1268713	0.60582131	1	nonDEG	0.5260818	0.14876766	1
-0.2426113	0.47851965	0.99305902	nonDEG	-0.2365982	0.59549194	1
-0.6798575	0.00545609	0.09431693	nonDEG	-0.1452738	0.7038128	1
0.35205555	0.14629986	0.68147492	nonDEG	-0.1552216	0.67289957	1
2.28698269	0.36227165	0.92354131	nonDEG	-2.9565838	0.27459308	1
-0.4201114	0.3449105	0.91869633	nonDEG	0.12005795	0.82457675	1
-0.436273	0.09165899	0.55224625	nonDEG	-0.3993506	0.29206842	1
-0.205153	0.40218856	0.95307326	nonDEG	0.03345987	0.92641415	1
-0.0874928	0.76690098	1	nonDEG	-0.1083505	0.79725368	1
0.74570187	0.26937669	0.85230023	nonDEG	-0.2000959	0.79584572	1
-0.2687888	0.44491525	0.97982211	nonDEG	-0.0312045	0.94947914	1
2.06904857	0.18884052	0.75431819	nonDEG	-1.8309832	0.19109879	1
0.18462435	0.45680209	0.98426953	nonDEG	-0.257766	0.50747744	1
-0.2254684	0.43996791	0.97664117	nonDEG	0.22645777	0.57409032	1
-0.4071962	0.40418515	0.95404159	nonDEG	-0.4161818	0.46859916	1
-0.4242725	0.27455867	0.85818661	nonDEG	0.2093142	0.65784892	1
0.4994484	0.80682936	1	nonDEG	-1.3277727	0.4193217	1
0.28185247	0.67900119	1	nonDEG	0.58331378	0.35985694	1
0.6904893	0.00598169	0.10079857	nonDEG	-0.1630589	0.65645597	1
-0.363316	0.59860388	1	nonDEG	-0.5396221	0.5112778	1
0.39039668	0.46023996	0.98479636	nonDEG	-0.7524136	0.23304643	1
0.16641656	0.49810292	1	nonDEG	0.26891521	0.46065959	1
0.61708032	0.22846188	0.80924485	nonDEG	0.44761337	0.44413175	1
0.67848042	0.32529008	0.90946768	nonDEG	0.34343836	0.62175835	1
-0.8140886	0.24406679	0.82615639	nonDEG	0.62826106	0.45145818	1
-1.0794401	0.14022999	0.66900939	nonDEG	-0.7537509	0.47119132	1
0.7524245	0.00487903	0.08699809	nonDEG	-0.1036378	0.77666477	1
0.01684509	0.94921776	1	nonDEG	-0.1887873	0.60796279	1
0.1533425	0.75816375	1	nonDEG	-0.5188385	0.3793134	1
1.33551794	0.02947448	0.29533671	nonDEG	0.28342121	0.59192161	1
0.40041713	0.13055915	0.65446487	nonDEG	0.19220931	0.59804518	1
-0.0237422	0.93133871	1	nonDEG	0.32260523	0.39391079	1
0.93582688	0.16363551	0.714154	nonDEG	-0.5972762	0.45371904	1
-0.0674916	0.79703777	1	nonDEG	0.19925454	0.61180437	1
0.07042219	0.85131205	1	nonDEG	0.00068209	1	1
-0.1367347	0.66711903	1	nonDEG	0.35583435	0.38933028	1
0.31631591	0.24907365	0.83023667	nonDEG	0.25109809	0.49388365	1
0.10823819	0.82553887	1	nonDEG	0.25604064	0.71965412	1
-0.5903263	0.23511596	0.81632019	nonDEG	0.64059609	0.27349407	1
0.18693656	0.5166163	1	nonDEG	-0.0196314	0.96138741	1
-0.0150706	0.95910849	1	nonDEG	-0.131267	0.74828613	1
0.50029844	0.24850391	0.82990493	nonDEG	-0.0193097	0.97007422	1
-0.0879947	0.73999842	1	nonDEG	0.12949442	0.72759561	1

0.20568149	0.83754638	1	nonDEG	0.70720033	0.41485291	1
1.1218733	0.15872301	0.70615789	nonDEG	-0.6955015	0.61617382	1
-0.5747963	0.69892507	1	nonDEG	-0.0967865	1	1
0.12485606	0.80248907	1	nonDEG	0.60236733	0.29823345	1
0.49544826	0.63380233	1	nonDEG	-0.0812949	0.95237123	1
0.64317967	0.01314692	0.17449596	nonDEG	-0.0166839	0.9661682	1
-0.0691148	0.84935307	1	nonDEG	-0.2364981	0.61331977	1
-0.2063605	0.44472101	0.97966661	nonDEG	0.31884533	0.41652784	1
-0.2051196	0.40671447	0.95617157	nonDEG	0.09561732	0.78942945	1
0.54931423	0.03986578	0.35376105	nonDEG	0.08055939	0.82563835	1
-0.0442494	0.87517268	1	nonDEG	-0.2407093	0.52742602	1
0.41234204	0.10854344	0.60254412	nonDEG	-0.0723241	0.8430682	1
-0.1335272	0.64479253	1	nonDEG	0.02970205	0.94003494	1
0.89039944	0.14608443	0.68076602	nonDEG	0.18797075	0.77475974	1
0.7396264	0.00650544	0.1069439	nonDEG	-0.0537531	0.88662649	1
0.00324518	0.99184101	1	nonDEG	-0.3626257	0.38120265	1
-0.4534685	0.47209621	0.99002994	nonDEG	-0.0649554	0.94394257	1
-0.1905605	0.44130578	0.97752398	nonDEG	0.00727944	0.98563348	1
1.6006067	0.01529685	0.19252837	nonDEG	-0.4966111	0.56641067	1
-0.245585	0.31471585	0.89809705	nonDEG	0.27489681	0.45665143	1
-0.0439444	0.85895364	1	nonDEG	-0.0492978	0.89120021	1
0.06707709	0.83242437	1	nonDEG	-0.2742076	0.53036053	1
-0.4245278	0.23109021	0.80988866	nonDEG	-0.4945847	0.32761524	1
-0.5613973	0.72159904	1	nonDEG	-3.0900219	0.2421371	1
-0.2923761	0.41228623	0.95908664	nonDEG	-0.1602064	0.72711958	1
-0.6996196	0.26997723	0.85268624	nonDEG	0.85334283	0.22339179	1
1.03003103	0.05181111	0.41200022	nonDEG	0.71045516	0.12631204	1
-0.4502093	0.09024913	0.54780294	nonDEG	0.09151245	0.81758955	1
0.04491388	0.90235251	1	nonDEG	0.57056318	0.17152376	1
-0.0708659	0.82516865	1	nonDEG	-0.0080549	0.98564598	1
-0.4903076	0.18884218	0.75431819	nonDEG	0.53100132	0.25442961	1
0.58310467	0.03665123	0.33741237	nonDEG	0.1023863	0.77994128	1
0.1073657	0.6846973	1	nonDEG	0.33871255	0.38083474	1
0.27204825	0.32625229	0.91057556	nonDEG	-0.1710172	0.65190066	1
0.32477105	0.22454073	0.80564761	nonDEG	0.15842246	0.66622173	1
0.12524004	0.81921165	1	nonDEG	-0.8923564	0.29122286	1
1.09485054	0.01293889	0.1729042	nonDEG	0.20284751	0.67668538	1
0.57205842	0.02278874	0.24974682	nonDEG	-0.4143379	0.26934108	1
0.0075841	0.99328302	1	nonDEG	0.04118185	0.9510213	1
-4.4594669	0.27575909	0.85842562	nonDEG	4.04369377	0.34951307	1
-0.1525538	0.52421104	1	nonDEG	-0.0984154	0.78589228	1
-0.2690075	0.5816059	1	nonDEG	0.88483431	0.09758349	1
0.36933861	0.14859732	0.68531592	nonDEG	-0.0773035	0.83286214	1

-0.1858199	0.58453658	1	nonDEG	-0.2516548	0.56615759	1
0.41343093	0.12323801	0.64008537	nonDEG	-0.514852	0.16663074	1
0.35772872	0.33446278	0.91780851	nonDEG	0.0460329	0.91778201	1
-0.0273568	0.93947659	1	nonDEG	-0.1384137	0.75421526	1
0.0427251	0.87992333	1	nonDEG	-0.183835	0.63947715	1
-0.5448473	0.3875206	0.94454537	nonDEG	0.26854892	0.69281684	1
0.02868629	0.91632803	1	nonDEG	0.07595539	0.83716269	1
0.50693963	0.06476245	0.46550937	nonDEG	-0.1047682	0.77549972	1
0.08998324	0.72200639	1	nonDEG	-0.1743088	0.63169077	1
0.30777393	0.25843693	0.83909237	nonDEG	0.23406649	0.51757911	1
-0.1043955	0.7232334	1	nonDEG	0.12693668	0.74225055	1
-0.4104606	0.22101236	0.80072941	nonDEG	-0.0205911	0.96633802	1
-0.6892029	0.17700969	0.742306	nonDEG	-2.0084601	0.00406386	0.52015192
0.22695299	0.35965186	0.92284417	nonDEG	0.42255688	0.25292674	1
0.27318897	0.26041143	0.84309382	nonDEG	0.15766797	0.70691817	1
0.01157681	0.98505082	1	nonDEG	0.00482723	1	1
0.10347977	0.69579878	1	nonDEG	0.11234217	0.7593229	1
0.66801843	0.01543838	0.19381256	nonDEG	0.06312147	0.86541566	1
0.11764803	0.70417542	1	nonDEG	0.30413079	0.44223582	1
-0.8829647	0.07335779	0.4944241	nonDEG	-0.5179948	0.34754741	1
-0.4983069	0.111146	0.60931602	nonDEG	0.52681623	0.20429094	1
-0.0751858	0.77239429	1	nonDEG	-0.0459475	0.90473895	1
1.47192247	0.34623638	0.91869633	nonDEG	-0.70231	0.72104262	1
0.86886651	0.19594043	0.76693341	nonDEG	-0.7005197	0.34602388	1
-0.1422555	0.59988287	1	nonDEG	0.06191571	0.86724853	1
0.28578713	0.41101491	0.95836418	nonDEG	0.11801184	0.78220893	1
-0.3976996	0.13842107	0.66616778	nonDEG	0.12606887	0.7363138	1
0.12886092	0.71303298	1	nonDEG	-0.2074841	0.63280035	1
0.97720492	0.51966725	1	nonDEG	-0.1106485	0.95622069	1
0.31363152	0.51763463	1	nonDEG	-0.136136	0.8336747	1
0.08412843	0.73839946	1	nonDEG	-0.2283167	0.54197708	1
1.74621891	0.16729517	0.72214115	nonDEG	-1.3772496	0.36607818	1
0.05796477	0.81344463	1	nonDEG	0.34438717	0.36105586	1
0.13643224	0.66013668	1	nonDEG	0.24020621	0.55569919	1
0.18199548	0.47411351	0.9909109	nonDEG	0.30811782	0.43702941	1
-0.1594842	0.88640684	1	nonDEG	-0.4062887	0.78540018	1
-0.441201	0.3106255	0.89435467	nonDEG	0.0421284	0.94414319	1
-0.0982546	0.74088397	1	nonDEG	-0.516985	0.22082413	1
-0.0161913	0.96786687	1	nonDEG	-0.2848501	0.52190677	1
-0.3369724	0.34296903	0.91869633	nonDEG	0.4267183	0.37003514	1
0.01015806	0.96865476	1	nonDEG	-0.1526906	0.67609999	1
-0.0008931	0.99978604	1	nonDEG	-0.2197364	0.59091177	1
-0.2412261	0.61553747	1	nonDEG	0.86369581	0.20109077	1

1.41649495	0.04539123	0.38135631	nonDEG	0.56689115	0.39234716	1
0.08716981	0.78893377	1	nonDEG	0.09823275	0.81152897	1
-0.8220692	0.04929023	0.40106555	nonDEG	0.25679551	0.62451171	1
-0.4641248	0.29750767	0.88261266	nonDEG	-0.043657	0.94242861	1
0.22893771	0.36561332	0.9257152	nonDEG	0.27709007	0.44750412	1
0.24076971	0.39232529	0.94700427	nonDEG	0.22205794	0.56004536	1
-4.0731046	0.35610395	0.91924469	nonDEG	4.9879797	0.15720006	1
-0.4623234	0.39414407	0.94836216	nonDEG	0.1302601	0.83754356	1
-3.9929335	0.2663129	0.85062515	nonDEG	3.28816312	0.63905585	1
0.15936644	0.62075059	1	nonDEG	0.20095641	0.61601114	1
0.42485995	0.11387889	0.61671829	nonDEG	0.04267601	0.90838026	1
0.76182061	0.00249102	0.0538027	nonDEG	0.1908668	0.65878159	1
0.26219436	0.28156738	0.86272502	nonDEG	-0.0431786	0.90373317	1
-0.1945592	0.56711819	1	nonDEG	-0.0962431	0.82700439	1
0.24991925	0.70874475	1	nonDEG	-0.6209571	0.41314841	1
-0.2475095	0.30349873	0.88814105	nonDEG	-0.0201486	0.95535357	1
-0.685577	0.24641569	0.82801409	nonDEG	-0.7871343	0.31336512	1
-0.2801099	0.31985511	0.90307822	nonDEG	0.01778302	0.9650858	1
2.4358774	1	1	nonDEG	2.79573391	0.18200431	1
0.67083819	0.00598736	0.1008363	nonDEG	-0.4899531	0.17355207	1
0.76603722	0.00386858	0.07468016	nonDEG	-0.9733978	0.00921268	0.74508797
0.12898405	0.64292339	1	nonDEG	0.49405594	0.17979259	1
-0.2991558	0.54518764	1	nonDEG	-0.3137134	0.60949719	1
-0.0492006	0.90310532	1	nonDEG	-0.1382432	0.77497067	1
-0.4882497	0.10535415	0.59393353	nonDEG	-0.0044882	0.99420318	1
-0.7104932	0.15252151	0.69147041	nonDEG	-0.2109211	0.74225658	1
-0.3065216	0.20470011	0.7787757	nonDEG	-0.0367468	0.9209337	1
0.25228721	0.31876572	0.90147301	nonDEG	-0.1263906	0.73876821	1
0.09337349	0.74226406	1	nonDEG	-0.9093955	0.02855153	1
3.30316929	0.63736355	1	nonDEG	-3.1627704	0.64217117	1
-0.0195497	0.94048991	1	nonDEG	0.06180948	0.8664696	1
0.3679117	0.14681107	0.68223696	nonDEG	0.0369261	0.92099201	1
-0.2176926	0.48106424	0.99426825	nonDEG	0.29925258	0.43964889	1
0.25371757	0.51290698	1	nonDEG	0.06880952	0.88893585	1
0.6082679	0.01614259	0.19925444	nonDEG	0.22822976	0.58723011	1
0.38596182	0.13935447	0.6678262	nonDEG	-0.0913128	0.8038426	1
0.17345951	0.69182869	1	nonDEG	0.5182717	0.31798236	1
-0.0709194	0.77585379	1	nonDEG	-0.1347908	0.7293095	1
0.04496785	0.90908196	1	nonDEG	0.0262508	0.95668969	1
-0.3398411	0.38486681	0.94140822	nonDEG	0.05091081	0.92218745	1
-0.0627676	0.91512726	1	nonDEG	1.02062728	0.07732691	1
0.3016846	0.23088008	0.80934492	nonDEG	0.19690958	0.59516361	1
-0.0040278	0.98837965	1	nonDEG	0.06328511	0.87397654	1

-0.4655279	0.05861252	0.4410772	nonDEG	-0.0604384	0.88072613	1
0.08803987	0.72406942	1	nonDEG	0.03792594	0.91816403	1
-0.3730427	0.13263047	0.65746616	nonDEG	-0.2305608	0.52484595	1
-0.2119145	0.38818411	0.94506715	nonDEG	0.26134803	0.46929347	1
0.34509076	0.78413024	1	nonDEG	0.96414435	0.34928502	1
-0.1108448	0.6654511	1	nonDEG	-0.0735608	0.8413436	1
-0.3707274	0.27979054	0.86183461	nonDEG	0.18837061	0.72007336	1
0.62292238	0.020671	0.23432171	nonDEG	-0.2168216	0.5573626	1
0.22103601	0.36845838	0.92777953	nonDEG	-0.1659507	0.652262	1
-0.0927829	0.70863129	1	nonDEG	-0.1007946	0.78257247	1
0.04708357	0.8657165	1	nonDEG	0.27949093	0.4714239	1
1.31302432	0.05239299	0.41495753	nonDEG	-0.545696	0.49054424	1
0.10554971	0.66644996	1	nonDEG	-0.0690487	0.84897131	1
-0.1984125	0.52701902	1	nonDEG	-0.1112871	0.79017154	1
0.12514075	0.62434755	1	nonDEG	0.45734575	0.25227374	1
0.19405439	0.43680231	0.97402891	nonDEG	-0.1685175	0.64776182	1
-0.0368805	0.88644129	1	nonDEG	0.50102003	0.16915598	1
-0.1801372	0.53218064	1	nonDEG	0.15214163	0.6940362	1
-0.0168829	0.96544037	1	nonDEG	0.24046626	0.58349014	1
0.37867881	0.30514967	0.89006022	nonDEG	0.03942477	0.93106964	1
0.79972071	0.00318031	0.06483741	nonDEG	-0.1350882	0.71559264	1
-0.0490726	0.8868427	1	nonDEG	0.08756751	0.84490865	1
0.08149425	0.94634589	1	nonDEG	-0.6807762	0.48920549	1
-0.2127946	0.39182596	0.94686517	nonDEG	-0.0184196	0.95984931	1
0.18444998	0.49064089	0.99934949	nonDEG	0.04381443	0.90541411	1
-0.7156342	0.01888025	0.22100029	nonDEG	0.58573164	0.13760728	1
-0.3289443	0.30574939	0.89022852	nonDEG	-0.0932843	0.8377531	1
-0.2470437	0.41938279	0.96282267	nonDEG	-0.0749001	0.85474133	1
0.26609568	0.31259541	0.89535767	nonDEG	-0.1412767	0.71790473	1
-0.2533967	0.32685869	0.91069401	nonDEG	-0.4140123	0.26566016	1
-0.061209	0.82316881	1	nonDEG	-0.2525015	0.49693478	1
-0.294334	0.3467723	0.91869633	nonDEG	-0.0155666	0.97122475	1
-0.1350886	0.63990328	1	nonDEG	0.08425986	0.82172658	1
0.38999322	0.32787146	0.9118557	nonDEG	0.07460415	0.87464184	1
0.2137608	0.40281765	0.9535508	nonDEG	0.06606861	0.85574179	1
-0.2431822	0.62568595	1	nonDEG	-0.0323631	0.96885334	1
0.63092748	0.39157074	0.94686517	nonDEG	-0.0910616	0.91373817	1
-0.0692864	0.9117903	1	nonDEG	-1.5018231	0.03862324	1
0.1349851	0.58059241	1	nonDEG	0.02364531	0.94962443	1
-0.1021517	0.68995811	1	nonDEG	-0.0580095	0.87503481	1
0.19627022	0.53625341	1	nonDEG	-0.0261693	0.95042244	1
-1.7921412	0.05545824	0.42772728	nonDEG	1.22292494	0.19732594	1
0.08842036	0.71918465	1	nonDEG	-0.0766017	0.83224103	1

-0.4847977	0.11503607	0.6194525	nonDEG	-0.0148872	0.97348785	1
-0.0057862	1	1	nonDEG	-0.2842986	0.71358366	1
0.13080506	0.68240086	1	nonDEG	0.07032602	0.86391303	1
-0.1056757	0.79353252	1	nonDEG	0.34630957	0.48154402	1
4.23476872	0.34552608	0.91869633	nonDEG	1.46723372	0.55334942	1
0.37695131	0.30163742	0.88648699	nonDEG	0.29305502	0.50917469	1
-0.182633	0.94928441	1	nonDEG	1.77056996	0.18549596	1
-0.0744065	0.8146285	1	nonDEG	-0.090207	0.82832718	1
-0.9753537	0.07910294	0.51520168	nonDEG	-1.1936397	0.08543146	1
0.40688647	0.11945849	0.63063362	nonDEG	0.20929314	0.62675165	1
-0.0782257	0.85383164	1	nonDEG	0.48538432	0.32044655	1
-0.3305983	0.51455864	1	nonDEG	0.49514012	0.39618019	1
0.10100052	0.73017529	1	nonDEG	0.13561106	0.72482903	1
-0.0925958	0.74032564	1	nonDEG	-0.1324723	0.72814628	1
0.11513868	0.65548555	1	nonDEG	0.12985129	0.72210343	1
3.3031378	0.63708648	1	nonDEG	-0.8472588	1	1
0.58150998	0.02275597	0.24961389	nonDEG	-0.2805699	0.50021361	1
-0.1250288	0.81458342	1	nonDEG	-0.7184545	0.25002991	1
0.54671079	0.03243913	0.31304778	nonDEG	0.00018908	1	1
0.24383441	0.33032935	0.91411259	nonDEG	-0.0258877	0.94400689	1
0.07850583	0.79485959	1	nonDEG	0.08264834	0.83593522	1
-0.217304	0.45162338	0.98329655	nonDEG	-0.2404523	0.54583311	1
0.7077848	0.00704964	0.11334479	nonDEG	-0.8642291	0.05805822	1
0.04602449	0.87940335	1	nonDEG	-0.3027115	0.4909848	1
0.24836793	0.51429939	1	nonDEG	0.2778961	0.54269633	1
0.49419987	0.05045965	0.40607126	nonDEG	0.06477465	0.87030334	1
-0.052608	0.94358551	1	nonDEG	-0.5483197	0.48307483	1
-0.0357922	0.90740674	1	nonDEG	0.21925763	0.58394379	1
-0.0779279	0.75481356	1	nonDEG	0.29788892	0.41088445	1
0.27630311	0.3762993	0.93394411	nonDEG	0.15974879	0.68517026	1
-0.5561541	0.35335684	0.91917773	nonDEG	0.13254263	0.86726208	1
-0.5329899	0.12673522	0.64672526	nonDEG	-0.2414508	0.59744349	1
0.67402352	0.41064635	0.95836418	nonDEG	-0.7728073	0.46309509	1
-0.1867173	0.52014402	1	nonDEG	-0.6148212	0.15152656	1
-0.8225829	0.58254915	1	nonDEG	-2.775584	0.14387271	1
0.32369786	0.2745409	0.85818661	nonDEG	0.08396966	0.82956345	1
-0.2187366	0.45202306	0.98329655	nonDEG	0.02592491	0.94805377	1
-0.0850259	0.76385142	1	nonDEG	0.22502351	0.55217662	1
0.33645802	0.18477253	0.75256203	nonDEG	-0.050154	0.89617274	1
0.07054177	0.79323276	1	nonDEG	0.02529603	0.94720648	1
-0.234564	0.37373414	0.93237716	nonDEG	-0.5189943	0.18320519	1
-0.5966222	0.78382001	1	nonDEG	-0.5421102	0.85134561	1
0.69968573	0.09811865	0.57137375	nonDEG	-1.567858	0.00366312	0.49008216

0.68240882	0.0559843	0.43043138	nonDEG	-0.8378099	0.05912511	1
-0.1211559	0.64845666	1	nonDEG	0.07058362	0.86761439	1
-0.213187	0.46410715	0.98553614	nonDEG	0.52775342	0.1800381	1
-0.0049239	0.98757427	1	nonDEG	0.05111263	0.89612982	1
0.12212815	0.62000305	1	nonDEG	0.31038385	0.39576742	1
-0.1183981	0.68376253	1	nonDEG	0.11779313	0.76790438	1
-0.0664081	0.81482731	1	nonDEG	-0.6574575	0.09519987	1
-0.6910714	0.51988823	1	nonDEG	0.86233371	0.44261386	1
-0.0281908	0.90797703	1	nonDEG	-0.189318	0.61877256	1
0.1141769	0.74244021	1	nonDEG	0.05699812	0.89537578	1
-0.0520802	0.83282494	1	nonDEG	-0.1083851	0.76378554	1
-0.2619025	0.3294345	0.91339798	nonDEG	0.37506457	0.34119242	1
0.09391101	0.77022969	1	nonDEG	0.29381898	0.4820935	1
-1.4746916	0.12460545	0.64340522	nonDEG	0.64705138	0.51485436	1
-1.3650261	0.41025976	0.95836418	nonDEG	-0.3009808	1	1
-0.1512452	0.54502077	1	nonDEG	-0.4198388	0.26181457	1
-0.0242473	0.9366288	1	nonDEG	-0.2073183	0.60297686	1
0.13822366	0.68520012	1	nonDEG	-0.3829418	0.42722418	1
-0.1522333	0.53843116	1	nonDEG	-0.051127	0.88737896	1
0.02474303	0.92167474	1	nonDEG	0.18202418	0.61305873	1
0.3703403	0.13286907	0.65773207	nonDEG	0.15205988	0.70106564	1
0.30928248	0.5768022	1	nonDEG	0.21498855	0.73836705	1
0.88076067	0.23974991	0.8216528	nonDEG	0.85410296	0.38565951	1
-0.4098793	0.15547975	0.69827131	nonDEG	0.72713688	0.07315089	1
0.13285214	0.85765301	1	nonDEG	-0.2075242	0.80222019	1
-0.0115786	0.97954087	1	nonDEG	-0.6538761	0.22995449	1
0.38404032	0.11366192	0.61607445	nonDEG	0.08547526	0.81948381	1
-0.2913207	0.36133244	0.92354131	nonDEG	0.13443337	0.74207518	1
0.01512404	0.95707006	1	nonDEG	0.23088939	0.57844981	1
-0.4577881	0.07496168	0.50188576	nonDEG	-0.0178129	0.96221562	1
2.02666265	0.27406459	0.8576791	nonDEG	0.64028995	0.71590425	1
-0.4722325	0.08792556	0.54219536	nonDEG	-0.0061166	0.9888177	1
-0.0994503	0.71160037	1	nonDEG	0.31992206	0.37980845	1
0.07944058	0.81013278	1	nonDEG	0.26033247	0.56709973	1
-0.2921969	0.88434102	1	nonDEG	0.90874549	0.44996583	1
0.26202429	0.44975614	0.98329655	nonDEG	0.17860371	0.66791456	1
0.7656614	0.12414001	0.64205162	nonDEG	-0.0405561	0.94847741	1
0.15532083	0.72050877	1	nonDEG	-0.4245723	0.43544957	1
0.03603412	0.88690056	1	nonDEG	0.10900199	0.80107706	1
0.53691672	0.20208644	0.77400024	nonDEG	-0.2729127	0.60467575	1
0.41555851	0.13616015	0.66318137	nonDEG	0.21332413	0.55831778	1
-0.3774194	0.38272345	0.93894257	nonDEG	0.11014958	0.85365983	1
-0.2240106	0.6891378	1	nonDEG	-0.4993847	0.44860891	1

-0.2513692	0.36679474	0.92635678	nonDEG	-0.692415	0.07621385	1
-0.0618896	0.80429703	1	nonDEG	0.10541461	0.77101914	1
0.17456701	0.51113373	1	nonDEG	-0.062241	0.86605026	1
-1.138976	0.09686068	0.56836385	nonDEG	-0.6494046	0.39908544	1
0.14219024	0.55774469	1	nonDEG	-0.2028719	0.57155465	1
0.23709686	0.33370267	0.91729884	nonDEG	0.00079776	0.99876349	1
-0.9305368	0.3661646	0.92599625	nonDEG	-0.0358408	1	1
-0.2258104	0.36111594	0.92354131	nonDEG	0.0990313	0.81898276	1
-0.0700761	0.92129337	1	nonDEG	-0.6252193	0.36270399	1
-0.3559043	0.25159782	0.83371668	nonDEG	0.85724238	0.04816181	1
0.05135618	0.92007154	1	nonDEG	0.08998082	0.87917316	1
0.24915729	0.62704764	1	nonDEG	-0.3792164	0.53288843	1
0.05315787	0.87167026	1	nonDEG	0.13092553	0.75476264	1
0.08189978	0.74494535	1	nonDEG	0.2940304	0.41504145	1
-0.3826687	0.45667663	0.98426953	nonDEG	0.23829689	0.69214275	1
0.086075	0.86203167	1	nonDEG	-0.2987467	0.60056934	1
-0.0184938	0.98446655	1	nonDEG	0.37627171	0.60827897	1
0.07952915	0.87326611	1	nonDEG	0.61992167	0.25822878	1
-0.1316708	0.64863631	1	nonDEG	-0.4396126	0.29166278	1
-0.1295902	0.60406444	1	nonDEG	0.0185418	0.95971061	1
-0.0765913	0.75537563	1	nonDEG	0.0808783	0.82388814	1
-0.302666	0.57471869	1	nonDEG	0.10825661	0.85740106	1
-0.044019	0.86920898	1	nonDEG	-0.1110725	0.80629015	1
0.22636739	0.36127113	0.92354131	nonDEG	0.1670453	0.64456587	1
0.79112365	0.18681232	0.75310345	nonDEG	-0.3863072	0.56316891	1
0.04521911	0.89375825	1	nonDEG	-0.0719021	0.87296397	1
0.10871565	0.79576651	1	nonDEG	-0.119941	0.82770683	1
0.1909322	0.48633669	0.99711758	nonDEG	-0.3365821	0.36896282	1
0.68297241	0.32631073	0.91057556	nonDEG	-0.2049251	0.83723079	1
-0.3296727	0.34377735	0.91869633	nonDEG	-0.1397693	0.75645197	1
0.08389599	0.81669963	1	nonDEG	0.22872883	0.59530073	1
1.7741159	0.2421791	0.82422088	nonDEG	1.20319685	0.46906225	1
-0.4100198	0.19324457	0.76320458	nonDEG	-0.3410698	0.42956931	1
-0.4026265	0.19748849	0.76905736	nonDEG	-0.0137	0.97545423	1
-0.4209057	0.15029475	0.68725272	nonDEG	-0.1868875	0.67436805	1
-0.1308277	0.65993562	1	nonDEG	-0.2141821	0.59767787	1
0.24183938	0.32555405	0.90964177	nonDEG	-0.2244485	0.53337654	1
-0.4230998	0.31512824	0.89824365	nonDEG	0.37912063	0.47319132	1
-0.0669311	0.79662142	1	nonDEG	-0.0394425	0.91513982	1
5.15705653	0.05044208	0.40607126	nonDEG	-0.3986856	0.89622114	1
-0.0713951	0.81012962	1	nonDEG	-0.3969579	0.31665783	1
-0.8596375	0.04674159	0.38914754	nonDEG	-0.338347	0.56292865	1
0.00655864	0.98047575	1	nonDEG	0.05529973	0.8813305	1

-0.3068468	0.38662333	0.94337374	nonDEG	-0.5041767	0.21458129	1
-0.3090428	0.23619122	0.81815704	nonDEG	0.47034358	0.19963937	1
-0.1007717	0.68233709	1	nonDEG	9.50E-05	0.9999911	1
0.06738496	0.8366632	1	nonDEG	0.19218754	0.63821495	1
-0.0702444	0.83072046	1	nonDEG	0.00428538	0.99459509	1
-0.2068162	0.43662596	0.97402891	nonDEG	-0.3855276	0.33145502	1
-0.4439282	0.12348138	0.64067117	nonDEG	0.06375807	0.87792719	1
-3.958172	0.27711045	0.85917372	nonDEG	3.14199857	0.6428654	1
0.77479467	0.03423748	0.32354346	nonDEG	0.17871834	0.67562102	1
0.8422301	0.46896669	0.98754723	nonDEG	0.4563625	0.69735628	1
0.30496059	0.35193428	0.91917773	nonDEG	0.46287611	0.22731715	1
-0.6627229	0.37429298	0.93251128	nonDEG	-0.1730066	0.84802204	1
-0.1606314	0.58792907	1	nonDEG	0.40825411	0.31378746	1
1.1888582	0.08169511	0.52307859	nonDEG	0.10287222	0.90416995	1
0.25799136	0.29771107	0.88261266	nonDEG	0.22128519	0.57472488	1
-0.299787	0.40014298	0.95204649	nonDEG	0.45485609	0.30797923	1
-0.5383421	0.03435607	0.32416934	nonDEG	-0.0205405	0.96153908	1
0.2527222	0.82140188	1	nonDEG	-0.1174124	0.9178006	1
-0.0169333	1	1	nonDEG	0.09852768	0.93406128	1
0.84368784	0.36907253	0.92832738	nonDEG	0.56924263	0.53113732	1
-0.3787018	0.12037457	0.63156406	nonDEG	-0.1581844	0.66731743	1
-0.0703585	0.79935442	1	nonDEG	0.36787558	0.34146891	1
0.02127436	0.97010805	1	nonDEG	0.13442712	0.81836477	1
0.54296241	0.02897644	0.29203612	nonDEG	0.16866913	0.6494948	1
-0.0931143	0.74557762	1	nonDEG	0.20356855	0.58949834	1
-0.097073	0.71631344	1	nonDEG	0.37918029	0.31924853	1
-0.419073	0.24872462	0.82990493	nonDEG	-0.1718382	0.72204792	1
-0.0882832	0.73377957	1	nonDEG	0.19857057	0.58994937	1
-0.1978822	0.7187277	1	nonDEG	-0.332876	0.7178361	1
-0.0482477	0.87362434	1	nonDEG	-0.4477844	0.25901911	1
-0.0111055	0.96698662	1	nonDEG	-0.0774186	0.83134415	1
-0.4676223	0.66579781	1	nonDEG	-0.8739344	0.51858907	1
0.33532241	0.41074062	0.95836418	nonDEG	0.23173254	0.63742008	1
0.4323306	0.09415398	0.55959186	nonDEG	0.28410788	0.54382438	1
-0.6567903	0.38405445	0.94057051	nonDEG	0.17106314	0.85863325	1
-0.2504494	0.41475028	0.96053724	nonDEG	0.03000585	0.95001487	1
-0.1721178	0.55409384	1	nonDEG	-0.8662446	0.02618327	1
-0.3243875	0.33038344	0.91411259	nonDEG	0.3665887	0.45570077	1
-0.0232612	0.92885719	1	nonDEG	-0.1085244	0.77051669	1
0.2571786	0.72010668	1	nonDEG	-0.0753947	0.92361454	1
-0.3691511	0.30384828	0.88863396	nonDEG	0.11425563	0.82609158	1
-0.063017	0.79942037	1	nonDEG	-0.1092271	0.76357339	1
-0.1442036	0.55987857	1	nonDEG	0.02178552	0.95322229	1

-0.007338	0.98208738	1	nonDEG	0.22867201	0.57625737	1
0.24882809	0.33305568	0.91702453	nonDEG	0.24716415	0.50068867	1
-0.2581216	0.29125054	0.87342689	nonDEG	-0.0665922	0.85304818	1
-0.1211146	0.62875769	1	nonDEG	-0.0454573	0.90413379	1
0.34015589	0.28341579	0.86569297	nonDEG	0.03675855	0.9319416	1
0.03267099	0.90878347	1	nonDEG	-0.2549287	0.5040394	1
0.44340172	0.6180002	1	nonDEG	-0.5659218	0.5567355	1
-0.3153566	0.35127443	0.91917773	nonDEG	-0.4235353	0.30052113	1
-0.1023672	0.75911385	1	nonDEG	-0.0113088	0.9798956	1
-0.4037813	0.38027354	0.93639703	nonDEG	0.13847659	0.80818841	1
-0.222536	0.36312145	0.92401525	nonDEG	0.16331579	0.70552501	1
0.08912854	0.72334197	1	nonDEG	0.08732873	0.8339871	1
0.20345931	0.68773628	1	nonDEG	0.00858198	1	1
-0.593553	0.01613242	0.19925444	nonDEG	-0.0019094	0.99603335	1
-0.4079064	0.31880279	0.90147301	nonDEG	-0.0261392	0.9594792	1
-0.5642113	0.28955364	0.87132471	nonDEG	0.09238996	0.90087582	1
-0.0648679	0.81129624	1	nonDEG	0.30132237	0.41341122	1
0.13742987	0.6268469	1	nonDEG	-0.0436712	0.91272277	1
-0.7829088	0.03776604	0.34240671	nonDEG	-0.0700773	0.89248016	1
0.14165619	0.5839895	1	nonDEG	-0.2073726	0.57695913	1
0.24905903	0.32020894	0.90328008	nonDEG	0.22592203	0.53716482	1
0.04620635	0.88551632	1	nonDEG	-0.1552198	0.70619308	1
0.09267251	0.77349518	1	nonDEG	-0.1562718	0.7005327	1
0.51904575	0.2699414	0.85268624	nonDEG	-0.1036362	0.86383172	1
0.28289786	0.27811639	0.86008704	nonDEG	-0.0373253	0.91957893	1
-0.397767	0.16346156	0.714154	nonDEG	-0.2388062	0.54782343	1
0.57963643	0.11745031	0.62683992	nonDEG	-0.2604473	0.60823557	1
-0.3824609	0.48908567	0.99836609	nonDEG	-0.0047247	1	1
-2.1968012	1	1	nonDEG	0	1	1
-0.0431012	0.86125615	1	nonDEG	0.44513196	0.30946605	1
0.37753152	0.16845238	0.72437474	nonDEG	0.35869537	0.38804756	1
0.15756024	0.56300614	1	nonDEG	0.12000833	0.74377485	1
-0.8063547	0.04311446	0.36950411	nonDEG	0.38509567	0.48060605	1
-0.446789	0.17182257	0.7304382	nonDEG	0.47930198	0.26347154	1
-0.3873845	0.13241626	0.65711072	nonDEG	0.15402358	0.69273373	1
1.45294314	0.50106264	1	nonDEG	-0.2261377	1	1
-0.1937204	0.49437208	1	nonDEG	0.15912163	0.67756918	1
0.89428541	0.23678084	0.81866798	nonDEG	0.28168167	0.70901448	1
0.08092325	0.8653082	1	nonDEG	-0.4896908	0.48046523	1
0.87754636	0.01141753	0.16051603	nonDEG	-0.3568616	0.38954287	1
0.78400134	0.11606264	0.62292948	nonDEG	-0.0516667	0.92815928	1
0.2240487	0.44183823	0.97799065	nonDEG	0.22640774	0.55839275	1
0.07474493	0.76254972	1	nonDEG	-0.0073941	0.98414889	1

2.59703797	0.1256281	0.64431252	nonDEG	-0.6419538	0.61574893	1
0.15637256	0.68610108	1	nonDEG	0.30299463	0.48906084	1
0.47213749	0.1317749	0.65533095	nonDEG	-0.3319181	0.40548668	1
0.26402914	0.28092312	0.86186717	nonDEG	0.26663769	0.45917975	1
0.32616962	0.20046497	0.77325509	nonDEG	0.57409523	0.13785892	1
0.17991729	0.72737986	1	nonDEG	0.12378206	0.84122495	1
0.35032082	0.43259134	0.97234509	nonDEG	-0.4667119	0.38063426	1
-0.2961349	0.26912488	0.85230023	nonDEG	-0.0235841	0.94986128	1
-0.0917596	0.72441082	1	nonDEG	0.05289899	0.88712202	1
0.28500705	0.24732791	0.82884679	nonDEG	0.32285098	0.37783837	1
-0.2521892	0.39552673	0.9493779	nonDEG	0.12784611	0.74340048	1
0.07318533	0.95382878	1	nonDEG	-0.5279036	0.51548517	1
0.40754593	0.20087316	0.77325509	nonDEG	0.14149344	0.74351629	1
-0.0212747	0.93756993	1	nonDEG	-0.288492	0.43935862	1
-0.3153407	0.62555257	1	nonDEG	-0.8970857	0.22098971	1
0.70833297	0.00389616	0.07487047	nonDEG	0.26919039	0.49086636	1
-0.8050201	0.25652271	0.83849359	nonDEG	0.72183602	0.35688621	1
-0.0829548	0.74467254	1	nonDEG	0.38961084	0.27904994	1
-0.1307532	0.7835572	1	nonDEG	-0.2012698	0.72758865	1
-0.7126966	0.15636759	0.70062224	nonDEG	-0.0700856	0.91048112	1
0.03084859	0.95927719	1	nonDEG	-1.1445446	0.1301795	1
-0.7217767	0.39611011	0.9493779	nonDEG	0.02856638	1	1
0.11408946	0.80161379	1	nonDEG	-0.3078739	0.56348809	1
0.79812492	0.00801761	0.12508453	nonDEG	0.04523673	0.90501129	1
-0.2636261	0.35549283	0.91917773	nonDEG	0.0419227	0.91459817	1
-0.0864308	0.77181669	1	nonDEG	0.19152996	0.62630162	1
0.31983856	0.30561093	0.89022852	nonDEG	0.2778274	0.55208275	1
0.1334393	0.74106804	1	nonDEG	0.34975087	0.45717696	1
-0.3568954	0.56397889	1	nonDEG	-0.2339379	0.76357533	1
0.39377701	0.11824359	0.62833449	nonDEG	0.05095199	0.90454417	1
0.38306734	0.16284403	0.714154	nonDEG	0.21194063	0.59930285	1
-0.0910197	0.79203933	1	nonDEG	0.13838264	0.74314939	1
0.00556017	0.99072343	1	nonDEG	-0.3337319	0.51981905	1
-0.0878923	0.72424708	1	nonDEG	0.19799505	0.59564425	1
-0.5626562	0.55225406	1	nonDEG	0.44323591	0.69656049	1
-0.5641523	0.20140901	0.77336631	nonDEG	-1.192691	0.05038723	1
0.09403225	0.73118623	1	nonDEG	0.01376768	0.97026795	1
1.44301928	0.09945356	0.57437345	nonDEG	-0.4982668	0.58699676	1
-0.0792834	0.76023007	1	nonDEG	0.05966946	0.87084774	1
-0.2071198	0.52553064	1	nonDEG	-0.2114866	0.62240309	1
0.1471585	0.89560974	1	nonDEG	-0.5399732	0.61650683	1
-0.0467907	0.91956206	1	nonDEG	-0.2589867	0.63022341	1
1.1033547	0.48009615	0.99357121	nonDEG	0.06260475	1	1

-0.1116087	0.73453577	1	nonDEG	-0.307341	0.54725598	1
-0.0248501	0.95067185	1	nonDEG	-0.0652981	0.89183504	1
-1.5615564	0.04563657	0.38287119	nonDEG	-3.6143458	0.01045328	0.77777211
-0.1689928	0.4894848	0.99867487	nonDEG	0.14719488	0.68424086	1
0.86378837	0.1088708	0.60347345	nonDEG	-0.0782392	0.89624866	1
-0.3174904	0.21384988	0.79218906	nonDEG	0.2327495	0.52430377	1
0.00091424	0.99736301	1	nonDEG	0.11027782	0.85245682	1
0.05330999	0.85064506	1	nonDEG	-0.2638269	0.49992525	1
-0.3150378	0.23175695	0.81104765	nonDEG	0.00852333	0.98276891	1
-0.3489373	0.68663208	1	nonDEG	-0.4532019	0.6433396	1
-0.1578831	0.55808344	1	nonDEG	0.18015493	0.62438348	1
0.12306639	0.67654253	1	nonDEG	-0.4734194	0.25508798	1
-0.485944	0.09694367	0.56851089	nonDEG	-0.0377468	0.91770978	1
-0.0494629	0.8736937	1	nonDEG	0.18534807	0.62845664	1
-0.5032959	0.04456069	0.37706873	nonDEG	0.14443877	0.7103752	1
0.17973852	0.46237343	0.98479636	nonDEG	0.09755958	0.7891227	1
1.3393218	0.01729556	0.20816186	nonDEG	0.16877328	0.76965875	1
0.0365804	0.88758239	1	nonDEG	0.11353137	0.75972413	1
-0.1189351	0.70849724	1	nonDEG	-0.3074466	0.46297157	1
0.14638314	0.82156029	1	nonDEG	-0.6418012	0.4178566	1
0.81471447	0.0947539	0.56132341	nonDEG	-0.1887131	0.72542141	1
-1.1940864	0.06158296	0.45368887	nonDEG	0.22562061	0.76156655	1
0.3342086	0.60468549	1	nonDEG	-0.454417	0.6071399	1
0.25166998	0.94251294	1	nonDEG	-2.5369936	0.13856933	1
0.00521707	0.98762418	1	nonDEG	-0.1612173	0.70032165	1
0.16629697	0.53037173	1	nonDEG	-0.3399088	0.39000787	1
-0.1358173	0.64963859	1	nonDEG	-0.8082777	0.05941246	1
-0.2646308	0.28210791	0.86339916	nonDEG	0.14798125	0.69571441	1
-0.1568721	0.89555391	1	nonDEG	-0.7986042	0.44126834	1
0.166204	0.64340036	1	nonDEG	-0.5169518	0.26522776	1
0.01783825	0.95022549	1	nonDEG	0.01838782	0.96269348	1
1.03416418	0.18637034	0.75310345	nonDEG	0.71780682	0.375749	1
-0.1384858	0.57253594	1	nonDEG	0.37338776	0.30572416	1
0.60478109	0.11243705	0.61251561	nonDEG	-0.0812674	0.86282015	1
-0.1204955	0.63158053	1	nonDEG	-0.0656807	0.85433601	1
0.57197354	0.0194508	0.22517191	nonDEG	0.24747977	0.49464562	1
-0.2922501	0.31522049	0.89833262	nonDEG	-0.4074371	0.29122694	1
0.7227932	0.3223672	0.90520806	nonDEG	-0.644379	0.43625025	1
0.07778197	0.79203893	1	nonDEG	0.06828549	0.85381204	1
0.27937164	0.87034196	1	nonDEG	-2.2362479	0.1638287	1
-0.0269771	0.91862463	1	nonDEG	0.30012233	0.43132303	1
0.11831719	0.83109404	1	nonDEG	-0.4133369	0.52055278	1
-0.401029	0.13187796	0.65569249	nonDEG	-0.0799287	0.82498046	1

-0.3679793	0.80571768	1	nonDEG	-0.0375164	1	1
-0.2802286	0.29462941	0.87816091	nonDEG	0.33680403	0.40063211	1
-0.1185801	0.69668129	1	nonDEG	-0.2821735	0.48745887	1
-0.7842268	0.04656554	0.38845083	nonDEG	-0.1601956	0.76776285	1
-0.2532908	0.3185877	0.90147301	nonDEG	-0.1183383	0.7476517	1
0.06617001	0.80076249	1	nonDEG	0.0071743	0.98518899	1
0.43384064	0.78341515	1	nonDEG	-2.6355205	0.24017485	1
-0.2860378	0.34467029	0.91869633	nonDEG	-0.2982364	0.49271511	1
-0.9695277	0.07094218	0.48669278	nonDEG	-0.5749444	0.41686283	1
0.67693475	0.33549743	0.91869633	nonDEG	-1.3783315	0.09362429	1
-0.7553882	0.03594074	0.33324703	nonDEG	-0.2063893	0.7140701	1
-0.0021846	1	1	nonDEG	-0.0282816	0.96294175	1
-0.2218158	0.4462828	0.98124647	nonDEG	-0.6482965	0.10912847	1
-0.296135	0.25635993	0.83846016	nonDEG	-0.1901495	0.6120715	1
-0.1417615	0.68469277	1	nonDEG	0.33525423	0.44240611	1
0.196881	0.4986526	1	nonDEG	-0.6098766	0.13204926	1
0.20192608	0.65660761	1	nonDEG	-0.1003224	0.86276548	1
-0.1955694	0.41844409	0.96254612	nonDEG	0.21898313	0.61592578	1
0.18849486	0.50801752	1	nonDEG	-0.0811233	0.83847349	1
0.30928663	0.35325683	0.91917773	nonDEG	-0.5408671	0.21409965	1
0.39885984	0.12583912	0.64460739	nonDEG	0.23188252	0.52279593	1
0.22804954	0.60790905	1	nonDEG	-0.2955273	0.58415052	1
-0.151119	0.59247338	1	nonDEG	0.23821964	0.53533865	1
0.34671849	0.14949879	0.68626577	nonDEG	0.10534279	0.76817132	1
-0.3767046	0.18607847	0.75310345	nonDEG	-0.1686979	0.66811906	1
0.56733883	0.20379373	0.77713519	nonDEG	-0.8673725	0.10775452	1
-1.3012045	0.10995145	0.60566492	nonDEG	-0.9039265	0.54941484	1
0.22686719	0.36514677	0.92544853	nonDEG	-0.1112581	0.78073502	1
-0.41084	0.77682061	1	nonDEG	0.32654083	1	1
1.47603767	0.11013901	0.60578707	nonDEG	-1.226375	0.13437373	1
-0.1226603	0.62972783	1	nonDEG	0.08259704	0.82162711	1
0.1703509	0.50921851	1	nonDEG	-0.1272254	0.72994133	1
0.25842738	0.67300194	1	nonDEG	0.61658137	0.34327466	1
0.07824442	0.79261661	1	nonDEG	-0.0371355	0.93339787	1
0.36286218	0.22719814	0.80901407	nonDEG	0.10178958	0.78723441	1
-0.4359624	0.33533382	0.91869633	nonDEG	-0.9511178	0.20014426	1
-0.3507319	0.42113953	0.96396459	nonDEG	0.50904811	0.38816559	1
0.21317158	0.42206637	0.96420737	nonDEG	-0.0769387	0.8334773	1
-0.7621388	0.02555326	0.26914578	nonDEG	-0.0246622	0.95725302	1
0.19132544	0.44834953	0.98329655	nonDEG	0.55537959	0.16307607	1
0.4515895	0.07384154	0.49675982	nonDEG	0.01804442	0.96103221	1
-0.9232052	0.18333496	0.75072226	nonDEG	-0.108578	0.92639022	1
0.19354193	0.59609718	1	nonDEG	-1.423676	0.00238233	0.38006158

1.19527305	0.07051637	0.48583328	nonDEG	-0.3860104	0.6107477	1
0.15593505	0.52091995	1	nonDEG	0.18402304	0.60865478	1
0.79237812	0.00356874	0.07070004	nonDEG	0.295786	0.42134876	1
-0.0170616	0.97305218	1	nonDEG	-0.0373144	0.94637338	1
-0.2214374	0.40038687	0.95217618	nonDEG	0.00192372	0.9965583	1
-0.4010187	0.1884818	0.7541065	nonDEG	-0.0437929	0.91460903	1
0.30895792	0.49213132	0.99992089	nonDEG	-1.1192795	0.04464975	1
0.4254175	0.125156	0.64413767	nonDEG	-0.4164974	0.26995927	1
0.25001914	1	1	nonDEG	1.20664959	0.66163542	1
-0.2693753	0.29122851	0.87342689	nonDEG	0.38476912	0.33202877	1
0.35017586	0.20070078	0.77325509	nonDEG	-0.0210937	0.95748678	1
0.28337942	0.41029766	0.95836418	nonDEG	0.38771413	0.33909726	1
0.31411552	0.33379224	0.91729884	nonDEG	-0.3230961	0.41987863	1
-0.3120781	0.38915554	0.94606909	nonDEG	0.07724905	0.86825321	1
-0.0936654	0.88482734	1	nonDEG	-1.6915076	0.02267214	1
0.19166568	0.51418175	1	nonDEG	-0.0112953	0.97898756	1
0.48484751	0.07410154	0.49784845	nonDEG	-0.1212994	0.74552105	1
-0.3159436	0.32105826	0.90420302	nonDEG	-0.0603754	0.88596963	1
-0.8638846	0.1330843	0.65824407	nonDEG	-0.1579494	0.84236729	1
0.02562381	0.92454538	1	nonDEG	-0.686568	0.07659101	1
-0.2480441	0.31075193	0.89435467	nonDEG	0.15372049	0.67794496	1
-0.1077545	0.87004725	1	nonDEG	-0.4397457	0.53037362	1
-0.0184188	0.94411726	1	nonDEG	-0.1288186	0.72291886	1
0.59047927	0.02628184	0.27436565	nonDEG	0.19462546	0.59551523	1
0.26241986	0.30525079	0.89006022	nonDEG	-0.1051509	0.77521559	1
-0.391909	0.58800596	1	nonDEG	0.40198037	0.61080771	1
0.13022429	0.78149774	1	nonDEG	0.34025309	0.52899457	1
0.6354905	0.01633639	0.20052881	nonDEG	-0.0058071	0.98826657	1
0.19454466	0.44432999	0.97965485	nonDEG	0.25877352	0.52130656	1
0.34237339	0.19254203	0.76192362	nonDEG	-0.0110069	0.97660662	1
0.64530152	0.16022337	0.70930196	nonDEG	-0.2567878	0.6336201	1
0.3579333	0.13571854	0.66261751	nonDEG	0.29733863	0.41455254	1
1.11051493	0.09971676	0.57443019	nonDEG	-2.1444213	0.00507947	0.56641847
0.23111877	0.62721778	1	nonDEG	0.39836609	0.46521581	1
-0.4125469	0.09152251	0.55189083	nonDEG	-0.2350915	0.5103731	1
0.0015001	0.99640882	1	nonDEG	-0.7362521	0.04819451	1
0.2025075	0.56850976	1	nonDEG	0.33271957	0.44290702	1
-0.7371825	0.29150798	0.8736995	nonDEG	0.05541614	0.95661561	1
-0.2978307	0.22683837	0.80856968	nonDEG	0.26798591	0.51878989	1
-0.1542086	0.60801365	1	nonDEG	0.29442252	0.44598311	1
-0.20804	0.6897793	1	nonDEG	-0.4187683	0.51124266	1
0.42896174	0.13159813	0.65533095	nonDEG	0.03818097	0.92628078	1
-1.033218	0.06397728	0.46254672	nonDEG	-0.3606721	0.61406931	1

0.06030361	0.83896778	1	nonDEG	0.42241484	0.26323385	1
0.39773614	0.14680475	0.68223696	nonDEG	0.091594	0.80824985	1
-0.3163723	0.2287042	0.80924485	nonDEG	-0.0367349	0.92315413	1
0.17544174	0.58747161	1	nonDEG	-0.1455132	0.74467022	1
-0.4190491	0.41096027	0.95836418	nonDEG	-0.1092971	0.860316	1
0.07535034	0.7591903	1	nonDEG	-0.3781327	0.29615181	1
0.36352989	0.21041059	0.78707463	nonDEG	0.06043995	0.87079923	1
-0.1472361	0.64119809	1	nonDEG	0.1647505	0.72193238	1
0.01956957	1	1	nonDEG	-0.5253384	0.70893822	1
-0.0429856	0.86795418	1	nonDEG	0.1750975	0.63011306	1
-0.0744946	1	1	nonDEG	-0.4442142	0.84389697	1
-0.2056214	0.44892284	0.98329655	nonDEG	0.22969141	0.5363458	1
0.16761769	0.50117196	1	nonDEG	-0.1307051	0.73041377	1
0.2830252	0.31445437	0.89780061	nonDEG	0.00112226	0.99908821	1
0.01753089	0.94635479	1	nonDEG	-0.3265274	0.37072675	1
-0.0313514	0.89615294	1	nonDEG	0.38672879	0.35436081	1
0.07661805	0.76636434	1	nonDEG	-0.039207	0.91712988	1
0.03920828	0.9536193	1	nonDEG	-0.0053924	1	1
0.83364298	0.08815231	0.54280584	nonDEG	-0.2368528	0.66636045	1
0.00714098	0.98018178	1	nonDEG	0.48531447	0.21345632	1
0.11122078	0.72891924	1	nonDEG	0.1231493	0.76497847	1
0.14671401	0.64874935	1	nonDEG	0.09758288	0.81341473	1
-0.1433509	0.56311624	1	nonDEG	-0.0413185	0.91034955	1
-0.2901141	0.65871912	1	nonDEG	0.58560577	0.3680331	1
-0.3980099	0.14375738	0.67635628	nonDEG	0.60756731	0.14367799	1
0.01708365	0.96524702	1	nonDEG	0.21729645	0.62027553	1
0.32679224	0.48638959	0.99711758	nonDEG	-0.288186	0.59803816	1
-0.1925953	0.72955566	1	nonDEG	0.05959127	0.93499706	1
-1.6682646	0.00498711	0.08828356	nonDEG	0.05740182	0.96501344	1
-0.2031388	0.47939413	0.99334042	nonDEG	0.27853885	0.46728003	1
0.22183464	0.64646592	1	nonDEG	-0.4496542	0.47885823	1
-0.1612297	0.52804014	1	nonDEG	-0.0570887	0.8766532	1
-1.1157022	0.14811989	0.68428235	nonDEG	-0.1336938	0.93341616	1
0.18355224	0.46313773	0.98479636	nonDEG	0.05779142	0.87903005	1
0.63352417	0.01221302	0.16746122	nonDEG	-0.0742824	0.84079061	1
0.27493351	0.29858361	0.88387501	nonDEG	0.23073018	0.54976048	1
-0.0002668	1	1	nonDEG	-0.4797672	0.32160533	1
-0.1258356	0.60982118	1	nonDEG	0.01878473	0.95906994	1
0.59716397	0.01606234	0.19884742	nonDEG	0.14030507	0.69990376	1
0.29274253	0.73606304	1	nonDEG	-1.0749189	0.27611267	1
-0.1087518	0.70895924	1	nonDEG	-0.0415393	0.91645592	1
0.19254597	0.55041197	1	nonDEG	-0.6762578	0.10776237	1
0.29405178	0.28423518	0.86665624	nonDEG	-0.4879527	0.20264336	1

-0.388465	0.27215055	0.85514356	nonDEG	-0.6694394	0.15772199	1
-0.0556589	0.83082396	1	nonDEG	0.17678086	0.63615979	1
-0.0998462	0.76302762	1	nonDEG	0.24458289	0.55419069	1
0.42659481	0.0837555	0.52900194	nonDEG	-0.1872798	0.60332235	1
0.24218259	0.35023471	0.91917773	nonDEG	-0.1945966	0.59748957	1
-0.3754424	0.1700691	0.72771285	nonDEG	0.08197397	0.83031182	1
0.755807	0.00320148	0.06517875	nonDEG	0.2070227	0.59887721	1
0.51912657	0.1067341	0.59816202	nonDEG	0.49910648	0.18581712	1
-1.1580221	0.06189625	0.45519951	nonDEG	-0.4366287	0.51642077	1
0.82492042	0.07785836	0.510602	nonDEG	-0.3592721	0.53510293	1
-0.0856122	0.73385558	1	nonDEG	-0.0579331	0.87409316	1
0.07157575	0.77110956	1	nonDEG	-0.5077201	0.18472591	1
0.07454465	0.76749372	1	nonDEG	-0.212979	0.58960773	1
0.09909598	0.84296122	1	nonDEG	0.18008647	0.74693863	1
0.48206501	0.16608888	0.71927717	nonDEG	0.50195865	0.21420195	1
0.53594719	0.05866276	0.44117151	nonDEG	0.19427205	0.65134591	1
-0.3520999	0.3550988	0.91917773	nonDEG	-0.0058631	0.99651705	1
-0.1553447	0.68398057	1	nonDEG	-0.1489857	0.80454907	1
-0.4052767	0.14387017	0.67658051	nonDEG	0.5720018	0.13461203	1
-0.230567	0.49199055	0.99983549	nonDEG	0.15289866	0.72570652	1
0.06040202	0.81141812	1	nonDEG	0.01387238	0.96988093	1
0.05247168	0.84466477	1	nonDEG	-0.2571379	0.48677066	1
0.0348337	0.95023101	1	nonDEG	-0.9057127	0.16615058	1
0.94610479	0.00726366	0.11608849	nonDEG	0.06054055	0.90571265	1
-1.6263872	0.08198645	0.52389822	nonDEG	1.07866453	0.38751496	1
-0.1277925	0.74188273	1	nonDEG	-0.2069844	0.69317024	1
-0.2517043	0.34587472	0.91869633	nonDEG	0.18771483	0.6144298	1
0.26948393	0.2739526	0.8576791	nonDEG	-0.2697409	0.47100465	1
-0.3956169	0.45310194	0.98329655	nonDEG	-0.3502789	0.58676949	1
0.75348852	0.18965155	0.75575547	nonDEG	-0.5030133	0.44439619	1
-0.1878985	0.47996335	0.99343606	nonDEG	0.09816075	0.79097067	1
-0.321917	0.40552772	0.95544464	nonDEG	-0.4168924	0.41139168	1
-0.3673952	0.22909015	0.80924485	nonDEG	-0.1165415	0.79455297	1
0.52245283	0.04699991	0.3901947	nonDEG	-0.1460915	0.69984292	1
0.60007999	0.14750207	0.68332382	nonDEG	0.18733072	0.71136471	1
0.16382447	0.54753684	1	nonDEG	0.19040584	0.61525861	1
0.96622497	0.11488732	0.61909498	nonDEG	0.62630757	0.2756386	1
0.49387098	0.07413865	0.49784845	nonDEG	0.39609433	0.26995451	1
-0.102753	0.7507437	1	nonDEG	-0.0647552	0.87757299	1
-0.5340247	0.21372483	0.79192515	nonDEG	-0.2198009	0.69351664	1
-0.4225662	0.14910797	0.6856099	nonDEG	-0.1739752	0.68940705	1
-0.2504672	0.33130181	0.91476214	nonDEG	-0.1182364	0.75629067	1
0.47832258	0.52290164	1	nonDEG	0.93157737	0.19272056	1

0.12336143	0.68183622	1	nonDEG	0.04322475	0.91508232	1
0.16205383	0.56371466	1	nonDEG	-0.1161151	0.75875001	1
-0.1483152	0.61841888	1	nonDEG	0.24313005	0.53647846	1
0.11768761	0.96014495	1	nonDEG	-2.3896239	0.08198655	1
-0.232433	0.61200565	1	nonDEG	0.21739118	0.6935051	1
-0.3608731	0.21141396	0.78802424	nonDEG	-0.0938304	0.80424865	1
0.40490095	0.20758565	0.78320256	nonDEG	-0.0882469	0.82969467	1
0.22545131	0.36703619	0.926574	nonDEG	0.01099008	0.9767558	1
0.02831943	0.93208414	1	nonDEG	0.31917643	0.46611856	1
-0.1718033	0.55743154	1	nonDEG	0.1014829	0.79227483	1
0.38315893	0.21822256	0.79665849	nonDEG	-0.1463683	0.72093896	1
-0.2815361	0.28231415	0.86375455	nonDEG	0.05423411	0.88801965	1
-0.3146998	0.21505782	0.7942651	nonDEG	0.18668918	0.66411498	1
-1.7310064	0.02966394	0.29612643	nonDEG	-2.031067	0.06054941	1
-0.5057062	0.47652195	0.99238326	nonDEG	0.20178493	0.81635006	1
0.71747602	0.07696083	0.50793676	nonDEG	-0.1172866	0.80170048	1
-0.0931757	0.69920876	1	nonDEG	0.18185493	0.62166599	1
-0.1519671	0.55029507	1	nonDEG	-0.223512	0.56967538	1
-0.4606447	0.20496692	0.77892413	nonDEG	1.56865367	0.00106548	0.2294705
2.29708396	0.40324101	0.95382347	nonDEG	-1.1984208	0.63878667	1
-0.3754105	0.28976751	0.87161191	nonDEG	0.01361349	0.97790665	1
-0.0255828	0.92135599	1	nonDEG	0.23970627	0.50837498	1
-0.4737795	0.07591878	0.50462249	nonDEG	0.04549433	0.90616	1
0.30656723	0.20948179	0.78572136	nonDEG	0.08049241	0.82747995	1
0.2568863	0.33853377	0.91869633	nonDEG	-0.4153733	0.26875217	1
0.18422043	0.68939833	1	nonDEG	0.60730861	0.23496261	1
0.34556579	0.31389546	0.89672669	nonDEG	0.10477677	0.80203881	1
-0.2720231	0.34314721	0.91869633	nonDEG	0.26878301	0.50982953	1
-0.692557	0.25289274	0.83393344	nonDEG	0.12971872	0.86364067	1
-0.5562681	0.08372155	0.52900194	nonDEG	-0.2018719	0.67246555	1
-0.0384367	0.89298835	1	nonDEG	0.21394148	0.5709854	1
0.43206934	0.24194278	0.82401585	nonDEG	0.3347486	0.43132637	1
-0.2071371	0.41401976	0.95994306	nonDEG	-0.3029011	0.40882992	1
0.71083261	0.08087509	0.52035682	nonDEG	0.36200452	0.42561271	1
-0.0556148	0.84130194	1	nonDEG	0.17693356	0.63256428	1
-0.3086563	0.22411581	0.8048125	nonDEG	-0.140388	0.71687879	1
-0.3616156	0.13672237	0.66368058	nonDEG	-0.0374188	0.92114867	1
-0.4071629	0.18512423	0.75274479	nonDEG	-0.1149523	0.78924117	1
-0.0144151	0.96247513	1	nonDEG	-0.2932229	0.44802363	1
0.26068715	0.55147537	1	nonDEG	-0.517258	0.34248133	1
-0.2364099	0.39923167	0.95204649	nonDEG	-0.1897767	0.640775	1
0.78128801	0.05105772	0.40866995	nonDEG	-0.0952535	0.83873556	1
-0.079924	0.74424483	1	nonDEG	-0.0407733	0.91517053	1

-0.0591066	0.80984469	1	nonDEG	0.37025101	0.305571	1
0.38973729	0.12593579	0.64488149	nonDEG	0.25913173	0.47185236	1
-0.3101382	0.20805958	0.78375764	nonDEG	-0.068811	0.85104125	1
-0.0866018	0.73473096	1	nonDEG	0.08963931	0.82673354	1
5.13E-15	1	1	nonDEG	5.73622615	0.03403597	1
0.15468255	0.70464508	1	nonDEG	-0.2427829	0.62773257	1
-0.6601072	0.0695619	0.48286555	nonDEG	1.12519038	0.00904048	0.73723709
-0.2616078	0.31487504	0.89811491	nonDEG	-0.3851322	0.29841667	1
0.01371633	0.95556227	1	nonDEG	0.22260965	0.53681321	1
-0.112872	0.65909859	1	nonDEG	0.11586624	0.75713583	1
-0.1972193	0.44098019	0.97744796	nonDEG	-0.2790555	0.46406522	1
-0.268983	0.38850331	0.94533384	nonDEG	-0.0853237	0.83759038	1
-0.224706	0.63422378	1	nonDEG	0.11094926	0.84094588	1
0.27488895	0.270929	0.85403991	nonDEG	-0.0311898	0.93192541	1
0.13829474	0.66946405	1	nonDEG	-0.1278928	0.76160303	1
0.52629713	0.03568937	0.33217176	nonDEG	0.03506927	0.93010125	1
0.55933333	0.12139373	0.63399257	nonDEG	-0.7210075	0.11808518	1
-0.0989412	0.68682802	1	nonDEG	-0.2970893	0.41219197	1
0.16353208	0.86938268	1	nonDEG	-1.7021931	0.10147551	1
-2.4320718	0.11624877	0.62336023	nonDEG	-0.0161321	1	1
0.10612896	0.67107313	1	nonDEG	-0.0439203	0.91113441	1
0.12818562	0.71071568	1	nonDEG	0.49620442	0.23698703	1
0.20025685	0.41789966	0.96254612	nonDEG	-0.1222505	0.74395363	1
0.20441113	0.48111209	0.99426825	nonDEG	-0.0879704	0.82095794	1
-0.007673	0.98255076	1	nonDEG	0.291139	0.4602006	1
0.42322069	0.20822546	0.7838989	nonDEG	0.22539628	0.56462899	1
-0.0177448	0.9777588	1	nonDEG	-0.879095	0.19276439	1
-0.4867868	0.04832009	0.39590737	nonDEG	0.30205268	0.42022038	1
-0.9129946	0.56774995	1	nonDEG	-4.5917122	0.13553306	1
-1.2540443	0.04711511	0.39049038	nonDEG	0.89731444	0.25132502	1
0.53859595	0.15876954	0.70625816	nonDEG	-0.5891212	0.17414063	1
-0.1961205	0.45149054	0.98329655	nonDEG	0.35922732	0.39839632	1
0.10657537	0.6806496	1	nonDEG	0.10941194	0.76702689	1
-0.3968726	0.13044706	0.65446487	nonDEG	-0.4088711	0.26779261	1
-0.3373677	0.18685334	0.75310345	nonDEG	-0.0289085	0.93773512	1
-0.0635803	0.80205968	1	nonDEG	-0.0074773	0.98457753	1
-0.2101581	0.66195609	1	nonDEG	-0.3482758	0.56283301	1
-0.4156336	0.15312837	0.69289056	nonDEG	0.13643848	0.75726395	1
-0.0638074	0.84028006	1	nonDEG	0.2268234	0.57130062	1
0.09355481	0.83043518	1	nonDEG	0.2672922	0.66759157	1
0.01736999	0.94441249	1	nonDEG	-0.137596	0.70278925	1
0.14712762	0.62674948	1	nonDEG	-0.1663205	0.67241619	1
0.56707613	0.06941975	0.48258181	nonDEG	0.24516281	0.56816775	1

0.54526759	0.04732453	0.39178482	nonDEG		-0.2796042	0.5244414	1
0.52101449	0.37642621	0.93394411	nonDEG		0.87547171	0.17151425	1
0.02706384	0.93462338	1	nonDEG		0.00644609	0.98973873	1
0.03363239	0.90930064	1	nonDEG		-0.0644319	0.87124101	1
-1.4555922	0.34884385	0.91917773	nonDEG		-0.3460407	1	1
-0.1431524	0.63162059	1	nonDEG		-0.1535342	0.72684738	1
0.47275056	0.13580291	0.66261751	nonDEG		-0.0145273	0.97145074	1
-0.3400173	0.497022	1	nonDEG		-0.2212679	0.71165212	1
-0.2114649	0.39985016	0.95204649	nonDEG		0.20436619	0.57644704	1
-0.6509236	0.03220693	0.31197349	nonDEG		0.00044697	0.99956726	1
0.01343271	0.9575069	1	nonDEG		-0.0621541	0.86536265	1
-0.2605373	0.39123336	0.94686517	nonDEG		-0.004346	0.99363811	1
-0.541695	0.03620987	0.33500389	nonDEG		0.4446878	0.3870594	1
-0.2846182	0.24980623	0.83131849	nonDEG		0.07151393	0.84573197	1
0.01719181	0.94922638	1	nonDEG		0.10791085	0.770112	1
0.05231895	0.84737357	1	nonDEG		-0.0847315	0.82395129	1
0.1901312	0.42961895	0.97110382	nonDEG		0.24175691	0.55236316	1
0.10127086	0.75098113	1	nonDEG		0.05667483	0.8910232	1
-0.0331427	0.92128096	1	nonDEG		0.29299345	0.48946048	1
0.06451275	0.8031411	1	nonDEG		-0.0764346	0.83302819	1
0.06027488	0.88659211	1	nonDEG		-0.3960036	0.43215177	1
0.2777933	0.29243588	0.8754342	nonDEG		-0.0695395	0.85086051	1
0.69959267	0.03814296	0.34416209	nonDEG		-0.5060182	0.26331024	1
-0.229153	0.41565464	0.961017	nonDEG		0.17864175	0.63916998	1
0.06033774	0.81101004	1	nonDEG		0.20501491	0.60403612	1
-0.1499762	0.61710906	1	nonDEG		0.55123908	0.19741312	1
-0.1597232	0.51556388	1	nonDEG		-0.1425648	0.69702791	1
0.14177865	0.65435313	1	nonDEG		0.03922394	0.92381978	1
0.18765641	0.47996156	0.99343606	nonDEG		0.28167447	0.45008197	1
0.24302762	0.33567768	0.91869633	nonDEG		0.31886354	0.37974831	1
-0.0592125	0.80984045	1	nonDEG		0.18607455	0.65039386	1
0.43531226	0.0991861	0.57396016	nonDEG		0.23963905	0.51865003	1
-0.0100222	1	1	nonDEG		0.17801521	0.79574262	1
0.08712252	0.82824836	1	nonDEG		0.16819735	0.72405162	1
-0.2595977	0.32799912	0.91205876	nonDEG		-0.2393879	0.54401108	1
0.16096074	0.76344985	1	nonDEG		-0.4256485	0.49247567	1
-0.3807176	0.13757601	0.66493192	nonDEG		0.26568106	0.50430426	1
-0.5471265	0.59964792	1	nonDEG		0.71999358	0.50022307	1
0.23934781	0.50018986	1	nonDEG		0.40206224	0.36618226	1
-0.1553108	0.63447993	1	nonDEG		0.44108882	0.28572095	1
0.76080671	0.04989094	0.40372166	nonDEG		0.10363771	0.79506905	1
0.89760273	0.08308144	0.52757433	nonDEG		-0.5934974	0.32491312	1
0.38278801	0.75247284	1	nonDEG		-2.1557149	0.09718803	1

0.40132602	0.24235045	0.82439569	nonDEG	-0.0627848	0.8842735	1
0.18234617	0.54706888	1	nonDEG	0.10856681	0.78082935	1
0.19125622	0.64276472	1	nonDEG	0.18518958	0.70371843	1
0.06450674	0.90343572	1	nonDEG	0.1742252	0.77232072	1
-0.1335956	0.57908621	1	nonDEG	0.21441824	0.54867662	1
0.48915728	0.55465491	1	nonDEG	0.15064838	0.88461583	1
0.03132321	0.97501557	1	nonDEG	-0.3036671	0.73998836	1
-0.2138834	0.45318418	0.98329655	nonDEG	-0.003802	0.99414895	1
-0.7314263	0.52683273	1	nonDEG	1.60861714	0.20199068	1
-0.4884772	0.06355371	0.46171022	nonDEG	-0.048348	0.89763841	1
1.01084916	0.3888697	0.94576196	nonDEG	-0.6955606	0.58470333	1
-0.5910218	0.17208569	0.73081805	nonDEG	-0.1166986	0.83611667	1
0.1619475	0.52569444	1	nonDEG	0.1459118	0.69130694	1
0.18954326	0.70670206	1	nonDEG	-0.9716117	0.12507234	1
-0.1334621	0.69504974	1	nonDEG	0.28506432	0.52921557	1
0.99978323	0.06026902	0.44787964	nonDEG	0.40582171	0.47588081	1
-0.2179127	0.60533518	1	nonDEG	0.13837291	0.80136106	1
-0.1396828	0.62241649	1	nonDEG	-0.4832452	0.23732787	1
0.56444625	0.77789352	1	nonDEG	0.43390567	0.91988578	1
-0.3843043	0.58460268	1	nonDEG	-0.518927	0.51159471	1
-0.381159	0.21632807	0.79477343	nonDEG	-0.1181683	0.77735113	1
-0.3667985	0.22918812	0.80924485	nonDEG	-0.1745025	0.66929841	1
0.11440905	0.65372429	1	nonDEG	0.16059664	0.68545914	1
0.5537952	0.1194386	0.63063362	nonDEG	-0.2247523	0.607954	1
0.23950443	0.74457506	1	nonDEG	0.44847773	0.56227758	1
0.04052474	0.88062658	1	nonDEG	0.26880321	0.48044433	1
0.24365017	0.6616931	1	nonDEG	-0.6764304	0.31075316	1
0.44584026	0.09754812	0.57010527	nonDEG	-0.1043013	0.78099001	1
-0.1490305	0.60222328	1	nonDEG	-0.1274851	0.73284385	1
-0.4549063	0.5600888	1	nonDEG	-1.7365337	0.07903899	1
-0.4879934	0.43264435	0.97234509	nonDEG	0.32814838	0.65214777	1
-0.1579799	0.55069117	1	nonDEG	0.68366071	0.08617641	1
0.03187949	0.90717744	1	nonDEG	-0.0456638	0.90150179	1
0.14429701	0.60095826	1	nonDEG	0.15067172	0.68146245	1
-0.0825274	0.82186202	1	nonDEG	0.05101195	0.91123554	1
-0.0528684	0.83059313	1	nonDEG	-0.4429391	0.23478598	1
0.37804353	0.43582944	0.97394991	nonDEG	-0.6808737	0.2199758	1
0.0261926	0.96686538	1	nonDEG	0.26361848	0.69980172	1
0.50120786	0.30874871	0.89262089	nonDEG	-1.0390052	0.07792041	1
-0.3551744	0.33912368	0.91869633	nonDEG	0.09908714	0.83903786	1
1.32132211	0.01152305	0.16150043	nonDEG	-0.5960616	0.30882595	1
0.01807397	0.95284227	1	nonDEG	-0.4739149	0.23431062	1
0.34677346	0.24040014	0.82215843	nonDEG	-0.6500566	0.11001735	1

-0.2181855	0.39982666	0.95204649	nonDEG	0.18595469	0.62773219	1
-0.2556845	0.64227135	1	nonDEG	-0.9042371	0.16494892	1
0.32461058	0.18060929	0.74592623	nonDEG	0.2145498	0.55808966	1
2.50348011	0.21316349	0.79100638	nonDEG	-1.2081703	0.50276904	1
0.36178039	0.18585134	0.75310345	nonDEG	0.23918172	0.51522048	1
-0.1579479	0.55412046	1	nonDEG	0.27991142	0.44980146	1
-0.110457	0.82209429	1	nonDEG	-0.0130779	0.99047589	1
0.37249869	0.14586202	0.68031971	nonDEG	-0.1600753	0.66021669	1
0.14163306	0.56870634	1	nonDEG	0.11301223	0.76321038	1
-0.0012323	0.99696655	1	nonDEG	0.17955964	0.6238698	1
-0.2233375	0.47835582	0.99305902	nonDEG	-0.3245141	0.47607533	1
0.39749646	0.17859057	0.74433965	nonDEG	-0.1920972	0.62161184	1
0.05423757	0.83097131	1	nonDEG	-0.0409753	0.91099728	1
-0.1853485	0.52922401	1	nonDEG	-0.2417579	0.5883214	1
0.01720631	0.9623024	1	nonDEG	-0.1464644	0.73128756	1
0.06788141	0.88081499	1	nonDEG	0.29331101	0.57936874	1
0.13977302	0.74697751	1	nonDEG	-0.9284044	0.06421889	1
-0.4388855	0.57951105	1	nonDEG	0.25456208	0.80838872	1
-0.0251123	0.98355641	1	nonDEG	0.86362402	0.20700232	1
-0.4963174	0.57182671	1	nonDEG	-1.0030289	0.38626393	1
0.26343729	0.3173884	0.90027476	nonDEG	0.17494318	0.64391036	1
3.62183136	0.12739166	0.64810083	nonDEG	-0.4469605	0.84513176	1
0.01179432	0.96682267	1	nonDEG	-0.6008503	0.12212283	1
-0.3527358	0.14348811	0.67589095	nonDEG	0.18408222	0.65739278	1
0.02070199	1	1	nonDEG	-0.8073473	0.57169616	1
-0.6976379	0.00453518	0.08292628	nonDEG	-0.4015856	0.29590093	1
-0.2519152	0.58099453	1	nonDEG	-0.6776142	0.22243044	1
-0.235385	0.37940822	0.93602044	nonDEG	-0.0633319	0.86810196	1
-0.3684574	0.41886941	0.96282267	nonDEG	0.47729114	0.32075664	1
-0.1726614	0.75634708	1	nonDEG	-1.2079049	0.06339939	1
0.25612713	0.36240068	0.92354131	nonDEG	-0.0814055	0.82684924	1
0.09366192	0.7016052	1	nonDEG	-0.1803173	0.63240814	1
0.03825891	0.90797235	1	nonDEG	0.08727731	0.83373613	1
0.40848422	0.6682159	1	nonDEG	-1.9322297	0.0808095	1
0.47077394	0.06773289	0.47541505	nonDEG	0.16778193	0.65195875	1
-0.0115428	1	1	nonDEG	-0.2488773	0.73006475	1
-0.0397247	0.96446339	1	nonDEG	-0.7551215	0.35905329	1
-0.5852942	0.54903678	1	nonDEG	-1.3980514	0.255444	1
4.96497364	0.09016093	0.54766713	nonDEG	-0.7517129	0.74406428	1
-0.0843538	0.72542962	1	nonDEG	-0.0226512	0.95210503	1
0.27731693	0.28327723	0.86547663	nonDEG	0.30023802	0.40980275	1
1.8599056	0.38196866	0.93796465	nonDEG	-5.619924	0.0925872	1
0.33035762	0.47244938	0.99012003	nonDEG	-0.4190049	0.46946903	1

-0.2177147	0.50697006	1	nonDEG	-0.1506484	0.72620704	1
-0.1797721	0.48150104	0.9944442	nonDEG	0.16892696	0.69849657	1
-0.1665108	0.55107282	1	nonDEG	-0.5809414	0.12262784	1
-0.0989614	0.69275205	1	nonDEG	-0.061698	0.86732719	1
-2.5460703	0.02614805	0.27364824	nonDEG	1.1699276	0.47546579	1
-0.2952979	0.6296214	1	nonDEG	0.02112797	0.985491	1
-0.1923432	0.42981807	0.97110382	nonDEG	0.26731169	0.46825617	1
0.00827796	0.98820712	1	nonDEG	0.09468451	0.84314929	1
0.26568282	0.28004553	0.86186717	nonDEG	0.16304261	0.71321947	1
0.17150599	0.48856034	0.99826154	nonDEG	0.22886924	0.52718946	1
-0.6982189	0.06868869	0.47917687	nonDEG	0.19324975	0.68786931	1
0.32104013	0.35394578	0.91917773	nonDEG	-0.4175068	0.34178418	1
0.31723456	0.36197824	0.92354131	nonDEG	-0.7307042	0.0951273	1
0.97705771	0.08890101	0.54533586	nonDEG	0.20641115	0.79989028	1
-0.3907101	0.41197592	0.95908664	nonDEG	-2.1257826	0.00098387	0.21777569
-0.1075889	0.71018789	1	nonDEG	-0.049473	0.89900704	1
0.30180913	0.23641632	0.81851818	nonDEG	0.10678802	0.80431158	1
0.61458014	0.07754698	0.51025426	nonDEG	0.08472476	0.84035022	1
0.25981911	0.39303971	0.94736887	nonDEG	0.1042621	0.84003812	1
0.04170822	0.87248618	1	nonDEG	-0.2221426	0.54642165	1
0.07043422	0.77175123	1	nonDEG	0.17622128	0.64680067	1
-0.2333704	0.4568702	0.98426953	nonDEG	-0.2477842	0.54917424	1
0.01864443	0.97455478	1	nonDEG	-0.1539445	0.78717891	1
-0.6927101	0.09771905	0.57010527	nonDEG	0.31374895	0.55997329	1
-0.4328467	0.25227083	0.83371668	nonDEG	0.27035561	0.56017731	1
-0.4897334	0.16836077	0.7242982	nonDEG	-0.2081203	0.66573435	1
0.35977643	0.14667683	0.68223696	nonDEG	0.21371409	0.63071862	1
0.70083775	0.20364481	0.77666791	nonDEG	-0.0907267	0.88566026	1
-0.1339821	0.71541033	1	nonDEG	-0.0621182	0.89463139	1
-0.0628143	0.87133999	1	nonDEG	0.05163254	0.92363266	1
0.31547249	0.19871708	0.77105997	nonDEG	0.09772005	0.79249817	1
0.82674808	0.00448531	0.08226984	nonDEG	-0.3048444	0.42694794	1
0.44996928	0.24535384	0.82672898	nonDEG	-0.1805944	0.6993463	1
0.85364391	0.04371162	0.37304382	nonDEG	-1.0198782	0.05038021	1
0.39838407	0.60265219	1	nonDEG	-2.0726328	0.01773101	0.95781535
-0.43206	0.79348784	1	nonDEG	0.7188253	0.69229906	1
-0.0797496	0.83471828	1	nonDEG	0.18752629	0.68850212	1
-0.3391079	0.25005396	0.83188159	nonDEG	0.43623192	0.35547736	1
-0.5861777	0.09632598	0.56635521	nonDEG	-0.4618889	0.31717509	1
-0.0187852	0.94358714	1	nonDEG	0.2782566	0.44465158	1
-0.3763467	0.20528795	0.77904149	nonDEG	0.15908165	0.68311989	1
-0.1020947	0.70044628	1	nonDEG	0.01951432	0.96014626	1
-1.4199303	0.01447644	0.18585783	nonDEG	0.11797029	0.89554314	1

-0.5925764	0.0244835	0.26171745	nonDEG	0.20317538	0.58806076	1
-0.4513615	0.20159696	0.77336631	nonDEG	0.39894559	0.36847369	1
-0.2517789	0.3645629	0.9254434	nonDEG	0.09941012	0.79325775	1
0.27818866	0.30671572	0.8907373	nonDEG	0.26770935	0.48236657	1
0.00153834	1	1	nonDEG	0.04854689	0.93518744	1
-0.0711235	0.78364922	1	nonDEG	-0.036346	0.92075597	1
-0.6801906	0.00883235	0.1336841	nonDEG	0.09482614	0.79988522	1
-0.1851593	0.49746984	1	nonDEG	-0.2033117	0.58772328	1
0.18033167	0.46558191	0.98613495	nonDEG	-0.0564626	0.87565261	1
0.09115819	0.72926556	1	nonDEG	0.42031616	0.27696641	1
0.5866317	0.07684001	0.50776501	nonDEG	0.41257	0.3027834	1
0.42785653	0.08713989	0.53984	nonDEG	-0.0482246	0.89483408	1
-2.0065215	0.01563592	0.19496226	nonDEG	1.66275534	0.05899107	1
-0.0901922	0.78858789	1	nonDEG	-0.5402451	0.20880822	1
-0.4043528	0.12002542	0.63091411	nonDEG	-0.177164	0.62526836	1
0.11980843	0.7161375	1	nonDEG	0.49687593	0.25795139	1
0.08836436	0.79276247	1	nonDEG	-1.0810929	0.01260424	0.82534259
0.11775927	0.86568665	1	nonDEG	0.32216851	0.63666882	1
-0.4174032	0.09408222	0.55941963	nonDEG	-0.3314374	0.38317316	1
0.06418858	0.80146183	1	nonDEG	0.1496903	0.68019153	1
-0.2060645	0.39287511	0.94732169	nonDEG	-0.0046407	0.99025303	1
-0.2764076	0.30449234	0.88945724	nonDEG	0.48730733	0.21306393	1
0.10991626	0.70794746	1	nonDEG	-0.1012322	0.80877619	1
0.13769793	0.63948105	1	nonDEG	0.2812595	0.46387892	1
0.37775794	0.19257614	0.76192362	nonDEG	0.21100119	0.57657584	1
-0.3274881	0.39624429	0.9493779	nonDEG	0.14519937	0.76830456	1
0.50485853	0.11157066	0.61039373	nonDEG	0.2024109	0.6058847	1
2.02039276	0.06488843	0.4657179	nonDEG	-1.7276953	0.07059169	1
1.15847561	0.45935345	0.98479636	nonDEG	-6.1788609	0.01326642	0.84289973
0.02458523	0.96785084	1	nonDEG	-0.1369129	0.81866156	1
0.02044562	0.93724147	1	nonDEG	-0.318962	0.38197366	1
0.12371162	0.69717539	1	nonDEG	-0.1644857	0.68706761	1
0.49329776	0.31757124	0.90044709	nonDEG	-0.0019158	1	1
-0.1386147	0.58032434	1	nonDEG	0.32170684	0.44690458	1
0.14488805	0.56652083	1	nonDEG	0.64778219	0.07528242	1
0.36191315	0.32851412	0.91257619	nonDEG	-0.0089854	0.98846997	1
-0.3745317	0.54377609	1	nonDEG	-0.1001512	0.89833923	1
0.1071231	0.6906242	1	nonDEG	0.12352568	0.74202921	1
0.11878101	0.74843724	1	nonDEG	0.16888764	0.70226154	1
-0.032987	0.8926289	1	nonDEG	-0.0740831	0.83858677	1
-1.3660407	0.06587504	0.46899524	nonDEG	0.11195458	0.92535922	1
0.11812949	0.83737047	1	nonDEG	0.08892714	0.88850457	1
0.11238263	0.6956833	1	nonDEG	0.05345336	0.88807783	1

0.21827262	0.3774981	0.93497951	nonDEG	0.27266511	0.45550654	1
1.2903025	0.02401482	0.25884688	nonDEG	0.31800674	0.67321139	1
-1.2286147	0.03345032	0.3192039	nonDEG	-1.3776696	0.07471256	1
-0.0245979	0.95192113	1	nonDEG	-0.0363663	0.94401372	1
-0.3279689	0.19993269	0.7730545	nonDEG	-0.4027073	0.33427943	1
0.80542465	0.01013006	0.14719589	nonDEG	-0.4316482	0.2451115	1
0.01584746	0.95267771	1	nonDEG	0.11983929	0.74289035	1
0.62243595	0.0106192	0.15285633	nonDEG	-0.0357311	0.92763813	1
-0.0637042	0.80241035	1	nonDEG	0.09629784	0.79995153	1
0.04258078	0.86605501	1	nonDEG	0.19106777	0.5990473	1
-0.0132134	0.95879248	1	nonDEG	0.1287905	0.74783107	1
-0.6345584	0.07542462	0.50315553	nonDEG	-0.0429993	0.93371307	1
1.25613676	0.0033891	0.06796431	nonDEG	0.17314362	0.72024179	1
-0.218117	0.39024888	0.94686517	nonDEG	-0.2762539	0.45706764	1
0.59321814	0.01560695	0.1948486	nonDEG	-0.1240708	0.73191987	1
0.62804446	0.05954545	0.4449133	nonDEG	-0.0378384	0.93922042	1
0.34735342	0.68500365	1	nonDEG	-0.187009	0.85306971	1
-0.3007336	0.42277586	0.96503828	nonDEG	-0.0479093	0.92386853	1
-0.3449687	0.31111437	0.89459817	nonDEG	-0.1044697	0.80924471	1
-0.3940136	0.78582191	1	nonDEG	0.44828642	0.78608613	1
-1.8940698	0.00973083	0.14316223	nonDEG	0.59717049	0.52915595	1
-0.374488	0.13032919	0.65446487	nonDEG	0.35047486	0.3398004	1
-0.1253355	0.61989202	1	nonDEG	-0.360894	0.34747763	1
-0.1327628	0.6798728	1	nonDEG	-0.2495753	0.56988566	1
0.26097191	0.41871578	0.96263754	nonDEG	0.38949358	0.3264925	1
0.09354962	0.72311013	1	nonDEG	0.28914567	0.48031198	1
0.36563723	0.13708999	0.66374713	nonDEG	-0.2572855	0.52112534	1
0.40009671	0.24801128	0.82968229	nonDEG	-1.141109	0.00848768	0.71595639
0.00713187	0.989462	1	nonDEG	-0.9880658	0.0599583	1
-0.6082496	0.05089512	0.40803418	nonDEG	0.11455729	0.81530836	1
-0.479014	0.6942251	1	nonDEG	0.41684368	0.71737031	1
-0.3975457	0.23428937	0.81498817	nonDEG	0.32929604	0.42459524	1
0.17804593	0.61227655	1	nonDEG	-0.0684261	0.88249329	1
-0.3301327	0.20756344	0.78320256	nonDEG	0.02855494	0.94107614	1
-0.2695391	0.86660583	1	nonDEG	1.22184092	0.30366538	1
-0.1008311	0.81608356	1	nonDEG	0.42240406	0.41611918	1
0.32293872	0.53469776	1	nonDEG	-0.4438151	0.47324836	1
0.10588255	0.66896804	1	nonDEG	-0.0148951	0.96764888	1
0.13138163	0.66766567	1	nonDEG	-0.3297221	0.42961766	1
-0.0415492	0.92735049	1	nonDEG	0.09645677	0.86484805	1
-1.3473887	0.35521183	0.91917773	nonDEG	1.6794115	0.27469837	1
0.68202451	0.02264386	0.2489707	nonDEG	-0.0912646	0.82764345	1
0.49485402	0.1964792	0.76773973	nonDEG	-0.3377262	0.45349759	1

-0.1369373	0.7863735	1	nonDEG	0.56779027	0.31484104	1
0.6454832	0.01180165	0.16414912	nonDEG	-0.1213072	0.74278124	1
-0.1330721	0.63655543	1	nonDEG	0.21089718	0.59689622	1
0.12473139	0.71612738	1	nonDEG	-0.5458633	0.27400953	1
-0.2293093	0.61679815	1	nonDEG	-1.1123973	0.04181884	1
-0.2029361	0.41109212	0.95836418	nonDEG	0.12092111	0.7374634	1
-0.4517107	0.90214436	1	nonDEG	-0.0481616	1	1
1.15717645	0.10741596	0.59991077	nonDEG	0.72923628	0.30225407	1
-0.0331315	0.90708489	1	nonDEG	-0.407056	0.37138046	1
0.88303884	0.61664739	1	nonDEG	0.12961243	1	1
-0.2347841	0.51229118	1	nonDEG	-0.2889948	0.53810801	1
0.02304441	0.9304209	1	nonDEG	0.20409174	0.58200086	1
-0.0366552	0.91217281	1	nonDEG	7.36E-05	1	1
0.07144106	0.84635052	1	nonDEG	-0.0577287	0.89786229	1
0.49212845	0.31799502	0.90091957	nonDEG	0.69171234	0.16020445	1
0.53106694	0.12698338	0.64755605	nonDEG	-1.2829758	0.00300562	0.4301506
-0.1770335	0.79941051	1	nonDEG	0.11753585	0.89210602	1
0.01604832	0.95709412	1	nonDEG	-0.0206874	0.95813883	1
0.22946387	0.56666084	1	nonDEG	-0.0379183	0.94530428	1
0.23559815	0.45236946	0.98329655	nonDEG	-0.0633762	0.88237134	1
-0.1151083	0.67200626	1	nonDEG	-0.1078278	0.77298811	1
0.16285549	0.90218777	1	nonDEG	-0.0308049	1	1
0.52669378	0.32656654	0.91057556	nonDEG	0.85836789	0.09946948	1
0.14019671	0.57349549	1	nonDEG	0.15719273	0.70717163	1
-0.2530321	0.49677047	1	nonDEG	0.23011933	0.61641216	1
-0.1917152	0.55626485	1	nonDEG	0.5649106	0.19458202	1
-0.1102327	0.65177686	1	nonDEG	-0.1657282	0.64545573	1
-0.1396017	0.57829354	1	nonDEG	-0.2628736	0.52040175	1
0.23838212	0.37359727	0.93232638	nonDEG	-0.1649884	0.65250113	1
0.27206501	0.58000417	1	nonDEG	-0.3686998	0.52243302	1
0.89159414	0.00427948	0.07988807	nonDEG	-0.1096856	0.77673427	1
5.13E-15	1	1	nonDEG	4.04375954	0.34899467	1
-0.1102858	0.68014536	1	nonDEG	-0.0965155	0.7902081	1
-1.7605515	0.27350187	0.85674092	nonDEG	0.50395994	0.81866699	1
0.56376508	0.30798188	0.89206467	nonDEG	-0.0216955	0.97591277	1
0.35982231	0.57011087	1	nonDEG	0.21985085	0.76526682	1
-0.580747	0.14663869	0.68223696	nonDEG	-0.1732608	0.74332788	1
0.18691441	0.4772811	0.99294124	nonDEG	0.18548934	0.62401171	1
0.82908752	0.55962232	1	nonDEG	-1.3208395	0.34017214	1
0.1043975	0.68275123	1	nonDEG	-0.0542829	0.88230181	1
-0.360039	0.34609937	0.91869633	nonDEG	-0.325754	0.51090255	1
0.23996979	0.39956672	0.95204649	nonDEG	-0.3233735	0.40591794	1
0.01554982	0.95302005	1	nonDEG	-0.1893701	0.60442549	1

0.31066555	0.65633831	1	nonDEG	-0.1367961	0.87592168	1
0.32734862	0.36353138	0.92416624	nonDEG	0.09622467	0.8285522	1
0.00640577	0.98771652	1	nonDEG	-0.1950149	0.65246289	1
0.36604357	0.18510962	0.75274479	nonDEG	-0.1155971	0.78685019	1
-0.1472634	0.68774389	1	nonDEG	-0.5874065	0.22939389	1
-0.1484358	0.61685542	1	nonDEG	-0.0374292	0.94243477	1
0.47887087	0.05045437	0.40607126	nonDEG	0.11134304	0.75895349	1
-0.0278207	0.91474982	1	nonDEG	0.00977296	0.97964312	1
-0.8571507	0.30763762	0.89189099	nonDEG	0.36217743	0.74399902	1
-0.177386	0.74345992	1	nonDEG	-0.317028	0.61950972	1
0.69790622	0.00696245	0.11249589	nonDEG	-0.1624839	0.66240924	1
-0.749164	0.08517523	0.53491714	nonDEG	-0.5739636	0.31083161	1
4.79701752	0.18764914	0.75310345	nonDEG	0.34219336	0.92128248	1
-0.2261853	0.39031303	0.94686517	nonDEG	0.07760228	0.85221163	1
-0.5494354	0.17642747	0.74114157	nonDEG	-0.0872523	0.86904758	1
-0.4320884	0.12098165	0.63294336	nonDEG	0.01397658	0.97294935	1
-0.5098677	0.12900494	0.65221148	nonDEG	0.39352065	0.36470037	1
0.47044652	0.11005286	0.60569191	nonDEG	0.19691274	0.60013317	1
0.02837066	0.96160968	1	nonDEG	-0.3139968	0.60020483	1
-0.1804584	0.5893077	1	nonDEG	0.01006885	0.98377416	1
-0.3050678	0.38718949	0.94412832	nonDEG	0.32171403	0.50420581	1
0.08942931	0.88147522	1	nonDEG	0.34043053	0.59722614	1
0.03704524	0.92731971	1	nonDEG	-0.7170679	0.18117621	1
-0.10792	0.67999569	1	nonDEG	-0.1968911	0.63029142	1
-0.2458658	0.43696746	0.97402891	nonDEG	0.03954239	0.92621105	1
0.46931488	0.06543187	0.46742269	nonDEG	0.03706687	0.92578846	1
0.18987144	0.45874832	0.98479636	nonDEG	0.0442284	0.90282137	1
0.02914357	0.91391403	1	nonDEG	0.14141843	0.73363676	1
0.08100437	0.79009368	1	nonDEG	-0.0520301	0.89872423	1
0.15617232	0.51695372	1	nonDEG	0.02685944	0.94105145	1
0.88346955	0.36985126	0.92861845	nonDEG	-1.460133	0.21147594	1
0.09983675	0.67886087	1	nonDEG	0.37864124	0.29442057	1
0.53206446	0.06490636	0.4657179	nonDEG	-0.0217416	0.9589233	1
-0.3735345	0.59819329	1	nonDEG	-0.9850123	0.21388551	1
-0.0082795	0.98138162	1	nonDEG	-0.3016331	0.4653227	1
-0.4430348	0.46321669	0.98479636	nonDEG	0.41676887	0.54671786	1
-0.025799	0.94336419	1	nonDEG	-0.0081585	0.98731906	1
-0.2536907	0.89654859	1	nonDEG	-0.4136569	0.82420339	1
0.59280861	0.15872042	0.70615789	nonDEG	0.11590307	0.80990246	1
-0.3911027	0.13956149	0.66785296	nonDEG	-0.2299474	0.53141921	1
-0.2647408	0.36654249	0.92627904	nonDEG	-0.0815234	0.84362886	1
0.19788889	0.43040601	0.9716987	nonDEG	0.51917532	0.1593184	1
-0.0892948	0.7353112	1	nonDEG	-0.1964544	0.6001001	1

0.21008947	0.48090351	0.99426825	nonDEG	0.47532819	0.21611035	1
0.60514963	0.32545553	0.90960093	nonDEG	-0.3744263	0.5819143	1
0.25429927	0.31493126	0.89811491	nonDEG	0.13340914	0.74086223	1
0.6191325	0.39444636	0.94862012	nonDEG	-0.7523342	0.34009326	1
0.26567752	0.28043065	0.86186717	nonDEG	-0.0236457	0.9545981	1
-0.1821297	0.6510543	1	nonDEG	-0.2924422	0.58458037	1
-2.2798446	0.00502209	0.08868937	nonDEG	0.49628457	0.67167588	1
0.12273396	0.64239052	1	nonDEG	0.31200409	0.43293785	1
3.24170627	0.00337481	0.06777013	nonDEG	-2.84681	0.00946976	0.74892342
-0.190825	0.4627076	0.98479636	nonDEG	0.19379224	0.59695325	1
0.13649366	0.57845056	1	nonDEG	0.06524216	0.85697735	1
0.9449767	0.0093874	0.13942164	nonDEG	-0.6582048	0.07973551	1
0.16641527	0.52807774	1	nonDEG	-0.0734961	0.84079957	1
-0.8674549	0.0081029	0.12584561	nonDEG	-0.1756058	0.68428071	1
0.73315892	0.82948544	1	nonDEG	1.20381281	0.43473544	1
0.1357456	0.67316283	1	nonDEG	-0.2734228	0.53593696	1
0.17356948	0.47849688	0.99305902	nonDEG	0.12465355	0.72960228	1
-0.1477052	0.94983504	1	nonDEG	-0.0184942	1	1
-0.3931374	0.10771608	0.60084562	nonDEG	0.08346881	0.82613409	1
2.11269569	0.16558916	0.71835826	nonDEG	2.92352318	0.02783252	1
-0.2775216	0.35089806	0.91917773	nonDEG	-0.0135597	0.97554904	1
0.13226719	0.73255871	1	nonDEG	0.26937131	0.55059724	1
-0.1922791	0.67637026	1	nonDEG	-0.0826293	0.88227035	1
-0.0698194	0.78831508	1	nonDEG	0.09322999	0.79850418	1
-0.1471344	0.59992896	1	nonDEG	0.18870019	0.61709405	1
0.21508762	0.70288552	1	nonDEG	-1.4726137	0.03451197	1
-0.577881	0.51565518	1	nonDEG	-1.4002832	0.3270422	1
-0.4054484	0.27901015	0.86056653	nonDEG	-0.1871743	0.67777081	1
0.29111479	0.81080069	1	nonDEG	-0.107541	0.92501197	1
0.96071575	0.83547469	1	nonDEG	-1.1984205	0.63878528	1
0.31387029	0.24867123	0.82990493	nonDEG	0.19451239	0.60321751	1
2.21273334	0.09825151	0.57137375	nonDEG	-0.1609321	0.92248203	1
0.05074343	0.84136126	1	nonDEG	0.27883592	0.4464151	1
-0.3023703	0.24391782	0.82612635	nonDEG	-0.055203	0.89179583	1
0.62804458	0.09913077	0.57395365	nonDEG	-0.0935481	0.8285205	1
-0.372144	0.22939014	0.80924485	nonDEG	-0.3318275	0.43527802	1
0.28141801	0.26017364	0.84251257	nonDEG	-0.0524682	0.88560573	1
-0.854905	0.32465205	0.90838968	nonDEG	-0.1408516	0.90547084	1
0.0095876	0.97865327	1	nonDEG	0.0605098	0.88427583	1
-0.339227	0.27676576	0.85887436	nonDEG	-0.1473862	0.73461376	1
0.29909862	0.26704696	0.85072996	nonDEG	0.37687911	0.30199676	1
-0.1148662	0.8946814	1	nonDEG	-0.5061573	0.60113214	1
-0.1067437	0.68851479	1	nonDEG	-0.1067451	0.77498783	1

-0.0860879	0.73971094	1	nonDEG	0.16448498	0.66159987	1
0.13518127	0.78507647	1	nonDEG	0.29782182	0.60250068	1
-0.0992424	0.70125532	1	nonDEG	0.10084178	0.80174805	1
-0.3522263	1	1	nonDEG	0.91991847	0.81254758	1
2.12430637	0.14377654	0.67635628	nonDEG	-1.3522359	0.44049566	1
-0.2341288	0.48305224	0.99583236	nonDEG	0.06070874	0.88690977	1
-0.2807597	0.28779148	0.87007405	nonDEG	0.05236256	0.88752799	1
0.46696034	0.06598891	0.4693976	nonDEG	0.07227756	0.84269346	1
0.7070432	0.00508999	0.0894593	nonDEG	0.04734246	0.91056277	1
0.11353374	0.72005869	1	nonDEG	0.25238693	0.52659827	1
-0.1451946	0.61736381	1	nonDEG	0.11864855	0.76201538	1
0.15471281	0.58741383	1	nonDEG	-0.4288215	0.26870247	1
0.32676967	0.23803597	0.81997906	nonDEG	0.06590202	0.85717641	1
0.16541043	0.50508803	1	nonDEG	0.0580682	0.87343306	1
-0.5683036	0.02340185	0.25478073	nonDEG	0.24881192	0.50909701	1
-0.3124389	0.22340471	0.80391288	nonDEG	0.21495094	0.55299255	1
0.26221358	0.41927342	0.96282267	nonDEG	0.29977072	0.46628678	1
0.28268678	0.44475846	0.97966661	nonDEG	0.13807304	0.77241514	1
-0.0156883	0.9696619	1	nonDEG	-0.3686407	0.44946632	1
-0.2480801	0.51111473	1	nonDEG	0.67498665	0.15318292	1
0.50644976	0.12006378	0.63091411	nonDEG	-0.0502265	0.90531471	1
0.31734139	0.21500952	0.7942651	nonDEG	0.17936894	0.63513254	1
-1.9739568	0.01289388	0.17249823	nonDEG	0.83990959	0.37860259	1
0.22137888	0.42044077	0.96335095	nonDEG	0.69194655	0.06944809	1
0.00691663	0.97799886	1	nonDEG	-0.0654554	0.85621811	1
-0.4490995	0.64601999	1	nonDEG	0.57078951	0.54987898	1
0.05298843	0.86119237	1	nonDEG	0.09785008	0.80219611	1
-0.8982813	0.006391	0.10558063	nonDEG	0.06696973	0.857966	1
0.27152208	0.7999246	1	nonDEG	-0.4673845	0.68394474	1
0.65595615	0.00962792	0.14200211	nonDEG	0.00439061	0.99095316	1
-0.0629629	0.8252228	1	nonDEG	-0.0232437	0.95268778	1
0.04430774	0.89634673	1	nonDEG	0.19336652	0.65571718	1
-0.0214058	0.94638452	1	nonDEG	-0.1783116	0.67850127	1
0.47847918	0.09310166	0.55653319	nonDEG	0.27479602	0.47024911	1
0.09543364	0.70975456	1	nonDEG	0.01886658	0.9611458	1
0.21249844	0.39470305	0.94862012	nonDEG	0.3633148	0.38426206	1
0.71061588	0.00446112	0.08197942	nonDEG	0.07050153	0.84648749	1
-0.2041917	0.4735843	0.99070103	nonDEG	0.17143101	0.65969253	1
0.33999635	0.44640861	0.98124647	nonDEG	-0.7198508	0.19157447	1
0.0049072	0.99058528	1	nonDEG	-0.0032809	0.99559428	1
-0.309543	0.41171305	0.95874233	nonDEG	-0.1600858	0.75397034	1
-0.3147345	0.38767798	0.94456147	nonDEG	-0.1160564	0.82117209	1
1.17787559	0.38864786	0.94541434	nonDEG	-0.7164621	0.60032212	1

-0.2292153	0.34840881	0.91917773	nonDEG	0.19267404	0.59579223	1
-0.2004747	0.77583255	1	nonDEG	-0.0170411	1	1
-0.175753	0.47317162	0.99066207	nonDEG	0.0547096	0.88140097	1
5.13E-15	1	1	nonDEG	5.89180296	0.0702488	1
-1.1721175	0.15351912	0.69375682	nonDEG	0.39857516	0.71991745	1
0.35165187	0.82896289	1	nonDEG	0.94201603	0.45008728	1
0.34117581	0.24144075	0.82326812	nonDEG	-0.4037713	0.29628874	1
-0.2777391	0.2763353	0.85842562	nonDEG	-0.0785812	0.830994	1
-0.4285862	0.156597	0.70108546	nonDEG	0.03005417	0.94203445	1
-0.5686563	0.12658758	0.64648377	nonDEG	0.1460869	0.75271357	1
0.30759375	0.41207388	0.95908664	nonDEG	-0.1300888	0.77754652	1
-1.2938733	0.31960624	0.90293668	nonDEG	-1.1405671	0.58738534	1
-0.3080629	0.23697461	0.81883661	nonDEG	-0.2817607	0.47075269	1
-0.0673959	0.78316372	1	nonDEG	-0.003412	0.99298175	1
-0.6181349	0.09274385	0.55536636	nonDEG	-0.6715928	0.19473866	1
1.08000137	0.00422809	0.07917988	nonDEG	-0.3048486	0.51117982	1
0.35237302	0.40298265	0.9535508	nonDEG	-0.148589	0.76745401	1
-0.1925078	0.45170684	0.98329655	nonDEG	0.08763829	0.81113364	1
-0.3259484	0.25651811	0.83849359	nonDEG	-0.0175332	0.96404988	1
-0.1543867	0.5748282	1	nonDEG	0.76802433	0.03668969	1
0.26989226	0.37922776	0.93595625	nonDEG	0.17979087	0.66019734	1
0.18791331	0.44143687	0.97752398	nonDEG	0.03059025	0.93195009	1
-0.6564175	0.70957953	1	nonDEG	-0.688603	0.80289229	1
0.06556866	0.83600876	1	nonDEG	-0.4199494	0.37614899	1
-0.057758	0.91702797	1	nonDEG	-0.3049166	0.61808614	1
-0.64687	0.02929247	0.29425173	nonDEG	0.07401656	0.85533216	1
-0.0400887	0.93552304	1	nonDEG	0.20575295	0.70358884	1
0.32562831	0.21593055	0.79477343	nonDEG	-0.341261	0.37595078	1
0.1096849	0.68276765	1	nonDEG	0.23398651	0.5379895	1
-0.0325373	0.8975814	1	nonDEG	0.00630159	0.98680076	1
0.15666167	0.55136715	1	nonDEG	-0.1356817	0.71980455	1
-0.0826673	0.86089304	1	nonDEG	-0.2653211	0.6358436	1
0.1398445	0.74714746	1	nonDEG	0.18176086	0.71773223	1
-0.4814586	0.16754751	0.7227101	nonDEG	-0.4256558	0.36473872	1
-0.236364	0.36222267	0.92354131	nonDEG	-0.0705575	0.85738813	1
-0.1515809	0.67767563	1	nonDEG	-0.141131	0.76813931	1
0.05524077	0.82510433	1	nonDEG	0.04342402	0.90538203	1
1.45548626	0.27462124	0.85818661	nonDEG	-1.0547602	0.36924963	1
-0.0657621	0.79792106	1	nonDEG	0.07879607	0.82896038	1
0.15460228	0.52689939	1	nonDEG	0.1173205	0.75074453	1
-0.2566509	0.82960078	1	nonDEG	-0.7173589	0.56030979	1
1.22204851	0.14438798	0.67739245	nonDEG	-0.2149716	0.82992514	1
0.40440131	0.11370843	0.61607445	nonDEG	0.22429083	0.53933455	1

-0.2126493	0.46266564	0.98479636	nonDEG	-0.0887226	0.8397352	1
-0.3846121	0.30259683	0.88711796	nonDEG	-0.3127476	0.52569173	1
0.23833259	0.58357577	1	nonDEG	-0.4280679	0.44929487	1
-0.3512177	0.21779146	0.7961707	nonDEG	-0.0162341	0.96744444	1
-0.1676271	0.49359225	1	nonDEG	-0.0658922	0.8554886	1
-0.1324111	0.60870141	1	nonDEG	0.19498111	0.60480621	1
-0.1494964	0.54643921	1	nonDEG	-0.1100278	0.79601472	1
0.04783094	0.9747517	1	nonDEG	-0.0959781	0.92585013	1
-0.0952772	0.69718566	1	nonDEG	0.04635149	0.8998387	1
-0.0040701	0.98723908	1	nonDEG	-0.095559	0.792159	1
-0.3316438	0.71317547	1	nonDEG	0.18583169	0.8880641	1
-0.0571169	0.97033131	1	nonDEG	-0.7088134	0.45730391	1
0.48584206	0.30606601	0.89026796	nonDEG	0.06052649	0.91897265	1
-1.0853641	0.25535555	0.83750042	nonDEG	-1.2648851	0.39722043	1
-0.5104519	0.44749206	0.98254542	nonDEG	-1.3615598	0.10053573	1
-0.0425873	0.87968896	1	nonDEG	-0.1613344	0.67140191	1
0.06102989	0.81311631	1	nonDEG	-0.0106902	0.97778551	1
0.17998433	0.46422815	0.98553614	nonDEG	-0.0186133	0.95929673	1
0.12322934	0.61682084	1	nonDEG	0.15479528	0.66868732	1
-0.1883886	0.51457996	1	nonDEG	-0.380862	0.29404049	1
1.16960778	0.39463873	0.94862012	nonDEG	-1.5615139	0.33249871	1
0.05629829	0.81928196	1	nonDEG	0.13335206	0.71316045	1
0.76553898	0.04542658	0.38154438	nonDEG	0.15658186	0.74676865	1
-0.9664057	0.11365241	0.61607445	nonDEG	-0.4343259	0.55677004	1
-1.8775355	0.13352957	0.65907076	nonDEG	1.64211233	0.24702032	1
-0.0753175	0.84893431	1	nonDEG	0.38851068	0.40942476	1
-0.0150544	1	1	nonDEG	0.69697878	0.48588368	1
0.01970062	0.94905794	1	nonDEG	0.21173698	0.59873785	1
-0.2048567	0.43356686	0.97299701	nonDEG	-0.218816	0.60264847	1
1.0046419	0.077793	0.51054069	nonDEG	0.2221008	0.730228	1
0.17613637	0.47917225	0.99332737	nonDEG	-0.0834737	0.81723234	1
-0.0934833	0.73232855	1	nonDEG	0.10075919	0.79495617	1
-0.7293345	0.03916772	0.34952124	nonDEG	0.56013153	0.2551357	1
-0.2028072	0.44221101	0.97851624	nonDEG	0.03443409	0.93014105	1
-0.7394144	0.54137364	1	nonDEG	0.21219235	0.91906213	1
-0.3147322	0.32805703	0.91213362	nonDEG	-0.2000721	0.63346645	1
0.2424424	0.33881946	0.91869633	nonDEG	0.01019306	0.97768983	1
0.02367549	0.93244333	1	nonDEG	-0.0946979	0.80023109	1
0.17796719	0.50761179	1	nonDEG	-0.2010019	0.60300325	1
0.05559044	0.83179668	1	nonDEG	0.31466118	0.42233634	1
0.31959782	0.35414882	0.91917773	nonDEG	-0.1398383	0.74929176	1
-0.2993596	0.24847408	0.82990493	nonDEG	0.00340538	0.99325586	1
0.32849709	0.38216299	0.93812152	nonDEG	-0.1951139	0.67849174	1

0.36150111	0.47160181	0.98975108	nonDEG	0.32212328	0.56159633	1
0.17294441	0.4914673	0.99967567	nonDEG	-0.2603651	0.50271731	1
-0.0120299	0.96388005	1	nonDEG	-0.023639	0.94941195	1
0.05218346	0.88853153	1	nonDEG	-0.1555253	0.7380561	1
-0.2706407	0.30197397	0.8866758	nonDEG	-0.2638916	0.48954975	1
0.06086612	0.83073382	1	nonDEG	0.09729574	0.81302053	1
-0.3823958	0.18305789	0.75019383	nonDEG	0.03321191	0.93666026	1
0.86794914	0.00230907	0.05080479	nonDEG	0.18196223	0.66929983	1
0.30470546	0.24296967	0.82500393	nonDEG	0.0526032	0.88655532	1
-0.3793599	0.12317546	0.63998628	nonDEG	-0.0318091	0.93218202	1
-1.2754937	0.04038013	0.35647711	nonDEG	0.46130361	0.56639664	1
0.0607585	0.88356591	1	nonDEG	-0.6701324	0.19117813	1
0.27024117	0.71444564	1	nonDEG	-0.9931423	0.21264435	1
-0.0769913	0.75800867	1	nonDEG	-0.0641936	0.86151901	1
-0.1628908	0.53244956	1	nonDEG	0.10301106	0.77823484	1
0.51818852	0.2207196	0.80037926	nonDEG	-0.2413387	0.63091442	1
0.04927652	0.85501938	1	nonDEG	-0.2639141	0.65330113	1
0.1611661	0.51145426	1	nonDEG	0.34865414	0.34410269	1
0.10937471	0.66988423	1	nonDEG	0.03389714	0.92897768	1
-0.5760108	0.02439256	0.26131467	nonDEG	0.438009	0.3232615	1
-0.4879134	0.07458008	0.50012823	nonDEG	0.08369635	0.8202134	1
-0.0072792	0.98254344	1	nonDEG	0.1178775	0.76232859	1
-0.3908859	0.66079644	1	nonDEG	1.01246267	0.26685212	1
0.02477907	1	1	nonDEG	0.6157356	0.47581978	1
-0.3825447	0.13443391	0.65981274	nonDEG	-0.0245619	0.94737452	1
0.40084477	0.14919725	0.6856099	nonDEG	-0.2759814	0.46264162	1
0.0878873	0.73681769	1	nonDEG	0.1127884	0.75875236	1
-0.1112763	0.65528405	1	nonDEG	-0.1143129	0.75186673	1
0.43792097	0.33532228	0.91869633	nonDEG	0.48964486	0.38805113	1
0.17817151	0.5164041	1	nonDEG	0.03878659	0.92094344	1
0.45067533	0.5047367	1	nonDEG	-0.4215954	0.59434659	1
-0.0278913	0.93496392	1	nonDEG	0.026395	0.95481389	1
0.15793423	0.83579443	1	nonDEG	-0.4147064	0.57364306	1
0.07698635	0.86033979	1	nonDEG	-1.1455305	0.03947653	1
-0.035616	0.92025219	1	nonDEG	-0.1993525	0.68077612	1
-0.0588776	0.82284846	1	nonDEG	0.0968999	0.7902329	1
0.23321008	0.40016263	0.95204649	nonDEG	-0.9607941	0.03142456	1
0.48074415	0.07883893	0.51427855	nonDEG	-0.1000239	0.78960063	1
0.31773424	0.21331765	0.79115104	nonDEG	-0.0449392	0.90198149	1
-0.0481738	0.87061161	1	nonDEG	0.44180898	0.23702025	1
-1.1190987	0.22483161	0.80603814	nonDEG	-0.3387251	0.80004606	1
0.37613012	0.16022481	0.70930196	nonDEG	0.01145782	0.97698228	1
0.37802113	0.34218015	0.91869633	nonDEG	0.32516743	0.48230175	1

-0.0460877	0.85816722	1	nonDEG	-0.3632381	0.32095406	1
0.29472898	0.33695362	0.91869633	nonDEG	0.40658566	0.29288147	1
-0.1135574	0.92818285	1	nonDEG	-2.0464877	0.11255102	1
0.38443045	0.18842918	0.75399845	nonDEG	-0.513442	0.1900069	1
0.51777396	0.30459438	0.88957713	nonDEG	0.637564	0.25290702	1
-0.012256	0.96844379	1	nonDEG	0.27385071	0.47747327	1
0.40436229	0.20355725	0.7766358	nonDEG	0.0209563	0.96025552	1
-1.3336851	0.09165316	0.55224625	nonDEG	0.47147078	0.61283065	1
0.09410057	0.74817921	1	nonDEG	0.10485535	0.78721434	1
-0.1132219	0.64272906	1	nonDEG	-0.0574383	0.87587063	1
0.19226433	0.45115315	0.98329655	nonDEG	0.01592831	0.96819246	1
0.15502223	0.57850357	1	nonDEG	0.18359559	0.6275927	1
0.0231227	0.98253176	1	nonDEG	-1.2976717	0.16327722	1
0.08566286	0.74008161	1	nonDEG	-0.1880714	0.62841274	1
0.23595629	0.38579713	0.94210008	nonDEG	0.19851453	0.59147513	1
0.45808925	0.06791427	0.47555692	nonDEG	-0.0336922	0.93214053	1
0.0503054	0.90052514	1	nonDEG	0.3547475	0.433028	1
-0.8682736	0.58170029	1	nonDEG	0.90766449	0.67764731	1
0.11429934	0.64562023	1	nonDEG	0.03787699	0.91577524	1
-0.1206995	0.6632123	1	nonDEG	-0.1295702	0.73360408	1
0.62384644	0.24296352	0.82500393	nonDEG	-1.7471063	0.00145954	0.27900964
-0.3367317	0.27878414	0.86049314	nonDEG	0.0700579	0.86805203	1
-0.2565168	0.46502513	0.98594705	nonDEG	-0.3355948	0.44997533	1
-0.4477123	0.08041835	0.51953337	nonDEG	0.20882809	0.58119728	1
0.14134357	0.83555068	1	nonDEG	-1.3231195	0.15717432	1
0.157142	0.57620033	1	nonDEG	0.06633403	0.86030327	1
-0.390196	0.1874426	0.75310345	nonDEG	0.04548488	0.91130474	1
-0.0699343	0.80692892	1	nonDEG	0.28112573	0.55916028	1
0.33417128	0.18165422	0.74732027	nonDEG	0.05190432	0.88683378	1
0.65452677	0.13988692	0.66813245	nonDEG	-0.287494	0.58330438	1
-0.916986	0.14029134	0.66901406	nonDEG	-0.3731084	0.69294217	1
0.02896146	0.95344488	1	nonDEG	-0.0848538	0.8772224	1
-0.1701325	0.51851093	1	nonDEG	0.11849765	0.74635291	1
-0.0997709	0.69116242	1	nonDEG	0.18280635	0.63232975	1
0.14544514	0.75071663	1	nonDEG	0.11184119	0.83540121	1
0.14819263	0.61210837	1	nonDEG	0.12949058	0.7269589	1
-0.3865299	0.18115075	0.74659063	nonDEG	-0.2340205	0.5591498	1
-0.3287798	0.17106719	0.7298619	nonDEG	0.1561838	0.67025196	1
-0.8389432	0.18033346	0.74551844	nonDEG	0.52690812	0.47821367	1
0.58774439	0.43408091	0.973123	nonDEG	0.0052354	1	1
5.13E-15	1	1	nonDEG	4.04373201	0.34921148	1
-0.0310575	0.89927308	1	nonDEG	-0.0864643	0.81527136	1
0.18447782	0.91152977	1	nonDEG	-3.0749718	0.01783907	0.95781535

-0.3432269	0.17832992	0.74433965	nonDEG	-0.0388515	0.91737974	1
0.2080812	0.39497892	0.94889705	nonDEG	-0.3405605	0.37568918	1
-0.2874133	0.48091537	0.99426825	nonDEG	-0.1617139	0.75648666	1
-0.1970185	0.448725	0.98329655	nonDEG	0.23507635	0.52779293	1
0.33790251	0.91046889	1	nonDEG	0.75012337	0.72427563	1
-0.2057094	0.4627546	0.98479636	nonDEG	0.16149872	0.67113331	1
0.02871419	1	1	nonDEG	-1.1387542	0.20375623	1
-0.445275	0.13132759	0.65483621	nonDEG	0.04259144	0.91741026	1
-0.1147739	0.66521762	1	nonDEG	-0.3416259	0.35764273	1
0.12482501	0.95305428	1	nonDEG	-3.4319128	0.0210843	1
0.5134601	0.03413146	0.32289859	nonDEG	0.04049059	0.9116207	1
-0.0260436	0.93944228	1	nonDEG	-0.1336966	0.75598743	1
0.21421907	0.43771943	0.97451579	nonDEG	0.15503877	0.69126769	1
-0.3525435	0.27876764	0.86049314	nonDEG	-0.366289	0.44169888	1
0.24380851	0.581366	1	nonDEG	0.32848582	0.50901072	1
0.24491883	0.33023759	0.91411259	nonDEG	0.25983392	0.47263726	1
-1.4824037	0.06319034	0.4607003	nonDEG	-1.0792595	0.33807715	1
0.01762933	0.94290627	1	nonDEG	0.26009966	0.47352358	1
-0.7295816	0.04517683	0.38020603	nonDEG	-0.0526776	0.92627146	1
0.19575145	0.53567855	1	nonDEG	0.13720787	0.73511016	1
-0.1486346	0.63515884	1	nonDEG	0.09048202	0.82817989	1
-0.3182142	0.4070962	0.95661757	nonDEG	0.18897054	0.6954147	1
-0.428671	0.25062359	0.83264958	nonDEG	0.35155566	0.45502148	1
-0.7852164	0.1317171	0.65533095	nonDEG	1.03975925	0.09015016	1
0.02680022	0.94305587	1	nonDEG	0.4891109	0.28249849	1
-0.0227372	0.94142127	1	nonDEG	0.02083617	0.96152454	1
0.16833755	0.50665223	1	nonDEG	-0.0004858	0.99953245	1
-0.0146199	0.96514454	1	nonDEG	0.09502444	0.81728974	1
-0.0820266	0.73761849	1	nonDEG	0.01407619	0.96962851	1
-0.2825899	0.29482068	0.8785531	nonDEG	0.19138843	0.63092057	1
-0.1705988	0.6703725	1	nonDEG	0.22069319	0.65280869	1
-0.1782073	0.53319674	1	nonDEG	-0.6290563	0.11046647	1
-0.1001096	0.7063472	1	nonDEG	0.00254509	0.99522859	1
0.09665392	0.72780756	1	nonDEG	-0.0365377	0.92141689	1
0.22556313	0.48024171	0.99366289	nonDEG	-0.0148932	0.97258895	1
1.00634877	0.00337258	0.06777013	nonDEG	0.23389432	0.65271548	1
0.68191302	0.01175406	0.16383895	nonDEG	0.19845587	0.60532663	1
-0.1604124	0.54375428	1	nonDEG	0.18906409	0.60300621	1
-0.1493394	0.57468177	1	nonDEG	-0.0476259	0.89751237	1
-0.0084144	0.97493407	1	nonDEG	-0.581448	0.12426451	1
-0.7407875	0.46388023	0.98544722	nonDEG	0.03409834	1	1
0.10489827	0.90475576	1	nonDEG	-1.2622099	0.21757654	1
0.02274128	0.93307855	1	nonDEG	-0.4074968	0.27103867	1

0.32652866	0.34521666	0.91869633	nonDEG	0.41725794	0.3159554	1
0.08395746	1	1	nonDEG	-1.2480106	0.71186826	1
-0.2912722	0.4974833	1	nonDEG	-0.2346966	0.6736125	1
0.3235906	0.20364345	0.77666791	nonDEG	0.26294775	0.4720669	1
-0.1368824	0.66712681	1	nonDEG	-0.1266375	0.77004545	1
-0.0782249	0.77899726	1	nonDEG	0.10587248	0.77113113	1
-0.0502985	0.84495427	1	nonDEG	0.17516521	0.64471208	1
-0.2537078	0.33160085	0.91533029	nonDEG	-0.0355493	0.92431068	1
-0.7215754	0.37813509	0.93529818	nonDEG	0.95980057	0.26730007	1
-0.0935144	0.79475436	1	nonDEG	0.50336919	0.23118818	1
0.31432914	0.50484345	1	nonDEG	-0.0594571	0.9169358	1
-0.2264517	0.37525849	0.93344578	nonDEG	-0.0004165	1	1
-1.2678072	0.04709334	0.39045749	nonDEG	-0.4308407	0.55304588	1
-0.4300422	0.13710541	0.66374713	nonDEG	-0.1030825	0.79956073	1
-0.5391902	0.06041352	0.44807419	nonDEG	0.03033778	0.93895362	1
0.07067381	0.78160456	1	nonDEG	-0.0154729	0.96712518	1
0.03820663	0.88034607	1	nonDEG	-0.1077369	0.76568623	1
-0.9198583	0.24631543	0.82796071	nonDEG	-1.6125212	0.12693765	1
0.03981689	1	1	nonDEG	-0.4967313	0.82698344	1
-0.1367722	0.61389345	1	nonDEG	0.0381919	0.91969845	1
0.61536169	0.03630349	0.3355268	nonDEG	-0.1511916	0.70678443	1
0.25950304	0.3090892	0.89265303	nonDEG	0.47674597	0.18622548	1
-0.0462442	0.95880556	1	nonDEG	0.46558234	0.53211638	1
-0.2174206	0.54942711	1	nonDEG	-0.0683115	0.8857178	1
0.39533161	0.14287832	0.67504331	nonDEG	0.02390604	0.95286882	1
-0.4874308	0.31755899	0.90044709	nonDEG	0.12875831	0.83036326	1
0.13178905	0.61019795	1	nonDEG	0.25705678	0.50328338	1
0.05697926	0.85018985	1	nonDEG	0.08389971	0.83043484	1
0.08713315	0.86051634	1	nonDEG	-0.1607229	0.79595397	1
0.0607067	0.80667947	1	nonDEG	0.44758484	0.22195895	1
0.26209646	0.32665369	0.91057556	nonDEG	0.58419208	0.12994744	1
0.54421282	0.23774089	0.81948793	nonDEG	0.11017579	0.82166813	1
0.50982463	0.07015164	0.48475818	nonDEG	-0.4129175	0.27709213	1
0.04127069	0.96110341	1	nonDEG	-0.1747312	0.83018294	1
0.78072207	0.01773591	0.21190239	nonDEG	-0.8252559	0.06861624	1
-0.1450495	0.56136423	1	nonDEG	0.20635998	0.57361396	1
-0.0835879	0.75161456	1	nonDEG	-0.1445647	0.70124855	1
-0.3788617	0.24710296	0.82862105	nonDEG	0.05276622	0.90785683	1
-0.0390926	0.87387968	1	nonDEG	-0.0184168	0.95975911	1
-0.5785156	0.20234671	0.77400965	nonDEG	-0.2969825	0.6188146	1
0.11969591	0.66025825	1	nonDEG	0.35326754	0.44957498	1
-0.036215	0.90445869	1	nonDEG	0.22447212	0.56372734	1
0.28796151	0.27828046	0.86017414	nonDEG	0.42000946	0.29207138	1

0.72258111	0.06298406	0.45963999	nonDEG	0.14558924	0.73832852	1
0.29449098	0.44472335	0.97966661	nonDEG	-0.3392781	0.45822909	1
0.22418589	0.48359792	0.99599492	nonDEG	-0.1407604	0.73372226	1
-0.0162072	1	1	nonDEG	-0.0437898	0.97703129	1
0.16133388	0.5414571	1	nonDEG	0.28739467	0.42938115	1
-0.2644419	0.40673236	0.95617157	nonDEG	-0.131944	0.75609631	1
-0.1846147	0.48444862	0.99623334	nonDEG	-0.0695476	0.85084836	1
0.50484072	0.07823068	0.51213419	nonDEG	-0.0976107	0.79316649	1
0.37961701	0.11668317	0.62477914	nonDEG	0.07537583	0.85059939	1
-0.1434297	0.55895502	1	nonDEG	0.17994159	0.63305557	1
-0.4900079	0.14870771	0.68531592	nonDEG	-0.2047765	0.66371409	1
0.31758255	0.29531284	0.87921873	nonDEG	-0.6193163	0.13026297	1
-0.0021274	1	1	nonDEG	0.71724276	0.36847033	1
-0.1404898	0.63108121	1	nonDEG	-0.1335316	0.73507804	1
6.39757586	0.00362473	0.07147255	nonDEG	-1.6337278	0.29478018	1
0.31445442	0.31600373	0.89925896	nonDEG	0.04041793	0.92029434	1
-0.0168911	0.94550916	1	nonDEG	0.22954897	0.53235792	1
-0.0450057	0.88158671	1	nonDEG	-0.0273764	0.94594337	1
-0.0884674	0.71743316	1	nonDEG	-0.0600862	0.87044747	1
-0.3689815	0.38566035	0.94196091	nonDEG	0.3630354	0.48062486	1
0.25930719	0.37941275	0.93602044	nonDEG	-0.1087515	0.78040469	1
0.31733146	0.6998689	1	nonDEG	0.16324151	0.8401934	1
-0.4349514	0.20219374	0.77400024	nonDEG	-0.1833863	0.69250142	1
1.10371382	0.03236683	0.31266985	nonDEG	-1.1296356	0.04824125	1
0.08796088	0.78114525	1	nonDEG	0.07776144	0.84755619	1
-0.4448786	0.20615331	0.78085836	nonDEG	-0.1383813	0.7578308	1
-0.1265616	0.81748076	1	nonDEG	0.2222638	0.7294896	1
-0.244493	0.31103827	0.89455408	nonDEG	-0.0356776	0.92442839	1
0.18611755	0.59041332	1	nonDEG	-0.4058493	0.38255956	1
-0.032371	0.92832052	1	nonDEG	-0.6493386	0.16213122	1
-0.1528109	0.68479505	1	nonDEG	-0.3773001	0.48405114	1
0.20886808	0.40016141	0.95204649	nonDEG	-0.0625203	0.867751	1
0.11029924	0.64587166	1	nonDEG	0.05332039	0.88144074	1
0.49965618	0.14344182	0.67589095	nonDEG	-0.8622187	0.059059	1
0.10182847	0.72085411	1	nonDEG	0.4662918	0.20134387	1
0.13648643	0.84663969	1	nonDEG	0.06867458	0.93387559	1
-0.2475153	0.33057508	0.91430575	nonDEG	-0.2422125	0.51260413	1
0.2552118	0.31940068	0.90247016	nonDEG	0.18866462	0.61185845	1
0.69942035	0.05972563	0.44543966	nonDEG	-0.5578643	0.28249094	1
0.04166257	0.88993456	1	nonDEG	0.67833347	0.08129149	1
-0.0596237	0.84443918	1	nonDEG	0.3325751	0.40912185	1
-0.1165749	0.63200114	1	nonDEG	-0.3587847	0.34957954	1
-0.0165205	0.94940701	1	nonDEG	0.23145849	0.52669389	1

-0.8913368	0.08183907	0.52341091	nonDEG	0.16283528	0.80224807	1
-0.3380425	0.18460882	0.75241575	nonDEG	-0.0844265	0.82147834	1
-0.5136151	0.19961238	0.77239601	nonDEG	-0.2066356	0.69348884	1
-0.1154713	0.64679942	1	nonDEG	-0.1134378	0.765126	1
-0.1049466	0.73292776	1	nonDEG	0.11071624	0.78554169	1
-0.0547802	0.84798321	1	nonDEG	-0.1147805	0.76347907	1
0.09974132	0.67964301	1	nonDEG	0.01697995	0.96734994	1
0.01429559	0.9539306	1	nonDEG	-0.2065361	0.56947917	1
0.10339489	0.67237878	1	nonDEG	0.35976079	0.31837917	1
-0.3281272	0.29409657	0.87719441	nonDEG	0.01687357	0.96994779	1
-0.1901428	0.60890892	1	nonDEG	0.06961354	0.88721706	1
0.27450764	0.34542695	0.91869633	nonDEG	-0.1761374	0.6286068	1
0.74219537	0.04291906	0.3689034	nonDEG	-0.4529715	0.30198095	1
0.37138946	0.54218958	1	nonDEG	-1.0061185	0.12994279	1
0.09286615	0.9113516	1	nonDEG	-0.3476198	0.68010559	1
0.4515079	0.36728277	0.92691278	nonDEG	-0.7462637	0.19535347	1
-0.2570579	0.32084362	0.9041486	nonDEG	-0.0889807	0.8130841	1
0.12865422	0.65403423	1	nonDEG	0.24873583	0.50864505	1
0.0586907	0.82522379	1	nonDEG	0.44933078	0.24127739	1
-0.4158847	0.15395325	0.69445075	nonDEG	0.26159013	0.57989815	1
0.14111845	0.57839091	1	nonDEG	-0.0004955	0.9995481	1
0.11236534	0.86874882	1	nonDEG	-0.3165253	0.69924456	1
0.18422397	0.46524297	0.98605384	nonDEG	0.18358073	0.61881638	1
0.32358713	0.18162454	0.74732027	nonDEG	0.05579598	0.88272053	1
0.23173715	0.46139222	0.98479636	nonDEG	-0.0822878	0.83462329	1
0.15113022	0.54147285	1	nonDEG	0.24576217	0.49450089	1
0.16022882	0.75920159	1	nonDEG	-0.2862031	0.65017154	1
-0.0770721	0.81172318	1	nonDEG	-0.1606149	0.70029004	1
0.05947656	0.87457861	1	nonDEG	-0.192923	0.67513175	1
0.08568345	0.81953836	1	nonDEG	-0.5222248	0.27447518	1
0.04446384	0.92888699	1	nonDEG	-0.5663098	0.30089903	1
-0.000254	0.99991673	1	nonDEG	0.17415953	0.69241254	1
0.47363726	0.22968026	0.80924485	nonDEG	0.09715355	0.83270443	1
0.44992972	0.06678504	0.47261651	nonDEG	-0.1600935	0.65733856	1
0.22847217	0.35997017	0.92312867	nonDEG	0.25642129	0.48683297	1
-0.1608134	0.53845276	1	nonDEG	-0.0440539	0.9042424	1
-0.4108742	0.24681736	0.82856236	nonDEG	-0.518559	0.24424291	1
-0.0819206	0.74792422	1	nonDEG	0.12306638	0.76104075	1
0.20019443	0.44352527	0.97907473	nonDEG	0.49389351	0.23001892	1
-0.0354983	0.88680626	1	nonDEG	-0.0644871	0.86223486	1
0.17159994	0.56865804	1	nonDEG	0.04720889	0.90623877	1
-0.1820868	0.46689961	0.98651098	nonDEG	-0.0284557	0.93821254	1
-0.4303218	0.08094372	0.52062532	nonDEG	0.14659384	0.75354559	1

0.85869096	0.82604058	1	nonDEG	0.41898698	0.87828528	1
0.09109165	0.72556125	1	nonDEG	0.45721682	0.22100718	1
-0.3327412	0.73991145	1	nonDEG	-1.9643348	0.06307834	1
0.07767481	0.75007018	1	nonDEG	-0.062058	0.86487773	1
-0.4149301	0.17124512	0.72989601	nonDEG	0.5365199	0.22440472	1
-0.054515	0.89277922	1	nonDEG	0.19333661	0.70474433	1
0.30857987	0.41835702	0.96254612	nonDEG	-0.1846726	0.69182716	1
0.1056855	0.71850645	1	nonDEG	-0.0570728	0.88515564	1
0.20361657	0.44649513	0.98124647	nonDEG	-0.2438908	0.53998985	1
0.26487702	0.38496784	0.94140822	nonDEG	0.10922097	0.79387954	1
0.41891242	0.24767921	0.82913671	nonDEG	0.04175277	0.92752417	1
-0.0259525	0.92339766	1	nonDEG	0.30830111	0.41450247	1
-0.6385729	0.09863789	0.57217752	nonDEG	-0.1962447	0.70559721	1
-0.1091518	0.69744192	1	nonDEG	0.2136468	0.61427427	1
0.47563205	0.09458829	0.56106882	nonDEG	-0.3093698	0.41718128	1
-0.2779467	0.29510368	0.87912946	nonDEG	0.28760821	0.489172	1
-0.0927786	0.80859312	1	nonDEG	0.14950103	0.75568507	1
0.00837023	0.97489864	1	nonDEG	0.19168419	0.5991983	1
-0.1231919	0.71513503	1	nonDEG	-0.0243112	0.96008482	1
-0.3262465	0.1808911	0.74646106	nonDEG	0.00210075	0.99541751	1
0.31460967	0.45067675	0.98329655	nonDEG	0.45447695	0.33879909	1
-0.0451567	0.89673861	1	nonDEG	0.607687	0.17192493	1
-0.0411716	0.87329577	1	nonDEG	-0.3554181	0.32895067	1
0.12149869	0.68384495	1	nonDEG	0.34045848	0.38629667	1
-0.3415267	0.1658437	0.71910791	nonDEG	0.35076219	0.39016192	1
-0.3268725	0.19792716	0.77002482	nonDEG	-0.1716126	0.63515821	1
0.21797517	0.51321007	1	nonDEG	0.43971944	0.35143543	1
-0.102122	0.68433675	1	nonDEG	0.19706885	0.58467627	1
-0.0504045	0.84424704	1	nonDEG	-0.0931085	0.80064806	1
1.40393079	0.00480919	0.08607335	nonDEG	-1.1898683	0.03975201	1
0.55975237	0.69158737	1	nonDEG	0.9521231	0.39770649	1
-0.8508023	0.00940703	0.13958347	nonDEG	0.20992144	0.62482472	1
0.12954815	0.76243466	1	nonDEG	-0.8203258	0.09817461	1
1.03728445	0.01851648	0.21778129	nonDEG	-0.5410976	0.27899543	1
0.09410473	0.69948074	1	nonDEG	0.330898	0.35662494	1
-0.1590009	0.67934593	1	nonDEG	0.41101682	0.38018764	1
-1.0367705	0.02873102	0.29080021	nonDEG	0.97020319	0.06829232	1
0.94780644	0.08242749	0.52512072	nonDEG	0.07436038	0.90558323	1
-0.1079606	0.92771434	1	nonDEG	0.07695674	0.94291719	1
-0.423065	0.54650847	1	nonDEG	1.04768647	0.15511586	1
0.47666097	0.21336732	0.79119796	nonDEG	-0.2146828	0.63899711	1
-0.2136923	0.4455968	0.98041193	nonDEG	-0.0845477	0.82947953	1
0.61965027	0.0140543	0.18258802	nonDEG	0.01569731	0.96611114	1

0.28974093	0.26199722	0.84580943	nonDEG	-0.2616859	0.47926973	1
0.8290508	0.07288392	0.49302153	nonDEG	-0.1701135	0.78003185	1
-1.6846051	0.00699512	0.11283801	nonDEG	-0.3024873	0.70107028	1
-0.0781592	0.78792386	1	nonDEG	-0.7252276	0.08052898	1
0.20579756	0.40498775	0.95502941	nonDEG	0.11439555	0.75069337	1
-0.1851033	0.44497021	0.97983978	nonDEG	0.1331929	0.70836118	1
0.10029558	0.7057222	1	nonDEG	0.25187436	0.50474723	1
-3.6154412	0.05367476	0.41969541	nonDEG	2.52647869	0.37564392	1
-0.3626771	0.86374481	1	nonDEG	-5.415346	0.0691079	1
0.092037	0.73065538	1	nonDEG	0.21265774	0.55891826	1
-0.1644837	0.605703	1	nonDEG	-0.0217956	0.96018276	1
0.44145761	0.67341175	1	nonDEG	-1.7290949	0.14930718	1
-0.1064904	0.82845849	1	nonDEG	0.1746626	0.74834084	1
-0.1357262	0.72397052	1	nonDEG	0.20682582	0.65992005	1
0.353674	0.64487605	1	nonDEG	1.25164683	0.17327134	1
-0.7729277	0.00438379	0.08142707	nonDEG	0.91779451	0.02180673	1
0.04216303	0.86789214	1	nonDEG	-0.3603774	0.35712443	1
-0.1667368	0.49677189	1	nonDEG	-0.101941	0.7834093	1
0.05883436	0.80980385	1	nonDEG	0.18826449	0.64606266	1
0.41966123	0.08167513	0.52307859	nonDEG	-0.0807115	0.83172393	1
-0.3609322	0.17909444	0.744789	nonDEG	0.26977321	0.50379667	1
0.3969877	0.15588319	0.69965621	nonDEG	0.2881385	0.43767296	1
0.0136495	0.95675986	1	nonDEG	0.14378645	0.69008788	1
-0.0472678	0.9161636	1	nonDEG	0.0756233	0.9004436	1
0.016668	0.96419158	1	nonDEG	0.01213963	0.98034452	1
-1.2013309	0.23329083	0.81342102	nonDEG	0.54627709	0.66792307	1
-0.005061	0.98395665	1	nonDEG	0.09712522	0.7946834	1
0.0739557	0.79754087	1	nonDEG	0.35765367	0.36859717	1
0.73902066	0.04044419	0.35690547	nonDEG	-0.1726916	0.69134886	1
-0.00777	0.98024357	1	nonDEG	-0.4781314	0.22028198	1
-0.2705405	0.67368789	1	nonDEG	-1.2801238	0.12758996	1
0.40963246	0.2487525	0.82990493	nonDEG	0.19920936	0.64414936	1
-1.7692166	0.13643875	0.66368058	nonDEG	-0.0007672	1	1
0.17111354	0.6657392	1	nonDEG	-0.219589	0.64798526	1
-0.1192035	0.7777567	1	nonDEG	-0.1518772	0.7666962	1
-0.1281091	0.59818347	1	nonDEG	0.20223344	0.57686915	1
-0.456326	0.18511909	0.75274479	nonDEG	0.37603621	0.39672181	1
-0.2578375	0.33707591	0.91869633	nonDEG	0.2054127	0.58559998	1
0.34169787	0.80116795	1	nonDEG	-2.2456494	0.11734197	1
-0.1027119	0.74828936	1	nonDEG	-0.1792659	0.66590051	1
-0.3633258	0.25219959	0.83371668	nonDEG	-0.1776379	0.67813171	1
-0.5109621	0.05227431	0.41435203	nonDEG	0.55944956	0.12531242	1
0.0735124	0.77516051	1	nonDEG	-0.0316077	0.93214366	1

-0.186081	0.52138356	1	nonDEG	0.11296731	0.77621896	1
0.13785932	0.60312529	1	nonDEG	-0.0650526	0.85750306	1
0.19840581	0.42002887	0.96294915	nonDEG	0.13352852	0.72008321	1
-0.4873688	0.22920427	0.80924485	nonDEG	-0.6477587	0.14071206	1
-0.0869445	0.93056495	1	nonDEG	-2.1062508	0.03544113	1
-0.1409703	0.67393291	1	nonDEG	-0.0173131	0.97002831	1
0.08073454	0.97026496	1	nonDEG	-1.0413442	0.28129781	1
-0.2044424	0.53060421	1	nonDEG	0.15620582	0.70896369	1
-0.2446478	0.43962799	0.97632833	nonDEG	0.14051193	0.72950426	1
0.03360769	0.89385768	1	nonDEG	0.63719137	0.08318476	1
-0.0908876	1	1	nonDEG	-1.683201	0.44206769	1
0.45981584	0.36172224	0.92354131	nonDEG	0.41872847	0.51162716	1
-0.0374612	0.94472241	1	nonDEG	0.12378742	0.82502558	1
0.16977961	0.6190787	1	nonDEG	0.08573434	0.84050893	1
-0.7676742	0.00252642	0.0542886	nonDEG	0.1472803	0.71272622	1
-0.2439358	0.38522814	0.94140822	nonDEG	-0.0325966	0.93156614	1
-0.4164118	0.09829716	0.57137375	nonDEG	0.13559433	0.7318153	1
0.44073211	0.08347432	0.52850582	nonDEG	-0.2264177	0.53289068	1
0.84421707	0.32330887	0.90698398	nonDEG	0.21711346	0.8453995	1
-0.2727218	0.41830036	0.96254612	nonDEG	-0.0259082	0.95651696	1
0.16181402	0.59053085	1	nonDEG	0.36942615	0.35701874	1
-1.556985	0.07267672	0.49207223	nonDEG	0.85376737	0.37605348	1
-0.5191213	0.07170414	0.48874697	nonDEG	0.21274997	0.6228755	1
-0.1612466	0.50553776	1	nonDEG	0.06751484	0.85419592	1
0.55809456	0.24521772	0.8265359	nonDEG	-0.053131	0.92367562	1
0.76640908	0.63594119	1	nonDEG	-0.1133323	0.97075635	1
-0.1872511	0.45031334	0.98329655	nonDEG	0.12863826	0.72950847	1
0.55617399	0.03073083	0.3025702	nonDEG	-0.3171497	0.42497487	1
0.11284332	0.65275052	1	nonDEG	0.21917487	0.55368721	1
5.13E-15	1	1	nonDEG	0	1	1
-0.1485721	0.57654968	1	nonDEG	-0.1684257	0.64456545	1
-0.009617	0.97969566	1	nonDEG	0.25820804	0.53062056	1
0.08732	0.76254258	1	nonDEG	-0.0506357	0.89566181	1
-0.1500898	0.6080784	1	nonDEG	-0.0466199	0.9127013	1
0.82926567	0.01666322	0.20298236	nonDEG	0.0016561	0.99854977	1
-0.357359	0.25938002	0.84107657	nonDEG	-0.3060977	0.46972884	1
-0.6448436	0.70494779	1	nonDEG	1.28462874	0.36944563	1
0.38187194	0.13664301	0.66368058	nonDEG	-0.2638637	0.46869975	1
-0.1417877	0.59374193	1	nonDEG	0.0184243	0.96113072	1
-0.0398435	0.87130491	1	nonDEG	0.25872262	0.47202107	1
0.7248943	0.00379567	0.07384488	nonDEG	0.19860067	0.59070648	1
0.03708003	0.89352151	1	nonDEG	0.15557398	0.69861233	1
-0.0964699	0.70927545	1	nonDEG	0.35112191	0.33506464	1

-1.8441467	0.02481767	0.26366198	nonDEG		1.57277019	0.09832454	1
-0.2657662	0.38009689	0.93639703	nonDEG		0.22769354	0.56431352	1
0.19936182	0.44532439	0.98032637	nonDEG		-0.1159441	0.75227532	1
0.87512787	0.25362497	0.83508169	nonDEG		-0.0005381	1	1
-0.0323578	0.89648625	1	nonDEG		-0.015311	0.96727183	1
1.35894642	0.16440506	0.71511831	nonDEG		-0.5279927	0.60062654	1
-1.6898953	0.03808085	0.34388407	nonDEG		0.88373222	0.35960811	1
0.47359292	0.3729594	0.93165867	nonDEG		-0.3997012	0.50108097	1
-0.3586362	0.17860713	0.74433965	nonDEG		0.15462591	0.67962179	1
-0.236237	0.39650128	0.9493779	nonDEG		-0.0261219	0.94780417	1
1.69336464	0.60688995	1	nonDEG		-1.4177185	0.61865527	1
-0.096681	0.71731664	1	nonDEG		0.05471563	0.88369629	1
-0.0060563	1	1	nonDEG		-0.0149444	1	1
0.20260952	0.482967	0.99581563	nonDEG		0.53929588	0.14623112	1
-0.2248522	0.39887915	0.95191286	nonDEG		0.29403426	0.43190459	1
1.42228121	0.00463886	0.08424634	nonDEG		-0.7541852	0.26546837	1
-0.252931	0.33419444	0.91780851	nonDEG		0.2335038	0.52393737	1
0.82558849	0.02937116	0.29475369	nonDEG		0.12701764	0.7599811	1
-0.8218919	0.57717542	1	nonDEG		-7.7164805	0.0071026	0.66803023
-0.0277537	0.90824807	1	nonDEG		0.13565246	0.70451916	1
-0.001878	0.99490468	1	nonDEG		0.18885584	0.60065136	1
-0.2171911	0.47828845	0.99305902	nonDEG		-0.6343917	0.13665633	1
-0.8354902	0.00374773	0.07335733	nonDEG		0.2048276	0.60165698	1
-0.1592538	0.76174241	1	nonDEG		0.39749256	0.51692498	1
1.69574952	0.171469	0.72989601	nonDEG		-1.6997415	0.18008185	1
-1.6418749	0.02640983	0.2749218	nonDEG		0.38014209	0.72554723	1
0.15497876	0.60290547	1	nonDEG		0.30809739	0.44898328	1
1.66438124	0.03504302	0.32853056	nonDEG		-0.4241017	0.60580255	1
0.1670318	0.51918037	1	nonDEG		0.25964091	0.47246354	1
-0.0755196	0.89781085	1	nonDEG		0.49529827	0.45734229	1
0.08313648	0.76002838	1	nonDEG		-0.0322263	0.9317758	1
-3.4670124	0.01642285	0.20111157	nonDEG		3.32638324	0.00507863	0.56641847
0.71777825	0.03524265	0.3297865	nonDEG		-0.5491045	0.23682536	1
-0.177035	0.49100236	0.99949565	nonDEG		-0.0680074	0.85041913	1
-0.3631485	0.1402551	0.66900939	nonDEG		-0.1199819	0.73946707	1
-0.5954216	0.18503875	0.75270911	nonDEG		0.43836414	0.41362461	1
0.27216505	0.2849548	0.86717396	nonDEG		0.16849558	0.64189007	1
-0.0568458	0.82414036	1	nonDEG		0.30633637	0.39789126	1
0.26665989	0.43750693	0.97423703	nonDEG		0.2369182	0.57840987	1
0.52325171	0.06481928	0.46553181	nonDEG		-0.179836	0.6388079	1
0.06552198	0.83843499	1	nonDEG		0.24327918	0.54922999	1
-0.2599346	0.2869095	0.8695006	nonDEG		0.13467743	0.71028979	1
0.20464261	0.43436417	0.973123	nonDEG		-0.2350092	0.52445173	1

-0.177427	0.49092212	0.99946448	nonDEG		-0.1055844	0.77803991	1
0.28585513	0.31780208	0.90063298	nonDEG		-0.2831793	0.46709272	1
-0.046217	0.87691433	1	nonDEG		0.28906607	0.45958757	1
-0.2042528	0.57696683	1	nonDEG		0.34910941	0.43236871	1
0.12901662	0.700916	1	nonDEG		-0.1205347	0.77813632	1
0.89781651	0.06427811	0.46390865	nonDEG		-0.5963159	0.27448784	1
-0.2943444	0.53386133	1	nonDEG		0.11745112	0.83596008	1
-0.463975	0.0722097	0.49026324	nonDEG		0.05297793	0.88515338	1
-0.0354281	0.92468869	1	nonDEG		-0.1057741	0.81997084	1
-0.3108112	0.72612207	1	nonDEG		-1.2989202	0.16166828	1
0.15805739	0.62972209	1	nonDEG		-0.5280429	0.22731514	1
0.18056678	0.75912315	1	nonDEG		0.05566761	0.93108846	1
-1.4161938	0.06027096	0.44787964	nonDEG		-1.8052822	0.1180504	1
0.11314829	0.91334495	1	nonDEG		0.81881522	0.34976	1
0.20513262	0.5555658	1	nonDEG		0.15154215	0.71225421	1
0.06940835	0.81421802	1	nonDEG		-0.4215846	0.27660545	1
-0.0301424	0.92426869	1	nonDEG		0.11936477	0.77549335	1
0.25443395	0.30912343	0.89265303	nonDEG		-0.1099058	0.76341537	1
0.20111128	0.44559285	0.98041193	nonDEG		0.2925365	0.44528406	1
0.69977772	0.01603458	0.19867089	nonDEG		-0.5232855	0.17274467	1
0.05280521	0.8624417	1	nonDEG		0.07420816	0.87020765	1
0.18016013	0.45339764	0.98339152	nonDEG		0.35039441	0.41117946	1
-0.1450764	0.60939745	1	nonDEG		0.13758191	0.71788879	1
-0.163218	0.77568816	1	nonDEG		-0.3190763	0.63871113	1
0.45088959	0.57579962	1	nonDEG		-0.9833338	0.25919128	1
0.2218301	0.46354857	0.98522895	nonDEG		-0.1111346	0.78421348	1
0.20948358	0.40323108	0.95382347	nonDEG		0.16073744	0.6853345	1
0.56242377	0.33829281	0.91869633	nonDEG		-0.1036306	0.8894394	1
-0.0311251	0.92611323	1	nonDEG		-0.0217773	0.96067144	1
0.0928897	0.70478057	1	nonDEG		-0.4735101	0.20619967	1
-0.1012962	0.85039126	1	nonDEG		0.34037539	0.5818575	1
0.39404136	0.11916875	0.63063362	nonDEG		0.1590344	0.67339828	1
-0.3484192	0.16592768	0.71910791	nonDEG		-0.1491609	0.68665904	1
-0.1202075	0.70980885	1	nonDEG		0.41231161	0.3045738	1
1.28084325	0.01316881	0.17462252	nonDEG		1.13082984	0.09050272	1
-0.1562203	0.64632235	1	nonDEG		-0.5848449	0.18407441	1
0.12861627	0.6564469	1	nonDEG		0.40876073	0.26664298	1
0.04937528	0.85452574	1	nonDEG		0.01911523	0.95897064	1
-0.2431651	0.32410771	0.90754391	nonDEG		-0.1826154	0.61324971	1
-0.2217134	0.65424043	1	nonDEG		0.20668533	0.71375572	1
-0.0929982	0.74645804	1	nonDEG		0.14018825	0.72792347	1
-0.0228834	0.94260225	1	nonDEG		-0.7676347	0.06875938	1
0.76264482	0.00271449	0.05716154	nonDEG		0.03920671	0.92555976	1

-0.2997936	0.33442171	0.91780851	nonDEG	0.27468193	0.50680584	1
0.13507554	0.71898297	1	nonDEG	-0.1526049	0.75445608	1
-0.861416	0.76734315	1	nonDEG	0.09202392	1	1
0.0304778	0.90991902	1	nonDEG	0.12375954	0.73511676	1
-0.6992437	0.6435053	1	nonDEG	-6.831186	0.00197368	0.34792358
-0.1153391	0.64250451	1	nonDEG	0.12268805	0.73530067	1
-0.6459477	0.44133711	0.97752398	nonDEG	0.13059276	0.90914565	1
0.78388937	0.69229091	1	nonDEG	-0.077029	1	1
-0.00759	0.98877615	1	nonDEG	0.38558942	0.47677675	1
-0.8358525	0.38997387	0.94686517	nonDEG	0.82722736	0.43523244	1
-1.6359113	0.61798314	1	nonDEG	1.73808106	0.61378258	1
-0.2662392	0.7046441	1	nonDEG	0.31575951	0.67980604	1
-0.5290545	0.10886392	0.60347345	nonDEG	-0.1493442	0.72942261	1
0.08384904	0.75047063	1	nonDEG	-0.0997889	0.78635357	1
-0.828814	0.27383494	0.85750738	nonDEG	0.56100367	0.50819829	1
-5.4187164	0.11205176	0.61181363	nonDEG	3.14234139	0.64148328	1
0.202348	0.46674708	0.98639597	nonDEG	-0.3754068	0.34235157	1
0.30454043	0.28020738	0.86186717	nonDEG	0.31690097	0.38833523	1
-1.4089702	0.04802759	0.39505006	nonDEG	-0.2311823	0.81880037	1
-0.2236407	0.63605354	1	nonDEG	-0.1641195	0.77714487	1
-0.4126843	0.30834085	0.89225736	nonDEG	-0.016844	0.97973164	1
-0.1367515	0.64189002	1	nonDEG	0.51401776	0.18242346	1
-0.432768	0.11413893	0.61721821	nonDEG	-0.0817971	0.8313372	1
-0.0043538	0.98699088	1	nonDEG	0.01599319	0.9692879	1
0.15100615	0.54349404	1	nonDEG	0.38327028	0.31002619	1
0.77734092	0.68388108	1	nonDEG	0.58403093	0.7828808	1
-0.3432414	0.25276871	0.83393344	nonDEG	-0.0273186	0.94811207	1
-0.1113603	0.75664499	1	nonDEG	0.02121328	0.96619938	1
-0.0018391	0.99613657	1	nonDEG	0.02303992	0.95181664	1
-0.0250785	0.96490482	1	nonDEG	-0.1095365	0.84247746	1
-0.0167076	0.95761228	1	nonDEG	-0.1793044	0.64873359	1
0.11675736	0.67459396	1	nonDEG	-0.0029535	0.99509177	1
0.53764028	0.49066196	0.99934949	nonDEG	-1.2572141	0.15863939	1
-0.0059253	0.98345082	1	nonDEG	0.62942197	0.08379914	1
5.13E-15	1	1	nonDEG	0	1	1
0.17061004	0.50506239	1	nonDEG	0.02141153	0.95359063	1
0.1099525	0.69504412	1	nonDEG	-0.1984451	0.59906507	1
-0.4059554	0.14226979	0.67359585	nonDEG	0.00099763	0.99913294	1
0.06077209	0.89777089	1	nonDEG	-0.1137585	0.84722601	1
0.09575508	0.86101231	1	nonDEG	0.43809238	0.44491318	1
0.61693206	0.01479398	0.18837366	nonDEG	0.07709553	0.84550239	1
-0.0196491	0.94965128	1	nonDEG	0.07573043	0.84802792	1
0.04922413	0.87929432	1	nonDEG	0.2554171	0.5189909	1

-0.0534349	0.86186224	1	nonDEG	-0.0441195	0.91439051	1
1.31511286	0.34617883	0.91869633	nonDEG	-0.1133936	0.92849544	1
-0.768934	0.83554001	1	nonDEG	-3.16245	0.63923484	1
-0.5949605	0.06518264	0.46677686	nonDEG	0.28198727	0.50877286	1
-1.8103685	0.1254697	0.64431252	nonDEG	2.52319142	0.02749405	1
-0.4752306	0.09230044	0.55374621	nonDEG	0.11857321	0.76727973	1
-0.1768806	0.4980201	1	nonDEG	-0.2893599	0.44266002	1
-0.5821331	0.11685876	0.62512815	nonDEG	0.2856189	0.54975104	1
0.11140763	0.72398681	1	nonDEG	0.31097486	0.43975742	1
-0.1622593	0.66862451	1	nonDEG	-0.1396375	0.77457162	1
-0.2800327	0.39623952	0.9493779	nonDEG	0.4593026	0.29778691	1
-0.1346498	0.5907162	1	nonDEG	-0.0430334	0.90860453	1
-0.1712705	0.48899204	0.99836609	nonDEG	0.30814091	0.43676965	1
-1.0420991	0.18949886	0.75564367	nonDEG	-3.0766436	0.03187938	1
5.13E-15	1	1	nonDEG	0	1	1
-0.4622287	0.26582998	0.85062515	nonDEG	-0.1607771	0.76846433	1
1.42149983	0.03904414	0.34897353	nonDEG	-1.5423786	0.04955928	1
0.24447285	0.32650001	0.91057556	nonDEG	0.01752117	0.96485918	1
-0.0838235	0.80061476	1	nonDEG	-0.2595856	0.55317159	1
-0.2106526	0.57560074	1	nonDEG	-0.0548628	0.91025842	1
0.8462704	0.19453644	0.76452875	nonDEG	0.1939029	0.77745144	1
0.01444941	0.9875785	1	nonDEG	0.23769354	0.73490968	1
0.02569045	0.92038634	1	nonDEG	0.01090776	0.97709465	1
0.71917736	0.0217317	0.24178361	nonDEG	0.29830975	0.48419758	1
-0.1316509	0.59194365	1	nonDEG	0.08639263	0.81145111	1
0.0524661	0.83798059	1	nonDEG	0.06809917	0.8566741	1
-0.0757536	0.76045887	1	nonDEG	0.4004607	0.28618374	1
-0.1210125	0.77127884	1	nonDEG	0.05635344	0.91515335	1
0.41181057	0.109434	0.60460927	nonDEG	0.24684104	0.55791608	1
0.43777297	0.09747746	0.57005142	nonDEG	-0.4284035	0.23901827	1
0.34313697	0.1821059	0.74823282	nonDEG	0.05973642	0.87530513	1
-0.0835882	0.87642903	1	nonDEG	-0.4820128	0.44440688	1
-0.5334312	0.25057136	0.83257002	nonDEG	0.57249444	0.32337328	1
-0.1736101	0.69139821	1	nonDEG	-0.3711758	0.49471689	1
-0.1453549	0.60275194	1	nonDEG	-0.2247903	0.58542288	1
-0.0181402	0.98073449	1	nonDEG	-0.6909591	0.27131407	1
0.28044827	0.28549666	0.86772002	nonDEG	-0.3510127	0.41428331	1
0.18172404	0.70771383	1	nonDEG	-0.728179	0.20839052	1
0.54949699	0.05531867	0.42687452	nonDEG	-0.0590164	0.87796202	1
4.23474369	0.34506714	0.91869633	nonDEG	-4.0861567	0.35229895	1
-0.4910844	0.09546611	0.56377669	nonDEG	0.0660149	0.87361579	1
0.78865288	0.0088305	0.1336841	nonDEG	-0.5865836	0.16484015	1
0.20505679	0.41247113	0.95908664	nonDEG	0.13048931	0.7182451	1

0.08662937	0.75022813	1	nonDEG	0.0592906	0.87390166	1
0.49024136	0.36567	0.92576013	nonDEG	0.11388438	0.88624812	1
-0.4638755	0.24237007	0.82439569	nonDEG	-0.5599938	0.28891201	1
0.10326199	0.72927612	1	nonDEG	0.27866472	0.48118704	1
0.64217761	0.8926829	1	nonDEG	0.82226076	0.72218731	1
0.00163606	0.99743472	1	nonDEG	0.55845566	0.14099116	1
0.01270105	0.98019128	1	nonDEG	0.38992075	0.43183575	1
-0.0459485	0.8993515	1	nonDEG	0.0919317	0.84589976	1
0.32074289	0.22058854	0.80037926	nonDEG	-0.0280162	0.93952943	1
0.54147522	0.06091367	0.45078869	nonDEG	0.08287969	0.82216206	1
0.12267819	0.71130312	1	nonDEG	0.3905198	0.33569684	1
0.92808398	0.00810501	0.12584561	nonDEG	-0.480553	0.26182426	1
-0.3689702	0.30098821	0.8855319	nonDEG	0.31513522	0.48603341	1
0.03145547	0.93079076	1	nonDEG	0.12352681	0.7748795	1
0.17265617	0.71798138	1	nonDEG	0.67020973	0.21082589	1
0.00714039	0.98779946	1	nonDEG	0.23098768	0.61474961	1
0.08811831	0.76161247	1	nonDEG	0.30855889	0.45787097	1
0.31090323	0.3348797	0.91849589	nonDEG	-0.117485	0.76207177	1
-0.5875836	0.03117065	0.30557203	nonDEG	0.96737638	0.02080028	1
-3.1444188	0.64615164	1	nonDEG	4.98798293	0.15716726	1
-0.2975493	0.86046601	1	nonDEG	1.87574967	0.24323498	1
0.70422506	0.00395044	0.07536309	nonDEG	0.14417143	0.70607959	1
2.15959038	0.01615556	0.19933068	nonDEG	-0.4053998	0.64506507	1
1.61653494	0.04633104	0.38704295	nonDEG	-0.0929456	0.9153641	1
-0.282658	0.50028394	1	nonDEG	-0.0054313	0.99558422	1
0.74103208	0.25455995	0.8362895	nonDEG	-0.6781716	0.37256518	1
0.70452852	0.05326153	0.41819366	nonDEG	-0.86358	0.04158493	1
-0.2452986	0.66456741	1	nonDEG	-0.859453	0.20812642	1
0.54369879	0.17771921	0.74368492	nonDEG	-0.1844274	0.69893169	1
-0.0840121	0.74173678	1	nonDEG	0.13131233	0.74936294	1
0.42967838	0.07481607	0.50125404	nonDEG	-0.2629828	0.46981172	1
-0.2625763	0.37532517	0.93344578	nonDEG	0.00023066	1	1
-0.4078645	0.10481963	0.59272458	nonDEG	0.23724522	0.51295314	1
-0.089139	0.97110205	1	nonDEG	-0.3857754	0.78413141	1
-0.1952664	0.4543317	0.98394385	nonDEG	0.41818396	0.29177896	1
-0.475116	0.09906077	0.57383715	nonDEG	-0.0528654	0.89641804	1
-0.0182315	0.94378581	1	nonDEG	0.03242995	0.93193171	1
0.19047268	0.43311334	0.97242402	nonDEG	0.25981591	0.46990624	1
0.99613612	0.11671865	0.62485546	nonDEG	-0.1677264	0.81667561	1
0.74326789	0.00263041	0.05579158	nonDEG	-0.3547052	0.32147901	1
0.23104061	0.37806448	0.93529818	nonDEG	-0.1341804	0.7143628	1
-0.5146005	0.15262719	0.69158473	nonDEG	-0.1103835	0.81897213	1
-0.2356225	0.38184136	0.93796465	nonDEG	-0.1789253	0.63330244	1

-0.5267175	0.2108913	0.7877265	nonDEG		0.0458222	0.93179426	1
0.09488971	0.70121468	1	nonDEG		0.16659142	0.64597772	1
-0.3598025	0.44667649	0.98139482	nonDEG		0.27881473	0.61999357	1
0.09965831	0.69699659	1	nonDEG		-0.0241648	0.95044538	1
0.46191395	0.06574235	0.46861718	nonDEG		0.04910858	0.89296917	1
-0.0373356	0.88715695	1	nonDEG		0.57268357	0.11178941	1
-0.8584726	0.00780957	0.12268188	nonDEG		0.12585592	0.77006796	1
0.33048124	0.24284294	0.82500393	nonDEG		0.20388293	0.59291999	1
-0.3213054	0.27818558	0.86008704	nonDEG		-0.082356	0.84150397	1
-0.3920201	0.20133889	0.77336631	nonDEG		-0.0067356	0.98853796	1
0.22565975	0.89930233	1	nonDEG		-2.6758173	0.13567584	1
-0.3382328	0.19195428	0.76035391	nonDEG		0.07920384	0.842626	1
0.63677065	0.02199004	0.24410441	nonDEG		-0.2122555	0.59441092	1
-0.061958	0.82980561	1	nonDEG		0.60910814	0.10896448	1
0.61169083	0.59407453	1	nonDEG		-0.3729965	0.7596159	1
0.02003689	0.94158797	1	nonDEG		0.28931196	0.43807606	1
-0.3715609	0.21854771	0.79677948	nonDEG		-0.0271392	0.94945131	1
0.04266772	0.88729594	1	nonDEG		0.21739419	0.58569213	1
0.11305995	0.73903375	1	nonDEG		0.12307442	0.7713831	1
2.09884519	0.00566264	0.09675126	nonDEG		-0.4241361	0.57810943	1
0.30354421	0.24499567	0.8265359	nonDEG		-0.2084748	0.57388724	1
-0.0990182	0.75458752	1	nonDEG		0.46260724	0.24734831	1
0.18432652	0.53363043	1	nonDEG		-0.1415422	0.72060339	1
-0.7527482	0.41723468	0.96233775	nonDEG		-0.177524	0.88390293	1
0.35461791	0.15466933	0.69601198	nonDEG		-0.0519046	0.88803266	1
-0.1580179	0.51776649	1	nonDEG		-0.1405856	0.70238079	1
-0.1215927	0.79389069	1	nonDEG		-0.5609632	0.32341472	1
0.08069023	0.75420789	1	nonDEG		-0.1739581	0.63248186	1
-0.1186724	0.6315545	1	nonDEG		0.01332121	0.97108344	1
-0.2044476	0.79438737	1	nonDEG		-1.5069761	0.07244594	1
-0.3871973	0.17948429	0.74514618	nonDEG		0.05162228	0.89792041	1
-0.1697106	0.58211526	1	nonDEG		-0.0263065	0.95088316	1
0.01908936	0.94135482	1	nonDEG		-0.2474926	0.51415427	1
-0.4674075	0.74675404	1	nonDEG		-1.2920996	0.35910676	1
0.4499975	0.85333644	1	nonDEG		-1.1813368	0.56145948	1
-0.4081841	0.2316096	0.81081356	nonDEG		0.15234657	0.74474466	1
-0.3693329	0.23371757	0.81396091	nonDEG		-0.1500935	0.70008546	1
-0.3963998	0.14230705	0.67359585	nonDEG		0.09974581	0.78931325	1
0.8582031	0.15502946	0.69699333	nonDEG		-0.9558211	0.16395456	1
1.5589579	0.01974887	0.22747822	nonDEG		-0.8988694	0.26705223	1
0.93349177	0.34977896	0.91917773	nonDEG		1.16522434	0.24309493	1
0.22545574	0.41990465	0.96294915	nonDEG		0.23016898	0.55361182	1
-0.001291	0.99821143	1	nonDEG		-0.3366087	0.40590134	1

0.32709489	0.3221366	0.90516173	nonDEG	-0.0991948	0.81645399	1
-0.1421173	0.68784747	1	nonDEG	-0.1868557	0.67889772	1
-0.0243123	0.92089647	1	nonDEG	-0.0045448	0.99040431	1
-0.8549238	0.01432788	0.18539904	nonDEG	0.03666169	0.94421648	1
-1.4515741	0.01058972	0.15255616	nonDEG	-0.4156177	0.62694621	1
-0.3164721	0.91836779	1	nonDEG	-1.2296089	0.45417018	1
0.19188793	0.5031584	1	nonDEG	0.18139565	0.64460502	1
0.09769694	0.69079751	1	nonDEG	0.16952606	0.64873303	1
-0.586403	0.47821644	0.99305902	nonDEG	-0.3544361	0.72074697	1
0.09113814	0.89123724	1	nonDEG	-1.410024	0.07310025	1
-0.5035042	0.07235024	0.49087779	nonDEG	-0.0334659	0.93404963	1
-0.333981	0.19831071	0.77049875	nonDEG	-0.2103161	0.59174431	1
-0.0133312	0.97454456	1	nonDEG	-0.1463422	0.75979784	1
-0.117402	0.78379392	1	nonDEG	0.47925767	0.33489236	1
-1.1986487	0.02043901	0.23231812	nonDEG	0.70017166	0.26382292	1
-0.4978077	0.10704554	0.59914672	nonDEG	0.06322816	0.87976486	1
0.20939976	0.4854379	0.99670526	nonDEG	0.14191495	0.72617477	1
0.19144269	0.47492421	0.99125	nonDEG	-0.0291738	0.93783879	1
0.49969342	0.47201802	0.99002994	nonDEG	0.85320414	0.40652516	1
-0.0123965	0.96282339	1	nonDEG	0.03305024	0.92881335	1
-1.5231047	0.47069131	0.9890565	nonDEG	-0.0163273	1	1
-0.0131593	0.95754857	1	nonDEG	0.1488236	0.68073474	1
-0.0459907	0.85040019	1	nonDEG	-0.0986678	0.78484155	1
-0.1095916	0.78686845	1	nonDEG	-0.449415	0.37248832	1
-0.9113479	0.00586642	0.09936795	nonDEG	0.21940972	0.60867665	1
-0.1486438	0.96812392	1	nonDEG	-0.1610725	0.94901635	1
0.22056697	0.69748045	1	nonDEG	0.17309341	0.82398885	1
-0.3276124	0.34992646	0.91917773	nonDEG	0.25181335	0.56100992	1
-2.1829552	0.06027715	0.44787964	nonDEG	2.23838096	0.05961532	1
0.30153808	0.53470034	1	nonDEG	-0.4404053	0.44618863	1
0.15008009	0.80003531	1	nonDEG	-0.4924293	0.45938795	1
0.30897905	0.33190457	0.91573933	nonDEG	0.21160524	0.58528304	1
-0.2851406	0.47204156	0.99002994	nonDEG	-0.3290672	0.53519141	1
0.00562805	0.9833781	1	nonDEG	-0.0880906	0.81296362	1
-0.4792037	0.05166251	0.41137146	nonDEG	0.82589827	0.0604713	1
-0.0814662	0.78361106	1	nonDEG	-0.0154807	0.97013695	1
0.31252604	0.19644122	0.76773594	nonDEG	0.13747282	0.70233842	1
0.01688909	0.94558548	1	nonDEG	0.19471908	0.63054974	1
-0.8148737	0.10089799	0.57894377	nonDEG	0.38987326	0.58915349	1
-0.1473998	1	1	nonDEG	-1.6398038	0.42555217	1
-0.5951704	0.71429138	1	nonDEG	-0.7779623	0.79776986	1
0.26962945	0.33347063	0.91729884	nonDEG	-0.1674483	0.65278814	1
0.62487915	0.19348507	0.76338677	nonDEG	0.13035157	0.81420011	1

0.58095753	0.14858111	0.68531592	nonDEG	-0.0470695	0.92305648	1
-0.3484419	0.26369423	0.847847	nonDEG	-0.364198	0.40324542	1
0.11744948	0.87078516	1	nonDEG	1.22085526	0.15276226	1
0.06305797	0.79905353	1	nonDEG	-0.0621881	0.86832355	1
-0.2412207	0.33832224	0.91869633	nonDEG	0.13354387	0.71287791	1
0.66441076	0.18449481	0.75232257	nonDEG	-0.1632409	0.78017101	1
-0.1032373	0.77190585	1	nonDEG	-0.12113	0.79604603	1
-0.1390721	0.56625639	1	nonDEG	0.11504652	0.7749049	1
-0.0754952	0.76527499	1	nonDEG	0.11966665	0.74174189	1
0.23651223	0.4746255	0.99095745	nonDEG	0.30858551	0.44512437	1
-0.1937656	0.43533004	0.97358028	nonDEG	0.01767773	0.96275056	1
-0.2476401	0.52671621	1	nonDEG	0.32283021	0.50199563	1
-0.1541555	0.60594497	1	nonDEG	0.58119175	0.13181663	1
-0.126396	0.63341507	1	nonDEG	0.07729664	0.84316067	1
0.36755625	0.50497373	1	nonDEG	-0.9616142	0.14314303	1
-0.0550654	0.82752118	1	nonDEG	-0.0447465	0.90213988	1
-0.2577725	0.31859864	0.90147301	nonDEG	-0.2466928	0.50616083	1
-0.2573015	0.45104001	0.98329655	nonDEG	0.69253865	0.11377419	1
0.38614103	0.35184216	0.91917773	nonDEG	-0.8636873	0.08852178	1
-0.0720064	0.77834668	1	nonDEG	0.39413898	0.27451116	1
0.34277873	0.31817419	0.90098849	nonDEG	-0.7073285	0.10280708	1
-0.1403922	0.7186452	1	nonDEG	-0.3075693	0.53977374	1
-0.3127547	0.48844523	0.99826154	nonDEG	0.75745738	0.1765934	1
0.07762553	0.79761054	1	nonDEG	-0.0671369	0.86500384	1
-0.9236195	0.5853378	1	nonDEG	1.26391718	0.45688709	1
-0.2948785	0.32608838	0.9105298	nonDEG	0.16847265	0.67088306	1
0.84392372	0.00635187	0.10520171	nonDEG	0.08176785	0.82791327	1
0.02293041	0.95645653	1	nonDEG	0.49244341	0.30920242	1
1.32494778	0.07400835	0.49754092	nonDEG	0.22091287	0.73135842	1
-0.4120399	0.20444191	0.77834447	nonDEG	-0.4435342	0.29546104	1
0.04190981	0.88491318	1	nonDEG	-0.2481924	0.52361932	1
-0.2653737	0.30074844	0.88542723	nonDEG	-0.0945002	0.79314598	1
-0.1150386	0.71677329	1	nonDEG	0.06971354	0.86352485	1
0.20464562	0.47755244	0.99294124	nonDEG	0.2243819	0.58490261	1
0.23595807	0.35856534	0.92154288	nonDEG	0.08325364	0.84297743	1
-0.8106136	0.01230318	0.1681125	nonDEG	-0.2277058	0.6107723	1
-0.0756401	0.83501079	1	nonDEG	-0.0814198	0.86222679	1
-0.1018775	0.73335044	1	nonDEG	0.31398865	0.42822143	1
0.83175622	0.00457031	0.08341316	nonDEG	0.11835151	0.75363365	1
-0.8394462	0.45725015	0.98426953	nonDEG	1.9782592	0.02324023	1
-0.2521848	0.41161707	0.95872812	nonDEG	-0.2469021	0.55434718	1
-0.1433637	0.55982326	1	nonDEG	0.36725654	0.31312754	1
-0.2838029	0.24883216	0.82990493	nonDEG	0.22024774	0.56121845	1

-0.0798099	0.81006731	1	nonDEG	0.33871339	0.40590318	1
0.12923903	0.60252841	1	nonDEG	0.18293021	0.62283438	1
-0.225848	0.43927826	0.97619691	nonDEG	0.02129998	0.95726753	1
0.18641363	1	1	nonDEG	-0.2064198	1	1
0.1278109	0.62823311	1	nonDEG	-0.291204	0.42806989	1
0.0422922	0.86721561	1	nonDEG	0.40913646	0.26008221	1
0.11782947	0.64237346	1	nonDEG	-0.0834841	0.82326721	1
0.38904819	0.16214713	0.71301481	nonDEG	0.0469418	0.89972759	1
0.31546273	0.24004863	0.82187354	nonDEG	0.45062865	0.24146645	1
0.4399474	0.34063869	0.91869633	nonDEG	-0.2934619	0.59542263	1
-0.2414013	0.37089651	0.92943023	nonDEG	0.02081693	0.95816614	1
1.96018242	0.00935583	0.13903391	nonDEG	1.26684476	0.10026319	1
0.05937568	0.80772811	1	nonDEG	-0.0172452	0.96288178	1
0.12327132	0.62920608	1	nonDEG	0.22153363	0.54747043	1
0.00474313	0.9848908	1	nonDEG	-0.0562903	0.8758024	1
-0.3003266	0.34459163	0.91869633	nonDEG	-1.3399247	0.00240331	0.38006158
-2.0448307	0.13650461	0.66368058	nonDEG	1.84814373	0.17725404	1
0.23796883	0.44519151	0.98025377	nonDEG	-0.0775372	0.87089664	1
-2.1031544	0.00286929	0.05973755	nonDEG	1.16255741	0.23603735	1
0.02635129	0.96512006	1	nonDEG	0.81452618	0.15721664	1
-0.8857334	0.54229449	1	nonDEG	-5.0038913	0.06976147	1
-0.3875835	0.18016347	0.74523429	nonDEG	-0.0890413	0.81583112	1
0.19600235	0.44934815	0.98329655	nonDEG	0.05711402	0.88886884	1
0.14336882	0.58165894	1	nonDEG	0.28636262	0.44421996	1
-0.0290941	0.9063339	1	nonDEG	0.05245848	0.88564807	1
1.10252842	1	1	nonDEG	-0.8471802	1	1
0.31715577	0.20219438	0.77400024	nonDEG	0.03656104	0.93195237	1
0.10035457	0.70583319	1	nonDEG	0.01208539	0.97535134	1
0.00492902	0.98420593	1	nonDEG	0.04300141	0.9060053	1
-0.9559859	0.31444968	0.89780061	nonDEG	-2.5302244	0.06221987	1
0.56923665	0.45700959	0.98426953	nonDEG	0.55793894	0.47975454	1
-0.1591979	0.57292763	1	nonDEG	-0.5652962	0.17152651	1
0.14564444	0.55534815	1	nonDEG	0.366331	0.32112656	1
-0.0907618	0.72297975	1	nonDEG	-0.2903609	0.4239811	1
-0.2356824	0.49146087	0.99967567	nonDEG	-0.5010531	0.25837854	1
-0.8407857	0.00436885	0.08135018	nonDEG	-0.3505442	0.38226167	1
-0.2596897	0.55147726	1	nonDEG	0.2196489	0.67589506	1
0.18687263	0.46596767	0.98613495	nonDEG	0.29942892	0.44907661	1
-0.5264374	0.03941704	0.35123659	nonDEG	-0.0971275	0.79056427	1
0.16781118	0.51637986	1	nonDEG	-0.0351826	0.92407305	1
0.2339266	0.36655969	0.92627904	nonDEG	0.37217121	0.31785275	1
0.5433362	0.06609859	0.46990497	nonDEG	-0.4117254	0.32175684	1
0.73296887	0.1190856	0.63063362	nonDEG	-0.3382137	0.54712711	1

0.28660399	0.2776024	0.8596126	nonDEG	0.27057781	0.45684997	1
-0.1082033	0.6529634	1	nonDEG	-0.0149515	0.97164338	1
1.59998835	0.1818017	0.74759887	nonDEG	-0.36782	0.86146046	1
-0.7668204	0.06559267	0.46791166	nonDEG	-0.011634	0.98488736	1
-0.7469761	0.22753478	0.80922828	nonDEG	-1.1400051	0.14637716	1
0.82017185	0.07696953	0.50793676	nonDEG	-0.0544626	0.91692973	1
-1.4140505	0.30357287	0.88818144	nonDEG	-0.7475058	0.6515742	1
0.82413586	0.01661398	0.20269336	nonDEG	-1.0933685	0.00707581	0.66803023
-0.1640193	0.62878424	1	nonDEG	-0.0410062	0.92753264	1
1.38602171	0.19921923	0.77158465	nonDEG	-0.6077863	0.62026744	1
0.15534825	0.52804592	1	nonDEG	-0.1686869	0.65821391	1
1.34304385	0.11807024	0.62825856	nonDEG	-0.8506417	0.29814862	1
-0.2343515	0.33737614	0.91869633	nonDEG	0.11280049	0.75463357	1
-2.4330352	0.01206391	0.16626845	nonDEG	1.80745297	0.02747827	1
0.20039158	0.46537689	0.98613495	nonDEG	0.12568127	0.73450922	1
-0.4544309	0.24795545	0.8295898	nonDEG	-0.009563	0.99166016	1
0.24779346	0.33378994	0.91729884	nonDEG	0.27735987	0.44518527	1
0.05662944	0.8954634	1	nonDEG	-0.060986	0.90747005	1
0.43876611	0.20772916	0.78320256	nonDEG	-1.4308269	0.00592479	0.60336628
0.17374498	0.48951657	0.99867487	nonDEG	0.15819958	0.67497728	1
-0.3529727	0.16957965	0.72663669	nonDEG	0.13900597	0.70329171	1
0.21381513	0.47233358	0.99012003	nonDEG	-0.0817458	0.83479217	1
-0.9449234	0.01226715	0.16791309	nonDEG	0.69007512	0.13585539	1
-0.4641782	0.25416408	0.8358301	nonDEG	-0.4771948	0.35609279	1
-0.1040971	0.69351575	1	nonDEG	-0.0980625	0.79408997	1
-0.1510106	0.61490235	1	nonDEG	0.31680499	0.41945854	1
0.75192054	0.01831886	0.21640605	nonDEG	0.19949508	0.66238028	1
0.07610815	0.82069549	1	nonDEG	0.19311779	0.6581566	1
0.03493889	0.91879647	1	nonDEG	0.7976706	0.03961428	1
-0.9124748	0.25582851	0.83793955	nonDEG	-0.5346136	0.58003058	1
-0.0305237	0.90774847	1	nonDEG	0.19692286	0.60418212	1
0.40206166	0.09830546	0.57137375	nonDEG	-0.3953119	0.29296174	1
-0.5010434	0.07916208	0.51535882	nonDEG	0.91895594	0.01697718	0.93945691
0.04880298	0.9407522	1	nonDEG	-0.5010192	0.44094435	1
-0.2911896	0.2720755	0.85514137	nonDEG	-0.1612766	0.67115068	1
-0.6586388	0.04146429	0.36189359	nonDEG	0.44952951	0.34072657	1
-0.018892	0.96372546	1	nonDEG	0.0934758	0.83918773	1
-0.5016506	0.18092373	0.74649105	nonDEG	0.26623589	0.5765939	1
-0.2136288	0.56555905	1	nonDEG	-0.064305	0.89904335	1
0.46476051	0.30213393	0.88691415	nonDEG	0.09333873	0.84827391	1
0.37911613	0.40771377	0.9572283	nonDEG	0.41284814	0.49200311	1
0.16252714	0.80173218	1	nonDEG	1.39186031	0.05116655	1
-0.0010438	0.99794843	1	nonDEG	0.68266704	0.07530269	1

-0.199071	0.60684828	1	nonDEG	0.04097891	0.9335751	1
0.43096474	0.08181689	0.52341091	nonDEG	-0.0601075	0.87994384	1
-0.4305483	0.19039222	0.75718453	nonDEG	0.17398241	0.69153929	1
0.3792131	0.6200206	1	nonDEG	-1.7662082	0.04883927	1
-0.0292165	0.90671803	1	nonDEG	0.13418186	0.71380974	1
-0.2668695	0.47515315	0.99128578	nonDEG	-0.0458696	0.92965477	1
-1.1216045	0.10561442	0.59483127	nonDEG	0.30968436	0.71898721	1
0.30900555	0.21202192	0.78921843	nonDEG	0.19192411	0.59331492	1
-0.1782351	0.521414	1	nonDEG	0.27311002	0.4901836	1
-0.3548055	0.57220897	1	nonDEG	0.4337784	0.50868456	1
-1.7749186	0.29746839	0.88261266	nonDEG	-1.347119	0.73442621	1
0.65592632	0.01444051	0.1856454	nonDEG	0.1516247	0.68758163	1
0.29995055	0.23071897	0.80924485	nonDEG	0.09516198	0.80203348	1
-0.1399801	0.56106075	1	nonDEG	0.14590383	0.68433195	1
0.31457794	0.36370562	0.92430334	nonDEG	-0.2669111	0.53922063	1
0.6840208	0.04843974	0.39666692	nonDEG	0.26999359	0.53210533	1
-0.6475511	0.00730092	0.11651775	nonDEG	-0.2033597	0.590121	1
-0.5480403	0.53519683	1	nonDEG	-2.0628313	0.08219457	1
0.04250556	0.89605792	1	nonDEG	-0.0372414	0.92908258	1
0.24302172	0.55994406	1	nonDEG	-0.4935323	0.40887104	1
-0.106278	0.74264341	1	nonDEG	0.39103021	0.34024749	1
-0.0482973	0.87556184	1	nonDEG	0.06567665	0.8712104	1
-0.112378	0.95935173	1	nonDEG	-1.4602294	0.34237215	1
0.49210667	0.06774551	0.47541505	nonDEG	0.01569941	0.96757371	1
-0.3172806	0.19635209	0.76773594	nonDEG	0.21863169	0.5455508	1
0.11416543	0.65154319	1	nonDEG	-0.1965564	0.61316672	1
0.67977781	0.02500673	0.26557474	nonDEG	0.15579513	0.6895034	1
0.695495	0.11933049	0.63063362	nonDEG	-1.4462271	0.0094956	0.74892342
0.10415153	0.68699492	1	nonDEG	0.15465042	0.67192152	1
-0.0055104	0.9819887	1	nonDEG	0.13684727	0.70298903	1
0.13932943	0.56764014	1	nonDEG	0.17500944	0.63014288	1
-1.0544156	0.05407701	0.42113858	nonDEG	0.36119784	0.58232949	1
0.18280874	0.47137859	0.98955292	nonDEG	0.02113544	0.95436228	1
0.56825272	0.09856497	0.57188242	nonDEG	0.20382852	0.64419474	1
-0.3611172	0.15201335	0.6902855	nonDEG	-0.2669727	0.47493102	1
-0.3097997	0.52184531	1	nonDEG	-0.2606117	0.63628784	1
0.24975993	0.33542287	0.91869633	nonDEG	0.08303456	0.84901322	1
-0.6568097	0.085093	0.53469642	nonDEG	0.02898845	0.95956468	1
0.41490774	0.126344	0.64619186	nonDEG	0.06304043	0.88174279	1
0.46498097	0.07734056	0.50992805	nonDEG	0.090074	0.80856126	1
0.31520516	0.23126556	0.81040671	nonDEG	0.15625528	0.70035956	1
-0.324285	0.25126246	0.83326749	nonDEG	-0.0130415	0.97590696	1
0.16843961	0.49178881	0.99981697	nonDEG	0.47389269	0.31415861	1

0.38536846	0.66160897	1	nonDEG	-1.2076843	0.25043911	1
-0.5468727	0.04293923	0.36894687	nonDEG	0.1266794	0.74109418	1
-0.2444959	0.48361602	0.99599492	nonDEG	-0.5167689	0.25940313	1
-0.1845048	0.4945206	1	nonDEG	0.2632554	0.4767254	1
-0.1720365	0.51266249	1	nonDEG	-0.2697334	0.47324741	1
0.01528224	0.95058833	1	nonDEG	-0.1638379	0.65094757	1
0.1423036	0.62002175	1	nonDEG	0.12573632	0.74072478	1
0.48842263	0.16294127	0.714154	nonDEG	0.23311451	0.57495501	1
0.29272611	0.2809662	0.86186717	nonDEG	0.2515535	0.53442544	1
-0.329513	0.19363618	0.76362433	nonDEG	0.14046555	0.7021782	1
-0.3757089	0.11834231	0.62843136	nonDEG	0.30724342	0.40421855	1
1.34436017	0.00544227	0.09418874	nonDEG	0.27579423	0.57546841	1
-0.0821722	0.74814603	1	nonDEG	0.12302373	0.76182662	1
-0.0765103	0.84120826	1	nonDEG	0.07586778	0.87399356	1
-0.3037473	0.21635329	0.79477343	nonDEG	-0.4019051	0.27438058	1
-0.4104155	0.54826319	1	nonDEG	-1.0409692	0.19850525	1
-0.5770851	0.03223737	0.31200237	nonDEG	-0.2969878	0.55319896	1
-0.1461146	0.69157711	1	nonDEG	-0.8377602	0.07819655	1
-0.030244	0.90493003	1	nonDEG	-0.2177859	0.55482239	1
-0.3805639	0.21714019	0.79497451	nonDEG	-0.1668002	0.68730034	1
0.00689659	0.97947984	1	nonDEG	-0.4356963	0.24573521	1
0.17786129	0.80683998	1	nonDEG	1.1217067	0.17193987	1
0.05691298	0.82491799	1	nonDEG	-0.2282629	0.55259663	1
0.13145519	1	1	nonDEG	0.59440576	0.59159176	1
-0.2957275	0.2755543	0.85835498	nonDEG	0.16045895	0.68366659	1
-0.0115912	0.9667588	1	nonDEG	-0.112507	0.79909047	1
0.22243576	0.45376478	0.98383025	nonDEG	0.34236158	0.38443928	1
0.51518135	0.31872596	0.90147301	nonDEG	0.12339425	0.84815344	1
-0.3075204	0.23689918	0.81880142	nonDEG	0.15417566	0.71769356	1
0.35939808	0.2522875	0.83371668	nonDEG	-0.3765391	0.38021784	1
0.27944768	0.27274954	0.85574694	nonDEG	0.26606545	0.46359222	1
0.12987945	0.75718645	1	nonDEG	-0.0971605	0.85042983	1
0.23374794	0.43693587	0.97402891	nonDEG	-0.2475841	0.618292	1
-0.1382396	0.71549795	1	nonDEG	0.0514077	0.91822652	1
0.11986783	0.75137226	1	nonDEG	0.1672888	0.71055839	1
-0.1134312	0.64363442	1	nonDEG	-0.0025369	0.99495436	1
0.0747625	0.80362394	1	nonDEG	-0.0543226	0.89112574	1
0.11595895	0.90770069	1	nonDEG	0.08941889	0.934769	1
-1.005479	0.13501014	0.6610751	nonDEG	0.20458189	0.80202157	1
0.10370095	0.73104168	1	nonDEG	0.37049694	0.35004593	1
-0.4651813	0.33762213	0.91869633	nonDEG	0.89892292	0.10624331	1
0.49532598	0.07220629	0.49026324	nonDEG	0.04345286	0.90984638	1
0.38910725	0.1197024	0.63063362	nonDEG	0.18394454	0.61579203	1

-0.0324913	0.89504729	1	nonDEG	-0.4060585	0.26620426	1
-0.2637473	0.41154983	0.95872812	nonDEG	0.45625504	0.26339042	1
2.67696414	0.13776774	0.66503679	nonDEG	-2.3333117	0.2028765	1
-0.2777393	0.36984449	0.92861845	nonDEG	0.4140882	0.31608886	1
-0.2261482	0.43569824	0.97391956	nonDEG	-0.0707102	0.85751475	1
-0.16068	0.67187754	1	nonDEG	0.09842557	0.83242099	1
-0.1341515	0.57872195	1	nonDEG	0.05301265	0.88166833	1
0.25409312	0.30404728	0.88905908	nonDEG	0.15159492	0.67547786	1
0.4266779	0.15125078	0.68852201	nonDEG	-0.3374156	0.38466481	1
0.33615638	0.19468588	0.7646157	nonDEG	0.25050203	0.50129262	1
-0.6497204	0.3935433	0.94783987	nonDEG	-0.0230062	1	1
-0.0374463	0.88406293	1	nonDEG	-0.0772259	0.83736711	1
-0.2699415	0.78782709	1	nonDEG	0.6213067	0.54769841	1
-0.0987991	0.82210593	1	nonDEG	0.12960174	0.80994146	1
0.03699595	0.91634259	1	nonDEG	0.11235403	0.79378082	1
0.15066091	0.62244696	1	nonDEG	-0.583638	0.14954388	1
0.2793995	0.27135293	0.85427653	nonDEG	-0.1056777	0.77510618	1
-0.5590346	0.02264566	0.2489707	nonDEG	-0.0084504	0.98362124	1
0.20342877	0.44384311	0.97927727	nonDEG	0.19653004	0.59047094	1
0.29698139	0.31207309	0.89535767	nonDEG	0.10340033	0.79342068	1
-0.2445983	0.38116648	0.93728367	nonDEG	0.5915663	0.11253057	1
0.23856821	0.33622284	0.91869633	nonDEG	0.3545241	0.33889456	1
0.83444674	0.0869668	0.53971705	nonDEG	0.64703299	0.1869912	1
-0.6033239	0.14712872	0.68306613	nonDEG	-0.3577828	0.4856824	1
-2.5597528	0.20928649	0.78572136	nonDEG	0.83804782	1	1
0.11383881	0.74897418	1	nonDEG	-0.2160015	0.63345278	1
0.12562133	0.61800363	1	nonDEG	-0.1161876	0.75263353	1
0.47832413	0.0660657	0.46978455	nonDEG	0.12515475	0.73590687	1
0.51476977	0.10517479	0.59326319	nonDEG	0.16467697	0.68075618	1
0.34324336	0.22476433	0.80603814	nonDEG	0.27070909	0.49423828	1
-0.3298524	0.20664659	0.78183639	nonDEG	-0.1117737	0.77884487	1
-0.3979342	0.20133798	0.77336631	nonDEG	-0.4713154	0.26149676	1
-0.0569837	0.87591821	1	nonDEG	-0.2983958	0.50306771	1
0.13203632	0.75307262	1	nonDEG	-0.8770693	0.106019	1
0.11450432	0.63763354	1	nonDEG	0.08508124	0.81260182	1
-0.4637294	0.07035826	0.48541759	nonDEG	0.06288846	0.87183171	1
0.17063105	0.50274515	1	nonDEG	-0.1029785	0.77833368	1
0.04381906	0.88688145	1	nonDEG	0.16960155	0.66470605	1
0.20554652	0.42941944	0.97101957	nonDEG	-0.0473825	0.90109364	1
0.15919438	0.65725278	1	nonDEG	0.25478833	0.58926262	1
0.40289441	0.24541927	0.82672898	nonDEG	0.11804393	0.77986089	1
0.27971687	0.27578702	0.85842562	nonDEG	0.35562077	0.3258383	1
0.5531219	0.059869	0.44628162	nonDEG	-0.1313236	0.73944472	1

0.10316941	0.69480645	1	nonDEG	0.28128017	0.44990279	1
0.3166397	0.24096676	0.82268787	nonDEG	0.11269551	0.75716979	1
-0.4292403	0.09569366	0.56466739	nonDEG	0.10466697	0.77761548	1
0.16813524	0.572991	1	nonDEG	-0.0754881	0.8469724	1
1.1316611	0.48007196	0.99357121	nonDEG	-0.6264629	0.7165879	1
0.28842572	0.39206605	0.946931	nonDEG	-0.9342946	0.02411369	1
0.97445017	0.14085314	0.67020778	nonDEG	0.00704543	1	1
0.31332739	0.21745278	0.79539695	nonDEG	0.30872708	0.39154253	1
0.41252349	0.09201213	0.55257985	nonDEG	0.09771622	0.80873999	1
0.01803465	0.9546504	1	nonDEG	0.04083899	0.91916005	1
0.21153944	0.50603549	1	nonDEG	0.48435061	0.21989772	1
0.8018684	0.00332089	0.06697784	nonDEG	-0.3495289	0.33736457	1
-0.7447848	0.58047741	1	nonDEG	1.82302285	0.08249342	1
-0.2607241	0.42580091	0.96729323	nonDEG	-0.3626579	0.40892478	1
0.09832355	0.75441324	1	nonDEG	-0.2879402	0.47735567	1
-0.6437784	0.04234427	0.36576454	nonDEG	-0.4075374	0.36586947	1
-0.4617089	0.67540726	1	nonDEG	-0.5123271	0.72046479	1
-0.2048179	0.52651655	1	nonDEG	-0.1322695	0.75796736	1
0.37477908	0.13775015	0.66503679	nonDEG	0.23971132	0.51475383	1
0.14829543	0.61443861	1	nonDEG	-0.197376	0.61243205	1
0.55820388	0.60501446	1	nonDEG	-0.6949886	0.58159545	1
-0.0243776	0.93197945	1	nonDEG	-0.1394251	0.71529736	1
-0.0592286	0.82382015	1	nonDEG	0.08379643	0.82199016	1
-0.2159944	0.39399095	0.94821576	nonDEG	-0.1890758	0.60175895	1
0.16533324	0.52356376	1	nonDEG	-0.3615073	0.32444071	1
-0.1355053	0.74612075	1	nonDEG	-0.5129306	0.33589962	1
-0.9313403	0.13686569	0.66368058	nonDEG	0.40598596	0.51574187	1
0.16384228	0.52196829	1	nonDEG	-0.1185103	0.74623764	1
0.20617705	0.42759633	0.9694269	nonDEG	0.13599658	0.71228495	1
-0.3712677	0.75705993	1	nonDEG	2.04320864	0.04108049	1
0.14780898	0.64609102	1	nonDEG	-0.1745119	0.67323734	1
0.07619499	0.80525538	1	nonDEG	-0.2231467	0.6165457	1
-0.4070976	0.43715131	0.97420873	nonDEG	0.23721963	0.72866369	1
0.17275612	0.50872433	1	nonDEG	0.45298089	0.26579952	1
0.11467438	0.64880352	1	nonDEG	0.13128525	0.71912747	1
0.42209412	0.08751104	0.54054722	nonDEG	0.03532297	0.92240045	1
-0.1393567	0.64426979	1	nonDEG	-0.2902538	0.46627131	1
0.51310275	0.61266956	1	nonDEG	0.2828398	0.80859992	1
-0.0441813	0.88299404	1	nonDEG	0.03138974	0.93158294	1
0.04791824	0.84327441	1	nonDEG	0.21708689	0.57279361	1
-0.2253083	0.50091607	1	nonDEG	-0.0023985	0.99815498	1
0.06518377	0.81137216	1	nonDEG	0.40239314	0.30209057	1
0.04525411	0.88194288	1	nonDEG	-0.8959111	0.03019086	1

0.40357541	0.46054321	0.98479636	nonDEG	-0.6336649	0.34492009	1
2.23527319	0.06333905	0.46113624	nonDEG	-3.514497	0.02170169	1
0.20413517	0.44827119	0.98329655	nonDEG	-0.2048009	0.57535025	1
0.13624504	0.67971571	1	nonDEG	0.17078714	0.67816852	1
0.01821791	0.95890512	1	nonDEG	0.04954157	0.9128937	1
0.25347518	0.31863788	0.90147301	nonDEG	-0.2951223	0.41884042	1
-0.8105167	0.20424737	0.7780588	nonDEG	-1.0877992	0.18153898	1
-0.0642065	0.86294588	1	nonDEG	0.34143697	0.51707739	1
-0.8076316	0.00922145	0.13766588	nonDEG	0.07931168	0.8326179	1
0.38641076	0.19410216	0.76383173	nonDEG	0.13763594	0.71784798	1
2.52964271	0.05318465	0.41796662	nonDEG	-2.4061326	0.119621	1
0.02298641	0.93755874	1	nonDEG	0.00337125	0.99422656	1
-0.3620461	0.30018849	0.88467981	nonDEG	0.56211068	0.18987664	1
0.1104225	0.67872462	1	nonDEG	0.05528776	0.88187102	1
0.65899768	0.01230621	0.1681125	nonDEG	-0.2525909	0.5013209	1
0.41197806	0.26374465	0.847847	nonDEG	-0.1731385	0.70494171	1
0.37194819	0.70886083	1	nonDEG	0.94894023	0.52185067	1
0.37824134	0.13989498	0.66813245	nonDEG	0.35286989	0.33886972	1
0.97093848	0.33893843	0.91869633	nonDEG	-2.2150533	0.05220877	1
0.05825249	0.83472049	1	nonDEG	-0.0103835	0.97896995	1
0.31797752	0.33069649	0.91437719	nonDEG	-0.2966702	0.49599958	1
-0.3304961	0.24553955	0.82672898	nonDEG	-0.1990649	0.60368848	1
0.22084863	0.43126198	0.97178962	nonDEG	0.54817814	0.1400208	1
0.0999201	0.73254561	1	nonDEG	-0.3110591	0.45016157	1
-0.1645575	0.52969024	1	nonDEG	-0.1075241	0.77816274	1
0.36790536	0.1859744	0.75310345	nonDEG	-0.9181479	0.03191517	1
-1.5173521	0.20763851	0.78320256	nonDEG	-3.507268	0.1026333	1
-0.3167614	0.28193526	0.8632233	nonDEG	-0.3721154	0.35311611	1
-1.5766528	0.0806843	0.52017157	nonDEG	0.33290157	0.77480146	1
-0.1521011	0.53969564	1	nonDEG	0.06489207	0.86417627	1
-0.1415338	0.67330353	1	nonDEG	0.42616309	0.31084694	1
-0.2019804	0.42387866	0.96620031	nonDEG	0.06306655	0.86404561	1
0.4587721	0.06352469	0.46171022	nonDEG	0.09869574	0.78587937	1
0.48920662	0.08884174	0.54531086	nonDEG	0.20576456	0.59230679	1
0.10292134	0.70782529	1	nonDEG	-0.0332727	0.92839818	1
0.53475333	0.05167471	0.41137146	nonDEG	0.10565258	0.77752187	1
0.62943996	0.08332766	0.52831277	nonDEG	-0.5327119	0.26498612	1
-0.2689977	0.57684269	1	nonDEG	-0.2105852	0.70570847	1
0.08047688	0.77364044	1	nonDEG	0.3081417	0.40559676	1
-0.3289786	0.17980802	0.74514618	nonDEG	-0.1028982	0.79611833	1
0.10754178	0.6848517	1	nonDEG	-0.0365962	0.92070112	1
0.14288776	0.79018958	1	nonDEG	-0.5775112	0.36691846	1
-0.2314777	0.40391828	0.95387055	nonDEG	-0.1474343	0.70020941	1

0.24166327	0.3647205	0.92544853	nonDEG	-0.3803318	0.30139891	1
0.09047129	0.84113234	1	nonDEG	-1.5594517	0.00751308	0.67511746
0.41913822	0.21114869	0.7877265	nonDEG	-0.8414342	0.05087841	1
0.29327707	0.90789834	1	nonDEG	-2.6726528	0.18270265	1
-0.4556996	0.61192755	1	nonDEG	-2.9058472	0.01536085	0.89532248
0.60307167	0.03715338	0.34008606	nonDEG	-0.0412812	0.9138624	1
0.27002673	0.51787935	1	nonDEG	0.18528365	0.71061529	1
0.04595766	0.85134138	1	nonDEG	0.16438479	0.65920261	1
-0.2204794	0.48462327	0.99628662	nonDEG	0.21767393	0.59428515	1
-0.2645031	0.50202764	1	nonDEG	0.75048708	0.12605138	1
-0.3291566	0.17543292	0.73906264	nonDEG	0.27847158	0.50631753	1
-0.3301612	0.63190319	1	nonDEG	-0.6219189	0.44811234	1
-0.2759199	0.62224179	1	nonDEG	-0.4345607	0.5146245	1
-0.7247981	0.29235047	0.8754342	nonDEG	-0.2534687	0.76738025	1
-0.1925141	0.46758397	0.986734	nonDEG	-0.3139222	0.39238649	1
-0.1272127	0.62539833	1	nonDEG	0.40474575	0.35840056	1
-0.1319011	0.58964619	1	nonDEG	0.06481566	0.85746948	1
-0.0168966	0.94824668	1	nonDEG	0.10582779	0.78292949	1
0.5201252	0.04632564	0.38704295	nonDEG	0.14600727	0.75239249	1
-0.4577601	0.18338922	0.75072226	nonDEG	0.50831582	0.23555219	1
-0.1846219	0.55071542	1	nonDEG	0.01912417	0.96054063	1
0.19094862	0.50367627	1	nonDEG	-0.0096885	0.98368511	1
-1.3855191	0.13483815	0.660956	nonDEG	-0.4106148	0.74374532	1
0.35729009	0.1418995	0.67279424	nonDEG	-0.0244808	0.94952207	1
0.17374884	0.54320333	1	nonDEG	0.086339	0.82691324	1
0.08697336	1	1	nonDEG	0.03389178	1	1
-0.1725691	0.58597001	1	nonDEG	0.45376353	0.30543394	1
0.55783029	0.0469955	0.3901947	nonDEG	0.29556847	0.46019997	1
0.57976583	0.1118803	0.61140603	nonDEG	-0.2834961	0.52841207	1
0.70998888	0.04484432	0.379033	nonDEG	0.18878964	0.7375469	1
0.19462308	0.44259884	0.97891316	nonDEG	0.22808427	0.54049447	1
0.2320503	0.39250836	0.94717646	nonDEG	0.1566417	0.68391984	1
2.18017651	0.41727352	0.96233775	nonDEG	-5.0477493	0.16377014	1
0.25007167	0.44031168	0.97696228	nonDEG	0.58631278	0.16569765	1
0.21932829	0.58810006	1	nonDEG	0.08370462	0.8677284	1
0.07708171	0.85005274	1	nonDEG	0.00738367	0.99219013	1
0.75757011	0.04706915	0.39043916	nonDEG	-0.7261494	0.13424287	1
-0.0194031	0.93835482	1	nonDEG	0.17953508	0.62893647	1
0.08062128	0.77496044	1	nonDEG	-0.2097023	0.58512157	1
2.39411304	1	1	nonDEG	1.77720115	0.61411792	1
-0.5686329	0.22405552	0.8047792	nonDEG	-0.3770855	0.52505958	1
-0.2120847	0.38724753	0.94412832	nonDEG	0.22997753	0.5397268	1
0.18408818	0.53013153	1	nonDEG	-0.3539866	0.36683749	1

0.05659194	0.8225905	1	nonDEG	0.51060189	0.21519778	1
0.15917672	0.54143922	1	nonDEG	0.02715954	0.94318062	1
0.21771975	0.58678801	1	nonDEG	0.04280455	0.93361653	1
0.02749266	0.94148681	1	nonDEG	-0.0495524	0.91361755	1
0.43020716	0.08486342	0.53370952	nonDEG	0.23041392	0.57946734	1
-2.8423441	0.01059223	0.15255616	nonDEG	0.82517511	0.7111102	1
-0.1524133	0.56575849	1	nonDEG	0.00091801	0.9991225	1
2.08761947	0.16986279	0.72725288	nonDEG	-0.7711636	0.54473984	1
-0.1657625	0.51586229	1	nonDEG	-0.1243086	0.74899499	1
-0.0897641	0.77826469	1	nonDEG	0.07194412	0.8617794	1
3.24350454	0.63649381	1	nonDEG	-3.1016412	0.64981609	1
0.45676088	0.09997065	0.57533122	nonDEG	0.31048191	0.42121069	1
0.30951874	0.21093406	0.7877265	nonDEG	-0.1909558	0.60478663	1
0.58547567	0.0441862	0.3753022	nonDEG	-0.3029469	0.48167649	1
-0.688498	0.06871451	0.47924345	nonDEG	-0.9416309	0.08488406	1
0.01079944	0.96502837	1	nonDEG	0.26414431	0.46340705	1
0.51669502	0.12923228	0.65245846	nonDEG	0.13961273	0.7365573	1
0.18279941	0.47159835	0.98975108	nonDEG	-0.1446162	0.74219712	1
-0.3404507	0.55670519	1	nonDEG	-0.4775796	0.49223575	1
-5.2768712	0.0505265	0.40607413	nonDEG	3.83376511	0.46342812	1
0.6506511	0.0468815	0.38987133	nonDEG	-0.2395637	0.55636365	1
0.28426418	0.28525958	0.86735764	nonDEG	-0.3987002	0.28111321	1
0.25931146	0.41933268	0.96282267	nonDEG	-0.0753819	0.85913117	1
0.14761654	0.57974894	1	nonDEG	0.2968987	0.51999196	1
-0.2143563	0.51053405	1	nonDEG	0.32866919	0.40066776	1
-0.4948669	0.13891991	0.66717926	nonDEG	0.15030583	0.72444769	1
-0.2289231	0.42207315	0.96420737	nonDEG	0.03923765	0.92031918	1
-0.1457552	0.87103812	1	nonDEG	-0.4347756	0.63375407	1
-0.2844037	0.59905953	1	nonDEG	-0.8987256	0.17412965	1
-0.2969612	0.24492291	0.8265359	nonDEG	-0.1695773	0.64285182	1
-0.0460123	0.88712715	1	nonDEG	0.49899484	0.19651055	1
0.26670302	0.31994517	0.90313771	nonDEG	-0.165317	0.65613029	1
0.24087938	0.33219293	0.91610564	nonDEG	-0.1101801	0.76019568	1
-0.3957711	0.38396162	0.94051418	nonDEG	0.51310796	0.50277571	1
-0.3863351	0.30868349	0.89257767	nonDEG	-0.8891673	0.07829354	1
0.91564896	0.49795372	1	nonDEG	-0.8878311	0.49956491	1
-0.0303681	0.90408332	1	nonDEG	0.20039874	0.57772016	1
-0.3020022	0.34176439	0.91869633	nonDEG	0.05296431	0.90120302	1
1.06278253	0.00395406	0.07536309	nonDEG	-0.3655437	0.38770441	1
-0.2903054	0.26585675	0.85062515	nonDEG	-0.6755204	0.06851407	1
-0.1678906	0.64450558	1	nonDEG	0.12617159	0.78794805	1
0.43966774	0.0909989	0.55042461	nonDEG	0.00428819	0.9922087	1
0.0513042	0.90672528	1	nonDEG	-0.3026907	0.56069525	1

0.5207311	0.13965107	0.66785296	nonDEG	0.01143663	0.98008722	1
-1.0867526	0.06725707	0.47390635	nonDEG	-0.5857341	0.38596971	1
0.21214025	0.60633474	1	nonDEG	-0.0532989	0.91982414	1
0.09637937	0.73206328	1	nonDEG	-0.1417378	0.71152017	1
0.0953763	0.71525269	1	nonDEG	-1.0090658	0.00814632	0.69918191
-0.2542778	0.30532289	0.89006022	nonDEG	0.13659627	0.73727842	1
0.47068671	0.25583072	0.83793955	nonDEG	-0.361862	0.46950857	1
0.88699692	0.0098625	0.1445207	nonDEG	0.07552919	0.85027497	1
-0.0219367	0.9473023	1	nonDEG	-0.9738961	0.0178011	0.95781535
-0.0024702	0.99532418	1	nonDEG	0.27922009	0.46012022	1
0.12090693	0.6463849	1	nonDEG	-0.115782	0.75251912	1
0.48165714	0.12083288	0.6327947	nonDEG	-0.0973195	0.8107576	1
0.2002199	0.43200705	0.97231377	nonDEG	0.15405824	0.67988745	1
0.6271022	0.01191615	0.16515005	nonDEG	0.05934024	0.87691782	1
-0.0780886	0.80104314	1	nonDEG	-0.4494789	0.25479248	1
-0.0909723	0.73007	1	nonDEG	-0.6393737	0.08846065	1
-0.0429406	0.87124222	1	nonDEG	-0.0541868	0.8835716	1
0.95662459	0.00577736	0.09819851	nonDEG	-0.3449852	0.46928828	1
-0.8732596	0.34354474	0.91869633	nonDEG	-0.1755898	0.93144339	1
0.1550058	0.65542559	1	nonDEG	0.72390068	0.07732877	1
-0.3573498	0.16979943	0.72708731	nonDEG	-0.2878314	0.44151348	1
-0.0339409	0.89287808	1	nonDEG	0.33176391	0.36606453	1
-0.5486048	0.26314467	0.84740606	nonDEG	0.7007629	0.22226151	1
-0.0959718	0.71274458	1	nonDEG	-0.0471008	0.89834954	1
-0.4576873	0.28452049	0.8670425	nonDEG	0.35083549	0.49075496	1
0.06727714	0.90279047	1	nonDEG	-0.0574005	0.93038053	1
0.43710618	0.35562268	0.91924469	nonDEG	0.10326593	0.86526003	1
0.02893014	0.90625827	1	nonDEG	-0.0924422	0.80543996	1
0.4358278	0.09175617	0.55224625	nonDEG	0.0226744	0.95232573	1
-0.01304	0.96172519	1	nonDEG	-0.288618	0.47660876	1
0.09042835	0.71849256	1	nonDEG	0.12737622	0.72838621	1
0.55508229	0.12092161	0.63285535	nonDEG	-0.0398867	0.92809888	1
-0.2183718	0.42936829	0.97101957	nonDEG	0.06330881	0.87248114	1
0.7577366	0.01588407	0.19738751	nonDEG	0.04426717	0.91438031	1
-0.1238577	0.67661963	1	nonDEG	-0.0434666	0.9127918	1
-0.2314278	0.34769615	0.91917773	nonDEG	0.06283802	0.86381281	1
-0.1977766	0.53471526	1	nonDEG	-0.0830667	0.85016927	1
-0.0607403	0.89355977	1	nonDEG	-0.1375046	0.79347916	1
0.6572166	0.08618937	0.53797919	nonDEG	-0.2265096	0.64802628	1
0.68109685	0.04444791	0.37643902	nonDEG	-0.2724998	0.56438813	1
-0.6632895	0.03011846	0.29847765	nonDEG	0.18661381	0.67719727	1
-0.0277503	0.91455936	1	nonDEG	0.11570823	0.7572097	1
0.1499073	0.53584566	1	nonDEG	-0.1545464	0.67449482	1

-0.0994411	0.71436653	1	nonDEG	-0.0222216	0.95383293	1
-0.2256208	0.36363753	0.92428987	nonDEG	0.29720282	0.44780285	1
-1.2254838	0.39844388	0.95139829	nonDEG	-0.1561999	1	1
-0.1334267	0.80766644	1	nonDEG	-0.0483567	0.94846716	1
-0.9635946	0.06231542	0.45672559	nonDEG	0.11517887	0.86548756	1
-0.529648	0.03814671	0.34416209	nonDEG	-0.0008941	0.99921123	1
0.07675955	0.82206683	1	nonDEG	0.41264204	0.31358674	1
-0.1788724	0.46700724	0.98651834	nonDEG	-0.0090996	0.9804817	1
-0.1674666	0.76317302	1	nonDEG	-0.2227914	0.72294018	1
-0.0281222	0.92048998	1	nonDEG	0.17894962	0.63232148	1
0.3775978	0.17247486	0.73141563	nonDEG	0.13653922	0.72037694	1
0.41720115	0.2004998	0.77325509	nonDEG	0.19941603	0.65979269	1
0.40082886	0.11657794	0.62455648	nonDEG	0.0644013	0.85982897	1
0.10451489	0.71413612	1	nonDEG	-0.044572	0.90930697	1
0.16261197	0.6991383	1	nonDEG	-1.3280089	0.01051338	0.77777211
0.64756315	0.05950445	0.4448328	nonDEG	0.12636186	0.75982134	1
0.33456462	0.91258075	1	nonDEG	-0.9658283	0.65915809	1
0.41442269	0.24564008	0.82672898	nonDEG	-0.3471056	0.43263545	1
0.24115281	0.32384068	0.90721721	nonDEG	0.13481676	0.73903081	1
-0.3442459	0.49162215	0.99972374	nonDEG	-0.8793797	0.14752595	1
0.26150526	0.39451199	0.94862012	nonDEG	0.0484927	0.90332151	1
-0.3039363	0.52135863	1	nonDEG	0.49507912	0.37040729	1
0.12211345	0.79733604	1	nonDEG	0.6298683	0.24403985	1
-0.0256716	0.93355086	1	nonDEG	-0.2223079	0.57076119	1
0.03656807	0.88257472	1	nonDEG	0.18120973	0.62104596	1
0.07488087	0.79507811	1	nonDEG	0.26300675	0.4892677	1
-1.7873797	0.03988777	0.35376105	nonDEG	1.18598863	0.22463863	1
0.0897682	0.75268413	1	nonDEG	-0.0747138	0.84468274	1
0.59007894	0.09654896	0.56709992	nonDEG	0.40429804	0.31688659	1
0.19929492	0.40760646	0.95712903	nonDEG	-0.3836365	0.29597858	1
0.120327	0.67596208	1	nonDEG	0.06057866	0.87691642	1
0.06774645	1	1	nonDEG	1.05286443	0.39231928	1
0.44958348	0.14234314	0.67359585	nonDEG	0.07971948	0.84366975	1
0.00761454	0.97787733	1	nonDEG	0.07133621	0.84628482	1
0.38795722	0.30186879	0.88648699	nonDEG	0.17016469	0.69759994	1
0.09268785	0.78131908	1	nonDEG	0.21189458	0.606411	1
0.28381077	0.460802	0.98479636	nonDEG	-0.6681323	0.16941576	1
-0.3329788	0.2252581	0.80634403	nonDEG	0.23791901	0.51727419	1
-0.6946364	0.4865577	0.99711758	nonDEG	0.6237169	0.57060426	1
-0.578946	0.01912099	0.22290298	nonDEG	0.08717002	0.81174374	1
9.97E-05	1	1	nonDEG	-0.1868629	0.60764592	1
-0.311771	0.45934043	0.98479636	nonDEG	0.11111808	0.83502528	1
0.82764824	0.02091248	0.23614981	nonDEG	0.60370425	0.19437333	1

-0.3770655	0.12273415	0.63859502	nonDEG	0.11460881	0.75056441	1
0.22170959	0.37897281	0.93586897	nonDEG	0.13262585	0.77625792	1
0.31551057	0.19555211	0.76634006	nonDEG	0.21932018	0.59402312	1
0.43562775	0.25095538	0.83275514	nonDEG	-0.6348884	0.17499447	1
0.14293142	0.62579612	1	nonDEG	0.28758705	0.54057395	1
-0.1339151	0.59075514	1	nonDEG	-0.3632756	0.31554986	1
-0.1817097	1	1	nonDEG	0.41200993	0.88714458	1
-0.2806285	0.31150373	0.89482226	nonDEG	-0.3709217	0.31411403	1
-0.0258801	0.92312505	1	nonDEG	0.13392943	0.72000535	1
0.01082938	0.97323462	1	nonDEG	0.16989289	0.67487285	1
0.41470571	0.10138216	0.58073682	nonDEG	-0.3070188	0.39748164	1
0.05170791	0.83424708	1	nonDEG	-0.2901252	0.43338063	1
1.34088977	0.01148689	0.16110651	nonDEG	0.60275337	0.40756532	1
0.08507573	0.72781031	1	nonDEG	0.08233443	0.81946647	1
0.65031472	0.07191957	0.4897356	nonDEG	-1.5170024	0.00051945	0.14642867
-0.1905145	0.44641817	0.98124647	nonDEG	0.09275817	0.80890648	1
-0.2432268	0.66619866	1	nonDEG	0.28098355	0.67875629	1
-0.3604207	0.14739313	0.68332382	nonDEG	0.08917562	0.83212956	1
0.08660466	0.76013796	1	nonDEG	0.31329255	0.42227117	1
-0.5243959	0.18426196	0.75193897	nonDEG	-0.3399344	0.5275664	1
-0.2875252	0.29994	0.88467981	nonDEG	0.37232499	0.3212558	1
-0.0331327	0.92665398	1	nonDEG	-0.1182664	0.77742178	1
0.08894655	0.77487076	1	nonDEG	0.69768698	0.07348579	1
-0.2066734	0.56804333	1	nonDEG	0.4097373	0.35464574	1
-0.1556602	0.82601999	1	nonDEG	-0.1015144	0.89837396	1
-0.114165	0.73663584	1	nonDEG	0.14804123	0.72255491	1
-0.1107761	0.67972285	1	nonDEG	-0.2682734	0.4741878	1
-0.2965646	0.26799347	0.85190792	nonDEG	-0.1440204	0.7153552	1
0.02654823	0.91886015	1	nonDEG	-0.4849756	0.20557128	1
0.61391717	0.05626902	0.43190589	nonDEG	0.10432384	0.79572036	1
0.17151158	0.49673236	1	nonDEG	0.3644727	0.34069894	1
-0.8894605	0.00343036	0.06855832	nonDEG	-0.0161186	0.97576578	1
0.23200431	0.43673163	0.97402891	nonDEG	-0.2919688	0.44447352	1
0.09492827	0.69867496	1	nonDEG	-0.1414364	0.70407872	1
0.51019684	0.79744518	1	nonDEG	-2.8182412	0.16606899	1
0.35709952	0.17477823	0.73757113	nonDEG	0.17538959	0.65660666	1
1.5319067	0.21540127	0.79470372	nonDEG	0.08800342	0.95328327	1
0.18429261	0.50475795	1	nonDEG	-0.3704697	0.36777268	1
-0.365707	0.17293914	0.73254035	nonDEG	-0.1402461	0.75783029	1
-0.1817228	0.66625488	1	nonDEG	-0.8507728	0.13679036	1
-0.3520491	0.50101072	1	nonDEG	-0.1567498	0.84266272	1
-0.1031574	0.83811307	1	nonDEG	-0.9523356	0.11629908	1
-0.1146979	0.67530609	1	nonDEG	-0.3659738	0.33061505	1

-0.918904	0.00456619	0.08338966	nonDEG	0.22241558	0.62045484	1
-0.0965222	0.80007892	1	nonDEG	0.00709078	0.9928823	1
0.3215748	0.45289741	0.98329655	nonDEG	0.49863134	0.29607193	1
0.07817781	0.80293283	1	nonDEG	-0.0411507	0.91974961	1
0.56625708	0.20592937	0.7803135	nonDEG	-0.0774326	0.8793334	1
0.06274565	0.79357082	1	nonDEG	0.07295247	0.8395337	1
6.08434459	0.0252287	0.26681248	nonDEG	-5.9292678	0.03622471	1
-0.0058723	0.98968979	1	nonDEG	0.13506684	0.75976943	1
0.17052957	0.62747284	1	nonDEG	0.27683373	0.51209636	1
0.66115547	0.00815568	0.1262735	nonDEG	-0.0380527	0.91912506	1
-0.250961	0.41704282	0.9621727	nonDEG	0.06432356	0.8868161	1
-0.0808045	0.77374673	1	nonDEG	0.23013902	0.54045801	1
0.1446791	0.6636325	1	nonDEG	0.15549546	0.71259176	1
0.11712843	0.68525808	1	nonDEG	0.07127135	0.85590364	1
0.14416467	0.58031348	1	nonDEG	0.28326089	0.43769583	1
0.02762052	0.91573133	1	nonDEG	0.0945881	0.79539862	1
0.18100614	0.48658552	0.99711758	nonDEG	0.04689137	0.89743933	1
-0.1541854	0.56240376	1	nonDEG	-0.0769839	0.8422217	1
-0.0394223	0.90581415	1	nonDEG	-0.4505251	0.28991928	1
-0.1690614	0.57426573	1	nonDEG	-0.2724729	0.53645484	1
-0.16248	0.70293094	1	nonDEG	0.22103569	0.67539648	1
0.0316965	0.89836268	1	nonDEG	-0.0869928	0.82223669	1
-0.2272706	0.47014425	0.98861261	nonDEG	-0.1954629	0.64255125	1
-0.0289681	0.90777221	1	nonDEG	0.32583037	0.39061547	1
-0.042491	0.91433265	1	nonDEG	0.4255056	0.34943775	1
-0.0574798	0.8227868	1	nonDEG	0.03202916	0.93101655	1
-0.2317784	0.53283435	1	nonDEG	-0.1416051	0.77526655	1
-0.0852527	0.75348508	1	nonDEG	0.17254051	0.64327693	1
0.6282719	0.03160686	0.30779829	nonDEG	-0.2158036	0.63048997	1
-0.5413307	0.07887734	0.5143012	nonDEG	-0.2501369	0.53824505	1
0.4998375	0.13618911	0.66318137	nonDEG	-0.4465857	0.26157727	1
0.00801083	0.98208777	1	nonDEG	0.4000109	0.31425374	1
-0.2332938	0.60048369	1	nonDEG	-1.2221006	0.03961644	1
-0.4752729	0.07154394	0.48822021	nonDEG	0.15502453	0.6820385	1
-0.1834503	0.51772489	1	nonDEG	0.30604029	0.45524767	1
-0.9426772	0.02827318	0.28790528	nonDEG	0.53213628	0.31968394	1
0.25059052	0.88374383	1	nonDEG	-0.0851435	1	1
-0.4330788	0.39391003	0.94818621	nonDEG	-0.2829081	0.65423916	1
-0.0133063	1	1	nonDEG	0.30373295	0.82898922	1
-0.0332294	0.96496345	1	nonDEG	-0.2550421	0.77468694	1
0.35721675	0.32977679	0.91380796	nonDEG	0.34652504	0.44006454	1
0.83515712	0.01236739	0.16837141	nonDEG	0.53690756	0.20400152	1
0.81389959	0.01503373	0.19076641	nonDEG	-0.2009615	0.6746181	1

0.12391584	0.82463033	1	nonDEG	0.41863553	0.50739606	1
-0.6043819	0.61614172	1	nonDEG	-1.4518763	0.31091741	1
-0.0181299	0.94391811	1	nonDEG	-0.1816711	0.62009862	1
-0.75298	0.0198092	0.22788708	nonDEG	0.24116996	0.5707763	1
0.23587326	0.37529085	0.93344578	nonDEG	-0.3427251	0.35554904	1
-0.1221638	0.67702896	1	nonDEG	-0.6792194	0.0740763	1
-0.1769849	0.70330756	1	nonDEG	0.36770952	0.49382833	1
0.50802074	0.19077224	0.75822114	nonDEG	0.38924916	0.38071765	1
1.33209476	0.55484329	1	nonDEG	2.00126835	0.27621024	1
0.15542138	0.54647408	1	nonDEG	-0.4184443	0.25983587	1
-0.1868836	0.46748349	0.986734	nonDEG	0.00052799	0.99973698	1
-0.3470933	0.28235034	0.86377551	nonDEG	-0.2729464	0.52219174	1
-0.021638	0.93330993	1	nonDEG	-0.1477777	0.71346402	1
-0.3302304	0.25381891	0.83543996	nonDEG	-0.5370521	0.22192816	1
-0.2752689	0.32709529	0.91107605	nonDEG	-0.2409983	0.53045302	1
0.0957664	0.7280178	1	nonDEG	0.58198567	0.17401726	1
0.08043655	0.79222456	1	nonDEG	-0.707196	0.08397368	1
0.29562118	0.30229616	0.88691415	nonDEG	0.24951858	0.50652554	1
-0.0125557	0.97126728	1	nonDEG	-0.1516072	0.72056773	1
-2.8023827	0.00323659	0.06575697	nonDEG	1.35859339	0.26288004	1
0.68625307	0.13390433	0.6590887	nonDEG	-0.1255372	0.81795332	1
0.64077561	0.01239314	0.16844031	nonDEG	-0.0566439	0.88774007	1
0.07802626	0.90330074	1	nonDEG	0.48944665	0.45735832	1
-0.0681629	0.78022764	1	nonDEG	0.07175846	0.87736171	1
0.64933289	0.01345903	0.17656877	nonDEG	-0.1578884	0.66763507	1
0.21650996	0.48928516	0.99854875	nonDEG	-0.015855	0.9702195	1
-1.3103506	0.38347367	0.93981697	nonDEG	-0.397115	0.8903437	1
1.00797492	0.46703713	0.98651834	nonDEG	-1.0466282	0.36753627	1
0.29359805	0.31509981	0.89824365	nonDEG	-0.0111099	0.98047237	1
-0.1911478	0.43954062	0.97629962	nonDEG	0.28704474	0.52103739	1
0.14396643	0.72342456	1	nonDEG	0.44972149	0.47630036	1
0.38494207	0.37493343	0.93302328	nonDEG	-0.2828526	0.59228005	1
0.13211305	0.62818286	1	nonDEG	-0.1316986	0.72300702	1
-0.7214	0.22235231	0.80228334	nonDEG	-0.574227	0.41039259	1
-1.912738	0.05320421	0.4180087	nonDEG	1.23099354	0.2319207	1
-0.4078272	0.11852397	0.62868959	nonDEG	-0.1815231	0.64063862	1
0.02698174	0.91260354	1	nonDEG	-0.1474977	0.69816697	1
0.22105427	0.44638514	0.98124647	nonDEG	0.11901366	0.7512682	1
6.11533777	0.00876093	0.13308212	nonDEG	-1.8252967	0.20277462	1
-0.0008959	1	1	nonDEG	-0.0704686	0.90488887	1
0.11624847	0.7370711	1	nonDEG	0.25213797	0.56611992	1
0.0682357	0.85599851	1	nonDEG	0.43942583	0.31592921	1
1.30871038	0.05248903	0.41538295	nonDEG	-1.4180235	0.02948881	1

-3.6811888	0.46241232	0.98479636	nonDEG	0	1	1
5.13E-15	1	1	nonDEG	0	1	1
-0.1530865	0.54816725	1	nonDEG	0.41864493	0.24869917	1
-0.1630148	0.68624314	1	nonDEG	0.16093085	0.77418775	1
-0.005064	0.98480248	1	nonDEG	0.09761768	0.79287599	1
0.0784145	0.87235134	1	nonDEG	-0.095709	0.87500352	1
0.51766196	0.2721219	0.85514356	nonDEG	-0.7211021	0.2174276	1
0.62082708	0.01748805	0.20970704	nonDEG	-0.3181305	0.41334812	1
-0.4263069	0.19371192	0.76371839	nonDEG	0.10391704	0.83806649	1
0.6463678	0.01840613	0.21679329	nonDEG	0.08327893	0.84469333	1
-0.3857422	0.1205919	0.63225378	nonDEG	-0.048506	0.89916253	1
-0.1174993	0.70606077	1	nonDEG	0.29571508	0.46566204	1
0.33753713	0.17302655	0.73256216	nonDEG	-0.2260097	0.62941167	1
0.0869851	0.88900846	1	nonDEG	0.10262299	0.87185544	1
0.26304043	0.28814829	0.87024354	nonDEG	-0.2498868	0.48867811	1
-0.0943733	0.79428602	1	nonDEG	-0.3453805	0.48954562	1
1.02511072	0.10432809	0.59120592	nonDEG	-1.0835571	0.13710123	1
-0.485226	0.08142399	0.52257268	nonDEG	-0.288548	0.46053556	1
0.7671012	0.52980735	1	nonDEG	-0.7259003	0.58265235	1
-0.4987363	0.70042098	1	nonDEG	0.95981733	0.45644121	1
1.08864741	0.20819637	0.7838989	nonDEG	-1.9444335	0.04259958	1
-0.5433983	0.76043761	1	nonDEG	-0.4543998	0.76742394	1
0.70208588	0.35621887	0.91924469	nonDEG	0.02135221	0.98082787	1
-0.1264024	0.69021687	1	nonDEG	0.39761816	0.32675754	1
-0.9689785	0.83293076	1	nonDEG	1.81007596	0.41370798	1
0.33652797	0.2840791	0.86663475	nonDEG	-0.0838264	0.83149582	1
0.594839	0.2322432	0.81180331	nonDEG	0.20339988	0.74988598	1
-0.0226459	0.97373364	1	nonDEG	-0.7913517	0.22803265	1
0.77594995	0.00494975	0.08792926	nonDEG	-0.1098041	0.76388516	1
-0.0996347	0.81532376	1	nonDEG	0.24444654	0.6540914	1
1.8569476	0.29046697	0.87282735	nonDEG	1.72011019	0.15167911	1
1.02033721	0.00456525	0.08338966	nonDEG	0.08570717	0.87303279	1
-0.2514483	0.36034493	0.92312867	nonDEG	-0.3648144	0.36760009	1
0.61789509	0.02927586	0.29424792	nonDEG	0.10106013	0.79703581	1
0.29645225	0.27949781	0.86140452	nonDEG	0.31305913	0.46603649	1
-0.0965345	0.89008677	1	nonDEG	-0.4032567	0.58749839	1
-0.1249786	0.69838282	1	nonDEG	0.13387177	0.73981388	1
0.22474304	0.45597045	0.98394385	nonDEG	-0.1613252	0.67029988	1
0.06693622	1	1	nonDEG	-0.688768	0.6814799	1
1.35606452	0.00304398	0.0627547	nonDEG	-1.1825726	0.00793237	0.69089048
0.10822741	0.71135979	1	nonDEG	-0.1817418	0.66297099	1
-0.6118039	0.34944943	0.91917773	nonDEG	0.33485636	0.64850681	1
0.28244909	0.274822	0.85835498	nonDEG	-0.0056381	0.98948298	1

-0.0055875	0.98218688	1	nonDEG	0.09857152	0.78673553	1
-0.7610434	0.20049769	0.77325509	nonDEG	1.49735547	0.00408149	0.52015192
0.13001155	0.59963651	1	nonDEG	0.54735824	0.1654309	1
-0.5592696	0.03049244	0.30112943	nonDEG	0.14681186	0.69383035	1
-0.0197155	0.94515824	1	nonDEG	0.20416047	0.60348661	1
0.89055753	0.068737	0.47928674	nonDEG	-0.0524822	0.92592329	1
0.61327244	0.28654609	0.86884647	nonDEG	-0.0118287	0.98734609	1
1.15272637	0.24520835	0.8265359	nonDEG	0.29653141	0.78313911	1
0.72385216	0.08072534	0.52017157	nonDEG	-2.0523677	0.00069851	0.17727188
-0.1294569	0.69274289	1	nonDEG	-0.2573341	0.56275149	1
-0.1907614	0.91195952	1	nonDEG	-1.01024	0.62242615	1
-0.1608142	0.92484666	1	nonDEG	-3.3563528	0.05736763	1
-0.6859392	0.1133236	0.61540924	nonDEG	1.01690291	0.04284921	1
0.58336567	0.14152534	0.67177078	nonDEG	-0.6360888	0.19965131	1
1.08314945	0.29065283	0.8729277	nonDEG	-5.1081755	0.00037925	0.11630021
-0.1788843	0.57381278	1	nonDEG	-0.1493512	0.72096933	1
-0.0369635	0.93146802	1	nonDEG	0.97353123	0.02563008	1
0.19925917	0.66053571	1	nonDEG	0.47293428	0.38749958	1
-2.2851247	1	1	nonDEG	0	1	1
-0.2996812	0.67783587	1	nonDEG	0.51812715	0.59465099	1
0.32121303	0.20770652	0.78320256	nonDEG	-0.015226	0.96671053	1
-0.1642651	0.73291125	1	nonDEG	0.81379223	0.19870631	1
0.39435725	0.11063453	0.60775241	nonDEG	-0.2101148	0.56482421	1
0.34977312	0.15685025	0.70153162	nonDEG	-0.0726338	0.84819457	1
-0.3977731	0.42294018	0.96526365	nonDEG	0.75550731	0.14968127	1
1.57720007	0.00254343	0.05452677	nonDEG	0.94718561	0.30780047	1
3.3819027	0.13233896	0.65711072	nonDEG	-0.229994	0.91591809	1
0.84706543	0.00293304	0.06080692	nonDEG	-0.06358	0.87389463	1
0.59883205	0.21081414	0.7877265	nonDEG	-0.9171367	0.11288324	1
0.25246035	0.5849614	1	nonDEG	-1.349383	0.00839624	0.71081252
-0.2399077	0.41750915	0.96239407	nonDEG	0.22226226	0.56708781	1
0.39039757	0.26091329	0.84406883	nonDEG	0.31803548	0.46088252	1
1.43442933	0.12976434	0.65346088	nonDEG	-0.8159724	0.40110702	1
0.45136023	0.57622229	1	nonDEG	-0.261449	0.77034414	1
0.53016923	0.05997453	0.44667826	nonDEG	0.46006585	0.24508895	1
0.19774957	0.60173916	1	nonDEG	-0.5876194	0.25100587	1
-0.1825054	0.78857975	1	nonDEG	-0.9993009	0.21723196	1
-0.2210374	0.41416429	0.96004586	nonDEG	0.77049709	0.05182263	1
-0.2087878	0.40205071	0.95307326	nonDEG	0.22111814	0.56571144	1
-0.1193343	0.65927968	1	nonDEG	-0.0324464	0.93347906	1
0.72765328	0.45320047	0.98329655	nonDEG	-1.8744969	0.09145313	1
-0.3819602	0.50291639	1	nonDEG	-0.7840001	0.26054792	1
0.23217035	0.3426853	0.91869633	nonDEG	-0.1568419	0.73870865	1

-0.2243016	0.41609518	0.96166196	nonDEG	-0.3607386	0.37180928	1
-0.4885788	0.06796503	0.47559369	nonDEG	-0.0847091	0.84007651	1
0.89498013	0.42674983	0.96847735	nonDEG	-3.5533038	0.01056634	0.77960522
-0.4340047	0.32684786	0.91069401	nonDEG	0.71434904	0.28465403	1
0.18974384	0.62612461	1	nonDEG	0.11493627	0.86147811	1
-0.2260331	0.43454158	0.973123	nonDEG	-0.3771678	0.35443891	1
0.18533522	0.61646444	1	nonDEG	-0.2020376	0.66273378	1
1.46577215	0.04673238	0.38914754	nonDEG	-0.1588025	0.83364079	1
-0.786342	0.12391923	0.6417591	nonDEG	-0.4178606	0.50423003	1
1.30753933	0.39454913	0.94862012	nonDEG	-2.9405949	0.02901355	1
-0.4383683	0.67017471	1	nonDEG	1.48152955	0.10817869	1
-0.2937748	0.2578719	0.83898637	nonDEG	0.07714766	0.83299165	1
-0.8797976	0.38630203	0.94282419	nonDEG	1.73929159	0.06304934	1
-0.9797825	0.34288153	0.91869633	nonDEG	1.8844826	0.05355363	1
-2.3277844	0.022743	0.24961389	nonDEG	-1.7198518	0.20359055	1
-0.7108168	0.28571256	0.86801776	nonDEG	0.24369205	0.76110055	1
-0.4485455	0.19196536	0.76035391	nonDEG	-0.1686509	0.71368881	1
-0.4549258	0.18105382	0.7465461	nonDEG	0.00830708	0.98765528	1
0.03668642	0.96421624	1	nonDEG	0.11198151	0.91739605	1
0.16875338	0.82437318	1	nonDEG	0.02467473	0.98873887	1
0.91650918	0.32645796	0.91057556	nonDEG	0.95374571	0.32950169	1
-1.6945256	0.01648907	0.2015034	nonDEG	0.23374779	0.82707657	1
0.47610433	0.06552935	0.46766512	nonDEG	0.37936688	0.3338818	1
-0.7376519	0.31535887	0.89842779	nonDEG	0.34941927	0.7195745	1
0.84732687	0.06785876	0.47541505	nonDEG	0.47027666	0.3358521	1
-0.0551145	0.96431357	1	nonDEG	1.01536139	0.38671026	1
0.33094133	0.3265683	0.91057556	nonDEG	-0.5610063	0.18654164	1
-1.4254333	0.00684541	0.11109256	nonDEG	-0.1470419	0.84437521	1

DEG/nonDEG	Comparison_4	Comparison_4	Comparison_4	DEG/nonDEG	Comparison_5	Comparison_5
nonDEG	0.24339973	0.57818015	1	nonDEG	-0.240335	0.5134336
nonDEG	0.71116535	0.10000978	1	nonDEG	-0.5966393	0.10231555
nonDEG	0.19873701	0.69672319	1	nonDEG	-0.3067707	0.51616181
nonDEG	0.13858842	0.76708692	1	nonDEG	-0.2332017	0.56441063
nonDEG	-0.0815305	0.8581278	1	nonDEG	0.18377889	0.62725151
nonDEG	-0.7578404	0.15159025	1	nonDEG	0.97172347	0.01130357
nonDEG	0.45742448	0.40197354	1	nonDEG	-0.6411634	0.23448405
nonDEG	-0.2012836	0.63847139	1	nonDEG	0.20153697	0.57634008
nonDEG	0.78470337	0.11028985	1	nonDEG	-0.1471008	0.74370022
nonDEG	-1.1125659	0.09284782	1	nonDEG	0.71118679	0.22697271
nonDEG	-0.4610434	0.3890173	1	nonDEG	0.03384189	0.94824649
nonDEG	0.93969975	0.09236065	1	nonDEG	-0.0668908	0.89679112
nonDEG	-0.8263946	0.15727623	1	nonDEG	0.87871542	0.14736892
nonDEG	0.55413154	0.23421962	1	nonDEG	-0.1888096	0.64409732
nonDEG	0.75759278	0.23411698	1	nonDEG	-0.1480387	0.79468441
nonDEG	-0.3866696	0.46089679	1	nonDEG	-0.708592	0.18720913
nonDEG	-0.4130493	0.47816247	1	nonDEG	0.3267724	0.55304295
nonDEG	1.03870873	0.27947637	1	nonDEG	0.10817529	0.90337395
nonDEG	1.18925865	0.14758276	1	nonDEG	1.69671024	0.00089672
nonDEG	0.27300334	0.55769393	1	nonDEG	0.29829193	0.42221151
nonDEG	0.65182217	0.19760137	1	nonDEG	0.02277769	0.95992643
nonDEG	1.85921433	0.01270652	0.85364221	nonDEG	-0.5094522	0.36456124
nonDEG	0.9276843	0.04902434	1	nonDEG	-0.3818532	0.31053843
nonDEG	-0.5245019	0.65483661	1	nonDEG	2.02467942	0.08754439
nonDEG	-1.531139	0.00963871	0.79482907	nonDEG	2.36142519	0.00027029
nonDEG	0.52223535	0.22222558	1	nonDEG	-0.1391369	0.70447983
nonDEG	0.02966701	0.94987115	1	nonDEG	-0.5323758	0.18217389
nonDEG	-1.1179526	0.21902158	1	nonDEG	0.53097998	0.53814626
nonDEG	0.2769711	0.607303	1	nonDEG	0.24435514	0.65898144
nonDEG	-1.8558883	0.00327695	0.65691312	nonDEG	1.72930455	0.0039274
nonDEG	-0.1501274	0.77825596	1	nonDEG	-0.0373912	0.9430366
nonDEG	1.27307157	0.34054676	1	nonDEG	0.15784251	0.89978158
nonDEG	1.27556315	0.12691678	1	nonDEG	0.52260394	0.49586893
nonDEG	0.40088005	0.41289321	1	nonDEG	0.21085031	0.62076508
nonDEG	-0.0385352	0.93600959	1	nonDEG	0.30988227	0.44557133
nonDEG	-0.2325508	0.61138482	1	nonDEG	-0.5252317	0.18261288
nonDEG	-0.9693199	0.16954056	1	nonDEG	1.64293662	0.02097076
nonDEG	-1.4095754	0.21771546	1	nonDEG	1.92255825	0.11240316
nonDEG	-0.2101324	0.62871622	1	nonDEG	0.4333215	0.27395282
nonDEG	2.20347998	0.02853149	1	nonDEG	-2.0973406	0.06948297
nonDEG	-0.8220167	0.10900042	1	nonDEG	1.06758175	0.03806526

nonDEG	0.18093359	0.74499956	1	nonDEG	-0.3398239	0.45366032
nonDEG	-1.2077419	0.01960298	0.97153571	nonDEG	0.77035583	0.11569015
nonDEG	-0.2494412	0.68999912	1	nonDEG	0.06706257	0.88540795
nonDEG	0.72651895	0.17288973	1	nonDEG	0.2165685	0.62833952
nonDEG	0.15968927	0.76608102	1	nonDEG	0.02587099	0.95475268
nonDEG	1.56382593	0.00429178	0.68407511	nonDEG	-0.4530693	0.27064283
nonDEG	0.41315745	0.41261576	1	nonDEG	-1.6862981	0.00035318
nonDEG	0.40139644	0.34556919	1	nonDEG	-0.3691215	0.30484342
nonDEG	0.88863026	0.06688528	1	nonDEG	-0.5654181	0.18078164
nonDEG	1.17189354	0.05074283	1	nonDEG	0.21751831	0.67936066
nonDEG	0.34325928	0.42623667	1	nonDEG	0.07893825	0.8325186
nonDEG	-0.3307376	0.46838604	1	nonDEG	0.61627187	0.1562819
nonDEG	-0.266391	0.52458831	1	nonDEG	-0.638991	0.08529879
nonDEG	0.34315778	0.48777013	1	nonDEG	0.05648586	0.90098229
nonDEG	0.26206893	0.54435614	1	nonDEG	-0.5464771	0.13992272
nonDEG	0.66929773	0.20046055	1	nonDEG	-0.1464471	0.77902057
DEG	0.6643951	0.49728457	1	nonDEG	-0.5943678	0.36956567
nonDEG	0.8749768	0.23049921	1	nonDEG	0.10776071	0.88362445
nonDEG	1.71480391	0.00242396	0.61776954	nonDEG	-0.3129211	0.52124517
nonDEG	0.48008565	0.45167063	1	nonDEG	-0.1644892	0.77126702
nonDEG	1.21185908	0.00776606	0.75581042	nonDEG	-0.6771073	0.08785593
nonDEG	-1.0598036	0.26384538	1	nonDEG	-1.4431696	0.06252372
nonDEG	-1.6934522	0.09732488	1	nonDEG	0.44953512	0.54049151
nonDEG	-0.4933487	0.29650027	1	nonDEG	0.46903216	0.28447111
nonDEG	1.05159564	0.09454529	1	nonDEG	-0.9623571	0.12471872
nonDEG	0.08208101	0.87824286	1	nonDEG	0.13073285	0.78774106
nonDEG	0.41666844	0.3636711	1	nonDEG	-0.2450262	0.50723996
nonDEG	-0.8679648	0.06215859	1	nonDEG	0.99087612	0.02325647
nonDEG	-0.4453372	0.68898714	1	nonDEG	0.29465659	0.80899349
nonDEG	-3.0017558	0.00106434	0.51315728	nonDEG	3.24305051	0.00016167
nonDEG	0.0518334	0.91450631	1	nonDEG	0.32632463	0.43507626
nonDEG	-0.3180287	0.59100979	1	nonDEG	0.6601952	0.22247532
nonDEG	-0.4209196	0.33720928	1	nonDEG	0.36341508	0.3212797
nonDEG	0.53874238	0.31745764	1	nonDEG	-0.2127106	0.61165661
nonDEG	-1.1813362	0.17535679	1	nonDEG	1.83023308	0.02055793
nonDEG	-1.1997451	0.05563458	1	nonDEG	1.4526601	0.02172667
nonDEG	0.29769776	0.48923378	1	nonDEG	0.00664555	0.98615125
nonDEG	0.50008761	0.4047203	1	nonDEG	-0.3888088	0.3731541
nonDEG	-0.325748	0.45768269	1	nonDEG	-0.3217446	0.42979872
nonDEG	0.26345647	0.53901865	1	nonDEG	0.08970138	0.81138998
nonDEG	0.33578835	0.57098103	1	nonDEG	-0.2277254	0.69251371
nonDEG	0.842578	0.06631628	1	nonDEG	-0.3057006	0.44056378
nonDEG	-0.6020816	0.19664403	1	nonDEG	-0.1695245	0.65360096

nonDEG	0.69808555	0.17880266	1	nonDEG	-0.3755515	0.32401154
nonDEG	0.39462604	0.41051847	1	nonDEG	-0.075771	0.84499604
nonDEG	0.4774108	0.35721764	1	nonDEG	0.14028332	0.71024964
nonDEG	0.32934546	0.69304161	1	nonDEG	-0.8317677	0.35662463
nonDEG	-1.1101168	0.14554691	1	nonDEG	1.07893689	0.08111804
nonDEG	-0.4358913	0.30143325	1	nonDEG	0.3573158	0.3598418
nonDEG	-2.0874362	0.00833231	0.77871384	nonDEG	2.01949456	0.01033321
nonDEG	-0.2895466	0.49073501	1	nonDEG	-0.384896	0.29941604
nonDEG	-2.732581	0.31590641	1	nonDEG	3.77102572	0.06027271
nonDEG	1.62943452	0.03440584	1	nonDEG	-0.4609581	0.4042537
nonDEG	1.35905869	0.00375817	0.67010784	nonDEG	-0.2438049	0.50554899
nonDEG	0.69736691	0.10202792	1	nonDEG	-0.2637276	0.46280253
nonDEG	-0.8259509	0.37692624	1	nonDEG	0.6296582	0.51062046
nonDEG	0.06553901	0.90454788	1	nonDEG	-0.5154999	0.22305982
nonDEG	0.00828355	0.98556896	1	nonDEG	0.09041083	0.81662754
nonDEG	0.98714794	0.02432199	1	nonDEG	-0.2094286	0.57483236
nonDEG	-0.2507194	0.59707084	1	nonDEG	0.05962005	0.88469313
nonDEG	0.69273669	0.133545	1	nonDEG	-0.2132881	0.5726672
nonDEG	-0.0508133	0.96952953	1	nonDEG	0.75772692	0.35671569
nonDEG	-0.5272959	0.31483846	1	nonDEG	0.40812009	0.41484307
nonDEG	0.36340498	0.42264099	1	nonDEG	0.0018814	0.99728004
nonDEG	2.92741415	0.00768062	0.75581042	nonDEG	-0.2916697	0.76358599
nonDEG	0.28797626	0.50831757	1	nonDEG	-0.2307913	0.535657
nonDEG	0.3515691	0.44857157	1	nonDEG	-0.7114604	0.05319981
nonDEG	0.05425731	0.91082837	1	nonDEG	0.34931302	0.43397313
nonDEG	0.87655224	0.15263939	1	nonDEG	0.45714844	0.37474748
nonDEG	-1.2488676	0.07138629	1	nonDEG	1.91691541	0.00411455
nonDEG	0.36410837	0.40197022	1	nonDEG	-0.1409536	0.69764315
nonDEG	-0.2911645	0.52058864	1	nonDEG	-0.3146629	0.42018062
nonDEG	0.76688787	0.4801756	1	nonDEG	-0.7170007	0.50618754
nonDEG	-0.5423162	0.28339282	1	nonDEG	-0.0077762	0.98571326
nonDEG	-1.258404	0.0052547	0.72753356	nonDEG	-0.5699969	0.17250909
nonDEG	1.41806509	0.02778331	1	nonDEG	-0.6148733	0.08838014
nonDEG	-1.2874084	0.09861668	1	nonDEG	1.99614027	0.01721253
nonDEG	1.60429794	0.00046149	0.41795603	nonDEG	-0.1024052	0.79291512
nonDEG	0.90410366	0.03745417	1	nonDEG	-0.0485308	0.89480584
nonDEG	-0.6152289	0.30912519	1	nonDEG	-0.0684946	0.88975427
nonDEG	-1.0450401	0.03994167	1	nonDEG	1.14277558	0.05567465
nonDEG	0.45065386	0.33177138	1	nonDEG	-0.1553551	0.68047633
nonDEG	-0.8906118	0.07155078	1	nonDEG	0.369742	0.37853612
nonDEG	-0.7689554	0.09882612	1	nonDEG	0.54274823	0.16786767
nonDEG	0.48436091	0.290329	1	nonDEG	0.0260451	0.94800093
nonDEG	-1.8123523	0.02276219	1	nonDEG	1.54019999	0.06640604

nonDEG	-0.4627912	0.40692503	1	nonDEG	0.6341601	0.25490304
nonDEG	1.26692173	0.08836604	1	nonDEG	-0.0642751	0.93874626
nonDEG	0.15041227	0.73145145	1	nonDEG	-0.0106755	0.97779054
nonDEG	0.45346545	0.43672301	1	nonDEG	0.05077617	0.92129745
nonDEG	0.23392415	0.60650029	1	nonDEG	-0.1670108	0.68397753
nonDEG	1.30067856	0.05328562	1	nonDEG	-1.1592736	0.01106242
nonDEG	-0.3366741	0.42072107	1	nonDEG	-0.1720793	0.63204864
nonDEG	-0.3510765	0.41674346	1	nonDEG	0.2832005	0.44607862
nonDEG	0.16810453	0.76849523	1	nonDEG	-0.2497784	0.5674061
nonDEG	0.9303107	0.03890019	1	nonDEG	-0.3800813	0.29435522
nonDEG	-1.3878277	0.02521815	1	nonDEG	1.76318037	0.02135375
nonDEG	-1.4515037	0.1512691	1	nonDEG	-0.242711	0.91392114
nonDEG	1.00222047	0.19847867	1	nonDEG	-0.1019255	0.89533756
nonDEG	1.49468367	0.09741167	1	nonDEG	0.44273411	0.5095376
nonDEG	-0.4086358	0.3784572	1	nonDEG	0.11583693	0.76921169
nonDEG	-0.1932017	0.67087079	1	nonDEG	-0.4750052	0.22236949
nonDEG	0.3972941	0.42507969	1	nonDEG	0.15257497	0.7247147
nonDEG	-0.6886817	0.14323003	1	nonDEG	0.3504674	0.38121343
nonDEG	1.58204874	0.02137794	1	nonDEG	-0.2718723	0.6701989
nonDEG	-1.1340624	0.09385657	1	nonDEG	0.12250811	0.86360635
nonDEG	-2.0933065	0.01632724	0.91787008	nonDEG	2.46680514	0.01771528
nonDEG	0.34879772	0.43488107	1	nonDEG	-0.3250429	0.40065293
nonDEG	-0.8184975	0.0913744	1	nonDEG	0.41872738	0.35886776
nonDEG	0.24404588	0.56199503	1	nonDEG	0.12394593	0.72778692
nonDEG	0.69841517	0.66643251	1	nonDEG	-0.0944426	0.94129453
nonDEG	2.02573092	0.00014175	0.20864629	nonDEG	-0.9001414	0.06031074
nonDEG	-0.0041969	0.99274086	1	nonDEG	-0.4274487	0.28646918
nonDEG	-2.0825099	0.0227659	1	nonDEG	1.20327308	0.33470035
nonDEG	0.80990104	0.49805801	1	nonDEG	0.62645776	0.58462294
nonDEG	-0.6102399	0.21206696	1	nonDEG	0.54361679	0.22673787
nonDEG	-0.2132981	0.63499928	1	nonDEG	-0.1528916	0.69840396
nonDEG	-0.2676789	0.53867543	1	nonDEG	-0.2201082	0.54502411
nonDEG	1.11951807	0.01272968	0.85364221	nonDEG	-0.0459483	0.9001993
nonDEG	-1.8500902	0.00945148	0.79482907	nonDEG	1.43666001	0.06141037
nonDEG	0.48819919	0.66008647	1	nonDEG	0.17279352	0.89845736
nonDEG	-0.3594067	0.46575136	1	nonDEG	0.44462277	0.29234437
nonDEG	0.25834134	0.63847657	1	nonDEG	0.53125125	0.3419408
nonDEG	-0.351753	0.64584623	1	nonDEG	1.3221405	0.06085826
nonDEG	2.02902066	0.26319079	1	nonDEG	-0.0724887	0.98245977
nonDEG	0.96191499	0.10822023	1	nonDEG	-0.1491881	0.77078035
nonDEG	-1.7376205	0.11579823	1	nonDEG	1.37806401	0.25715873
nonDEG	-0.6998554	0.15532691	1	nonDEG	-0.239605	0.69683393
nonDEG	-0.5728808	0.17038691	1	nonDEG	0.26258062	0.48051867

nonDEG	-0.0356306	0.93539772	1	nonDEG	0.13215726	0.72559892
nonDEG	-0.335583	0.4517493	1	nonDEG	-0.2852499	0.46417679
nonDEG	-0.4646664	0.32010003	1	nonDEG	-0.3904826	0.37473623
nonDEG	-1.770811	0.02543791	1	nonDEG	1.16457803	0.15592278
nonDEG	-0.508924	0.25526294	1	nonDEG	0.23114252	0.52925409
nonDEG	-0.4146265	0.65192084	1	nonDEG	0.9073198	0.29397866
nonDEG	0.18890485	0.66473765	1	nonDEG	-0.1549305	0.66813544
nonDEG	0.34251502	0.53404775	1	nonDEG	-0.1799363	0.72508004
nonDEG	4.77440113	0.01102488	0.84185458	nonDEG	-1.8590077	0.22320842
nonDEG	-0.3467471	0.44276993	1	nonDEG	-0.0506777	0.89476194
nonDEG	-0.3618103	0.46902963	1	nonDEG	-0.3520489	0.40576864
nonDEG	-0.9493403	0.30392627	1	nonDEG	0.1202109	0.90224499
nonDEG	-0.3979082	0.3697468	1	nonDEG	-0.2769267	0.50609441
nonDEG	-1.2883757	0.16798007	1	nonDEG	2.12857301	0.01795498
nonDEG	1.34015365	0.01384791	0.88049374	nonDEG	-0.1860931	0.68796094
nonDEG	-0.30083	0.47700712	1	nonDEG	-0.4637108	0.20276616
nonDEG	-0.6680874	0.30368926	1	nonDEG	0.9090861	0.19360115
nonDEG	0.43065381	0.3539404	1	nonDEG	-0.127005	0.76042144
nonDEG	0.87182944	0.09953904	1	nonDEG	-0.3746686	0.33387802
nonDEG	-3.2764771	0.00188268	0.61198441	nonDEG	2.42381345	0.00058672
nonDEG	-0.1767891	0.84504691	1	nonDEG	-1.268279	0.20594466
nonDEG	1.12262091	0.03929438	1	nonDEG	-0.2005925	0.69276236
nonDEG	-0.9258063	0.06530363	1	nonDEG	0.42121751	0.32622601
nonDEG	1.25541611	0.00339179	0.65691312	nonDEG	-0.8836152	0.01749531
nonDEG	-0.7726437	0.15753372	1	nonDEG	0.673535	0.17699105
nonDEG	2.04904	0.00252873	0.61776954	nonDEG	-0.0955364	0.8283546
nonDEG	0.28754455	0.50948229	1	nonDEG	-0.3176819	0.41094098
nonDEG	-0.5091172	0.27549995	1	nonDEG	0.20086466	0.59275125
nonDEG	-0.5239452	0.37409324	1	nonDEG	1.08552005	0.06138042
nonDEG	-0.0195439	0.96552907	1	nonDEG	-0.0209332	0.95440338
nonDEG	-1.6736503	0.13488474	1	nonDEG	2.24904206	0.06332889
nonDEG	-2.8796852	0.01134359	0.84185458	nonDEG	1.37170097	0.19823883
nonDEG	0.52374577	0.49175215	1	nonDEG	-0.635269	0.25640132
nonDEG	-1.2593778	0.01612926	0.91521859	nonDEG	1.04652864	0.05665774
nonDEG	-0.253058	0.59611875	1	nonDEG	-0.4694979	0.23821371
nonDEG	-0.1454447	0.74516698	1	nonDEG	0.28546386	0.468175
nonDEG	2.31627288	0.0303606	1	nonDEG	-2.1165637	0.0501321
nonDEG	-0.4781567	0.27938783	1	nonDEG	-0.0126669	0.97453817
nonDEG	0.74767655	0.11304545	1	nonDEG	-0.1298073	0.72331726
nonDEG	-0.1171852	0.78780172	1	nonDEG	0.3976816	0.31861171
nonDEG	0.28706148	0.51775774	1	nonDEG	0.00829309	0.98302261
nonDEG	0.06715305	0.90413043	1	nonDEG	0.20159552	0.69495354
nonDEG	1.29640411	0.11730626	1	nonDEG	-0.0096755	0.99284978

nonDEG	0.48007656	0.31148991	1	nonDEG	-0.1558271	0.69201752
nonDEG	-0.3380795	0.4327486	1	nonDEG	0.18122205	0.61804187
nonDEG	-1.2720759	0.13508582	1	nonDEG	1.90264814	0.05854945
nonDEG	-1.9309658	0.01643118	0.91787008	nonDEG	2.75705169	0.00153229
nonDEG	0.47750168	0.52976439	1	nonDEG	-0.0097224	0.99448477
nonDEG	0.44516428	0.30807046	1	nonDEG	-0.4188959	0.26393003
nonDEG	-0.1664043	0.69799063	1	nonDEG	0.28225001	0.49910576
nonDEG	1.91137371	0.08455751	1	nonDEG	-2.1084648	0.03653927
nonDEG	-0.8006058	0.10608948	1	nonDEG	0.9870527	0.09172176
nonDEG	0.35597532	0.43123655	1	nonDEG	-0.391147	0.28510687
nonDEG	0.28533035	0.56976034	1	nonDEG	0.40021316	0.37214618
nonDEG	-0.1510815	0.7994275	1	nonDEG	0.18547078	0.75153552
nonDEG	0.23209742	0.65631341	1	nonDEG	-0.6472295	0.15245827
nonDEG	-0.1640156	0.69859915	1	nonDEG	-0.3302601	0.3644113
nonDEG	-1.4968807	0.05664194	1	nonDEG	1.30077603	0.09658469
nonDEG	1.56537717	0.02262862	1	nonDEG	-0.5530778	0.39255068
nonDEG	1.73893777	0.09030128	1	nonDEG	-0.5511251	0.59466123
nonDEG	-0.4787749	0.26148268	1	nonDEG	0.60643041	0.14220105
nonDEG	1.43743966	0.07871599	1	nonDEG	-0.5898253	0.46419855
nonDEG	0.40462165	0.39189667	1	nonDEG	-0.1825225	0.62334841
nonDEG	-0.266994	0.80305875	1	nonDEG	0.78919175	0.46735227
nonDEG	0.36387278	0.47828761	1	nonDEG	-0.4335985	0.26550599
nonDEG	-1.2027933	0.06477201	1	nonDEG	1.64078757	0.00989086
nonDEG	-2.660219	0.0770725	1	nonDEG	3.32578191	0.02618452
nonDEG	0.53552505	0.29630378	1	nonDEG	-0.1202985	0.76492242
nonDEG	0.99025195	0.19415642	1	nonDEG	-0.0889747	0.89676196
nonDEG	0.29570378	0.87744814	1	nonDEG	0.73266206	0.62839533
nonDEG	1.15310421	0.00962959	0.79482907	nonDEG	0.10497729	0.78614755
DEG	0.31854669	0.71643774	1	nonDEG	1.15533901	0.09565645
nonDEG	-0.369396	0.42050388	1	nonDEG	0.93822106	0.01400052
nonDEG	-0.3068994	0.58832415	1	nonDEG	-0.1850964	0.7603675
nonDEG	0.14385146	0.81821349	1	nonDEG	0.57621297	0.30627579
nonDEG	3.1164262	0.00053804	0.44242691	nonDEG	-1.6898821	0.04832105
nonDEG	-0.4097424	0.37070584	1	nonDEG	0.17421045	0.67938782
nonDEG	1.63017433	0.04910227	1	nonDEG	-0.1744448	0.81725539
nonDEG	0.50486094	0.43868743	1	nonDEG	-0.7494384	0.09092697
nonDEG	-0.11536	0.82901804	1	nonDEG	-0.7798191	0.13571704
nonDEG	0.90557492	0.12296105	1	nonDEG	0.13483275	0.79187576
nonDEG	0.94569155	0.03104722	1	nonDEG	-0.4102199	0.27842351
nonDEG	1.85327616	0.02256918	1	nonDEG	-0.8800947	0.11952791
nonDEG	1.39532185	0.02032211	0.98886386	nonDEG	-1.7525911	6.77E-06
nonDEG	0.21724558	0.62579678	1	nonDEG	-0.000899	0.99896461
nonDEG	-0.8395924	0.21628985	1	nonDEG	1.07826531	0.08501975

nonDEG	-1.2961929	0.03305699	1	nonDEG	1.4187471	0.02949144
nonDEG	-0.6941858	0.2335275	1	nonDEG	0.74877342	0.16567603
nonDEG	0.55463736	0.45835963	1	nonDEG	0.52838045	0.36745032
nonDEG	1.82951347	0.07550367	1	nonDEG	-1.4717532	0.13031602
nonDEG	-0.2576487	0.58102488	1	nonDEG	-0.3177635	0.50839192
nonDEG	0.58000555	0.2118804	1	nonDEG	-0.3918623	0.34184315
nonDEG	-1.4665747	0.21596111	1	nonDEG	1.04321094	0.41835541
nonDEG	-0.0059822	0.99011868	1	nonDEG	-0.2813424	0.5227212
nonDEG	-0.0310528	0.95549095	1	nonDEG	-0.0472957	0.92538348
nonDEG	0.80995944	0.08137746	1	nonDEG	-0.5870975	0.17265528
nonDEG	1.12493579	0.05221874	1	nonDEG	-0.8126751	0.13761121
nonDEG	-0.8550389	0.19455802	1	nonDEG	0.30895487	0.65898707
nonDEG	-0.1376867	0.76526555	1	nonDEG	0.01242296	0.97694927
nonDEG	2.00321721	0.01979751	0.97952777	nonDEG	-1.8952608	0.01677228
nonDEG	-0.5777244	0.40064534	1	nonDEG	0.44065604	0.55449997
nonDEG	-0.9796635	0.12309081	1	nonDEG	1.22137269	0.04165602
nonDEG	1.00261687	0.10489192	1	nonDEG	-0.5811956	0.19381865
nonDEG	0.23022692	0.87128021	1	nonDEG	0.33973002	0.75100996
nonDEG	0.15586044	0.76462356	1	nonDEG	0.49536051	0.25625028
nonDEG	-1.3116355	0.05597318	1	nonDEG	0.25883771	0.66375315
nonDEG	-1.1719159	0.25868022	1	nonDEG	1.47274042	0.20605626
nonDEG	-1.3650551	0.13344897	1	nonDEG	1.08538068	0.16762694
nonDEG	0.5851765	0.19249225	1	nonDEG	-0.0988467	0.79726128
nonDEG	-1.6547232	0.00692744	0.75126991	nonDEG	0.41906439	0.47951544
nonDEG	0.42179364	0.34941309	1	nonDEG	0.01735012	0.96402396
nonDEG	-0.8227268	0.27361062	1	nonDEG	1.10683688	0.15963388
nonDEG	-1.0173847	0.04167729	1	nonDEG	1.03547767	0.02833025
nonDEG	0.21594085	0.69281803	1	nonDEG	-0.2804995	0.56485508
nonDEG	1.04326132	0.31451005	1	nonDEG	0.75991734	0.44477852
nonDEG	0.9638647	0.05588883	1	nonDEG	-0.3611033	0.40060713
nonDEG	-1.2278671	0.08800938	1	nonDEG	0.3284687	0.67769519
nonDEG	-1.6384151	0.02655018	1	nonDEG	1.93422681	0.02555803
nonDEG	2.02449811	0.00927074	0.79328284	nonDEG	-1.447609	0.01091453
nonDEG	0.30176979	0.60703747	1	nonDEG	0.40392337	0.44760094
nonDEG	-0.7686968	0.5291723	1	nonDEG	0.43207174	0.76911345
nonDEG	0.30966034	0.48384332	1	nonDEG	-0.4058524	0.30832102
nonDEG	-1.287403	0.09202875	1	nonDEG	1.62774148	0.03544021
nonDEG	-0.4121866	0.36256427	1	nonDEG	0.2743184	0.46536539
nonDEG	0.48330577	0.30506095	1	nonDEG	-0.1182966	0.7429546
nonDEG	-0.1299111	0.76461469	1	nonDEG	0.86849027	0.02175409
nonDEG	0.2076353	0.73741578	1	nonDEG	-0.2736163	0.65543329
nonDEG	-0.7448823	0.43361246	1	nonDEG	0.59859469	0.52081156
nonDEG	-0.4134763	0.34887327	1	nonDEG	0.22960955	0.52829235

nonDEG	-0.2803039	0.52452408	1	nonDEG	-0.5131255	0.17661088
nonDEG	-0.1786138	0.68477529	1	nonDEG	0.33385448	0.40452925
nonDEG	-0.1799903	0.71449445	1	nonDEG	-0.1374811	0.7676553
nonDEG	-0.7990139	0.77578128	1	nonDEG	0.35517604	1
nonDEG	-0.7549486	0.19614632	1	nonDEG	0.67550489	0.10342488
nonDEG	-0.7523782	0.10830696	1	nonDEG	0.52457024	0.19769066
nonDEG	-0.0197369	0.96515803	1	nonDEG	-0.3466633	0.37750232
nonDEG	0.82054683	0.1681726	1	nonDEG	-0.2951423	0.58721031
nonDEG	0.26307142	0.59093446	1	nonDEG	-0.2650148	0.48944345
nonDEG	-1.2756503	0.01632369	0.91787008	nonDEG	0.68792291	0.16721175
nonDEG	0.95368481	0.29618645	1	nonDEG	-2.4807045	3.43E-06
nonDEG	-0.5915031	0.36400191	1	nonDEG	0.23423472	0.72860383
nonDEG	-0.356275	0.47243228	1	nonDEG	0.32031177	0.46629999
nonDEG	1.14756794	0.09486459	1	nonDEG	-1.1493844	0.02342663
nonDEG	-0.1889855	0.66301131	1	nonDEG	-0.0387766	0.91484483
nonDEG	0.41382093	0.37503411	1	nonDEG	-0.1200867	0.7710488
nonDEG	-1.0001235	0.36969098	1	nonDEG	1.63107525	0.13730428
nonDEG	0.28549474	0.53420792	1	nonDEG	-0.0840296	0.83735442
nonDEG	0.55446147	0.36186579	1	nonDEG	-0.2289442	0.67891403
nonDEG	-0.6505215	0.17081468	1	nonDEG	0.74914335	0.13979413
nonDEG	-0.7861413	0.10823198	1	nonDEG	0.75011115	0.0843075
nonDEG	-0.3966896	0.49559182	1	nonDEG	0.35638013	0.46203066
nonDEG	-1.593276	0.18178783	1	nonDEG	1.46238998	0.20478073
nonDEG	1.40706276	0.00334884	0.65691312	nonDEG	-0.5876212	0.14884626
nonDEG	-0.5077175	0.25506209	1	nonDEG	0.72563298	0.0598065
nonDEG	-0.3593528	0.40611631	1	nonDEG	-0.3369093	0.36356529
nonDEG	4.24567397	0.35366379	1	nonDEG	1.23475753	0.55928426
nonDEG	0.6527186	0.51016908	1	nonDEG	-0.3114763	0.78488512
nonDEG	0.11411506	0.79498784	1	nonDEG	-0.2075808	0.56868541
nonDEG	1.00781256	0.25621981	1	nonDEG	-1.169019	0.13554702
nonDEG	-0.654998	0.67876187	1	nonDEG	1.65637258	0.21849039
nonDEG	0.91501086	0.03135156	1	nonDEG	-0.1215838	0.73835732
nonDEG	0.62607849	0.48344444	1	nonDEG	0.6263478	0.45573183
nonDEG	-0.9901502	0.19324764	1	nonDEG	1.81298589	0.04179375
nonDEG	0.79278813	0.19338341	1	nonDEG	-0.4274386	0.41626755
nonDEG	-0.1805136	0.9047008	1	nonDEG	-0.2776651	0.8415434
nonDEG	-0.8637921	0.07008866	1	nonDEG	0.51280315	0.19596824
nonDEG	0.62472054	0.19749174	1	nonDEG	0.02447503	0.95428596
nonDEG	-1.5019925	0.01875058	0.95501418	nonDEG	0.47918785	0.45361736
nonDEG	-0.6437565	0.51925121	1	nonDEG	1.26852647	0.15513353
nonDEG	0.16699695	0.73490856	1	nonDEG	0.02564969	0.94355587
nonDEG	-0.8930655	0.48805469	1	nonDEG	-0.6503789	0.75413919
nonDEG	-0.5183672	0.25325948	1	nonDEG	0.36930338	0.330773

nonDEG	1.39547124	0.04225775	1	nonDEG	-0.6862203	0.28480228
nonDEG	0.63627688	0.60059014	1	nonDEG	-2.0339919	0.25410275
nonDEG	-0.3711867	0.5444498	1	nonDEG	-0.0758841	0.90731283
nonDEG	0.04440955	0.93202052	1	nonDEG	0.64728636	0.16515597
nonDEG	0.75782521	0.1382861	1	nonDEG	0.22254405	0.64136785
nonDEG	-0.2318225	0.60509454	1	nonDEG	0.02736457	0.94134976
nonDEG	-0.3698941	0.41449593	1	nonDEG	0.64318082	0.11687075
nonDEG	0.25897217	0.60583626	1	nonDEG	-0.8697226	0.04120257
nonDEG	1.02030735	0.05263851	1	nonDEG	-0.952314	0.04932842
nonDEG	-0.2903977	0.56812267	1	nonDEG	-0.8313998	0.0698313
nonDEG	0.67154542	0.24975094	1	nonDEG	-0.0826185	0.883448
nonDEG	-0.9528132	0.24558296	1	nonDEG	1.05405417	0.21203564
nonDEG	0.82304299	0.1386292	1	nonDEG	0.33695568	0.48299971
nonDEG	-0.2822835	0.5065275	1	nonDEG	-0.4675976	0.21870489
nonDEG	0.59598636	0.46665648	1	nonDEG	-0.6812946	0.425958
nonDEG	-0.5117658	0.22990108	1	nonDEG	0.01082818	0.97749146
nonDEG	0.51141523	0.3161443	1	nonDEG	0.08508328	0.8243881
nonDEG	-0.7105072	0.48324633	1	nonDEG	0.32982712	0.78838881
nonDEG	0.88023714	0.26022098	1	nonDEG	-0.7931861	0.31642179
nonDEG	-0.9604971	0.10166962	1	nonDEG	0.69360942	0.17856977
nonDEG	-0.1945203	0.7049133	1	nonDEG	0.3289066	0.52730348
nonDEG	-0.605629	0.22297675	1	nonDEG	0.46875753	0.3209309
nonDEG	-0.9639482	0.04994917	1	nonDEG	0.19920329	0.6730757
nonDEG	-0.2149152	0.65006735	1	nonDEG	0.68186246	0.09777502
nonDEG	-0.9846464	0.37220486	1	nonDEG	0.87828047	0.60095279
nonDEG	1.08240303	0.01201961	0.85229819	nonDEG	-0.5909608	0.10590186
nonDEG	0.14599546	0.82032702	1	nonDEG	-0.5197505	0.43112181
nonDEG	0.7005355	0.12705838	1	nonDEG	-0.3789874	0.32337226
nonDEG	-0.0226688	0.96773499	1	nonDEG	0.45919625	0.26073093
nonDEG	-0.7356529	0.11304606	1	nonDEG	0.72044874	0.08980103
nonDEG	0.45067282	0.30394339	1	nonDEG	0.21599077	0.55460646
nonDEG	-0.1442188	0.73784511	1	nonDEG	-0.1763162	0.62916987
nonDEG	-0.3581524	0.39373629	1	nonDEG	-0.5930789	0.09924199
nonDEG	0.35514395	0.60509606	1	nonDEG	-0.6376348	0.18016781
nonDEG	-0.1991909	0.65167787	1	nonDEG	-0.5530789	0.14134248
nonDEG	-0.2168366	0.6415573	1	nonDEG	0.56863162	0.18818385
nonDEG	0.32999484	0.46992658	1	nonDEG	0.08549153	0.82306724
nonDEG	-0.3269391	0.4602739	1	nonDEG	0.68244461	0.13765496
nonDEG	0.63002169	0.20225392	1	nonDEG	-0.2004798	0.59009323
nonDEG	-1.5346592	0.00602856	0.73793371	nonDEG	0.36481346	0.52168958
nonDEG	0.36458888	0.39559002	1	nonDEG	-0.1306795	0.71961971
nonDEG	-0.3794885	0.55632821	1	nonDEG	0.63832059	0.30979133
nonDEG	1.35791857	0.07822967	1	nonDEG	-1.1112691	0.00790592

nonDEG	0.56954706	0.1871599	1	nonDEG	0.00361843	0.99255803
nonDEG	-7.3111475	0.01370375	0.87892073	nonDEG	7.40792191	0.00145863
nonDEG	-1.1239702	0.08353977	1	nonDEG	0.36069337	0.47675556
nonDEG	-0.6828451	0.23059308	1	nonDEG	0.17964919	0.74956172
nonDEG	0.00917576	0.98374448	1	nonDEG	-0.1580438	0.67264975
nonDEG	0.5886772	0.44031949	1	nonDEG	-0.1288882	0.85789461
nonDEG	-0.4690351	0.27486355	1	nonDEG	0.05268669	0.88882297
nonDEG	0.39910003	0.44507253	1	nonDEG	-0.1710209	0.6971176
nonDEG	-0.1423031	0.73289472	1	nonDEG	-0.572776	0.1585277
nonDEG	1.22866749	0.01678148	0.9251498	nonDEG	0.03263878	0.94466476
nonDEG	0.65909772	0.18121981	1	nonDEG	0.42064674	0.2503227
nonDEG	0.202515	0.67152355	1	nonDEG	0.23288465	0.52618612
nonDEG	0.67097868	0.12714954	1	nonDEG	-0.2747529	0.45780765
nonDEG	-0.1837768	0.67729218	1	nonDEG	-0.1166199	0.75197376
nonDEG	0.51520773	0.22864864	1	nonDEG	-0.2237951	0.54311911
nonDEG	-0.6626333	0.14923699	1	nonDEG	0.37323745	0.34195517
nonDEG	1.36301182	0.01665376	0.92156012	nonDEG	-0.5958854	0.18335341
nonDEG	-0.286363	0.51496503	1	nonDEG	-0.0147826	0.97194204
nonDEG	-0.6272464	0.21317178	1	nonDEG	0.32346668	0.48244735
nonDEG	-0.3399885	0.42230896	1	nonDEG	-0.8047925	0.03193874
nonDEG	-0.2570301	0.55627317	1	nonDEG	0.2366658	0.5344168
nonDEG	0.97913109	0.07752664	1	nonDEG	-0.3086051	0.48767849
nonDEG	-1.6063496	0.16450837	1	nonDEG	2.03312893	0.10456165
nonDEG	-0.3490527	0.66237223	1	nonDEG	0.80497894	0.3752894
nonDEG	0.34552318	0.45205731	1	nonDEG	-0.2501864	0.49973833
nonDEG	0.3334043	0.60041235	1	nonDEG	-0.4964118	0.29052915
nonDEG	-0.675518	0.20183378	1	nonDEG	-0.4799017	0.34905122
nonDEG	0.36347091	0.42799123	1	nonDEG	0.142871	0.72376841
nonDEG	1.13600724	0.10421226	1	nonDEG	-1.1118347	0.06809275
nonDEG	-2.4566945	0.07843217	1	nonDEG	2.88760718	0.02823435
nonDEG	-1.2894969	0.05400392	1	nonDEG	1.26996585	0.05045605
nonDEG	0.75571601	0.16998267	1	nonDEG	0.00897604	0.9823452
nonDEG	0.42587502	0.35593948	1	nonDEG	-0.2102371	0.58546738
nonDEG	0.62545136	0.22867326	1	nonDEG	-0.0737587	0.85036695
nonDEG	-0.3816823	0.388631	1	nonDEG	0.60671797	0.12518563
nonDEG	-0.164712	0.71149938	1	nonDEG	-0.4785405	0.19746923
nonDEG	0.31472353	0.54935262	1	nonDEG	-0.0680293	0.89693394
nonDEG	-0.3740694	0.40665336	1	nonDEG	-0.2419606	0.51916313
nonDEG	-0.7217041	0.24088258	1	nonDEG	0.68276431	0.38843997
nonDEG	0.61805771	0.196175	1	nonDEG	0.02546255	0.9528163
nonDEG	-1.9528742	0.19668754	1	nonDEG	1.08424116	0.6443996
nonDEG	-0.6446358	0.1482716	1	nonDEG	0.80072131	0.06408125
nonDEG	0.96681173	0.02808434	1	nonDEG	-0.4084604	0.33014381

nonDEG	-2.283701	0.06626841	1	nonDEG	1.35380086	0.30844913
nonDEG	1.35830446	0.01430145	0.88752292	nonDEG	-0.8302977	0.02946457
nonDEG	0.40874826	0.36458324	1	nonDEG	-0.2236398	0.56805611
nonDEG	0.26649526	0.54209434	1	nonDEG	-0.4847842	0.1949122
nonDEG	1.51658552	0.04243398	1	nonDEG	-1.103974	0.02977779
nonDEG	0.08604255	0.84711527	1	nonDEG	0.0678592	0.85642662
nonDEG	-0.639152	0.22687428	1	nonDEG	-0.1026274	0.82230548
nonDEG	-0.7276212	0.11413335	1	nonDEG	0.70137508	0.07233386
nonDEG	-0.4379822	0.36477452	1	nonDEG	-0.9323874	0.06027896
nonDEG	-1.0028121	0.11237496	1	nonDEG	1.08021629	0.07242527
nonDEG	-0.8757663	0.16251081	1	nonDEG	0.84854759	0.09750506
nonDEG	1.75428635	0.00306934	0.65006007	nonDEG	-0.3889721	0.37842674
nonDEG	-0.7166986	0.15953517	1	nonDEG	1.08780177	0.04059773
nonDEG	1.73099775	0.07151522	1	nonDEG	1.16667777	0.16386401
nonDEG	-0.6862461	0.28058515	1	nonDEG	0.74351034	0.18282382
nonDEG	-0.5002373	0.64830954	1	nonDEG	1.19464485	0.30361687
nonDEG	-0.3297307	0.50117619	1	nonDEG	0.28495446	0.5228018
nonDEG	1.55143903	0.04909977	1	nonDEG	-0.2091228	0.70432984
nonDEG	-0.07153	0.86827228	1	nonDEG	-0.4593114	0.23056318
nonDEG	-1.0613366	0.10543257	1	nonDEG	1.441271	0.01407476
nonDEG	0.86181218	0.09869429	1	nonDEG	-0.3230746	0.37208605
nonDEG	-0.1611324	0.81198317	1	nonDEG	0.85527338	0.16540071
nonDEG	-0.46687	0.3043305	1	nonDEG	-0.3055424	0.42239073
nonDEG	-0.844479	0.15212994	1	nonDEG	0.14130831	0.80474261
nonDEG	-0.2978637	0.50044332	1	nonDEG	-0.4580269	0.24630829
nonDEG	-1.6217008	0.00743816	0.75263895	nonDEG	2.09338726	0.00439391
nonDEG	-2.7011768	0.02859668	1	nonDEG	2.61312148	0.01702734
nonDEG	1.26119347	0.03605402	1	nonDEG	-0.2134456	0.62709534
nonDEG	0.54188903	0.27224643	1	nonDEG	-0.8705232	0.02307726
nonDEG	0.34568314	0.58628468	1	nonDEG	0.11055684	0.86946493
nonDEG	0.71598368	0.10367807	1	nonDEG	-0.6042591	0.09619313
nonDEG	-0.4804273	0.27490418	1	nonDEG	0.66646367	0.08257155
nonDEG	-1.4567351	0.20760742	1	nonDEG	0.89945962	0.48842464
nonDEG	-0.49279	0.2798665	1	nonDEG	0.21801488	0.55974938
nonDEG	1.28734933	0.0151131	0.89340262	nonDEG	-0.5946918	0.15497618
nonDEG	-0.485006	0.27686298	1	nonDEG	-0.1732749	0.63287676
nonDEG	-0.3132511	0.45795202	1	nonDEG	-0.3523919	0.35502464
nonDEG	0.20085617	0.75611649	1	nonDEG	0.23772696	0.67853947
nonDEG	0.87498703	0.08071692	1	nonDEG	-0.179217	0.6387296
nonDEG	0.95930798	0.17978242	1	nonDEG	0.07517533	0.90361747
nonDEG	-0.5334905	0.23698247	1	nonDEG	0.47071064	0.29236707
nonDEG	-0.6553729	0.40638931	1	nonDEG	0.6313675	0.47103251
nonDEG	0.30959701	0.49744908	1	nonDEG	-0.0034513	0.99506556

nonDEG	0.26440229	0.53611444	1	nonDEG	0.00395355	0.99207664
nonDEG	0.7511374	0.40370349	1	nonDEG	0.59754043	0.40818446
nonDEG	0.55418969	0.30707888	1	nonDEG	0.38844098	0.4011081
nonDEG	0.69899038	0.16186505	1	nonDEG	-0.5783848	0.19210144
nonDEG	-0.4441218	0.29784725	1	nonDEG	-0.0621548	0.8650822
nonDEG	0.38100482	0.48214043	1	nonDEG	-0.161766	0.67655458
nonDEG	0.19982273	0.64056904	1	nonDEG	0.00571209	0.98803751
nonDEG	-0.3430523	0.43047884	1	nonDEG	-0.5486694	0.13822922
nonDEG	-1.4449082	0.05362187	1	nonDEG	0.17878722	0.78567235
nonDEG	0.92250783	0.04442998	1	nonDEG	-0.2137142	0.60692413
nonDEG	1.40802882	0.30195041	1	nonDEG	-1.532084	0.05746841
nonDEG	-2.5064687	0.03504007	1	nonDEG	2.07235766	0.14730056
nonDEG	-1.1610727	0.07435805	1	nonDEG	1.06527888	0.11358075
nonDEG	0.32456929	0.52845817	1	nonDEG	-0.5099753	0.17816211
nonDEG	-0.3380705	0.43731017	1	nonDEG	0.07069261	0.84961101
nonDEG	1.02955207	0.16876741	1	nonDEG	-0.8709722	0.1058308
nonDEG	-1.7406202	0.23784833	1	nonDEG	1.90729656	0.20834271
nonDEG	-0.5067096	0.23845294	1	nonDEG	0.6260633	0.12007751
nonDEG	0.47714981	0.62659765	1	nonDEG	-1.9250707	0.0273155
nonDEG	2.44437294	0.18225217	1	nonDEG	2.80708922	0.04718293
nonDEG	0.4067144	0.34592747	1	nonDEG	-0.1745475	0.632893
nonDEG	-0.5062278	0.56931639	1	nonDEG	1.18729984	0.21833849
nonDEG	0.99014348	0.04346564	1	nonDEG	-0.4938789	0.18075773
nonDEG	0.46673918	0.28763639	1	nonDEG	-0.2451477	0.50633818
nonDEG	2.2345835	0.03275961	1	nonDEG	-0.2323574	0.82558476
nonDEG	0.59782304	0.3279376	1	nonDEG	-0.0958276	0.87137516
nonDEG	0.1801935	0.72677432	1	nonDEG	-0.4144583	0.30992272
nonDEG	-0.096099	0.82435618	1	nonDEG	-0.2122296	0.57276344
nonDEG	0.80879907	0.11215989	1	nonDEG	-0.2303336	0.59460529
nonDEG	-0.6301375	0.19121583	1	nonDEG	0.05770717	0.90086081
nonDEG	0.84930857	0.07277358	1	nonDEG	0.11015171	0.76879702
nonDEG	-0.1960279	0.65857697	1	nonDEG	-0.2853119	0.4493422
nonDEG	-2.1916289	0.0265436	1	nonDEG	1.49041833	0.12133544
nonDEG	-1.1657467	0.01781105	0.9394793	nonDEG	0.76100282	0.13788815
nonDEG	0.59070512	0.43716683	1	nonDEG	-0.2373737	0.72355862
nonDEG	0.26487993	0.57462769	1	nonDEG	-0.2827019	0.44615579
nonDEG	-1.0928715	0.07072231	1	nonDEG	0.7525442	0.1766156
nonDEG	-0.4927376	0.35016089	1	nonDEG	0.32905094	0.51370846
nonDEG	-1.8748507	0.0041538	0.68407511	nonDEG	1.59230384	0.00015818
nonDEG	0.60537759	0.45157693	1	nonDEG	0.39307555	0.61287816
nonDEG	-0.4444197	0.44275102	1	nonDEG	0.47159287	0.37185826
nonDEG	1.10030726	0.03455265	1	nonDEG	-0.25401	0.58722457
nonDEG	-0.4712965	0.29991919	1	nonDEG	-0.2256266	0.56515114

nonDEG	0.47119353	0.37775468	1	nonDEG	-0.3334577	0.48429012
nonDEG	0.01744759	0.9866377	1	nonDEG	0.08783311	0.89028222
nonDEG	0.13677009	0.78846127	1	nonDEG	-0.2242256	0.59040721
nonDEG	-0.7429397	0.2385292	1	nonDEG	0.87460997	0.1484043
nonDEG	1.61670946	0.03863412	1	nonDEG	-0.4830224	0.4666414
nonDEG	0.16671476	0.72211581	1	nonDEG	-0.1400175	0.73852917
nonDEG	0.84497159	0.10743832	1	nonDEG	-0.444032	0.32502674
nonDEG	-0.5804148	0.23527708	1	nonDEG	0.59888466	0.21207173
nonDEG	1.36323131	0.01479328	0.89056822	nonDEG	-0.7975751	0.11800013
nonDEG	1.51017559	0.07024112	1	nonDEG	-0.7623753	0.20986132
nonDEG	1.0638896	0.40786316	1	nonDEG	-0.1075402	0.95671612
nonDEG	-0.7114292	0.12372614	1	nonDEG	0.37479685	0.3698975
nonDEG	4.31410641	0.00012131	0.18795539	nonDEG	-2.0169973	0.0717538
nonDEG	1.27570965	0.0473079	1	nonDEG	-0.0898825	0.87836562
nonDEG	-0.7867098	0.10514619	1	nonDEG	0.67598156	0.11058837
nonDEG	0.73868712	0.14443119	1	nonDEG	-0.135422	0.7675092
nonDEG	0.31633429	0.60896212	1	nonDEG	0.03771039	0.95018068
nonDEG	0.13846001	0.8086247	1	nonDEG	-0.0999939	0.8525325
nonDEG	0.54620192	0.25570837	1	nonDEG	0.12838763	0.73289087
nonDEG	-0.9135758	0.1644569	1	nonDEG	-1.4761877	0.09617312
nonDEG	1.60823952	0.02578325	1	nonDEG	-1.3237758	0.0021372
nonDEG	0.0712824	0.89021354	1	nonDEG	0.49517069	0.2806058
nonDEG	1.06493523	0.09329067	1	nonDEG	-0.2425333	0.67742762
nonDEG	-0.8921838	0.18570732	1	nonDEG	0.73712348	0.28450927
nonDEG	1.7524106	0.06263339	1	nonDEG	0.29098982	0.7383074
nonDEG	-0.7482183	0.58369172	1	nonDEG	-0.2458016	0.92332136
nonDEG	-0.5129814	0.29152703	1	nonDEG	-0.1205957	0.79217129
nonDEG	-1.1302674	0.39114681	1	nonDEG	1.20939957	0.36156068
nonDEG	-0.7598404	0.11002886	1	nonDEG	0.47812818	0.1972045
nonDEG	0.56036424	0.18285001	1	nonDEG	-0.0699805	0.85037986
nonDEG	0.63666518	0.14660771	1	nonDEG	-0.4009303	0.27654593
nonDEG	-1.0118213	0.03937877	1	nonDEG	1.48833259	0.00071658
nonDEG	-0.4723718	0.40979182	1	nonDEG	1.38352752	0.00499499
nonDEG	0.52707418	0.30977745	1	nonDEG	0.16792905	0.71739825
nonDEG	-0.2912582	0.50046232	1	nonDEG	-0.514098	0.1579776
nonDEG	-0.7434747	0.62986579	1	nonDEG	0.88669575	0.52249343
nonDEG	-1.1884876	0.12088374	1	nonDEG	0.38074108	0.61978601
nonDEG	0.16635017	0.72881683	1	nonDEG	-0.1640192	0.67730032
nonDEG	0.44770465	0.50851964	1	nonDEG	-0.1601624	0.75811794
nonDEG	-0.148526	0.74521592	1	nonDEG	0.09827896	0.80119899
nonDEG	0.39517054	0.50088547	1	nonDEG	-0.3407168	0.39828328
nonDEG	-0.4719796	0.27781833	1	nonDEG	-0.171467	0.65439085
nonDEG	0.13264223	0.77773176	1	nonDEG	-0.1849311	0.61668843

nonDEG	1.84804735	0.0421097	1	nonDEG	-1.095776	0.21229982
nonDEG	0.65687391	0.13334385	1	nonDEG	0.20830934	0.58222639
nonDEG	-0.7540056	0.26291704	1	nonDEG	0.48992993	0.50798783
nonDEG	0.29172395	0.61718631	1	nonDEG	0.31252453	0.55595316
nonDEG	0.3658949	0.52998336	1	nonDEG	-0.6795585	0.15136615
nonDEG	0.42748494	0.34838861	1	nonDEG	-0.2817144	0.45096283
nonDEG	-0.0890494	0.89520727	1	nonDEG	-0.2176987	0.70117121
nonDEG	-0.8549912	0.18045032	1	nonDEG	0.77673436	0.12939421
nonDEG	1.34494224	0.02369383	1	nonDEG	0.23061394	0.62176435
nonDEG	0.43681791	0.4255203	1	nonDEG	-0.6050819	0.13795146
nonDEG	0.63228144	0.2788859	1	nonDEG	-0.8444709	0.06959928
nonDEG	0.07963668	0.86764046	1	nonDEG	0.15088652	0.71677572
nonDEG	1.41008407	0.2314276	1	nonDEG	-0.4434325	0.62427819
nonDEG	-0.4620037	0.28003915	1	nonDEG	0.43232769	0.24032259
nonDEG	-1.0875043	0.11877934	1	nonDEG	1.3458687	0.143526
nonDEG	0.90217135	0.17332972	1	nonDEG	0.0593466	0.9244113
nonDEG	0.02151708	0.96070018	1	nonDEG	-0.3255712	0.36365662
nonDEG	-1.7375347	0.04879057	1	nonDEG	1.05567378	0.18348861
nonDEG	0.37130271	0.4552468	1	nonDEG	-0.0420458	0.92027156
nonDEG	1.63337149	0.25504866	1	nonDEG	-1.1499815	0.42028118
nonDEG	2.87685993	0.01546086	0.90129159	nonDEG	-1.6997225	0.13633063
nonDEG	0.4775528	0.47919058	1	nonDEG	-1.2008849	0.13223695
nonDEG	-0.3026202	0.48629476	1	nonDEG	-0.1918087	0.60531312
nonDEG	1.07402009	0.06360689	1	nonDEG	0.18395811	0.70095823
nonDEG	-0.7548586	0.14250395	1	nonDEG	0.56536853	0.29964634
nonDEG	-0.9324656	0.0880856	1	nonDEG	0.5641134	0.27492488
nonDEG	-0.937054	0.17190956	1	nonDEG	0.83395717	0.20131156
nonDEG	0.62786609	0.37291123	1	nonDEG	-1.0114164	0.06795156
nonDEG	-2.2174859	0.0089561	0.79328284	nonDEG	0.74499029	0.30286406
nonDEG	-0.9984925	0.39814674	1	nonDEG	0.92386701	0.40864249
nonDEG	0.0397651	0.92868867	1	nonDEG	-0.2355473	0.54970119
nonDEG	-0.1375776	0.74413738	1	nonDEG	0.11056304	0.76058322
nonDEG	1.25403336	0.12292662	1	nonDEG	-0.3000379	0.62224919
nonDEG	-0.2704516	0.56748113	1	nonDEG	0.0286849	0.94439128
nonDEG	-1.2992262	0.00916304	0.79328284	nonDEG	0.58335599	0.21760467
nonDEG	-1.5893502	0.00772842	0.75581042	nonDEG	1.80026737	0.01189739
nonDEG	-0.4352957	0.30275688	1	nonDEG	0.21296094	0.56008294
nonDEG	0.7926851	0.11128912	1	nonDEG	-0.8546527	0.02002009
nonDEG	-2.6451316	0.05918091	1	nonDEG	2.33072596	0.3254124
nonDEG	0.91086328	0.1059866	1	nonDEG	0.09238742	0.85610795
nonDEG	-0.5701767	0.30194444	1	nonDEG	0.00962344	0.9892421
nonDEG	-1.50795	0.00618286	0.73793371	nonDEG	2.44770004	0.00067434
nonDEG	4.23094436	0.00071305	0.50181787	nonDEG	-0.9090112	0.38700824

nonDEG	0.44641505	0.44548018	1	nonDEG	-0.4734381	0.24715964
nonDEG	0.20404245	0.64922017	1	nonDEG	-0.0164398	0.96434239
nonDEG	1.26474149	0.14721184	1	nonDEG	-0.2589831	0.6818331
nonDEG	-0.3802239	0.42814751	1	nonDEG	-0.2896555	0.50283127
nonDEG	0.94762579	0.03390469	1	nonDEG	-0.2766374	0.46162437
nonDEG	0.03451415	1	1	nonDEG	-0.7730385	0.47966104
nonDEG	0.20393903	0.66566238	1	nonDEG	0.6959272	0.08546942
nonDEG	-0.411342	0.35003191	1	nonDEG	0.31077092	0.41346518
nonDEG	-2.1474171	0.20502836	1	nonDEG	0.0579623	1
nonDEG	-1.4364951	0.03524371	1	nonDEG	0.25869577	0.60279452
nonDEG	-0.5222763	0.38414628	1	nonDEG	-0.1764855	0.76566674
nonDEG	1.11819767	0.10482898	1	nonDEG	0.0390032	0.94783444
nonDEG	0.45546269	0.4471661	1	nonDEG	0.05492936	0.92066572
nonDEG	-0.4669985	0.409176	1	nonDEG	0.64374574	0.25049514
nonDEG	-0.2181366	0.60631475	1	nonDEG	-0.009471	0.97952563
nonDEG	-0.3254168	0.46733683	1	nonDEG	-0.380701	0.31814097
nonDEG	0.37210585	0.55597011	1	nonDEG	-0.6781397	0.1897764
nonDEG	1.34591545	0.00157235	0.5714603	nonDEG	-0.4196121	0.30917612
nonDEG	1.23230828	0.35918967	1	nonDEG	-1.0025723	0.39079345
nonDEG	0.87255589	0.08274811	1	nonDEG	-0.3469942	0.38508143
nonDEG	1.73608126	0.0018746	0.61198441	nonDEG	-0.5996839	0.23387365
nonDEG	0.72459175	0.18450773	1	nonDEG	-0.4020327	0.42985202
nonDEG	0.4370237	0.3869693	1	nonDEG	0.40295308	0.37444235
nonDEG	-0.5099011	0.25599344	1	nonDEG	0.27551928	0.45949162
nonDEG	-0.8392115	0.10258434	1	nonDEG	0.50479692	0.29272653
nonDEG	-0.2529277	0.55830003	1	nonDEG	0.22145986	0.54404399
nonDEG	-0.3171011	0.46904258	1	nonDEG	-0.006416	0.98612295
nonDEG	-1.4034407	0.03640939	1	nonDEG	1.50868103	0.03977764
nonDEG	0.29584545	0.49599063	1	nonDEG	0.17274382	0.63029196
nonDEG	0.31315955	0.57390004	1	nonDEG	-0.4708839	0.38131859
nonDEG	-0.5587234	0.21749952	1	nonDEG	0.19427343	0.62511506
nonDEG	-0.8867662	0.1942522	1	nonDEG	0.55666559	0.29443554
nonDEG	-0.7283402	0.14972544	1	nonDEG	0.40127492	0.35063156
nonDEG	-0.5805048	0.21190067	1	nonDEG	0.07519733	0.84545697
nonDEG	-1.7919578	0.04337263	1	nonDEG	1.83788655	0.0839556
nonDEG	-0.4410595	0.29801871	1	nonDEG	0.67799678	0.06317295
nonDEG	-0.3616818	0.63556407	1	nonDEG	0.10798086	0.88450067
nonDEG	-0.1860767	0.68354694	1	nonDEG	0.17143107	0.64989062
nonDEG	0.9618013	0.04501351	1	nonDEG	-0.212648	0.55753813
nonDEG	0.8486963	0.06086733	1	nonDEG	-0.4016734	0.32131375
nonDEG	-1.5786822	0.03229851	1	nonDEG	1.19931342	0.05043517
nonDEG	-0.5757288	0.60591463	1	nonDEG	1.64983709	0.11140811
nonDEG	0.6418566	0.15812505	1	nonDEG	-0.4681952	0.19894943

nonDEG	0.17865759	0.76105858	1	nonDEG	-0.4466225	0.41985271
nonDEG	1.10977138	0.11799215	1	nonDEG	-0.0017682	0.99673875
nonDEG	-0.7477536	0.11656571	1	nonDEG	0.34224399	0.34721581
nonDEG	0.15649101	0.71566023	1	nonDEG	0.06889778	0.85195869
nonDEG	-0.5577621	0.19326511	1	nonDEG	-0.4374778	0.25832202
nonDEG	-0.2788053	0.52321378	1	nonDEG	-0.3376755	0.35222475
nonDEG	-0.6442296	0.15819754	1	nonDEG	-0.0326066	0.93544537
nonDEG	-0.0353595	0.94640119	1	nonDEG	-0.2155799	0.67199716
nonDEG	0.66997054	0.15036086	1	nonDEG	-0.2569515	0.49865868
nonDEG	-0.393882	0.42865649	1	nonDEG	-0.4569071	0.30162378
nonDEG	0.55001986	0.38368345	1	nonDEG	-0.2585221	0.66979277
nonDEG	0.56229041	0.21836238	1	nonDEG	0.10870301	0.79825018
nonDEG	0.29810763	0.50913466	1	nonDEG	-0.1515411	0.70918818
nonDEG	0.19366475	0.68742516	1	nonDEG	-0.0520117	0.9055912
nonDEG	0.44382919	0.39400018	1	nonDEG	0.47488625	0.31211136
nonDEG	-0.3901224	0.405949	1	nonDEG	0.03434661	0.93459125
nonDEG	0.26043016	0.5459247	1	nonDEG	0.18682354	0.61186027
nonDEG	-1.4477482	0.04020377	1	nonDEG	1.66650037	0.0075974
nonDEG	-1.8669139	0.02065213	0.99114571	nonDEG	1.5851523	0.02853066
nonDEG	0.72760403	0.12192355	1	nonDEG	-0.1111551	0.78371253
nonDEG	2.56159192	9.96E-05	0.17256328	nonDEG	-0.2794393	0.54923694
nonDEG	-1.0409846	0.07263199	1	nonDEG	0.50701582	0.39813543
nonDEG	0.85738969	0.21576434	1	nonDEG	0.03337195	0.96391914
nonDEG	-0.4448421	0.29459609	1	nonDEG	0.16619605	0.65483686
nonDEG	-0.8436549	0.09136328	1	nonDEG	1.13152841	0.00178292
nonDEG	-1.3093632	0.08752256	1	nonDEG	0.23834818	0.75671878
nonDEG	0.92188137	0.16991167	1	nonDEG	-0.8592641	0.11638576
nonDEG	0.18458327	0.69782852	1	nonDEG	-0.0069875	0.98934531
nonDEG	-1.2776931	0.01816294	0.94303121	nonDEG	0.73246435	0.16578885
nonDEG	1.16583026	0.09238795	1	nonDEG	-0.2183212	0.73499582
nonDEG	0.309468	0.64763785	1	nonDEG	-0.4562664	0.49746292
nonDEG	0.24515188	0.7331462	1	nonDEG	-0.0492476	0.95466375
nonDEG	0.28003743	0.5650803	1	nonDEG	-0.3192597	0.38548524
nonDEG	2.94741441	0.00386962	0.67135178	nonDEG	-0.224955	0.77786449
nonDEG	-1.2446198	0.05475677	1	nonDEG	-0.1524239	0.85156449
nonDEG	-0.294152	0.52408269	1	nonDEG	-0.3082843	0.46638687
nonDEG	-0.0033185	0.99624907	1	nonDEG	0.26621999	0.55177217
nonDEG	-0.0192393	0.96503035	1	nonDEG	-0.1645822	0.64969644
nonDEG	1.07545521	0.07466515	1	nonDEG	-0.1953749	0.72309834
nonDEG	0.76451844	0.0957155	1	nonDEG	-1.0243758	0.00861722
nonDEG	-0.4894073	0.35131483	1	nonDEG	-0.0469942	0.91929638
nonDEG	-0.5482227	0.25500675	1	nonDEG	0.23208822	0.54577814
nonDEG	0.26463324	0.61092565	1	nonDEG	-0.0720536	0.85947257

nonDEG	0.45595388	0.33420963	1	nonDEG	-0.5202438	0.21695111
nonDEG	-0.1521541	0.72620402	1	nonDEG	0.07538762	0.84219539
nonDEG	0.32586661	0.5698922	1	nonDEG	-0.1353658	0.78841578
nonDEG	-0.4290052	0.34171411	1	nonDEG	0.26175656	0.50315988
nonDEG	1.40328546	0.06646752	1	nonDEG	0.00432919	0.99548535
nonDEG	-1.1045726	0.12848482	1	nonDEG	1.27607548	0.03857943
nonDEG	-2.0101893	0.08973656	1	nonDEG	2.90047968	0.03191215
nonDEG	-0.2399325	0.6807554	1	nonDEG	-0.33805	0.53533246
nonDEG	0.74417688	0.29561281	1	nonDEG	-1.0033858	0.17883671
nonDEG	0.68570619	0.15251373	1	nonDEG	-0.6665431	0.06741831
nonDEG	-0.0310924	0.94388074	1	nonDEG	-0.0237047	0.95063407
nonDEG	-1.3962024	0.02214127	1	nonDEG	0.68864673	0.15820862
nonDEG	-0.3853601	0.44360889	1	nonDEG	0.61695732	0.18740022
nonDEG	0.30239242	0.51011726	1	nonDEG	0.19771166	0.61656813
nonDEG	0.1173356	0.79885209	1	nonDEG	-0.0056955	0.98836509
nonDEG	1.33987585	0.03503788	1	nonDEG	-0.3409519	0.4435104
nonDEG	-0.6668118	0.15068823	1	nonDEG	-0.1433672	0.72088033
nonDEG	-0.6862473	0.17815557	1	nonDEG	0.95502419	0.08406184
nonDEG	-0.3797713	0.46920999	1	nonDEG	-0.3018665	0.47182538
nonDEG	0.48843319	0.28181764	1	nonDEG	0.05881446	0.87933287
nonDEG	0.31560528	0.70092591	1	nonDEG	-0.3746117	0.67858416
nonDEG	0.64095335	0.44035437	1	nonDEG	-1.163351	0.18130049
nonDEG	0.4491096	0.34862662	1	nonDEG	-0.306977	0.49628694
nonDEG	-0.3372978	0.44168644	1	nonDEG	-0.4898919	0.18656533
nonDEG	0.51880866	0.41247626	1	nonDEG	0.51578948	0.34571021
nonDEG	0.21845844	0.61203664	1	nonDEG	0.52212485	0.17574998
nonDEG	-0.6680922	0.13905827	1	nonDEG	0.21947266	0.55618954
nonDEG	0.4880921	0.29134188	1	nonDEG	-1.2668742	0.00136483
nonDEG	0.25351146	0.55999326	1	nonDEG	0.00739035	0.9840197
nonDEG	0.16045519	0.72427345	1	nonDEG	-0.4408316	0.25556477
nonDEG	0.52619293	0.35233441	1	nonDEG	0.04127848	0.91568684
nonDEG	0.83588937	0.23024076	1	nonDEG	0.43417393	0.44888778
nonDEG	1.47396951	0.03159951	1	nonDEG	-1.0003714	0.06305109
nonDEG	-0.8287562	0.12938567	1	nonDEG	0.53976263	0.27581633
nonDEG	-1.5055335	0.53645216	1	nonDEG	2.78843263	0.17731822
nonDEG	-0.9000642	0.21727333	1	nonDEG	1.29937364	0.07732703
nonDEG	1.51300546	0.19486797	1	nonDEG	-1.8528046	0.15024892
nonDEG	0.54207396	0.44087564	1	nonDEG	-0.028203	0.97349259
nonDEG	0.409479	0.38702831	1	nonDEG	-0.2750053	0.44994843
nonDEG	0.16868504	0.71278198	1	nonDEG	0.10483608	0.77474966
nonDEG	0.33208943	0.52899188	1	nonDEG	0.20007834	0.67017152
nonDEG	1.42647991	0.00591439	0.73793371	nonDEG	-0.1892529	0.69249122
nonDEG	2.11341543	0.23234305	1	nonDEG	0.94174207	0.53408874

nonDEG	-1.0473032	0.04032473	1	nonDEG	0.94366936	0.05714052
nonDEG	0.31815424	0.77440866	1	nonDEG	1.88900959	0.04392079
nonDEG	0.63150042	0.55520028	1	nonDEG	0.85389002	0.39108523
nonDEG	-1.5066518	0.01380855	0.87989141	nonDEG	1.98177488	0.00341845
nonDEG	-4.0207301	0.00267918	0.61776954	nonDEG	4.70710596	0.00062608
nonDEG	-0.4834049	0.29392853	1	nonDEG	0.49906228	0.25928579
nonDEG	0.59369386	0.24587804	1	nonDEG	-0.0240385	0.96005572
nonDEG	-4.1784626	0.00095994	0.51194803	nonDEG	3.2689604	0.00131994
nonDEG	-1.4179653	0.16680996	1	nonDEG	-0.7137442	0.57991721
nonDEG	-0.521384	0.26312753	1	nonDEG	-0.7652897	0.12120328
nonDEG	-1.8504243	0.0147362	0.89005739	nonDEG	1.91761098	4.81E-06
nonDEG	1.04974326	0.02802261	1	nonDEG	-0.4954458	0.24624044
nonDEG	-0.0878288	0.84092568	1	nonDEG	-0.3172912	0.39564683
nonDEG	0.37059892	0.45575527	1	nonDEG	0.19678623	0.60861398
nonDEG	0.6333018	0.24072573	1	nonDEG	-0.0859078	0.81325315
nonDEG	-2.2874759	0.16063842	1	nonDEG	-0.0190577	1
nonDEG	0.24803394	0.61365964	1	nonDEG	0.27760217	0.48082467
nonDEG	0.92708924	0.04358787	1	nonDEG	-0.3684067	0.35133548
nonDEG	-0.006603	0.98804523	1	nonDEG	0.26722012	0.50152047
nonDEG	1.03072484	0.20175059	1	nonDEG	-0.8689995	0.10581742
nonDEG	-0.6972138	0.46812719	1	nonDEG	-0.9743228	0.42221066
nonDEG	0.25008183	0.62715727	1	nonDEG	-0.1789679	0.65140857
nonDEG	1.76806313	0.00268317	0.61776954	nonDEG	-0.3847367	0.30253748
nonDEG	1.09478617	0.11831361	1	nonDEG	-0.0337269	0.93992924
nonDEG	0.0666151	0.90669727	1	nonDEG	-0.6939732	0.11577613
nonDEG	-0.1445041	0.7361817	1	nonDEG	-0.4299079	0.23771097
nonDEG	1.54088313	0.0571931	1	nonDEG	-1.3728287	0.09812179
nonDEG	2.81729026	0.00694218	0.75126991	nonDEG	-1.2933691	0.16833586
nonDEG	-0.3360698	0.46163505	1	nonDEG	-0.4189047	0.26571476
nonDEG	-1.660877	0.16641164	1	nonDEG	0.96025939	0.42021007
nonDEG	-0.6274544	0.24416806	1	nonDEG	0.30719424	0.54828843
nonDEG	-0.2723479	0.53913173	1	nonDEG	0.10310051	0.77559824
nonDEG	-0.2177208	0.81065939	1	nonDEG	-0.0577672	0.9558015
nonDEG	0.38486305	0.40566438	1	nonDEG	-0.1055898	0.78934367
nonDEG	0.6744613	0.17494316	1	nonDEG	-0.3548261	0.35119605
nonDEG	0.28989808	0.53213488	1	nonDEG	-0.2182864	0.55657254
nonDEG	0.15099635	0.79046122	1	nonDEG	-0.6808974	0.22769321
nonDEG	-0.1894452	0.78048675	1	nonDEG	1.33321168	0.01398123
nonDEG	-1.3551982	0.13458649	1	nonDEG	0.17388996	0.88266905
nonDEG	0.07143943	0.89623631	1	nonDEG	0.32737146	0.49938786
nonDEG	-0.5556313	0.19057966	1	nonDEG	0.53961401	0.13857808
nonDEG	-0.6315126	0.28541512	1	nonDEG	0.29289803	0.63263844
nonDEG	-1.4031627	0.0973086	1	nonDEG	1.42418153	0.08275

nonDEG	-2.2151103	0.02286884	1	nonDEG	2.29112452	0.0108607
nonDEG	-0.3175033	0.49356917	1	nonDEG	0.40740574	0.29392422
nonDEG	0.55618505	0.21133224	1	nonDEG	-0.0298856	0.93949795
nonDEG	0.01782203	0.97187571	1	nonDEG	0.37547491	0.44065526
nonDEG	-0.2851352	0.53619498	1	nonDEG	0.16130922	0.68697921
nonDEG	0.69178925	0.11951669	1	nonDEG	-0.4924374	0.21169356
nonDEG	-0.1833768	0.66948574	1	nonDEG	-0.1954863	0.59454782
nonDEG	0.72084668	0.09337668	1	nonDEG	-0.2179747	0.55010944
nonDEG	-0.5628154	0.23855378	1	nonDEG	0.89143369	0.02723386
nonDEG	-0.9427966	0.07788268	1	nonDEG	0.25655817	0.56334605
nonDEG	-0.0066267	0.98831494	1	nonDEG	0.20962793	0.57377087
nonDEG	0.51782689	0.25391135	1	nonDEG	-0.0223375	0.95395148
nonDEG	-0.5368035	0.41973504	1	nonDEG	-0.000785	1
nonDEG	0.64210124	0.20429517	1	nonDEG	-0.0456973	0.91236454
nonDEG	0.31441426	0.48604674	1	nonDEG	-0.1447182	0.69366102
nonDEG	-2.1728932	0.03424935	1	nonDEG	1.62513417	0.0935978
nonDEG	0.36684806	0.42079659	1	nonDEG	-0.6019925	0.12377156
nonDEG	-0.9941598	0.31927318	1	nonDEG	-0.081444	0.93876131
nonDEG	0.23053536	0.61478632	1	nonDEG	0.0878827	0.81877685
nonDEG	0.82560031	0.09270416	1	nonDEG	-0.0452358	0.90151533
nonDEG	0.42313281	0.47127418	1	nonDEG	0.08636766	0.85519473
nonDEG	-1.3052996	0.08885101	1	nonDEG	1.52123556	0.0253311
nonDEG	-1.5160999	0.08544755	1	nonDEG	0.35759434	0.66380726
nonDEG	0.18982902	0.6589454	1	nonDEG	0.08219939	0.82427655
nonDEG	-0.4097784	0.34286055	1	nonDEG	0.14148773	0.70597095
nonDEG	-0.6301128	0.29713396	1	nonDEG	0.29554737	0.60298375
nonDEG	-0.6890308	0.44378659	1	nonDEG	0.41714764	0.68961592
nonDEG	-0.1288605	0.76660526	1	nonDEG	-0.6236945	0.08757967
nonDEG	0.51042283	0.90403897	1	nonDEG	0.87034132	0.57808357
nonDEG	-0.5357469	0.45770869	1	nonDEG	-0.6674891	0.40207643
nonDEG	-1.4853892	0.05681565	1	nonDEG	1.51040025	0.03505787
nonDEG	0.26600284	0.56943943	1	nonDEG	0.53575473	0.18875088
nonDEG	0.27560885	0.51847117	1	nonDEG	-0.0296717	0.93630359
nonDEG	1.85591443	0.00046851	0.41795603	nonDEG	-0.4492444	0.27950985
nonDEG	-0.3393395	0.74058875	1	nonDEG	-1.1531447	0.21412867
nonDEG	-0.4896515	0.27231813	1	nonDEG	-0.2014463	0.59235696
nonDEG	1.20639851	0.04298046	1	nonDEG	-0.0339859	0.95362791
nonDEG	1.47863839	0.22003078	1	nonDEG	-0.8341683	0.42706693
nonDEG	0.94224102	0.15708504	1	nonDEG	0.28390976	0.61997634
nonDEG	1.13539149	0.01440511	0.88752292	nonDEG	0.34482722	0.35797377
nonDEG	2.16145398	0.0133716	0.86906821	nonDEG	-1.134511	0.14556337
nonDEG	-0.9157037	0.14182299	1	nonDEG	0.21234827	0.75195386
nonDEG	-0.6749843	0.16395267	1	nonDEG	-0.2354375	0.57708343

nonDEG	-0.6438873	0.25526878	1	nonDEG	-0.1916027	0.7033496
nonDEG	-0.3530753	0.40699514	1	nonDEG	0.75620061	0.0441544
nonDEG	-1.9755211	0.09613593	1	nonDEG	1.69827088	0.16564602
nonDEG	1.66466678	0.03472863	1	nonDEG	-0.195589	0.63779712
nonDEG	-1.0403022	0.10205693	1	nonDEG	0.70907708	0.26627866
nonDEG	-0.3868037	0.39822807	1	nonDEG	-0.3683694	0.37251062
nonDEG	1.18231563	0.06341588	1	nonDEG	-0.2909604	0.62160515
nonDEG	0.3732003	0.46664319	1	nonDEG	-0.5724765	0.21522598
nonDEG	-0.4698034	0.50103385	1	nonDEG	-0.1318028	0.85230352
nonDEG	-0.4652831	0.57441087	1	nonDEG	0.98328104	0.18307358
nonDEG	-0.5211553	0.21816402	1	nonDEG	0.16554725	0.6572604
nonDEG	2.26636824	0.00303248	0.64690646	nonDEG	-1.3635008	0.04957259
nonDEG	0.78265996	0.12134223	1	nonDEG	-0.4913951	0.22448776
nonDEG	-0.5382285	0.22038726	1	nonDEG	-0.5743517	0.12576201
nonDEG	0.96269682	0.03707504	1	nonDEG	-0.2782263	0.4518782
nonDEG	-0.3820306	0.37321251	1	nonDEG	-0.2029193	0.59225435
nonDEG	0.02345833	0.95872084	1	nonDEG	-0.4124428	0.254402
nonDEG	0.44954039	0.32731387	1	nonDEG	0.21750686	0.57237227
nonDEG	-0.2731745	0.53177726	1	nonDEG	0.06949598	0.85545947
nonDEG	0.9118153	0.24295975	1	nonDEG	0.05943131	0.92002686
nonDEG	1.10150679	0.06390907	1	nonDEG	-0.5047904	0.24175473
nonDEG	-0.1869521	0.67177163	1	nonDEG	0.21367842	0.57222367
nonDEG	-0.2911365	0.51468769	1	nonDEG	-0.1079415	0.78186739
nonDEG	0.91250492	0.109049	1	nonDEG	-0.0421771	0.93657623
nonDEG	0.32280821	0.45971298	1	nonDEG	-0.245137	0.49975091
nonDEG	-0.1195677	0.79828213	1	nonDEG	-0.0667829	0.86711235
nonDEG	-0.3520174	0.42318928	1	nonDEG	0.4641803	0.22949863
nonDEG	1.32698313	0.16853771	1	nonDEG	0.67528247	0.43831428
nonDEG	-0.9040346	0.16209451	1	nonDEG	0.94589851	0.07226141
nonDEG	-1.5745476	0.04185201	1	nonDEG	1.53548118	0.02310373
nonDEG	2.50199353	0.01171351	0.85229819	nonDEG	-0.5512713	0.54708697
nonDEG	0.23683877	0.58349325	1	nonDEG	0.03139278	0.93154291
nonDEG	-0.5757396	0.19794976	1	nonDEG	0.19924271	0.59659152
nonDEG	-4.3795747	0.20510516	1	nonDEG	-5.13E-15	1
nonDEG	-0.5388293	0.25704204	1	nonDEG	-0.3905066	0.4078477
nonDEG	-0.9973025	0.16240955	1	nonDEG	0.82295984	0.24802076
nonDEG	0.54813398	0.21541845	1	nonDEG	-0.0225389	0.95238879
nonDEG	-0.0075435	1	1	nonDEG	2.15645156	0.10031953
nonDEG	-0.553298	0.49677114	1	nonDEG	1.08002273	0.16243492
nonDEG	-1.3996546	0.09721547	1	nonDEG	1.60280988	0.05760633
nonDEG	0.10040604	0.83827461	1	nonDEG	0.0379497	0.92760835
nonDEG	0.61399111	0.15383349	1	nonDEG	-0.5766887	0.11714113
nonDEG	-0.4995949	0.26602304	1	nonDEG	0.38708873	0.31229599

nonDEG	-0.7016757	0.11293243	1	nonDEG	0.66173387	0.08768851
nonDEG	0.36285405	0.56789049	1	nonDEG	0.22564835	0.69979315
nonDEG	0.88725866	0.0788806	1	nonDEG	-0.3991648	0.28406756
nonDEG	0.41707955	0.36361458	1	nonDEG	0.1759318	0.62696312
nonDEG	0.37002472	0.48414167	1	nonDEG	0.01413043	0.97673904
nonDEG	0.21115492	0.69889278	1	nonDEG	-0.1266452	0.81387705
nonDEG	-0.4433556	0.35103634	1	nonDEG	-0.0972494	0.82798662
nonDEG	0.06681884	0.94298004	1	nonDEG	0.4084302	0.60200493
nonDEG	0.23676447	0.59389254	1	nonDEG	0.23668891	0.52399084
nonDEG	0.43323711	0.30537711	1	nonDEG	-0.4727346	0.20344005
nonDEG	-0.0043627	0.992349	1	nonDEG	-0.4156869	0.29416783
nonDEG	0.32032517	0.56276267	1	nonDEG	-0.6076921	0.25196184
nonDEG	-1.2615634	0.0265232	1	nonDEG	0.66858452	0.20387195
nonDEG	-0.1223898	0.78298506	1	nonDEG	-0.1596905	0.70185261
nonDEG	-1.0451397	0.04464082	1	nonDEG	1.14289859	0.05050861
nonDEG	-0.1728161	0.70453391	1	nonDEG	-0.4726897	0.22798003
nonDEG	0.50253328	0.27619204	1	nonDEG	-0.3195828	0.43072485
nonDEG	-1.167289	0.10697149	1	nonDEG	0.11503617	0.88169259
nonDEG	0.55869065	0.21908608	1	nonDEG	0.04036342	0.91971363
nonDEG	-0.0705667	0.8875867	1	nonDEG	0.30668543	0.4787236
nonDEG	0.67189901	0.14018477	1	nonDEG	-0.1742719	0.66390697
nonDEG	0.43555335	0.55931629	1	nonDEG	-0.8646397	0.31036899
nonDEG	0.22575092	0.63260484	1	nonDEG	0.01828871	0.96642361
nonDEG	1.07753657	0.11049037	1	nonDEG	-0.1522209	0.79783423
nonDEG	-0.4170864	0.32632081	1	nonDEG	-0.1357907	0.71530083
nonDEG	-0.1441338	0.75397959	1	nonDEG	-0.0785528	0.84139161
nonDEG	0.5092071	0.27518822	1	nonDEG	-0.1707497	0.64079463
nonDEG	1.18592912	0.15029239	1	nonDEG	-0.9617921	0.21722832
nonDEG	-1.1325391	0.08688547	1	nonDEG	0.14684467	0.83116172
nonDEG	0.52013334	0.44201727	1	nonDEG	-0.7762787	0.26874672
nonDEG	0.64848011	0.17818726	1	nonDEG	-0.2326727	0.52067808
nonDEG	0.91261573	0.16654933	1	nonDEG	-0.6962462	0.22500758
nonDEG	0.63860088	0.41805817	1	nonDEG	-0.7340442	0.2753363
nonDEG	0.20719659	0.74629635	1	nonDEG	-0.1334783	0.84547919
nonDEG	0.8171039	0.0659416	1	nonDEG	-0.2700687	0.49897335
nonDEG	-0.9587802	0.14222045	1	nonDEG	0.87901199	0.23366231
nonDEG	0.12031523	0.77909312	1	nonDEG	-0.1780595	0.63359396
nonDEG	-0.8053075	0.62037893	1	nonDEG	-0.2667448	0.92533114
nonDEG	1.27401866	0.09890373	1	nonDEG	-0.6921941	0.13707058
nonDEG	2.33460842	0.00131454	0.52845676	nonDEG	0.01669862	0.97827278
nonDEG	-0.2756243	0.52613749	1	nonDEG	-0.832335	0.03391433
nonDEG	-0.8555264	0.06460216	1	nonDEG	1.271336	0.00131189
nonDEG	-0.9299858	0.23792384	1	nonDEG	1.68861366	0.0368355

nonDEG	0.37191327	0.4753948	1	nonDEG	-0.7739559	0.04129328
nonDEG	-0.4149325	0.33992611	1	nonDEG	-0.7629521	0.05988278
nonDEG	-0.5705731	0.21569416	1	nonDEG	0.25889576	0.5494541
DEG	2.41095704	4.55E-06	0.03026052	DEG	-0.6000619	0.17513552
nonDEG	0.93353944	0.09142807	1	nonDEG	-0.5119138	0.22493088
nonDEG	-1.1261896	0.24492193	1	nonDEG	0.27197218	0.80799085
nonDEG	-0.4471014	0.31972932	1	nonDEG	0.57499959	0.28422517
nonDEG	0.48651281	0.29335773	1	nonDEG	0.02014208	0.95903499
nonDEG	0.93206336	1	1	nonDEG	-0.4105758	1
nonDEG	0.98687239	0.11553224	1	nonDEG	-1.0975079	0.07543974
nonDEG	0.29589537	0.51706224	1	nonDEG	-0.5993817	0.11230272
nonDEG	-2.0511929	0.03811406	1	nonDEG	1.03415249	0.31042427
nonDEG	0.05024192	0.91351987	1	nonDEG	-0.5672503	0.16157053
nonDEG	1.31129481	0.02537305	1	nonDEG	-0.4499065	0.36163435
nonDEG	0.65966678	0.83834992	1	nonDEG	-4.1272843	0.40821845
nonDEG	0.02387261	0.95763256	1	nonDEG	-0.0009127	0.99818134
nonDEG	0.06526785	0.89917967	1	nonDEG	-0.8345974	0.10572304
nonDEG	0.22541205	0.60190771	1	nonDEG	-0.1335704	0.72361396
nonDEG	1.0827026	0.09073186	1	nonDEG	-0.0374644	0.93250224
nonDEG	0.24039177	0.65267315	1	nonDEG	0.43506653	0.33342495
nonDEG	0.69579448	0.1735219	1	nonDEG	-0.2585897	0.55476197
nonDEG	0.33714659	0.53760643	1	nonDEG	-1.3058574	0.02090405
nonDEG	0	1	1	nonDEG	2.21604745	1
nonDEG	0.44180607	0.3768779	1	nonDEG	-0.0369058	0.93570146
nonDEG	-0.3120525	0.4799323	1	nonDEG	0.29544833	0.42733224
nonDEG	-0.6481895	0.25231265	1	nonDEG	-0.09715	0.88078561
DEG	-0.2723655	0.77048136	1	nonDEG	2.58652623	0.0016799
nonDEG	1.28857744	0.04363265	1	nonDEG	-0.0520164	0.9257766
nonDEG	2.04336847	0.02080669	0.99114571	nonDEG	-0.5838876	0.44812402
nonDEG	3.64372526	0.04531257	1	nonDEG	1.62804898	0.25505294
nonDEG	0.75185073	0.25108124	1	nonDEG	-0.4145529	0.28496441
nonDEG	-0.5890876	0.19802323	1	nonDEG	0.46696269	0.2759401
nonDEG	0.16661954	0.71312154	1	nonDEG	-0.3033895	0.4513928
nonDEG	0.25533145	0.62804161	1	nonDEG	0.19338037	0.60554051
nonDEG	0.07812218	0.86047333	1	nonDEG	-0.2543306	0.51878851
nonDEG	-0.3783575	0.37180492	1	nonDEG	-0.111073	0.76759527
nonDEG	0.41751159	0.32658804	1	nonDEG	0.00856178	0.98229784
nonDEG	0.33740878	0.45790196	1	nonDEG	-0.1180497	0.75292475
nonDEG	-2.4986812	0.0779013	1	nonDEG	1.09749836	0.50171271
nonDEG	-1.4967315	0.19035714	1	nonDEG	1.64855515	0.16260976
nonDEG	-1.0418021	0.12554223	1	nonDEG	0.81233463	0.29592827
nonDEG	0.64971608	0.15001219	1	nonDEG	0.12561307	0.75268201
nonDEG	-1.1389781	0.08712586	1	nonDEG	1.28998596	0.03493389

nonDEG	1.24287776	0.06731357	1	nonDEG	-0.4285072	0.42656811
nonDEG	-1.3720646	0.01263397	0.85364221	nonDEG	1.33276442	0.00507371
nonDEG	1.79864472	0.00457293	0.68407511	nonDEG	-0.4495847	0.35633212
nonDEG	1.18799996	0.01869199	0.95501418	nonDEG	-0.6801614	0.11630456
nonDEG	0.10518117	0.80929917	1	nonDEG	0.26008028	0.4703908
nonDEG	0.73596892	0.11280382	1	nonDEG	-0.2462651	0.50400179
nonDEG	-1.5512911	0.01849622	0.95194095	nonDEG	1.38329886	0.0146829
nonDEG	1.63917556	0.10964549	1	nonDEG	-0.6980689	0.25838905
nonDEG	0.57657027	0.29427865	1	nonDEG	-0.3289854	0.43453631
nonDEG	-0.0749247	0.85995384	1	nonDEG	0.06891702	0.8495833
nonDEG	1.68684042	0.01787114	0.9394793	nonDEG	-0.3952892	0.3562143
nonDEG	0.39098271	0.50586651	1	nonDEG	-0.3437565	0.4533292
nonDEG	-0.0572687	0.90679496	1	nonDEG	0.49338845	0.24251744
nonDEG	1.63422074	0.08149472	1	nonDEG	-0.341687	0.71646937
nonDEG	0.24017136	0.62735199	1	nonDEG	-0.3652563	0.3747574
nonDEG	1.13593213	0.06229803	1	nonDEG	-0.7150503	0.1099346
nonDEG	-0.1149988	0.84661317	1	nonDEG	-0.010303	0.98832532
nonDEG	-0.8840339	0.09127403	1	nonDEG	0.48405979	0.25873752
nonDEG	-0.4781784	0.30076417	1	nonDEG	0.71732809	0.06460503
nonDEG	-1.0266384	0.49338314	1	nonDEG	-0.5022923	0.80464842
nonDEG	-0.658337	0.38449441	1	nonDEG	0.83960075	0.30911847
nonDEG	0.15065921	0.85961148	1	nonDEG	-1.3625773	0.10867152
nonDEG	-0.6655355	0.21957807	1	nonDEG	0.70656385	0.14927519
nonDEG	-0.6321756	0.30148462	1	nonDEG	-1.4467528	0.01360233
DEG	5.95330106	0.01785406	0.9394793	nonDEG	0.91516816	0.51109021
nonDEG	0.73933276	0.32502354	1	nonDEG	-0.9668913	0.0284359
nonDEG	0.1210227	0.79096367	1	nonDEG	-0.1294237	0.74528718
nonDEG	-0.1155118	0.79385306	1	nonDEG	0.04570711	0.90895746
nonDEG	-0.4266648	0.48687588	1	nonDEG	0.79663721	0.2310073
nonDEG	-0.3171565	0.47045275	1	nonDEG	0.14596133	0.71813106
nonDEG	0.60593782	0.17416806	1	nonDEG	0.06560126	0.86400476
nonDEG	1.05188181	0.03200788	1	nonDEG	-1.4008562	0.000399
nonDEG	-0.2920799	0.62167909	1	nonDEG	0.41622741	0.47390178
DEG	1.40803625	0.01291943	0.8619311	nonDEG	-0.2587087	0.61247608
nonDEG	0.17604286	0.69128551	1	nonDEG	0.04440809	0.90398225
nonDEG	-2.2386726	0.00738065	0.75263895	nonDEG	2.76709198	0.00656175
nonDEG	-1.5738325	0.12557362	1	nonDEG	1.84086659	0.09559497
nonDEG	0.06344784	0.91934838	1	nonDEG	0.66792606	0.21264127
nonDEG	5.20079063	0.05971194	1	nonDEG	-2.3732128	0.31948772
nonDEG	-0.7400782	0.11621977	1	nonDEG	-0.2125279	0.60140741
nonDEG	-0.9234592	0.09428253	1	nonDEG	1.53952318	0.01392227
nonDEG	0.71345708	0.2862353	1	nonDEG	0.64731589	0.2818521
nonDEG	0.64013568	0.37613024	1	nonDEG	-0.2386289	0.75671503

nonDEG	0.83879034	0.65590138	1	nonDEG	-0.6732089	0.74105282
nonDEG	0.58302593	0.20979882	1	nonDEG	-0.0936397	0.80581331
nonDEG	-2.7149327	0.03737061	1	nonDEG	2.50022783	0.02272565
nonDEG	-0.5119223	0.2346565	1	nonDEG	0.18996006	0.61722448
nonDEG	-1.5108714	0.06789795	1	nonDEG	2.00803586	0.02365682
nonDEG	-1.3797463	0.03475153	1	nonDEG	1.95337092	0.00278245
nonDEG	-0.0597623	0.89493625	1	nonDEG	-0.646752	0.09341405
nonDEG	-1.4939493	0.06122391	1	nonDEG	0.76464538	0.40430136
nonDEG	-0.0856751	0.88473026	1	nonDEG	0.10942417	0.84918751
nonDEG	1.4389827	0.14518151	1	nonDEG	-0.9467674	0.17895122
nonDEG	0.77065329	0.11670836	1	nonDEG	0.14277422	0.74832891
nonDEG	-0.0245066	0.95970233	1	nonDEG	-0.0614519	0.87250249
nonDEG	-0.6512233	0.66185418	1	nonDEG	2.17012049	0.12227293
nonDEG	1.27437002	0.53284634	1	nonDEG	-2.4221014	0.26417301
nonDEG	0.73623293	0.19702669	1	nonDEG	-0.2042626	0.65874688
nonDEG	-1.1226742	0.07889885	1	nonDEG	1.10673262	0.15685662
nonDEG	0.75278251	0.16728692	1	nonDEG	-0.5117451	0.28003732
nonDEG	1.20489797	0.06482021	1	nonDEG	0.1698848	0.71729294
nonDEG	0.79574486	0.24688424	1	nonDEG	-0.4183039	0.37211176
nonDEG	0.24360535	0.60263938	1	nonDEG	-0.5415466	0.14224666
nonDEG	-0.3377747	0.44424873	1	nonDEG	0.73871101	0.0660715
nonDEG	1.93035366	0.01795498	0.94060016	nonDEG	-0.7317915	0.2573404
nonDEG	-0.2643111	0.61342673	1	nonDEG	0.08836253	0.84116401
nonDEG	-0.5139974	0.27136795	1	nonDEG	0.40818306	0.33725609
nonDEG	-0.6341487	0.2041701	1	nonDEG	0.6365684	0.15144
nonDEG	-0.8553815	0.0490432	1	nonDEG	1.52691076	0.01249203
nonDEG	0.15841053	0.76021266	1	nonDEG	-0.5235605	0.25993961
nonDEG	0.49776427	0.44852441	1	nonDEG	-0.9886694	0.02959269
nonDEG	-0.0104181	0.98161189	1	nonDEG	-0.3593592	0.34436328
nonDEG	-0.0160694	0.97915543	1	nonDEG	0.13058757	0.78494404
nonDEG	-0.8995334	0.11362339	1	nonDEG	0.84981895	0.12572208
nonDEG	3.0569631	0.00644631	0.73793371	nonDEG	-0.4591936	0.6320698
nonDEG	-1.0934694	0.14734833	1	nonDEG	0.28472978	0.64692009
nonDEG	0.18210673	0.82321147	1	nonDEG	0.94202786	0.17269455
nonDEG	-1.2206164	0.01229423	0.85229819	nonDEG	0.48156194	0.25656951
nonDEG	1.27455622	0.25504205	1	nonDEG	-2.8522161	0.03746583
nonDEG	0.37399244	0.41708996	1	nonDEG	-0.6199492	0.14588563
nonDEG	0.30160093	0.49379616	1	nonDEG	-0.1660144	0.66027576
nonDEG	-0.690789	0.2837765	1	nonDEG	0.88813693	0.22506493
nonDEG	-0.4752531	0.272178	1	nonDEG	0.3050718	0.41077214
nonDEG	0.28715174	0.55940202	1	nonDEG	-0.3717535	0.36909816
nonDEG	0.56031631	0.19364255	1	nonDEG	-0.1822999	0.61774559
nonDEG	-0.2641686	0.54544648	1	nonDEG	0.12135251	0.73991026

DEG	-1.8748984	0.03083136	1	nonDEG	1.78701903	0.0353104
nonDEG	-1.2552866	0.1449824	1	nonDEG	0.86799346	0.31910999
nonDEG	0.9191779	0.04782789	1	nonDEG	0.09824401	0.79423143
nonDEG	-0.4327296	0.46731838	1	nonDEG	-1.1255295	0.04011585
nonDEG	-1.2888353	0.32841647	1	nonDEG	0.33986277	0.81050003
nonDEG	0.8851884	0.26710099	1	nonDEG	0.10029825	0.90177432
nonDEG	-0.5414149	0.3183939	1	nonDEG	-0.0991809	0.85602868
nonDEG	0.28642447	0.52366819	1	nonDEG	-0.1794698	0.65513325
nonDEG	-0.8789943	0.27859962	1	nonDEG	1.10483702	0.17169748
nonDEG	-0.0396646	0.95168505	1	nonDEG	-1.3071348	0.00410829
nonDEG	-0.2058809	0.73673087	1	nonDEG	0.90366401	0.1773588
nonDEG	-0.1003456	0.81180371	1	nonDEG	-0.1355729	0.71742372
nonDEG	1.13206501	0.29126038	1	nonDEG	0.81204171	0.43355882
nonDEG	-0.5545511	0.20766105	1	nonDEG	0.22228851	0.57573889
nonDEG	-0.1289589	0.76615876	1	nonDEG	-0.1225702	0.73954139
nonDEG	-2.4277145	0.00493573	0.70646871	nonDEG	3.42323827	0.00176684
nonDEG	-1.4415412	0.06104494	1	nonDEG	0.69691237	0.25244637
nonDEG	1.54064783	0.22092586	1	nonDEG	-0.9879289	0.44750954
nonDEG	0.51203669	0.25646848	1	nonDEG	0.12442934	0.74953711
nonDEG	0.88734436	0.16026509	1	nonDEG	-0.0032098	1
nonDEG	-0.1042711	0.83616121	1	nonDEG	0.62037373	0.19727593
nonDEG	0.36913689	0.44500099	1	nonDEG	-0.5130056	0.20554809
nonDEG	-0.6054725	0.25592466	1	nonDEG	-0.9313507	0.08906037
nonDEG	1.53451289	0.00228313	0.61776954	nonDEG	-1.3976526	0.00093649
nonDEG	-0.4568122	0.49606879	1	nonDEG	0.36959776	0.55366015
nonDEG	0.49277252	0.57418862	1	nonDEG	-0.2915166	0.72236976
nonDEG	-1.6075712	0.027145	1	nonDEG	1.93522055	0.01360963
nonDEG	0.49666361	0.46363718	1	nonDEG	-0.2405757	0.70485394
nonDEG	-0.1810701	0.68663175	1	nonDEG	0.122218	0.7664461
nonDEG	-0.3343726	0.43940844	1	nonDEG	0.02524475	0.94604127
nonDEG	0.50161335	0.24787202	1	nonDEG	-0.4253458	0.24507482
nonDEG	-1.3505215	0.06055789	1	nonDEG	1.6418861	0.02193784
nonDEG	1.2468416	0.00565102	0.73793371	nonDEG	-0.5454736	0.14192391
nonDEG	0.11170354	0.87742345	1	nonDEG	-0.754561	0.43437725
nonDEG	-0.4519053	0.3650506	1	nonDEG	0.11554251	0.80649634
nonDEG	0.53966171	0.22126721	1	nonDEG	-0.4794116	0.21835848
nonDEG	0.12079347	1	1	nonDEG	0.17104347	0.95734347
nonDEG	0.67554398	0.21562997	1	nonDEG	-1.6132707	0.00112816
nonDEG	0.44627299	0.30170471	1	nonDEG	0.060495	0.87093949
nonDEG	-0.2069333	0.63124559	1	nonDEG	-0.2070169	0.56516428
nonDEG	-0.0471071	0.91747336	1	nonDEG	0.17212185	0.67860035
nonDEG	3.34812592	0.00387682	0.67135178	nonDEG	0.82546676	0.37738644
nonDEG	-0.2248917	0.60148781	1	nonDEG	0.12586501	0.74500935

nonDEG	-0.8254959	0.12860108	1	nonDEG	0.82307847	0.14466036
nonDEG	0.33004322	0.46898853	1	nonDEG	-0.8168331	0.03235954
nonDEG	0.21844416	0.62114444	1	nonDEG	-0.072498	0.84393021
nonDEG	-0.2771873	0.51430766	1	nonDEG	-0.5896911	0.10270282
nonDEG	0.15863827	0.73394659	1	nonDEG	-0.0079436	0.98419327
nonDEG	-0.2178901	0.6456124	1	nonDEG	-0.1210317	0.7796007
nonDEG	-0.4976309	0.52082165	1	nonDEG	0.47723382	0.55646754
nonDEG	2.5081449	6.85E-05	0.14400617	nonDEG	-1.0434561	0.05755285
nonDEG	0.46147494	0.41640886	1	nonDEG	-0.0548232	0.89797365
DEG	1.14282081	0.09777635	1	nonDEG	0.16648784	0.77906653
nonDEG	0.70484234	0.34064916	1	nonDEG	-0.5125313	0.33862602
nonDEG	0.45854378	0.29129343	1	nonDEG	-0.1609878	0.66839279
nonDEG	-0.542497	0.24675891	1	nonDEG	0.07248244	0.85574518
nonDEG	-1.1601755	0.00959792	0.79482907	nonDEG	1.84608912	0.00319201
nonDEG	0.47530555	0.30530656	1	nonDEG	-0.3943682	0.34924097
nonDEG	-1.4113124	0.07614693	1	nonDEG	1.7742373	0.020819
nonDEG	-2.1008654	0.07289468	1	nonDEG	2.91392548	0.03030821
nonDEG	-0.4839848	0.24750324	1	nonDEG	0.57007121	0.12567287
nonDEG	1.79006971	9.73E-05	0.17256328	nonDEG	-0.3819965	0.30037936
nonDEG	1.0397926	0.10958138	1	nonDEG	-0.8683825	0.15365771
nonDEG	0.14750283	0.76185861	1	nonDEG	-0.9472534	0.0287269
nonDEG	0.11750511	0.79641848	1	nonDEG	0.06519609	0.85791783
nonDEG	-0.4088894	0.45035926	1	nonDEG	0.25298515	0.64634265
nonDEG	-0.9884289	0.48775273	1	nonDEG	0.6885845	0.65322711
nonDEG	0.66488421	0.13021358	1	nonDEG	-0.593872	0.102272
nonDEG	-0.2670083	0.5863389	1	nonDEG	0.63112841	0.17709843
nonDEG	0.46973628	0.30868942	1	nonDEG	-0.1633155	0.65884769
nonDEG	-0.0888997	0.84999963	1	nonDEG	0.63867098	0.15931257
nonDEG	0.0387101	0.93479156	1	nonDEG	-0.0419759	0.91129093
nonDEG	0.56365789	0.2052057	1	nonDEG	-0.1431205	0.70006758
nonDEG	1.42408282	0.3758122	1	nonDEG	-1.0203187	0.43775548
nonDEG	-0.2107467	0.7382115	1	nonDEG	-0.0921729	0.89464402
nonDEG	-0.7453496	0.13747143	1	nonDEG	-0.0105119	0.9878922
nonDEG	-2.7098223	0.02012571	0.98541756	nonDEG	2.13007174	0.0607816
nonDEG	-0.0810116	0.89727223	1	nonDEG	-0.1454605	0.83885924
nonDEG	-0.6542634	0.22460627	1	nonDEG	0.53442018	0.29026659
nonDEG	-0.3885693	0.36742585	1	nonDEG	-0.3942686	0.28123469
nonDEG	-0.4553851	0.31701159	1	nonDEG	0.19545975	0.66689545
nonDEG	-1.5799484	0.08538578	1	nonDEG	0.96041119	0.35894534
nonDEG	1.60686421	0.1542409	1	nonDEG	0.05575347	0.95259521
nonDEG	-0.55104	0.29139236	1	nonDEG	0.17196603	0.7291498
nonDEG	1.51620191	0.01912925	0.96926999	nonDEG	0.01794085	0.97626222
nonDEG	0.57698986	0.23652233	1	nonDEG	-0.3172756	0.37655491

nonDEG	0.73336691	0.2373531	1	nonDEG	-0.5408706	0.27964728
nonDEG	0.12516435	0.79925028	1	nonDEG	0.27496132	0.55985632
nonDEG	-3.3579362	0.02644004	1	nonDEG	3.56352993	0.00944298
nonDEG	1.43109376	0.15721549	1	nonDEG	-0.0792576	0.93574886
nonDEG	-0.5674428	0.19295661	1	nonDEG	0.2318225	0.53911823
nonDEG	-0.6522624	0.18197873	1	nonDEG	0.44303121	0.2571508
nonDEG	-1.1593496	0.09976384	1	nonDEG	1.28442548	0.08359862
nonDEG	-0.6374227	0.15465019	1	nonDEG	0.28557552	0.44562188
nonDEG	-1.9426805	0.1130441	1	nonDEG	2.12055648	0.12068818
nonDEG	0.57655831	0.28815983	1	nonDEG	-0.2855862	0.52936042
nonDEG	0.19752446	0.64583229	1	nonDEG	0.05209487	0.88717234
nonDEG	0.36008459	0.42616316	1	nonDEG	0.45462486	0.22061201
nonDEG	-0.425379	0.38411269	1	nonDEG	-0.3503553	0.41320526
nonDEG	-0.8261794	0.08632586	1	nonDEG	1.17938312	0.03676176
nonDEG	0.29690483	0.49729202	1	nonDEG	-0.1149748	0.75048488
nonDEG	1.31415871	0.50362355	1	nonDEG	-1.0431437	0.62207174
nonDEG	-1.1360086	0.21244138	1	nonDEG	1.96878964	0.03102876
nonDEG	1.06946984	0.14563938	1	nonDEG	-0.0720618	0.92101209
nonDEG	-0.0394724	0.92997617	1	nonDEG	0.14873579	0.70387101
nonDEG	-1.3858684	0.36439222	1	nonDEG	1.0828934	0.50840427
nonDEG	-1.0936538	0.02257434	1	nonDEG	1.70985091	0.00569242
nonDEG	0.29470785	0.62232857	1	nonDEG	0.05761482	0.90320296
nonDEG	-0.3032183	0.87101426	1	nonDEG	-1.0822394	0.51926098
nonDEG	0.51665547	0.23871164	1	nonDEG	-0.2344585	0.51803251
nonDEG	-0.3318944	0.46262845	1	nonDEG	-0.3402164	0.3733102
nonDEG	-0.358589	0.71438442	1	nonDEG	-1.3567801	0.11731072
nonDEG	0.57127319	0.23084297	1	nonDEG	-0.1720819	0.66283701
nonDEG	-0.9595386	0.47424383	1	nonDEG	0.27625641	0.85932458
nonDEG	-0.2821714	0.52747058	1	nonDEG	0.24717709	0.55144942
nonDEG	-0.3355126	0.47307806	1	nonDEG	-0.5069565	0.24842778
nonDEG	-1.3622478	0.12367513	1	nonDEG	0.20570419	0.89086439
nonDEG	-0.5272421	0.34366352	1	nonDEG	0.53579281	0.3400579
nonDEG	1.62745796	0.01473868	0.89005739	nonDEG	-0.3571276	0.48743265
nonDEG	1.70241256	0.01888936	0.96042141	nonDEG	0.01886554	0.97715148
nonDEG	2.05392398	0.02108264	0.99864599	nonDEG	0.60712079	0.43520825
nonDEG	0.50994429	0.29290093	1	nonDEG	-0.2210065	0.55583831
nonDEG	0.55511201	0.45126569	1	nonDEG	0.05421557	0.93889432
nonDEG	-1.4275704	0.06207502	1	nonDEG	1.53327577	0.06324581
nonDEG	-0.2917214	0.50869211	1	nonDEG	0.08027873	0.83693123
nonDEG	-0.6818245	0.12438093	1	nonDEG	0.55554315	0.13622649
nonDEG	0.30944371	0.56938055	1	nonDEG	-0.7175333	0.10252341
nonDEG	0.58748891	0.48133915	1	nonDEG	-0.5369278	0.20647736
nonDEG	0.508328	0.26518185	1	nonDEG	-0.0685634	0.8501155

nonDEG	0.56227247	0.2920148	1	nonDEG	-0.7370592	0.06886287
nonDEG	1.47005272	0.19045246	1	nonDEG	-1.2106886	0.248344
nonDEG	1.65571599	0.00028042	0.34458183	nonDEG	-0.9005964	0.02789008
nonDEG	0.61830617	0.14947391	1	nonDEG	-0.0734687	0.84256496
nonDEG	0.32372111	0.57701831	1	nonDEG	-0.4496715	0.40400357
nonDEG	-0.3140025	0.48809474	1	nonDEG	-0.3560132	0.42616137
nonDEG	0.46982719	0.42326735	1	nonDEG	-0.2488498	0.55226242
nonDEG	-0.8104517	0.08089567	1	nonDEG	-0.2163365	0.6251365
nonDEG	0.57593835	0.4017988	1	nonDEG	-0.437759	0.33758121
nonDEG	-0.3055539	0.49237287	1	nonDEG	-0.4099448	0.31318437
nonDEG	-0.0756054	0.8640883	1	nonDEG	-0.3539713	0.35678398
nonDEG	0	1	1	nonDEG	2.21584419	1
DEG	0.25734733	0.82394803	1	nonDEG	2.74552462	0.00854769
nonDEG	-0.9209449	0.07339914	1	nonDEG	0.90679966	0.08520078
nonDEG	-0.3223211	0.48430163	1	nonDEG	-0.476606	0.27479983
nonDEG	0.70147239	0.10784264	1	nonDEG	-0.2898647	0.45437675
nonDEG	0.54767712	0.66926196	1	nonDEG	0.39987723	0.71874083
nonDEG	0.03773487	0.96461539	1	nonDEG	-0.4339637	0.49029474
nonDEG	-0.3208278	0.47942394	1	nonDEG	-0.1850586	0.62390803
nonDEG	1.92726473	0.0023359	0.61776954	nonDEG	-1.2377775	0.00569017
nonDEG	-1.6041571	0.00749085	0.75263895	nonDEG	0.66672637	0.2305327
nonDEG	2.01411144	0.00235024	0.61776954	nonDEG	-0.1358584	0.75366384
nonDEG	0.19974429	0.73648926	1	nonDEG	-0.1716546	0.64333773
nonDEG	0.36700765	0.42404393	1	nonDEG	-0.0741688	0.83682642
nonDEG	0.88421493	0.075533	1	nonDEG	-0.3506297	0.36606217
nonDEG	0.43143107	0.32695099	1	nonDEG	0.15181963	0.67516699
nonDEG	2.15748862	0.20668038	1	nonDEG	-0.2410271	0.88163004
nonDEG	0.29322708	0.5689214	1	nonDEG	-0.1986608	0.6260155
nonDEG	-0.9407143	0.10576507	1	nonDEG	1.0544455	0.00931873
nonDEG	0.78307709	0.08267151	1	nonDEG	-0.5488851	0.13171274
nonDEG	0.56910113	0.317222	1	nonDEG	-0.0830272	0.86192722
nonDEG	0.36563918	0.62945608	1	nonDEG	-0.008523	0.99057993
nonDEG	-0.2738045	0.5197263	1	nonDEG	0.07215024	0.84433066
nonDEG	-0.0938003	0.8372353	1	nonDEG	-0.6692875	0.08551984
nonDEG	0.67904155	0.20067709	1	nonDEG	-0.4944169	0.32466445
nonDEG	2.27100733	0.00471272	0.69368911	nonDEG	-0.8743412	0.20797292
nonDEG	0.35248268	0.4358661	1	nonDEG	-0.1311415	0.71823438
nonDEG	-0.9571971	0.23949247	1	nonDEG	1.32609402	0.13429281
nonDEG	-0.2855242	0.50425715	1	nonDEG	-0.4731769	0.21733697
nonDEG	-1.3449169	0.01131727	0.84185458	nonDEG	1.83140026	0.00635793
nonDEG	0.01510189	0.97255374	1	nonDEG	0.11630398	0.75143962
nonDEG	-1.2873972	0.04766828	1	nonDEG	1.54245643	0.01450644
nonDEG	1.64462034	0.01344356	0.87172874	nonDEG	-0.7713991	0.2027461

nonDEG	-0.4135204	0.35427223	1	nonDEG	-0.21293	0.61990423
nonDEG	0.72571199	0.1383236	1	nonDEG	0.00351302	0.99509829
nonDEG	-0.5002192	0.26354196	1	nonDEG	0.11931724	0.7631816
nonDEG	-1.2728097	0.1567447	1	nonDEG	0.36800424	0.73456221
nonDEG	-0.0301358	0.96099633	1	nonDEG	-0.8272723	0.13432806
nonDEG	0.28144157	0.61487834	1	nonDEG	-0.5307421	0.21419879
nonDEG	-0.4097275	0.32669899	1	nonDEG	0.28211664	0.43047244
nonDEG	1.07637732	0.1730675	1	nonDEG	-0.8086469	0.28873411
nonDEG	0.63204068	0.20944312	1	nonDEG	-0.2581284	0.50618193
nonDEG	0.07411211	0.87770676	1	nonDEG	0.23922362	0.56427511
nonDEG	-0.5276506	0.26384484	1	nonDEG	0.01517614	0.97214763
nonDEG	0.63469852	0.13187254	1	nonDEG	0.00488547	0.99011242
nonDEG	1.4530554	0.01943463	0.97153571	nonDEG	-0.2639407	0.59408638
nonDEG	0.61614108	0.19118357	1	nonDEG	0.17108436	0.64877605
nonDEG	0.37711879	0.68115449	1	nonDEG	0.03550416	0.98017436
nonDEG	-0.6533002	0.18838259	1	nonDEG	0.17387909	0.67692996
nonDEG	-0.3385456	0.43864766	1	nonDEG	0.27472186	0.48355257
nonDEG	-0.6961287	0.26291124	1	nonDEG	0.46348718	0.44930986
nonDEG	0.30593957	0.68115631	1	nonDEG	0.08040867	0.91701041
nonDEG	0.52231249	0.21990524	1	nonDEG	-0.0030479	0.9937464
nonDEG	-0.3522244	0.4145197	1	nonDEG	0.09959584	0.78408147
nonDEG	0.30575214	0.68276439	1	nonDEG	0.05327845	0.92526467
nonDEG	-0.2561138	0.58095135	1	nonDEG	-0.0411843	0.92369883
nonDEG	-1.6835899	0.11220849	1	nonDEG	2.1212248	0.02945458
nonDEG	0.18554679	0.67816595	1	nonDEG	0.223728	0.56110078
nonDEG	0.23101784	0.60874797	1	nonDEG	0.08791836	0.81089836
nonDEG	-0.2702276	0.55253664	1	nonDEG	-0.3061842	0.43428188
nonDEG	-0.4660571	0.34618601	1	nonDEG	-0.2412824	0.56817588
nonDEG	0.28186658	0.51928619	1	nonDEG	0.01076888	0.97763194
nonDEG	1.46864154	0.19109242	1	nonDEG	0.69204456	0.50955125
nonDEG	-1.0480561	0.16985785	1	nonDEG	0.73658542	0.30594612
nonDEG	-0.3440112	0.41925903	1	nonDEG	-0.2475346	0.5026205
nonDEG	-0.4453645	0.32104106	1	nonDEG	0.50427935	0.18147035
nonDEG	1.75169816	0.00510118	0.71853359	nonDEG	-1.1256817	0.05315896
nonDEG	-0.2451317	0.5833912	1	nonDEG	-0.3592106	0.36207147
nonDEG	-0.2693985	0.59022635	1	nonDEG	-0.3147891	0.51618149
nonDEG	-0.1496878	0.74222423	1	nonDEG	-0.2905772	0.4529148
nonDEG	-0.3425053	0.4265002	1	nonDEG	-0.3216999	0.41595984
nonDEG	0.09377036	0.87469605	1	nonDEG	-0.136362	0.78747361
nonDEG	-0.6882892	0.13902204	1	nonDEG	0.68614186	0.08523964
nonDEG	-0.9642126	0.08135266	1	nonDEG	0.58826591	0.2236458
nonDEG	-0.2435218	0.59121339	1	nonDEG	0.08839606	0.83707433
nonDEG	2.27296014	0.00239043	0.61776954	nonDEG	-0.9495667	0.12748487

nonDEG	1.32717793	0.03242893	1	nonDEG	-0.3671488	0.49981979
nonDEG	1.41187959	0.03062233	1	nonDEG	-0.2791785	0.57379951
DEG	3.10681631	3.83E-05	0.12599308	nonDEG	-0.2194136	0.69384008
nonDEG	-0.4113	0.33493949	1	nonDEG	0.77864727	0.03496211
nonDEG	-0.549483	0.3185302	1	nonDEG	0.60803211	0.12559179
nonDEG	-0.1814752	0.66522001	1	nonDEG	0.21604408	0.54816079
nonDEG	-0.7494218	0.13878827	1	nonDEG	0.77910884	0.0444943
nonDEG	-2.7283629	0.00130728	0.52845676	nonDEG	2.72921528	3.65E-06
nonDEG	-0.6554314	0.16129451	1	nonDEG	0.24390222	0.55182841
nonDEG	1.93995951	0.09018245	1	nonDEG	-0.6812305	0.52195838
nonDEG	1.02740438	0.1253249	1	nonDEG	-0.2105852	0.722125
nonDEG	-2.9907373	0.00968168	0.79503069	nonDEG	2.82604715	0.00572158
nonDEG	0.36232927	0.59583531	1	nonDEG	-0.8926914	0.0462384
nonDEG	-1.617242	0.00996528	0.80027302	nonDEG	1.04191738	0.04693886
nonDEG	0.33373665	0.43485577	1	nonDEG	0.18857181	0.61352539
nonDEG	0.47314761	0.29995729	1	nonDEG	-0.7976546	0.04402144
nonDEG	0.33910561	0.53084073	1	nonDEG	-0.0485574	0.90987141
nonDEG	0.20317265	0.66635109	1	nonDEG	-0.1228222	0.74953014
nonDEG	0.09156635	0.8572727	1	nonDEG	-0.20075	0.6068147
nonDEG	0.40378661	0.44564455	1	nonDEG	-0.7553924	0.08566101
DEG	1.41396669	0.00145691	0.54987298	nonDEG	-0.3831423	0.30595063
nonDEG	0.52632885	0.27084665	1	nonDEG	-0.267054	0.47608972
nonDEG	0.84189956	0.30139215	1	nonDEG	-0.4383096	0.33450353
nonDEG	0.35871936	0.54798691	1	nonDEG	-0.6562367	0.15166894
nonDEG	-2.5768941	0.00655922	0.73793371	nonDEG	1.58044001	0.00492721
nonDEG	-0.3412793	0.42064157	1	nonDEG	-0.2461545	0.50212012
nonDEG	-0.5347292	0.23669165	1	nonDEG	0.07889969	0.84382474
nonDEG	-0.0555585	0.89869087	1	nonDEG	0.01983263	0.95965907
nonDEG	-0.3316381	0.68018128	1	nonDEG	0.31420589	0.7050408
nonDEG	1.18290587	0.10420224	1	nonDEG	-0.7532388	0.04785838
nonDEG	-0.3221311	0.45281219	1	nonDEG	-0.5127113	0.16030216
nonDEG	-0.2231726	0.60584392	1	nonDEG	0.01342372	0.97303078
nonDEG	0.14662866	0.73171987	1	nonDEG	0.1130431	0.75819858
nonDEG	-0.5338333	0.55272287	1	nonDEG	1.3419369	0.20149445
nonDEG	-0.252696	0.56471034	1	nonDEG	0.06764871	0.8596139
nonDEG	-0.9976256	0.0935166	1	nonDEG	1.01793081	0.13740817
nonDEG	-0.902365	0.07494674	1	nonDEG	0.03791719	0.93521601
nonDEG	-1.4793278	0.004296	0.68407511	nonDEG	1.2679452	0.01000983
nonDEG	-0.5534264	0.38816749	1	nonDEG	0.397733	0.52279471
nonDEG	0.08065702	0.86358276	1	nonDEG	0.24840205	0.54918135
nonDEG	0.89861728	0.19035749	1	nonDEG	0.25544236	0.67972774
nonDEG	0.33485403	0.48218937	1	nonDEG	-0.0024678	0.99492956
nonDEG	0.94420852	0.06923865	1	nonDEG	-0.2363862	0.57944377

nonDEG	-0.2746221	0.56070682	1	nonDEG	0.60804932	0.13631974
nonDEG	-1.0204194	0.04582785	1	nonDEG	0.77160704	0.04421311
nonDEG	0.74741752	0.10855549	1	nonDEG	-0.5000312	0.18846225
nonDEG	0.73669727	0.12982297	1	nonDEG	0.04011857	0.92427154
nonDEG	0.59172089	0.21983215	1	nonDEG	-0.3205138	0.39553255
nonDEG	0.08551319	0.86038507	1	nonDEG	0.09575546	0.7945634
nonDEG	0.7762283	0.10293723	1	nonDEG	-0.0925361	0.82675308
nonDEG	0.0685042	0.88269009	1	nonDEG	-0.4151278	0.30917385
nonDEG	0.52218785	0.45762	1	nonDEG	0.49764637	0.43201104
nonDEG	-1.7871115	0.00710044	0.75136371	nonDEG	2.33210923	0.002489
nonDEG	0.30367507	0.479529	1	nonDEG	0.05532062	0.88112136
nonDEG	-0.4580623	0.29888316	1	nonDEG	0.11246366	0.7913578
nonDEG	-0.1769772	0.68483089	1	nonDEG	0.10308638	0.78854622
DEG	0.01842786	0.97768687	1	nonDEG	0.97880259	0.10839104
nonDEG	-0.4498267	0.3307208	1	nonDEG	0.42907188	0.29249512
nonDEG	-0.7435495	0.09448455	1	nonDEG	0.12334957	0.73841729
nonDEG	0.46780863	0.40461553	1	nonDEG	0.18376009	0.69651256
nonDEG	-0.4428352	0.35865724	1	nonDEG	-0.2127028	0.58420831
nonDEG	-1.3010622	0.02623747	1	nonDEG	0.45304332	0.3889274
nonDEG	0.52818577	0.25394804	1	nonDEG	-0.4736381	0.23729039
nonDEG	-1.0716341	0.66214015	1	nonDEG	-0.191015	1
nonDEG	-0.3185088	0.46393996	1	nonDEG	0.19211399	0.63624134
nonDEG	0.43400146	0.35603473	1	nonDEG	0.13539534	0.7394665
nonDEG	-1.517029	0.08747758	1	nonDEG	1.27105397	0.17895175
nonDEG	-0.1482664	0.74885793	1	nonDEG	-0.0438484	0.91504523
nonDEG	-1.5316659	0.09537355	1	nonDEG	1.66635417	0.0319843
nonDEG	-0.6593747	0.29305934	1	nonDEG	1.29500009	0.04321685
nonDEG	-0.2187111	0.75641684	1	nonDEG	1.09713125	0.08230198
nonDEG	-0.7143297	0.12106522	1	nonDEG	0.57614374	0.19166244
nonDEG	0.61447065	0.20755653	1	nonDEG	-0.2163783	0.60484413
nonDEG	-1.2200965	0.10797462	1	nonDEG	1.02854232	0.11196091
nonDEG	-0.759859	0.07458427	1	nonDEG	0.17354535	0.65902999
nonDEG	-1.4242213	0.00257697	0.61776954	nonDEG	2.10818105	0.0005223
nonDEG	-0.9214852	0.49314439	1	nonDEG	0.65506905	0.71632096
nonDEG	0.66512389	0.14052693	1	nonDEG	-0.2212817	0.54562006
nonDEG	0.3074264	0.50217172	1	nonDEG	0.01124514	0.97727429
nonDEG	-1.6625595	0.03417974	1	nonDEG	0.52651143	0.36529756
nonDEG	0.76383525	0.11292127	1	nonDEG	-0.2023958	0.61168561
nonDEG	1.00308248	0.05906382	1	nonDEG	-0.1506346	0.73178066
nonDEG	-0.1854965	0.70919676	1	nonDEG	-0.2232329	0.61541532
nonDEG	0.57167501	0.32271877	1	nonDEG	0.30735175	0.5530609
nonDEG	1.1355078	0.02447131	1	nonDEG	-0.6147232	0.10272775
nonDEG	1.22919345	0.05970831	1	nonDEG	-0.7111794	0.14469751

nonDEG	0.39041932	0.42683161	1	nonDEG	-0.2993687	0.47166907
nonDEG	0.14025264	0.89050296	1	nonDEG	-0.317465	0.79397883
DEG	1.31884226	0.00461059	0.68407511	nonDEG	0.83743038	0.13951944
nonDEG	-0.2109417	0.63495407	1	nonDEG	-0.4380744	0.22416099
nonDEG	-0.7460734	0.17984142	1	nonDEG	0.79271455	0.0723776
nonDEG	-1.0473756	0.16430929	1	nonDEG	-1.0669606	0.05792968
nonDEG	-0.23298	0.73747845	1	nonDEG	0.54348588	0.48851526
nonDEG	0.73173381	0.48727447	1	nonDEG	0.05504814	0.96766965
nonDEG	-0.0984705	0.85373566	1	nonDEG	0.35708206	0.45139794
nonDEG	-0.0611763	0.88897177	1	nonDEG	-0.1521799	0.68809597
nonDEG	0.64158673	0.38138864	1	nonDEG	-1.3249311	0.07408993
nonDEG	-0.4381956	0.36755864	1	nonDEG	0.36580503	0.35316444
nonDEG	0.20874985	0.68292658	1	nonDEG	0.12763851	0.78421714
nonDEG	-1.5926991	0.34580188	1	nonDEG	-4.3848811	0.36692603
nonDEG	0.40056023	0.36052913	1	nonDEG	-0.2986799	0.41564081
nonDEG	0.65888576	0.15618049	1	nonDEG	-0.3834214	0.32588094
nonDEG	1.08649153	0.29703796	1	nonDEG	-0.4099771	0.72768196
nonDEG	-0.4578355	0.40046814	1	nonDEG	0.44838968	0.37426328
nonDEG	-0.2175363	0.61230306	1	nonDEG	0.1537005	0.6814407
nonDEG	-0.1926114	0.67426041	1	nonDEG	-0.050405	0.91363809
nonDEG	-0.7409136	0.13121562	1	nonDEG	0.35664924	0.33782055
nonDEG	0.902	0.03792382	1	nonDEG	-0.564994	0.12460057
nonDEG	0.92007399	0.08360477	1	nonDEG	-0.9708518	0.01778285
nonDEG	-0.4773133	0.4240306	1	nonDEG	0.7553667	0.17339065
nonDEG	-0.4141324	0.62799677	1	nonDEG	0.03394204	0.97155714
nonDEG	0.41595896	0.62931925	1	nonDEG	0.61642303	0.44384398
nonDEG	0.25085854	0.56197985	1	nonDEG	0.16749638	0.64773763
nonDEG	0.12732025	0.87565601	1	nonDEG	0.04956313	0.95832833
nonDEG	0.65369491	0.12698244	1	nonDEG	0.00129367	0.99785102
nonDEG	-0.2471262	0.66420123	1	nonDEG	0.47682633	0.27396885
nonDEG	0.49363682	0.37612434	1	nonDEG	0.10035435	0.79159152
nonDEG	-0.3846031	0.40409534	1	nonDEG	0.0305243	0.93846483
nonDEG	-1.3280031	0.01060295	0.82576758	nonDEG	1.94398742	0.00359302
nonDEG	-0.6548119	0.25630919	1	nonDEG	0.03680872	0.92987721
nonDEG	-1.4240472	0.1662758	1	nonDEG	1.73976629	0.12841082
nonDEG	-0.438352	0.30820442	1	nonDEG	0.46388833	0.21352133
nonDEG	1.14663207	0.03142449	1	nonDEG	0.0349275	0.94026544
nonDEG	-1.4949847	0.04178943	1	nonDEG	0.49449726	0.43423375
nonDEG	-0.3957343	0.36804349	1	nonDEG	0.16367153	0.68372961
nonDEG	0.50167027	0.42835633	1	nonDEG	-0.647808	0.18576209
nonDEG	-1.7246226	0.01279351	0.85597279	nonDEG	2.26107233	0.00089352
nonDEG	0.53756646	0.25266257	1	nonDEG	-0.2056412	0.61488166
nonDEG	0.80830617	0.18773714	1	nonDEG	-0.4303216	0.4301057

nonDEG	-1.3480467	0.02459833	1	nonDEG	1.18014274	0.05832444
nonDEG	-0.9454226	0.11350192	1	nonDEG	-0.001452	1
nonDEG	-0.2374105	0.59624597	1	nonDEG	-0.4207736	0.314575
nonDEG	-0.3068041	0.51458143	1	nonDEG	-0.320832	0.43941034
nonDEG	0.29739291	0.49593659	1	nonDEG	0.04715704	0.89654462
nonDEG	-2.9176084	0.01248065	0.85350212	nonDEG	2.49767868	0.02906858
nonDEG	0.95616161	0.1355796	1	nonDEG	0.61092762	0.23604029
nonDEG	0.62297291	0.21330076	1	nonDEG	-0.2301184	0.53409558
nonDEG	0.81175002	0.37234117	1	nonDEG	-0.178155	0.80706037
nonDEG	-0.7703766	0.36296811	1	nonDEG	0.72091648	0.39507855
nonDEG	-0.9409404	0.03195187	1	nonDEG	0.62349147	0.10345091
nonDEG	-2.1759218	0.02138843	1	nonDEG	1.90242242	0.08968602
nonDEG	-0.9121721	0.15028326	1	nonDEG	1.52659296	0.03174902
nonDEG	-0.862602	0.08002312	1	nonDEG	0.37325962	0.357831
nonDEG	-0.4524719	0.30037739	1	nonDEG	-0.599126	0.11638497
nonDEG	-0.0823724	0.87013117	1	nonDEG	0.09171939	0.84546986
nonDEG	1.13187967	0.04277353	1	nonDEG	-0.5544699	0.16460334
nonDEG	-0.1870065	0.66670879	1	nonDEG	-0.0875229	0.81962534
nonDEG	1.77948743	0.00377684	0.67010784	nonDEG	-0.2733694	0.62878441
nonDEG	-0.5325	0.30911118	1	nonDEG	0.59116503	0.18938869
nonDEG	-1.1746423	0.83903168	1	nonDEG	0.74197103	0.86030069
nonDEG	-1.1701594	0.17213574	1	nonDEG	1.85921102	0.01546997
nonDEG	-0.6797668	0.1589195	1	nonDEG	0.50689366	0.22725581
nonDEG	-0.797926	0.16978447	1	nonDEG	0.30231994	0.43924622
nonDEG	-1.2698093	0.01008035	0.80421551	nonDEG	0.22613411	0.56733237
nonDEG	0.88911861	0.05603489	1	nonDEG	-0.3440993	0.39619979
nonDEG	-0.4655365	0.30210337	1	nonDEG	-0.1694126	0.65225065
nonDEG	0.83234891	0.05997912	1	nonDEG	-0.4343541	0.24057126
nonDEG	0.28400292	0.51557589	1	nonDEG	-0.0556059	0.88160967
nonDEG	-0.5729488	0.18700468	1	nonDEG	0.23462926	0.52620539
nonDEG	0.76370964	0.13708296	1	nonDEG	-0.4007513	0.38609077
nonDEG	0.42189906	0.33587595	1	nonDEG	0.03551656	0.92541764
nonDEG	-0.2914936	0.49304693	1	nonDEG	0.04504178	0.90108039
nonDEG	0.29477208	0.56928862	1	nonDEG	-0.2698511	0.57117786
nonDEG	-0.5765497	0.23935988	1	nonDEG	0.37114539	0.38419961
nonDEG	-0.2610467	0.77531578	1	nonDEG	0.36601315	0.66718142
nonDEG	2.18844472	0.06593088	1	nonDEG	0.46231582	0.67886541
nonDEG	1.76631387	0.06278935	1	nonDEG	-1.2870899	0.14037909
nonDEG	-1.4437841	0.35899167	1	nonDEG	3.16768492	0.05966742
nonDEG	-0.5211688	0.33346142	1	nonDEG	-0.1973993	0.65923573
nonDEG	1.56257018	0.01564069	0.90639029	nonDEG	-0.507685	0.38001623
nonDEG	-0.8931909	0.08057691	1	nonDEG	0.91386245	0.10595024
nonDEG	-0.7061848	0.16939326	1	nonDEG	0.40155871	0.37125988

nonDEG	0.71369567	0.09670411	1	nonDEG	-0.5778431	0.11633978
nonDEG	-0.3555817	0.40797342	1	nonDEG	-0.505393	0.16669941
nonDEG	0.17545475	0.69333234	1	nonDEG	0.12678435	0.73262694
nonDEG	-0.4184974	0.37046201	1	nonDEG	0.80129535	0.06173719
nonDEG	2.74679759	0.00843356	0.78074119	nonDEG	-1.962915	0.04921253
nonDEG	0.18701808	0.65762049	1	nonDEG	0.03058195	0.93249232
nonDEG	-0.3511391	0.44246251	1	nonDEG	-0.2883939	0.47934188
nonDEG	-2.0426119	0.03535454	1	nonDEG	2.8930849	0.00211103
nonDEG	0.60950471	0.17663749	1	nonDEG	-0.1512857	0.6846373
nonDEG	1.3129632	0.2999231	1	nonDEG	0.22745758	0.86619537
nonDEG	-0.1520878	0.74639175	1	nonDEG	-0.4852734	0.2377799
nonDEG	0.79407592	0.07413523	1	nonDEG	-0.382283	0.29467624
nonDEG	-3.0869467	0.01795636	0.94060016	nonDEG	0.87556414	0.5829351
nonDEG	-0.538945	0.21154486	1	nonDEG	0.0432009	0.9080224
nonDEG	2.39976657	0.43013483	1	nonDEG	-2.675742	0.42377367
nonDEG	-0.8825566	0.14295389	1	nonDEG	2.18639195	0.00020075
nonDEG	0.22573104	0.61858825	1	nonDEG	0.01619355	0.96559828
nonDEG	0.46707947	0.63520707	1	nonDEG	0.19043951	0.85579181
nonDEG	0.20632584	0.83400246	1	nonDEG	1.53440703	0.02744349
nonDEG	0.62823722	0.30488212	1	nonDEG	-0.3645197	0.3732995
nonDEG	-1.0263328	0.03256269	1	nonDEG	1.07503471	0.08374947
nonDEG	0.31874078	0.55550148	1	nonDEG	0.02212776	0.96587763
nonDEG	-0.6349351	0.1406736	1	nonDEG	0.08870325	0.82815477
nonDEG	-0.1981693	0.65102773	1	nonDEG	-0.3177168	0.38950609
nonDEG	-1.5359809	0.02129153	0.99968341	nonDEG	1.03143603	0.10131124
nonDEG	0.3835268	0.422495	1	nonDEG	0.19536724	0.65612564
nonDEG	-1.0623667	0.10045116	1	nonDEG	0.65107901	0.32250906
nonDEG	0.63602272	0.24531867	1	nonDEG	-0.106444	0.79771114
nonDEG	-0.5840164	0.33273068	1	nonDEG	0.47359486	0.42354381
nonDEG	-0.3357829	0.54546872	1	nonDEG	-0.3909122	0.52243006
nonDEG	-1.4515224	0.01491846	0.89057059	nonDEG	1.01200428	0.06906897
nonDEG	1.10489743	0.55860992	1	nonDEG	2.00345638	0.16684349
nonDEG	0.64333551	0.20735633	1	nonDEG	-0.0013594	0.99978083
nonDEG	-0.712481	0.22484086	1	nonDEG	1.35119477	0.04167406
nonDEG	-1.7341082	0.00071184	0.50181787	nonDEG	1.90534065	0.00081678
nonDEG	-0.5338344	0.2335117	1	nonDEG	0.50932352	0.2057163
nonDEG	0.2469669	0.59960554	1	nonDEG	-0.2525914	0.49613041
nonDEG	-0.5918488	0.19096377	1	nonDEG	0.55573764	0.13371977
nonDEG	0.54383671	0.32775786	1	nonDEG	0.44000882	0.35361391
nonDEG	1.60050662	0.00552566	0.73274684	nonDEG	-0.5763746	0.23702801
nonDEG	-1.4495423	0.00617511	0.73793371	nonDEG	1.97766251	0.00102309
nonDEG	-0.5384552	0.67468138	1	nonDEG	0.86682021	0.42717777
nonDEG	-0.8627584	0.40471566	1	nonDEG	0.80451931	0.48477307

nonDEG	-1.7729896	0.08971813	1	nonDEG	2.03323293	0.08118065
nonDEG	0.69618629	0.14997865	1	nonDEG	-0.5279512	0.21631077
nonDEG	-0.9063594	0.13936681	1	nonDEG	0.57468054	0.236337
nonDEG	0.20582486	0.69300101	1	nonDEG	-0.4371647	0.29476419
nonDEG	0.94464802	0.1020042	1	nonDEG	-0.4526998	0.24964341
nonDEG	-0.2498732	0.56261111	1	nonDEG	-0.468142	0.2008111
nonDEG	-1.0352064	0.05799855	1	nonDEG	0.75238339	0.05507348
nonDEG	-0.2079111	0.62689715	1	nonDEG	-0.6654673	0.07909964
nonDEG	0.4164225	0.32524222	1	nonDEG	-0.1646949	0.64922639
nonDEG	-0.3049783	0.65300539	1	nonDEG	0.53929866	0.4599876
nonDEG	-0.2308793	0.74379305	1	nonDEG	1.48963824	0.01131216
nonDEG	-0.2392201	0.58907925	1	nonDEG	-0.2912275	0.46358111
nonDEG	0.47374151	0.4411555	1	nonDEG	-0.3815579	0.39388493
nonDEG	-0.9300617	0.25061822	1	nonDEG	0.929424	0.24523745
nonDEG	0.39982158	0.48869921	1	nonDEG	-1.6245721	0.00049068
nonDEG	-1.5451013	0.01494419	0.89057059	nonDEG	0.42151803	0.48999602
nonDEG	2.22215578	0.02323709	1	nonDEG	-0.6984708	0.45437858
nonDEG	1.54006531	0.06988085	1	nonDEG	-0.4688944	0.58000801
nonDEG	0.52862379	0.23251518	1	nonDEG	-0.2048648	0.58523361
nonDEG	0.01918944	0.96670675	1	nonDEG	0.08888161	0.81210649
nonDEG	-0.5273362	0.29510893	1	nonDEG	-0.0024551	0.99603663
nonDEG	-1.6702509	0.0957585	1	nonDEG	2.22550829	0.01110877
nonDEG	3.03621283	0.00715619	0.75239696	nonDEG	-0.0896391	0.92350963
nonDEG	0.33690743	0.45646396	1	nonDEG	-0.0459867	0.89922186
nonDEG	0.1118279	0.80676292	1	nonDEG	-0.0113058	0.97801987
nonDEG	-0.5497679	0.20521356	1	nonDEG	0.45270998	0.22863611
nonDEG	0.51885818	0.25443183	1	nonDEG	0.32764612	0.3908332
nonDEG	1.61183535	0.14961139	1	nonDEG	-0.4177055	0.69642736
nonDEG	-0.0883198	0.87908771	1	nonDEG	0.20034491	0.71917538
nonDEG	-1.5217141	0.02693224	1	nonDEG	0.61928107	0.42531139
nonDEG	1.04997422	0.07500914	1	nonDEG	-0.0805644	0.83066148
nonDEG	0.38359259	0.37115049	1	nonDEG	-0.0529505	0.88416431
nonDEG	0.74416773	0.46641649	1	nonDEG	0.08712017	0.95886646
nonDEG	-0.929246	0.0569026	1	nonDEG	0.65469887	0.10087019
nonDEG	-0.2528165	0.5873047	1	nonDEG	-0.4694896	0.23122723
nonDEG	-0.3066079	0.50190066	1	nonDEG	-0.0224743	0.95329294
nonDEG	1.28378823	0.0654205	1	nonDEG	-0.9835704	0.05606574
nonDEG	1.45793159	0.01307984	0.86352262	nonDEG	-1.0511404	0.00974222
nonDEG	-0.1608066	0.73016038	1	nonDEG	-0.143555	0.74748402
nonDEG	-0.3888987	0.39900884	1	nonDEG	0.53193471	0.14846882
nonDEG	2.73172749	0.08060173	1	nonDEG	-1.2553181	0.35892927
nonDEG	-0.0885705	0.84659468	1	nonDEG	-0.3728262	0.35597531
nonDEG	-0.9352031	0.29943186	1	nonDEG	0.55244162	0.54595124

nonDEG	-0.6859617	0.12983604	1	nonDEG	0.30259492	0.46062013
nonDEG	-2.2381761	0.28262066	1	nonDEG	3.05895082	0.04339131
nonDEG	0	1	1	nonDEG	3.05420555	0.69366678
nonDEG	0.34211835	0.44614585	1	nonDEG	0.33796946	0.37246873
nonDEG	0.31344511	0.52176359	1	nonDEG	0.0410669	0.93166566
nonDEG	0.74325864	0.67479237	1	nonDEG	-1.631053	0.43692251
nonDEG	1.23016397	0.11907164	1	nonDEG	-0.2147995	0.7690042
nonDEG	-0.0349402	0.95030006	1	nonDEG	-0.2104278	0.56097467
nonDEG	0.86369947	0.06360834	1	nonDEG	-0.4230505	0.25363486
nonDEG	0.2072404	0.62674359	1	nonDEG	-0.0094212	0.98035692
nonDEG	-0.3353321	0.43744186	1	nonDEG	-0.1216284	0.7471463
nonDEG	0.36288275	0.48133064	1	nonDEG	-0.2581578	0.50296711
nonDEG	-0.4601073	0.29080841	1	nonDEG	0.27270854	0.4597121
nonDEG	0.4207131	0.349557	1	nonDEG	-0.2606633	0.47144158
nonDEG	-0.6984281	0.19035744	1	nonDEG	-0.1883252	0.70701603
nonDEG	-0.858874	0.05294632	1	nonDEG	0.4213399	0.27730682
nonDEG	-1.6849663	0.09629533	1	nonDEG	0.72034662	0.52036871
nonDEG	-0.3615407	0.40200814	1	nonDEG	0.41494622	0.25697954
nonDEG	2.6100637	0.11516659	1	nonDEG	-0.7628318	0.5494304
nonDEG	-0.0091659	0.98557514	1	nonDEG	0.17672876	0.65463605
nonDEG	0.44374199	0.59743901	1	nonDEG	-0.3112471	0.71890254
nonDEG	1.30432188	0.05048364	1	nonDEG	-0.3927207	0.49934237
nonDEG	-0.4762241	0.2749578	1	nonDEG	-0.1523018	0.6913304
nonDEG	-0.4773559	0.27057687	1	nonDEG	0.2281525	0.52767569
nonDEG	-2.9953079	0.00854947	0.78407444	nonDEG	4.25489256	0.00011595
nonDEG	-0.5008469	0.26908622	1	nonDEG	1.27993264	0.01019579
nonDEG	1.70948146	0.01585751	0.91041716	nonDEG	-0.5140602	0.34776098
nonDEG	0.89418309	0.11755969	1	nonDEG	-0.4320778	0.33700786
nonDEG	-0.2583789	0.54963402	1	nonDEG	-0.3916225	0.2846313
nonDEG	-0.6138607	0.72977807	1	nonDEG	0.3135094	0.93230186
nonDEG	-0.3241439	0.45910562	1	nonDEG	-0.5285048	0.15574201
nonDEG	0.13417038	0.96346651	1	nonDEG	-1.1619666	0.55171082
nonDEG	-1.4853129	0.00139381	0.53989834	nonDEG	-0.770367	0.04194055
nonDEG	-2.4438575	0.00501539	0.71327584	nonDEG	2.71096368	0.00657215
nonDEG	-1.0622878	0.34955369	1	nonDEG	0.97410906	0.38680038
nonDEG	1.05373281	0.24230426	1	nonDEG	-0.5818191	0.42940711
nonDEG	-0.4307938	0.3391827	1	nonDEG	0.32144544	0.38522542
nonDEG	-0.4423203	0.30358307	1	nonDEG	-0.0170104	0.96596699
nonDEG	0.77701217	0.11301069	1	nonDEG	-0.2241263	0.5479962
nonDEG	0.88747601	0.07431596	1	nonDEG	-0.3455121	0.40022771
nonDEG	0.41090677	0.8641702	1	nonDEG	2.03789333	0.10402216
nonDEG	1.2926386	0.02886301	1	nonDEG	-1.5274465	0.00034738
nonDEG	0.62806399	0.44269639	1	nonDEG	-1.0980331	0.01799456

nonDEG	-1.6442088	0.05276053	1	nonDEG	2.12280045	0.00086168
nonDEG	1.58044144	0.00608857	0.73793371	nonDEG	-0.721975	0.16593139
nonDEG	1.0974411	0.14852591	1	nonDEG	-0.2138559	0.73084277
nonDEG	-0.9531268	0.0966096	1	nonDEG	0.36344574	0.42740062
nonDEG	-1.2930652	0.41537818	1	nonDEG	3.35333371	0.0104455
nonDEG	-1.0237736	0.02978459	1	nonDEG	0.60691336	0.12317517
nonDEG	1.03344308	0.28183682	1	nonDEG	0.02721929	0.98186723
nonDEG	1.45166211	0.02480997	1	nonDEG	-0.0217318	0.96264226
nonDEG	1.01529349	0.06771185	1	nonDEG	0.10351181	0.81429199
nonDEG	-0.7004517	0.23978337	1	nonDEG	0.50991492	0.35026707
nonDEG	-0.4018123	0.35668638	1	nonDEG	-0.2956738	0.42746167
nonDEG	1.34733565	0.11014063	1	nonDEG	0.21977244	0.73684696
nonDEG	-1.5217848	0.11426368	1	nonDEG	1.93066977	0.01526359
nonDEG	0.85716118	0.3419737	1	nonDEG	0.25217096	0.76156254
nonDEG	0.17960456	0.6701456	1	nonDEG	-0.0799527	0.82680569
nonDEG	-0.4142845	0.33308301	1	nonDEG	-0.4742031	0.19210683
nonDEG	-0.9003361	0.09820306	1	nonDEG	0.88280826	0.13915925
nonDEG	-0.5928986	0.2273906	1	nonDEG	0.56309286	0.12473323
nonDEG	-0.4345885	0.31062707	1	nonDEG	-0.0776501	0.83033857
nonDEG	-0.1561332	0.9015503	1	nonDEG	-0.0954781	0.95760633
nonDEG	-0.4259967	0.48101412	1	nonDEG	-1.7737338	0.0234549
nonDEG	-0.3583497	0.59573968	1	nonDEG	0.43857589	0.50601741
nonDEG	0.52912333	0.30830677	1	nonDEG	-0.3337571	0.46811444
nonDEG	-0.3834669	0.39068206	1	nonDEG	-0.2206025	0.54776333
nonDEG	-0.4375926	0.34088854	1	nonDEG	0.30305884	0.43569358
nonDEG	-0.5471514	0.47872271	1	nonDEG	0.66614283	0.38562354
nonDEG	0.90656394	0.21241378	1	nonDEG	-0.269124	0.6264572
nonDEG	-0.3108541	0.4783672	1	nonDEG	-0.0246022	0.95544949
nonDEG	0.1618407	0.71940233	1	nonDEG	-0.0063844	0.98673161
nonDEG	-0.3113773	0.48355918	1	nonDEG	-0.0836575	0.84020244
nonDEG	0.99920607	0.11013592	1	nonDEG	-0.5390741	0.35323486
nonDEG	-0.366989	0.614144	1	nonDEG	0.48517941	0.4762215
nonDEG	-1.5565873	0.00505237	0.71507998	nonDEG	0.78031344	0.08779157
nonDEG	-0.116879	0.7907688	1	nonDEG	-0.5991435	0.11160318
nonDEG	1.81448055	0.01747952	0.93380915	nonDEG	-0.83276	0.06010722
nonDEG	-0.1856304	0.65982126	1	nonDEG	-0.1888287	0.60813618
nonDEG	1.19207491	0.08732653	1	nonDEG	-1.2522445	0.01489631
nonDEG	0.19085526	0.69479973	1	nonDEG	-0.0671307	0.87199732
nonDEG	1.40969021	0.10111746	1	nonDEG	0.10190505	0.90612508
nonDEG	-0.6154703	0.21417892	1	nonDEG	-0.2522144	0.53653379
nonDEG	-1.2398218	0.04596586	1	nonDEG	1.77461869	0.00991413
nonDEG	-0.4683886	0.33093007	1	nonDEG	-0.0082196	0.98618868
nonDEG	0.36259239	0.51169644	1	nonDEG	-0.173657	0.74203497

nonDEG	-0.04595	0.93149291	1	nonDEG	-0.4249805	0.41119468
nonDEG	2.10107485	0.10405319	1	nonDEG	-0.278852	0.81093855
nonDEG	-0.560401	0.45524334	1	nonDEG	1.12564508	0.07037806
nonDEG	-1.8869487	0.14893241	1	nonDEG	1.96193188	0.18963944
nonDEG	-0.4252763	0.3214388	1	nonDEG	-0.0199633	0.95727962
nonDEG	2.20336674	0.34634649	1	nonDEG	-6.4291926	0.05935038
nonDEG	0.07931558	0.85603655	1	nonDEG	0.04383095	0.9047255
nonDEG	-0.5540429	0.28461335	1	nonDEG	-0.1565907	0.70854392
nonDEG	-1.4205043	0.06275458	1	nonDEG	-0.8169043	0.16805304
nonDEG	-0.6805765	0.42407431	1	nonDEG	0.28401871	0.72876321
nonDEG	-0.8767411	0.30724319	1	nonDEG	0.39174967	0.65778738
nonDEG	-0.3038677	0.49412665	1	nonDEG	-0.3788255	0.32836966
nonDEG	-0.5424617	0.24172257	1	nonDEG	0.06200284	0.87109751
nonDEG	-0.229417	0.59997713	1	nonDEG	-0.5458637	0.13829644
nonDEG	-0.3070041	0.57899975	1	nonDEG	-0.3106607	0.56566073
nonDEG	-1.3863644	0.10348175	1	nonDEG	0.86099136	0.25421792
nonDEG	0.57116718	0.29905099	1	nonDEG	-0.3225543	0.39373187
nonDEG	-0.098556	0.82775822	1	nonDEG	0.0155249	0.97031132
nonDEG	-0.4233334	0.36142259	1	nonDEG	0.12723945	0.74156353
nonDEG	0.45735143	0.36598238	1	nonDEG	-0.2539501	0.57333292
nonDEG	0.89202838	0.26906532	1	nonDEG	-1.838614	0.04229341
nonDEG	-0.4081679	0.34757575	1	nonDEG	0.36772293	0.34280692
nonDEG	0.51626132	0.43278846	1	nonDEG	-0.2252314	0.69723703
nonDEG	-0.7697884	0.09613002	1	nonDEG	0.26213005	0.47864042
nonDEG	-0.6693498	0.24143977	1	nonDEG	-0.1925951	0.6756048
nonDEG	-3.8967087	0.01098153	0.84185458	nonDEG	3.08129036	0.02942661
nonDEG	1.84681329	0.06749202	1	nonDEG	-1.4026381	0.10717906
nonDEG	-2.5466686	0.0269884	1	nonDEG	1.79939522	0.17677357
nonDEG	-0.3889996	0.38152624	1	nonDEG	0.21989369	0.56275827
nonDEG	-0.9977321	0.17957198	1	nonDEG	1.28211216	0.03398541
nonDEG	0.89033379	0.07953366	1	nonDEG	-0.4830683	0.2277201
nonDEG	-0.7919524	0.0805777	1	nonDEG	0.39727586	0.27453636
nonDEG	0.95188424	0.04403899	1	nonDEG	-1.0504081	0.01637621
nonDEG	0.14276374	0.74381129	1	nonDEG	0.17711319	0.62935502
nonDEG	2.44664921	0.00776184	0.75581042	nonDEG	-0.3098337	0.65983326
nonDEG	-1.2433231	0.10024842	1	nonDEG	1.32861131	0.14922522
nonDEG	0.66393099	0.2115176	1	nonDEG	-0.5667515	0.24109636
nonDEG	-0.125657	0.76968011	1	nonDEG	0.03466495	0.92748657
nonDEG	0.66018757	0.48931249	1	nonDEG	-1.8509564	0.01524027
nonDEG	-0.2257099	0.60849559	1	nonDEG	-0.1182902	0.74547527
nonDEG	-0.7663256	0.19085341	1	nonDEG	0.63608464	0.27606419
nonDEG	-0.785137	0.24982731	1	nonDEG	0.52590622	0.41399594
nonDEG	-0.2912406	0.54568586	1	nonDEG	-0.1581177	0.73637656

nonDEG	-0.1539684	0.73564904	1	nonDEG	-0.1513225	0.7198079
nonDEG	-0.4587219	0.36286454	1	nonDEG	0.24551071	0.57890772
nonDEG	0.27924347	0.63416114	1	nonDEG	-0.7317022	0.18867385
nonDEG	0.12578598	0.81461329	1	nonDEG	-0.2365771	0.59064481
nonDEG	-0.8579504	0.21524266	1	nonDEG	0.032565	0.9763425
nonDEG	1.1734891	0.2398145	1	nonDEG	0.77514751	0.30271203
nonDEG	0.73217011	0.09361339	1	nonDEG	-0.2492468	0.51328066
nonDEG	-1.0874289	0.39019826	1	nonDEG	1.73255646	0.08056558
nonDEG	1.69795684	0.01099084	0.84185458	nonDEG	-0.7578047	0.21302721
nonDEG	-0.7305746	0.51596813	1	nonDEG	0.73405556	0.51708328
nonDEG	-1.3765765	0.00455139	0.68407511	nonDEG	1.00156523	0.01195339
nonDEG	1.55454357	0.00164093	0.58201664	nonDEG	-0.3314626	0.4117051
nonDEG	-0.0568304	0.9769325	1	nonDEG	0.4860746	0.62645174
nonDEG	2.37086201	0.05956073	1	nonDEG	-0.1697885	0.88841611
nonDEG	-0.5277531	0.36388475	1	nonDEG	0.43544776	0.45263665
nonDEG	1.61551052	0.62363475	1	nonDEG	2.69960509	0.22417635
nonDEG	-2.4379097	0.00365119	0.67010784	nonDEG	2.83009736	3.26E-06
nonDEG	1.08160953	0.03997406	1	nonDEG	-0.4151925	0.38386742
nonDEG	0.20306261	0.64330597	1	nonDEG	-0.3429579	0.36312671
nonDEG	0.80766193	0.07720798	1	nonDEG	-0.4231288	0.29099675
DEG	-1.801233	0.13096753	1	nonDEG	0.19116444	0.87741512
nonDEG	1.2540381	0.0046203	0.68407511	nonDEG	-0.5135317	0.15114038
nonDEG	0.52790266	0.28300995	1	nonDEG	-0.3300301	0.37260853
DEG	7.8982849	6.53E-05	0.14400617	nonDEG	-0.4018198	0.7031007
nonDEG	-0.4478415	0.35653348	1	nonDEG	-0.4069545	0.32960138
nonDEG	0.77827593	0.07202162	1	nonDEG	0.34157932	0.40013655
nonDEG	-0.4101736	0.38504957	1	nonDEG	-0.1395435	0.75686387
nonDEG	1.07645213	0.10825172	1	nonDEG	-0.0511263	0.92473517
nonDEG	0.75493291	0.19035299	1	nonDEG	-0.2114588	0.70032937
nonDEG	0.6836869	0.26724663	1	nonDEG	0.21407767	0.70912197
nonDEG	-0.0779212	0.9059145	1	nonDEG	0.09059391	0.88790847
nonDEG	-1.2630323	0.09496417	1	nonDEG	1.71079085	0.02993662
nonDEG	-0.1913376	0.71052406	1	nonDEG	0.36382138	0.36641875
nonDEG	-0.348512	0.45233595	1	nonDEG	-0.3718153	0.35484221
nonDEG	0.78334916	0.10776697	1	nonDEG	-0.4253279	0.26564228
nonDEG	-1.0129811	0.03280137	1	nonDEG	0.48521133	0.25958048
nonDEG	0.48339985	0.27447648	1	nonDEG	-0.6379792	0.09792331
nonDEG	-0.6285338	0.2278465	1	nonDEG	-0.042724	0.92563979
nonDEG	-0.1269176	0.7715478	1	nonDEG	-0.2159456	0.56553302
nonDEG	-1.538565	0.1084347	1	nonDEG	0.68475782	0.39235198
nonDEG	-0.3869125	0.38514495	1	nonDEG	-0.2965404	0.4471436
nonDEG	1.61492379	0.1954054	1	nonDEG	-1.5168366	0.19876899
nonDEG	-0.3619594	0.45733971	1	nonDEG	-0.3765589	0.36682668

nonDEG	-0.4953986	1	1	nonDEG	4.24506945	0.10468967
nonDEG	1.54102695	0.18925147	1	nonDEG	-0.0650767	0.95379542
nonDEG	0.83201751	0.11957728	1	nonDEG	-0.4290348	0.35112893
nonDEG	-0.3410872	0.43836239	1	nonDEG	0.90012858	0.0277933
nonDEG	0.06032844	0.89605078	1	nonDEG	-0.0965757	0.80897178
nonDEG	0.38676423	0.36469513	1	nonDEG	-0.0859211	0.81793166
nonDEG	2.23359611	0.03238069	1	nonDEG	-1.1733734	0.13825225
nonDEG	-0.1724984	0.69138044	1	nonDEG	-0.831995	0.04556567
nonDEG	-0.4034036	0.36913898	1	nonDEG	0.62455355	0.12396508
nonDEG	-0.4142142	0.4105078	1	nonDEG	0.21997134	0.56339149
nonDEG	0.31761514	0.63762657	1	nonDEG	-0.0953698	0.88124255
nonDEG	-3.0482441	0.00212087	0.61198441	nonDEG	3.0583033	0.00021561
nonDEG	0.31705553	0.68133145	1	nonDEG	-0.0440153	0.94897514
nonDEG	0.29918843	0.55630427	1	nonDEG	-0.2305322	0.57021259
nonDEG	-0.6584243	0.2360655	1	nonDEG	-0.3938493	0.37792409
nonDEG	-0.5637761	0.22417188	1	nonDEG	0.30800455	0.44767016
nonDEG	0.56919204	0.22402934	1	nonDEG	0.14502039	0.72397329
nonDEG	-0.6589003	0.13670148	1	nonDEG	0.07126778	0.85794489
nonDEG	0.70982896	0.12949557	1	nonDEG	-0.5339886	0.2049516
nonDEG	0.92398252	0.20582473	1	nonDEG	-0.7128871	0.33060389
nonDEG	0.31945154	0.44362079	1	nonDEG	-0.0668329	0.85312452
nonDEG	0.47735335	0.36211882	1	nonDEG	0.19617137	0.67650989
nonDEG	1.99835675	0.01955813	0.97153571	nonDEG	-0.2153762	0.77140685
nonDEG	0.74539901	0.2422166	1	nonDEG	0.29886697	0.59565289
nonDEG	0.28566198	0.51166501	1	nonDEG	-0.0783845	0.83739135
nonDEG	-0.1271691	0.77723728	1	nonDEG	0.2904833	0.42687721
nonDEG	1.42903519	0.02847919	1	nonDEG	0.13434278	0.7991787
nonDEG	0.23829197	0.62128964	1	nonDEG	-0.3271635	0.47258174
nonDEG	-0.2283929	0.61081635	1	nonDEG	-1.0212546	0.00805341
nonDEG	0.87532677	0.08201854	1	nonDEG	-0.0396461	0.92944142
nonDEG	-0.6490604	0.2010599	1	nonDEG	0.42616102	0.38492867
nonDEG	1.65822263	0.22129755	1	nonDEG	-0.3115159	0.85329651
nonDEG	1.42271861	0.01042026	0.82021951	nonDEG	-0.8098306	0.06544793
DEG	1.47805699	0.01249565	0.85350212	nonDEG	0.05631494	0.8975546
nonDEG	1.72389055	0.00292166	0.62843199	nonDEG	-0.765924	0.12032385
nonDEG	-1.5090473	0.005819	0.73793371	nonDEG	1.28676633	0.0284589
nonDEG	-2.0851508	0.17377514	1	nonDEG	2.63987871	0.09463695
nonDEG	0.92598892	0.2489529	1	nonDEG	-0.8093969	0.22000147
nonDEG	0.39392429	0.50915495	1	nonDEG	-0.6499711	0.17917723
nonDEG	0.40075225	0.34920994	1	nonDEG	0.21853283	0.54737179
nonDEG	-0.8360505	0.49110076	1	nonDEG	0.95478288	0.41942679
nonDEG	-0.4191874	0.3767	1	nonDEG	-0.1703116	0.67957995
nonDEG	-0.9836977	0.03827262	1	nonDEG	1.67209875	0.00282877

nonDEG	0.77539635	0.1348194	1	nonDEG	-0.6035555	0.15822615
nonDEG	-1.278863	0.06786975	1	nonDEG	0.26935535	0.71024898
nonDEG	0.44990773	0.3317741	1	nonDEG	-0.2669843	0.47303643
nonDEG	-0.6768351	0.14615358	1	nonDEG	0.47200358	0.26863718
nonDEG	-1.3358947	0.03243639	1	nonDEG	1.12610516	0.0196549
nonDEG	0.42386301	0.42934344	1	nonDEG	0.00126039	1
nonDEG	-0.3384166	0.7770461	1	nonDEG	0.89254291	0.38663091
nonDEG	0.37731262	0.4660919	1	nonDEG	-0.3730517	0.35411198
nonDEG	0.13443785	0.83087881	1	nonDEG	-0.2777467	0.64885281
nonDEG	-0.2288837	0.60243213	1	nonDEG	-0.1304933	0.75059603
nonDEG	0.11342252	0.94382463	1	nonDEG	0.8147526	0.41401966
nonDEG	3.31783778	0.07188675	1	nonDEG	-0.6521957	0.69860146
nonDEG	0.66113696	0.3368026	1	nonDEG	-0.692889	0.204076
nonDEG	0.65864747	0.42952545	1	nonDEG	-0.3489171	0.58376584
nonDEG	-1.5384271	0.00422215	0.68407511	nonDEG	1.21906946	0.01734775
nonDEG	-0.9455694	0.21148059	1	nonDEG	0.83325333	0.36873325
nonDEG	-2.723431	0.00029229	0.34458183	nonDEG	-3.630559	0.00051213
nonDEG	-0.065135	0.88344483	1	nonDEG	-0.0671727	0.85959016
nonDEG	0.99510848	0.03564229	1	nonDEG	-0.1201508	0.76981908
nonDEG	-0.5028877	0.3365992	1	nonDEG	-0.1783049	0.69208795
nonDEG	-0.0408714	0.93197313	1	nonDEG	0.35043898	0.41723443
nonDEG	2.58005937	0.01900955	0.96486592	nonDEG	-1.5386693	0.15786879
nonDEG	-0.6157757	0.19774282	1	nonDEG	0.50365551	0.21195369
nonDEG	-1.1852478	0.61317893	1	nonDEG	-4.1272444	0.40854809
nonDEG	0.3639246	0.44656468	1	nonDEG	-0.3118553	0.40554302
nonDEG	-1.0033889	0.40120713	1	nonDEG	-0.0903734	0.97560912
nonDEG	-0.4009244	0.40701048	1	nonDEG	0.1628446	0.72580583
nonDEG	0.79159915	0.63787607	1	nonDEG	0.07776356	0.97389837
nonDEG	0.47412673	0.29231384	1	nonDEG	-0.5405738	0.16964014
nonDEG	0.10933188	0.81832256	1	nonDEG	-0.290076	0.45206121
nonDEG	-0.3899017	0.36062396	1	nonDEG	0.5766425	0.13680131
nonDEG	0.83060512	0.09368672	1	nonDEG	-0.5721695	0.17601809
nonDEG	1.2261901	0.20697242	1	nonDEG	0.41480591	0.60377366
nonDEG	0.67278843	0.16750765	1	nonDEG	-0.0900983	0.81651801
nonDEG	1.50362835	0.01161284	0.85229819	nonDEG	-0.3454943	0.48067226
nonDEG	0.0471906	0.92420625	1	nonDEG	0.24545223	0.567285
nonDEG	0.45579353	0.37436047	1	nonDEG	-0.336564	0.42873916
nonDEG	-0.9039222	0.13353296	1	nonDEG	0.74011242	0.19189133
nonDEG	-0.4779249	0.26355964	1	nonDEG	-0.0504821	0.89255669
nonDEG	-0.7080515	0.20177347	1	nonDEG	0.76783626	0.16457978
nonDEG	-0.509513	0.27213705	1	nonDEG	0.31597373	0.40717819
nonDEG	-0.7700974	0.16010863	1	nonDEG	0.30636237	0.59722596
nonDEG	0.96156231	0.05651142	1	nonDEG	-0.0426539	0.92000859

nonDEG	1.10212667	0.02817833	1	nonDEG	-0.7514882	0.10353913
nonDEG	0.6097027	0.31464214	1	nonDEG	-0.9287044	0.05104528
nonDEG	1.34067277	0.0626992	1	nonDEG	-0.4208998	0.38929379
nonDEG	1.12271781	0.0305521	1	nonDEG	-0.5015721	0.26651381
nonDEG	-0.9205428	0.04886377	1	nonDEG	0.57140255	0.13937673
nonDEG	-1.7205968	0.01655383	0.91951594	nonDEG	1.3435178	0.03101736
nonDEG	-1.1075958	0.13252317	1	nonDEG	0.89602917	0.24805215
nonDEG	0	1	1	nonDEG	-5.13E-15	1
nonDEG	-0.8249802	0.36209344	1	nonDEG	0.9993914	0.28712905
nonDEG	0.65313037	0.18939302	1	nonDEG	-0.4518454	0.29681781
nonDEG	-1.8388885	0.10866453	1	nonDEG	0.84457238	0.45234521
nonDEG	-0.3555531	0.42229061	1	nonDEG	-0.4550649	0.28591272
nonDEG	-1.4466483	0.03132941	1	nonDEG	1.34215506	0.02270223
nonDEG	-0.0781534	0.90211702	1	nonDEG	0.54430732	0.33452183
nonDEG	-0.3216037	0.44272703	1	nonDEG	-0.25847	0.47307436
nonDEG	-4.917598	0.10260431	1	nonDEG	5.1922524	0.20664557
nonDEG	-1.6013359	0.0250991	1	nonDEG	1.34639491	0.0663519
nonDEG	1.7011821	0.00017692	0.23674055	nonDEG	-0.5531545	0.13536627
nonDEG	-0.2395658	0.57455603	1	nonDEG	0.30459735	0.40252464
nonDEG	0.55730383	0.2388184	1	nonDEG	0.16136732	0.68652099
nonDEG	-0.3180268	0.53780258	1	nonDEG	0.26647669	0.51068545
nonDEG	-0.8369443	0.1134414	1	nonDEG	0.58557629	0.22240282
nonDEG	0.2324946	0.61401841	1	nonDEG	-0.5617095	0.13386677
nonDEG	0.44432876	0.64038872	1	nonDEG	-1.2930819	0.14910778
nonDEG	1.55748817	0.00091217	0.51194803	nonDEG	-0.5195226	0.18492683
nonDEG	-1.316604	0.19223543	1	nonDEG	-0.4555975	0.73360065
nonDEG	-0.230397	0.76355306	1	nonDEG	0.66706053	0.34988994
nonDEG	-0.3406078	0.45411801	1	nonDEG	-0.3612125	0.37920508
nonDEG	-0.5311141	0.2404455	1	nonDEG	0.3240248	0.3874003
nonDEG	-0.6454488	0.20817922	1	nonDEG	1.06716599	0.02244285
nonDEG	1.59115482	0.00379046	0.67010784	nonDEG	0.20410708	0.69016412
nonDEG	0.16150202	0.77440897	1	nonDEG	-0.1128537	0.8087636
nonDEG	0.86039394	0.17398986	1	nonDEG	-0.4913345	0.17934931
nonDEG	-3.5541498	0.00462417	0.68407511	nonDEG	2.40131194	0.0126316
nonDEG	2.74153875	0.00116091	0.51605879	nonDEG	-0.7128402	0.30387021
nonDEG	-0.0744545	0.86305542	1	nonDEG	0.27955	0.44527183
nonDEG	-0.204484	0.70647515	1	nonDEG	-0.5382405	0.30989084
nonDEG	-0.9720341	0.37181522	1	nonDEG	0.06645276	0.96815302
nonDEG	0.16597831	0.74444043	1	nonDEG	-0.0776705	0.83696627
nonDEG	-0.2948402	0.49263268	1	nonDEG	1.16333275	0.00210625
nonDEG	0.87666577	0.08502252	1	nonDEG	-0.0707894	0.87765026
nonDEG	1.69115854	0.05260914	1	nonDEG	-1.4469377	0.04184348
nonDEG	-0.1682474	0.70153157	1	nonDEG	-0.4141878	0.26640151

nonDEG	-0.765751	0.14456792	1	nonDEG	0.36548487	0.46279509
nonDEG	-0.2330137	0.58961287	1	nonDEG	-0.2574886	0.48329705
nonDEG	-1.0225822	0.30657591	1	nonDEG	0.99016262	0.40214152
nonDEG	-0.6015934	0.16704028	1	nonDEG	-0.0325583	0.92995592
nonDEG	-0.6747686	0.14785181	1	nonDEG	0.82395117	0.03462038
nonDEG	0.31317165	0.49176445	1	nonDEG	-0.4755227	0.18466239
nonDEG	-0.9276696	0.06635384	1	nonDEG	0.78251437	0.09445287
nonDEG	-2.9949597	0.00494171	0.70646871	nonDEG	1.20248614	0.13404689
nonDEG	0.70845412	0.12703441	1	nonDEG	-0.1462954	0.72442039
nonDEG	-1.1982882	0.0163409	0.91787008	nonDEG	0.89087351	0.02512918
nonDEG	-4.0010682	0.00097385	0.51194803	nonDEG	4.80242747	4.99E-05
nonDEG	-1.0542595	0.16239032	1	nonDEG	1.66621059	0.03272835
nonDEG	1.44829084	0.02045189	0.9902683	nonDEG	-0.3766473	0.46424388
nonDEG	0.67075952	0.17730746	1	nonDEG	0.20592585	0.62710507
nonDEG	0.60455774	0.3193709	1	nonDEG	-0.4805515	0.22246389
nonDEG	0.5151715	0.324158	1	nonDEG	0.10525013	0.82202811
nonDEG	0.28166919	0.58484533	1	nonDEG	-0.1301672	0.73420755
nonDEG	-0.3228969	0.47712551	1	nonDEG	0.31535818	0.42542071
nonDEG	0.19160799	0.65967784	1	nonDEG	-0.2080131	0.58743881
nonDEG	-0.7782864	0.26476889	1	nonDEG	0.93720781	0.1616508
nonDEG	-0.5340472	0.45467346	1	nonDEG	1.18170215	0.1022054
nonDEG	-0.485329	0.31071747	1	nonDEG	-0.2285748	0.56481373
nonDEG	-0.20756	0.63909063	1	nonDEG	-0.0103935	0.97880197
nonDEG	0.26097559	0.53688508	1	nonDEG	-0.0182416	0.96200575
nonDEG	1.21425519	0.08078046	1	nonDEG	0.0024394	0.99682259
nonDEG	-0.3039165	0.5397581	1	nonDEG	0.21798489	0.63899986
nonDEG	-0.9598345	0.3879009	1	nonDEG	1.37380861	0.21415981
nonDEG	0.04792067	0.92500867	1	nonDEG	0.21898512	0.62103744
nonDEG	1.26798343	0.18481529	1	nonDEG	0.31806059	0.67336386
nonDEG	-1.6719501	0.09219248	1	nonDEG	2.44193198	0.03039536
nonDEG	-1.8323239	0.02488513	1	nonDEG	1.40174241	0.1456347
nonDEG	1.30001518	0.08596434	1	nonDEG	-0.2652727	0.71586623
nonDEG	-0.4115372	0.3367566	1	nonDEG	0.23575567	0.51936202
nonDEG	-0.460125	0.28244573	1	nonDEG	0.2393795	0.51316854
nonDEG	0.23127619	0.59991898	1	nonDEG	0.00526343	0.98872083
nonDEG	0.20502117	0.63177042	1	nonDEG	-0.3282654	0.38097323
nonDEG	-0.8346725	0.29556918	1	nonDEG	1.01356154	0.20200174
nonDEG	0.14264405	0.82874245	1	nonDEG	0.0397265	0.94758149
nonDEG	-1.0506507	0.13554279	1	nonDEG	1.09082793	0.13570195
nonDEG	-3.5815968	0.0061607	0.73793371	nonDEG	0.72969508	0.49985528
nonDEG	0.80240926	0.19183851	1	nonDEG	-0.2714955	0.61371583
nonDEG	-0.2934166	0.52910391	1	nonDEG	-0.082318	0.85212465
nonDEG	0.12452022	0.77627131	1	nonDEG	-0.6719681	0.0739488

nonDEG	-1.8758959	0.01529242	0.89858993	nonDEG	1.4144766	0.06617151
nonDEG	1.49409826	0.06969462	1	nonDEG	-1.2607469	0.03214936
nonDEG	-0.76201	0.11811973	1	nonDEG	0.66360875	0.07310821
nonDEG	1.60949465	0.06984055	1	nonDEG	-0.6507578	0.39568682
nonDEG	1.3333603	0.02359829	1	nonDEG	-0.4786568	0.2793693
nonDEG	-0.2478274	0.59874666	1	nonDEG	0.29352441	0.44566183
nonDEG	-0.2734239	0.53304064	1	nonDEG	-0.428615	0.24007005
nonDEG	1.56337735	0.00132837	0.52845676	nonDEG	-0.5436943	0.1659368
nonDEG	-0.3377946	0.43378644	1	nonDEG	0.05889225	0.87369346
nonDEG	1.53099374	0.12421279	1	nonDEG	-0.1735559	0.87010916
nonDEG	-1.0944037	0.04181895	1	nonDEG	0.79879277	0.09058138
nonDEG	0.76677115	0.12224361	1	nonDEG	-0.2774663	0.45181583
nonDEG	1.92639477	0.00081563	0.50181787	nonDEG	-1.2469099	0.02393835
nonDEG	-1.5790936	0.02341324	1	nonDEG	2.01451484	0.01462071
nonDEG	-2.2667274	0.18573927	1	nonDEG	2.81776916	0.13512552
nonDEG	-0.4661666	0.32050498	1	nonDEG	-0.4634574	0.30374256
nonDEG	-1.2198039	0.0490848	1	nonDEG	1.46467543	0.00392373
nonDEG	-0.1814912	0.67935083	1	nonDEG	-0.6881505	0.08463816
nonDEG	0.32745359	0.46923543	1	nonDEG	0.21857189	0.61784257
nonDEG	0.98941072	0.34424877	1	nonDEG	-0.0044756	1
nonDEG	0.43589737	0.4801483	1	nonDEG	-0.4542054	0.41310663
nonDEG	-0.4823986	0.3037202	1	nonDEG	0.11743566	0.75368059
nonDEG	0.00054905	0.99930912	1	nonDEG	-0.229617	0.5707805
nonDEG	0.08491378	0.84900671	1	nonDEG	0.78144898	0.0598305
nonDEG	-0.2061625	0.62706986	1	nonDEG	-0.2685633	0.45922515
nonDEG	0.06065677	0.89771648	1	nonDEG	-0.8422804	0.03832141
nonDEG	-0.3266001	0.54860195	1	nonDEG	-9.32E-05	1
nonDEG	-0.768705	0.46932129	1	nonDEG	-0.3892215	0.63485103
nonDEG	-0.9066828	0.22296337	1	nonDEG	0.97890599	0.11416775
nonDEG	1.00638768	0.19151128	1	nonDEG	-1.7771267	0.01304085
nonDEG	0.17830147	0.67776969	1	nonDEG	0.25812499	0.52847884
nonDEG	1.16706837	0.05885224	1	nonDEG	-0.0981144	0.86160804
nonDEG	1.1875889	0.10628529	1	nonDEG	0.1604462	0.76241232
nonDEG	-0.2521477	0.57652948	1	nonDEG	-0.4055153	0.28743458
nonDEG	-1.4639477	0.03197436	1	nonDEG	1.05434366	0.05286546
nonDEG	0.29787902	0.49549999	1	nonDEG	0.19105597	0.60494128
nonDEG	-0.8364172	0.29578762	1	nonDEG	0.50384726	0.53739508
nonDEG	2.75431084	0.02158729	1	nonDEG	-0.5257503	0.60812641
nonDEG	-0.5368363	0.25416158	1	nonDEG	-0.0412721	0.92844957
nonDEG	-0.3157217	0.45764322	1	nonDEG	-0.3147094	0.41917662
nonDEG	-0.8115287	0.08878079	1	nonDEG	0.29908457	0.45554533
nonDEG	-0.4240292	0.36575707	1	nonDEG	0.02159268	0.96419182
nonDEG	-0.4832752	0.26212526	1	nonDEG	0.07867815	0.82861727

nonDEG	1.29347559	0.00320454	0.65616368	nonDEG	-0.6735209	0.0650484
nonDEG	0.84884219	0.17416546	1	nonDEG	-0.2218985	0.57481249
nonDEG	-0.5801487	0.18706751	1	nonDEG	0.12941394	0.72912289
nonDEG	0.6814354	0.15715379	1	nonDEG	-0.6200251	0.14771846
nonDEG	-2.0536145	0.01874796	0.95501418	nonDEG	2.08650512	0.04150919
nonDEG	0.13723234	0.78722392	1	nonDEG	-0.7886114	0.06326109
nonDEG	0.41109973	0.62100917	1	nonDEG	-0.9508092	0.28679475
nonDEG	-0.2629042	0.54211444	1	nonDEG	-0.0796918	0.84198981
nonDEG	-2.016942	0.04115259	1	nonDEG	3.42909304	0.00426794
nonDEG	-0.2528718	0.5895799	1	nonDEG	-0.2384229	0.61436693
nonDEG	-0.7007688	0.13421028	1	nonDEG	-0.0241969	0.95363757
nonDEG	-0.8246212	0.16569426	1	nonDEG	1.05126858	0.10330619
nonDEG	1.90507891	0.04393135	1	nonDEG	-0.1187198	0.89962744
nonDEG	2.12363127	0.03587447	1	nonDEG	0.25111888	0.79058797
nonDEG	0.25231455	0.57399071	1	nonDEG	-0.3028663	0.44762357
nonDEG	0.55769943	0.365756	1	nonDEG	-0.0244438	0.96797595
nonDEG	1.11518106	0.02207086	1	nonDEG	-0.029209	0.93875738
nonDEG	0.68377819	0.13886413	1	nonDEG	-0.2741923	0.45699751
nonDEG	0.0504177	0.90698373	1	nonDEG	0.23124893	0.54708804
nonDEG	0.01671704	0.96916127	1	nonDEG	0.20655268	0.57138201
nonDEG	1.1040155	0.04513122	1	nonDEG	-0.4959004	0.31015056
nonDEG	-0.3078944	0.46816266	1	nonDEG	0.00556867	0.98843617
nonDEG	0.5547384	0.25873525	1	nonDEG	-0.6202713	0.10947438
nonDEG	1.40354668	0.03869123	1	nonDEG	-0.5409745	0.27321275
nonDEG	-0.4922028	0.2917059	1	nonDEG	-0.3676661	0.38518598
nonDEG	0.314073	0.68220076	1	nonDEG	0.10532217	0.89115364
nonDEG	-0.9143257	0.05034166	1	nonDEG	0.76030716	0.07357245
nonDEG	-0.4634439	0.30075075	1	nonDEG	-0.0017319	0.99679623
nonDEG	-0.103392	0.81866675	1	nonDEG	-0.2506809	0.50874951
nonDEG	-0.523993	0.23519076	1	nonDEG	0.20793055	0.59748964
nonDEG	-0.0476586	0.96212373	1	nonDEG	0.50583707	0.520294
nonDEG	0.78624341	0.06822038	1	nonDEG	-0.204919	0.57639072
nonDEG	-0.2454036	0.90369434	1	nonDEG	2.07930262	0.10861748
nonDEG	0.54125774	0.34119679	1	nonDEG	-1.0202539	0.00991865
nonDEG	-0.6505273	0.16291357	1	nonDEG	0.832893	0.07386926
nonDEG	0.48879183	0.34581821	1	nonDEG	-0.1271957	0.76571322
nonDEG	1.15865042	0.05139926	1	nonDEG	0.13079319	0.80554989
nonDEG	0.610376	0.35028541	1	nonDEG	-0.5792083	0.35436577
nonDEG	1.54122324	0.09252332	1	nonDEG	-0.3493664	0.68821651
nonDEG	0.76394444	0.16860451	1	nonDEG	-0.2815473	0.54878171
nonDEG	2.14577639	0.00601477	0.73793371	nonDEG	-0.2709225	0.6667192
nonDEG	-0.003258	0.99447633	1	nonDEG	-0.0594534	0.86958267
nonDEG	0.04275397	0.92662445	1	nonDEG	0.38578728	0.36403748

nonDEG	-0.6105727	0.21644093	1	nonDEG	0.61303623	0.15017526
nonDEG	0.12291309	0.79273106	1	nonDEG	0.06797123	0.85218635
nonDEG	2.7910159	0.01391843	0.8811712	nonDEG	-1.344077	0.23684971
nonDEG	1.93722959	0.0150776	0.89340262	nonDEG	0.63049442	0.31051713
nonDEG	-2.0177686	0.04001464	1	nonDEG	0.32528785	0.78072664
nonDEG	-0.0459724	0.91401502	1	nonDEG	-0.5521422	0.15641637
nonDEG	0.26786386	0.66528763	1	nonDEG	0.30320183	0.5721884
nonDEG	0.3003636	0.48801151	1	nonDEG	-0.0118702	0.97530874
nonDEG	0.66456104	0.14603265	1	nonDEG	-0.1138058	0.77914626
nonDEG	0.47958165	0.35741533	1	nonDEG	-0.0285382	0.95454263
nonDEG	-0.0650467	0.89130541	1	nonDEG	-0.5899647	0.15590155
nonDEG	-0.9981788	0.21510653	1	nonDEG	0.66223385	0.50672677
nonDEG	-0.4938417	0.36761673	1	nonDEG	0.29961512	0.56929195
nonDEG	-0.1277617	0.76300132	1	nonDEG	0.14905038	0.68892916
nonDEG	0.959909	0.05020729	1	nonDEG	-0.4908738	0.25353599
nonDEG	-0.3919862	0.39461823	1	nonDEG	-0.5844604	0.14505921
nonDEG	0.7205935	0.13572858	1	nonDEG	-0.1745576	0.64759493
nonDEG	0.12635437	0.77247284	1	nonDEG	-0.1106664	0.76419898
nonDEG	-0.1407962	0.74699051	1	nonDEG	0.60069963	0.10205111
nonDEG	1.62038614	0.01708429	0.93079586	nonDEG	-0.9361426	0.09435789
nonDEG	1.13837736	0.04435485	1	nonDEG	-0.2197547	0.66202
nonDEG	-0.3163564	0.58818775	1	nonDEG	0.43243079	0.36154295
nonDEG	-0.2741904	0.51923367	1	nonDEG	-0.1328177	0.7264962
nonDEG	0.91782095	0.06245822	1	nonDEG	-0.2461983	0.54213288
nonDEG	0.12038418	0.77917007	1	nonDEG	-0.3060629	0.39737345
nonDEG	-1.6291758	0.00113303	0.51315728	nonDEG	2.15499894	1.42E-06
nonDEG	-0.3381987	0.43492604	1	nonDEG	0.18699546	0.6118736
nonDEG	-1.1158021	0.04906389	1	nonDEG	1.3463436	0.02411614
nonDEG	-0.9551138	0.06232182	1	nonDEG	0.17751865	0.68869827
nonDEG	-0.3169572	0.51362486	1	nonDEG	0.23412076	0.60306429
nonDEG	-0.0380548	0.93651424	1	nonDEG	-0.371221	0.38183702
nonDEG	1.67223002	0.00380135	0.67010784	nonDEG	-0.4609292	0.29693663
nonDEG	1.01776041	0.13087196	1	nonDEG	-1.9190375	0.00999573
nonDEG	-0.7781879	0.27615179	1	nonDEG	0.7698614	0.26369094
nonDEG	-0.8740281	0.17109352	1	nonDEG	-0.7038998	0.33529031
nonDEG	0.08884808	0.83939997	1	nonDEG	0.41050281	0.27221906
nonDEG	0.11853285	0.87443366	1	nonDEG	0.07412813	0.92169446
nonDEG	-0.1979438	0.70919326	1	nonDEG	0.15564556	0.75763792
nonDEG	-1.0071933	0.08839507	1	nonDEG	0.17566102	0.67273341
nonDEG	-0.986055	0.11121779	1	nonDEG	0.94986979	0.13382487
nonDEG	0.81281994	0.12547869	1	nonDEG	-0.5604761	0.15826956
nonDEG	-1.6761314	0.04136508	1	nonDEG	2.57519273	0.009685
nonDEG	-0.2059704	0.6359634	1	nonDEG	-0.4577641	0.22463379

nonDEG	0.42798434	0.47198728	1	nonDEG	-0.4639312	0.2976162
nonDEG	-1.5040458	0.00070176	0.50181787	nonDEG	-0.0365351	0.94054061
nonDEG	1.21889926	0.26889351	1	nonDEG	0.52597266	0.60167249
nonDEG	1.57707837	0.02600567	1	nonDEG	0.37337653	0.47908031
nonDEG	0.8892968	0.08128637	1	nonDEG	-0.5301758	0.19416884
nonDEG	4.51860586	0.00081491	0.50181787	nonDEG	-1.075625	0.36561858
nonDEG	-2.2020335	0.05638587	1	nonDEG	3.06393842	0.00535856
nonDEG	0.18705337	0.66393435	1	nonDEG	-0.010006	0.97993358
nonDEG	0.06650351	0.90858259	1	nonDEG	0.84562358	0.11018909
nonDEG	-2.2324577	0.11555716	1	nonDEG	3.28413735	0.03342959
nonDEG	1.37716613	0.00179192	0.61198441	nonDEG	-0.8287041	0.02755229
nonDEG	-0.5809629	0.180397	1	nonDEG	0.12432044	0.74532156
nonDEG	0.08716579	0.84317233	1	nonDEG	0.13770207	0.71262792
nonDEG	-0.4261692	0.37503207	1	nonDEG	-0.3584524	0.33915615
nonDEG	2.31627126	0.01449744	0.88827361	nonDEG	-1.3711861	0.13010573
nonDEG	0.92753958	0.15748785	1	nonDEG	-0.3230027	0.59968076
nonDEG	-0.3962807	0.3689193	1	nonDEG	0.20665068	0.57198941
nonDEG	0.59334152	0.42953384	1	nonDEG	0.25830943	0.72765949
nonDEG	0.5448861	0.25567828	1	nonDEG	0.04915712	0.90608234
nonDEG	0.22525281	0.61250026	1	nonDEG	-0.2007264	0.59152791
nonDEG	-0.6751542	0.5691275	1	nonDEG	1.25913467	0.49829325
nonDEG	1.03931295	0.26769859	1	nonDEG	0.5681004	0.51919435
nonDEG	-0.4419936	0.53055709	1	nonDEG	0.46847592	0.43508671
DEG	-1.3185839	0.05374278	1	nonDEG	0.34621647	0.48095019
nonDEG	-1.4536202	0.01316029	0.86478986	nonDEG	1.43030168	0.00151218
nonDEG	0.44539228	0.36049623	1	nonDEG	-0.4193531	0.33940623
nonDEG	-0.6235254	0.17295691	1	nonDEG	0.00412181	0.99260816
nonDEG	0.05477146	0.90936035	1	nonDEG	-0.7742012	0.06593308
nonDEG	-0.137389	0.77103521	1	nonDEG	-0.3217987	0.47950551
nonDEG	-1.0375293	0.020401	0.9902683	nonDEG	1.97343006	0.00360042
nonDEG	1.34900729	0.01025346	0.80925385	nonDEG	-0.6471463	0.09381459
nonDEG	-0.6039083	0.36449908	1	nonDEG	0.61212782	0.29351042
nonDEG	-0.3644515	0.4374567	1	nonDEG	-0.2199414	0.5838025
nonDEG	0.66612764	0.16969038	1	nonDEG	-0.1992647	0.59776882
nonDEG	-1.2201934	0.15336584	1	nonDEG	0.30161168	0.8180314
nonDEG	0.56400623	0.22118147	1	nonDEG	-0.7715506	0.05614723
nonDEG	0.51693583	0.3260093	1	nonDEG	-0.4604478	0.24215164
nonDEG	-0.3503754	0.45472409	1	nonDEG	0.72331393	0.10309203
nonDEG	-0.8041413	0.08001509	1	nonDEG	0.65350183	0.08740134
nonDEG	-3.8581247	0.00854317	0.78407444	nonDEG	4.13191202	0.01063023
nonDEG	-0.954319	0.10910404	1	nonDEG	1.71992224	0.00606102
nonDEG	-0.3155107	0.46814933	1	nonDEG	0.00625591	0.98739061
nonDEG	0.6051921	0.21429963	1	nonDEG	-0.6047085	0.16560925

nonDEG	-0.0971646	0.83657981	1	nonDEG	0.15408009	0.67321432
nonDEG	0.68061073	0.12472195	1	nonDEG	-0.3283294	0.38733872
nonDEG	0.28961236	0.50890753	1	nonDEG	-0.2032361	0.57887115
nonDEG	1.44173768	0.14013754	1	nonDEG	-0.6093957	0.51813318
nonDEG	-1.8759693	0.00538827	0.72967447	nonDEG	1.07932962	0.02374995
nonDEG	0.24911297	0.67645483	1	nonDEG	-0.1683784	0.71540346
nonDEG	0.50098511	0.23612504	1	nonDEG	0.06824123	0.85058212
nonDEG	0.42902139	0.3606471	1	nonDEG	-0.3642119	0.3829469
nonDEG	-0.6023452	0.24343621	1	nonDEG	-0.0922094	0.81645963
nonDEG	-0.3828543	0.37686588	1	nonDEG	-0.0215728	0.95497387
nonDEG	-1.8492548	0.00197806	0.61198441	nonDEG	1.88141867	0.01042278
nonDEG	0.72544142	0.13358533	1	nonDEG	-0.3148055	0.45443766
nonDEG	0.26555351	0.54663649	1	nonDEG	-0.0840081	0.81874908
nonDEG	-0.4445836	0.34652152	1	nonDEG	0.49272543	0.23222107
nonDEG	0.05456192	0.92100414	1	nonDEG	0.32973522	0.51921427
nonDEG	-0.3353962	0.45377856	1	nonDEG	-0.1527526	0.67882489
nonDEG	0.60108513	0.21429278	1	nonDEG	-0.1611901	0.67134811
nonDEG	-2.8528404	0.00638629	0.73793371	nonDEG	3.52497883	0.00774039
nonDEG	-0.0454639	0.91901906	1	nonDEG	-0.5705729	0.151105
nonDEG	-1.5696104	0.03259466	1	nonDEG	1.96814854	0.00479785
nonDEG	3.97557284	2.99E-05	0.12599308	nonDEG	-0.8562277	0.28633865
nonDEG	-1.2962395	0.04163956	1	nonDEG	1.20218802	0.08793099
nonDEG	-1.4891618	0.23056116	1	nonDEG	1.45055213	0.30828612
nonDEG	-0.1636589	0.70435674	1	nonDEG	-0.1009455	0.7942507
nonDEG	-1.4259871	0.00369139	0.67010784	nonDEG	2.04539629	0.00045847
nonDEG	0.59747443	0.27785688	1	nonDEG	0.17585129	0.72064382
nonDEG	-0.3130308	0.49289544	1	nonDEG	-0.1054486	0.78646674
nonDEG	-0.3622101	0.46098267	1	nonDEG	0.99014474	0.03229951
nonDEG	0.75811841	0.2951463	1	nonDEG	0.27038269	0.67760113
nonDEG	0.76013535	0.27801201	1	nonDEG	-0.4109883	0.51075576
nonDEG	-0.552656	0.25296946	1	nonDEG	0.24778626	0.56092515
nonDEG	-0.3213967	0.49291922	1	nonDEG	-0.0010761	1
nonDEG	1.54072218	0.01415154	0.88752292	nonDEG	0.21670783	0.70498918
nonDEG	0.61903522	0.37467807	1	nonDEG	-0.576251	0.21318478
nonDEG	1.44981133	0.04716278	1	nonDEG	-1.2515985	0.08132977
nonDEG	1.48869295	0.0023473	0.61776954	nonDEG	-0.2927148	0.47296532
nonDEG	1.526798	0.01190531	0.85229819	nonDEG	-0.1741897	0.74681758
nonDEG	-1.3376052	0.04651142	1	nonDEG	1.97689235	0.00774245
nonDEG	0.57042328	0.3102977	1	nonDEG	0.03547634	0.94554892
nonDEG	0.12154494	0.79532984	1	nonDEG	0.23788775	0.56880153
nonDEG	-1.103217	0.16960875	1	nonDEG	1.45600957	0.17714561
nonDEG	0.40937752	0.50132868	1	nonDEG	-0.0960029	0.86329561
nonDEG	0.70628376	0.14290717	1	nonDEG	-0.1881856	0.67848412

nonDEG	1.00177347	0.03223104	1	nonDEG	-0.6144602	0.13160829
nonDEG	-0.1688836	0.69219378	1	nonDEG	0.13768361	0.70537278
nonDEG	1.97481759	0.00542439	0.72967447	nonDEG	-0.0201421	0.97046869
nonDEG	-1.5026946	0.16149419	1	nonDEG	0.97425646	0.43374803
nonDEG	0.72338407	0.32279572	1	nonDEG	0.33359909	0.57034243
nonDEG	-1.9836679	0.00734807	0.75263895	nonDEG	1.58275051	0.06839916
nonDEG	-1.0323497	0.07699486	1	nonDEG	0.82003736	0.08737632
nonDEG	1.37448067	0.03313159	1	nonDEG	-0.0875688	0.88641342
nonDEG	0.23018928	0.6413146	1	nonDEG	-0.0483636	0.905661
nonDEG	-0.2191983	0.6819051	1	nonDEG	0.04663266	0.93118225
nonDEG	0.41996241	0.31927263	1	nonDEG	-0.5009584	0.23390011
nonDEG	-0.3261185	0.45163948	1	nonDEG	-0.3523212	0.3377388
nonDEG	0.18547786	0.86667285	1	nonDEG	-0.2681069	0.78774013
nonDEG	0.98950188	0.06303703	1	nonDEG	0.12161857	0.79596113
nonDEG	0.28945069	0.60240897	1	nonDEG	-0.4817144	0.26534936
nonDEG	-0.226566	0.59525735	1	nonDEG	-0.5394068	0.13869576
nonDEG	-1.5972494	0.22609054	1	nonDEG	2.70623334	0.06562914
nonDEG	0.35039411	0.6134998	1	nonDEG	0.59202963	0.34900099
nonDEG	0.76409911	0.08352199	1	nonDEG	-0.5106	0.16761591
nonDEG	2.15847573	0.01576966	0.91028043	nonDEG	-0.5870677	0.37503128
nonDEG	0.89231871	0.09782624	1	nonDEG	-0.1612672	0.72856978
nonDEG	-0.3080096	0.47250542	1	nonDEG	-0.0874031	0.81169339
nonDEG	-0.5098239	0.28606644	1	nonDEG	0.29455581	0.47397389
nonDEG	-1.526644	0.02121349	0.99960842	nonDEG	0.05734972	0.9092211
nonDEG	0.52917279	0.57316609	1	nonDEG	-0.4572868	0.65321341
nonDEG	0.86483371	0.10317458	1	nonDEG	0.17226586	0.7022167
nonDEG	-0.009105	0.98563205	1	nonDEG	0.10832205	0.78889359
nonDEG	0.63393675	0.51873907	1	nonDEG	0.2732527	0.7777064
nonDEG	-0.7564235	0.20714029	1	nonDEG	0.94151295	0.08291872
nonDEG	-0.7139918	0.10176506	1	nonDEG	-0.2770842	0.57060357
nonDEG	0.34825598	0.41818376	1	nonDEG	0.03763057	0.91775816
nonDEG	0.08271452	0.85576198	1	nonDEG	0.00596892	0.987289
nonDEG	0.2914119	0.54272229	1	nonDEG	-0.237933	0.56687833
nonDEG	-0.2291169	0.60037418	1	nonDEG	-0.6431561	0.12699552
nonDEG	0.27704397	0.67343222	1	nonDEG	0.54310339	0.33062301
nonDEG	0.91666024	0.09752035	1	nonDEG	-0.4467866	0.26023133
nonDEG	0.69558079	0.16750043	1	nonDEG	-0.3709005	0.32251734
nonDEG	0.3172885	0.4788595	1	nonDEG	-0.4973714	0.22908186
nonDEG	-1.2569553	0.02203049	1	nonDEG	1.44606214	0.01653372
nonDEG	0.68421233	0.21127234	1	nonDEG	-0.2430296	0.56673511
nonDEG	-2.7852781	0.0076789	0.75581042	nonDEG	2.68920026	0.0103312
nonDEG	0.26552587	0.5583288	1	nonDEG	-0.3496922	0.4308122
nonDEG	-1.0589848	0.14088789	1	nonDEG	1.30210576	0.09332759

nonDEG	-0.6557722	0.330847	1	nonDEG	0.74038369	0.26483465
nonDEG	0.61652011	0.19626475	1	nonDEG	0.09599829	0.81684343
nonDEG	0.01909955	0.96690491	1	nonDEG	-0.2166092	0.5728135
nonDEG	-1.3277671	0.02943292	1	nonDEG	1.65529354	0.00179327
nonDEG	-0.2640086	0.54656066	1	nonDEG	0.63902655	0.11128196
nonDEG	0.38900348	0.39128164	1	nonDEG	0.02011823	0.96032382
nonDEG	-0.284811	0.89533237	1	nonDEG	1.12727857	0.39719514
nonDEG	-0.2532341	0.57260511	1	nonDEG	0.60115192	0.12884343
nonDEG	0.18896091	0.67415166	1	nonDEG	0.07660138	0.84879208
nonDEG	-0.3053138	0.56638682	1	nonDEG	0.70548469	0.17586284
nonDEG	0.88260108	0.05499046	1	nonDEG	-0.3221547	0.43374865
nonDEG	0.81979635	0.06528639	1	nonDEG	-0.3523168	0.33294714
nonDEG	0.74948619	0.16096952	1	nonDEG	-0.4883234	0.23015587
nonDEG	-0.6206722	0.197284	1	nonDEG	0.51853169	0.18145359
nonDEG	1.0060802	0.02531428	1	nonDEG	-0.4414968	0.24564642
nonDEG	-0.3073482	0.4843046	1	nonDEG	-0.4065352	0.2642601
nonDEG	0.40213124	0.43169951	1	nonDEG	-0.1974057	0.60613713
nonDEG	0.80782971	0.09629202	1	nonDEG	-0.6882201	0.11879344
nonDEG	4.1947202	0.00205942	0.61198441	nonDEG	-0.6374248	0.57702963
nonDEG	0.69539808	0.16915237	1	nonDEG	-0.002842	0.99707271
nonDEG	0.32811303	0.59002735	1	nonDEG	0.00776102	0.99340971
nonDEG	-0.7547956	0.23484836	1	nonDEG	0.15644812	0.79909142
nonDEG	0.28597983	0.49835174	1	nonDEG	0.70523921	0.06600804
nonDEG	-0.3167781	0.44798525	1	nonDEG	-0.2694239	0.46139686
nonDEG	0.23368038	0.59109399	1	nonDEG	-0.0185498	0.95939898
DEG	0.70983541	0.18514452	1	nonDEG	0.15404246	0.67767523
nonDEG	-2.657335	0.00375804	0.67010784	nonDEG	3.03934815	0.00317865
nonDEG	1.00448073	0.10908699	1	nonDEG	0.05585465	0.9064753
nonDEG	0.41333778	0.378906	1	nonDEG	-0.3621128	0.32053294
nonDEG	-0.4183957	0.34603843	1	nonDEG	0.26300748	0.47557646
nonDEG	0.07682827	0.939307	1	nonDEG	0.77655266	0.25351424
nonDEG	0.4082537	0.45353361	1	nonDEG	0.39820415	0.40797007
nonDEG	0.47511954	0.28658432	1	nonDEG	0.02173036	0.95343215
nonDEG	-0.1686987	0.69804555	1	nonDEG	0.03674284	0.92274353
nonDEG	0.45642721	0.29676159	1	nonDEG	-0.2326855	0.5201285
nonDEG	0.74605035	0.11748839	1	nonDEG	-0.4548134	0.30640922
nonDEG	-0.3437107	0.53336406	1	nonDEG	0.20398709	0.73972645
nonDEG	-1.0565801	0.09568666	1	nonDEG	1.29552308	0.01831007
nonDEG	-0.3180723	0.46499257	1	nonDEG	-0.0593764	0.8717782
nonDEG	-0.0002402	1	1	nonDEG	0.04691613	0.92105977
nonDEG	1.62105577	0.00596612	0.73793371	nonDEG	-0.2124378	0.67500321
nonDEG	2.16009062	0.05561534	1	nonDEG	-0.7800828	0.48065394
nonDEG	-2.7368664	0.0194827	0.97153571	nonDEG	2.69065881	0.0132686

nonDEG	1.0798157	0.1136258	1	nonDEG	-0.7551565	0.26845944
nonDEG	0.77548402	0.24520561	1	nonDEG	-0.9322323	0.16243884
nonDEG	-0.6386784	0.1693414	1	nonDEG	0.42091717	0.2726992
nonDEG	0.21192253	0.67984357	1	nonDEG	-0.243516	0.53592933
nonDEG	-1.0745964	0.10721978	1	nonDEG	0.73408243	0.25904849
nonDEG	1.18126686	0.08753019	1	nonDEG	-0.2279031	0.65117188
nonDEG	-1.2467282	0.09298739	1	nonDEG	0.56668357	0.44922044
nonDEG	1.75973093	0.09460121	1	nonDEG	-0.915108	0.35114427
nonDEG	0.50188664	0.55060243	1	nonDEG	1.45396546	0.02681147
nonDEG	4.70280594	0.22895117	1	nonDEG	1.0088705	0.6880429
nonDEG	1.52187593	0.006652	0.73793371	nonDEG	-0.4963728	0.3074965
nonDEG	-0.3921	0.36753454	1	nonDEG	0.19977689	0.59225848
nonDEG	0.46120902	0.43933978	1	nonDEG	-0.7899244	0.16473439
nonDEG	-1.5860346	0.0895589	1	nonDEG	0.80354624	0.30215856
nonDEG	1.52146793	0.00289339	0.62843199	nonDEG	-0.7788396	0.1000857
nonDEG	0.37258884	0.47200625	1	nonDEG	-0.8365034	0.04118289
nonDEG	1.75604031	0.00539313	0.72967447	nonDEG	-0.2772607	0.62473118
nonDEG	1.39531875	0.04315506	1	nonDEG	-0.8759382	0.08593516
nonDEG	0.00017032	1	1	nonDEG	-0.9890505	0.02590464
nonDEG	-0.0063534	0.98876398	1	nonDEG	0.21631849	0.55840351
nonDEG	1.38347646	0.00692068	0.75126991	nonDEG	-1.0280764	0.02809296
nonDEG	-0.5755954	0.19471125	1	nonDEG	0.44126689	0.22995192
nonDEG	-1.6400605	0.00434368	0.68407511	nonDEG	1.50012974	0.01484156
nonDEG	0.92529303	0.05525882	1	nonDEG	-0.6485788	0.10278246
nonDEG	-0.1841675	0.68449415	1	nonDEG	-0.6516152	0.092404
nonDEG	-0.642688	0.27665598	1	nonDEG	0.26977239	0.66135234
nonDEG	-0.1084577	0.81688067	1	nonDEG	-0.1753953	0.67736725
nonDEG	-0.0685094	0.87997371	1	nonDEG	-0.4365131	0.26410837
nonDEG	-1.4157078	0.02685214	1	nonDEG	0.99179527	0.09307598
nonDEG	-1.8985213	0.03976615	1	nonDEG	1.85789809	0.05491828
nonDEG	-1.6275136	0.00993337	0.80027302	nonDEG	1.20729336	0.00253492
nonDEG	0.28000794	0.53151823	1	nonDEG	-0.0645986	0.86030082
nonDEG	0.36064332	0.47752102	1	nonDEG	-0.1587808	0.7296884
nonDEG	-0.1510995	0.74277224	1	nonDEG	-0.0857858	0.82034311
nonDEG	1.65416425	0.0449151	1	nonDEG	0.32594273	0.65304888
nonDEG	-1.1327435	0.09427188	1	nonDEG	1.02876651	0.02305192
nonDEG	0.8032806	0.15183034	1	nonDEG	-0.2970596	0.56757953
nonDEG	1.30808228	0.04492263	1	nonDEG	-0.5207109	0.23994256
nonDEG	0.78486318	0.08319267	1	nonDEG	-0.3510018	0.35464939
nonDEG	0.80292722	0.14446724	1	nonDEG	0.09338454	0.80927118
nonDEG	-0.4265032	0.32936851	1	nonDEG	0.17598296	0.63825386
nonDEG	0.20996968	0.67431461	1	nonDEG	-0.0396078	0.92630842
nonDEG	-0.5653952	0.3442614	1	nonDEG	-0.0625177	0.89793514

nonDEG	0.7527253	0.37358789	1	nonDEG	-0.4755686	0.4335813
nonDEG	-0.1841291	0.69621699	1	nonDEG	0.15120113	0.68771515
nonDEG	-0.9119444	0.29459093	1	nonDEG	0.60774716	0.49659962
nonDEG	-0.5439229	0.24754472	1	nonDEG	0.7152735	0.10535864
nonDEG	-0.1478029	0.73204196	1	nonDEG	-0.0738771	0.83971865
nonDEG	-0.1936746	0.67177798	1	nonDEG	-0.4929104	0.27460297
nonDEG	-1.0898357	0.13857114	1	nonDEG	1.18949414	0.12849937
nonDEG	-1.6806103	0.0075584	0.75581042	nonDEG	0.55768766	0.40812158
nonDEG	-0.2787202	0.51864303	1	nonDEG	-0.0331544	0.9282224
nonDEG	-0.2555342	0.63382136	1	nonDEG	-0.1001373	0.8575362
nonDEG	0.50123717	0.25500527	1	nonDEG	-0.0180969	0.9603719
nonDEG	1.17273275	0.00919616	0.79328284	nonDEG	-0.5426551	0.13738271
nonDEG	1.94547754	0.03352767	1	nonDEG	0.62201635	0.38235582
nonDEG	-0.0912841	0.83985072	1	nonDEG	-0.678524	0.07712085
nonDEG	-1.8801602	0.06233709	1	nonDEG	1.18122764	0.3117687
nonDEG	1.09578307	0.06728382	1	nonDEG	-0.1970291	0.69529876
nonDEG	0.50168045	0.45497844	1	nonDEG	0.19385001	0.69790103
nonDEG	-0.7250028	0.25422291	1	nonDEG	0.98731812	0.06854039
nonDEG	0.67409747	0.17719935	1	nonDEG	-0.4424628	0.30732225
nonDEG	1.81225785	0.01294685	0.8619311	nonDEG	0.05248377	0.93771849
nonDEG	1.10475988	0.07564839	1	nonDEG	-1.4158543	0.00285237
nonDEG	0.22216646	0.6790658	1	nonDEG	-0.1087042	0.78829
nonDEG	-0.7716679	0.14792965	1	nonDEG	0.70487004	0.15809096
nonDEG	-0.4077151	0.35608094	1	nonDEG	-0.2198499	0.60572153
nonDEG	-0.4037609	0.38619042	1	nonDEG	0.04651991	0.91790057
nonDEG	-0.483789	0.28467735	1	nonDEG	0.04790728	0.90615799
nonDEG	3.44087093	0.00798707	0.76341314	nonDEG	-2.5292009	0.03895257
nonDEG	0	1	1	nonDEG	3.96671205	0.43604295
nonDEG	0.54073752	0.21811588	1	nonDEG	-0.1179775	0.75253889
nonDEG	-1.3598215	0.03418032	1	nonDEG	1.58321049	0.0326531
nonDEG	-0.1412454	0.74082615	1	nonDEG	-0.5011831	0.17799944
nonDEG	0.83823746	0.12166403	1	nonDEG	0.27165512	0.57055905
nonDEG	-0.4355716	0.40717505	1	nonDEG	-0.230647	0.58709914
nonDEG	0.88682024	0.04207579	1	nonDEG	-0.5374563	0.13660401
nonDEG	-0.23771	0.59722633	1	nonDEG	-0.1048077	0.78219805
nonDEG	-1.2555794	0.04058729	1	nonDEG	-0.7420681	0.23878996
nonDEG	-0.2325853	0.59815287	1	nonDEG	-0.2746147	0.45817577
nonDEG	0.99364886	0.18767942	1	nonDEG	-0.9692148	0.23633328
nonDEG	-0.4226588	0.32369644	1	nonDEG	0.65303449	0.12218916
nonDEG	0.43333133	0.37607355	1	nonDEG	-0.2697524	0.48307369
nonDEG	0.46412979	0.39755368	1	nonDEG	0.11558661	0.81356407
nonDEG	1.61774842	0.06171234	1	nonDEG	-0.9045584	0.05869054
nonDEG	-0.0207756	0.9620222	1	nonDEG	-0.0580649	0.87853966

nonDEG	0.10466077	0.818458	1	nonDEG	-0.706337	0.0719508
nonDEG	0.2440487	0.57952554	1	nonDEG	0.1094027	0.76379912
nonDEG	0.28906965	0.52709526	1	nonDEG	-0.168549	0.67719074
nonDEG	0.30213458	0.66186351	1	nonDEG	0.24454263	0.64213234
nonDEG	0.96481119	0.37019632	1	nonDEG	-1.4995976	0.21193573
nonDEG	-0.0185835	0.96691923	1	nonDEG	0.06101598	0.88210149
nonDEG	-1.6659534	0.00321582	0.65616368	nonDEG	-0.6358316	0.17832648
nonDEG	-0.3403772	0.45673051	1	nonDEG	0.12498228	0.73367222
nonDEG	-0.5398772	0.22609769	1	nonDEG	0.20103797	0.59174727
nonDEG	-0.5282885	0.2259007	1	nonDEG	0.35097071	0.33594708
nonDEG	0.67228878	0.33976343	1	nonDEG	-0.9575122	0.11869283
nonDEG	0.66230211	0.22187509	1	nonDEG	-0.2999925	0.47367158
nonDEG	1.57401127	0.00822739	0.77656086	nonDEG	-0.249861	0.60739297
nonDEG	-0.2871162	0.51947504	1	nonDEG	-0.1377364	0.7082166
nonDEG	-3.1914122	0.01135284	0.84185458	nonDEG	2.75242299	0.0091236
nonDEG	0.61957939	0.21837928	1	nonDEG	-0.5767503	0.15426613
nonDEG	0.7639979	0.07591985	1	nonDEG	-0.3162254	0.38124484
nonDEG	-0.4405054	0.30655894	1	nonDEG	0.57506645	0.1123455
nonDEG	-0.6021767	0.16612095	1	nonDEG	0.22386549	0.56247471
nonDEG	-0.3741479	0.41762495	1	nonDEG	0.523321	0.20719467
nonDEG	0.04155374	0.92293522	1	nonDEG	0.46649642	0.20941619
nonDEG	0.76267859	0.10529975	1	nonDEG	0.23457368	0.52398285
nonDEG	-0.1816615	0.7346242	1	nonDEG	0.01116368	0.98557993
nonDEG	0.90908946	0.24250753	1	nonDEG	-0.5918455	0.44050309
nonDEG	0.0476028	0.91624173	1	nonDEG	-0.6346787	0.10937933
nonDEG	1.52290922	0.06652703	1	nonDEG	-1.3153312	0.09949306
nonDEG	-1.2390197	0.04754742	1	nonDEG	0.7813207	0.20222414
nonDEG	2.2261588	0.00583424	0.73793371	nonDEG	-0.0127449	0.98634747
nonDEG	-0.6501941	0.49445394	1	nonDEG	0.24291403	0.83928514
nonDEG	1.01849244	0.1436304	1	nonDEG	-0.5444139	0.14975498
nonDEG	-1.4794118	0.01388326	0.88083911	nonDEG	0.72070327	0.12262489
nonDEG	0	1	1	nonDEG	7.23089863	0.03186161
nonDEG	0.78697728	0.2222256	1	nonDEG	0.06577183	0.91166755
nonDEG	-0.6180757	0.3610427	1	nonDEG	0.24503076	0.72761352
nonDEG	-0.5461121	0.23447395	1	nonDEG	0.01912599	0.96101896
nonDEG	-0.3724854	0.42790854	1	nonDEG	-0.3689638	0.3552878
nonDEG	-0.9446291	0.23375311	1	nonDEG	1.17461676	0.08677439
nonDEG	0.09141682	0.85323991	1	nonDEG	0.34716056	0.43310912
nonDEG	0.83189329	0.10123966	1	nonDEG	-0.6556833	0.07600016
nonDEG	-0.5740701	0.18026764	1	nonDEG	0.41063652	0.26337278
nonDEG	0.38981166	0.37297838	1	nonDEG	-0.5220859	0.15728628
nonDEG	0.67058233	0.15071206	1	nonDEG	-0.6444981	0.10968085
nonDEG	0.74976085	0.13508733	1	nonDEG	-0.1962171	0.59538713

nonDEG	-1.4017096	0.04233952	1	nonDEG	2.13465536	0.00308165
nonDEG	-0.4656585	0.46579984	1	nonDEG	0.6060997	0.31490306
nonDEG	0.74923561	0.43383764	1	nonDEG	-0.0081583	1
nonDEG	-0.6800716	0.12923184	1	nonDEG	0.20574015	0.58587333
nonDEG	0.28210387	0.50493273	1	nonDEG	-0.169853	0.63905379
nonDEG	1.16923751	0.17827162	1	nonDEG	-0.2289968	0.79578994
nonDEG	0.41202114	0.39510292	1	nonDEG	-0.5668097	0.18565316
nonDEG	-0.319149	0.46089957	1	nonDEG	-0.2185201	0.56410201
nonDEG	-0.8916253	0.08560542	1	nonDEG	0.45773287	0.31390583
nonDEG	0.56438866	0.24352035	1	nonDEG	-0.4615422	0.26689244
nonDEG	-0.167277	0.73696923	1	nonDEG	-0.3989396	0.40502977
nonDEG	6.43014531	0.00453671	0.68407511	nonDEG	-0.3714941	0.82612935
nonDEG	1.39505829	0.04756278	1	nonDEG	-0.3571935	0.5729266
nonDEG	-0.7312543	0.12861083	1	nonDEG	0.1385568	0.77499233
nonDEG	1.74225716	0.02009601	0.98541756	nonDEG	-0.3020013	0.62161891
nonDEG	-0.4132686	0.4313968	1	nonDEG	0.3620092	0.42604699
nonDEG	-1.4152254	0.06741303	1	nonDEG	2.02011311	0.02679856
nonDEG	-0.8721829	0.30815308	1	nonDEG	1.16879738	0.15321954
nonDEG	-0.2541466	0.56103366	1	nonDEG	-0.9415115	0.03622478
nonDEG	-0.1014377	0.81556924	1	nonDEG	-0.3094137	0.43053745
nonDEG	0.03497486	0.94550308	1	nonDEG	-0.2182606	0.5972769
nonDEG	0.74221727	0.3255734	1	nonDEG	-0.0320218	0.96248975
nonDEG	0.59326679	0.53269934	1	nonDEG	-1.1835151	0.09853929
nonDEG	0.55811951	0.19500863	1	nonDEG	-0.1726539	0.64445245
nonDEG	-0.6505826	0.3129784	1	nonDEG	0.12365487	0.84071491
nonDEG	0.38519388	0.51766187	1	nonDEG	-0.2512445	0.62023204
nonDEG	1.35355567	0.02258805	1	nonDEG	-0.3515689	0.51452262
nonDEG	0.60543452	0.19420322	1	nonDEG	-0.5211243	0.19041816
nonDEG	1.36541077	0.39525143	1	nonDEG	-4.3846388	0.03821598
DEG	0.68673886	0.11196179	1	nonDEG	0.82605372	0.02819582
nonDEG	0.29126898	0.61192989	1	nonDEG	0.07480813	0.88881745
nonDEG	1.63476585	0.00867731	0.7876977	nonDEG	0.25604957	0.63229417
nonDEG	-0.337969	0.42882388	1	nonDEG	0.19625865	0.60267555
nonDEG	0.0683218	0.91297473	1	nonDEG	0.18739067	0.74801375
nonDEG	0.25423128	0.66435019	1	nonDEG	0.17585402	0.72688197
nonDEG	-1.4962372	0.01724168	0.93133556	nonDEG	-0.3684402	0.34083269
nonDEG	-0.07318	0.88273246	1	nonDEG	-0.3048913	0.50166514
nonDEG	3.37539554	3.24E-05	0.12599308	nonDEG	0.00725753	0.99232703
nonDEG	0.82464075	0.13780009	1	nonDEG	0.09551475	0.80974317
nonDEG	0.52379399	0.46428024	1	nonDEG	-0.2249808	0.74475528
nonDEG	-0.1000249	0.85536678	1	nonDEG	0.29354597	0.56130629
nonDEG	2.17231773	0.00052261	0.44242691	nonDEG	-0.276041	0.56081543
nonDEG	-0.8898912	0.14158367	1	nonDEG	0.56859324	0.33361008

nonDEG	-0.3396709	0.48161407	1	nonDEG	-0.1952769	0.64220805
nonDEG	-0.0341276	0.93987881	1	nonDEG	0.53621388	0.18323251
nonDEG	-0.523576	0.3051231	1	nonDEG	-0.2629499	0.55914833
nonDEG	-2.5347392	0.08516006	1	nonDEG	3.16167305	0.03331492
nonDEG	-0.398268	0.34566744	1	nonDEG	0.48898952	0.17250264
nonDEG	-0.6436718	0.150863	1	nonDEG	-0.5797152	0.20456817
nonDEG	0.10693176	0.83022806	1	nonDEG	-0.0367557	0.92652923
nonDEG	1.16323003	0.22660473	1	nonDEG	-1.1115444	0.03500456
nonDEG	1.9073898	0.42893861	1	nonDEG	1.36168087	0.5749617
nonDEG	-0.6320121	0.19174921	1	nonDEG	0.49212648	0.20924865
nonDEG	0.94287478	0.26448902	1	nonDEG	0.1852708	0.82508285
nonDEG	-0.2817208	0.52797992	1	nonDEG	0.11501157	0.76815164
nonDEG	-1.4911328	0.13389142	1	nonDEG	1.99239675	0.01542302
nonDEG	-1.3673714	0.00739263	0.75263895	nonDEG	0.1743731	0.69862507
nonDEG	0.14883775	0.78755754	1	nonDEG	-0.1899955	0.63588644
nonDEG	-0.3566882	0.53018605	1	nonDEG	-0.1028693	0.83213236
nonDEG	-1.3856033	0.05350698	1	nonDEG	2.15105082	0.0067612
nonDEG	-0.9623251	0.16368561	1	nonDEG	0.52025955	0.37849189
nonDEG	-0.6105989	0.1993797	1	nonDEG	0.18676606	0.67272177
nonDEG	-1.5810009	0.03272986	1	nonDEG	2.26509952	0.02230205
nonDEG	-1.7179204	0.07615234	1	nonDEG	1.96479037	0.02141753
nonDEG	-0.2343832	0.58822132	1	nonDEG	-0.4446237	0.22650127
nonDEG	0.96221697	0.03084853	1	nonDEG	-0.2601519	0.49788393
nonDEG	1.07542815	0.49357556	1	nonDEG	-0.4170067	0.79280194
nonDEG	-1.1220907	0.19779705	1	nonDEG	1.16012473	0.12839432
nonDEG	-0.2553532	0.5592048	1	nonDEG	-0.0570937	0.89258245
nonDEG	-0.3294196	0.44113608	1	nonDEG	0.13900471	0.70448275
nonDEG	-0.6942761	0.21967529	1	nonDEG	0.40510009	0.45531703
nonDEG	-0.2614573	0.84724477	1	nonDEG	-1.3633766	0.56153058
nonDEG	-0.9424652	0.04009368	1	nonDEG	0.71237405	0.08548155
nonDEG	-0.5395654	0.25647329	1	nonDEG	0.58870782	0.17488645
nonDEG	1.21245485	0.02715315	1	nonDEG	-0.5258999	0.26271375
nonDEG	-1.4565414	0.22375761	1	nonDEG	1.98878525	0.04412855
nonDEG	-0.4502902	0.37267391	1	nonDEG	-0.3779009	0.37924364
nonDEG	-0.3705055	0.40992145	1	nonDEG	-0.3673368	0.37724549
nonDEG	-1.1658349	0.03444517	1	nonDEG	0.14141681	0.72854398
nonDEG	-0.7301911	0.52634677	1	nonDEG	0.47306062	0.66734871
nonDEG	-0.0587548	0.89472274	1	nonDEG	0.06314855	0.86963384
nonDEG	0.67781987	0.11202873	1	nonDEG	-0.6919999	0.06022963
nonDEG	-0.4283956	0.32458973	1	nonDEG	-0.1445519	0.69721397
nonDEG	1.60736974	0.01242012	0.85229819	nonDEG	-0.6221297	0.25712054
nonDEG	1.26160003	0.10505397	1	nonDEG	-0.4250159	0.56817775
nonDEG	3.50044567	0.00282587	0.62843199	nonDEG	0.56474411	0.53699323

nonDEG	-0.977505	0.18272231	1	nonDEG	0.37658297	0.63741918
nonDEG	-1.0359006	0.49406577	1	nonDEG	1.55712074	0.26589435
nonDEG	-0.5486242	0.2353193	1	nonDEG	0.54819021	0.15602183
nonDEG	-0.5571425	0.26296794	1	nonDEG	0.5306322	0.33770308
nonDEG	-1.7601621	0.44169388	1	nonDEG	1.43924794	0.52251844
nonDEG	0.71020818	0.09314945	1	nonDEG	-0.0141388	0.96918813
nonDEG	0.43478673	0.3759438	1	nonDEG	-0.3559377	0.36441012
nonDEG	0.26906448	0.61696937	1	nonDEG	0.0932768	0.85265472
nonDEG	-1.4899597	0.00202173	0.61198441	nonDEG	1.33062349	0.00735668
nonDEG	-0.9217206	0.13422849	1	nonDEG	0.25811673	0.66700959
nonDEG	-0.0212422	0.96157123	1	nonDEG	-0.2890109	0.43910573
nonDEG	-0.5446368	0.29293099	1	nonDEG	0.34679562	0.462109
nonDEG	1.22922824	0.02122847	0.99960842	nonDEG	-0.3914824	0.39419512
nonDEG	0.69444609	0.20869577	1	nonDEG	-0.3510253	0.36623772
nonDEG	-2.7678805	0.00280142	0.62843199	nonDEG	-0.5121087	0.4541883
nonDEG	1.30284142	0.0073746	0.75263895	nonDEG	-0.7447226	0.06731324
nonDEG	1.7815865	0.01241427	0.85229819	nonDEG	-0.8775248	0.05529795
nonDEG	-0.3386533	0.44777662	1	nonDEG	-0.1230271	0.73874751
nonDEG	-0.5529097	0.20854113	1	nonDEG	0.24056085	0.52095962
nonDEG	-0.1877718	0.67454523	1	nonDEG	-0.138725	0.74442262
nonDEG	1.13391488	0.10894303	1	nonDEG	-0.1407717	0.82541003
nonDEG	-2.3344266	0.00897355	0.79328284	nonDEG	1.45761499	0.05237818
nonDEG	-1.4271715	0.0066476	0.73793371	nonDEG	1.55122708	0.00093156
nonDEG	-0.184051	0.65987708	1	nonDEG	0.01690782	0.96348449
nonDEG	-0.141075	0.74758232	1	nonDEG	0.08560466	0.84483031
nonDEG	-0.425334	0.34522955	1	nonDEG	0.20516653	0.63240115
nonDEG	-0.5494522	0.25518891	1	nonDEG	-0.098888	0.81943621
nonDEG	0.88595566	0.37388235	1	nonDEG	-0.7461007	0.24691357
nonDEG	-3.0995883	0.01934708	0.97153571	nonDEG	1.62954723	0.3505019
nonDEG	0.13847779	0.78640875	1	nonDEG	-0.2146907	0.65311466
nonDEG	0.67709138	0.68750164	1	nonDEG	3.05627445	0.01138734
nonDEG	-0.2440387	0.56595653	1	nonDEG	0.15406448	0.67904175
nonDEG	0.77526885	0.14948768	1	nonDEG	-0.230028	0.6236713
nonDEG	0.57639922	0.40433467	1	nonDEG	-0.5746179	0.27489339
nonDEG	-0.3003188	0.55601707	1	nonDEG	-0.3832057	0.45100671
nonDEG	-0.8548358	0.07358101	1	nonDEG	-0.0434128	0.91338625
nonDEG	-1.1284858	0.16700643	1	nonDEG	1.60731623	0.05474305
nonDEG	-1.159843	0.03542633	1	nonDEG	0.98930445	0.05758728
nonDEG	1.12653243	0.07696312	1	nonDEG	-0.3090478	0.50900799
nonDEG	0.19411428	0.89689798	1	nonDEG	0.13829001	0.95073452
nonDEG	-0.4427215	0.29175636	1	nonDEG	0.44929812	0.21860239
nonDEG	0.96135318	0.07355811	1	nonDEG	0.01846898	0.96496968
nonDEG	-1.8455527	0.07982655	1	nonDEG	1.76950002	0.10129512

nonDEG	-0.3534552	0.43671238	1	nonDEG	0.48188906	0.22710318
nonDEG	-0.8210526	0.12474744	1	nonDEG	1.00457398	0.0324987
nonDEG	-0.3256897	0.49260259	1	nonDEG	-0.1939447	0.64717267
nonDEG	0.48380924	0.38355458	1	nonDEG	0.77186314	0.22801056
nonDEG	-0.9611984	0.04074389	1	nonDEG	0.58885344	0.15247476
nonDEG	-0.2396417	0.80898627	1	nonDEG	-0.3687702	0.75430308
nonDEG	-0.3358136	0.43613894	1	nonDEG	-0.2416386	0.50461851
nonDEG	-0.5482576	0.28740702	1	nonDEG	0.53292299	0.15608546
nonDEG	-1.1691104	0.0350336	1	nonDEG	0.75483108	0.18536606
nonDEG	-1.0552103	0.04943359	1	nonDEG	1.92145641	0.0007581
nonDEG	-1.217969	0.25549706	1	nonDEG	1.31308902	0.12598412
nonDEG	0.251491	0.5996182	1	nonDEG	-0.1975716	0.62223067
nonDEG	-0.5220403	0.24187734	1	nonDEG	0.77590052	0.05838375
nonDEG	-1.2913279	0.00718818	0.75263895	nonDEG	1.63735981	0.00490559
nonDEG	-0.8272667	0.15368032	1	nonDEG	-0.0871137	0.8901576
nonDEG	0.40462832	0.45420541	1	nonDEG	-0.2447389	0.54428393
nonDEG	0.25063942	0.61607038	1	nonDEG	0.23650061	0.59008388
nonDEG	1.46218507	0.06773829	1	nonDEG	-1.439906	0.02005008
nonDEG	-0.9467325	0.06779066	1	nonDEG	0.79350438	0.09849868
nonDEG	0.58645001	0.19134196	1	nonDEG	0.67506448	0.06463986
nonDEG	-0.2553959	0.56430384	1	nonDEG	-0.5249069	0.16321951
nonDEG	0.69618535	0.10441586	1	nonDEG	-0.8336575	0.02580036
nonDEG	-1.0871139	0.07556756	1	nonDEG	0.68870643	0.27044859
nonDEG	-0.4093671	0.38250225	1	nonDEG	0.40253343	0.27399742
nonDEG	0.68892582	0.25689137	1	nonDEG	0.21825213	0.69598455
nonDEG	0.96258606	0.04986562	1	nonDEG	0.08999002	0.81354788
nonDEG	-0.8619726	0.19915993	1	nonDEG	0.75717498	0.30945008
nonDEG	-0.405787	0.39969639	1	nonDEG	-0.6172926	0.21166844
nonDEG	0.55406989	0.21797804	1	nonDEG	-0.2435937	0.52471277
nonDEG	-1.4562801	0.00780794	0.75581042	nonDEG	0.8553029	0.05792233
nonDEG	0.25955207	0.58787941	1	nonDEG	0.04272869	0.90619592
nonDEG	1.34470093	0.08081102	1	nonDEG	-0.5067899	0.40435091
nonDEG	1.67590571	0.07373297	1	nonDEG	-1.9422878	0.0731931
nonDEG	-0.7501188	0.09449684	1	nonDEG	0.89239782	0.04472496
nonDEG	-0.8377473	0.32948242	1	nonDEG	1.15096575	0.16877258
nonDEG	-0.4584964	0.30658532	1	nonDEG	0.47472116	0.21512392
nonDEG	0.52956485	0.28009057	1	nonDEG	-0.2187292	0.56165825
nonDEG	-1.1057174	0.23727605	1	nonDEG	1.39173743	0.1185601
nonDEG	-0.3564419	0.42542687	1	nonDEG	0.31188189	0.39118893
nonDEG	0.44653208	0.34100638	1	nonDEG	-0.4852116	0.23911522
nonDEG	-0.665305	0.13532471	1	nonDEG	0.46996323	0.23650734
nonDEG	0.5646891	0.30375263	1	nonDEG	0.08003384	0.84953487
nonDEG	0.89761932	0.1102726	1	nonDEG	-1.0312585	0.02803895

nonDEG	0.1832418	0.69031319	1	nonDEG	-0.4587471	0.22114325
nonDEG	0.09699392	0.85655506	1	nonDEG	-0.5492799	0.19382946
nonDEG	-0.6707163	0.14510735	1	nonDEG	-0.0016421	0.99892104
nonDEG	1.35162794	0.1344876	1	nonDEG	-0.0365721	0.97180559
nonDEG	0.04953484	0.93140218	1	nonDEG	0.06514096	0.89889124
nonDEG	-0.7021166	0.21153401	1	nonDEG	0.91843698	0.06532651
nonDEG	0.48182285	0.30783543	1	nonDEG	0.06117589	0.88139696
nonDEG	-0.7132108	0.23051358	1	nonDEG	1.23529991	0.02615453
nonDEG	0.27255566	0.55412493	1	nonDEG	-0.8282328	0.03289129
nonDEG	-0.3968523	0.37445845	1	nonDEG	-0.0252789	0.94591117
nonDEG	0.19038362	0.73717632	1	nonDEG	0.16346983	0.7588036
nonDEG	-0.4295669	0.32614542	1	nonDEG	-0.4407279	0.23015073
nonDEG	0.50622034	0.25295858	1	nonDEG	-0.1300465	0.71847128
nonDEG	0.39799173	0.39856806	1	nonDEG	-0.5319198	0.17883718
nonDEG	1.94435283	0.00543866	0.72967447	nonDEG	-0.938654	0.13878602
nonDEG	0.63387683	0.1845088	1	nonDEG	-0.011178	0.97575992
nonDEG	0.72633515	0.25919873	1	nonDEG	-0.8417395	0.13397871
nonDEG	-1.2244556	0.0207436	0.99114571	nonDEG	1.34099809	0.01907593
nonDEG	1.89982547	0.01311168	0.86352262	nonDEG	-0.5065767	0.30747982
nonDEG	-1.6491709	0.00438317	0.68407511	nonDEG	1.90448501	0.00639571
nonDEG	1.92485099	0.02216123	1	nonDEG	-1.3812391	0.06088356
nonDEG	0.03785665	0.93440957	1	nonDEG	-0.9329207	0.02127835
nonDEG	0.64105856	0.40655329	1	nonDEG	-0.9868452	0.08357023
nonDEG	1.03571225	0.44221119	1	nonDEG	-0.0575484	0.98187359
nonDEG	-0.7607015	0.17895946	1	nonDEG	-0.2425808	0.6467955
nonDEG	0.80611405	0.12278377	1	nonDEG	-0.2205157	0.63201254
nonDEG	-0.1736421	0.69789377	1	nonDEG	0.15081115	0.68157987
nonDEG	-0.5299818	0.23775737	1	nonDEG	0.33858574	0.38556014
nonDEG	-0.4393184	0.29803009	1	nonDEG	0.05934118	0.8728779
nonDEG	-1.4192934	0.09326472	1	nonDEG	1.47198346	0.10565447
nonDEG	-1.8113967	0.0028774	0.62843199	nonDEG	1.4221079	0.00555958
nonDEG	-0.2129182	0.62724392	1	nonDEG	-0.1568419	0.66971833
nonDEG	0.4774898	0.25967319	1	nonDEG	-0.1849331	0.60657721
nonDEG	-0.2856754	0.77188533	1	nonDEG	0.04956812	0.98300678
nonDEG	-1.1904456	0.01377501	0.87965814	nonDEG	1.24408612	0.00947604
nonDEG	0.49301599	0.301348	1	nonDEG	-0.224798	0.55558821
nonDEG	-0.7855821	0.13543605	1	nonDEG	0.3164943	0.44816583
nonDEG	0.37964727	0.43760048	1	nonDEG	-0.0135269	0.97234336
nonDEG	-0.4334487	0.44341762	1	nonDEG	0.00299967	0.99777225
nonDEG	-0.0435326	0.92327309	1	nonDEG	-0.7688857	0.04900501
nonDEG	0.48674202	0.28971408	1	nonDEG	-0.059273	0.8813838
nonDEG	1.64502148	0.01860258	0.95407879	nonDEG	0.03831356	0.95064604
nonDEG	-0.9060238	0.0686688	1	nonDEG	0.42736365	0.31269498

nonDEG	0.48366084	0.27633205	1	nonDEG	-0.0746846	0.84600428
nonDEG	-1.0504255	0.05477684	1	nonDEG	1.08647191	0.05320535
nonDEG	0.84140372	0.04616741	1	nonDEG	-0.1553226	0.67258562
nonDEG	-0.3708101	0.41294082	1	nonDEG	-0.4191332	0.27238368
nonDEG	-0.1437995	0.78842108	1	nonDEG	-0.1081195	0.82311298
nonDEG	0.71051257	0.17168701	1	nonDEG	-0.4298938	0.34578664
nonDEG	0.43812309	0.71079167	1	nonDEG	1.48379126	0.20576078
nonDEG	-2.3111708	0.01203478	0.85229819	nonDEG	1.79765565	0.07916186
nonDEG	-0.2533965	0.57855168	1	nonDEG	-0.0312796	0.93144825
nonDEG	-1.7672679	0.0237374	1	nonDEG	0.44941299	0.43625336
nonDEG	-0.3344208	0.51476571	1	nonDEG	-0.3247211	0.50977595
nonDEG	0.15891405	0.73278597	1	nonDEG	0.05341881	0.89915843
nonDEG	-1.0464763	0.11254191	1	nonDEG	0.4098648	0.46952073
nonDEG	1.22832594	0.14535125	1	nonDEG	-0.9598527	0.04550002
nonDEG	0.32186801	0.57793996	1	nonDEG	0.32816845	0.46869362
nonDEG	0.40350857	0.48103333	1	nonDEG	-0.0369206	0.9496535
nonDEG	0.12166679	0.85461327	1	nonDEG	0.25990065	0.66957879
nonDEG	-1.7313044	0.00268605	0.61776954	nonDEG	1.75828754	0.00136687
nonDEG	-0.1108491	0.8184903	1	nonDEG	-0.0011625	0.99964977
nonDEG	-3.8367142	0.46909333	1	nonDEG	-5.13E-15	1
nonDEG	-0.3887992	0.44236071	1	nonDEG	-0.0821192	0.85708953
nonDEG	0.45585157	0.32472745	1	nonDEG	-0.0178808	0.96127237
nonDEG	-0.3483782	0.41665889	1	nonDEG	0.04671205	0.8992214
nonDEG	0.91580419	0.10451171	1	nonDEG	-0.0123935	0.9739412
nonDEG	0.60505335	0.30102828	1	nonDEG	-1.2255898	0.01000356
nonDEG	0.31548251	0.49201433	1	nonDEG	-0.0740656	0.85447003
nonDEG	0.81286679	0.1015841	1	nonDEG	-0.394659	0.3483657
nonDEG	-0.5832315	0.21890222	1	nonDEG	0.39983883	0.3249835
nonDEG	0.55233252	0.30893305	1	nonDEG	-0.4660544	0.25530235
nonDEG	0.73042105	0.34132537	1	nonDEG	-0.7597606	0.42461564
nonDEG	-1.631278	0.2618942	1	nonDEG	0.64486917	0.73056467
nonDEG	0.35537592	0.42959254	1	nonDEG	-0.0831615	0.83777961
nonDEG	-0.460717	0.32062995	1	nonDEG	-0.1947905	0.61668022
nonDEG	0.67759751	0.11166774	1	nonDEG	-0.0460774	0.89911869
nonDEG	0.7536169	0.24404442	1	nonDEG	-0.8768746	0.0947066
nonDEG	-0.5157326	0.26595652	1	nonDEG	0.49103467	0.26517653
nonDEG	2.24797544	0.00735202	0.75263895	nonDEG	-0.1614601	0.84518327
nonDEG	0.03602809	0.9694975	1	nonDEG	0.0647123	0.92290364
nonDEG	-0.4505831	0.34880852	1	nonDEG	-0.1927702	0.65058955
nonDEG	0.21893021	0.61816905	1	nonDEG	-0.2284423	0.53591637
nonDEG	-1.7849029	0.04279216	1	nonDEG	1.871074	0.05769021
nonDEG	1.50156465	0.00671997	0.74093303	nonDEG	-0.7671902	0.13207701
nonDEG	-0.0139954	0.98866839	1	nonDEG	-0.9145076	0.28654544

nonDEG	3.11012841	0.11923303	1	nonDEG	-0.8424986	0.72179548
nonDEG	-0.7359764	0.12208691	1	nonDEG	0.40844889	0.34139468
nonDEG	-0.2972651	0.50771743	1	nonDEG	0.32614592	0.43119148
nonDEG	0.55026533	0.37188846	1	nonDEG	-0.5033552	0.31573447
nonDEG	0.06580315	0.8894205	1	nonDEG	-0.0013804	0.99926742
nonDEG	-2.0029848	0.0092966	0.79328284	nonDEG	1.33332519	0.02537518
nonDEG	-0.7497551	0.29506829	1	nonDEG	1.23311158	0.07561205
nonDEG	-1.4250978	0.17260274	1	nonDEG	1.35783502	0.21441928
nonDEG	-1.4479749	0.03600942	1	nonDEG	0.44517985	0.59121015
nonDEG	-0.7214751	0.10305327	1	nonDEG	0.59857442	0.13968609
nonDEG	-0.6851552	0.14839313	1	nonDEG	0.52998062	0.17788987
nonDEG	0.23459744	0.63661984	1	nonDEG	-0.087873	0.814313
nonDEG	-0.4109479	0.34601001	1	nonDEG	0.01969597	0.95770621
nonDEG	-1.2915772	0.0675958	1	nonDEG	1.15912428	0.15937736
nonDEG	-0.7235166	0.14254075	1	nonDEG	0.28944224	0.44744351
nonDEG	-0.4761337	0.282087	1	nonDEG	0.37303738	0.33592432
nonDEG	-0.3447267	0.43101586	1	nonDEG	-0.2897858	0.47666086
nonDEG	-0.0336297	0.9393112	1	nonDEG	-0.1917703	0.60896169
nonDEG	0.33079653	0.56472289	1	nonDEG	-0.1742019	0.70824486
nonDEG	-0.7085843	0.22689725	1	nonDEG	0.8106477	0.19978794
nonDEG	-1.8269578	0.01354683	0.87457296	nonDEG	1.64397712	0.003461
nonDEG	0.45213557	0.42425694	1	nonDEG	-0.036971	0.94343223
nonDEG	0.25068526	0.64305808	1	nonDEG	0.33535013	0.49166278
nonDEG	0.25812181	1	1	nonDEG	1.21180459	0.48824983
nonDEG	-0.5870076	0.31284445	1	nonDEG	0.51909555	0.33524728
nonDEG	1.10180642	0.01359465	0.87573927	nonDEG	-0.2328225	0.52117509
nonDEG	-0.7345483	0.34468495	1	nonDEG	0.24252544	0.86602673
nonDEG	1.50119786	0.00869601	0.7876977	nonDEG	-0.542249	0.18700024
nonDEG	0.62400049	0.37278238	1	nonDEG	-0.2699995	0.55716172
nonDEG	-0.5277788	0.24194026	1	nonDEG	0.29313205	0.42077389
nonDEG	0.38332237	0.53938336	1	nonDEG	-1.3291647	0.01260396
nonDEG	0.61826812	0.21870565	1	nonDEG	0.20762387	0.63719942
nonDEG	-0.4235475	0.3688385	1	nonDEG	-0.0763864	0.83779933
nonDEG	1.73172711	0.02022497	0.98576638	nonDEG	-0.328552	0.59207569
nonDEG	1.1924364	0.01703256	0.93079586	nonDEG	-0.0790869	0.85447085
nonDEG	-0.0433498	0.92076286	1	nonDEG	0.06394708	0.86116243
nonDEG	2.41558529	0.01591851	0.91172155	nonDEG	-0.7361743	0.38554556
nonDEG	-0.0039019	1	1	nonDEG	0.57527509	0.48867584
nonDEG	7.22981172	0.0004637	0.41795603	nonDEG	-1.7574647	0.19945011
nonDEG	0.0505704	0.92336469	1	nonDEG	-0.6255064	0.16173909
nonDEG	-0.9697354	0.03170254	1	nonDEG	1.35056265	0.00876864
nonDEG	-0.637046	0.17005492	1	nonDEG	0.58792944	0.13945947
nonDEG	-0.0376082	0.96567729	1	nonDEG	0.08779981	0.93871002

nonDEG	0.67165599	0.13369519	1	nonDEG	-0.4728258	0.21898859
nonDEG	0.83567381	0.38662955	1	nonDEG	-0.7483573	0.49633649
nonDEG	-0.3315187	0.48416612	1	nonDEG	0.41673595	0.25445691
nonDEG	-0.1400877	0.75517267	1	nonDEG	-0.7142117	0.06812636
nonDEG	-2.4974375	0.0112314	0.84185458	nonDEG	2.35480465	0.00636165
nonDEG	-0.2728934	0.6231956	1	nonDEG	0.27424483	0.59182357
nonDEG	-0.0919075	0.8352747	1	nonDEG	-0.5225895	0.16657978
nonDEG	1.05667287	0.24607115	1	nonDEG	-0.5030185	0.55806041
nonDEG	0.69312382	0.22108635	1	nonDEG	-0.4976119	0.34619468
nonDEG	-0.2566084	0.54609249	1	nonDEG	0.30568831	0.41249594
nonDEG	-1.5054039	0.02813656	1	nonDEG	1.98037146	0.00807468
nonDEG	1.19230165	0.12306026	1	nonDEG	-0.3109303	0.67892516
nonDEG	0.15533275	0.75481801	1	nonDEG	-0.3786341	0.42425603
nonDEG	-0.5001571	0.51133833	1	nonDEG	0.95765132	0.19092653
nonDEG	-2.0609517	0.03361885	1	nonDEG	1.22704352	0.24670911
nonDEG	1.68096024	0.09382614	1	nonDEG	-0.3907485	0.60128291
nonDEG	0.06547567	0.88948622	1	nonDEG	-1.1721889	0.00477267
nonDEG	-0.5261601	0.22411894	1	nonDEG	0.10347259	0.78596098
nonDEG	-0.4914089	0.40929869	1	nonDEG	0.23647166	0.71196079
nonDEG	-1.3381394	0.02078018	0.99114571	nonDEG	1.50320587	0.01156299
nonDEG	-0.920855	0.26882978	1	nonDEG	1.30074188	0.09302076
nonDEG	-0.3664235	0.43637849	1	nonDEG	0.0486404	0.90160498
nonDEG	-0.3107508	0.47608559	1	nonDEG	0.16754387	0.64885771
nonDEG	0.6542922	0.20893751	1	nonDEG	-0.2411575	0.52339118
nonDEG	-0.232487	0.59025067	1	nonDEG	-0.4093498	0.28945794
nonDEG	-0.2445465	0.58604543	1	nonDEG	-0.0267409	0.94996626
nonDEG	0.59944301	0.25111133	1	nonDEG	-0.2440416	0.52722081
nonDEG	-1.4129451	0.01823485	0.94395982	nonDEG	1.79783754	0.00301196
nonDEG	0.83609521	0.15612036	1	nonDEG	0.03338825	0.95132149
nonDEG	-0.4583988	0.34182753	1	nonDEG	0.62892036	0.14101715
nonDEG	0.41235239	0.45405837	1	nonDEG	0.46447447	0.33743751
nonDEG	-1.2883434	0.07280328	1	nonDEG	0.18594893	0.73302306
nonDEG	1.61778849	0.0244886	1	nonDEG	-0.3844283	0.47123475
nonDEG	0.44235906	0.3370138	1	nonDEG	-0.3202231	0.38230467
nonDEG	1.97655091	0.02071834	0.99114571	nonDEG	-0.3659982	0.65865822
nonDEG	1.46621422	0.05096535	1	nonDEG	0.21855924	0.73224568
nonDEG	-0.5812289	0.21151201	1	nonDEG	0.23133345	0.55372015
nonDEG	0.78130412	0.54548404	1	nonDEG	0.00803671	1
nonDEG	0.911085	0.07704507	1	nonDEG	-0.3227553	0.48171782
nonDEG	0.69780418	0.23718166	1	nonDEG	-0.4636188	0.36663876
nonDEG	-0.9084042	0.0924721	1	nonDEG	0.76527787	0.11063246
nonDEG	-1.201966	0.02493184	1	nonDEG	1.24471653	0.00268226
nonDEG	-1.6400972	0.0452886	1	nonDEG	-0.3614742	0.70569181

nonDEG	-0.006163	0.98948205	1	nonDEG	-0.4124974	0.2817444
nonDEG	-0.4095427	0.38160668	1	nonDEG	0.10168429	0.82128705
nonDEG	-1.2821176	0.01475417	0.89005739	nonDEG	1.43123475	0.00114225
nonDEG	-0.9752459	0.11572324	1	nonDEG	1.71189881	0.00397912
nonDEG	0.29834309	0.53475911	1	nonDEG	-0.1412347	0.74639602
nonDEG	1.55450922	0.09265369	1	nonDEG	-0.351089	0.67212161
nonDEG	-1.4704414	0.03103673	1	nonDEG	1.69447059	0.01665969
nonDEG	0.68713247	0.34979749	1	nonDEG	-1.0421018	0.16887138
nonDEG	-1.0431789	0.02610834	1	nonDEG	-0.4009074	0.32819564
nonDEG	-0.6154839	0.17712142	1	nonDEG	0.84014567	0.02426874
nonDEG	0.58176192	0.25572745	1	nonDEG	-0.6339687	0.11394946
nonDEG	1.05815465	0.02198013	1	nonDEG	-0.0413963	0.9165436
nonDEG	0.18177499	0.76784136	1	nonDEG	-0.5606366	0.39857038
nonDEG	-0.3053673	0.50325151	1	nonDEG	0.06964322	0.86832446
nonDEG	1.86754768	0.00252641	0.61776954	nonDEG	-0.7640636	0.06850272
nonDEG	0.46380852	0.29553914	1	nonDEG	0.14731869	0.71795087
nonDEG	0.46413646	0.34024485	1	nonDEG	-0.3480365	0.42654975
nonDEG	0.28195989	0.5617157	1	nonDEG	-0.0919272	0.83256899
nonDEG	-0.8130057	0.07772823	1	nonDEG	0.96131973	0.0183813
nonDEG	-1.2885454	0.15795527	1	nonDEG	1.31781115	0.17331163
nonDEG	2.41450438	0.03259361	1	nonDEG	0.15975951	0.8098266
nonDEG	-6.5018122	5.14E-06	0.03026052	DEG	6.18667208	0.0002126
nonDEG	2.03907254	0.00037857	0.3842997	nonDEG	-0.1234493	0.80671273
nonDEG	-0.4947817	0.29075268	1	nonDEG	0.66620948	0.11160887
nonDEG	-0.6144195	0.1734231	1	nonDEG	0.54676917	0.15139627
nonDEG	-0.0119345	0.99245293	1	nonDEG	-0.975824	0.22015582
nonDEG	0.13054433	0.79410204	1	nonDEG	0.15938577	0.66285023
nonDEG	0.05761865	0.8971575	1	nonDEG	0.3070637	0.39679204
nonDEG	0.07756475	0.88399472	1	nonDEG	0.18592465	0.68697097
nonDEG	0.02852503	0.94821156	1	nonDEG	-0.2187426	0.5793018
nonDEG	-0.1350399	0.74888017	1	nonDEG	-0.1946145	0.59048247
nonDEG	-0.0005161	1	1	nonDEG	0.15269375	0.74565044
nonDEG	-1.9206935	0.00206427	0.61198441	nonDEG	2.50406282	0.00042941
nonDEG	-0.1652175	0.71016487	1	nonDEG	-0.628254	0.09588251
nonDEG	-0.110352	0.84728396	1	nonDEG	1.07250096	0.02603401
nonDEG	-0.3990661	0.4080039	1	nonDEG	0.25648154	0.56343352
nonDEG	1.43766977	0.00582533	0.73793371	nonDEG	-0.2093898	0.63863747
nonDEG	0.77032697	0.09296751	1	nonDEG	-0.343612	0.34300383
nonDEG	-2.2035119	0.09939024	1	nonDEG	2.5038465	0.08282917
nonDEG	-0.9399985	0.07956125	1	nonDEG	1.222397	0.0352137
nonDEG	0.50874606	0.29158008	1	nonDEG	-0.4505844	0.25943307
nonDEG	-0.9693802	0.02792367	1	nonDEG	0.59841954	0.12034187
nonDEG	1.21307836	0.09754032	1	nonDEG	0.06602575	0.9106385

nonDEG	-0.0001177	1	1	nonDEG	0.5794021	0.28426009
nonDEG	-1.0360232	0.08293726	1	nonDEG	1.5209616	0.0050498
nonDEG	0.80460022	0.06747268	1	nonDEG	-0.135121	0.70877128
nonDEG	-1.3058586	0.02693971	1	nonDEG	1.74754502	0.00061229
nonDEG	0.31922914	0.47815911	1	nonDEG	-0.1649726	0.66077764
nonDEG	0.4025186	0.40344941	1	nonDEG	-0.3564471	0.39867597
nonDEG	-1.1141534	0.1329675	1	nonDEG	1.39868369	0.0393671
nonDEG	2.00629861	0.01757751	0.93380915	nonDEG	-0.7095113	0.09338795
nonDEG	0.58239704	0.36403355	1	nonDEG	0.28631386	0.61545341
nonDEG	0.75038902	0.09292833	1	nonDEG	-0.0264122	0.94286261
nonDEG	-0.1315074	0.76187676	1	nonDEG	0.29021338	0.44204721
nonDEG	1.57372053	0.00084497	0.50181787	nonDEG	-1.1234494	0.00419804
nonDEG	-1.6911921	0.30891335	1	nonDEG	2.41634836	0.16925501
nonDEG	-1.9065824	0.0306063	1	nonDEG	3.01017153	5.23E-06
nonDEG	-0.7267439	0.10697606	1	nonDEG	0.71564946	0.06353599
nonDEG	0.23395621	0.590308	1	nonDEG	-0.2435426	0.51386611
nonDEG	0.35477462	0.42716843	1	nonDEG	-0.2008595	0.58024065
nonDEG	-0.1512277	0.72539403	1	nonDEG	0.06040901	0.87149056
nonDEG	2.7705026	0.00920559	0.79328284	nonDEG	-0.992733	0.32423441
nonDEG	-0.4386043	0.29483148	1	nonDEG	0.20275281	0.58063412
nonDEG	0.38814366	0.4015083	1	nonDEG	-0.3120902	0.40894897
nonDEG	1.45204475	0.02877645	1	nonDEG	-0.0954954	0.86767635
nonDEG	1.30171119	0.11867406	1	nonDEG	-0.4383476	0.59096825
nonDEG	-1.4443697	0.056508	1	nonDEG	1.29333792	0.03670787
nonDEG	-0.2299367	0.59848311	1	nonDEG	-0.1724679	0.66584671
nonDEG	0.72384351	0.16779719	1	nonDEG	-0.1674908	0.70099245
nonDEG	0.52560888	0.22280014	1	nonDEG	0.06985991	0.84971476
nonDEG	0.58996956	0.19635283	1	nonDEG	-0.0959544	0.80525073
nonDEG	-2.2266546	0.00198435	0.61198441	nonDEG	2.27267798	0.00914053
nonDEG	0.47049108	0.29001997	1	nonDEG	-0.4205957	0.24098816
nonDEG	-0.5561233	0.22985929	1	nonDEG	-0.1761615	0.68124144
nonDEG	0.61802742	0.32696124	1	nonDEG	-0.2154481	0.5698384
nonDEG	0.06724841	0.88886447	1	nonDEG	-0.5043862	0.23571656
nonDEG	0.07390544	0.86427213	1	nonDEG	0.06977376	0.84939175
nonDEG	0.36576239	0.50180926	1	nonDEG	-0.6556973	0.12093411
nonDEG	0.88359155	0.36788315	1	nonDEG	-0.5953295	0.5119394
nonDEG	0.50815608	0.36059114	1	nonDEG	-0.266713	0.59826349
nonDEG	1.42563062	0.07312161	1	nonDEG	0.03689681	0.95759117
nonDEG	-0.2657049	0.56142858	1	nonDEG	-0.2500658	0.54983961
nonDEG	-1.6067916	0.00292453	0.62843199	nonDEG	-0.8157103	0.03080755
nonDEG	0.87352546	0.05513346	1	nonDEG	-0.5308624	0.14583492
nonDEG	0.23935151	0.63093382	1	nonDEG	-0.1204684	0.76866796
nonDEG	-1.3375583	0.01710434	0.93079586	nonDEG	1.15929661	0.03473316

nonDEG	-0.8474212	0.31472654	1	nonDEG	0.81190554	0.24370053
nonDEG	-0.5586283	0.22339776	1	nonDEG	0.26873254	0.48468958
nonDEG	-0.775092	0.3087679	1	nonDEG	0.31567615	0.69425567
nonDEG	0.20577542	0.67353628	1	nonDEG	-0.0303634	0.9443036
nonDEG	-1.0902869	0.04710214	1	nonDEG	1.42789985	0.01585191
nonDEG	0.74415831	0.09430131	1	nonDEG	-0.9781934	0.00901347
nonDEG	-0.8427728	0.09831559	1	nonDEG	0.5594535	0.20148107
nonDEG	1.66102007	0.02631274	1	nonDEG	-1.4715348	0.06109048
nonDEG	-0.7151165	0.30110254	1	nonDEG	0.01341619	0.99248697
nonDEG	0.44387979	0.30675747	1	nonDEG	0.17202876	0.652068
nonDEG	-1.4114473	0.10855022	1	nonDEG	1.45528749	0.13529781
DEG	1.13645959	0.22479777	1	nonDEG	1.59827848	0.0439059
nonDEG	2.77250258	0.00129335	0.52845676	nonDEG	-1.0883189	0.15863956
nonDEG	0.0071797	0.98698483	1	nonDEG	0.28226084	0.43998183
nonDEG	-0.8083016	0.29605382	1	nonDEG	-0.2955471	0.61655726
nonDEG	2.796342	0.00126048	0.52845676	nonDEG	-0.9480629	0.22050848
nonDEG	-2.3085735	0.02506051	1	nonDEG	1.72869864	0.08887665
nonDEG	1.66441317	0.00082075	0.50181787	nonDEG	-0.1197307	0.78661352
nonDEG	-0.786621	0.06922096	1	nonDEG	0.4422041	0.25549201
nonDEG	1.71633145	0.02361862	1	nonDEG	-0.4608006	0.47153312
nonDEG	0.28703097	0.51162978	1	nonDEG	0.07567553	0.83856359
nonDEG	-0.3382417	0.59610728	1	nonDEG	0.62627221	0.23350203
nonDEG	-0.6121439	0.39562714	1	nonDEG	0.62707756	0.36581802
nonDEG	1.2272074	0.09963488	1	nonDEG	-0.9812479	0.02561162
nonDEG	0.34253921	0.53759622	1	nonDEG	-0.1376845	0.75174538
nonDEG	0.27997137	0.8179223	1	nonDEG	-1.1426861	0.3519175
nonDEG	0.28448379	0.58674527	1	nonDEG	0.00755582	0.98798896
nonDEG	-0.4190682	0.34173398	1	nonDEG	-0.6256153	0.10043045
nonDEG	2.04623565	0.12323382	1	nonDEG	-1.6454863	0.06693192
nonDEG	0.1373341	0.80157073	1	nonDEG	0.52922387	0.27093062
nonDEG	-0.9069786	0.04542805	1	nonDEG	1.00536273	0.00845645
nonDEG	-0.5712576	0.52003421	1	nonDEG	-0.6801829	0.53639367
nonDEG	1.10479718	0.18875908	1	nonDEG	-0.700348	0.25343095
nonDEG	0.66900764	0.1636375	1	nonDEG	-0.3206092	0.46161706
nonDEG	-0.3119733	0.46858739	1	nonDEG	0.10029109	0.793018
nonDEG	-0.2157015	0.74306731	1	nonDEG	0.6589356	0.25569258
nonDEG	-0.5197681	0.51483305	1	nonDEG	0.33828262	0.68227075
nonDEG	0.4302517	0.41861113	1	nonDEG	-0.5417141	0.17700063
nonDEG	-0.239578	0.58534017	1	nonDEG	-0.4716473	0.20148332
nonDEG	-0.1095816	0.81851704	1	nonDEG	0.0039761	0.99210954
nonDEG	-0.1006492	0.90016517	1	nonDEG	-0.5121076	0.44118465
nonDEG	-0.3749446	0.42010063	1	nonDEG	-0.111228	0.80184781
nonDEG	-0.3265972	0.52689736	1	nonDEG	-0.5232201	0.32835357

nonDEG	0.90709542	0.24741291	1	nonDEG	-0.3273301	0.53282391
nonDEG	-0.4582069	0.31440731	1	nonDEG	0.39488758	0.31925575
DEG	-2.4171488	3.24E-07	0.00952827	DEG	2.28970289	0.00095235
nonDEG	0.90801005	0.12824218	1	nonDEG	-0.5912699	0.19916474
nonDEG	0.93287273	0.12684182	1	nonDEG	-0.2167223	0.61438504
nonDEG	1.13707506	0.01482314	0.89056822	nonDEG	-0.4396356	0.22024472
nonDEG	-0.0410916	0.92549397	1	nonDEG	-0.053948	0.88313169
nonDEG	0.3815678	0.44679326	1	nonDEG	-0.4523644	0.27335348
nonDEG	0.93629644	0.05424466	1	nonDEG	0.05986381	0.88565684
nonDEG	-0.5974794	0.29686943	1	nonDEG	-0.1649214	0.74246923
nonDEG	-0.8747852	0.05061325	1	nonDEG	0.44571641	0.24261455
nonDEG	-0.1751696	0.69451418	1	nonDEG	-0.0201351	0.95862766
nonDEG	-0.5143538	0.23662793	1	nonDEG	0.18775319	0.62753337
nonDEG	0.87409418	0.11432855	1	nonDEG	-0.6394719	0.20163834
nonDEG	0.96111996	0.02494458	1	nonDEG	-0.5999733	0.10774323
nonDEG	-1.4261357	0.00787451	0.75757446	nonDEG	1.79355023	0.00501325
nonDEG	1.82002985	0.0284669	1	nonDEG	-2.0688519	0.00980954
nonDEG	0.03797094	0.93157543	1	nonDEG	-0.1644686	0.669471
nonDEG	0.98294543	0.06722757	1	nonDEG	-0.4748879	0.32589083
nonDEG	-0.2843717	1	1	nonDEG	-1.0749688	0.85739536
nonDEG	-0.5917891	0.17472253	1	nonDEG	0.77800896	0.07985962
nonDEG	1.52041463	0.14625435	1	nonDEG	0.74053502	0.43142789
nonDEG	0.43751849	0.31526205	1	nonDEG	-0.1497073	0.67614082
nonDEG	0.31815999	0.65630862	1	nonDEG	-0.3808638	0.59838831
nonDEG	0.03485721	1	1	nonDEG	0.85107692	0.8074171
nonDEG	2.30974126	0.03239715	1	nonDEG	0.4291346	0.61677904
nonDEG	1.63990068	0.36794723	1	nonDEG	1.54796499	0.42813015
nonDEG	-1.263628	0.02405695	1	nonDEG	1.48428236	0.02299103
nonDEG	-0.1299194	0.78948313	1	nonDEG	0.08092647	0.85124352
nonDEG	0.03796774	0.93032394	1	nonDEG	-0.0561362	0.87824552
nonDEG	0.81322514	0.24518274	1	nonDEG	-0.0006312	1
nonDEG	0.36363149	0.43769118	1	nonDEG	-0.4198799	0.25709718
nonDEG	-0.9759009	0.13450313	1	nonDEG	0.49150237	0.46494465
nonDEG	0.29983122	0.58679742	1	nonDEG	-0.0879298	0.84863216
nonDEG	0.08363036	0.86791383	1	nonDEG	-0.4485857	0.28623469
nonDEG	2.22844258	0.01647835	0.91876144	nonDEG	0.19664523	0.81048772
nonDEG	0.73710631	0.11703892	1	nonDEG	-0.4687461	0.20585688
nonDEG	-0.9838263	0.06694398	1	nonDEG	0.98431875	0.04445663
nonDEG	-0.3941064	0.43927383	1	nonDEG	-0.2268285	0.58234635
nonDEG	2.18059016	0.00185668	0.61198441	nonDEG	-0.244554	0.67747141
nonDEG	0.79911974	0.2685886	1	nonDEG	-0.3277087	0.64444529
nonDEG	1.90660192	0.07429012	1	nonDEG	0.16680669	0.89629425
nonDEG	0.91077236	0.36791998	1	nonDEG	-0.0489251	0.96825339

nonDEG	0.29076521	0.71868437	1	nonDEG	-0.8174789	0.29188459
nonDEG	1.20257902	0.00959171	0.79482907	nonDEG	-0.5755173	0.14780192
nonDEG	-0.535542	0.21977558	1	nonDEG	0.38028859	0.29468785
nonDEG	2.29441531	0.20984466	1	nonDEG	0.67847879	0.7165096
nonDEG	-1.4171748	0.08632609	1	nonDEG	0.02164722	1
nonDEG	-1.8871989	0.03314376	1	nonDEG	1.06159274	0.07589804
nonDEG	-1.9873239	0.02825145	1	nonDEG	1.54262106	0.0437129
nonDEG	0.57479635	0.33303049	1	nonDEG	-0.3128317	0.56544857
nonDEG	0.61551544	0.19385386	1	nonDEG	-0.3071708	0.41293797
nonDEG	-3.8057721	0.00666418	0.73793371	nonDEG	4.90829902	0.00269638
nonDEG	-1.3014636	0.08933843	1	nonDEG	0.12871738	0.81186422
nonDEG	0.41347734	0.49063075	1	nonDEG	0.70311305	0.17687685
nonDEG	-0.4008893	0.42944137	1	nonDEG	-0.2916545	0.55075386
nonDEG	-1.3144925	0.07304561	1	nonDEG	1.47878181	0.05561221
nonDEG	0.67285293	0.37206483	1	nonDEG	-0.8949706	0.08455236
nonDEG	-0.7848888	0.07044055	1	nonDEG	0.57372144	0.10999384
nonDEG	0.64362076	0.34993672	1	nonDEG	0.61331316	0.30698031
nonDEG	0.43097904	0.32899846	1	nonDEG	-0.3962438	0.27658751
nonDEG	0.46225823	0.51370632	1	nonDEG	-0.3125971	0.59505944
nonDEG	-0.4275173	0.31606002	1	nonDEG	-0.2114684	0.55975315
nonDEG	0.27276725	0.58906911	1	nonDEG	0.20489864	0.64630777
nonDEG	1.44802227	0.08407602	1	nonDEG	-1.6040913	8.15E-05
nonDEG	-0.9127945	0.1382423	1	nonDEG	0.71611566	0.19843218
nonDEG	-0.3755576	0.49412581	1	nonDEG	-0.336231	0.50313176
nonDEG	1.40675281	0.02234172	1	nonDEG	-0.419137	0.40371502
nonDEG	-0.365902	0.4601794	1	nonDEG	0.31318688	0.45852233
nonDEG	1.21722657	0.34751157	1	nonDEG	0.62389279	0.61233904
nonDEG	-0.051792	0.90729649	1	nonDEG	0.1756394	0.6522018
DEG	1.4719824	0.00428538	0.68407511	nonDEG	-0.6151101	0.17890119
nonDEG	-2.0521406	0.01467377	0.89005739	nonDEG	2.21878112	0.00011351
nonDEG	0.00671266	1	1	nonDEG	0.57553466	0.57807574
nonDEG	-0.1629132	0.71253059	1	nonDEG	-0.1866465	0.62357397
nonDEG	0.51384513	0.33182253	1	nonDEG	0.13786443	0.73049797
nonDEG	-3.1305884	0.64058281	1	nonDEG	-5.13E-15	1
nonDEG	1.67524058	0.19816299	1	nonDEG	-0.4269992	0.76978654
nonDEG	1.3409549	0.0344146	1	nonDEG	-0.9345717	0.05073443
nonDEG	-0.228072	0.62533627	1	nonDEG	-0.2678466	0.53740029
nonDEG	0.21463518	0.61909207	1	nonDEG	0.15279894	0.68777165
nonDEG	-0.2919888	0.50786161	1	nonDEG	-0.599492	0.11181937
nonDEG	-0.5784093	0.17240991	1	nonDEG	0.45430429	0.21000649
nonDEG	0.9136966	0.27737656	1	nonDEG	-1.2623769	0.03450905
nonDEG	-1.5009638	0.0226448	1	nonDEG	1.04621101	0.11368232
nonDEG	-3.1309068	0.63941523	1	nonDEG	3.96663327	0.43069549

nonDEG	1.2623593	0.01813102	0.94303121	nonDEG	-0.338811	0.44283989
nonDEG	0.67249335	0.27562236	1	nonDEG	-0.0487695	0.93810763
nonDEG	-0.754171	0.26459814	1	nonDEG	1.51522801	0.07016013
nonDEG	-1.7981064	0.15965153	1	nonDEG	1.77696785	0.14696957
nonDEG	-0.6714542	0.24950283	1	nonDEG	0.60476411	0.24162496
nonDEG	0.53077072	0.29512742	1	nonDEG	-0.1306182	0.73920826
nonDEG	0.13208649	0.79486334	1	nonDEG	0.09314237	0.83466715
nonDEG	-0.1473993	0.79976476	1	nonDEG	-0.0521603	0.90189869
nonDEG	-0.6091522	0.18936702	1	nonDEG	0.100203	0.81764051
nonDEG	-0.2314215	0.59787701	1	nonDEG	0.05762239	0.87776744
nonDEG	1.43701811	0.09825158	1	nonDEG	-0.9365724	0.26203058
nonDEG	1.098743	0.04592102	1	nonDEG	-0.8017702	0.11337277
nonDEG	-0.6275627	0.25035726	1	nonDEG	0.53441331	0.33179232
nonDEG	-0.1592367	0.70803016	1	nonDEG	-0.2643707	0.46650891
nonDEG	1.36762797	0.04118831	1	nonDEG	-1.5508994	0.00638783
nonDEG	1.03502937	0.02566684	1	nonDEG	-0.4056785	0.3737237
nonDEG	-1.0110134	0.16757418	1	nonDEG	0.74461219	0.33988201
nonDEG	0.66118103	0.13376751	1	nonDEG	-0.4312477	0.30807326
nonDEG	0.61071731	0.19689862	1	nonDEG	-0.3825058	0.31635439
nonDEG	-1.3342061	0.09090651	1	nonDEG	0.46496506	0.56643523
nonDEG	0.06581928	0.88937347	1	nonDEG	-1.8281761	0.0001607
nonDEG	-0.8861973	0.04707478	1	nonDEG	0.47377375	0.19872967
nonDEG	-0.0835402	0.85007207	1	nonDEG	-0.3442919	0.36562744
nonDEG	1.00848915	0.04768255	1	nonDEG	-0.0969407	0.82346483
nonDEG	-0.7279163	0.27392922	1	nonDEG	-0.2863978	0.67682275
nonDEG	-0.1785432	0.69480384	1	nonDEG	0.17054325	0.67287424
nonDEG	-0.5747252	0.2280343	1	nonDEG	-0.2696803	0.57378114
nonDEG	1.22841055	0.13164146	1	nonDEG	0.57455288	0.41380581
nonDEG	0.75057256	0.09478747	1	nonDEG	-0.1413932	0.70609165
nonDEG	-1.6668202	0.12352549	1	nonDEG	1.14057263	0.05945955
nonDEG	-0.66315	0.5207541	1	nonDEG	1.14098855	0.21847537
nonDEG	0.23362187	0.71959309	1	nonDEG	-0.3020682	0.5158813
nonDEG	0.76351059	0.13889305	1	nonDEG	0.21212255	0.56888263
nonDEG	1.0895745	0.02609519	1	nonDEG	-0.4404427	0.29609241
nonDEG	-1.4233268	0.05237007	1	nonDEG	1.64691787	0.05641511
nonDEG	-1.3449685	0.06432112	1	nonDEG	1.13397645	0.05977417
nonDEG	-0.6775553	0.22609467	1	nonDEG	0.70841501	0.18717102
nonDEG	-0.5786196	0.38964168	1	nonDEG	0.34104075	0.5679975
nonDEG	-1.2688601	0.11095699	1	nonDEG	1.33282515	0.12556503
nonDEG	-1.2211825	0.02313721	1	nonDEG	1.17654005	0.01838939
nonDEG	0.39062008	0.3685887	1	nonDEG	-0.1116933	0.76538384
nonDEG	0.65652081	0.17561796	1	nonDEG	-0.0012936	0.99818309
nonDEG	-0.5205849	0.51896666	1	nonDEG	1.77821765	0.05540438

nonDEG	-1.2364959	0.05293056	1	nonDEG	0.23718113	0.73683457
nonDEG	-0.2206651	0.77080037	1	nonDEG	0.21836836	0.57763236
nonDEG	-1.7820303	0.00105087	0.51315728	nonDEG	2.42347107	0.00040764
nonDEG	-0.4147341	0.35068944	1	nonDEG	0.22702112	0.53923308
nonDEG	0.25730574	0.70417688	1	nonDEG	-0.14832	0.82704828
nonDEG	0.23993628	0.69629613	1	nonDEG	-0.2268272	0.67057631
nonDEG	0.6455806	0.25160073	1	nonDEG	-0.1255416	0.80994939
nonDEG	-0.4747455	0.28177863	1	nonDEG	-0.1603471	0.69318716
nonDEG	-1.4015798	0.3065622	1	nonDEG	3.15752131	0.00390724
nonDEG	-0.6858796	0.13887687	1	nonDEG	-0.0558767	0.87952614
nonDEG	-0.3989148	0.41655568	1	nonDEG	-0.4585891	0.30800958
nonDEG	-0.0361531	0.93628053	1	nonDEG	-0.0090163	0.98112099
nonDEG	0.66443489	0.16237339	1	nonDEG	-0.466702	0.21274386
nonDEG	1.35273732	0.23106152	1	nonDEG	-0.4819865	0.47741813
nonDEG	1.44580241	0.0129704	0.8619311	nonDEG	-0.2511895	0.64222182
nonDEG	0.21037248	0.63170461	1	nonDEG	0.26035044	0.48033502
nonDEG	1.94466417	0.00337452	0.65691312	nonDEG	-0.3124617	0.59123494
nonDEG	-0.1854552	0.67359239	1	nonDEG	-0.3241396	0.39432255
nonDEG	-2.4346575	0.01196684	0.85229819	nonDEG	2.30346724	0.03881555
nonDEG	-0.2781257	0.51106049	1	nonDEG	-0.4085128	0.26871028
nonDEG	0.52293986	0.28674318	1	nonDEG	-0.6022637	0.1788646
nonDEG	0.49193123	0.47679364	1	nonDEG	-1.8291538	0.0173865
nonDEG	0.12430218	0.78521026	1	nonDEG	0.10980668	0.78309409
nonDEG	0.3427749	0.44851281	1	nonDEG	-0.2676967	0.49848103
nonDEG	-1.4687212	0.03231511	1	nonDEG	1.49479323	0.03559176
nonDEG	-0.4795769	0.53091453	1	nonDEG	0.3907118	0.62290601
nonDEG	-0.7407198	0.10622461	1	nonDEG	-0.1197378	0.7943625
nonDEG	-1.3207451	0.41007084	1	nonDEG	-1.1294822	0.79820461
nonDEG	-1.0003615	0.0736742	1	nonDEG	0.24280221	0.65206126
nonDEG	1.56416416	0.03272563	1	nonDEG	0.35897134	0.60064569
nonDEG	-2.714862	0.03592978	1	nonDEG	2.81245196	0.04978564
nonDEG	-1.4665942	0.15253767	1	nonDEG	1.9860582	0.05458966
nonDEG	-0.4471955	0.59534857	1	nonDEG	1.03997601	0.16267388
nonDEG	-0.5838219	0.16917372	1	nonDEG	0.19616707	0.60073752
nonDEG	-1.7862961	0.04987402	1	nonDEG	1.38369653	0.16502464
nonDEG	1.5900108	0.07227094	1	nonDEG	-1.007503	0.24548695
nonDEG	0.58563045	0.42323069	1	nonDEG	-0.4418684	0.33871998
nonDEG	-0.2503534	0.59460306	1	nonDEG	-0.2508776	0.58117517
nonDEG	-1.067072	0.08579855	1	nonDEG	1.47697888	0.05679702
nonDEG	1.37764985	0.07160549	1	nonDEG	-1.4052074	0.00552722
nonDEG	1.44747984	0.01429997	0.88752292	nonDEG	-0.4810511	0.30132805
nonDEG	0.59023156	0.1830626	1	nonDEG	-0.1878181	0.60446577
nonDEG	0.58926272	0.20581475	1	nonDEG	-0.2790874	0.44281116

nonDEG	1.59790617	0.00427005	0.68407511	nonDEG	-0.0570388	0.90677442
nonDEG	-1.7014231	0.01640753	0.91787008	nonDEG	1.86394954	0.00021264
nonDEG	2.04873242	0.03406447	1	nonDEG	-0.8327742	0.36753374
nonDEG	-0.8554964	0.08425306	1	nonDEG	0.94324869	0.02989311
nonDEG	-0.3370208	0.42914964	1	nonDEG	0.18842391	0.60817179
nonDEG	0.54250469	0.2367751	1	nonDEG	-0.4852973	0.20712097
nonDEG	0.85294322	0.49786158	1	nonDEG	-0.2024233	0.86920974
nonDEG	0.72798241	0.12207804	1	nonDEG	-0.3436527	0.42033725
nonDEG	0.97690272	0.24190709	1	nonDEG	0.73070828	0.28846634
nonDEG	0.75758129	0.24338792	1	nonDEG	-0.0821276	0.87397222
nonDEG	-1.073611	0.23210893	1	nonDEG	0.60931234	0.55325665
nonDEG	0.64984949	0.17383751	1	nonDEG	0.18531526	0.62055628
nonDEG	-0.2563334	0.58171118	1	nonDEG	-1.4988486	0.00101549
nonDEG	1.31956769	0.0054777	0.72967447	nonDEG	-0.3203069	0.38251306
nonDEG	0.07681986	0.86026712	1	nonDEG	-0.2668367	0.47745341
nonDEG	-1.198741	0.05198364	1	nonDEG	0.7642343	0.21031436
nonDEG	0.38224046	0.43092915	1	nonDEG	0.04559538	0.90564502
nonDEG	-0.4384244	0.61504511	1	nonDEG	0.7224796	0.37041028
nonDEG	0.45457334	0.56550546	1	nonDEG	-0.5019333	0.52461699
nonDEG	2.34847422	0.01213828	0.85229819	nonDEG	-0.2533468	0.759112
nonDEG	3.62082944	0.00011657	0.18795539	nonDEG	0.1254788	0.90326303
nonDEG	-1.6095497	0.01824495	0.94395982	nonDEG	0.51521182	0.4547361
nonDEG	-0.6515389	0.24114341	1	nonDEG	0.39997432	0.3710881
nonDEG	-0.5443366	0.23515687	1	nonDEG	0.285927	0.50189332
nonDEG	-1.1582484	0.01658558	0.91951594	nonDEG	1.20527403	0.01889817
DEG	1.01856723	0.02906966	1	nonDEG	0.43987583	0.25502253
nonDEG	-0.4108879	0.40708789	1	nonDEG	0.01056341	0.97930349
nonDEG	-0.1267647	0.77091686	1	nonDEG	-0.0672292	0.85542622
nonDEG	4.92827513	0.12751168	1	nonDEG	0.48113081	0.80918513
nonDEG	0.6839049	0.11556495	1	nonDEG	-0.0430019	0.90684108
nonDEG	0.5114126	0.32051534	1	nonDEG	-0.5872349	0.23757767
nonDEG	0.46305995	0.27483962	1	nonDEG	-0.2830509	0.44020743
nonDEG	0.80227118	0.3192877	1	nonDEG	-0.7782384	0.12944546
nonDEG	-0.321425	0.45753788	1	nonDEG	-0.0007776	0.99853011
nonDEG	-0.828498	0.20596337	1	nonDEG	0.91221929	0.13595538
nonDEG	0.80516475	0.05745154	1	nonDEG	-0.6195135	0.11440937
nonDEG	0.81025465	0.24946596	1	nonDEG	0.17053465	0.79507973
nonDEG	-0.3756083	0.40158713	1	nonDEG	-0.2526965	0.4864817
nonDEG	0.41105293	0.34283368	1	nonDEG	-0.4596886	0.21450836
nonDEG	0.93387964	0.05526613	1	nonDEG	-0.3457516	0.40873842
nonDEG	-1.3170252	0.10636429	1	nonDEG	1.20826789	0.13435599
nonDEG	0.33071781	0.69122022	1	nonDEG	0.00141801	1
nonDEG	-1.5137074	0.02067851	0.99114571	nonDEG	1.18289673	0.07484557

nonDEG	2.2058739	0.0005965	0.4621112	nonDEG	-0.5451038	0.28613338
nonDEG	0.06081548	0.88913361	1	nonDEG	0.11949047	0.74819023
nonDEG	-0.6412969	0.16536788	1	nonDEG	0.30816678	0.46485743
nonDEG	0.21209249	0.62715136	1	nonDEG	-0.1164669	0.76265144
nonDEG	0.36965433	0.44953667	1	nonDEG	-0.5861182	0.18139173
nonDEG	0.40990379	0.40190577	1	nonDEG	-0.1733287	0.66722603
nonDEG	0.96588458	0.09239655	1	nonDEG	-0.2355535	0.62802493
nonDEG	-1.2299838	0.0131013	0.86352262	nonDEG	-0.4410955	0.29366584
nonDEG	-0.178878	0.68046043	1	nonDEG	-0.0374058	0.92251344
nonDEG	1.96608598	0.04006441	1	nonDEG	-0.0460105	0.96635855
nonDEG	0.80174919	0.10673074	1	nonDEG	0.19093476	0.65283013
nonDEG	-0.0542622	0.90835995	1	nonDEG	0.2261975	0.60025912
nonDEG	0.78913273	0.1102218	1	nonDEG	-0.0480351	0.89520075
nonDEG	-0.4101865	0.35618096	1	nonDEG	0.01115529	0.97667516
nonDEG	0.32743168	0.44146396	1	nonDEG	-0.2208585	0.56624805
nonDEG	-0.1967212	0.67200547	1	nonDEG	-0.1964765	0.62256793
nonDEG	-1.1754802	0.03512724	1	nonDEG	1.79035067	0.00529997
nonDEG	-0.2329287	0.65032939	1	nonDEG	0.33641526	0.46175692
nonDEG	0.52302762	0.27796542	1	nonDEG	-0.4655357	0.20901052
nonDEG	-0.0393823	0.93469766	1	nonDEG	-0.5587182	0.16801668
nonDEG	-0.3008451	0.48238107	1	nonDEG	0.09359361	0.80376233
nonDEG	0.37408589	0.61399872	1	nonDEG	-0.2097651	0.77866094
nonDEG	-0.7580511	0.26187833	1	nonDEG	0.13999721	0.83473315
nonDEG	1.34347693	0.01134483	0.84185458	nonDEG	-0.3062034	0.4607841
nonDEG	-0.1940885	0.65885198	1	nonDEG	0.22943996	0.53899344
nonDEG	2.71512409	0.00894238	0.79328284	nonDEG	-0.4348984	0.59839908
nonDEG	-0.4145724	0.51291202	1	nonDEG	0.47287922	0.42731436
nonDEG	0.37697943	0.53104512	1	nonDEG	-0.5606274	0.25543236
nonDEG	0.92307714	0.06676185	1	nonDEG	-0.2969676	0.42311201
nonDEG	-0.4841947	0.28689805	1	nonDEG	-0.4636374	0.29889211
nonDEG	0.40076336	0.37766146	1	nonDEG	-0.1288739	0.73235592
nonDEG	0.81167617	0.0841067	1	nonDEG	-0.2396725	0.51808792
nonDEG	0.11641999	0.85970482	1	nonDEG	-0.5898967	0.28804091
nonDEG	-1.7775236	0.01606307	0.91466279	nonDEG	0.1115109	0.80369059
nonDEG	1.26833473	0.03031802	1	nonDEG	-0.5928566	0.25594201
nonDEG	0	1	1	nonDEG	4.27052358	0.36079387
nonDEG	0.69577776	0.21521632	1	nonDEG	-0.1582917	0.76575
nonDEG	-0.7691913	0.1515784	1	nonDEG	0.13943137	0.76601951
nonDEG	-0.7343338	0.11800874	1	nonDEG	0.11322573	0.80501388
nonDEG	0.48417334	0.45102758	1	nonDEG	-0.2249391	0.68906896
nonDEG	-0.5937819	0.45003932	1	nonDEG	0.57338481	0.50996185
nonDEG	-0.0724347	0.8690579	1	nonDEG	-0.1741685	0.63167081
nonDEG	0.19787719	0.78834613	1	nonDEG	0.43623493	0.53463874

DEG	1.88808174	0.01613501	0.91521859	nonDEG	1.28386847	0.04333376
nonDEG	-0.9250792	0.05140527	1	nonDEG	0.70601946	0.08898429
nonDEG	0.14242524	0.74716293	1	nonDEG	-0.1194758	0.74446894
nonDEG	1.24703474	0.13820585	1	nonDEG	0.09948858	0.90891775
nonDEG	-0.0051691	0.99088887	1	nonDEG	0.04718553	0.89905926
nonDEG	-2.3246398	0.02896879	1	nonDEG	2.07959659	0.01891107
nonDEG	-0.6737253	0.14353737	1	nonDEG	0.31101814	0.43406483
nonDEG	0.60945555	0.23010854	1	nonDEG	-0.4185593	0.24725343
nonDEG	2.08621173	0.00122327	0.52498817	nonDEG	-0.8342036	0.1517136
nonDEG	0.29965885	0.56634037	1	nonDEG	-0.2607647	0.56259564
nonDEG	0.59141181	0.47904904	1	nonDEG	0.87452039	0.23291417
nonDEG	0.52508026	0.22131859	1	nonDEG	-0.0946937	0.79910131
nonDEG	-1.3995522	0.01056822	0.82576758	nonDEG	1.57002013	0.01157647
nonDEG	-1.3329547	0.03047995	1	nonDEG	0.53573924	0.33032514
nonDEG	0.26530117	0.54979368	1	nonDEG	-0.1480896	0.68837024
nonDEG	0.19057441	0.72827718	1	nonDEG	0.06347486	0.86863498
nonDEG	-0.5496748	0.31329924	1	nonDEG	0.25000093	0.64171939
nonDEG	-0.5246973	0.23853718	1	nonDEG	0.21792837	0.6487769
nonDEG	0.13660228	0.76388904	1	nonDEG	-0.8996211	0.02441554
nonDEG	0.42511712	0.36548637	1	nonDEG	-0.1679626	0.65975554
nonDEG	1.09837062	0.06598107	1	nonDEG	-0.3550562	0.5201531
nonDEG	0.64994366	0.21858097	1	nonDEG	-0.7052827	0.13932182
nonDEG	-0.867877	0.19185547	1	nonDEG	0.76226602	0.19816733
nonDEG	1.72059333	0.10519291	1	nonDEG	0.56827615	0.5323049
nonDEG	0.06349224	0.95841786	1	nonDEG	0.45318132	0.58612504
nonDEG	0.4533944	0.46154165	1	nonDEG	-0.0872696	0.88538576
nonDEG	-0.8730653	0.09350054	1	nonDEG	1.25235847	0.02207346
nonDEG	-0.3745161	0.38353251	1	nonDEG	-0.1911466	0.62313754
nonDEG	-0.5472171	0.20150719	1	nonDEG	0.39087186	0.28921896
nonDEG	-0.2555512	0.55179202	1	nonDEG	-0.2880795	0.42635579
nonDEG	0.52545289	0.24441695	1	nonDEG	-0.6456441	0.09587208
nonDEG	0.33340803	0.57866339	1	nonDEG	-0.5054205	0.3595331
nonDEG	-0.0689335	0.87576659	1	nonDEG	-0.6813864	0.06852643
nonDEG	1.54512321	0.02609965	1	nonDEG	-0.7226977	0.25894269
nonDEG	-0.8482551	0.08202471	1	nonDEG	0.93329093	0.04811408
nonDEG	0.6545369	0.16487218	1	nonDEG	-0.1608292	0.66156312
nonDEG	0.34770882	0.52101322	1	nonDEG	0.29239293	0.54604844
nonDEG	0.40625101	0.39005838	1	nonDEG	0.22807727	0.58875993
nonDEG	-0.9869061	0.03909314	1	nonDEG	0.94506087	0.01451558
nonDEG	-1.2998044	0.02086956	0.9925346	nonDEG	-0.0303461	0.94954215
nonDEG	0.23373437	0.63532451	1	nonDEG	-0.3774359	0.35195721
nonDEG	-0.0045362	0.99215979	1	nonDEG	0.26174163	0.49195831
nonDEG	-0.3224843	0.44978876	1	nonDEG	0.09971308	0.79997146

nonDEG	0.84242041	0.09310295	1	nonDEG	-0.456898	0.27996169
nonDEG	-0.3282446	0.58751036	1	nonDEG	0.31277833	0.60016397
nonDEG	-0.3764333	0.41260273	1	nonDEG	0.04652717	0.91445146
nonDEG	0.78997624	0.30960387	1	nonDEG	-0.6343914	0.29396465
nonDEG	-0.4686731	0.31555083	1	nonDEG	-0.2079086	0.60616554
nonDEG	-1.5714532	0.01943424	0.97153571	nonDEG	1.74304348	0.0002392
nonDEG	0.29315907	0.48768316	1	nonDEG	-0.0829833	0.82032447
nonDEG	-0.4760092	0.28486766	1	nonDEG	1.33802731	0.01984356
nonDEG	1.10202877	0.08442844	1	nonDEG	-0.6797313	0.2570798
nonDEG	0.28867175	0.57647038	1	nonDEG	-0.0180991	0.97182222
nonDEG	1.33770077	0.02483615	1	nonDEG	-0.8164593	0.13490422
nonDEG	0.51389385	0.25208686	1	nonDEG	-0.1277534	0.73268501
nonDEG	1.68000417	0.01632743	0.91787008	nonDEG	-0.9346306	0.15780155
nonDEG	-3.6295414	0.05471463	1	nonDEG	2.26385428	0.41919959
nonDEG	0.61250402	0.37377876	1	nonDEG	-0.3227816	0.64241989
nonDEG	2.64503635	0.01830977	0.94399538	nonDEG	-0.5148895	0.63133435
nonDEG	0.88873893	0.07033543	1	nonDEG	-0.5707166	0.16474766
nonDEG	-1.123573	0.05634542	1	nonDEG	0.18991007	0.7083422
nonDEG	-0.1702077	0.70117271	1	nonDEG	0.02570823	0.94655356
nonDEG	-4.1115476	0.00097268	0.51194803	nonDEG	0.76792535	0.50346838
nonDEG	0.45036811	0.39892374	1	nonDEG	-0.3560023	0.47595086
nonDEG	0.43680916	0.31985061	1	nonDEG	-0.8938209	0.01999358
nonDEG	0.80976217	0.06943022	1	nonDEG	-0.320544	0.41013155
nonDEG	-0.1273924	0.78488597	1	nonDEG	0.02923797	0.94254403
nonDEG	-0.3333443	0.62799129	1	nonDEG	0.28772603	0.67892055
nonDEG	-0.7638736	0.09762969	1	nonDEG	0.34727373	0.39902304
nonDEG	1.24647883	0.00881627	0.7914707	nonDEG	-0.5221058	0.22085105
nonDEG	0.68756872	0.23056485	1	nonDEG	-0.1627427	0.69606253
nonDEG	0.75018606	0.43476307	1	nonDEG	-0.3762477	0.71641305
nonDEG	0.05194857	0.92623742	1	nonDEG	0.18157458	0.71213562
nonDEG	0.35462653	0.42077776	1	nonDEG	-0.1692772	0.67674684
nonDEG	1.72444036	0.00900052	0.79328284	nonDEG	-0.4588437	0.43218647
nonDEG	3.42415167	0.00383393	0.67135178	nonDEG	-1.1831468	0.26302754
nonDEG	-0.2181351	0.6150663	1	nonDEG	-0.2874242	0.44221734
nonDEG	1.60981025	0.00616416	0.73793371	nonDEG	-0.6085319	0.2048347
nonDEG	-0.5600194	0.19382693	1	nonDEG	-0.3327653	0.41521595
nonDEG	-0.6990705	0.22524383	1	nonDEG	0.97848552	0.02510875
nonDEG	-0.4268634	0.31811186	1	nonDEG	-0.4000706	0.27186221
nonDEG	0.09088169	0.84445474	1	nonDEG	0.26177571	0.5161842
nonDEG	-0.6889099	0.15468828	1	nonDEG	0.89994381	0.03326947
nonDEG	0.55239342	0.4887498	1	nonDEG	0.37906089	0.59135598
nonDEG	0.01173648	0.9839664	1	nonDEG	0.3315484	0.4377436
nonDEG	1.83437224	0.00657876	0.73793371	nonDEG	-0.6132036	0.31487509

nonDEG	-0.1373564	0.84007797	1	nonDEG	0.4880666	0.39971953
nonDEG	3.05410393	0.64743114	1	nonDEG	-3.2223569	0.67934871
nonDEG	-0.346949	0.53117305	1	nonDEG	0.23973644	0.64718339
nonDEG	0.15501348	0.77996468	1	nonDEG	0.09505082	0.85821142
nonDEG	-0.1242305	0.77583435	1	nonDEG	-0.2178498	0.56064145
nonDEG	0.33676917	0.47483733	1	nonDEG	-0.5767461	0.13283551
nonDEG	-0.2859871	0.610505	1	nonDEG	0.52278053	0.32518822
nonDEG	-0.1212275	0.79094199	1	nonDEG	-0.5828898	0.13967205
nonDEG	-0.3741534	0.41007951	1	nonDEG	0.10466667	0.79203066
nonDEG	-0.1018574	0.82013657	1	nonDEG	-0.202118	0.60065242
nonDEG	-0.6838831	0.30558401	1	nonDEG	0.80544072	0.15278022
nonDEG	0.11005523	0.80347467	1	nonDEG	-0.1174708	0.74619434
nonDEG	-0.4688621	0.34826566	1	nonDEG	0.64920292	0.08367981
nonDEG	-1.5539478	0.05438699	1	nonDEG	0.96386918	0.06242137
nonDEG	-0.6392668	0.5863316	1	nonDEG	1.02712794	0.40886756
nonDEG	1.44830018	0.04959637	1	nonDEG	-0.6059223	0.22706789
nonDEG	0.69815159	0.11802536	1	nonDEG	-1.0358561	0.01202977
nonDEG	-0.6982068	0.14937057	1	nonDEG	0.71814408	0.11546263
nonDEG	-0.5331255	0.26580503	1	nonDEG	0.3104743	0.49033087
nonDEG	-0.4780973	0.27667966	1	nonDEG	0.39839033	0.29144372
nonDEG	-0.3708578	0.3976398	1	nonDEG	-0.0247974	0.94711027
nonDEG	-0.3606916	0.51358583	1	nonDEG	0.27751375	0.59127844
nonDEG	-0.4580097	0.28388873	1	nonDEG	0.2927782	0.4380035
nonDEG	2.26539665	0.0586007	1	nonDEG	-1.4149613	0.32374455
nonDEG	-0.652061	0.60686009	1	nonDEG	1.29010509	0.29146764
nonDEG	-0.0033211	0.99512986	1	nonDEG	-0.1059295	0.77577587
nonDEG	2.08140311	0.06375899	1	nonDEG	-1.6600044	0.08696697
nonDEG	-0.5869058	0.24143885	1	nonDEG	0.71162157	0.09042277
nonDEG	1.32538885	0.05132347	1	nonDEG	-1.3407872	0.03492521
nonDEG	-0.9067818	0.40786006	1	nonDEG	0.94276271	0.42148186
nonDEG	-0.2684305	0.52744291	1	nonDEG	0.17908574	0.62709894
nonDEG	-1.947461	0.1382296	1	nonDEG	2.13306506	0.12561024
nonDEG	-0.6669859	0.30381309	1	nonDEG	0.13488049	0.83998838
nonDEG	-1.4502223	0.08896505	1	nonDEG	2.17617598	0.00600098
nonDEG	0.81947885	0.22400912	1	nonDEG	0.09419795	0.83234119
nonDEG	0.16981552	0.80634007	1	nonDEG	-0.1667433	0.807609
nonDEG	-1.0455865	0.17648009	1	nonDEG	1.19660696	0.01183363
nonDEG	2.54407103	0.08553951	1	nonDEG	-2.1993165	0.12589797
nonDEG	0.51454155	0.44638518	1	nonDEG	-0.2437263	0.70780204
nonDEG	1.33150234	0.10018956	1	nonDEG	-0.4635371	0.45973159
nonDEG	1.06123816	0.19950224	1	nonDEG	0.10111885	0.8538044
nonDEG	0.83277228	0.06990155	1	nonDEG	-0.3171396	0.38561985
nonDEG	0.47417789	0.26956489	1	nonDEG	0.81579997	0.0256818

nonDEG	-0.8061969	0.14613296	1	nonDEG	0.66388933	0.21902518
nonDEG	2.28946601	0.00630741	0.73793371	nonDEG	-1.1414507	0.10446427
nonDEG	-0.4933489	0.47302903	1	nonDEG	0.92781572	0.14402896
nonDEG	-2.0788173	0.00528864	0.72753356	nonDEG	0.99385066	0.14902702
nonDEG	0.89035132	0.11537054	1	nonDEG	-0.3905762	0.42129683
nonDEG	-0.5462905	0.24445699	1	nonDEG	-0.1761301	0.69182079
nonDEG	0.18399167	0.73338417	1	nonDEG	0.01185284	0.98351188
nonDEG	-0.3911933	0.35924803	1	nonDEG	0.06074739	0.87003786
nonDEG	1.40388813	0.00478821	0.69782277	nonDEG	-0.6140949	0.13844737
nonDEG	0.55979422	0.65732433	1	nonDEG	0.40712121	0.74444718
nonDEG	-0.7424509	0.29362432	1	nonDEG	0.85369798	0.2367726
nonDEG	-0.9251745	0.14383775	1	nonDEG	0.44715006	0.38376743
nonDEG	0.04296321	0.9269845	1	nonDEG	-0.272918	0.48448711
nonDEG	-0.4260909	0.33472579	1	nonDEG	0.16688402	0.64464431
nonDEG	0.11447479	0.80412601	1	nonDEG	-0.0847593	0.82428296
nonDEG	0.11565287	0.81406115	1	nonDEG	-0.4452793	0.25698175
nonDEG	-0.4006535	0.34949863	1	nonDEG	0.26488744	0.48114661
nonDEG	-2.8172685	0.01238204	0.85229819	nonDEG	2.78144287	0.00502339
nonDEG	-0.4327922	0.3300654	1	nonDEG	0.32831657	0.37921545
nonDEG	-0.1598308	0.73693676	1	nonDEG	-0.7020931	0.0612245
nonDEG	0.76898398	0.41130977	1	nonDEG	-0.5305784	0.57464594
nonDEG	-0.0438411	0.94052669	1	nonDEG	0.10863705	0.83086484
nonDEG	-0.7259173	0.09848204	1	nonDEG	0.29582842	0.45865164
nonDEG	0.64186023	0.26973314	1	nonDEG	-0.8518385	0.13591954
nonDEG	0.83096737	0.32776233	1	nonDEG	0.29278021	0.6995247
nonDEG	0.64310831	0.17213871	1	nonDEG	-0.9125065	0.04094778
nonDEG	1.65630898	0.30499348	1	nonDEG	-0.3473277	0.80604095
nonDEG	1.5037795	0.03757088	1	nonDEG	-0.0833353	0.91812745
nonDEG	0.2668249	0.5484551	1	nonDEG	0.13639919	0.71052791
nonDEG	-0.4991895	0.28131882	1	nonDEG	0.59204474	0.12403368
nonDEG	-0.2345154	0.59896776	1	nonDEG	0.35790719	0.37117758
nonDEG	-0.9831965	0.17998043	1	nonDEG	1.61407607	0.0429887
nonDEG	1.37645088	0.05265833	1	nonDEG	-0.2498259	0.69127869
nonDEG	-0.3389233	0.44286801	1	nonDEG	0.17274141	0.66231045
nonDEG	-0.9958038	0.23162281	1	nonDEG	0.86207573	0.28822111
nonDEG	0.39226813	0.39113841	1	nonDEG	-0.1380427	0.73553451
nonDEG	-0.2622537	0.54558529	1	nonDEG	-0.164902	0.66835689
nonDEG	-1.7097994	0.09372071	1	nonDEG	2.18436007	0.07773492
nonDEG	0.16441543	0.79053499	1	nonDEG	0.46279925	0.40150965
nonDEG	0.20471701	0.6792059	1	nonDEG	-0.1641952	0.6909745
nonDEG	-0.6648544	0.14594409	1	nonDEG	0.8661012	0.07211707
nonDEG	-0.7955403	0.25742833	1	nonDEG	-0.545562	0.51635714
nonDEG	-0.8242602	0.14132393	1	nonDEG	0.10466315	0.83027501

nonDEG	0.88746599	0.18538095	1	nonDEG	-0.3056134	0.57981477
nonDEG	-0.1984615	0.64619593	1	nonDEG	-0.5821451	0.15681605
nonDEG	0.01312248	0.97970017	1	nonDEG	-0.1714302	0.6629741
nonDEG	0.13863937	0.78739101	1	nonDEG	-0.092254	0.84413132
nonDEG	-1.0716798	0.07342892	1	nonDEG	0.98394216	0.09203412
nonDEG	-1.0675898	0.07657349	1	nonDEG	0.66356897	0.22709481
nonDEG	1.63028618	0.00691039	0.75126991	nonDEG	-0.4220908	0.41164714
nonDEG	-0.0783967	0.86772241	1	nonDEG	0.46750341	0.26856565
nonDEG	-1.0792646	0.06969746	1	nonDEG	1.03340375	0.07814497
nonDEG	0.77782823	0.16335145	1	nonDEG	-0.516356	0.2032795
nonDEG	0.67622626	0.16110147	1	nonDEG	-0.2314499	0.5795239
nonDEG	-0.6928941	0.18421723	1	nonDEG	1.26052021	0.00350909
nonDEG	1.18063172	0.03972698	1	nonDEG	-0.0327178	0.93617514
nonDEG	-0.2474082	0.56488992	1	nonDEG	0.21993313	0.55357347
nonDEG	0.2096473	0.63601934	1	nonDEG	0.18261962	0.62430675
nonDEG	-0.9303772	0.13067763	1	nonDEG	0.45571551	0.44720566
nonDEG	-0.4265758	0.31429406	1	nonDEG	-0.1260546	0.73152016
nonDEG	0.32958592	0.48846542	1	nonDEG	-0.1678605	0.69177652
nonDEG	0.24859975	0.56732213	1	nonDEG	-0.164434	0.64997356
nonDEG	-0.1135036	0.79863744	1	nonDEG	0.84141615	0.02511663
nonDEG	-0.4102015	0.34127867	1	nonDEG	-0.1839418	0.61665479
nonDEG	-0.1491595	0.74076991	1	nonDEG	-0.3207235	0.39905842
nonDEG	-0.3111066	0.51073616	1	nonDEG	-0.574525	0.18689046
nonDEG	0.65441285	0.14754114	1	nonDEG	-0.1484619	0.6965916
nonDEG	-2.2950099	0.01717923	0.93079586	nonDEG	0.92240167	0.24369982
nonDEG	-0.5038592	0.2421054	1	nonDEG	0.15234983	0.69123632
nonDEG	-0.2537604	0.7672767	1	nonDEG	0.01502809	0.99192297
nonDEG	1.11610963	0.15272535	1	nonDEG	-0.0720511	0.92599929
nonDEG	-0.647837	0.22290293	1	nonDEG	0.49920585	0.26173784
nonDEG	0.65139728	0.18726211	1	nonDEG	0.24715919	0.56463564
nonDEG	0.54007705	0.36853741	1	nonDEG	-0.8555424	0.15191476
nonDEG	-0.1096637	0.79804066	1	nonDEG	-0.5613651	0.12828669
nonDEG	-0.9665082	0.06919318	1	nonDEG	0.88082814	0.01989714
nonDEG	-0.4846919	0.29982118	1	nonDEG	0.12497735	0.75784661
nonDEG	-0.3176185	0.47946283	1	nonDEG	0.15474267	0.68380469
nonDEG	-0.8984754	0.73167236	1	nonDEG	-0.1522785	1
nonDEG	-0.5466395	0.23170505	1	nonDEG	0.68264979	0.06032724
nonDEG	-2.0821111	0.01628799	0.91787008	nonDEG	2.05873946	0.00911233
nonDEG	0.09957015	0.82047533	1	nonDEG	-0.0277048	0.94283814
nonDEG	-0.9171188	0.0328085	1	nonDEG	1.06372524	0.00324095
nonDEG	-0.3378591	0.4803243	1	nonDEG	0.09716015	0.8032075
nonDEG	0.34261252	0.46617232	1	nonDEG	-0.1222391	0.76086701
nonDEG	-0.778581	0.10164245	1	nonDEG	1.19109739	0.0285761

nonDEG	0.24501129	0.62622712	1	nonDEG	-0.2667162	0.47817038
nonDEG	0.29454134	0.62966187	1	nonDEG	-0.2801437	0.58146988
nonDEG	0.68468323	0.6150403	1	nonDEG	-0.3062297	0.82568962
nonDEG	-0.1225437	0.79377602	1	nonDEG	0.27791265	0.45226838
nonDEG	0.70171379	0.18138979	1	nonDEG	-0.5266934	0.19832675
nonDEG	1.39451238	0.16369804	1	nonDEG	-0.2417343	0.83765904
nonDEG	0.39096908	0.57270594	1	nonDEG	-0.4972433	0.41814425
nonDEG	0.52260246	0.27561662	1	nonDEG	-0.3866034	0.32754929
nonDEG	0.92645862	0.08554717	1	nonDEG	0.1753871	0.69703909
nonDEG	-0.1645414	0.71855755	1	nonDEG	-0.7686255	0.05473943
nonDEG	-0.3318259	0.44336782	1	nonDEG	0.01999669	0.95701062
nonDEG	2.31227486	0.05760575	1	nonDEG	0.36805302	0.70362192
nonDEG	-0.8333869	0.13063049	1	nonDEG	-0.0059109	0.99135134
nonDEG	0.65398976	0.22623679	1	nonDEG	-0.0506371	0.9213754
nonDEG	0.61440956	0.27708022	1	nonDEG	0.00406008	0.99507013
nonDEG	0.66479363	0.25405808	1	nonDEG	-0.1261012	0.78454457
nonDEG	-1.3064769	0.0606743	1	nonDEG	1.17205072	0.09045053
nonDEG	-0.6631159	0.11688662	1	nonDEG	0.58839294	0.1019901
nonDEG	0.73715733	0.29166249	1	nonDEG	-1.2015747	0.04396
nonDEG	-0.3676072	0.41074498	1	nonDEG	-0.2598434	0.49968178
nonDEG	0.38008167	0.5149594	1	nonDEG	-0.4037556	0.35247553
nonDEG	0.64851417	0.1470532	1	nonDEG	-0.4087618	0.28233364
nonDEG	-0.3878801	0.37474772	1	nonDEG	-0.6187124	0.10071041
nonDEG	0.66072587	0.49357718	1	nonDEG	-0.3619477	0.66812812
nonDEG	0.62907031	0.18559683	1	nonDEG	-0.0464199	0.90083091
nonDEG	0.23375292	0.76292995	1	nonDEG	0.94470109	0.18595179
nonDEG	-0.2427004	0.59914155	1	nonDEG	0.1714025	0.67738191
nonDEG	-0.4661529	0.2862698	1	nonDEG	-0.3458509	0.33860221
nonDEG	0.46399586	0.53651043	1	nonDEG	-0.1249773	0.87306621
nonDEG	0.37233882	0.4016001	1	nonDEG	-0.1531476	0.70811943
nonDEG	0.8282701	0.07514609	1	nonDEG	0.04487298	0.9089203
nonDEG	1.03208034	0.03501151	1	nonDEG	-0.1949701	0.64555166
nonDEG	-0.1964051	0.65472931	1	nonDEG	-0.3995365	0.30616961
nonDEG	1.81271433	0.00105524	0.51315728	nonDEG	-0.4706441	0.31943679
nonDEG	0.21377801	0.7168787	1	nonDEG	-0.2717246	0.63036599
nonDEG	-0.6938257	0.32018726	1	nonDEG	1.91164243	0.00010274
nonDEG	0.82518485	0.09030016	1	nonDEG	-0.0639879	0.86023452
nonDEG	0.0056506	0.99680135	1	nonDEG	0.53937998	0.24754839
nonDEG	-0.0545493	0.91097295	1	nonDEG	-1.0655427	0.01182272
nonDEG	-0.7795346	0.16362054	1	nonDEG	-0.464882	0.43578255
nonDEG	-0.2884402	0.5390409	1	nonDEG	0.32764304	0.39871228
nonDEG	-0.5499601	0.28834781	1	nonDEG	-0.3396472	0.45273453
nonDEG	-0.7568641	0.20114729	1	nonDEG	0.07238544	0.88663393

nonDEG	-0.3892346	0.35282372	1	nonDEG	-0.5473501	0.17201676
nonDEG	-0.2146276	0.73232885	1	nonDEG	-0.7983037	0.19139851
nonDEG	-0.2164818	0.61553445	1	nonDEG	-0.4360616	0.25391052
nonDEG	0.74759704	0.31355373	1	nonDEG	0.61893024	0.34806969
nonDEG	-0.3237829	0.4504794	1	nonDEG	0.33217081	0.36262523
nonDEG	-1.523208	0.07541964	1	nonDEG	0.59247651	0.44079733
nonDEG	1.53611211	0.06735603	1	nonDEG	-0.4424797	0.57028854
nonDEG	0.6287484	0.15289463	1	nonDEG	-0.159793	0.65974888
nonDEG	-0.8911068	0.26563074	1	nonDEG	1.36878862	0.06762384
nonDEG	0.40764375	0.35581183	1	nonDEG	-0.0090576	0.98073436
nonDEG	0.74293083	0.14519421	1	nonDEG	-0.0853594	0.82042509
nonDEG	-2.7781299	0.04041163	1	nonDEG	2.79786197	0.0738797
nonDEG	0.34285398	0.5219176	1	nonDEG	-0.3026459	0.39920475
nonDEG	-0.6183624	0.19739256	1	nonDEG	0.06147585	0.88586887
nonDEG	0.07534253	0.88116605	1	nonDEG	-0.1958527	0.67419178
nonDEG	-0.50152	0.31303465	1	nonDEG	0.05337816	0.90482172
nonDEG	-0.0164141	0.96982106	1	nonDEG	0.36750452	0.3222575
nonDEG	0.74159098	0.1017455	1	nonDEG	-0.3188601	0.3765878
nonDEG	-1.3530624	0.020573	0.99114571	nonDEG	0.24043846	0.67297638
nonDEG	0.35463309	0.46751103	1	nonDEG	0.12361601	0.76664565
nonDEG	0.10138304	0.86697521	1	nonDEG	-0.3910357	0.49960727
nonDEG	0.82831333	0.17424841	1	nonDEG	-0.4785768	0.23015653
nonDEG	0.616029	0.17024614	1	nonDEG	-0.0956072	0.79659987
nonDEG	3.09626415	0.00939864	0.79482907	nonDEG	-1.501349	0.04281116
nonDEG	0.79701126	0.33189365	1	nonDEG	0.49597582	0.50020836
nonDEG	1.35146309	0.10955324	1	nonDEG	-0.077221	0.92705132
nonDEG	-1.4441856	0.02312619	1	nonDEG	1.27755724	0.00506059
nonDEG	1.83237033	0.0839971	1	nonDEG	-2.1385848	0.04358165
nonDEG	-0.5781349	0.35340498	1	nonDEG	-0.3313588	0.51066342
nonDEG	-0.1072821	0.80923468	1	nonDEG	0.1810734	0.62408484
nonDEG	-1.4608351	0.01197268	0.85229819	nonDEG	1.28755729	0.04569453
nonDEG	-1.2480849	0.07951146	1	nonDEG	1.76738563	0.01356667
nonDEG	1.03692464	0.14538731	1	nonDEG	-0.3712167	0.51148309
nonDEG	-1.6193844	0.05893968	1	nonDEG	0.67158055	0.35403987
nonDEG	-0.3814239	0.6668402	1	nonDEG	0.44279088	0.59295908
nonDEG	0.70272254	0.13652075	1	nonDEG	-0.4365067	0.30142213
nonDEG	1.08890164	0.09467963	1	nonDEG	-1.5574056	0.00491072
nonDEG	-1.0489703	0.27240136	1	nonDEG	0.71259919	0.53331074
nonDEG	-0.372601	0.38238799	1	nonDEG	0.10966299	0.76475709
nonDEG	-1.0648909	0.12368864	1	nonDEG	-0.1174302	0.88776399
nonDEG	-0.6080061	0.16955507	1	nonDEG	-0.5301259	0.29410573
nonDEG	0.60827874	0.58593598	1	nonDEG	1.0530341	0.32844382
nonDEG	0.96106836	0.03699042	1	nonDEG	-0.2634829	0.46997068

nonDEG	0.75685165	0.09394413	1	nonDEG	-0.1442082	0.69344872
nonDEG	0.99686067	0.14916883	1	nonDEG	-0.6290324	0.36268701
nonDEG	1.00644634	0.04500695	1	nonDEG	-0.8213448	0.06819088
nonDEG	-0.2211554	0.61006482	1	nonDEG	0.03384969	0.9266055
nonDEG	-1.3584743	0.05204211	1	nonDEG	0.85743004	0.09940882
nonDEG	-0.6173164	0.16752013	1	nonDEG	0.34877632	0.38887974
nonDEG	-1.0653068	0.11787816	1	nonDEG	1.16269037	0.04625244
nonDEG	-0.535636	0.20568714	1	nonDEG	0.11119057	0.76135759
nonDEG	-0.5915797	0.24850172	1	nonDEG	0.2373678	0.60203758
nonDEG	-0.3750191	0.54726578	1	nonDEG	-1.1131937	0.1201897
nonDEG	1.30434405	0.09192549	1	nonDEG	-1.0294391	0.16851347
nonDEG	0.03889722	0.93391919	1	nonDEG	-0.6807742	0.09803438
nonDEG	-0.7555466	0.19012376	1	nonDEG	-0.0616685	0.90007149
nonDEG	-0.3565638	0.40537716	1	nonDEG	-0.6218314	0.08738234
nonDEG	0.36852291	0.38226375	1	nonDEG	-0.0175907	0.96157294
nonDEG	1.78919063	0.01474363	0.89005739	nonDEG	-0.9725094	0.09253857
nonDEG	-2.0627149	0.03971265	1	nonDEG	2.17547204	0.08432111
nonDEG	0.9898408	0.09626925	1	nonDEG	-0.7212438	0.18698762
nonDEG	2.58818191	0.04851085	1	nonDEG	-0.3470617	0.81203563
nonDEG	-0.241131	0.65285282	1	nonDEG	0.3241699	0.52467948
nonDEG	-0.3020011	0.49075636	1	nonDEG	-0.0692557	0.84880086
nonDEG	-1.5966552	0.03507222	1	nonDEG	1.2320573	0.05393527
nonDEG	-1.4119515	0.02451507	1	nonDEG	0.9736898	0.0462995
nonDEG	1.29255001	0.12162509	1	nonDEG	0.05933395	0.95134557
nonDEG	-1.3337637	0.10084515	1	nonDEG	1.67631583	0.04239955
nonDEG	1.00417749	0.0294962	1	nonDEG	-0.5650089	0.13665362
nonDEG	-1.7272028	0.00709108	0.75136371	nonDEG	1.61144225	0.01886732
nonDEG	0.58204948	0.16850218	1	nonDEG	-0.4162833	0.26952265
nonDEG	0.41376312	0.4842764	1	nonDEG	0.27874398	0.59166467
DEG	1.40795946	0.01944046	0.97153571	nonDEG	0.30092315	0.54634303
nonDEG	-0.345414	0.46129599	1	nonDEG	-0.1591195	0.68991539
nonDEG	0.91377862	0.08814893	1	nonDEG	-0.2502744	0.60451699
nonDEG	0.17550055	0.68213687	1	nonDEG	-0.2003673	0.58746478
nonDEG	-1.337148	0.00256368	0.61776954	nonDEG	-0.414908	0.33783574
nonDEG	-1.8188114	0.13781424	1	nonDEG	1.96907828	0.13893023
nonDEG	-3.2773958	0.00081089	0.50181787	nonDEG	4.16067861	0.00030979
nonDEG	-0.6399824	0.35290593	1	nonDEG	0.71807953	0.29081489
nonDEG	-1.3173033	0.05103913	1	nonDEG	0.86422345	0.12824304
nonDEG	0.50104963	0.27490366	1	nonDEG	0.04430851	0.90536018
nonDEG	1.34939862	0.00611814	0.73793371	nonDEG	-0.2909455	0.42932693
nonDEG	0.79900178	0.07394399	1	nonDEG	-0.2732369	0.49331425
nonDEG	1.12679398	0.06163167	1	nonDEG	-0.5149296	0.34799802
nonDEG	-1.1665552	0.04159056	1	nonDEG	1.32505214	0.01182531

nonDEG	0.90970897	0.08231099	1	nonDEG	-0.102725	0.81887751
nonDEG	1.3292945	0.0088452	0.7914707	nonDEG	-0.7423588	0.05144183
nonDEG	2.58210758	0.01958633	0.97153571	nonDEG	-1.8992223	0.12059642
nonDEG	0.81466841	0.07741207	1	nonDEG	-0.5453771	0.13434957
nonDEG	0.49896046	0.32838211	1	nonDEG	-0.7343695	0.05723608
nonDEG	-0.9612582	0.08326358	1	nonDEG	1.36792814	0.00843067
nonDEG	-0.0037097	0.99395702	1	nonDEG	-0.3653817	0.31991912
nonDEG	-2.5314654	0.03079005	1	nonDEG	2.49267811	0.00752963
nonDEG	-1.4775299	0.06116538	1	nonDEG	1.06416879	0.22779987
nonDEG	2.33173122	0.01671882	0.92342469	nonDEG	-0.9762293	0.29616702
nonDEG	-0.017451	0.96920996	1	nonDEG	-0.5284986	0.17822148
nonDEG	-1.4637471	0.01446283	0.88827361	nonDEG	2.59926946	0.00116315
nonDEG	0.43224692	0.35800357	1	nonDEG	0.02742515	0.94056729
nonDEG	-1.2563237	0.02109983	0.99864599	nonDEG	1.37805284	0.00973696
nonDEG	0.66721034	0.1349338	1	nonDEG	0.02660607	0.9468146
nonDEG	-0.2690203	0.54040991	1	nonDEG	-0.4834828	0.20798361
nonDEG	0.58051157	0.42294294	1	nonDEG	0.47062751	0.51166607
nonDEG	0.36593818	0.40440584	1	nonDEG	-0.1805046	0.61614956
nonDEG	-1.9942917	0.02884954	1	nonDEG	2.90407872	0.00339176
nonDEG	0.55083778	0.28177516	1	nonDEG	0.28268018	0.51518433
nonDEG	0.37187521	0.38271186	1	nonDEG	-0.0919548	0.79898083
nonDEG	0.6022138	0.16484257	1	nonDEG	-0.2672728	0.47184209
nonDEG	0.52019498	0.26966744	1	nonDEG	-0.1871879	0.64550707
nonDEG	1.00847316	0.06002286	1	nonDEG	-0.0557874	0.90131051
nonDEG	-2.5811379	0.00212805	0.61198441	nonDEG	2.0815957	0.00660354
nonDEG	0.53503267	0.52067624	1	nonDEG	-1.0447995	0.10580443
nonDEG	-0.5111326	0.254273	1	nonDEG	0.21917474	0.5470638
nonDEG	1.29536752	0.15816177	1	nonDEG	0.05682126	0.96157986
nonDEG	0.18716306	0.70690225	1	nonDEG	-0.4303132	0.29622244
nonDEG	-0.0434302	0.92037747	1	nonDEG	-0.4614077	0.21268012
nonDEG	0.75999199	0.0989119	1	nonDEG	0.2365438	0.52202385
nonDEG	-1.7997662	0.05418203	1	nonDEG	1.09317014	0.30823377
nonDEG	-0.1844606	0.67037774	1	nonDEG	0.02802054	0.94101763
nonDEG	-1.7865193	0.00415472	0.68407511	nonDEG	1.50637763	0.04141013
nonDEG	0.35228757	0.42619417	1	nonDEG	-0.3764962	0.2998766
nonDEG	0.37682925	0.49241363	1	nonDEG	-0.352733	0.43017542
nonDEG	-0.7833317	0.35661133	1	nonDEG	2.01911049	0.02554419
nonDEG	-0.2211087	0.61100315	1	nonDEG	-0.1883357	0.6152277
nonDEG	-1.1965965	0.1137243	1	nonDEG	0.2969163	0.70085612
nonDEG	-0.2740319	0.52138358	1	nonDEG	0.17170934	0.63522273
nonDEG	-0.1875606	0.72674673	1	nonDEG	-0.0017743	1
nonDEG	-1.0567142	0.09837757	1	nonDEG	0.58712418	0.36589134
nonDEG	-0.2309866	0.59571301	1	nonDEG	-0.0290761	0.93738675

nonDEG	-0.1280309	0.79036568	1	nonDEG	-0.0966656	0.83001335
nonDEG	-0.3486125	0.42310311	1	nonDEG	0.10321786	0.7756234
nonDEG	0.43852281	0.3723823	1	nonDEG	-0.6618011	0.12051622
nonDEG	1.17404802	0.08995561	1	nonDEG	-0.3554317	0.57534194
nonDEG	-2.5172653	0.02227305	1	nonDEG	0.9396393	0.52449744
nonDEG	-0.0279575	0.94944917	1	nonDEG	0.13462252	0.72102107
nonDEG	-0.0951116	0.82710139	1	nonDEG	-0.0870012	0.81235652
nonDEG	1.08587668	0.19652472	1	nonDEG	-0.2905301	0.64671086
nonDEG	0.17175665	0.70284972	1	nonDEG	-0.1579581	0.66876033
nonDEG	0.51912946	0.4208754	1	nonDEG	-0.8740907	0.03301127
nonDEG	-0.4981439	0.2700213	1	nonDEG	0.07839419	0.84250257
nonDEG	0.40030892	0.38911651	1	nonDEG	-0.4962499	0.22014626
nonDEG	1.50625807	0.00190126	0.61198441	nonDEG	-0.307723	0.44875412
nonDEG	0.44506326	0.53615462	1	nonDEG	0.14790473	0.82336484
nonDEG	-0.2747223	0.5693788	1	nonDEG	-1.0022676	0.0441975
nonDEG	-1.1807196	0.43017263	1	nonDEG	0.75486407	0.64574582
nonDEG	1.13239626	0.18514236	1	nonDEG	-1.5729661	0.20574479
nonDEG	-0.2400982	0.56979259	1	nonDEG	0.56716592	0.11344343
nonDEG	-0.2467462	0.6535442	1	nonDEG	0.27204227	0.59804924
nonDEG	-1.2021446	0.04140636	1	nonDEG	1.38251106	0.01832063
nonDEG	0.06047808	0.89304483	1	nonDEG	-0.1800739	0.62255848
nonDEG	-4.0293104	0.3526449	1	nonDEG	-5.13E-15	1
nonDEG	0.08523568	0.84947775	1	nonDEG	0.43327741	0.23808958
nonDEG	0.23863838	0.61410814	1	nonDEG	-0.2691126	0.47974215
nonDEG	0.45007718	0.33794241	1	nonDEG	-0.138321	0.70008858
nonDEG	0.48348555	0.26238373	1	nonDEG	-0.1356485	0.71491008
nonDEG	-0.761989	0.10205944	1	nonDEG	0.48342206	0.17778121
nonDEG	1.34697062	0.1120311	1	nonDEG	-0.1162125	0.87605866
nonDEG	0.73023836	0.25468197	1	nonDEG	-0.0899135	0.88247252
nonDEG	0.38288692	0.65452019	1	nonDEG	-0.2111678	0.79719366
nonDEG	-0.8365738	0.35189992	1	nonDEG	1.74693704	0.07267599
nonDEG	-0.5559331	0.35432479	1	nonDEG	-0.0326436	0.95582868
nonDEG	-0.8887434	0.08485181	1	nonDEG	0.39247955	0.43999305
nonDEG	-0.5682557	0.2199621	1	nonDEG	0.19180329	0.66488739
nonDEG	-0.4483703	0.3114341	1	nonDEG	0.25361938	0.49018888
nonDEG	-0.9214475	0.06765601	1	nonDEG	0.24908902	0.59697299
nonDEG	-0.3537821	0.43331482	1	nonDEG	0.02901183	0.93738911
nonDEG	0.30121532	0.56386454	1	nonDEG	-0.5170724	0.3091037
nonDEG	0.69407555	0.14150481	1	nonDEG	-0.2982305	0.51188279
nonDEG	-1.3072329	0.10455648	1	nonDEG	1.60956672	0.03069733
nonDEG	0.38165567	0.4178596	1	nonDEG	0.04234599	0.9076381
nonDEG	2.71254081	0.00337961	0.65691312	nonDEG	-1.5607554	0.05491515
nonDEG	-0.6139215	0.2409084	1	nonDEG	0.74578131	0.12984703

nonDEG	0.81150778	0.14361622	1	nonDEG	0.1436218	0.7352895
nonDEG	0.30109883	0.50793181	1	nonDEG	0.10808545	0.77912698
nonDEG	0.45521717	0.35752096	1	nonDEG	-0.0447885	0.9138007
nonDEG	-0.6247965	0.38529582	1	nonDEG	0.77677516	0.29089081
nonDEG	0.75910089	0.12348093	1	nonDEG	0.00218628	0.99554101
nonDEG	2.79531589	0.0026346	0.61776954	nonDEG	-0.8041576	0.34298358
nonDEG	1.19550202	0.01013951	0.80674895	nonDEG	-0.3228803	0.37175239
nonDEG	-0.516933	0.30676268	1	nonDEG	0.22236731	0.6415765
nonDEG	0.54168351	0.29685712	1	nonDEG	-0.2905651	0.4699728
nonDEG	-1.1552394	0.04798798	1	nonDEG	0.69376883	0.20173148
nonDEG	0.96543301	0.18599916	1	nonDEG	-0.7317503	0.17915874
nonDEG	-0.0807108	0.85356403	1	nonDEG	0.01503585	0.96769741
nonDEG	0.4156486	0.32848873	1	nonDEG	-0.1297109	0.72340981
nonDEG	-1.8449756	0.01427404	0.88752292	nonDEG	0.58507874	0.26231202
nonDEG	-1.1010463	0.02578277	1	nonDEG	1.21823563	0.00701154
nonDEG	-0.6722782	0.16688934	1	nonDEG	0.50712589	0.22292357
nonDEG	1.03279137	0.06671728	1	nonDEG	-1.1535954	0.01869063
nonDEG	0.44957189	0.31081432	1	nonDEG	-0.2026707	0.57669801
nonDEG	2.59591858	0.00869357	0.7876977	nonDEG	-0.8709406	0.33404737
nonDEG	-1.4224573	0.17710993	1	nonDEG	0.97818273	0.32613743
nonDEG	1.83421436	0.00087302	0.50393775	nonDEG	-1.727214	0.00184793
nonDEG	-1.3182546	0.04150202	1	nonDEG	1.05619418	0.08218802
nonDEG	-0.37679	0.37024765	1	nonDEG	0.03628685	0.92189929
nonDEG	3.78489991	0.46876184	1	nonDEG	-3.987536	0.44091986
nonDEG	-0.989226	0.06793597	1	nonDEG	0.99097546	0.06993289
nonDEG	0.92221207	0.27075953	1	nonDEG	-0.046425	0.93848361
nonDEG	-0.0638129	0.88603352	1	nonDEG	-0.5262267	0.14829549
nonDEG	0.40853244	0.35642584	1	nonDEG	-0.3625383	0.33581847
nonDEG	1.02767655	0.24417503	1	nonDEG	-1.5858913	0.06681025
nonDEG	0.79035636	0.27349147	1	nonDEG	-0.4571089	0.48074773
nonDEG	-0.5343153	0.30999348	1	nonDEG	0.04567019	0.92627567
nonDEG	0.45042132	0.31095683	1	nonDEG	-0.0551866	0.88451998
nonDEG	0.35540944	0.45456808	1	nonDEG	-0.2248482	0.60227236
nonDEG	-0.7042806	0.09705748	1	nonDEG	0.49210737	0.17269475
nonDEG	0.1563623	0.93305977	1	nonDEG	1.26746962	0.20270258
nonDEG	0.49182099	0.25780736	1	nonDEG	0.05476742	0.88257492
nonDEG	-0.0855016	0.89470974	1	nonDEG	0.17579446	0.77514959
nonDEG	-0.3122226	0.48613775	1	nonDEG	-0.0115471	0.97495532
nonDEG	0.27761881	0.53961386	1	nonDEG	-0.3147306	0.38569474
nonDEG	-0.8335962	0.08453448	1	nonDEG	-1.0109866	0.02256943
nonDEG	-1.1271544	0.06978403	1	nonDEG	0.09924426	0.86634432
nonDEG	0.224494	0.72131993	1	nonDEG	-0.006934	0.9867944
nonDEG	0.15858234	0.73099949	1	nonDEG	-0.0355793	0.92765772

nonDEG	-0.5477289	0.5209511	1	nonDEG	0.88000155	0.31198497
DEG	-2.1678614	5.00E-05	0.14400617	nonDEG	2.17305096	0.00141448
DEG	3.43574043	0.00314207	0.65616368	nonDEG	-0.418215	0.69144461
nonDEG	1.74405377	0.00819858	0.77656086	nonDEG	-1.4613859	0.02154058
nonDEG	1.32802358	0.17254778	1	nonDEG	-1.6261504	0.03404818
nonDEG	-1.9612617	0.00763248	0.75581042	nonDEG	2.49459648	0.01629097
nonDEG	0.53249118	0.47205849	1	nonDEG	-0.2556867	0.64244983
nonDEG	-1.0248751	0.047116	1	nonDEG	0.47574651	0.32947232
nonDEG	-0.7068618	0.19661442	1	nonDEG	0.03870175	0.93645993
nonDEG	-0.4580194	0.29926599	1	nonDEG	-0.3878986	0.34147842
nonDEG	1.13000166	0.02535299	1	nonDEG	-0.4114981	0.27046733
nonDEG	1.52349408	0.27536678	1	nonDEG	-0.0438924	0.99120686
nonDEG	0.94468941	0.14577852	1	nonDEG	0.45589466	0.41940814
nonDEG	1.11682827	0.0771865	1	nonDEG	0.05114569	0.93001936
nonDEG	0.63415251	0.2105143	1	nonDEG	-0.3392856	0.45137921
nonDEG	-0.1259639	0.77074752	1	nonDEG	-0.2885563	0.44781079
nonDEG	-0.2381373	0.57919737	1	nonDEG	-0.1316994	0.72868784
nonDEG	0.08553614	0.86616359	1	nonDEG	-0.0479733	0.90485013
nonDEG	-0.8231908	0.16686324	1	nonDEG	0.76739029	0.26141084
nonDEG	-0.236235	0.68330137	1	nonDEG	0.08469392	0.86893698
nonDEG	0.58296231	0.30514211	1	nonDEG	-0.2513479	0.55742875
nonDEG	2.14463835	0.0042441	0.68407511	nonDEG	-1.0245042	0.14510973
nonDEG	0.81447408	0.13226639	1	nonDEG	0.25887054	0.59135943
nonDEG	0.80712227	0.12352783	1	nonDEG	-0.403209	0.31252805
nonDEG	-0.5943844	0.16344679	1	nonDEG	0.59007316	0.145213
nonDEG	-0.246755	0.57653888	1	nonDEG	-0.2195788	0.6028287
nonDEG	1.79650873	0.0580327	1	nonDEG	0.40545851	0.58714438
nonDEG	-0.7454273	0.11779927	1	nonDEG	0.08430737	0.87552612
nonDEG	1.21759966	0.16558562	1	nonDEG	0.55459214	0.48400638
nonDEG	2.0567921	0.00973867	0.79637955	nonDEG	-1.8507953	0.03154243
nonDEG	1.47145797	0.020705	0.99114571	nonDEG	-1.1878919	0.01328533
nonDEG	0.51347665	0.30932522	1	nonDEG	-0.6573352	0.10153753
nonDEG	0.85498035	0.15384387	1	nonDEG	-0.6210918	0.32850446
nonDEG	-1.055012	0.03465641	1	nonDEG	0.8037582	0.05731834
nonDEG	-0.6709602	0.1681575	1	nonDEG	-0.5355875	0.23887248
nonDEG	-0.4501094	0.31064851	1	nonDEG	-0.0550505	0.88085432
nonDEG	-0.1103461	0.79865297	1	nonDEG	-0.4345156	0.23130723
nonDEG	-2.0477248	0.04087072	1	nonDEG	2.16956801	0.04902215
nonDEG	0.23303907	0.59723961	1	nonDEG	0.33872038	0.35052695
nonDEG	1.34939055	0.00782345	0.75581042	nonDEG	0.00755418	0.9872158
nonDEG	-0.8564335	0.17896287	1	nonDEG	0.72919637	0.14434475
nonDEG	1.2566873	0.06899458	1	nonDEG	-0.6606953	0.29436381
nonDEG	1.68263734	0.08916096	1	nonDEG	-0.1610281	0.83751189

nonDEG	-1.2185281	0.09342501	1	nonDEG	2.29195793	0.00437234
nonDEG	0.60212561	0.48607935	1	nonDEG	-0.1060045	0.84428147
nonDEG	2.2733826	0.01170679	0.85229819	nonDEG	-0.7790029	0.34193725
nonDEG	0.52703073	0.231154	1	nonDEG	-0.0285799	0.94017228
nonDEG	0.1909316	0.72179621	1	nonDEG	-0.0626191	0.88873786
nonDEG	-0.2569528	0.55931822	1	nonDEG	0.31248618	0.42103484
nonDEG	-1.6168023	0.12887431	1	nonDEG	1.22351489	0.24422371
nonDEG	-0.0805858	0.85229244	1	nonDEG	0.04585682	0.90167726
nonDEG	-0.7563572	0.5975088	1	nonDEG	-0.4643958	0.80629334
nonDEG	-0.30773	0.47797632	1	nonDEG	0.24054322	0.52355542
nonDEG	0.05229731	0.96239743	1	nonDEG	-0.4053255	0.66829011
nonDEG	1.78456724	0.00073634	0.50181787	nonDEG	-0.3683256	0.41686552
nonDEG	0.2852619	0.76271437	1	nonDEG	-0.5523804	0.45255036
nonDEG	-2.3915994	0.0015974	0.57348672	nonDEG	1.88197311	0.00032552
nonDEG	-0.372456	0.39696899	1	nonDEG	0.30935401	0.39743004
nonDEG	-0.6218882	0.14418324	1	nonDEG	0.20902865	0.58833435
nonDEG	0.97340719	0.12729012	1	nonDEG	-0.3655817	0.41145456
nonDEG	-0.7196742	0.25950352	1	nonDEG	0.06226258	0.91424201
nonDEG	0.45852489	0.7371187	1	nonDEG	-0.5970357	0.49903467
nonDEG	-0.2847163	0.51911111	1	nonDEG	0.04614983	0.89997836
nonDEG	-0.4229325	0.33506208	1	nonDEG	0.09104231	0.80403829
nonDEG	1.43501087	0.00603008	0.73793371	nonDEG	-0.6459215	0.12428274
nonDEG	0.25281188	0.65633137	1	nonDEG	-0.3773944	0.48871531
nonDEG	1.86996431	0.00558555	0.73507048	nonDEG	-0.4268365	0.35730201
nonDEG	1.1849675	0.02621316	1	nonDEG	0.21630798	0.57818538
nonDEG	1.62295545	0.04354382	1	nonDEG	-0.5635332	0.42461783
nonDEG	0.09117721	0.84438222	1	nonDEG	-0.282993	0.48140884
nonDEG	-0.2491615	0.56651773	1	nonDEG	-0.4143677	0.25535853
nonDEG	1.50828314	0.14111448	1	nonDEG	-1.4028638	0.1602781
nonDEG	0.82939167	0.08406643	1	nonDEG	-0.2616532	0.48054304
nonDEG	0.38664017	0.40092132	1	nonDEG	0.00465183	0.99181899
nonDEG	-0.0612478	0.94859362	1	nonDEG	-0.8228664	0.39119481
nonDEG	0.67383835	0.33787255	1	nonDEG	-0.5755459	0.4272877
nonDEG	1.79996285	0.02946056	1	nonDEG	-1.6075204	0.02987052
nonDEG	-0.5261017	0.28556368	1	nonDEG	-0.1914929	0.64606583
nonDEG	0.62946675	0.26259631	1	nonDEG	-0.4092953	0.34464256
nonDEG	0.80428674	0.10638536	1	nonDEG	0.02467276	0.95893593
nonDEG	1.49803758	0.05387037	1	nonDEG	-0.5686803	0.42955346
nonDEG	-0.6238093	0.51026103	1	nonDEG	-0.310172	0.79997559
nonDEG	0.03614706	0.96519378	1	nonDEG	0.07234443	0.91795742
nonDEG	1.60812632	0.08222337	1	nonDEG	-1.3511409	0.04313667
nonDEG	-1.0006045	0.12968146	1	nonDEG	-0.4705984	0.46360085
nonDEG	0.9653951	0.04448796	1	nonDEG	-0.1826908	0.6514411

nonDEG	-0.8056409	0.29104973	1	nonDEG	0.37447156	0.65353996
nonDEG	-0.225072	0.6067827	1	nonDEG	-0.324453	0.38535981
nonDEG	2.46665347	0.00369338	0.67010784	nonDEG	-0.5549882	0.45359331
nonDEG	-1.5312283	0.28702293	1	nonDEG	0.78951279	0.71259402
nonDEG	-0.6129477	0.26071593	1	nonDEG	0.09317437	0.86043988
nonDEG	0.34083817	0.43145625	1	nonDEG	-0.2583925	0.50126635
nonDEG	-0.5872839	0.18801403	1	nonDEG	0.59643267	0.10282105
nonDEG	-0.5174993	0.2581461	1	nonDEG	-0.4440604	0.34413498
nonDEG	-0.0830272	0.94097852	1	nonDEG	0.4945219	0.75814109
nonDEG	-0.7534503	0.21395425	1	nonDEG	1.44497722	0.00443652
nonDEG	0.37665817	0.4221924	1	nonDEG	0.02074341	0.95664345
nonDEG	0.50640699	0.2361076	1	nonDEG	-0.0691639	0.84934422
nonDEG	0.60480366	0.33172444	1	nonDEG	-0.1488285	0.78540642
nonDEG	0.74435759	0.18248193	1	nonDEG	-0.7146242	0.13438064
nonDEG	-1.2403454	0.11900007	1	nonDEG	0.21090484	0.80800775
nonDEG	1.54410674	0.02830207	1	nonDEG	0.15412447	0.79627319
nonDEG	0.23109623	0.62655556	1	nonDEG	-0.0827651	0.83979698
nonDEG	0.78977226	0.5685634	1	nonDEG	0.14668778	0.96550765
nonDEG	-0.2168351	0.67940985	1	nonDEG	0.23175681	0.6404595
nonDEG	0.61472468	0.34259248	1	nonDEG	-0.295802	0.64497577
nonDEG	0.33141819	0.49956879	1	nonDEG	-0.1613852	0.68092717
nonDEG	0.03060442	0.94573961	1	nonDEG	-0.2496412	0.49029637
nonDEG	1.00846631	0.1476862	1	nonDEG	-0.9350915	0.16565147
nonDEG	1.23399555	0.06115267	1	nonDEG	-0.3994571	0.37504586
nonDEG	0.02130779	0.96192018	1	nonDEG	-0.1058744	0.79156288
nonDEG	-0.4509568	0.28925756	1	nonDEG	-0.1058489	0.78673463
nonDEG	-0.78862	0.10761953	1	nonDEG	0.18880907	0.68347368
nonDEG	-0.103558	0.91787097	1	nonDEG	0.04412931	0.97266637
nonDEG	-0.7212225	0.09552311	1	nonDEG	0.26574884	0.49302006
nonDEG	-0.1738164	0.75723234	1	nonDEG	0.56999772	0.24296653
nonDEG	-2.1085154	0.02542835	1	nonDEG	2.20651923	0.06137094
nonDEG	0.30460547	0.5598066	1	nonDEG	-0.0834965	0.87012938
nonDEG	1.5913582	0.01575609	0.91028043	nonDEG	-0.3781196	0.50651945
nonDEG	-0.9996791	0.04705581	1	nonDEG	0.73543189	0.11629296
nonDEG	1.54561687	0.01733867	0.93380915	nonDEG	-0.4856504	0.36802422
nonDEG	0.46793873	0.27808446	1	nonDEG	-0.0408759	0.91167855
nonDEG	0.11335151	0.90557439	1	nonDEG	-0.6208491	0.29509192
nonDEG	0.68279242	0.12174152	1	nonDEG	0.06642656	0.86044817
nonDEG	0.52568404	0.24528072	1	nonDEG	0.33914879	0.39316472
nonDEG	-0.1587709	0.70630511	1	nonDEG	-0.2023404	0.57646148
nonDEG	-0.3690836	0.40789473	1	nonDEG	-0.2323081	0.55805075
nonDEG	0.1493562	0.73110886	1	nonDEG	0.03340048	0.92848928
nonDEG	1.3656229	0.0363969	1	nonDEG	-0.1983522	0.68023478

nonDEG	1.13457124	0.06072407	1	nonDEG	-0.886197	0.12867145
nonDEG	1.52757198	0.00084218	0.50181787	nonDEG	-0.9146437	0.01186961
nonDEG	0.59760327	0.32488468	1	nonDEG	-0.8882676	0.07768891
nonDEG	0.74268286	0.10417904	1	nonDEG	-0.7936192	0.0391522
nonDEG	-0.3742667	0.45982597	1	nonDEG	-0.0262805	0.95883756
nonDEG	0.28208628	0.539728	1	nonDEG	-0.325975	0.3928264
nonDEG	0.61668691	0.23924011	1	nonDEG	-0.2089132	0.64678347
nonDEG	0.33907225	0.63186179	1	nonDEG	0.20028905	0.75880719
nonDEG	0.66949793	0.42359013	1	nonDEG	0.55977236	0.45440801
nonDEG	0.07226293	0.88506465	1	nonDEG	0.87355931	0.02754374
nonDEG	-1.2999931	0.00318812	0.65616368	nonDEG	1.02746514	0.0134258
nonDEG	-1.4569666	0.00606435	0.73793371	nonDEG	1.78790131	0.00834859
nonDEG	0.8058323	0.45952906	1	nonDEG	0.87434767	0.42466567
nonDEG	1.34071696	0.00494353	0.70646871	nonDEG	-0.3495728	0.38974402
nonDEG	-0.7280292	0.13923472	1	nonDEG	-0.0091794	0.98457023
nonDEG	0.38640276	0.46224283	1	nonDEG	-0.3272549	0.52319017
nonDEG	-0.0561948	0.8976677	1	nonDEG	0.57217832	0.1137859
nonDEG	-0.814991	0.2328005	1	nonDEG	0.67433165	0.3179507
nonDEG	-0.0910533	0.83271411	1	nonDEG	-0.1500028	0.68128385
nonDEG	0.34438474	0.54336673	1	nonDEG	-0.2849197	0.60516787
nonDEG	2.82893022	0.05323673	1	nonDEG	-1.46357	0.26263287
nonDEG	0.79004573	0.3508168	1	nonDEG	-1.2864743	0.13439711
nonDEG	0.27208794	0.5163697	1	nonDEG	-0.276282	0.44509513
nonDEG	1.42908698	0.00911202	0.79328284	nonDEG	-0.4810796	0.18730296
nonDEG	0.06760671	0.87727961	1	nonDEG	0.22413668	0.54420245
nonDEG	0.51753816	0.31442778	1	nonDEG	0.08821167	0.84709169
nonDEG	0.52638931	0.29935542	1	nonDEG	-0.4971319	0.19877275
nonDEG	-0.6106122	0.25567519	1	nonDEG	0.6061607	0.24332374
nonDEG	-2.5333714	0.01451339	0.88827361	nonDEG	3.16172419	0.01110745
nonDEG	-0.2523241	0.59408852	1	nonDEG	-0.1746129	0.67717826
nonDEG	1.39753194	0.29203314	1	nonDEG	-0.6678137	0.57415155
nonDEG	1.6921428	0.03378854	1	nonDEG	-0.0859702	0.91210851
nonDEG	-0.4793473	0.64713464	1	nonDEG	0.26043981	0.79969178
nonDEG	-0.3062282	0.6182478	1	nonDEG	-0.110436	0.85756735
nonDEG	0.16582408	0.73189349	1	nonDEG	0.12523382	0.73846369
nonDEG	1.0995692	0.08672489	1	nonDEG	0.39282203	0.48783679
nonDEG	-2.028972	0.08345206	1	nonDEG	2.2600268	0.07335157
nonDEG	-0.6290034	0.19140211	1	nonDEG	0.76210971	0.0820072
nonDEG	1.18766845	0.15968478	1	nonDEG	0.11915461	0.88667408
nonDEG	-0.9475235	0.27424668	1	nonDEG	1.51705419	0.12314022
nonDEG	1.39023139	0.00697731	0.75126991	nonDEG	-0.884032	0.05611856
nonDEG	-0.3385565	0.45268192	1	nonDEG	-0.0210315	0.95566088
nonDEG	0.89329818	0.39665734	1	nonDEG	-0.0435366	0.98124568

nonDEG	0.22657781	0.76938723	1	nonDEG	-0.1778409	0.83002082
nonDEG	-0.4941517	0.34301221	1	nonDEG	0.40359318	0.38537022
nonDEG	1.23134391	0.06461371	1	nonDEG	-0.0246287	0.95679308
nonDEG	0.17362554	0.72439466	1	nonDEG	-0.2294207	0.54930498
nonDEG	0.87774322	0.21695943	1	nonDEG	-0.7955605	0.12721511
nonDEG	-0.3167514	0.49582975	1	nonDEG	-0.0972678	0.82508778
nonDEG	0.38493437	0.40333591	1	nonDEG	-0.4768589	0.21641651
nonDEG	-0.0680475	0.87590414	1	nonDEG	0.36834606	0.38969052
nonDEG	-0.7409164	0.13825116	1	nonDEG	0.19980265	0.63393873
nonDEG	2.70545334	0.00626972	0.73793371	nonDEG	-2.8846952	0.00354627
nonDEG	0.6326257	0.18189168	1	nonDEG	-0.4592632	0.26313658
nonDEG	1.13122508	0.06691806	1	nonDEG	-0.9682524	0.10778482
nonDEG	0.066584	0.89419972	1	nonDEG	-0.0581159	0.89539683
nonDEG	-0.9586657	0.05490563	1	nonDEG	0.33032342	0.47967326
nonDEG	-0.0219954	0.96818767	1	nonDEG	-0.7598981	0.15127973
nonDEG	0.86479216	0.44548457	1	nonDEG	0.19510483	0.88029762
nonDEG	0.4074939	0.36972812	1	nonDEG	-0.3830998	0.29271488
nonDEG	0.26477134	0.59393799	1	nonDEG	-0.4608661	0.30587561
nonDEG	-0.2470325	0.59338643	1	nonDEG	0.09791271	0.817297
nonDEG	-0.9408153	0.0839718	1	nonDEG	0.8924639	0.05972378
nonDEG	-0.3906944	0.40554139	1	nonDEG	0.53646062	0.15681509
nonDEG	-0.1027001	0.80809323	1	nonDEG	-0.44687	0.21615074
nonDEG	1.29118385	0.01518713	0.89448687	nonDEG	-0.1587276	0.71972212
nonDEG	0.38279829	0.36602357	1	nonDEG	-0.3768957	0.30097574
nonDEG	-0.1209316	0.8085493	1	nonDEG	-0.1384884	0.77977867
nonDEG	-0.0939847	0.84791591	1	nonDEG	-0.1369924	0.7383159
nonDEG	-0.5673773	0.31217686	1	nonDEG	-0.3444143	0.46823686
nonDEG	0.42712889	0.46763297	1	nonDEG	0.09667735	0.8210741
nonDEG	0.16320422	0.70809357	1	nonDEG	-0.0711508	0.84626034
nonDEG	-0.464295	0.30166331	1	nonDEG	-0.2905553	0.44195048
nonDEG	0.26501766	0.54079922	1	nonDEG	-0.3077508	0.39927759
nonDEG	2.17303465	0.05200716	1	nonDEG	0.4997714	0.6208699
nonDEG	0.70065266	0.24281226	1	nonDEG	-0.5028419	0.39939469
nonDEG	0.84180126	0.10588257	1	nonDEG	-0.1191372	0.80512006
nonDEG	0.5782516	0.26055205	1	nonDEG	-0.3128139	0.42574798
nonDEG	-0.3002976	0.50422282	1	nonDEG	-0.1844619	0.67868066
nonDEG	-0.2676794	0.53692788	1	nonDEG	-0.0602758	0.86956678
nonDEG	0.60068625	0.52555379	1	nonDEG	-0.5761311	0.39475215
nonDEG	0.274389	0.5630867	1	nonDEG	-0.2035771	0.59336247
nonDEG	0.90546943	0.31873778	1	nonDEG	-0.6501117	0.47602985
nonDEG	3.95540573	3.91E-06	0.03026052	DEG	-1.3120681	0.07231865
nonDEG	-0.1266618	0.76627398	1	nonDEG	-0.5013273	0.17906849
nonDEG	0.37248952	0.4084676	1	nonDEG	-0.3068878	0.44253109

nonDEG	0.25170606	0.76570369	1	nonDEG	0.28293285	0.72527844
nonDEG	-0.3103311	0.53549442	1	nonDEG	0.68030847	0.07406441
nonDEG	-0.6714928	0.12903148	1	nonDEG	0.5842675	0.14525666
nonDEG	-0.4582919	0.2995492	1	nonDEG	0.47360318	0.21096367
nonDEG	-1.3454172	0.15305124	1	nonDEG	1.78903449	0.06990102
nonDEG	-0.5791938	0.23429349	1	nonDEG	0.56331592	0.17834685
nonDEG	-0.5144021	0.23710421	1	nonDEG	0.0813472	0.82545289
nonDEG	-1.4871239	0.06228216	1	nonDEG	0.07499795	0.89358469
nonDEG	1.60159847	0.10882886	1	nonDEG	0.8560648	0.32328849
nonDEG	1.41217464	0.07253527	1	nonDEG	-0.7536751	0.25995774
nonDEG	0.14502542	0.77122613	1	nonDEG	-0.6776867	0.11407702
nonDEG	1.40150146	0.02301704	1	nonDEG	-1.1248757	0.10862301
nonDEG	1.32021915	0.02777647	1	nonDEG	-0.2954829	0.54869337
nonDEG	0.22648915	0.61073521	1	nonDEG	0.28985871	0.46034989
nonDEG	0.18259967	0.66672488	1	nonDEG	-0.0518625	0.88510469
nonDEG	-0.9600094	0.2267226	1	nonDEG	0.65949369	0.46286008
nonDEG	1.27325195	0.1478667	1	nonDEG	-1.1803328	0.23592803
nonDEG	-1.2041758	0.00945073	0.79482907	nonDEG	1.09678357	0.00522011
nonDEG	-0.6993396	0.39872202	1	nonDEG	0.5755633	0.45401864
nonDEG	-0.1483533	0.85323523	1	nonDEG	-0.1648021	0.83635416
nonDEG	-1.0286541	0.10347626	1	nonDEG	0.7023877	0.28224315
nonDEG	-0.5282531	0.32722933	1	nonDEG	0.46173484	0.35979129
nonDEG	2.04421245	0.00809516	0.77124119	nonDEG	-0.2650318	0.68232425
nonDEG	1.4076322	0.13472582	1	nonDEG	-1.9118322	0.01863905
nonDEG	-0.3800599	0.37346343	1	nonDEG	0.33745136	0.3717039
nonDEG	1.0299038	0.13617304	1	nonDEG	-0.2103969	0.72682443
nonDEG	0.80902495	0.2739609	1	nonDEG	-0.1674056	0.80161687
nonDEG	-1.0722143	0.02652625	1	nonDEG	0.99980113	0.03180731
DEG	2.16237535	0.00111464	0.51315728	nonDEG	-1.2562556	0.05437436
nonDEG	1.57031119	0.05113618	1	nonDEG	-0.7451586	0.32015382
nonDEG	-1.1002929	0.06272165	1	nonDEG	1.32473378	0.01573438
nonDEG	-1.2088854	0.01232481	0.85229819	nonDEG	1.45008633	0.00552223
nonDEG	0.43661658	0.34699465	1	nonDEG	-0.0571306	0.8903932
nonDEG	-1.3841079	0.07360552	1	nonDEG	1.47323272	0.03088174
nonDEG	-1.0109391	0.24390248	1	nonDEG	1.58659474	0.07309782
nonDEG	-0.1943607	0.65674706	1	nonDEG	0.09127022	0.80142569
nonDEG	-1.064931	0.24341871	1	nonDEG	1.81641486	0.09442746
nonDEG	-3.289908	0.64165682	1	nonDEG	-5.13E-15	1
nonDEG	-0.7046577	0.27521333	1	nonDEG	0.15596487	0.80500984
nonDEG	-1.138166	0.15139135	1	nonDEG	1.02965816	0.21018979
nonDEG	-0.8975552	0.22405675	1	nonDEG	2.43359684	0.00602925
nonDEG	-0.19097	0.66083193	1	nonDEG	-0.295187	0.42898998
nonDEG	0.45075731	0.29461127	1	nonDEG	-0.2453984	0.51659533

nonDEG	-0.2524813	0.5739758	1	nonDEG	0.36840945	0.31320454
nonDEG	0.09121466	0.8344605	1	nonDEG	0.02279731	0.95064421
nonDEG	0.03412918	0.94333921	1	nonDEG	-0.1187652	0.79098806
nonDEG	-0.1121161	0.80069124	1	nonDEG	0.30138204	0.40667844
nonDEG	0.42201882	0.3173855	1	nonDEG	0.01408313	0.96982978
nonDEG	0.08409581	0.94095089	1	nonDEG	0.54369618	0.57763915
nonDEG	-0.5145052	0.31336379	1	nonDEG	-0.0372601	0.93603444
nonDEG	0.66270363	0.18206194	1	nonDEG	-0.2381199	0.5191353
nonDEG	-0.431226	0.35828574	1	nonDEG	0.50771999	0.1718308
nonDEG	-1.7128682	0.09311986	1	nonDEG	1.01959918	0.54707247
nonDEG	1.76812556	0.00593526	0.73793371	nonDEG	-0.3706515	0.49843791
nonDEG	0.25438551	0.57348043	1	nonDEG	-0.0700642	0.85070808
nonDEG	0.55818539	0.29990005	1	nonDEG	0.18277838	0.70300695
nonDEG	-0.522407	0.69511905	1	nonDEG	1.87933346	0.15357905
nonDEG	-0.0094598	1	1	nonDEG	0.25268436	0.72702961
nonDEG	0.46898566	0.36606777	1	nonDEG	-0.9468625	0.01573062
nonDEG	0.85812434	0.5219282	1	nonDEG	1.02224491	0.45232846
nonDEG	-0.854045	0.04850725	1	nonDEG	0.57909733	0.1222211
nonDEG	-0.4852696	0.29701432	1	nonDEG	0.22674333	0.54606266
nonDEG	-0.1556679	0.71351546	1	nonDEG	-0.0745191	0.83584968
nonDEG	-0.2303655	0.58325547	1	nonDEG	0.06740561	0.85570502
nonDEG	-0.7050565	0.14043159	1	nonDEG	0.83985369	0.02150748
nonDEG	-0.2769033	0.53128492	1	nonDEG	-0.5146763	0.1519513
nonDEG	0.5558857	0.43440029	1	nonDEG	0.10757927	0.88093297
DEG	-2.5245306	0.05789787	1	nonDEG	2.41668918	0.182684
nonDEG	-0.8637823	0.19367776	1	nonDEG	1.5182551	0.06509477
nonDEG	-0.038792	0.93234375	1	nonDEG	-0.528581	0.21317636
nonDEG	1.4869424	0.06519662	1	nonDEG	0.07841214	0.91482164
nonDEG	0.50050908	0.2642733	1	nonDEG	0.27193758	0.48620987
nonDEG	0.53199013	0.33490421	1	nonDEG	-0.2035607	0.69103779
nonDEG	-0.2947366	0.53961985	1	nonDEG	0.35195189	0.35808699
nonDEG	0.8097437	0.09483173	1	nonDEG	0.0684021	0.86839776
nonDEG	-1.2401114	0.11330267	1	nonDEG	0.16289719	0.77324215
nonDEG	-1.4990868	0.00123048	0.52498817	nonDEG	1.69602248	0.00616001
nonDEG	0.30888806	0.53918127	1	nonDEG	0.24490949	0.59198548
nonDEG	0.49669144	0.27375177	1	nonDEG	-0.3108856	0.40257182
nonDEG	0.26569203	0.54681538	1	nonDEG	0.12552815	0.72993298
nonDEG	-1.7410219	0.00824674	0.77656086	nonDEG	0.22425196	0.63750959
nonDEG	0.33092509	0.46456961	1	nonDEG	0.30055581	0.44528454
nonDEG	-0.2872448	0.51423762	1	nonDEG	0.08294174	0.82819707
nonDEG	0.87629418	0.04466975	1	nonDEG	-0.3833727	0.29329747
nonDEG	-0.7402608	0.13598739	1	nonDEG	0.7378507	0.07238404
nonDEG	0.40185533	0.35804866	1	nonDEG	-0.0934	0.79629761

nonDEG	-0.2593251	0.55248823	1	nonDEG	-0.3567701	0.33099091
nonDEG	2.10375174	0.00031842	0.35799251	nonDEG	0.00493957	0.9930641
nonDEG	1.27101201	0.03725302	1	nonDEG	-0.4295506	0.36338053
nonDEG	1.24033442	0.0083977	0.78074119	nonDEG	-0.3460556	0.39181118
nonDEG	0.32928158	0.44910921	1	nonDEG	-0.2462446	0.49952477
nonDEG	1.00433539	0.03958893	1	nonDEG	-0.9376017	0.01658569
nonDEG	-0.0940666	0.8518311	1	nonDEG	-0.2152748	0.65179742
nonDEG	-0.1448299	0.76344139	1	nonDEG	-0.3746521	0.39235269
nonDEG	-0.5290425	0.21608674	1	nonDEG	0.10019421	0.79433135
nonDEG	0.04895006	0.93329459	1	nonDEG	0.08619506	0.87502558
nonDEG	0.20212371	0.65417233	1	nonDEG	-0.0029498	0.99368091
nonDEG	1.62293566	0.05573747	1	nonDEG	0.40935398	0.57878619
nonDEG	-0.0176902	0.96734749	1	nonDEG	0.11220473	0.76094935
nonDEG	0.70336193	0.32630956	1	nonDEG	-1.073461	0.05678902
nonDEG	0.08882807	0.88481324	1	nonDEG	0.40827589	0.44901566
nonDEG	-1.1454152	0.10319925	1	nonDEG	2.04408171	0.01887684
nonDEG	-1.0974522	0.16721339	1	nonDEG	-0.1539892	0.78574828
nonDEG	0.95947805	0.13975824	1	nonDEG	-0.795532	0.11245228
nonDEG	-1.4758225	0.08142415	1	nonDEG	2.12131256	0.03144145
nonDEG	-0.9955157	0.14874868	1	nonDEG	1.91689281	0.01413364
nonDEG	0.04946766	0.97141571	1	nonDEG	0.18670396	0.83312772
nonDEG	-0.4031936	0.3412756	1	nonDEG	0.33302013	0.40311371
DEG	-1.9785031	0.05282225	1	nonDEG	1.715901	0.11072487
nonDEG	-0.2886887	0.6059022	1	nonDEG	-0.2725405	0.58833027
nonDEG	-0.2854886	0.5096847	1	nonDEG	-0.0016816	0.99721967
nonDEG	1.04290628	0.32314565	1	nonDEG	-0.5297228	0.68104569
nonDEG	-1.1348826	0.03206646	1	nonDEG	0.17095961	0.71023036
nonDEG	1.03385087	0.03882309	1	nonDEG	-1.0661232	0.01534317
nonDEG	1.06225162	0.06554455	1	nonDEG	0.12748955	0.80943275
nonDEG	0.56684956	0.35548025	1	nonDEG	-0.5234781	0.29989612
nonDEG	0.05896648	0.91347528	1	nonDEG	-0.366075	0.31458611
nonDEG	1.95086763	0.01053888	0.82576758	nonDEG	-0.4116107	0.6019984
nonDEG	0.8322969	0.06899346	1	nonDEG	-0.2624115	0.47563063
nonDEG	-0.7365437	0.2101337	1	nonDEG	0.78476072	0.18423735
nonDEG	0.75321527	0.13164291	1	nonDEG	-0.5244258	0.23658735
nonDEG	0.4497233	0.29933676	1	nonDEG	-0.0185995	0.95953376
nonDEG	0.35836151	0.46506163	1	nonDEG	-0.533716	0.1931827
nonDEG	-2.0374345	0.00355348	0.67010784	nonDEG	2.19950263	0.00380987
nonDEG	0.57432261	0.19338299	1	nonDEG	-0.3714273	0.32504955
nonDEG	-0.2128778	0.68689824	1	nonDEG	0.61730799	0.2482733
nonDEG	0.67582348	0.65393991	1	nonDEG	1.64817498	0.12099885
nonDEG	1.37347921	0.16175176	1	nonDEG	0.19164551	0.83125041
nonDEG	0.84215658	0.16497561	1	nonDEG	-0.1304362	0.81997607

nonDEG	1.71430047	0.0390427	1	nonDEG	-0.1204332	0.8760753
nonDEG	-0.87973	0.18749352	1	nonDEG	0.76446428	0.24403379
nonDEG	-0.3518484	0.44652612	1	nonDEG	-0.5893092	0.13900983
nonDEG	-0.6029804	0.19610635	1	nonDEG	-0.1304047	0.77712301
nonDEG	-0.1707375	0.69310209	1	nonDEG	-0.1983107	0.60085714
nonDEG	0.64518162	0.31695316	1	nonDEG	-0.1934983	0.74163021
nonDEG	-1.0650479	0.09770242	1	nonDEG	1.92651396	0.00335288
nonDEG	0.62454438	0.24158366	1	nonDEG	-0.4210137	0.32138385
nonDEG	-0.5526213	0.24519481	1	nonDEG	-0.1016322	0.78940627
nonDEG	1.16589899	0.03274716	1	nonDEG	-0.8692094	0.08935166
nonDEG	0.13800395	0.80257597	1	nonDEG	0.0237485	0.96713047
nonDEG	-0.2342188	0.60235983	1	nonDEG	0.4877051	0.18697095
nonDEG	-1.6140329	0.00569469	0.73793371	nonDEG	1.92884247	1.33E-05
nonDEG	0.41411219	0.35921946	1	nonDEG	-0.5600159	0.1497091
nonDEG	0.97265904	0.12085855	1	nonDEG	-0.8546096	0.1099631
nonDEG	0.5394329	0.32007772	1	nonDEG	0.02144892	0.96524325
nonDEG	-0.4635459	0.28209673	1	nonDEG	0.14520378	0.68998644
nonDEG	-0.727019	0.10792293	1	nonDEG	0.61874706	0.11655591
nonDEG	-0.6629833	0.35537553	1	nonDEG	1.29199554	0.06657729
nonDEG	-0.4826202	0.33913229	1	nonDEG	0.14942289	0.75201982
nonDEG	1.51504433	0.01218676	0.85229819	nonDEG	-0.051897	0.93008181
nonDEG	2.1043535	0.02892684	1	nonDEG	0.76103	0.371857
nonDEG	-1.0025107	0.04454453	1	nonDEG	1.32681633	0.01106888
nonDEG	-0.5154083	0.322628	1	nonDEG	0.73492376	0.12844284
nonDEG	1.35948243	0.15280881	1	nonDEG	-0.5635337	0.52845988
nonDEG	-0.6943171	0.183872	1	nonDEG	0.78377927	0.07485021
nonDEG	-0.9094638	0.07954652	1	nonDEG	0.16515151	0.74711989
nonDEG	0.74184158	0.23268995	1	nonDEG	0.35067999	0.47472961
nonDEG	-0.6672349	0.15617438	1	nonDEG	0.82117334	0.04665179
nonDEG	-0.7202951	0.19372065	1	nonDEG	-0.3108597	0.50266651
nonDEG	-0.5610027	0.32829325	1	nonDEG	1.01610892	0.07217414
nonDEG	-1.1770066	0.09289144	1	nonDEG	1.09434715	0.14358026
nonDEG	-1.185357	0.0454256	1	nonDEG	1.45929636	0.02648308
nonDEG	-1.0302491	0.23682168	1	nonDEG	0.72527827	0.50470872
nonDEG	-1.0670456	0.03403483	1	nonDEG	1.18144712	0.04402788
nonDEG	0.33836884	0.43166901	1	nonDEG	-0.3468561	0.33930084
nonDEG	-0.7020101	0.13579092	1	nonDEG	-0.1040256	0.79843398
nonDEG	0.03621344	0.94660363	1	nonDEG	0.1230479	0.8011269
nonDEG	-0.8595219	0.19997341	1	nonDEG	1.25734602	0.04512365
nonDEG	0.01850573	0.96830074	1	nonDEG	-0.3776063	0.33395241
nonDEG	-0.6504033	0.18436183	1	nonDEG	-0.055554	0.91223131
nonDEG	0.21287897	0.64051296	1	nonDEG	-0.2977134	0.47566148
nonDEG	-0.5461393	0.55095642	1	nonDEG	0.18988501	0.85516897

nonDEG	0.56067558	0.23670253	1	nonDEG	-0.2789598	0.4523725
nonDEG	1.12120671	0.07863758	1	nonDEG	-0.2597498	0.67068938
nonDEG	-0.7576396	0.13620641	1	nonDEG	1.10768018	0.00266783
nonDEG	-1.2173517	0.03553028	1	nonDEG	0.29525866	0.50416104
nonDEG	-3.3046251	0.00208682	0.61198441	nonDEG	2.57224672	0.00017246
nonDEG	-0.0106156	0.98352284	1	nonDEG	0.1976232	0.6497829
nonDEG	-0.2286448	0.63225585	1	nonDEG	-0.2722218	0.51895561
nonDEG	-0.3282647	0.43801349	1	nonDEG	0.1435489	0.69799117
nonDEG	0.34623355	0.46835855	1	nonDEG	-0.4656869	0.28688717
nonDEG	-0.0762937	0.85622412	1	nonDEG	0.07667053	0.8383513
nonDEG	0.62069629	0.53239178	1	nonDEG	-1.239476	0.01890436
nonDEG	0.14301655	0.74828417	1	nonDEG	-0.016326	0.96428902
nonDEG	-0.2432163	0.62942834	1	nonDEG	-0.0897155	0.83551526
nonDEG	-0.3579432	0.40177291	1	nonDEG	0.22784955	0.52489534
nonDEG	0.38567564	0.41103702	1	nonDEG	0.09678483	0.78726359
nonDEG	0.45770597	0.57804237	1	nonDEG	0.11742177	0.88153604
nonDEG	-0.8774763	0.32394267	1	nonDEG	1.19748654	0.24974723
nonDEG	-0.2563453	0.58514838	1	nonDEG	0.17080684	0.69411018
nonDEG	-0.0516746	0.90292407	1	nonDEG	-0.191675	0.59746134
nonDEG	-0.7810565	0.16734265	1	nonDEG	-1.2926641	0.04747985
nonDEG	-0.4858646	0.6034692	1	nonDEG	0.05730004	0.95219037
nonDEG	-0.4017058	0.34544561	1	nonDEG	-0.5750518	0.11278857
nonDEG	0.60432319	0.33508889	1	nonDEG	0.09301328	0.86791098
nonDEG	-0.6820347	0.17060031	1	nonDEG	0.26218568	0.59182212
nonDEG	1.39054252	0.04065447	1	nonDEG	-0.4371423	0.44857648
nonDEG	0.87918385	0.3946868	1	nonDEG	0.82450154	0.36823102
nonDEG	-0.3389185	0.46242891	1	nonDEG	-0.3684594	0.38076956
nonDEG	-0.2690536	0.74723294	1	nonDEG	-0.0194747	0.99076973
nonDEG	0.36366614	0.52073444	1	nonDEG	-0.0457167	0.92233526
nonDEG	-1.1251095	0.10336256	1	nonDEG	1.55044237	0.00312566
nonDEG	1.37904296	0.03323345	1	nonDEG	0.38344056	0.4992832
nonDEG	-0.4107903	0.47401163	1	nonDEG	0.83207015	0.10221507
nonDEG	0.1981164	0.76694385	1	nonDEG	0.17997857	0.72083786
nonDEG	0.6227093	0.21421659	1	nonDEG	-0.6879866	0.06885847
nonDEG	0.49134714	0.2952883	1	nonDEG	-0.2932493	0.42192256
nonDEG	-0.0532931	0.90336815	1	nonDEG	-0.0610114	0.8700631
nonDEG	-0.3596572	0.40484807	1	nonDEG	0.01201924	0.97522286
nonDEG	0.42358692	0.43873874	1	nonDEG	-0.4100847	0.35726768
nonDEG	-2.749296	0.00236729	0.61776954	nonDEG	2.22390876	0.00970466
nonDEG	0.25418877	0.6157546	1	nonDEG	0.01360852	0.97049671
nonDEG	0.68640785	0.15088433	1	nonDEG	-0.5861475	0.20195089
nonDEG	0.19879932	0.65890535	1	nonDEG	0.31671701	0.39669067
nonDEG	0.15303542	0.71930287	1	nonDEG	-0.0284459	0.93816282

nonDEG	0.58746371	0.24601043	1	nonDEG	-0.0080054	0.98775819
nonDEG	3.12963265	0.00727003	0.75263895	nonDEG	-2.3819863	0.03059036
nonDEG	0.79604244	0.14784867	1	nonDEG	-0.9703027	0.06507503
nonDEG	-0.4116621	0.38386862	1	nonDEG	-0.2656026	0.52802873
nonDEG	-0.588973	0.22928652	1	nonDEG	0.7330887	0.09860915
nonDEG	-1.9800595	0.13089733	1	nonDEG	1.32587615	0.37217201
nonDEG	-5.6941933	0.03366378	1	nonDEG	3.05440224	0.69968085
nonDEG	4.74769656	0.24066683	1	nonDEG	0.53858342	0.90333586
nonDEG	1.40975719	0.00528396	0.72753356	nonDEG	-0.1682825	0.6768581
nonDEG	-0.8456653	0.15258337	1	nonDEG	0.01462715	0.98063943
nonDEG	-0.4115769	0.41080868	1	nonDEG	-0.185217	0.67763137
nonDEG	0.10665045	0.80134484	1	nonDEG	-0.3125969	0.40735682
nonDEG	-0.5393654	0.42500366	1	nonDEG	0.7166463	0.22735384
nonDEG	1.24671888	0.03494499	1	nonDEG	0.51885883	0.33602381
nonDEG	0.5482391	0.20720255	1	nonDEG	-0.0127582	0.97337346
nonDEG	0.18144527	0.82132019	1	nonDEG	0.08654512	0.91511976
nonDEG	-0.5934601	0.2939843	1	nonDEG	0.79885112	0.07628183
nonDEG	-1.009258	0.04844744	1	nonDEG	0.90342173	0.02921693
nonDEG	0.40042571	0.41365594	1	nonDEG	-0.0505839	0.89698006
nonDEG	0.87297612	0.23109199	1	nonDEG	0.11485949	0.84472943
nonDEG	-0.4107772	0.35540067	1	nonDEG	-0.0854752	0.83820127
nonDEG	-0.7371192	0.35200386	1	nonDEG	1.81301566	0.01825198
nonDEG	1.38681248	0.43323223	1	nonDEG	-0.7807686	0.64994494
nonDEG	0.58465384	0.24201707	1	nonDEG	-0.3940021	0.42661825
nonDEG	-0.5409292	0.31361548	1	nonDEG	0.16379437	0.74486466
nonDEG	-0.0264898	0.95605321	1	nonDEG	0.17123488	0.68478125
nonDEG	0.9023503	0.05315965	1	nonDEG	-0.260103	0.47368607
nonDEG	0.84714987	0.40012002	1	nonDEG	0.24806573	0.81771548
nonDEG	0.35370952	0.48934639	1	nonDEG	-0.1820972	0.70338371
nonDEG	1.24109631	0.05945921	1	nonDEG	-0.7593384	0.22201084
nonDEG	2.23300104	0.02786808	1	nonDEG	-0.9480668	0.32087468
nonDEG	-0.8953544	0.11602072	1	nonDEG	0.69255523	0.26214708
nonDEG	0.21738665	0.69755657	1	nonDEG	-0.0728118	0.86505298
nonDEG	1.69179694	0.00546372	0.72967447	nonDEG	-0.3564017	0.38942181
nonDEG	-0.0007147	1	1	nonDEG	0.17198733	0.77829018
nonDEG	0.35917535	0.47290979	1	nonDEG	-0.5843244	0.15214151
nonDEG	-0.5638295	0.5191364	1	nonDEG	1.7163471	0.00939816
nonDEG	1.2240851	0.02305157	1	nonDEG	-0.3915684	0.43521413
nonDEG	0.84288234	0.636942	1	nonDEG	0.43833726	0.78443745
nonDEG	0.44250798	0.40540066	1	nonDEG	0.06773935	0.86766265
nonDEG	-0.4426439	0.33360034	1	nonDEG	0.35169843	0.34367086
nonDEG	-1.3415508	0.04006835	1	nonDEG	0.79407828	0.15383619
nonDEG	1.20245947	0.04574929	1	nonDEG	-1.7613975	0.00715667

nonDEG	-0.0165014	0.97037196	1	nonDEG	-0.2106441	0.58552751
DEG	0.88781172	0.06259993	1	nonDEG	0.63428748	0.10400201
nonDEG	2.07845152	0.03236935	1	nonDEG	-1.2687891	0.162708
nonDEG	-0.8157417	0.09803232	1	nonDEG	1.63092791	0.00554152
nonDEG	4.33102442	0.01224534	0.85229819	nonDEG	-2.4274151	0.16131489
nonDEG	0.77644845	0.12208287	1	nonDEG	-0.5655746	0.22497781
nonDEG	-0.350477	0.43312287	1	nonDEG	0.22645746	0.5662621
nonDEG	0.14302837	0.75371032	1	nonDEG	0.11590356	0.75375317
nonDEG	1.59466553	0.10324405	1	nonDEG	-0.2652274	0.79113951
nonDEG	-1.1771282	0.07419537	1	nonDEG	2.09647644	0.00115188
nonDEG	0.23376125	0.65128722	1	nonDEG	-0.4557905	0.27808248
nonDEG	1.19195817	0.06547882	1	nonDEG	-0.3895024	0.46164087
nonDEG	-1.1260398	0.03359328	1	nonDEG	1.1026194	0.08688193
nonDEG	-0.7087835	0.17929935	1	nonDEG	-0.5953569	0.35403212
nonDEG	-2.1051821	0.01070203	0.83005619	nonDEG	-0.7864045	0.29819342
nonDEG	-0.3275507	0.55067546	1	nonDEG	-0.0973295	0.85812107
nonDEG	0.29062352	0.72101685	1	nonDEG	-0.2834214	0.74171966
nonDEG	1.00008395	0.05810467	1	nonDEG	-0.5974788	0.09709209
nonDEG	-0.2133192	0.63659006	1	nonDEG	-0.4865193	0.22565306
nonDEG	-3.0148029	0.00323189	0.65616368	nonDEG	1.1010285	0.26550201
nonDEG	1.307752	0.01506013	0.89340262	nonDEG	-0.6080236	0.09775416
nonDEG	-0.1472729	0.86084422	1	nonDEG	1.20394402	0.09023303
nonDEG	-1.539362	0.15054982	1	nonDEG	1.94248312	0.1059944
nonDEG	-0.4517718	0.29685403	1	nonDEG	0.53286196	0.14416154
nonDEG	0.06163673	0.88734503	1	nonDEG	-0.0679881	0.85863309
nonDEG	-1.0525102	0.03433681	1	nonDEG	0.96910571	0.01136213
nonDEG	-1.2599264	0.02050395	0.99114571	nonDEG	0.4818228	0.23986402
nonDEG	-0.1973541	0.64136194	1	nonDEG	-0.1023871	0.77600003
nonDEG	0.61776418	0.16475464	1	nonDEG	-1.481921	0.00019688
nonDEG	-0.9841375	0.25679141	1	nonDEG	1.03704842	0.18899985
nonDEG	-1.6124831	0.17515622	1	nonDEG	2.14441795	0.08176928
nonDEG	-1.4564958	0.09187372	1	nonDEG	1.55350274	0.05838204
nonDEG	-1.6137393	0.14253005	1	nonDEG	1.28235462	0.27716251
nonDEG	0.38985143	0.41478835	1	nonDEG	0.21246315	0.57372866
nonDEG	0.72937125	0.23183717	1	nonDEG	-0.5311994	0.33824098
nonDEG	-1.7226968	0.00397386	0.68407511	nonDEG	1.72141325	0.02478682
nonDEG	0.74622869	0.43808615	1	nonDEG	-0.725834	0.44195685
nonDEG	0.55863617	0.45963064	1	nonDEG	-0.344198	0.5314213
nonDEG	-1.3476781	0.02697525	1	nonDEG	0.58514962	0.37451265
nonDEG	0.0428902	0.92330898	1	nonDEG	-0.0471557	0.90041473
nonDEG	0.71435148	0.13964373	1	nonDEG	-0.0796097	0.83493925
nonDEG	0.79830416	0.09469501	1	nonDEG	-0.0718709	0.84371037
nonDEG	0.13354696	0.85879992	1	nonDEG	-0.4205396	0.62136835

nonDEG	0.38098014	0.37243878	1	nonDEG	-0.317313	0.38288435
nonDEG	0.22795799	0.59456696	1	nonDEG	0.11261243	0.75723422
nonDEG	0.53193929	0.48217598	1	nonDEG	-0.7091916	0.17597263
nonDEG	-1.1926206	0.20011629	1	nonDEG	0.75962141	0.45778564
nonDEG	0.72151698	0.34973263	1	nonDEG	-0.1089351	0.88609523
nonDEG	2.20891098	0.00029262	0.34458183	nonDEG	-1.352179	0.01795034
nonDEG	-0.5886531	0.18771518	1	nonDEG	0.21229969	0.55372776
nonDEG	-0.5766452	0.27427625	1	nonDEG	-0.2167977	0.69514306
nonDEG	-1.1306521	0.17632865	1	nonDEG	1.57454934	0.07217925
nonDEG	0.02957417	0.9499536	1	nonDEG	-0.3153948	0.45534782
nonDEG	0.27769696	0.53639151	1	nonDEG	-0.7041298	0.06853142
nonDEG	-0.219484	0.62663318	1	nonDEG	-0.4162098	0.29323532
nonDEG	-1.0888163	0.02814536	1	nonDEG	0.36469113	0.37260638
nonDEG	0.34860544	0.61089461	1	nonDEG	0.30676891	0.66019312
nonDEG	-0.3832941	0.48711014	1	nonDEG	-0.0009972	0.99980851
nonDEG	-0.2784631	0.52222246	1	nonDEG	-0.172915	0.64156335
nonDEG	0.80488979	0.26011322	1	nonDEG	0.35448426	0.58603607
nonDEG	1.01987502	0.07345283	1	nonDEG	0.2214099	0.59735116
nonDEG	0.39591026	0.43788634	1	nonDEG	-0.428983	0.33401663
nonDEG	-0.5628804	0.21906333	1	nonDEG	0.17086554	0.72489971
nonDEG	0.48858621	0.27422039	1	nonDEG	-0.6549747	0.10027856
nonDEG	0.64284618	0.21324316	1	nonDEG	-0.0939518	0.8423633
nonDEG	-1.3407361	0.02933877	1	nonDEG	-0.4473381	0.52383505
nonDEG	0.74546019	0.19407333	1	nonDEG	-0.5838197	0.16951335
nonDEG	0.83722234	0.44646239	1	nonDEG	0.14712739	0.92015517
nonDEG	1.21240247	0.23085949	1	nonDEG	-0.7688377	0.47175238
nonDEG	-1.1400967	0.09129607	1	nonDEG	0.46213079	0.53610646
nonDEG	-0.5470857	0.21432397	1	nonDEG	0.21644298	0.55343878
nonDEG	0.8592274	0.18386578	1	nonDEG	-0.0802923	0.8726455
nonDEG	-0.943829	0.05843841	1	nonDEG	0.13615898	0.72027019
nonDEG	-1.5181031	0.01605669	0.91466279	nonDEG	0.88321309	0.06294943
nonDEG	0.15689586	0.90889788	1	nonDEG	1.10623455	0.26645633
nonDEG	0.32739423	0.45903724	1	nonDEG	0.21279376	0.56763751
nonDEG	-0.5020092	0.73636815	1	nonDEG	-0.0750982	1
nonDEG	2.07485362	0.02463978	1	nonDEG	-0.4640313	0.50995146
nonDEG	-0.8617466	0.07185263	1	nonDEG	0.59588456	0.14389454
nonDEG	-0.4846766	0.46265592	1	nonDEG	-0.5541833	0.26081396
nonDEG	0.37558701	0.43183929	1	nonDEG	-0.237885	0.53099853
nonDEG	1.52624868	0.12127987	1	nonDEG	-0.4454746	0.66095127
nonDEG	-1.6298966	0.01353194	0.87457296	nonDEG	1.1453811	0.01839742
nonDEG	1.12446752	0.07578643	1	nonDEG	-0.702376	0.22545666
nonDEG	0.14036896	0.83475157	1	nonDEG	-0.3940347	0.56268519
nonDEG	-0.5569715	0.19908252	1	nonDEG	0.16823644	0.65522874

nonDEG	0.08985011	0.84833734	1	nonDEG	-0.306293	0.39786325
nonDEG	0.15148542	0.74077878	1	nonDEG	-0.098215	0.79567318
nonDEG	-1.5709083	0.04662299	1	nonDEG	0.73146276	0.22833749
nonDEG	0.91106004	0.03351943	1	nonDEG	-0.31487	0.383883
nonDEG	-0.6180748	0.22337527	1	nonDEG	0.35856091	0.43948908
nonDEG	1.00022611	0.06320468	1	nonDEG	-0.4153842	0.28220584
nonDEG	-1.1851573	0.0530794	1	nonDEG	1.06508163	0.04295768
nonDEG	0.29417474	0.60785063	1	nonDEG	-0.136075	0.81269596
nonDEG	-0.6790437	0.35393111	1	nonDEG	1.44811154	0.07376644
nonDEG	0.84257776	0.15141738	1	nonDEG	-0.6565689	0.23919484
nonDEG	0.64627611	0.17113983	1	nonDEG	-0.2787231	0.44945016
nonDEG	0.81167332	0.13581679	1	nonDEG	-0.152462	0.71191859
nonDEG	-0.0584269	0.89361322	1	nonDEG	-0.0449746	0.90313882
nonDEG	0.04038497	0.92870947	1	nonDEG	-0.4315978	0.27604852
nonDEG	-0.4704204	0.41745545	1	nonDEG	0.85140856	0.18908859
nonDEG	-0.1216595	0.7880719	1	nonDEG	-0.332179	0.39784204
nonDEG	1.0730469	0.1404745	1	nonDEG	-0.8280077	0.14795664
nonDEG	0.86969546	0.1135706	1	nonDEG	-0.5705108	0.1813784
nonDEG	-1.7829871	0.00983251	0.80027302	nonDEG	1.42002128	0.02632131
nonDEG	1.24218918	0.24239031	1	nonDEG	-0.6654825	0.17352088
nonDEG	-1.0101283	0.10648132	1	nonDEG	0.88002231	0.16827791
nonDEG	-1.8613937	0.06983637	1	nonDEG	2.05905206	0.05917424
nonDEG	0.25672911	0.65898379	1	nonDEG	0.00091284	1
nonDEG	-0.5939999	0.68433257	1	nonDEG	1.6118901	0.20108121
nonDEG	-0.5587963	0.20493943	1	nonDEG	0.07481781	0.84755925
nonDEG	-0.8782928	0.05367145	1	nonDEG	0.31915739	0.38544654
nonDEG	-0.7017316	0.2802987	1	nonDEG	1.10216139	0.07445551
nonDEG	0.20406137	0.76016113	1	nonDEG	-0.3057347	0.60356227
nonDEG	-0.7761652	0.22672815	1	nonDEG	0.78056277	0.22055955
nonDEG	-0.238402	0.57786944	1	nonDEG	0.14543692	0.68801834
nonDEG	2.01348626	0.17099621	1	nonDEG	-2.4228177	0.0973509
nonDEG	0.62815504	0.1783162	1	nonDEG	-0.2999492	0.40516066
nonDEG	-0.3594982	0.43092717	1	nonDEG	-0.2535759	0.53568283
nonDEG	-0.4189603	0.42723689	1	nonDEG	0.06093002	0.89591416
nonDEG	-0.4779751	0.30007098	1	nonDEG	0.47836223	0.22153293
nonDEG	0.26352602	0.56110724	1	nonDEG	-0.2393952	0.51163298
nonDEG	0.96982847	0.06840703	1	nonDEG	-0.294637	0.55050934
nonDEG	-0.8731959	0.07295001	1	nonDEG	0.97780545	0.04316661
nonDEG	-0.5050388	0.25926514	1	nonDEG	0.02236575	0.95350193
nonDEG	2.43945024	0.00210166	0.61198441	nonDEG	-1.6811681	0.01766622
nonDEG	-0.3220613	0.50592036	1	nonDEG	-0.1195868	0.77152116
nonDEG	1.17581528	0.13434166	1	nonDEG	-0.0774823	0.92852663
nonDEG	0.18919556	0.68667266	1	nonDEG	-0.187372	0.62466201

nonDEG	-0.3816017	0.36947592	1	nonDEG	0.35845706	0.3249964
nonDEG	0.20077777	0.6498456	1	nonDEG	0.35469604	0.35517841
nonDEG	-0.7013568	0.16028875	1	nonDEG	0.39589858	0.40310052
nonDEG	-0.3015195	0.48617146	1	nonDEG	0.09716246	0.79270619
nonDEG	0.93999615	0.06450178	1	nonDEG	-0.1693082	0.69003937
nonDEG	-0.7108367	0.16691726	1	nonDEG	0.04934421	0.91360638
nonDEG	0.5933497	0.50189443	1	nonDEG	-0.7677103	0.39560064
nonDEG	-0.5283953	0.21844998	1	nonDEG	0.10966942	0.76889336
nonDEG	2.2880789	0.00035432	0.37253089	nonDEG	-0.8696015	0.05201488
nonDEG	0.06681669	0.9208443	1	nonDEG	0.43797461	0.46204767
nonDEG	-0.4454745	0.44058164	1	nonDEG	0.69023769	0.16229507
nonDEG	-0.5038613	0.48542309	1	nonDEG	-0.1853424	0.81015064
nonDEG	0.53321085	0.20735321	1	nonDEG	0.2206302	0.54127757
nonDEG	1.42010976	0.02569837	1	nonDEG	-0.5474647	0.29024217
nonDEG	0.05548794	0.89459467	1	nonDEG	0.11458743	0.75949712
nonDEG	-0.3423803	0.43617102	1	nonDEG	0.04517144	0.90995315
nonDEG	-0.4091819	0.42247802	1	nonDEG	0.24318206	0.58692147
nonDEG	0.21506047	0.65803091	1	nonDEG	-0.3042531	0.47562325
nonDEG	-0.1256365	0.77099704	1	nonDEG	-0.4912667	0.21417539
nonDEG	0.01659837	0.97238143	1	nonDEG	-0.1628454	0.70383998
nonDEG	-1.5074617	0.01192844	0.85229819	nonDEG	0.73743458	0.14900538
nonDEG	0.94685695	0.0579017	1	nonDEG	-1.2037467	0.00770989
nonDEG	-0.0850759	0.85188832	1	nonDEG	0.21701019	0.63660066
nonDEG	-1.2590839	0.01173554	0.85229819	nonDEG	2.16274109	0.00072016
nonDEG	-0.778071	0.0983995	1	nonDEG	0.09870059	0.80660424
nonDEG	-1.1023023	0.07539457	1	nonDEG	1.09207062	0.07051227
nonDEG	0.44529098	0.49189734	1	nonDEG	-0.6549432	0.15839011
nonDEG	0.66926953	0.11346974	1	nonDEG	-0.3975135	0.27257384
nonDEG	-0.1038305	0.81317488	1	nonDEG	0.11899145	0.76030141
nonDEG	0.42030517	0.44644663	1	nonDEG	-0.3534541	0.49693182
nonDEG	0.72979508	0.35033209	1	nonDEG	-0.9215215	0.11749927
nonDEG	0.92906071	0.07787224	1	nonDEG	-0.1679168	0.66855281
nonDEG	0.53324674	0.26876487	1	nonDEG	-0.2230727	0.56741917
nonDEG	-0.4956023	0.2779184	1	nonDEG	0.20276268	0.61458729
nonDEG	0.0898397	0.83451732	1	nonDEG	0.19196816	0.6035037
nonDEG	-0.8451407	0.13300189	1	nonDEG	0.81858722	0.14836171
nonDEG	-0.6278941	0.18782226	1	nonDEG	0.11176654	0.81748748
nonDEG	-0.612147	0.15765046	1	nonDEG	0.00585544	0.98895209
nonDEG	-1.8701811	0.00150778	0.55484247	nonDEG	2.18944835	6.89E-05
nonDEG	-1.8091546	0.09891848	1	nonDEG	2.26511467	0.05235394
nonDEG	-0.6075236	0.37829634	1	nonDEG	0.34089363	0.61388714
nonDEG	-0.5238972	0.27541718	1	nonDEG	0.1079817	0.78897599
nonDEG	-0.5794858	0.23766403	1	nonDEG	0.26913605	0.50324793

nonDEG	0.64147218	0.14225945	1	nonDEG	-0.4021558	0.28030991
nonDEG	0.39876234	0.38802436	1	nonDEG	0.18811026	0.66761717
nonDEG	-0.2119527	0.62812618	1	nonDEG	-0.3357426	0.37103588
nonDEG	0.73405045	0.19089584	1	nonDEG	-0.4226794	0.37880987
nonDEG	0.46338138	0.33703374	1	nonDEG	-0.1603808	0.6604745
nonDEG	0.24359373	0.58512309	1	nonDEG	-0.4381595	0.23949499
nonDEG	0.01654141	0.96982695	1	nonDEG	0.30756493	0.40103319
nonDEG	0.88079777	0.21455419	1	nonDEG	-0.699666	0.14995159
nonDEG	0.24046707	0.63097684	1	nonDEG	-0.040762	0.93211234
nonDEG	0.0285201	0.94805473	1	nonDEG	0.22828207	0.53337981
nonDEG	0.40790339	0.35511569	1	nonDEG	-0.2012483	0.57708955
nonDEG	-0.4958592	0.26731273	1	nonDEG	0.19400317	0.60249411
nonDEG	0.74470999	0.11144327	1	nonDEG	-0.1766551	0.63010932
nonDEG	0.48945951	0.25063152	1	nonDEG	0.44433388	0.23429673
nonDEG	1.02822982	0.11444733	1	nonDEG	-0.6366958	0.26390325
nonDEG	0.27545781	0.62660506	1	nonDEG	-0.2496425	0.63252493
nonDEG	-0.1779667	0.68495768	1	nonDEG	-0.6562878	0.07899559
nonDEG	-1.090617	0.15852971	1	nonDEG	0.54641967	0.53680767
nonDEG	0.90410292	0.12458926	1	nonDEG	-0.4688584	0.32285585
nonDEG	0.66349601	0.22444344	1	nonDEG	0.00999091	0.98624479
nonDEG	0.28289796	0.54310842	1	nonDEG	0.10376539	0.78126885
nonDEG	-0.7108551	0.16066274	1	nonDEG	0.38238307	0.37253732
nonDEG	-0.3457772	0.4637425	1	nonDEG	-0.2586827	0.49880961
nonDEG	-0.8877908	0.08455142	1	nonDEG	1.08692194	0.00345856
nonDEG	-0.4778306	0.2817375	1	nonDEG	0.14177372	0.7106749
nonDEG	0.44283002	0.3253149	1	nonDEG	0.14272525	0.71277767
nonDEG	0.54945258	0.32082746	1	nonDEG	-0.1477386	0.77374531
nonDEG	-1.0660352	0.02910424	1	nonDEG	1.21294289	0.04210421
nonDEG	-0.9066493	0.07293682	1	nonDEG	0.70803405	0.23039465
nonDEG	2.13422696	6.46E-05	0.14400617	nonDEG	-0.7620112	0.08035396
nonDEG	1.32458203	0.02656704	1	nonDEG	0.32574356	0.50848128
nonDEG	-0.7124524	0.11168658	1	nonDEG	0.48706572	0.18091165
nonDEG	-0.3757084	0.67672144	1	nonDEG	0.31735928	0.73949884
nonDEG	1.24013098	0.01983773	0.97987069	nonDEG	-0.8061665	0.02692012
nonDEG	0.57956057	0.19234253	1	nonDEG	-0.1528324	0.68609768
nonDEG	0.09001543	0.84369951	1	nonDEG	0.23907969	0.51124635
nonDEG	-0.2558119	0.56354093	1	nonDEG	0.12624553	0.73274637
nonDEG	-0.6041915	0.19115622	1	nonDEG	0.22235411	0.57865994
nonDEG	0.48713358	0.35076694	1	nonDEG	-0.3904123	0.36028307
nonDEG	0.58562293	0.4360266	1	nonDEG	-0.6263887	0.33101457
nonDEG	0.97505001	0.02213483	1	nonDEG	-0.5056818	0.16085497
nonDEG	-0.3979585	0.35442022	1	nonDEG	0.11706064	0.7539917
nonDEG	-0.4084068	0.36909097	1	nonDEG	0.04780486	0.90972923

nonDEG	0.2081112	0.66091307	1	nonDEG	-0.4446675	0.24138679
nonDEG	-0.177732	0.70720833	1	nonDEG	-0.2907588	0.5132128
DEG	2.04418432	0.00790352	0.75788792	nonDEG	0.25877453	0.69449979
nonDEG	-0.4261338	0.31232482	1	nonDEG	0.41739232	0.25114658
nonDEG	0.4995374	0.26505711	1	nonDEG	0.24903529	0.5239412
nonDEG	-0.4541834	0.29661373	1	nonDEG	-0.0053847	0.98855617
nonDEG	0.67829157	0.14758805	1	nonDEG	-0.3612894	0.34418423
nonDEG	-1.2457206	0.01431346	0.88752292	nonDEG	0.09834411	0.84489735
nonDEG	-0.6516181	0.25192502	1	nonDEG	-0.233954	0.62242132
nonDEG	0.43072432	0.36934661	1	nonDEG	0.11248318	0.76693523
nonDEG	-0.2484068	0.58315426	1	nonDEG	0.4439439	0.25255315
nonDEG	-0.5449395	0.22539734	1	nonDEG	0.4110819	0.28655388
nonDEG	0.62733199	0.29238028	1	nonDEG	-0.1510752	0.7857777
nonDEG	0.21454661	0.72468584	1	nonDEG	-0.0416875	0.94892968
nonDEG	-0.4441281	0.32372079	1	nonDEG	0.1555863	0.70634457
nonDEG	-0.644852	0.29782381	1	nonDEG	0.29935677	0.59287582
nonDEG	1.24839254	0.01255306	0.85364221	nonDEG	-0.90147	0.01534744
nonDEG	-1.070931	0.01656087	0.91951594	nonDEG	1.00832903	0.03868282
nonDEG	0.9620474	0.15247401	1	nonDEG	-0.1695763	0.75112839
nonDEG	0.36897693	0.60252067	1	nonDEG	-0.7240481	0.40593941
nonDEG	1.6332022	0.11040855	1	nonDEG	0.07906972	0.93730729
nonDEG	0.69022429	0.26470319	1	nonDEG	-0.448872	0.41562052
nonDEG	1.5490354	0.24553894	1	nonDEG	0.37718126	0.7693854
nonDEG	1.21301998	0.04273429	1	nonDEG	-0.3987128	0.46039189
nonDEG	-0.1868961	0.68005632	1	nonDEG	0.39072763	0.37216103
nonDEG	-1.3516859	0.0031925	0.65616368	nonDEG	-0.405014	0.27497371
nonDEG	-0.4798714	0.27494729	1	nonDEG	0.05771363	0.87436687
nonDEG	-0.1992269	0.64181597	1	nonDEG	-0.248317	0.49368917
nonDEG	0.10306332	0.88952496	1	nonDEG	0.66851888	0.31321168
nonDEG	0.28736767	0.5217031	1	nonDEG	-0.0286704	0.94304048
nonDEG	-0.1990856	0.65328038	1	nonDEG	-0.5743501	0.1459443
nonDEG	-0.1990212	0.63482004	1	nonDEG	-0.2119679	0.55551813
nonDEG	-0.371308	0.50026357	1	nonDEG	0.05495763	0.92330134
nonDEG	-1.3551037	0.25218275	1	nonDEG	-0.4189397	0.7683893
nonDEG	-0.5586298	0.22762304	1	nonDEG	0.20804665	0.57220265
nonDEG	0.88025795	0.45075066	1	nonDEG	-1.7495716	0.1434941
nonDEG	-0.4894038	0.71751088	1	nonDEG	1.06780221	0.43031411
nonDEG	1.38513877	0.24056919	1	nonDEG	0.06816521	0.965824
nonDEG	0.50920849	0.23687985	1	nonDEG	-0.4503307	0.22467417
nonDEG	-0.2798876	0.52288316	1	nonDEG	-0.1338545	0.73770583
nonDEG	1.41146368	0.04556992	1	nonDEG	-1.0993142	0.12400807
nonDEG	0.77369702	0.12281811	1	nonDEG	-0.2985927	0.50259969
nonDEG	0.45602392	0.32920168	1	nonDEG	0.00140182	0.99706199

nonDEG	-0.676223	0.11711709	1	nonDEG	0.51941719	0.15268805
nonDEG	-0.451903	0.39604825	1	nonDEG	-0.171034	0.69445995
nonDEG	0.92679462	0.10162031	1	nonDEG	-0.2252836	0.66571418
nonDEG	-0.2287369	0.69243825	1	nonDEG	0.54232324	0.32679453
nonDEG	-0.4481768	0.33554457	1	nonDEG	0.53311461	0.22482299
nonDEG	0.30426214	0.56092469	1	nonDEG	-0.053619	0.9032915
nonDEG	0.09551276	0.87964712	1	nonDEG	-0.6386684	0.30749558
nonDEG	-0.6372349	0.17123458	1	nonDEG	0.51990091	0.21184521
nonDEG	-1.8828673	0.04743503	1	nonDEG	2.17688319	0.03582224
nonDEG	-1.8653333	0.00748465	0.75263895	nonDEG	2.40986451	0.00188897
nonDEG	-0.9053332	0.06472922	1	nonDEG	0.20917899	0.61161471
nonDEG	0.4543878	0.41593102	1	nonDEG	-0.2626958	0.59880107
nonDEG	-0.246968	0.56376141	1	nonDEG	-0.1167364	0.75025213
nonDEG	2.12536119	0.02583898	1	nonDEG	-0.5691442	0.43918451
nonDEG	-0.3909747	0.47976175	1	nonDEG	0.58913881	0.26535715
nonDEG	-0.6943557	0.160306	1	nonDEG	0.28942353	0.45523134
nonDEG	-0.2728116	0.59466071	1	nonDEG	0.6866256	0.08047548
nonDEG	0.2791195	0.64689895	1	nonDEG	-0.3463756	0.53823171
nonDEG	0.14115747	0.77056346	1	nonDEG	-0.3568923	0.34318234
nonDEG	-1.4417128	0.03193452	1	nonDEG	2.28985487	0.00214822
nonDEG	0.83111403	0.1382895	1	nonDEG	-0.311209	0.50427473
nonDEG	0.98660217	0.06224365	1	nonDEG	-0.4627192	0.26629175
nonDEG	-0.5918802	0.20217228	1	nonDEG	0.18370276	0.67014013
nonDEG	0.47433142	0.40661997	1	nonDEG	-0.4424231	0.42564453
nonDEG	-0.0681564	0.87923239	1	nonDEG	-0.5677634	0.13660611
nonDEG	0.07260552	0.90892494	1	nonDEG	-0.6925501	0.20335485
nonDEG	-0.4866931	0.31159595	1	nonDEG	-0.4620414	0.2789081
nonDEG	-0.0197902	0.96716953	1	nonDEG	0.09458167	0.82088638
nonDEG	1.02462819	0.01775555	0.9394793	nonDEG	-0.1404858	0.70530553
nonDEG	-0.6785459	0.14165343	1	nonDEG	-0.1837917	0.68269664
nonDEG	-0.3628294	0.58859452	1	nonDEG	-0.3336673	0.63462118
nonDEG	-0.134794	0.75829327	1	nonDEG	-0.1768618	0.642327
nonDEG	1.95256138	0.00696523	0.75126991	nonDEG	-0.5872548	0.35487415
nonDEG	3.05487282	0.02993618	1	nonDEG	-0.7147782	0.62074083
nonDEG	-0.2854741	0.5102964	1	nonDEG	0.09543046	0.82242662
nonDEG	1.07791466	0.09772438	1	nonDEG	-0.5404747	0.23104345
nonDEG	-0.2180582	0.6161418	1	nonDEG	-0.7718551	0.03719001
nonDEG	-1.0113424	0.24862079	1	nonDEG	0.5116554	0.57987458
nonDEG	2.3715436	0.09795512	1	nonDEG	0.47595875	0.73578441
nonDEG	0.26515174	0.6969602	1	nonDEG	-0.6157727	0.3178296
nonDEG	-0.3527505	0.41063317	1	nonDEG	0.19433586	0.59681554
nonDEG	0.60943092	0.21202759	1	nonDEG	0.69636998	0.09512391
nonDEG	-0.4487309	0.38103141	1	nonDEG	0.54620877	0.17560883

nonDEG	-0.0762267	0.86646897	1	nonDEG	-0.1784885	0.6559126
nonDEG	0.18035858	0.71453595	1	nonDEG	-0.3357322	0.41843067
nonDEG	-0.1086037	0.79707089	1	nonDEG	-0.5449471	0.15051898
nonDEG	-0.5517646	0.21871791	1	nonDEG	0.20080177	0.58839166
nonDEG	-1.9944692	0.02262098	1	nonDEG	1.53593211	0.0062518
nonDEG	-1.9841355	0.06173585	1	nonDEG	2.02440339	0.09745626
nonDEG	0.09212445	0.84256031	1	nonDEG	0.12662825	0.76062277
nonDEG	-0.7816697	0.1906904	1	nonDEG	1.05537653	0.10160893
nonDEG	-0.2557307	0.5640379	1	nonDEG	0.25352437	0.53039447
nonDEG	-1.5990525	0.03717446	1	nonDEG	1.36552233	0.08734828
nonDEG	-0.4841373	0.40955437	1	nonDEG	-0.1306543	0.83298608
nonDEG	0.87986032	0.311291	1	nonDEG	0.05523023	0.90065727
nonDEG	1.60897907	0.01417981	0.88752292	nonDEG	-0.7936471	0.14377214
nonDEG	-0.4913423	0.2797587	1	nonDEG	-0.1065122	0.78395004
nonDEG	0.41192046	0.37885571	1	nonDEG	0.29593332	0.41255406
nonDEG	-0.7074646	0.22580089	1	nonDEG	0.68523031	0.14576871
nonDEG	0.43422173	0.38245466	1	nonDEG	-0.1162928	0.77343712
nonDEG	-0.69938	0.26785445	1	nonDEG	-0.0208291	0.97657299
nonDEG	-1.6184986	0.06934899	1	nonDEG	1.56348774	0.06719349
nonDEG	0.53353602	0.27251806	1	nonDEG	-0.1957619	0.60749772
nonDEG	0.8909514	0.20207758	1	nonDEG	0.67062969	0.25649329
nonDEG	0.22136645	0.80679336	1	nonDEG	-0.5264838	0.56303921
nonDEG	-1.0129046	0.42582405	1	nonDEG	0.58979708	0.73329561
nonDEG	-0.6320453	0.23099936	1	nonDEG	-0.1918101	0.68783314
nonDEG	-1.4086481	0.03934709	1	nonDEG	1.9976229	0.00233717
nonDEG	0.11134901	0.88260929	1	nonDEG	-0.3468436	0.61015978
nonDEG	-1.8777508	0.10995158	1	nonDEG	2.21742598	0.0040776
nonDEG	-0.1930341	0.6578132	1	nonDEG	-0.3648686	0.32929521
nonDEG	-0.6256401	0.14913564	1	nonDEG	0.34311367	0.36586774
nonDEG	-0.0581014	0.89933243	1	nonDEG	-0.2200022	0.57015612
nonDEG	1.23766347	0.0876265	1	nonDEG	-1.5522447	0.0614537
nonDEG	-0.24421	0.59984117	1	nonDEG	0.1699662	0.67949331
nonDEG	-0.6387184	0.19421701	1	nonDEG	0.06696439	0.85951329
nonDEG	-1.0370229	0.07032294	1	nonDEG	0.85068775	0.13147305
nonDEG	-0.4861535	0.2676694	1	nonDEG	0.05698029	0.88021715
nonDEG	1.22464193	0.06833707	1	nonDEG	-0.7006833	0.15156971
nonDEG	0.17980445	0.6720985	1	nonDEG	0.01440207	0.96909625
nonDEG	0.47939884	0.26260175	1	nonDEG	-0.4133643	0.25813499
nonDEG	-0.4539789	0.28182131	1	nonDEG	-0.4005232	0.2918541
nonDEG	-2.164509	0.00541971	0.72967447	nonDEG	0.8350952	0.17170783
nonDEG	0.92983681	0.08098353	1	nonDEG	-0.1460089	0.70388612
nonDEG	-1.2810345	0.03404991	1	nonDEG	2.09149567	0.00221868
nonDEG	0.38040932	0.40003397	1	nonDEG	-0.2633624	0.47283475

nonDEG	-0.6123343	0.25592675	1	nonDEG	-0.1614802	0.71872284
nonDEG	3.11961822	0.00249913	0.61776954	nonDEG	-1.8679384	0.05352069
nonDEG	-0.6493528	0.14364084	1	nonDEG	0.84268145	0.05215995
nonDEG	-1.8493228	0.02721808	1	nonDEG	2.81573745	0.0001526
nonDEG	0.91701762	0.25273679	1	nonDEG	0.25241541	0.73922182
nonDEG	0.41790791	0.36626278	1	nonDEG	-0.173183	0.65800396
nonDEG	0.09349928	0.82941844	1	nonDEG	-0.3360811	0.36731595
nonDEG	-0.6493104	0.25458128	1	nonDEG	0.69575025	0.19152505
nonDEG	-0.5943969	0.31163177	1	nonDEG	0.2739014	0.70628798
nonDEG	-0.1584892	0.79578261	1	nonDEG	-0.4605395	0.47452968
nonDEG	-1.1388238	0.09720999	1	nonDEG	1.4136149	0.02265787
nonDEG	-1.2619512	0.1336208	1	nonDEG	0.26117711	0.75357968
nonDEG	-0.3685514	0.39124593	1	nonDEG	0.40728486	0.27371815
nonDEG	0.62114662	0.25592991	1	nonDEG	0.05384209	0.9160953
nonDEG	-0.2835146	0.5203953	1	nonDEG	-0.2653845	0.49107117
nonDEG	-1.0359485	0.03882647	1	nonDEG	1.14207704	0.04558077
nonDEG	-0.9911279	0.05614198	1	nonDEG	0.66461401	0.10274392
nonDEG	-0.6593073	0.37063435	1	nonDEG	-0.2699563	0.77827642
nonDEG	0.60551709	0.22507215	1	nonDEG	-0.8335809	0.05106611
nonDEG	1.22641309	0.10275148	1	nonDEG	-0.8393225	0.23844236
nonDEG	3.01905951	0.00040397	0.39641127	nonDEG	-1.3234955	0.06004774
nonDEG	1.06727399	0.10604268	1	nonDEG	-0.6679505	0.1249698
nonDEG	-0.5055871	0.45058371	1	nonDEG	0.8085399	0.25850878
nonDEG	0.20268036	0.78372968	1	nonDEG	-0.3518271	0.62268029
nonDEG	2.04514902	0.02514738	1	nonDEG	-1.1392502	0.16903089
nonDEG	-0.7712196	0.08109863	1	nonDEG	0.26044393	0.52711085
nonDEG	-0.2066154	0.63800228	1	nonDEG	0.0436558	0.91155685
nonDEG	-0.4856747	0.26547455	1	nonDEG	-0.3648723	0.37260937
nonDEG	0.76706376	0.09412013	1	nonDEG	-0.2290369	0.56193817
nonDEG	0.05555556	0.90823679	1	nonDEG	-0.0880449	0.85576156
nonDEG	0.80018386	0.0636124	1	nonDEG	-0.1226941	0.73867485
nonDEG	-0.9568605	0.30224311	1	nonDEG	1.91920616	0.01936617
nonDEG	-1.1218155	0.00926255	0.79328284	nonDEG	0.79948126	0.03354999
nonDEG	-0.8520695	0.16699226	1	nonDEG	0.93153826	0.0935296
nonDEG	-1.7037495	0.11760032	1	nonDEG	1.938369	0.06497736
nonDEG	-0.379823	0.38284992	1	nonDEG	0.23894749	0.55175385
nonDEG	-0.9059027	0.04337394	1	nonDEG	-0.2981534	0.45727438
nonDEG	0.37931674	0.62310978	1	nonDEG	-0.2864679	0.724575
nonDEG	0.88126631	0.18882726	1	nonDEG	-0.0282238	0.95738738
nonDEG	0.82590642	0.05821147	1	nonDEG	-0.3083505	0.40235305
nonDEG	-0.4345848	0.33797324	1	nonDEG	0.23203552	0.52486425
nonDEG	-0.4457866	0.41845504	1	nonDEG	0.0127208	0.98543059
nonDEG	-0.3862184	0.38456257	1	nonDEG	0.37035141	0.35231789

nonDEG	0.4033536	0.39320165	1	nonDEG	-0.4315183	0.25591835
nonDEG	-0.3255748	1	1	nonDEG	2.18076668	0.14542029
nonDEG	-1.3737542	0.01146593	0.84810435	nonDEG	0.74191909	0.11926359
nonDEG	-0.9083102	0.09809199	1	nonDEG	0.55256482	0.26954368
nonDEG	0.29994873	0.53428102	1	nonDEG	-0.1340795	0.75480673
nonDEG	1.06427052	0.16267624	1	nonDEG	0.29551118	0.67183705
nonDEG	-0.7386137	0.25352638	1	nonDEG	0.65820272	0.27597952
nonDEG	1.0070529	0.10101887	1	nonDEG	0.09141017	0.87153926
nonDEG	-0.8398168	0.49972972	1	nonDEG	0.97076624	0.42434857
nonDEG	0.02494076	0.96505742	1	nonDEG	-0.0039501	0.99453685
nonDEG	-0.7105567	0.21255832	1	nonDEG	0.21055109	0.70618653
nonDEG	-0.6071105	0.15785362	1	nonDEG	0.05214984	0.89030047
nonDEG	-0.2411624	0.58916864	1	nonDEG	-0.3981742	0.31436954
nonDEG	-0.8376926	0.06211805	1	nonDEG	0.9665127	0.01577672
nonDEG	-1.038454	0.07371777	1	nonDEG	1.2732367	0.0035151
nonDEG	0.66511673	0.33331183	1	nonDEG	0.0112101	0.99101705
nonDEG	0.14847967	0.86788795	1	nonDEG	-0.5261558	0.58501518
nonDEG	-0.5864473	0.18267427	1	nonDEG	0.01272222	0.97707743
nonDEG	-1.3168983	0.20418064	1	nonDEG	5.19225127	4.84E-05
nonDEG	0.95024574	0.03526545	1	nonDEG	-0.3613483	0.39899885
nonDEG	-1.1141988	0.07170027	1	nonDEG	0.42718651	0.50229937
nonDEG	0.1899145	0.68805982	1	nonDEG	-0.2184701	0.60829478
nonDEG	-0.406814	0.34845589	1	nonDEG	-0.287413	0.4322043
nonDEG	0.5035538	0.28569672	1	nonDEG	-0.1219629	0.73960533
nonDEG	0.35797774	0.40858524	1	nonDEG	0.08388224	0.81823947
nonDEG	0.58288221	0.20821958	1	nonDEG	0.7764472	0.04527149
nonDEG	-2.0480941	0.03030129	1	nonDEG	1.10497855	0.22499097
nonDEG	-0.6342042	0.28074522	1	nonDEG	0.62394569	0.31548708
nonDEG	-0.4946909	0.35940692	1	nonDEG	0.25757206	0.62536061
nonDEG	-0.7537535	0.18460242	1	nonDEG	1.12637221	0.00536988
nonDEG	0.40342212	0.58345805	1	nonDEG	0.06896397	0.92580006
nonDEG	0.48032834	0.28195399	1	nonDEG	-0.2682701	0.45557487
nonDEG	-0.1327466	0.77035395	1	nonDEG	-0.5311474	0.16974868
nonDEG	0.18958881	0.81887773	1	nonDEG	0.12737192	0.88708917
nonDEG	0.49203219	0.29506234	1	nonDEG	0.53180913	0.17180616
nonDEG	-0.2928568	0.48733393	1	nonDEG	0.20654574	0.56766843
nonDEG	-2.8547603	0.00951569	0.79482907	nonDEG	2.75123046	0.00430493
nonDEG	-1.3976963	0.08676296	1	nonDEG	2.01267024	0.00441148
nonDEG	-0.7943083	0.22206391	1	nonDEG	0.93757302	0.07980096
nonDEG	1.25198393	0.08349397	1	nonDEG	-0.4640251	0.48509823
nonDEG	-0.5951291	0.21854472	1	nonDEG	-0.1996243	0.63527098
nonDEG	0.28777563	0.55066854	1	nonDEG	-0.0285922	0.94989523
nonDEG	0.06952882	0.87587204	1	nonDEG	-0.1066479	0.78092284

nonDEG	0.11156788	0.83722599	1	nonDEG	-0.0358853	0.94647932
nonDEG	-0.2829742	0.5144324	1	nonDEG	0.53461171	0.1749452
nonDEG	-2.2787977	0.12722679	1	nonDEG	0.52055393	0.71430644
nonDEG	1.56325551	0.00701788	0.75126991	nonDEG	-0.477053	0.32400481
nonDEG	-0.1716982	0.69965423	1	nonDEG	0.0493313	0.89269858
nonDEG	0.33360604	0.48558617	1	nonDEG	-0.4555149	0.25997359
nonDEG	-0.0811325	0.8655067	1	nonDEG	0.06146853	0.89901455
nonDEG	1.28763556	0.22644836	1	nonDEG	0.68290184	0.46581976
nonDEG	0.90969742	0.21706941	1	nonDEG	-0.455455	0.30307587
nonDEG	0.95204933	0.03974029	1	nonDEG	-0.3844542	0.28327129
nonDEG	0.26032903	0.55987896	1	nonDEG	0.14836842	0.70746263
nonDEG	1.9521577	0.02336055	1	nonDEG	-0.8400914	0.30598983
nonDEG	0.90267381	0.08250018	1	nonDEG	-0.2320733	0.60057598
nonDEG	1.57240733	0.00246462	0.61776954	nonDEG	-0.4482418	0.35758233
nonDEG	-0.8475893	0.41776175	1	nonDEG	1.38952482	0.18922472
nonDEG	0.53400687	0.23829149	1	nonDEG	-0.3510576	0.38085361
nonDEG	-1.9782197	0.19472356	1	nonDEG	2.79269617	0.16117963
nonDEG	-0.2628975	0.53662886	1	nonDEG	0.06220251	0.86369473
nonDEG	4.09775602	0.00662762	0.73793371	nonDEG	0.58511332	0.59895778
nonDEG	0.18155068	0.703407	1	nonDEG	-0.3408807	0.3534375
nonDEG	0.75266619	0.4820962	1	nonDEG	-1.4071114	0.08279945
nonDEG	-1.3012893	0.08607183	1	nonDEG	0.0798161	0.94340667
nonDEG	0.46081929	0.45187417	1	nonDEG	0.0663433	0.91275589
nonDEG	-0.7696208	0.11030019	1	nonDEG	0.22570947	0.58822993
nonDEG	-0.0873571	0.85382377	1	nonDEG	-0.0065828	0.9872178
nonDEG	0.40617468	0.37892699	1	nonDEG	0.1254829	0.74030605
nonDEG	-3.5300487	0.0107144	0.83005619	nonDEG	4.17076038	0.00046868
nonDEG	0.66898256	0.21399263	1	nonDEG	-0.331536	0.49471878
nonDEG	1.68325746	0.00166409	0.58320471	nonDEG	-0.5025731	0.22587317
nonDEG	-1.1531205	0.09211036	1	nonDEG	1.06673228	0.12638672
nonDEG	3.09091557	4.41E-06	0.03026052	DEG	-1.4179388	0.02708316
nonDEG	0.64471084	0.74993338	1	nonDEG	0.15448418	1
nonDEG	-0.5964298	0.37369275	1	nonDEG	0.83427789	0.17080082
nonDEG	0.34088153	0.59558867	1	nonDEG	-0.8120005	0.08916639
nonDEG	0.67265199	0.41927838	1	nonDEG	-0.4623436	0.45332981
nonDEG	-0.7837393	0.13914306	1	nonDEG	0.11714351	0.82540714
nonDEG	0.55300183	0.27425762	1	nonDEG	-0.2980862	0.52668593
nonDEG	-0.5284298	0.30204527	1	nonDEG	-0.3606477	0.43659626
nonDEG	-0.5663263	0.18637337	1	nonDEG	0.64557034	0.08254795
nonDEG	2.45977068	0.03033398	1	nonDEG	-1.6029687	0.07823209
nonDEG	-1.5865592	0.01990931	0.98175885	nonDEG	1.10742826	0.08273605
nonDEG	0.26666565	0.52431377	1	nonDEG	-0.513882	0.16710562
nonDEG	0.22018345	0.65243928	1	nonDEG	-0.345038	0.37470619

nonDEG	-0.9803375	0.38716766	1	nonDEG	1.01225648	0.4027249
nonDEG	0.79131143	0.10753239	1	nonDEG	-0.7863686	0.07686432
nonDEG	2.59959888	0.12037451	1	nonDEG	-2.086536	0.23163701
nonDEG	-0.467151	0.34761636	1	nonDEG	-0.1333113	0.76013225
nonDEG	0.09303579	0.84200071	1	nonDEG	0.18127617	0.66188186
nonDEG	0.31916363	0.51369897	1	nonDEG	-0.3103857	0.4622487
nonDEG	0.11478411	0.91969002	1	nonDEG	-1.2559351	0.1336306
nonDEG	-0.9627702	0.26681794	1	nonDEG	1.05922051	0.16685626
nonDEG	-2.6135114	0.05858022	1	nonDEG	1.00460622	0.39230733
nonDEG	-0.869349	0.538492	1	nonDEG	-5.0330298	0.0010139
nonDEG	0.81237089	0.16395053	1	nonDEG	-0.4197451	0.39236592
nonDEG	-5.8400335	0.00358544	0.67010784	nonDEG	3.82851334	0.00675472
nonDEG	-0.4246867	0.5004872	1	nonDEG	0.456758	0.43609557
nonDEG	1.79966608	0.17498321	1	nonDEG	-1.9219349	0.21572238
nonDEG	-0.9496257	0.25615642	1	nonDEG	0.5180701	0.54853822
nonDEG	-0.5029696	0.32871606	1	nonDEG	-0.1678563	0.7091915
nonDEG	-2.4604489	0.00284826	0.62843199	nonDEG	1.5437601	0.11130646
nonDEG	-1.2815564	0.02711293	1	nonDEG	2.38607465	0.00189938
nonDEG	0.81789694	0.10778473	1	nonDEG	-0.0698306	0.86303034
nonDEG	-0.9984205	0.04358664	1	nonDEG	1.22012804	0.0307625
nonDEG	0.44407739	0.578035	1	nonDEG	0.39156999	0.5074261
nonDEG	-0.3930531	0.36365334	1	nonDEG	0.21961337	0.54695511
nonDEG	-1.9295439	0.19480384	1	nonDEG	2.72988532	0.08690871
nonDEG	-0.4009836	0.36586571	1	nonDEG	0.39561102	0.29794873
nonDEG	-0.0119318	0.97924221	1	nonDEG	0.4691561	0.21457157
nonDEG	-0.7280102	0.22920121	1	nonDEG	0.28676961	0.56834843
nonDEG	-0.548923	0.19157571	1	nonDEG	0.5376146	0.16056674
nonDEG	-0.3800453	0.37165859	1	nonDEG	-0.0163317	0.96739016
nonDEG	-0.2117773	0.65203115	1	nonDEG	0.21210244	0.61754746
nonDEG	-0.324722	0.47790904	1	nonDEG	-0.7423657	0.05863795
nonDEG	0.44783311	0.3760574	1	nonDEG	0.94674838	0.03133223
nonDEG	-0.5124717	0.22944775	1	nonDEG	0.6777627	0.07447387
nonDEG	0.22436307	0.66913411	1	nonDEG	-0.4127501	0.26025074
nonDEG	-0.1218062	0.79806572	1	nonDEG	-0.0541368	0.90154236
nonDEG	-1.6766607	0.17444002	1	nonDEG	0.54385808	0.6621186
nonDEG	1.03562607	0.12234782	1	nonDEG	-1.2406639	0.02625993
nonDEG	-1.7918767	0.6188065	1	nonDEG	2.84314809	0.3561664
nonDEG	-0.8308668	0.0726592	1	nonDEG	-0.1623461	0.66101422
nonDEG	-0.5955766	0.58281797	1	nonDEG	0.35037066	0.7996702
nonDEG	-0.0905165	0.84430899	1	nonDEG	-0.2203427	0.56387374
nonDEG	-0.1451168	0.7500592	1	nonDEG	-0.129346	0.72885849
nonDEG	0.94172555	0.16185699	1	nonDEG	-0.7559039	0.1888236
nonDEG	-0.1866098	0.66961326	1	nonDEG	0.03660917	0.92304203

nonDEG	-1.1071624	0.10790727	1	nonDEG	1.37564264	0.03033603
nonDEG	0.25899068	0.58870445	1	nonDEG	-0.2258095	0.62425655
nonDEG	-0.1630924	0.72145365	1	nonDEG	0.03609689	0.93411438
nonDEG	-0.3438114	0.47851096	1	nonDEG	0.04376488	0.93635553
nonDEG	-0.204751	0.63922642	1	nonDEG	0.36075082	0.35074156
nonDEG	-0.2050407	0.63605745	1	nonDEG	0.2675245	0.47023686
DEG	0.40570256	0.3610574	1	nonDEG	-0.110591	0.76562897
nonDEG	-0.425634	0.47252906	1	nonDEG	0.127197	0.82672251
nonDEG	0.56392118	0.46432664	1	nonDEG	-1.2235773	0.0706594
nonDEG	-0.7135115	0.21272342	1	nonDEG	0.40430158	0.45957092
nonDEG	1.86284016	0.0179943	0.94091332	nonDEG	-1.0088804	0.17842187
nonDEG	-0.052302	0.90470051	1	nonDEG	-0.1081627	0.77696623
nonDEG	0.06727619	0.88850939	1	nonDEG	-0.6395407	0.11192304
nonDEG	-0.3890436	0.37259919	1	nonDEG	0.12598751	0.75058781
nonDEG	0.49226686	0.30407215	1	nonDEG	-0.2927609	0.44077275
nonDEG	0.82095234	0.25483458	1	nonDEG	-0.0951899	0.89048845
nonDEG	0.55891783	0.34016968	1	nonDEG	0.2355607	0.67690862
nonDEG	0.73031045	0.15354109	1	nonDEG	-0.196835	0.64198301
nonDEG	0.44036275	0.53662941	1	nonDEG	0.93176037	0.1816862
nonDEG	-0.4283222	0.35035608	1	nonDEG	0.22559221	0.58159526
nonDEG	-0.0383007	0.93903814	1	nonDEG	0.04809321	0.91238577
nonDEG	-0.1035054	0.86209633	1	nonDEG	-0.0975899	0.81056924
nonDEG	1.47955572	0.32270291	1	nonDEG	-3.2078173	0.05831766
nonDEG	-0.3285424	0.6144513	1	nonDEG	-1.1434704	0.11301985
nonDEG	0.0175487	0.96914213	1	nonDEG	0.23266591	0.54589313
nonDEG	-0.472823	0.35178931	1	nonDEG	-0.8457438	0.1053463
nonDEG	-0.1542441	0.76984732	1	nonDEG	0.13003821	0.79586221
nonDEG	0.09040998	0.84155638	1	nonDEG	0.35868715	0.48116015
nonDEG	-0.5623468	0.1883648	1	nonDEG	0.67005884	0.07621116
nonDEG	-0.2337707	0.58295654	1	nonDEG	-0.1395841	0.70458425
nonDEG	1.52457124	0.02783711	1	nonDEG	-0.9113433	0.17518381
nonDEG	0.28479313	0.59297154	1	nonDEG	-1.152885	0.02298801
nonDEG	-0.5494639	0.2783841	1	nonDEG	-0.0681806	0.90778642
nonDEG	-3.6494109	0.46208804	1	nonDEG	5.08601564	0.23104483
nonDEG	0.90337234	0.15107665	1	nonDEG	-0.1017788	0.87346251
nonDEG	-1.5294252	0.01216179	0.85229819	nonDEG	-0.1192856	0.74253971
nonDEG	-2.2793097	0.10168939	1	nonDEG	3.33612358	0.00414301
nonDEG	1.28011888	0.22766573	1	nonDEG	-0.7422043	0.52265748
nonDEG	-0.2824961	0.56221669	1	nonDEG	-0.2658296	0.58277615
nonDEG	-0.4860643	0.24437004	1	nonDEG	0.36498023	0.30969695
nonDEG	-1.447611	0.13663171	1	nonDEG	0.50142505	0.61313844
nonDEG	-0.2102747	0.89333518	1	nonDEG	3.80066834	5.51E-05
nonDEG	-0.2656905	0.53031461	1	nonDEG	-0.0913379	0.83107302

nonDEG	-1.2368358	0.11351306	1	nonDEG	0.25075373	0.63566908
nonDEG	0.18855915	0.66131848	1	nonDEG	-0.3220705	0.44305139
nonDEG	-1.8672419	0.0274808	1	nonDEG	0.52252792	0.49095286
nonDEG	-0.0566826	0.90932598	1	nonDEG	-0.7396649	0.1305545
nonDEG	-0.0570438	0.92213364	1	nonDEG	1.28228911	0.01279248
nonDEG	-0.3389709	0.46374239	1	nonDEG	0.10871398	0.79314809
nonDEG	-0.2299534	0.60671179	1	nonDEG	0.7989166	0.09350777
nonDEG	-0.1290067	0.7722337	1	nonDEG	0.18551464	0.64181801
nonDEG	-0.5450369	0.38818293	1	nonDEG	0.86304979	0.10362751
nonDEG	0.46624791	0.33522611	1	nonDEG	0.42064658	0.32458748
nonDEG	-0.5110283	0.36436814	1	nonDEG	0.09183574	0.85732835
nonDEG	0.26783968	0.69620542	1	nonDEG	-0.8920117	0.2237986
nonDEG	1.95030989	0.26883681	1	nonDEG	-0.2380892	0.8971507
DEG	6.75372989	0.0037861	0.67010784	nonDEG	2.14154921	0.10207122
nonDEG	-0.4753267	0.33373611	1	nonDEG	1.34391976	0.00134381
nonDEG	0.99642343	0.05188595	1	nonDEG	-0.3829651	0.34280305
nonDEG	-0.7611929	0.07687874	1	nonDEG	0.68123146	0.08619536
nonDEG	-0.0283971	0.94711493	1	nonDEG	-0.1915924	0.59858813
nonDEG	0.19220251	0.8110126	1	nonDEG	-1.0058909	0.16186019
nonDEG	1.71147523	0.0237606	1	nonDEG	-0.9731172	0.18050483
nonDEG	-0.001612	0.99926967	1	nonDEG	-0.7810173	0.08743407
nonDEG	0.96537891	0.03932639	1	nonDEG	-0.5287486	0.16104545
DEG	6.46184813	0.04315438	1	nonDEG	-2.186629	0.38450423
nonDEG	0.56116541	0.4495662	1	nonDEG	0.32357503	0.63131819
nonDEG	0.20340689	0.63844459	1	nonDEG	0.01079222	0.98105996
nonDEG	0.39845299	0.44075131	1	nonDEG	-0.7137979	0.1031006
nonDEG	-0.3367396	0.44203678	1	nonDEG	0.15283015	0.68232841
nonDEG	-0.5478282	0.28838618	1	nonDEG	0.79987279	0.08146524
nonDEG	0.98625178	0.25044275	1	nonDEG	-0.5275394	0.55891229
nonDEG	-0.5650707	0.19879534	1	nonDEG	0.51557346	0.16490426
nonDEG	0.10860376	0.79961218	1	nonDEG	-0.1892692	0.60284193
nonDEG	-0.6590818	0.30094317	1	nonDEG	0.77767426	0.08833118
nonDEG	-0.2713028	0.51534424	1	nonDEG	-0.1151172	0.75111935
nonDEG	1.13791058	0.05188118	1	nonDEG	-0.5097604	0.29161633
nonDEG	-1.0366268	0.10916901	1	nonDEG	-0.8252777	0.18396194
nonDEG	-1.2470347	0.17835006	1	nonDEG	0.90830866	0.20877305
nonDEG	-0.5817598	0.18330427	1	nonDEG	-0.116735	0.76539848
DEG	3.1567108	0.00430401	0.68407511	nonDEG	-1.926045	0.04640102
nonDEG	-0.1874116	0.73665195	1	nonDEG	0.30305452	0.53605867
nonDEG	-0.0374407	0.93982999	1	nonDEG	-0.6593564	0.12081572
nonDEG	-0.555858	0.45239543	1	nonDEG	0.38992269	0.56837346
nonDEG	-0.0395766	0.93085956	1	nonDEG	-0.1016176	0.79062227
nonDEG	1.47542136	0.01760468	0.93380915	nonDEG	-0.5020518	0.39030055

nonDEG	-1.1622184	0.01216017	0.85229819	nonDEG	-1.4477363	0.00062199
nonDEG	0.27365247	0.53238094	1	nonDEG	-0.1426883	0.70528577
nonDEG	-0.0185103	0.96928724	1	nonDEG	-0.8967334	0.01446964
nonDEG	4.70280755	0.22893743	1	nonDEG	-1.8363818	0.59034623
nonDEG	-2.2680375	0.097373	1	nonDEG	-0.986792	0.53174018
nonDEG	-0.7777853	0.12698991	1	nonDEG	0.0316972	0.94485233
nonDEG	-0.0625704	0.89295049	1	nonDEG	-0.2881844	0.46847421
nonDEG	0.12196851	0.78912975	1	nonDEG	-0.4863766	0.22305294
nonDEG	0.1259637	1	1	nonDEG	2.12787788	0.19405413
nonDEG	0.27629289	0.81290167	1	nonDEG	-0.4482151	0.745705
nonDEG	-0.308381	0.48658053	1	nonDEG	-0.2159398	0.56370391
nonDEG	-0.4881526	0.32276052	1	nonDEG	0.34585281	0.41975045
nonDEG	0.14804394	0.76099677	1	nonDEG	-1.695111	0.00061922
nonDEG	-0.4274002	0.33597398	1	nonDEG	0.84191792	0.04062238
nonDEG	-0.0071288	0.99055133	1	nonDEG	0.11275351	0.79798153
nonDEG	-1.1303152	0.11915779	1	nonDEG	0.00661738	0.99400528
nonDEG	0.68174892	0.28254935	1	nonDEG	-0.5644009	0.32574996
nonDEG	-0.0743381	0.8618448	1	nonDEG	0.181139	0.62236085
nonDEG	0.76811298	0.15434673	1	nonDEG	-0.1449142	0.73631133
nonDEG	0.02464856	0.95471742	1	nonDEG	-0.1256969	0.73460124
nonDEG	-0.0066358	0.99339131	1	nonDEG	-0.8884606	0.19634143
nonDEG	-0.1847617	0.68441314	1	nonDEG	0.19992388	0.6237643
nonDEG	-0.0723127	0.87878486	1	nonDEG	-0.0562303	0.88948511
nonDEG	-0.6433122	0.1557994	1	nonDEG	0.53265843	0.22136331
nonDEG	-0.1395435	0.8399858	1	nonDEG	-0.9561213	0.19451262
nonDEG	0.66650345	0.40904477	1	nonDEG	-1.1791194	0.09883026
nonDEG	0.51062937	0.40305782	1	nonDEG	0.43645777	0.49819869
nonDEG	-0.7051514	0.12200274	1	nonDEG	0.05566368	0.88079106
nonDEG	0.20318858	0.71617933	1	nonDEG	-0.1662501	0.74431185
nonDEG	-0.2943613	0.48124953	1	nonDEG	0.69556122	0.15849725
nonDEG	0.62250649	0.24895256	1	nonDEG	-0.2884604	0.49661939
nonDEG	-0.1887327	0.72368825	1	nonDEG	-0.7039294	0.18022969
nonDEG	0.04926913	0.94325944	1	nonDEG	-0.1405459	0.8159138
nonDEG	0.39572976	0.47587818	1	nonDEG	-0.5289857	0.31396242
nonDEG	1.88682444	0.01710769	0.93079586	nonDEG	-1.0075188	0.18788465
nonDEG	-1.3871334	0.20962752	1	nonDEG	1.68769275	0.15186521
nonDEG	0.3306751	0.85667059	1	nonDEG	0.98110297	0.52254076
nonDEG	0.18410304	0.7527579	1	nonDEG	-0.4444874	0.39052812
nonDEG	-0.0744233	0.8986021	1	nonDEG	-0.2082699	0.63761569
nonDEG	0.51482874	0.38751316	1	nonDEG	-0.6671976	0.2220192
nonDEG	-0.5493282	0.22230189	1	nonDEG	0.56829792	0.22657161
nonDEG	-0.0082505	0.98772335	1	nonDEG	0.11674045	0.82373747
nonDEG	-0.3609851	0.42199024	1	nonDEG	0.12121448	0.75324382

nonDEG	-0.2274344	0.58726957	1	nonDEG	0.23194757	0.53541549
nonDEG	-0.3749055	0.82350086	1	nonDEG	1.93787394	0.21345095
nonDEG	-0.1019853	0.81603418	1	nonDEG	-0.0527842	0.89484691
nonDEG	-0.0195841	0.96625259	1	nonDEG	-0.2381003	0.57582452
nonDEG	0.41595902	0.43252769	1	nonDEG	-0.406963	0.39943384
nonDEG	-0.1171645	0.82102087	1	nonDEG	-0.8602285	0.06871665
nonDEG	-0.4398605	0.31851124	1	nonDEG	-0.3185156	0.39638148
nonDEG	-0.4658682	0.30955005	1	nonDEG	0.08432449	0.83803501
nonDEG	-0.9646561	0.12398501	1	nonDEG	0.9465378	0.03706387
nonDEG	1.89526741	0.1007849	1	nonDEG	0.81481559	0.40870468
nonDEG	-0.4313856	0.33147789	1	nonDEG	0.19210066	0.61263785
DEG	-0.139266	0.8663067	1	nonDEG	0.87095623	0.27267466
nonDEG	-0.9628038	0.0553852	1	nonDEG	-0.5394948	0.31584528
nonDEG	0.41424925	0.46084733	1	nonDEG	-0.8270865	0.0793219
nonDEG	0.44023691	0.59055734	1	nonDEG	-0.8308238	0.26081817
nonDEG	0.49496542	0.43831182	1	nonDEG	-0.6421873	0.30474286
nonDEG	-0.3569392	0.43884757	1	nonDEG	0.38597977	0.34252311
nonDEG	0.37723493	0.47760129	1	nonDEG	-1.3867886	0.00736866
nonDEG	-0.294356	0.51944557	1	nonDEG	0.01796245	0.967917
nonDEG	-0.8947834	0.28266485	1	nonDEG	-1.1208649	0.10951679
nonDEG	-0.1950181	0.66056356	1	nonDEG	0.25122622	0.51069629
nonDEG	0.21080915	0.660024	1	nonDEG	0.11031433	0.80704932
nonDEG	-0.1750526	0.68227317	1	nonDEG	0.28764113	0.42792788
nonDEG	0.15629224	0.73210351	1	nonDEG	0.11445069	0.77142926
nonDEG	0.00722875	0.98764761	1	nonDEG	0.0989152	0.795618
nonDEG	-0.5057079	0.26025736	1	nonDEG	0.03392848	0.9304936
nonDEG	-0.651986	0.1706683	1	nonDEG	-0.3951055	0.36763711
nonDEG	0.73783498	0.22147047	1	nonDEG	-0.0696592	0.90839211
nonDEG	-0.3771613	0.38650181	1	nonDEG	0.55315035	0.13227948
nonDEG	-0.2374074	0.58084559	1	nonDEG	0.11641169	0.76910307
nonDEG	-0.2616657	0.63976803	1	nonDEG	-0.4359576	0.38375185
nonDEG	0.19486439	0.71667456	1	nonDEG	0.2127172	0.70205034
DEG	1.84209592	3.85E-05	0.12599308	nonDEG	-0.15585	0.86124272
nonDEG	1.97656768	0.0963949	1	nonDEG	-0.6350443	0.71175115
nonDEG	-0.0881068	0.88975116	1	nonDEG	-1.087709	0.08380857
nonDEG	-0.4769456	0.3010302	1	nonDEG	0.16845219	0.73610342
nonDEG	-0.305986	0.65922715	1	nonDEG	-0.0334679	0.96519264
DEG	2.04413792	0.00734367	0.75263895	nonDEG	-1.65218	0.02022999
nonDEG	-1.2489364	0.02398318	1	nonDEG	0.33638571	0.44288517
nonDEG	1.31606761	0.03675206	1	nonDEG	-1.0751427	0.08480841
nonDEG	-1.5886474	0.12911286	1	nonDEG	1.31414794	0.10242086
nonDEG	0.07177738	0.87714986	1	nonDEG	-0.2277307	0.59287038
nonDEG	-0.7095751	0.12212803	1	nonDEG	0.28773016	0.48404046

nonDEG	-0.1591408	0.73295246	1	nonDEG	0.14112395	0.74221191
nonDEG	-0.3622408	0.41372855	1	nonDEG	-0.0457475	0.91030698
nonDEG	-0.8377897	0.08041979	1	nonDEG	-0.1113222	0.758729
nonDEG	-0.2112085	0.64305921	1	nonDEG	-0.1315234	0.76148275
nonDEG	0.02819347	1	1	nonDEG	1.28040967	0.18399581
nonDEG	-0.3088675	0.64196061	1	nonDEG	0.59957606	0.23574135
nonDEG	0.51635703	0.28509285	1	nonDEG	-0.9677481	0.02389418
nonDEG	-0.6355744	0.153014	1	nonDEG	0.0276494	0.94404735
nonDEG	-0.5769111	0.2585522	1	nonDEG	0.67000497	0.07879203
nonDEG	0.74345257	0.22022964	1	nonDEG	-0.3310554	0.49997366
DEG	0.46411608	0.38888359	1	nonDEG	0.51026145	0.34841077
nonDEG	-1.5748578	0.13097932	1	nonDEG	2.25625419	0.04897473
nonDEG	0.5600072	0.33837924	1	nonDEG	-0.1608875	0.76310345
nonDEG	-0.9117632	0.14023684	1	nonDEG	-0.1404401	0.83845559
nonDEG	-0.2399748	0.81963747	1	nonDEG	1.39888407	0.12423699
nonDEG	0.31307287	0.45923886	1	nonDEG	-0.1611398	0.65142388
nonDEG	-0.5674151	0.18596634	1	nonDEG	0.25210029	0.50174126
nonDEG	1.16857832	0.13932752	1	nonDEG	-0.8267211	0.26342516
nonDEG	0.00936905	0.98397107	1	nonDEG	-0.4844748	0.23310759
nonDEG	-0.841654	0.07945891	1	nonDEG	-0.1601986	0.6634411
nonDEG	1.95893826	0.01332605	0.86906821	nonDEG	-0.9752363	0.20121857
nonDEG	-0.2403635	0.58787631	1	nonDEG	0.29201387	0.49324581
nonDEG	-0.3659216	0.3978513	1	nonDEG	0.76263243	0.04872246
nonDEG	1.02256717	0.22498489	1	nonDEG	-0.6410111	0.36149421
nonDEG	-0.0895893	0.83263535	1	nonDEG	-0.1339589	0.72123501
nonDEG	0.25464334	0.57143169	1	nonDEG	-1.0276744	0.01348559
nonDEG	0.07277021	0.88333655	1	nonDEG	-0.209182	0.65346603
nonDEG	0.62600147	0.4151768	1	nonDEG	-0.7777328	0.28393403
nonDEG	0.88934274	0.19348943	1	nonDEG	-0.6387084	0.34188348
nonDEG	0.13750466	0.80250455	1	nonDEG	-0.1244262	0.78740076
nonDEG	-0.8708981	0.15999764	1	nonDEG	0.63472747	0.33620994
nonDEG	-3.7160119	0.39581646	1	nonDEG	2.21587283	1
nonDEG	-0.7311467	0.24299885	1	nonDEG	-0.1029215	0.85112952
nonDEG	0.92826176	0.14635463	1	nonDEG	-0.5522166	0.29784563
nonDEG	1.92733765	0.01374358	0.87955945	nonDEG	-0.9581808	0.20049528
nonDEG	-0.4590547	0.3186451	1	nonDEG	0.77374456	0.06735549
nonDEG	-0.0475207	0.91739577	1	nonDEG	0.064048	0.8724601
nonDEG	1.07603314	0.02044587	0.9902683	nonDEG	-0.5769709	0.13354596
nonDEG	-0.1345581	0.80928849	1	nonDEG	-0.7881131	0.12086287
nonDEG	-0.4492583	0.31361959	1	nonDEG	0.51729753	0.19252419
nonDEG	-0.6590103	0.16172947	1	nonDEG	0.49610847	0.26363042
nonDEG	0.12283089	0.84138076	1	nonDEG	0.04632595	0.93940797
nonDEG	-0.3267397	0.62595256	1	nonDEG	-0.9922082	0.15527522

nonDEG	-0.7382377	0.11097664	1	nonDEG	0.81875552	0.07489091
nonDEG	0.00232004	0.99615599	1	nonDEG	0.07294396	0.84448716
nonDEG	-0.5577402	0.22224295	1	nonDEG	0.84162464	0.0260046
nonDEG	-0.1203407	0.8087078	1	nonDEG	0.09138335	0.83577372
nonDEG	1.76053321	0.02164952	1	nonDEG	-0.8510451	0.26111737
nonDEG	0.79522371	0.33433214	1	nonDEG	-1.4654271	0.04885005
nonDEG	0.24928837	0.61982266	1	nonDEG	-0.6516199	0.1728704
nonDEG	-0.1249102	0.77234384	1	nonDEG	-0.0262372	0.94231937
nonDEG	-0.4812273	0.26685366	1	nonDEG	-0.5949837	0.11666266
nonDEG	-0.5000175	0.32963922	1	nonDEG	0.63732814	0.19786499
nonDEG	0.07231603	0.8672998	1	nonDEG	0.10469372	0.77643876
nonDEG	-0.5864467	0.19107401	1	nonDEG	0.42191202	0.3113105
nonDEG	-0.421094	0.48974024	1	nonDEG	0.57073777	0.26258391
nonDEG	0.74338272	0.25550959	1	nonDEG	0.08245099	0.89885713
nonDEG	-0.0845306	0.85651238	1	nonDEG	0.24376816	0.52576829
nonDEG	0.68793007	0.11252126	1	nonDEG	-0.4170356	0.26679684
nonDEG	0.53179732	0.4821771	1	nonDEG	-0.0731292	0.92100439
nonDEG	0.57507993	0.59658854	1	nonDEG	1.35084159	0.16419156
nonDEG	0.33639427	0.47560344	1	nonDEG	-0.2762061	0.52256206
nonDEG	-0.2870697	0.80619899	1	nonDEG	-2.7728861	0.05010497
nonDEG	0.46449845	0.35909541	1	nonDEG	0.5026588	0.24694148
nonDEG	-0.1597112	0.71723011	1	nonDEG	0.03242657	0.93229427
nonDEG	-0.1859862	0.69531365	1	nonDEG	-0.0112774	0.97885349
nonDEG	1.60749221	0.02621661	1	nonDEG	-0.9790096	0.1600726
nonDEG	-0.5353808	0.45826121	1	nonDEG	0.54827073	0.41176251
nonDEG	-0.168605	0.70781521	1	nonDEG	-0.0074151	0.98649944
nonDEG	-0.3100867	0.58744882	1	nonDEG	0.13633019	0.80807786
nonDEG	0.08470309	0.91599981	1	nonDEG	-0.9785766	0.1623988
nonDEG	-0.0785803	0.86477135	1	nonDEG	-0.3913385	0.34363838
nonDEG	-0.5510826	0.34606394	1	nonDEG	0.23355469	0.58879056
nonDEG	-0.5736225	0.32340154	1	nonDEG	0.19591417	0.73160382
nonDEG	-0.1844531	0.6762951	1	nonDEG	0.37844041	0.2958852
nonDEG	0.3899231	0.38767563	1	nonDEG	-0.3293362	0.39226606
nonDEG	-0.340497	0.44759521	1	nonDEG	0.21852011	0.57913693
nonDEG	0.45305314	0.31582548	1	nonDEG	-0.5169118	0.17131437
nonDEG	-0.6239165	0.20347479	1	nonDEG	0.19647153	0.66535128
nonDEG	-0.3355671	0.44342721	1	nonDEG	0.40815942	0.27291064
nonDEG	0.4197998	0.36248926	1	nonDEG	0.43267559	0.29358996
nonDEG	0.27127137	0.66552447	1	nonDEG	0.35714686	0.59018985
nonDEG	6.42457135	0.03979303	1	nonDEG	-1.16895	0.6472572
nonDEG	-0.7243617	0.33778418	1	nonDEG	-1.3340237	0.06356935
nonDEG	0.0583966	0.89630587	1	nonDEG	-0.092453	0.80634696
nonDEG	-0.0667382	0.8964192	1	nonDEG	0.19563417	0.63791458

nonDEG	0.42472313	0.43688523	1	nonDEG	-0.4124404	0.38306989
nonDEG	-0.9500347	0.36823388	1	nonDEG	-1.8958075	0.26757155
nonDEG	-0.2487622	0.63122676	1	nonDEG	-0.5255751	0.28775355
nonDEG	0.58514906	0.21370493	1	nonDEG	-0.4413585	0.27130692
nonDEG	2.6060188	0.02166084	1	nonDEG	-0.392107	0.71402203
nonDEG	-2.0057395	0.19953974	1	nonDEG	2.32695579	0.16681016
nonDEG	-0.1212711	0.78662908	1	nonDEG	0.24791373	0.51691699
nonDEG	0.09899541	0.819157	1	nonDEG	-0.3758459	0.31077305
nonDEG	2.05928707	0.0126104	0.85364221	nonDEG	-1.0449041	0.19636519
nonDEG	0.29148042	0.50059961	1	nonDEG	0.17795604	0.63209422
nonDEG	-0.4286884	0.39144115	1	nonDEG	0.24922489	0.51898087
nonDEG	-0.6032109	0.2173492	1	nonDEG	0.52810473	0.29134557
DEG	0.18285624	0.72016673	1	nonDEG	-0.8073516	0.05679373
nonDEG	-1.2884313	0.06354731	1	nonDEG	1.31806402	0.00088757
nonDEG	1.19613471	0.21825376	1	nonDEG	-1.9719998	0.12258447
nonDEG	0.40106517	0.51146456	1	nonDEG	-0.0234339	0.96812852
nonDEG	0.57442554	0.33537833	1	nonDEG	-0.4229138	0.44780822
nonDEG	0.37781964	0.70728304	1	nonDEG	-0.2744104	0.79643313
nonDEG	0.07938161	0.91766151	1	nonDEG	-0.8864505	0.14971394
nonDEG	0.93764822	0.13662969	1	nonDEG	-0.4303111	0.41623337
nonDEG	0.47669772	0.50412313	1	nonDEG	-1.6108632	0.02270068
nonDEG	0.05448378	0.90011994	1	nonDEG	-0.0915574	0.81168422
nonDEG	0.45511948	0.45488102	1	nonDEG	-0.8278278	0.13616098
nonDEG	-0.4055071	0.33873728	1	nonDEG	0.58019974	0.12860983
nonDEG	-1.8396647	0.08749177	1	nonDEG	1.22638496	0.12740979
nonDEG	-0.1584471	0.72866713	1	nonDEG	-0.0462384	0.91975914
nonDEG	2.19963925	0.2057094	1	nonDEG	0.57425241	0.72088708
nonDEG	-0.0797314	0.89757011	1	nonDEG	0.56450444	0.29183891
nonDEG	-0.1553424	0.73105432	1	nonDEG	0.29109326	0.456525
nonDEG	-0.708931	0.19607489	1	nonDEG	0.46174604	0.33383408
nonDEG	-0.3240252	0.58765754	1	nonDEG	0.25299234	0.6936577
nonDEG	-0.0968992	0.8222837	1	nonDEG	-0.0313934	0.93263497
nonDEG	0.42012062	0.73525213	1	nonDEG	0.32121785	0.81490928
nonDEG	-0.271017	0.52659753	1	nonDEG	0.45374564	0.21754104
nonDEG	1.25006509	0.27684902	1	nonDEG	-1.0189709	0.56522444
nonDEG	0.57737516	0.45624632	1	nonDEG	-1.1452701	0.09969998
nonDEG	-0.8323996	0.10331637	1	nonDEG	-0.0841584	0.82013038
nonDEG	-0.052235	0.95329237	1	nonDEG	-0.4663907	0.59169448
nonDEG	-0.2829801	0.50820759	1	nonDEG	0.01086553	0.97810927
nonDEG	0.695718	0.38362964	1	nonDEG	-1.2347377	0.08499771
nonDEG	-0.258418	0.54458181	1	nonDEG	0.16892954	0.63845608
nonDEG	0.09777574	0.82598041	1	nonDEG	0.25608438	0.49319719
nonDEG	0.58468004	0.44639987	1	nonDEG	-1.1179934	0.10685545

nonDEG	0.53558873	0.29297977	1	nonDEG	0.31825502	0.53554806
nonDEG	0.00707606	0.98740014	1	nonDEG	0.19672828	0.59562809
nonDEG	-0.1062382	0.88258972	1	nonDEG	0.19260665	0.63056723
DEG	3.0853871	0.00492132	0.70646871	nonDEG	1.11561247	0.24212638
DEG	1.94333227	0.00187727	0.61198441	nonDEG	0.4821751	0.29396398
DEG	1.91828875	0.01536773	0.90015177	nonDEG	1.04774188	0.02644627
DEG	1.65557949	0.00107348	0.51315728	nonDEG	-0.0470722	0.91421146
DEG	-1.6526186	0.08777774	1	nonDEG	1.30345462	0.32238827
DEG	1.52051333	0.11321655	1	nonDEG	0.96913119	0.24818888
DEG	0.92011303	0.33784093	1	nonDEG	-0.7488858	0.43738022
DEG	1.38240676	0.13833355	1	nonDEG	1.69009678	0.02078049
DEG	1.43162721	0.07542706	1	nonDEG	-0.5022021	0.5285738
DEG	-2.1571574	0.12931194	1	nonDEG	2.44920803	0.17327362
DEG	0.98635801	0.48858404	1	nonDEG	1.70843123	0.29895062
DEG	0.12538418	0.77695042	1	nonDEG	1.10471935	0.00316316
DEG	2.14200933	0.06666135	1	nonDEG	1.00246522	0.33852559
DEG	1.70049095	0.00148096	0.55187146	nonDEG	-0.5190779	0.30808795
DEG	0.41726352	0.39737587	1	nonDEG	0.47409534	0.23184678
DEG	0.60681415	0.21422201	1	nonDEG	0.26884785	0.53008006
DEG	0.14220177	0.83038928	1	nonDEG	1.83392508	0.00428459
DEG	1.88854906	0.04340199	1	nonDEG	1.47309713	0.08986072
DEG	1.99287546	0.03718832	1	nonDEG	-0.147505	0.88269023
DEG	1.7475667	0.05255429	1	nonDEG	0.1803205	0.83243219
DEG	1.36713731	0.01129211	0.84185458	nonDEG	0.18332819	0.68428352
DEG	3.32876844	0.03750884	1	nonDEG	0.27001607	0.82642875
DEG	1.7463237	0.09355453	1	nonDEG	2.99301642	0.0005626
DEG	-0.0432794	0.95216348	1	nonDEG	1.52679315	0.05608995
DEG	1.48364346	0.03074535	1	nonDEG	1.03887032	0.05067177
DEG	0.97534743	0.10756967	1	nonDEG	0.45458837	0.43905469
nonDEG	-0.112847	0.81542074	1	nonDEG	1.92625556	7.50E-07
nonDEG	-2.4132962	0.00990776	0.80027302	nonDEG	3.78280008	3.82E-09
nonDEG	-4.6519457	0.00094599	0.51194803	nonDEG	5.26134705	2.51E-06
nonDEG	-0.8338773	0.51217695	1	nonDEG	4.27076584	1.36E-05
nonDEG	0.08060564	0.85166228	1	nonDEG	0.06332892	0.86242581
nonDEG	0.06380937	1	1	nonDEG	1.67079385	0.09264993
nonDEG	-0.0159764	0.97054943	1	nonDEG	-0.1557056	0.67184563
nonDEG	-0.3222422	0.44361296	1	nonDEG	0.0383106	0.91898824
nonDEG	-0.1406659	0.74208198	1	nonDEG	-0.2974191	0.42891543
nonDEG	0.51017043	0.62383437	1	nonDEG	0.07296926	0.97391
nonDEG	0.05210379	0.90284399	1	nonDEG	-0.2276674	0.52870455
nonDEG	0.48152296	0.26040373	1	nonDEG	-0.6943677	0.08433365
nonDEG	-0.2699962	0.55229607	1	nonDEG	0.04356843	0.91144149
nonDEG	0.06001524	0.89302334	1	nonDEG	-0.4002573	0.28152487

nonDEG	-0.0994937	0.82461228	1	nonDEG	-0.3677649	0.32464236
nonDEG	0.94886452	0.0290908	1	nonDEG	0.40411848	0.29373751
nonDEG	0.27591592	0.60952686	1	nonDEG	-0.1642533	0.75180007
nonDEG	0.00869011	0.99038076	1	nonDEG	0.11527417	0.80977012
nonDEG	-0.5882103	0.44795087	1	nonDEG	-0.3843497	0.66906152
nonDEG	0.4573969	0.44976585	1	nonDEG	0.23135323	0.68650265
nonDEG	-0.1428215	0.80309255	1	nonDEG	-0.1761772	0.73699223
nonDEG	0.11633976	0.80844382	1	nonDEG	-0.1628754	0.67760685
nonDEG	0.30124481	0.62964859	1	nonDEG	0.17434692	0.77358311
nonDEG	-0.3490272	0.77675935	1	nonDEG	0.79772427	0.5242522
nonDEG	-0.2851654	0.53070049	1	nonDEG	-0.4132483	0.28962968
nonDEG	-0.3018602	0.49114884	1	nonDEG	-0.0688119	0.85405228
nonDEG	0.71670606	0.21667056	1	nonDEG	-0.1822427	0.73810973
nonDEG	0.09826498	0.86831095	1	nonDEG	0.72344204	0.14687134
nonDEG	-0.0271129	0.95665074	1	nonDEG	0.236296	0.62000565
nonDEG	0.01285699	0.97669715	1	nonDEG	-0.1953134	0.60077373
nonDEG	0.0029972	0.9951404	1	nonDEG	-0.2099976	0.60385654
nonDEG	-0.3680208	0.5773054	1	nonDEG	0.60059581	0.36745223
nonDEG	0.43897866	0.35116387	1	nonDEG	0.01612155	0.96681805
nonDEG	0.2150492	0.65237033	1	nonDEG	0.0229563	0.95570545
nonDEG	-0.0606786	0.88988703	1	nonDEG	-0.0976359	0.79629429
nonDEG	0.24075939	0.65080221	1	nonDEG	-0.4491371	0.29479364
nonDEG	-0.0923794	0.83434267	1	nonDEG	-0.0704476	0.85203245
nonDEG	-3.1659898	0.12881825	1	nonDEG	2.13196117	0.57422161
nonDEG	0.22152126	0.71763675	1	nonDEG	0.28639997	0.59714267
nonDEG	-0.6384733	0.27645987	1	nonDEG	0.13798387	0.81775929
nonDEG	-0.4356659	0.32554703	1	nonDEG	-0.3113684	0.4751475
nonDEG	0	1	1	nonDEG	-5.13E-15	1
nonDEG	0.77831349	0.37781332	1	nonDEG	0.31052862	0.72813435
nonDEG	-0.0963827	0.82638326	1	nonDEG	-0.067415	0.85262518
nonDEG	-2.1176793	0.30678881	1	nonDEG	3.00783534	0.09614638
nonDEG	-0.462904	0.32589257	1	nonDEG	0.23994993	0.54977296
nonDEG	-0.6240978	0.22653735	1	nonDEG	0.21877145	0.67653628
nonDEG	-0.5056679	0.40595781	1	nonDEG	0.41541912	0.41764157
nonDEG	0.19905398	0.65226435	1	nonDEG	-0.0094847	0.98484024
nonDEG	0.42864452	0.41516784	1	nonDEG	0.04079584	0.92858153
nonDEG	0.19443776	0.68040907	1	nonDEG	-0.0808278	0.82661655
nonDEG	0.00699517	0.99032237	1	nonDEG	-0.2182538	0.64637767
nonDEG	0.450153	0.53105904	1	nonDEG	0.19583391	0.77303486
nonDEG	-1.4644995	0.21310931	1	nonDEG	1.64314912	0.19500074
nonDEG	3.07514276	0.02816873	1	nonDEG	-0.6067707	0.63383884
nonDEG	0.05164589	0.91799551	1	nonDEG	-0.229801	0.62319284
nonDEG	-0.4585341	0.31200956	1	nonDEG	-0.0609893	0.88697152

nonDEG	0.63600119	0.63713881	1	nonDEG	-1.747701	0.25160047
nonDEG	-0.2246435	0.66647192	1	nonDEG	-0.2447963	0.65243981
nonDEG	-0.1387377	0.85807979	1	nonDEG	0.43588415	0.53255042
nonDEG	-0.04046	0.92588505	1	nonDEG	0.03739403	0.91843723
nonDEG	0.33277799	0.85414507	1	nonDEG	0.01482794	1
nonDEG	-0.0978992	0.82719069	1	nonDEG	-1.0453934	0.01878195
nonDEG	-0.1102824	0.88600969	1	nonDEG	-0.0768955	0.91485569
nonDEG	1.56610235	0.04315196	1	nonDEG	-0.5494518	0.45441038
nonDEG	0.29638011	0.59358962	1	nonDEG	0.16481406	0.74272386
nonDEG	-0.4152835	0.35884546	1	nonDEG	-0.1064283	0.80026879
nonDEG	-0.0035891	0.99452857	1	nonDEG	-0.0085303	0.9825574
nonDEG	-0.4294605	0.30320778	1	nonDEG	0.25288413	0.48161541
nonDEG	1.82459206	0.04091394	1	nonDEG	-1.2311194	0.15010014
nonDEG	-0.262399	0.62583946	1	nonDEG	0.10556799	0.81875085
nonDEG	0.22624436	0.59188343	1	nonDEG	0.01308388	0.97207375
nonDEG	0.17128937	0.70369153	1	nonDEG	-0.2248993	0.57204118
nonDEG	0.00913072	0.98337595	1	nonDEG	0.01426021	0.969084
nonDEG	0.36208349	0.39171695	1	nonDEG	-0.2234475	0.53716822
nonDEG	0.42769081	0.4886723	1	nonDEG	0.0632343	0.91605778
nonDEG	-0.1213635	0.78781367	1	nonDEG	-0.3868182	0.37782346
nonDEG	-0.0470554	0.913911	1	nonDEG	-0.431879	0.24468052
nonDEG	-0.0525517	0.9067102	1	nonDEG	-0.3416875	0.39982058
nonDEG	-0.2451857	0.87400532	1	nonDEG	1.4767325	0.17669309
nonDEG	-1.5209467	0.38481695	1	nonDEG	0.986319	0.49579362
nonDEG	0.05136062	0.92032101	1	nonDEG	0.45436428	0.28474312
nonDEG	0.1318479	0.85174471	1	nonDEG	-0.7810232	0.30293275
nonDEG	0.03962632	0.92732796	1	nonDEG	0.18415807	0.60778633
nonDEG	0.05322494	0.92197182	1	nonDEG	0.19614691	0.6507656
nonDEG	0.97889183	0.03354243	1	nonDEG	-0.5563386	0.16964414
nonDEG	2.6759006	0.13948704	1	nonDEG	-0.690946	0.73086008
nonDEG	-0.4115713	0.73935985	1	nonDEG	0.67784693	0.57388382
nonDEG	-0.2241794	0.61317986	1	nonDEG	0.02848412	0.93979157
nonDEG	-0.1942053	0.65142668	1	nonDEG	-0.2272601	0.53283448
nonDEG	-0.4673671	0.33339585	1	nonDEG	-0.0671349	0.88915631
nonDEG	-0.2962679	0.87930098	1	nonDEG	-0.4299722	0.86470345
nonDEG	-0.5097917	0.63562215	1	nonDEG	1.61488663	0.18057073
nonDEG	-0.0698132	0.87309091	1	nonDEG	-0.2623168	0.48260721
nonDEG	0.95022624	0.4505368	1	nonDEG	1.69680912	0.1183662
nonDEG	-0.4330093	0.76345085	1	nonDEG	0.3572369	0.90535948
nonDEG	0.67722002	0.15268426	1	nonDEG	-0.0505333	0.88865536
nonDEG	-0.0220863	0.96278095	1	nonDEG	-0.0810501	0.84863828
nonDEG	-0.0844983	0.84953405	1	nonDEG	-0.7533378	0.10177877
nonDEG	-0.0414606	0.9217671	1	nonDEG	-0.0407187	0.91250468

nonDEG	0.25253416	0.66747426	1	nonDEG	-0.6989407	0.1823669
nonDEG	0.47259774	0.33124409	1	nonDEG	0.42533343	0.32497162
nonDEG	-0.1250232	0.76781063	1	nonDEG	-0.3737948	0.30781812
nonDEG	-0.2309565	0.69056846	1	nonDEG	0.74713838	0.16882663
nonDEG	0.08486399	0.84338678	1	nonDEG	0.01081996	0.9778716
nonDEG	-0.8041415	0.17839567	1	nonDEG	0.81967779	0.14194569
nonDEG	-0.229628	0.59709487	1	nonDEG	0.0620965	0.86642586
nonDEG	-0.2875005	0.65367886	1	nonDEG	-0.5881452	0.37337914
nonDEG	-0.0776351	0.85607485	1	nonDEG	-0.1754971	0.6296979
nonDEG	0.14717079	0.72704841	1	nonDEG	-0.1811539	0.61591423
nonDEG	-0.3413918	0.66636601	1	nonDEG	0.20172697	0.80766045
nonDEG	-0.0652962	0.87786327	1	nonDEG	-0.131668	0.72722559
nonDEG	-0.891689	0.81519765	1	nonDEG	-1.1151013	1
nonDEG	-0.1547982	0.77325842	1	nonDEG	0.14324902	0.76085001
nonDEG	0.10558965	0.83938454	1	nonDEG	-0.0278432	0.95651487
nonDEG	0.19973691	0.8781091	1	nonDEG	0.27211345	0.83036736
nonDEG	0.20111669	0.6436098	1	nonDEG	0.10024384	0.7862892
nonDEG	-0.077905	1	1	nonDEG	0.06531346	0.96642322
nonDEG	0.00165417	1	1	nonDEG	0.1163409	0.80482408
nonDEG	-0.6081476	0.58113658	1	nonDEG	0.65430988	0.58965557
nonDEG	-0.1115729	0.79134977	1	nonDEG	0.03619305	0.92075834
nonDEG	-0.0135963	0.97746972	1	nonDEG	-0.4570011	0.27169049
nonDEG	0.20934743	0.64026037	1	nonDEG	-0.134945	0.7207712
nonDEG	-0.8523829	0.68534149	1	nonDEG	0.75739666	0.73906724
nonDEG	-0.3201625	0.63992811	1	nonDEG	0.36583393	0.61307214
nonDEG	-0.2920397	0.50573338	1	nonDEG	0.16993801	0.67326272
nonDEG	0.4613555	0.61547946	1	nonDEG	-3.8799676	0.00171626
nonDEG	-0.0246353	0.9554782	1	nonDEG	-0.1374349	0.70191037
nonDEG	-0.3812093	0.67614065	1	nonDEG	0.11937261	0.9300378
nonDEG	0.80515361	0.15862601	1	nonDEG	-0.1943594	0.71061155
nonDEG	0.04497699	0.929434	1	nonDEG	0.08771006	0.85372758
nonDEG	-0.9482531	0.54197448	1	nonDEG	0.14355129	1
nonDEG	-0.3209661	0.46120392	1	nonDEG	-0.1388245	0.71160899
nonDEG	-0.3539938	0.59702521	1	nonDEG	-0.38838	0.52653357
nonDEG	-3.6175543	0.02848311	1	nonDEG	3.69787169	0.00080286
nonDEG	-0.1485404	0.81002809	1	nonDEG	0.31492758	0.63706008
nonDEG	-0.1553349	0.71424089	1	nonDEG	-0.1306053	0.72204758
nonDEG	0.04362175	0.95470866	1	nonDEG	0.13215465	0.86261904
nonDEG	0.33611262	0.58817487	1	nonDEG	0.00414232	1
nonDEG	-0.3007258	0.56124574	1	nonDEG	-1.1036945	0.0205213
nonDEG	-0.0225308	0.95854649	1	nonDEG	-0.242361	0.50666471
nonDEG	-0.204218	0.69826356	1	nonDEG	0.08009448	0.87438016
nonDEG	-0.3017002	0.49698238	1	nonDEG	0.129538	0.72458005

nonDEG	-0.1100423	0.79488295	1	nonDEG	-0.2397244	0.50724479
nonDEG	0.59871013	0.21166234	1	nonDEG	-0.0644627	0.88337966
nonDEG	0.53027793	0.22711384	1	nonDEG	-0.1993844	0.61281017
nonDEG	0.31041017	0.56428748	1	nonDEG	0.04648772	0.92954079
nonDEG	-0.1013779	0.82169176	1	nonDEG	0.36368235	0.31938777
nonDEG	0.94992035	0.59143279	1	nonDEG	1.52636155	0.23563501
nonDEG	0.18154842	0.71979533	1	nonDEG	0.64726065	0.12104257
nonDEG	0.5244031	0.61769421	1	nonDEG	0.13727815	0.90952497
nonDEG	0.7003758	0.61370579	1	nonDEG	-3.7397418	0.0033525
nonDEG	0.00413184	0.99266986	1	nonDEG	0.02372087	0.94817674
nonDEG	0.10317654	0.80695602	1	nonDEG	-0.0102742	0.97764577
nonDEG	2.01680682	0.05690113	1	nonDEG	-0.5089223	0.60919229
nonDEG	0.26614839	0.77596052	1	nonDEG	0.34759521	0.69776169
nonDEG	-0.2092906	0.62974017	1	nonDEG	0.22075912	0.54548707
nonDEG	-0.136843	0.75046363	1	nonDEG	-0.5112222	0.16669837
nonDEG	0.24203851	0.59903351	1	nonDEG	-0.2192412	0.57910651
nonDEG	0.0133401	0.97804297	1	nonDEG	-0.1604858	0.66984204
nonDEG	-0.2759419	0.51847539	1	nonDEG	-0.0591075	0.87342595
nonDEG	0.07227386	0.89829528	1	nonDEG	-0.1613068	0.7601861
nonDEG	1.01518587	0.18890171	1	nonDEG	-1.1144948	0.16402607
nonDEG	-2.0728756	0.0380708	1	nonDEG	1.2553756	0.34405291
nonDEG	-0.285728	0.52986629	1	nonDEG	0.17368522	0.65745208
nonDEG	-0.2986677	0.56931956	1	nonDEG	-0.5808495	0.2299355
nonDEG	0.16602703	0.74926006	1	nonDEG	-0.2019168	0.65325403
nonDEG	-0.3807945	0.38523361	1	nonDEG	-0.176101	0.65104051
nonDEG	0.05739351	0.92573384	1	nonDEG	0.2008224	0.72097737
nonDEG	0.14982588	0.73015538	1	nonDEG	0.15148208	0.68897309
nonDEG	0.35907584	0.3969923	1	nonDEG	-0.1265679	0.72591568
nonDEG	-0.3197415	0.46647306	1	nonDEG	0.10793027	0.77397494
nonDEG	-0.1622505	0.70889032	1	nonDEG	-0.1825886	0.6175699
nonDEG	-0.0201185	0.9645851	1	nonDEG	-0.347107	0.35867457
nonDEG	0.25551151	0.55647445	1	nonDEG	-0.1781982	0.62870494
nonDEG	2.63663077	0.11781009	1	nonDEG	-1.2582895	0.51962482
nonDEG	-0.3735124	0.69246205	1	nonDEG	0.62988224	0.52141862
nonDEG	0.26491211	0.54092209	1	nonDEG	0.17308372	0.64646019
nonDEG	0.06761225	0.87264965	1	nonDEG	0.09538006	0.79275879
nonDEG	-0.4066877	0.3698232	1	nonDEG	0.22481721	0.55256534
nonDEG	-0.0119514	0.97814272	1	nonDEG	0.09786721	0.7905577
nonDEG	0.23794278	0.61186501	1	nonDEG	-0.4092667	0.31730684
nonDEG	0.97399866	0.29817974	1	nonDEG	-0.4327528	0.65853779
nonDEG	-0.4757179	0.29777566	1	nonDEG	0.05095636	0.88847261
nonDEG	0.11834743	0.7980712	1	nonDEG	-0.2632976	0.50130709
nonDEG	1.07566179	0.17481619	1	nonDEG	-0.1767811	0.82351936

nonDEG	0.31692099	0.48212701	1	nonDEG	-0.0634646	0.87780838
nonDEG	0.86728183	0.18816299	1	nonDEG	0.04568756	0.94415704
nonDEG	0.39455839	0.41718584	1	nonDEG	-0.0682438	0.87148491
nonDEG	-3.4073184	0.1918694	1	nonDEG	5.4318843	0.00116193
nonDEG	-0.375286	0.41621238	1	nonDEG	0.31340018	0.45783751
nonDEG	0.56209446	0.36899783	1	nonDEG	-0.509672	0.35794368
nonDEG	0.20336619	0.79920385	1	nonDEG	-0.1973353	0.80369282
nonDEG	0.15889144	0.72523315	1	nonDEG	0.16566859	0.64912931
nonDEG	-0.0230339	0.95774904	1	nonDEG	0.03413092	0.92710589
nonDEG	-0.2938194	0.69342366	1	nonDEG	-1.309939	0.02868015
nonDEG	-0.2573326	0.57721825	1	nonDEG	-0.1180199	0.74557166
nonDEG	-0.5799222	0.32207029	1	nonDEG	-0.1087671	0.85536221
nonDEG	0.15238022	0.72122239	1	nonDEG	0.07247181	0.84307951
nonDEG	-0.1936668	0.73548071	1	nonDEG	0.22599635	0.70142816
nonDEG	-0.0690806	0.87311037	1	nonDEG	0.20505753	0.58109858
nonDEG	1.69906429	0.55753816	1	nonDEG	-1.1474651	0.82857131
nonDEG	0.28179801	0.51642444	1	nonDEG	-0.1903952	0.60150643
nonDEG	0.41802315	0.72768904	1	nonDEG	1.9395544	0.06761246
nonDEG	-0.3682572	0.39634979	1	nonDEG	-0.7975669	0.0378757
nonDEG	-0.3144186	0.70140687	1	nonDEG	0.28058137	0.77857089
nonDEG	0.35565988	0.81770293	1	nonDEG	-3.9952059	0.03198823
nonDEG	-0.7654855	0.30000817	1	nonDEG	0.33066364	0.69170528
nonDEG	0.10408602	0.80742438	1	nonDEG	-0.0531828	0.88321237
nonDEG	-0.1479838	0.73187691	1	nonDEG	-0.0373598	0.91835609
nonDEG	0.03956973	0.92908514	1	nonDEG	-0.256709	0.48463341
nonDEG	0.67203814	0.3271945	1	nonDEG	-0.1328589	0.84755709
nonDEG	-0.2548513	0.54913572	1	nonDEG	-0.0094171	0.97998063
nonDEG	5.36026322	0.0478158	1	nonDEG	-0.073142	1
nonDEG	0.39366837	0.433159	1	nonDEG	-0.3173633	0.47053917
nonDEG	-0.4523103	0.40387351	1	nonDEG	0.12069815	0.78573368
nonDEG	-0.2678221	0.57625943	1	nonDEG	-0.448626	0.32354996
nonDEG	0.07891653	0.91899215	1	nonDEG	0.20509323	0.77057907
nonDEG	0.07668654	0.86290889	1	nonDEG	-0.087336	0.82236229
nonDEG	1.03125804	0.04589684	1	nonDEG	0.01552672	0.97386018
nonDEG	0.17972324	0.68364861	1	nonDEG	-0.1305841	0.72648345
nonDEG	0.28535905	0.50611458	1	nonDEG	0.0927412	0.80111066
nonDEG	0.21590321	0.62157355	1	nonDEG	-0.1359736	0.70981094
nonDEG	-1.756463	0.31244336	1	nonDEG	0.2946853	0.94639392
nonDEG	0.88599141	0.43014215	1	nonDEG	-0.5382843	0.63820917
nonDEG	0.05031279	0.90646941	1	nonDEG	0.049455	0.89153554
nonDEG	0.23969468	0.67035737	1	nonDEG	0.12503986	0.79618475
nonDEG	0.14164269	0.74712954	1	nonDEG	-0.2568825	0.49913558
nonDEG	-0.2072068	0.67451735	1	nonDEG	-0.4565358	0.35723845

nonDEG	-0.3612145	0.41967663	1	nonDEG	0.0763885	0.85789916
nonDEG	0.07397845	0.87927033	1	nonDEG	0.20286393	0.63316604
nonDEG	-0.1931619	0.65335168	1	nonDEG	-0.1988216	0.59143843
nonDEG	-0.1463996	0.74223059	1	nonDEG	-0.460393	0.24896593
nonDEG	5.31129365	0.04517266	1	nonDEG	-1.0009636	0.54726133
nonDEG	-0.0771064	0.89791681	1	nonDEG	-0.0638786	0.89591802
nonDEG	0.0010492	0.99846537	1	nonDEG	0.04401929	0.90820244
nonDEG	0.00598143	0.9892833	1	nonDEG	-0.4513611	0.24263274
nonDEG	1.70347098	0.032276	1	nonDEG	-1.0893935	0.08645036
nonDEG	-0.0075773	0.98627007	1	nonDEG	0.0137136	0.97059236
nonDEG	-0.1077556	0.83366809	1	nonDEG	0.09370746	0.85520814
nonDEG	-0.5569461	0.24857495	1	nonDEG	0.14484943	0.75089346
nonDEG	0.09588456	0.82659144	1	nonDEG	-0.1343639	0.72868054
nonDEG	-0.9040315	0.36439647	1	nonDEG	1.02007751	0.34617773
nonDEG	0.0305759	0.94655946	1	nonDEG	-0.4636284	0.22093488
nonDEG	0.23808792	0.58359834	1	nonDEG	0.01087679	0.97627055
nonDEG	0.06673648	0.87699445	1	nonDEG	-0.029016	0.93854539
nonDEG	-0.7944338	0.2612571	1	nonDEG	-0.0194859	0.98019806
nonDEG	-0.2665244	0.56728567	1	nonDEG	-0.1501383	0.69234664
nonDEG	-3.386731	0.0121716	0.85229819	nonDEG	3.68372921	0.00870836
nonDEG	0.36367258	0.49812082	1	nonDEG	-0.3011494	0.55929129
nonDEG	-0.0351227	0.95334539	1	nonDEG	0.04642762	0.93130257
nonDEG	0.08374383	0.87096665	1	nonDEG	0.20498093	0.66241543
nonDEG	0	1	1	nonDEG	3.05432933	0.69742631
nonDEG	-0.132716	0.76147644	1	nonDEG	-0.4472055	0.24780695
nonDEG	-0.3117301	0.51927503	1	nonDEG	-1.0084313	0.03128025
nonDEG	0.16653888	0.73239324	1	nonDEG	0.13468151	0.75241799
nonDEG	0.22324748	0.61542669	1	nonDEG	-0.2400695	0.51095307
nonDEG	0.05337799	0.90023906	1	nonDEG	0.20251048	0.57919848
nonDEG	-0.6060004	0.25952543	1	nonDEG	0.04868885	0.91486819
nonDEG	0.31247676	0.45435953	1	nonDEG	0.00197543	0.99646077
nonDEG	-0.0177743	0.96856217	1	nonDEG	0.02512033	0.95059841
nonDEG	-0.4302128	0.35375542	1	nonDEG	0.38228223	0.41030853
nonDEG	-0.2499591	0.57813892	1	nonDEG	-0.0992968	0.79605513
nonDEG	-0.2212785	0.5992829	1	nonDEG	0.10042664	0.78420207
nonDEG	-0.1292535	0.76163241	1	nonDEG	0.08696663	0.81170632
nonDEG	0.01752958	0.96947347	1	nonDEG	0.25546543	0.48928568
nonDEG	-0.3344031	0.47938905	1	nonDEG	1.32555518	0.00067785
nonDEG	0.26274723	0.55166621	1	nonDEG	0.04955349	0.89277565
nonDEG	0.10921334	0.82029665	1	nonDEG	-0.4090924	0.32779787
nonDEG	0.04070245	0.92672436	1	nonDEG	0.06247433	0.86950737
nonDEG	-0.4273018	0.41013371	1	nonDEG	0.53193534	0.25170437
nonDEG	-0.3654509	0.44988047	1	nonDEG	0.19460765	0.66605026

nonDEG	0.00971333	0.98226747	1	nonDEG	-0.1450515	0.68631055
nonDEG	-0.2446324	0.58801514	1	nonDEG	-0.0024869	0.99737883
nonDEG	-0.342893	0.43295675	1	nonDEG	0.12671032	0.7329612
nonDEG	0.34617209	0.56106347	1	nonDEG	-0.1827578	0.74166305
nonDEG	0.09532809	0.82357307	1	nonDEG	0.16861813	0.64526611
nonDEG	0.03782787	0.94062377	1	nonDEG	0.31622588	0.40324534
nonDEG	-0.4278446	0.67446658	1	nonDEG	1.12258531	0.28341106
nonDEG	-0.3804704	0.55051806	1	nonDEG	0.68801002	0.27801705
nonDEG	0.56343495	0.24544629	1	nonDEG	-0.2471052	0.55215827
nonDEG	-0.3087883	0.49036292	1	nonDEG	-0.110791	0.78714745
nonDEG	0.37858556	0.56303862	1	nonDEG	-0.0102614	0.98804984
nonDEG	-1.1375308	0.47236187	1	nonDEG	2.04397168	0.15746108
nonDEG	0.7665638	0.33367406	1	nonDEG	-0.1181482	0.89236264
nonDEG	-0.1019501	0.81291176	1	nonDEG	-0.4912556	0.17757664
nonDEG	0.08371893	0.84428644	1	nonDEG	0.0356787	0.92302409
nonDEG	0.21154128	0.66442216	1	nonDEG	-0.5639025	0.15872004
nonDEG	-0.3196764	0.47891679	1	nonDEG	0.30260656	0.47227672
nonDEG	0.0579217	0.89239969	1	nonDEG	-0.3511838	0.34584835
nonDEG	0.11817019	0.78221901	1	nonDEG	-0.2676583	0.45799747
nonDEG	-0.0195726	0.97437442	1	nonDEG	0.07837841	0.88114261
nonDEG	0.639604	0.39170064	1	nonDEG	-1.2740355	0.12707364
nonDEG	0.01979428	0.96662878	1	nonDEG	-0.1425231	0.70982061
nonDEG	0.18509193	0.90834031	1	nonDEG	0.06720102	1
nonDEG	0.15830935	0.78072959	1	nonDEG	-0.6554876	0.2269435
nonDEG	0.32971263	0.64817671	1	nonDEG	0.13248954	0.85808842
nonDEG	0.11868727	0.78111758	1	nonDEG	-0.0667293	0.8537927
nonDEG	4.18083819	0.36194155	1	nonDEG	-4.3849822	0.36796373
nonDEG	1.03858258	0.04181925	1	nonDEG	-0.968713	0.0720934
nonDEG	0.14659333	0.73487177	1	nonDEG	-0.1751335	0.63325105
nonDEG	0.09676459	0.83914691	1	nonDEG	-0.2376602	0.59074193
nonDEG	0.07202414	0.88799288	1	nonDEG	-0.6089985	0.22862458
nonDEG	0.14851829	0.72886831	1	nonDEG	-0.0612892	0.8697991
nonDEG	-2.0449026	0.2198014	1	nonDEG	-0.4380113	1
nonDEG	-0.1938708	0.67975958	1	nonDEG	-0.373483	0.44132155
nonDEG	0.46202467	0.35094435	1	nonDEG	-0.4329854	0.33161428
nonDEG	-0.1229828	0.84156448	1	nonDEG	0.32652651	0.47085319
nonDEG	0.4568829	0.4041309	1	nonDEG	-0.2011018	0.68631823
nonDEG	-0.049195	0.9115543	1	nonDEG	0.73335572	0.15408077
nonDEG	0.55235328	0.3540498	1	nonDEG	-0.07206	0.90305821
nonDEG	0.13998977	0.76700015	1	nonDEG	-0.0752101	0.86086254
nonDEG	0.06424392	0.88016152	1	nonDEG	-0.2250933	0.5341105
nonDEG	-0.1651673	0.87201158	1	nonDEG	0.60685784	0.43837598
nonDEG	-0.2244569	0.60401621	1	nonDEG	0.00785766	0.98445202

nonDEG	0.27363863	0.65996175	1	nonDEG	-0.2364753	0.70062368
nonDEG	1.71730055	0.00056235	0.4474317	nonDEG	-1.2049267	0.00160782
nonDEG	-0.0163981	0.9776869	1	nonDEG	-0.2756041	0.57556674
nonDEG	-0.0786582	0.85334198	1	nonDEG	-0.0123553	0.97342082
nonDEG	-0.2452637	0.6513222	1	nonDEG	0.43867498	0.31815934
nonDEG	-0.0940383	0.95674263	1	nonDEG	1.75616221	0.26557038
nonDEG	0.71647605	0.39158166	1	nonDEG	-2.0686129	0.03957846
nonDEG	1.61552905	0.62369472	1	nonDEG	0.41410221	0.8612619
nonDEG	-0.7281108	0.49744044	1	nonDEG	1.76291628	0.0987961
nonDEG	-0.2379238	0.61007082	1	nonDEG	-0.3948566	0.29368295
nonDEG	0.00673788	0.9884825	1	nonDEG	0.18534221	0.62533321
nonDEG	-0.2842207	0.70676536	1	nonDEG	-0.0342918	0.96353101
nonDEG	-0.0735439	0.88297595	1	nonDEG	0.06506194	0.88892766
nonDEG	-0.019635	0.96498052	1	nonDEG	-0.0649691	0.87732545
nonDEG	0.30690559	0.47464912	1	nonDEG	-0.2129772	0.56006405
nonDEG	-0.3596929	0.92274571	1	nonDEG	-1.1628023	0.73450209
nonDEG	0.37843648	0.82675559	1	nonDEG	0.43915869	0.74521783
nonDEG	-0.0617651	0.95800613	1	nonDEG	0.31202148	0.70520578
nonDEG	-2.9410035	0.00201677	0.61198441	nonDEG	3.0317357	0.00414353
nonDEG	-0.096761	0.82051564	1	nonDEG	0.03202274	0.92992528
nonDEG	0.07281304	0.86442569	1	nonDEG	-0.005129	0.9889948
nonDEG	-0.2376597	0.58280348	1	nonDEG	-0.2160427	0.54868401
nonDEG	0.15183469	0.72728311	1	nonDEG	-0.2613316	0.48853283
nonDEG	0.21111294	0.63643356	1	nonDEG	-0.0316835	0.93859522
nonDEG	0.40126466	0.36388628	1	nonDEG	-0.060296	0.87709305
nonDEG	-0.1074277	0.84128822	1	nonDEG	-0.2357154	0.66670103
nonDEG	-0.0917119	0.90444178	1	nonDEG	-0.0689297	0.92177642
nonDEG	0.07238801	0.88688637	1	nonDEG	0.12108361	0.78722246
nonDEG	-0.1800747	0.72290386	1	nonDEG	0.36397954	0.41078643
nonDEG	1.86665857	0.01757353	0.93380915	nonDEG	-1.0904692	0.15572006
nonDEG	-0.0649589	0.88226164	1	nonDEG	-0.1669933	0.64743286
nonDEG	-0.6025719	0.42565261	1	nonDEG	1.25065955	0.14265832
nonDEG	-0.0550553	0.89644338	1	nonDEG	-0.7803368	0.03214353
nonDEG	-0.2648257	0.60887612	1	nonDEG	0.4932622	0.33254675
nonDEG	-0.2507842	0.59624288	1	nonDEG	-0.2948229	0.51928637
nonDEG	-0.022277	0.96445333	1	nonDEG	-0.2447893	0.52796691
nonDEG	-0.0272323	0.94977008	1	nonDEG	-0.0822471	0.82108413
nonDEG	-0.2315646	0.79034902	1	nonDEG	0.16510906	0.87682018
nonDEG	-0.0182269	0.96774643	1	nonDEG	-0.276982	0.47720343
nonDEG	-0.0432844	0.92429349	1	nonDEG	-0.3973248	0.35574005
nonDEG	0.14574508	0.78126009	1	nonDEG	-0.4024538	0.34733488
nonDEG	0.04229489	0.92145336	1	nonDEG	-0.1510972	0.68389231
nonDEG	0.44945754	0.51509749	1	nonDEG	-0.0018432	1

nonDEG	1.00196128	0.1202157	1	nonDEG	-0.2810742	0.62659188
nonDEG	0.11589018	0.78693712	1	nonDEG	-0.2723467	0.46078356
nonDEG	-0.275523	0.55234742	1	nonDEG	0.28289054	0.5369462
nonDEG	-0.2101347	0.65913867	1	nonDEG	-0.1542889	0.73285547
nonDEG	-0.050192	0.91276694	1	nonDEG	-0.4597478	0.2608939
nonDEG	-0.2719653	0.70143251	1	nonDEG	-0.3878481	0.61608034
nonDEG	-0.43733	0.32806507	1	nonDEG	0.05167529	0.89946227
nonDEG	0.10776889	0.83354912	1	nonDEG	-0.2905944	0.48825933
nonDEG	-0.2088098	0.63521517	1	nonDEG	-0.117095	0.76526697
nonDEG	-0.0496667	0.90723296	1	nonDEG	-0.0277711	0.93973578
nonDEG	-0.2431916	0.66106183	1	nonDEG	-0.3328753	0.58355791
nonDEG	0.11262282	0.79901982	1	nonDEG	0.05780223	0.88328595
nonDEG	-0.1713702	0.70846741	1	nonDEG	0.12679544	0.75970541
nonDEG	0.51816632	0.4523496	1	nonDEG	-0.474017	0.49063841
nonDEG	4.9622284	0.08509275	1	nonDEG	-0.6489669	0.70064867
nonDEG	0.4214342	0.35667674	1	nonDEG	-0.2811076	0.46550556
nonDEG	-2.7960518	0.05644177	1	nonDEG	3.89524449	0.00404792
nonDEG	0.26512511	1	1	nonDEG	0.02830174	1
nonDEG	-0.1788167	0.68273462	1	nonDEG	-0.0331589	0.93631532
nonDEG	0.03648677	0.9412472	1	nonDEG	-0.1827846	0.68661693
nonDEG	-0.9580069	0.36392462	1	nonDEG	0.54102145	0.60965698
nonDEG	-0.0775756	0.86711023	1	nonDEG	0.37928462	0.37398652
nonDEG	0.36465855	0.47039713	1	nonDEG	-0.011411	0.97965619
nonDEG	1.45624985	0.04259527	1	nonDEG	-0.0470403	0.94912138
nonDEG	-0.1500144	0.77860168	1	nonDEG	0.26944462	0.59680097
nonDEG	0.34758477	0.82074677	1	nonDEG	0.81418623	0.66325498
nonDEG	0.18052094	0.73985244	1	nonDEG	-0.0340715	0.94682358
nonDEG	-0.1109704	0.8526719	1	nonDEG	0.26529736	0.63637724
nonDEG	0.25858045	0.60461488	1	nonDEG	-0.484148	0.26730553
nonDEG	1.09872315	0.11968491	1	nonDEG	-0.6535106	0.34642859
nonDEG	0.4342203	0.44909317	1	nonDEG	-0.6870357	0.22025195
nonDEG	0.07151726	0.87462577	1	nonDEG	-0.4668194	0.24926068
nonDEG	-0.0922784	0.88041825	1	nonDEG	-0.3990851	0.49208524
nonDEG	-0.3152853	0.71636848	1	nonDEG	1.24254845	0.10701579
nonDEG	-0.8597246	0.2918704	1	nonDEG	-0.6584128	0.49593408
nonDEG	0.62430436	0.38695727	1	nonDEG	-0.4911825	0.47684598
nonDEG	0.54747793	0.19844559	1	nonDEG	-0.3475807	0.38103556
nonDEG	-0.1127127	0.81586305	1	nonDEG	-0.1410529	0.73795205
nonDEG	-0.2575874	0.54068225	1	nonDEG	-0.2080098	0.58299008
nonDEG	0.93890415	0.03273374	1	nonDEG	-0.6495458	0.07675004
nonDEG	0.3023592	0.53247214	1	nonDEG	-0.3502574	0.38673959
nonDEG	-0.418212	0.3746623	1	nonDEG	0.15132519	0.7297933
nonDEG	0.55433502	0.46879033	1	nonDEG	0.48298839	0.44944504

nonDEG	0.17410167	0.69940251	1	nonDEG	-0.2295568	0.55124816
nonDEG	-0.772859	1	1	nonDEG	1.04290274	0.77154061
nonDEG	0.03770992	0.93109842	1	nonDEG	-0.3077631	0.42824402
nonDEG	0.21099449	0.62307972	1	nonDEG	-0.3303248	0.38194266
nonDEG	2.69070235	0.24142724	1	nonDEG	0.79087326	0.73031483
nonDEG	0.11221903	0.789639	1	nonDEG	-0.1672451	0.65098181
nonDEG	-0.1790865	0.71729128	1	nonDEG	0.09980371	0.82200223
nonDEG	-0.4374565	0.33965986	1	nonDEG	0.51311791	0.16270631
nonDEG	0.24896474	0.5821575	1	nonDEG	0.39101337	0.28527643
nonDEG	-0.0949381	0.8443104	1	nonDEG	-0.1077987	0.80663671
nonDEG	-0.0958445	0.82076981	1	nonDEG	0.17064622	0.64214797
nonDEG	-0.2642316	0.55900156	1	nonDEG	0.17565858	0.67802041
nonDEG	0.86518479	0.05861653	1	nonDEG	-0.3163568	0.43112346
nonDEG	0.22548378	0.5918685	1	nonDEG	-0.0953982	0.79302245
nonDEG	-2.2188978	0.04978173	1	nonDEG	1.33176669	0.37833453
nonDEG	-0.0732909	0.87023997	1	nonDEG	0.22601484	0.55803242
nonDEG	-0.3077024	0.69049379	1	nonDEG	0.5300823	0.46619285
nonDEG	-0.276386	0.5396721	1	nonDEG	0.13118502	0.73143914
nonDEG	0.0034964	0.99398352	1	nonDEG	-0.2232738	0.54172123
nonDEG	-0.0670105	0.87404384	1	nonDEG	0.0999999	0.78172983
nonDEG	-0.1173859	0.79709387	1	nonDEG	-0.1863719	0.68577462
nonDEG	0.10303065	0.81291808	1	nonDEG	-0.1705022	0.64767716
nonDEG	-0.3792758	0.43336865	1	nonDEG	0.05154495	0.91651634
nonDEG	-0.3481818	0.45220702	1	nonDEG	0.46095436	0.26197644
nonDEG	1.87715299	0.01712034	0.93079586	nonDEG	-0.8280282	0.2783032
nonDEG	0.0845494	0.84223974	1	nonDEG	-0.3879673	0.28170519
nonDEG	0.13155246	0.80308559	1	nonDEG	-0.9280729	0.04049323
nonDEG	0.10507248	0.89605106	1	nonDEG	0.55817403	0.42373323
nonDEG	0.18292675	0.71160891	1	nonDEG	-0.1049061	0.81216401
nonDEG	0.2671217	0.75737682	1	nonDEG	0.23514134	0.8070776
nonDEG	-0.1195201	0.78648991	1	nonDEG	-0.0959911	0.80177043
nonDEG	-0.0167149	0.9709817	1	nonDEG	0.17278168	0.65490981
nonDEG	-0.0455436	0.94640738	1	nonDEG	0.18474499	0.76113793
nonDEG	-0.2051164	0.74412572	1	nonDEG	-1.0075775	0.13587823
nonDEG	0.03611778	1	1	nonDEG	-1.706496	0.33695057
nonDEG	-0.200512	0.64414561	1	nonDEG	0.23193328	0.52865568
nonDEG	-0.0256164	0.9525173	1	nonDEG	0.11395337	0.75279374
nonDEG	0.20367096	0.65762036	1	nonDEG	0.00120069	0.99765131
nonDEG	-0.3715319	0.52511422	1	nonDEG	-0.1134819	0.81573566
nonDEG	-0.1085585	1	1	nonDEG	-0.5239782	1
nonDEG	-0.397196	0.78959076	1	nonDEG	1.36142814	0.30333366
nonDEG	0.50223208	0.32643527	1	nonDEG	-0.1247731	0.77806678
nonDEG	-0.0010724	0.99822959	1	nonDEG	-0.3198179	0.40510782

nonDEG	0.23372697	0.6283977	1	nonDEG	-0.0815323	0.84866172
nonDEG	2.10375891	0.14711005	1	nonDEG	0.8439514	0.53727441
nonDEG	0.1544222	0.73528957	1	nonDEG	0.13297196	0.7268923
nonDEG	0.517951	0.57325091	1	nonDEG	-0.3984992	0.68618933
nonDEG	-0.4760063	0.32860107	1	nonDEG	0.40772171	0.40715768
nonDEG	-0.6554247	0.23427815	1	nonDEG	0.77624576	0.10810328
nonDEG	0.35185251	0.42653593	1	nonDEG	-0.1527362	0.68386796
nonDEG	-0.0575709	0.91099858	1	nonDEG	-0.4857887	0.31482285
nonDEG	0.31305224	0.47338766	1	nonDEG	0.09332879	0.80676666
nonDEG	0.60962981	0.2264295	1	nonDEG	0.28283498	0.57671322
nonDEG	-0.0891739	0.84546857	1	nonDEG	-0.5534116	0.13675472
nonDEG	0.85376859	0.05212566	1	nonDEG	-0.2692856	0.4772977
nonDEG	-0.0605277	0.8853969	1	nonDEG	-0.0503333	0.89026939
nonDEG	-0.0673876	0.9173617	1	nonDEG	-0.7896719	0.21768465
nonDEG	0.09140424	0.83287283	1	nonDEG	0.34042432	0.35784104
nonDEG	0.1943583	0.73142373	1	nonDEG	0.41776091	0.41261872
nonDEG	-0.5505993	0.29899552	1	nonDEG	0.0133018	0.9770272
nonDEG	-0.3602584	0.4932526	1	nonDEG	-0.1474114	0.72818519
nonDEG	-0.3931645	0.46442426	1	nonDEG	-0.0765299	0.88846492
nonDEG	0.40304916	0.35233745	1	nonDEG	-0.0518434	0.88744993
nonDEG	-0.1496271	0.72557786	1	nonDEG	-0.4384067	0.23182892
nonDEG	-0.1171229	0.79421922	1	nonDEG	-0.2100793	0.57700121
nonDEG	0.07298113	0.87346103	1	nonDEG	-1.168617	0.00528135
nonDEG	0.32648823	0.4620308	1	nonDEG	-0.131907	0.72631845
nonDEG	-0.3811476	0.3988163	1	nonDEG	0.02101586	0.95616842
nonDEG	-0.2606528	0.54699236	1	nonDEG	-0.0324127	0.92884897
nonDEG	-0.0635547	0.91249961	1	nonDEG	0.05947796	0.90441693
nonDEG	0.20938255	0.70234537	1	nonDEG	-0.7390287	0.10724034
nonDEG	0.00455731	0.99223213	1	nonDEG	-0.2769789	0.44059022
nonDEG	0.07755438	0.86365227	1	nonDEG	-0.2981898	0.42065915
nonDEG	1.24819387	0.01232854	0.85229819	nonDEG	-1.0196262	0.0325422
nonDEG	0.21035294	0.61533614	1	nonDEG	-0.4016324	0.26353664
nonDEG	1.98123991	0.04200702	1	nonDEG	-0.401383	0.67232599
nonDEG	0.33366997	0.58131123	1	nonDEG	-0.182272	0.76312453
nonDEG	-1.1693929	0.30648439	1	nonDEG	2.65882706	0.0097456
nonDEG	0.35091422	0.5432025	1	nonDEG	-0.3598993	0.4077173
nonDEG	-0.1948904	0.67422645	1	nonDEG	0.24051312	0.57410249
nonDEG	-0.0638818	0.88257511	1	nonDEG	-0.0436357	0.90522844
nonDEG	0.91494153	0.10001807	1	nonDEG	-0.6272644	0.19451971
nonDEG	-0.1671356	0.70833824	1	nonDEG	0.10991468	0.77940667
nonDEG	-0.0560737	0.89740457	1	nonDEG	0.21667327	0.55447854
nonDEG	1.05085846	0.02605605	1	nonDEG	-0.5399356	0.18497906
nonDEG	0.43938535	0.37136256	1	nonDEG	-0.3658379	0.31005264

nonDEG	-0.3349766	0.55000697	1	nonDEG	0.9885912	0.05045087
nonDEG	-0.3408741	0.43920689	1	nonDEG	0.11878862	0.75683124
nonDEG	2.22720163	0.01441068	0.88752292	nonDEG	-0.3357801	0.68649034
nonDEG	-0.1626053	0.69971647	1	nonDEG	-0.0042688	0.99114687
nonDEG	-0.0550577	0.90164073	1	nonDEG	-0.1538124	0.69608328
nonDEG	-0.0801274	0.8535833	1	nonDEG	-0.2127453	0.57688969
nonDEG	-0.1338852	0.76198338	1	nonDEG	-0.1479617	0.70640295
nonDEG	0.02840719	0.94667571	1	nonDEG	-0.2457025	0.49352629
nonDEG	-0.1642933	0.70120021	1	nonDEG	-0.4628959	0.21890986
nonDEG	-0.287619	0.5557771	1	nonDEG	0.68381832	0.12165882
nonDEG	0.00634695	0.98848477	1	nonDEG	0.30815928	0.40083497
nonDEG	-0.2067518	0.63758227	1	nonDEG	0.04290839	0.90630377
nonDEG	-0.0766553	0.9534679	1	nonDEG	-0.2831989	0.77985361
nonDEG	-0.0235739	0.9581562	1	nonDEG	-0.2556277	0.53004945
nonDEG	-0.0436271	0.91839507	1	nonDEG	-0.0679543	0.85193783
nonDEG	-0.1518064	0.7287379	1	nonDEG	-0.0628181	0.86682896
nonDEG	-0.0822317	0.86505375	1	nonDEG	-0.6303258	0.20143941
nonDEG	0.24005919	0.57633619	1	nonDEG	-0.0924354	0.79914904
nonDEG	1.06378211	0.20293461	1	nonDEG	-1.0307614	0.20971686
nonDEG	0.03696317	0.9324671	1	nonDEG	-0.3674883	0.33038569
nonDEG	0.05684378	0.90649693	1	nonDEG	-0.0561553	0.89655491
nonDEG	-0.1567381	0.72779546	1	nonDEG	-0.0663137	0.87423346
nonDEG	0.19354766	0.66666849	1	nonDEG	-0.4892775	0.21313148
nonDEG	-0.1405334	1	1	nonDEG	2.22208801	0.18218255
nonDEG	-0.1021586	0.81964313	1	nonDEG	-0.1942626	0.62856628
nonDEG	-0.1679024	0.69313541	1	nonDEG	9.71E-05	1
nonDEG	-0.4801414	0.2768459	1	nonDEG	0.17086207	0.65619143
nonDEG	0.0454596	0.91963366	1	nonDEG	-0.2354222	0.5424599
nonDEG	-0.0363851	0.93730706	1	nonDEG	-0.1906577	0.61598449
nonDEG	1.23838844	0.13135338	1	nonDEG	0.32454227	0.66080874
nonDEG	0.40639158	0.3755883	1	nonDEG	-0.483095	0.23305921
nonDEG	-0.1233743	0.77182311	1	nonDEG	0.00417976	0.99119599
nonDEG	0.00690247	0.98740859	1	nonDEG	-0.045676	0.89975173
nonDEG	-0.0643279	0.92070916	1	nonDEG	0.09407743	0.88725667
nonDEG	-4.4182871	0.18857526	1	nonDEG	5.60390919	0.1499188
nonDEG	0.29234564	0.50016201	1	nonDEG	-0.0763918	0.83574236
nonDEG	0.33306175	0.42834922	1	nonDEG	-0.2557	0.48225705
nonDEG	0.09064219	0.83186346	1	nonDEG	0.00378629	0.99215563
nonDEG	-0.2465371	0.62358497	1	nonDEG	-0.0098624	0.98458949
nonDEG	0.1095025	0.80274237	1	nonDEG	-0.3390236	0.3944999
nonDEG	-0.0626792	0.88382264	1	nonDEG	-0.5376117	0.15932656
nonDEG	-1.6692364	0.07586345	1	nonDEG	1.72129766	0.03566652
nonDEG	-0.0614446	0.9047716	1	nonDEG	0.19164798	0.66665189

nonDEG	-0.3262	0.51018251	1	nonDEG	-0.1948286	0.62764288
nonDEG	-1.0690172	0.08504541	1	nonDEG	0.22295597	0.63397219
nonDEG	0.46284507	0.36857602	1	nonDEG	-0.2562774	0.59481205
nonDEG	-0.1590719	0.71096628	1	nonDEG	0.08894945	0.80611752
nonDEG	0.06591253	0.94542629	1	nonDEG	-1.0223259	0.24123889
nonDEG	-0.3514942	0.42533834	1	nonDEG	-0.205568	0.60297664
nonDEG	-0.4323268	0.34955435	1	nonDEG	-0.3132862	0.50866482
nonDEG	-0.2025364	0.67597757	1	nonDEG	-0.1457458	0.77315265
nonDEG	-0.0110916	0.98663197	1	nonDEG	0.04973772	0.92150069
nonDEG	0.35074831	0.46303612	1	nonDEG	-0.0830046	0.82102661
nonDEG	-0.2650617	0.55606075	1	nonDEG	0.15154933	0.713468
nonDEG	-0.0899884	0.84491875	1	nonDEG	0.20254173	0.60634951
nonDEG	-0.1708078	0.69903187	1	nonDEG	-0.1430133	0.7206197
nonDEG	0.40689617	0.37831212	1	nonDEG	-0.2403282	0.52488117
nonDEG	0.23021395	0.60504988	1	nonDEG	-0.1829267	0.6132393
nonDEG	0.17525419	0.71178175	1	nonDEG	0.16368769	0.70248649
nonDEG	-0.984222	0.30344276	1	nonDEG	-0.6184649	0.68729389
nonDEG	2.23430362	1	1	nonDEG	3.05718384	0.29474799
nonDEG	0.18112342	0.87020572	1	nonDEG	0.85274268	0.37329098
nonDEG	-1.5562764	0.37541447	1	nonDEG	1.3874893	0.59742567
nonDEG	-1.2031123	0.1571209	1	nonDEG	1.38670651	0.18124122
nonDEG	0.68757485	0.38936157	1	nonDEG	-0.6387791	0.44868422
nonDEG	0.13308343	0.75995007	1	nonDEG	-0.0965192	0.79061076
nonDEG	-0.0339531	0.93671802	1	nonDEG	0.08618156	0.81112021
nonDEG	0.10742032	0.81959399	1	nonDEG	-0.016069	0.96913197
nonDEG	-0.2406963	0.57156235	1	nonDEG	0.04955208	0.89176264
nonDEG	0.53659719	0.52567324	1	nonDEG	0.62633279	0.43487024
nonDEG	-0.0351619	0.9339973	1	nonDEG	-0.0607992	0.86756721
nonDEG	0.12734283	0.76715258	1	nonDEG	-0.0240536	0.94980963
nonDEG	-0.2267706	0.89207766	1	nonDEG	0.36040225	0.90525749
nonDEG	0	1	1	nonDEG	3.05429873	0.6964888
nonDEG	0.40262138	0.72832452	1	nonDEG	-0.8396441	0.53668301
nonDEG	1.23475328	0.01233889	0.85229819	nonDEG	-0.8416329	0.05401091
nonDEG	0.30988256	0.47480299	1	nonDEG	-0.4239393	0.27095919
nonDEG	-0.4103043	0.52640185	1	nonDEG	0.25343974	0.69097411
nonDEG	-0.0847544	0.85099327	1	nonDEG	0.20645796	0.61041463
nonDEG	-0.2624879	0.81682222	1	nonDEG	0.48289599	0.6299467
nonDEG	0.1948406	0.6557603	1	nonDEG	-0.1626058	0.67041042
nonDEG	-0.0924964	0.83096911	1	nonDEG	0.43668138	0.25131264
nonDEG	0.09732218	0.81660228	1	nonDEG	-0.2803865	0.43425244
nonDEG	-0.0401883	0.92685979	1	nonDEG	-0.0643519	0.86619026
nonDEG	1.86988315	0.06891892	1	nonDEG	-0.1504536	0.87908713
nonDEG	-0.809923	0.40743425	1	nonDEG	0.52759416	0.51586916

nonDEG	-0.1136903	0.79153619	1	nonDEG	0.25967703	0.47803514
nonDEG	0.82346465	0.30004986	1	nonDEG	-0.9327829	0.32103772
nonDEG	-0.0098285	0.98243927	1	nonDEG	-0.4252773	0.25270612
nonDEG	-0.3118417	0.57262142	1	nonDEG	0.65157542	0.18187914
nonDEG	-4.1307028	0.26628878	1	nonDEG	4.52142667	0.35516787
nonDEG	3.27299479	0.02362064	1	nonDEG	-0.3519249	0.82341042
nonDEG	-0.2315452	0.80608123	1	nonDEG	-0.7106287	0.52035545
nonDEG	-0.2635857	0.55289982	1	nonDEG	0.11981283	0.76956268
nonDEG	0.0574012	0.96595657	1	nonDEG	0.01337265	1
nonDEG	0.04633698	0.91487302	1	nonDEG	-0.0140053	0.97061274
nonDEG	-0.5663651	0.6646783	1	nonDEG	1.21533333	0.31286723
nonDEG	-0.0498533	0.90870065	1	nonDEG	-0.0616399	0.86748155
nonDEG	-0.1360698	0.75245799	1	nonDEG	0.0557259	0.8794219
nonDEG	0.05842594	0.89072675	1	nonDEG	-0.0920817	0.80416721
nonDEG	-0.0472491	0.91483613	1	nonDEG	-0.5874091	0.15425813
nonDEG	0.22697195	0.62240795	1	nonDEG	-0.3364949	0.38537333
nonDEG	-0.072066	0.86719059	1	nonDEG	0.07866816	0.82950621
nonDEG	-0.2005173	0.89690137	1	nonDEG	0.50330901	0.63553496
nonDEG	0.57363553	0.56634185	1	nonDEG	-0.1602511	0.87253001
nonDEG	0.05473166	0.91159562	1	nonDEG	0.51354003	0.23023149
nonDEG	-0.2456581	0.56911433	1	nonDEG	-0.1986454	0.60663591
nonDEG	-0.1104946	0.80586976	1	nonDEG	-0.3028441	0.40324699
nonDEG	-0.063919	0.90324854	1	nonDEG	-0.4065061	0.41820443
nonDEG	0.09030403	0.84727579	1	nonDEG	-0.3476863	0.38346102
nonDEG	-0.3132742	0.58297617	1	nonDEG	-0.0606251	0.91645478
nonDEG	0.11827611	0.7972231	1	nonDEG	0.18835572	0.6549646
nonDEG	0.18514624	0.84806624	1	nonDEG	0.02700956	0.98646988
nonDEG	0.72353374	0.09188714	1	nonDEG	-0.4176997	0.25989065
nonDEG	-0.4359243	0.36144652	1	nonDEG	-0.0405435	0.92770329
nonDEG	-0.0758462	0.85759486	1	nonDEG	0.21436401	0.55445167
nonDEG	0.17584633	0.70048997	1	nonDEG	-0.2702593	0.474923
nonDEG	0.04729557	0.96046453	1	nonDEG	0.92670672	0.14164798
nonDEG	0.04636446	0.92814083	1	nonDEG	-0.4646925	0.28597447
nonDEG	-0.4089116	0.74812438	1	nonDEG	0.37242109	0.77069728
nonDEG	-0.2678933	0.5831629	1	nonDEG	0.02365942	0.96015945
nonDEG	0.40748192	0.4355763	1	nonDEG	0.1227321	0.80304802
nonDEG	-0.3112596	0.46932414	1	nonDEG	-0.7964845	0.04031247
nonDEG	0.72611028	0.13887968	1	nonDEG	0.22584221	0.57040795
nonDEG	-0.5435616	0.22586244	1	nonDEG	0.62632305	0.09391349
nonDEG	0.14073677	0.74933502	1	nonDEG	-0.2979118	0.42138416
nonDEG	0.38607978	0.37543647	1	nonDEG	-0.2278982	0.54275851
nonDEG	-0.117902	0.7871027	1	nonDEG	-0.2168681	0.55084866
nonDEG	0.56079344	0.51032595	1	nonDEG	-0.1618685	0.84854891

nonDEG	-0.3626229	0.7173829	1	nonDEG	-0.5237634	0.63955566
nonDEG	-0.3137631	0.70589252	1	nonDEG	-0.1649103	0.84399536
nonDEG	0.0916237	0.91737591	1	nonDEG	1.07950676	0.12254762
nonDEG	0.18958019	0.82288084	1	nonDEG	0.77859045	0.27024235
nonDEG	0.3418517	0.43357841	1	nonDEG	-0.1654516	0.65230819
nonDEG	0.15460518	0.79508009	1	nonDEG	-0.9257571	0.04979494
nonDEG	0.51684322	0.23289919	1	nonDEG	-0.2116894	0.56450092
nonDEG	-0.3745308	0.40174676	1	nonDEG	-0.4259488	0.29114947
nonDEG	-0.3324833	0.46417763	1	nonDEG	0.22684387	0.56221237
nonDEG	-0.0673715	0.87549588	1	nonDEG	0.10000279	0.78340602
nonDEG	-0.0106218	0.98078995	1	nonDEG	0.04811613	0.89623997
nonDEG	0.4516864	0.85179092	1	nonDEG	-5.9373	0.12321724
nonDEG	0.32096045	0.52642046	1	nonDEG	-0.384162	0.36730551
nonDEG	0.23176919	1	1	nonDEG	-0.2153056	1
nonDEG	-0.2495152	0.67668861	1	nonDEG	-0.2074429	0.7433086
nonDEG	0.28821516	0.56976888	1	nonDEG	-0.3101328	0.46656115
nonDEG	-0.2458511	0.58081558	1	nonDEG	0.24912831	0.50462371
nonDEG	0.33755473	0.46953689	1	nonDEG	-0.2182346	0.5568355
nonDEG	0.07320843	0.86683266	1	nonDEG	0.00627643	0.98767697
nonDEG	-0.0510543	0.93558402	1	nonDEG	0.27981178	0.60897652
nonDEG	0.8962722	0.19609069	1	nonDEG	-0.6472313	0.34079946
nonDEG	-0.3412731	0.47107882	1	nonDEG	-0.3183114	0.47254871
nonDEG	0.24775843	0.64751797	1	nonDEG	-0.9844262	0.08918533
nonDEG	-0.3450281	0.53675738	1	nonDEG	-0.1026551	0.82601865
nonDEG	-0.1392695	0.74475113	1	nonDEG	-0.0886599	0.80698904
nonDEG	0.73491672	0.08934917	1	nonDEG	-0.4858637	0.20858913
nonDEG	0.02619578	0.98091308	1	nonDEG	-0.3946653	0.6191891
nonDEG	0.43559229	0.3210337	1	nonDEG	-0.2417313	0.50247222
nonDEG	-0.0508141	0.90464338	1	nonDEG	-0.0606336	0.87048464
nonDEG	0.56865479	0.62096328	1	nonDEG	-0.5862233	0.62971565
nonDEG	0.01480893	0.97272004	1	nonDEG	-0.2275471	0.53498641
nonDEG	-0.4363189	0.34612791	1	nonDEG	-0.4985599	0.22228543
nonDEG	-0.147553	0.73219251	1	nonDEG	-0.0889543	0.81336451
nonDEG	-0.0465626	0.91379561	1	nonDEG	-0.0794987	0.82864519
nonDEG	-0.6035853	0.22452916	1	nonDEG	-0.2416526	0.6181091
nonDEG	0.1047934	0.82966642	1	nonDEG	0.08792733	0.83608181
nonDEG	0.09522662	0.89607507	1	nonDEG	0.13025182	0.84212083
nonDEG	-0.2870063	0.50294428	1	nonDEG	0.31109639	0.3921874
nonDEG	0.39390573	0.53185362	1	nonDEG	0.56110637	0.36790569
nonDEG	-0.1066901	0.83677871	1	nonDEG	-0.0513454	0.91151753
nonDEG	0.43995647	0.53705825	1	nonDEG	0.12063705	0.868055
nonDEG	-0.2212633	0.67834637	1	nonDEG	0.22552672	0.66374347
nonDEG	-0.0448511	0.91832631	1	nonDEG	0.17853437	0.62465893

nonDEG	0.1238301	0.79971977	1	nonDEG	0.25322872	0.55978476
nonDEG	-0.1865578	0.66304038	1	nonDEG	0.24901537	0.50923446
nonDEG	-0.2022278	0.6592158	1	nonDEG	-0.2971193	0.44152325
nonDEG	0.22519246	0.71623774	1	nonDEG	-0.3096221	0.70852328
nonDEG	-0.5306336	0.49172818	1	nonDEG	-0.2492769	0.74879979
nonDEG	0.30017942	0.4860222	1	nonDEG	-0.0796662	0.82732648
nonDEG	-0.9533971	0.27677425	1	nonDEG	0.94396771	0.31271181
nonDEG	-0.4649809	0.40385981	1	nonDEG	0.01809801	0.97008559
nonDEG	-0.2141922	0.61820208	1	nonDEG	0.1030336	0.79469697
nonDEG	-0.5233771	0.21478189	1	nonDEG	0.25512494	0.49342535
nonDEG	-0.3715461	0.44316548	1	nonDEG	-0.0220894	0.96822813
nonDEG	0.0948308	0.87910986	1	nonDEG	0.13799922	0.81088767
nonDEG	0.00841694	0.98494034	1	nonDEG	-0.102631	0.77933203
nonDEG	0.04808957	0.91824582	1	nonDEG	-0.1445294	0.69423936
nonDEG	-0.3539054	0.57697703	1	nonDEG	0.18045581	0.77265363
nonDEG	-0.1337514	0.76634288	1	nonDEG	0.52365386	0.17709605
nonDEG	0.22921682	0.7450183	1	nonDEG	0.23052899	0.72100559
nonDEG	0.20392254	0.66333106	1	nonDEG	-0.4107857	0.28891116
nonDEG	-0.0364947	0.93579016	1	nonDEG	0.38182401	0.34984448
nonDEG	0.06211573	0.88740305	1	nonDEG	0.3393696	0.36082918
nonDEG	0.17106992	0.700853	1	nonDEG	-0.09508	0.80710016
nonDEG	0.05379207	0.89875208	1	nonDEG	-0.6443388	0.07567736
nonDEG	0.21261805	0.6227208	1	nonDEG	-0.1923422	0.59818288
nonDEG	-0.1550107	0.77679421	1	nonDEG	0.16425598	0.74556826
nonDEG	0.16096598	0.75795485	1	nonDEG	-0.6470979	0.19191967
nonDEG	0.00584484	0.98953834	1	nonDEG	-0.1644193	0.64985327
nonDEG	0.19326789	0.66542969	1	nonDEG	-0.2363206	0.5192814
nonDEG	0.213845	0.6968022	1	nonDEG	-0.302772	0.5158901
nonDEG	-1.1228881	0.1246669	1	nonDEG	0.7785051	0.33078018
nonDEG	0.15482011	0.7161729	1	nonDEG	-0.1286776	0.72094599
nonDEG	-0.2267975	0.77114468	1	nonDEG	0.2712115	0.74008506
nonDEG	0.43400963	0.53168836	1	nonDEG	0.14213689	0.84691329
nonDEG	0.00971869	0.98438876	1	nonDEG	-0.0108504	0.97985443
nonDEG	1.07019554	0.08868211	1	nonDEG	-0.5744274	0.336524
nonDEG	0.00873552	0.98394366	1	nonDEG	-0.1085088	0.76460166
nonDEG	-0.0981256	0.83600288	1	nonDEG	0.61234446	0.13783101
nonDEG	-0.1402208	0.77015622	1	nonDEG	0.1854259	0.66690943
nonDEG	0.05576958	0.93113895	1	nonDEG	-0.7028705	0.26876382
nonDEG	-0.1298691	0.77636574	1	nonDEG	0.20976956	0.58622932
nonDEG	-0.3502426	0.7365952	1	nonDEG	0.40245727	0.71642405
nonDEG	-0.2903246	0.51397627	1	nonDEG	-0.1330577	0.73187756
nonDEG	0.02029722	0.97934512	1	nonDEG	0.01248711	0.98759859
nonDEG	2.74595359	0.05151669	1	nonDEG	-0.7142122	0.6086164

nonDEG	-0.6597059	0.34051375	1	nonDEG	-0.1780286	0.741581
nonDEG	-0.0912614	0.91998491	1	nonDEG	0.98267552	0.19928001
nonDEG	0.74318685	0.76168324	1	nonDEG	0.51769014	0.86339416
nonDEG	1.1746448	0.34943765	1	nonDEG	0.79148833	0.51343633
nonDEG	0.30941919	0.55590717	1	nonDEG	-0.1435027	0.76416299
nonDEG	0.31708986	0.4684123	1	nonDEG	-0.0520593	0.890473
nonDEG	0.20709441	0.82059329	1	nonDEG	0.24519631	0.77140612
nonDEG	1.30507352	0.23946919	1	nonDEG	0.03250939	0.97047349
nonDEG	-3.1301136	0.64234073	1	nonDEG	5.19225799	0.20599923
nonDEG	0.26986951	0.62320196	1	nonDEG	0.02441681	0.96057594
nonDEG	0.77022897	0.15108441	1	nonDEG	-0.0615153	0.88840438
nonDEG	-0.0576082	0.89361549	1	nonDEG	-0.0048515	0.98950923
nonDEG	-0.5909067	0.2684401	1	nonDEG	-0.06762	0.87924147
nonDEG	0.21834581	0.61039502	1	nonDEG	-0.0217595	0.95300565
nonDEG	-0.4396004	0.4264105	1	nonDEG	0.44303159	0.38321241
nonDEG	-1.0805192	0.21810749	1	nonDEG	0.63717342	0.50111513
nonDEG	1.46540867	0.43783718	1	nonDEG	0.11381526	1
nonDEG	-0.4243789	0.50100164	1	nonDEG	-0.6325401	0.29803602
nonDEG	-0.3540039	0.446115	1	nonDEG	-0.1840629	0.62782822
nonDEG	-0.4259519	0.78522389	1	nonDEG	0.53544185	0.68831609
nonDEG	0.06640715	0.89125152	1	nonDEG	-0.1949467	0.63291738
nonDEG	-0.025151	0.96603112	1	nonDEG	-0.143018	0.79068766
nonDEG	0.1969811	0.70061525	1	nonDEG	-0.5586	0.25390526
nonDEG	0.41728056	0.32619585	1	nonDEG	0.22606908	0.55671364
nonDEG	-0.0980174	0.81930314	1	nonDEG	0.07477904	0.84345258
nonDEG	0.55135112	0.24218887	1	nonDEG	-0.3845878	0.32405893
nonDEG	0.22771949	0.72043493	1	nonDEG	-0.2811006	0.655083
nonDEG	-0.4636036	0.42924812	1	nonDEG	0.70137913	0.21961485
nonDEG	0.10609694	0.81264315	1	nonDEG	-1.1095466	0.01198134
nonDEG	0.24120387	0.60834098	1	nonDEG	-0.2441366	0.55085988
nonDEG	0.14455536	0.73529709	1	nonDEG	-0.1364912	0.70558811
nonDEG	-0.0675206	0.87624683	1	nonDEG	0.08856783	0.8098703
nonDEG	0.01775101	0.970891	1	nonDEG	0.06677375	0.87165602
nonDEG	-0.01459	0.97540502	1	nonDEG	-0.3194094	0.40922531
nonDEG	-0.1136184	0.8255931	1	nonDEG	-0.0898244	0.83173329
nonDEG	-0.0928809	0.82911074	1	nonDEG	-0.1217113	0.75076818
nonDEG	0.39568357	0.57662315	1	nonDEG	0.00834376	0.99309669
nonDEG	0.63428641	0.32177687	1	nonDEG	-0.9442501	0.12812658
nonDEG	0.5534419	0.51794655	1	nonDEG	0.04001885	0.97791762
nonDEG	0.04193299	0.9316267	1	nonDEG	-0.0860919	0.84242131
nonDEG	0.35153644	0.49594478	1	nonDEG	-0.6644646	0.13936629
nonDEG	-0.0847408	0.84738727	1	nonDEG	-0.2233399	0.5560296
nonDEG	0.15443027	0.87637734	1	nonDEG	-0.3908313	0.68089599

nonDEG	-0.0240228	0.95611178	1	nonDEG	0.09431068	0.79825629
nonDEG	-0.1508252	0.73176109	1	nonDEG	-0.1448661	0.72324048
nonDEG	-0.3817529	0.40040138	1	nonDEG	0.56534513	0.13898046
nonDEG	-0.1103969	0.82034845	1	nonDEG	-0.1665248	0.71411446
nonDEG	0.13186157	0.78284401	1	nonDEG	-0.2824293	0.48796796
nonDEG	0	1	1	nonDEG	3.96662902	0.43041039
nonDEG	0.05939813	0.89207888	1	nonDEG	-0.3273883	0.40997746
nonDEG	0.57123346	0.19300594	1	nonDEG	-0.4822164	0.2268846
nonDEG	-0.3338213	0.61651104	1	nonDEG	-0.3406865	0.63507873
nonDEG	-0.1503723	0.73450662	1	nonDEG	0.14451897	0.69325451
nonDEG	0.73531571	0.10118381	1	nonDEG	0.08898269	0.81433811
nonDEG	-0.5171295	0.25740466	1	nonDEG	0.22133656	0.58234626
nonDEG	-0.3268749	0.44656319	1	nonDEG	0.22635879	0.55774547
nonDEG	0.01337628	0.97938245	1	nonDEG	-1.2398903	0.00879202
nonDEG	0.59114778	0.57282765	1	nonDEG	0.43556692	0.67021715
nonDEG	0.03011446	0.96042794	1	nonDEG	0.81304472	0.10211098
nonDEG	0.09035302	0.83630183	1	nonDEG	-0.291098	0.417925
nonDEG	0.21027416	0.6351554	1	nonDEG	-0.087038	0.82645819
nonDEG	-0.2018277	0.6397801	1	nonDEG	0.14674783	0.69430963
nonDEG	-0.3476527	0.55169546	1	nonDEG	0.2584163	0.64936841
nonDEG	-0.0083244	0.98597492	1	nonDEG	-0.0733473	0.84617231
nonDEG	-0.3744389	0.38847172	1	nonDEG	0.24134499	0.51807246
nonDEG	0.13381813	0.75666222	1	nonDEG	-0.0963863	0.79239512
nonDEG	0.09558283	0.82177282	1	nonDEG	-0.0107764	0.97767573
nonDEG	0.77864832	0.53117747	1	nonDEG	1.83891333	0.09388286
nonDEG	-0.0063864	0.98896937	1	nonDEG	-0.089104	0.80431097
nonDEG	-0.1913193	0.65909994	1	nonDEG	-0.1164169	0.75342377
nonDEG	-0.2102091	0.68222999	1	nonDEG	-0.2329881	0.63718938
nonDEG	1.21200309	0.0223762	1	nonDEG	-0.3489806	0.40281165
nonDEG	-0.2143431	0.62605901	1	nonDEG	-0.3682647	0.32312421
nonDEG	-0.1454274	0.73907234	1	nonDEG	-0.2186931	0.54652553
nonDEG	0.33712624	0.60482936	1	nonDEG	0.03527209	0.95728531
nonDEG	0.16683154	0.71788826	1	nonDEG	-0.4306296	0.252582
nonDEG	0.13767852	0.78756722	1	nonDEG	-0.2371815	0.57268712
nonDEG	-0.284819	0.51804545	1	nonDEG	0.2927438	0.41994115
nonDEG	0.3442442	0.41304313	1	nonDEG	-0.1236791	0.73424004
nonDEG	0.66155465	0.80807405	1	nonDEG	1.88468634	0.28110908
nonDEG	-0.1644534	0.69779409	1	nonDEG	-0.0361042	0.92262772
nonDEG	-0.0521533	0.90432779	1	nonDEG	-0.1611586	0.6602833
nonDEG	0.70565965	0.58586234	1	nonDEG	-1.5352929	0.27903924
nonDEG	0.63788505	0.19609661	1	nonDEG	0.10400187	0.81350025
nonDEG	0.00713805	0.98703371	1	nonDEG	0.03776999	0.91688195
nonDEG	-0.0038973	0.9934284	1	nonDEG	-0.4481692	0.2113948

nonDEG	0.96670223	0.02354459	1	nonDEG	0.04295546	0.90612487
nonDEG	4.74765954	0.2403385	1	nonDEG	-2.7373011	0.45962104
nonDEG	-0.6390905	0.23050651	1	nonDEG	0.78576621	0.14186351
nonDEG	-0.239176	0.5731968	1	nonDEG	0.05956999	0.87180834
nonDEG	-0.0916416	1	1	nonDEG	1.99939273	0.22916345
nonDEG	-0.1781403	0.68906271	1	nonDEG	0.14471456	0.71040297
nonDEG	0.00676582	0.98782665	1	nonDEG	0.07142531	0.84360513
nonDEG	-0.5638221	0.31829981	1	nonDEG	0.50216023	0.35577506
nonDEG	-0.3801683	0.38733541	1	nonDEG	-0.0676557	0.86826936
nonDEG	0.11427964	0.87239689	1	nonDEG	0.59879535	0.37593577
nonDEG	-0.1608211	0.7206523	1	nonDEG	-0.4910288	0.246657
nonDEG	0.15552432	0.72103791	1	nonDEG	-0.0583113	0.87888886
nonDEG	0.07051649	0.87192027	1	nonDEG	0.13712919	0.71440879
nonDEG	-0.320123	0.45579529	1	nonDEG	0.19113243	0.61895739
nonDEG	-0.0742011	1	1	nonDEG	2.62457638	0.17427319
nonDEG	-0.3917463	0.44148168	1	nonDEG	-0.0469804	0.92868032
nonDEG	-0.3596098	0.401113	1	nonDEG	0.03008251	0.9351458
nonDEG	-1.4235873	0.66570824	1	nonDEG	1.82127173	0.41754716
nonDEG	-0.4975397	0.56724416	1	nonDEG	0.24044512	0.81746911
nonDEG	-0.5677943	0.70846024	1	nonDEG	0.11773734	0.96415028
nonDEG	0.92597833	0.04309663	1	nonDEG	-0.0063788	0.9886417
nonDEG	0.08539854	0.84440826	1	nonDEG	0.13033991	0.72044448
nonDEG	0.28242472	0.50114178	1	nonDEG	0.15697904	0.66982897
nonDEG	0.14344605	0.77865152	1	nonDEG	-0.4549921	0.30509983
nonDEG	0.4458213	0.83783757	1	nonDEG	0.69160484	0.67125887
nonDEG	-0.0598574	0.88883545	1	nonDEG	-0.3426672	0.35370997
nonDEG	0.36636531	0.41352446	1	nonDEG	0.04524751	0.89978503
nonDEG	-0.8914249	0.64258478	1	nonDEG	-2.1845264	0.48187267
nonDEG	0.56615644	0.41023856	1	nonDEG	0.24470477	0.69656051
nonDEG	-0.4456611	0.37517911	1	nonDEG	-0.2772288	0.53733243
nonDEG	-0.0196481	0.96378797	1	nonDEG	0.4260262	0.23958441
nonDEG	-0.1787833	0.67060489	1	nonDEG	-0.0288527	0.94498117
nonDEG	-0.1676102	0.7179034	1	nonDEG	-0.3011381	0.43081446
nonDEG	-1.4421107	0.25216298	1	nonDEG	0.48471077	0.81457221
nonDEG	-0.0198335	0.96313813	1	nonDEG	-0.1554908	0.66765658
nonDEG	-0.5179548	0.27131301	1	nonDEG	-0.0060058	0.98760858
nonDEG	0.37677545	0.42354979	1	nonDEG	-0.1929455	0.60459887
nonDEG	0.03827737	0.93422508	1	nonDEG	0.40642251	0.28502293
nonDEG	0.24650493	0.60357698	1	nonDEG	-0.4048797	0.34121364
nonDEG	-0.251673	0.55927018	1	nonDEG	-0.1292527	0.72314761
nonDEG	0.07715052	0.8784883	1	nonDEG	-0.7883656	0.05615965
nonDEG	0.07419279	0.86533594	1	nonDEG	-0.135989	0.72059105
nonDEG	-0.1444789	0.74026948	1	nonDEG	0.03424795	0.92499883

nonDEG	-0.286223	0.5125904	1	nonDEG	0.08756523	0.8360934
nonDEG	0.35774022	0.56989122	1	nonDEG	-0.0982178	0.88080914
nonDEG	-0.2135401	0.61544296	1	nonDEG	0.06052568	0.87637912
nonDEG	0	1	1	nonDEG	5.23392321	0.20510509
nonDEG	-0.2706886	0.73840641	1	nonDEG	-0.3545847	0.5946515
nonDEG	0.76442179	0.23428436	1	nonDEG	-0.225462	0.69045389
nonDEG	-0.4157543	0.33946629	1	nonDEG	0.24230168	0.52328322
nonDEG	-0.0015619	0.99753741	1	nonDEG	0.01184421	0.97455846
nonDEG	-0.3209181	0.46138065	1	nonDEG	0.37030729	0.32316018
nonDEG	0.5378998	0.39591695	1	nonDEG	0.2269501	0.72747239
nonDEG	-0.4956839	0.2464806	1	nonDEG	0.02601635	0.94390014
nonDEG	-0.2051586	0.62553545	1	nonDEG	0.09263632	0.79765594
nonDEG	0.07708215	0.85513133	1	nonDEG	0.0635922	0.86163838
nonDEG	0.58008299	0.19537444	1	nonDEG	0.15149522	0.67809376
nonDEG	0.27890246	0.51058714	1	nonDEG	0.15077703	0.67370723
nonDEG	-0.150453	0.72523686	1	nonDEG	-0.3840279	0.31733922
nonDEG	-0.0821524	0.84702049	1	nonDEG	-0.2866419	0.43013089
nonDEG	-0.3810643	0.49370289	1	nonDEG	-0.186988	0.70242905
nonDEG	1.21670095	0.25141353	1	nonDEG	-1.6399328	0.2327146
nonDEG	0.44574987	0.40874199	1	nonDEG	0.35432864	0.41876265
nonDEG	-2.2713292	0.33247951	1	nonDEG	-3.3286948	0.63892947
nonDEG	-0.0374373	0.94333332	1	nonDEG	-0.1019584	0.83428676
nonDEG	0.41787995	0.65000761	1	nonDEG	0.21919501	0.78928684
nonDEG	-0.4250754	0.34076147	1	nonDEG	0.07577595	0.84341536
nonDEG	-0.0386774	0.9322896	1	nonDEG	-0.0494576	0.90186868
nonDEG	0.11821036	0.79722441	1	nonDEG	-0.0596228	0.87550867
nonDEG	0.08127814	0.87019606	1	nonDEG	-0.7147532	0.0922987
nonDEG	0.09748614	0.81967139	1	nonDEG	-0.4194639	0.25126315
nonDEG	-0.1535889	0.72102617	1	nonDEG	0.24934437	0.50472778
nonDEG	-0.0362967	0.93896504	1	nonDEG	-0.4909002	0.25919082
nonDEG	-0.0360882	0.93872263	1	nonDEG	0.40635653	0.30655111
nonDEG	0.19086491	0.65978533	1	nonDEG	-0.3857515	0.2976341
nonDEG	0.0975661	0.82051544	1	nonDEG	0.08550103	0.81535528
nonDEG	-0.109122	0.80543958	1	nonDEG	0.12898652	0.74920953
nonDEG	-0.0413398	0.9224847	1	nonDEG	-0.1978414	0.58771739
nonDEG	-0.2594677	0.538728	1	nonDEG	-0.441493	0.24359813
nonDEG	-0.0390759	0.92956239	1	nonDEG	-0.3564014	0.34899019
nonDEG	-0.9071146	0.49624335	1	nonDEG	-0.2873234	0.8515929
nonDEG	0.10529195	0.80957579	1	nonDEG	0.10775865	0.76903635
nonDEG	3.54628788	0.15805826	1	nonDEG	0.39044812	0.86108955
nonDEG	-0.6329826	0.33604315	1	nonDEG	0.90013161	0.14578151
nonDEG	0.92506816	0.32852025	1	nonDEG	-0.7392252	0.43942877
nonDEG	0.34670577	0.64449097	1	nonDEG	0.46560772	0.51675219

nonDEG	0.4380115	0.72326264	1	nonDEG	-0.5048627	0.70033591
nonDEG	0.25051121	0.68238225	1	nonDEG	0.03269641	0.94465671
nonDEG	-0.5387167	0.30877994	1	nonDEG	-0.2822609	0.60817366
nonDEG	-0.5300854	0.26215571	1	nonDEG	0.05947847	0.88112579
nonDEG	0.48296215	0.28301856	1	nonDEG	-0.0916656	0.80612952
nonDEG	0.75512976	1	1	nonDEG	2.48106437	0.35883743
nonDEG	-0.1233291	0.77381245	1	nonDEG	-0.2592713	0.48729706
nonDEG	0.04646934	0.92460621	1	nonDEG	0.13274917	0.76517831
nonDEG	-0.9959625	0.11779878	1	nonDEG	1.56982989	0.00516789
nonDEG	-0.305394	0.48688131	1	nonDEG	0.1975098	0.62921022
nonDEG	-0.4196372	0.32650591	1	nonDEG	0.17594889	0.64279855
nonDEG	1.51426557	0.17390972	1	nonDEG	0.77513471	0.43269495
nonDEG	0.63211835	0.28699785	1	nonDEG	-0.2964785	0.62297269
nonDEG	-0.1295705	0.7784433	1	nonDEG	-0.8128126	0.05432499
nonDEG	0.01277283	0.97729073	1	nonDEG	-0.0390378	0.91766344
nonDEG	0.16944242	0.69235665	1	nonDEG	-0.1306253	0.71851134
nonDEG	0.09370784	0.92573044	1	nonDEG	0.98600066	0.33129614
nonDEG	0.23965877	0.63793697	1	nonDEG	-0.0076156	0.98875359
nonDEG	0.08467784	0.91088986	1	nonDEG	0.68008539	0.15877693
nonDEG	-0.0408437	0.92593483	1	nonDEG	0.02838433	0.93824943
nonDEG	-0.2607167	0.5563398	1	nonDEG	-0.1105836	0.76137426
nonDEG	0.39605138	0.39085147	1	nonDEG	0.23345485	0.59794239
nonDEG	-0.1157068	0.78634204	1	nonDEG	0.11271328	0.75816215
nonDEG	5.4709461	0.11674006	1	nonDEG	-0.7566031	0.8071621
nonDEG	-0.054648	0.90542268	1	nonDEG	0.31650554	0.4369988
nonDEG	-0.5706088	0.3629527	1	nonDEG	-0.0749717	0.8958277
nonDEG	-0.309023	0.46520068	1	nonDEG	-0.0144276	0.97044857
nonDEG	0.12007878	0.78541626	1	nonDEG	0.11572646	0.76159294
nonDEG	-2.0757495	0.17826744	1	nonDEG	1.0088746	0.68807749
nonDEG	0.09459119	0.83815126	1	nonDEG	-0.4013629	0.31503875
nonDEG	3.94176992	0.08169354	1	nonDEG	-1.6688093	0.47294731
nonDEG	1.08267397	0.33039724	1	nonDEG	-0.988301	0.51808769
nonDEG	0.16100428	0.73052041	1	nonDEG	-0.1400209	0.72058829
nonDEG	-0.104465	0.87874354	1	nonDEG	0.50448176	0.42492702
nonDEG	0.17393479	0.75396475	1	nonDEG	0.15488371	0.76796502
nonDEG	-0.0801042	0.85438126	1	nonDEG	0.28430386	0.46024558
nonDEG	0.00278427	0.99598264	1	nonDEG	-0.1332279	0.71983632
nonDEG	-0.3883724	0.38944443	1	nonDEG	0.29906737	0.41584611
nonDEG	-0.0442918	0.91758957	1	nonDEG	-0.2145482	0.56264848
nonDEG	0.26146196	0.74149063	1	nonDEG	-0.0097459	0.99546421
nonDEG	-0.1272441	0.77037787	1	nonDEG	-0.5201844	0.21005317
nonDEG	-1.9347319	0.22752288	1	nonDEG	1.75201812	0.32723771
nonDEG	0.26496335	0.64021616	1	nonDEG	-0.3862104	0.48059702

nonDEG	0.39140606	0.75057761	1	nonDEG	0.03452578	1
nonDEG	0.05824849	0.9074433	1	nonDEG	-0.0077466	0.98747965
nonDEG	-0.0224247	0.95840885	1	nonDEG	0.14513582	0.6887362
nonDEG	0.74768913	0.34966905	1	nonDEG	0.54794383	0.47216526
nonDEG	-0.1090745	0.80683331	1	nonDEG	-0.280501	0.54682594
nonDEG	0.49529248	0.63508957	1	nonDEG	-0.5194911	0.62065352
nonDEG	0.33558946	0.44933788	1	nonDEG	-0.0187527	0.96487658
nonDEG	-0.1521731	0.72489789	1	nonDEG	0.35751517	0.34858274
nonDEG	0.61553264	0.17260348	1	nonDEG	-0.0392034	0.9209445
nonDEG	-0.0243579	0.955148	1	nonDEG	-0.3973706	0.27208519
nonDEG	1.05016006	0.25627251	1	nonDEG	-0.9210138	0.30059396
nonDEG	0.52673783	0.2270712	1	nonDEG	-0.4082117	0.28196702
nonDEG	0.19280805	0.64609836	1	nonDEG	-0.1074831	0.76492589
nonDEG	-0.1247403	0.79492137	1	nonDEG	-0.3623309	0.36614347
nonDEG	1.34548637	0.37527862	1	nonDEG	0.22231274	0.89887512
nonDEG	-0.0209446	1	1	nonDEG	-6.0719476	0.03471074
nonDEG	0.33228952	0.44854621	1	nonDEG	0.00970033	0.97922549
nonDEG	-0.2008319	0.63447543	1	nonDEG	0.15231415	0.67304169
nonDEG	0.17671761	0.69537167	1	nonDEG	-0.117518	0.75578532
nonDEG	0.32883078	0.46081934	1	nonDEG	-0.4663973	0.21786392
nonDEG	0.21755417	0.61452402	1	nonDEG	-0.1039601	0.77614312
nonDEG	0.43508113	0.33048523	1	nonDEG	-0.1839316	0.63238071
nonDEG	-0.1995901	0.89154613	1	nonDEG	0.47209896	0.69708917
nonDEG	-0.008868	0.99202315	1	nonDEG	-0.7962967	0.20688026
nonDEG	-0.7799799	0.48752668	1	nonDEG	0.2040033	0.89800917
nonDEG	0.08514576	0.84612991	1	nonDEG	-0.0597623	0.87739163
nonDEG	-1.1746223	0.83900643	1	nonDEG	-3.2220295	0.6806353
nonDEG	-0.2238676	0.72593137	1	nonDEG	-0.375332	0.56554381
nonDEG	-0.2437042	0.6403597	1	nonDEG	-0.499934	0.31595399
nonDEG	0.42455727	0.34979426	1	nonDEG	-0.1262771	0.75314815
nonDEG	-0.4118068	0.33199907	1	nonDEG	0.02492106	0.94596584
nonDEG	0.15539555	0.9647079	1	nonDEG	-1.0059927	0.59202306
nonDEG	0.14934627	0.74568531	1	nonDEG	0.14052814	0.75003615
nonDEG	-0.400122	0.39571527	1	nonDEG	-0.2251139	0.56034725
nonDEG	-0.2670062	0.56422988	1	nonDEG	0.32739289	0.44312441
nonDEG	-0.144563	0.73310364	1	nonDEG	0.13894444	0.70542054
nonDEG	-4.0287365	0.35693411	1	nonDEG	3.05444315	0.70095946
nonDEG	0.49005971	0.35351558	1	nonDEG	0.59874751	0.22071008
nonDEG	0.96881521	0.26198516	1	nonDEG	-0.6136153	0.51113088
nonDEG	0.2751084	0.53269316	1	nonDEG	0.05791081	0.87941558
nonDEG	0.09264501	0.8560682	1	nonDEG	-0.4744319	0.26408386
nonDEG	-0.2457924	0.62358744	1	nonDEG	0.00949922	0.98478906
nonDEG	-0.13589	0.76325979	1	nonDEG	-0.2597658	0.50454125

nonDEG	-0.4205515	0.48815575	1	nonDEG	0.70070128	0.14756031
nonDEG	0.01989183	0.96863409	1	nonDEG	-0.4289878	0.3783541
nonDEG	-0.0803356	0.85842395	1	nonDEG	-0.0670581	0.86813386
nonDEG	-0.3064191	0.49447753	1	nonDEG	0.11978299	0.75019214
nonDEG	0.326031	0.79891796	1	nonDEG	1.04842408	0.36833726
nonDEG	0.09551959	0.82469855	1	nonDEG	0.07733339	0.83316529
nonDEG	-0.1617652	1	1	nonDEG	-1.7932053	0.44740775
nonDEG	0.03215681	0.94104382	1	nonDEG	0.02000899	0.95681265
nonDEG	0.62712897	0.16467675	1	nonDEG	-0.1933787	0.59964816
nonDEG	-0.3709256	0.48829526	1	nonDEG	-0.2018754	0.70242366
nonDEG	-0.1447711	0.89946795	1	nonDEG	0.41015547	0.69664409
nonDEG	-0.2665069	0.5412043	1	nonDEG	-0.0638689	0.87007273
nonDEG	0.01516248	0.97201425	1	nonDEG	0.02970084	0.93494838
nonDEG	-1.9336589	0.06866224	1	nonDEG	1.80097852	0.12409195
nonDEG	-0.2567567	0.56601603	1	nonDEG	-0.5939138	0.1250719
nonDEG	-0.4048039	0.58118525	1	nonDEG	-0.0953375	0.88753126
nonDEG	0.28726876	0.49334776	1	nonDEG	0.01313789	0.97206456
nonDEG	-3.6493908	0.46220827	1	nonDEG	7.36348292	0.00978549
nonDEG	-0.1323178	0.88192097	1	nonDEG	-0.2523115	0.81101657
nonDEG	-0.1079772	0.80325727	1	nonDEG	0.107924	0.76978735
nonDEG	0.65661382	0.58406162	1	nonDEG	0.26863918	0.85506156
nonDEG	0.04443056	0.91719579	1	nonDEG	-0.1121522	0.75525362
nonDEG	-0.0333652	0.93704052	1	nonDEG	0.04284746	0.90523135
nonDEG	-1.0042901	0.57601288	1	nonDEG	-0.1842733	1
nonDEG	-0.1288804	0.75944101	1	nonDEG	0.16660515	0.64974655
nonDEG	-0.0443999	1	1	nonDEG	0.16805291	0.92400779
nonDEG	0.28060221	0.51497049	1	nonDEG	-0.0125857	0.9725617
nonDEG	-0.236367	0.65949237	1	nonDEG	0.29742698	0.54597826
nonDEG	0.39063117	0.47948837	1	nonDEG	0.09358371	0.85381616
nonDEG	-0.676391	0.29191825	1	nonDEG	-0.2117937	0.74109852
nonDEG	-0.0880037	0.90183831	1	nonDEG	0.31444294	0.6154369
nonDEG	0.18339315	0.67349562	1	nonDEG	-0.2552119	0.49140222
nonDEG	-0.0895634	0.8424724	1	nonDEG	0.19966896	0.60419119
nonDEG	0.15239341	0.73077417	1	nonDEG	-0.1262512	0.72994197
nonDEG	0.09505261	0.85568626	1	nonDEG	-0.0364237	0.94550574
nonDEG	0.29871082	0.48957212	1	nonDEG	-0.1026024	0.78469802
nonDEG	0.09568798	0.83184985	1	nonDEG	-0.1516333	0.67677945
nonDEG	0.57709297	0.37280794	1	nonDEG	-0.6846641	0.28223183
nonDEG	-0.1032808	0.84870078	1	nonDEG	0.28599569	0.5553602
nonDEG	-0.1224099	0.77126706	1	nonDEG	0.09143934	0.79978793
nonDEG	-0.1116012	0.79792212	1	nonDEG	-0.1242438	0.73519145
nonDEG	-0.0838871	0.91987623	1	nonDEG	0.65682553	0.43931905
nonDEG	-0.4333293	0.46923044	1	nonDEG	0.48104178	0.41306576

nonDEG	0.81069795	0.33451985	1	nonDEG	-1.9425511	0.01391568
nonDEG	0.02919734	1	1	nonDEG	0.80456098	0.54928304
nonDEG	0.25807142	0.55319962	1	nonDEG	0.20499999	0.59017446
nonDEG	-0.0650653	0.88706023	1	nonDEG	0.32138061	0.45676893
nonDEG	0.38041984	0.42677594	1	nonDEG	-0.2036659	0.63631481
nonDEG	0.18659774	0.6724678	1	nonDEG	0.16682078	0.66233334
nonDEG	0.21793195	0.62882974	1	nonDEG	-0.0874745	0.81489321
nonDEG	0.77510057	0.37455312	1	nonDEG	-0.883807	0.30651199
nonDEG	-0.1864842	0.66477287	1	nonDEG	0.4656883	0.20879694
nonDEG	0.66546414	0.43454614	1	nonDEG	-0.2705764	0.76925888
nonDEG	0.03272663	0.97705098	1	nonDEG	0.9333104	0.13697452
nonDEG	-0.0123012	0.97812966	1	nonDEG	0.09677746	0.79491889
nonDEG	0.21150725	0.72032708	1	nonDEG	-0.7032087	0.24011585
nonDEG	1.59670812	0.00112973	0.51315728	nonDEG	-1.1498615	0.00179684
nonDEG	0.24252197	0.57081044	1	nonDEG	-0.0450958	0.90291827
nonDEG	-0.1281769	0.76283231	1	nonDEG	0.3244575	0.37311779
nonDEG	-0.2998268	0.51925407	1	nonDEG	-0.0986865	0.8117976
nonDEG	-0.2093587	0.62532198	1	nonDEG	0.04829705	0.8948401
nonDEG	-0.3404002	0.62908078	1	nonDEG	0.41920289	0.55112193
nonDEG	0.10650622	1	1	nonDEG	-5.2862389	0.11372411
nonDEG	-0.0200997	0.96526711	1	nonDEG	-0.001279	0.99915243
nonDEG	0.21672232	0.854555	1	nonDEG	0.53931572	0.58195375
nonDEG	-0.2701326	0.54636131	1	nonDEG	0.13765302	0.72333999
nonDEG	0.65277659	0.50118554	1	nonDEG	-0.0615571	0.96464252
nonDEG	0.02510169	0.95700892	1	nonDEG	-0.1940573	0.63207005
nonDEG	-0.1213758	0.78878305	1	nonDEG	-0.1827928	0.66732747
nonDEG	-0.6043193	0.55595092	1	nonDEG	-0.8027529	0.38747432
nonDEG	-0.2178583	0.62480742	1	nonDEG	0.08484363	0.81394165
nonDEG	0.37049979	0.67074117	1	nonDEG	0.50413708	0.53860921
nonDEG	0.26167912	0.54507854	1	nonDEG	-0.0105718	0.97783021
nonDEG	0.08793437	0.85822503	1	nonDEG	0.3872222	0.36192028
nonDEG	0.1500246	0.72641088	1	nonDEG	-0.1178781	0.74375171
nonDEG	-0.1020922	0.81165238	1	nonDEG	0.14191765	0.70021448
nonDEG	-0.1995515	0.75695411	1	nonDEG	0.83969917	0.21131461
nonDEG	-0.4233359	0.56281181	1	nonDEG	0.81784743	0.24707793
nonDEG	-0.0578316	0.89274271	1	nonDEG	-0.14211	0.69674448
nonDEG	0.37795912	0.6234326	1	nonDEG	0.5282754	0.46605705
nonDEG	0.29820141	1	1	nonDEG	2.61125983	0.19145191
nonDEG	-0.4450906	0.3991746	1	nonDEG	0.09112509	0.85949817
nonDEG	0.2363715	0.60200696	1	nonDEG	-0.243403	0.52186861
nonDEG	-0.0872644	0.84495958	1	nonDEG	-0.2858462	0.48062935
nonDEG	-0.1830873	0.66119281	1	nonDEG	-0.1423807	0.69483536
nonDEG	-0.4814259	0.2674555	1	nonDEG	-0.350743	0.35560606

nonDEG	-0.0888283	0.84852936	1	nonDEG	-0.2514712	0.54203486
nonDEG	-0.1282517	0.80264835	1	nonDEG	0.23680343	0.61690504
nonDEG	0.15916902	0.71845295	1	nonDEG	-0.1380144	0.71033416
nonDEG	0.04545353	0.91807665	1	nonDEG	-0.2373384	0.52515004
nonDEG	-2.3005478	1	1	nonDEG	2.21598318	1
nonDEG	0.02251203	0.97094743	1	nonDEG	-0.3184977	0.5580809
nonDEG	0.46335115	0.29570058	1	nonDEG	0.01840073	0.96161853
nonDEG	0.16410553	0.69876663	1	nonDEG	-0.0637629	0.86006961
nonDEG	0.15066262	0.74884484	1	nonDEG	0.01012473	0.98063158
nonDEG	0.22228868	0.78608928	1	nonDEG	-0.3462416	0.70621621
nonDEG	0.35483851	0.48053011	1	nonDEG	-0.2767019	0.53214543
nonDEG	-4.130708	0.26650553	1	nonDEG	5.08601919	0.23144326
nonDEG	-0.0722042	0.87285476	1	nonDEG	-0.0205415	0.95607738
nonDEG	-0.0637236	0.89351527	1	nonDEG	-0.1007779	0.81481969
nonDEG	0.36900997	0.54096924	1	nonDEG	0.24425694	0.66880089
nonDEG	0.31684518	0.58689862	1	nonDEG	-0.1701874	0.7441801
nonDEG	-2.0911262	0.51151918	1	nonDEG	4.75409514	0.01677665
nonDEG	-1.2977839	0.07872765	1	nonDEG	1.32969798	0.07179431
nonDEG	-0.392576	0.3554691	1	nonDEG	0.04632702	0.89853356
nonDEG	0.28063492	0.78198754	1	nonDEG	0.50289492	0.60174825
nonDEG	0.59441965	0.39700349	1	nonDEG	-0.291082	0.66925828
nonDEG	-0.2779231	0.525275	1	nonDEG	0.07282072	0.84423058
nonDEG	-0.7692506	0.17761814	1	nonDEG	0.18044972	0.73455589
nonDEG	-0.1728213	0.82635638	1	nonDEG	0.60892538	0.47474749
nonDEG	-0.1459826	0.86296526	1	nonDEG	0.00034272	1
nonDEG	-0.0194388	1	1	nonDEG	0.65986398	0.51790577
nonDEG	-0.3333709	0.43314318	1	nonDEG	0.01016472	0.97853327
nonDEG	-0.0821057	0.845458	1	nonDEG	0.11524471	0.74961472
nonDEG	-0.048986	0.95144655	1	nonDEG	0.89850777	0.16308134
nonDEG	0.20658091	0.70885215	1	nonDEG	-0.8640798	0.12529327
nonDEG	-0.2073557	0.62421787	1	nonDEG	-0.0636946	0.86169054
nonDEG	-0.2642688	0.53622979	1	nonDEG	-0.001622	0.99722211
nonDEG	0.29413296	0.73710171	1	nonDEG	-0.8537405	0.41125855
nonDEG	-0.1398556	0.76649407	1	nonDEG	0.06033093	0.88085453
nonDEG	0.0299031	0.95539007	1	nonDEG	-0.0609614	0.89982478
nonDEG	-0.2295545	0.61028435	1	nonDEG	-0.4624883	0.34163412
nonDEG	-0.3446979	0.41025242	1	nonDEG	-0.0467262	0.90195348
nonDEG	0.30438643	0.67136654	1	nonDEG	0.31096498	0.5579983
nonDEG	-0.1621404	0.78477341	1	nonDEG	-0.1917194	0.74310381
nonDEG	-0.1937122	0.66264695	1	nonDEG	-0.0609384	0.87240614
nonDEG	0.1321667	0.76948009	1	nonDEG	0.02093664	0.95634723
nonDEG	-0.0581474	0.91416228	1	nonDEG	0.10847073	0.82884435
nonDEG	-0.0368851	0.93316296	1	nonDEG	-0.1316708	0.72110759

nonDEG	-2.5502119	0.03326749	1	nonDEG	2.67975399	0.03461751
nonDEG	-0.4196993	0.78381522	1	nonDEG	0.22778163	0.85120473
nonDEG	-1.4642274	0.5033955	1	nonDEG	1.47460638	0.58010967
nonDEG	-0.1505701	0.80276318	1	nonDEG	-0.8638185	0.14088591
nonDEG	-1.1882511	0.30306872	1	nonDEG	1.13855545	0.40250034
nonDEG	0.05779212	0.90377607	1	nonDEG	-0.5561854	0.17285672
nonDEG	-0.2652465	0.62747024	1	nonDEG	0.98809974	0.03947463
nonDEG	-0.0249758	0.95771971	1	nonDEG	-0.406425	0.29011852
nonDEG	-0.0614434	0.88993765	1	nonDEG	-0.1642086	0.6696148
nonDEG	-0.2033436	0.63207824	1	nonDEG	0.05721128	0.87748213
nonDEG	-0.1539073	0.72430629	1	nonDEG	0.36851068	0.35280798
nonDEG	0.02823824	0.94809066	1	nonDEG	0.19812043	0.59226608
nonDEG	-0.1788265	0.69168663	1	nonDEG	0.12988026	0.74706783
nonDEG	-0.7220381	0.23228371	1	nonDEG	0.5570456	0.33577835
nonDEG	-0.3682632	0.39581195	1	nonDEG	0.18756055	0.62282374
nonDEG	0.33426596	0.46201899	1	nonDEG	0.06260644	0.86843895
nonDEG	0.26926819	0.78961419	1	nonDEG	-1.3778681	0.21257624
nonDEG	0.27328753	0.57684459	1	nonDEG	-0.510525	0.20186641
nonDEG	1.03602697	0.23861185	1	nonDEG	-1.7012005	0.01369087
nonDEG	0.09517836	0.82693834	1	nonDEG	-0.0450961	0.90109026
nonDEG	0.13884064	0.74740977	1	nonDEG	0.16357515	0.65272085
nonDEG	0.07981729	0.86947848	1	nonDEG	-0.4439228	0.29234338
nonDEG	0.4787651	0.37887564	1	nonDEG	-0.0320661	0.94897865
nonDEG	3.1665595	0.23224078	1	nonDEG	-0.0737049	1
nonDEG	-0.2754464	0.59353044	1	nonDEG	0.13741483	0.77933548
nonDEG	0.73593933	0.35521381	1	nonDEG	-0.0943219	0.90141649
nonDEG	-0.6695632	0.13928766	1	nonDEG	0.7394442	0.09248969
nonDEG	0.10782757	0.81228393	1	nonDEG	-0.1727747	0.64296292
nonDEG	-0.5101258	0.26149247	1	nonDEG	-0.1576912	0.72693036
nonDEG	-0.2398204	0.59043441	1	nonDEG	0.09707579	0.80858457
nonDEG	-0.0580686	0.910545	1	nonDEG	-0.5080341	0.30162254
nonDEG	-0.1714571	0.69306402	1	nonDEG	0.28806199	0.46081472
nonDEG	-0.3404287	0.44955412	1	nonDEG	0.08582698	0.82471092
nonDEG	0.20486944	0.63993648	1	nonDEG	-0.487835	0.21185258
nonDEG	-0.2293843	0.58166904	1	nonDEG	-0.01705	0.96261958
nonDEG	-0.020446	0.9915345	1	nonDEG	0.01319986	1
nonDEG	-0.6670848	0.22319069	1	nonDEG	0.37865527	0.44846987
nonDEG	-0.0198759	0.96400243	1	nonDEG	0.11415058	0.75449627
nonDEG	0.33445469	0.58116308	1	nonDEG	-0.3844071	0.52198269
nonDEG	-4.0292575	0.35303627	1	nonDEG	4.90094585	0.14496517
nonDEG	0.23514398	0.58801505	1	nonDEG	-0.1099779	0.76146209
nonDEG	-0.0645806	0.90868016	1	nonDEG	-0.4764131	0.3756435
nonDEG	-0.0091643	0.98339108	1	nonDEG	-0.1631074	0.65422324

nonDEG	0.29444903	0.53892986	1	nonDEG	0.01528179	0.97347874
nonDEG	-0.2436263	0.57568662	1	nonDEG	0.29379377	0.42601837
nonDEG	-0.2247287	0.64741775	1	nonDEG	0.19588193	0.68599527
nonDEG	-0.0614496	0.89913971	1	nonDEG	0.04771645	0.91762532
nonDEG	0.23391304	0.59447281	1	nonDEG	0.06960732	0.85215647
nonDEG	-0.564748	0.43987466	1	nonDEG	-0.4506076	0.58326531
nonDEG	0.03998897	0.92485758	1	nonDEG	0.0570301	0.87460697
nonDEG	-0.3764247	0.37597435	1	nonDEG	0.36777899	0.31307699
nonDEG	-0.0656239	0.87957113	1	nonDEG	0.22192368	0.54759499
nonDEG	-0.0544758	0.89767531	1	nonDEG	-0.1779171	0.62362186
nonDEG	-0.1617188	0.70495416	1	nonDEG	0.12644315	0.73420728
nonDEG	0.05176085	0.91885452	1	nonDEG	-0.0932631	0.83586332
nonDEG	0.80933385	0.36200027	1	nonDEG	1.94519333	0.0196893
nonDEG	-0.0585004	0.9005747	1	nonDEG	-0.5959293	0.1511263
nonDEG	0.29488769	0.59009351	1	nonDEG	-0.5022187	0.2759446
nonDEG	0.21706385	0.73326795	1	nonDEG	-0.2634712	0.68299518
nonDEG	0.19217317	0.65075629	1	nonDEG	-0.0203563	0.95749421
nonDEG	-0.4096612	0.34675467	1	nonDEG	-0.0178008	0.96108321
nonDEG	-0.2125465	0.6195452	1	nonDEG	0.22736039	0.55463679
nonDEG	0.19142139	0.74735052	1	nonDEG	0.35822177	0.55300223
nonDEG	0.10897181	0.81485264	1	nonDEG	-0.6320491	0.13146379
nonDEG	0.03896138	0.9286537	1	nonDEG	0.1999032	0.57968552
nonDEG	0.30907983	1	1	nonDEG	0.2556291	0.93158641
nonDEG	-1.9804983	0.04320867	1	nonDEG	3.17048789	0.00046998
nonDEG	0.24116228	0.56971287	1	nonDEG	-0.17489	0.63652945
nonDEG	-0.3991537	0.40273596	1	nonDEG	0.19267215	0.67074601
nonDEG	0.19134425	0.64797076	1	nonDEG	-0.609852	0.09463214
nonDEG	-0.079796	0.86975232	1	nonDEG	0.24577555	0.56071351
nonDEG	-0.4826807	0.71881092	1	nonDEG	1.47127846	0.17285201
nonDEG	-1.1256456	0.15302575	1	nonDEG	0.18100018	0.82483828
nonDEG	-0.058358	0.89323691	1	nonDEG	0.15898663	0.6618365
nonDEG	-1.9131963	0.53532896	1	nonDEG	0.74187403	0.86010151
nonDEG	-0.0160368	0.97251247	1	nonDEG	-0.5462915	0.17601083
nonDEG	-0.0505374	0.91323327	1	nonDEG	-0.6494014	0.11700807
nonDEG	0.09569296	0.84487086	1	nonDEG	-0.4694074	0.25068135
nonDEG	-0.3312397	0.85926168	1	nonDEG	0.9083634	0.43424824
nonDEG	-0.3121504	0.59751462	1	nonDEG	0.47624747	0.40377275
nonDEG	0.4862106	0.29629743	1	nonDEG	0.0931803	0.80900789
nonDEG	0.12970795	0.79511067	1	nonDEG	0.01041156	0.98514369
nonDEG	0.01451147	0.97799194	1	nonDEG	-0.2188767	0.65684477
nonDEG	-0.1541401	0.7191745	1	nonDEG	0.14396048	0.69900379
nonDEG	0.21519006	0.63775538	1	nonDEG	-0.00605	0.98901188
nonDEG	-1.1168835	0.14022699	1	nonDEG	0.79043777	0.21076856

nonDEG	-0.6211058	0.36119783	1	nonDEG	-0.7321884	0.33780006
nonDEG	-0.0441455	0.92322834	1	nonDEG	-0.1397557	0.73552781
nonDEG	-0.1373358	0.80659196	1	nonDEG	-0.1181287	0.83844223
nonDEG	0.37297339	0.51976405	1	nonDEG	-0.243856	0.67245394
nonDEG	-0.1756288	0.68635398	1	nonDEG	0.00858718	0.98193264
nonDEG	-0.1757546	0.68083349	1	nonDEG	-0.1452853	0.69190805
nonDEG	-2.7325802	0.31589151	1	nonDEG	5.06440702	0.00505639
nonDEG	0.1114004	0.87383767	1	nonDEG	-0.0793144	0.90934692
nonDEG	0.49376635	1	1	nonDEG	-3.987563	0.44114685
nonDEG	-0.0202455	0.96463486	1	nonDEG	-0.0914567	0.82521605
nonDEG	-0.1815795	0.67870125	1	nonDEG	0.19354362	0.61500576
nonDEG	-0.6873343	0.17137657	1	nonDEG	0.33823021	0.38356445
nonDEG	-0.1684847	0.69696348	1	nonDEG	0.071941	0.84493063
nonDEG	-0.0323229	0.94858108	1	nonDEG	-0.2682925	0.56104627
nonDEG	0.08020852	0.95099127	1	nonDEG	0.93186046	0.28195613
nonDEG	0.26325232	0.541291	1	nonDEG	-0.0085032	0.98176309
nonDEG	1.53527186	0.07470459	1	nonDEG	-0.2781091	0.72969698
nonDEG	-0.0637594	0.88315614	1	nonDEG	0.01204301	0.97589065
nonDEG	-5.090254	0.07558548	1	nonDEG	6.9314093	0.01699083
nonDEG	-0.0483919	0.91084893	1	nonDEG	-0.1997394	0.59651042
nonDEG	0.1459514	0.76010369	1	nonDEG	0.51611459	0.27057235
nonDEG	-0.1108897	0.79181877	1	nonDEG	-0.3205748	0.37004239
nonDEG	0.20291589	0.75234305	1	nonDEG	0.21518846	0.69492531
nonDEG	0.26354138	0.61071316	1	nonDEG	-0.091079	0.85146264
nonDEG	0.36098333	0.46979687	1	nonDEG	-0.3683934	0.40466
nonDEG	0.59091997	0.36266082	1	nonDEG	0.23371073	0.70080266
nonDEG	0.2231039	0.61072835	1	nonDEG	-5.35E-05	1
nonDEG	0.11039358	0.80637586	1	nonDEG	0.12129634	0.74709544
nonDEG	-0.4113039	0.35490564	1	nonDEG	0.4560456	0.21042034
nonDEG	0	1	1	nonDEG	-5.13E-15	1
nonDEG	0.13666213	0.74699777	1	nonDEG	0.03107343	0.93212613
nonDEG	-0.3188236	0.46529849	1	nonDEG	0.14307928	0.69680515
nonDEG	0.99814372	0.15482362	1	nonDEG	-0.5877643	0.37050012
nonDEG	-0.2539274	0.63019043	1	nonDEG	0.07679318	0.88742265
nonDEG	0.20219545	0.70438899	1	nonDEG	-0.5072643	0.27574085
nonDEG	0.33322725	0.48342933	1	nonDEG	0.26235787	0.55207344
nonDEG	0.16179521	0.77771057	1	nonDEG	-0.1288189	0.80830536
nonDEG	0.0650154	0.88553768	1	nonDEG	0.20881568	0.57022601
nonDEG	0.17194677	0.7525961	1	nonDEG	-0.1148289	0.81677247
nonDEG	-0.0737041	0.89515938	1	nonDEG	0.48374997	0.32501475
nonDEG	-0.2237464	0.67153863	1	nonDEG	-0.3311185	0.55644232
nonDEG	-0.238774	0.59365232	1	nonDEG	0.01127436	0.97623522
nonDEG	0.18156808	0.69098339	1	nonDEG	-0.2009998	0.59392442

nonDEG	0.43096622	0.39547624	1	nonDEG	-0.5186416	0.21818196
nonDEG	-0.1172371	0.78350277	1	nonDEG	0.20421285	0.57738563
nonDEG	0.40848111	0.33635864	1	nonDEG	-0.4176172	0.2539411
nonDEG	0.31814601	0.47657864	1	nonDEG	-0.2208218	0.5685419
nonDEG	-1.0675621	0.47061763	1	nonDEG	-1.1117014	0.53987905
nonDEG	0.11455314	0.78797192	1	nonDEG	0.01980944	0.95733263
nonDEG	0.35218184	0.57390706	1	nonDEG	-0.1703521	0.7394133
nonDEG	-0.0485581	0.90954664	1	nonDEG	0.06336039	0.86375588
nonDEG	0.22061009	0.61843542	1	nonDEG	0.1809454	0.63877986
nonDEG	0.19744933	0.64455178	1	nonDEG	-0.0708721	0.8441627
nonDEG	-0.256232	0.56594135	1	nonDEG	0.02152441	0.95694873
nonDEG	0.55929385	0.51779273	1	nonDEG	-1.1657012	0.18602327
nonDEG	0.23549466	0.58367914	1	nonDEG	-0.1677978	0.64883333
nonDEG	0.30623974	0.50316702	1	nonDEG	-0.0900457	0.82769625
nonDEG	0.09904688	0.84519803	1	nonDEG	-0.7700218	0.08568827
nonDEG	-0.205337	0.63314112	1	nonDEG	0.33691754	0.36276859
nonDEG	-0.1824483	0.67027942	1	nonDEG	-0.3614538	0.32149399
nonDEG	-0.1367058	0.7509977	1	nonDEG	-0.0184813	0.96280502
nonDEG	-0.5710053	0.26509253	1	nonDEG	0.16711126	0.74138419
nonDEG	-0.3264094	0.50790203	1	nonDEG	0.05524315	0.90703134
nonDEG	-0.2701322	0.53007598	1	nonDEG	-0.0666432	0.85478933
nonDEG	-0.0667918	0.89168969	1	nonDEG	0.19729069	0.64663836
nonDEG	-0.0715857	1	1	nonDEG	0.00719018	1
nonDEG	0.01355114	0.97505555	1	nonDEG	-0.0691733	0.85817761
nonDEG	-0.1523793	0.71584451	1	nonDEG	0.34825786	0.33115288
nonDEG	0.36039338	0.44717997	1	nonDEG	-0.5312972	0.23064999
nonDEG	0.32635454	0.53040007	1	nonDEG	-0.5904744	0.22566059
nonDEG	-0.0249405	0.95749474	1	nonDEG	-0.0662023	0.87384184
nonDEG	0.58105891	0.23645901	1	nonDEG	-0.4164216	0.34225042
nonDEG	0.15077305	0.72333964	1	nonDEG	0.11380171	0.75429108
nonDEG	0.16783089	0.68871809	1	nonDEG	0.2670549	0.47535594
nonDEG	-0.1589719	0.72322184	1	nonDEG	0.00840684	0.98525409
nonDEG	0.20333754	0.63362579	1	nonDEG	-0.114687	0.75447401
nonDEG	-0.4572844	0.38089444	1	nonDEG	0.16549339	0.74854993
nonDEG	0.02409854	0.95495291	1	nonDEG	0.1874096	0.60795207
nonDEG	-0.3953776	0.55306623	1	nonDEG	0.62070861	0.34965719
nonDEG	0.09037839	0.94274999	1	nonDEG	-0.188408	0.85799522
nonDEG	0.65683754	0.32944103	1	nonDEG	0.41123024	0.48304787
nonDEG	-0.0961408	0.82403019	1	nonDEG	-0.1328091	0.71368591
nonDEG	-0.2129323	0.62220523	1	nonDEG	0.36997059	0.30911415
nonDEG	-0.0310419	0.94638195	1	nonDEG	-0.0387971	0.92982274
nonDEG	-1.532492	0.13187084	1	nonDEG	4.47162433	0.00035619
nonDEG	-0.0336643	0.93746707	1	nonDEG	-0.0393989	0.91364572

nonDEG	-0.1523071	0.75187651	1	nonDEG	0.42100344	0.33670432
nonDEG	1.90961696	0.01829832	0.94399538	nonDEG	-1.0421116	0.18741672
nonDEG	-0.1060302	0.81338438	1	nonDEG	-0.1824185	0.66416474
nonDEG	-0.0645454	0.90265247	1	nonDEG	-0.3776097	0.46871197
nonDEG	-1.4755361	0.49903927	1	nonDEG	0.63655352	0.88701115
nonDEG	-0.3850297	0.4321612	1	nonDEG	-0.1206832	0.79226037
nonDEG	-1.2701804	0.414507	1	nonDEG	1.92932081	0.22209111
nonDEG	-0.2354613	0.61101305	1	nonDEG	0.28273702	0.50680312
nonDEG	1.64311582	0.04887761	1	nonDEG	-0.7067649	0.30607798
nonDEG	-0.4221681	0.41435028	1	nonDEG	-0.5459838	0.19438569
nonDEG	-0.3821227	0.46724391	1	nonDEG	-0.2314451	0.67141509
nonDEG	-0.621031	0.33882228	1	nonDEG	0.9027625	0.18353521
nonDEG	-0.1139908	0.79028474	1	nonDEG	0.09031989	0.81170479
nonDEG	-0.1237041	0.77475474	1	nonDEG	0.09751574	0.79883753
nonDEG	-0.1291774	0.75874319	1	nonDEG	-0.0454302	0.89957237
nonDEG	1.64627828	0.61973138	1	nonDEG	-4.1272178	0.40876757
nonDEG	-0.1841736	0.69319557	1	nonDEG	-0.1942742	0.59876394
nonDEG	-0.1196234	0.88407636	1	nonDEG	0.36215939	0.66070843
nonDEG	-0.1791629	0.67651487	1	nonDEG	0.15977185	0.66617128
nonDEG	-0.2327221	0.58385473	1	nonDEG	0.01777843	0.96140218
nonDEG	-0.077708	0.86889242	1	nonDEG	-0.3183232	0.46295335
nonDEG	0.20762544	0.6382397	1	nonDEG	-0.2791545	0.47765806
nonDEG	-0.0256985	0.96091546	1	nonDEG	-0.0970743	0.80082758
nonDEG	-0.0617532	0.90020689	1	nonDEG	0.02354642	0.9557585
nonDEG	-0.6861028	0.17918544	1	nonDEG	0.31231516	0.52930237
nonDEG	0.08790098	0.86026889	1	nonDEG	-0.2765849	0.52176278
nonDEG	0.97067738	0.20216576	1	nonDEG	0.10034416	0.89328971
nonDEG	0.19466775	0.66785785	1	nonDEG	-0.4519182	0.30036987
nonDEG	0.33325405	0.43491588	1	nonDEG	0.06537258	0.86227724
nonDEG	-0.3295806	0.45458814	1	nonDEG	-0.0526781	0.8987073
nonDEG	0.17945451	0.82496065	1	nonDEG	0.27078566	0.74968665
nonDEG	0.35847129	0.48054132	1	nonDEG	-0.5131463	0.28944662
nonDEG	-1.1619531	0.43280771	1	nonDEG	0.81593446	0.61980769
nonDEG	0.33817313	0.47754088	1	nonDEG	0.15707241	0.69096108
nonDEG	-0.1686674	1	1	nonDEG	4.90071078	0.06153282
nonDEG	-0.2552044	0.55386889	1	nonDEG	-0.0276035	0.94386738
nonDEG	0.10564338	0.81645094	1	nonDEG	-0.1352766	0.74151989
nonDEG	-0.1717732	0.68168056	1	nonDEG	-0.6310064	0.08606625
nonDEG	0.25001066	0.59258408	1	nonDEG	-0.2057595	0.59804292
nonDEG	-0.4035538	0.36434733	1	nonDEG	0.20515718	0.60018157
nonDEG	0.53613834	0.21943729	1	nonDEG	-0.0852252	0.8157756
nonDEG	-3.7692016	0.40031261	1	nonDEG	5.49080042	0.16514022
nonDEG	0.17333884	0.73193006	1	nonDEG	-0.0382317	0.929493

nonDEG	0.39778301	0.43098747	1	nonDEG	0.78183555	0.06780751
nonDEG	0.16202513	0.73886161	1	nonDEG	-0.2839113	0.45554224
nonDEG	-0.3486769	0.43609722	1	nonDEG	-0.0720873	0.85839766
nonDEG	-0.2914877	0.51108527	1	nonDEG	0.19768442	0.63746973
nonDEG	-0.2901803	0.50454393	1	nonDEG	0.15651967	0.67227553
nonDEG	-0.0026147	0.99695967	1	nonDEG	-0.072717	0.86284685
nonDEG	0.41778458	0.33432946	1	nonDEG	0.15827601	0.66658267
nonDEG	0.24343108	0.88063162	1	nonDEG	-0.0081115	1
nonDEG	-0.1594557	0.72723609	1	nonDEG	0.27453909	0.46890005
nonDEG	-0.043746	0.92929389	1	nonDEG	-0.2061056	0.65005497
nonDEG	0.01611724	0.97011695	1	nonDEG	0.12921941	0.71890088
nonDEG	0.48896694	0.28749061	1	nonDEG	0.06502617	0.86207435
nonDEG	-0.370129	0.45400397	1	nonDEG	0.12155647	0.79227095
nonDEG	0.48394614	0.63935937	1	nonDEG	1.11587263	0.26523441
nonDEG	0.67905761	0.78516706	1	nonDEG	2.47987519	0.09907234
nonDEG	0.29775328	0.49221065	1	nonDEG	0.00098888	0.99804191
nonDEG	-0.2014855	0.67195271	1	nonDEG	0.10333952	0.81187284
nonDEG	-0.1089202	0.84286797	1	nonDEG	0.33347102	0.46399768
nonDEG	0.05472888	0.89733251	1	nonDEG	-0.0162167	0.96504124
nonDEG	0.04163665	0.9221882	1	nonDEG	-0.345404	0.33663369
nonDEG	-0.3077755	0.51167867	1	nonDEG	-0.158236	0.69165044
nonDEG	-0.1869917	0.77983305	1	nonDEG	-0.1280801	0.86216771
nonDEG	-1.8527071	0.13942103	1	nonDEG	1.77202808	0.09264448
nonDEG	0.11882954	0.79920308	1	nonDEG	-0.0930891	0.81593108
nonDEG	0.57594394	0.56480646	1	nonDEG	-0.6311556	0.51388904
nonDEG	0.51069648	0.362076	1	nonDEG	-0.0516215	0.91829653
nonDEG	-0.0665314	0.88360491	1	nonDEG	-0.269989	0.46617343
nonDEG	-0.0470512	0.91819487	1	nonDEG	0.01679836	0.96657742
nonDEG	0.03794415	0.94114358	1	nonDEG	-0.4151126	0.37234229
nonDEG	0.00433314	0.99298505	1	nonDEG	0.13859562	0.709257
nonDEG	0.00599408	1	1	nonDEG	0.2241206	0.95922011
nonDEG	0.02614192	0.95242434	1	nonDEG	0.08263454	0.8247548
nonDEG	-0.0073294	0.98677879	1	nonDEG	0.00284708	0.99456135
nonDEG	-0.3147889	0.52237261	1	nonDEG	-0.1157263	0.783693
nonDEG	0.30029715	0.84952191	1	nonDEG	-3.2916874	0.11301914
nonDEG	-0.3971976	0.40560067	1	nonDEG	0.10277391	0.82064906
nonDEG	-0.4468035	0.51426587	1	nonDEG	0.48752904	0.44563096
nonDEG	-0.1412476	0.77388965	1	nonDEG	1.21992848	0.00357187
nonDEG	0.05922363	0.91084279	1	nonDEG	-0.4049789	0.34212997
nonDEG	-0.4642567	0.40170703	1	nonDEG	1.05232062	0.03610029
nonDEG	-0.3132999	0.45198473	1	nonDEG	-0.0330987	0.92787917
nonDEG	0.27483207	0.65302225	1	nonDEG	-0.2479264	0.63542281
nonDEG	0.78564899	0.26686618	1	nonDEG	0.04122486	0.95760664

nonDEG	0.47940704	0.26493246	1	nonDEG	0.01087891	0.97755919
nonDEG	0.62695229	0.14456	1	nonDEG	-0.2964772	0.41371275
nonDEG	-0.4127241	0.34188837	1	nonDEG	0.26765853	0.47902781
nonDEG	1.7915437	0.05057191	1	nonDEG	-0.8816656	0.32559162
nonDEG	-0.0426455	0.92084764	1	nonDEG	0.14207599	0.70683151
nonDEG	0.15654637	0.71474161	1	nonDEG	-0.2246838	0.54015372
nonDEG	0.82950481	0.46994396	1	nonDEG	-1.8009371	0.2846139
nonDEG	0.11237314	0.83113366	1	nonDEG	-0.396004	0.34273212
nonDEG	0.50557093	0.52078702	1	nonDEG	0.25280356	0.73807319
nonDEG	-0.3290151	0.47809141	1	nonDEG	0.26617572	0.4763706
nonDEG	0.10555781	0.85743952	1	nonDEG	0.16275767	0.76949712
nonDEG	0.04669929	0.94530507	1	nonDEG	-0.0309456	0.97020785
nonDEG	-0.0931548	0.83817817	1	nonDEG	0.07375871	0.85884713
nonDEG	-0.2236608	0.61668251	1	nonDEG	-0.2229336	0.57267716
nonDEG	-0.1817695	0.7687763	1	nonDEG	0.55714472	0.33834863
nonDEG	-0.142248	0.82858751	1	nonDEG	0.2605544	0.69932174
nonDEG	-0.1579153	0.85400629	1	nonDEG	-0.7778434	0.42733833
nonDEG	-0.3459764	0.53258919	1	nonDEG	-0.5883134	0.29932909
nonDEG	0.75915754	0.18720957	1	nonDEG	-0.04035	0.93673378
nonDEG	0.14309166	0.7344025	1	nonDEG	-0.3349759	0.35915972
nonDEG	0.02623858	0.95182223	1	nonDEG	-0.106558	0.77033461
nonDEG	-0.2896975	0.66109937	1	nonDEG	0.21197043	0.7553129
nonDEG	0.29560873	0.58548296	1	nonDEG	-0.0923254	0.82798077
nonDEG	0.05878061	0.89081531	1	nonDEG	-0.0951144	0.79646478
nonDEG	0.01236929	1	1	nonDEG	-0.4982847	0.54084952
nonDEG	-0.1861988	0.70766408	1	nonDEG	0.0328838	0.95052565
nonDEG	-0.0222761	0.97458891	1	nonDEG	-0.0524758	0.92312825
nonDEG	0.03202156	0.94147869	1	nonDEG	0.23826329	0.52051475
nonDEG	1.61735632	0.10973798	1	nonDEG	-0.6575567	0.43160642
nonDEG	-0.0536399	0.91450611	1	nonDEG	0.09442568	0.83832768
nonDEG	-0.0705457	0.88249688	1	nonDEG	-0.4485088	0.34403986
nonDEG	-7.276724	0.01433904	0.88752292	nonDEG	4.92120436	0.26329714
nonDEG	0.25094648	0.59665526	1	nonDEG	0.25448853	0.53999628
nonDEG	0.13468513	0.76338841	1	nonDEG	-0.0688862	0.86976483
nonDEG	0.13258912	0.78487119	1	nonDEG	-0.0017637	0.99777881
nonDEG	0.33827957	0.4525185	1	nonDEG	-0.0392787	0.925787
nonDEG	0.03732324	0.93327621	1	nonDEG	0.01732013	0.96360961
nonDEG	-0.408361	0.47535197	1	nonDEG	-0.6329725	0.28209403
nonDEG	0.23450764	0.57504181	1	nonDEG	-0.2652699	0.45929075
nonDEG	-1.5188773	0.61527601	1	nonDEG	1.98279711	0.38945742
nonDEG	0.25344779	0.56487491	1	nonDEG	-0.1298938	0.73552721
nonDEG	0.45238333	0.46461135	1	nonDEG	0.5676891	0.28047368
nonDEG	0.19061931	0.6993209	1	nonDEG	-0.4950031	0.27745144

nonDEG	0.2755555	0.54847225	1	nonDEG	-0.2510305	0.53688014
nonDEG	-0.388722	0.3553131	1	nonDEG	-0.5762716	0.12157981
nonDEG	0.39755079	0.36831008	1	nonDEG	-0.2670386	0.47296366
nonDEG	-0.1699363	0.70340787	1	nonDEG	-0.0180551	0.96695449
nonDEG	-0.2532223	0.59459467	1	nonDEG	0.28602426	0.49084432
nonDEG	0.36269698	0.42266615	1	nonDEG	-0.5103531	0.1727036
nonDEG	0.41929993	0.35803703	1	nonDEG	-0.4779418	0.2103417
nonDEG	-3.1295757	0.64435681	1	nonDEG	5.8868559	0.04421669
nonDEG	-0.6955871	0.15019314	1	nonDEG	-0.6856544	0.18091094
nonDEG	-0.0805578	0.96839799	1	nonDEG	-0.247097	0.9093041
nonDEG	-0.1971958	0.64163774	1	nonDEG	-0.1223743	0.74205046
nonDEG	0.10953023	0.92781213	1	nonDEG	0.37186396	0.70793071
nonDEG	-0.1696633	0.70622917	1	nonDEG	-0.5305877	0.19151885
nonDEG	-0.9816744	0.28703575	1	nonDEG	0.78078534	0.36974297
nonDEG	-0.3015651	0.508556	1	nonDEG	0.06166499	0.86483408
nonDEG	0.41678619	0.42710448	1	nonDEG	-0.3477678	0.43412405
nonDEG	0.2250299	0.62550828	1	nonDEG	-0.2465562	0.4932452
nonDEG	0.36423722	0.74923729	1	nonDEG	-0.9567247	0.38440701
nonDEG	-0.209228	0.85135285	1	nonDEG	-1.83892	0.25238924
nonDEG	0.04576999	0.97127013	1	nonDEG	0.10982817	0.90572549
nonDEG	0.18301301	0.68222019	1	nonDEG	-0.0118698	0.97466546
nonDEG	-0.0772453	0.85932814	1	nonDEG	0.12639155	0.72647019
nonDEG	-0.3385807	0.59994256	1	nonDEG	-0.1949168	0.77808645
nonDEG	-0.1833592	0.69003757	1	nonDEG	-0.2366088	0.55735428
nonDEG	-0.0107373	0.98037503	1	nonDEG	-0.1425071	0.6974359
nonDEG	0.11526312	0.80860674	1	nonDEG	-0.358738	0.4003871
nonDEG	0.1487602	0.77657221	1	nonDEG	0.19418116	0.69076137
nonDEG	0.04352207	0.91852241	1	nonDEG	-0.3298669	0.36115688
nonDEG	0.07246764	0.95097482	1	nonDEG	0.75954439	0.29315203
nonDEG	0.5040513	0.24851796	1	nonDEG	-0.179276	0.63633563
nonDEG	-0.2468894	0.58783916	1	nonDEG	0.22687755	0.57037136
nonDEG	-0.1829509	0.88293531	1	nonDEG	-0.189359	0.96252263
nonDEG	-0.4983032	0.35792004	1	nonDEG	-0.5201006	0.35281464
nonDEG	0.27847919	0.603223	1	nonDEG	0.03514184	0.92857025
nonDEG	-0.5311919	0.57868967	1	nonDEG	1.72270825	0.05867977
nonDEG	0.19280175	0.70401101	1	nonDEG	-0.0403144	0.91761081
nonDEG	1.83585724	0.00435556	0.68407511	nonDEG	-0.285387	0.63040899
nonDEG	-0.5282264	0.32256758	1	nonDEG	-0.290637	0.5887335
nonDEG	0.25760443	0.54643071	1	nonDEG	0.10634865	0.77413084
nonDEG	-0.3525407	0.68703781	1	nonDEG	0.27307019	0.76052257
nonDEG	0.00127805	0.99939988	1	nonDEG	0.28888637	0.48119951
nonDEG	-0.0999394	0.81484364	1	nonDEG	0.07219699	0.84299675
nonDEG	0.26256949	0.54770553	1	nonDEG	-0.0880754	0.81005166

nonDEG	-0.5046424	0.29320301	1	nonDEG	-0.0132036	0.97802571
nonDEG	-0.1266375	0.7670162	1	nonDEG	-0.0689966	0.85050807
nonDEG	0.17300382	0.6913552	1	nonDEG	-0.0041387	0.99110143
nonDEG	0.21302629	0.62412464	1	nonDEG	-0.0640684	0.86095538
nonDEG	0.13502616	0.78429897	1	nonDEG	-0.0228282	0.95687874
nonDEG	0.18860476	0.66405467	1	nonDEG	0.02787433	0.94104204
nonDEG	-0.1270228	0.92147971	1	nonDEG	1.28723727	0.24260674
nonDEG	0.26022572	0.58141099	1	nonDEG	-0.4773966	0.27050458
nonDEG	-0.1308003	0.77022284	1	nonDEG	0.36707113	0.34946139
nonDEG	-0.0080251	0.99090632	1	nonDEG	-0.1793379	0.74946095
nonDEG	0.15148282	0.76569286	1	nonDEG	-0.4458651	0.25362259
nonDEG	-0.2033536	0.67619144	1	nonDEG	-0.1760601	0.6790342
nonDEG	-0.0696514	0.92158026	1	nonDEG	0.25723166	0.69256851
nonDEG	0.23089057	0.59840921	1	nonDEG	-0.1538494	0.68623666
nonDEG	-0.0891907	0.87906866	1	nonDEG	0.36147028	0.5095848
nonDEG	0.27938567	0.69545773	1	nonDEG	-0.0296579	0.97294996
nonDEG	-0.3740472	0.38177662	1	nonDEG	0.00223677	0.99602593
nonDEG	-0.3234551	0.46585249	1	nonDEG	0.2712159	0.4706231
nonDEG	0.55460542	0.28439755	1	nonDEG	0.03696201	0.93816127
nonDEG	0.28889077	0.51695764	1	nonDEG	-0.3815461	0.3112211
nonDEG	-0.2001337	0.64695724	1	nonDEG	-0.0827936	0.82121241
nonDEG	-0.0955573	0.8425405	1	nonDEG	0.06047107	0.89236783
nonDEG	-0.0316552	0.94624359	1	nonDEG	0.3729475	0.35676769
nonDEG	-0.3531885	0.58737986	1	nonDEG	0.21223652	0.73325585
nonDEG	-0.1723565	0.69492422	1	nonDEG	-0.0011481	0.99878791
nonDEG	0.49178133	0.26105094	1	nonDEG	-0.226872	0.54640612
nonDEG	1.43278963	0.06244168	1	nonDEG	-1.4162839	0.03745883
nonDEG	-0.1200945	0.88740059	1	nonDEG	1.15383293	0.08874318
nonDEG	0	1	1	nonDEG	-5.13E-15	1
nonDEG	0.48003987	0.42419982	1	nonDEG	-0.8870343	0.08330636
nonDEG	-0.7801488	0.09382595	1	nonDEG	0.07229297	0.84585695
nonDEG	-0.3112891	0.45789752	1	nonDEG	0.26919806	0.45347841
nonDEG	-0.3550943	0.56772458	1	nonDEG	0.69096797	0.23265614
nonDEG	-0.2514917	0.58011996	1	nonDEG	-0.5597602	0.19751303
nonDEG	0.41475669	0.40110103	1	nonDEG	-0.0954948	0.8226125
nonDEG	-1.0224017	0.80535256	1	nonDEG	2.98937451	0.06301146
nonDEG	0.12275847	0.77244029	1	nonDEG	-0.0274722	0.94138286
nonDEG	-0.0020681	1	1	nonDEG	-0.5390168	0.31635904
nonDEG	-0.2174434	0.78499975	1	nonDEG	-0.2717504	0.69511278
nonDEG	0.3170996	0.47032739	1	nonDEG	0.90646677	0.02560059
nonDEG	-0.5115911	0.38688333	1	nonDEG	0.65903352	0.22738156
nonDEG	-0.0617538	0.88965581	1	nonDEG	0.04617235	0.90623003
nonDEG	-0.1966543	0.64834313	1	nonDEG	0.16149358	0.66436141

nonDEG	-1.7053145	0.43323233	1	nonDEG	3.51135784	0.05676373
nonDEG	0.09926206	0.83099487	1	nonDEG	0.04754889	0.9126604
nonDEG	0.04793555	0.91610942	1	nonDEG	0.47523924	0.22404959
nonDEG	-0.0907833	0.83384199	1	nonDEG	-0.0767957	0.83438705
nonDEG	0.02246021	0.96263407	1	nonDEG	-0.6134371	0.12624523
nonDEG	-0.2598827	0.70119032	1	nonDEG	1.21518376	0.03631143
nonDEG	0.37818159	0.51769675	1	nonDEG	0.00529671	0.99587312
nonDEG	0.21515264	0.63235232	1	nonDEG	-0.0076898	0.9856462
nonDEG	0.191001	0.65211336	1	nonDEG	-0.0299648	0.93375014
nonDEG	-0.1454913	0.74382883	1	nonDEG	-0.2804666	0.46254698
nonDEG	0.18216711	0.67541809	1	nonDEG	-0.2815502	0.46200236
nonDEG	0.62108645	0.47532866	1	nonDEG	0.24043205	0.79165067
nonDEG	0.04314377	0.93000874	1	nonDEG	-0.2197743	0.5951522
nonDEG	-0.0065005	0.98895928	1	nonDEG	0.13096483	0.7239865
nonDEG	0.12519505	0.88632627	1	nonDEG	0.66173654	0.35652825
nonDEG	0.0075173	0.98836019	1	nonDEG	-0.6798478	0.14421447
nonDEG	-1.1046926	0.22365598	1	nonDEG	1.68233874	0.09060359
nonDEG	-0.1817715	0.66735229	1	nonDEG	-0.4963164	0.18575656
nonDEG	0.14777348	0.80380857	1	nonDEG	-0.084753	0.88670233
nonDEG	0.24658475	0.69605441	1	nonDEG	-0.1463543	0.81551185
nonDEG	0.44088305	0.587503	1	nonDEG	0.53634294	0.43733722
nonDEG	-0.352094	0.73980366	1	nonDEG	0.15554864	0.9294707
nonDEG	0.47286835	0.41048962	1	nonDEG	-0.2503123	0.64239522
nonDEG	-0.1689975	0.70243041	1	nonDEG	0.24799369	0.53714166
nonDEG	0.19739326	0.64403826	1	nonDEG	-0.2984333	0.42497382
nonDEG	0.05863091	0.89193195	1	nonDEG	-0.0027593	0.99502365
nonDEG	-0.3065134	0.57228258	1	nonDEG	-0.0899999	0.83938428
nonDEG	0.03693631	0.94355791	1	nonDEG	-0.9952136	0.05077641
nonDEG	0.55085637	0.47671587	1	nonDEG	-0.2330774	0.77402523
nonDEG	-0.617374	0.24235044	1	nonDEG	0.27016099	0.52533687
nonDEG	-0.5296058	0.24926114	1	nonDEG	-0.0120441	0.97662344
nonDEG	-0.3243459	0.50431314	1	nonDEG	0.22745134	0.61928117
nonDEG	-0.1559997	0.79465015	1	nonDEG	0.08859084	0.87280559
nonDEG	-0.0996958	0.81753932	1	nonDEG	-0.1770261	0.63043676
nonDEG	-0.0328153	1	1	nonDEG	1.02377192	0.33798992
nonDEG	0.7809279	0.25852105	1	nonDEG	0.68097178	0.25731141
nonDEG	-0.0541903	0.89872602	1	nonDEG	-0.1511665	0.68111238
nonDEG	-0.5910888	0.56094456	1	nonDEG	-0.3275618	0.85463497
nonDEG	-0.0003059	1	1	nonDEG	-0.0096615	0.97925206
nonDEG	-0.0971354	0.8437702	1	nonDEG	-0.4020701	0.41461444
nonDEG	-0.114906	0.94843589	1	nonDEG	-0.5206297	0.76552861
nonDEG	0.83314428	0.14197224	1	nonDEG	-0.3888693	0.46456672
nonDEG	0.34948731	0.80353973	1	nonDEG	0.43047473	0.73449018

nonDEG	0.33382908	0.55754707	1	nonDEG	-0.5955293	0.19531721
nonDEG	-0.0388042	0.94008234	1	nonDEG	0.01987695	0.96934729
nonDEG	3.53483341	0.01129001	0.84185458	nonDEG	-0.7436107	0.65704147
nonDEG	-0.0742381	0.86163238	1	nonDEG	-0.1098188	0.76126143
nonDEG	-0.7874264	0.3222826	1	nonDEG	1.71368977	0.0149021
nonDEG	0.09335958	0.8305066	1	nonDEG	0.3745172	0.32691902
nonDEG	0.59128182	0.42243791	1	nonDEG	-0.5459369	0.2974861
nonDEG	0.23525988	0.59253483	1	nonDEG	-0.0223501	0.95362706
nonDEG	-0.0574575	0.89502073	1	nonDEG	-0.1463391	0.70060735
nonDEG	0.27001921	0.79532133	1	nonDEG	-0.007895	1
nonDEG	-0.1572321	0.70876719	1	nonDEG	0.15858123	0.66251578
nonDEG	-0.0028194	0.99616327	1	nonDEG	-0.0069896	0.98624099
nonDEG	0.5361642	0.22348722	1	nonDEG	0.27419155	0.48157464
nonDEG	0.05656043	0.89419629	1	nonDEG	-0.1595859	0.66912754
nonDEG	0.26697934	0.54926008	1	nonDEG	0.10597937	0.76865847
nonDEG	-0.009391	0.98301633	1	nonDEG	-0.3713463	0.31895227
nonDEG	-0.3210115	0.5995967	1	nonDEG	0.57182443	0.32067359
nonDEG	0.0025846	0.99554495	1	nonDEG	-0.3624354	0.31696939
nonDEG	0.28760215	0.53823441	1	nonDEG	-0.0698611	0.86918196
nonDEG	0.21673865	0.81386732	1	nonDEG	-0.0345846	0.97681814
nonDEG	0.30679718	0.59000793	1	nonDEG	0.3825276	0.45953305
nonDEG	0.7984853	0.37339657	1	nonDEG	-0.9449852	0.35614042
nonDEG	-0.3720435	0.72690686	1	nonDEG	0.20875235	0.82958714
nonDEG	2.6268654	0.28142349	1	nonDEG	-0.2433331	0.96424886
nonDEG	0.20456097	0.65654629	1	nonDEG	-0.2917144	0.4453993
nonDEG	0.44755857	0.36357268	1	nonDEG	-0.3329448	0.44626642
nonDEG	-0.1113809	0.82870203	1	nonDEG	0.72906039	0.08990826
nonDEG	0.09178082	0.84425719	1	nonDEG	-0.3701093	0.34145516
nonDEG	1.05470728	0.33347057	1	nonDEG	0.91424609	0.36754511
nonDEG	0.07298624	0.89127076	1	nonDEG	0.10859842	0.82517357
nonDEG	-0.1743791	0.69304983	1	nonDEG	0.01923882	0.96192061
nonDEG	-0.6703321	0.43340445	1	nonDEG	0.02333947	1
nonDEG	0.03878871	0.92839109	1	nonDEG	-0.1074768	0.76659586
nonDEG	-0.4332947	0.39258278	1	nonDEG	0.48697997	0.28101156
nonDEG	0.52125646	0.22829796	1	nonDEG	0.05125335	0.88873918
nonDEG	0.19370931	0.65903056	1	nonDEG	-0.3871808	0.30380945
nonDEG	0.09530193	0.82943356	1	nonDEG	0.02704242	0.94549837
nonDEG	-0.0950154	0.91842636	1	nonDEG	0.5426112	0.52780933
nonDEG	-0.1769096	0.68080123	1	nonDEG	0.13617431	0.7207018
nonDEG	1.78112509	0.35297227	1	nonDEG	0.06451799	1
nonDEG	-0.2062262	0.65267478	1	nonDEG	0.11084558	0.78137467
nonDEG	0.14931087	0.83240891	1	nonDEG	0.74814813	0.27532411
nonDEG	0.19463613	0.64730506	1	nonDEG	-0.0956284	0.79145711

nonDEG	1.65144642	0.25088724	1	nonDEG	-1.5074578	0.27963677
nonDEG	-0.0490547	0.91657821	1	nonDEG	-0.121706	0.74495721
nonDEG	0.17165253	0.71079642	1	nonDEG	-0.0201031	0.9628042
nonDEG	0.25105819	0.66126112	1	nonDEG	0.02463688	0.96090477
nonDEG	0.33910133	0.43040505	1	nonDEG	0.0221649	0.95206151
nonDEG	-0.2315144	0.59274335	1	nonDEG	0.07483188	0.83725522
nonDEG	-2.4132346	1	1	nonDEG	5.34539273	0.07690721
nonDEG	0.07988916	0.87321902	1	nonDEG	0.34480218	0.42915248
nonDEG	1.07602713	0.1483166	1	nonDEG	-0.9329373	0.21408641
nonDEG	1.26115361	0.14920466	1	nonDEG	-0.9710811	0.2924733
nonDEG	1.9250457	0.01941793	0.97153571	nonDEG	-0.481036	0.49091259
nonDEG	0.86672171	0.09427661	1	nonDEG	-0.3901984	0.43350205
nonDEG	0.38551839	0.40323722	1	nonDEG	0.32836427	0.40555091
nonDEG	0.31647157	0.45766475	1	nonDEG	-0.0727499	0.83928591
nonDEG	-0.0641728	0.89489296	1	nonDEG	-0.4474643	0.31064887
nonDEG	-0.0699532	0.87777414	1	nonDEG	0.39313312	0.30478806
nonDEG	-0.2504792	0.69830188	1	nonDEG	-0.332902	0.60193238
nonDEG	0.33256381	0.55995614	1	nonDEG	-0.674943	0.14635566
nonDEG	-0.2163128	0.6391521	1	nonDEG	-0.005904	0.99028449
nonDEG	0.17396279	0.72656556	1	nonDEG	0.24745745	0.56252906
nonDEG	-0.5009388	0.24721438	1	nonDEG	0.01901309	0.95924318
nonDEG	0.12879641	0.8284569	1	nonDEG	-0.0652607	0.91390013
nonDEG	-0.2789144	0.51656491	1	nonDEG	0.25065954	0.50057138
nonDEG	-0.0053048	0.99037167	1	nonDEG	-0.0659465	0.8565176
nonDEG	0.48178103	0.25794371	1	nonDEG	-0.103612	0.77509236
nonDEG	0.23115201	0.68063634	1	nonDEG	-0.0268463	0.96172133
nonDEG	-0.7471592	0.69330851	1	nonDEG	1.89515816	0.16144801
nonDEG	-0.1002979	0.82963851	1	nonDEG	0.14746335	0.70742557
nonDEG	1.02177066	0.57807379	1	nonDEG	-0.6380445	0.82045954
nonDEG	0.45392222	0.68406549	1	nonDEG	0.4767161	0.706501
nonDEG	-0.0545999	0.89943722	1	nonDEG	-0.2125014	0.57249598
nonDEG	-0.0558007	0.89702588	1	nonDEG	0.07892326	0.8301532
nonDEG	-0.0717121	0.92148747	1	nonDEG	0.00459385	1
nonDEG	-0.3419284	0.4787827	1	nonDEG	0.10024742	0.79705475
nonDEG	-0.1557633	0.71348373	1	nonDEG	0.06162914	0.8681434
nonDEG	0.53788085	0.57712793	1	nonDEG	-0.1048979	0.90875891
nonDEG	-0.3070568	0.62217056	1	nonDEG	-1.0747359	0.07318532
nonDEG	-0.2110837	0.61470397	1	nonDEG	0.36086122	0.31492657
nonDEG	0.34125204	0.47142314	1	nonDEG	0.04169667	0.92198408
nonDEG	-0.0733831	0.87473776	1	nonDEG	-0.4891781	0.21655608
nonDEG	-0.2251417	0.6158908	1	nonDEG	-0.1726342	0.66093809
nonDEG	0.67889483	0.45345846	1	nonDEG	0.02510766	0.98685376
nonDEG	0.49782691	0.25828894	1	nonDEG	0.29905432	0.43517343

nonDEG	-0.0901698	0.93074364	1	nonDEG	0.52408421	0.56552782
nonDEG	-0.0835955	0.84660817	1	nonDEG	-0.0385305	0.91547682
nonDEG	-0.4851717	0.24875371	1	nonDEG	0.2242058	0.56914098
nonDEG	0.08685886	0.88087235	1	nonDEG	-0.0999551	0.8554967
nonDEG	0.01569383	0.97164791	1	nonDEG	0.09154033	0.80176424
nonDEG	-0.1128125	0.80243248	1	nonDEG	0.16093626	0.69030027
nonDEG	0.86155982	0.16169713	1	nonDEG	-0.5958828	0.27724192
nonDEG	-0.4728888	0.27999672	1	nonDEG	0.20501195	0.59791825
nonDEG	-1.3427566	0.58383369	1	nonDEG	1.0660139	0.71283941
nonDEG	0.19681982	0.68735395	1	nonDEG	-0.6015807	0.13720886
nonDEG	0.22286874	0.61350693	1	nonDEG	-0.3641475	0.33098678
nonDEG	-0.1343659	0.76139625	1	nonDEG	-0.2337633	0.57956126
nonDEG	0.6508372	0.26057645	1	nonDEG	0.06008653	0.91733204
nonDEG	0.0987389	0.84509085	1	nonDEG	-0.337747	0.47910572
nonDEG	0.08951333	0.94433733	1	nonDEG	3.11278915	0.00960196
nonDEG	0.08788421	0.84891982	1	nonDEG	-0.0705866	0.86253049
nonDEG	-0.0032719	0.99438548	1	nonDEG	-0.0125969	0.97278638
nonDEG	0.88958865	0.04864203	1	nonDEG	0.08486462	0.82354806
nonDEG	0.22771802	0.78318649	1	nonDEG	-0.296084	0.78129909
nonDEG	0.56651318	0.20247879	1	nonDEG	-0.0664047	0.85799045
nonDEG	0.00806292	0.98557029	1	nonDEG	-0.6115665	0.09218242
nonDEG	0.58498446	0.42564896	1	nonDEG	-0.3413832	0.64663477
nonDEG	0.24292632	0.57748321	1	nonDEG	0.07571897	0.84063873
nonDEG	-0.2259151	0.59781773	1	nonDEG	-0.2574375	0.47636619
nonDEG	-0.0075597	0.98663777	1	nonDEG	0.0017588	0.99693501
nonDEG	-0.0199799	1	1	nonDEG	-0.4465059	0.63157568
nonDEG	-0.1158904	0.84244523	1	nonDEG	0.14799215	0.79906148
nonDEG	-0.3364163	0.43525031	1	nonDEG	0.04096036	0.91097203
nonDEG	-0.4065507	0.38058381	1	nonDEG	-0.3053101	0.42302998
nonDEG	-0.4969411	0.24769915	1	nonDEG	0.1871298	0.6098192
nonDEG	-0.1791556	0.79004715	1	nonDEG	0.38848651	0.55453962
nonDEG	-0.118741	0.78653312	1	nonDEG	-0.448285	0.23029405
nonDEG	0.28964487	0.6735429	1	nonDEG	0.43804672	0.48046025
nonDEG	-0.3901386	0.50814102	1	nonDEG	0.04529561	0.94698713
nonDEG	0.0904188	0.83619264	1	nonDEG	0.03130087	0.93235791
nonDEG	0.70493693	0.09781174	1	nonDEG	0.00605226	0.98738541
nonDEG	-0.4916915	0.30694394	1	nonDEG	-0.2963651	0.53366588
nonDEG	0.48736338	0.61174265	1	nonDEG	-0.1530673	0.88081378
nonDEG	0.0396706	0.93731262	1	nonDEG	-0.7444737	0.06585018
nonDEG	-0.2000266	0.64740556	1	nonDEG	0.17205131	0.65685471
nonDEG	0.53830914	0.39377873	1	nonDEG	-0.3080533	0.57729585
nonDEG	-0.4472009	0.32532589	1	nonDEG	0.16909733	0.65512378
nonDEG	0.40411065	0.55747567	1	nonDEG	-0.3573338	0.6251503

nonDEG	-0.2599427	0.54712539	1	nonDEG	-0.1927898	0.60722796
nonDEG	-0.3222117	0.4635329	1	nonDEG	0.22320454	0.57188031
nonDEG	0.13271559	0.75728501	1	nonDEG	0.14749661	0.68535001
nonDEG	-0.2235952	0.66515854	1	nonDEG	0.08349703	0.86236045
nonDEG	-0.1167449	0.85609264	1	nonDEG	-0.6161679	0.36890969
nonDEG	0.09614432	0.82313114	1	nonDEG	-0.1703263	0.64254283
nonDEG	0.03402147	0.94117267	1	nonDEG	0.0220059	0.95690414
nonDEG	-0.0128337	0.97960861	1	nonDEG	-0.0541509	0.88883489
nonDEG	-1.0805667	0.47770935	1	nonDEG	1.03254674	0.58185194
nonDEG	-0.1549732	0.71116586	1	nonDEG	-0.0664401	0.85332719
nonDEG	1.63556987	0.18581155	1	nonDEG	-1.4483207	0.41211199
nonDEG	0.02916417	0.94606833	1	nonDEG	-0.1850455	0.6095719
nonDEG	-0.0283404	0.94916472	1	nonDEG	0.11281361	0.7580523
nonDEG	-0.0848956	0.84187042	1	nonDEG	0.2479862	0.49503612
nonDEG	-0.0024728	0.99611919	1	nonDEG	0.10764746	0.76314227
nonDEG	0.23019794	0.65994635	1	nonDEG	-0.7094835	0.09628728
nonDEG	-0.0562573	0.89560582	1	nonDEG	0.06727913	0.85219613
nonDEG	-0.1467553	0.86254287	1	nonDEG	0.04144876	0.96528066
nonDEG	0.11589032	0.85579871	1	nonDEG	0.0058137	0.99583601
nonDEG	-0.3732173	0.40913497	1	nonDEG	-0.4786421	0.22992569
nonDEG	-0.2842528	0.53284839	1	nonDEG	0.22467439	0.58461322
nonDEG	0.0799249	0.85828654	1	nonDEG	-0.1707434	0.66371909
nonDEG	0.03972403	0.92752383	1	nonDEG	0.13466601	0.71617731
nonDEG	-1.047567	0.18594319	1	nonDEG	2.19941866	0.0066425
nonDEG	-0.3631874	0.45355311	1	nonDEG	0.09494933	0.83143541
nonDEG	-0.2284561	0.63936776	1	nonDEG	-0.5407881	0.26542761
nonDEG	-0.0119098	0.98873576	1	nonDEG	0.05529344	0.93236394
nonDEG	0.23307737	0.72236367	1	nonDEG	-0.0951446	0.8928271
nonDEG	1.00222322	0.30329405	1	nonDEG	-0.681666	0.51169378
nonDEG	0.0760035	0.85777404	1	nonDEG	-0.1612415	0.6662574
nonDEG	0.1273058	0.84181945	1	nonDEG	0.09343315	0.86227579
nonDEG	0.12646499	0.76684656	1	nonDEG	-0.2818944	0.45220624
nonDEG	-0.5501413	0.60835129	1	nonDEG	0.9486885	0.40733098
nonDEG	-0.2668062	0.54659756	1	nonDEG	0.11305355	0.75781099
nonDEG	0.0058079	0.9905031	1	nonDEG	0.18601959	0.66948137
nonDEG	-0.2935169	0.50561403	1	nonDEG	-0.1241577	0.73539751
nonDEG	-0.1316733	0.80584204	1	nonDEG	1.48570258	0.00053495
nonDEG	0.01481758	0.97274587	1	nonDEG	-0.1156323	0.75022536
nonDEG	0.40701	0.37018517	1	nonDEG	-0.5270167	0.17342091
nonDEG	0.52165122	0.59639052	1	nonDEG	0.4539942	0.66826577
nonDEG	0.04301695	0.92491714	1	nonDEG	-0.0736619	0.85403919
nonDEG	0.46519704	0.30021181	1	nonDEG	-0.1324286	0.73369095
nonDEG	-0.1437818	0.73569956	1	nonDEG	0.1763645	0.62771442

nonDEG	0.11979691	0.82458208	1	nonDEG	0.49246034	0.30721893
nonDEG	-0.526006	0.25301976	1	nonDEG	0.18586287	0.63609291
nonDEG	0.05624111	0.90149533	1	nonDEG	-0.2150232	0.60531934
nonDEG	-0.2589018	0.55592347	1	nonDEG	0.01082372	0.97781971
nonDEG	0.07699717	0.85851522	1	nonDEG	-0.120527	0.73895486
nonDEG	0.16363428	0.70708829	1	nonDEG	0.11682933	0.7516219
nonDEG	0.54031213	0.23171443	1	nonDEG	-0.6134063	0.1046764
nonDEG	-0.3803052	0.37300559	1	nonDEG	-0.2087651	0.58483301
nonDEG	1.74798809	0.02828193	1	nonDEG	-1.2054559	0.10819006
nonDEG	-0.0105224	0.9899719	1	nonDEG	-0.3285115	0.59576253
nonDEG	0.23008997	0.58912858	1	nonDEG	0.04828022	0.89365565
nonDEG	-0.1029272	0.82463026	1	nonDEG	0.67900625	0.0854487
nonDEG	-0.1848786	0.68623676	1	nonDEG	0.11454625	0.7572541
nonDEG	-0.3150975	0.59987875	1	nonDEG	0.03600343	0.95512248
nonDEG	-0.6141349	0.1678057	1	nonDEG	0.02438886	0.95547522
nonDEG	-0.7416479	0.1271389	1	nonDEG	0.16594928	0.673789
nonDEG	-0.4329793	0.44383304	1	nonDEG	0.58299823	0.24112611
nonDEG	0.25028293	0.69674198	1	nonDEG	0.07577447	0.88250318
nonDEG	-0.0907702	0.83565578	1	nonDEG	0.20350056	0.57964349
nonDEG	0.12889835	0.80467756	1	nonDEG	-0.6606763	0.18522379
nonDEG	0.13604235	0.75093981	1	nonDEG	-0.1379732	0.70461044
nonDEG	0.10371297	0.80815938	1	nonDEG	-0.0735889	0.84190662
nonDEG	0.4140372	0.58443296	1	nonDEG	0.19574337	0.79267195
nonDEG	0.54335089	0.39513618	1	nonDEG	-0.9187604	0.08345062
nonDEG	-0.6267057	0.60259284	1	nonDEG	-0.0749029	1
nonDEG	-0.0666927	0.91077083	1	nonDEG	-0.3865162	0.4657002
nonDEG	0.31834543	0.47465437	1	nonDEG	0.57283972	0.12347662
nonDEG	0.07238513	0.86994619	1	nonDEG	0.3698305	0.31565691
nonDEG	0.39577235	0.62101895	1	nonDEG	0.18717022	0.80537095
nonDEG	0.2503425	0.68421436	1	nonDEG	0.46313199	0.39048167
nonDEG	-0.2327771	0.59169563	1	nonDEG	0.14981293	0.68743699
nonDEG	0.15437827	0.7818686	1	nonDEG	0.08636401	0.87268601
nonDEG	0.29106713	0.54298677	1	nonDEG	-0.3299537	0.41661428
nonDEG	-0.0651986	0.88007633	1	nonDEG	0.17533725	0.63858028
nonDEG	-0.2878436	0.60978703	1	nonDEG	-0.5580396	0.30070549
nonDEG	-0.4018231	0.34160006	1	nonDEG	0.22637258	0.52925415
nonDEG	-0.3558481	0.56662923	1	nonDEG	0.65711977	0.26073916
nonDEG	-0.3123282	0.47710263	1	nonDEG	-0.4164774	0.26357553
nonDEG	0.12245168	0.78490817	1	nonDEG	-0.0034106	0.99416039
nonDEG	0.68501957	0.24152731	1	nonDEG	-0.2108476	0.72342291
nonDEG	-0.0512758	0.91646148	1	nonDEG	0.19737054	0.63449208
nonDEG	0.23896132	0.57780791	1	nonDEG	-0.16394	0.64967344
nonDEG	-0.6034136	0.44102334	1	nonDEG	0.56366473	0.48996259

nonDEG	-0.0889401	0.84276529	1	nonDEG	-0.0384557	0.92363252
nonDEG	-0.0091491	0.98401351	1	nonDEG	-0.0442443	0.91023987
nonDEG	-0.0759063	0.86025641	1	nonDEG	-0.4683643	0.24527571
nonDEG	1.83086685	0.3213785	1	nonDEG	-0.2819937	0.89048952
nonDEG	0.03347848	0.96124381	1	nonDEG	-0.3511181	0.54498438
nonDEG	0.72488773	0.10231709	1	nonDEG	-0.2635659	0.4867551
nonDEG	0.09980573	0.82621577	1	nonDEG	-0.4785731	0.24601601
nonDEG	-0.0939855	0.83027341	1	nonDEG	-0.0410677	0.90975636
nonDEG	-0.3596996	0.44878743	1	nonDEG	-0.5677692	0.23540279
nonDEG	0.15174553	0.72512622	1	nonDEG	0.10018082	0.78841306
nonDEG	-0.3237748	0.47405773	1	nonDEG	0.26398502	0.50599242
nonDEG	0.1892258	0.67368086	1	nonDEG	-0.1011826	0.78463344
nonDEG	0.16394953	0.75838295	1	nonDEG	-0.4643964	0.2888941
nonDEG	2.06816281	0.09807894	1	nonDEG	0.31763159	0.78700117
nonDEG	0.74758255	0.37420334	1	nonDEG	-0.9532165	0.30236151
nonDEG	-0.134825	0.79510141	1	nonDEG	0.16938113	0.73536107
nonDEG	0.13623353	0.7579252	1	nonDEG	-0.260065	0.47672878
nonDEG	0.32212338	0.50169597	1	nonDEG	-0.3803476	0.34139872
nonDEG	0.04380068	0.93796926	1	nonDEG	-0.1311145	0.81578933
nonDEG	-3.1305677	0.64065911	1	nonDEG	4.9211971	0.26253096
nonDEG	-0.0583287	0.90559394	1	nonDEG	0.07566456	0.86446207
nonDEG	-0.1327796	0.75367791	1	nonDEG	0.02970559	0.93507057
nonDEG	0.34407686	0.43871156	1	nonDEG	0.02771068	0.94465705
nonDEG	-0.1550055	0.73227011	1	nonDEG	-0.3622375	0.36057361
nonDEG	0.05825866	0.89139694	1	nonDEG	0.20984794	0.56303996
nonDEG	-0.5231114	0.36047824	1	nonDEG	-0.315933	0.59509602
nonDEG	-0.1951168	0.67238629	1	nonDEG	-0.1036805	0.8098861
nonDEG	0.05502362	0.90607676	1	nonDEG	-0.3974421	0.29507166
nonDEG	-0.1836631	0.82379141	1	nonDEG	0.84382995	0.24491642
nonDEG	0.01880657	0.97248978	1	nonDEG	0.18919664	0.64515761
nonDEG	0.01251753	0.97794249	1	nonDEG	-0.0931901	0.80637464
nonDEG	-0.5655231	0.23656007	1	nonDEG	-0.0170611	0.9736917
nonDEG	-0.1116231	0.80220377	1	nonDEG	0.21243459	0.58330866
nonDEG	-0.5481088	0.27341586	1	nonDEG	0.10085273	0.83634257
nonDEG	-0.0483865	0.90901483	1	nonDEG	0.02584683	0.94440791
nonDEG	0.1438111	0.74707372	1	nonDEG	-0.0351657	0.92502022
nonDEG	0.23034391	0.60446432	1	nonDEG	-0.2405258	0.51167981
nonDEG	0.03443306	0.94356672	1	nonDEG	-0.2244078	0.5807641
nonDEG	0.00755625	0.98792891	1	nonDEG	0.28976358	0.44686215
nonDEG	0.13276263	0.83325132	1	nonDEG	-0.33583	0.57991259
nonDEG	-0.0269755	0.95243642	1	nonDEG	0.13506011	0.7150028
nonDEG	-0.7115448	0.1768797	1	nonDEG	0.0965833	0.85440425
nonDEG	-0.1453018	0.74368643	1	nonDEG	-0.245649	0.49707186

nonDEG	0.12258001	0.77537155	1	nonDEG	-0.3487585	0.34023894
nonDEG	-0.1217663	0.77401329	1	nonDEG	0.02390336	0.94769876
nonDEG	0.08970714	0.83871154	1	nonDEG	-0.1746165	0.63912883
nonDEG	0.09375022	0.85041848	1	nonDEG	-0.1521311	0.70924216
nonDEG	-5.7378281	0.03953079	1	nonDEG	3.92997983	0.43957209
nonDEG	0.01124347	0.98785278	1	nonDEG	-0.0628537	0.91040074
nonDEG	-0.4800331	0.26435278	1	nonDEG	-0.7500759	0.17119569
nonDEG	0.40293158	0.33495358	1	nonDEG	-0.274906	0.44446257
nonDEG	-0.0657946	0.87790043	1	nonDEG	-0.1257403	0.72840063
nonDEG	-0.0127637	0.97710028	1	nonDEG	-0.1143056	0.75794255
nonDEG	0.29453444	0.49918048	1	nonDEG	0.07496195	0.83659622
nonDEG	0.0505653	0.91339175	1	nonDEG	-0.2135218	0.6359475
nonDEG	0.44765125	0.44509788	1	nonDEG	-0.1848577	0.73844647
nonDEG	-0.1990791	0.64308357	1	nonDEG	0.05624321	0.87711896
nonDEG	0.25574365	0.55832711	1	nonDEG	-0.4787312	0.20499566
nonDEG	0.05282367	0.91545371	1	nonDEG	-0.2686779	0.52893912
nonDEG	-0.1933625	0.72894046	1	nonDEG	-0.0945871	0.85949877
nonDEG	0.16682017	0.69834568	1	nonDEG	0.04414592	0.9045997
nonDEG	0.60826052	0.62079943	1	nonDEG	-1.090598	0.38222014
nonDEG	-0.0741938	1	1	nonDEG	-3.2224449	0.67900441
nonDEG	-0.1995996	0.66729965	1	nonDEG	0.02208108	0.95587428
nonDEG	-0.2667018	0.55601155	1	nonDEG	-0.3752353	0.397249
nonDEG	0.35327285	0.45840085	1	nonDEG	-0.1427102	0.73386215
nonDEG	-0.3178774	0.46920552	1	nonDEG	0.15749969	0.68685844
nonDEG	0.1818656	0.67309031	1	nonDEG	0.02133107	0.95614845
nonDEG	-0.0863589	0.84338468	1	nonDEG	-0.0745014	0.85063301
nonDEG	1.47770795	0.01149902	0.84842013	nonDEG	-0.0678289	0.90805477
nonDEG	0.33239292	0.47534592	1	nonDEG	-0.3093918	0.45397209
nonDEG	5.31129503	0.04513746	1	nonDEG	-2.4575505	0.2751873
nonDEG	0.18546444	0.81246816	1	nonDEG	0.10151392	0.90624755
nonDEG	0.09122021	0.85711267	1	nonDEG	0.53269117	0.22955473
nonDEG	0.37672418	0.46390995	1	nonDEG	-0.0620161	0.88836202
nonDEG	-0.0048063	0.99146349	1	nonDEG	-0.180727	0.61781683
nonDEG	0.19025947	0.66136001	1	nonDEG	0.09186528	0.80465562
nonDEG	0.35470634	0.40671466	1	nonDEG	-0.1882321	0.60964227
nonDEG	-0.0474582	0.91546782	1	nonDEG	-0.2061697	0.56996922
nonDEG	0.37491385	0.56491863	1	nonDEG	0.01216957	0.99001842
nonDEG	0.20568113	0.67060666	1	nonDEG	-0.084006	0.8283057
nonDEG	-0.0868665	0.84367176	1	nonDEG	-0.1291405	0.75254141
nonDEG	-0.6577165	0.31600939	1	nonDEG	1.02927414	0.03885439
nonDEG	0.13766245	0.74667358	1	nonDEG	-0.1021848	0.78061601
nonDEG	0.23493366	0.59052745	1	nonDEG	0.03306555	0.93154611
nonDEG	-0.5795527	0.20815427	1	nonDEG	0.32550582	0.39762808

nonDEG	0.35356209	0.52239886	1	nonDEG	-0.3436843	0.44946837
nonDEG	-0.7442341	0.26673444	1	nonDEG	-0.0214114	0.97796091
nonDEG	-0.1830132	0.68930902	1	nonDEG	-0.0134179	0.9773232
nonDEG	-0.1189648	0.78404419	1	nonDEG	0.12426007	0.73795348
nonDEG	0.29820057	1	1	nonDEG	-1.075017	0.85728017
nonDEG	-0.2108224	0.74288114	1	nonDEG	0.16469212	0.77938472
nonDEG	-0.0468328	0.91275294	1	nonDEG	-0.3138879	0.4064417
nonDEG	-0.4626082	0.50613084	1	nonDEG	0.9128329	0.17624024
nonDEG	0.02676147	0.95018954	1	nonDEG	-0.4872321	0.19619586
nonDEG	0.5062022	0.23882234	1	nonDEG	-0.0243763	0.9468548
nonDEG	0.13437577	0.75365759	1	nonDEG	-0.0199197	0.95635396
nonDEG	0.16400565	0.72112458	1	nonDEG	-0.3367142	0.41122363
nonDEG	0.60379773	0.34855192	1	nonDEG	-0.7153714	0.16008737
nonDEG	-0.0532265	0.9014721	1	nonDEG	-0.0217555	0.9529656
nonDEG	-0.1463024	0.73420948	1	nonDEG	-0.1466051	0.69233608
nonDEG	-0.1084788	0.79922649	1	nonDEG	-0.0519719	0.88674434
nonDEG	-0.2534118	0.59179076	1	nonDEG	-0.1967653	0.61727644
nonDEG	-0.0289276	0.95097988	1	nonDEG	-0.0376184	0.93162233
nonDEG	-0.1998049	0.67421215	1	nonDEG	-0.048645	0.9166975
nonDEG	0.06415297	0.88493011	1	nonDEG	-0.1638422	0.67266392
nonDEG	0.55401251	0.29623929	1	nonDEG	-0.0531073	0.91596564
nonDEG	-0.2575567	0.54055575	1	nonDEG	0.2064077	0.56905195
nonDEG	0.55666162	0.22820545	1	nonDEG	-0.2281732	0.54099682
nonDEG	-0.0088208	0.98457495	1	nonDEG	0.09619902	0.79295692
nonDEG	0.20965153	0.67972968	1	nonDEG	-0.5151115	0.23779619
nonDEG	0.24295778	0.65624382	1	nonDEG	-0.2778191	0.54291603
nonDEG	0.40516954	0.35328597	1	nonDEG	-0.0592788	0.89535678
nonDEG	-0.3203932	0.48212206	1	nonDEG	0.16368596	0.70118847
nonDEG	-0.3890437	0.36401153	1	nonDEG	-0.0135047	0.97349702
nonDEG	-0.1672696	0.69793319	1	nonDEG	-0.3075683	0.41478041
nonDEG	0.12862333	0.79896873	1	nonDEG	-0.5013818	0.21902785
nonDEG	-0.2153495	0.6154798	1	nonDEG	0.27517392	0.44916771
nonDEG	-0.2352808	0.74778589	1	nonDEG	-0.484648	0.53237115
nonDEG	0.01935589	0.97029418	1	nonDEG	-0.7401293	0.11701717
nonDEG	0.28902001	0.51471928	1	nonDEG	0.03430798	0.92536258
nonDEG	0.70566696	0.28361879	1	nonDEG	0.11842322	0.8501331
nonDEG	0.12392879	0.79630474	1	nonDEG	-0.2548038	0.52130751
nonDEG	0.30349751	0.70912932	1	nonDEG	-0.7453123	0.39649751
nonDEG	-0.551969	0.25866976	1	nonDEG	0.25415698	0.59681388
nonDEG	-0.07476	0.86842443	1	nonDEG	-0.0296635	0.94303643
nonDEG	-0.2019467	0.67619385	1	nonDEG	-0.1487848	0.72207861
nonDEG	-0.0123248	1	1	nonDEG	0.41297997	0.5392056
nonDEG	2.72978376	0.04637452	1	nonDEG	-0.7392733	0.6000821

nonDEG	-0.4596729	0.33386509	1	nonDEG	0.42451256	0.34008277
nonDEG	-0.4259702	0.33053702	1	nonDEG	0.32893734	0.4321633
nonDEG	-0.2809219	0.59899922	1	nonDEG	0.28469782	0.57326845
nonDEG	-1.0803604	0.14643833	1	nonDEG	1.5772239	0.02485613
nonDEG	-0.0722192	0.86712226	1	nonDEG	-0.2524374	0.48660041
nonDEG	-0.1400788	0.92015384	1	nonDEG	0.08772045	0.95816509
nonDEG	0.9030435	0.33472551	1	nonDEG	-0.9797053	0.20198998
nonDEG	-0.0202433	0.96598006	1	nonDEG	0.09231147	0.81808646
nonDEG	-0.6484087	0.60991677	1	nonDEG	-1.5921166	0.25166467
nonDEG	-0.0992998	0.81611264	1	nonDEG	-0.0092165	0.98182371
nonDEG	0.46130228	0.70013898	1	nonDEG	0.08370617	0.94783713
nonDEG	0.03355041	0.95773107	1	nonDEG	0.18727353	0.74582091
nonDEG	-0.1920448	0.65389428	1	nonDEG	0.00088015	0.99831068
nonDEG	0.77843877	0.28747291	1	nonDEG	-0.124986	0.86361744
nonDEG	0.00903113	0.98770768	1	nonDEG	-0.4952182	0.2836702
nonDEG	-1.1746182	0.07697427	1	nonDEG	1.55313166	0.01357189
nonDEG	0.16050047	0.77882678	1	nonDEG	-0.3145522	0.54058795
nonDEG	-0.0896513	0.84644728	1	nonDEG	0.29410852	0.45389062
nonDEG	-0.9207785	0.70820158	1	nonDEG	2.58473387	0.27233743
nonDEG	0.10527231	0.93506374	1	nonDEG	0.56146685	0.53711426
nonDEG	0.25261658	0.57401136	1	nonDEG	-0.3713191	0.38100426
nonDEG	0.08790027	0.84830994	1	nonDEG	-0.0910014	0.82830897
nonDEG	-0.6124966	0.17788747	1	nonDEG	0.02539762	0.94527499
nonDEG	-0.1842275	0.70844889	1	nonDEG	0.22919308	0.62362076
nonDEG	0.08614488	0.93396742	1	nonDEG	0.06873134	0.94719911
nonDEG	-0.0542596	0.90418784	1	nonDEG	-0.0814726	0.83058522
nonDEG	0.32645032	0.66931052	1	nonDEG	0.29863753	0.69122413
nonDEG	-0.0443829	0.91806279	1	nonDEG	0.21728795	0.55763683
nonDEG	0.125473	0.76642201	1	nonDEG	0.20118905	0.58303963
nonDEG	0.69206657	0.52033266	1	nonDEG	0.07887504	0.97665488
nonDEG	-0.0401565	0.96268729	1	nonDEG	-0.0470802	0.96810845
nonDEG	-0.4086936	0.416853	1	nonDEG	0.20307752	0.64080499
nonDEG	-0.0289406	0.94595844	1	nonDEG	0.15992303	0.65970481
nonDEG	-0.1004969	0.81078204	1	nonDEG	0.28359168	0.42876292
nonDEG	0.15660871	0.75416999	1	nonDEG	-0.2380346	0.61156
nonDEG	0.17957903	0.69652979	1	nonDEG	-0.1269212	0.74840245
nonDEG	-0.3401477	0.59962817	1	nonDEG	0.30248477	0.64292449
nonDEG	-0.6076181	0.46402443	1	nonDEG	0.86001304	0.2728649
nonDEG	0.90639867	0.16760203	1	nonDEG	-0.6386168	0.31790007
nonDEG	0.07250606	0.88714194	1	nonDEG	-0.4859238	0.31274056
nonDEG	0.10751672	0.87306614	1	nonDEG	1.54163541	0.00453878
nonDEG	0.22951065	0.62077097	1	nonDEG	0.35414519	0.38360535
nonDEG	0.18963438	0.67742364	1	nonDEG	0.07497652	0.84986585

nonDEG	0.14248951	0.77252917	1	nonDEG	-0.5636821	0.19007008
nonDEG	0.07339538	0.92579554	1	nonDEG	-0.1108061	0.89611181
nonDEG	-0.1785313	0.68394826	1	nonDEG	-0.1643053	0.65164074
nonDEG	0.13283017	1	1	nonDEG	0.73634752	0.6960824
nonDEG	-0.2080372	0.62326866	1	nonDEG	0.29558432	0.4220593
nonDEG	-0.1586258	0.7055406	1	nonDEG	-0.3084857	0.39466342
nonDEG	0.41758815	0.58317777	1	nonDEG	-0.7633739	0.22712746
nonDEG	-0.0287516	0.94652965	1	nonDEG	-0.3003089	0.39938135
nonDEG	-0.1746477	0.68944017	1	nonDEG	-0.0337847	0.92886299
nonDEG	-0.0329267	0.93934881	1	nonDEG	-0.0745768	0.83747443
nonDEG	-0.1140438	0.82702377	1	nonDEG	-0.4409598	0.36339268
nonDEG	0.35426075	0.43186197	1	nonDEG	-0.1744934	0.66303399
nonDEG	-0.0856109	0.83993743	1	nonDEG	0.01853324	0.95959643
nonDEG	0.3166258	0.52553236	1	nonDEG	0.05352333	0.8980474
nonDEG	0.23757769	0.60886991	1	nonDEG	-0.4218194	0.32787152
nonDEG	-0.360657	0.52147096	1	nonDEG	-0.2881509	0.60661143
nonDEG	0.54122393	0.2304814	1	nonDEG	0.99640503	0.00762198
nonDEG	-0.8154837	0.42842141	1	nonDEG	0.64921369	0.57975304
nonDEG	-0.6175865	0.41265768	1	nonDEG	0.40267791	0.61152333
nonDEG	1.64124268	0.15147386	1	nonDEG	-1.7949107	0.16800534
nonDEG	0.0266334	0.95271888	1	nonDEG	-0.2201875	0.56471461
nonDEG	0.65525149	0.7670774	1	nonDEG	-1.8593212	0.49554043
nonDEG	0.22314276	0.6100991	1	nonDEG	0.45714843	0.26869023
nonDEG	0.14002851	0.7852218	1	nonDEG	-0.401344	0.34059731
nonDEG	0.48578849	0.84681055	1	nonDEG	-2.6659826	0.28367128
nonDEG	0.96969325	0.13432111	1	nonDEG	-0.1532174	0.79393837
nonDEG	0.66756067	0.2839596	1	nonDEG	-0.1358689	0.82448761
nonDEG	0.09949099	0.81689614	1	nonDEG	-0.0494739	0.89305262
nonDEG	-0.6183423	0.21327821	1	nonDEG	1.14133025	0.0455479
nonDEG	0.60347718	0.42431607	1	nonDEG	0.09546403	0.90145125
nonDEG	-0.1184657	0.77934376	1	nonDEG	-0.1182247	0.74984614
nonDEG	0.02584727	0.95318383	1	nonDEG	0.07157542	0.84351343
nonDEG	-0.1971235	0.67020518	1	nonDEG	0.2710642	0.52788093
nonDEG	-0.1120095	1	1	nonDEG	-0.0779185	1
nonDEG	-0.3445323	0.42140543	1	nonDEG	-0.1108057	0.76060735
nonDEG	-0.372144	0.64120291	1	nonDEG	0.52133657	0.52901792
nonDEG	-0.6452216	0.49875876	1	nonDEG	0.26221705	0.81654349
nonDEG	1.56100929	0.15408859	1	nonDEG	-0.8336339	0.64004546
nonDEG	-0.9724169	0.83621513	1	nonDEG	-3.2219472	0.68096017
nonDEG	0.14226077	0.74978257	1	nonDEG	-0.3377656	0.35680694
nonDEG	-0.292013	0.4981252	1	nonDEG	0.09916985	0.78985472
nonDEG	6.01791652	0.06045757	1	nonDEG	-0.3546938	0.90483955
nonDEG	-0.5085194	0.44794611	1	nonDEG	0.84042935	0.19244495

nonDEG	0.02716293	0.95685134	1	nonDEG	-0.2246794	0.61705043
nonDEG	0.04884109	0.92519812	1	nonDEG	-0.167055	0.68657932
nonDEG	0.9506661	0.03076602	1	nonDEG	-0.2502705	0.51550058
nonDEG	-0.2235731	0.61640308	1	nonDEG	0.16637882	0.66488628
nonDEG	0.4916319	0.72917733	1	nonDEG	-0.6344247	0.72573561
nonDEG	0.45190082	0.56488935	1	nonDEG	0.31656319	0.68328726
nonDEG	0.09270707	0.83152065	1	nonDEG	-0.4074471	0.26095928
nonDEG	-0.0372396	0.94457187	1	nonDEG	0.08708506	0.86294741
nonDEG	-0.4595171	0.38240008	1	nonDEG	-0.1071465	0.79261886
nonDEG	-0.1193007	0.77944195	1	nonDEG	-0.0476402	0.89513854
nonDEG	0.21752679	0.66610856	1	nonDEG	0.11191687	0.81783941
nonDEG	0.07839401	0.87468908	1	nonDEG	0.01461142	0.97748008
nonDEG	0.21538667	0.65484195	1	nonDEG	0.3420997	0.41261012
nonDEG	0.58390931	0.55767333	1	nonDEG	-0.3582199	0.66953533
nonDEG	0.71405866	0.44371779	1	nonDEG	1.86743329	0.03089004
nonDEG	-0.2229152	0.60710257	1	nonDEG	0.56575624	0.13581953
nonDEG	-0.3535476	0.47757186	1	nonDEG	-0.2443775	0.53387425
nonDEG	-0.060215	0.9024272	1	nonDEG	-0.2193091	0.62882816
nonDEG	-0.7219372	0.24392762	1	nonDEG	0.08194307	0.87091653
nonDEG	0.0096498	0.98257986	1	nonDEG	0.04483806	0.90310885
nonDEG	-0.2331393	0.62155678	1	nonDEG	-0.3120126	0.4487566
nonDEG	-0.0777754	0.87183887	1	nonDEG	0.27091464	0.5291725
nonDEG	-0.405987	0.51851102	1	nonDEG	-0.4620212	0.50003496
nonDEG	-0.3733434	0.51257422	1	nonDEG	1.16487655	0.03102967
nonDEG	0.3335893	0.47729483	1	nonDEG	-0.3477788	0.42480209
nonDEG	0.01838226	0.976144	1	nonDEG	-0.2534991	0.6135397
nonDEG	-0.478567	0.36542631	1	nonDEG	-0.263871	0.54164104
nonDEG	-0.4998534	0.48871678	1	nonDEG	0.42848448	0.55131496
nonDEG	-0.4639259	0.36465512	1	nonDEG	0.79025933	0.09122289
nonDEG	-0.3963695	0.49883901	1	nonDEG	0.42323571	0.45745768
nonDEG	0.06063647	0.89267091	1	nonDEG	0.02594939	0.94515618
nonDEG	-0.2036703	0.62887231	1	nonDEG	0.33827529	0.35168122
nonDEG	-0.5970538	0.23714539	1	nonDEG	0.85341768	0.0583621
nonDEG	0.0717801	0.90429988	1	nonDEG	0.61702234	0.22132636
nonDEG	0.34672408	0.67749087	1	nonDEG	0.32095509	0.70030334
nonDEG	0.46520279	0.84621771	1	nonDEG	-1.2735189	0.52045012
nonDEG	-0.003444	1	1	nonDEG	0.11167359	0.82429409
nonDEG	0.12145081	0.81865412	1	nonDEG	-0.6974303	0.08989524
nonDEG	0.4203305	0.42269602	1	nonDEG	0.06310601	0.89322741
nonDEG	-0.2307369	0.58947372	1	nonDEG	0.25063639	0.49880495
nonDEG	0.11222347	0.79659704	1	nonDEG	-0.1145854	0.76647394
nonDEG	-0.0818942	0.85046141	1	nonDEG	0.00205048	0.99635912
nonDEG	0.67279038	0.37675344	1	nonDEG	-0.8040463	0.32541152

nonDEG	-0.1480108	0.73156907	1	nonDEG	0.44530504	0.26057552
nonDEG	0.11358722	0.80749515	1	nonDEG	-0.2731509	0.53797938
nonDEG	0.08411157	0.84245283	1	nonDEG	-0.3459353	0.34863459
nonDEG	-0.3588735	0.40978055	1	nonDEG	0.02447917	0.94827777
nonDEG	0.24703095	0.66726002	1	nonDEG	-0.1326432	0.81134582
nonDEG	-0.1102284	0.79248956	1	nonDEG	0.04004692	0.9116264
nonDEG	0.18486532	0.6806491	1	nonDEG	-0.2781823	0.46964093
nonDEG	0.13104052	0.75716512	1	nonDEG	-0.1657547	0.6516177
nonDEG	0.63774376	0.14690688	1	nonDEG	-0.4139288	0.27196386
nonDEG	-0.1355205	0.75988879	1	nonDEG	-0.7812443	0.04333347
nonDEG	-0.5781033	0.19257934	1	nonDEG	-0.1903697	0.64820778
nonDEG	0.42467885	0.33440903	1	nonDEG	0.08356873	0.82341762
nonDEG	0.14993181	0.88835848	1	nonDEG	0.00127657	1
nonDEG	0.53782582	0.24067872	1	nonDEG	-0.5242424	0.21302918
nonDEG	0.01547687	0.97140054	1	nonDEG	-0.4153043	0.24934757
nonDEG	-0.3444207	0.46573138	1	nonDEG	-0.3258831	0.45726146
nonDEG	-0.1612402	0.73608494	1	nonDEG	0.30352149	0.49738678
nonDEG	-0.3546663	0.59224872	1	nonDEG	-0.0216568	0.98387992
nonDEG	0.16673933	0.71239994	1	nonDEG	-0.2632631	0.48328868
nonDEG	-0.202587	0.63210317	1	nonDEG	0.1472403	0.68462219
nonDEG	0.07586757	0.866224	1	nonDEG	-0.1398173	0.70715099
nonDEG	-0.0677705	0.87869909	1	nonDEG	-0.2874689	0.42929291
nonDEG	-0.5659573	0.24006559	1	nonDEG	0.7622522	0.07072385
nonDEG	-0.0046434	0.99242762	1	nonDEG	0.06956393	0.85108088
nonDEG	0.09671807	0.81952423	1	nonDEG	-0.1441448	0.69495633
nonDEG	-0.5745138	0.32626438	1	nonDEG	0.25927484	0.65135389
nonDEG	-0.2477282	0.56942998	1	nonDEG	0.06631171	0.86919443
nonDEG	0.04385	1	1	nonDEG	1.73271802	0.11758599
nonDEG	5.76136264	0.08205384	1	nonDEG	-1.4550559	0.47238968
nonDEG	-0.0691172	0.92443115	1	nonDEG	0.4289958	0.48599213
nonDEG	0.01276233	0.97646071	1	nonDEG	0.1927883	0.58992805
nonDEG	-0.1405368	0.7470962	1	nonDEG	0.31774002	0.42263199
nonDEG	-0.4914961	0.4260634	1	nonDEG	0.28288435	0.65215712
nonDEG	-0.059805	0.9046138	1	nonDEG	-0.3661923	0.37190948
nonDEG	-0.0319391	0.94230318	1	nonDEG	-0.8501193	0.02450397
nonDEG	-0.262211	0.60219243	1	nonDEG	0.24063683	0.62286119
nonDEG	0.34265651	0.66692349	1	nonDEG	-0.4414226	0.61420612
nonDEG	-0.09112	0.83360914	1	nonDEG	-0.2656903	0.48221419
nonDEG	-0.1786815	0.70753194	1	nonDEG	0.08988878	0.83758108
nonDEG	0.01106263	0.97991806	1	nonDEG	0.06147091	0.86753313
nonDEG	2.00233592	0.02182384	1	nonDEG	-0.8003028	0.28551614
nonDEG	0.07774908	0.91149988	1	nonDEG	-0.3021948	0.68355048
nonDEG	-0.2776862	0.51730414	1	nonDEG	0.18469758	0.62241799

nonDEG	0.21181674	0.66255385	1	nonDEG	-0.4619081	0.29052953
nonDEG	-1.7162069	0.02892554	1	nonDEG	1.99631602	0.0009503
nonDEG	2.83353917	0.006144	0.73793371	nonDEG	-1.9707549	0.05230058
nonDEG	0.34846498	0.49544678	1	nonDEG	-0.4396639	0.35657703
nonDEG	0.3165532	0.51613711	1	nonDEG	-0.8156781	0.03282726
nonDEG	0.22305161	0.63651485	1	nonDEG	-0.434372	0.3115804
nonDEG	0.18449829	0.66128225	1	nonDEG	-0.5161771	0.15315976
nonDEG	0.1135892	0.81600269	1	nonDEG	-0.4964528	0.22325183
nonDEG	-0.1753281	0.69002251	1	nonDEG	0.17850995	0.62794283
nonDEG	-0.2399182	0.57617395	1	nonDEG	0.0602723	0.86879327
nonDEG	0.29807209	0.54663755	1	nonDEG	-0.6349743	0.12694881
nonDEG	0.37507232	0.46808712	1	nonDEG	-0.5690236	0.24935206
nonDEG	-0.0834879	0.88060654	1	nonDEG	0.25429183	0.61604971
nonDEG	-0.0140416	0.97418707	1	nonDEG	0.20929796	0.56384444
nonDEG	-0.2705412	0.53006945	1	nonDEG	0.24043364	0.50716014
nonDEG	0.20419307	0.70883399	1	nonDEG	0.01247585	0.98477668
nonDEG	1.18954987	0.19309927	1	nonDEG	-0.2497733	0.78579963
nonDEG	0.05221344	0.92891306	1	nonDEG	-0.1155364	0.82424445
nonDEG	0.04801547	0.92412382	1	nonDEG	0.53579426	0.22359907
nonDEG	-0.1371517	1	1	nonDEG	-0.7071166	0.75157424
nonDEG	-0.6731501	0.50714075	1	nonDEG	-0.2106571	0.85723656
nonDEG	-0.2922329	0.49793064	1	nonDEG	-0.0662696	0.85537313
nonDEG	0.40310929	0.36741027	1	nonDEG	-0.2604586	0.479638
nonDEG	0.55042122	0.25992722	1	nonDEG	-0.2661868	0.52433826
nonDEG	-0.3822409	0.38325428	1	nonDEG	-0.1586184	0.69567008
nonDEG	-0.2717843	0.56042101	1	nonDEG	-0.3294261	0.39934191
nonDEG	-0.2254018	0.62259535	1	nonDEG	0.17678256	0.62895984
nonDEG	-0.1811536	0.70115121	1	nonDEG	0.7884276	0.06456706
nonDEG	0.59250484	0.32019414	1	nonDEG	-0.1834581	0.74260681
nonDEG	0.09874192	0.84742087	1	nonDEG	0.08555133	0.81912919
nonDEG	-0.3020082	0.87650048	1	nonDEG	0.48428654	0.77541293
nonDEG	0.20896786	0.66261182	1	nonDEG	0.4230845	0.32564938
nonDEG	-0.1479448	0.77080561	1	nonDEG	0.07240846	0.86640996
nonDEG	0.37258845	0.40262181	1	nonDEG	-0.3205782	0.39368296
nonDEG	-4.0058174	0.02323863	1	nonDEG	4.05763362	0.01383553
nonDEG	-0.7324933	0.23118301	1	nonDEG	0.51365389	0.37767821
nonDEG	-0.4060414	0.59755049	1	nonDEG	0.83399825	0.23101874
nonDEG	-0.0379072	0.93146704	1	nonDEG	0.13159542	0.72508156
nonDEG	-0.1278127	0.78420267	1	nonDEG	0.30501084	0.44414249
nonDEG	-0.0718142	0.90942224	1	nonDEG	-0.1789999	0.75956841
nonDEG	0.82709031	0.57358493	1	nonDEG	-3.6995222	0.07617477
nonDEG	-0.5882467	0.21516561	1	nonDEG	0.04334422	0.91626955
nonDEG	0.04605429	0.92722236	1	nonDEG	0.11322193	0.80490408

nonDEG	-0.0685965	0.91578415	1	nonDEG	-0.2835525	0.69034024
nonDEG	-0.4735535	0.26987529	1	nonDEG	0.08192332	0.81876114
nonDEG	-0.1517664	0.73939842	1	nonDEG	-0.174162	0.66834996
nonDEG	0.40924856	0.42477735	1	nonDEG	0.3670937	0.34107586
nonDEG	1.01878169	0.08775672	1	nonDEG	-0.0869218	0.87450188
nonDEG	-0.039534	0.92676906	1	nonDEG	-0.1206669	0.74294586
nonDEG	0.41909733	0.91283013	1	nonDEG	0.37356853	0.94922423
nonDEG	-1.1028405	0.1640457	1	nonDEG	-0.473351	0.62936514
nonDEG	0.20765515	0.68098667	1	nonDEG	0.12972923	0.73786679
nonDEG	-1.3467546	0.38153321	1	nonDEG	-0.0196702	1
nonDEG	0.05600605	0.91546082	1	nonDEG	0.46859921	0.32054464
nonDEG	0.17247442	0.68870779	1	nonDEG	-0.1242902	0.73313712
nonDEG	-0.1907348	0.6840349	1	nonDEG	0.20326721	0.64998073
nonDEG	0.01145418	0.9847047	1	nonDEG	0.2056525	0.65518919
nonDEG	-0.4131111	0.43141795	1	nonDEG	0.05780727	0.91519204
nonDEG	-0.1159052	0.81418829	1	nonDEG	0.77702192	0.08561874
nonDEG	-0.0278601	0.97999255	1	nonDEG	-0.4108181	0.61313365
nonDEG	-0.0527435	0.90229467	1	nonDEG	-0.050046	0.89471898
nonDEG	0.02983907	0.95996606	1	nonDEG	-0.4366977	0.36710662
nonDEG	-0.3152797	0.50600689	1	nonDEG	0.28145964	0.49667699
nonDEG	0.08740708	0.85019793	1	nonDEG	0.20909981	0.60411839
nonDEG	0.16804597	0.92594686	1	nonDEG	1.51694263	0.16549155
nonDEG	0.5475788	0.28680467	1	nonDEG	-0.0630742	0.89355681
nonDEG	0.18097579	0.72787087	1	nonDEG	-0.3187833	0.44329544
nonDEG	0.03838845	0.9390557	1	nonDEG	-0.1069106	0.81860845
nonDEG	-0.0322514	0.94607372	1	nonDEG	-0.0670211	0.86585407
nonDEG	0.05103192	0.90533749	1	nonDEG	0.02344001	0.94864278
nonDEG	0.62827908	0.27177724	1	nonDEG	-0.9096499	0.06554893
nonDEG	-0.1618426	0.70047928	1	nonDEG	-0.0051512	0.98949422
nonDEG	0.58846197	0.34103903	1	nonDEG	0.00333794	1
nonDEG	-0.3119544	0.47508727	1	nonDEG	-0.0345118	0.93715842
nonDEG	-4.0293623	0.35226213	1	nonDEG	-5.13E-15	1
nonDEG	-0.2203482	0.62433819	1	nonDEG	0.38111574	0.34034795
nonDEG	-5.1429396	0.07447417	1	nonDEG	3.05440319	0.69971038
nonDEG	-0.5568397	0.49342803	1	nonDEG	0.36187101	0.64930396
nonDEG	0.369161	0.61974296	1	nonDEG	0.87297204	0.164494
nonDEG	0.40796861	0.45976451	1	nonDEG	0.45078266	0.34992255
nonDEG	-0.2043965	0.63551103	1	nonDEG	-0.1314734	0.72009365
nonDEG	1.22452748	0.47779089	1	nonDEG	1.15175465	0.37642804
nonDEG	-0.3155919	0.44972318	1	nonDEG	0.3101152	0.38713785
nonDEG	0.45114082	0.38964895	1	nonDEG	-0.1289013	0.79193725
nonDEG	1.11037791	0.01004385	0.80348109	nonDEG	-0.3914102	0.2819405
nonDEG	-0.057375	0.89267189	1	nonDEG	0.15694859	0.66630838

nonDEG	-0.5081904	0.5938363	1	nonDEG	1.13915076	0.18918639
nonDEG	-0.4319932	0.38707021	1	nonDEG	0.2064736	0.66485152
nonDEG	0.20019644	0.67227393	1	nonDEG	-0.2430408	0.56034766
nonDEG	-0.3801713	0.44472915	1	nonDEG	0.37819785	0.34983266
nonDEG	0.1826668	0.7499259	1	nonDEG	0.42302777	0.40673996
nonDEG	0.92758399	0.17422017	1	nonDEG	-0.573756	0.32697096
nonDEG	-0.1029771	0.81294339	1	nonDEG	0.12537892	0.7325563
nonDEG	0.24085753	0.58599672	1	nonDEG	0.09970238	0.79541383
nonDEG	0.14631322	0.90973869	1	nonDEG	-0.3578731	0.77219397
nonDEG	0.09997003	0.89946845	1	nonDEG	-0.0866908	0.91379436
nonDEG	0.32511928	0.4516004	1	nonDEG	-0.0852223	0.81711582
nonDEG	0.61146826	0.32127054	1	nonDEG	0.10096612	0.86425602
nonDEG	-2.6136308	0.31833191	1	nonDEG	4.09334484	0.04547119
nonDEG	0.23815755	0.61725726	1	nonDEG	-0.3200405	0.39471937
nonDEG	0.02723428	0.96137265	1	nonDEG	0.6160632	0.20678677
nonDEG	-0.0338184	0.93948096	1	nonDEG	0.28502801	0.45246302
nonDEG	0.02061162	0.96799049	1	nonDEG	-0.3846805	0.40423451
nonDEG	-0.3833543	0.36426374	1	nonDEG	0.17693757	0.63729916
nonDEG	0.4349625	0.50921229	1	nonDEG	-0.7739764	0.24644178
nonDEG	0.21711029	0.63534218	1	nonDEG	-0.1536864	0.71055511
nonDEG	0.32878527	0.52713862	1	nonDEG	-0.1339464	0.76234029
nonDEG	-0.9884275	0.07914352	1	nonDEG	1.07967044	0.10473291
nonDEG	0.99280882	0.0894146	1	nonDEG	-0.3866519	0.42280366
nonDEG	-0.0245655	0.95806904	1	nonDEG	0.05386867	0.88308646
nonDEG	0.3617628	0.42630135	1	nonDEG	-0.1378989	0.73018915
nonDEG	0.06526864	0.89499575	1	nonDEG	-0.2949411	0.47742742
nonDEG	-0.0959863	0.82114641	1	nonDEG	0.02987247	0.93493948
nonDEG	0.22837179	0.66905079	1	nonDEG	-0.4384425	0.33648575
nonDEG	0.27263628	0.54677396	1	nonDEG	-0.0145121	0.97167051
nonDEG	-0.1091123	0.80150279	1	nonDEG	0.1401926	0.70074673
nonDEG	0.32043227	0.90308578	1	nonDEG	0.46033305	0.80678582
nonDEG	-0.0391914	0.92816723	1	nonDEG	-0.3618457	0.32571602
nonDEG	-0.3284142	0.46422256	1	nonDEG	0.18915875	0.60238493
nonDEG	-0.0035395	1	1	nonDEG	1.47121652	0.12730959
nonDEG	0.95600361	0.02960845	1	nonDEG	-0.2484205	0.52114579
nonDEG	0.09147437	0.90451368	1	nonDEG	-0.195935	0.78139053
nonDEG	0.42305246	0.35435736	1	nonDEG	-0.0973711	0.81232307
nonDEG	-1.5668648	0.25939149	1	nonDEG	0.66915694	0.77911203
nonDEG	0.12352269	0.80637217	1	nonDEG	0.11303327	0.80808587
nonDEG	0.3950711	0.34310718	1	nonDEG	0.02841439	0.93800135
nonDEG	-0.1503629	0.74158577	1	nonDEG	0.11318777	0.78481147
nonDEG	-0.2207799	0.62852174	1	nonDEG	-0.3264147	0.40896815
nonDEG	-0.1179045	0.78148594	1	nonDEG	0.2201317	0.54798628

nonDEG	-0.1459406	0.73728337	1	nonDEG	-0.1109493	0.76520988
nonDEG	1.48514177	0.01866398	0.95501418	nonDEG	-0.3498044	0.53943526
nonDEG	0.0825283	0.86801313	1	nonDEG	-0.6570141	0.116275
nonDEG	0.23939082	0.80318132	1	nonDEG	-1.3806063	0.18827736
nonDEG	-0.128909	0.7861798	1	nonDEG	-0.1723476	0.66258791
nonDEG	0.25942896	0.66185204	1	nonDEG	0.32179494	0.5107471
nonDEG	-1.4731399	0.23705872	1	nonDEG	1.2855956	0.35214338
nonDEG	-0.1793878	0.69235886	1	nonDEG	-0.3251682	0.37194587
nonDEG	-0.2250839	0.82770387	1	nonDEG	-1.0401509	0.37206662
nonDEG	-0.0873944	0.83713812	1	nonDEG	-0.0090782	0.98073531
nonDEG	0.31877559	0.45738741	1	nonDEG	-0.2637849	0.46587121
nonDEG	-0.3646617	0.45992128	1	nonDEG	0.14874627	0.73947122
nonDEG	-0.241571	0.56562023	1	nonDEG	0.18949445	0.60260597
nonDEG	0.75341378	0.0944328	1	nonDEG	0.06125918	0.87330325
nonDEG	-0.2973999	0.90925066	1	nonDEG	-2.9918528	0.27121235
nonDEG	0.56652437	0.22917883	1	nonDEG	-0.0999619	0.80046242
nonDEG	-0.1348023	0.75602827	1	nonDEG	0.04748702	0.896708
nonDEG	-2.1418923	0.29517886	1	nonDEG	1.06134551	0.70094771
nonDEG	0.16278489	0.71973454	1	nonDEG	0.10288086	0.78328184
nonDEG	-1.3928213	0.36984037	1	nonDEG	-1.994016	0.22964826
nonDEG	-0.1518335	0.7430815	1	nonDEG	0.1542461	0.69761216
nonDEG	-0.3018011	0.54369713	1	nonDEG	0.09627665	0.83844546
nonDEG	0.28314339	0.60670804	1	nonDEG	-0.0170414	0.97690842
nonDEG	-0.1841944	0.66390135	1	nonDEG	0.26109356	0.48514533
nonDEG	0.07130022	0.86651277	1	nonDEG	-0.2780261	0.46055754
nonDEG	0.86494992	0.27223133	1	nonDEG	-0.565621	0.5011809
nonDEG	0.51604095	0.7565147	1	nonDEG	0.44152704	0.73732013
nonDEG	0.62632406	0.17560388	1	nonDEG	-0.0955659	0.81658945
nonDEG	1.4845874	0.11728176	1	nonDEG	-2.4706636	0.0365523
nonDEG	2.7995227	0.11423475	1	nonDEG	-1.4374519	0.3638544
nonDEG	-0.415833	0.34200265	1	nonDEG	0.15605904	0.67442048
nonDEG	-1.3176946	0.23114903	1	nonDEG	0.70364746	0.62302723
nonDEG	-0.0403676	0.92979728	1	nonDEG	-0.0652082	0.86820996
nonDEG	0.07060534	0.87880512	1	nonDEG	-0.101338	0.78510376
nonDEG	-0.1648139	0.74183113	1	nonDEG	-0.0926617	0.84574754
nonDEG	0.46766939	0.30980979	1	nonDEG	0.14608979	0.72577346
nonDEG	-0.1207954	0.77704042	1	nonDEG	-0.1465664	0.68821855
nonDEG	0.24109824	0.82958962	1	nonDEG	0.49801018	0.64119693
nonDEG	0.13784416	0.7573432	1	nonDEG	-0.1366904	0.7379914
nonDEG	0.3319332	0.48690847	1	nonDEG	-0.1437115	0.72440643
nonDEG	0.21355364	0.62173446	1	nonDEG	-1.0565519	0.00540315
nonDEG	1.27960665	0.0978503	1	nonDEG	-0.8869171	0.1813566
nonDEG	0.12393188	0.7741812	1	nonDEG	0.04441956	0.90627782

nonDEG	-0.1145465	0.79278137	1	nonDEG	-0.1222956	0.74150637
nonDEG	-0.5298195	0.40278691	1	nonDEG	-0.6036644	0.38860465
nonDEG	0.2810994	0.5594235	1	nonDEG	-0.4016307	0.31368589
nonDEG	-1.7918765	0.61880088	1	nonDEG	-2.3845229	1
nonDEG	-4.5766207	0.23095485	1	nonDEG	5.45625622	0.08522173
nonDEG	0.20294103	0.64992045	1	nonDEG	-0.2043069	0.60940218
nonDEG	-0.1511897	0.71987665	1	nonDEG	0.05693586	0.87760866
nonDEG	0.0400845	0.92490997	1	nonDEG	-0.1346639	0.72983919
nonDEG	0.46514871	0.4144601	1	nonDEG	-0.2641536	0.58263652
nonDEG	-0.0482683	0.91315701	1	nonDEG	-0.3930811	0.349011
nonDEG	0.21022146	0.63109585	1	nonDEG	-0.2543375	0.51192724
nonDEG	0.80101231	0.15285817	1	nonDEG	-0.4776749	0.34194597
nonDEG	0.01775751	0.96696252	1	nonDEG	-0.5294716	0.1465089
nonDEG	-0.1332377	0.75613209	1	nonDEG	0.15491939	0.66990856
nonDEG	-0.0024792	0.99583583	1	nonDEG	0.08347871	0.82450364
nonDEG	-0.1583971	0.70563061	1	nonDEG	0.08088892	0.82062374
nonDEG	-0.2267461	0.61407509	1	nonDEG	-0.477665	0.30353736
nonDEG	-0.1330247	0.80071668	1	nonDEG	-0.5975766	0.25876339
nonDEG	0.22024624	0.68466769	1	nonDEG	-0.074984	0.88385434
nonDEG	-0.9173503	0.08821041	1	nonDEG	1.24539985	0.01697495
nonDEG	-0.3065097	0.51083216	1	nonDEG	-0.0994289	0.81937897
nonDEG	-0.1574981	0.7202277	1	nonDEG	0.09787551	0.79115849
nonDEG	-0.4600733	0.7211155	1	nonDEG	-0.5423545	0.70640575
nonDEG	-0.0262176	0.95556282	1	nonDEG	-0.6888745	0.08320228
nonDEG	0.19446947	0.64859873	1	nonDEG	-0.1381267	0.70370456
nonDEG	-1.0277907	0.42548072	1	nonDEG	2.53793212	0.06707756
nonDEG	0.01077396	0.9815032	1	nonDEG	-0.129137	0.7372494
nonDEG	2.01731834	0.0102108	0.80925385	nonDEG	-0.9838189	0.1985682
nonDEG	-1.4829028	0.23991658	1	nonDEG	2.21161215	0.08749349
nonDEG	-0.1042351	0.8261394	1	nonDEG	-0.2252035	0.57210091
nonDEG	0.5387394	0.22779755	1	nonDEG	-0.0238503	0.9489249
nonDEG	-0.0808063	0.86103561	1	nonDEG	-0.395736	0.33461175
nonDEG	-0.205853	0.66622829	1	nonDEG	0.28966271	0.4785646
nonDEG	-0.3767187	0.37495532	1	nonDEG	0.19192357	0.59845976
nonDEG	-0.0345314	0.9391412	1	nonDEG	0.00640423	0.98687527
nonDEG	-0.423107	0.38103519	1	nonDEG	-0.3055502	0.45016301
nonDEG	-0.2602483	0.55115906	1	nonDEG	0.35774805	0.36273888
nonDEG	0.04115808	0.92471999	1	nonDEG	0.041663	0.91113149
nonDEG	0.58774217	0.25882086	1	nonDEG	-0.0026341	0.99785044
nonDEG	0.40924026	0.38117758	1	nonDEG	-0.7728414	0.07607408
nonDEG	0.29141495	0.60409046	1	nonDEG	0.3167819	0.52755019
nonDEG	-0.2858364	0.60796649	1	nonDEG	0.66258685	0.174265
nonDEG	-0.6756812	0.67810714	1	nonDEG	0.91063465	0.51035438

nonDEG	0.1741778	0.68657724	1	nonDEG	-0.0338281	0.92563239
nonDEG	0.32301525	0.74313128	1	nonDEG	-0.1779222	0.83698469
nonDEG	0.08938562	0.8362149	1	nonDEG	-0.041009	0.90994999
nonDEG	-5.8964598	0.07666214	1	nonDEG	-5.13E-15	1
nonDEG	0.64033589	0.50169179	1	nonDEG	-1.0901578	0.21390178
nonDEG	-1.6592567	0.15375104	1	nonDEG	0.19379322	0.94837722
nonDEG	-0.1392183	0.75153035	1	nonDEG	0.11379801	0.76758952
nonDEG	0.22694377	0.5898766	1	nonDEG	0.03078413	0.93264873
nonDEG	0.08213101	0.8541845	1	nonDEG	0.31884722	0.40843786
nonDEG	0.2027191	0.68482181	1	nonDEG	0.29113854	0.51440793
nonDEG	-0.2887123	0.57693509	1	nonDEG	0.21915211	0.67391361
nonDEG	1.32325942	0.50757154	1	nonDEG	-5.1872279	0.09747003
nonDEG	0.25075235	0.58195233	1	nonDEG	-0.1418823	0.70293235
nonDEG	-0.2262174	0.61474887	1	nonDEG	0.20156907	0.60308288
nonDEG	0.39190551	0.48599676	1	nonDEG	-0.0609937	0.90775828
nonDEG	-0.7187737	0.14964347	1	nonDEG	0.52256095	0.23909488
nonDEG	-0.4396194	0.4415276	1	nonDEG	0.46088081	0.40586959
nonDEG	0.00615422	0.98926401	1	nonDEG	-0.1336602	0.71174823
nonDEG	0.27709939	0.53159657	1	nonDEG	0.16623842	0.6639498
nonDEG	-0.319403	0.45392456	1	nonDEG	-0.8441099	0.02403383
nonDEG	-0.2150733	0.62862027	1	nonDEG	0.13802888	0.70472414
nonDEG	0.04873969	0.9125279	1	nonDEG	-0.1345416	0.72391495
nonDEG	1.04825282	0.49346007	1	nonDEG	-0.3482903	0.80535931
nonDEG	-0.2107802	0.72287094	1	nonDEG	0.70228682	0.17363504
nonDEG	0.41546757	0.53634533	1	nonDEG	-0.5322943	0.45060912
nonDEG	0.28258655	0.52036991	1	nonDEG	-0.1802667	0.65456983
nonDEG	0.24335844	0.67131287	1	nonDEG	-0.5901551	0.29150907
nonDEG	0.10952378	0.80744566	1	nonDEG	0.04266168	0.90988888
nonDEG	-0.5536899	0.20312562	1	nonDEG	0.5006251	0.17460101
nonDEG	-0.0304694	0.94295635	1	nonDEG	-0.1932911	0.59630982
nonDEG	0.28710259	0.50815728	1	nonDEG	-0.1890302	0.6058596
nonDEG	-0.4412385	0.51574669	1	nonDEG	0.79554097	0.22703575
nonDEG	-0.1043337	0.84542965	1	nonDEG	-0.1752486	0.74348561
nonDEG	0.43492806	0.38718348	1	nonDEG	-0.1672509	0.7273062
nonDEG	-0.100724	0.82525104	1	nonDEG	0.15132653	0.69293678
nonDEG	-0.0205186	0.97241243	1	nonDEG	-0.1668866	0.73434495
nonDEG	-0.1332458	0.76305846	1	nonDEG	-0.1315884	0.72138985
nonDEG	1.34989442	0.39081716	1	nonDEG	-2.837582	0.12494332
nonDEG	0.09414551	0.82906666	1	nonDEG	0.07542762	0.84295505
nonDEG	-0.3375655	0.4437815	1	nonDEG	0.25311809	0.49920607
nonDEG	-2.1588849	0.1996534	1	nonDEG	1.27918721	0.6240016
nonDEG	-1.2602387	0.23560173	1	nonDEG	0.178091	0.93637879
nonDEG	-0.102653	0.81109127	1	nonDEG	-0.2789115	0.4413691

nonDEG	0.43048594	0.38652696	1	nonDEG	-0.7806305	0.06977217
nonDEG	0.22729378	0.67748923	1	nonDEG	0.02813845	0.95779306
nonDEG	0.74263508	0.21445379	1	nonDEG	-0.5097029	0.33495324
nonDEG	0.18132527	0.67570443	1	nonDEG	-0.1269725	0.72968114
nonDEG	-0.0136798	0.97499289	1	nonDEG	-0.3683742	0.31310654
nonDEG	-0.010862	0.98058561	1	nonDEG	-0.3264008	0.37933463
nonDEG	0.3494586	0.46714939	1	nonDEG	-0.283741	0.44677567
nonDEG	-0.5588445	0.57809599	1	nonDEG	0.89870025	0.39203922
nonDEG	0.21349711	0.62296808	1	nonDEG	-0.1669984	0.64557451
nonDEG	-0.1190321	0.78404828	1	nonDEG	0.25356169	0.49173332
nonDEG	0.4226119	0.70331115	1	nonDEG	0.0426185	0.98146227
nonDEG	0.09952973	0.95905566	1	nonDEG	0.81000612	0.45498698
nonDEG	-0.3551917	0.55478664	1	nonDEG	0.11388454	0.85408618
nonDEG	1.31509226	0.35507317	1	nonDEG	-0.0959831	0.93457653
nonDEG	0.84714373	0.38621414	1	nonDEG	-1.499017	0.21052461
nonDEG	0.05552174	0.89730163	1	nonDEG	-0.1317356	0.72564273
nonDEG	0.01506064	0.97282822	1	nonDEG	0.07302363	0.84221011
nonDEG	-0.1163984	0.7860666	1	nonDEG	0.01681078	0.96322175
nonDEG	-0.0033971	0.99395146	1	nonDEG	-0.1640628	0.64965573
nonDEG	0.66769117	0.11508668	1	nonDEG	-0.1228019	0.73464593
nonDEG	-0.3966318	0.89655308	1	nonDEG	0.13755842	1
nonDEG	0.00186851	0.99683833	1	nonDEG	-0.0625867	0.86431862
nonDEG	-0.4198851	0.45714309	1	nonDEG	0.28047761	0.56655475
nonDEG	0.93954608	0.23687688	1	nonDEG	0.12322115	0.86726571
nonDEG	-0.6311521	0.65050482	1	nonDEG	-0.4700777	0.81427404
nonDEG	-0.7436547	0.11995255	1	nonDEG	0.36938161	0.47472991
nonDEG	-1.3405469	0.29003621	1	nonDEG	1.24628964	0.33508731
nonDEG	-0.1242034	0.76914857	1	nonDEG	0.64582839	0.08559197
nonDEG	0.1636556	0.73008282	1	nonDEG	0.08409734	0.81839867
nonDEG	-0.5126066	0.44888682	1	nonDEG	0.11621886	0.93568527
nonDEG	0.15066576	0.72119519	1	nonDEG	-0.0609586	0.86555439
nonDEG	-0.3356103	0.44896131	1	nonDEG	0.2566108	0.49514662
nonDEG	0.03269772	0.9496402	1	nonDEG	-0.7016439	0.16444007
nonDEG	0.30146844	0.50929763	1	nonDEG	-0.5099557	0.2045359
nonDEG	-1.6061643	0.29786751	1	nonDEG	0.66130715	0.79477192
nonDEG	-0.0439281	0.92421816	1	nonDEG	0.19863743	0.61739778
nonDEG	0.38858436	0.37749159	1	nonDEG	-0.1401229	0.71440522
nonDEG	0.15867269	0.70487928	1	nonDEG	-0.2132896	0.55805224
nonDEG	0.17072266	0.69277317	1	nonDEG	0.03524126	0.9228224
nonDEG	0.10332492	0.8219316	1	nonDEG	-0.5988209	0.12161425
nonDEG	0.06679447	0.89175505	1	nonDEG	0.03419785	0.93922285
nonDEG	-0.0700186	0.86966087	1	nonDEG	0.08393024	0.81905458
nonDEG	-0.4237447	0.43134477	1	nonDEG	0.58532272	0.25167818

nonDEG	-0.6612831	0.23865618	1	nonDEG	0.73864781	0.17683802
nonDEG	0.18777269	0.67144318	1	nonDEG	-0.0836624	0.81993416
nonDEG	-0.0460324	0.91291613	1	nonDEG	-0.118447	0.7406319
nonDEG	-0.3050401	0.56177036	1	nonDEG	-0.0733901	0.89163638
nonDEG	0.08015105	0.85863774	1	nonDEG	0.0417689	0.91400135
nonDEG	-0.1142088	0.80040616	1	nonDEG	0.01305751	0.97267708
nonDEG	-0.1368742	0.7687435	1	nonDEG	-0.158327	0.69197588
nonDEG	0.5620729	0.22865239	1	nonDEG	-0.4441394	0.26093648
nonDEG	0.03024203	0.94429357	1	nonDEG	-0.2249944	0.5407705
nonDEG	0.15537633	0.72942564	1	nonDEG	-0.2402982	0.51957226
nonDEG	0.48179775	0.59476507	1	nonDEG	-0.543756	0.52874582
nonDEG	0.68848659	0.2167493	1	nonDEG	-0.2639323	0.61622188
nonDEG	0.54657006	0.55201483	1	nonDEG	-0.106817	0.92659988
nonDEG	0.33814805	0.43373427	1	nonDEG	0.00786145	0.983133
nonDEG	0.26753615	0.52057519	1	nonDEG	-0.2432881	0.49795021
nonDEG	-0.3942419	0.49457432	1	nonDEG	0.57125154	0.28739182
nonDEG	0.56443476	0.39086368	1	nonDEG	-0.6226315	0.14031995
nonDEG	0.08011178	0.86143934	1	nonDEG	-0.480443	0.23135126
nonDEG	0.02011968	0.96357584	1	nonDEG	-0.0687883	0.8542053
nonDEG	0.43900927	0.43810299	1	nonDEG	-1.0197838	0.03014125
nonDEG	0.33620379	0.45565901	1	nonDEG	-0.5960533	0.12116533
nonDEG	-0.2280423	0.60552673	1	nonDEG	0.16038427	0.68179503
nonDEG	-0.843022	0.3730094	1	nonDEG	1.37806466	0.13972236
nonDEG	-0.4921408	0.57274648	1	nonDEG	0.45766698	0.59058117
nonDEG	0.08953563	0.83105425	1	nonDEG	-0.2022838	0.57376664
nonDEG	0.01323766	0.9758432	1	nonDEG	0.01728447	0.96341153
nonDEG	-0.1348292	0.75164301	1	nonDEG	0.0635163	0.86261053
nonDEG	0.07492327	0.86299122	1	nonDEG	0.02388835	0.94764713
nonDEG	-0.8195936	0.18826897	1	nonDEG	0.12481937	0.83615187
nonDEG	-0.2908648	0.51324533	1	nonDEG	-0.0071026	0.98644444
nonDEG	-0.5035534	0.58915706	1	nonDEG	0.53625898	0.59092026
nonDEG	-0.0328162	0.94887834	1	nonDEG	-0.138612	0.75621076
nonDEG	0.68581679	0.37188404	1	nonDEG	-0.308872	0.69538527
nonDEG	1.756643	0.01170185	0.85229819	nonDEG	-1.1463326	0.08173448
nonDEG	0.52317125	0.33024877	1	nonDEG	-0.0232609	0.9621771
nonDEG	0.09437258	0.83167496	1	nonDEG	-0.1233745	0.75286222
nonDEG	0.16299999	0.74023825	1	nonDEG	0.44067183	0.2479062
nonDEG	-0.1587615	0.71078418	1	nonDEG	-0.0001418	1
nonDEG	-0.2085773	0.62176609	1	nonDEG	-0.260976	0.47576334
nonDEG	-0.1946537	0.6473364	1	nonDEG	-0.2209516	0.55320753
nonDEG	-0.3418654	0.85246633	1	nonDEG	1.66718516	0.22807475
nonDEG	-0.2463052	0.56924287	1	nonDEG	0.25190846	0.48918773
nonDEG	-0.1402655	0.77839571	1	nonDEG	-0.3033881	0.52988689

nonDEG	0.151996	0.72389113	1	nonDEG	0.10387981	0.78198451
nonDEG	-0.2302514	0.59123925	1	nonDEG	-0.3972201	0.33508025
nonDEG	1.91577065	0.14399829	1	nonDEG	-0.7921868	0.53330853
nonDEG	0.28201716	0.51688466	1	nonDEG	0.07018879	0.85363288
nonDEG	-0.4923745	0.46982935	1	nonDEG	0.63918886	0.32203816
nonDEG	-0.310729	0.46881203	1	nonDEG	0.02693506	0.94542772
nonDEG	0.28431819	0.52348995	1	nonDEG	0.01096365	0.97860538
nonDEG	0.86853853	0.31512191	1	nonDEG	-0.4340499	0.64064318
nonDEG	-0.0855583	0.84337079	1	nonDEG	-0.1581131	0.68162613
nonDEG	0.03536254	0.93754595	1	nonDEG	-0.1951692	0.6105349
nonDEG	0.25942826	0.58020929	1	nonDEG	-0.2191151	0.56776404
nonDEG	-0.0123787	0.97806576	1	nonDEG	0.06963914	0.85355808
nonDEG	0.00526077	1	1	nonDEG	-0.2682999	0.84053387
nonDEG	0.04118703	0.92712469	1	nonDEG	-0.0434569	0.90652963
nonDEG	0.26501825	0.53385106	1	nonDEG	0.02491874	0.94745056
nonDEG	0.02454695	0.95775408	1	nonDEG	-0.0145098	0.96986626
nonDEG	0.00684294	0.99098744	1	nonDEG	-1.033768	0.03259484
nonDEG	-2.2381732	0.28257697	1	nonDEG	0.5564696	0.87343664
nonDEG	-0.0592424	0.89108391	1	nonDEG	-0.022146	0.95135067
nonDEG	0.2501115	0.55456238	1	nonDEG	0.02192032	0.95298962
nonDEG	-0.2357266	0.62777722	1	nonDEG	0.88454853	0.02269649
nonDEG	-0.0088501	0.98654164	1	nonDEG	-0.1127199	0.79425989
nonDEG	0.24502646	0.60831969	1	nonDEG	0.06028462	0.89128785
nonDEG	0.08269669	0.85894991	1	nonDEG	-0.1079932	0.79557149
nonDEG	0.36228839	0.7647124	1	nonDEG	-0.2814567	0.79600762
nonDEG	-0.0558811	0.8955491	1	nonDEG	0.05950388	0.87039584
nonDEG	0.13716822	0.75217709	1	nonDEG	-0.3740138	0.32115544
nonDEG	0.08001311	0.89162696	1	nonDEG	-0.7131935	0.12517711
nonDEG	-0.2277061	0.60472335	1	nonDEG	-0.1197543	0.74943661
nonDEG	-0.1334709	0.82060327	1	nonDEG	-0.0226509	0.97182933
nonDEG	1.48279493	0.09654808	1	nonDEG	-0.5069402	0.48410013
nonDEG	0.16496712	0.76945771	1	nonDEG	-0.0021255	1
nonDEG	-0.1960937	0.63951154	1	nonDEG	-0.0916481	0.80203271
nonDEG	-0.0238102	0.95836302	1	nonDEG	-0.3605706	0.3331953
nonDEG	0.16903348	0.77318733	1	nonDEG	-0.1157752	0.83356038
nonDEG	0.34950614	0.41277066	1	nonDEG	-0.3349224	0.38884545
nonDEG	0.1727369	0.69861097	1	nonDEG	0.22869875	0.55054283
nonDEG	0.19751368	0.65465233	1	nonDEG	0.07361364	0.83993991
nonDEG	-0.3316869	0.72004541	1	nonDEG	0.47921853	0.5684849
nonDEG	-0.9794117	0.24474432	1	nonDEG	-3.0485958	0.0152479
nonDEG	-4.0293411	0.35241864	1	nonDEG	2.21589574	1
nonDEG	0.07728703	0.85869415	1	nonDEG	0.0159386	0.96523416
nonDEG	3.40682456	0.02480404	1	nonDEG	-4.3272381	0.02690773

nonDEG	-0.3033203	0.48126927	1	nonDEG	0.26860505	0.45747805
nonDEG	-0.1658264	0.71389016	1	nonDEG	0.09381774	0.80261036
nonDEG	0.26800566	0.6320534	1	nonDEG	0.31421764	0.53422852
nonDEG	0.10985491	0.80501071	1	nonDEG	-0.6303347	0.09884558
nonDEG	-2.2998792	0.23669764	1	nonDEG	1.29638506	0.75341367
nonDEG	0.13080373	0.75779234	1	nonDEG	-0.006183	0.98728628
nonDEG	0.48012752	0.65885686	1	nonDEG	1.66408315	0.10363103
nonDEG	0.17765338	0.68463685	1	nonDEG	-0.5964334	0.13941845
nonDEG	0.54346667	0.22733265	1	nonDEG	-0.3017233	0.43233167
nonDEG	2.37779199	0.22554446	1	nonDEG	1.5532525	0.26349778
nonDEG	-0.1389636	0.75040903	1	nonDEG	0.05030065	0.89057555
nonDEG	-0.0171483	0.972155	1	nonDEG	-0.3847662	0.40402633
nonDEG	-0.3378324	0.44735725	1	nonDEG	0.20187964	0.59590461
nonDEG	1.13551579	0.03821537	1	nonDEG	-0.0482255	0.91663753
nonDEG	-0.1646642	0.74436771	1	nonDEG	0.00962065	0.98607153
nonDEG	-0.1853479	0.66508825	1	nonDEG	-0.3512056	0.35426665
nonDEG	2.46953225	0.02278227	1	nonDEG	-1.5525017	0.17141629
nonDEG	0.05231812	0.90346793	1	nonDEG	-0.1828898	0.6150841
nonDEG	0.49292481	0.40194103	1	nonDEG	-0.163678	0.7447643
nonDEG	-0.329876	0.46257106	1	nonDEG	-0.1768925	0.66633846
nonDEG	-0.1539374	0.73587283	1	nonDEG	0.13800402	0.75046931
nonDEG	-0.1737226	0.74088356	1	nonDEG	0.02390234	0.96692795
nonDEG	-0.016638	0.97692362	1	nonDEG	-0.3008224	0.53144215
nonDEG	-0.2949356	0.6368811	1	nonDEG	0.0236177	0.97472874
nonDEG	-0.4347651	0.3806984	1	nonDEG	-0.0851291	0.85308291
nonDEG	-0.045733	0.92093318	1	nonDEG	-0.014388	0.97262842
nonDEG	-0.2245538	0.60186734	1	nonDEG	0.23569375	0.51696783
nonDEG	-0.0760501	0.8673243	1	nonDEG	-0.1962826	0.63941452
nonDEG	-0.0368188	0.93324391	1	nonDEG	-0.111715	0.76494152
nonDEG	0.30287899	0.52963334	1	nonDEG	-0.0939375	0.81868015
nonDEG	0.0878312	0.86698901	1	nonDEG	-0.0591103	0.90579362
nonDEG	0.37243424	0.39290833	1	nonDEG	-0.1786728	0.63635316
nonDEG	0.11946241	0.78061744	1	nonDEG	0.12604645	0.73221643
nonDEG	0.26774877	0.52080182	1	nonDEG	-0.0950587	0.79561294
nonDEG	-0.0533549	0.90587682	1	nonDEG	0.03229175	0.93805034
nonDEG	-0.4763795	0.42921676	1	nonDEG	0.68542198	0.1677413
nonDEG	0.02617801	0.95559403	1	nonDEG	-0.3043724	0.47745765
nonDEG	-0.26625	0.5245994	1	nonDEG	0.01574893	0.9663681
nonDEG	-0.0080064	0.98570368	1	nonDEG	0.04126236	0.91169264
nonDEG	0.60414767	0.17195855	1	nonDEG	0.00807375	0.98324254
nonDEG	0.77675663	0.47607326	1	nonDEG	-1.3043298	0.28734695
nonDEG	-0.0106249	1	1	nonDEG	1.65446215	0.23054175
nonDEG	0.07760694	0.85548879	1	nonDEG	-0.067179	0.85243065

nonDEG	-0.3103431	0.49380545	1	nonDEG	-0.2830629	0.54314833
nonDEG	-0.0741969	1	1	nonDEG	2.14147701	0.455253
nonDEG	0.15825724	0.79143911	1	nonDEG	-0.1054926	0.85623063
nonDEG	-0.058901	0.89065126	1	nonDEG	-0.0314141	0.9309433
nonDEG	-0.094865	0.85337452	1	nonDEG	0.11085175	0.80490739
nonDEG	0.57026754	0.19278616	1	nonDEG	0.05489532	0.88215519
nonDEG	-0.269225	0.53336222	1	nonDEG	0.29666838	0.41624669
nonDEG	0.322592	0.45421344	1	nonDEG	-0.6573703	0.07809192
nonDEG	-1.0334228	0.22955091	1	nonDEG	1.0619454	0.27203773
nonDEG	-0.4883481	0.28582055	1	nonDEG	-0.2544589	0.58273906
nonDEG	0.21956365	0.71355701	1	nonDEG	-0.255068	0.66543825
nonDEG	0.10825761	0.79904168	1	nonDEG	-0.0439153	0.90453289
nonDEG	1.80003343	0.01934177	0.97153571	nonDEG	-1.2751751	0.07674651
nonDEG	0.05888919	0.89811053	1	nonDEG	-0.5496562	0.1789407
nonDEG	0.44317967	0.29901046	1	nonDEG	-0.3416204	0.3507122
nonDEG	0.17377798	0.68368237	1	nonDEG	0.00151324	0.9973991
nonDEG	0.05815428	0.89058323	1	nonDEG	0.09784168	0.78764004
nonDEG	1.71399661	0.21187274	1	nonDEG	-1.2611894	0.38738894
nonDEG	-1.7462701	0.39242873	1	nonDEG	1.62008269	0.56742618
nonDEG	0.18250462	0.6686495	1	nonDEG	-0.1409269	0.69509681
nonDEG	0.26225543	0.62614526	1	nonDEG	-0.7016154	0.15174994
nonDEG	-0.1756405	0.68118497	1	nonDEG	-0.6588404	0.0698347
nonDEG	-0.6808408	0.40488718	1	nonDEG	0.63725051	0.4398251
nonDEG	0.21879534	0.65637558	1	nonDEG	-0.129101	0.79519653
nonDEG	-0.0286182	0.95022348	1	nonDEG	-0.3015508	0.42428517
nonDEG	-0.3257744	0.61731033	1	nonDEG	0.47910416	0.45867872
nonDEG	-0.2457725	0.58570943	1	nonDEG	-0.1590406	0.67035935
nonDEG	-0.0266637	0.95172467	1	nonDEG	0.11113382	0.76594002
nonDEG	0.48840785	0.46394413	1	nonDEG	0.33427576	0.53971719
nonDEG	0.02748011	0.95119252	1	nonDEG	-0.5511533	0.14727761
nonDEG	0.00330165	0.99469023	1	nonDEG	-0.7774171	0.0515529
nonDEG	-0.1374932	0.79409448	1	nonDEG	1.4192472	0.04387326
nonDEG	-0.3042675	0.48569541	1	nonDEG	0.3550105	0.34246739
nonDEG	0.99184429	0.14772249	1	nonDEG	-0.7085275	0.3715389
nonDEG	0.34528306	0.49110348	1	nonDEG	-0.4206794	0.36280702
nonDEG	0.04159116	0.92395129	1	nonDEG	-0.2669186	0.46972003
nonDEG	0.00023778	1	1	nonDEG	0.02933152	0.93651955
nonDEG	0.03561647	0.9499922	1	nonDEG	0.4687147	0.34033423
nonDEG	0.2569195	0.55701997	1	nonDEG	-0.0834262	0.82416124
nonDEG	0.50380143	0.42454946	1	nonDEG	0.18833447	0.738553
nonDEG	0.12963926	0.81564048	1	nonDEG	-0.8594403	0.04449018
nonDEG	-0.017357	0.96810589	1	nonDEG	-0.0892789	0.80744056
nonDEG	-0.2160739	0.65538883	1	nonDEG	-0.2419582	0.56854893

nonDEG	-0.3752522	0.45399853	1	nonDEG	-0.0459103	0.92740216
nonDEG	0.0165537	0.97740658	1	nonDEG	0.26719864	0.57582812
nonDEG	-0.1091322	0.80902942	1	nonDEG	0.11665334	0.77245699
nonDEG	0.29170335	0.74416862	1	nonDEG	-0.1713392	0.86864416
nonDEG	-0.1152173	0.78586211	1	nonDEG	-0.0257232	0.94323367
nonDEG	0.28331607	0.53527067	1	nonDEG	-0.1120891	0.78474711
nonDEG	-0.2295991	0.58517274	1	nonDEG	0.26321511	0.46810426
nonDEG	0.01686495	0.96928613	1	nonDEG	-0.1245414	0.73198821
nonDEG	0.05689893	0.90749344	1	nonDEG	-0.4003159	0.32151842
nonDEG	-0.2216095	0.61826656	1	nonDEG	0.12963026	0.73210169
nonDEG	0.88511469	0.06192846	1	nonDEG	-0.7036902	0.07950311
nonDEG	1.24311872	0.00842161	0.78074119	nonDEG	-0.3247199	0.43141001
nonDEG	0.35300656	0.68215008	1	nonDEG	-0.0936531	0.9064435
nonDEG	-0.0121568	0.97989458	1	nonDEG	0.09542503	0.81364148
nonDEG	-4.5766696	0.23051023	1	nonDEG	3.96667393	0.43343853
nonDEG	-0.167	0.70551593	1	nonDEG	0.21646197	0.58632941
nonDEG	-0.1055525	0.80853871	1	nonDEG	-0.4027494	0.26944708
nonDEG	-0.0796335	0.86196933	1	nonDEG	0.19204056	0.6363378
nonDEG	-0.0020225	0.99655629	1	nonDEG	-0.0637998	0.86015141
nonDEG	-0.0986187	0.86034281	1	nonDEG	-0.427789	0.43592804
nonDEG	0.93890649	0.03061845	1	nonDEG	-0.756471	0.04144575
nonDEG	0.3426182	0.66619988	1	nonDEG	-0.7694164	0.3752727
nonDEG	-0.1655829	0.74830422	1	nonDEG	0.3540344	0.45118866
nonDEG	0.83263938	0.2059061	1	nonDEG	-0.3185977	0.62788489
nonDEG	-0.1879172	0.67356287	1	nonDEG	-0.4038973	0.35022334
nonDEG	0.62905145	0.18728575	1	nonDEG	-0.0462617	0.91339736
nonDEG	0.29583325	0.66594548	1	nonDEG	-0.3782698	0.60894637
nonDEG	0.19748489	0.65595475	1	nonDEG	-0.2619345	0.47371289
nonDEG	-0.1291554	0.82216342	1	nonDEG	-0.1711193	0.75564231
nonDEG	0.47998607	0.3608903	1	nonDEG	0.67962615	0.12157228
nonDEG	0.41485961	0.46966309	1	nonDEG	-0.3989004	0.412158
nonDEG	-0.3636767	0.40615857	1	nonDEG	0.21689089	0.55362577
nonDEG	-0.0835799	0.84694907	1	nonDEG	0.09105173	0.80250031
nonDEG	-0.6346294	0.21225832	1	nonDEG	1.41844424	0.00571703
nonDEG	-0.2291074	0.58420059	1	nonDEG	-0.2612811	0.46974584
nonDEG	-0.5975785	0.45561703	1	nonDEG	0.15148784	0.8809457
nonDEG	0.19168038	0.65346509	1	nonDEG	-0.0477316	0.89556203
nonDEG	-0.2794169	0.53449418	1	nonDEG	-0.0258556	0.94755435
nonDEG	0.41901089	0.45709022	1	nonDEG	-0.6519563	0.17125278
nonDEG	-0.2066416	0.63171093	1	nonDEG	-0.3694669	0.35604791
nonDEG	-0.0489464	0.91220543	1	nonDEG	-0.4545329	0.26868037
nonDEG	0.38778157	0.45496669	1	nonDEG	-0.7215611	0.11515139
nonDEG	0.15292826	0.71975966	1	nonDEG	0.26793111	0.46650171

nonDEG	0.64811032	0.34868917	1	nonDEG	-0.2808712	0.66732324
nonDEG	0.18900311	0.66263809	1	nonDEG	0.24763575	0.49682892
nonDEG	0.31274773	0.58630074	1	nonDEG	0.05966086	0.91404638
nonDEG	0.41435015	0.3688618	1	nonDEG	-0.2503769	0.52530393
nonDEG	-0.1726467	0.70541815	1	nonDEG	-0.212743	0.60899232
nonDEG	0.35900031	0.39530663	1	nonDEG	0.32984668	0.36612064
nonDEG	-0.2499648	0.60412059	1	nonDEG	-0.191625	0.61674574
nonDEG	0.34509455	0.42133564	1	nonDEG	-0.0075155	0.98398742
nonDEG	0.01229126	0.97763587	1	nonDEG	-0.0977926	0.79117173
nonDEG	-0.0266999	0.9561331	1	nonDEG	-0.2587693	0.5588059
nonDEG	0.18695512	0.72220427	1	nonDEG	-0.2991122	0.54098944
nonDEG	-0.1717937	0.69063452	1	nonDEG	0.29106938	0.43726534
nonDEG	0.33319201	0.50501563	1	nonDEG	0.45021514	0.32908242
nonDEG	0.19441428	0.81972998	1	nonDEG	-0.8613122	0.35507423
nonDEG	0.40110885	0.65487833	1	nonDEG	-0.9696917	0.28307273
nonDEG	0.50456451	0.43069221	1	nonDEG	0.645878	0.25092576
nonDEG	0.0425201	0.9216181	1	nonDEG	-0.1289203	0.72336924
nonDEG	-0.2047912	0.62675852	1	nonDEG	0.09687758	0.79400251
nonDEG	0.1596095	0.7270183	1	nonDEG	-0.8023117	0.04122041
nonDEG	0.1261036	0.81740788	1	nonDEG	-0.4885171	0.26299272
nonDEG	-0.1223652	0.77359595	1	nonDEG	0.21801464	0.54936587
nonDEG	0.65042299	0.44121792	1	nonDEG	-0.4202614	0.58175964
nonDEG	-0.3408924	0.43882416	1	nonDEG	0.22147371	0.54997939
nonDEG	0.60145868	0.18140637	1	nonDEG	-0.4236006	0.24518466
nonDEG	-0.010484	0.98268306	1	nonDEG	0.0170213	0.96734157
nonDEG	0.00271602	0.99531224	1	nonDEG	-0.0893429	0.80688224
nonDEG	0.37999626	0.58547818	1	nonDEG	-1.0477855	0.13783927
nonDEG	0.15407981	0.73338302	1	nonDEG	0.12295841	0.76879023
nonDEG	0.02453037	0.96498567	1	nonDEG	0.47217117	0.3057242
nonDEG	0.96646671	0.07213809	1	nonDEG	0.0869782	0.8538929
nonDEG	-0.0266293	0.97392352	1	nonDEG	0.32445259	0.5890003
nonDEG	0.0958525	0.85399767	1	nonDEG	-0.4127343	0.31108635
nonDEG	-0.1093936	0.82529891	1	nonDEG	-0.1721337	0.71873005
nonDEG	-0.2690074	0.5382903	1	nonDEG	0.11221734	0.76358711
nonDEG	-0.26225	0.54431317	1	nonDEG	-0.0863501	0.81275142
nonDEG	0.27223736	0.52809165	1	nonDEG	0.23840308	0.5170885
nonDEG	1.19876856	0.00517212	0.72223055	nonDEG	-0.6803651	0.06795628
nonDEG	0.05119928	0.91685498	1	nonDEG	-0.1410466	0.72790217
nonDEG	0.12933126	0.7965213	1	nonDEG	-0.239149	0.5883373
nonDEG	-0.1685153	0.70942682	1	nonDEG	0.2101249	0.58697681
nonDEG	-0.4238571	0.33527707	1	nonDEG	0.25879168	0.48106849
nonDEG	-0.0117707	0.97852082	1	nonDEG	0.06151597	0.86785722
nonDEG	0.36097926	0.51802819	1	nonDEG	-0.3727782	0.37427909

nonDEG	-0.5032579	0.77820292	1	nonDEG	-0.8481459	0.88884066
nonDEG	-0.1545602	0.73106274	1	nonDEG	-0.5085741	0.18753212
nonDEG	0.19429534	0.84324582	1	nonDEG	0.38561683	0.68068266
nonDEG	0.35024519	0.42054847	1	nonDEG	-0.3034626	0.41068556
nonDEG	0.08055508	0.86812103	1	nonDEG	-0.8820958	0.02589108
nonDEG	-0.3504582	0.53257964	1	nonDEG	0.03198331	0.95874036
nonDEG	0.18540897	0.71480706	1	nonDEG	-0.0258342	0.95798139
nonDEG	0.06430416	0.88856853	1	nonDEG	-0.1942051	0.63335165
nonDEG	-0.294111	0.50956781	1	nonDEG	-0.0971707	0.79112569
nonDEG	-0.0509888	0.917284	1	nonDEG	-0.1783978	0.69363324
nonDEG	-0.1673729	0.7359248	1	nonDEG	0.48939711	0.24266909
nonDEG	-0.228111	0.59379226	1	nonDEG	-0.0293219	0.93732451
nonDEG	0.57388539	0.287652	1	nonDEG	-0.5328424	0.29681295
nonDEG	-0.0791798	0.86495486	1	nonDEG	-0.2514674	0.52560138
nonDEG	-0.1029002	0.8058999	1	nonDEG	-0.0321082	0.93046483
nonDEG	0.08666887	0.8601312	1	nonDEG	-0.5365998	0.17566021
nonDEG	-0.473467	0.37148778	1	nonDEG	0.27162647	0.59191327
nonDEG	-0.1964307	0.64129945	1	nonDEG	-0.2538892	0.47832325
nonDEG	-0.0739869	0.88357863	1	nonDEG	-0.0704354	0.87754286
nonDEG	0.22605569	0.60281906	1	nonDEG	-0.1104284	0.76277674
nonDEG	-0.1423374	0.77699553	1	nonDEG	-0.6806826	0.1812807
nonDEG	-0.5855508	0.2381097	1	nonDEG	0.01731569	0.97100674
nonDEG	0.15582994	0.70953669	1	nonDEG	-0.0840072	0.83000218
nonDEG	-0.1621137	0.71309783	1	nonDEG	-0.0330844	0.9328742
nonDEG	0.33543231	0.51163098	1	nonDEG	-0.6290493	0.13727115
nonDEG	0.12424313	0.77125692	1	nonDEG	0.10242244	0.781952
nonDEG	-0.6511324	0.2159643	1	nonDEG	-0.4831089	0.31454617
nonDEG	-0.0851725	0.84051611	1	nonDEG	-0.0791028	0.82830281
nonDEG	0.18342351	0.66406008	1	nonDEG	-0.6176695	0.08983033
nonDEG	0.44466689	0.60738965	1	nonDEG	-0.4316518	0.62198262
nonDEG	-0.035427	1	1	nonDEG	-2.3609155	0.22394256
nonDEG	-0.0094504	0.98521529	1	nonDEG	0.32564905	0.40138714
nonDEG	0.45644124	0.41059148	1	nonDEG	0.0843921	0.87476261
nonDEG	-0.2792286	0.58133262	1	nonDEG	-0.1384558	0.78338574
nonDEG	-0.0284031	0.94706599	1	nonDEG	-0.080101	0.82672723
nonDEG	-0.0114082	0.98352429	1	nonDEG	0.06594948	0.89344448
nonDEG	1.06865707	0.13009091	1	nonDEG	-0.2111562	0.765804
nonDEG	-0.6809648	0.3013932	1	nonDEG	0.5452247	0.42984162
nonDEG	0.71509982	0.42172397	1	nonDEG	0.83673328	0.3144933
nonDEG	-0.7092542	0.34425144	1	nonDEG	-0.3158204	0.71800171
nonDEG	-0.0059248	0.99093779	1	nonDEG	0.13544143	0.77107231
nonDEG	0.20304782	0.63875138	1	nonDEG	-0.2515469	0.50452055
nonDEG	-0.2174085	0.61297207	1	nonDEG	0.24441599	0.49885392

nonDEG	-0.119653	0.78214768	1	nonDEG	0.30224288	0.41348104
nonDEG	-0.1862811	0.77800694	1	nonDEG	0.49985431	0.37888649
nonDEG	0.43080259	0.63860152	1	nonDEG	0.44688957	0.59718241
nonDEG	0.95878725	0.03644469	1	nonDEG	-0.1461108	0.7043736
nonDEG	-0.1013265	0.81279398	1	nonDEG	0.03585899	0.92131431
nonDEG	0.10831354	0.80272026	1	nonDEG	-0.3352216	0.36672054
nonDEG	-0.2927422	0.49997874	1	nonDEG	-0.0169359	0.96555619
nonDEG	-4.7993659	0.20488253	1	nonDEG	6.49137964	0.03880526
nonDEG	5.19484317	0.13933533	1	nonDEG	-0.6486773	0.81892612
nonDEG	-0.0071534	0.98733363	1	nonDEG	-0.1320238	0.71369885
nonDEG	0.11352694	0.80330259	1	nonDEG	-0.3414172	0.41093078
nonDEG	-1.7821599	0.43280455	1	nonDEG	5.09236274	0.00728837
nonDEG	-0.0947341	0.85139619	1	nonDEG	0.07531333	0.90814716
nonDEG	-0.7583762	0.14653433	1	nonDEG	0.21097805	0.69150267
nonDEG	-0.4245447	0.62294174	1	nonDEG	-0.325907	0.67085618
nonDEG	0.71037808	0.23209909	1	nonDEG	-0.0671391	0.91125506
nonDEG	0.19918885	0.67506154	1	nonDEG	-0.2120034	0.5942903
nonDEG	0.43613433	0.32169432	1	nonDEG	-0.0353158	0.93658001
nonDEG	-0.2942997	0.54938749	1	nonDEG	-0.2320183	0.59160485
nonDEG	0.09709663	0.83825608	1	nonDEG	-0.4047421	0.32375637
nonDEG	0.15646923	0.75577675	1	nonDEG	-0.5551818	0.18957495
nonDEG	0.03459838	0.94708365	1	nonDEG	-0.0016565	0.99777263
nonDEG	0.03333598	0.93778111	1	nonDEG	-0.0618215	0.86428169
nonDEG	0.63423652	0.29450073	1	nonDEG	-0.1630642	0.75611175
nonDEG	0.05744121	0.90478779	1	nonDEG	-0.2382192	0.58571853
nonDEG	-6.6955366	0.00371733	0.67010784	nonDEG	7.87633283	0.0020314
nonDEG	0.05274254	0.9066048	1	nonDEG	-0.0761441	0.8384727
nonDEG	0.02253233	0.96276761	1	nonDEG	-0.4735898	0.24931361
nonDEG	0.55761652	0.30342635	1	nonDEG	0.0952007	0.85472518
nonDEG	0.16074469	0.71429185	1	nonDEG	-0.2265707	0.58928907
nonDEG	1.65491397	0.0691837	1	nonDEG	-0.2654787	0.75816815
nonDEG	-0.2878117	0.53653631	1	nonDEG	-0.3664633	0.41618998
nonDEG	1.97495071	0.09427081	1	nonDEG	-0.786154	0.5145561
nonDEG	-0.2439226	0.6325685	1	nonDEG	0.153973	0.76075209
nonDEG	0.16312566	0.77042148	1	nonDEG	-0.1754116	0.74508281
nonDEG	0.08523777	0.84249677	1	nonDEG	-0.0345063	0.92428069
nonDEG	-0.1955628	0.68459268	1	nonDEG	0.16664172	0.71292872
nonDEG	-0.0585691	0.89294131	1	nonDEG	-0.2946281	0.43241729
nonDEG	-5.220667	0.05985856	1	nonDEG	6.6825763	0.00973637
nonDEG	-0.2038956	0.67802309	1	nonDEG	-0.0221443	0.96444957
nonDEG	0.17365427	0.71385578	1	nonDEG	0.11305239	0.78743557
nonDEG	0.04367217	0.91708017	1	nonDEG	-0.815009	0.05262995
nonDEG	-0.0747682	0.87067553	1	nonDEG	-0.4967611	0.21861406

nonDEG	-0.117876	0.79042514	1	nonDEG	0.3179188	0.41232757
nonDEG	0.02702789	0.94984611	1	nonDEG	0.04937417	0.89439219
nonDEG	-0.3135534	0.48547914	1	nonDEG	0.13439744	0.71815905
nonDEG	0.35990244	0.48352695	1	nonDEG	0.51824385	0.26819264
nonDEG	2.42025417	0.02577855	1	nonDEG	-0.4692691	0.6520022
nonDEG	0.23138476	0.60363293	1	nonDEG	-0.2827805	0.48510523
nonDEG	-1.0752262	0.4024767	1	nonDEG	2.51534396	0.07081092
nonDEG	0.10280906	0.82039003	1	nonDEG	-0.4064613	0.35225258
nonDEG	0.43573878	0.32051608	1	nonDEG	-0.2574712	0.50044902
nonDEG	-0.0964192	0.82765379	1	nonDEG	-0.7081467	0.05607228
nonDEG	2.26314028	0.18530591	1	nonDEG	-3.1221575	0.14972189
nonDEG	-0.7444803	0.28709201	1	nonDEG	0.75465153	0.24506527
nonDEG	-0.3617187	0.55170004	1	nonDEG	0.01880014	0.98380854
nonDEG	-0.0791393	0.86894502	1	nonDEG	-0.1881034	0.6803971
nonDEG	0.34605479	0.47869827	1	nonDEG	-0.2187707	0.5878822
nonDEG	0.67744876	0.11804065	1	nonDEG	-0.6816258	0.06660217
nonDEG	-0.0288927	0.95013175	1	nonDEG	-0.2684894	0.47669457
nonDEG	-0.0193286	0.96449054	1	nonDEG	0.06926052	0.85244017
nonDEG	0.88505927	0.33367881	1	nonDEG	-0.5091649	0.39025289
nonDEG	0.05592009	0.91117832	1	nonDEG	-0.663986	0.1713206
nonDEG	-0.072868	0.86973089	1	nonDEG	-0.1944453	0.6371273
nonDEG	0.69844075	0.38674978	1	nonDEG	-0.9438459	0.27168978
nonDEG	0.03824893	0.93661511	1	nonDEG	-0.0604007	0.87460765
nonDEG	0.15837772	0.72042866	1	nonDEG	-0.1095915	0.76775262
nonDEG	-0.4458166	0.42363808	1	nonDEG	0.05540629	0.92426462
nonDEG	-1.7611683	0.39990134	1	nonDEG	1.96483526	0.41302851
nonDEG	0.05836565	0.89686679	1	nonDEG	-0.2879099	0.44408334
nonDEG	0.16272947	0.74136843	1	nonDEG	-0.3437172	0.41352571
nonDEG	-0.3019282	0.49841211	1	nonDEG	0.21616858	0.56822306
nonDEG	0	1	1	nonDEG	-5.13E-15	1
nonDEG	-0.0767681	0.85524241	1	nonDEG	-0.0795749	0.82572113
nonDEG	-0.2335111	0.60591652	1	nonDEG	0.16679208	0.68684295
nonDEG	-0.1698388	0.69148901	1	nonDEG	-0.1103418	0.77168242
nonDEG	0.23937958	0.60250297	1	nonDEG	-0.2348838	0.53425692
nonDEG	-0.1768282	0.69148851	1	nonDEG	-0.0914007	0.82533348
nonDEG	0.32342811	0.49879525	1	nonDEG	0.26448083	0.5276195
nonDEG	-1.6292601	0.14537885	1	nonDEG	-0.9686413	0.63645491
nonDEG	-0.1839683	0.66206706	1	nonDEG	0.37099185	0.3095344
nonDEG	0.0804977	0.84982965	1	nonDEG	-0.0724481	0.85506647
nonDEG	-0.0118562	0.978171	1	nonDEG	-0.162903	0.65666584
nonDEG	-0.3061416	0.49373014	1	nonDEG	-0.1216218	0.74911731
nonDEG	-0.113312	0.82043611	1	nonDEG	0.2829599	0.50841356
nonDEG	-0.0678903	0.87349583	1	nonDEG	-0.6375729	0.07846065

nonDEG	0.2881597	0.82349835	1	nonDEG	-0.991166	0.52965648
nonDEG	-0.1282189	0.76921801	1	nonDEG	0.06297267	0.87279855
nonDEG	0.10420259	0.8050147	1	nonDEG	0.09891935	0.7833027
nonDEG	-0.4786247	0.61468926	1	nonDEG	0.45554831	0.65156875
nonDEG	-0.1215099	0.7804596	1	nonDEG	0.12561438	0.73849676
nonDEG	0.03749786	1	1	nonDEG	-1.4943458	0.24766088
nonDEG	0.98193487	0.28364043	1	nonDEG	-0.9341943	0.3448901
nonDEG	0.4029147	0.58837265	1	nonDEG	0.13700193	0.85562681
nonDEG	0.03148482	0.9416407	1	nonDEG	-0.3085669	0.39335734
nonDEG	0.25465094	0.57492702	1	nonDEG	-0.404352	0.31221748
nonDEG	1.91584656	0.47283224	1	nonDEG	0.25405182	0.95136675
nonDEG	-0.0499971	0.90580347	1	nonDEG	0.22719554	0.5262607
nonDEG	0.11039635	0.92163001	1	nonDEG	-0.0965841	0.92428298
nonDEG	-0.379713	0.3675502	1	nonDEG	-0.2669279	0.49231064
nonDEG	0.03552081	0.93365144	1	nonDEG	-0.6395144	0.11174627
nonDEG	-0.7321823	0.34600673	1	nonDEG	0.75644293	0.27716448
nonDEG	-0.0365811	0.93078708	1	nonDEG	0.24890517	0.4951683
nonDEG	-0.3166564	0.48757474	1	nonDEG	-0.160414	0.70990489
nonDEG	4.70280101	0.22899326	1	nonDEG	2.26465329	0.22578003
nonDEG	-0.0753199	0.86167404	1	nonDEG	-0.034078	0.92549008
nonDEG	0.09038818	0.83099004	1	nonDEG	-0.0397254	0.91243047
nonDEG	0.58575838	0.22134868	1	nonDEG	-0.0764458	0.83643191
nonDEG	0.31051427	0.46732775	1	nonDEG	-0.2724091	0.47244321
nonDEG	-0.2723562	0.68133438	1	nonDEG	0.06007249	0.92767235
nonDEG	-1.9598775	0.36241245	1	nonDEG	2.26384492	0.41912967
nonDEG	-0.8721579	0.47232095	1	nonDEG	0.55486981	0.6728624
nonDEG	-0.019696	0.96604191	1	nonDEG	-0.073745	0.84985791
nonDEG	-1.364397	0.20257357	1	nonDEG	0.9287175	0.41111697
nonDEG	0.00497258	0.99114465	1	nonDEG	-0.0790773	0.83008894
nonDEG	-0.1518192	0.83127698	1	nonDEG	-1.3653698	0.07628045
nonDEG	-0.1240574	0.76983586	1	nonDEG	-0.1441247	0.70299213
nonDEG	-1.9994906	0.03569921	1	nonDEG	3.93143066	0.00242683
nonDEG	-0.2148181	0.69652644	1	nonDEG	-0.2919133	0.56843752
nonDEG	-0.0826452	0.84513016	1	nonDEG	-0.0677251	0.85154134
nonDEG	0.18772877	0.65917566	1	nonDEG	-0.3519146	0.33875131
nonDEG	-0.7734107	0.22365825	1	nonDEG	0.34884952	0.58931118
nonDEG	0.2084123	0.6336221	1	nonDEG	-0.1483346	0.69873985
nonDEG	-0.024883	0.95356876	1	nonDEG	-0.3530414	0.3294854
nonDEG	-0.0951442	0.83528132	1	nonDEG	-0.2991719	0.46552332
nonDEG	-0.2556253	0.55439293	1	nonDEG	-0.0196461	0.95975129
nonDEG	-0.4524987	0.32328155	1	nonDEG	-0.0275666	0.95335494
nonDEG	0.29939068	0.48375832	1	nonDEG	-0.2461885	0.4959667
nonDEG	0.10729644	0.80290778	1	nonDEG	-0.0872666	0.80913871

nonDEG	0.03639208	0.93334329	1	nonDEG	-0.0492954	0.89129081
nonDEG	0.0341511	0.93598348	1	nonDEG	0.00118367	0.99791067
nonDEG	-0.2923511	0.49616203	1	nonDEG	-0.1555631	0.6967425
nonDEG	0.22332825	0.62710677	1	nonDEG	0.21501237	0.58929339
nonDEG	0.00277228	0.99825008	1	nonDEG	0.33124234	0.43541342
nonDEG	-0.7209496	0.33065586	1	nonDEG	0.00328261	1
nonDEG	0.11250932	0.84467463	1	nonDEG	0.17719594	0.73833363
nonDEG	0.23041271	0.58771366	1	nonDEG	-0.2542588	0.48562655
nonDEG	-0.134997	0.7940603	1	nonDEG	0.47731129	0.30212199
nonDEG	-0.1115651	0.92478804	1	nonDEG	2.30095446	0.04372632
nonDEG	-0.3128575	0.47522901	1	nonDEG	0.38041318	0.29439403
nonDEG	-0.2251801	0.7434836	1	nonDEG	-0.0715044	0.9304169
nonDEG	1.62685577	0.18652481	1	nonDEG	-1.4326155	0.38982894
nonDEG	-1.3168004	0.17834006	1	nonDEG	0.03021074	1
nonDEG	0.0034276	0.99584487	1	nonDEG	-0.1773674	0.66490181
nonDEG	0.71318722	0.09476429	1	nonDEG	-0.4383503	0.23205738
nonDEG	0.36646446	0.46423725	1	nonDEG	-0.2946696	0.53353183
nonDEG	0.2070105	0.62794485	1	nonDEG	-0.1968658	0.59549206
nonDEG	-0.1842424	0.6725108	1	nonDEG	-0.3405758	0.3685727
nonDEG	0.50908384	0.2609831	1	nonDEG	-0.0940351	0.81405077
nonDEG	-0.0686031	0.88750579	1	nonDEG	-0.4014705	0.33401158
nonDEG	-0.3935969	0.42414898	1	nonDEG	-0.2136148	0.60245093
nonDEG	-0.1709113	0.69408341	1	nonDEG	0.06980332	0.85767562
nonDEG	0.11449866	0.87823377	1	nonDEG	0.04938419	0.95518631
nonDEG	1.31542826	0.11264191	1	nonDEG	-0.2855642	0.73401381
nonDEG	0.43491245	0.33352009	1	nonDEG	-0.4183547	0.28119897
nonDEG	-0.6621217	0.14285155	1	nonDEG	0.20240871	0.57836579
nonDEG	0.31162559	0.68616266	1	nonDEG	0.3142998	0.66955154
nonDEG	0.11580563	0.80109032	1	nonDEG	-0.0004588	1
nonDEG	-0.2366477	0.60216194	1	nonDEG	0.63086567	0.11244026
nonDEG	-0.6670593	0.32498318	1	nonDEG	-0.3818822	0.60772109
nonDEG	-0.352058	0.43046413	1	nonDEG	0.01419351	0.97068924
nonDEG	0.51014496	0.26474437	1	nonDEG	-0.4593388	0.24978077
nonDEG	-0.0314857	0.94670022	1	nonDEG	-0.3394676	0.429499
nonDEG	1.14046281	0.07943553	1	nonDEG	-0.5977559	0.18333788
nonDEG	0.39278992	0.40301414	1	nonDEG	0.31762724	0.43911979
nonDEG	-0.0981951	0.81822255	1	nonDEG	-0.2420973	0.50688844
nonDEG	-0.1569843	0.71850097	1	nonDEG	-0.4622569	0.20538718
nonDEG	-0.0028494	0.99508759	1	nonDEG	0.19955567	0.58082114
nonDEG	-0.766233	0.22368977	1	nonDEG	1.14147788	0.09998009
nonDEG	-0.1482874	0.73541099	1	nonDEG	-0.352976	0.36433083
nonDEG	0.7105045	0.12365654	1	nonDEG	0.26555295	0.49637291
nonDEG	0.43406194	0.44599876	1	nonDEG	-0.2652392	0.58226246

nonDEG	-0.0061032	0.99095576	1	nonDEG	-0.4683929	0.27725381
nonDEG	-0.2384336	0.65764244	1	nonDEG	0.37898706	0.43682821
nonDEG	0.93635154	0.68517428	1	nonDEG	0.05953993	1
nonDEG	-0.2442046	0.58038545	1	nonDEG	-0.0161563	0.96861316
nonDEG	1.39247547	0.73838749	1	nonDEG	5.09862802	0.00020544
nonDEG	0.11029686	0.79552923	1	nonDEG	-0.054073	0.88141794
nonDEG	0.133131	0.92181726	1	nonDEG	-0.7727561	0.4898666
nonDEG	-0.9977071	0.56177045	1	nonDEG	1.11888428	0.64294344
nonDEG	-0.5323649	0.3609254	1	nonDEG	-0.2231284	0.67047843
nonDEG	-0.860982	0.44339427	1	nonDEG	1.44211066	0.19535591
nonDEG	0.85575935	0.80584739	1	nonDEG	-2.0211086	0.53016024
nonDEG	-0.8909624	0.28835706	1	nonDEG	0.51947922	0.62846888
nonDEG	0.22193003	0.64377015	1	nonDEG	-0.0627106	0.8852425
nonDEG	0.54473764	0.21685605	1	nonDEG	-0.2838068	0.45407015
nonDEG	-0.5435811	0.56081285	1	nonDEG	1.00859939	0.28738343
nonDEG	1.93520321	0.37827413	1	nonDEG	-1.3227727	0.64971493
nonDEG	0.12012143	0.79114821	1	nonDEG	0.43470445	0.26320166
nonDEG	0.12433519	0.77499126	1	nonDEG	-0.5437969	0.15380793
nonDEG	1.93216972	0.03777953	1	nonDEG	-1.5365561	0.11688662
nonDEG	0.08505922	0.89925072	1	nonDEG	0.2767503	0.65904235
nonDEG	-0.0065034	0.99287946	1	nonDEG	-0.2983653	0.62650596
nonDEG	0.22242643	0.61311632	1	nonDEG	-0.8847788	0.02611669
nonDEG	0.13096454	0.75675826	1	nonDEG	-0.0417877	0.90958622
nonDEG	0.10241331	0.83371164	1	nonDEG	-0.0680114	0.86521379
nonDEG	0.07739146	0.87095111	1	nonDEG	-0.6025296	0.14436583
nonDEG	-6.0260309	0.05934832	1	nonDEG	6.06547191	0.10258364
nonDEG	0.2300488	0.60446794	1	nonDEG	-0.2159295	0.58584232
nonDEG	-0.4315909	0.39026625	1	nonDEG	0.85549526	0.05892589
nonDEG	-0.1274009	0.76837964	1	nonDEG	0.03849783	0.92008513
nonDEG	0.58665705	0.28085041	1	nonDEG	-0.1819049	0.70962706
nonDEG	0.15134687	0.72984329	1	nonDEG	0.12552871	0.74852543
nonDEG	-0.1117094	0.79530609	1	nonDEG	0.03132234	0.93194456
nonDEG	0.93914305	0.36517453	1	nonDEG	0.47678329	0.65162981
nonDEG	-0.2031414	0.62945224	1	nonDEG	-0.1556879	0.67175048
nonDEG	4.18075503	0.36137967	1	nonDEG	-4.3849095	0.36721722
nonDEG	0.28386461	0.51619836	1	nonDEG	-0.0493929	0.90113089
nonDEG	-0.0817139	0.85560937	1	nonDEG	-0.0763031	0.84668582
nonDEG	0.19378799	0.65248905	1	nonDEG	-0.1450268	0.69694804
nonDEG	0.00250108	1	1	nonDEG	0.05289875	0.92967227
nonDEG	0.15123077	0.80378538	1	nonDEG	-0.6020807	0.31229819
nonDEG	0.08337867	0.866472	1	nonDEG	-0.5117038	0.22985983
nonDEG	0.06087595	0.89311303	1	nonDEG	0.12102801	0.76144016
nonDEG	-0.2551683	0.56280334	1	nonDEG	0.07115876	0.86254601

nonDEG	0.19266176	0.66145553	1	nonDEG	-0.2806347	0.47592843
nonDEG	-1.062973	0.48624304	1	nonDEG	0.36421393	0.84568344
nonDEG	2.23464547	1	1	nonDEG	-2.384686	1
nonDEG	0.27362599	0.55050762	1	nonDEG	-0.4517461	0.30355251
nonDEG	-2.0786655	0.13227043	1	nonDEG	0.22504147	0.9496423
nonDEG	0.26856869	0.62410751	1	nonDEG	-0.3721882	0.45783963
nonDEG	0.25066918	0.56211934	1	nonDEG	-0.0352455	0.92274871
nonDEG	-0.158595	0.7529091	1	nonDEG	0.15678011	0.73071023
nonDEG	-0.1676407	0.70645313	1	nonDEG	-0.1775279	0.68186337
nonDEG	0.29742341	0.5653768	1	nonDEG	0.01446552	0.97877655
nonDEG	-0.1290871	0.78459544	1	nonDEG	-0.3887694	0.39599848
nonDEG	0.28637913	0.51443371	1	nonDEG	0.11551489	0.76115243
nonDEG	-0.1328375	0.77323449	1	nonDEG	-0.1518245	0.68090939
nonDEG	3.25006562	0.03076266	1	nonDEG	-4.0763895	0.01601821
nonDEG	0	1	1	nonDEG	-5.13E-15	1
nonDEG	0.08236598	0.89811285	1	nonDEG	0.16467784	0.76959359
nonDEG	0.79708949	0.38696199	1	nonDEG	0.42325469	0.64536246
nonDEG	0.2339918	0.63940653	1	nonDEG	-0.0877169	0.83060257
nonDEG	0.07302038	0.88323548	1	nonDEG	0.24151103	0.57549444
nonDEG	-0.6260776	0.24419455	1	nonDEG	0.23861303	0.65404685
nonDEG	-0.1993567	0.77982278	1	nonDEG	-0.2771405	0.69449468
nonDEG	-0.4756639	0.52827506	1	nonDEG	1.07690561	0.15015542
nonDEG	0.08283996	0.84821748	1	nonDEG	-0.4838564	0.18356017
nonDEG	-0.6846223	0.15606203	1	nonDEG	0.39762378	0.33209741
nonDEG	0.00385291	0.99328193	1	nonDEG	-0.4060558	0.28514922
nonDEG	-0.0077287	0.98615523	1	nonDEG	-0.019736	0.95872681
nonDEG	-0.0866552	0.84561012	1	nonDEG	-0.0291294	0.93620185
nonDEG	0.08369389	0.87970348	1	nonDEG	-0.889871	0.10423132
nonDEG	-0.0386586	0.9382065	1	nonDEG	-0.5714056	0.14986541
nonDEG	-0.0213569	0.96043952	1	nonDEG	0.29691996	0.4344721
nonDEG	-0.3952588	0.36910821	1	nonDEG	0.26450479	0.48169197
nonDEG	0.11020946	0.88430957	1	nonDEG	-0.1350098	0.8595992
nonDEG	0.53103368	0.35328734	1	nonDEG	-1.0341478	0.07858325
nonDEG	0.38115874	0.51440558	1	nonDEG	-0.8080608	0.16507768
nonDEG	-0.3684529	0.43435443	1	nonDEG	-0.3022257	0.48013856
nonDEG	1.01448936	0.1047981	1	nonDEG	-0.7970913	0.18972878
nonDEG	0.38365322	0.42422723	1	nonDEG	0.35205992	0.33173666
nonDEG	0.90304733	0.12138589	1	nonDEG	-0.5090204	0.3662818
nonDEG	-0.4239454	0.32571092	1	nonDEG	-0.1372535	0.73183341
nonDEG	0	1	1	nonDEG	-5.13E-15	1
nonDEG	0.47277189	0.32348611	1	nonDEG	-0.447293	0.2916704
nonDEG	-0.3414419	0.50804372	1	nonDEG	0.47367066	0.29479515
nonDEG	-0.1089708	0.79701686	1	nonDEG	-0.0978664	0.7883116

nonDEG	0.09321376	0.83131626	1	nonDEG	-0.0460912	0.90206983
nonDEG	-0.3552854	0.65303714	1	nonDEG	1.06268649	0.05680562
nonDEG	0.54289298	0.35211117	1	nonDEG	-0.1758556	0.75272889
nonDEG	-0.1989878	0.64898344	1	nonDEG	0.00402706	0.9930569
nonDEG	-5.3828095	0.04442388	1	nonDEG	4.92119342	0.2621438
nonDEG	-0.3386359	0.42158273	1	nonDEG	-0.221719	0.56125823
nonDEG	-0.3441334	0.53702002	1	nonDEG	-0.3595492	0.52161736
nonDEG	-0.1200684	0.81608828	1	nonDEG	-0.18934	0.68986144
nonDEG	-0.1066764	0.80027102	1	nonDEG	0.12367797	0.73265443
nonDEG	-0.0845117	0.8401642	1	nonDEG	-0.0807323	0.8237517
nonDEG	-0.2658899	0.54895958	1	nonDEG	-0.3358506	0.42092831
nonDEG	-0.3851369	0.4345354	1	nonDEG	-0.0468487	0.92413956
nonDEG	0.02991839	0.95416783	1	nonDEG	-0.1729294	0.69480235
nonDEG	-0.178814	0.72252932	1	nonDEG	-0.1447865	0.76593068
nonDEG	-0.2334004	0.67402229	1	nonDEG	-0.2793305	0.59455978
nonDEG	0.01908683	0.97163531	1	nonDEG	-0.2405662	0.59940106
nonDEG	-0.459976	0.3273014	1	nonDEG	0.14170846	0.73377502
nonDEG	0.91002972	0.03834133	1	nonDEG	-0.5091354	0.17848424
nonDEG	0.57439131	0.3243968	1	nonDEG	-0.1947729	0.73255179
nonDEG	0.18361738	0.93307097	1	nonDEG	-5.3601288	0.19519413
nonDEG	-5.6686583	0.01336422	0.86906821	nonDEG	5.14213572	0.00235973
nonDEG	0.13346142	0.7761521	1	nonDEG	-0.4081359	0.31448259
nonDEG	0.22919356	0.8667607	1	nonDEG	-0.8175292	0.51404939
nonDEG	-0.8311453	0.37837233	1	nonDEG	0.21307749	0.86149742
nonDEG	0.31688732	0.56078134	1	nonDEG	-0.3738802	0.50640959
nonDEG	0.0975236	0.9172245	1	nonDEG	-0.0290756	0.98658804
nonDEG	0.50680768	0.38168841	1	nonDEG	-0.3919854	0.50655994
nonDEG	0.67070367	0.36135271	1	nonDEG	-0.5628693	0.47849126
nonDEG	-0.514384	0.31609677	1	nonDEG	0.64997764	0.16813746
nonDEG	0.11963871	0.81096155	1	nonDEG	-0.2061987	0.61628952
nonDEG	-0.0699716	0.87327901	1	nonDEG	0.09599814	0.79525252
nonDEG	0.9022288	0.21150785	1	nonDEG	-0.653454	0.26886982
nonDEG	-0.0827858	0.85346516	1	nonDEG	-0.1890335	0.63080158
nonDEG	1.09870311	0.47324706	1	nonDEG	-0.3873509	0.79327369
nonDEG	0.02880791	0.95072832	1	nonDEG	-0.8578284	0.02031374
nonDEG	0.30862499	0.48475985	1	nonDEG	-0.1975887	0.59903929
nonDEG	-0.2336458	0.59946186	1	nonDEG	-0.0454377	0.90741534
nonDEG	0.00611737	0.98884397	1	nonDEG	-0.0852156	0.81649956
nonDEG	-0.7813445	0.36079716	1	nonDEG	0.96209902	0.25449933
nonDEG	-0.0982637	0.82372935	1	nonDEG	-0.5710809	0.14067666
nonDEG	0.16548435	0.69389678	1	nonDEG	-0.292379	0.42026596
nonDEG	0.24422172	0.63149143	1	nonDEG	-0.0066444	0.99162863
nonDEG	0.4205181	0.31415718	1	nonDEG	-0.2454912	0.49715252

nonDEG	0.33392776	0.54027673	1	nonDEG	-0.8919099	0.11648702
nonDEG	-0.2454988	0.56963317	1	nonDEG	0.09144854	0.80388644
nonDEG	-0.6898574	0.2643299	1	nonDEG	0.39554762	0.52536468
nonDEG	-0.1917945	0.67393745	1	nonDEG	-0.0709216	0.86205076
nonDEG	-0.1355282	0.74992686	1	nonDEG	-0.0639711	0.85912993
nonDEG	-0.4109541	0.33388404	1	nonDEG	-0.2056185	0.58117958
nonDEG	-0.1667787	0.72326028	1	nonDEG	0.50297938	0.23505172
nonDEG	-0.3147743	0.47245991	1	nonDEG	-0.0835112	0.81829793
nonDEG	0.24070056	0.60262867	1	nonDEG	0.03420847	0.93288529
nonDEG	0.19383065	0.69057399	1	nonDEG	-0.2281416	0.5863745
nonDEG	0.08076441	1	1	nonDEG	2.15268996	0.28332605
nonDEG	0.28236492	0.55771498	1	nonDEG	-0.5219179	0.20874595
nonDEG	-0.4928461	0.28230925	1	nonDEG	0.5298749	0.16411647
nonDEG	-0.2610542	0.54714688	1	nonDEG	-0.2568944	0.49086692
nonDEG	-1.0291859	0.46385804	1	nonDEG	1.88601977	0.18644508
nonDEG	-0.0393384	0.93058446	1	nonDEG	-0.4180904	0.27311618
nonDEG	0.26437874	0.55650339	1	nonDEG	-0.6094553	0.13291966
nonDEG	-0.1984106	0.65705199	1	nonDEG	0.01579855	0.96920067
nonDEG	-0.5490025	0.24865849	1	nonDEG	0.15195756	0.74479565
nonDEG	-0.9471614	0.23691206	1	nonDEG	0.71836494	0.49030799
nonDEG	-0.0875911	0.83689275	1	nonDEG	0.08572037	0.81372773
nonDEG	-0.0960044	0.82466952	1	nonDEG	-0.572797	0.14554371
nonDEG	-0.2938845	0.51864842	1	nonDEG	0.2483755	0.54842221
nonDEG	0.8146749	0.43875506	1	nonDEG	-0.0876336	0.9314516
nonDEG	0.10440744	0.81155033	1	nonDEG	0.17864275	0.62935865
nonDEG	-0.0234035	0.95737317	1	nonDEG	-0.1301479	0.72272125
nonDEG	0.46613881	0.44962803	1	nonDEG	-0.2860598	0.63897298
nonDEG	-0.2502043	0.55708552	1	nonDEG	0.33712286	0.35786489
nonDEG	-0.5067967	0.25358917	1	nonDEG	0.75139442	0.04837243
nonDEG	3.67174992	5.71E-05	0.14400617	nonDEG	-1.2057505	0.09831589
nonDEG	0.12814352	0.77725134	1	nonDEG	-0.3314696	0.40068129
nonDEG	0.25938917	0.5762199	1	nonDEG	-0.5245556	0.18345991
nonDEG	-0.0273393	0.95020062	1	nonDEG	0.10924087	0.76582633
nonDEG	-0.1506638	1	1	nonDEG	0.05796836	1
nonDEG	-4.1756057	0.28471695	1	nonDEG	5.34183656	0.07967179
nonDEG	0.4349064	0.40687272	1	nonDEG	-0.0120752	0.97954534
nonDEG	0.66813471	0.11822967	1	nonDEG	-0.3040263	0.40977142
nonDEG	0.05178047	0.90392278	1	nonDEG	-0.647995	0.10155261
nonDEG	0.70811651	0.33560507	1	nonDEG	-0.0955828	0.90487758
nonDEG	-0.8938069	0.36700929	1	nonDEG	1.07288297	0.25640347
nonDEG	-1.4558245	0.14132334	1	nonDEG	0.99627608	0.2848948
nonDEG	-0.4126002	0.35505571	1	nonDEG	-0.0765047	0.84416656
nonDEG	-0.1728986	0.69583138	1	nonDEG	0.22696594	0.55013424

nonDEG	0.22317713	0.62771831	1	nonDEG	-0.1166532	0.78053141
nonDEG	0.14600922	0.77052644	1	nonDEG	0.29896716	0.52685004
nonDEG	-0.1066309	0.8046431	1	nonDEG	0.12116877	0.74093565
nonDEG	0.74213913	0.21583535	1	nonDEG	-0.6436475	0.16605822
nonDEG	1.53400476	0.06671797	1	nonDEG	-0.8323669	0.28122002
nonDEG	1.46267301	0.40440243	1	nonDEG	-1.4671973	0.57759138
nonDEG	-0.1421466	0.74520277	1	nonDEG	-0.0287727	0.94115615
nonDEG	0.12250684	0.78293262	1	nonDEG	-0.6724597	0.08080265
nonDEG	0.84967835	0.42700054	1	nonDEG	-1.4014434	0.27353552
nonDEG	2.22186054	0.00435848	0.68407511	nonDEG	-0.3959169	0.5677538
nonDEG	0.36590769	0.3991279	1	nonDEG	-0.352485	0.34123527
nonDEG	0.23703254	0.5945954	1	nonDEG	-0.1015508	0.7809685
nonDEG	0.31680971	0.51053336	1	nonDEG	-0.1155261	0.79105715
nonDEG	0.43525273	0.44169816	1	nonDEG	-0.5204034	0.27695875
nonDEG	0.20472405	0.74991341	1	nonDEG	-0.7982461	0.23985425
nonDEG	-0.0875411	0.84746916	1	nonDEG	-0.2091174	0.61561788
nonDEG	-0.2089729	0.63452736	1	nonDEG	-0.0406812	0.9182285
nonDEG	-0.041476	0.92227874	1	nonDEG	-0.0817962	0.8235331
nonDEG	-0.826858	0.45789949	1	nonDEG	0.49988019	0.59077247
nonDEG	0.05501009	0.89676383	1	nonDEG	-0.1294523	0.72472124
nonDEG	2.20754717	0.24549184	1	nonDEG	-2.5340853	0.27963731
nonDEG	0.13346974	0.75485593	1	nonDEG	-0.0895489	0.80467913
nonDEG	-0.1063147	0.80349376	1	nonDEG	0.05974835	0.86937562
nonDEG	0.3620101	0.51149357	1	nonDEG	-0.280781	0.59066758
nonDEG	1.57008424	0.03213192	1	nonDEG	-1.028179	0.14263045
nonDEG	-1.7474377	0.22335338	1	nonDEG	0.3511643	1
nonDEG	-0.3329785	0.69508408	1	nonDEG	0.20235973	0.79632755
nonDEG	-0.0884261	0.85301994	1	nonDEG	-0.2386629	0.59354297
nonDEG	-0.9862489	0.44382486	1	nonDEG	0.73444024	0.61123317
nonDEG	0.06010418	0.9431503	1	nonDEG	-0.73601	0.38806593
nonDEG	0.70054746	0.33852698	1	nonDEG	-0.6175139	0.41255829
nonDEG	-0.168065	0.69879953	1	nonDEG	-0.2484392	0.5258464
nonDEG	0.42123581	0.48895417	1	nonDEG	-0.1255585	0.82684749
nonDEG	0.03523435	0.93537818	1	nonDEG	0.03214221	0.92947611
nonDEG	0.31192919	0.57422335	1	nonDEG	-0.6133752	0.17522439
nonDEG	0.13021013	0.76729143	1	nonDEG	-0.1653351	0.66850492
nonDEG	-0.0419945	0.92313901	1	nonDEG	-0.3807694	0.29917274
nonDEG	-0.1003483	0.83158908	1	nonDEG	-0.3583841	0.38497788
nonDEG	0.70060816	0.37182645	1	nonDEG	-0.4230259	0.53147236
nonDEG	1.29301102	0.66781877	1	nonDEG	0.44562764	0.92372137
nonDEG	-3.2903053	0.64300646	1	nonDEG	4.9212213	0.26509893
nonDEG	-0.22814	0.58928111	1	nonDEG	0.45601473	0.23056303
nonDEG	-0.3082363	0.60742718	1	nonDEG	0.52601011	0.35629724

nonDEG	-0.5854292	0.27059565	1	nonDEG	0.15287714	0.77682769
nonDEG	0.17879127	0.71152174	1	nonDEG	-0.265935	0.52894062
nonDEG	0.88316719	0.31607151	1	nonDEG	-0.6448979	0.3249038
nonDEG	-0.1468611	0.73732167	1	nonDEG	0.15761091	0.66764297
nonDEG	0.05953981	0.88792779	1	nonDEG	-0.0722612	0.84325434
nonDEG	0.66215645	0.26323467	1	nonDEG	-0.4023422	0.47544559
nonDEG	0.11751014	0.82058722	1	nonDEG	-0.1440294	0.76069009
nonDEG	0.1913937	0.71005446	1	nonDEG	-0.6530923	0.13539797
nonDEG	0.41870211	0.35583852	1	nonDEG	-0.0499359	0.89739603
nonDEG	-0.0512457	0.91012507	1	nonDEG	-0.2859481	0.49015682
nonDEG	-0.0212198	0.962218	1	nonDEG	-0.01531	0.96759419
nonDEG	0.71487952	0.13621604	1	nonDEG	-0.8211446	0.06409369
nonDEG	0.01167615	0.97957464	1	nonDEG	0.96861552	0.02030304
nonDEG	-0.1322205	0.76453701	1	nonDEG	-0.0599855	0.87497899
nonDEG	0.55947952	0.40439512	1	nonDEG	-0.1257418	0.84569443
nonDEG	0.03258623	0.93908079	1	nonDEG	0.01407092	0.97020056
nonDEG	0.52762534	0.22859146	1	nonDEG	-0.1765523	0.64243375
nonDEG	-0.2198667	0.64543936	1	nonDEG	-0.451494	0.35192136
nonDEG	0.7025553	0.18877307	1	nonDEG	-0.1567385	0.74695078
nonDEG	-0.1738039	0.68433025	1	nonDEG	-0.0764275	0.84257167
nonDEG	0.035876	0.93562582	1	nonDEG	0.1098631	0.77030674
nonDEG	0.14276791	0.80452095	1	nonDEG	-0.3104295	0.57054321
nonDEG	-0.0326951	0.95787822	1	nonDEG	-0.3691574	0.49046672
nonDEG	-0.03496	0.93716587	1	nonDEG	-0.2665271	0.49292773
nonDEG	0.42527233	0.85270334	1	nonDEG	-0.6942008	0.7439924
nonDEG	-0.0307359	0.9458968	1	nonDEG	-0.0710425	0.86026591
nonDEG	-0.4516251	0.28863663	1	nonDEG	-0.0893735	0.83175366
nonDEG	-0.6277841	0.25667723	1	nonDEG	0.17869681	0.75780522
nonDEG	-0.9619811	0.24780305	1	nonDEG	1.57242558	0.04531179
nonDEG	0.46145543	0.333799	1	nonDEG	-0.0786546	0.85421652
nonDEG	0.10480767	0.80835063	1	nonDEG	-0.0820299	0.82861483
nonDEG	0.15759705	0.71398218	1	nonDEG	0.09945881	0.78421229
nonDEG	-0.3321737	0.45912818	1	nonDEG	0.08207288	0.84497028
nonDEG	-0.0883814	0.84773852	1	nonDEG	-0.1327319	0.72275285
nonDEG	0.17560428	0.72902198	1	nonDEG	-0.4667174	0.24462014
nonDEG	0.62314457	0.19351211	1	nonDEG	-0.8335786	0.07188722
nonDEG	-0.2803775	0.59680324	1	nonDEG	0.76452165	0.12250288
nonDEG	-0.1205281	0.78043742	1	nonDEG	-0.0747202	0.84281379
nonDEG	-0.5590637	0.18044319	1	nonDEG	0.32081459	0.37104099
nonDEG	-0.6718852	0.50606655	1	nonDEG	0.3081314	0.78958224
nonDEG	0.31115138	0.49574338	1	nonDEG	-0.1053143	0.80209561
nonDEG	0.02410935	0.95554787	1	nonDEG	-0.0847271	0.81537644
nonDEG	0.20333108	0.67099486	1	nonDEG	-0.6392283	0.11236635

nonDEG	-0.2726866	0.54160798	1	nonDEG	0.00538269	0.99169769
nonDEG	0.29424135	0.5559931	1	nonDEG	-0.5129746	0.25249166
nonDEG	0.21290859	0.63583223	1	nonDEG	0.07474078	0.85292545
nonDEG	-0.1213859	1	1	nonDEG	1.06133973	0.70090976
nonDEG	0.06918832	0.86915551	1	nonDEG	0.13647864	0.70308426
nonDEG	-0.2339243	0.58226768	1	nonDEG	-0.5129292	0.15854961
nonDEG	-0.1835884	0.68067702	1	nonDEG	0.07687681	0.83972609
nonDEG	-0.2535599	0.54781554	1	nonDEG	0.01835653	0.96154708
nonDEG	-0.0637426	0.89078214	1	nonDEG	-0.6972594	0.0766526
nonDEG	-0.4540207	0.47864529	1	nonDEG	0.57835945	0.35589435
nonDEG	0.15167457	0.73123498	1	nonDEG	0.11616955	0.76244655
nonDEG	-1.1966657	0.22576866	1	nonDEG	-0.6226815	0.53506438
nonDEG	0.17835801	0.69090925	1	nonDEG	-0.1962943	0.6142959
nonDEG	-0.0868741	0.8391183	1	nonDEG	0.01617836	0.96460033
nonDEG	0.13880934	0.74584222	1	nonDEG	0.02397377	0.94765303
nonDEG	0.87307224	0.07578699	1	nonDEG	-0.3490081	0.42143207
nonDEG	-3.0684862	0.02442699	1	nonDEG	3.93266896	0.0178813
nonDEG	-0.4246749	0.47410641	1	nonDEG	0.54684552	0.34210171
nonDEG	0.92250935	0.4923426	1	nonDEG	-0.5704932	0.65973267
nonDEG	-0.2858088	0.63274432	1	nonDEG	0.4371367	0.43453549
nonDEG	5.79316643	0.01755615	0.93380915	nonDEG	-1.0719268	0.59662409
nonDEG	0.33084927	0.47092901	1	nonDEG	-0.3296425	0.40830015
nonDEG	0.34559027	0.4896012	1	nonDEG	-0.3038251	0.4630334
nonDEG	-0.3188491	0.48106876	1	nonDEG	-0.3354303	0.41373892
nonDEG	-0.0287952	0.94645296	1	nonDEG	0.10856167	0.76666507
nonDEG	-2.3014949	1	1	nonDEG	-5.13E-15	1
nonDEG	0.24235955	0.66631915	1	nonDEG	-0.6670844	0.1617588
nonDEG	-0.3445321	0.42128125	1	nonDEG	0.36103272	0.3252804
nonDEG	-0.1844679	0.67669389	1	nonDEG	0.04801813	0.89841728
nonDEG	0.87641819	0.69599635	1	nonDEG	0.45047264	0.82735766
nonDEG	-0.4920263	0.55341059	1	nonDEG	0.18139923	0.84214159
nonDEG	0.26061608	0.5713904	1	nonDEG	0.00186517	0.99743295
nonDEG	-0.0248548	0.95461969	1	nonDEG	-0.4608016	0.2125444
nonDEG	0.16130702	0.70184255	1	nonDEG	-0.0677953	0.8508873
nonDEG	1.00701248	0.0285908	1	nonDEG	-0.4182718	0.27136441
nonDEG	0.54619658	0.26820868	1	nonDEG	1.24037435	0.05239115
nonDEG	0.00842755	0.99151435	1	nonDEG	-0.1193064	0.82767069
nonDEG	-0.1599556	0.72141196	1	nonDEG	-0.0085026	0.98265427
nonDEG	0.3545661	0.40186179	1	nonDEG	0.04166035	0.90992182
nonDEG	-0.192587	0.64970868	1	nonDEG	0.19491345	0.58959547
nonDEG	-0.33496	0.44767904	1	nonDEG	0.00718648	0.98513231
nonDEG	0.06635967	0.88809596	1	nonDEG	0.0710919	0.85486092
nonDEG	-0.1311726	0.82211646	1	nonDEG	-0.168144	0.66822865

nonDEG	-0.3005081	0.4767366	1	nonDEG	0.04834113	0.89306685
nonDEG	0.34000791	0.4991823	1	nonDEG	-0.1853403	0.65314974
nonDEG	0.76766021	0.62611203	1	nonDEG	-0.6825006	0.76456262
nonDEG	0.56958739	0.30889267	1	nonDEG	-0.5585022	0.29223401
nonDEG	0.52778166	0.67088159	1	nonDEG	0.69885664	0.53983161
nonDEG	-0.5356588	0.35141052	1	nonDEG	0.51132215	0.34756003
nonDEG	1.87255975	0.07420335	1	nonDEG	-0.23859	0.82135152
nonDEG	-0.0430073	0.9198726	1	nonDEG	0.10008803	0.78736078
nonDEG	-0.0003269	1	1	nonDEG	0.01003151	0.98366048
nonDEG	0.6359125	0.60013423	1	nonDEG	-1.1585273	0.39852153
nonDEG	0.5705842	0.25318535	1	nonDEG	-0.1372944	0.74669532
nonDEG	-0.3015227	0.78316166	1	nonDEG	0.0563425	1
nonDEG	-0.1985206	0.64184978	1	nonDEG	0.14087391	0.70101428
nonDEG	-1.5264226	0.14347885	1	nonDEG	2.80066914	0.00516042
nonDEG	-0.1574214	0.71149966	1	nonDEG	-0.0459488	0.90094763
nonDEG	0.12687158	0.81855779	1	nonDEG	-0.5661258	0.29513041
nonDEG	-0.2130235	0.61472241	1	nonDEG	0.08131796	0.82345109
nonDEG	-0.0858203	0.88280599	1	nonDEG	-0.1216703	0.82652083
nonDEG	0.59612209	0.2851218	1	nonDEG	0.7161997	0.08230422
nonDEG	-0.2747051	0.53423015	1	nonDEG	-0.0274975	0.94196764
nonDEG	0.22131607	0.60482533	1	nonDEG	-0.2170736	0.55046861
nonDEG	-0.0062644	0.98903402	1	nonDEG	0.31570557	0.39362708
nonDEG	-0.07731	0.8670405	1	nonDEG	0.33548427	0.40107359
nonDEG	0.31467721	0.58235762	1	nonDEG	0.1731106	0.73782308
nonDEG	-0.0620616	0.8872081	1	nonDEG	0.25031484	0.51148269
nonDEG	-0.0650146	0.88172595	1	nonDEG	-0.4935624	0.22245547
nonDEG	-0.6704516	0.18686458	1	nonDEG	0.42733141	0.31814818
nonDEG	-0.2923225	0.54338494	1	nonDEG	0.03273211	0.94787899
nonDEG	-0.3918577	0.34581348	1	nonDEG	-0.6256461	0.09275485
nonDEG	0.41044397	0.69850583	1	nonDEG	0.2171705	0.84704945
nonDEG	0.17696283	0.69106286	1	nonDEG	-0.4469087	0.23477742
nonDEG	-0.0599791	0.89319718	1	nonDEG	0.00601644	0.98699235
nonDEG	0.3672574	0.43918117	1	nonDEG	-1.6426938	0.00016329
nonDEG	-0.2835691	0.71195306	1	nonDEG	0.48884731	0.4900203
nonDEG	-0.0918269	0.83722668	1	nonDEG	0.18296853	0.63895704
nonDEG	-0.3478808	0.4984245	1	nonDEG	-0.8970846	0.080735
nonDEG	1.12318873	0.01181329	0.85229819	nonDEG	-0.2433954	0.520571
nonDEG	0.03382169	0.94931266	1	nonDEG	-0.4998854	0.33838336
nonDEG	0.12941287	0.80513059	1	nonDEG	0.35049652	0.44995566
nonDEG	-0.5024757	0.34537412	1	nonDEG	-0.0169202	0.97802022
nonDEG	-0.6592673	0.31733115	1	nonDEG	1.2069669	0.02295186
nonDEG	-1.4398502	0.06034772	1	nonDEG	1.23286604	0.26725168
nonDEG	-0.0505325	0.90967712	1	nonDEG	-0.4209043	0.27163701

nonDEG	0.1332463	0.80221261	1	nonDEG	-0.2210972	0.66091224
nonDEG	0.18420413	0.71551696	1	nonDEG	-0.4030058	0.34744304
nonDEG	-0.0334607	0.96119902	1	nonDEG	0.14430229	0.82255004
nonDEG	0.33499631	0.74556716	1	nonDEG	-0.453128	0.79410683
nonDEG	0.00509965	0.99081111	1	nonDEG	-0.1534251	0.69126115
nonDEG	0.12929024	0.81886407	1	nonDEG	0.21857041	0.66482859
nonDEG	0.17031289	0.85994435	1	nonDEG	0.29100368	0.73969519
nonDEG	-0.0362659	0.93603878	1	nonDEG	-0.4902601	0.20887052
nonDEG	-0.18426	0.68643507	1	nonDEG	-0.1355279	0.74260956
nonDEG	-0.1816739	0.79757152	1	nonDEG	0.69332922	0.31353459
nonDEG	1.33200442	0.73504555	1	nonDEG	-0.7936711	1
nonDEG	-0.3241799	0.45501569	1	nonDEG	0.10880045	0.76809941
nonDEG	0.31245032	0.50115272	1	nonDEG	-0.4195282	0.3008814
nonDEG	0.09960689	0.81797227	1	nonDEG	-0.1668118	0.64606611
nonDEG	0.28264958	0.56089641	1	nonDEG	0.18449947	0.665395
nonDEG	0.0632842	0.89106103	1	nonDEG	-0.222758	0.54871744
nonDEG	0.41240602	0.37336531	1	nonDEG	-0.3786583	0.33508497
nonDEG	1.17886585	0.30228113	1	nonDEG	-0.2953871	0.86212336
nonDEG	-0.2207491	0.64490229	1	nonDEG	-0.1411281	0.75356997
nonDEG	1.63224459	0.01300201	0.86208573	nonDEG	-0.7771446	0.12241763
nonDEG	-0.398307	0.37437143	1	nonDEG	0.29572598	0.46971359
nonDEG	0.05627132	0.89972619	1	nonDEG	-0.7632682	0.06462385
nonDEG	-0.0040329	1	1	nonDEG	0.71564919	0.80748019
nonDEG	0.23696542	0.62567467	1	nonDEG	-0.2986533	0.47912765
nonDEG	0.1241288	0.77180208	1	nonDEG	0.04315054	0.9055142
nonDEG	0.10666875	0.81050391	1	nonDEG	0.0670452	0.85538339
nonDEG	-0.3869608	0.37304328	1	nonDEG	0.22775864	0.55172646
nonDEG	0.63828591	0.35482799	1	nonDEG	0.19484189	0.78001141
nonDEG	-0.2266836	0.61921139	1	nonDEG	0.22156041	0.57045051
nonDEG	-0.1933586	0.66221748	1	nonDEG	0.15460707	0.67973027
nonDEG	0.08333138	0.84746332	1	nonDEG	-0.0622443	0.86768607
nonDEG	0.6384263	0.30868263	1	nonDEG	-0.5810432	0.3487384
nonDEG	-0.00049	0.99977453	1	nonDEG	0.01842343	0.96049611
nonDEG	-0.2568179	0.59765432	1	nonDEG	0.34683035	0.40405565
nonDEG	0.51543172	0.26110928	1	nonDEG	-0.1209549	0.75964363
nonDEG	1.02510966	0.06314191	1	nonDEG	-0.0079473	0.98764199
nonDEG	-0.4129864	0.40797087	1	nonDEG	-0.1524955	0.72158356
nonDEG	0.14384182	0.7963393	1	nonDEG	-0.1385996	0.79701799
nonDEG	0.67972048	0.18707776	1	nonDEG	-0.3728404	0.37967259
nonDEG	-0.2867864	0.50504008	1	nonDEG	0.16688259	0.64914725
nonDEG	-0.1831437	0.69078023	1	nonDEG	-0.3997049	0.30140449
nonDEG	0.22789364	0.60603331	1	nonDEG	-0.0255104	0.94575982
nonDEG	-0.4268422	0.43078391	1	nonDEG	-0.2275315	0.60691943

nonDEG	0.93395989	0.38836199	1	nonDEG	0.83155728	0.4135629
nonDEG	0.25039635	0.61701508	1	nonDEG	-0.7989719	0.07092672
nonDEG	0.24002645	0.63567297	1	nonDEG	-0.009957	0.98506093
nonDEG	-0.1255021	0.76333626	1	nonDEG	0.02011454	0.95724379
nonDEG	0.17423396	0.68451856	1	nonDEG	0.32946505	0.36893164
nonDEG	0.02660479	0.95211863	1	nonDEG	-0.0112744	0.97651758
nonDEG	-0.0377247	0.93105879	1	nonDEG	-0.084791	0.81826271
nonDEG	-0.3690079	0.41201386	1	nonDEG	0.15333042	0.71110462
nonDEG	-0.2792981	0.53958526	1	nonDEG	0.0798118	0.82885881
nonDEG	-0.0078687	0.98566075	1	nonDEG	-0.0573639	0.87505363
nonDEG	-0.2127438	0.62911682	1	nonDEG	-0.1197962	0.75893638
nonDEG	-0.3560613	0.46210764	1	nonDEG	0.1325503	0.75986245
nonDEG	0.03201939	0.94682839	1	nonDEG	-0.336909	0.38809288
nonDEG	0.05212539	0.9207178	1	nonDEG	-0.2980165	0.54108714
nonDEG	0.34535131	0.46568466	1	nonDEG	-0.3511776	0.38845553
nonDEG	0.50983139	0.54126561	1	nonDEG	0.77867732	0.28885718
nonDEG	0.40526454	0.52152646	1	nonDEG	-0.1810526	0.72323506
nonDEG	0.37358208	0.47146248	1	nonDEG	-0.0798362	0.87143493
nonDEG	-0.1292986	0.76563231	1	nonDEG	0.33003182	0.37277727
nonDEG	0.19318765	0.67306979	1	nonDEG	0.21193272	0.60385488
nonDEG	0.21723892	0.62645707	1	nonDEG	0.25729316	0.51116834
nonDEG	0.19531662	0.73283508	1	nonDEG	-1.4691895	0.00081431
nonDEG	0.35105341	0.4391981	1	nonDEG	0.003916	0.99215542
nonDEG	0.17721882	0.91629559	1	nonDEG	-2.5873369	0.14571531
nonDEG	-0.0753922	0.86634707	1	nonDEG	-0.1274028	0.7302614
nonDEG	0.39031868	0.42139975	1	nonDEG	-0.2372395	0.51976738
nonDEG	-0.2577035	0.55249159	1	nonDEG	-0.4009414	0.31464199
nonDEG	-0.6333435	0.35949612	1	nonDEG	0.24598195	0.72365163
nonDEG	0.24707604	0.64000515	1	nonDEG	-0.6598683	0.14256957
nonDEG	-0.0917489	0.85005559	1	nonDEG	0.00339099	0.99583951
nonDEG	-0.2040554	0.63404179	1	nonDEG	0.06876701	0.85087922
nonDEG	-0.0431974	0.94213004	1	nonDEG	0.11772785	0.82011931
nonDEG	0.5476642	0.34581523	1	nonDEG	-0.6542229	0.14495169
nonDEG	0.04485818	0.93561571	1	nonDEG	0.04225815	0.9359253
nonDEG	-0.343704	0.49189601	1	nonDEG	-0.2626758	0.59379691
nonDEG	0.2437481	0.57510632	1	nonDEG	-0.0461307	0.90112742
nonDEG	0.11484769	0.79694768	1	nonDEG	-0.0636325	0.87385148
nonDEG	0.26228688	0.76896657	1	nonDEG	0.55802633	0.52381341
nonDEG	0.48390598	0.50998821	1	nonDEG	-0.1090148	0.88981212
nonDEG	-0.1740279	0.69167674	1	nonDEG	-0.0608333	0.87580866
nonDEG	-0.2284929	0.68473689	1	nonDEG	-0.3318368	0.57835411
nonDEG	-0.1721881	0.6950037	1	nonDEG	0.27625761	0.46100781
nonDEG	0.3453855	0.44011957	1	nonDEG	-0.485365	0.21024796

nonDEG	0.07684342	0.86097984	1	nonDEG	0.45110819	0.22108433
nonDEG	0.16404933	0.70990232	1	nonDEG	-0.6738258	0.08847494
nonDEG	-3.2901143	0.64235586	1	nonDEG	-5.13E-15	1
nonDEG	-0.0925815	0.83933556	1	nonDEG	-0.4250553	0.29851074
nonDEG	0.09224948	0.8296447	1	nonDEG	0.02710621	0.94431197
nonDEG	0.20350139	0.67730426	1	nonDEG	-0.0208661	0.9655683
nonDEG	0.00905247	0.98345962	1	nonDEG	-0.0317999	0.93140081
nonDEG	-0.1825596	0.66933871	1	nonDEG	0.06349317	0.86091801
nonDEG	-0.1113016	0.79664369	1	nonDEG	0.34058901	0.39032849
nonDEG	-0.0333465	0.94461349	1	nonDEG	-0.6170897	0.14793835
nonDEG	0.86591525	0.32659444	1	nonDEG	0.05479845	0.9619497
nonDEG	0.76917016	0.0774205	1	nonDEG	-0.0951383	0.79324915
nonDEG	-1.0405733	0.39253005	1	nonDEG	-2.0721095	0.26076352
nonDEG	0.11207909	0.84369361	1	nonDEG	0.5626741	0.22594227
nonDEG	-0.1565028	0.74005873	1	nonDEG	0.00578489	0.99153474
nonDEG	0.21660901	0.61264185	1	nonDEG	-0.1591463	0.67073473
nonDEG	0.04291653	0.92746598	1	nonDEG	-0.4478571	0.28668465
nonDEG	-0.1347841	0.77441526	1	nonDEG	-0.0423531	0.91258039
nonDEG	-0.3424436	0.41951476	1	nonDEG	0.1510225	0.6806274
nonDEG	-0.305336	0.48992491	1	nonDEG	0.02360209	0.95368663
nonDEG	-0.1193392	0.77714957	1	nonDEG	-0.2996989	0.41075319
nonDEG	-0.1694188	0.69677411	1	nonDEG	-0.2737625	0.45805654
nonDEG	-1.0442726	0.13041088	1	nonDEG	0.09957738	0.89237842
nonDEG	0.61190368	0.26788515	1	nonDEG	-0.4242888	0.41248275
nonDEG	-3.1298099	0.64347562	1	nonDEG	3.54695909	0.53590515
nonDEG	-0.1776744	0.73162164	1	nonDEG	0.39177774	0.40244325
nonDEG	-0.1486937	0.72641436	1	nonDEG	-0.1085546	0.76540044
nonDEG	0.07680903	0.86538617	1	nonDEG	-0.1716219	0.65746176
nonDEG	-0.2921464	0.52388163	1	nonDEG	0.13544784	0.75298846
nonDEG	-0.1269035	0.78609182	1	nonDEG	-0.403227	0.34036572
nonDEG	0.05218622	0.90864078	1	nonDEG	0.07905601	0.83058171
nonDEG	0.73226644	0.10043981	1	nonDEG	-0.1753646	0.64436466
nonDEG	0.14922673	0.77386286	1	nonDEG	0.11567291	0.81033059
nonDEG	0.07776644	0.90590159	1	nonDEG	0.4499049	0.43149984
nonDEG	-0.1750117	0.68512941	1	nonDEG	0.14435817	0.69237531
nonDEG	0.35089629	0.4533583	1	nonDEG	-0.5334668	0.1667328
nonDEG	-0.0903875	0.82928819	1	nonDEG	0.14646891	0.68158767
nonDEG	-0.0502644	0.91041315	1	nonDEG	-0.2945579	0.47601995
nonDEG	0.16351689	0.70631096	1	nonDEG	0.00699056	0.98524435
nonDEG	-0.345328	0.49901759	1	nonDEG	0.06065262	0.90306066
nonDEG	-0.0439037	0.92557437	1	nonDEG	-0.0077864	0.98729645
nonDEG	0.11678036	0.78926394	1	nonDEG	-0.2258991	0.54553485
nonDEG	-0.3198876	0.48841081	1	nonDEG	0.42746937	0.28977005

nonDEG	-0.2101475	0.62252199	1	nonDEG	0.08106313	0.82173114
nonDEG	-0.299457	0.47351004	1	nonDEG	0.16783128	0.64200668
nonDEG	0.17196097	0.68737721	1	nonDEG	-0.1831028	0.6167767
nonDEG	-0.0933804	0.83320909	1	nonDEG	0.07306166	0.85306773
nonDEG	-4.9175993	0.10262505	1	nonDEG	-5.13E-15	1
nonDEG	0.67754612	0.14947183	1	nonDEG	-0.3233301	0.44141565
nonDEG	-0.252799	0.81142064	1	nonDEG	-0.0708904	0.95433603
nonDEG	-0.4156985	0.34259802	1	nonDEG	-0.0874164	0.81581399
nonDEG	0.21331007	0.67304991	1	nonDEG	-0.459502	0.27525032
nonDEG	0.21724707	0.6164907	1	nonDEG	-0.2858371	0.45710948
nonDEG	-0.5104433	0.24475666	1	nonDEG	-0.3400319	0.41297659
nonDEG	-0.0076052	0.98660385	1	nonDEG	0.21898541	0.54884651
nonDEG	-0.3298871	0.77131397	1	nonDEG	-3.918862	0.02729286
nonDEG	0.19569259	0.69405161	1	nonDEG	-0.1665115	0.71781107
nonDEG	-0.1638776	0.71087605	1	nonDEG	0.19010944	0.63107195
nonDEG	0.4830813	0.33421372	1	nonDEG	0.20111828	0.62036855
nonDEG	-1.2710485	0.31813572	1	nonDEG	2.17291961	0.05890544
nonDEG	0.11932325	0.80014043	1	nonDEG	0.01721466	0.96873526
nonDEG	-0.2240541	0.61566276	1	nonDEG	-0.4117593	0.290083
nonDEG	-0.0691198	0.87375316	1	nonDEG	0.10360515	0.78473454
nonDEG	-6.2006891	0.02587063	1	nonDEG	5.23392279	0.20505509
nonDEG	0.14552727	0.73572314	1	nonDEG	0.06114882	0.87076499
nonDEG	-0.0089497	0.98383534	1	nonDEG	0.11952947	0.74385708
nonDEG	-0.0141028	0.9747898	1	nonDEG	0.11880024	0.75389928
nonDEG	0.26237362	0.5410825	1	nonDEG	0.02630312	0.94250534
nonDEG	-0.2132559	0.72968599	1	nonDEG	0.29195303	0.62752518
nonDEG	0.65124368	0.3018945	1	nonDEG	-0.0181753	0.96837079
nonDEG	0.15687604	0.71311549	1	nonDEG	0.11322592	0.7578395
nonDEG	-0.1710392	0.69011143	1	nonDEG	0.01401885	0.97036096
nonDEG	-1.9430145	0.04630817	1	nonDEG	1.18925464	0.35118305
nonDEG	-0.0798251	0.86454381	1	nonDEG	0.33259533	0.42637486
nonDEG	-0.030962	0.95269822	1	nonDEG	0.18433854	0.6669693
nonDEG	0.36513202	0.59883917	1	nonDEG	-0.9289385	0.16339799
nonDEG	-0.0511984	0.91886554	1	nonDEG	-0.3621854	0.40064519
nonDEG	-0.0940726	0.82568772	1	nonDEG	0.2498282	0.49521324
nonDEG	-0.1228159	0.77621656	1	nonDEG	-0.4342309	0.23211509
nonDEG	0.34104737	0.42486475	1	nonDEG	0.13181117	0.72085605
nonDEG	-1.2974354	0.2843274	1	nonDEG	0.12065717	1
nonDEG	0.53731508	0.21589473	1	nonDEG	-0.1649835	0.65790202
nonDEG	0.06442913	0.89512105	1	nonDEG	-0.4298464	0.31087713
nonDEG	-0.0699952	0.88579425	1	nonDEG	0.10849583	0.80546885
nonDEG	0.59337076	0.33967882	1	nonDEG	-0.9175036	0.12472489
nonDEG	0.38150358	0.4634602	1	nonDEG	-0.5693183	0.2418746

nonDEG	0.88002657	0.19569086	1	nonDEG	-0.7196939	0.23590371
nonDEG	1.12947228	0.63075754	1	nonDEG	-2.955194	0.37383596
nonDEG	-0.0237831	0.95551758	1	nonDEG	-0.0526876	0.88455219
nonDEG	-0.2568744	0.55229517	1	nonDEG	0.83829289	0.02823261
nonDEG	0.13998894	0.77433975	1	nonDEG	0.08890514	0.83530807
nonDEG	-0.0116544	0.97861418	1	nonDEG	-0.1047789	0.77106149
nonDEG	0.80056198	0.4031445	1	nonDEG	0.62556474	0.49696489
nonDEG	-0.5387056	0.35226533	1	nonDEG	-0.2242456	0.68577079
nonDEG	1.87853513	0.01416246	0.88752292	nonDEG	-0.9832694	0.18012823
nonDEG	-0.3206214	0.45250396	1	nonDEG	-0.1190365	0.75606142
nonDEG	1.23953487	0.67202431	1	nonDEG	-5.6773769	0.15940056
nonDEG	-0.1564874	0.71492919	1	nonDEG	-0.168317	0.66023052
nonDEG	-0.4779039	0.30869645	1	nonDEG	-0.0332075	0.94399576
nonDEG	-0.1400574	0.74365922	1	nonDEG	0.0994341	0.78337023
nonDEG	-0.1261369	0.77188286	1	nonDEG	0.03762951	0.92438644
nonDEG	-0.1564314	0.75741941	1	nonDEG	0.17239935	0.7168614
nonDEG	-0.2516462	0.87331263	1	nonDEG	-0.8617064	0.60440573
nonDEG	-0.1545223	0.72078957	1	nonDEG	-0.5833373	0.10949875
nonDEG	-1.692688	0.26960018	1	nonDEG	1.11415151	0.6546134
nonDEG	0.21228572	0.6209911	1	nonDEG	-0.1978729	0.59462487
nonDEG	0.31418559	0.51412618	1	nonDEG	-0.1171324	0.7660885
nonDEG	0.23339714	0.57570793	1	nonDEG	0.56301484	0.12327758
nonDEG	-0.2286294	0.59947928	1	nonDEG	-0.3308521	0.36093516
nonDEG	-0.1105302	0.81174614	1	nonDEG	-0.3138177	0.45303103
nonDEG	0.15346557	0.72645345	1	nonDEG	0.13760479	0.70521177
nonDEG	0.44719851	0.33813174	1	nonDEG	-0.2124611	0.56173658
nonDEG	-2.3013586	1	1	nonDEG	7.54974257	0.00128334
nonDEG	0.03803166	0.93341424	1	nonDEG	0.05841068	0.88583674
nonDEG	0.22727745	0.87470636	1	nonDEG	-0.8648834	0.55011231
nonDEG	-0.0431721	0.92200413	1	nonDEG	-0.1788086	0.62365213
nonDEG	-0.3656414	0.42637138	1	nonDEG	-0.468926	0.30706227
nonDEG	-0.2124921	0.62445059	1	nonDEG	0.1756074	0.63134662
nonDEG	0.56976789	0.19296233	1	nonDEG	-0.3774703	0.33045954
nonDEG	-0.173497	0.6821162	1	nonDEG	0.14809102	0.68236045
nonDEG	0.30650794	0.49215997	1	nonDEG	0.07722103	0.84003737
nonDEG	-0.1249024	0.77318479	1	nonDEG	-0.0537997	0.88462961
nonDEG	-0.4629957	0.40432309	1	nonDEG	0.42708079	0.40699667
nonDEG	0.08385842	0.88974719	1	nonDEG	-0.2201062	0.71480932
nonDEG	-0.0854226	0.83887718	1	nonDEG	-0.0185333	0.96002245
nonDEG	0.31196465	0.50651435	1	nonDEG	-0.4803364	0.19995205
nonDEG	0.02418509	0.95451442	1	nonDEG	0.02787133	0.93883
nonDEG	-0.2299599	0.73999128	1	nonDEG	0.70201298	0.27370848
nonDEG	0.2331033	0.58381717	1	nonDEG	-0.036801	0.92109423

nonDEG	-0.3760482	0.40563324	1	nonDEG	0.39827632	0.31147254
nonDEG	0.87073746	0.15357081	1	nonDEG	0.25685249	0.62144331
nonDEG	0.60132924	0.16742369	1	nonDEG	-0.0545572	0.91064283
nonDEG	-0.0646537	1	1	nonDEG	3.17931551	0.14037865
nonDEG	2.24034548	0.09726026	1	nonDEG	-0.4593231	0.74377583
nonDEG	0.00918921	0.98419842	1	nonDEG	-0.2471805	0.5343785
nonDEG	-0.2544038	0.63438959	1	nonDEG	0.29095676	0.55974138
nonDEG	-0.1853625	0.67248138	1	nonDEG	0.25684284	0.49497672
nonDEG	0.00047494	1	1	nonDEG	-0.3046878	0.474407
nonDEG	-0.1628214	0.7523227	1	nonDEG	-0.6353152	0.19777303
nonDEG	-0.0457501	0.92700663	1	nonDEG	-0.3919289	0.3172504
nonDEG	0.31847535	0.73057776	1	nonDEG	1.14345131	0.16731411
nonDEG	0.10529448	0.89768099	1	nonDEG	0.66280423	0.42556962
nonDEG	0.18765514	0.83230852	1	nonDEG	-0.4985664	0.55681813
nonDEG	1.08162726	0.01234658	0.85229819	nonDEG	-0.2573335	0.48405206
nonDEG	-0.5357517	0.29980763	1	nonDEG	-0.1676629	0.69624535
nonDEG	-0.0323607	0.94002588	1	nonDEG	0.07151954	0.84405283
nonDEG	-0.2332575	0.59410692	1	nonDEG	0.15653304	0.66320029
nonDEG	0.34103304	0.56924121	1	nonDEG	-0.5236279	0.29164225
nonDEG	-0.1701674	0.69218709	1	nonDEG	-0.9405828	0.02505317
nonDEG	0.49447346	0.24441354	1	nonDEG	-0.4446112	0.23363803
nonDEG	-0.0605227	0.90936571	1	nonDEG	-0.4166417	0.33576781
nonDEG	0.43428285	0.73984087	1	nonDEG	0.74173381	0.55230578
nonDEG	-0.0800954	0.86093359	1	nonDEG	0.02908108	0.93752888
nonDEG	-0.2921374	0.52793014	1	nonDEG	0.02531771	0.95123194
nonDEG	-1.791872	0.61866448	1	nonDEG	1.54405371	0.59937366
nonDEG	-0.1658138	0.72896782	1	nonDEG	-0.317268	0.51413725
nonDEG	-0.5151878	0.26002899	1	nonDEG	0.27175209	0.47052843
nonDEG	0.04689214	0.92237888	1	nonDEG	-0.6713582	0.10400129
nonDEG	0.34525569	0.59341017	1	nonDEG	-0.9393938	0.0543564
nonDEG	-0.3126021	0.47272684	1	nonDEG	0.06885868	0.85129453
nonDEG	-0.2743768	0.53280252	1	nonDEG	0.15558198	0.67836498
nonDEG	0	1	1	nonDEG	-5.13E-15	1
nonDEG	-0.2045876	0.65671947	1	nonDEG	-0.38231	0.37377319
nonDEG	-0.2635292	0.62993395	1	nonDEG	0.29159969	0.56268506
nonDEG	-0.2562411	0.62382699	1	nonDEG	0.75080205	0.09711934
nonDEG	-0.5941726	0.35750081	1	nonDEG	0.55321771	0.37113515
nonDEG	-0.161689	0.7204192	1	nonDEG	-0.2976089	0.44460002
nonDEG	0.03175691	0.94262294	1	nonDEG	-0.0904824	0.81272891
nonDEG	-4.0289668	0.35520104	1	nonDEG	-5.13E-15	1
nonDEG	0.26059116	0.69522358	1	nonDEG	0.60623321	0.32492898
nonDEG	-0.0418278	0.92469634	1	nonDEG	-0.2786859	0.44464061
nonDEG	0.28295658	0.60371419	1	nonDEG	-0.2939543	0.56046533

nonDEG	-0.0163874	0.97212046	1	nonDEG	-0.1939181	0.60568904
nonDEG	0.04296815	0.92376709	1	nonDEG	-0.3019307	0.4243005
nonDEG	0.40571925	0.46173279	1	nonDEG	-0.4958274	0.34407218
nonDEG	0.25587421	0.59846636	1	nonDEG	-0.3714724	0.42102121
nonDEG	0.16738151	0.75063804	1	nonDEG	-0.5087108	0.2535859
nonDEG	1.41314432	0.3785057	1	nonDEG	-0.7758118	0.73319139
nonDEG	-0.1779212	0.67393772	1	nonDEG	0.26337965	0.46765234
nonDEG	-1.5637198	0.32333642	1	nonDEG	-1.2057305	0.82518543
nonDEG	0.12240713	0.79656433	1	nonDEG	-0.0719703	0.86072117
nonDEG	0.05384134	0.90562145	1	nonDEG	-0.0079272	0.98692491
nonDEG	0	1	1	nonDEG	-5.13E-15	1
nonDEG	-0.327958	0.45541606	1	nonDEG	-0.0899034	0.80081767
nonDEG	0.08635936	0.84420127	1	nonDEG	-0.3173466	0.38835159
nonDEG	0.32622833	0.55455403	1	nonDEG	-0.1449919	0.75367327
nonDEG	0.59006542	0.42561171	1	nonDEG	0.03951326	0.95656491
nonDEG	-0.1307694	0.75946329	1	nonDEG	-0.1755041	0.62776802
nonDEG	-0.0808739	0.86082279	1	nonDEG	-0.1691717	0.6925307
nonDEG	-0.2791195	0.57096232	1	nonDEG	0.2788794	0.44426263
nonDEG	0.68962319	0.34674961	1	nonDEG	-0.5390016	0.45965826
nonDEG	1.43730786	0.47508019	1	nonDEG	-1.8828131	0.43277461
nonDEG	-0.3364939	0.49572161	1	nonDEG	-0.0553689	0.91790646
nonDEG	-0.0570689	0.89387001	1	nonDEG	0.23287049	0.52399091
nonDEG	-0.2031239	0.66620438	1	nonDEG	0.3490153	0.42079299
nonDEG	-0.415037	0.44973333	1	nonDEG	-0.2791312	0.53797325
nonDEG	-0.3042504	0.5128773	1	nonDEG	-0.2117609	0.59027957
nonDEG	0.2420423	0.59118292	1	nonDEG	-0.3414533	0.41515819
nonDEG	0.08957383	0.83930249	1	nonDEG	-0.3148053	0.42426746
nonDEG	0.26801257	0.82045321	1	nonDEG	0.50325608	0.63457906
nonDEG	0.90113979	0.18267365	1	nonDEG	-0.1412248	0.83065743
nonDEG	0.13222693	0.76679995	1	nonDEG	0.08970621	0.81962921
nonDEG	0.91281434	0.03225443	1	nonDEG	-0.2099588	0.56611183
nonDEG	-0.0989391	0.81399214	1	nonDEG	0.17525148	0.63348658
nonDEG	-0.0387854	0.92796164	1	nonDEG	0.29911436	0.41452316
nonDEG	0.26269487	0.74059611	1	nonDEG	-1.200593	0.04139023
nonDEG	0.85170193	0.11840918	1	nonDEG	-0.3359059	0.50090149
nonDEG	-2.7627043	0.08894024	1	nonDEG	3.59028152	0.13998189
nonDEG	-0.0118843	0.97801287	1	nonDEG	-0.2089159	0.5626914
nonDEG	0.15162244	0.75311802	1	nonDEG	0.10935406	0.80927599
nonDEG	0.44902164	0.32561679	1	nonDEG	-0.5822173	0.23250842
nonDEG	0.43342018	0.32142691	1	nonDEG	0.00963799	0.98003941
nonDEG	-0.0470657	0.92838648	1	nonDEG	-0.5584923	0.26504823
nonDEG	-0.3889494	0.38215283	1	nonDEG	0.21498479	0.55550388
nonDEG	0.20851141	0.69496417	1	nonDEG	0.22539	0.63923709

nonDEG	-0.325434	0.49522238	1	nonDEG	0.29337727	0.51892712
nonDEG	1.72281407	0.02537319	1	nonDEG	-1.1566805	0.10839092
nonDEG	-0.4541258	0.4568058	1	nonDEG	0.17132028	0.77481011
nonDEG	0.34979359	0.40749848	1	nonDEG	-0.0152062	0.96773802
nonDEG	0.01474777	0.97463083	1	nonDEG	0.4007167	0.30527847
nonDEG	0.1020686	0.83775726	1	nonDEG	-0.1162436	0.77885736
nonDEG	-0.2321586	0.69424074	1	nonDEG	0.27977224	0.61712295
nonDEG	-0.4258759	0.34704671	1	nonDEG	-0.1046708	0.80797506
nonDEG	0.8484851	0.06041465	1	nonDEG	0.1832682	0.63847696
nonDEG	-0.2248743	0.59457082	1	nonDEG	0.25615787	0.48084212
nonDEG	1.18797887	0.00559312	0.73507048	nonDEG	-0.7736336	0.0350673
nonDEG	-0.2143191	0.64918391	1	nonDEG	0.09701618	0.81740837
nonDEG	-0.1883304	0.66409201	1	nonDEG	0.06945922	0.84956361
nonDEG	-0.4549949	0.31057252	1	nonDEG	0.30357601	0.40966965
nonDEG	0.96923444	0.02594516	1	nonDEG	-0.5577252	0.1222483
nonDEG	0.28543018	0.50044351	1	nonDEG	-0.3957429	0.27051743
nonDEG	0.18192188	0.66459046	1	nonDEG	0.0203532	0.95568971
nonDEG	-0.3633111	0.55003136	1	nonDEG	0.60490222	0.30905963
nonDEG	-0.9879175	0.44865286	1	nonDEG	1.03082464	0.52746001
nonDEG	-0.227841	0.60710654	1	nonDEG	-0.706403	0.11813825
nonDEG	0.1442767	0.73397063	1	nonDEG	0.12856162	0.72278707
nonDEG	0.0875945	0.84097901	1	nonDEG	-0.5400992	0.14061778
nonDEG	-0.2240906	0.68310757	1	nonDEG	0.18460431	0.71939295
nonDEG	0.27237503	0.51472192	1	nonDEG	0.02786898	0.93873177
nonDEG	-0.1344501	0.80030062	1	nonDEG	-0.1798158	0.7250177
nonDEG	-0.182783	0.7876859	1	nonDEG	0.16634636	0.80860344
nonDEG	-0.1601669	0.80193507	1	nonDEG	0.40264433	0.47018549
nonDEG	0.08154458	0.8528377	1	nonDEG	-0.0092292	0.98036167
nonDEG	-0.40245	0.36079367	1	nonDEG	0.16539133	0.65657531
nonDEG	-0.2777321	0.55998002	1	nonDEG	0.59897426	0.12838039
nonDEG	0.04468883	0.9167458	1	nonDEG	-0.1517564	0.67524856
nonDEG	-0.209198	0.66596338	1	nonDEG	0.07445281	0.87219738
nonDEG	-0.0810156	0.8551503	1	nonDEG	0.12087251	0.74224434
nonDEG	-0.2657168	0.54840332	1	nonDEG	0.14471218	0.70673166
nonDEG	0.1628864	0.70832393	1	nonDEG	-0.0362415	0.92612195
nonDEG	0.10553185	0.80618977	1	nonDEG	-0.0300503	0.93473802
nonDEG	0.02828067	0.9544177	1	nonDEG	0.065444	0.87383658
nonDEG	0.14061703	0.81846634	1	nonDEG	0.06127315	0.9142685
nonDEG	-0.1769356	0.754848	1	nonDEG	0.56408551	0.24557914
nonDEG	0.07238782	0.89134562	1	nonDEG	-0.2180566	0.62376782
nonDEG	0.47964303	0.3456527	1	nonDEG	0.03494216	0.93300649
nonDEG	-0.1838213	0.67110788	1	nonDEG	0.11511214	0.75395825
nonDEG	0.2491215	0.57436776	1	nonDEG	0.19199838	0.6120897

nonDEG	-0.1020593	0.81285197	1	nonDEG	0.18702112	0.61134718
nonDEG	-0.0846738	0.85294221	1	nonDEG	-0.2197305	0.54915569
nonDEG	0.25582263	1	1	nonDEG	-1.3020726	0.58070187
nonDEG	-0.1772718	0.81051224	1	nonDEG	-0.2375756	0.74010911
nonDEG	0.51572151	0.43774901	1	nonDEG	-0.4153416	0.52445065
nonDEG	0.57947912	0.22113572	1	nonDEG	-0.3077719	0.43458419
nonDEG	-0.1938338	0.66357504	1	nonDEG	-0.6338765	0.14574135
nonDEG	0.14697286	0.73247995	1	nonDEG	0.05175485	0.88726061
nonDEG	0.66419967	0.31158302	1	nonDEG	-0.4027333	0.5169698
nonDEG	-0.3351134	0.4360303	1	nonDEG	0.38559604	0.29416386
nonDEG	-0.2420973	0.59037244	1	nonDEG	-0.2207298	0.55210341
nonDEG	-0.2586548	0.59315313	1	nonDEG	0.19274515	0.61370236
nonDEG	-0.3032463	0.47710295	1	nonDEG	0.36315939	0.33063959
nonDEG	0.28679751	0.50400584	1	nonDEG	-0.2182411	0.55631962
nonDEG	0.75968241	0.12966322	1	nonDEG	-0.4226532	0.3535621
nonDEG	0.80523675	0.05975499	1	nonDEG	-0.2599642	0.47544635
nonDEG	-4.0291185	0.35406811	1	nonDEG	3.05429913	0.69650097
nonDEG	0.35396795	0.40758323	1	nonDEG	-0.245043	0.501243
nonDEG	-0.2977202	0.52497475	1	nonDEG	-0.2910936	0.44347867
nonDEG	0.75129203	0.22541252	1	nonDEG	0.10337809	0.8582104
nonDEG	-0.23213	0.61088716	1	nonDEG	0.10400512	0.80368794
nonDEG	-0.3440511	0.56076414	1	nonDEG	-0.0928235	0.89211338
nonDEG	-0.5683368	0.32090154	1	nonDEG	0.51859103	0.39180804
nonDEG	-0.0985626	0.82331464	1	nonDEG	-0.2571306	0.53048976
nonDEG	0.05042652	0.9099934	1	nonDEG	-0.2042282	0.59503911
nonDEG	-0.0723457	0.8667549	1	nonDEG	-0.1983137	0.6004573
nonDEG	0.26479429	0.80688277	1	nonDEG	-0.6190294	0.58831455
nonDEG	0.04649284	0.91329047	1	nonDEG	0.02470821	0.94704217
nonDEG	-0.3800756	0.39020908	1	nonDEG	-0.0603039	0.88615918
nonDEG	0.21791935	0.63496188	1	nonDEG	-0.2902215	0.46557621
nonDEG	-0.1799885	0.67794222	1	nonDEG	-0.0096816	0.98094283
nonDEG	-1.6914996	0.28813792	1	nonDEG	2.6757619	0.07969825
nonDEG	-0.3210498	0.47812975	1	nonDEG	0.356709	0.36909204
nonDEG	-0.1280226	0.75927056	1	nonDEG	0.2261579	0.52662141
nonDEG	0.04943016	0.91689153	1	nonDEG	-0.3174473	0.46027961
nonDEG	-0.0926204	0.83865837	1	nonDEG	-0.4287078	0.31463915
nonDEG	-0.0197858	0.97170383	1	nonDEG	0.43034462	0.35078298
nonDEG	0.26519914	0.52952782	1	nonDEG	-0.0746124	0.83753749
nonDEG	0.78813703	0.48051288	1	nonDEG	-4.5292937	0.00802725
nonDEG	0.03815929	0.93255554	1	nonDEG	0.33166737	0.40436913
nonDEG	-0.0886101	0.83896676	1	nonDEG	-0.0022127	0.99571557
nonDEG	0.13708655	0.80652328	1	nonDEG	-0.3962511	0.46718435
nonDEG	-0.97632	0.11211267	1	nonDEG	-0.3324644	0.57243615

nonDEG	-0.0574741	0.89318466	1	nonDEG	0.06205031	0.86832818
nonDEG	-0.5136024	0.34933591	1	nonDEG	0.00542414	0.99017795
nonDEG	-0.4532624	0.34978256	1	nonDEG	0.03042261	0.93995032
nonDEG	0.54650393	0.28685701	1	nonDEG	-0.0913056	0.84974741
nonDEG	0.18166234	0.71495202	1	nonDEG	-0.3136937	0.41398002
nonDEG	-0.0994602	0.814171	1	nonDEG	0.01734952	0.96205841
nonDEG	-2.7571565	0.23623549	1	nonDEG	1.19289054	0.80504806
nonDEG	0.30961629	0.48134909	1	nonDEG	0.03363621	0.92923716
nonDEG	0.02730429	0.94908309	1	nonDEG	-0.0335354	0.92654865
nonDEG	0.17204111	0.70205317	1	nonDEG	-0.0687584	0.85771212
nonDEG	0.0633511	0.88356687	1	nonDEG	0.0687271	0.86621219
nonDEG	-0.2896338	0.50341898	1	nonDEG	-0.0223825	0.95232396
nonDEG	-1.6608566	0.06631879	1	nonDEG	0.69522311	0.33313045
nonDEG	-0.1241911	0.77126667	1	nonDEG	0.0782175	0.82904523
nonDEG	-0.1256104	0.81393719	1	nonDEG	0.81801932	0.10377545
nonDEG	-0.2714505	0.53599612	1	nonDEG	-0.0960321	0.79142
nonDEG	0.04642968	0.95011755	1	nonDEG	-0.2014229	0.77959332
nonDEG	0.33472493	0.51881536	1	nonDEG	-0.296877	0.47239307
nonDEG	-0.3441684	0.4432295	1	nonDEG	0.09703661	0.80259671
nonDEG	-0.0580602	0.92583339	1	nonDEG	0.12269584	0.82840762
nonDEG	-0.3326174	0.4706064	1	nonDEG	0.22723416	0.58153443
nonDEG	0.3074034	0.57492905	1	nonDEG	-0.1998076	0.68057369
nonDEG	-0.381264	0.36519415	1	nonDEG	-0.1884753	0.62582227
nonDEG	-0.1877877	0.68760695	1	nonDEG	-0.7231622	0.13550146
nonDEG	-0.5385508	0.5296577	1	nonDEG	0.5694891	0.53271047
nonDEG	-0.5674875	0.21398295	1	nonDEG	0.62552615	0.13087555
nonDEG	0.24491105	0.56273646	1	nonDEG	0.16411542	0.64745414
nonDEG	0.48039611	0.29452002	1	nonDEG	-0.1121452	0.7707904
nonDEG	0.34658055	0.43887434	1	nonDEG	-0.1899202	0.61173424
nonDEG	0.33693198	0.43951583	1	nonDEG	0.2145329	0.5769963
nonDEG	0.13272957	0.77912698	1	nonDEG	-0.3949262	0.31450881
nonDEG	0.75852739	0.15271066	1	nonDEG	-0.2477576	0.57158801
nonDEG	0.3710804	0.39324093	1	nonDEG	-0.0886374	0.80653562
nonDEG	0.12773341	0.77752195	1	nonDEG	-0.0102863	0.97981533
nonDEG	1.33199646	0.73500769	1	nonDEG	-0.7790706	1
nonDEG	-0.1969737	0.65998997	1	nonDEG	0.2218874	0.54718214
nonDEG	-0.3341428	0.78874543	1	nonDEG	-4.4089476	0.03713521
nonDEG	0.23200512	0.63241691	1	nonDEG	0.29497623	0.47862198
nonDEG	0.22243147	0.66495144	1	nonDEG	-0.0824726	0.83028046
nonDEG	0.36084861	0.54113995	1	nonDEG	1.0088814	0.06064069
nonDEG	0.38614715	0.64469472	1	nonDEG	0.31009728	0.63521778
nonDEG	0.44682305	0.48914628	1	nonDEG	-0.0491841	0.94097126
nonDEG	0.48464151	0.26996678	1	nonDEG	-0.1817654	0.63270122

nonDEG	0.51221662	0.30957394	1	nonDEG	-0.7262209	0.15793397
nonDEG	-0.035565	0.94807816	1	nonDEG	-0.1715654	0.73286025
nonDEG	-0.3356706	0.51304299	1	nonDEG	-0.0089329	0.99017154
nonDEG	-0.1173334	0.79256596	1	nonDEG	0.03756958	0.92819989
nonDEG	-0.3224309	0.56848153	1	nonDEG	0.32952435	0.55896009
nonDEG	-0.0502235	0.90732495	1	nonDEG	-0.0356681	0.92189449
nonDEG	3.78493654	0.46895232	1	nonDEG	-3.9875747	0.44124526
nonDEG	-0.1133884	0.8192793	1	nonDEG	0.1838	0.68196199
nonDEG	0.05416588	0.90692698	1	nonDEG	-0.5625164	0.20033269
nonDEG	0.07883901	0.85693496	1	nonDEG	-0.1302277	0.73988042
nonDEG	0.2192237	0.65923392	1	nonDEG	-0.0861997	0.83436228
nonDEG	-0.1309114	0.75743542	1	nonDEG	0.05124023	0.88990496
nonDEG	-0.3214898	0.48169698	1	nonDEG	0.42066751	0.30287763
nonDEG	-0.0169915	0.96982729	1	nonDEG	-0.0652832	0.86174779
nonDEG	-0.2788731	0.50914744	1	nonDEG	0.0022867	0.99568632
nonDEG	-0.2004572	0.64124115	1	nonDEG	0.01446419	0.96921325
nonDEG	-0.1318345	0.75597591	1	nonDEG	-0.0029128	0.99432988
nonDEG	-0.0936009	0.8338639	1	nonDEG	0.04391734	0.91136941
nonDEG	0.29230326	0.54338461	1	nonDEG	-0.2448296	0.57866436
nonDEG	0.05180883	0.91794245	1	nonDEG	-0.1922082	0.64996979
nonDEG	0.04564775	0.93646643	1	nonDEG	-0.1051959	0.84127173
nonDEG	0.00849214	0.98527306	1	nonDEG	0.05201036	0.88739962
nonDEG	0.31304644	0.4863525	1	nonDEG	-0.1313745	0.73712898
nonDEG	-0.1957276	0.66250083	1	nonDEG	-0.2553451	0.50671621
nonDEG	-0.5233812	0.29689069	1	nonDEG	-0.2697805	0.61788456
nonDEG	-0.1076135	0.80372524	1	nonDEG	0.14415863	0.69846884
nonDEG	0.18925064	0.72883159	1	nonDEG	-0.1864036	0.70748804
nonDEG	-0.0327847	0.93970573	1	nonDEG	-0.2218301	0.54067923
nonDEG	0.44067082	0.39962553	1	nonDEG	0.87188233	0.27331371
nonDEG	0.58348768	0.18621315	1	nonDEG	-0.313853	0.4237966
nonDEG	0.17413007	0.68752077	1	nonDEG	-0.184932	0.62644819
nonDEG	-0.0681473	0.87671334	1	nonDEG	-0.1077476	0.78390142
nonDEG	0.96257907	0.13598872	1	nonDEG	1.13052247	0.08332483
nonDEG	0.28704042	0.51880985	1	nonDEG	-0.277642	0.4634844
nonDEG	0.00043742	1	1	nonDEG	-0.7021866	0.11719188
nonDEG	0.24692667	0.64574072	1	nonDEG	-1.0701352	0.05246218
nonDEG	-1.0351482	0.44310241	1	nonDEG	-0.1170689	1
nonDEG	0.17942861	0.79312848	1	nonDEG	0.19368695	0.77976084
nonDEG	0.69665872	0.51434691	1	nonDEG	0.64463447	0.55704544
nonDEG	-0.1397266	0.89676601	1	nonDEG	0.94195746	0.2312309
nonDEG	-0.1853262	0.70354618	1	nonDEG	-0.1087898	0.81379049
nonDEG	0.22556385	0.68772382	1	nonDEG	-0.146476	0.78870385
nonDEG	-0.9315758	0.06520071	1	nonDEG	0.9388016	0.01504752

nonDEG	-0.4149736	0.55093488	1	nonDEG	0.6117303	0.35426738
nonDEG	0.37217203	1	1	nonDEG	2.06569086	0.29419513
nonDEG	0.05847101	0.89255672	1	nonDEG	0.01749459	0.9632474
nonDEG	-0.0155189	0.97416932	1	nonDEG	0.36804347	0.33083289
nonDEG	-0.25956	0.58134304	1	nonDEG	0.39496356	0.3425743
nonDEG	1.00201826	0.02648322	1	nonDEG	-0.2403925	0.5435247
nonDEG	-0.3244234	0.58468585	1	nonDEG	0.19624427	0.72904021
nonDEG	-0.6396619	0.19149452	1	nonDEG	0.40579774	0.39731953
nonDEG	-0.9922669	0.58725235	1	nonDEG	1.60235646	0.19886604
nonDEG	0.24347521	0.56787218	1	nonDEG	-0.1299186	0.71969027
nonDEG	0.20812153	0.63209161	1	nonDEG	0.05472837	0.88321494
nonDEG	0.30276267	0.52160059	1	nonDEG	0.1687878	0.68767959
nonDEG	-0.2084222	0.64810116	1	nonDEG	0.10808994	0.77063228
nonDEG	-0.2298096	0.64909879	1	nonDEG	0.26467091	0.53172277
nonDEG	0.13489518	0.7559799	1	nonDEG	-0.8951218	0.01753558
nonDEG	-0.630103	0.21060476	1	nonDEG	-0.1211787	0.79578867
nonDEG	0.34325932	0.46413884	1	nonDEG	-0.1809691	0.66902612
nonDEG	-0.3765813	0.38927225	1	nonDEG	-0.0697065	0.86323108
nonDEG	0.32951185	0.47200522	1	nonDEG	-0.0938151	0.81532923
nonDEG	-0.7577504	0.50626082	1	nonDEG	1.91136665	0.185449
nonDEG	-0.0710812	0.90337354	1	nonDEG	0.0491355	0.93626749
nonDEG	-0.388207	0.42271817	1	nonDEG	-0.0459065	0.9071114
nonDEG	-0.0512089	0.95173542	1	nonDEG	-0.146452	0.83798747
nonDEG	-0.2524758	0.64384137	1	nonDEG	-0.1844707	0.67817663
nonDEG	0.2425369	0.56835688	1	nonDEG	0.2097716	0.5890469
nonDEG	-0.1265786	0.78381757	1	nonDEG	-0.4377213	0.32271231
nonDEG	-4.4549555	0.19238093	1	nonDEG	6.40655608	0.02170241
nonDEG	-0.6250654	0.69516583	1	nonDEG	-1.8857164	0.52035678
nonDEG	0.23400401	0.63309949	1	nonDEG	-0.4437758	0.32931997
nonDEG	0.42791615	0.43469738	1	nonDEG	-0.4850134	0.26715318
nonDEG	-0.7804469	0.25363735	1	nonDEG	-0.1737781	0.77638257
nonDEG	0.5688656	0.31455763	1	nonDEG	-0.3069911	0.46750255
nonDEG	0.19186771	0.6565415	1	nonDEG	0.18723828	0.61741545
nonDEG	0.87662223	0.30239653	1	nonDEG	-1.1093994	0.22198706
nonDEG	-2.0444501	0.07050778	1	nonDEG	4.75013998	0.00012178
nonDEG	-0.2131759	0.63418201	1	nonDEG	0.0009569	0.99815985
nonDEG	0.39451482	0.37466716	1	nonDEG	0.48619861	0.21684177
nonDEG	0.12250221	0.77659505	1	nonDEG	-0.1652233	0.66110714
nonDEG	-0.5425174	1	1	nonDEG	-3.745066	0.51473558
nonDEG	0.55199324	0.43405345	1	nonDEG	-0.4911698	0.38288017
nonDEG	-0.0890477	0.84055369	1	nonDEG	-0.0628167	0.86666711
nonDEG	-0.0402374	0.93365844	1	nonDEG	-0.2842616	0.52575682
nonDEG	-0.2468924	0.68774238	1	nonDEG	0.61522198	0.2878845

nonDEG	10.1410501	0.0001596	0.22373747	nonDEG	-7.2884338	0.00085217
nonDEG	0	1	1	nonDEG	3.96666163	0.43260467
nonDEG	0.18412047	0.67010477	1	nonDEG	-0.2401293	0.53932598
nonDEG	-0.561073	0.38333442	1	nonDEG	-0.3195386	0.63161337
nonDEG	-0.1587779	0.71091917	1	nonDEG	0.1646982	0.65281843
nonDEG	0.09661271	0.88048287	1	nonDEG	0.30443678	0.58380312
nonDEG	0.38100134	0.55669471	1	nonDEG	-0.0576527	0.92664178
nonDEG	-0.0796719	0.85768023	1	nonDEG	-0.0243362	0.94889675
nonDEG	0.53410507	0.30930776	1	nonDEG	-0.5013415	0.21782048
nonDEG	0.05051584	0.91920742	1	nonDEG	-0.345445	0.37718023
nonDEG	0.73611558	0.1269474	1	nonDEG	-0.037264	0.92643995
nonDEG	-0.1920758	0.74112257	1	nonDEG	0.07039446	0.8965817
nonDEG	-0.3291461	0.54081173	1	nonDEG	-0.1881552	0.65879005
nonDEG	-0.639959	0.32796675	1	nonDEG	0.29496111	0.66057223
nonDEG	-0.0581817	0.89261829	1	nonDEG	0.20588748	0.5759768
nonDEG	0.0915175	0.87460392	1	nonDEG	0.69319338	0.14205176
nonDEG	-1.1938553	0.39259641	1	nonDEG	0.94965113	0.56093362
nonDEG	0.73354937	0.08831528	1	nonDEG	0.28563138	0.43052412
nonDEG	1.18365823	0.41446875	1	nonDEG	1.48695514	0.23698425
nonDEG	-1.0262964	0.47412381	1	nonDEG	0.76902519	0.60278234
nonDEG	-0.4508552	0.56169168	1	nonDEG	2.47396894	0.00666012
nonDEG	-1.1326782	0.7137399	1	nonDEG	2.54816962	0.17774241
nonDEG	-1.3926314	0.04292515	1	nonDEG	-1.9483533	0.01213883
nonDEG	-0.0725067	0.8759201	1	nonDEG	-0.2736962	0.5023147
nonDEG	-0.7563544	0.75849674	1	nonDEG	-0.1136744	1
nonDEG	-0.0268959	0.95151369	1	nonDEG	0.01496088	0.97009985
nonDEG	-0.8058743	0.28016501	1	nonDEG	0.93896289	0.17044263
nonDEG	0.98063593	0.12619861	1	nonDEG	-0.163163	0.78613547
nonDEG	-0.3693005	0.39664632	1	nonDEG	0.03668042	0.92480742
nonDEG	-0.6122241	0.27549891	1	nonDEG	1.2454197	0.03201987
nonDEG	-2.5028936	0.04094469	1	nonDEG	0.90873995	0.68385299
nonDEG	-0.5937293	0.31390877	1	nonDEG	0.54551926	0.24957391
nonDEG	0.10082178	0.84454569	1	nonDEG	0.84415364	0.05447643
nonDEG	-0.1990852	0.66802175	1	nonDEG	-0.0831344	0.82360438
nonDEG	-0.6411217	0.19261118	1	nonDEG	0.01142748	0.97659919
nonDEG	0.78636538	0.34747444	1	nonDEG	-0.4219702	0.61128798
nonDEG	-0.0119655	0.97951656	1	nonDEG	-0.2704895	0.48524874
nonDEG	0.02311735	0.95776485	1	nonDEG	-0.0326627	0.93261903
nonDEG	0.92241263	0.56851544	1	nonDEG	-1.8507227	0.13727825
nonDEG	-0.1291259	0.8443139	1	nonDEG	0.57020762	0.37911643
nonDEG	0.12821545	0.78011027	1	nonDEG	-0.0028568	0.9953653
nonDEG	-0.064835	0.94955077	1	nonDEG	-0.499812	0.55515492
nonDEG	0.29376811	0.53838471	1	nonDEG	-0.2943755	0.47040098

nonDEG	-0.1400304	0.75118186	1	nonDEG	0.00157752	0.99700807
nonDEG	-1.291392	0.01928546	0.97153571	nonDEG	1.55614576	0.01072596
nonDEG	-0.1224106	0.79249535	1	nonDEG	-0.0903332	0.80853774
nonDEG	0.39200422	0.37077656	1	nonDEG	-0.1125489	0.76503456
nonDEG	0.14268401	0.80028367	1	nonDEG	0.05710215	0.91222622
nonDEG	-0.7761512	0.24699472	1	nonDEG	0.50009054	0.460063
nonDEG	0.54786008	0.49467951	1	nonDEG	0.55033628	0.44983942
nonDEG	-0.9473652	0.30370371	1	nonDEG	1.52123784	0.0394029
nonDEG	1.0752237	0.05099719	1	nonDEG	-0.3278132	0.44635187
nonDEG	0.61956826	0.22965553	1	nonDEG	-0.3118429	0.52279536
nonDEG	2.97192768	0.1333924	1	nonDEG	-3.5544398	0.09410238
nonDEG	-2.3010649	1	1	nonDEG	7.29987282	0.01071533
nonDEG	-0.2416207	0.6427738	1	nonDEG	-0.2442275	0.67115244
nonDEG	0.01951376	0.97437308	1	nonDEG	0.6275873	0.21170274
nonDEG	5.13423871	0.00117449	0.51605879	nonDEG	0.17343656	0.8858452
nonDEG	0.11573532	0.8080209	1	nonDEG	0.25793023	0.54181292
nonDEG	-0.2516277	0.58209764	1	nonDEG	-0.8729642	0.08498757
nonDEG	-0.3115598	0.60453907	1	nonDEG	-0.0638257	0.91960491
nonDEG	6.53806042	0.03465405	1	nonDEG	-0.8474521	0.65229937
nonDEG	-0.3439865	0.75681161	1	nonDEG	0.89992036	0.31300227
nonDEG	-0.1836046	0.67240437	1	nonDEG	0.21053177	0.58080079
nonDEG	-0.6148502	0.35032416	1	nonDEG	0.51413979	0.37637269
nonDEG	-0.0814161	0.84914189	1	nonDEG	-0.4614507	0.2058656
nonDEG	0.0928827	0.83304234	1	nonDEG	-0.2847212	0.43983962
nonDEG	-1.0934022	0.07002313	1	nonDEG	1.08792244	0.11520806
nonDEG	-1.8042073	0.07157545	1	nonDEG	2.17548103	0.00026966
nonDEG	-1.7523815	0.25778024	1	nonDEG	1.58807739	0.40961688
nonDEG	0.09570819	0.83126028	1	nonDEG	0.17244096	0.63473562
nonDEG	0.70245658	0.2776024	1	nonDEG	0.11794783	0.82443822
nonDEG	0.0099326	0.99455658	1	nonDEG	0.91192363	0.09521949
nonDEG	0.10688392	0.8019953	1	nonDEG	-0.6016819	0.12561668
nonDEG	-0.5379161	0.26116546	1	nonDEG	-0.2033779	0.68884514
nonDEG	0.02091077	1	1	nonDEG	1.26031616	0.38694064
nonDEG	0.83019872	0.33903793	1	nonDEG	-0.143068	0.86701436
nonDEG	-0.0178344	0.96964287	1	nonDEG	-0.4913577	0.19764658
nonDEG	-0.1891994	0.75177393	1	nonDEG	-0.1183429	0.83459147
nonDEG	-0.1192406	0.91608389	1	nonDEG	1.06213783	0.27173587
nonDEG	0.07077586	0.88721202	1	nonDEG	-0.6101888	0.15923001
nonDEG	0.21029023	0.63832758	1	nonDEG	-0.1214342	0.73759393
nonDEG	0.07427118	0.86624487	1	nonDEG	-0.0560404	0.88172702
nonDEG	-0.416183	0.79531757	1	nonDEG	-0.8835077	0.74923856
nonDEG	1.88213361	0.01806716	0.94141084	nonDEG	-1.3032602	0.09080964
nonDEG	-0.3651614	0.52519464	1	nonDEG	-0.2296111	0.63142429

nonDEG	0.6800153	0.13279602	1	nonDEG	0.44971934	0.22614864
nonDEG	0.393961	0.39228913	1	nonDEG	-0.1707269	0.64023387
nonDEG	-5.372868	0.13445766	1	nonDEG	7.59606204	0.00102614
nonDEG	0.24147716	0.70845919	1	nonDEG	-0.8100463	0.06538927
nonDEG	-0.2520568	0.71046299	1	nonDEG	0.17761	0.72991348
nonDEG	-0.0447589	0.91965532	1	nonDEG	0.25214761	0.48734562
nonDEG	0.26797466	0.59195007	1	nonDEG	0.38786152	0.37222548
nonDEG	0.30631065	0.71735043	1	nonDEG	-0.6631397	0.45223414
nonDEG	0.88149845	0.15465897	1	nonDEG	-0.5255108	0.38010006
nonDEG	2.86777166	0.03536732	1	nonDEG	0.05347817	0.97921126
nonDEG	-1.0440291	0.33214125	1	nonDEG	1.60299372	0.21010309
nonDEG	-0.0032229	0.99468168	1	nonDEG	-0.1481323	0.72294969
nonDEG	-1.0222674	0.31302781	1	nonDEG	1.9093888	0.13946548
nonDEG	-1.2757434	0.30621359	1	nonDEG	1.87243753	0.18871368
nonDEG	5.59076899	8.03E-05	0.15769123	nonDEG	-4.9178017	0.00016841
nonDEG	0.03690591	0.98625201	1	nonDEG	0.70316927	0.39954224
nonDEG	0.02614023	0.96273054	1	nonDEG	0.31665239	0.51652106
nonDEG	-0.3150335	0.52473142	1	nonDEG	0.43734637	0.34447181
nonDEG	0.4819889	0.62440517	1	nonDEG	0.09045292	0.90981124
nonDEG	0.84953067	0.28431566	1	nonDEG	-0.4485394	0.52219923
nonDEG	-1.9609496	0.10495769	1	nonDEG	1.65894827	0.19434515
nonDEG	1.87411993	0.04770112	1	nonDEG	-0.5450469	0.52457251
nonDEG	-0.1201888	0.79988631	1	nonDEG	-0.1340947	0.75561344
nonDEG	-0.3941464	0.69030858	1	nonDEG	0.05811295	0.96458921
nonDEG	0.090658	0.85109622	1	nonDEG	-0.343035	0.45426076
nonDEG	-0.9724591	0.39133874	1	nonDEG	0.86413402	0.51921535
nonDEG	-0.1709538	0.70899868	1	nonDEG	0.05239996	0.89488677
nonDEG	0.20869155	0.78962504	1	nonDEG	-0.1333291	0.86550424

Comparison_5	DEG/nonDEG	Comparison_6	Comparison_6	Comparison_6	DEG/nonDEG	Comparison_7
1	nonDEG	-0.2714563	0.06049815	0.21275892	nonDEG	0.29974413
1	nonDEG	-1.7900355	6.12E-22	1.68E-19	DEG	-0.0506874
1	nonDEG	0.29212484	0.27914075	0.59440322	nonDEG	0.20020435
1	nonDEG	-0.4767925	0.00387217	0.02656369	DEG	-0.0842464
1	nonDEG	-0.2985711	0.14760308	0.39373751	nonDEG	0.18258671
0.90740781	nonDEG	0.6101457	0.25027631	0.55302116	nonDEG	-1.5031264
1	nonDEG	-0.1716166	0.54534586	0.84652975	nonDEG	-0.1679297
1	nonDEG	0.03107444	0.85495153	1	nonDEG	0.05155457
1	nonDEG	0.82679176	0.00398036	0.02716854	DEG	-0.0105483
1	nonDEG	-0.7694058	5.15E-05	0.00076135	DEG	-0.7692517
1	nonDEG	-0.0488276	0.90412897	1	nonDEG	-0.3030894
1	nonDEG	0.75607836	7.61E-07	1.94E-05	DEG	-1.1626352
1	nonDEG	0.79874579	0.00219712	0.01686155	DEG	-0.2785129
1	nonDEG	-0.1327153	0.712227	0.98014448	nonDEG	-0.2439894
1	nonDEG	0.22172785	0.44043063	0.7694859	nonDEG	-0.7429611
1	nonDEG	0.13073283	0.63431945	0.92288872	nonDEG	0.18800045
1	nonDEG	-0.3925797	0.14493104	0.38897118	nonDEG	-0.0232855
1	nonDEG	-2.6777788	1.22E-18	2.15E-16	DEG	0.11187563
0.32983857	nonDEG	0.10702069	1	1	nonDEG	-2.6790295
1	nonDEG	-0.0222228	0.89082856	1	nonDEG	-0.2812087
1	nonDEG	-0.7851906	0.0002347	0.00270211	DEG	-0.0092461
1	nonDEG	-0.0257911	0.92495208	1	nonDEG	0.52572534
1	nonDEG	0.41880036	0.00824202	0.04790463	DEG	-0.391841
1	nonDEG	-0.377523	0.43387695	0.76233811	nonDEG	0.37915569
0.20403103	nonDEG	-0.70232	0.0477981	0.17922916	nonDEG	-0.0135069
1	nonDEG	-0.2187324	0.16291993	0.41986928	nonDEG	-0.2742778
1	nonDEG	-0.0361263	0.81653891	1	nonDEG	0.22093644
1	nonDEG	-0.11203	0.4543655	0.78286702	nonDEG	-0.139688
1	nonDEG	0.85055457	4.42E-07	1.21E-05	DEG	-0.2973628
0.64232638	nonDEG	0.66731147	0.13129009	0.36377766	nonDEG	0.14613073
1	nonDEG	0.63830371	0.02049843	0.09639828	nonDEG	0.4233522
1	nonDEG	-0.9881719	0.23821894	0.53562419	nonDEG	0.11772834
1	nonDEG	-0.5800978	0.02208035	0.10175696	nonDEG	0.04389358
1	nonDEG	1.18527272	0.00165573	0.01342991	DEG	-0.796056
1	nonDEG	0.25745097	0.24867035	0.55092298	nonDEG	-0.0448114
1	nonDEG	-0.3145214	0.04879963	0.18226493	nonDEG	0.17895271
1	nonDEG	-0.4906357	0.16255107	0.4197061	nonDEG	1.18441246
1	nonDEG	-1.0321844	0.04899928	0.18277873	nonDEG	0.68661371
1	nonDEG	-0.1820529	0.23236771	0.52665286	nonDEG	-0.0068364
1	nonDEG	0.33678966	0.27446974	0.58794402	nonDEG	0.13546014
1	nonDEG	0.21667483	0.4102666	0.7385702	nonDEG	0.391101

1	nonDEG	0.12857502	0.42135119	0.75040989	nonDEG	0.04164399
1	nonDEG	0.15610698	0.57348315	0.87168372	nonDEG	0.24245383
1	nonDEG	-0.1407396	0.85372903	1	nonDEG	-0.545041
1	nonDEG	-0.1283003	0.70555609	0.97540095	nonDEG	0.02162192
1	nonDEG	0.1534211	0.40486117	0.73305296	nonDEG	-0.217054
1	nonDEG	-0.1599782	0.32574497	0.64377202	nonDEG	0.2228704
0.22795628	nonDEG	-0.0631517	0.69474448	0.96711664	nonDEG	-0.6792972
1	nonDEG	-0.0700145	0.65597437	0.938623	nonDEG	0.26281399
1	nonDEG	0.00847505	0.95751678	1	nonDEG	0.52351118
1	nonDEG	-0.4223226	0.18784555	0.4607937	nonDEG	-0.0362876
1	nonDEG	-0.3648571	0.01241627	0.06585005	nonDEG	-0.3571613
1	nonDEG	0.37088542	0.06180707	0.2164571	nonDEG	-0.6835922
1	nonDEG	-0.1745068	0.30131551	0.61847471	nonDEG	0.52133545
1	nonDEG	-0.4372231	0.05851942	0.20783913	nonDEG	-0.1572824
1	nonDEG	-1.1721658	4.02E-15	4.83E-13	DEG	-0.0533761
1	nonDEG	0.4182017	0.10719079	0.31813588	nonDEG	0.35197143
1	nonDEG	-0.46651	0.15004963	0.39813528	nonDEG	0.01981628
1	nonDEG	0.06785076	0.77725487	1	nonDEG	-1.2878702
1	nonDEG	-0.2555015	0.20066388	0.47882237	nonDEG	0.16123424
1	nonDEG	0.03630646	0.8751571	1	nonDEG	-0.3889428
1	nonDEG	-0.5320858	0.00042326	0.00441386	DEG	0.04874098
1	nonDEG	-1.9980644	0.0003511	0.00377225	DEG	1.10490979
1	nonDEG	-1.6136731	0.0348919	0.14198821	nonDEG	1.79457403
1	nonDEG	0.5293528	0.01690978	0.08313412	nonDEG	-0.1475503
1	nonDEG	0.34612597	0.22650448	0.5176386	nonDEG	0.12573648
1	nonDEG	0.281652	0.12478006	0.35179087	nonDEG	-0.4742153
1	nonDEG	0.32348028	0.03917734	0.15468638	nonDEG	0.08559655
1	nonDEG	-0.2043018	0.32907353	0.6479997	nonDEG	-0.0343845
1	nonDEG	2.11048138	2.44E-12	1.99E-10	DEG	0.90050396
0.16923514	nonDEG	-0.5394505	0.10470912	0.31284303	nonDEG	0.69303054
1	nonDEG	0.13971663	0.47766197	0.80639355	nonDEG	0.00144814
1	nonDEG	-0.1929164	0.38494631	0.71174692	nonDEG	0.29534302
1	nonDEG	-1.0424999	4.02E-08	1.41E-06	DEG	0.50801611
1	nonDEG	-0.6031656	0.00397085	0.0271225	DEG	0.63639422
1	nonDEG	-0.0510163	0.91221372	1	nonDEG	0.34135201
1	nonDEG	0.46125694	0.0362981	0.14632066	nonDEG	0.49181698
1	nonDEG	0.67313078	1.00E-05	0.00018545	DEG	-0.1564125
1	nonDEG	-0.2276063	0.20529977	0.48587667	nonDEG	0.09142824
1	nonDEG	0.34432264	0.0360214	0.14545405	nonDEG	0.58523678
1	nonDEG	0.30744739	0.0598758	0.21127696	nonDEG	-0.008851
1	nonDEG	0.47956405	0.04576884	0.17331991	nonDEG	-0.0188275
1	nonDEG	-0.2291439	0.11585766	0.33504259	nonDEG	-0.0610657
1	nonDEG	-0.0514772	0.80530529	1	nonDEG	-0.4693964

1	nonDEG	0.26957845	0.07433803	0.24725312	nonDEG	0.14466227
1	nonDEG	0.23461616	0.10235788	0.30802174	nonDEG	-0.578541
1	nonDEG	0.02565753	0.86075992	1	nonDEG	-0.2011803
1	nonDEG	0.24013629	0.30053238	0.61754414	nonDEG	0.45713362
1	nonDEG	-0.9919575	0.01100805	0.06002335	nonDEG	0.02388154
1	nonDEG	0.40739358	0.01692877	0.08321356	nonDEG	0.15218618
0.87665536	nonDEG	-0.5813298	0.14621854	0.39128513	nonDEG	0.78993379
1	nonDEG	-0.1510392	0.39885563	0.72624388	nonDEG	0.48879723
1	nonDEG	-0.5413529	0.55866519	0.85846877	nonDEG	0.82038406
1	nonDEG	-0.1512178	0.63671418	0.92495242	nonDEG	-0.3720978
1	nonDEG	0.0730211	0.68139435	0.95699481	nonDEG	-0.6271283
1	nonDEG	-0.2078498	0.21583991	0.50246014	nonDEG	-0.6165744
1	nonDEG	-0.5146761	0.12579856	0.35327518	nonDEG	-0.6489193
1	nonDEG	-0.8941607	2.42E-07	7.00E-06	DEG	0.04221383
1	nonDEG	-0.1175232	0.5449611	0.84636224	nonDEG	0.47377804
1	nonDEG	0.16315332	0.27878268	0.59383854	nonDEG	-0.5550468
1	nonDEG	-0.5662495	0.13048845	0.3626746	nonDEG	0.33559735
1	nonDEG	-0.0067015	0.96837286	1	nonDEG	-0.0457941
1	nonDEG	-0.0525352	0.90327526	1	nonDEG	-0.5754187
1	nonDEG	0.55834446	0.02322697	0.10563623	nonDEG	-0.613299
1	nonDEG	-0.4291551	0.00675161	0.04109173	DEG	0.24718619
1	nonDEG	-0.2741856	0.51290211	0.83922438	nonDEG	-0.4029647
1	nonDEG	-0.2736084	0.05606215	0.20163882	nonDEG	0.36606102
1	nonDEG	0.32985841	0.03489537	0.14198821	nonDEG	-0.1674437
1	nonDEG	-0.0430426	0.87962092	1	nonDEG	0.73883151
1	nonDEG	-1.2153921	2.15E-17	3.26E-15	DEG	-0.3890365
0.64883717	nonDEG	-1.0853518	0.00106818	0.0094832	DEG	0.37702203
1	nonDEG	0.63823689	0.00043205	0.00448965	DEG	0.26869133
1	nonDEG	0.63860376	2.41E-05	0.00039661	DEG	0.55840882
1	nonDEG	3.00040018	0.53625896	0.84234591	nonDEG	0.73212393
1	nonDEG	0.43696698	0.00359163	0.02510304	DEG	0.62373662
1	nonDEG	0.25197706	0.15579178	0.4076397	nonDEG	-0.5901956
1	nonDEG	-0.2243864	0.13214691	0.36559278	nonDEG	0.15867471
1	nonDEG	-0.2841667	0.4092524	0.73742083	nonDEG	0.87641888
1	nonDEG	-0.7585156	0.00328758	0.02335496	DEG	-0.7135173
1	nonDEG	-0.2745878	0.07262336	0.24300511	nonDEG	-0.3147553
1	nonDEG	0.80060977	2.42E-06	5.38E-05	DEG	0.94563149
1	nonDEG	-0.2751213	0.40674035	0.73466686	nonDEG	1.04104254
1	nonDEG	0.95803226	1.61E-11	1.13E-09	DEG	-0.4111239
1	nonDEG	0.20216194	0.38409137	0.71119352	nonDEG	0.153757
1	nonDEG	-0.1748905	0.24683518	0.54825569	nonDEG	-0.2294139
1	nonDEG	0.07086841	0.66578787	0.94526272	nonDEG	-0.2944552
1	nonDEG	0.09335894	0.80018219	1	nonDEG	0.08072202

1	nonDEG	-0.1251407	0.66481533	0.94491728	nonDEG	-0.1984243
1	nonDEG	-0.8113594	0.04292922	0.16550759	nonDEG	-0.5717109
1	nonDEG	-0.372031	0.00932973	0.05273767	nonDEG	-0.4994497
1	nonDEG	-1.18993	0.02863052	0.12345891	nonDEG	0.42728173
1	nonDEG	-0.0139394	0.95255892	1	nonDEG	-0.1091699
0.89780005	nonDEG	-0.2871292	0.07503602	0.2489065	nonDEG	0.08273659
1	nonDEG	0.08913239	0.61312932	0.90557465	nonDEG	0.33426022
1	nonDEG	-0.2166589	0.17540418	0.44055317	nonDEG	0.25188852
1	nonDEG	-0.5538686	0.00143912	0.01205301	DEG	-0.2639628
1	nonDEG	-0.0918888	0.54613276	0.84721517	nonDEG	-0.6175828
1	nonDEG	0.35084804	0.08168669	0.26465315	nonDEG	0.26745246
1	nonDEG	-2.5932717	1	1	nonDEG	0
1	nonDEG	-0.5497224	0.16539487	0.42420801	nonDEG	-0.2804583
1	nonDEG	-1.7800827	2.42E-06	5.37E-05	DEG	0.28372655
1	nonDEG	-0.0198214	0.8988748	1	nonDEG	-0.0225941
1	nonDEG	1.25231371	2.44E-16	3.29E-14	DEG	0.37863357
1	nonDEG	0.19924633	0.25703898	0.56322818	nonDEG	0.23478706
1	nonDEG	-0.3423815	0.04447712	0.16976039	nonDEG	0.44240267
1	nonDEG	-0.2882762	0.4567798	0.78479177	nonDEG	0.23890001
1	nonDEG	0.65513944	0.0015723	0.01291491	DEG	0.69787823
1	nonDEG	-0.7110676	0.06710962	0.22926631	nonDEG	-0.3099878
1	nonDEG	-0.2110758	0.24256618	0.54245713	nonDEG	-0.3968904
1	nonDEG	-0.3419352	0.27497305	0.58856897	nonDEG	-0.1467754
1	nonDEG	-0.52336	0.00107489	0.00952017	DEG	0.18825249
1	nonDEG	-0.6452032	0.22369412	0.51383669	nonDEG	0.04117934
1	nonDEG	-0.6931802	0.01748234	0.08527964	nonDEG	-0.3931685
1	nonDEG	0.94665488	2.82E-06	6.16E-05	DEG	-0.8935461
1	nonDEG	-0.6627231	0.3573668	0.68041142	nonDEG	1.57917128
1	nonDEG	-0.2757442	0.46097456	0.78925867	nonDEG	0.22615331
1	nonDEG	0.62167553	0.00373573	0.02582811	DEG	0.32783235
1	nonDEG	0.64102583	1.08E-05	0.00019787	DEG	0.62534363
1	nonDEG	0.43165958	0.00262715	0.01950585	DEG	0.05576881
1	nonDEG	-0.8878448	1.68E-05	0.00029002	DEG	-0.7358966
1	nonDEG	0.25441205	0.50102266	0.83007519	nonDEG	-0.5474772
1	nonDEG	0.4058806	0.58982917	0.88614345	nonDEG	-0.2479341
1	nonDEG	0.80649852	9.91E-05	0.00132114	DEG	-0.1528645
1	nonDEG	-1.4306707	4.86E-13	4.34E-11	DEG	-0.7974992
1	nonDEG	-0.1478621	0.73541784	0.99553804	nonDEG	0.62815443
1	nonDEG	0.01922712	1	1	nonDEG	1.40610229
1	nonDEG	-0.774572	0.00238555	0.01801649	DEG	-0.4844337
1	nonDEG	-1.8877253	0.24177863	0.54108413	nonDEG	3.43069774
1	nonDEG	0.54886652	0.00557461	0.03533068	DEG	0.93722689
1	nonDEG	-0.0234785	0.91783963	1	nonDEG	-0.0451215

1	nonDEG	-1.5982643	9.24E-22	2.50E-19	DEG	0.34696566
1	nonDEG	0.81611277	6.52E-05	0.00092886	DEG	0.65767646
1	nonDEG	0.96180873	1.88E-10	1.09E-08	DEG	0.78065005
1	nonDEG	0.02773521	0.94430729	1	nonDEG	-0.1703456
1	nonDEG	0.40181964	0.0359766	0.14534311	nonDEG	-0.6470304
1	nonDEG	-0.9179036	0.07480606	0.24839762	nonDEG	-0.2676684
1	nonDEG	0.10546455	0.49989109	0.82894246	nonDEG	-0.2031176
1	nonDEG	0.44238517	0.02493801	0.111556	nonDEG	-0.3888905
1	nonDEG	-0.7910445	0.0502613	0.18604833	nonDEG	-0.3971887
1	nonDEG	0.2276458	0.39111147	0.71808683	nonDEG	0.03415778
1	nonDEG	0.82673421	4.08E-08	1.43E-06	DEG	0.68016389
1	nonDEG	0.38812707	0.39800518	0.72532342	nonDEG	-0.0379156
1	nonDEG	0.52153305	0.06988549	0.23615231	nonDEG	0.44362926
1	nonDEG	-0.3107077	0.50642822	0.83531715	nonDEG	0.23830451
1	nonDEG	0.05214456	0.74491227	1	nonDEG	-0.3529024
1	nonDEG	0.24144689	0.1257963	0.35327518	nonDEG	0.17165964
1	nonDEG	0.15015122	0.63920231	0.92694457	nonDEG	0.02972517
1	nonDEG	-0.2685119	0.14483292	0.38888511	nonDEG	-0.2249132
1	nonDEG	0.16604701	0.26289458	0.57177276	nonDEG	-0.0923597
0.28787571	nonDEG	-0.3815734	0.29889333	0.61626304	nonDEG	-0.3678499
1	nonDEG	-0.1422689	0.69412193	0.96661568	nonDEG	-0.2168078
1	nonDEG	0.09447008	0.69226548	0.96504457	nonDEG	-0.4298375
1	nonDEG	-1.8080458	4.27E-11	2.80E-09	DEG	0.39696449
1	nonDEG	-1.3986824	1.74E-07	5.29E-06	DEG	0.37606699
1	nonDEG	-0.019447	0.9510474	1	nonDEG	0.16982487
1	nonDEG	-0.2619888	0.19159594	0.46572428	nonDEG	-0.2200312
1	nonDEG	0.45837983	0.00820999	0.04775613	DEG	-0.6885933
1	nonDEG	-0.2685725	0.18131756	0.44995428	nonDEG	0.09528925
1	nonDEG	-0.2186299	0.29148587	0.606511	nonDEG	-0.2958917
1	nonDEG	0.02376073	0.87304157	1	nonDEG	-0.0534435
1	nonDEG	0.59980135	0.47116594	0.79913892	nonDEG	-0.0749148
1	nonDEG	-1.6958634	0.00155873	0.01282849	DEG	0.63327769
1	nonDEG	-0.4266795	0.07639768	0.25202504	nonDEG	0.31825336
1	nonDEG	0.1550079	0.52552857	0.84234591	nonDEG	0.50910115
1	nonDEG	0.87285839	2.01E-08	7.57E-07	DEG	0.63058128
1	nonDEG	0.92162481	0.0001061	0.00139818	DEG	-0.9323936
1	nonDEG	0.51994495	0.24273036	0.54270063	nonDEG	0.50361469
1	nonDEG	-0.1105656	0.4604075	0.78852711	nonDEG	0.11917796
1	nonDEG	-0.2115601	0.13828339	0.37749672	nonDEG	-0.44225
1	nonDEG	-0.2547315	0.09028043	0.2819013	nonDEG	-0.0957981
1	nonDEG	-0.0844785	0.56535718	0.86455509	nonDEG	-0.1984796
1	nonDEG	-0.4703144	0.14731615	0.393237	nonDEG	1.07125715
1	nonDEG	-0.286022	0.33270839	0.65223429	nonDEG	-0.1743381

1	nonDEG	-0.4974734	0.00082135	0.0076421	DEG	-0.1379472
1	nonDEG	0.52399969	0.00475948	0.03117118	DEG	-0.1916432
1	nonDEG	-0.5119083	0.12414152	0.35069591	nonDEG	0.05209489
0.4063882	nonDEG	0.31547036	0.33644127	0.65647788	nonDEG	0.50983758
1	nonDEG	-1.9920707	3.07E-23	1.04E-20	DEG	-0.687396
1	nonDEG	0.18032609	0.2362159	0.53234019	nonDEG	-0.2473657
1	nonDEG	-0.2406432	0.19598116	0.47232824	nonDEG	-0.7192081
1	nonDEG	-0.960057	0.0167882	0.08268827	nonDEG	-0.0385525
1	nonDEG	-0.0484023	0.84182634	1	nonDEG	0.36366717
1	nonDEG	0.61698098	2.96E-05	0.000475	DEG	-0.5367732
1	nonDEG	-0.2126406	0.37354591	0.69940965	nonDEG	-1.2857411
1	nonDEG	0.286016	0.29987663	0.61734741	nonDEG	0.28008857
1	nonDEG	-0.4552968	0.00873144	0.049993	DEG	-1.2470034
1	nonDEG	0.31337112	0.08837757	0.27790058	nonDEG	0.06684339
1	nonDEG	0.43106618	0.18494111	0.45549077	nonDEG	-0.0268743
1	nonDEG	-0.9624216	0.00076091	0.00719812	DEG	0.42431354
1	nonDEG	0.78883129	0.46993441	0.7983176	nonDEG	-0.6450336
1	nonDEG	-0.599508	0.02781957	0.12076642	nonDEG	0.88094064
1	nonDEG	-1.8197664	0.00019493	0.00231296	DEG	-0.2309191
1	nonDEG	-0.3609726	0.01743554	0.08509363	nonDEG	0.09265953
1	nonDEG	-1.1946172	0.00939709	0.05305732	nonDEG	-1.7302529
1	nonDEG	-0.4601778	0.0117989	0.06338463	nonDEG	-0.1464569
0.86163597	nonDEG	-0.736279	0.00952346	0.0536782	nonDEG	-0.1026626
1	nonDEG	-0.0769179	0.87243308	1	nonDEG	0.13745749
1	nonDEG	-0.0684967	0.65681707	0.9392305	nonDEG	-0.5634582
[truncated: 487,384 more chars]
